# Harnessing Oxetane and Azetidine Sulfonyl Fluorides for Opportunities in Drug Discovery

**DOI:** 10.1021/jacs.4c14164

**Published:** 2024-12-12

**Authors:** Oliver L. Symes, Hikaru Ishikura, Callum S. Begg, Juan J. Rojas, Harry A. Speller, Anson M. Cherk, Marco Fang, Domingo Leung, Rosemary A. Croft, Joe I. Higham, Kaiyun Huang, Anna Barnard, Peter Haycock, Andrew J. P. White, Chulho Choi, James A. Bull

**Affiliations:** † Department of Chemistry, Imperial College London, Molecular Sciences Research Hub, White City Campus, Wood Lane, London W12 0BZ, U.K.; †† Medicine Design, Pfizer Research and Development, Groton, Connecticut 06340, United States

## Abstract

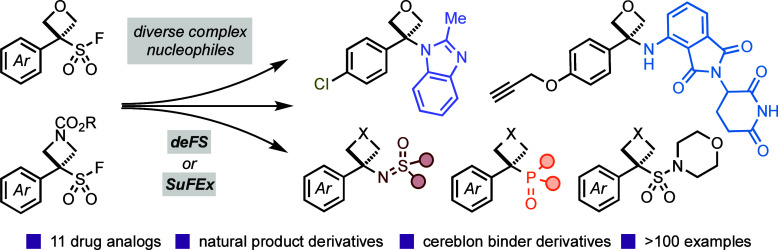

Four-membered heterocycles such as oxetanes and azetidines represent attractive and emergent design options in medicinal chemistry due to their small and polar nature and potential to significantly impact the physiochemical properties of drug molecules. The challenging preparation of these derivatives, especially in a divergent manner, has severely limited their combination with other medicinally and biologically important groups. Consequently, there is a substantial demand for mild and effective synthetic strategies to access new oxetane and azetidine derivatives and molecular scaffolds. Here, we report the development and use of oxetane sulfonyl fluorides (OSFs) and azetidine sulfonyl fluorides (ASFs), which behave as precursors to carbocations in an unusual defluorosulfonylation reaction pathway (deFS). The small-ring sulfonyl fluorides are activated under mild thermal conditions (60 °C), and the generated reactive intermediates couple with a broad range of nucleophiles. Oxetane and azetidine heterocyclic, -sulfoximine, and -phosphonate derivatives are prepared, several of which do not have comparable carbonyl analogs, providing new chemical motifs and design elements for drug discovery. Alternatively, a SuFEx pathway under anionic conditions accesses oxetane-sulfur(VI) derivatives. We demonstrate the synthetic utility of novel OSF and ASF reagents through the synthesis of 11 drug analogs, showcasing their potential for subsequent diversification and facile inclusion into medicinal chemistry programs. Moreover, we propose the application of the OSF and ASF reagents as linker motifs and demonstrate the incorporation of pendant groups suitable for common conjugation reactions. Productive deFS reactions with E3 ligase recruiters such as pomalidomide and related derivatives provide new degrader motifs and potential PROTAC linkers.

## Introduction

Precise control of molecular properties and conformation is essential in drug discovery.^1^ Effective interactions with targeted biological sites require complementary electron density surfaces, modulated through combinations of polar, aromatic, and hydrophobic sites. Small polar groups such as oxetanes,^2^ sulfoximines,^3^ and phosphine oxides^4^ have emerged as valuable bioisosteres that mimic the spatial electronic properties of more common functional groups and potentially enhance binding through tailoring polarity and H-bonding properties. These groups are also beneficial in providing increased three-dimensionality and in influencing metabolic stability, solubility, absorption, distribution, and p*K*_a_. Thus, these small polar motifs have provided genuine new design options for medicinal chemists.

**Figure 1:**
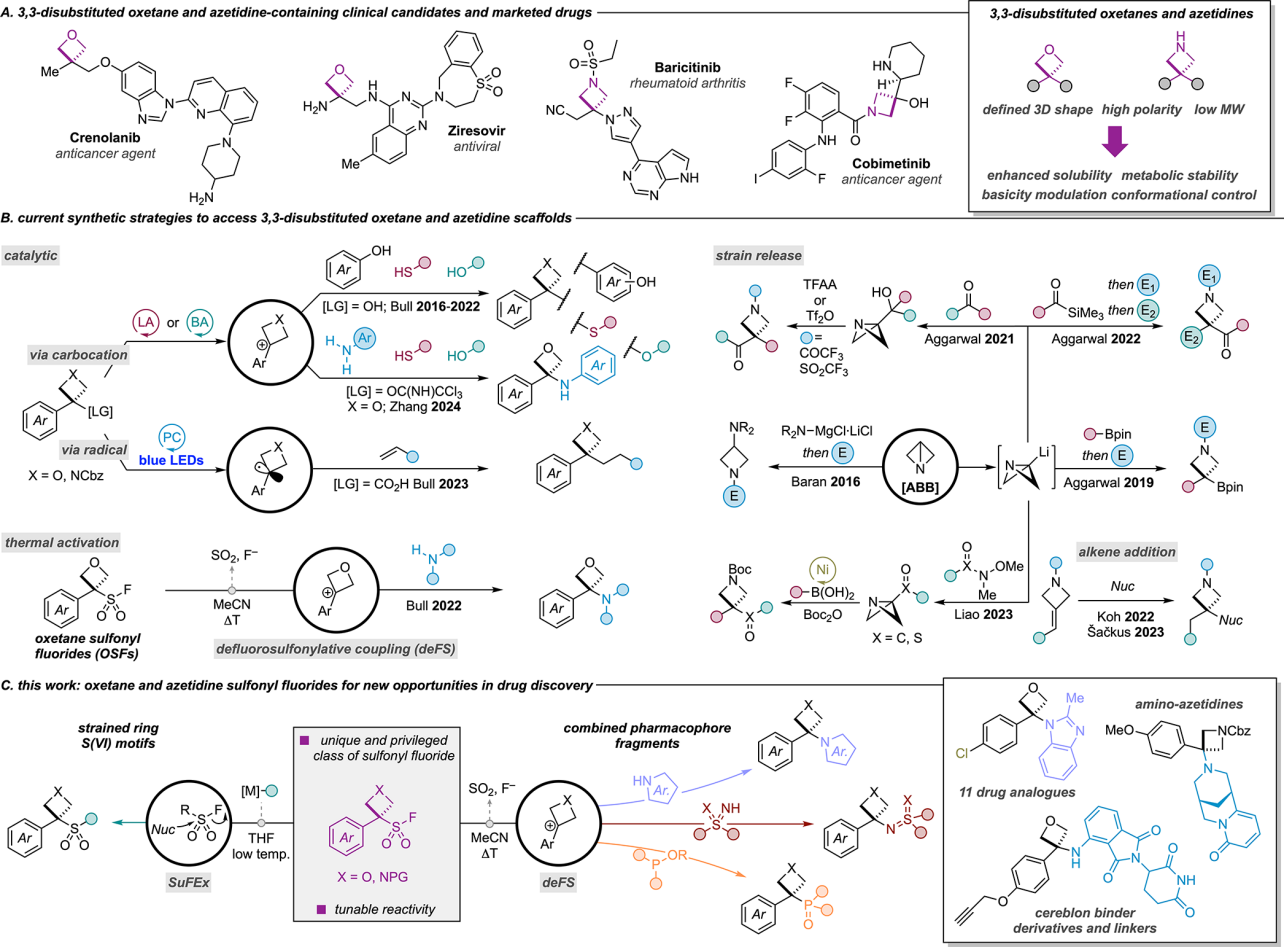
(A) Oxetanes and azetidines in clinical candidates and marketed drugs; (B) current strategies to access 3,3-disubstituted oxetanes and azetidines; and (C) this work: oxetane and azetidine SFs for new opportunities in drug discovery.

Oxetanes are a notable example of such a motif, with nine oxetane-containing compounds currently in clinical trials (Figure 1a).^2a^ Carreira’s seminal studies established oxetanes as valuable replacements for carbonyl and *gem*-dimethyl functionalities, expanding their medicinal chemistry potential by advancing the chemistry of oxetanone and oxetane-Michael acceptors.^5,6^ We have demonstrated the Lewis and Brønsted acid-catalyzed formation of oxetane carbocations from oxetanols, facilitating trapping with arenes, thiols, and alcohols to generate 3,3-disubstituted derivatives (Figure 1b).^7,8^ Very recently, Zhang and co-workers also developed a Lewis acid activation of oxetanyl trichloroacetimidates for the generation of oxetane carbocations.^9^ These advancements, along with the development of alternative electrophiles^10,11^ and other cross-coupling and radical processes,^12^ accentuate the significance of oxetanes in drug discovery.^13^

Similarly, azetidines are undergoing extensive investigation and though less represented than their five- and six-membered counterparts are rapidly gaining prominence in approved drugs (Figure 1a).^14^ Pioneering work by Baran stimulated the adoption of azabicyclo[1.1.0]butane (ABB) strain-release reagents, enabling late-stage “azetidinylation” of secondary amines (Figure 1b).^15^ Aggarwal leveraged 3-lithiated ABBs as nucleophiles in the modular synthesis of azetidine scaffolds.^16,17^ Radical processes^18^ and additions to alkylidene-azetidines^19^ have been developed to generate valuable 3,3-substiuted azetidine derivatives.

Recently, we introduced a unique deFS coupling process of OSFs with neutral amine nucleophiles.^20^ SFs typically react with nucleophiles in Sulfur–Fluoride Exchange (SuFEx) reactions to yield S(VI) derivatives such as sulfonamides and sulfonate esters.^21^ In contrast to sulfonyl chlorides, sulfonyl fluorides are notably more stable and can persist intact through further reactions. The combination of high stability with selective activation^22^ has made the SF functional group ideal for applications in drug discovery and chemical biology^23^ and was coined a “click reagent” by Sharpless in an influential report in 2014.^21a^ 3-Aryloxetane-3-sulfonyl fluorides did not show the expected SuFEx reactivity. Instead, these reagents underwent selective deFS upon mild thermal activation (60 °C) to generate oxetane carbocations that were trapped with a variety of amine nucleophiles to yield amino-oxetanes as potential isosteres of amides. Amine libraries were directly coupled, and the deFS reaction demonstrated high functional group tolerance.

Here, we demonstrate the potent capabilities of oxetane sulfonyl fluorides (OSFs) and azetidine sulfonyl fluorides (ASFs) as versatile, customizable reagents for the generation of a diverse array of novel pharmacophore motifs (Figure 1c). The facile generation of carbocations from OSF reagents is exploited to synthesize several novel oxetane analogs of biologically active molecules and marketed drugs. We present the inaugural synthesis of ASFs and demonstrate their compatibility with the deFS coupling chemistry, offering an attractive alternative to azabicyclo[1.1.0]butane (ABB) reagents and their derivatives. Control of the solvent and nucleophile strength unlocks the previously challenging SuFEx reaction pathway for these reagents, resulting in novel strained ring S(VI) motifs. Finally, we demonstrate the potential for forming new degrader motifs or PROTAC linker units through coupling with E3 ligase ligands. With a repertoire exceeding 100 novel oxetane and azetidine fragments, our study underlines the synthetic versatility of OSF and ASF reagents as a flexible platform to facilitate access to innovative chemical space in drug discovery.

## Results and Discussion

**Scheme 1:**
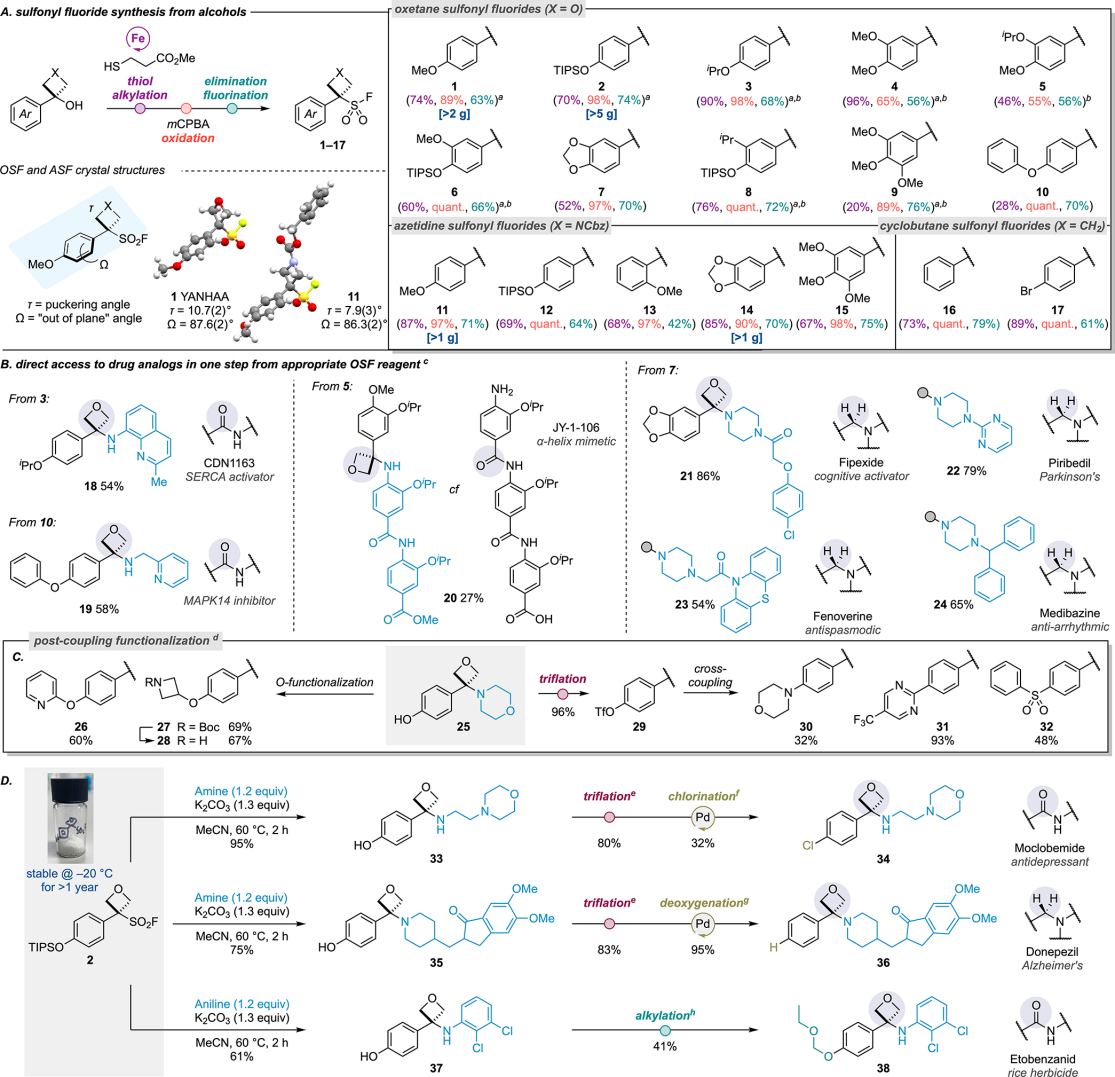
(A) Preparation of oxetane, azetidine, and cyclobutane-sulfonyl fluorides from tertiary alcohols and (B) direct access to oxetane analogs of biologically relevant compounds; (C) post-deFS arene functionalization; (D) use of OTIPS OSF 2 for rapid access to oxetane analogs of biologically relevant compounds Compounds **1**–**4**, **6**, **8**, **9**, **25**, and **29** were reported in ref (20). Sulfide prepared using Li-catalysis. deFS conditions: OSF (1.0 equiv), amine (1.2 equiv), K_2_CO_3_ (1.3 equiv), acetonitrile (0.3 M), 60 °C, 2–5 h. For full details of the reaction conditions, see the Supporting Information. Conditions: Tf_2_O (1.08 equiv), pyridine (2.0 equiv), CH_2_Cl_2_ (0.5 M), 0–25 °C, 3 h. Conditions: Pd_2_(dba)_3_ (1.5 mol %), *t*BuBrettPhos (4.5 mol %), KCl (2.0 equiv), KF (0.5 equiv), 1,4-dioxane (0.25 M), 130 °C, 16 h. Conditions: Pd(OAc)_2_ (2 mol %), dppf (2 mol %), Et_3_N (3.0 equiv), formic acid (2.0 equiv), DMF (0.1 M), 60 °C, 1 h. Conditions: chloromethyl ethyl ether (1.1 equiv), K_2_CO_3_ (2.0 equiv), acetone (0.2 M), 40 °C, 24 h.

Four-membered ring sulfonyl fluorides were prepared initially through a three-step sequence from the readily available tertiary alcohols (Scheme 1a). Thiol alkylation of the aryloxetanols used either a lithium triflimide catalyst or inexpensive iron chloride as a catalyst in a modified sequence to enable a more scalable and reliable process (see below and SI for further details). Subsequent oxidation, for which flash chromatography was not required, and an elimination/fluorination sequence readily provided the sulfonyl fluoride reagents. Large-scale preparation of PMP OSF derivative **1** on >2 g and OTIPS OSF derivative **2** on >5 g scale was achieved in single runs. Pleasingly, the azetidine derivatives proceeded in a similar manner and afforded a series of *N*-Cbz-ASFs (**11**–**15**) through the thiol alkylation–oxidation–elimination/fluorination sequence (Scheme 1a). Cyclobutane derivatives were also prepared through this sequence (**16**, **17**) and tolerated more electron-poor arenes. To date, heteroarene and electron-poor arene containing OSF and ASF reagents are not directly accessible. In each case, the OSF and ASF reagents were stable solids stored at −20 °C for >1 year. OSF **1** and ASF **1** were characterized by X-ray crystallography and displayed comparable conformations. Each of the OSF reagents was demonstrated to react through the unusual deFS pathway to generate amino-oxetanes (see SI for further details). The conditions involve simply warming at 60 °C in acetonitrile in the presence of K_2_CO_3_ as base, which minimizes the formation of a minor oxetane fluoride side product. Previously, we demonstrated that the suitable selection of OSF reagent and amine nucleophile could be applied to the preparation of 10 oxetane analogs of benzamide drugs, with the amino-oxetane providing potential bioisosteric replacement. Here, we applied alternative OSF reagents to form directly oxetane analogs of amides (Scheme 1b), including highly chelating nucleophiles that would have been challenging in Lewis acid catalysis and thus highlighting the ease and tolerance of these thermal conditions. Isopropyl OSF **3** reacted readily with 2-methylquinolin-8-amine to form an analog of CDN1163, a SERCA activator of interest for the treatment of diabetic-induced immune dysfunction.^24^ Phenoxyarylether OSF **10** reacted similarly with 2-picolylamine to form the oxetane analog of a MAPK14 inhibitor.^25^ Oligobenzamides have been applied as α-helix mimetics and show promising potential as anticancer agents but often suffer from poor aqueous solubility.^26^ JY-1-106 disrupts the Bcl-x_L_/Bak protein–protein interaction by mimicking the α-helical BH3 domain of Bak.^27^*meta*-Isopropoxy-containing OSF **5** allowed access to an oxetane analog of JY-1-106 with increased three-dimensionality.

Six of the nine current oxetane-containing clinical candidates are substituted in the 3-position with an amine functional group, whereby the oxetane plays a crucial role in reducing the basicity of the amine to appropriate levels. Consequently, the application of oxetanes as analogs of benzylic amines may have advantages in controlling p*K*_a_ as well as affecting metabolism at benzylic sites. Benzodioxole is a scaffold frequently seen across natural products and approved medicines, and several oxetane analogs of marketed drugs containing benzylic amines were efficiently accessed from benzodioxole OSF **7**. Analogs of fipexide (**21**), piribedil (**22**), fenoverine (**23**), and medibazine (**24**) were all prepared directly by reacting the suitable amine nucleophiles under the deFS conditions (Scheme 1b).

Employing OTIPS OSF **2** in the deFS coupling led to cleavage of the TIPS group by the liberated fluoride anion, unmasking the phenol group of amino-oxetane products and providing a handle for divergent functionalization. The revealed phenol functionality of morpholine-oxetane **25** was readily alkylated (**26**, **27**) or converted to the triflate and applied in palladium-catalyzed processes including Buchwald–Hartwig amination, Suzuki–Miyaura cross-coupling, and sulfonylation reactions (**30**–**32**, Scheme 1c).^28^ Through this strategy, OTIPS OSF reagent **2** was readily converted to diverse oxetane analogs of important biologically active compounds with variation of the arene (Scheme 1d). The reaction of OTIPS OSF **2** with a primary amine followed by deoxygenative chlorination^29^ gave aryl chloride **34**, an oxetane analog to moclobemide, a marketed antidepressant. The reaction with a secondary amine and subsequent deoxygenation gave an oxetane analog of donepezil (**36**), an Alzheimer’s medication. Similarly, reaction with 2,3-dichloroaniline and phenol alkylation gave an oxetane analog of the rice herbicide etobenzanid (**38**). These sequences demonstrate the particular value of the OTIPS OSF **2** reagent for the divergent preparation of oxetane derivatives.

### Merging Pharmacophores

The merger of polar functional groups into new combined pharmacophores presents opportunities for new design options for medicinal chemists. We envisaged that the mild functional group-tolerant conditions of the deFS reaction would enable the installation of oxetanes to combinations of functional groups by employing a new range of nucleophiles to form attractive novel motifs. Moreover, the incorporation of oxetane, with its similarity to the carbonyl functionality, may provide further isosteres for acyl-derivatives of functional groups. Investigation of temperature and equivalents of nucleophiles allowed optimization for each nucleophile class (see SI for further details).

**Scheme 2:**
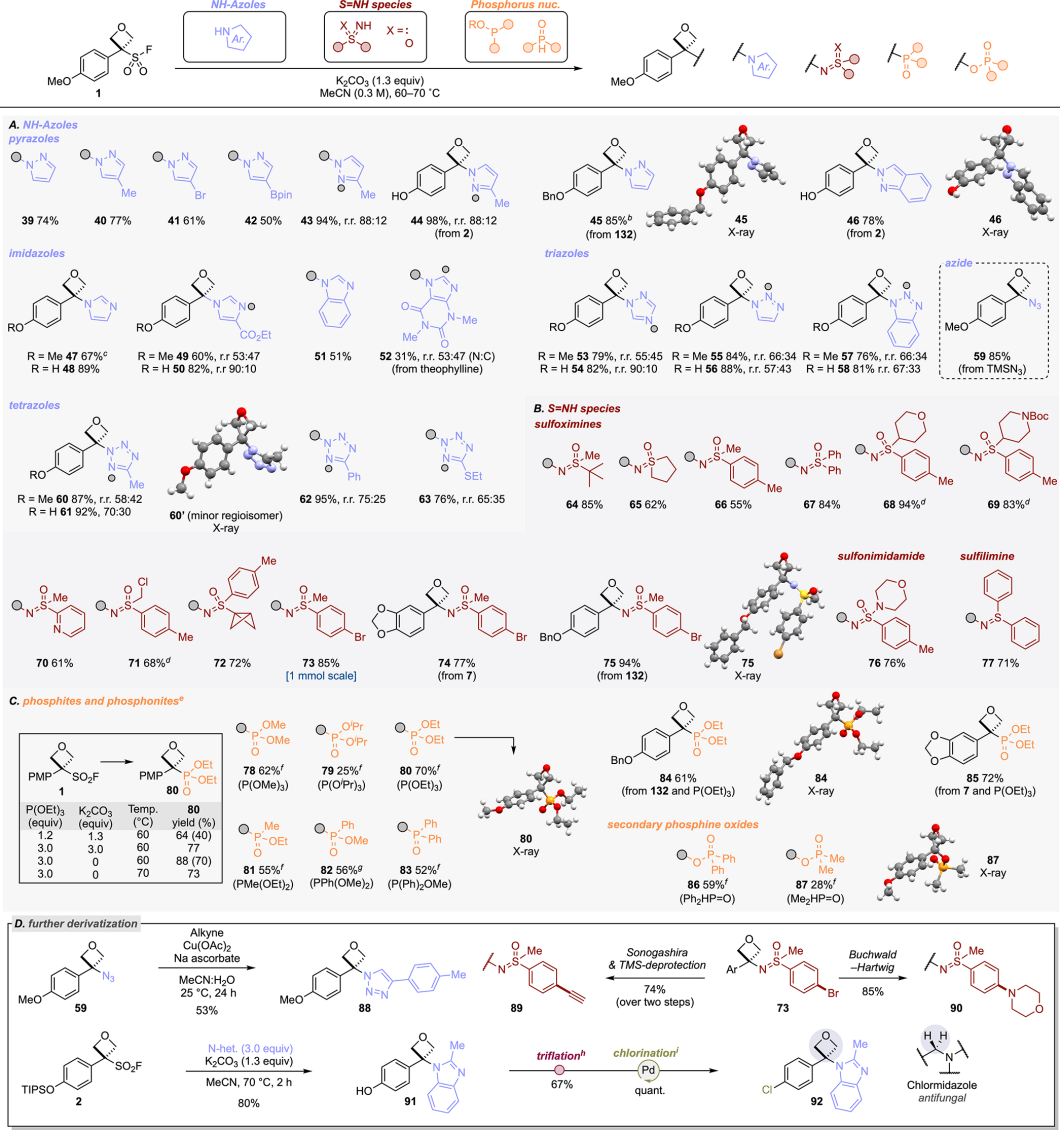
Defluorosulfonylative Coupling of Oxetane Sulfonyl Fluorides with a Diverse Array of Nucleophiles. All reactions in parts A–C performed on a 0.2 mmol scale unless otherwise stated. Conditions: NH-azoles: OSF (1.0 equiv), nucleophile (imidazole = 3.0 equiv; pyrazole = 1.2 equiv; triazole = 3.0 equiv, tetrazole = 3.0 equiv), K_2_CO_3_ (1.3 equiv), acetonitrile (0.3 M), 60 °C (pyrazoles) or 70 °C (imidazoles, triazoles, and tetrazoles), 2–5 h; sulfoximines, sulfonimidamide, and sulfilimine: OSF (1.0 equiv), S = NH nucleophile (3.0 equiv), K_2_CO_3_ (1.3 equiv), acetonitrile (0.3 M), 60 °C, 2 h; phosphorus nucleophiles: OSF (1.0 equiv), nucleophile (3.0 equiv), K_2_CO_3_ (3.0 equiv, only with secondary phosphine oxides), acetonitrile (0.3 M), 60 °C, 2 h. Reported regiomeric ratios (r.r.) determined by relative ^1^H NMR integrations of the crude reaction mixture. Reaction performed at 70 °C for 2 h. Compound **47** reported in ref (20). Reaction performed on a 0.07 mmol scale. Reactions performed in the absence of K_2_CO_3_. Reaction performed on a 0.1 mmol scale. Reaction performed on a 0.15 mmol scale. Tf_2_O (1.08 equiv), pyridine (2.0 equiv), CH_2_Cl_2_ (0.5 M), 0–25 °C, 3 h. Pd_2_(dba)_3_ (1.5 mol %), *t*BuBrettPhos (4.5 mol %), KCl (2.0 equiv), KF (0.5 equiv), 1,4-dioxane (0.25 M), 130 °C, 16 h.

First, we examined NH-azole nucleophiles, which are essential components of drug structures (Scheme 2a). Azoles containing electron-withdrawing groups were of particular interest, which may be mimicked by the electron-withdrawing nature of the oxetane.^30^ A range of substituted pyrazoles was well tolerated, providing oxetano-pyrazoles in moderate to excellent yields (**39**–**46**, 60 °C, 1.2 equiv pyrazole).^31^ Pyrazoles bearing 4-bromo and 4-Bpin groups were well tolerated and introduced an additional synthetic handle. The reaction with 3-methylpyrazole was regioselective at the less hindered nitrogen atom. OTIPS OSF **2** gave phenolic product **44** in quantitative yield, with similar regioselectivity. Azole oxetane derivatives **45**, **46**, and **60’** were further characterized by X-ray crystallography. Unlike typical conjugated diarylketones, the nonconjugated π systems in oxetanes **45** and **60’** are rotated nearly 90° (dihedral angles 89.9° and 79.1°, respectively) to minimize steric interactions, inducing a twisted conformation and creating an atropisomeric axis in the solid state, with one atropisomer crystallizing as a conglomerate.^32^

Slightly elevated temperatures (70 °C) improved the reactivity with imidazole nucleophiles, yielding oxetanimidazoles **47**–**52** from the corresponding PMP and OTIPS OSF reagents. Interestingly, a significant change in regioselectivity was observed when reacting OTIPS OSF **2** in comparison to PMP OSF **1** (**49**: r.r. 53:47; **50**: r.r. 90:10), which is seen also in later examples (**53**, **54**). This may be explained through formation of a different intermediate from OTIPS OSF **2** proceeding through the quinone methide rather than the carbocation intermediate,^33^ which changes the angle of attack of the nucleophile from ca. 90° to the arene plane in the carbocation (p orbital) to ca. 107° in the quinone methide (Bürgi–Dunitz angle; LUMO π* orbital). Benzimidazole and theophylline were also reactive (**51**, **52**), as well as 1,2,4- and 1,2,3-triazoles and benzotriazole, which generated the corresponding oxetano-triazoles in high yields (**53**–**58**). TMSN_3_, in the absence of K_2_CO_3_, afforded oxetane azide **59** in 85% yield, whereby the liberated fluoride anion presumably activates the azide nucleophile and in the process is effectively scavenged as TMSF. Substituted NH-tetrazoles gave a mixture of regioisomers with a general preference for the 3-substituted product due to steric influence of the carbon substituent (**60**–**63**). Notably, the corresponding carbonyl derivatives of such *N*-heterocycles are unstable and represent intermediates in acylation processes by acting as an effective leaving group, often with the azole providing a catalyst, including histidine catalysis.^34^ As such, these stable oxetane-heterocycle derivatives may mimic acylation intermediates previously unusable for drug discovery purposes.

Sulfoximines, the monoaza analogs of sulfones, have themselves become of significant interest in medicinal chemistry.^3^ The imine nitrogen can introduce a stereogenic center and has been shown to instill properties beneficial to drug compounds, including improved solubility, increased polarity, and hydrogen-bond donor and acceptor capabilities. Functionalization of this imine nitrogen can tune the chemical and biological features of sulfoximine derivatives.^35^ Pleasingly, the sulfoximine nitrogen trapped the oxetane carbocation to generate the corresponding *N*-oxetane-sulfoximine fragments (Scheme 2b). High yields were achieved with excess nucleophile (3.0 equiv, 60 °C, 85% **73**), though the reverse stoichiometry gave a similar yield (87%, **73**). A wide variety of sulfoximines were amenable to the deFS coupling, including bulky bis-alkyl- (**64**), cyclic- (**65**), alkyl-aryl- (**66**), and diarylsulfoximines (**67**). Saturated heterocycles (**68**, **69**) and electron-poor pyridyl (**70**) groups were well tolerated. Moreover, potentially reactive sites including a sensitive BCB sulfoximine (**72**) were unaffected and successfully coupled under the mild thermal conditions. 4-Bromophenylmethyl sulfoximine reacted readily with different OSF reagents (**7** and **132**, see Scheme 6), providing a handle for further diversification. *N*-Oxetane-sulfoximine **75** was characterized by X-ray crystallography, revealing a turn conformation about the Ar–C–N=S bond with a gauche arrangement (67.3°) with respect to the oxetane ring, stabilized by π-stacking interactions (*d* = 3.676 Å) between the oxetane and sulfoximine aryl groups in the solid state. Sulfonimidamides (the monoaza analogs of sulfonamides) and sulfilimines (the aza-analog of sulfoxides) also reacted successfully, to afford new oxetane functionalized derivatives (**76**, **77**).

Phosphorus functional groups have been underexplored as medicinal chemistry motifs. Phosphonate and phosphate groups have been deployed in pro-drugs to facilitate cell membrane permeability,^36^ while phosphine oxides are only recently becoming more extensively examined following the approval of Brigatinib.^37^ There is an increasing interest in the incorporation of these motifs into drug-like molecules to modulate Log*D* and solubility. We hypothesized that alkyl phosphite reagents could be used to trap the oxetane carbocation and provide the corresponding phosphorylated oxetane products. We expected the liberated fluoride to effect dealkylation in an Arbuzov mechanism to reveal the P=O bond. Triethylphosphite successfully reacted in this manner with PMP OSF **1** to form oxetane phosphonate **80** (Scheme 2c). Improved yields were afforded by performing the reaction in the absence of K_2_CO_3_, supporting dealkylation by the released fluoride (Scheme 2c, inset table). Trialkylphosphites reacted to provide a variety of methyl (**78**), ethyl (**80, 84**, **85**), and isopropyl (**79**) oxetane-phosphonates in good yields.^38^ Phosphonites were well tolerated and afforded the corresponding phosphinates in good yields (**81**, **82**). Bulky methoxydiphenylphosphine provided phosphine oxide (**83**). Secondary phosphine oxides were found to react through the oxygen rather than the phosphorus, confirmed by the small-molecule X-ray crystal structure of **87**. In solution, secondary phosphine oxides exist in equilibrium with their phosphinous acid form, which is presumably trapped through the oxygen atom preferentially by the oxetane carbocation, with oxidation to provide the more stable P(V) products observed.

The new oxetane-containing motifs were amenable to further derivatization (Scheme 2d). Oxetane azide **59** underwent copper-catalyzed azide–alkyne cycloaddition (CuAAC) to afford triazole **88**, expanding the potential scope of oxetane 1,2,3-triazoles available. The bromide handle of *N*-oxetane-sulfoximine **73** was further functionalized through palladium-catalyzed Sonogashira and Buchwald–Hartwig cross-coupling transformations in good yields. OTIPS OSF **2** reacted efficiently with 2-methyl benzimidazole with concomitant TIPS deprotection to afford oxetane-benzimidazole **91** in a high yield. The phenol was converted to an aryl chloride through triflation and palladium-catalyzed chlorination to generate an oxetane analog of chlormidazole (**92**), a marketed antifungal treatment.

### Unlocking SuFEx Reactivity

**Scheme 3:**
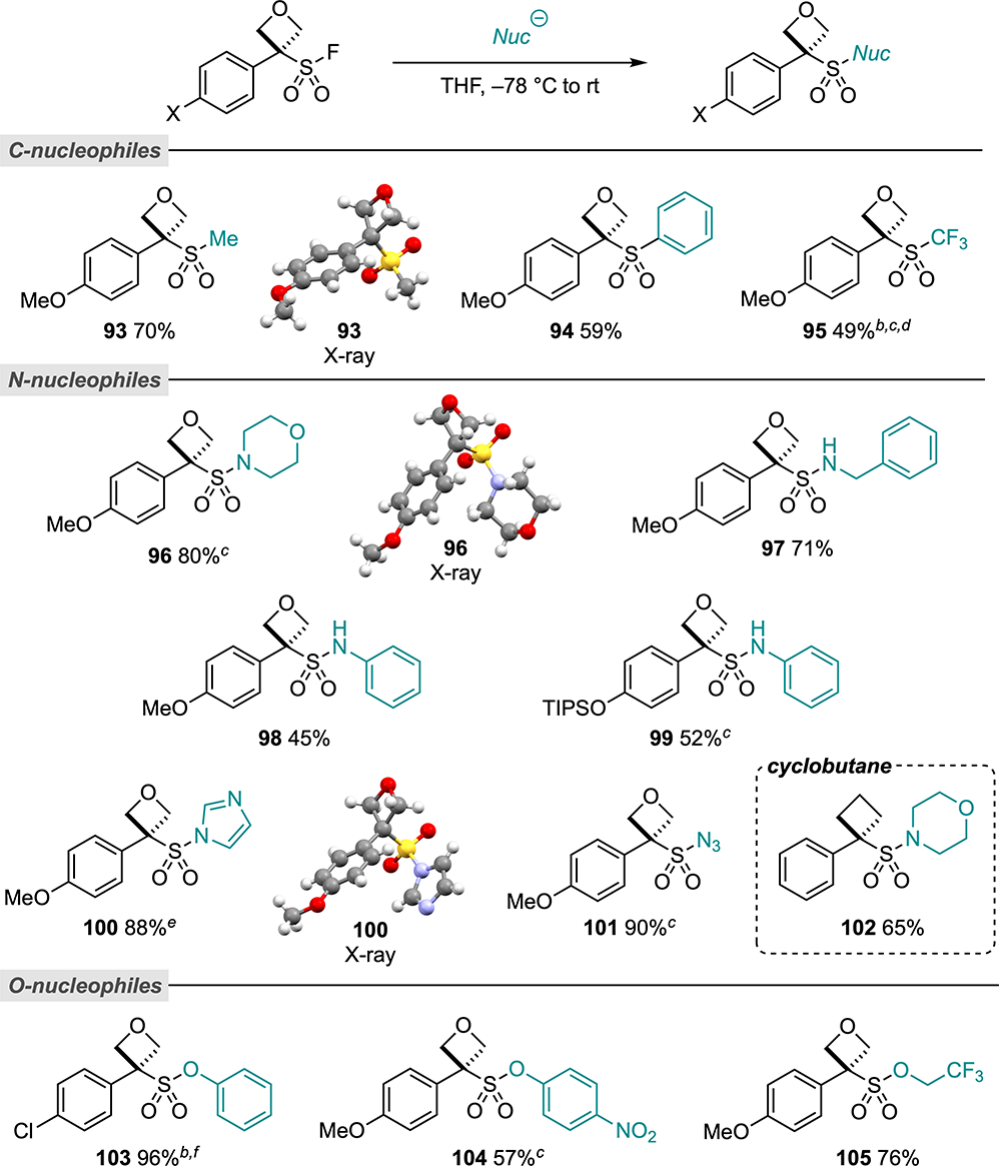
OSF SuFEx Scope Reactions performed on a 0.1 mmol scale unless otherwise specified. For full details of the reaction conditions, see the Supporting Information. Reaction performed in acetonitrile (0.3 M). Reaction performed on a 0.2 mmol scale. Degradation observed over extended periods in MeCN-*d*_3_ or on silica gel. Unstable in CDCl_3_. Reaction performed on a 0.06 mmol scale.

Extrusion of the anionic SO_2_F group in the deFS process is dependent primarily on the solvent polarity and temperature. Polar solvents (methanol and acetonitrile) effectively stabilize the oxetane carbocation to the extent that a mild rise in temperature promotes the entropically driven loss of SO_2_. We envisaged that the application of harder nucleophiles, with less polar solvents such as THF, would increase the rate of the SuFEx pathway over that of the deFS. Organolithium reagents MeLi and PhLi were applied in THF at −78 °C, with slow warming to 0 °C to cleanly afford the corresponding oxetane sulfones **93** and **94** (Scheme 3).

Interestingly, TMSCF_3_ reacted rapidly through the SuFEx pathway to afford the CF_3_-oxetanesulfone (**95**) even in acetonitrile at 60 °C. Low-molecular-weight sulfones such as **93** and **95** would have been challenging to obtain by the thiol alkylation from oxetanols since it would require gaseous thiol nucleophiles (e.g., MeSH). Oxetane sulfonamide motifs were accessed through the addition of lithium amide nucleophiles (amine + *n*BuLi) to a solution of OSF in THF at −78 °C to room temperature, producing another previously unknown substitution pattern on oxetanes. Good yields were obtained with secondary amines, benzylamine, and anilines (**96**, **97**, **98**). Morpholine sulfonamide **96** was characterized by X-ray crystallography. OTIPS OSF **2** reacted without the deprotection of the silyl group (**99**). The reaction with the anion of imidazole, formed with NaH, gave the sulfonyl imidazole, which could be isolated and characterized, including by X-ray crystallography. Sulfonyl imidazole **100** exhibited poor acidic stability (decomposition observed in CDCl_3_) with the extrusion of SO_2_.^39^ Treatment with HCl led to the formation of oxetanol and oxetane chloride through trapping of the oxetane carbocation by residual water or Cl anions.

Unlike the reaction with TMSN_3_, which gave oxetane azide **59** above, the reaction with the harder nucleophile NaN_3_ gave sulfonyl azide **101** in good yields. Cyclobutane-sulfonyl fluoride **16** also underwent SuFEx with deprotonated amine nucleophiles (**102**), and exhibited comparable reactivity to its OSF counterpart (see SI for comparison). Phenolates (NaH deprotonation) or phenols with Cs_2_CO_3_ generated sulfonate esters in good yields (**103**, **104**). Similarly, sodium trifluoroethanoate underwent SuFEx (**105**). As such, we envisage that a broad range of SuFEx chemistry is applicable to the OSF reagents when the nucleophiles are sufficiently reactive at room temperature.

### Azetidine Sulfonyl Fluorides (ASFs)

**Scheme 4:**
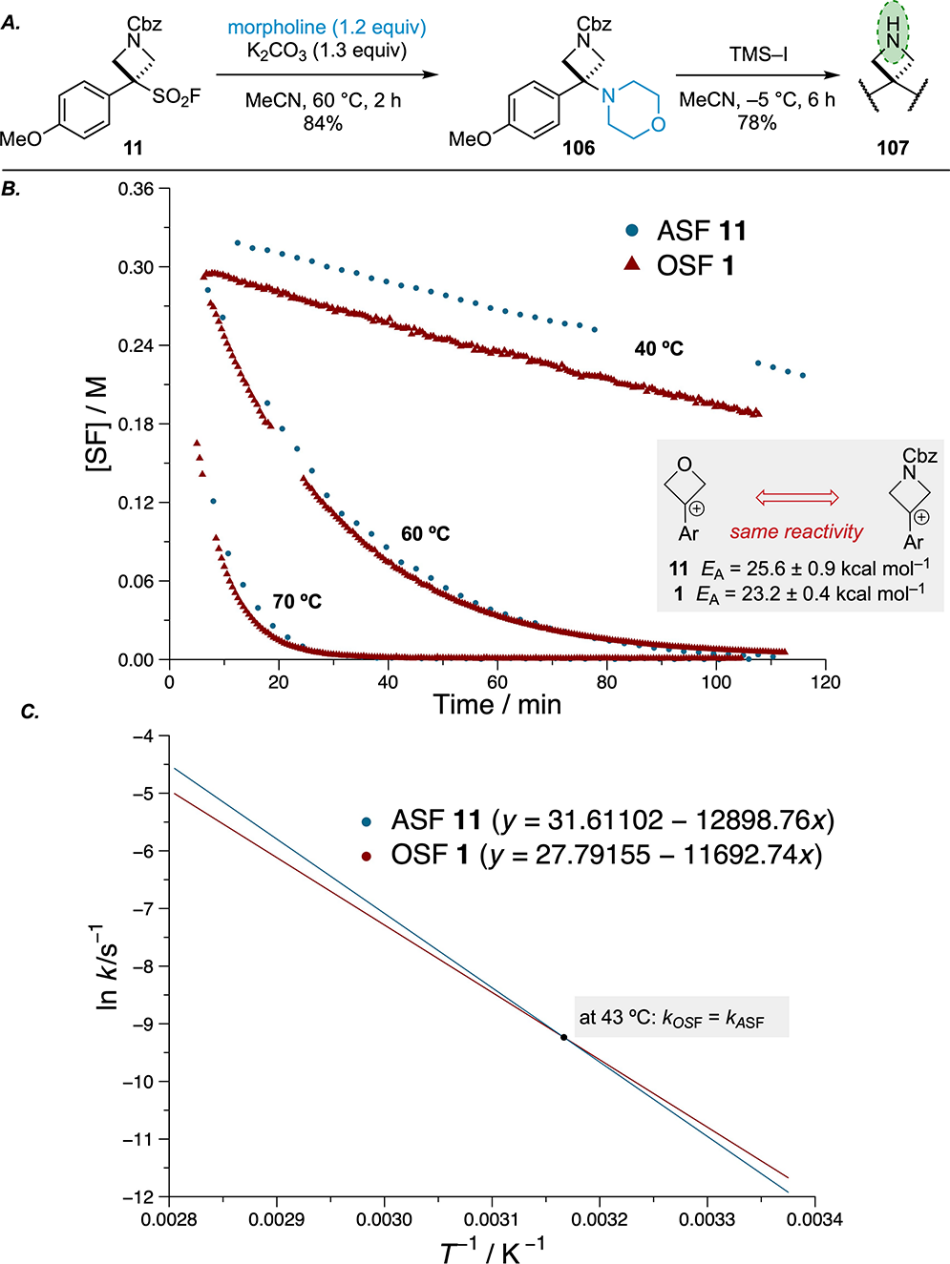
(A) deFS Reaction of PMP(Cbz)ASF 11, (B) Kinetic Profiles at Different Temperatures, and (C) Arrhenius Plots.^,^ Kinetic data for PMP OSF **1** taken from ref (20). Profiles at 40 °C do not overlap due to discrepancies in the absolute amounts of internal standard/PMP(Cbz)ASF **11**, but the rates of consumption are evidently very similar, seen by the parallel lines.

For the first time, we report the reactivity of ASFs as reagents for the mild synthesis of 3-aryl-3-substituted azetidines. Reacting PMP(Cbz)ASF **11** with morpholine in acetonitrile in the presence of triethylamine or K_2_CO_3_ effected the deFS reaction without any observable SuFEx reactivity (Scheme 4a). The Cbz group was readily removed by using TMSI to give NH azetidine-amine **107** in 78% yield, unveiling the additional vector for growth and further derivatization. The NH-amino-azetidines also represent potential isosteres of amidines with differences in conformation and subtle differences in p*K*_*a*_.^40^

The kinetics of the deFS process with PMP OSF (**1**) and PMP(Cbz)ASF (**11**) was examined by heating at 60 °C in MeCN-*d*_3_ in the presence of triethylamine and morpholine as a coupling partner. This study revealed a remarkably similar profile for the consumption of these reagents, with only a slightly larger activation energy for the deFS of the azetidine reagent (difference of 2.4 kcal mol^–1^; Scheme 4b).

The similar reactivity was also reflected in the comparable yield in the reaction of PMP OSF (**1**) and PMP(Cbz)ASF (**11**) with morpholine amino-oxetane (86%) vs amino-azetidine (**106**, 84%). Despite the small kinetic differences between PMP(Cbz)ASF **11** and PMP OSF **1**, the larger activation energy of PMP(Cbz)ASF **11**, coupled with an also larger Arrhenius pre-exponential factor, results in a situation where the rate of deFS of PMP(Cbz)ASF **11** is higher at higher temperatures and the rate of deFS of PMP OSF **1** is higher at lower temperatures (Scheme 4c). At 43 °C, the rates of deFS of both species appear to be equal (*k*_OSF_ = *k*_ASF_).

**Scheme 5:**
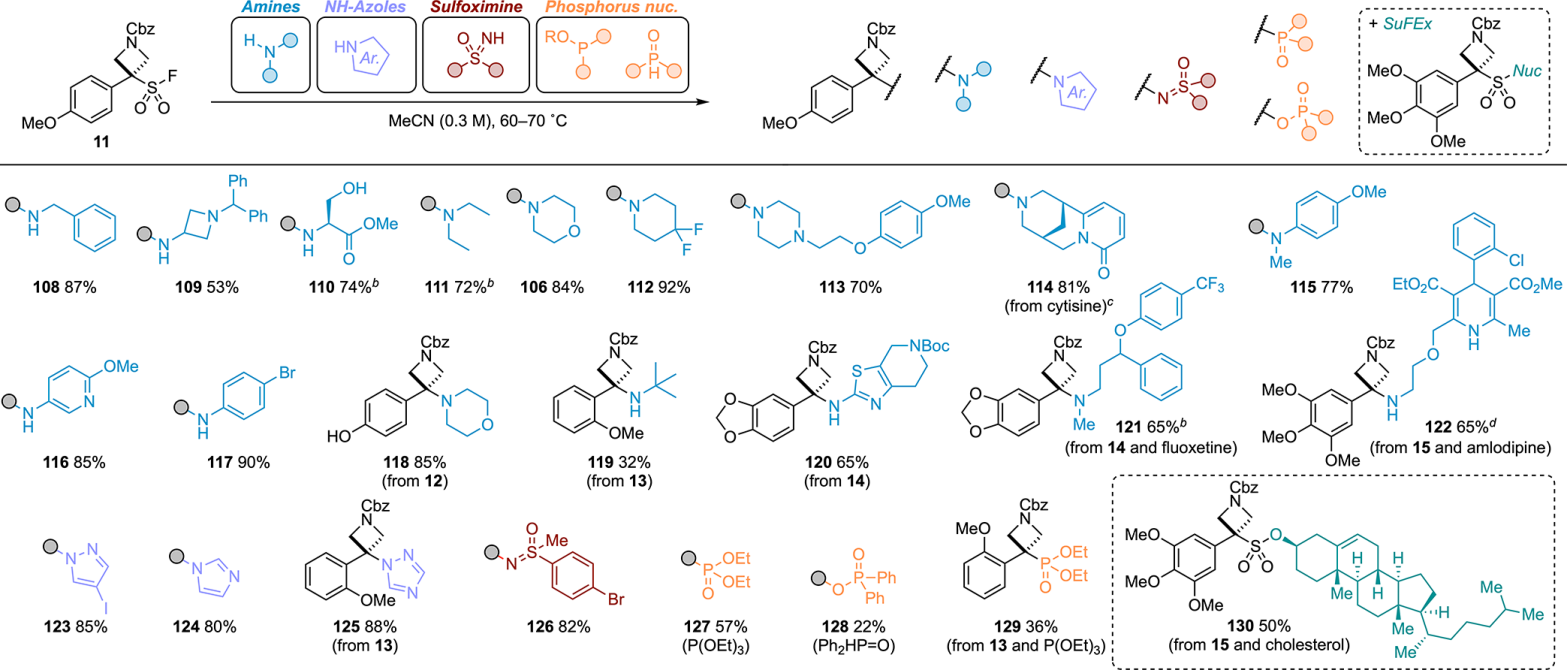
deFS and SuFEx Reaction of ASFs (11–15) with Different Nucleophiles. Conditions: Amines: ASF (1.0 equiv), amine (1.2 equiv), K_2_CO_3_ (1.3 equiv), acetonitrile (0.3 M), 60 °C, 2–5 h; NH-azoles: ASF (1.0 equiv), nucleophile (imidazole = 1.2 equiv; pyrazole = 1.2 equiv; triazole = 3.0 equiv), K_2_CO_3_ (1.3 equiv), acetonitrile (0.3 M), 60 °C, 2–5 h; sulfoximine: ASF (1.0 equiv), sulfoximine (3.0 equiv), K_2_CO_3_ (1.3 equiv), acetonitrile (0.3 M), 60 °C, 2 h; phosphorus nucleophiles: ASF (1.0 equiv), nucleophile (3.0 equiv), K_2_CO_3_ (3.0 equiv, only with phosphine oxide), acetonitrile (0.3 M), 60 °C, 2 h; SuFEx: ASF (1.0 equiv), alcohol (2.0 equiv), NaH (1.5 equiv), THF (0.3 M), 0 °C to rt, 21 h. Hydrochloride salt of fluoxetine and 2.6 equiv of K_2_CO_3_ used. Amine as limiting reagent and PMP(Cbz)ASF **11** in slight excess (1.2 equiv), performed on a 0.1 mmol scale. Benzenesulfonate salt of amlodipine and 2.6 equiv of K_2_CO_3_ used.

PMP(Cbz)ASF **11** was reacted with a diverse range of primary and secondary amines as well as anilines, providing amino-azetidines (Scheme 5). Like with OSFs, the functional group tolerance was broad, and sensitive functionalities such as free alcohols, tertiary amines, esters, and pyridines were well tolerated as well as complex late-stage examples, providing azetidine functionalized fluoxetine (**121**) and amlodipine (**122**). NH-Azoles, sulfoximines,^41^ and phosphorus-based nucleophiles were also successful (**123**–**129**) providing a series of novel motifs. Azetidine sulfonyl fluorides were amenable to SuFEx reactivity using alkoxides, shown here with cholesterol, demonstrating the potential for conjugation with complex molecules (**130**).

### Divergent Access to OSF and ASF Reagents and Linkers

**Scheme 6:**
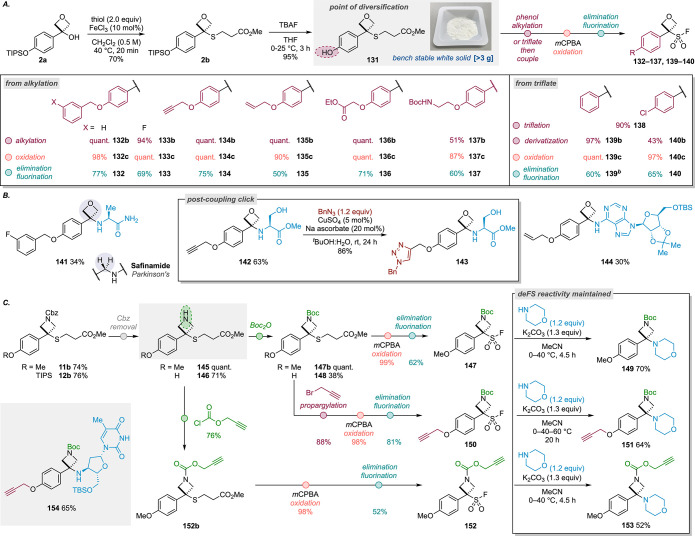
Divergent Synthesis of Oxetane and Azetidine Sulfonyl Fluorides. For full details of the reaction conditions, see the Supporting Information. Preparation of **139** was previously reported in ref (20).

To enable divergent access to the aryl OSF reagents for the deFS or SuFEx processes, we targeted phenol sulfide **131** as a strategic point for diversification (Scheme 6a). To access this material on a multi gram scale, we initially redeveloped the thiol alkylation. Employing an FeCl_3_ catalyst (10 mol %) generated sulfide **2b** in good yield and was scaled up to 14 mmol/6.0 g (see SI for further discussion). The TIPS group was readily removed to generate the diversifiable intermediate **131** as a bench stable white solid, which was stable to storage at room temperature for >6 months. From here, the phenol was effectively alkylated with benzyl bromide in quantitative yield by using K_2_CO_3_ in acetone. The oxidation–elimination/fluorination process afforded benzyl OSF **132**. Applying 3-fluorobenzyl bromide gave the corresponding OSF reagent (**133**), which was reacted with *H*-Ala-NH_2_ hydrochloride to generate an oxetane analog of the drug safinamide, which contains a benzylamine (**141**, Scheme 6b, see SI for X-ray crystal structure).

Given the potential to react with complex and functional biomolecules under mild conditions, we next reacted phenol **131** with alkyl halides to install propargylic, allyl, ester, and amine functionality to enable potential application as linkers, generating OSF reagents **134**–**137** (Scheme 6a). Allyl and ethyl ester OSFs (**135**, **136**) were further characterized by X-ray crystallography (see SI for the structures). Alternatively, the phenol was treated with Tf_2_O to afford aryl triflate **138**, which was applied to palladium-catalyzed deoxygenation and chlorination reactions to generate OSFs **139** and **140**.^42^ Propargylic OSF **134** readily reacted with serine methyl ester hydrochloride to provide amino-oxetane **142** in 63% yield (Scheme 6b). The propargylic ether was then demonstrated to undergo CuAAC under standard conditions (**143**). Allyl OSF **135** reacted similarly through a deFS pathway with an adenosine derivative (**144**).

A similar approach was applied to vary the ASF reagents and to modify the N-group. Removal of the Cbz was achieved using TMSI to provide the free NH azetidine in quantitative yield with PMP derivative **145**. Using the OTIPS derivative gave phenol **146**. In both cases, the azetidine nitrogen could be selectively functionalized with Boc anhydride. PMP derivative **145** was converted to PMP(Boc)ASF **147**, and pleasingly, the deFS reactivity of this ASF was retained, confirmed with efficient coupling with morpholine (**149**). Having proven that the Cbz protecting group, which was crucial in our catalytic methods,^8^ was not required for deFS reactivity, it greatly widens the potential for differently N-functionalized ASFs. Curiously, PMP(Boc)ASF **147** required lower reaction temperatures, from 0 °C and slowly warming to 40 °C to avoid unproductive formation of the arylazetidin-3-ol and azetidine fluoride.

Our attention turned to the incorporation of additional linking groups and click handles to allow potential applications as di- and trifunctional linking reagents considering the azetidine N-vector. From phenol **148** alkylation with propargyl bromide followed by conversion to the ASF gave trifunctional reagent **150**. This reagent performed well in the deFS reaction with morpholine (**151**) as well as with a derivative of thymidine (**154**). Next, we targeted the installation of a click handle through the azetidine nitrogen atom. The reaction of NH azetidine **145** with propargyl chloroformate formed the propargyl carbamate, which was converted to ASF **152**. The deFS reactivity was similarly maintained in the reaction with morpholine to generate an amino-azetidine bearing additional N-functionality (**153**).

**Scheme 7:**
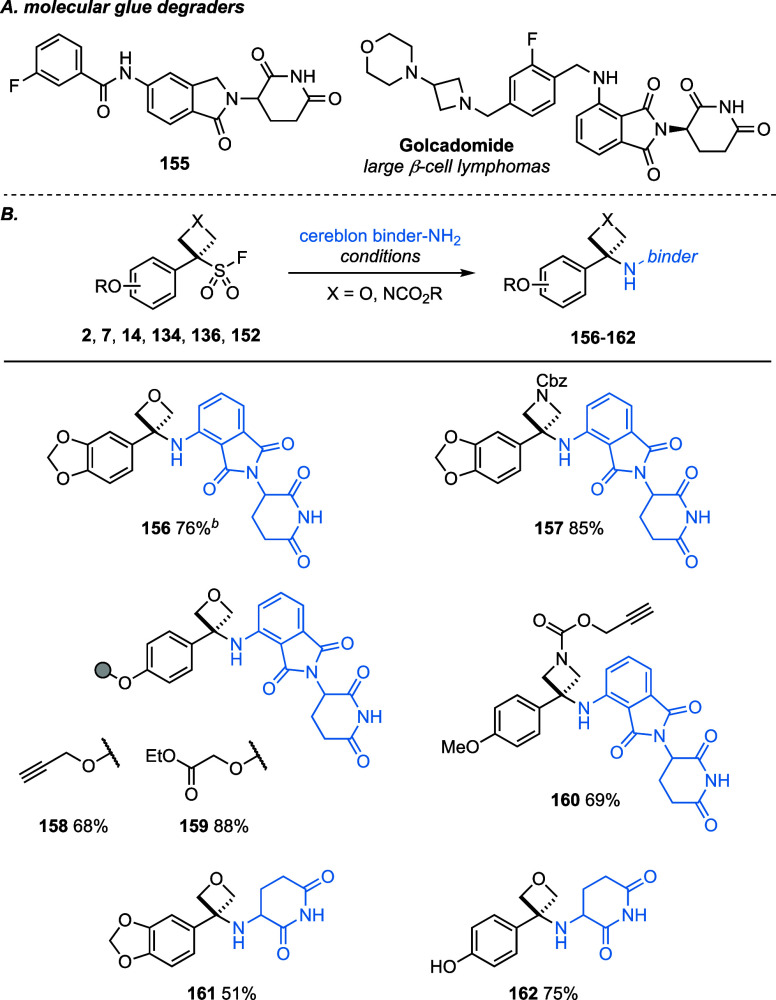
(A) Emergent Molecular Glue Degraders and (B) Multifunctional OSFs and ASFs as Potential PROTAC Linkers and Molecular Glue Precursors Reactions performed on a 0.1 mmol scale unless otherwise specified. For full details of the reaction conditions, see the Supporting Information. Reaction performed on 0.2 mmol scale.

Targeted protein degradation is a burgeoning field in drug discovery, involving the use of bifunctional small molecules to promote ubiquitination of a protein of interest by an E3 ligase and so initiate subsequent proteasomal degradation.^43^ The glutarimide scaffold is effective in binding the cereblon E3 ligase and is found in the most clinically successful degraders, thalomid (thalidomide), revlimid (lenalidomide), and pomalyst (pomalidomide). Proteolysis-targeting chimeras (PROTACs) offer exciting potential as new modalities in drug discovery and typically consist of an inhibitor, a linker, and an E3 ligase binder. Linker design is itself crucial both to efficacy and physicochemical properties of the PROTAC compounds.^44^ We envisaged that the use of functionalized OSF and ASF reagents would allow the introduction of cereblon-binding motifs (CBMs) and provide new linker designs that may offer potentially advantageous physicochemical or solubility properties. The inclusion of oxetanes or azetidine derivatives would also provide notably different conformations in comparison to amide, ether, or amine links, and hence how the binding elements are displayed.^45^ Moreover, the direct modification of lenalidomide or pomalidomide as small-molecule derivatives can have a significant effect on degradation potency and profiles, offering exciting new opportunities for drug discovery as molecular glues (e.g., **155**, Scheme 7a).^46^ Golcadomide is a pomalidomide-derived molecular glue degrader from Calgene/Bristol-Myers Squibb currently entering phase III clinical trials for large β-cell lymphoma (NCT06356129).^47^ Hence, there is significant interest in the generation of new cereblon-binding derivatives.

We first examined the reaction of benzodioxole OSF **7** and (Cbz)ASF **14** reagents to form pomalidomide derivatives (Scheme 7b). Pomalidomide is commonly employed in degrader design but suffers from limited synthetic derivatization as a nucleophile due to the electron-poor aniline and its low solubility in common organic solvents.^48^ Performing reactions under more dilute conditions (0.05 M acetonitrile) with slightly elevated temperature (80 °C) and prestirring facilitated greater dissolution in the reaction media. Subsequent addition of excess OSF or ASF led to effective formation of the desired amino-oxetane and azetidine in excellent yields (**156**, **157**).^49^ Using OSF reagents **134** and **136** functionalized with further reactive handles, pomalidomide-oxetanes **158** and **159** were generated, whereby the propargylic and ethyl ester tails provide the opportunity to attach an inhibitor for a protein of interest. Pomalidomide-azetidine **160** was cleanly afforded from ASF **152**, offering the potential for further functionalization through the alkyne handle. The exploration of novel, glutarimide-bearing CBMs is providing new design options for molecular glues and PROTACs.^50^ Commercially available and inexpensive 2-aminoglutarimide HCl salt reacted directly with OSFs 7 and 2, providing **161** and **162** with the necessary cereblon-binding motif.

## Conclusions

In conclusion, we have extensively demonstrated the powerful potential of oxetane sulfonyl fluorides (OSFs) and azetidine sulfonyl fluorides (ASFs) to access a broad chemical space of significant medicinal relevance. Divergent routes toward OSF and ASF reagents have been developed. Eleven oxetane analogues of marketed drugs and bioactive compounds have been synthesized using these reagents to demonstrate their synthetic utility in a drug discovery context. OSFs and ASFs have been shown to be compatible with a broad range of nucleophile types, including primary and secondary amines, anilines, NH-azoles, sulfoximines, sulfonimidamides, and phosphorus reagents, to enable merging of these valuable pharmacophores. The SuFEx reaction pathway of the OSFs can now be fully exploited to access novel oxetano-S(VI) motifs. These novel motifs provide new design options for medicinal chemistry that may find use as valuable novel bioisosteres or replacement groups for medicinal chemists. We propose these oxetane and azetidine structures as much broader, valuable chemical motifs beyond bioisosteres. These novel combinations of polar functional groups are likely to confer favorable properties in their own right and access new chemical and intellectual property space. Moreover, the OSF and ASF reagents readily react with functional molecules, enabling possible applications in the linkerology of PROTAC degraders or in molecular glues. We expect this work to highlight the value of these reagents in discovery for facile diversification and to offer new opportunities in drug discovery.

## Data Availability

The data underlying this study are available in the published article and its Supporting Information. Raw and processed characterization data for all novel compounds can be found at the Imperial College London Research Data Repository: 10.14469/hpc/14851.

## Supporting Information Available

Experimental details and procedures, characterization data, X-ray crystallography data and further discussion (PDF)
^1^H, ^13^C, ^19^F, ^31^P, and ^11^B NMR spectra for selected compounds (PDF)

## Notes

A version of this manuscript was deposited on the preprint repository ChemRxiv.^51^
