# Supplementary material for: Harnessing Oxetane and Azetidine Sulfonyl Fluorides for Opportunities in Drug Discovery
Source: J Am Chem Soc. 2024 Dec 12;146(51):35377–89. doi: 10.1021/jacs.4c14164 (PMC11673132; doi:10.1021/jacs.4c14164)
Supplement: Supplementary file 1 — ja4c14164_si_001.pdf [file ja4c14164_si_001.pdf]

SUPPORTING INFORMATION

Harnessing Oxetane and Azetidine Sulfonyl Fluorides for Opportunities in Drug Discovery

Oliver L. Symes,[1] Hikaru Ishikura,[1] Callum S. Begg,[1] Juan J. Rojas,[1] Harry A. Speller,[1] Anson M. Cherk,[1] Marco Fang,[1] Domingo Leung,[1] Rosemary A. Croft,[1] Joe I. Higham,[1] Kaiyun Huang,[1] Peter Haycock,[1] Andrew J. P. White,[1] Anna Barnard,[1] Chulho Choi[2] and James A. Bull[1]*

[1] Department of Chemistry, Imperial College London, Molecular Sciences Research Hub, White City Campus, Wood Lane, London W12 0BZ, UK.

[2] Medicine Design, Pfizer Research & Development, Eastern Point Rd., Groton, CT 06340, USA.

*e-mail: j.bull@imperial.ac.uk

General Experimental Conditions 4
Structures of Additional Compounds in SI 7
Additional reaction scope and unsuccessful substrates 8
Synthesis route to oxetane sulfonyl fluorides (OSFs) from oxetanone **1a** 9
  Oxetane thiol alkylation (TA) procedures 9
  Oxetane sulfinate elimination (OSE) procedures 10
Divergent route to oxetane sulfonyl fluorides from oxetane phenol sulfide **131** 11
Synthesis route to azetidine sulfonyl fluorides (ASFs) from N-Cbz-azetidinone 12
  Azetidine sulfinate elimination (ASE) procedures 12
Synthesis, handling and storage considerations of sulfonyl fluorides 12
Optimization of thiol alkylation with oxetanols 14
Optimization of deFS couplings between PMP OSF **1** and different nucleophile types 17
Optimization of SuFEx couplings between PMP OSF **1** and different nucleophile types 22
  SuFEx with amines 22
  SuFEx with NH-azoles (imidazole) 25
  SuFEx with NaN_3 26
  SuFEx comparison between cyclobutane sulfonyl fluoride **16** and OSF **139** 26
Defluorosulfonylative (deFS) coupling General Procedures 27
Kinetic analysis of PMP Azetidine Sulfonyl Fluoride ASF **11** 28
  Quantitative Reaction Monitoring by ^{1}H NMR 28
  Reaction profile of ASF **11** with morpholine 29
  Variable Time Normalization Analysis (VTNA) 30
  Arrhenius Plot and Determination of E_A and Half-Lives 31
  Comparison between the kinetics of ASF **11** and OSF **1** 34
X-Ray Crystallography Details 36
  Comments on Crystal Structures 40
  ORTEP Renders of Crystal Structures 41
  Crystal Structure Measurements 52
Experimental Details and Characterization Data 56
  Oxetane Sulfonyl Fluorides (**1–10**, **132–140**) 56
  Azetidine Sulfonyl Fluorides (**11–15**, **147**, **150**, **152**) 76
  Cyclobutane Sulfonyl Fluorides (**16** and **17**) 91
  Oxetane analogues of bioactive molecules (**18–24**, **34**, **36**, **38**, **92**, **141**) 95
  Post-deFS functionalization from phenol **25** (**26–32**) 106
  Oxetane deFS with NH-azole nucleophiles (**39–63**) 110
  Oxetane deFS with S=NH nucleophiles (**64–77**) 124
  Oxetane deFS with phosphorous nucleophiles (**78–87**) 130

Oxetane deFS with amines (**142**, **S23**, **144**, **S24**, **S26–28**) 134
Further derivatization of oxetane deFS products (**88–90**, **143**) 137
Oxetane SuFEx products (**93–105**, **S29–32**) 140
Azetidine deFS products (**106–129**, **149**, **151**, **153**) 147
Azetidine SuFEx product (**130**) 159
Linker and Molecular Glue-type deFS products (**156–162**) 161
References 165
^{1}H, ^{13}C, ^{19}F, ^{31}P and ^{11}B NMR Spectra for Selected Compounds 168

General Experimental Conditions

All non-aqueous reactions were carried out under an inert atmosphere (argon) with flame-dried glassware using standard techniques, unless otherwise specified. Anhydrous solvents were obtained by filtration through drying columns (CH_2Cl_2, THF) or used as supplied (MeCN, MeOH). Reactions in sealed tubes were run using Biotage microwave vials (0.2–0.5 mL, 2–5 mL) and aluminum caps with molded butyl septa.

Filtration was performed using Celite® Hyflo Supercel from Thermo Scientific. Flash chromatography was performed using 230–400 mesh silica from Sigma-Aldrich, with the indicated solvent system according to standard techniques. Automated flash column chromatography was performed using a Biotage® Selekt system. Purification traces are reported with the following information: column type; flow rate; sample mass; solvent A (least polar); solvent B (more polar); solvent C (most polar); UV wavelength detection and peak identities. Preparative thin layer chromatography (PTCL) was performed on Analtech UNIPLATE™ precoated, glass-backed silica gel plates (20 × 20 cm, 1500 microns) with the indicated solvent system. Analytical thin-layer chromatography (TLC) was performed on precoated glass-backed silica gel plates. Visualization of the developed chromatogram was performed by UV absorbance (254 nm) and stained with a ninhydrin, *p*-anisaldehyde, cerium ammonium molybdate (CAM) or phosphomolybdic acid solution in ethanol, followed by heating.

Infrared spectra (n_{max}, FTIR ATR) were obtained using an Agilent Technologies Cary 630 FTIR or a Perkin Elmer Spectrum 100 FTIR Spectrometer and recorded in reciprocal centimeters (cm^{-1}) (br = broad, w = weak, st = stretch, as = asymmetric, sy = symmetric).

Nuclear magnetic resonance spectra were recorded on either 400 or 500 MHz Bruker AvIII HD spectrometers with a SampleXpress automatic sample changer. The frequency used to record the NMR spectra is given in each assignment and spectrum (^{1}H NMR at 400 or 500 MHz; ^{13}C NMR at 101 MHz or 126 MHz; ^{19}F NMR at 377 MHz). Chemical shifts for ^{1}H NMR spectra are recorded in parts per million (ppm) from tetramethylsilane with the residual protic solvent resonance as the internal standard ($CHCl_3$: δ = 7.27 ppm, DMSO: δ = 2.50 ppm, CH_3OD: δ = 3.31 ppm, H_2O: δ = 4.79 ppm, CH_3CN: δ = 1.94 ppm, $(CH_3)_2CO$: δ = 2.05 ppm). Data is reported as follows: chemical shift (multiplicity [s = singlet, d = doublet, t = triplet, q = quartet, p = pentet, hept = heptet, m = multiplet and br = broad], coupling constant (in Hz), integration of equivalent nuclei and assignment). ^{13}C NMR spectra were recorded with complete proton decoupling. Chemical shifts are reported in parts per million (ppm) from tetramethylsilane with the solvent resonance as the internal standard ($^{13}CDCl_3$: δ = 77.0 ppm, $(^{13}CD_3)_2SO$: δ = 39.5 ppm, $^{13}CD_3OD$: δ = 49.0 ppm, $(^{13}CD_3)_2CO$: δ = 29.8 ppm). *J* values are reported in Hz. ^{19}F and ^{31}P NMR spectra were recorded with or without complete proton decoupling. Decoupling is indicated as $^{19}F\{^1H\}$ and $^{31}P\{^1H\}$ and where relevant this is stated in each assignment. ^{19}F, ^{31}P and ^{11}B NMR spectra are indirectly referenced to $CFCl_3$, H_3PO_4 and BF_3•OEt_2 automatically by direct measurement of the absolute frequency of the deuterium lock signal by the spectrometer hardware. All spectra were measured at 25 °C unless otherwise specified.

Assignments of ^{1}H and ^{13}C spectra were based upon the analysis of δ and *J* values, by analogy of previous examples, as well as DEPT, COSY, HSQC and HMBC experiments where appropriate.

NMR spectra are displayed as follows unless this would obscure signals: ^{1}H NMR spectra are displayed between 10.0 ppm and 0.0 ppm; ^{13}C NMR spectra are displayed between 210 ppm and 0 ppm; ^{19}F and ^{31}P NMR spectra are displayed for the full sweep width as acquired.

Melting points were obtained using an Optimelt MPA100 melting point apparatus and are uncorrected.

High resolution mass spectrometry (HRMS) analyses were performed through the Imperial College or EPSRC mass spectrometry service. HRMS analyses at Imperial College were performed using an electrospray ion source (ESI), nanospray ionization (NSI), electron impact ionization (EI) or

atmospheric pressure chemical ionization (APCI) using an atmospheric solids analysis probe (ASAP). ESI was performed using either: a Waters LCT Premier (ES-ToF) equipped with an ESI source operated in positive or negative ion mode; or a Waters UPLC with TUV and Thermo Q Exactive equipped with ESI source operated in positive mode. APCI was performed using a Thermo Scientific Q-Extractive/Dionex Ultimate 3000 using an ASAP to insert samples into the APCI source operated in positive or negative mode. The sample was introduced at ambient temperature and the temperature increased until the sample vaporized. HRMS analyses at the EPSRC UK National Mass Spectrometry Facility (NMSF) were performed using a nano-electrospray ion source (NSI), chemical ionization (CI) or atmospheric pressure chemical ionization (APCI) using an atmospheric solids analysis probe (ASAP). NSI was performed using a Thermo Scientific LTQ Orbitrap XL operated in positive or negative ion mode. CI was performed using a Finnigan MAT 95 XP operated in positive mode. APCI was performed using a Waters Xevo G2-S using an ASAP to insert samples into the APCI source operated in positive mode. The sample was introduced at ambient temperature and the temperature increased until the sample vaporized. The software used was either MassLynx 4.1 or Bruker Daltonics DataAnalysis 4.0. Please note: The MassLynx 4.1 software, used at the Imperial College mass spectrometry service, does not account for the electron and all the calibrations/references are calculated accordingly, i.e. $[M+H]^+$ is detected and the mass is calibrated to output [M+H]. In the cases where this software is used, we report the HRMS as [M+H].

Observed optical rotations (α') were measured at the indicated temperature (*T* °C) and converted to the corresponding specific rotations $[\alpha]_D^T$ in deg cm^2 g^{-1}, concentration (c) in g per 100 mL.

Where volumes have been quoted with respect to recrystallization, this refers to the amount of solvent used per unit mass crude material, with 1 volume equating to 1 mL per 1 g of crude product (i.e. 3 mL of solvent for 0.5 g crude material would equate to 6 volumes).

Reagents

Where the synthesis of a reagent is not stated, the reagent was commercially available. Commercial reagents were used as supplied or purified by standard techniques where necessary. All volatile amines were distilled over $MgSO_4$ or KOH pellets before use.

- The exact concentration of *n*-BuLi (1.6 M in hexanes, purchased from Sigma-Aldrich, CAS: 109-72-8), methyllithium (1.6 M in Et_2O, purchased from Sigma-Aldrich, CAS: 60-29-7), phenyllithium (1.9 M in dibutylether, purchased from Sigma-Aldrich, CAS: 591-51-5) and ^{i}PrMgCl•LiCl (1.3 M in THF, purchased from Sigma-Aldrich, CAS: 745038-86-2) was determined by titration with salicylaldehyde phenylhydrazone as indicator before each reaction using a literature procedure.[1] The average of three titrations was taken.
- Iron (III) chloride (reagent grade, 97%, CAS: 7705-08-0) was purchased from Sigma-Aldrich and stored under an argon atmosphere in a desiccator.
- In some cases, *m*CPBA was pre-washed before use. Procedure: a solution of commercial *m*CPBA (15 g, purity (≤77%, purchased from Sigma-Aldrich) in CH_2Cl_2 (300 mL) was washed with a phosphate buffer (pH = 7.5, 3 × 300 mL), then dried over Na_2SO_4, filtered and concentrated *in vacuo* using a rotatory evaporator to obtain pure *m*CPBA (free of benzoic acid and water). Special attention should be paid when handling pure *m*CPBA since it is shock-sensitive and potentially explosive. Do not heat on rotatory evaporators above 25 °C, do not scratch with a metal spatula when removing from round bottom flask (use for example a plastic spatula) and store at <4 °C. The washing of *m*CPBA is not a requirement, but can sometimes avoid the need for a purification step after the reaction work-up.

- Anhydrous potassium carbonate (≥98%; powder, –325 mesh, CAS: 584-08-7) was purchased from Sigma-Aldrich. In all cases K_2CO_3 was flame-dried as part of the reaction set-up.
- Sodium methoxide (pure, 5.4 M (30 wt%) solution in MeOH, CAS: 124-41-4, 67-56-1) was purchased from Acros Organics and stored under an argon atmosphere.
- Selectfluor™ (CAS: 140681-55-6) was purchased from Fluorochem and stored at <4 °C.
- **O**xetane **S**ulfonyl **F**luorides are abbreviated as OSF.
- **A**zetidine **S**ulfonyl **F**luorides are abbreviated as ASF.

Structures of Additional Compounds in SI

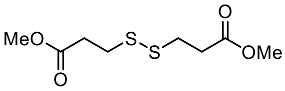

S1

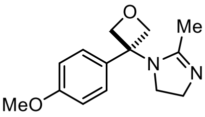

S2

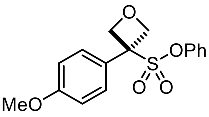

S3

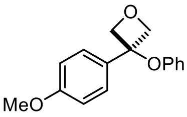

S4

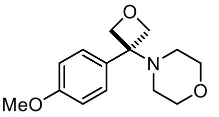

S5

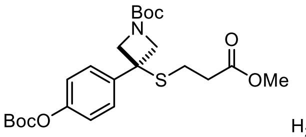

S6

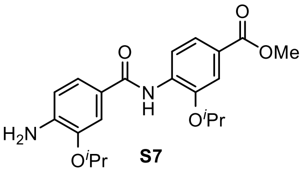

S7

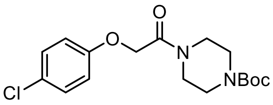

S8

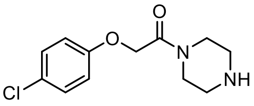

S9

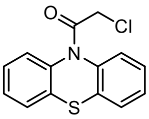

S10

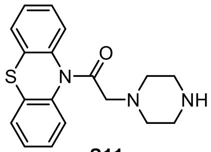

S11

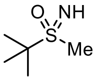

S12

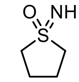

S13

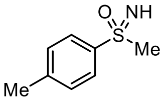

S14

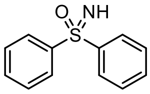

S15

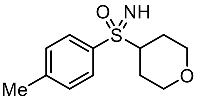

S16

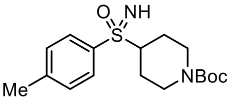

S17

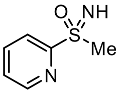

S18

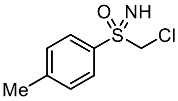

S19

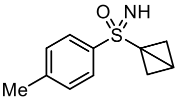

S20

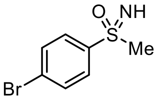

S21

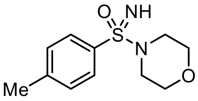

S22

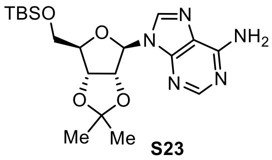

S23

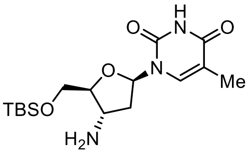

S24

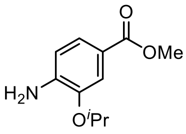

S25

Sulfoximimes and sulfonimidamide (**S12–S22**) were synthesized using methods previously reported in the literature.[2]

Additional reaction scope and unsuccessful substrates

Additional amino-oxetanes were prepared from OSFs **1**, **5**, and **7**. Additional oxetano-S(VI) systems were also generated from OSF **1** and cyclobutane sulfonyl fluoride **16** (Scheme **S1**).

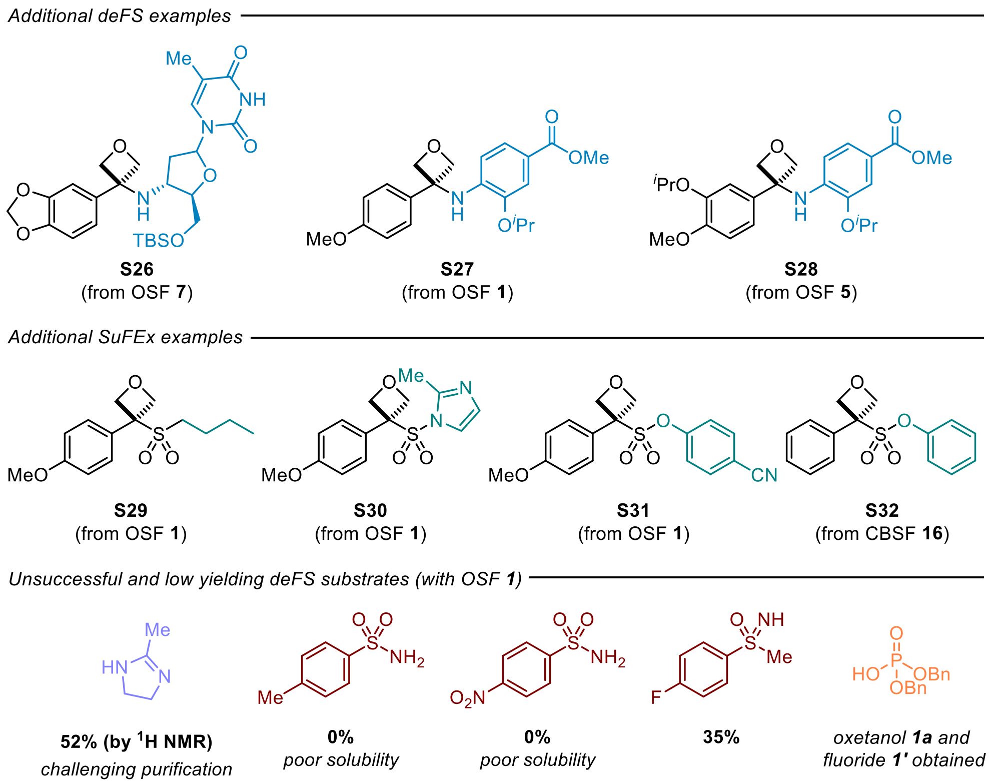

Scheme S1 Additional scope of amino-oxetane products and unsuccessful coupling partners.

Synthesis route to oxetane sulfonyl fluorides (OSFs) from oxetanone 1a

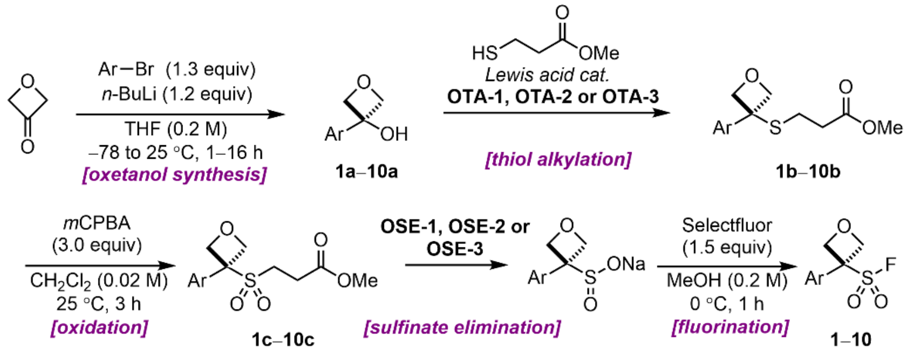

thiol alkylation (TA) conditions

reference	conditions
**OTA-1**	thiol (2.0 equiv), $FeCl_3$ (10 mol%), CH_2Cl_2 (0.5 M), 40 °C, 25 min
**OTA-2**	thiol (1.5 equiv), $FeCl_3$ (10 mol%), $CHCl_3$ (0.5 M), 60 °C, 60 min
**OTA-3**	thiol (2.0 equiv), $Li(NTf_2)$ (11 mol%), $^{n}Bu_4PF_6$ (5.5 mol%), $CHCl_3$ (0.5 M), 40 °C, 25 min

oxetane sulfinate elimination (SE) conditions

reference	conditions
**OSE-1**	NaH (1.05 equiv), THF (0.2 M), 25 °C, 1 h<br>*then* anhydrous MeOH quench (0.2 mL per 0.1 mmol sulfone)
**OSE-2**	5 M NaOH in MeOH (1.05 equiv), CH_2Cl_2 (0.1 M), 0 °C, 15 min
**OSE-3**	5.4 M NaOMe in MeOH (1.0 equiv), THF (0.5 M), 25 °C, 20 min

Scheme S2 General synthesis route to OSFs and the different thiol alkylation (TA) and oxetane sulfinate elimination (OSE) conditions used.

Oxetane thiol alkylation (TA) procedures

- **OTA-1:** A flame-dried reaction vessel with a stir bar was charged with $FeCl_3$ (10 mol%). Anhydrous CH_2Cl_2 (0.5 M) was added, followed sequentially by methyl 3-mercaptopropionate (2.0 equiv) and oxetanol (1.0 equiv). The reaction flask under argon was then placed into a pre-heated oil bath at 40 °C and stirred for 20 or 25 min. It is recommended to quench the reaction no later than 25 min after placing the reaction vessel into the oil bath to maximize yield while reducing formation of undesired ring-opened products (see Scheme **S3**).

- **OTA-2:** A flame-dried reaction vessel with a stir bar was charged with $FeCl_3$ (10 mol%). Anhydrous $CHCl_3$ (0.5 M) was added, followed sequentially by methyl 3-mercaptopropionate (1.5 equiv) and oxetanol (1.0 equiv). The reaction flask under argon was then placed into a pre-heated oil bath at 60 °C and stirred for 60 min. It is recommended to quench the reaction no later than 60 min after placing the reaction vessel into the oil bath to maximize yield while reducing formation of undesired ring-opened products (see Scheme **S3**).

- **OTA-3:** Oxetanol (1.0 equiv) was dissolved in non-anhydrous $CHCl_3$ (0.5 M) and methyl 3-mercaptopropionate (2.0 equiv) was added. Then, $Li(NTf_2)$ (11 mol%) and $^{n}Bu_4PF_6$ (5.5 mol%) were added sequentially to the solution. The reaction flask under argon was then placed into a preheated oil bath at 40 °C. There is a sharp change in color (most often from colorless to colorful) that indicates the beginning of the reaction. If there was no change in color after stirring for 15 min at 40 °C, the reaction was heated up at a rate of approximately 5 °C every 10 min until there was a change in color. It is recommended to quench the reaction

exactly 25 min after placing the reaction vessel into the oil bath to maximize yield while reducing formation of undesired ring-opened products (see Scheme **S3**).

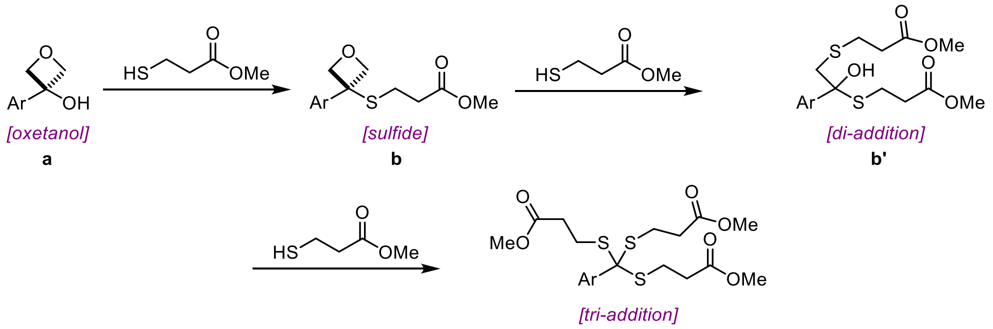

Scheme S3 Ring-opened side-products observed in the synthesis of oxetane sulfides.

Oxetane sulfinate elimination (OSE) procedures

- **OSE-1:** Oxetane sulfone (1.0 equiv) was added to a solution of NaH (60% dispersion in mineral oil, 1.05 equiv) in anhydrous THF (0.2 M) at 25 °C. The reaction was stirred at 25 °C for 1 h and then quenched with anhydrous MeOH (0.2 mL per 0.1 mmol of sulfone) and concentrated *in vacuo* using a rotary evaporator to afford the oxetane sulfinate salt.

- **OSE-2:** NaOH (5 M in MeOH, 1.05 equiv) was added to a solution of oxetane sulfone (1.0 equiv) in CH_2Cl_2 (0.1 M) at 0 °C. The reaction mixture was stirred at 0 °C for 15 min then filtered and washed with ice-cold hexane to afford oxetane sulfinate salt.

- **OSE-3:** NaOMe (5.4 M solution in MeOH, 1.0 equiv) was added dropwise to a solution of oxetane sulfone (1.0 equiv) in anhydrous THF (0.5 M) at 25 °C. The reaction mixture was stirred at 25 °C for 20 min, then concentrated *in vacuo* using a rotatory evaporator to afford the oxetane sulfinate salt.

Divergent route to oxetane sulfonyl fluorides from oxetane phenol sulfide 131

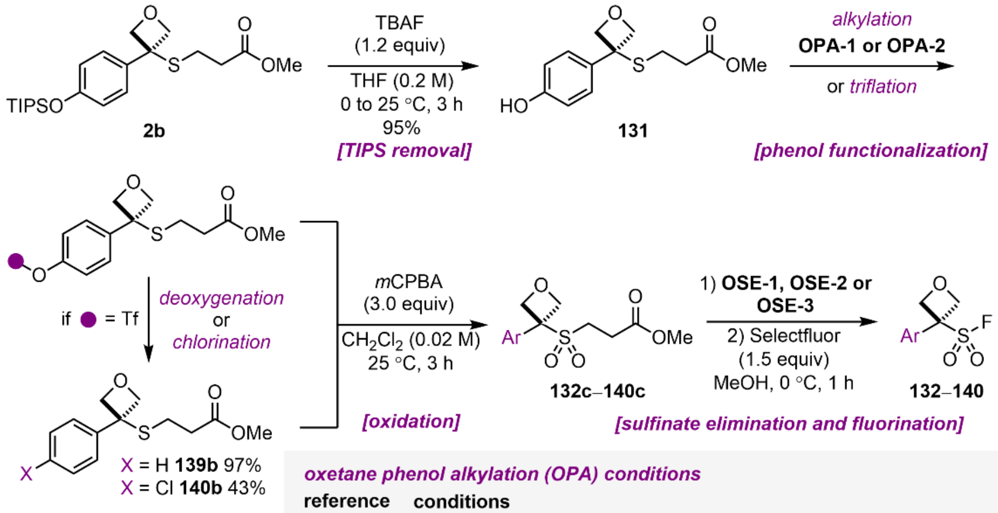

oxetane phenol alkylation (OPA) conditions

reference	conditions
**OPA-1**	alkyl halide (2.0 equiv), K_2CO_3 (3.0 equiv), acetone (0.2 M), 40 °C, 16 h
**OPA-2**	alkyl halide (2.0 equiv), K_2CO_3 (3.0 equiv), DMF (0.2 M), 60 °C, 16 h

triflation conditions
Tf_2O (1.2 equiv), pyridine (2.0 equiv)
CH_2Cl_2 (0.08 M), 0 to 25 °C, 1 h

deoxygenation conditions
HCOOH (2.0 equiv), Et_3N (3.0 equiv)
$Pd(OAc)_2$ (2 mol%), dppf (2 mol%)
DMF (0.1 M) 60 °C, 1 h

chlorination conditions
KCl (2.0 equiv), KF (0.5 equiv)
$Pd_2(dba)_3$ (1.5 mol%), ^{t}BuBrettPhos (4.5 mol%)
1,4-dioxane (0.25 M) 130 °C, 20 h

Scheme S4 Route to oxetane sulfonyl fluorides from oxetane phenol sulfide **131**.

<u>Notes on sulfinate elimination of oxetane sulfone **136c**</u>

Oxetane sulfone **136c** contains an ethyl ester pendant on the aryl group. It was found that performing the sulfinate elimination step on this sulfone with NaOMe (**OSE-3**) or NaH and quenching with MeOH (**OSE-1**) resulted in almost complete transesterification to the methyl ester. When NaOEt (21 wt. % in EtOH) is used as the base for elimination and anhydrous EtOH employed as the solvent for the fluorination step, no undesired transesterification is observed and the pendant ethyl ester is retained.

Synthesis route to azetidine sulfonyl fluorides (ASFs) from N-Cbz-azetidinone

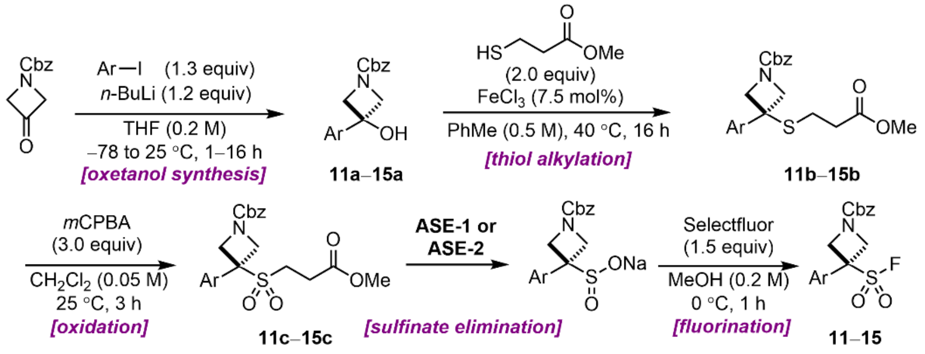

azetidine sulfinate elimination (ASE) conditions

reference	conditions
ASE-1	5.4 M NaOMe in MeOH (1.0 equiv), THF (0.4 M), 25 °C, 20 min
ASE-2	NaH (1.05 equiv), THF (0.2 M), 0–25 °C, 60 min *then* anhydrous MeOH quench (0.2 mL per 0.1 mmol sulfone)

Scheme S5 General synthesis route to ASFs and the different azetidine sulfinate elimination (ASE) conditions used

Azetidine sulfinate elimination (ASE) procedures

- **ASE-1:** NaOMe (5.4 M solution in MeOH, 1.0 equiv) was added dropwise to a solution of azetidine sulfone (1.0 equiv) in anhydrous THF (0.4 M) at 25 °C. The reaction mixture was stirred at 25 °C for 20 min, then concentrated *in vacuo* using a rotatory evaporator to afford the azetidine sulfinate salt.

- **ASE-2:** Azetidine sulfone (1.0 equiv) was added to a solution of NaH (60% dispersion in mineral oil, 1.05 equiv) in anhydrous THF (0.2 M) at 0 °C. The reaction was allowed to warm to 25 °C over 1 h and then quenched with anhydrous MeOH (0.2 mL per 0.1 mmol of sulfone) and concentrated *in vacuo* using a rotary evaporator to afford the azetidine sulfinate salt.

Synthesis, handling and storage considerations of sulfonyl fluorides

<u>Physical state of sulfinate salts:</u>

Usually, oxetane, azetidine and cyclobutane sulfinate salts precipitate upon addition of base. For some lipophilic examples there was a color change of the reaction mixture instead of precipitation.

Often the oxetane, azetidine and cyclobutane sulfinate salts afforded after elimination are solids, but they can also sometimes appear as residues and gums. When gums and residues are afforded, it is best to ***not*** transfer the sulfinate residue from the initial reaction flask, but instead to add the pre-cooled anhydrous MeOH directly to the residue, followed by the Selectfluor™, and perform the fluorination step in the same reaction vessel.

<u>Hazards of the sulfinate elimination:</u>

Special attention should be paid when concentrating the reaction mixture, since methyl acrylate (volatile and very toxic) is generated as a by-product. Only use rotatory evaporators in a ventilated space.

Concentrating sulfinate salts and sulfonyl fluorides:

Most oxetane, azetidine and cyclobutane sulfinate salts and sulfonyl fluorides are unstable to prolonged heating. When concentrating the reaction mixtures containing sulfinate salts and sulfonyl fluorides, set the rotary evaporator water bath to no higher than 20 °C to minimize potential decomposition and defluorosulfonylation.

Purification of sulfonyl fluorides:

Most oxetane, azetidine and cyclobutane sulfonyl fluorides are unstable on silica. Column chromatography should be performed in the shortest time possible (<15 min if possible) and using the shortest possible column or a Biotage® Selekt (or equivalent) system. As a reference, a 3 mmol-scale reaction can be purified with 2.5 cm of silica in a column of 1.5 cm diameter.

Storage recommendations:

Oxetane, azetidine and cyclobutane sulfonyl fluorides are remarkably stable at room temperature (25 °C), but very reactive at increased temperatures (*e.g.* 60 °C). The stability of the sulfonyl fluoride is, however, somewhat substrate dependent. For example, PMP OSF **1** can be left at room temperature under air ('bench'-conditions) for prolonged periods of time (>6 months) without significant loss in purity (still >85% pure after 6 months). Contrastingly, OTIPS OSF **2** degraded completely from a white solid to a purple gum after 30 days on the bench. Oxetane sulfonyl fluorides turn from white to pink as a sign of degradation.

It is recommended that the bulk of material is stored as the sulfone precursor and converted to the corresponding sulfonyl fluoride when required. However, all synthesized oxetane, azetidine and cyclobutane sulfonyl fluorides have been indefinitely stable (>12 months) in the freezer (–20 °C).

Optimization of thiol alkylation with oxetanols

Table S1 Optimization of thiol alkylation of PMP oxetanol **1a** with methyl 3-mercaptopropionate.

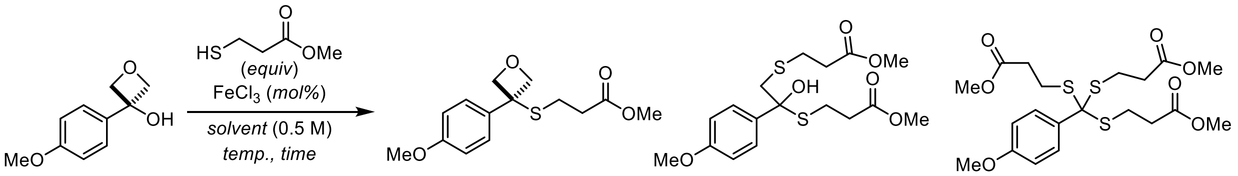

1a **1b** **1b'** **1b"**

Entry	Solvent (0.5 M)	$FeCl_3$ (mol%)	Thiol (equiv)	Temp. (°C)	Time	^{1}H NMR Yield (%)[a]				
						oxetanol **1a**	sulfide **1b**	di-addition **1b'**	tri-addition **1b"**	total
**1**[c]	CH_2Cl_2	5	2	40	1 h	44	47	9	0	N/A
**2**	CH_2Cl_2	10	2	40	1 h	0	51	2	13	66
**3**	CH_2Cl_2	10	2	70	1 h	0	54	15	7	76
**4**[b]	CH_2Cl_2	20	2	40	1 h	0	55	34	11	N/A
**5**	CH_2Cl_2	20	2	70	1 h	0	33	13	5	51
**6**	**CH_2Cl_2**	**10**	**2**	**40**	**25 min**	**0**	**84**	**0**	**12**	**96**
**7**	CH_2Cl_2	7.5	2	40	25 min	0	80	0	7	87
**8**[b*]	PhMe	5	2	40	1 h	69	21	11	0	N/A
**9**	PhMe	7.5	2	70	20 min	0	74	8	19	101
**10**	PhMe	10	2	70	20 min	0	67	7	27	101
**11**[c]	CH_2Cl_2	10	1.2	40	25 min	0	84	4	9	97
**12**[c]	PhMe	7.5	1.2	70	25 min	0	70	8	4	82
**13**[c]	CH_2Cl_2	10	1.5	40	20 min	0	61	0	9	90

All reactions performed on a 0.5 mmol scale unless otherwise stated. [a]%Yield given by analysis of crude ^{1}H NMR in comparison to 1,3,5-trimethoxybenzene as an internal standard. [b]Unable to determine ^{1}H NMR due to filtration issues during work-up, values presented are instead through relative distribution of the products. [c]Performed using an alternate reagent order of addition (see below for details). *Reaction performed on a 1.0 mmol scale.

Standard reagent order of addition: (1) reaction vessel flame-dried under argon (2) oxetanol **1a** added (3) $FeCl_3$ added (4) reaction vessel sealed (5) anhydrous solvent added (6) thiol added (7) reaction vessel placed in pre-heated oil bath

Alternate reagent order of addition: (1) reaction vessel flame-dried under argon (2) $FeCl_3$ added (3) anhydrous solvent added (4) thiol added (5) oxetanol **1a** added (6) reaction vessel sealed and placed in pre-heated oil bath.

Table S2 Optimization of thiol alkylation of *para*-OTIPS oxetanol **2a** with methyl 3-mercaptopropionate.

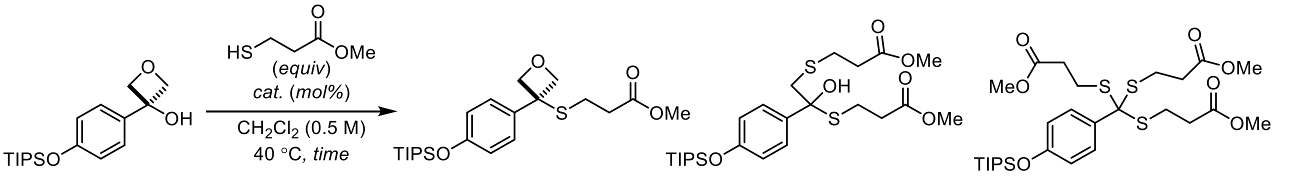

Entry	catalyst (mol%)	Thiol (equiv)	Time (min)	^{1}H NMR Yield (%)[a]				
				oxetanol **2a**	sulfide **2b**	di-addition **2b'**	tri-addition **2b"**	total
**1**	$Ca(NTf_2)_2/BuNPF_6$ (5/5)	2	25	0	64	0	17	81
**2**	$Ca(NTf_2)_2/BuNPF_6$ (10/10)	2	25	0	59	5	10	74
**3**	$Ca(NTf_2)_2/BuNPF_6$ (5/5)	2	15	0	55	0	9	64
**4**	$Ca(NTf_2)_2/BuNPF_6$ (5/5)	2	20	0	66	0	16	82
**5**	$Ca(NTf_2)_2/BuNPF_6$ (5/5)	1.5	20	4	56	9	8	77
**6**	**$FeCl_3$ (10)**	**2**	**20**	**0**	**77**	**0**	**20**	**97**
**7**	$FeCl_3$ (10)	1.5	20	0	76	0	11	87

All reactions performed on a 0.1 mmol scale. [a]%Yield given by analysis of crude ^{1}H NMR in comparison to 1,3,5-trimethoxybenzene as an internal standard. All reactions performed using the following order of addition: (1) reaction vessel flame dried (2) catalyst added (3) thiol added (4) oxetanol **2a** added (5) anhydrous CH_2Cl_2 added (6) vessel sealed and placed in pre-heated oil bath.

Table S3 Optimization of thiol alkylation of phenoxy oxetanol **10a** with methyl 3-mercaptopropionate.

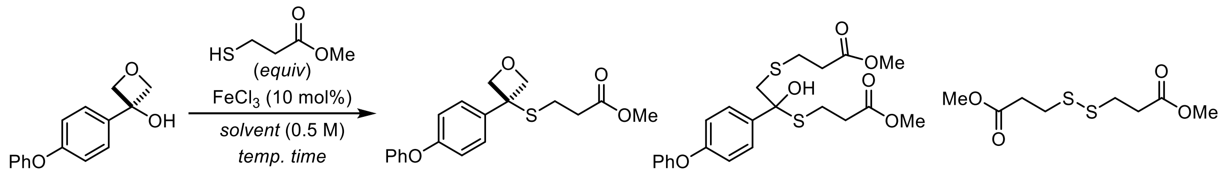

10a **10b** **10b'** **S1**

Entry	Solvent (0.5 M)	Thiol (equiv)	Temp. (°C)	Time	^{1}H NMR Yield (%)[a]			
					oxetanol **10a**	sulfide **10b**	di-addition **10b'**	disulfide **S1**
**1**	$CHCl_3$	2	60	1 h	0	36	10	3
**2**[b]	$CHCl_3$	2	60	20 min	0	4	21	12
**3**[b]	$CHCl_3$	2	60	30 min	0	16	22	5
**4**[b]	$CHCl_3$	2	60	40 min	0	13	17	6
**5**	PhMe	2	60	1 h	32	15	0	10
**6**	CH_2Cl_2	1.5	40	1 h	21	28	0	N.D.[c]
**7**	CH_2Cl_2	2	40	1 h	32	23	0	4
**8**	CH_2Cl_2	1.5	40	5 h	27	25	0	3
**9**	$CHCl_3$	1.5	60	20 min	29	31	0	3
**10**	$CHCl_3$	1.5	60	40 min	29	32	0	3
**11**	**$CHCl_3$**	**1.5**	**60**	**1 h**	**31**	**35**	**0**	**N.D.**[c]
**12**	$CHCl_3$	1.5	60	5 h	38	25	0	25

All reactions performed on a 0.2 mmol scale. [a]%Yield given by analysis of crude ^{1}H NMR in comparison to 1,3,5-trimethoxybenzene as an internal standard. [b]Reactions performed on a 0.1 mmol scale. [c]Peaks overlapped in ^{1}H NMR spectrum. N.D. = not determined.

Additional consideration for $FeCl_3$-catalyzed thiol alkylation conditions – the formation of dimethyl 3,3'-disulfanediyldipropionate:

$FeCl_3$ is known to also act as a mild oxidant. Therefore, under the optimized reaction conditions, $FeCl_3$ is capable of oxidizing methyl 3-mercaptopropanoate to its disulfide dimer dimethyl 3,3'-disulfanediyldipropionate **S1** (Scheme **S6**).

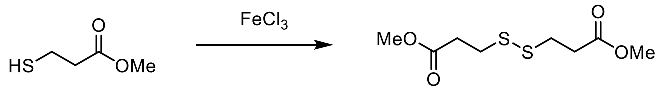

methyl 3-mercaptopropanoate **S1**

Scheme S6 Proposed oxidation of methyl 3-mercaptopropanoate to dimethyl 3,3'-disulfanediyldipropionate **S1**.

The dimethyl 3,3'-disulfanediyldipropionate disulfide **S1** (see below for characterization data) can be separated from the desired oxetane sulfide products by column chromatography and is visible by TLC when staining with cerium ammonium molybdate (CAM). If the disulfide does co-elute with the desired product, the disulfide is destroyed during the *m*CPBA oxidation step to the oxetane sulfone.

Characterization data for dimethyl 3,3'-disulfanediyldipropionate disulfide (**S1**):

Colorless oil; R_f = 0.15 (15% EtOAc/*n*-hexane); IR (film)/cm^{-1} 2951, 1729 (C=O st); 1434, 1353, 1238, 1169, 1135, 978; ^{1}H NMR (400 MHz, $CDCl_3$) δ 3.63 (s, 6 H, 2 × CO_2CH_3), 2.85 (t, *J* = 7.2 Hz, 4 H, 2 × SCH_2CH_2), 2.67 (t, *J* = 7.2 Hz, 4 H, 2 × SCH_2CH_2); ^{13}C NMR (101 MHz, $CDCl_3$) δ 172.0 (2 × C_q=O), 51.8 (2 × SCH_2CH_2), 33.8 (2 × SCH_2CH_2), 33.0 (2 × CO_2CH_3). The observed characterization data (IR, ^{1}H, ^{13}C) were consistent with that previously reported.[3]

Optimization of deFS couplings between PMP OSF 1 and different nucleophile types

Table S4 Optimization of deFS between PMP OSF **1** and pyrazole.

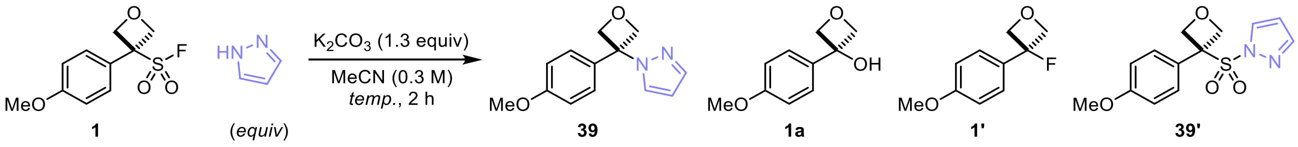

Entry	Pyrazole (equiv)	Temp. (°C)	^{1}H NMR Yield (%)[a]				
			OSF **1**	oxetano-pyrazole **39**	oxetanol **1a**	oxetane fluoride **1'**	sulfonamide **39'**
**1**[b]	1.2	60	1	85	0	4	1
**2**[b]	1.2	70	0	83	0	2	3
**3**[b]	3.0	60	1	71	0	5	10
**4**[c]	**1.2**	**60**	**3**	**87**	**0**	**8**	**trace**
**5**[c]	1.2	70	0	85	0	7	trace
**6**[c]	3.0	60	0	74	0	6	4

All reactions performed on 0.1 mmol scale. [a]%Yield given by analysis of crude ^{1}H NMR in comparison to 1,3,5-trimethoxybenzene as an internal standard. [b]Order of addition: K_2CO_3, OSF **1**, pyrazole, MeCN. [c]Order of addition: K_2CO_3, OSF **1**, MeCN, pyrazole.

Table S5 Investigation into the role of bases in deFS between PMP OSF **1** and 3-methylpyrazole.

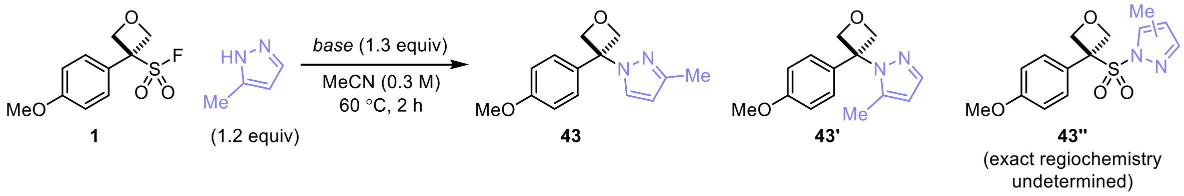

Entry	Base	^{1}H NMR Yield (%)[a]			**43:43'** ratio
		oxetano-pyrazole **43**	oxetano-pyrazole **43'**	sulfonamide **43"**	
**1**	K_2CO_3	72	11	0	87:13
**2**	Cs_2CO_3	44	8	27	85:15
**3**	No base	78	14	0	85:15

All reactions performed on 0.1 mmol scale. [a]%Yield given by analysis of crude ^{1}H NMR in comparison to 1,3,5-trimethoxybenzene as an internal standard.

Changing base from K_2CO_3 to Cs_2CO_3 had little effect on regioselectivity of the resulting amino-oxetane product, but did impact the rate of SuFEx reactivity to form the corresponding sulfonamide product. The effect is likely explained by the joint effects of the increased solubility and the larger cationic radius of Cs^+ compared to K^+. Cs_2CO_3 has been found to be the most soluble of the carbonate salts in polar aprotic organic solvents, which creates a more basic reaction media.[4] This results in a larger proportion of pyrazolate anions (pyrazole pK_a = 19.8 in DMSO),[5] which, coupled with the weaker ion-pairing of Cs^+, are highly "naked" strong nucleophiles. Overall, these effects lower the energy barrier for the SuFEx pathway and accelerate such reactivity. The precise regioisomer of the sulfonamide SuFEx product **43"** could not be determined by selective nOe experiments.

Table S6 Optimization of deFS between PMP OSF **1** and imidazole.

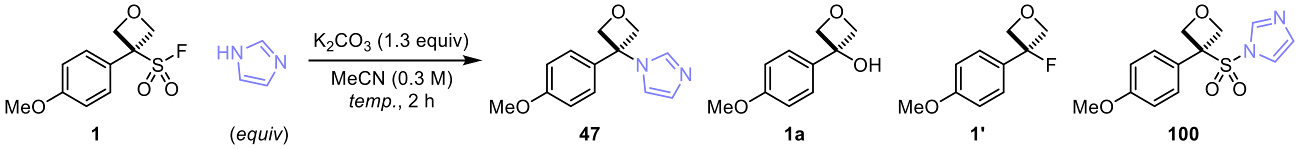

Entry	Imidazole (equiv)	Temp. (°C)	^{1}H NMR Yield (%)[a]				
			OSF **1**	oxetano-imidazole **47**	oxetanol **1a**	oxetane fluoride **1'**	sulfonamide **100**
**1**	1.2	60	0	46	0	9	16
**2**	3.0	60	0	69	0	0	13
**3**	1.2	70	0	51	0	0	0
**4**	**3.0**	**70**	**0**	**69**	**0**	**6**	**8**

All reactions performed on 0.1 mmol scale. [a]%Yield given by analysis of crude ^{1}H NMR in comparison to 1,3,5-trimethoxybenzene as an internal standard.

Table S7 Optimization of deFS between PMP OSF **1** and 1,2,4-triazole.

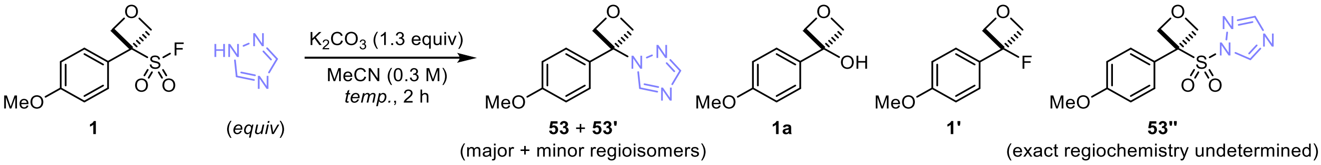

Entry	Triazole (equiv)	Temp. (°C)	^{1}H NMR Yield (%)[a]				
			OSF **1**	oxetano-triazoles **53** + **53'**	oxetanol **1a**	oxetane fluoride **1'**[b]	sulfonamide **53"**[b]
**1**	1.2	60	0	45	0	N.D.	N.D.
**2**	3.0	60	0	85	0	N.D.	N.D.
**3**	1.2	70	0	55	0	N.D.	N.D.
**4**	**3.0**	**70**	**0**	**87**	**0**	**N.D.**	**N.D.**

All reactions performed on 0.1 mmol scale. [a]%Yield given by analysis of crude ^{1}H NMR in comparison to 1,3,5-trimethoxybenzene as an internal standard. [b]Difficult to determine due to complex spectra. N.D. = not determined.

Table S8 Optimization of deFS between PMP OSF **1** and 5-methyl tetrazole.

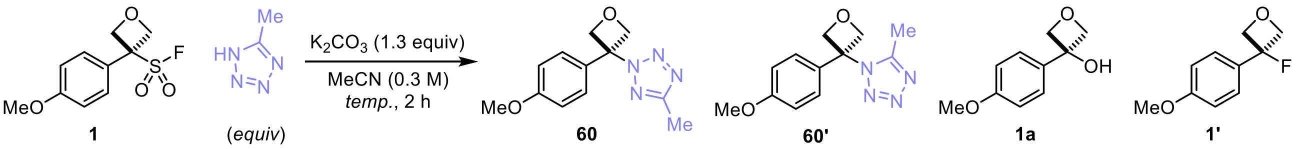

Entry	Tetrazole (equiv)	Temp. (°C)	^{1}H NMR Yield (%)[a]				
			OSF **1**	3-methyl oxetano-tetrazole **60**	2-methyl oxetano-tetrazole **60'**	oxetanol **1a**	oxetane fluoride **1'**
**1**	1.2	60	0	55	21	0	0
**2**	3.0	60	0	45	34	0	0
**3**	1.2	70	0	40	29	0	0
**4**	**3.0**	**70**	**0**	**51**	**39**	**0**	**0**

All reactions performed on 0.1 mmol scale. [a]%Yield given by analysis of crude ^{1}H NMR in comparison to 1,3,5-trimethoxybenzene as an internal standard.

Table S9 Optimization of deFS between PMP OSF **1** and lysidine.

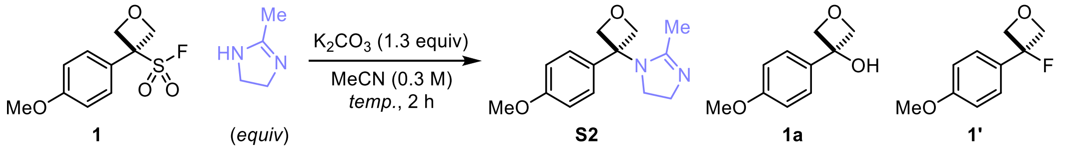

Entry	Lysidine (equiv)	Temp. (°C)	^{1}H NMR Yield (%)[a]			
			OSF **1**	amino-oxetane **S2**	oxetanol **1a**	oxetane fluoride **1'**
**1**	1.2	60	0	31	0	18
**2**	3.0	60	<1	54	0	0
**3**	1.2	70	0	32	0	0
**4**	**3.0**	**70**	**0**	**52**	**0**	**0**

All reactions performed on 0.1 mmol scale. [a]%Yield given by analysis of crude ^{1}H NMR in comparison to 1,3,5-trimethoxybenzene as an internal standard.

<u>Note on lysidine optimization:</u>

Despite positive results of observed amino-oxetane **S2** formation in the deFS with lysidine, purification of the products was very challenging due to the high polarity and co-elution with the lysidine starting material.

Table S10 Optimization of deFS between PMP OSF **1** and $TMSN_3$.

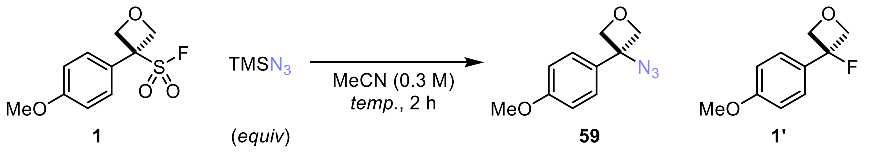

Entry	$TMSN_3$ (equiv)	Temp. (°C)	^{1}H NMR Yield (%)[a]		
			OSF **1**	oxetane azide **59**	oxetane fluoride **1'**
**1**	1.2	60	5	60	9
**2**	3.0	60	6	74	6
**3**	1.2	70	0	84	7
**4**	**3.0**	**70**	**0**	**87**	**7**

All reactions performed on 0.1 mmol scale. [a]%Yield given by analysis of crude ^{1}H NMR in comparison to 1,3,5-trimethoxybenzene as an internal standard.

Note on $TMSN_3$ optimization:

All reactions were performed in the absence of K_2CO_3. This is because the liberated fluoride anion from the deFS of PMP OSF **1** activates the $TMSN_3$ by removal of the TMS group. K_2CO_3 would hinder this process by trapping released fluoride anions as KF.

Table S11 Optimization of deFS between PMP OSF **1** and *p*Br-methyl sulfoximine **S21**.

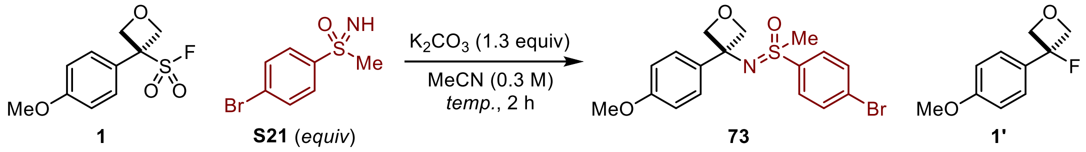

Entry	Sulfoximine (equiv)	Temp. (°C)	^{1}H NMR Yield (%)[a]		
			OSF **1**	oxetane-sulfoximine **73**	oxetane fluoride **1'**
**1**	1.2	60	6	72	2
**2**	**3.0**	**60**	**<1**	**93**	**<1**
**3**	1.2	70	0	82	8

All reactions performed on 0.1 mmol scale. [a]%Yield given by analysis of crude ^{1}H NMR in comparison to 1,3,5-trimethoxybenzene as an internal standard.

Note on sulfoximine optimization:

Conditions from Entry 2 (Table **S11**) can be used with reversed stoichiometry (*i.e.* 3.0 equiv OSF **1**, 1.0 equiv sulfoximine **S21**) to afford the corresponding oxetane-sulfoximine **73** in 87% yield.

Table S12 Optimization of deFS between PMP OSF **1** and P(OEt)$_3$.

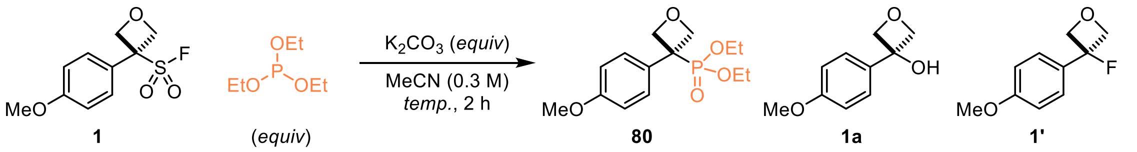

Entry	P(OEt)$_3$ (equiv)	K_2CO_3 (equiv)	Temp (°C)	^{1}H NMR Yield (%)[a]	
				OSF **1**	oxetane phosphonate **80**
**1**	1.2	1.3	60	4	64 (40)
**2**	3.0	3.0	60	3	77
**3**	**3.0**	**0**	**60**	**0**	**88 (70)**
**4**	3.0	0	70	0	73

All reactions performed on 0.1 mmol scale. [a]%Yield given by analysis of crude ^{1}H NMR in comparison to 1,3,5-trimethoxybenzene as an internal standard.

<u>Note on the proposed Michaelis-Arbuzov mechanism:</u>

Tri-alkyl phosphite reagents are used in the Michaelis-Arbuzov phosphorylation of alkyl halides to generate alkyl phosphonates. In the mechanism, the released halide ion performs the dealkylation of the phosphonium intermediate to generate the P=O double bond. We propose the released fluoride ion from the defluorosulfonylation of OSF and ASF reagents performs this crucial dealkylation step (Scheme **S7**).

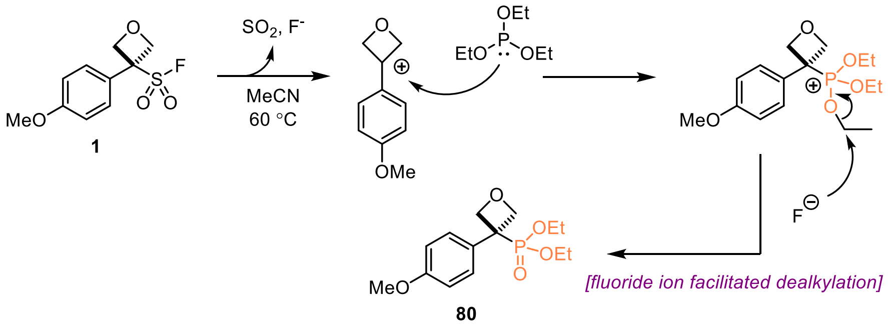

Scheme S7 Proposed Michaelis-Arbuzov mechanism with PMP OSF **1**.

K_2CO_3 is employed in the standard deFS reaction conditions to minimize formation of oxetane fluoride by trapping released fluoride ions. Performing this reaction in the absence of K_2CO_3 provides higher yields, presumably due to the higher concentration of fluoride ions in solution capable of dealkylating the phosphonium ion intermediate.

Optimization of SuFEx couplings between PMP OSF 1 and different nucleophile types

SuFEx with amines

We previously observed that the reactivity between OSFs and phenols can be switched between deFS coupling or SuFEx by changing the nature of the base. Standard conditions with K_2CO_3 affords a mixture of deFS and SuFEx products. Organic soluble bases (Et_3N) shifts selectivity towards the deFS oxetane ether product. Softer countercations of carbonate bases (Cs_2CO_3) leads to selective formation of the SuFEx derived sulfonate ester, while weaker bases ($KHCO_3$) unable to deprotonate the phenol but still capable of trapping fluoride ions yields the deFS oxetane ether in complete selectivity.

Pre-formation of sodium phenolate with NaH gives complete SuFEx selectivity in high yields. This can be rationalized by the proposed differences in rate between the SuFEx and deFS reactions, ie that the rate of SuFEx between the oxetane sulfonyl fluoride and phenoxide is faster than defluorosulfonylation, but the rate of defluorosulfonylation is faster than SuFEx between oxetane sulfonyl fluoride and phenol (Table **S13**).[6]

Table S13 Effect of base on the formation of sulfonate ester and oxetane ethers from OSF **1** and phenol.

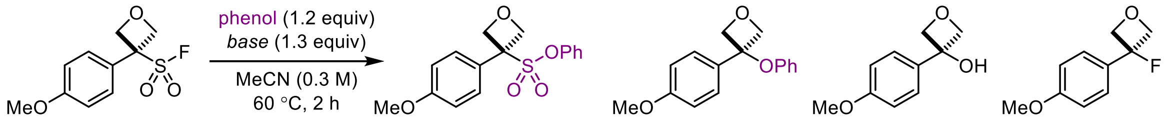

Entry	Base	^{1}H NMR Yield (%)[a]			
		sulfonate ester **S3**	oxetane ether **S4**	oxetanol **1a**	oxetane fluoride **1'**
**1**	K_2CO_3	54 (41)	36 (30)	0	17
**2**	Et_3N	9 (8)	50 (49)	0	31
**3**	Cs_2CO_3	88 (76)	11 (10)	0	5
**4**	$KHCO_3$	0	49 (39)	0	53
**5**[b]	NaH	100 (83)	0	0	4
**6**	TMS-phenol (no base)	2	67 (62)	0	8
**7**[c]	No base	N.D.	N.D. (3)	N.D.	N.D.

All reactions performed on 0.2 mmol scale. [a]%Yield given by analysis of crude ^{1}H NMR in comparison to 1,3,5-trimethoxybenzene as an internal standard. Isolated yields in parentheses. [b]1.205 equiv of NaH used. NaH was added portionwise to a solution of phenol in MeCN at 0 °C. The reaction was warmed to 25 °C, OSF **1** was added and the reaction vial was sealed. The reaction mixture was warmed up to 60 °C and stirred at this temperature for 2 h. [c]The ^{1}H NMR spectrum of the crude mixture of the reaction was too complex to determine *in situ* yields. N.D. = not determined.

Similarly, we envisaged that formally deprotonated amines would undergo SuFEx by outcompeting the rate of the deFS reaction. We found that subjecting the PMP OSF **1** to typical deFS conditions while in THF resulted in almost complete recovery of the OSF by ^{1}H NMR analysis (Entry 7, Table **S14**).

Table S14 Sensitivity of the deFS coupling towards air, high dilution, variable base loading, and solvent polarity.

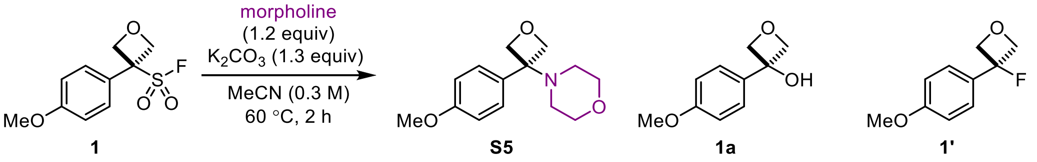

Entry	Deviations from standard	^{1}H NMR Yield (%)[a]			
		OSF **1**	amino-oxetane **S5**	oxetanol **1a**	oxetane fluoride **1'**
**1**	None	0	85	9	4
**2**	Under air	0	93	4	3
**3**	0.07 M	0	82	11	6
**4**	3.0 equiv K_2CO_3	0	76	17	9
**5**	0.5 equiv K_2CO_3	0	57	21	19
**6**	Under air, 0.07 M, 3.0 equiv K_2CO_3	0	92	3	2
**7**	**THF**	**98**	**2**	**0**	**0**

All reactions performed on 0.1 mmol scale. [a]%Yield given by analysis of crude ^{1}H NMR in comparison to 1,3,5-trimethoxybenzene as an internal standard.

This result demonstrates the essential role of MeCN in stabilising the 3° carbocation, something THF cannot do as a relatively less polar solvent. However, a stronger base than K_2CO_3 is required to overcome the energetic barrier for SuFEx. Morpholine was therefore fully deprotonated using *n*-BuLi at –78 °C and OSF **1** added directly as a solid, generating oxetane sulfonamide **96** in 80% yield (Scheme **S8**).

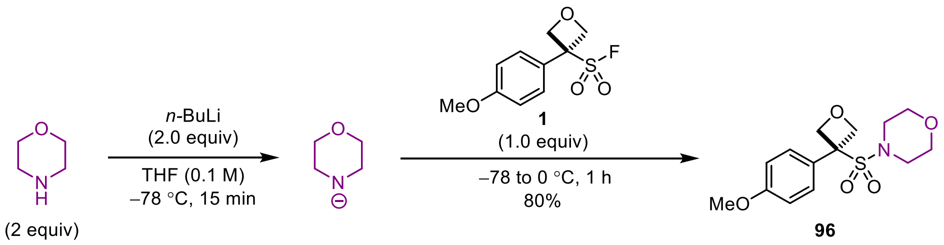

Scheme S8 Synthesis of PMP oxetane sulfonamide **96**.

The tolerance of this unique SuFEx reactivity with amines was investigated in reactions between PMP OSF **1** and benzylamine (Table **S15**).

Table S15 Optimization of SuFEx between PMP OSF **1** and benzylamine.

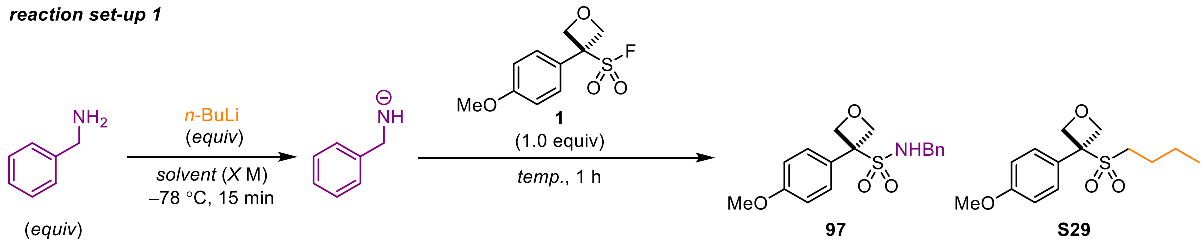

Entry	Solvent (conc.)	Amine/*n*-BuLi (equiv)	Temp (°C)	^{1}H NMR Yield (%)[a]		
				OSF **1**	sulfonamide **97**	sulfone **S29**
**1**	THF (0.1 M)	2.0	0 to –78	0	71 (62)	7
**2**[b]	1,4-dioxane (0.1 M)	2.0	rt	0	48*	20
**3**	Et_2O (0.1 M)	2.0	0 to –78	0	15*	0
**4**	CPME (0.1 M)	2.0	0 to –78	0	67	11
**5**	THF (0.3 M)	2.0	0 to –78	0	49	15
**6**	THF (0.5 M)	2.0	0 to –78	0	66	18
**7**	THF (0.1 M)	1.2	0 to –78	0	38*	23

All reactions performed on 0.1 mmol scale. [a]%Yield given by analysis of crude ^{1}H NMR in comparison to 1,3,5-trimethoxybenzene as an internal standard. [b]Deprotonation and subsequent reaction performed at room temperature due to high melting point of 1,4-dioxane of 12 °C. *Broad peaks used to determine yields.

The use of different apolar solvents is tolerated, albeit in lower yields (1,4-dioxane and Et_2O). CPME offers comparable results to THF as a process-friendly alternative solvent. A significant issue was the presence of oxetane sulfone **S29**, derived from the addition of *n*-BuLi through SuFEx. The structure of oxetane sulfone **S29** was confirmed through independent preparation and isolation. To prevent *n*-BuLi addition (through SuFEx), a series of modifications were made to the reaction set up to avoid the formation of the sulfone and provide sulfonamide **97** in higher and more consistent yields (Scheme **S9**).

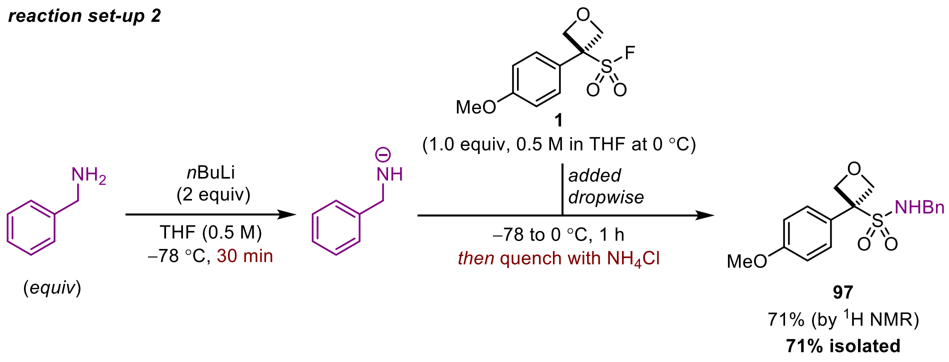

Scheme S9 Synthesis of PMP oxetane sulfonamide **97** under optimized conditions and reaction set-up.

Important changes in the optimized reaction set-up were the following:

- Benzylamine deprotonation time was increased from 15 to 30 min to ensure a more complete consumption of the *n*-BuLi and prevent formation of oxetane sulfone **S29**
- PMP OSF **1** added dropwise as a solution to avoid side reactions compared to adding directly as a solid.
- Quench the reaction with NH_4Cl rather than MeOH to prevent potential methylation/MeOH deprotonation pathways.

SuFEx with NH-azoles (imidazole)

A similar approach to the amines was taken with the NH-azole nucleophiles – creating a harder nucleophile and performing the reaction in an apolar solvent (Table **S16**).

Table S16 Optimization of SuFEx between PMP OSF **1** and imidazole.

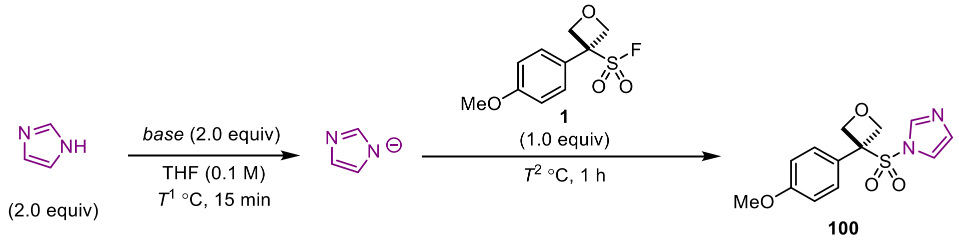

Entry	Base	T^1 (°C)	T^2 (°C)	sulfonamide **100**	
				^{1}H NMR Yield (%)[a]	Isolated yield (%)
**1**	*n*-BuLi	–78	–78 to 0	49	20
**2**	^{t}BuOK	0	0	67	52
**3**	**NaH**	**0**	**0**	**100**	**88**

All reactions performed on 0.25 mmol scale. [a]%Yield given by analysis of crude ^{1}H NMR in comparison to 1,3,5-trimethoxybenzene as an internal standard.

Pleasingly, simple deprotonation of imidazole prior to addition of OSF **1** directly as a solid provided the desired sulfonamide product **100** in high yields (Entry 3, Table **S16**).

SuFEx with NaN_3

NaN_3 was employed as a 'harder' nucleophile alternative to $TMSN_3$ (used in the deFS optimization to provide oxetane azide **59**). The addition of 15-crown-5 ether creates a more 'naked' N_3 anion, enabling a more efficient SuFEx process (Table **S17**).

Table S17 Optimization of SuFEx between PMP OSF **1** and NaN_3.

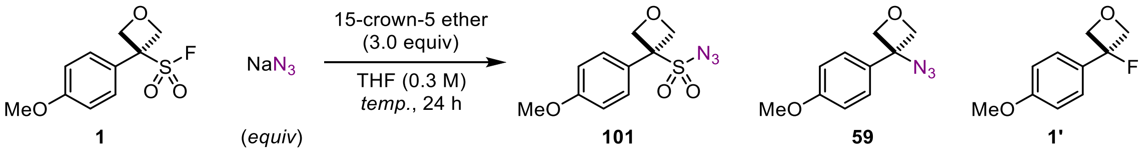

Entry	NaN_3 (equiv)	Temp. (°C)	^1H NMR Yield (%)[a]		
			sulfonyl azide **101**	oxetane azide **59**	oxetane fluoride **1'**
**1**	1.2	rt	80 (65)	2	2
**2**	**3.0**	**rt**	**88**	**<1**	**<1**
**3**	1.2	35	79	3	5
**4**	3.0	35	74	5	3

All reactions performed on 0.25 mmol scale. [a]%Yield given by analysis of crude ^1H NMR in comparison to 1,3,5-trimethoxybenzene as an internal standard. Isolated yields in parentheses.

SuFEx comparison between cyclobutane sulfonyl fluoride 16 and OSF 139

The influence of the type of 4-membered ring on SuFEx reactivity was probed in a head-to-head comparison with OSF **139** and cyclobutane sulfonyl fluoride **16** both bearing a phenyl ring (Table **S18**).

Table S18 Comparison of SuFEx reactivity between cyclobutane sulfonyl fluoride **16** and OSF **139**

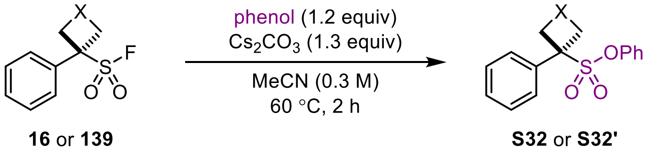

Entry	X =	sulfonyl fluoride	sulfonate ester	Isolated yield of sulfonate ester (%)
**1**	CH_2	**16**	**S32**	87
**2**	O	**139**	**S32'**	98

All reactions performed on a 0.117 mmol scale. Characterization data and yield for sulfonate ester **S32'** previously reported in ref. 6.

Both sulfonyl fluorides exhibit excellent SuFEx reactivity with phenol/Cs_2CO_3 under identical conditions. The slightly improved yield with OSF **139** may suggest that the more electron-withdrawing nature of the oxetane ring increases the electrophilic nature of the sulfur centre, but is more likely to be a reflection of the absence of a competing deFS pathway on this reagent at 60 °C.

Defluorosulfonylative (deFS) coupling General Procedures

- **deFS General Procedure A:** Anhydrous K_2CO_3 (1.3 equiv), nucleophile (1.0–3.0 equiv) and sulfonyl fluoride (1.0 equiv) were added sequentially to a flame-dried reaction tube under argon. The reaction vessel was sealed and anhydrous MeCN (0.3 M) was added by syringe. The reaction mixture was stirred at 60 or 70 °C for 2–18 h and then either a Celite or aqueous workup was performed. Purification by flash column chromatography afforded the desired product.
- **deFS General Procedure B:** Anhydrous K_2CO_3 (1.3 equiv), sulfonyl fluoride (1.0 equiv), anhydrous MeCN (0.3 M) and nucleophile (1.2–3.0 equiv) were added sequentially to a flame-dried reaction tube under argon. The reaction mixture was stirred at 60 or 70 °C for 2–18 h and then either a Celite or aqueous workup was performed. Purification by flash column chromatography afforded the desired product.
- **deFS General Procedure C:** Anhydrous K_2CO_3 (3.2–3.6 equiv), nucleophile (1.0–1.2 equiv) and anhydrous MeCN (0.05 M) were added to a flame-dried reaction tube under argon. The reaction mixture was stirred at 70 °C for 10 min before addition of the sulfonyl fluoride (1.0–3.4 equiv). The reaction mixture was then warmed to 80 °C and stirred at this temperature for 2–18 h. Purification by flash column chromatography afforded the desired product.

Remarks:

deFS General Procedure A is suitable for most nucleophilic coupling partners.

deFS General Procedure B is the same as **deFS General Procedure A** but with a modified order of addition of reagents. This is done to reduce the SuFEx side-products often observed with these nucleophiles, most notably pyrazoles.

deFS General Procedure C involves pre-stirring and heating of the nucleophile with K_2CO_3 in MeCN. This is typically done with nucleophilic coupling partners which have poor solubility in MeCN, as the additional pre-stirring and heating can enable improved dissolution of these nucleophiles. Excess sulfonyl fluoride is used in some cases to ensure full consumption of the nucleophilic starting material and to simplify purification.

Celite Workup:

The reaction mixture was left to cool to 25 °C, EtOAc was added and the mixture was filtered through a plug of Celite, eluting with further EtOAc (3 × 10 mL). The solvent was then removed *in vacuo* using a rotatory evaporator.

EtOH can be used as an alternative solvent for the Celite filtration if the product solubility appears poor in EtOAc.

Aqueous Workup:

The reaction mixture was left to cool to 25 °C and water and CH_2Cl_2 were added. The layers were separated, and the aqueous portion extracted with CH_2Cl_2 (3 ×). The organic extracts were combined, dried over Na_2SO_4, filtered and concentrated *in vacuo* using a rotatory evaporator.

Kinetic analysis of PMP Azetidine Sulfonyl Fluoride ASF 11

Kinetic Experiments

The reaction of azetidine sulfonyl fluorides with amines was monitored by *in situ* ^{1}H NMR to gain a better understanding of the reaction and its mechanism. Mesitylene was added to the reaction as an inert internal standard.

Quantitative Reaction Monitoring by ^{1}H NMR

General Procedure and Considerations: Mesitylene (ca. 15 mg, 0.13 mmol, 0.75 equiv) was added by syringe to a tared oven-dried vial, followed by triethylamine (27 µL, 0.195 mmol, 1.3 equiv; added by a Gilson micropipette), amine nucleophile (0.18 mmol, 1.2 equiv; added by a Gilson micropipette) and azetidine sulfonyl fluoride **11** (0.15 mmol, 1.0 equiv). CD_3CN (0.5 mL, 0.5 M) was added to the vial by syringe and after solvation, the mixture was transferred using a glass pipette to an oven-dried Young's NMR tube under Ar and the tube was sealed using a Young's valve. The sealed Young's NMR tube was inserted into a 500 MHz Bruker NMR AvIII HD standard spectrometer with the probe pre-heated at the relevant temperature (40, 60 or 70 ºC). The sample was locked to CD_3CN, shimmed and a spectrum was acquired every 2 min 43 s until all the starting material had been consumed. The start of the reaction was taken as the time point when the sample was inserted into the pre-heated probe. The time point of the first measurement was taken as the time-interval between insertion of the sample into the probe and acquisition of the first spectrum (8 mins). The ^{1}H NMR spectra were acquired with 8 number of scans and 16.7 s relaxation delay (d1) and processed using MestReNova (version 12.0.4-22023). All the spectra of each reaction were superimposed and phase and baseline corrected using MestReNova. Then, the relevant signals for each species were integrated on the superimposed spectra, with the aryl peaks of mesitylene being used as the internal standard integral. The absolute integral values were exported as a .txt file (Script: 1D Integral Series) and converted to the plotted concentrations by using the internal standard. For an example of the superimposed spectra of the standard reaction at 60 ºC, see Fig. **S1**, and for the corresponding reaction profile, see Fig. **S2**. All statistical analyses and graphical representations were performed with Microsoft Excel (version 16.64) and the open-source statistical program 'R', version 3.2.2 (April 08, 2015 – "Fire Safety").[7]

Notes:

The exact amount of mesitylene added was measured by mass and it varied slightly between each experiment. The amounts of triethylamine and amine nucleophile added were measured by volume using a Gilson micropipette.

Triethylamine was used as base instead of K_2CO_3 to allow for a homogeneous reaction in the NMR tube.

A broad signal (assigned as **H**Et_3N^+) appeared on almost all reactions and was shifted downfield over the course of the reaction, presumably due to changes in its concentration (*i.e.* changes in the equilibrium between protonated and neutral species) and pH of the reaction mixture (see *e.g.* Fig. **S1**). This led in many cases to an overlap with the investigated signals during several minutes. These values were omitted from the analyses since they showed an artificial sharp increase (and later decrease) in concentration.

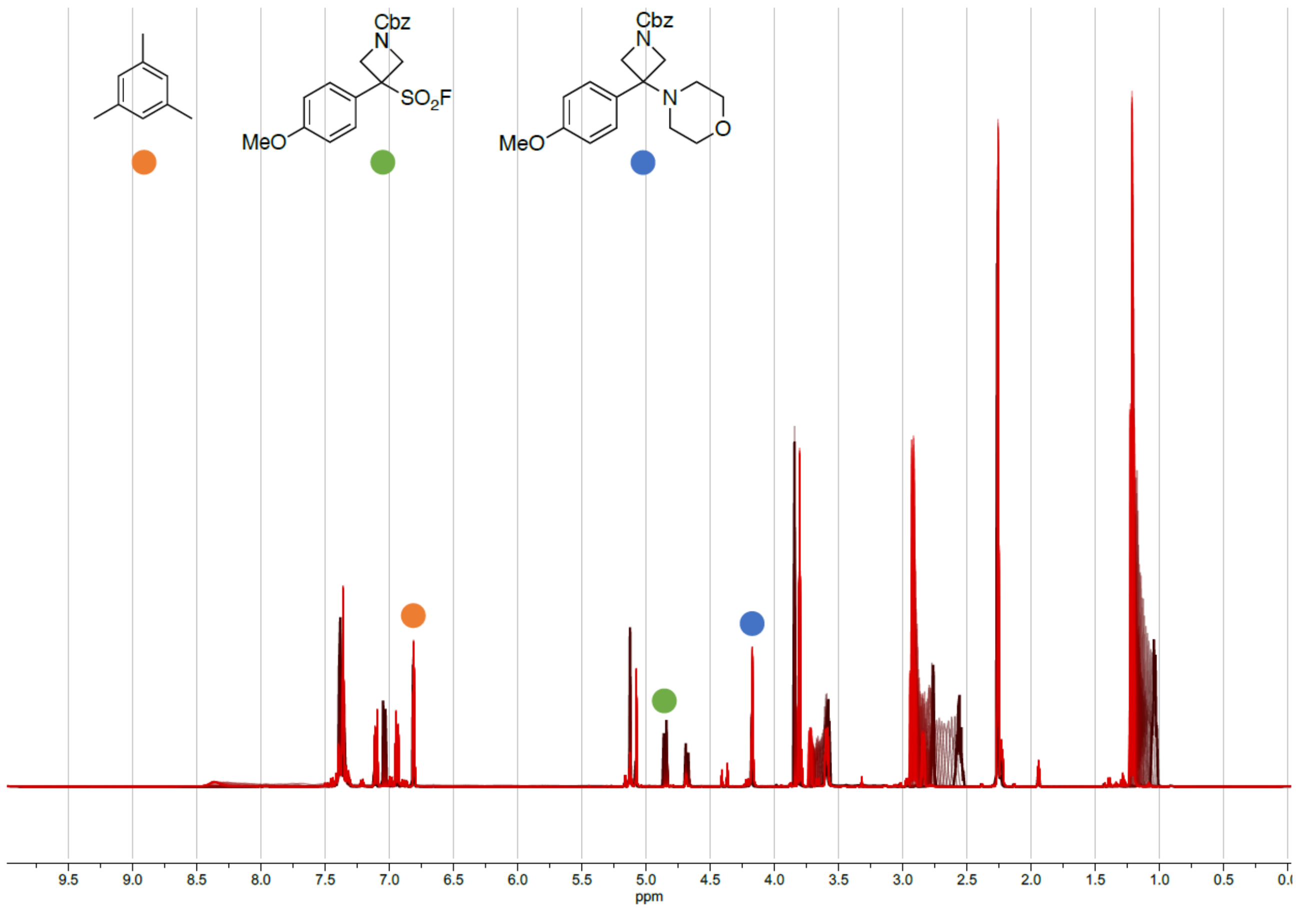

Fig. S1 Superimposed spectra for the reaction of ASF **11** with morpholine at 60 °C. The color of the signals go with time from dark red to bright red.

Reaction profile of ASF 11 with morpholine

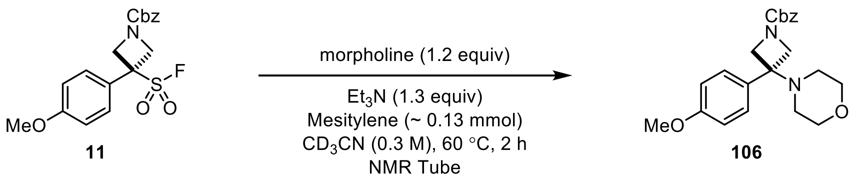

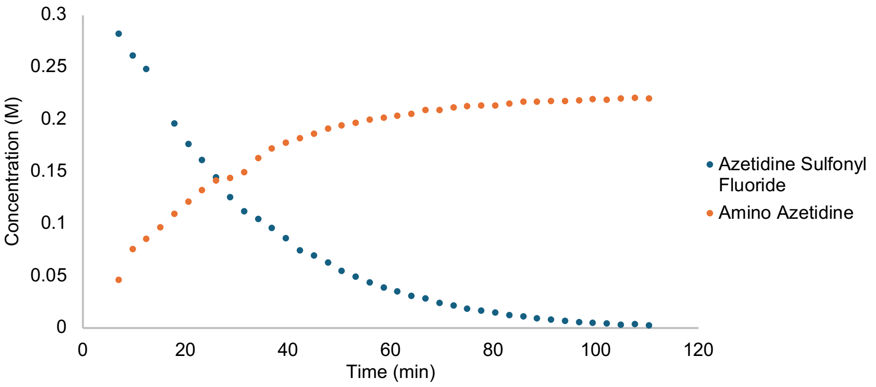

Fig. S2 Reaction profile of ASF **11** with morpholine to yield amino-azetidine **106**.

Variable Time Normalization Analysis (VTNA)

The defluorosulfonylative amination of oxetane sulfonyl fluorides was previously shown to be a first-order reaction, with first-order dependency on the concentration of OSF **1** and 0-order in all other components (amine, base).[6] The reaction of ASFs with amines was concluded to also proceed in a first-order fashion based on the following: 1) Similarity to the oxetane system; 2) the half-life of ASF **11** remains constant throughout the reaction, indicative of a first-order reaction (Fig. **S2**); 3) the plot of the sum of all components to the power of their respective order (1 in ASF **11**, 0 in base, 0 in amine) times the difference in time is linear (Fig. **S3**), suggesting a correct reaction order, as described by Burés.[8]

Variable Time Normalization Analysis (VTNA)[8] was employed to determine the kinetic constant *k*, activation energy E_A and the half-life $t_{1/2}$ of ASF **11**. First, the amination of ASF **11** with morpholine was monitored by *in situ* ^{1}H NMR at three different temperatures (40, 60 and 70 ºC, Fig. **S4**).

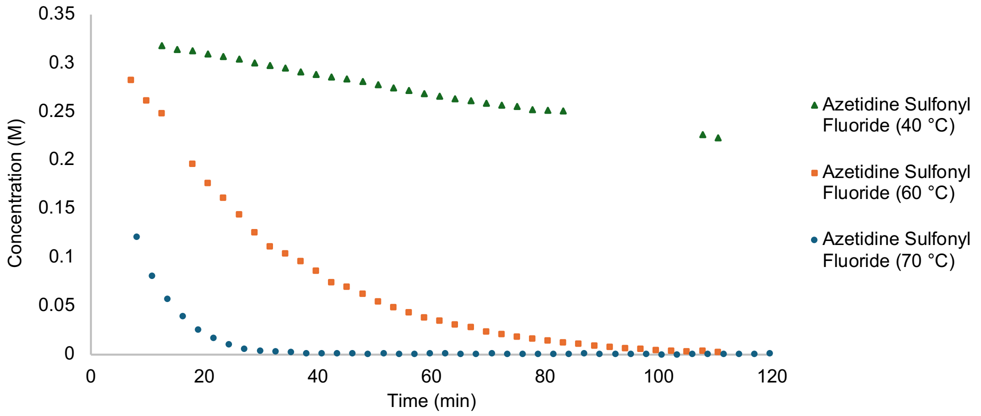

Fig. S4 Consumption of ASF **11** at 40 °C, 60 °C and 70 °C.

The kinetic constant of the rate-determining step of the reaction (k_{Obs}) was then determined by plotting the sum of all components to the power of their respective order times the difference in time, as described by Burés.[8] (Fig. **S3**):

$$\sum_{i=1}^{n}\left(\frac{[\mathrm{ASF}]_i + [\mathrm{ASF}]_{i-1}}{2}\right)^1 \left(\frac{[\mathrm{amine}]_i + [\mathrm{amine}]_{i-1}}{2}\right)^0 \left(\frac{[\mathrm{NEt_3}]_i + [\mathrm{NEt_3}]_{i-1}}{2}\right)^0 (t_i - t_{i-1}) =$$

$$\sum_{i=1}^{n}\left(\frac{[\mathrm{ASF}]_i + [\mathrm{ASF}]_{i-1}}{2}\right)^1 (t_i - t_{i-1})\,.$$

A linear regression was modelled on the curves with the (negative) slope equal to the kinetic constant of the reaction (see Table **S19** for values). A first order dependence on [ASF] **11** and zero order in all other components are in good accordance with an S_N1 mechanism and the formation of an azetidine carbocation.

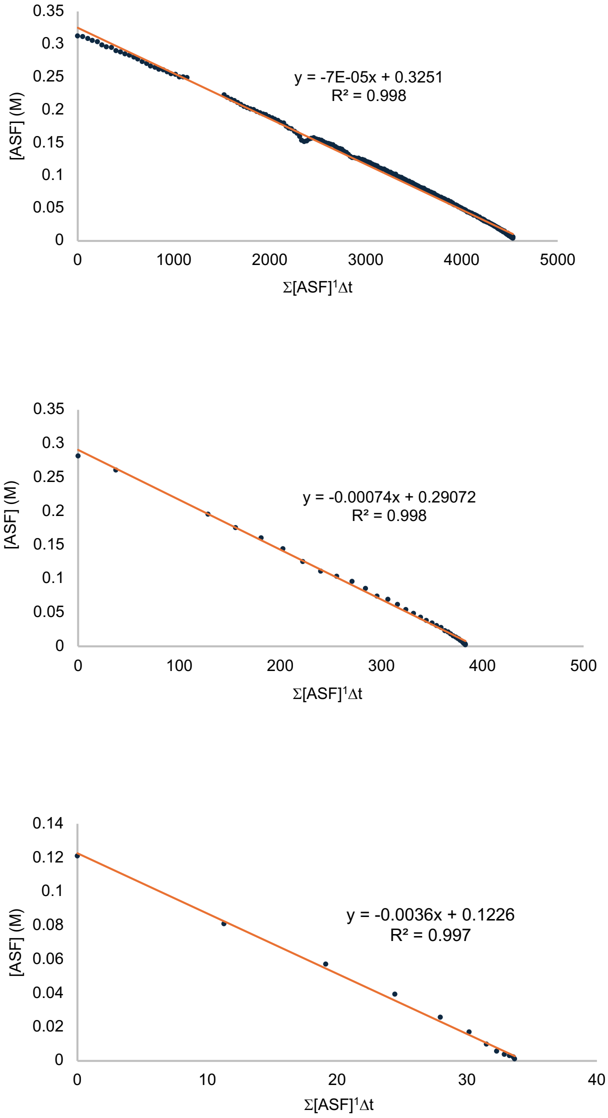

Fig. S3 VTNA analysis for the determination of k_{Obs}. *Top*: 40 ºC; *middle*: 60 ºC; *bottom*: 70 ºC. *t* is given in seconds.

Arrhenius Plot and Determination of E$_A$ and Half-Lives

The half-life $t_{1/2}$ of ASF **11** at different temperatures was then calculated (Table **S19**). The defluorosulfonylation of ASF **11** is a first order reaction and $t_{1/2}$ can be derived from the integrated rate law to:

$$t_{\frac{1}{2}} = \frac{\ln(2)}{k}.$$

Table S19 Half-lives of ASF **11** at different temperatures. The depicted errors correspond to the 95% confidence interval with *n* degrees of freedom calculated from the standard deviation times the factor t_s.[1]

Temperature / °C	k / 10^{-3} min^{-1}	$t_{1/2}$
70	213 ± 8	3.37 ± 0.13 min
60	44.3 ± 0.7	15.7 ± 0.3 min
40	4.164 ± 0.022	166.4 ± 1.0 min

An Arrhenius plot (ln(k) vs 1/T) was generated using the Arrhenius equation and the obtained kinetic constants (Fig. **S5**):

$$\ln(k) = \ln(A) - \frac{E_A}{R}\left(\frac{1}{T}\right),$$

A 'weighed' linear regression was modelled on the data points since each value has its own standard deviation. In this way, values with a smaller error are given more significance in the regression. The standard deviation of the ln(k) values was calculated from the standard deviation of the original k_{Obs} values (given by the standard deviation of the linear regression) using the conventional Gaussian equation for error propagation:

$$s_z^2 = \left(\frac{\partial f}{\partial a}\right)^2 s_a^2 + \left(\frac{\partial f}{\partial b}\right)^2 s_b^2 + \left(\frac{\partial f}{\partial c}\right)^2 s_c^2 + \cdots,$$

whereby s_z^2 is the squared standard deviation of the new function z ($z = f(a,b,c,\ldots)$) which depends on the values $a,b,c,\ldots$ and their squared standard deviations s_a^2, s_b^2, s_c^2 ... The weighed linear regression was calculated using the following formulae[9]:

$$intercept\ a = \frac{\sum_{i=1}^{n}\frac{x_i^2}{s_i^2}\sum_{i=1}^{n}\frac{y_i}{s_i^2} - \sum_{i=1}^{n}\frac{x_i}{s_i^2}\sum_{i=1}^{n}\frac{x_i y_i}{s_i^2}}{\sum_{i=1}^{n}\frac{1}{s_i^2}\sum_{i=1}^{n}\frac{x_i^2}{s_i^2} - \left(\sum_{i=1}^{n}\frac{x_i}{s_i^2}\right)^2}$$

$$slope\ b = \frac{\sum_{i=1}^{n}\frac{1}{s_i^2}\sum_{i=1}^{n}\frac{x_i y_i}{s_i^2} - \sum_{i=1}^{n}\frac{x_i}{s_i^2}\sum_{i=1}^{n}\frac{y_i}{s_i^2}}{\sum_{i=1}^{n}\frac{1}{s_i^2}\sum_{i=1}^{n}\frac{x_i^2}{s_i^2} - \left(\sum_{i=1}^{n}\frac{x_i}{s_i^2}\right)^2}.$$

[1]For linear regressions, t_s was determined using the degrees of freedom $\nu = n - 2$, whereby n is the number of data points. 2 degrees of freedom are lost due to the linear regression because the intercept and slope also depend on the data points n.

The standard deviation of intercept and slope were calculated using the error propagation formula:

$$s_a^2 = \frac{\sum_{i=1}^{n} \frac{x_i^2}{s_i^2}}{\sum_{i=1}^{n} \frac{1}{s_i^2} \sum_{i=1}^{n} \frac{x_i^2}{s_i^2} - \left(\sum_{i=1}^{n} \frac{x_i}{s_i^2}\right)^2}$$

$$s_b^2 = \frac{\sum_{i=1}^{n} \frac{1}{s_i^2}}{\sum_{i=1}^{n} \frac{1}{s_i^2} \sum_{i=1}^{n} \frac{x_i^2}{s_i^2} - \left(\sum_{i=1}^{n} \frac{x_i}{s_i^2}\right)^2}.$$

And the correlation coefficient R^2:

$$R^2 = \left(\frac{\sum_{i=1}^{n} \frac{1}{s_i^2} \sum_{i=1}^{n} \frac{x_i y_i}{s_i^2} - \sum_{i=1}^{n} \frac{x_i}{s_i^2} \sum_{i=1}^{n} \frac{y_i}{s_i^2}}{\sqrt{\sum_{i=1}^{n} \frac{1}{s_i^2} \sum_{i=1}^{n} \frac{x_i^2}{s_i^2} - \left(\sum_{i=1}^{n} \frac{x_i}{s_i^2}\right)^2} \sqrt{\sum_{i=1}^{n} \frac{1}{s_i^2} \sum_{i=1}^{n} \frac{y_i^2}{s_i^2} - \left(\sum_{i=1}^{n} \frac{y_i}{s_i^2}\right)^2}} \right)^2.$$

The activation energy is equal to the slope multiplied by the universal gas constant *R*, according to the Arrhenius equation. The error was calculated as described for the kinetic constants. The activation energy of ca. 107 kJ mol^{-1} is well in line with the observed reactivity.

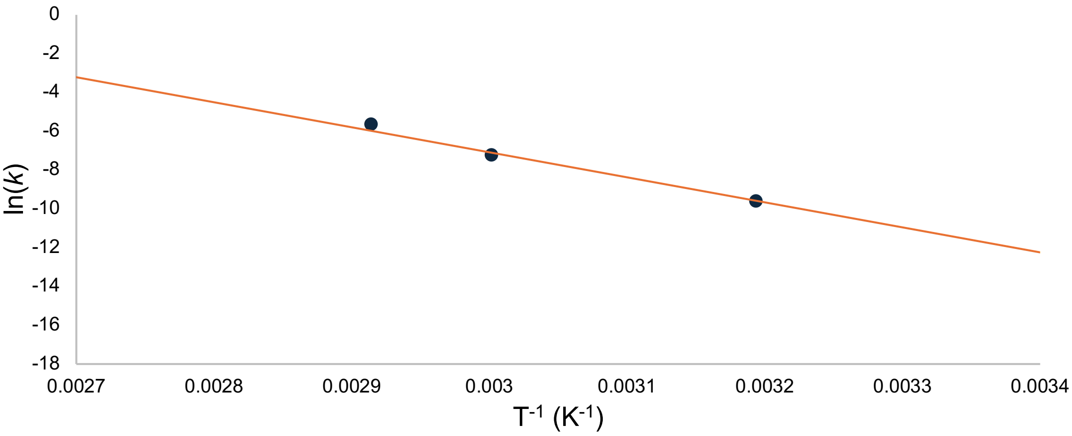

Fig. S5 Arrhenius plot to calculate the activation energy. ln refers to the natural logarithm. The equation and correlation coefficient R^2 are given for the modelled weighed linear regression. The error of E_A represents the 95% confidence interval, calculated from the standard deviation of the linear regression (s_b^2, *vide supra*) times the factor t_s.

Comparison between the kinetics of ASF 11 and OSF 1

The reaction profiles of ASF **11** and OSF **1**, as well as the derived kinetic values are very similar (Fig. **S6** and Table **S20**), reflecting the comparable reactivity observed with amine nucleophiles (see Scheme **3** in main text). Even the activation energy, calculated using the Arrhenius equation with only three values, differs only by 10 ± 6 kJ mol^{-1}. In previous works we have shown NCbz azetidines to show comparable reactivity to analogous oxetane substrates. This includes dehydration reactions of benzylic NCbz azetidinols and oxetanols with different nucleophiles such as aromatics,[10,11,12] thiols[13,14] and alcohols.[15] In this study we make comparable observations between azetidine and oxetane sulfonyl fluorides, now supported by experimental kinetic data. Taken together, these synthetic and kinetic data suggest that the reactivity of benzylic NCbz azetidine and oxetane carbocations, the proposed intermediates in the abovementioned studies, is nearly identical. This observation is important to note for the development of future synthetic methods that involve benzylic oxetane and azetidine carbocations: a reaction developed for oxetane substrates will most likely behave comparably with analogous NCbz azetidine compounds. The only aspect to bear in mind is that oxetanes are more prone to ring-opening than azetidines, especially by internal nucleophiles.[12,16]

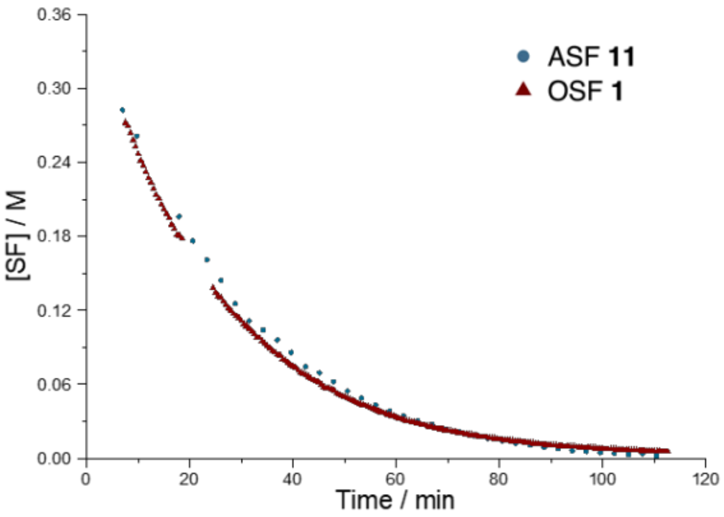

Fig. S6 Comparison of the rate of sulfonyl fluoride consumption between ASF **11** and OSF **1** in the reaction with morpholine at 60 ºC. Data for OSF **1** taken from ref. 6.

Table S20 Comparison of kinetic values between ASF **11** and OSF **1**.[6]

	ASF **11**	OSF **1**	Δ
k (70 °C) / 10^{-3} min^{-1}	213 ± 8	144.2 ± 1.3	69 ± 9
k (60 °C) / 10^{-3} min^{-1}	44.3 ± 0.7	39.7 ± 0.6	4.6 ± 1.3
k (40 °C) / 10^{-3} min^{-1}	4.164 ± 0.022	4.52 ± 0.03	0.36 ± 0.05
$t_{1/2}$ (70 °C) / min	3.37 ± 0.13	4.81 ± 0.04	1.44 ± 0.17
$t_{1/2}$ (60 °C) / min	15.7 ± 0.3	17.46 ± 0.03	1.8 ± 0.3
$t_{1/2}$ (40 °C) / min	166.4 ± 1.0	153.4 ± 0.9	13.0 ± 1.9
E_A / kJ mol^{-1}	107 ± 4	97.2 ± 1.6	10 ± 6

X-Ray Crystallography Details

Table **S21** provides a summary of the crystallographic data for the structures of **11**, **45**, **46**, **60'**, **75**, **80**, **84**, **87**, **93**, **96**, **100**, **135**, **136** and **141**. Data were collected using Agilent Xcalibur PX Ultra A [**11**, **84**, **87**, **96** and **100**] and Agilent Xcalibur 3 E [**45**, **46**, **60'**, **75**, **80**, **93**, **135**, **136** and **141**] diffractometers, and the structures were solved and refined using the OLEX2,[17] SHELXTL[18] and SHELX-2013[19] program systems. Whilst the absolute structure of **11** was determined by use of the Flack parameter [x = –0.02(3)], those of **45**, **60'**, **80**, **93** and **141**, could not be determined [Flack parameter x = –0.2(9), –0.4(10), 0.41(12), –0.05(4) and –0.2(5) respectively] and so arbitrary assignments were made. CCDC Deposition Numbers: 2388139–2388152.

Table S21 Crystal Data, Data Collection and Refinement Parameters for the crystal structures.

data	11	45	46	60'
**formula**	$C_{18}H_{18}FNO_5S$	$C_{19}H_{18}N_2O_2$	$C_{16}H_{14}N_2O_2$	$C_{12}H_{14}N_4O_2$
**formula weight**	379.39	306.35	266.29	246.27
**colour, habit**	colourless blocky needles	colourless blocky needles	colourless prisms	colourless blocks
**temperature / K**	173	173	173	173
**crystal system**	orthorhombic	monoclinic	orthorhombic	orthorhombic
**space group**	$Pna2_1$ (no. 33)	$P2_1$ (no. 4)	$Pbca$ (no. 61)	$P2_12_12_1$ (no.
a **/ Å**	26.2882(6)	9.7178(6)	8.1251(3)	6.4642(4)
b **/ Å**	10.8213(3)	7.6965(3)	12.0516(5)	6.9967(5)
c **/ Å**	6.10789(14)	11.3432(6)	25.9140(12)	26.5508(15)
**α / deg**	90	90	90	90
**β / deg**	90	111.418(6)	90	90
**γ / deg**	90	90	90	90
V **/ Å^3**	1737.53(7)	789.81(8)	2537.53(19)	1200.84(13)
Z	4	2	8	4
D_c **/ g cm^{-3}**	1.450	1.288	1.394	1.362
**radiation used**	Cu-Kα	Mo-Kα	Mo-Kα	Mo-Kα
**μ / mm^{-1}**	2.024	0.085	0.094	0.097
**no. of unique reflns**				
**measured (R_{int})**	2635 (0.0356)	3467 (0.0406)	2648 (0.0227)	2365 (0.0271)
**obs, $\|F_o\| > 4\sigma(\|F_o\|)$**	2340	2801	2180	2039
**completeness (%)** [a]	99.5	99.8	99.7	98.9
**no. of variables**	236	208	186	166
R_1**(obs),** wR_2**(all)** [b]	0.0360, 0.0913	0.0448, 0.0998	0.0399, 0.0949	0.0437, 0.0926
**CCDC code**	2388139	2388140	2388141	2388142

[a] Completeness to 0.84 Å resolution. [b] $R_1 = \Sigma||F_o| - |F_c||/\Sigma|F_o|$; $wR_2 = \{\Sigma[w(F_o^2 - F_c^2)^2] / \Sigma[w(F_o^2)^2]\}^{1/2}$; $w^{-1} = \sigma^2(F_o^2) + (aP)^2 + bP$. [c] There are two crystallographically independent molecules.

data	75	80	84	87
**formula**	$C_{23}H_{22}BrNO_3S$	$C_{14}H_{21}O_5P$	$C_{20}H_{25}O_5P$	$C_{12}H_{17}O_4P$
**formula weight**	472.38	300.28	376.37	256.22
**colour, habit**	colourless blocky needles	colourless blocks	colourless needles	colourless needles
**temperature / K**	173	173	173	173
**crystal system**	monoclinic	monoclinic	monoclinic	monoclinic
**space group**	$P2_1/n$ (no. 14)	*Cc* (no. 9)	$P2_1/n$ (no. 14)	$P2_1/c$ (no. 14)
a **/ Å**	13.27773(19)	9.7902(2)	8.85189(11)	5.65929(19)
b **/ Å**	10.62347(13)	15.1330(4)	8.83613(13)	30.4782(9)
c **/ Å**	15.0506(2)	20.1143(5)	24.9789(3)	14.7341(4)
**α / deg**	90	90	90	90
**β / deg**	104.0634(14)	93.835(2)	98.0605(12)	90.385(3)
**γ / deg**	90	90	90	90
**V / Å^3**	2059.33(5)	2973.34(12)	1934.46(4)	2541.35(13)
Z	4	8 [c]	4	8 [c]
**D_c / g cm^{-3}**	1.524	1.342	1.292	1.339
**radiation used**	Mo-Kα	Mo-Kα	Cu-Kα	Cu-Kα
**μ / mm^{-1}**	2.122	0.201	1.492	1.947
**no. of unique reflns**				
**measured (R_{int})**	4897 (0.0390)	6355 (0.0234)	3900 (0.0525)	5026 (0.0699)
**obs, $\|F_o\| > 4\sigma(\|F_o\|)$**	4131	5754	3132	3378
**completeness (%)** [a]	99.9	100	100	100
**no. of variables**	263	368	254	313
**R_1(obs), wR_2(all)** [b]	0.0349, 0.0789	0.0399, 0.1010	0.0460, 0.1230	0.0432, 0.1110
**CCDC code**	2388143	2388144	2388145	2388146

data	93	96	100	135
**formula**	$C_{11}H_{14}O_4S$	$C_{14}H_{19}NO_5S$	$C_{13}H_{14}N_2O_4S$	$C_{12}H_{13}FO_4S$
**formula weight**	242.28	313.36	294.32	272.28
**colour, habit**	colourless blocks	colourless blocky needles	colourless blocky needles	colourless blocks
**temperature / K**	173	173	173	173
**crystal system**	orthorhombic	Orthorhombic	monoclinic	triclinic
**space group**	$Pca2_1$ (no. 29)	Pbca	$P2_1/n$ (no. 14)	P–1 (no. 2)
***a* / Å**	7.6918(3)	6.54306(14)	11.58714(19)	8.1812(5)
***b* / Å**	15.6103(6)	17.6671(4)	6.03319(9)	10.3380(5)
***c* / Å**	18.4280(7)	25.0121(6)	19.9000(3)	16.2097(7)
**α / deg**	90	90	90	101.050(4)
**β / deg**	90	90	101.5023(15)	90.271(4)
**γ / deg**	90	90	90	111.699(5)
V / Å^3	2212.66(14)	1891.56(11)	1363.22(4)	1245.90(12)
Z	8 [c]	8	4	4 [c]
D_c / g cm^{-3}	1.455	1.440	1.434	1.452
**radiation used**	Mo-Kα	Cu-Kα	Cu-Kα	Mo-Kα
**μ / mm^{-1}**	0.288	2.195	2.262	0.276
**no. of unique reflns**				
**measured (R_{int})**	3840 (0.0210)	2820	2707 (0.0320)	9435 (0.0677)
**obs, $\|F_o\| > 4\sigma(\|F_o\|)$**	3471	2249	2405	4898
**completeness (%)** [a]	99.5	99.8	99.8	99.6
**no. of variables**	293	191	183	348
R_1**(obs),** wR_2**(all)** [b]	0.0453, 0.1132	0.0362,	0.0323, 0.0855	0.0415, 0.0988
**CCDC code**	2388147	2388148	2388149	2388150

data	**136**	**141**
**formula**	$C_{13}H_{15}FO_6S$	$C_{19}H_{21}FN_2O_3$
**formula weight**	318.31	344.38
**colour, habit**	colourless prisms	colourless prisms
**temperature / K**	173	173
**crystal system**	monoclinic	triclinic
**space group**	*C*2/*c* (no. 15)	*P*1 (no. 1)
a **/ Å**	23.2520(8)	6.0449(4)
b **/ Å**	7.3085(3)	8.6154(4)
c **/ Å**	16.4501(6)	17.1068(9)
**α / deg**	90	77.719(4)
**β / deg**	94.197(4)	81.542(5)
**γ / deg**	90	86.536(5)
V **/ Å^3**	2788.01(17)	860.67(8)
Z	8	2 [c]
D_c **/ g cm^{-3}**	1.517	1.329
**radiation used**	Mo-Kα	Mo-Kα
**μ / mm^{-1}**	0.269	0.098
**no. of unique reflns**		
**measured (R_{int})**	2924 (0.0219)	6957 (0.0263)
**obs, $\|F_o\| > 4\sigma(\|F_o\|)$**	2516	5830
**completeness (%)** [a]	99.9	99.8
**no. of variables**	191	488
R_1**(obs),** wR_2**(all)** [b]	0.0343, 0.0869	0.0480, 0.1087
**CCDC code**	2388151	2388152

Comments on Crystal Structures

Whilst the absolute structure of **11** was determined by use of the Flack parameter [*x* = –0.02(3)], those of **45** [*x* = –0.2(9)], **60’** [*x* = –0.4(10)], **80** [*x* = 0.41(12)], **93** [*x* = –0.05(4)], and **141** [*x* = –0.2(5)] could not and so arbitrary assignments were made.

The O11–H hydrogen atom in the structure of **46** was located from a Δ*F* map and refined freely subject to an O–H distance constraint of 0.90 Å. The structure of **80** was found to contain two crystallographically independent molecules (**80-A** and **80-B**) in the asymmetric unit. The O20- and O23-bound ethyl groups in the structure of **84** were found to be disordered. For the former, three orientations were identified of *ca*. 39, 37 and 24% occupancy, whilst for the latter two orientations were identified of *ca*. 71 and 29% occupancy. The geometries of each set of orientations were optimised, the thermal parameters of adjacent atoms were restrained to be similar, and only the non-hydrogen atoms of the major occupancy orientation of the O23-bound ethyl group were refined anisotropically (those of all of the minor occupancy orientations were refined isotropically). The structure of **87** was found to contain two crystallographically independent molecules (**87-A** and **87-B**) in the asymmetric unit. The structure of **93** was found to contain two crystallographically independent molecules (**93-A** and **93-B**) in the asymmetric unit.

The crystal of **135** that was studied was found to be a two component twin in a *ca*. 81:19 ratio, with the two lattices related by the approximate twin law [0.67 0.39 –0.33 0.00 –1.00 0.00 –1.67 –0.39 –0.67]. The structure was found to contain two crystallographically independent molecules (**135-A** and **135-B**) in the asymmetric unit. The O11-bound allyl group in molecule **135-B** was found to be disordered. Two orientations were identified of *ca*. 64 and 36% occupancy, their geometries were optimised, the thermal parameters of adjacent atoms were restrained to be similar, and only the non-hydrogen atoms of the major occupancy orientation were refined anisotropically (those of the minor occupancy orientation were refined isotropically). The SO_2F group in molecule **135-B** was found to be disordered in a manner that suggested distribution of the fluorine over the F16B and O17B sites in a *ca*. 62:38 ratio. Partial occupancy fluorine and oxygen atoms were modelled at each site, the bond lengths were restrained to appropriate distances, the thermal parameters of adjacent atoms were restrained to be similar, and only the atoms of the major occupancy orientation were refined anisotropically (those of the minor occupancy orientation were refined isotropically).

The structure of **141** was found to contain two crystallographically independent molecules (**141-A** and **141-B**) in the asymmetric unit. In both independent molecules the position of the fluorine atom of the C12-bound fluorophenyl group was found to be disordered across the two *meta* positions (*i.e.* C15 and C17). Partial occupancy atoms were modelled at each site in *ca*. 60:40 and 80:20 ratios for the C15 and C17 sites in molecules **141-A** and **141-B** respectively, with only the major occupancy fluorine atoms refined anisotropically (the minor occupancy atoms were refined isotropically). All six of the N–H hydrogen atoms across the two independent molecules were located from Δ*F* maps and refined freely subject to N–H distance constraints of 0.90 Å.

ORTEP Renders of Crystal Structures

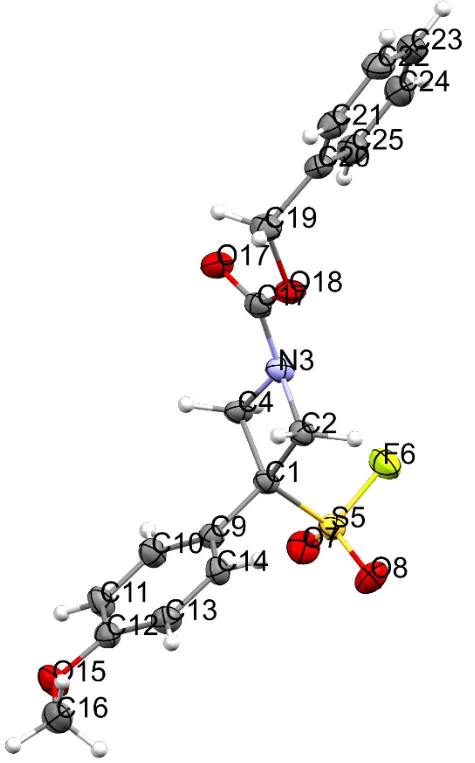

Fig. S7 The crystal structure of **11** (50% probability ellipsoids).

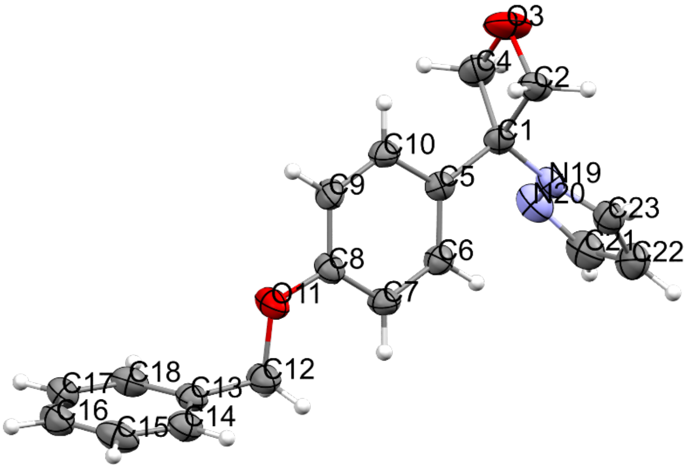

Fig. S8 The crystal structure of **45** (50% probability ellipsoids).

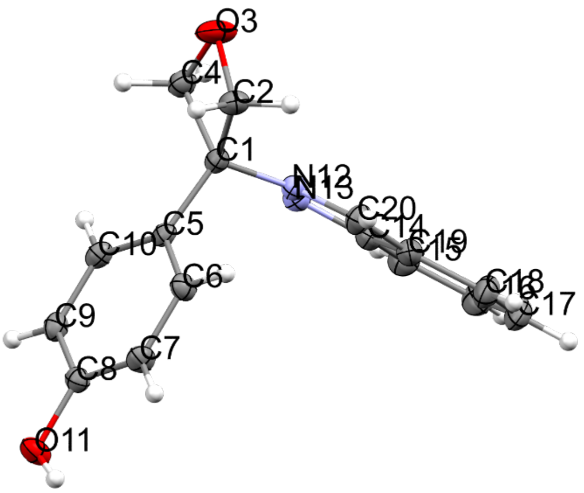

Fig. S9 The crystal structure of **46** (50% probability ellipsoids).

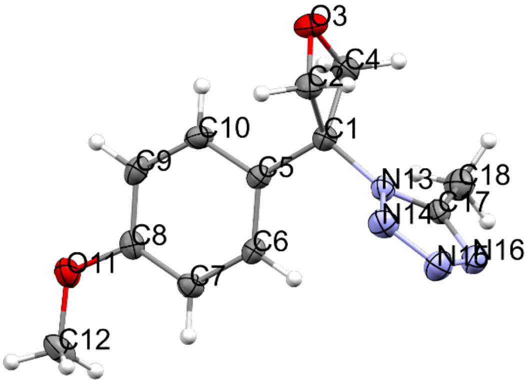

Fig. S10 The crystal structure of **60’** (50% probability ellipsoids).

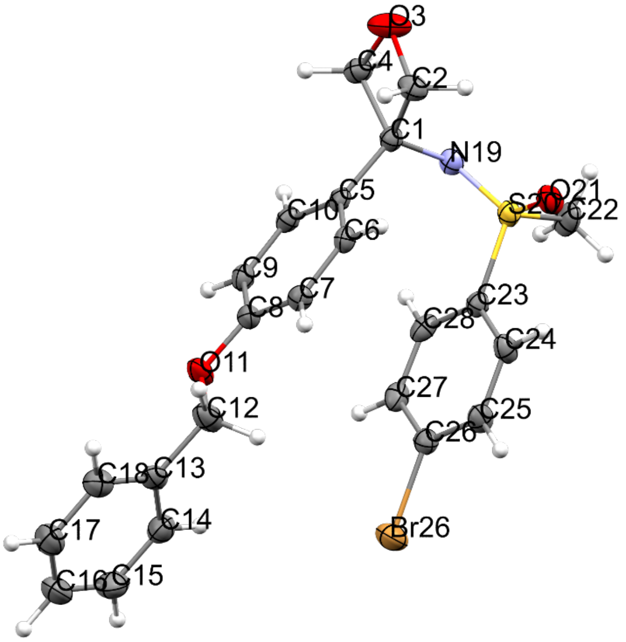

Fig. S11 The crystal structure of **75** (50% probability ellipsoids).

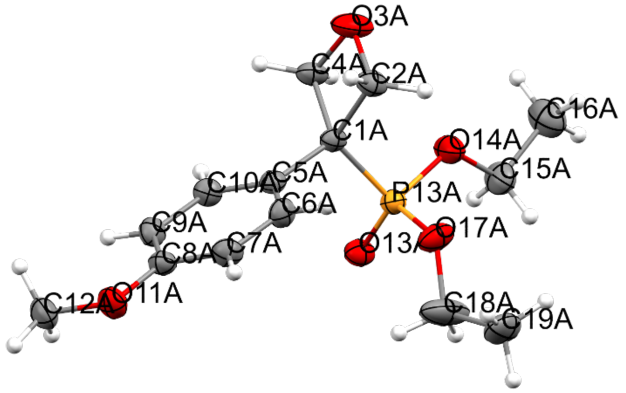

Fig. S12 The structure of **80-A**, one of the two independent molecules present in the crystal of **80** (50% probability ellipsoids).

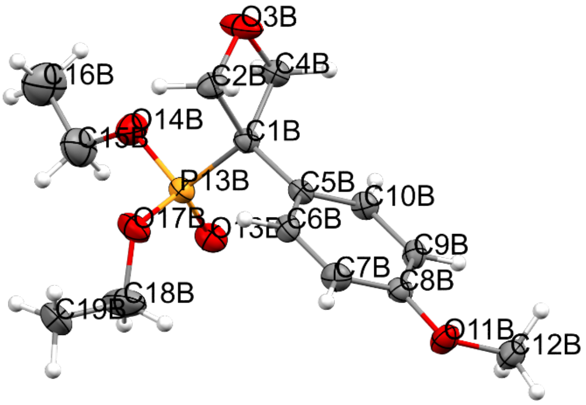

Fig. S13 The structure of **80-B**, one of the two independent molecules present in the crystal of **80** (50% probability ellipsoids).

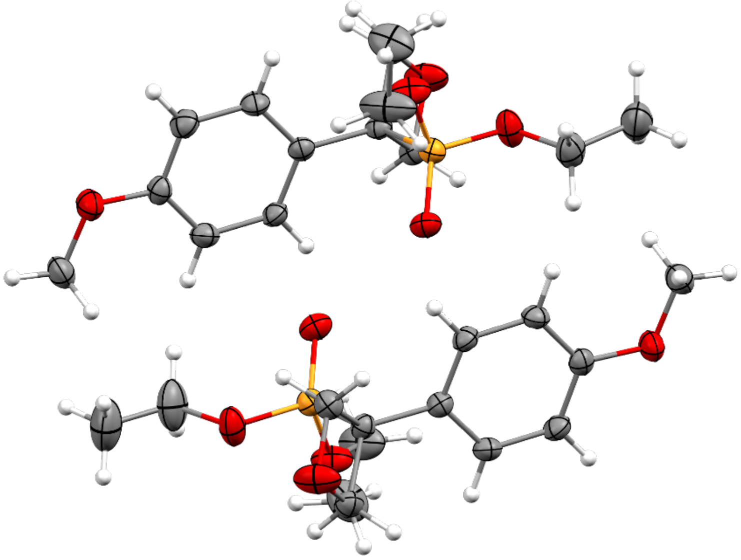

Fig. S14 The structure of the asymmetric unit of **80** (50% probability ellipsoids).

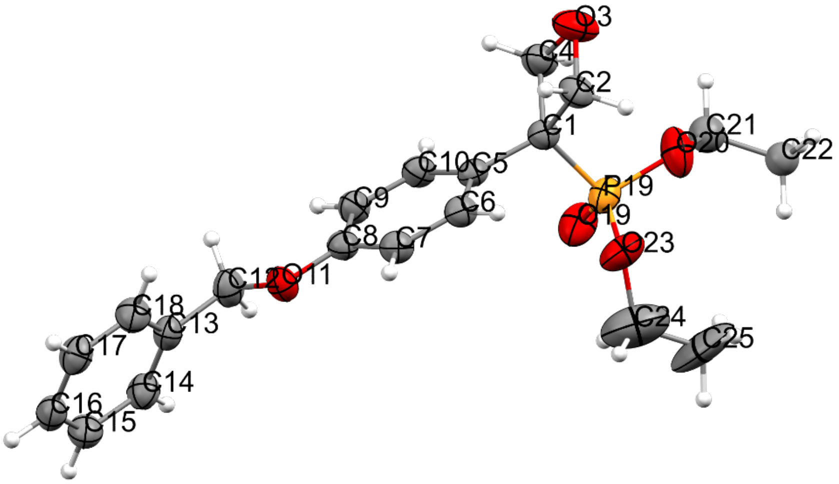

Fig. S15 The crystal structure of **84** (50% probability ellipsoids).

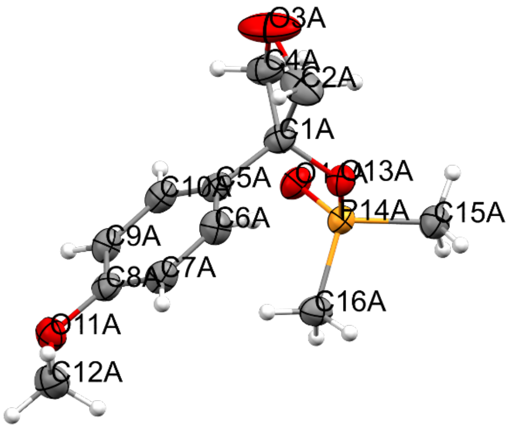

Fig. S16 The structure of **87-A**, one of the two independent molecules present in the crystal of **87** (50% probability ellipsoids).

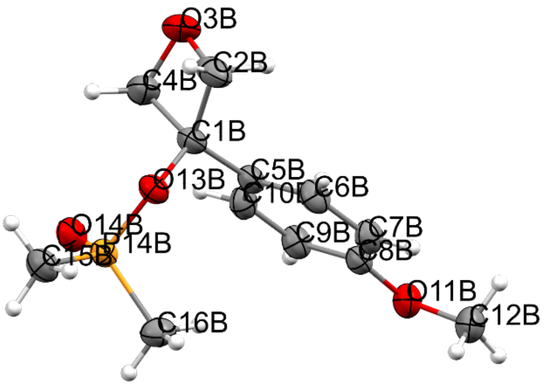

Fig. S17 The structure of **87-B**, one of the two independent molecules present in the crystal of **87** (50% probability ellipsoids).

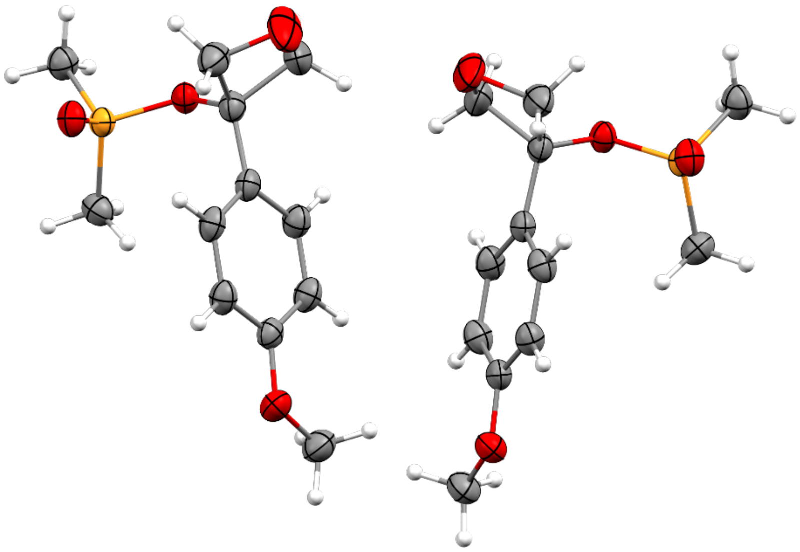

Fig. S18 The structure of the asymmetric unit of **87** (50% probability ellipsoids).

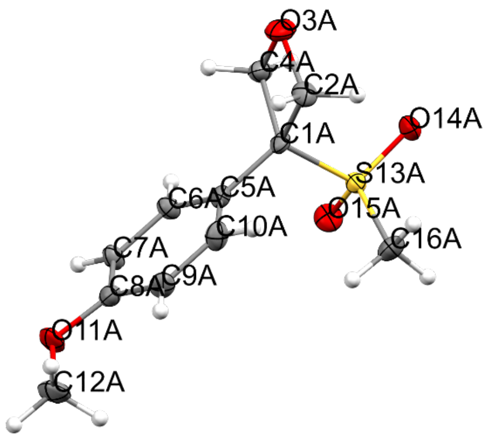

Fig. S19 The structure of **93-A**, one of the two independent molecules present in the crystal of **93** (50% probability ellipsoids).

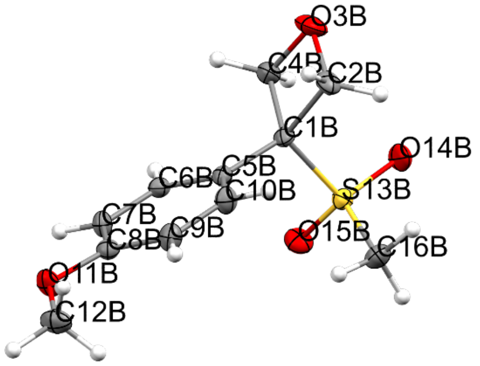

Fig. S20 The structure of **93-B**, one of the two independent molecules present in the crystal of **93** (50% probability ellipsoids).

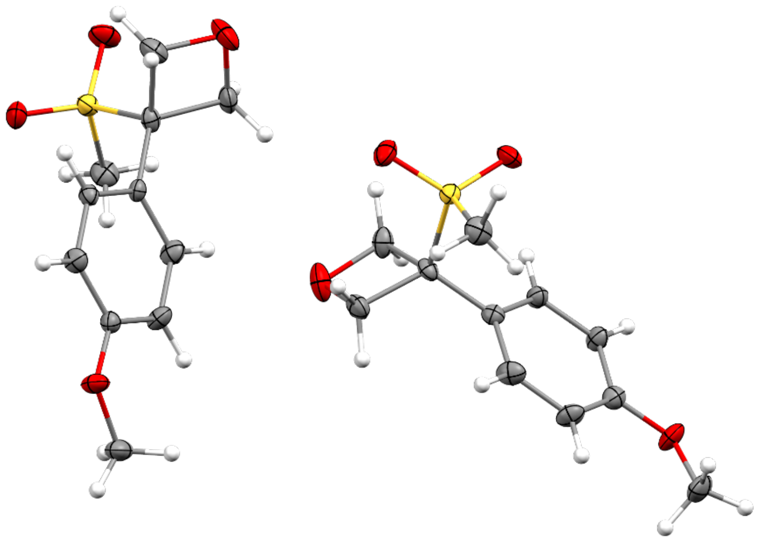

Fig. S21 The structure of the asymmetric unit of **93** (50% probability ellipsoids).

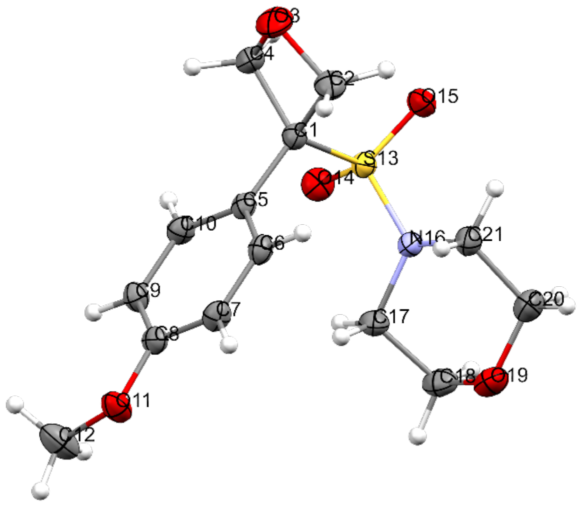

Fig. S22 The crystal structure of **96** (50% probability ellipsoids).

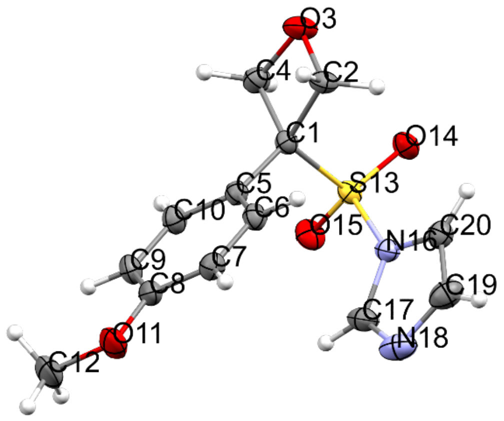

Fig. S23 The crystal structure of **100** (50% probability ellipsoids).

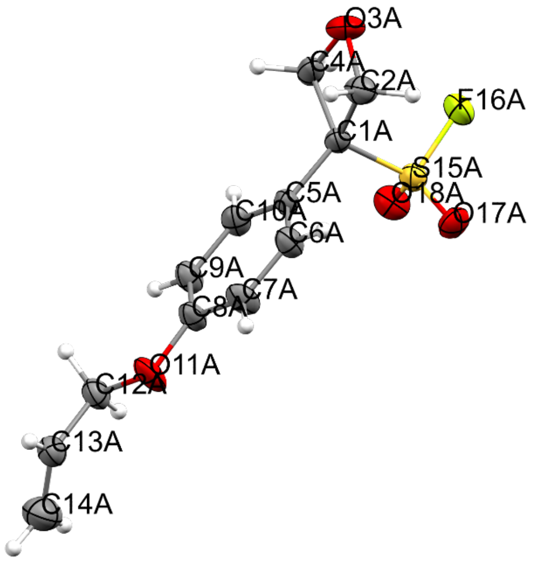

Fig. S24 The structure of **135-A**, one of the two independent molecules present in the crystal of **135** (50% probability ellipsoids).

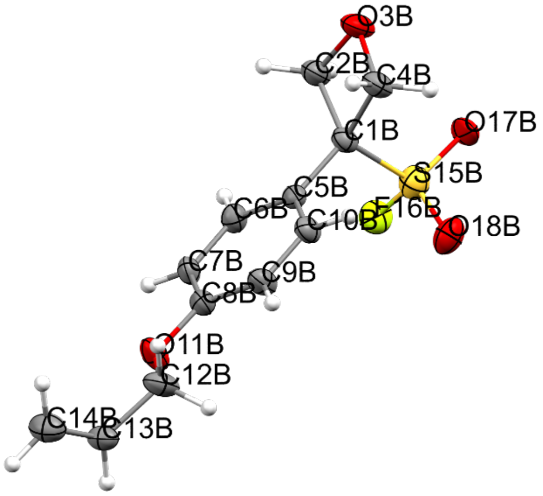

Fig. S25 The structure of **135-B**, one of the two independent molecules present in the crystal of **135** (50% probability ellipsoids, disorder removed O16``, F17``, C12``, C13`` and C14`` for clarity).

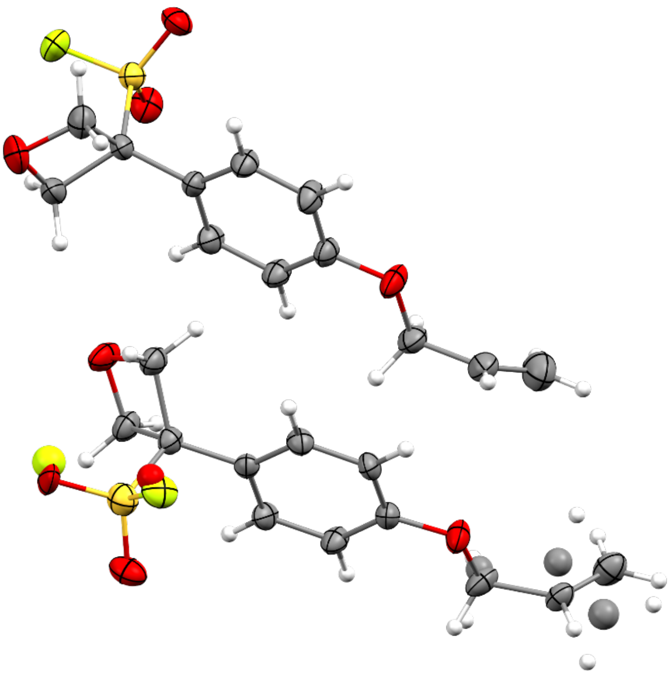

Fig. S26 The structure of the asymmetric unit of **135** (50% probability ellipsoids).

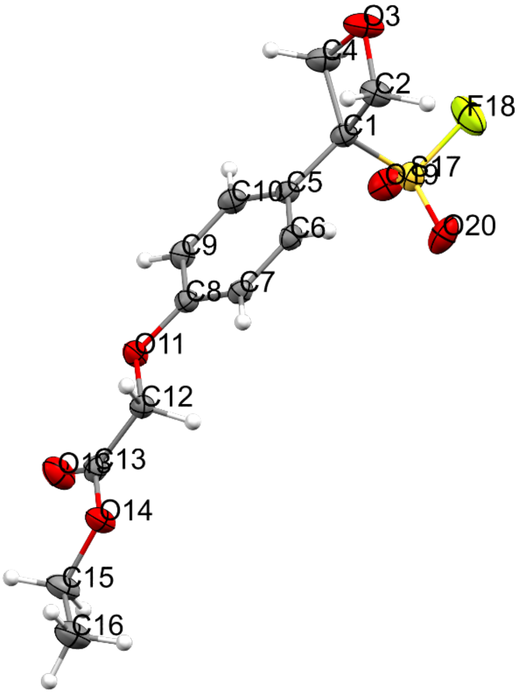

Fig. S27 The crystal structure of **136** (50% probability ellipsoids).

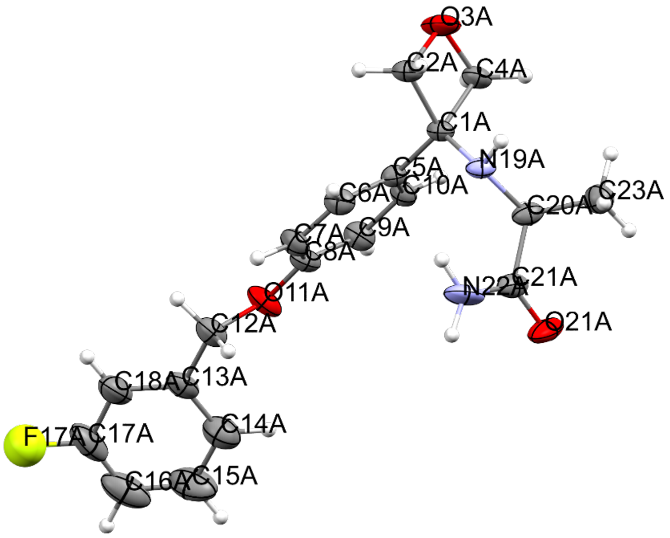

Fig. S28 The structure of **141-A**, one of the two independent molecules present in the crystal of **141** (50% probability ellipsoids, disorder removed F15A and H15A for clarity).

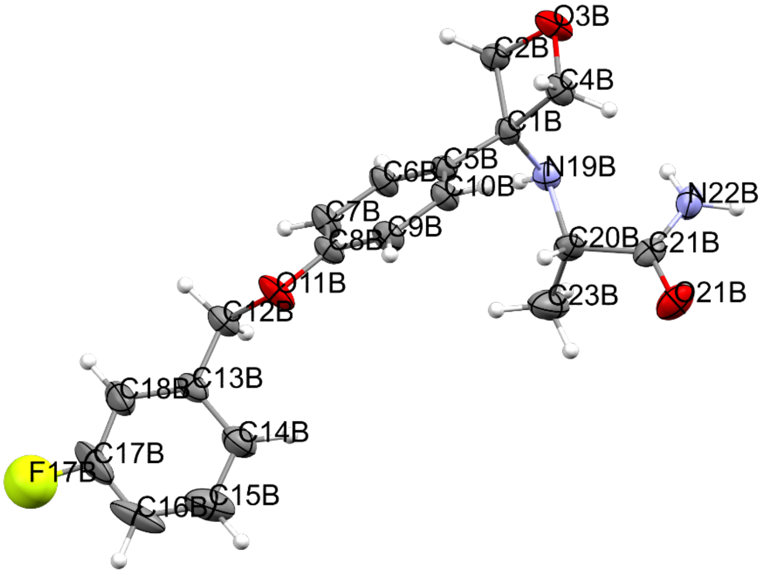

Fig. S29 The structure of **141-B**, one of the two independent molecules present in the crystal of **141** (50% probability ellipsoids, disorder removed F15B and H15B for clarity).

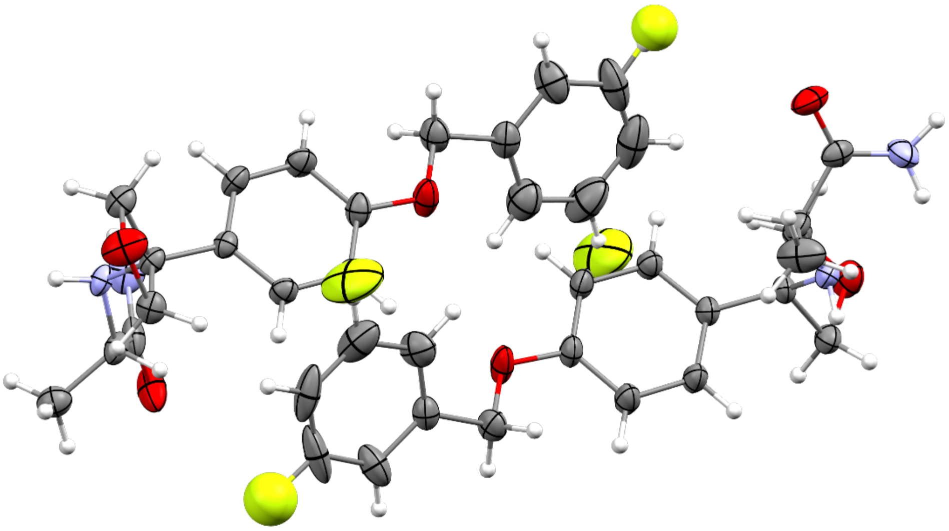

Fig. S30 The structure of the asymmetric unit of **141** (50% probability ellipsoids).

Crystal Structure Measurements

Dihedral angles and axial chirality

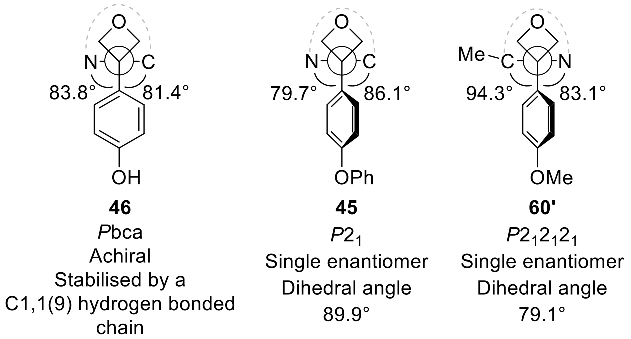

Fig. S31 Torsion and dihedral angles of **46** (left), **45** (middle) and **60’** (right) showing the how atropisomers are generated by the twisting of the 3,3` substituted aromatic rings and the measurement of the corresponding angle between the planes. Angles were measured in *Mercury* 2023.1.0 (Build 376230).

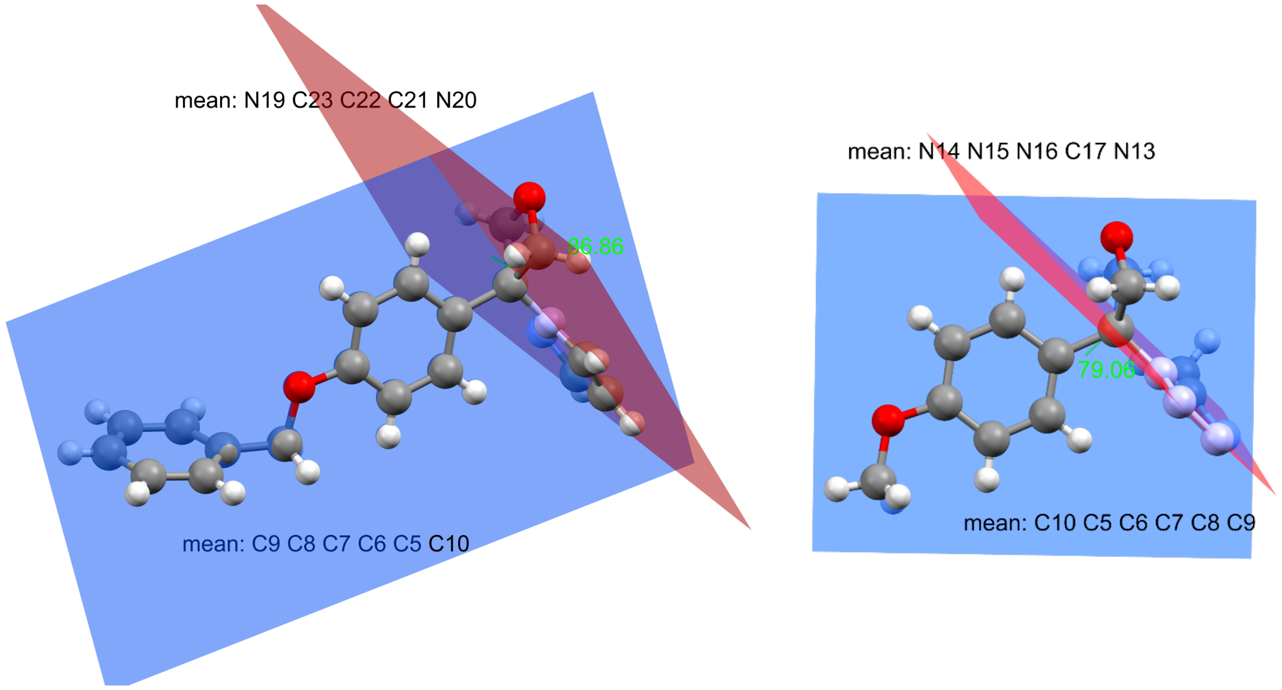

Fig. S32 The crystal structure of **45** (left) and **60’** (right) showing how atropisomers can be generated by the twisting of the 3,3` substituted aromatic rings and the measurement of the corresponding angle between the planes. Angles and planes were measured and generated in *Mercury* 2023.1.0 (Build 376230).

Turn Conformation

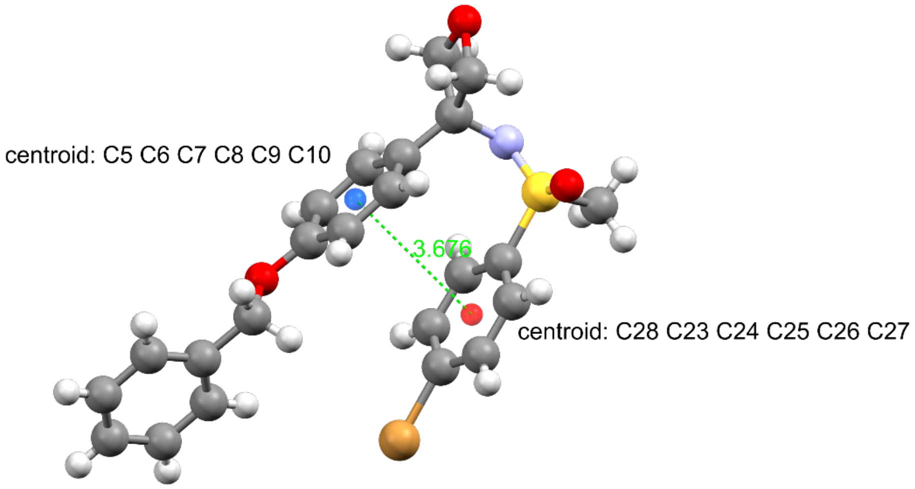

Fig. S33 The crystal structure of **75** and the measurement of the distance of π-stacking interactions via generation of centroids. Distances and centroids were measured and generated in *Mercury* 2023.1.0 (Build 376230).

Puckering and Out of Plane Angles

τ = torsion angle (1-2-3-4) ω = torsion angle (5-6-7-8)

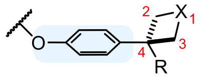

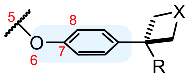

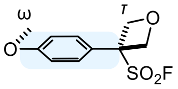

1 YANHAA
τ = puckering angle = 10.7(2)°
ω = "out of plane" angle = 8.4(2)°

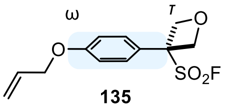

135
τ_A = 13.6(3)°
ω_A = 2.9(4)°
τ_B = 12.1(3)°
ω_B = 6.4(5)°

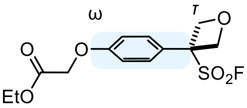

136
τ = 11.9(2)°
ω = 6.0(2)°

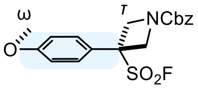

11
τ = 7.9(3)°
ω = 0.4(5)°

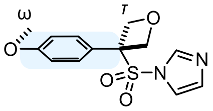

100
τ = 20.2(3)°
ω = 2.1(4)°

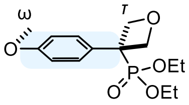

80
τ_A = 3.6(4)°
ω_A = 0.1(5)°
τ_B = 0.4(4)°
ω_B = 0.5(5)°

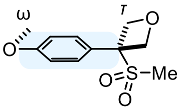

93
τ_A = 4.2(4)°
ω_A = 16.1(5)°
τ_B = 1.0(4)°
ω_B = 13.9(5)°

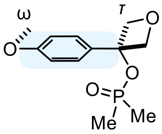

87
τ_A = 19.7(3)°
ω_A = 5.3(4)°
τ_B = 21.4(3)°
ω_B = 4.4(4)°

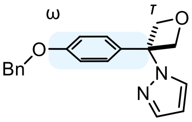

45
τ = 15.2(3)°
ω = 6.4(4)°

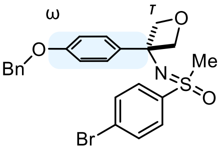

75
τ = 11.2(2)°
ω = 6.1(3)°

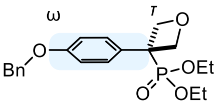

84
τ = 5.8(2)°
ω = 3.3(3)°

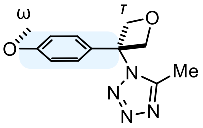

60`
τ = 21.2(3)°
ω = 2.1(4)°

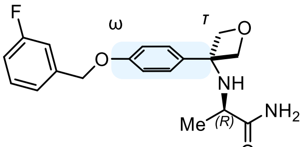

141
τ_A = 3.4(3)°
ω_A = 6.3(5)°
τ_B = 13.6(3)°
ω_B = 14.0(5)°

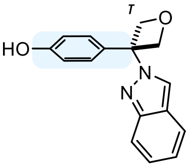

46
τ = 16.5(1)°

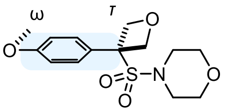

96
τ = 5.6(2)°
ω = 8.0(9)°

Fig. S34 Puckering angles (τ) and out of plane angles (ω) for para-aryl substituents of all crystal structures measured in *Mercury* 2023.1.0 (Build 376230). τ_A/ω_A and τ_B/ω_B refer to the angle measured for the stated crystallographically independent molecule in given crystal structures.

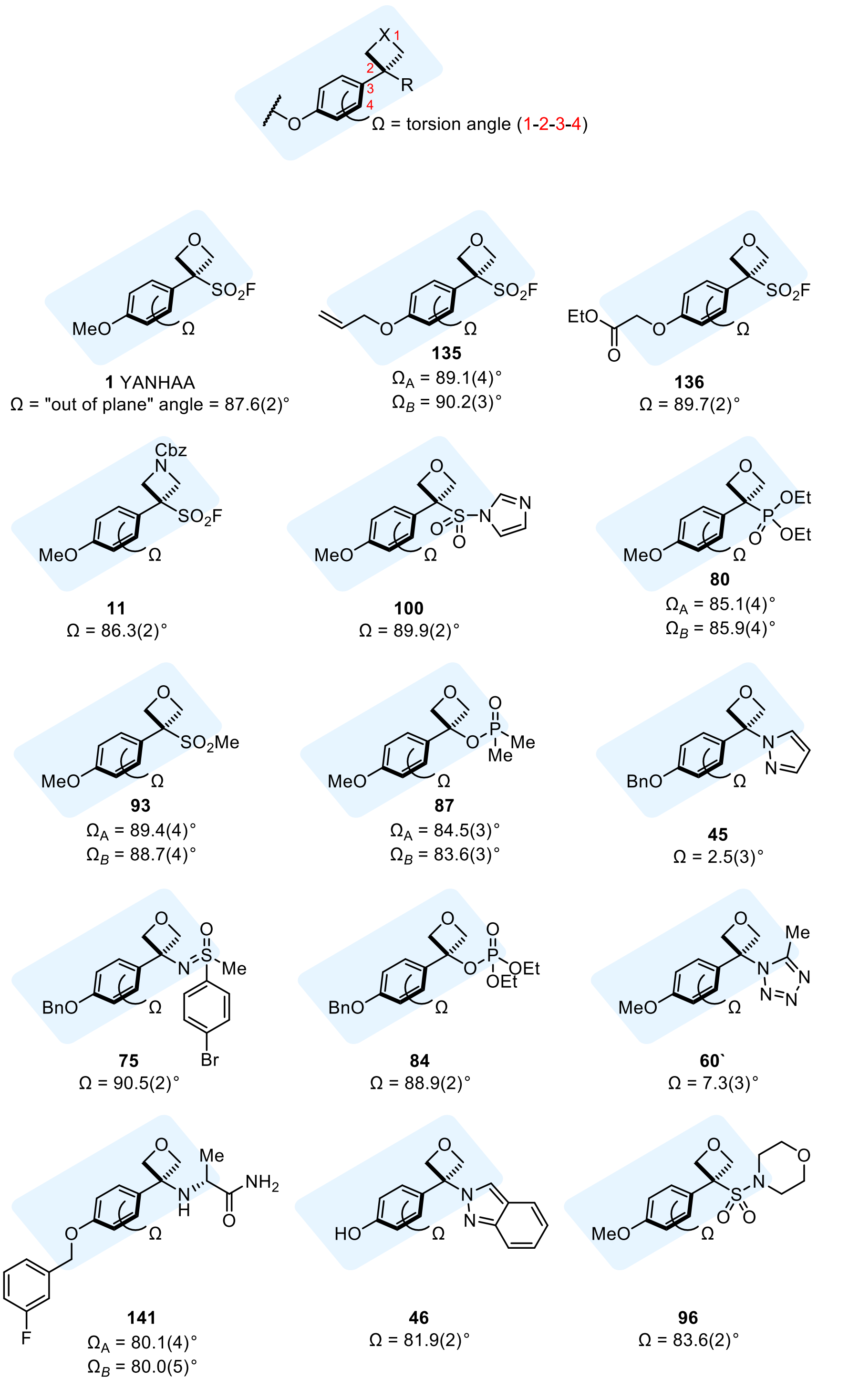

Fig. S35 Out of plane angles (Ω) of 4-membered rings versus the plane of the aromatic group (blue) of all crystal structures measured in *Mercury* 2023.1.0 (Build 376230). Ω_A and Ω_B refer to the angle measured for the stated crystallographically independent molecule in given crystal structures.

Experimental Details and Characterization Data

Oxetane Sulfonyl Fluorides (1–10, 132–140)

Oxetane Sulfonyl Fluoride 1

3-(4-Methoxyphenyl)oxetan-3-ol (1a)

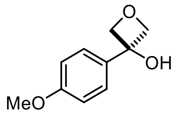

n-BuLi (1.59 M in hexanes, 100 mL, 160 mmol, 1.1 equiv) was added dropwise using a canula over 20 min to a solution of *p*-bromoanisole (21.8 mL, 174 mmol, 1.2 equiv) in anhydrous THF (660 mL, 0.22 M) at –78 °C in a 1 L 2-neck round bottom flask. After stirring for 1 h, oxetan-3-one (9.30 mL, 145 mmol, 1.0 equiv) was added dropwise to the reaction mixture. After a further 1 h of stirring at –78 °C, the reaction mixture was warmed up slowly to 25 °C over ~ 2 h and quenched with water (200 mL). The aqueous layer was extracted with Et_2O (3 × 200 mL). The organic layers were washed with brine (200 mL), dried over Na_2SO_4, filtered and concentrated *in vacuo* using a rotatory evaporator. Purification by recrystallization in toluene (8 volumes, 70 °C then 3 °C overnight) afforded oxetanol **1a** as a crystalline white solid (22.38 g, 86%). R_f = 0.20 (45% EtOAc/*n*-hexane); ^{1}H NMR (400 MHz, $CDCl_3$) δ 7.49–7.45 (m, 2 H, 2 × Ar-CH), 6.95–6.91 (m, 2 H, 2 × Ar-CH), 4.88 (d, *J* = 7.0 Hz, 2 H, C*H*HOC*H*H), 4.85 (d, *J* = 7.0 Hz, 2 H, CH*H*OCH*H*), 3.82 (s, 3 H, OCH_3), 3.32 (br s, 1 H, OH). The observed characterization data (R_f, ^{1}H) were consistent with that previously reported.[10]

Notes:

The rate of the dropwise addition of n-BuLi via the canula was controlled by employing a positive pressure on the bottle of n-BuLi with a balloon filled with argon.

One 100 mL bottle of n-BuLi was added to the reaction.

The crude material can alternatively be purified by flash column chromatography (45% EtOAc/n-hexane) and requires ~500 g of silica gel when performed on this scale.

Methyl 3-((3-(4-methoxyphenyl)oxetan-3-yl)thio)propanoate (1b)

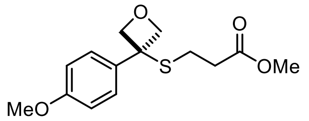

Prepared according to **oxetane thiol alkylation procedure 1 (OTA-1)**. Methyl 3-mercaptopropionate (6.65 mL, 60 mmol, 2.0 equiv) was added to a solution of $FeCl_3$ (487 mg, 3.0 mmol, 10 mol%) in anhydrous CH_2Cl_2 (60 mL, 0.5 M) in a 250 mL round-bottom flask. Oxetanol **1a** (5.40 g, 30 mmol, 1.0 equiv) was added to the resulting mixture and the flask was immediately placed into a pre-heated oil bath at 40 °C. The reaction was stirred at 40 °C for 20 min and quenched with sat. aq. $NaHCO_3$ (10 mL). The resulting mixture was filtered through cotton wool directly into a separatory funnel to remove residual $FeCl_3$ and aid phase separation during solvent-solvent extraction. The aqueous layer was extracted with CH_2Cl_2 (3 × 100 mL). The organic layers were washed with aq. NaOH (1 M, 100 mL), dried over Na_2SO_4, filtered and concentrated *in vacuo* using a rotatory evaporator. Purification by flash column chromatography (40% Et_2O/pentane) afforded oxetane sulfide **1b** as a white solid (6.25 g, 74%). R_f = 0.26 (50% Et_2O/pentane); ^{1}H NMR (400 MHz, $CDCl_3$) δ 7.16–7.12 (m, 2 H, 2 × Ar-CH), 6.92–6.89 (m, 2 H, 2 × Ar-CH), 5.19 (d, *J* = 6.5 Hz, 2 H, C*H*HOC*H*H), 4.90 (d, *J* = 6.5 Hz, 2 H, CH*H*OCH*H*), 3.83 (s, 3 H, Ar-C_qOCH_3), 3.68 (s, 3 H, CO_2CH_3), 2.67 (t, *J* = 7.4 Hz, 2 H, SCH_2), 2.42 (t, *J* = 7.4 Hz, 2 H, CH_2CO_2Me). The observed characterization data (R_f, ^{1}H) were consistent with that previously reported.[13]

Notes:

Oxetane sulfide can also be synthesized using ***oxetane thiol alkylation procedure 3 (OTA-3)***[6]

Methyl 3-((3-(4-methoxyphenyl)oxetan-3-yl)sulfonyl)propanoate (1c)

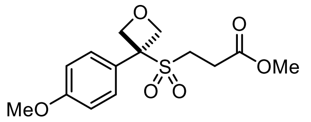

*m*CPBA (≤77%, 11.1 g, 49.5 mmol, 3.0 equiv) was added to a solution of oxetane sulfide **1b** (4.66 g, 16.5 mmol, 1.0 equiv) in CH_2Cl_2 (750 mL, 0.02 M) in a 2 L mL round-bottom flask. The reaction mixture was stirred at 25 °C for 3 h then aq. KOH (3 M, 200 mL) was added and the phases were separated. The aqueous portion was extracted with CH_2Cl_2 (4 × 100 mL). The organic extracts were combined, dried over Na_2SO_4, filtered and concentrated *in vacuo* using a rotatory evaporator to afford sulfone **1c** as a white solid (5.09 g, 98%); R_f = 0.59 (75% EtOAc/*n*-hexane); ^{1}H NMR (400 MHz, $CDCl_3$) δ 7.24–7.21 (m, 2 H, 2 × Ar-CH), 6.99–6.95 (m, 2 H, 2 × Ar-CH), 5.40 (d, *J* = 7.2 Hz, 2 H, C*H*HOC*H*H), 5.12 (d, *J* = 7.2 Hz, 2 H, CH*H*OCH*H*), 3.84 (s, 3 H, Ar-$C_q$$OCH_3$), 3.70 (s, 3 H, CO_2CH_3), 3.08 (dd, *J* = 8.2, 7.1 Hz, 2 H, SO_2CH_2), 2.71 (t, *J* = 8.2, 7.1 Hz, 2 H, CH_2CO_2Me). The observed characterization data (R_f, ^{1}H) were consistent with that previously reported.[6]

3-(4-Methoxyphenyl)oxetane-3-sulfonyl fluoride (1)

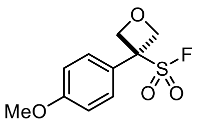

Prepared according to **oxetane sulfinate elimination procedure 3 (OSE-3)** from sulfone **1c** and submitting the sulfinate intermediate directly to the fluorination conditions. Sodium methoxide (5.4 M solution in MeOH, 1.19 mL, 6.42 mmol, 1.0 equiv) was added dropwise to a solution of oxetane sulfone **1c** (2.02 g, 6.42 mmol, 1.0 equiv) in anhydrous THF (12.8 mL, 0.5 M) in a 50 mL round bottom flask. The oxetane sulfinate salt precipitated immediately after the addition of base to form an off-white solid. The reaction mixture was stirred at 25 °C for 20 min, then concentrated *in vacuo* using a rotatory evaporator to afford oxetane sulfinate as a light-yellow solid, which was subjected directly to the next step without spectroscopical analysis. For the fluorination step, Selectfluor™ (3.41 g, 9.63 mmol, 1.5 equiv) was added to anhydrous MeOH (32 mL, 0.2 M) at 0 °C in a 50 mL round bottom flask, then stirred for 5 min. The sulfinate salt was added portionwise and the sulfinate flask was rinsed with ice-cold Selectfluor™/methanol solution and transferred to the reaction vessel. The reaction mixture was stirred at 0 °C for 1 h, then concentrated *in vacuo* using a rotatory evaporator. The residue was diluted with CH_2Cl_2 (100 mL) and filtered through a plug of Celite, eluting with further CH_2Cl_2. After concentration *in vacuo* using a rotatory evaporator, the crude residue was filtered through a pad of silica on a sintered funnel, eluting with further CH_2Cl_2 (200 mL). Concentration of the filtrate afforded oxetane sulfonyl fluoride **1** as a white solid (1.16 g, 70%). R_f = 0.60 (100% CH_2Cl_2); ^{1}H NMR (400 MHz, $CDCl_3$) δ 7.23–7.19 (m, 2 H, 2 × Ar-CH), 7.01–6.97 (m, 2 H, 2 × Ar-CH), 5.44 (d, ^2J = 7.9 Hz, 2 H, C*H*HOC*H*H), 5.24 (dd, ^2J = 7.9 Hz, $J_{H\text{-}F}$ = 1.5 Hz, 2 H, CH*H*OCH*H*), 3.86 (s, 3 H, OCH_3). ^{19}F{^{1}H} NMR (377 MHz, $CDCl_3$) δ 34.3. The observed characterization data (R_f, ^{1}H, ^{19}F) were consistent with that previously reported.[6]

Notes:

*This elimination-fluorination procedure can be performed on a larger scale (15.6 mmol) to afford **1** in a comparable yield (2.45 g, 63%)*

***1** can be further characterized by X-ray crystallography (CCDC reference 2094791).[6] Crystals suitable for X-ray analysis were grown by slow evaporation from acetone.*

Oxetane Sulfonyl Fluoride 2

3-(4-((Triisopropylsilyl)oxy)phenyl)oxetan-3-ol (2a)

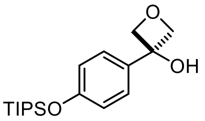

n-BuLi (1.52 M in hexanes, 89.5 mL, 136 mmol, 1.1 equiv) was added dropwise over 15 min to a solution of (4-bromophenoxy)triisopropylsilane (42.3 mL, 149 mmol, 1.2 equiv) in anhydrous THF (620 mL, 0.22 M) at –78 °C in a 2 L 3-neck round-bottom flask. After stirring for 1 h, oxetan-3-one (8.0 mL, 124 mmol, 1.0 equiv)

was added dropwise to the reaction mixture. After a further 15 min of stirring at –78 °C, the reaction mixture was warmed to 25 °C over ~1 h and quenched with water (200 mL). The aqueous layer was extracted with Et_2O (3 × 200 mL). The organic layers were washed with brine (150 mL), dried over Na_2SO_4, filtered and concentrated *in vacuo* using a rotatory evaporator. Purification by recrystallization in *n*-hexane (5 volumes, 70 °C then 3 °C overnight) to afford oxetanol **2a** as a crystalline off-white solid (30.5 g, 77%). R_f = 0.20 (20% EtOAc/pentane); ^{1}H NMR (400 MHz, $CDCl_3$) δ 7.43–7.40 (m, 2 H, 2 × Ar-CH), 6.94–6.90 (m, 2 H, 2 × Ar-CH), 4.93 (d, 2 H, *J* = 6.9 Hz, C*H*HOC*H*H), 4.89 (d, 2 H, *J* = 6.9 Hz, CH*H*OCH*H*), 2.50 (s, 1 H, OH), 1.31–1.22 (m, 3 H, 3 × SiCH), 1.11 (d, *J* = 7.3 Hz, 18 H, 6 × SiCHCH_3). The observed characterization data (R_f, ^{1}H) were consistent with that previously reported.[10]

Notes:

The rate of the dropwise addition of n-BuLi via the canula was controlled by employing a positive pressure on the bottle of n-BuLi with a balloon filled with argon.

The crude material can alternatively be purified by flash column chromatography (10–40% EtOAc/pentane) and requires ~500 g of silica gel when performed on this scale.

Methyl 3-((3-(4-((triisopropylsilyl)oxy)phenyl)oxetan-3-yl)thio)propanoate (2b)

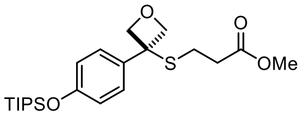

Prepared according to **oxetane thiol alkylation procedure 1 (OTA-1)**. Methyl 3-mercaptopropionate (3.51 mL, 32 mmol, 1.5 equiv) was added to a solution of $FeCl_3$ (342 mg, 2.1 mmol, 10 mol%) in anhydrous CH_2Cl_2 (42 mL, 0.5 M) in a 100 mL round-bottom flask. Oxetanol **2a** (6.81 g, 21.1 mmol, 1.0 equiv) was added to the resulting mixture and the flask was immediately placed into a pre-heated oil bath at 40 °C. The reaction was stirred at 40 °C for 20 min and quenched with sat. aq. $NaHCO_3$ (100 mL). The resulting mixture was filtered through cotton wool directly into a separatory funnel. The aqueous layer was extracted with CH_2Cl_2 (3 × 100 mL). The organic layers were washed with aq. NaOH (1 M, 100 mL), dried over Na_2SO_4, filtered and concentrated *in vacuo* using a rotatory evaporator. Purification by flash column chromatography (20% Et_2O/pentane) afforded oxetane sulfide **2b** as a colorless oil (6.25 g, 74%). R_f = 0.25 (20% Et_2O/pentane); ^{1}H NMR (400 MHz, $CDCl_3$) δ 7.08–7.04 (m, 2 H, 2 × Ar-CH), 6.89–6.86 (m, 2 H, 2 × Ar-CH), 5.20 (d, *J* = 6.4 Hz, 2 H, C*H*HOC*H*H), 4.89 (d, *J* = 6.4 Hz, 2 H, CH*H*OCH*H*), 3.67 (s, 3 H, CO_2CH_3), 2.65 (t, *J* = 7.4 Hz, 2 H, SCH_2), 2.38 (t, *J* = 7.4 Hz, 2 H, CH_2CO_2Me) 1.30–1.21 (m, 3 H, 3 × SiC*H*), 1.11 (d, *J* = 7.4 Hz, 18 H, 6 × SiCHCH_3). The observed characterization data (R_f, ^{1}H) were consistent with that previously reported.[6]

Methyl 3-((3-(4-((triisopropylsilyl)oxy)phenyl)oxetan-3-yl)sulfonyl)propanoate (2c)

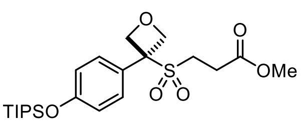

*m*CPBA (≤77%, 12.4 g, 55.2 mmol, 3.0 equiv) was added to a solution of oxetane sulfide **2b** (7.83 g, 18.4 mmol, 1.0 equiv) in CH_2Cl_2 (368 mL, 0.05 M) in a 1 L round-bottom flask. The reaction mixture was stirred at 25 °C for 3 h then aq. KOH (3 M, 200 mL) was added and the phases were separated. The aqueous portion was extracted with CH_2Cl_2 (4 × 100 mL). The organic extracts were combined, dried over Na_2SO_4, filtered and concentrated *in vacuo* using a rotatory evaporator to afford sulfone **2c** as a white solid (8.22 g, 98%). R_f = 0.36 (30% EtOAc/pentane). ^{1}H NMR (400 MHz, $CDCl_3$) δ 7.18–7.14 (m, 2 H, 2 × Ar-CH), 6.96–6.93 (m, 2 H, 2 × Ar-CH), 5.40 (d, *J* = 7.2 Hz, 2 H, C*H*HOC*H*H), 5.12 (d, *J* = 7.2 Hz, 2 H, CH*H*OCH*H*), 3.70 (s, 3 H, CO_2CH_3), 3.06 (dd, *J* = 8.3 Hz, *J* = 7.0 Hz, 2 H, SO_2CH_2), 2.67 (dd, *J* = 8.3 Hz, *J* = 7.0 Hz, 2 H, CH_2CO_2Me), 1.32–1.23 (m, 3 H, 3 × SiCH), 1.11 (d, J = 7.3 Hz, 18 H, 6 × SiCHCH_3). The observed characterization data (R_f, ^{1}H) were consistent with that previously reported.[6]

3-(4-((Triisopropylsilyl)oxy)phenyl)oxetane-3-sulfonyl fluoride (2)

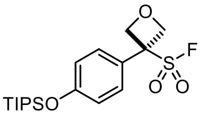

Prepared according to **oxetane sulfinate elimination procedure 3 (OSE-3)** from sulfone **2c** and submitting the sulfinate intermediate directly to the fluorination conditions. Sodium methoxide (5.4 M solution in MeOH, 3.31 mL, 17.9 mmol, 1.0 equiv) was added dropwise to a solution of oxetane sulfone **2c** (8.17 g, 17.9 mmol, 1.0 equiv) in anhydrous THF (36 mL, 0.5 M) in a 100 mL round bottom flask. The reaction mixture was stirred at 25 °C for 20 min, then concentrated *in vacuo* using a rotatory evaporator to afford oxetane sulfinate as a light-yellow solid, which was subjected directly to the next step without spectroscopical analysis. For the fluorination step, Selectfluor^{TM} (9.50 g, 26.9 mmol, 1.5 equiv) was added to anhydrous MeOH (90 mL, 0.2 M) at 0 °C in a 250 mL round bottom flask, then stirred for 5 min. The sulfinate salt was added portionwise and the sulfinate flask was rinsed with ice-cold Selectfluor^{TM}/methanol solution and transferred to the reaction vessel. The reaction mixture was stirred at 0 °C for 1 h, then concentrated *in vacuo* using a rotatory evaporator. The residue was diluted with CH_2Cl_2 (100 mL) and filtered through a plug of Celite, eluting with further CH_2Cl_2. After concentration *in vacuo* using a rotatory evaporator, the crude residue was filtered through a pad of silica on a sintered funnel, eluting with a 50% CH_2Cl_2/pentane solvent system (300 mL). Concentration of the filtrate afforded oxetane sulfonyl fluoride **2** as a white solid (5.13 g, 74%). R_f = 0.45 (50% CH_2Cl_2/pentane). ^{1}H NMR (400 MHz, $CDCl_3$) δ 7.15–7.11 (m, 2 H, 2 × Ar-CH), 6.97–6.93 (m, 2 H, 2 × Ar-CH), 5.42 (d, *J* = 7.7 Hz, 2 H, C*H*HOC*H*H), 5.23 (dd, ^2J = 7.7 Hz, $J_{H\text{-}F}$ = 1.5 Hz, 2 H, CH*H*OCH*H*), 1.32–1.23 (m, 3 H, 3 × SiCH), 1.11 (d, *J* = 7.4 Hz, 18 H, 6 × SiCHCH_3); ^{19}F{^{1}H} NMR (377 MHz, $CDCl_3$) δ 34.3. The observed characterization data (R_f, ^{1}H, ^{19}F) were consistent with that previously reported.[6]

Oxetane Sulfonyl Fluoride 5

3-(3-Isopropoxy-4-methoxyphenyl)oxetan-3-ol (5a)

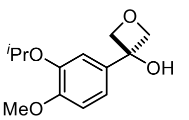

n-BuLi (1.59 M in hexanes, 2.50 mL, 3.97 mmol, 1.1 equiv) was added dropwise over 5 min to a solution of 4-bromo-2-isopropoxy-1-methoxybenzene (1.06 g, 4.33 mmol, 1.2 equiv) in anhydrous THF (19.7 mL, 0.22 M) at –78 °C in a 50 mL round-bottom flask. After stirring for 10 min, oxetan-3-one (0.23 mL, 3.6 mmol, 1.0 equiv) was added dropwise to the reaction mixture. After further 10 min of stirring at –78 °C, the reaction mixture was warmed to 25 °C over ~1 h and quenched with water (15 mL). The aqueous layer was extracted with Et_2O (3 × 10 mL). The organic layers were washed with brine (15 mL), dried over Na_2SO_4, filtered and concentrated *in vacuo* using a rotatory evaporator. Purification by flash column chromatography (30% EtOAc/pentane) afforded oxetanol **5a** as a colorless oil (663 mg, 77%). R_f = 0.25 (30% EtOAc/pentane); IR (film)/cm^{-1} 3389 (br OH), 2969, 2871, 1585, 1509, 1481, 1415, 1375, 1258, 1211, 1177, 1108, 1025, 969, 907, 862, 809; ^{1}H NMR (400 MHz, $CDCl_3$) δ 7.12 (s, 1 H, Ar-CH), 7.10 (d, *J* = 2.2 Hz, 1 H, Ar-CH), 6.91–6.88 (m, 1 H, Ar-CH), 4.91–4.87 (m, 4 H, CH_2OCH_2), 4.57 (hept, *J* = 6.1 Hz, 1 H, C*H*$(CH_3)_2$), 3.86 (s, 3 H, OCH_3), 2.66 (s, 1 H, OH), 1.38 (d, *J* = 6.1 Hz, 6 H, CH(C$H_3)_2$); ^{13}C NMR (101 MHz, $CDCl_3$) δ 150.1 (Ar-C_qOCH$_3$), 147.4 (Ar-C_q^{i}Pr), 134.9 (Ar-C_qC$_q$), 117.3 (Ar-CH), 112.7 (Ar-CH), 111.7 (Ar-CH), 85.5 (CH_2OCH_2), 75.7 (*C*H$(CH_3)_2$), 71.6 (C_q), 56.0 (Ar-C_qO*C*H$_3$), 22.1 (CH(*C*H$_3)_2$); HRMS (TOF-MS-ES$^+$) *m/z* calcd for $C_{13}H_{17}O_3^+$ [M–OH]$^+$: 221.1178, found: 221.1182.

Methyl 3-((3-(3-isopropoxy-4-methoxyphenyl)oxetan-3-yl)thio)propanoate (5b)

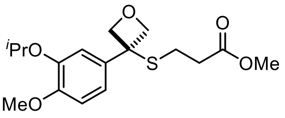

Prepared according to **oxetane thiol alkylation procedure 2 (OTA-3)**. Lithium bis(trifluoromethanesulfonimide) (63 mg, 0.22 mmol, 11 mol%) and tetrabutylammonium hexafluorophosphate (43 mg, 0.11 mmol, 5.5 mol%) were added sequentially to a solution of oxetanol **5a** (477 mg, 2.0 mmol, 1.0 equiv) and methyl 3-mercaptopropionate (0.43 mL, 4.0 mmol, 2.0 equiv) in non-anhydrous $CHCl_3$

(4 mL, 0.5 M). The reaction flask was placed into a preheated oil bath at 40 °C. The reaction was stirred for 15 min and then heated up to 70 °C in 1 h and 10 min. At 70 °C there was a color change from colorless to pink. From the moment of the color change, the reaction was stirred for further 25 min and quenched with sat. aq. $NaHCO_3$ (15 mL). The aqueous layer was extracted with CH_2Cl_2 (3 × 20 mL). The organic extracts were combined and washed with aq. NaOH (1 M, 20 mL), dried over Na_2SO_4, filtered and concentrated *in vacuo* using a rotatory evaporator. Purification by flash column chromatography (10% EtOAc/pentane) afforded oxetane sulfide **5b** as a colorless oil (315 mg, 46%). R_f = 0.57 (30% EtOAc/pentane); IR (film)/cm^{-1} 2946, 2976, 2247, 1732 (C=O st), 1509, 1436, 1355, 1244, 1174, 1138, 1108, 1023, 972, 906, 803, 724; ^{1}H NMR (400 MHz, $CDCl_3$) δ 6.85 (d, *J* = 8.0 Hz, 1 H, Ar-CH), 6.76 (d, *J* = 2.2 Hz, 1 H, Ar-CH), 6.74 (d, *J* = 2.1 Hz, 1 H, Ar-CH), 5.18 (d, *J* = 6.4 Hz, 2 H, C*H*HOC*H*H), 4.88 (d, *J* = 6.4 Hz, 2 H, CH*H*OCH*H*), 4.52 (hept, *J* = 6.1 Hz, 1 H, C*H*(CH_3)$_2$), 3.86 (s, 3 H, Ar-C_qOCH_3), 3.66 (s, 3 H, CO_2CH_3), 2.65 (t, *J* = 7.4 Hz, 2 H, SCH_2), 2.39 (t, *J* = 7.4 Hz, 2 H, C*H*$_2$$CO_2CH_3$), 1.37 (d, *J* = 6.0 Hz, 6 H, CH(C*H*$_3$)$_2$); ^{13}C NMR (101 MHz, $CDCl_3$) δ 172.0 (*C*O_2CH_3), 149.7 (Ar-*C*$_q$OMe), 147.3 (Ar-*C*$_q$^{i}Pr), 134.4 (Ar-*C*$_q$$C_q$), 119.0 (Ar-CH), 114.3 (Ar-CH), 111.4 (Ar-CH), 83.1 (CH_2OCH_2), 71.5 (*C*H(CH_3)$_2$), 55.8 (Ar-C_qO*C*H_3), 52.3 (C_q), 51.7 (CO_2*C*H_3), 34.0 (*C*$H_2CO_2CH_3$), 25.1 (SCH_2), 21.9 (CH(*C*H_3)$_2$); HRMS (TOF-MS-APCI$^+$) *m/z* calcd for $C_{17}H_{23}O_4S^+$ [M–OH]$^+$: 323.1312, found: 323.1315.

Methyl 3-((3-(3-isopropoxy-4-methoxyphenyl)oxetan-3-yl)sulfonyl) propanoate (5c)

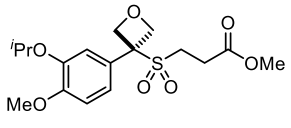

*m*CPBA (≤77%, 529 mg, 2.37 mmol, 3.0 equiv) was added to a solution of oxetane sulfide **5b** (269 mg, 0.79 mmol, 1.0 equiv) in CH_2Cl_2 (39.5 mL, 0.02 M) in a 100 mL round-bottom flask. The reaction mixture was stirred at 25 °C for 3 h then aq. KOH (3 M, 10 mL) was added and the phases were separated. The aqueous portion was extracted with CH_2Cl_2 (3 × 15 mL). The organic extracts were combined, dried over Na_2SO_4, filtered and concentrated *in vacuo* using a rotatory evaporator. Purification by flash column chromatography (70% Et_2O/pentane) afforded oxetane sulfone **5c** as a colorless oil (161 mg, 55%). R_f = 0.28 (50% EtOAc/pentane); IR (film)/cm^{-1} 2949, 2882, 2837, 1736 (C=O st), 1601, 1511, 1439, 1418, 1305 (SO_2 st as), 1249, 1214, 1174, 1135 (SO_2 st sy), 1103, 1022, 997, 932, 890, 807, 726, 632, 513; ^{1}H NMR (400 MHz, $CDCl_3$) δ 6.92 (d, *J* = 8.3 Hz, 1 H, Ar-CH), 6.85 (dd, *J* = 8.3, 2.2 Hz, 1 H, Ar-CH), 6.79 (d, *J* = 2.1 Hz, 1 H, Ar-CH), 5.39 (d, *J* = 7.2 Hz, 2 H, C*H*HOC*H*H), 5.11 (d, *J* = 7.2 Hz, 2 H, CH*H*OCH*H*), 4.53 (hept, *J* = 6.1 Hz, 1 H, C*H*(CH_3)$_2$), 3.88 (s, 3 H, Ar-C_qOCH_3), 3.69 (s, 3 H, CO_2CH_3), 3.07 (t, *J* = 8.3 Hz, 2 H, SCH_2), 2.68 (t, *J* = 8.2 Hz, 2 H, CH_2CO_2Me), 1.36 (d, *J* = 6.1 Hz, 6 H, CH(C*H*$_3$)$_2$); ^{13}C NMR (101 MHz, $CDCl_3$) δ 170.8 (*C*O_2Me), 151.3 (Ar-*C*$_q$OMe), 147.5 (Ar-*C*$_q$^{i}Pr), 125.7 (Ar-*C*$_q$$C_q$), 120.9 (Ar-CH), 115.6 (Ar-CH), 111.7 (Ar-CH), 76.2 (CH_2OCH_2), 71.7 (*C*H(CH_3)$_2$), 68.4 (Ar-C_qO*C*H_3), 56.0 (C_q), 52.4 (CO_2*C*H_3), 43.4 (*C*H_2CO_2Me), 25.9 (SCH_2), 21.9 (CH(*C*H_3)$_2$); HRMS (TOF-MS-ES$^+$) *m/z* calcd for $C_{19}H_{27}NO_7NaS^+$ [M+CH_3CN+Na]$^+$: 436.1406, found: 436.1417.

3-(3-Isopropoxy-4-methoxyphenyl)oxetane-3-sulfonyl fluoride (5) and 3-fluoro-3-(3-isopropoxy-4-methoxyphenyl)oxetane (5')

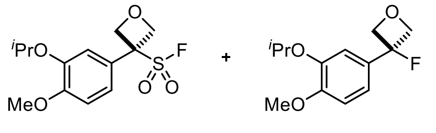

Prepared according to **oxetane sulfinate elimination procedure 3 (OSE-3)** from sulfone **5c** and submitting the sulfinate intermediate directly to the fluorination conditions. Sodium methoxide (5.4 M solution in MeOH, 72.2 µL, 0.39 mmol, 1.0 equiv) was added dropwise to a solution of oxetane sulfone **5c** (146 mg, 0.39 mmol, 1.0 equiv) in anhydrous THF (1.0 mL, 0.39 M) in a 10 mL round bottom flask. The reaction mixture was stirred at 25 °C for 20 min, then concentrated *in vacuo* using a rotatory evaporator to afford oxetane sulfinate as a yellow solid, which was subjected directly to the next step without spectroscopical analysis. For the fluorination step, Selectfluor^{TM} (207 mg, 0.585 mmol, 1.5 equiv) was added to anhydrous MeOH (1.95 mL, 0.2 M) at 0 °C in a 10 mL round bottom flask, then stirred for 5 min. The sulfinate salt was added portionwise and the sulfinate flask was rinsed with ice-cold Selectfluor^{TM}/methanol solution and

transferred to the reaction vessel. The reaction mixture was stirred at 0 °C for 1 h, then concentrated *in vacuo* using a rotatory evaporator. The residue was diluted with CH_2Cl_2 (30 mL) and filtered through a plug of Celite, eluting with further CH_2Cl_2. After concentration *in vacuo* using a rotary evaporator, purification by flash column chromatography (80% CH_2Cl_2/pentane, then 100% CH_2Cl_2) afforded an 85:15 mixture of oxetane sulfonyl fluoride **5** and oxetane fluoride **5'** as a white solid (67 mg, 56%). R_f = 0.47 (100% CH_2Cl_2); mp = 93–95 °C, IR (film)/cm^{-1} 2974, 2250, 1538, 1513, 1462, 1421, 1399 (SO_2 st as), 1267, 1252, 1211 (SO_2 st sy), 1143, 1108, 1024, 903, 787, 728, 540; ^{1}H NMR (400 MHz, $CDCl_3$) δ 7.12–7.03 (m, 0.29 H, 2 × Ar-CH, **5'**), 6.94 (d, *J* = 8.4 Hz, 1 H, Ar-CH, **5**), 6.85 (dd, *J* = 8.4, 2.3 Hz, 1 H, Ar-CH, **5**), 6.74 (d, *J* = 2.3 Hz, 1 H, Ar-CH, **5**), 5.42 (d, *J* = 7.5 Hz, 2 H, C*H*HOC*H*H, **5**), 5.23 (dd, *J* = 7.8, 1.5 Hz, 2 H, CH*H*OCH*H*, **5**), 5.10 (dd, ^2J = 7.7, $J_{H\text{-}F}$ = 1.2 Hz, 0.14 H, C*H*HOCHH, **5'**), 5.05 (dd, ^2J = 7.6, $J_{H\text{-}F}$ = 1.2 Hz, 0.14 H, CH*H*OCHH, **5'**), 4.90 (dd, ^2J = 7.6, $J_{H\text{-}F}$ = 1.2 Hz, 0.14 H, CHHOC*H*H, **5'**), 4.84 (dd, ^2J = 7.6, $J_{H\text{-}F}$ = 1.3 Hz, 0.14 H, CHHOCH*H*, **5'**), 4.52 (hept, *J* = 6.1 Hz, 1 H, C*H*(CH_3)$_2$, **5**), 3.89 (s, 3 H, OCH_3, **5**), 1.36 (d, *J* = 6.1 Hz, 6 H, CH(C*H*$_3$)$_2$, **5**); ^{13}C NMR (101 MHz, $CDCl_3$) δ 151.9 (Ar-*C*$_q$OCH$_3$, **5**), 147.6 (Ar-*C*$_q$^{i}Pr, **5**), 123.3 (Ar-*C*$_q$C$_q$, **5**), 121.4 (Ar-CH, **5**), 115.6 (Ar-CH, **5**), 111.9 (Ar-CH, **5**), 83.1 (d, $^2J_{C\text{–}F}$ = 25.8 Hz, CH_2OCH_2, **5'**), 77.1 (CH_2OCH_2, **5**), 71.9 (*C*H(CH_3)$_2$, **5**), 71.7 (*C*H(CH_3)$_2$, **5'**), 68.4 (d, $J_{C\text{-}F}$ = 14.9 Hz, C$_q$, **5**), 56.0 (OCH_3, **5**), 22.0 (CH(C*H*$_3$)$_2$, **5**), 21.9 (CH(C*H*$_3$)$_2$, **5**); ^{19}F{^{1}H} NMR (377 MHz, $CDCl_3$) δ 34.5 (**5**), –146.4 (**5'**); HRMS (FTMS–APCI$^+$) *m/z* calcd for $C_{13}H_{17}O_3^+$ [M–SO_2F]$^+$: 221.1172, found: 221.1173.

Notes:

*The R_f values of **5** and **5'** are very similar and attempts to further purify the obtained mixture by flash column chromatography led to significant loss in mass of sulfonyl fluoride **5** due to degradation on silica without improvement in purity.*

Oxetane Sulfonyl Fluoride 7

3-(Benzo[*d*][1,3]dioxol-5-yl)oxetan-3-ol (7a)

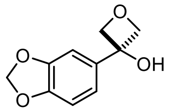

n-BuLi (1.58 M in hexanes, 27.8 mL, 44 mmol, 1.1 equiv) was added dropwise over 10 min to a solution of 5-bromobenzo[*d*][1,3]dioxole (5.68 mL, 48.0 mmol, 1.2 equiv) in anhydrous THF (180 mL, 0.22 M) at –78 °C in a 500 mL round bottom flask. After stirring for 1 h, oxetan-3-one (2.34 mL, 40 mmol, 1.0 equiv) was added dropwise to the reaction mixture. After a further 1 h of stirring at –78 °C, the reaction mixture was slowly warmed to 25 °C, stirred overnight and quenched with NH_4Cl (100 mL). The aqueous layer was extracted with EtOAc (3 × 100 mL). The organic layers were washed with brine (100 mL), dried over Na_2SO_4, filtered and concentrated *in vacuo* using a rotatory evaporator. Purification by recrystallization in toluene (6 volumes, 90 °C then 3 °C overnight) to afford oxetanol **7a** as a crystalline off-white solid (6.36 g, 81%). R_f = 0.13 (30% EtOAc/*n*-hexane); ^{1}H NMR (400 MHz, $CDCl_3$) δ 7.08–7.02 (m, 2 H, 2 × Ar-CH), 6.84 (d, *J* = 8.0 Hz, 1 H, Ar-CH), 5.99 (s, 2 H, OCH_2O), 4.88 (s, 4 H, CH_2OCH_2), 2.69 (s, 1 H, OH). The observed characterization data (R_f, ^{1}H, ^{19}F) were consistent with that previously reported.[10]

Methyl 3-((2H-(benzo[*d*][1,3]dioxol-5-yl)oxetan-3-yl)thio)propanoate (7b)

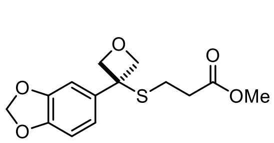

Prepared according to **oxetane thiol alkylation procedure 1 (OTA-1)**. Methyl 3-mercaptopropionate (7.18 mL, 64.8 mmol, 2.0 equiv) was added to a solution of $FeCl_3$ (526 mg, 3.24 mmol, 10 mol%) in anhydrous CH_2Cl_2 (65 mL, 0.5 M) in a 100 mL round-bottom flask. Oxetanol **7a** (6.30 g, 32.4 mmol, 1.0 equiv) was added to the resulting mixture and the flask was immediately placed into a pre-heated oil bath at 40 °C. The reaction was stirred at 40 °C for 25 min and quenched with sat. aq. $NaHCO_3$ (100 mL). The resulting mixture was filtered through cotton wool directly into a separatory funnel to remove residual $FeCl_3$ and aid phase separation during solvent-solvent extraction. The aqueous layer was extracted with CH_2Cl_2 (3 × 100 mL). The organic layers were washed with aq. NaOH (1 M, 100 mL), dried over Na_2SO_4, filtered and concentrated *in vacuo* using a rotatory evaporator. Purification by flash column chromatography (10–20% EtOAc/pentane) afforded oxetane sulfide **7b** as a white solid

(4.96 g, 52%). R_f = 0.25 (20% EtOAc/pentane); mp = 67–70 °C; IR (film)/cm^{-1} 2950, 2870, 1733 (C=O st), 1489, 1439, 1249, 1224, 1038; ^{1}H NMR (400 MHz, $CDCl_3$) δ 6.78 (d, *J* = 7.9 Hz, 1 H, Ar-CH), 6.70–6.61 (m, 2 H, 2 × Ar-CH), 5.98 (s, 2 H, OCH_2O), 5.15 (d, *J* = 6.4 Hz, 2 H, C*H*HOC*H*H), 4.85 (d, *J* = 6.4 Hz, 2 H, CH*H*OCH*H*), 3.67 (s, 3 H, CO_2CH_3), 2.67 (t, *J* = 7.4 Hz, 2 H, SCH_2), 2.43 (t, *J* = 7.4 Hz, 2 H, $CH_2CO_2CH_3$); ^{13}C NMR (101 MHz, $CDCl_3$) δ 172.1 (C_q=O), 148.1 (Ar-C_q-OCH_2O), 146.8 (Ar-C_q-OCH_2O), 136.1 (Ar-C_q-C_q), 119.6 (Ar-CH), 108.0 (Ar-CH), 107.0 (Ar-CH), 101.3 (OCH_2O), 83.1 (CH_2OCH_2), 52.6 (C_q), 51.9 (CO_2CH_3), 34.0 ($CH_3CO_2CH_3$), 25.2 (SCH_2). HRMS (TOF-MS-ES$^+$) *m/z* calcd for $C_{14}H_{20}NO_5S^+$ $[M+NH_4]^+$: 314.1062, found: 314.1054.

Methyl 3-((3-(benzo[*d*][1,3]dioxol-5-yl)oxetan-3-yl)sulfonyl)propanoate (7c)

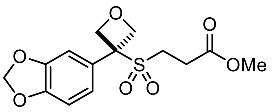

*m*CPBA (≤77%, 11.23 g, 50.1 mmol, 3.0 equiv) was added to a solution of oxetane sulfide **7b** (4.96 g, 16.7 mmol, 1.0 equiv) in CH_2Cl_2 (334 mL, 0.05 M) in a 1 L round-bottom flask. The reaction mixture was stirred at 25 °C for 3 h then aq. KOH (3 M, 200 mL) was added and the phases were separated. The aqueous portion was extracted with CH_2Cl_2 (4 × 100 mL). The organic extracts were combined, dried over Na_2SO_4, filtered and concentrated *in vacuo* using a rotatory evaporator to afford sulfone **7c** as a white solid (5.32 g, 97%). R_f = 0.28 (50% EtOAc/pentane); mp = 131–134 °C; IR (film)/cm^{-1} 2954, 2887, 1735 (C=O st), 1504, 1491, 1441, 1310 (SO_2 st as), 1256, 1231, 1139 (SO_2 st sy), 1034, 890; ^{1}H NMR (400 MHz, $CDCl_3$) δ 6.87 (d, *J* = 8.6 Hz, 1 H, Ar-CH), 6.75 (d, *J* = 6.1 Hz, 2 H, Ar-CH), 6.02 (s, 2 H, OCH_2O), 5.36 (d, *J* = 7.2 Hz, 2 H, C*H*HOC*H*H), 5.09 (d, *J* = 7.2 Hz, 2 H, CH*H*OCH*H*), 3.71 (s, 3 H, CO_2CH_3), 3.12 (t, *J* = 7.6 Hz, 2 H, SO_2CH_2), 2.75 (t, *J* = 7.6 Hz, 2 H, $CH_2CO_2CH_3$). ^{13}C NMR (101 MHz, $CDCl_3$) δ 170.8 (C_q=O), 148.7 (Ar-C_q-OCH_2O), 148.4 (Ar-C_q-OCH_2O), 127.2 (Ar-C_q-C_q), 122.1 (Ar-CH), 108.6 (2 × Ar-CH), 101.8 (OCH_2O), 76.3 (CH_2OCH_2), 68.7 (C_q), 52.5 (CO_2CH_3), 43.3 ($CH_2CO_2CH_3$), 25.9 (SO_2CH_2), HRMS (TOF-MS-ES$^+$) *m/z* calcd for $C_{14}H_{20}NO_7S^+$ $[M+NH_4]^+$: 346.0960, found: 346.0955.

3-(Benzo[*d*][1,3]dioxol-5-yl)oxetane-3-sulfonyl fluoride (7)

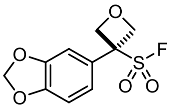

Prepared according to **oxetane sulfinate elimination procedure 2 (OSE-2)** from sulfone **7c** and submitting the sulfinate intermediate directly to the fluorination conditions. NaOH (5 M in MeOH, 2.95 mL, 14.8 mmol, 1.05 equiv) was added to a solution of sulfone **7c** (4.62 g, 14.8 mmol, 1.0 equiv) in anhydrous CH_2Cl_2 (140 mL, 0.1 M) at 0 °C in a 500 mL round-bottom flask. The oxetane sulfinate salt precipitated after 5 min of the start of addition of base to form a white solid. The reaction mixture was stirred at 0 °C for 15 min then filtered and washed with ice-cold *n*-hexane to afford the oxetane sulfinate as a white solid, which was subjected directly to the next step without spectroscopical analysis. For the fluorination step, Selectfluor™ (7.47 g, 21.1 mmol, 1.5 equiv) was added to anhydrous MeOH (70 mL, 0.2 M) at 0 ºC in a 250 mL round bottom flask, then stirred for 5 min. The sulfinate salt was added portionwise. The reaction mixture was stirred at 0 °C for 1 h then concentrated *in vacuo* using a rotatory evaporator. The residue was diluted with CH_2Cl_2 (50 mL) and filtered through a plug of Celite, eluting with further CH_2Cl_2. After concentration *in vacuo* using a rotatory evaporator, the crude residue was filtered through a pad of silica on a sintered funnel, eluting with a 70% CH_2Cl_2/pentane solvent system (200 mL). Concentration of the filtrate afforded oxetane sulfonyl fluoride **7** as a white solid (2.56 g, 70%). R_f = 0.32 (50% CH_2Cl_2/pentane); mp = 111–113 °C; IR (film)/cm^{-1} 2891, 1508, 1441, 1395 (SO_2 st as), 1262, 1232, 1213, 1034, 932, 889, 773, 626; ^{1}H NMR (400 MHz, $CDCl_3$) δ 6.88 (d, *J* = 8.1 Hz, 1 H, Ar-CH), 6.76–6.69 (m, 2 H, 2 × Ar-CH), 6.04 (s, 2 H, OCH_2O), 5.39 (d, *J* = 7.6 Hz, 2 H, C*H*HOC*H*H), 5.20 (dd, *J* = 7.9, $J_{H\text{-}F}$ = 1.6 Hz, 2 H, CH*H*OCH*H*). ^{13}C NMR (126 MHz, $CDCl_3$) δ 149.2 (Ar-C_q-OCH_2O), 148.4 (Ar-C_q-OCH_2O), 124.8 (Ar-C_q-C_q), 122.5 (Ar-CH), 108.8 (Ar-CH), 108.4 (Ar-CH), 101.9 (OCH_2O), 77.1 (CH_2OCH_2), 68.4 (d, $J_{C\text{-}F}$ = 14.8 Hz, C_q); ^{19}F{^{1}H} NMR (377 MHz, $CDCl_3$) δ 34.6; HRMS (FTMS–ES$^+$) *m/z* calcd for $C_{10}H_9O_3^+$ $[M–SO_2F]^+$: 177.0546, found: 177.0545.

Oxetane Sulfonyl Fluoride 10

3-(4-Phenoxyphenyl)oxetan-3-ol (10a)

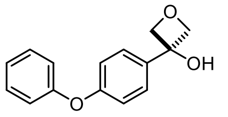

n-BuLi (1.53 M in *n*-hexane, 14.8 mL, 24.0 mmol, 1.1 equiv) was added dropwise over 5 min to a solution of diphenyl ether (4.18 mL, 24 mmol, 1.2 equiv) in anhydrous THF (100 mL, 0.22 M) at –78 °C in a 200 mL round-bottom flask. After stirring for 30 min, oxetan-3-one (1.29 mL, 20.0 mmol, 1.0 equiv) was added dropwise to the reaction mixture. After further 30 min of stirring at –78 °C, the reaction mixture was warmed to 25 °C over ~1 h and quenched with water (50 mL). The aqueous layer was extracted with Et_2O (3 × 50 mL). The organic layers were washed with brine (50 mL), dried over Na_2SO_4, filtered and concentrated *in vacuo* using a rotatory evaporator. Purification by flash column chromatography (20–40% EtOAc/*n*-hexane) afforded oxetanol **10a** as a colorless oil (3.94 g, 81%). R_f = 0.29 (40% EtOAc/ *n*-hexane). mp = 73–75 °C. IR (film)/cm^{-1} 3690 (OH st), 3061, 2951, 2873, 1587, 1508, 1484, 1228, 1167, 1106, 1068, 1016, 969, 868, 839, 692, 542. ^1H NMR (400 MHz, $CDCl_3$) δ 7.60–7.50 (m, 2 H, 2 × Ar-CH), 7.36 (dd, *J* = 8.6, 7.3 Hz, 2 H, 2 × Ar-CH), 7.19–7.10 (m, 1 H, Ar-CH), 7.04 (t, *J* = 8.2 Hz, 4 H, 4 × Ar-CH), 4.97–4.85 (m, 4 H, CH_2OCH_2), 3.01–2.87 (m, 1 H, OH); ^{13}C NMR (101 MHz, $CDCl_3$) δ 157.1 ($Ar_{(Ph)}C_q$-O), 156.9 (Ar-C_q-O), 137.0 (Ar-C_q-C_q), 129.9 (2 × Ar-CH), 126.2 (2 × Ar-CH), 123.6 (Ar-CH), 119.1 (2 × Ar-CH), 118.8 (2 × Ar-CH), 85.7 (CH_2OCH_2), 75.6 (C_q). HRMS (TOF-MS-ES$^+$) *m/z* calcd for $C_{15}H_{15}O_3^+$ $[M+H]^+$: 243.1021; found: 243.1026.

Methyl 3-((3-(4-phenoxyphenyl)oxetan-3-yl)thio)propanoate (10b)

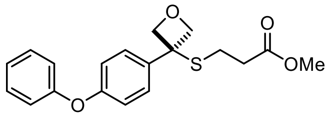

Prepared according to **oxetane thiol alkylation procedure 2 (OTA-2)**. $FeCl_3$ (64.9 mg, 0.4 mmol, 10 mol%) and methyl 3-mercaptopropionate (665 µL, 6.0 mmol, 1.5 equiv) were added sequentially to a solution of oxetanol **10a** (969 mg, 4.0 mmol, 1.0 equiv) in anhydrous $CHCl_3$ (8.0 mL, 0.5 M) in a 25 mL round bottom flask. The reaction mixture was stirred at 60 °C for 1 h, then quenched with sat. aq. $NaHCO_3$ (10 mL). The aqueous layer was extracted with CH_2Cl_2 (3 × 10 mL). The organic layers were washed with aq. NaOH (1 M, 40 mL), dried over Na_2SO_4, filtered, and solvent concentrated *in vacuo* using a rotary evaporator. Purification by flash chromatography (30% Et_2O/pentane) afforded oxetane sulfide **10b** as a colorless oil (379.4 mg, 28%). R_f = 0.20 (30% Et_2O/pentane). IR (film)/cm^{-1} 2950, 2870, 1735 (C=O st), 1587, 1588, 1506, 1489, 1236, 1170. ^1H NMR (400 MHz, $CDCl_3$) δ 7.42–7.32 (m, 2 H, 2 × Ar-CH), 7.17 (dd, *J* = 10.0, 7.9 Hz, 3 H, 3 × Ar-CH), 7.09–6.96 (m, 4 H, 4 × Ar-CH), 5.21 (d, *J* = 6.4 Hz, 2 H, C*H*HOC*H*H), 4.92 (d, *J* = 6.4 Hz, 2 H, CH*H*OCH*H*), 3.69 (s, 3 H, CO_2CH_3), 2.71 (t, *J* = 7.4 Hz, 2 H, SCH_2), 2.44 (t, *J* = 7.4 Hz, 2 H, C*H*$_2CO_2CH_3$). ^{13}C NMR (101 MHz, $CDCl_3$) δ 172.0 (C_q=O), 156.7 ($Ar_{(Ph)}$-C_q-O), 156.5 (Ar-C_q-O), 136.9 (Ar-C_q-C_q), 129.8 (2 × Ar-CH), 127.7 (2 × Ar-CH), 123.6 (Ar-CH), 119.1 (2 × Ar-CH), 118.7 (2 × Ar-CH), 83.1 (CH_2OCH_2), 52.1 (C_q), 51.9 (CH_3), 34.0 ($CH_2CO_2CH_3$), 25.1 (SCH_2). HRMS (TOF-MS-ES$^+$) *m/z* calcd for $C_{19}H_{20}O_4S^+$ $[M+H]^+$: 362.1426; found 362.1419.

Methyl 3-((3-(4-phenoxyphenyl)oxetan-3-yl)sulfonyl)propanoate (10c)

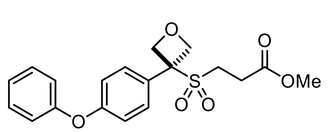

*m*CPBA (≤77%, 628 mg, 2.8 mmol, 3.0 equiv) was added to a solution of oxetane sulfide **10b** (322 mg, 0.93 mmol, 1.0 equiv) in CH_2Cl_2 (9.3 mL, 0.1 M) in a 25 mL round-bottom flask. The reaction mixture was stirred at 25 °C for 3 h then aq. KOH (3 M, 20 mL) was added and the phases were separated. The aqueous portion was extracted with CH_2Cl_2 (4 × 20 mL). The organic extracts were combined, dried over Na_2SO_4, filtered and concentrated *in vacuo* using a rotatory evaporator to afford sulfone **10c** as a pale-yellow solid (365 mg, quant.). R_f = 0.38 (50% EtOAc/*n*-hexane). mp = 117–118 °C. IR (film)/cm^{-1} 2952, 2926, 2885, 1739 (C=O st), 1588, 1508, 1489, 1308 (SO_2 st as), 1241, 1139 (SO_2 st sy). ^1H NMR (400 MHz, $CDCl_3$) δ 7.44–7.36 (m, 2 H, 2 × Ar-CH), 7.27–7.23 (m, 2 H, 2 × Ar-CH), 7.22–7.16 (m, 1 H, Ar-CH), 7.09–7.03 (m, 4 H, 4 × Ar-CH), 5.41 (d, *J* = 7.3 Hz, 2 H,

C*H*HOC*H*H), 5.14 (d, *J* = 7.3 Hz, 2 H, CH*H*OCH*H*), 3.72 (s, 3 H, CO_2CH_3), 3.14 (dd, *J* = 8.1, 7.1 Hz, 2 H, SCH_2), 2.75 (dd, *J* = 8.1, 7.1 Hz, 2 H, $CH_2CO_2CH_3$). ^{13}C NMR (101 MHz, $CDCl_3$) δ 170.8 (C_q=O), 158.7 ($Ar_{(Ph)}$-C_q-O), 155.9 (Ar-C_q-O), 130.0 (2 × Ar-CH), 129.7 (2 × Ar-CH), 127.7 (Ar-C_q-C_q), 124.3 (Ar-CH), 119.8 (2 × Ar-CH), 118.5 (2 × Ar-CH), 76.3 (CH_2OCH_2), 68.4 (C_q), 52.5 (CH_3), 43.3 (CH_2CO_2Me), 25.9 (SCH_2). HRMS (TOF-MS-ES$^+$) *m/z* calcd for $C_{19}H_{20}O_6S^+$ $[M+NH_4]^+$: 394.1324; found 394.1344.

3-(4-Phenoxyphenyl)oxetane-3-sulfonyl fluoride (10)

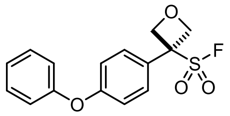

Prepared according to **oxetane sulfinate elimination procedure 1 (OSE-1)** from sulfone **10c** and submitting the sulfinate intermediate directly to the fluorination conditions. Oxetane sulfone **10c** (151 mg, 0.4 mmol, 1.0 equiv) was added to a solution of NaH (60% in mineral oil, 16.8 mg, 1.05 mmol, 1.05 equiv) in THF (2.0 mL, 0.2 M). The reaction mixture was stirred at 25 °C for 1 h then quenched with anhydrous MeOH (3.0 mL) and concentrated under reduced pressure to afford sulfinate salt as a yellow solid, which was subjected directly to the next step without spectroscopical analysis. Selectfluor™ (213 mg, 0.6 mmol, 1.5 equiv) was added to anhydrous MeOH (2.0 mL, 0.2 M) at 0 °C in a 10 mL round bottom flask, then stirred for 5 min. Sulfinate salt was added portionwise to the cooled reaction mixture. The reaction mixture was stirred at 0 °C for 1 h then concentrated under reduced pressure. The residue was diluted with CH_2Cl_2 (30 mL) and filtered through a plug of Celite, eluting with further CH_2Cl_2 (100 mL). After concentration *in vacuo* using a rotatory evaporator, purification by flash column chromatography through a short column (70% CH_2Cl_2/pentane) afforded oxetane sulfonyl fluoride **10** as a white solid (85.9 mg, 70%). R_f = 0.53 (70% CH_2Cl_2/pentane); mp = 93–95 °C; IR (film)/cm^{-1} 2954, 2892, 1724, 1590, 1508, 1489, 1340 (SO_2 st as), 1243, 1213 (SO_2 st sy), 1168; ^1H NMR (500 MHz, $CDCl_3$) δ 7.41 (m, 2 H, 2 × Ar-CH), 7.22 (m, 3 H, 3 × Ar-CH), 7.07 (m, 4 H, 4 × Ar-CH), 5.44 (d, *J* = 7.2 Hz, 2 H, C*H*HOC*H*H), 5.25 (d, *J* = 7.2 Hz, 2 H, CH*H*OCH*H*). ^{13}C NMR (126 MHz, $CDCl_3$) δ 159.3 ($Ar_{(Ph)}$-C_q-O), 155.7 (Ar-C_q-O), 130.0 (2 × Ar-CH), 129.8 (2 × Ar-CH), 125.5 (Ar-C_q-Cq), 124.5 (Ar-CH), 120.0 (2 × Ar-CH), 118.4 (2 × Ar-CH), 77.1 (CH_2OCH_2), 68.0 (d, $^2J_{C\text{-}F}$ = 14.1 Hz, C_q). $^{19}F\{^1H\}$ NMR (377 MHz, $CDCl_3$) δ 34.7; HRMS (TOF-MS-ES$^+$) *m/z* calcd for $C_{15}H_{13}O_2^+$ $[M–SO_2F]^+$: 225.0916; found: 225.0916.

Divergent access to oxetane sulfonyl fluorides from phenol sulfide **131**

Methyl 3-((3-(4-hydroxyphenyl)oxetan-3-yl)thio)propanoate (131)

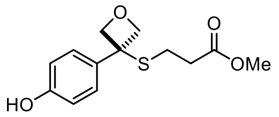

TBAF (1.0 M in THF, 4.63 mL, 16.0 mmol, 1.2 equiv) was added dropwise into a solution of oxetane sulfide **2b** (5.67 g, 13.4 mmol, 1.0 equiv) in THF (66.8 mL, 0.2 M) at 0 °C. The reaction mixture was warmed to room temperature and stirred for 3 h, then quenched with sat. aq. NH_4Cl (30 mL). The aqueous layer was extracted with EtOAc (3 × 30 mL). The combined organic layers were dried with Na_2SO_4, filtered and concentrated *in vacuo* using a rotatory evaporator. Purification by flash chromatography (40% EtOAc/*n*-hexane) afforded oxetane phenol sulfide **131** as a white solid (3.4 g, 95%). R_f = 0.22 (20% EtOAc/*n*-hexane). ^1H NMR (400 MHz, $CDCl_3$) δ 7.14–7.05 (m, 2 H, 2 × Ar-CH), 6.90–6.82 (m, 2 H, 2 × Ar-CH), 5.19 (d, *J* = 6.5 Hz, 2 H, C*H*HOC*H*H), 4.90 (d, *J* = 6.5 Hz, 2 H, CH*H*OCH*H*), 4.88 (br s, OH) 3.68 (s, 3 H, CO_2CH_3), 2.67 (t, *J* = 7.4 Hz, 2 H, SCH_2), 2.42 (t, *J* = 7.4 Hz, 2 H, $CH_2CO_2CH_3$). The observed characterization data (R_f, ^1H) were consistent with that previously reported.[6]

Oxetane Sulfonyl Fluoride 132

Methyl 3-((3-(4-(benzyloxy)phenyl)oxetan-3-yl)thio)propanoate (132b)

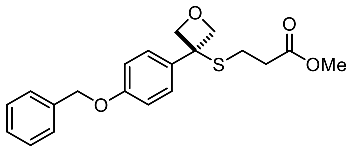

Benzyl bromide (1.19 mL, 10.0 mmol, 2.0 equiv) was added to a solution of phenol sulfide **131** (1.34 g, 5.0 mmol, 1.0 equiv) and K_2CO_3 (2.56 g, 15.0 mmol, 3.0 equiv) in non-anhydrous acetone (16.7 mL, 0.3 M) in a 50 mL round-bottom flask. The reaction mixture was stirred at 40 °C for 16 h then diluted with EtOAc (50 mL), filtered through a plug of Celite and concentrated *in vacuo* using a rotatory evaporator. If required, purification by flash column chromatography (30% EtOAc/*n*-hexane) afforded the oxetane sulfide **132b** as a white solid (1.79 g, quant.). R_f = 0.27 (30% EtOAc/*n*-hexane). ^{1}H NMR (400 MHz, $CDCl_3$) δ 7.49–7.30 (m, 5 H, 5 × Ph-CH), 7.16–7.10 (m, 2 H, 2 × Ar-CH), 7.02–6.95 (m, 2 H, 2 × Ar-CH), 5.19 (d, *J* = 6.4 Hz, 2 H, C*H*HOC*H*H), 5.08 (s, 2 H, $PhCH_2$), 4.90 (d, *J* = 6.4 Hz, 2 H, CH*H*OCH*H*), 3.68 (s, 3 H, CO_2CH_3), 2.68 (t, *J* = 7.4 Hz, 2 H, SCH_2), 2.43 (t, *J* = 7.4 Hz, 2 H, $CH_2CO_2CH_3$). The observed characterization data (R_f, ^{1}H) were consistent with that previously reported.[6]

Methyl 3-((3-(4-(benzyloxy)phenyl)oxetan-3-yl)sulfonyl)propanoate (132c)

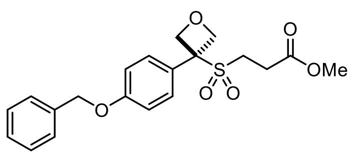

*m*CPBA (≤77%, 3.36 g, 15.0 mmol, 3.0 equiv) was added to a solution of oxetane sulfide **132b** (1.79 g, 5.0 mmol, 1.0 equiv) in CH_2Cl_2 (50 mL, 0.1 M) in a 100 mL round-bottom flask. The reaction mixture was stirred at 25 °C for 3 h then aq. KOH (3 M, 50 mL) was added and the phases were separated. The aqueous portion was extracted with CH_2Cl_2 (4 × 50 mL). The organic extracts were combined, dried over Na_2SO_4, filtered and concentrated *in vacuo* using a rotatory evaporator to afford sulfone **132c** as a white solid (1.92 g, 98%). R_f = 0.34 (50% EtOAc/*n*-hexane). ^{1}H NMR (400 MHz, $CDCl_3$) δ 7.47–7.33 (m, 5 H, 5 × Ph-CH), 7.24–7.20 (m, 2 H, 2 × Ar-CH), 7.08–7.02 (m, 2 H, 2 × Ar-CH), 5.40 (d, *J* = 7.2 Hz, 2 H, C*H*HOC*H*H), 5.13 (d, *J* = 7.2 Hz, 2 H, CH*H*OCH*H*), 5.10 (s, 2 H, $PhCH_2$), 3.71 (s, 3 H, CO_2CH_3), 3.09 (t, *J* = 7.6 Hz, 2 H, SCH_2), 2.73 (t, *J* = 7.6 Hz, 2 H, $CH_2CO_2CH_3$). The observed characterization data (R_f, ^{1}H) were consistent with that previously reported.[6]

3-(4-(Benzyloxy)phenyl)oxetane-3-sulfonyl fluoride (132)

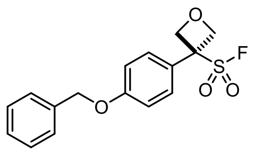

Prepared according to **oxetane sulfinate elimination procedure 1 (OSE-1)** from sulfone **132c** and submitting the sulfinate intermediate directly to the fluorination conditions. Oxetane sulfone **132c** (390 mg, 1.0 mmol, 1.0 equiv) was added to a solution of NaH (60% in mineral oil, 42 mg, 1.05 mmol, 1.05 equiv) in THF (5.0 mL, 0.2 M). The reaction mixture was stirred at 25 °C for 1 h then quenched with anhydrous MeOH (5.0 mL) and concentrated under reduced pressure to afford sulfinate salt as a white solid, which was subjected directly to the next step without spectroscopical analysis. Selectfluor™ (531 mg, 1.5 mmol, 1.5 equiv) was added to anhydrous MeOH (5.0 mL, 0.2 M) at 0 °C in a 25 mL round bottom flask, then stirred for 5 min. Sulfinate salt was added portionwise to the cooled reaction mixture. The reaction mixture was stirred at 0 °C for 1 h then concentrated under reduced pressure. The residue was diluted with CH_2Cl_2 (30 mL) and filtered through a plug of Celite, eluting with further CH_2Cl_2 (100 mL). After concentration under reduced pressure, purification by flash column chromatography through a short column (80% CH_2Cl_2/pentane) afforded oxetane sulfonyl fluoride **132** as a white solid (249 mg, 77%). R_f = 0.38 (80% CH_2Cl_2/pentane); ^{1}H NMR (400 MHz, $CDCl_3$) δ 7.45–7.38 (m, 5 H, 5 × Ph-CH), 7.22–7.20 (m, 2 H, 2 × Ar-CH), 7.07–7.05 (m, 2 H, 2 × Ar-CH), 5.43 (d, *J* = 7.5 Hz, 2 H, C*H*HOC*H*H), 5.23 (d, *J* = 7.5 Hz, 2 H, CH*H*OCH*H*), 5.11 (s, 2 H, $PhCH_2$); ^{19}F{^{1}H} NMR (377 MHz, $CDCl_3$) δ 34.4. The observed characterization data (R_f, ^{1}H) were consistent with that previously reported.[6]

Oxetane Sulfonyl Fluoride 133

Methyl 3-((3-(4-((3-fluorobenzyl)oxy)phenyl)oxetan-3-yl)thio)propanoate (133b)

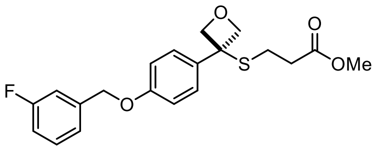

1-(Bromomethyl)-3-fluorobenzene (246 mL, 2.0 mmol, 2.0 equiv) was added to a solution of phenol sulfide **131** (270 mg, 1.0 mmol, 1.0 equiv) and K_2CO_3 (414 mg, 3.0 mmol, 3.0 equiv) in non-anhydrous acetone (5.0 mL, 0.2 M) in a 25 mL round-bottom flask. The reaction mixture was stirred at 40 °C for 16 h then diluted with EtOAc (30 mL). The organic layer was washed with water (3 × 30 mL) and brine (20 mL), dried with Na_2SO_4, filtered and concentrated *in vacuo* using a rotatory evaporator. Purification by flash column chromatography (30% EtOAc/pentane) afforded the oxetane sulfide **133b** as a colorless oil (354 mg, 94%). R_f = 0.30 (30% EtOAc/pentane); IR (film)/cm^{-1} 2950, 2870, 1733 (C=O st), 1608, 1592, 1510, 1239, 1221, 1180, 982, 783, 684; ^{1}H NMR (400 MHz, $CDCl_3$) δ 7.40–7.32 (m, 1 H, $Ar_{(Ph\text{-}F)}$-CH), 7.23–7.11 (m, 4 H, 2 × $Ar_{(Ph\text{-}F)}$-CH + 2 × Ar-CH), 7.03 (td, *J* = 8.5, 2.6 Hz, 1 H, $Ar_{(Ph\text{-}F)}$-CH), 6.99–6.94 (m, 2 H, 2 × Ar-CH), 5.19 (d, *J* = 6.4 Hz, 2 H, C*H*HOC*H*H), 5.07 (s, 2 H, Ar-C_q-OCH_2), 4.90 (d, *J* = 6.4 Hz, 2 H, CH*H*OCH*H*), 3.68 (s, 3 H, CO_2CH_3), 2.68 (t, *J* = 7.4 Hz, 2 H, SCH_2), 2.43 (t, *J* = 7.4 Hz, 2 H, $CH_2CO_2CH_3$); ^{13}C NMR (101 MHz, $CDCl_3$) δ 172.1 (C_q=O), 163.0 (d, $^1J_{C\text{-}F}$ = 246.5 Hz, $Ar_{(Ph\text{-}F)}$-C_q-F), 157.6 (Ar-C_q-OCH_2), 139.4 (d, $^3J_{C\text{-}F}$ = 7.3 Hz, $Ar_{(Ph\text{-}F)}$-C_q), 134.9 (Ar-C_q-C_q), 130.2 (d, $^3J_{C\text{-}F}$ = 8.2 Hz, $Ar_{(Ph\text{-}F)}$-CH), 127.6 (2 × Ar-CH), 122.7 (d, $^4J_{C\text{-}F}$ = 3.0 Hz, $Ar_{(Ph\text{-}F)}$-CH), 114.9 (2 × Ar-CH), 114.9 (d, $^2J_{C\text{-}F}$ = 21.2 Hz, $Ar_{(Ph\text{-}F)}$-CH), 114.2 (d, $^2J_{C\text{-}F}$ = 22.0 Hz, $Ar_{(Ph\text{-}F)}$-CH), 83.2 (CH_2OCH_2), 69.3 (d, $^5J_{C\text{-}F}$ = 2.0 Hz, Ar-C_q-OCH_2), 52.2 (C_q), 51.9 (CO_2CH_3), 34.1 ($CH_2CO_2CH_3$), 25.2 (SCH_2); ^{19}F{^{1}H} NMR (377 MHz, $CDCl_3$) δ –112.7; HRMS (TOF-MS-ES$^+$) *m/z* calcd for $C_{20}H_{21}O_4FNaS^+$ [M+Na]$^+$: 399.1042, found: 399.1042.

Methyl 3-((3-(4-((3-fluorobenzyl)oxy)phenyl)oxetan-3-yl)sulfonyl)propanoate (133c)

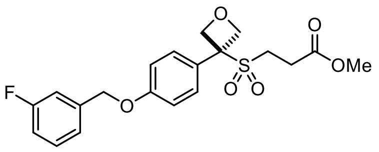

*m*CPBA (≤77%, 605 mg, 2.7 mmol, 3.0 equiv) was added to a solution of oxetane sulfide **133b** (337 mg, 0.8 mmol, 1.0 equiv) in CH_2Cl_2 (18 mL, 0.05 M) in a 50 mL round-bottom flask. The reaction mixture was stirred at 25 °C for 3 h then aq. KOH (3 M, 10 mL) was added and the phases were separated. The aqueous portion was extracted with CH_2Cl_2 (4 × 20 mL). The organic extracts were combined, dried over Na_2SO_4, filtered and concentrated *in vacuo* using a rotatory evaporator to afford sulfone **133c** as a white solid (372 mg, quant.). R_f = 0.51 (50% EtOAc/pentane); mp = 103–105 °C; IR (film)/cm^{-1} 2950, 2883, 1735 (C=O st), 1610, 1515, 1300 (SO_2 st as), 1251, 1182, 1131 (SO_2 st sy), 997, 814, 783; ^{1}H NMR (400 MHz, $CDCl_3$) δ 7.40–7.33 (m, 1 H, $Ar_{(Ph\text{-}F)}$-CH), 7.25–7.12 (m, 4 H, 2 × $Ar_{(Ph\text{-}F)}$-CH + 2 × Ar-CH), 7.06–6.99 (m, 3 H, $Ar_{(Ph\text{-}F)}$-CH + 2 × Ar-CH), 5.39 (d, *J* = 7.2 Hz, 2 H, C*H*HOC*H*H), 5.15–5.05 (m, 4 H, CH*H*OCH*H* + Ar-C_q-OCH_2), 3.70 (s, 3 H, CO_2CH_3), 3.09 (t, *J* = 7.6 Hz, 2 H, SO_2CH_2), 2.72 (t, *J* = 7.6 Hz, 2 H, $CH_2CO_2CH_3$); ^{13}C NMR (101 MHz, $CDCl_3$) δ 170.8 (C_q=O), 163.0 (d, $^1J_{C\text{-}F}$ = 246.5 Hz, $Ar_{(Ph\text{-}F)}$-C_q-F), 159.2 (Ar-C_q-OCH_2), 139.0 (d, $^3J_{C\text{-}F}$ = 7.3 Hz, $Ar_{(Ph\text{-}F)}$-C_q), 130.3 (d, $^3J_{C\text{-}F}$ = 8.2 Hz, $Ar_{(Ph\text{-}F)}$-CH), 129.6 (2 × Ar-CH), 126.1 (Ar-C_q-C_q), 122.7 (d, $^4J_{C\text{-}F}$ = 3.0 Hz, $Ar_{(Ph\text{-}F)}$-CH), 115.4 (2 × Ar-CH), 115.1 (d, $^2J_{C\text{-}F}$ = 21.2 Hz, $Ar_{(Ph\text{-}F)}$-CH), 114.2 (d, $^2J_{C\text{-}F}$ = 22.1 Hz, $Ar_{(Ph\text{-}F)}$-CH), 76.3 (CH_2OCH_2), 69.3 (Ar-C_q-OCH_2), 68.3 (C_q), 52.5 (CO_2CH_3), 43.3 ($CH_2CO_2CH_3$), 25.9 (SO_2CH_2); ^{19}F{^{1}H} NMR (377 MHz, $CDCl_3$) δ –112.5; HRMS (TOF-MS-ES$^+$) *m/z* calcd for $C_{20}H_{21}O_6FNaS^+$ [M+Na]$^+$: 431.0941, found: 431.0938.

3-(4-((3-Fluorobenzyl)oxy)phenyl)oxetane-3-sulfonyl fluoride (133)

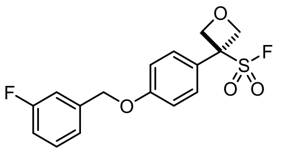

Prepared according to **oxetane sulfinate elimination procedure 1 (OSE-1)** from sulfone **133c** and submitting the sulfinate intermediate directly to the fluorination conditions. Oxetane sulfone **133c** (327 mg, 0.8 mmol, 1.0 equiv) was added to a solution of NaH (60% in mineral oil, 32 mg, 0.84 mmol, 1.05 equiv) in THF (5.0 mL, 0.2 M). The reaction mixture was stirred at 25 °C for 1 h then quenched with anhydrous MeOH (6.0 mL) and concentrated under reduced pressure to afford sulfinate salt as a pale-yellow solid, which was subjected directly to the next step without spectroscopical analysis. Selectfluor^{TM} (424.8 mg, 1.2 mmol, 1.5 equiv) was added to anhydrous MeOH (4.0 mL, 0.2 M) at 0 °C in a 10 mL round bottom flask, then stirred for 5 min. Sulfinate salt was added portionwise to the cooled reaction mixture. The reaction mixture was stirred at 0 °C for 1 h then concentrated under reduced pressure. The residue was diluted with CH_2Cl_2 (20 mL) and filtered through a plug of Celite, eluting with further CH_2Cl_2 (100 mL). After concentration *in vacuo* using a rotatory evaporator, the crude residue was filtered through a pad of silica on a sintered funnel, eluting with further 50% CH_2Cl_2/pentane (100 mL). Concentration of the filtrate afforded oxetane sulfonyl fluoride **133** as an off-white solid (186 mg, 69%). R_f = 0.26 (60% CH_2Cl_2/pentane); mp = 103–107 °C; IR (film)/cm^{-1} 2926, 2892, 1608, 1592, 1513, 1398, 1251, 1213, 1001, 908, 781, 731; ^{1}H NMR (500 MHz, $CDCl_3$) δ 7.37 (td, *J* = 8.0, 5.8 Hz, 1 H, $Ar_{(Ph-F)}$-CH), 7.22–7.18 (m, 3 H, $Ar_{(Ph-F)}$-CH + 2 × Ar-CH), 7.17–7.14 (m, 1 H, $Ar_{(Ph-F)}$-CH), 7.06–7.01 (m, 3 H, $Ar_{(Ph-F)}$-CH + 2 × Ar-CH), 5.42 (d, *J* = 8.0 Hz, 2 H, C*H*HOC*H*H), 5.22 (dd, *J* = 7.8, J_{H-F} = 1.3 Hz, 2 H, CH*H*OCH*H*), 5.10 (s, 2 H, Ar-C_q-OCH_2); ^{13}C NMR (126 MHz, $CDCl_3$) δ 163.0 (d, $^1J_{C-F}$ = 246.6 Hz, $Ar_{(Ph-F)}$-C_q-F), 159.7 (Ar-C_q-OCH_2), 138.8 (d, $^3J_{C-F}$ = 7.4 Hz, $Ar_{(Ph-F)}$-C_q), 130.3 (d, $^3J_{C-F}$ = 8.2 Hz, $Ar_{(Ph-F)}$-CH), 129.7 (2 × Ar-CH), 123.9 (Ar-C_q-C_q), 122.7 (d, $^4J_{C-F}$ = 3.0 Hz, $Ar_{(Ph-F)}$-CH), 115.5 (2 × Ar-CH), 115.1 (d, $^2J_{C-F}$ = 21.2 Hz, $Ar_{(Ph-F)}$-CH), 114.2 (d, $^2J_{C-F}$ = 22.1 Hz, $Ar_{(Ph-F)}$-CH), 77.1 (CH_2OCH_2), 69.4 (Ar-C_q-OCH_2), 68.1 (d, $^2J_{C-F}$ = 14.9 Hz, C_q); ^{19}F{^{1}H} NMR (377 MHz, $CDCl_3$) δ 34.4 (SO_2F), –112.5 (Ar-F); HRMS (FTMS–ES$^+$) *m/z* calcd for $C_{16}H_{14}FO_2^+$ $[M–SO_2F]^+$: 257.0972, found: 257.0975.

Oxetane Sulfonyl Fluoride 134

Methyl 3-((3-(4-(prop-2-yn-1-yloxy)phenyl)oxetan-3-yl)thio)propanoate (134b)

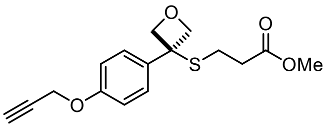

Propargyl bromide (~80% solution in toluene, 0.43 mL, 4.0 mmol, 2.0 equiv) was added to a solution of phenol sulfide **131** (540 mg, 2.0 mmol, 1.0 equiv) and K_2CO_3 (830 mg, 6.0 mmol, 3.0 equiv) in non-anhydrous acetone (10 mL, 0.2 M) in a 25 mL round-bottom flask. The reaction mixture was stirred at 40 °C for 16 h then diluted with EtOAc (50 mL). The organic layer was washed with water (3 × 50 mL) and brine (30 mL), dried with Na_2SO_4, filtered and concentrated *in vacuo* using a rotatory evaporator. If required, purification by flash column chromatography (20% EtOAc/pentane) afforded the oxetane sulfide **134b** as a yellow oil (612 mg, quant.). R_f = 0.18 (20% EtOAc/pentane); IR (film)/cm^{-1} 3285 (≡C-H st), 2948, 2870, 1734 (C=O st), 1608, 1511, 1434, 1217, 1019, 833; ^{1}H NMR (400 MHz, $CDCl_3$) δ 7.15 (d, *J* = 8.8 Hz, 2 H, 2 × Ar-CH), 6.97 (d, *J* = 8.8 Hz, 2 H, 2 × Ar-CH), 5.18 (d, *J* = 6.4 Hz, 2 H, C*H*HOC*H*H), 4.89 (d, *J* = 6.4 Hz, 2 H, CH*H*OCH*H*), 4.70 (d, *J* = 2.4 Hz, 2 H, C*H*$_2$C≡CH), 3.67 (s, 3 H, CO_2CH_3), 2.67 (t, *J* = 7.4 Hz, 2 H, SCH_2), 2.54 (t, *J* = 2.4 Hz, 1 H, C≡CH), 2.42 (t, J = 7.4 Hz, 2 H, C*H*$_2$$CO_2CH_3$); ^{13}C NMR (101 MHz, $CDCl_3$) δ 172.1 (C_q=O), 156.6 (Ar-C_q-OCH_2), 135.3 (Ar-C_q-C_q), 127.5 (2 × Ar-CH), 115.0 (2 × Ar-CH), 83.2 (CH_2OCH_2), 78.3 (C_q≡CH), 75.8 (C_q≡*C*H), 55.9 (*C*H_2C≡CH), 52.2 (C_q), 51.9 (CO_2*C*H_3), 34.1 (*C*$H_2CO_2CH_3$), 25.2 (SCH_2); HRMS (TOF-MS-ES$^+$) *m/z* calcd for $C_{16}H_{19}O_4S^+$ $[M+H]^+$: 307.1004, found: 307.1006.

Methyl 3-((3-(4-(prop-2-yn-1-yloxy)phenyl)oxetan-3-yl)sulfonyl)propanoate (134c)

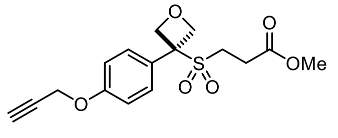

*m*CPBA (≤77%, 1.34 g, 6.0 mmol, 3.0 equiv) was added to a solution of oxetane sulfide **134b** (612 mg, 2.0 mmol, 1.0 equiv) in CH_2Cl_2 (40 mL, 0.05 M) in a 100 mL round-bottom flask. The reaction mixture was stirred at 25 °C for 3 h then aq. KOH (3 M, 50 mL) was added and the phases were separated. The aqueous portion was extracted with CH_2Cl_2 (4 × 60 mL). The organic extracts were combined, dried over Na_2SO_4, filtered and concentrated *in vacuo* using a rotatory evaporator to afford sulfone **134c** as a pale-orange solid (680 mg, quant.). R_f = 0.25 (30% EtOAc/pentane); mp = 124–126 °C; IR (film)/cm^{-1} 3272 (≡C-H st), 2921, 2854, 1737 (C=O st), 1609, 1512, 1441, 1368, 1305 (SO_2 st as), 1247, 1226, 1184, 1136 (SO_2 st sy), 1019, 838; ^{1}H NMR (400 MHz, $CDCl_3$) δ 7.24 (d, *J* = 9.0 Hz, 2 H, 2 × Ar-CH), 7.05 (d, *J* = 8.8 Hz, 2 H, 2 × Ar-CH), 5.39 (d, *J* = 7.2 Hz, 2 H, C*H*HOC*H*H), 5.12 (d, *J* = 7.3 Hz, 2 H, CH*H*OCH*H*), 4.73 (d, *J* = 2.4 Hz, 2 H, C*H*₂C≡CH), 3.70 (s, 3 H, CO_2CH_3), 3.09 (dd, *J* = 8.2, 7.1 Hz, 2 H, SO_2CH_2), 2.72 (t, *J* = 7.6 Hz, 2 H, $CH_2CO_2CH_3$), 2.56 (t, *J* = 2.4 Hz, 1 H, C≡CH); ^{13}C NMR (101 MHz, $CDCl_3$) δ 170.8 (C_q=O), 158.2 (Ar-C_q-OCH_2), 129.5 (2 × Ar-CH), 126.5 (Ar-C_q-C_q), 115.5 (2 × Ar-CH), 78.0 (C_q≡CH), 76.3 (CH_2OCH_2), 76.2 (C_q≡*C*H), 68.3 (C_q), 55.9 (*C*H_2C≡CH), 52.5 (CO_2*C*H_3), 43.3 (*C*$H_2CO_2CH_3$), 25.9 (SO_2CH_2); HRMS (TOF-MS-ES$^+$) *m/z* calcd for $C_{16}H_{19}O_6S^+$ [M+H]$^+$: 339.0902, found: 339.0913.

3-(4-(Prop-2-yn-1-yloxy)phenyl)oxetane-3-sulfonyl fluoride (134)

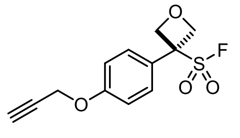

Prepared according to **oxetane sulfinate elimination procedure 3 (OSE-3)** from sulfone **134c** and submitting the sulfinate intermediate directly to the fluorination conditions. Sodium methoxide (5.4 M solution in MeOH, 0.35 mL, 1.88 mmol, 1.0 equiv) was added dropwise to a solution of oxetane sulfone **134c** (637 mg, 1.88 mmol, 1.0 equiv) in anhydrous THF (3.76 mL, 0.5 M) in a 25 mL round-bottom flask. A color change to dark brown was observed soon after addition of the base. The reaction mixture was stirred at 25 °C for 20 min, then concentrated *in vacuo* using a rotatory evaporator to afford oxetane sulfinate as a brown residue, which was subjected directly to the next step without spectroscopical analysis. For the fluorination step, pre-cooled (0 °C) anhydrous MeOH (9.4 mL, 0.2 M) and Selectfluor™ (998 mg, 2.82 mmol, 1.5 equiv) were added sequentially to the oxetane sulfinate salt at 0 °C, then stirred at 0 °C for 1 h. The reaction mixture was concentrated *in vacuo* using a rotary evaporator. The resulting residue was diluted with CH_2Cl_2 (20 mL) and filtered through a plug of Celite, eluting with further CH_2Cl_2 (40 mL). After concentration *in vacuo* using a rotatory evaporator, purification by flash column chromatography through a short column (60% CH_2Cl_2/pentane) afforded oxetane sulfonyl fluoride **134** as a white solid (354 mg, 75%). R_f = 0.41 (50% CH_2Cl_2/pentane); mp = 83–86 °C; IR (film)/cm^{-1} 3291 (≡C-H st), 2892, 1608, 1512, 1398, 1212, 1185, 1019, 836, 786, 618; ^{1}H NMR (400 MHz, $CDCl_3$) δ 7.22 (d, *J* = 8.8 Hz, 2 H, 2 × Ar-CH), 7.07 (d, *J* = 8.9 Hz, 2 H, 2 × Ar-CH), 5.43 (d, *J* = 7.6 Hz, 2 H, C*H*HOC*H*H), 5.23 (d, *J* = 6.6 Hz, 2 H, CH*H*OCH*H*), 4.74 (d, *J* = 2.4 Hz, 2 H, C*H*₂C≡CH), 2.56 (t, *J* = 2.4 Hz, 1 H, C≡CH). ^{13}C NMR (126 MHz, $CDCl_3$) δ 158.7 (Ar-C_q-OCH_2), 129.6 (2 × Ar-CH), 124.3 (Ar-C_q-C_q), 115.6 (2 × Ar-CH), 77.8 (C_q≡CH), 77.1 (CH_2OCH_2), 76.2 (C_q≡*C*H), 68.0 (d, $^2J_{C\text{-}F}$ = 14.7 Hz, C_q), 55.9 (*C*H_2C≡CH); ^{19}F{^{1}H} NMR (377 MHz, $CDCl_3$) δ 34.5; HRMS (TOF-MS-ES$^+$) *m/z* calcd for $C_{12}H_{11}O_2^+$ [M–SO_2F]$^+$: 187.0759, found: 187.0750.

Oxetane Sulfonyl Fluoride 135

Methyl 3-((3-(4-(allyloxy)phenyl)oxetan-3-yl)thio)propanoate (135b)

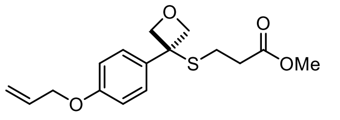

Allyl bromide (175 mL, 2.0 mmol, 2.0 equiv) was added to a solution of phenol sulfide **131** (270 mg, 1.0 mmol, 1.0 equiv) and K_2CO_3 (415 mg, 3.0 mmol, 3.0 equiv) in non-anhydrous acetone (5.0 mL, 0.2 M) in a 25 mL round-bottom flask. The reaction mixture was stirred at 40 °C for 16 h then diluted with EtOAc (30 mL). The organic layer was washed with water (3 × 30 mL) and brine (20 mL), dried with Na_2SO_4, filtered and concentrated *in vacuo* using a rotatory evaporator. If required, purification by flash column chromatography (20% EtOAc/pentane) afforded the oxetane sulfide **135b** as a colorless oil (307 mg, quant.). R_f = 0.53 (30% EtOAc/pentane); IR (film)/cm^{-1} 2950, 2870, 2339, 1753 (C=O st), 1608 (C=C st), 1511, 1436, 1243, 1180, 986, 829; ^{1}H NMR (400 MHz, $CDCl_3$) δ 7.13 (d, *J* = 8.2 Hz, 2 H, 2 × Ar-CH), 6.92 (d, *J* = 8.3 Hz, 2 H, 2 × Ar-CH), 6.14–5.99 (m, 1 H, CH_2CH=CH_2), 5.43 (dd, *J* = 17.3, 2.1 Hz, 1 H, CH_2CH=C*H*H), 5.34–5.25 (m, 1 H, CH=CH*H*), 5.19 (d, *J* = 6.3 Hz, 2 H, C*H*HOC*H*H), 4.90 (d, *J* = 6.3 Hz, 2 H, CH*H*OCH*H*), 4.60–4.49 (m, 2 H, C*H*$_2$CH=CH_2), 3.68 (s, 3 H, CO_2CH_3), 2.67 (t, *J* = 7.4 Hz, 2 H, SCH_2), 2.42 (t, *J* = 7.4 Hz, 2 H, C*H*$_2CO_2CH_3$); ^{13}C NMR (101 MHz, $CDCl_3$) δ 172.1 (C_q=O), 157.7 (Ar-*C*$_q$-OCH_2), 134.4 (Ar-*C*$_q$-C_q), 133.1 (CH_2*C*H=CH_2), 127.5 (2 × Ar-CH), 117.9 (CH_2CH=*C*H$_2$), 114.8 (2 × Ar-CH), 83.2 (CH_2OCH_2), 68.9 (*C*H$_2$CH=CH_2), 52.2 (C_q), 51.9 (CO_2CH_3), 34.1 (*C*H$_2CO_2CH_3$), 25.2 (SCH_2); HRMS (TOF-MS-ES$^+$) *m/z* calcd for $C_{16}H_{24}NO_4S^+$ $[M+NH_4]^+$: 326.1426, found: 326.1423.

Methyl 3-((3-(4-(allyloxy)phenyl)oxetan-3-yl)sulfonyl)propanoate (135c)

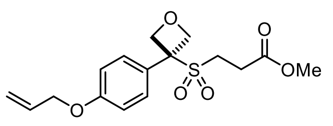

*m*CPBA (≤77%, 493 mg, 2.2 mmol, 2.2 equiv) was added to a solution of oxetane sulfide **135b** (307 mg, 1.0 mmol, 1.0 equiv) in CH_2Cl_2 (20 mL, 0.05 M) in a 50 mL round-bottom flask. The reaction mixture was stirred at 25 °C for 3 h then aq. KOH (3 M, 30 mL) was added and the phases were separated. The aqueous portion was extracted with CH_2Cl_2 (4 × 50 mL). The organic extracts were combined, dried over Na_2SO_4, filtered and concentrated *in vacuo* using a rotatory evaporator to afford sulfone **135c** as a white solid (306 mg, 90%). R_f = 0.19 (30% EtOAc/pentane); mp = 92–95 °C; IR (film)/cm^{-1} 2950, 2883, 2339, 1737 (C=O st), 1608 (C=C st), 1513, 1305 (SO_2 st as), 1251, 1183, 1139 (SO_2 st sy), 997, 930, 837; ^{1}H NMR (400 MHz, $CDCl_3$) δ 7.20 (d, *J* = 8.8 Hz, 2 H, 2 × Ar-CH), 6.97 (d, *J* = 8.8 Hz, 2 H, 2 × Ar-CH), 6.04 (ddt, *J* = 17.3, 10.5, 5.3 Hz, 1 H, CH_2C*H*=CH_2), 5.46–5.35 (m, 3 H, CH=C*H*H + C*H*HOC*H*H), 5.31 (dd, *J* = 10.5, 1.4 Hz, 1 H, CH=CH*H*), 5.11 (d, *J* = 7.2 Hz, 2 H, CH*H*OCH*H*), 4.56 (dt, *J* = 5.3, 1.4 Hz, 2 H, C*H*$_2$CH=CH_2), 3.69 (s, 3 H, CO_2CH_3), 3.07 (t, *J* = 7.7 Hz, 2 H, SO_2CH_2), 2.69 (t, *J* = 7.7 Hz, 2 H, C*H*$_2CO_2CH_3$); ^{13}C NMR (101 MHz, $CDCl_3$) δ 170.8 (C_q=O), 159.4 (Ar-*C*$_q$-OCH_2), 132.7 (CH_2*C*H=CH_2), 129.5 (2 × Ar-CH), 125.7 (Ar-*C*$_q$-C_q), 118.1 (CH_2CH=*C*H$_2$), 115.3 (2 × Ar-CH), 76.3 (CH_2OCH_2), 68.9 (*C*H$_2$CH=CH_2), 68.3 (C_q), 52.5 (CO_2CH_3), 43.3 (*C*H$_2CO_2CH_3$), 25.9 (SCH_2); HRMS (TOF-MS-ES$^+$) *m/z* calcd for $C_{16}H_{24}NO_6S^+$ $[M+NH_4]^+$: 358.1324, found: 358.1315.

3-(4-(Allyloxy)phenyl)oxetane-3-sulfonyl fluoride (135)

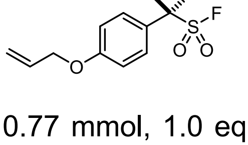

Prepared according to **oxetane sulfinate elimination procedure 3 (OSE-3)** from sulfone **135c** and submitting the sulfinate intermediate directly to the fluorination conditions. Sodium methoxide (5.4 M solution in MeOH, 0.14 mL, 0.77 mmol, 1.0 equiv) was added dropwise to a solution of oxetane sulfone **135c** (263 mg, 0.77 mmol, 1.0 equiv) in anhydrous THF (1.54 mL, 0.5 M) in a 10 mL round-bottom flask. A color change to dark brown was observed soon after addition of the base. The reaction mixture was stirred at 25 °C for 20 min, then concentrated *in vacuo* using a rotatory evaporator to afford oxetane sulfinate as a white paste, which was subjected directly to the next step without spectroscopical analysis. For the fluorination step, pre-cooled (0 °C) anhydrous MeOH (4.0 mL, 0.2 M) and Selectfluor™ (409 mg,

1.16 mmol, 1.5 equiv) were added sequentially to the oxetane sulfinate salt at 0 °C, then stirred at 0 °C for 1 h. The reaction mixture was concentrated *in vacuo* using a rotary evaporator. The resulting residue was diluted with CH_2Cl_2 (10 mL) and filtered through a plug of Celite, eluting with further CH_2Cl_2 (40 mL). After concentration *in vacuo* using a rotatory evaporator, purification by flash column chromatography through a short column (80% CH_2Cl_2/pentane) afforded oxetane sulfonyl fluoride **135** as a white solid (105 mg, 50%). R_f = 0.35 (50% CH_2Cl_2/pentane); mp = 62–64 °C; IR (film)/cm^{-1} 2991, 2892, 1610 (C=C st), 1513, 1398 (SO_2 st as), 1254, 1213 (SO_2 st sy), 1183, 997, 931, 833, 784; ^{1}H NMR (400 MHz, $CDCl_3$) δ 7.20 (d, *J* = 8.8 Hz, 2 H, 2 × Ar-CH), 7.00 (d, *J* = 8.8 Hz, 2 H, 2 × Ar-CH), 6.06 (ddt, *J* = 17.3, 10.6, 5.3 Hz, 1 H, CH_2CH=CH_2), 5.48–5.39 (m, 3 H, CH=C*H*H + C*H*HOC*H*H), 5.33 (dd, *J* = 10.6, 1.5 Hz, 1 H, CH=CH*H*), 5.27–5.21 (m, 2 H, CH*H*OCH*H*), 4.59 (dt, *J* = 5.3, 1.5 Hz, 2 H, C*H*$_2$CH=CH_2); ^{13}C NMR (126 MHz, $CDCl_3$) δ 159.8 (Ar-*C*$_q$-OCH_2), 132.6 (CH_2*C*H=CH_2), 129.5 (2 × Ar-CH), 123.4 (Ar-*C*$_q$-C_q), 118.2 (CH_2CH=*C*H$_2$), 115.4 (2 × Ar-CH), 77.1 (CH_2OCH_2), 68.9 (*C*H$_2$CH=CH_2), 68.1 (d, $^2J_{C\text{-}F}$ = 14.7 Hz, C_q); ^{19}F{^{1}H} NMR (377 MHz, $CDCl_3$) δ 34.4; HRMS (TOF-MS-ES$^+$) *m/z* calcd for $C_{12}H_{13}O_2^+$ [M–SO_2F]$^+$: 189.0916, found: 189.0915.

***135** was further characterized by X-ray crystallography (see Fig. **S24–S26**). Crystals suitable for X-ray analysis were grown by slow evaporation from $CDCl_3$.*

Oxetane Sulfonyl Fluoride 136

Methyl 3-((3-(4-(2-ethoxy-2-oxoethoxy)phenyl)oxetan-3-yl)thio)propanoate (136b)

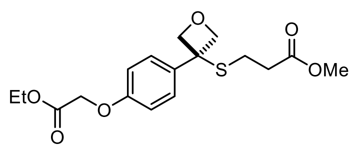

Ethyl 2-bromoacetate (338 mL, 3.0 mmol, 2.0 equiv) was added to a solution of phenol sulfide **131** (405 mg, 1.5 mmol, 1.0 equiv) and K_2CO_3 (623 mg, 4.5 mmol, 3.0 equiv) in non-anhydrous acetone (7.5 mL, 0.2 M) in a 25 mL round-bottom flask. The reaction mixture was stirred at 40 °C for 16 h then diluted with EtOAc (40 mL). The organic layer was washed with water (3 × 40 mL) and brine (30 mL), dried with Na_2SO_4, filtered and concentrated *in vacuo* using a rotatory evaporator. If required, purification by flash column chromatography (30% EtOAc/pentane) afforded the oxetane sulfide **136b** as a colorless oil (530 mg, quant.). R_f = 0.25 (30% EtOAc/pentane); IR (film)/cm^{-1} 2950, 2872, 1756 (C=O st), 1735 (C=O st), 1608, 1511, 1243, 1198, 1082, 984, 833; ^{1}H NMR (400 MHz, $CDCl_3$) δ 7.14 (d, *J* = 8.8 Hz, 2 H, 2 × Ar-CH), 6.90 (d, *J* = 8.8 Hz, 2 H, 2 × Ar-CH), 5.17 (d, *J* = 6.4 Hz, 2 H, C*H*HOC*H*H), 4.88 (d, *J* = 6.4 Hz, 2 H, CH*H*OCH*H*), 4.62 (s, 2 H, Ar-O-CH_2), 4.32–4.26 (d, *J* = 6.4 Hz, 2 H, CO_2C*H*$_2$$CH_3$), 3.66 (s, 3 H, CO_2CH_3), 2.66 (t, *J* = 7.4 Hz, 2 H, SCH_2), 2.41 (t, *J* = 7.4 Hz, 2 H, C*H*$_2$$CO_2CH_3$), 1.30 (t, *J* = 7.1 Hz, 3 H, CO_2CH_2C*H*$_3$); ^{13}C NMR (101 MHz, $CDCl_3$) δ 172.1 (*C*O$_2$$CH_3$), 168.7 (*C*O$_2$$CH_2CH_3$), 156.9 (Ar-*C*$_q$-$OCH_2$), 135.4 (Ar-*C*$_q$-$C_q$), 127.6 (2 × Ar-CH), 114.8 (2 × Ar-CH), 83.1 (CH_2OCH_2), 65.5 (Ar-O-CH_2), 61.5 (CO_2*C*H$_2$$CH_3$), 52.1 ($C_q$), 51.9 ($CO_2$*C*H$_3$), 34.0 (*C*H$_2$$CO_2CH_3$), 25.2 ($SCH_2$), 14.2 ($CO_2CH_2$*C*H$_3$); HRMS (TOF-MS-ES$^+$) *m/z* calcd for $C_{17}H_{26}NO_6S^+$ [M+NH_4]$^+$: 372.1481, found: 372.1476.

Methyl 3-((3-(4-(2-ethoxy-2-oxoethoxy)phenyl)oxetan-3-yl)sulfonyl)propanoate (136c)

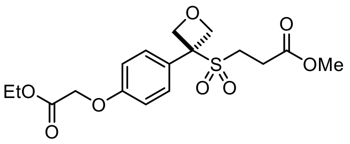

*m*CPBA (≤77%, 1.0 g, 4.5 mmol, 3.0 equiv) was added to a solution of oxetane sulfide **136b** (528 mg, 1.5 mmol, 1.0 equiv) in CH_2Cl_2 (30 mL, 0.05 M) in a 100 mL round-bottom flask. The reaction mixture was stirred at 25 °C for 3 h then aq. KOH (3 M, 40 mL) was added and the phases were separated. The aqueous portion was extracted with CH_2Cl_2 (4 × 50 mL). The organic extracts were combined, dried over Na_2SO_4, filtered and concentrated *in vacuo* using a rotatory evaporator to afford sulfone **136c** as a white solid (606 mg, quant.). R_f = 0.49 (50% Et_2O/pentane); mp = 131–133 °C; IR (film)/cm^{-1} 2952, 2885, 1735 (2 × C=O st), 1610, 1513, 1439, 1305 (SO_2 st as), 1200, 1183, 1136 (SO_2 st sy), 1081, 837; ^{1}H NMR (400 MHz, $CDCl_3$) δ 7.23 (d, *J* =

8.7 Hz, 2 H, 2 × Ar-CH), 6.97 (d, *J* = 8.7 Hz, 2 H, 2 × Ar-CH), 5.38 (d, *J* = 7.1 Hz, 2 H, C*H*HOC*H*H), 5.11 (d, *J* = 7.1 Hz, 2 H, CH*H*OCH*H*), 4.65 (s, 2 H, Ar-O-CH_2), 4.29 (q, *J* = 7.1 Hz, 2 H, $CO_2CH_2CH_3$), 3.70 (s, 3 H, CO_2CH_3), 3.08 (t, *J* = 7.6 Hz, 2 H, SO_2CH_2), 2.71 (t, *J* = 7.6 Hz, 2 H, $CH_2CO_2CH_3$), 1.31 (t, *J* = 7.1 Hz, 3 H, $CO_2CH_2CH_3$); ^{13}C NMR (101 MHz, $CDCl_3$) δ 170.8 (CO_2CH_3), 168.4 ($CO_2CH_2CH_3$), 158.5 (Ar-C_q-OCH_2), 129.6 (2 × Ar-CH), 126.7 (Ar-C_q-C_q), 115.2 (2 × Ar-CH), 76.3 (CH_2OCH_2), 68.2 (C_q), 65.3 (Ar-O-CH_2), 61.6 ($CO_2CH_2CH_3$), 52.5 (CO_2CH_3), 43.2 ($CH_2CO_2CH_3$), 25.9 (SO_2CH_2), 14.2 ($CO_2CH_2CH_3$); HRMS (TOF-MS-ES$^+$) *m/z* calcd for $C_{17}H_{23}O_8S^+$ [M+H]$^+$: 387.1114, found: 387.1131.

Ethyl 2-(4-(3-(fluorosulfonyl)oxetan-3-yl)phenoxy)acetate (136)

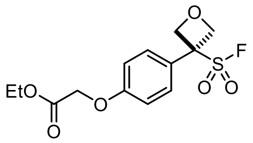

Sodium ethoxide (21 wt. % in ethanol, 379 mL, 1.17 mmol, 1.0 equiv) was added to a solution of oxetane sulfone **136c** (452 mg, 1.17 mmol, 1.0 equiv) in anhydrous CH_2Cl_2 (11.7 mL, 0.1 M) at 0 °C in a 50 mL round-bottom flask. A color change from a clear to yellow solution was observed soon after addition of the base. The reaction mixture was stirred at 0 °C for 15 min, then concentrated *in vacuo* using a rotatory evaporator to afford oxetane sulfinate as an orange solid, which was subjected directly to the next step without spectroscopical analysis. For the fluorination step, pre-cooled (0 °C) anhydrous EtOH (5.85 mL, 0.2 M) was added to the oxetane sulfinate salt and the resulting mixture sonicated for 2–5 min to aid solubility of the salt. Selectfluor^{TM} (621 mg, 1.76 mmol, 1.5 equiv) was added sequentially to the reaction mixture at 0 °C, then stirred at 0 °C for 1 h. The reaction mixture was concentrated *in vacuo* using a rotary evaporator. The resulting residue was diluted with CH_2Cl_2 (10 mL) and filtered through a plug of Celite, eluting with further CH_2Cl_2 (20 mL). After concentration *in vacuo* using a rotatory evaporator, purification by flash column chromatography through a short column (CH_2Cl_2) afforded oxetane sulfonyl fluoride **136** as a white solid (265 mg, 71%). R_f = 0.59 (CH_2Cl_2); mp = 127–129 °C; IR (film)/cm^{-1} 2890, 2359, 1751 (C=O st), 1610, 1515, 1395 (SO_2 st as), 1213 (SO_2 st sy), 1170, 1082, 831, 792, 624, 561; ^{1}H NMR (400 MHz, $CDCl_3$) δ 7.22 (d, *J* = 8.9 Hz, 2 H, 2 × Ar-CH), 7.00 (d, *J* = 8.9 Hz, 2 H, 2 × Ar-CH), 5.43 (d, *J* = 7.6 Hz, 2 H, C*H*HOC*H*H), 5.23 (dd, *J* = 7.8, $J_{H\text{-}F}$ = 1.5 Hz, 2 H, CH*H*OCH*H*), 4.67 (s, 2 H, OCH_2C_q=O), 4.30 (q, *J* = 7.2 Hz, 2 H, OCH_2CH_3), 1.32 (t, *J* = 7.1 Hz, 3 H, OCH_2CH_3); ^{13}C NMR (126 MHz, $CDCl_3$) δ 168.3 (C_q=O), 159.0 (Ar-C_q-OCH_2), 129.7 (2 × Ar-CH), 124.5 (Ar-C_q-C_q), 115.3 (2 × Ar-CH), 77.0 (CH_2OCH_2), 68.0 (d, $^2J_{C\text{-}F}$ = 14.9 Hz, C_q), 65.3 (Ar-O-CH_2), 61.6 ($CO_2CH_2CH_3$), 14.1 ($CO_2CH_2CH_3$); ^{19}F{^{1}H} NMR (377 MHz, $CDCl_3$) δ 34.6; HRMS (TOF-MS-ES$^+$) *m/z* calcd for $C_{13}H_{15}O_4^+$ [M–SO_2F]$^+$: 235.0970, found: 235.0967.

***136** was further characterized by X-ray crystallography (see Fig. **S27**). Crystals suitable for X-ray analysis were grown by slow evaporation from $CDCl_3$.*

Oxetane Sulfonyl Fluoride 137

Methyl 3-((3-(4-(2-((*tert*-butoxycarbonyl)amino)ethoxy)phenyl)oxetan-3-yl)thio)propanoate (137b)

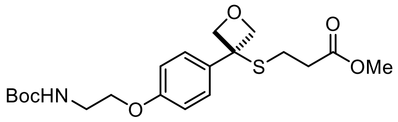

K_2CO_3 (830 mg, 6.0 mmol, 3.0 equiv), KI (332 mg, 2.0 mmol, 1.0 equiv), 2-(Boc-amino)ethyl bromide (1.03 g, 4.6 mmol, 2.3 equiv), and oxetane sulfide **131** (537 mg, 2.0 mmol, 1.0 equiv) were added to vial. Anhydrous DMF (10 mL, 0.2 M) was added, and the vial was sealed. The reaction mixture was stirred at 80 °C for 24 h. After cooling to room temperature, the reaction mixture was diluted with EtOAc (50 mL) and quenched with sat. aq. $Na_2S_2O_3$ (50 mL). The layers were separated, and the organic layer was washed with water (3 × 50 mL), followed by aq. NaOH (1M, 5 × 50 mL), followed by brine (50 mL). The organic layer was dried over anhydrous Na_2SO_4, filtered, and concentrated *in vacuo* using a rotary evaporator. Purification by flash column

chromatography (50% EtOAc/*n*-hexane) afforded oxetane sulfide **137b** as a yellow gum (418 mg, 51%). R_f = 0.33 (50% EtOAc/*n*-hexane); IR (film)/cm^{-1} 3375 (NH st), 2974, 2950, 2871, 1701 (2 × C=O st), 1507, 1364, 1243, 1162, 982, 909, 829; ^{1}H NMR (400 MHz, $CDCl_3$) δ 7.15 (d, *J* = 8.5 Hz, 2 H, 2 × Ar-CH), 6.90 (d, *J* = 8.5 Hz, 2 H, 2 × Ar-CH), 5.19 (d, *J* = 6.4 Hz, 2 H, C*H*HOC*H*H), 5.03 (s, 1 H, NH), 4.91 (d, *J* = 6.4 Hz, 2 H, CH*H*OCH*H*), 4.04 (t, *J* = 5.3 Hz, 2 H, OCH_2), 3.69 (s, 3 H, OCH_3), 3.56 (t, *J* = 5.3 Hz, 2 H, $NHCH_2$), 2.68 (t, *J* = 7.4 Hz, 2 H, SCH_2), 2.43 (t, *J* = 7.4 Hz, 2 H, CH_2CO_2Me), 1.46 (s, 9 H, 3 × CH_3); ^{13}C NMR (101 MHz, $CDCl_3$) δ 172.1 (C=O st), 157.7 (Ar-C_q-OCH_2), 155.9 (C=O), 134.7 (Ar-C_q-C_q), 127.6 (2 × Ar-CH), 114.5 (2 × Ar-CH), 83.2 (CH_2OCH_2), 79.6 ($C_q(CH_3)_3$), 67.3 (OCH_2), 52.2 (C_q), 51.9 (CO_2CH_3), 40.1 ($NHCH_2$), 34.1 (CH_2CO_2Me), 28.4 (3 × CH_3), 25.2 (SCH_2); HRMS (TOF-MS-ES$^+$) *m/z* calcd for $C_{20}H_{30}NO_6S^+$ [M+H]$^+$: 412.1794, found: 412.1798.

Methyl 3-((3-(4-(2-((*tert*-butoxycarbonyl)amino)ethoxy)phenyl)oxetan-3-yl)sulfonyl)propanoate (137c)

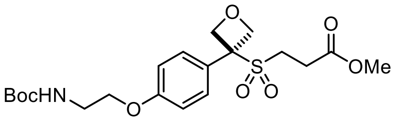

*m*CPBA (70%, 333 mg, 1.35 mmol, 3.0 equiv) was added to a solution of oxetane sulfide **137b** (185 mg, 0.45 mmol, 1.0 equiv) in CH_2Cl_2 (9 mL, 0.05 M). The reaction mixture was stirred at 25 °C for 3 h then aq. KOH (3 M, 20 mL) was added, and the phases were separated. The aqueous portion was extracted with CH_2Cl_2 (4 × 20 mL). The organic extracts were combined, dried over Na_2SO_4, filtered and concentrated *in vacuo*. Purification by automated flash column chromatography (70% EtOAc/*n*-hexane, see conditions below) afforded oxetane sulfone **137c** as a clear colorless gum (174 mg, 87%). R_f = 0.20 (60% EtOAc/*n*-hexane); IR (film)/cm^{-1} 3384 (NH st), 2978, 2885, 1707 (2 × C=O st), 1513, 1366, 1306 (SO_2 st as), 1250, 1172, 1138 (SO_2 st sy); ^{1}H NMR (400 MHz, $CDCl_3$) δ 7.23 (d, *J* = 8.6 Hz, 2 H, 2 × Ar-CH), 6.98 (d, *J* = 8.6 Hz, 2 H, 2 × Ar-CH), 5.40 (d, *J* = 7.2 Hz, 2 H, C*H*HOC*H*H), 5.13 (d, *J* = 7.2 Hz, 2 H, CH*H*OCH*H*), 5.02 (s, 1 H, NH), 4.06 (t, *J* = 5.2 Hz, 2 H, OCH_2), 3.72 (s, 3 H, OCH_3), 3.56 (t, *J* = 5.2 Hz, 2 H, NHC*H$_2$*), 3.09 (t, *J* = 7.6 Hz, 2 H, SO_2CH_2), 2.73 (t, *J* = 7.6 Hz, 2 H, C*H$_2$*CO_2CH_3), 1.47 (s, 9 H, $C(CH_3)_3$); ^{13}C NMR (101 MHz, $CDCl_3$) δ 170.8 (C=O), 159.4 (Ar-C_q-OCH_2), 155.9 (C=O), 129.5 (2 × Ar-CH), 125.9 (Ar-C_q-C_q), 115.0 (2 × Ar-CH), 79.7 ($C_q(CH_3)_3$), 76.3 (CH_2OCH_2), 68.3 (C_q), 67.4 (OCH_2), 52.5 (CO_2CH_3), 43.2 (CH_2CO_2Me), 40.0 ($NHCH_2$), 28.4 ($C_q(CH_3)_3$), 25.9 (SO_2CH_2); HRMS (TOF-MS-ES$^+$) *m/z* calcd for $C_{20}H_{30}NO_8S^+$ [M+H]$^+$: 444.1692, found: 444.1689.

Automated Column Conditions: Run on a Biotage® Selekt system. Column type: Biotage® Sfär® HC Duo 10 g. Flow rate: 40 mL/min. Sample mass: 300 mg. Solvent A: *n*-hexane, Solvent B: EtOAc. UV wavelength detection: l 200–400 nm. See trace and gradient below. From the left: peak 1 (yellow): impurity 1, peak 2 (green): impurity 2, peak 3 (red): oxetane sulfone **137c**

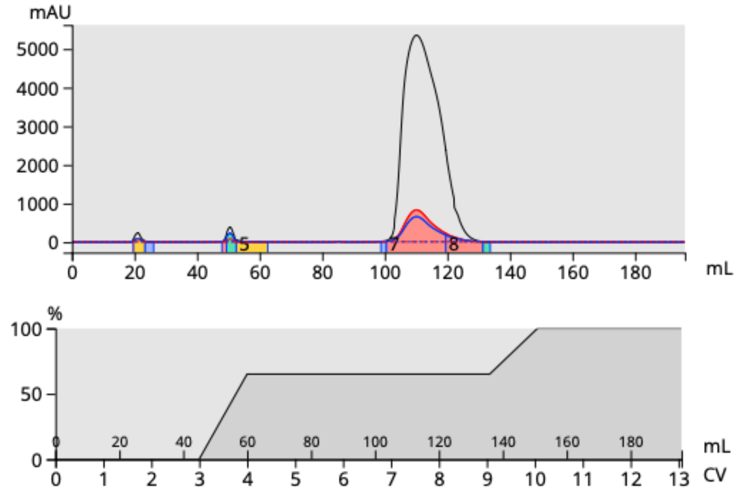

tert-Butyl (2-(4-(3-(fluorosulfonyl)oxetan-3-yl)phenoxy)ethyl)carbamate (137)

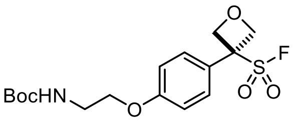

Prepared according to **oxetane sulfinate elimination procedure 2 (OSE-2)** from sulfone **137c** and submitting the sulfinate intermediate directly to the fluorination conditions. NaOH (5 M in MeOH, 90 mL, 0.45 mmol, 1.05 equiv) was added to a solution of sulfone **137c** (153 mg, 0.43 mmol, 1.0 equiv) in anhydrous CH_2Cl_2 (4.3 mL, 0.1 M) at 0 °C in a 10 mL round-bottom flask. The oxetane sulfinate salt precipitated after 5 min of the start of addition of base to form a white solid. The reaction mixture was stirred at 0 °C for 15 min then concentrated *in vacuo* using a rotary evaporator to afford the oxetane sulfinate as a white paste, which was subjected directly to the next step without spectroscopical analysis. For the fluorination step, pre-cooled (0 °C) anhydrous MeOH (2.15 mL, 0.2 M) and Selectfluor^{TM} (228 mg, 0.65 mmol, 1.5 equiv) were added sequentially to the oxetane sulfinate salt at 0 °C, then stirred at 0 °C for 1 h. The reaction mixture was concentrated *in vacuo* using a rotary evaporator. The resulting residue was diluted with CH_2Cl_2 (10 mL) and filtered through a plug of Celite, eluting with further CH_2Cl_2 (40 mL). After concentration *in vacuo* using a rotatory evaporator, purification by flash column chromatography through a short column (CH_2Cl_2) afforded oxetane sulfonyl fluoride **137** as a white gum (96.9 mg, 60%). R_f = 0.36 (CH_2Cl_2); IR (film)/cm^{-1} 3425 (NH st), 2978, 2339, 1705 (C=O st), 1610, 1513, 1400 (SO_2 st as), 1250, 1213 (SO_2 st sy), 1169, 1001, 781, 576; ^{1}H NMR (400 MHz, $CDCl_3$) δ 7.20 (d, *J* = 8.7 Hz, 2 H, 2 × Ar-CH), 6.98 (d, *J* = 8.7 Hz, 2 H, 2 × Ar-CH), 5.43 (d, *J* = 7.5 Hz, 2 H, C*H*HOC*H*H), 5.23 (d, *J* = 7.5 Hz, 2 H, CH*H*OCH*H*), 4.99 (br s, 1 H, NH), 4.06 (t, *J* = 5.1 Hz, 2 H, OCH_2), 3.56 (q, *J* = 5.5 Hz, 2 H, NHC*H*$_2$), 1.46 (s, 9 H, $C(CH_3)_3$); ^{13}C NMR (126 MHz, $CDCl_3$) δ 159.9 (Ar-*C*$_q$-OCH_2) 155.9 (C=O), 129.6 (2 × Ar-CH), 123.7 (Ar-*C*$_q$-C_q), 115.1 (2 × Ar-CH), 79.7 (*C*$_q$(CH_3)$_3$), 77.1 (CH_2OCH_2), 68.1 (d, $^2J_{C\text{-}F}$ = 14.7 Hz, C_q), 67.4 (OCH_2), 40.0 ($NHCH_2$), 28.4 (C_q(*C*H$_3$)$_3$); ^{19}F{^{1}H} NMR (377 MHz, $CDCl_3$) δ 34.4; HRMS (TOF-MS-ES$^+$) *m/z* calcd for $C_{16}H_{26}N_2O_6FS^+$ $[M+NH_4]^+$: 393.1496, found: 393.1477.

Oxetane Sulfonyl Fluoride 140

Methyl 3-((3-(4-(((trifluoromethyl)sulfonyl)oxy)phenyl)oxetan-3-yl)thio)propanoate (138)

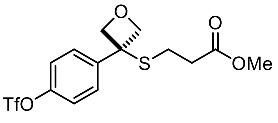

Pyridine (0.32 mL, 4.0 mmol, 2.0 equiv) and triflic anhydride (0.4 mL, 2.4 mmol, 1.2 equiv) were added sequentially to a solution of phenol sulfide **131** (536 mg, 2.0 mmol, 1.0 equiv) in anhydrous CH_2Cl_2 (20 mL, 0.1 M) in a 50 mL round-bottom flask at 0 °C. After warming to 25 °C and stirring for 1 h, the reaction mixture was quenched with water (50 mL). The phases were separated, and the aqueous layer was extracted with CH_2Cl_2 (3 × 50 mL). The combined organic layers were dried over anhydrous Na_2SO_4, filtered, and concentrated *in vacuo* using a rotary evaporator. Purification by flash column chromatography (50% Et_2O/pentane) afforded oxetane triflate **138** as a clear, colorless gum which solidified into a white solid in the freezer at –20 °C (1.82 g, 90%). R_f = 0.26 (50% Et_2O/pentane); ^{1}H NMR (400 MHz, $CDCl_3$) δ 7.37–7.27 (m, 4 H, 4 × Ar-CH), 5.17 (d, *J* = 6.6 Hz, 2 H, C*H*HOC*H*H), 4.93 (d, *J* = 6.6 Hz, 2 H, CH*H*OCH*H*), 3.68 (s, 3 H, OCH_3), 2.69 (t, *J* = 7.3 Hz, 2 H, SCH_2), 2.45 (t, *J* = 7.3 Hz, 2 H, C*H*2CO_2CH_3); ^{19}F{^{1}H} NMR (377 MHz, $CDCl_3$) δ –72.8; The observed characterization data (R_f, ^{1}H) were consistent with that previously reported.[6]

Methyl 3-((3-(4-chlorophenyl)oxetan-3-yl)thio)propanoate (140b)

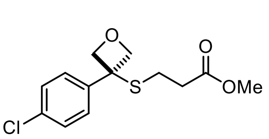

Using chlorination conditions developed by Buchwald,[20] oxetane triflate **138** (800 mg, 2.0 mmol, 1.0 equiv), KCl (300 mg, 4.0 mmol, 2.0 equiv), and KF (58 mg, 1.0 mmol, 0.5 equiv) were added to a reaction vial (1). The reaction vial (1) was sealed then evacuated and backfilled with Ar three times. $Pd_2(dba)_3$ (28 mg, 0.03 mmol, 1.5 mol%) and ^{t}BuBrettPhos (44 mg, 0.09 mmol, 4.5 mol%) were added to a

separate reaction vial (2). The reaction vial (2) was sealed then evacuated and backfilled with Ar three times. Anhydrous, degassed 1,4-dioxane (2 mL) was added to reaction vial (2) and the reaction mixture was stirred at 120 °C for 5 min. After cooling to 25 °C, the contents of reaction vial (2) were transferred to reaction vial (1) by syringe and the reaction mixture was diluted with further 1,4-dioxane (6 mL). The reaction mixture was then heated to 130 °C and stirred vigorously for 20 h. After cooling to 25 °C, the reaction mixture was filtered through a plug of Celite and eluted with Et_2O (25 mL). The filtrate was then concentrated *in vacuo*. Purification by flash column chromatography (20–30% Et_2O/pentane) afforded oxetane sulfide **140b** as a clear, yellow oil (245 mg, 43%). R_f = 0.25 (30% Et_2O/pentane); IR (film)/cm^{-1} 2949, 2871, 1733 (C=O st), 1491, 1436, 1358, 1245, 1219, 1172, 1013, 984, 926, 827, 528; ^{1}H NMR (400 MHz, $CDCl_3$) δ 7.35 (d, *J* = 8.5 Hz, 2 H, 2 × Ar-CH), 7.16 (d, *J* = 8.5 Hz, 2 H, 2 × Ar-CH), 5.16 (d, *J* = 6.5 Hz, 2 H, C*H*HOC*H*H), 4.89 (d, *J* = 6.5 Hz, 2 H, CH*H*OCH*H*), 3.67 (s, 3 H, CO_2CH_3), 2.66 (t, *J* = 7.3 Hz, 2 H, SCH_2), 2.43 (t, *J* = 7.3 Hz, 2 H, $CH_2CO_2CH_3$); ^{13}C NMR (101 MHz, $CDCl_3$) δ 171.9 (C_q=O), 140.9 (Ar-C_q-C_q), 133.3 (Ar-C_q-Cl), 128.9 (2 × Ar-CH), 127.7 (2 × Ar-CH), 82.8 (CH_2OCH_2), 52.0 (C_q), 51.9 (CO_2CH_3), 33.9 ($CH_2CO_2CH_3$), 25.1 (SCH_2); HRMS (TOF-MS-ES$^+$) *m/z* calcd for $C_{13}H_{16}O_3S^{35}Cl$ [M+H]: 287.0509; found 287.0520.

Methyl 3-((3-(4-chlorophenyl)oxetan-3-yl)sulfonyl)propanoate (140c)

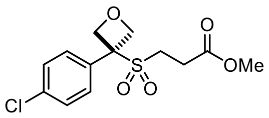

Washed *m*CPBA (421 mg, 2.49 mmol, 3.0 equiv) was added to a solution of oxetane sulfide **140b** (239 mg, 0.84 mmol, 1.0 equiv) in anhydrous CH_2Cl_2 (40 mL, 22 mM) in a 250 mL round bottom flask. The reaction mixture was stirred at 25 °C for 3 h and then aq. KOH (3 M, 20 mL) was added and the phases were separated. The aqueous portion was extracted with CH_2Cl_2 (3 × 30 mL). The organic extracts were combined and dried over Na_2SO_4, filtered, and concentrated *in vacuo* using a rotary evaporator to afford oxetane sulfone **140c** as a white solid (259 mg, 97%). R_f = 0.33 (50% EtOAc/ *n*-hexane); IR (film)/cm^{-1} 2953, 2887, 1738 (C=O st), 1494, 1438, 1365, 1311 (SO_2 st as), 1252, 1137 (SO_2 st sy), 1095, 1009, 931, 835, 716, 578, 521, 469; ^{1}H NMR (400 MHz, $CDCl_3$) δ 7.45 (d, *J* = 8.6 Hz, 2 H, 2 × Ar-CH), 7.26 (d, *J* = 8.6 Hz, 2 H, 2 × Ar-CH), 5.40 (d, *J* = 7.4 Hz, 2 H, C*H*HOC*H*H), 5.12 (d, *J* = 7.4 Hz, 2 H, CH*H*OCH*H*), 3.72 (s, 3 H, CO_2CH_3), 3.13 (t, *J* = 7.5 Hz, 2 H, SO_2CH_2), 2.77 (t, *J* = 7.5 Hz, 2 H, $CH_2CO_2CH_3$); ^{13}C NMR (101 MHz, $CDCl_3$) δ 170.8 (C_q=O), 135.8 (Ar-C_q-C_q), 132.3 (Ar-C_q-Cl), 129.6 (2 × Ar-CH), 129.4 (2 × Ar-CH), 76.2 (CH_2OCH_2), 68.4 (C_q), 52.5 (CO_2CH_3), 43.4 ($CH_2CO_2CH_3$), 25.8 (SO_2CH_2); HRMS (FTMS–APCI$^-$) *m/z* calcd for $C_{13}H_{15}O_5{}^{35}Cl_2S^-$ [M+Cl]$^-$: 353.0023; found 353.0020.

3-(4-Chlorophenyl)oxetane-3-sulfonyl fluoride (140)

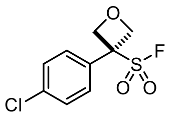

Prepared according to **oxetane sulfinate elimination procedure 3 (OSE-3)** from sulfone **140c** and submitting the sulfinate intermediate directly to the fluorination conditions. Sodium methoxide (5.4 M solution in MeOH, 0.14 mL, 0.75 mmol, 1.0 equiv) was added dropwise to a solution of oxetane sulfone **140c** (239 mg, 0.75 mmol, 1.0 equiv) in anhydrous THF (1.5 mL, 0.5 M) in a 10 mL round-bottom flask. A color change to deep yellow and precipitation of the oxetane sulfinate salt was observed immediately after addition of the base. The reaction mixture was stirred at 25 °C for 20 min, then concentrated *in vacuo* using a rotatory evaporator to afford oxetane sulfinate as a white solid, which was subjected directly to the next step without spectroscopical analysis. For the fluorination step, Selectfluor™ (399 mg, 1.05 mmol, 1.5 equiv) was added to anhydrous MeOH (3.75 mL, 0.2 M) at 0 °C in a 25 mL round bottom flask, then stirred for 5 min. The sulfinate salt was added portionwise and the sulfinate flask was rinsed with ice-cold Selectfluor™/methanol solution and transferred to the reaction vessel. The reaction mixture was stirred for at 0 °C for 1 h, then concentrated *in vacuo* using a rotatory evaporator. The residue was diluted with CH_2Cl_2 (10 mL) and filtered through a plug of Celite, eluting with further CH_2Cl_2 (20 mL). After concentrating *in vacuo* using a rotatory evaporator, purification by flash column chromatography through a very short column (80% CH_2Cl_2/pentane) afforded oxetane sulfonyl

fluoride **140** as a white solid (123 mg, 65%). R_f = 0.61 (100% CH_2Cl_2); mp = 105–107 °C; IR (film)/cm^{-1} 2963, 2893, 1598, 1494, 1400, 1214, 1168, 1096, 1003, 934, 833, 789, 755, 691, 591, 523, 464; ^{1}H NMR (400 MHz, $CDCl_3$) δ 7.48 (d, *J* = 8.5 Hz, 2 H, 2 × Ar-CH), 7.24 (d, *J* = 8.5 Hz, 2 H, 2 × Ar-CH), 5.44 (d, *J* = 7.6 Hz, 2 H, C*H*HOC*H*H), 5.23 (dd, *J* = 7.6 Hz, $J_{H–F}$ = 1.5 Hz, 2 H, CH*H*OCH*H*); ^{13}C NMR (101 MHz, $CDCl_3$) δ 136.6 (Ar-C_q-C_q), 130.2 (Ar-C_q-Cl), 129.6 (2 × Ar-CH), 129.5 (2 × Ar-CH), 76.9 (CH_2OCH_2), 67.9 (d, $^2J_{C-F}$ = 15.9 Hz, C_q); ^{19}F{^{1}H} NMR (377 MHz, $CDCl_3$) δ 35.4; HRMS (FTMS–APCI$^+$) *m/z* calcd for $C_9H_8O^{35}Cl^+$ $[M–SO_2F]^+$: 167.0258; found 167.0256.

Note on additional oxetane sulfonyl fluorides in the manuscript:

The following OSFs were prepared according to previously reported sequences: **3** (*para*-O^{i}Pr), **4** (3,4-dimethoxy), **6** (*meta*-methoxy, *para*-OTIPS), **8** (*meta*-^{i}Pr, *para*-OTIPS), **9** (3,4,5-trimethoxy) and **139** (phenyl).[6]

Azetidine Sulfonyl Fluorides (11–15, 147, 150, 152)

Azetidine Sulfonyl Fluoride 11

Benzyl 3-hydroxy-3-(4-methoxyphenyl)azetidine-1-carboxylate (11a)

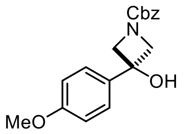

4-Methoxyphenylmagnesium bromide (0.5 M solution in THF, 100 mL, 50 mmol) was added dropwise using a canula to a solution of 1-Cbz-3-azetidinone (7.90 g, 38.5 mmol) in THF (50 mL) in a 500 mL round-bottom flask at 0 °C. The reaction mixture was stirred for 19 h at 25 °C and then cooled to 0 °C and quenched with sat. aq. NH_4Cl (300 mL). The aqueous portion was extracted with Et_2O (3 × 300 mL). The organic extracts were combined, dried over Na_2SO_4, filtered and concentrated *in vacuo* using a rotary evaporator. Purification by flash chromatography (70% Et_2O/*n*-hexane) afforded azetidinol **11a** as a white solid (9.39 g, 77%). R_f = 0.30 (30% EtOAc/*n*-hexane); ^{1}H NMR (400 MHz, $CDCl_3$) δ 7.41–7.30 (m, 7 H, 7 × Ar-CH), 6.93–6.89 (m, 2 H, 2 × Ar-CH), 5.11 (s, 2 H, $PhCH_2$), 4.32 (d, *J* = 9.2 Hz, 2 H, C*H*HNC*H*H), 4.23 (d, *J* = 9.2 Hz, 2 H, CH*H*NCH*H*), 3.81 (s, 3 H, OCH_3), 3.06 (s, 1 H, OH). The observed characterization data (R_f, ^{1}H) were consistent with that previously reported.[11]

Notes:

The rate of the dropwise addition of 4-methoxyphenylmagnesium bromide via the canula was controlled by employing a positive pressure on the bottle of 4-methoxyphenylmagnesium bromide with a balloon filled with argon.

Benzyl 3-((3-methoxy-3-oxopropyl)thio)-3-(4-methoxyphenyl)- azetidine-1-carboxylate (11b)

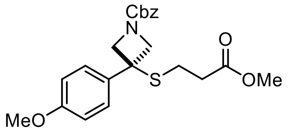

Azetidinol **11a** (3.12 g, 10.0 mmol) was added to a solution of $FeCl_3$ (135 mg, 0.75 mmol, 7.5 mol%) and methyl 3-mercaptoproprionate (2.18 mL, 20.0 mmol, 2.0 equiv) in anhydrous toluene (20 mL, 0.5 M) in a 50 mL round-bottom flask. The reaction mixture was stirred at 40 °C for 15 h, then quenched with sat. aq. $NaHCO_3$ (30 mL). The layers were separated, and the aqueous portion was extracted with CH_2Cl_2 (3 × 40 mL). The organic extracts were combined, dried over Na_2SO_4, filtered, and concentrated *in vacuo* using a rotary evaporator. Purification by flash column chromatography (40% Et_2O/pentane) afforded azetidine sulfide **11b** as a colorless oil (3.20 g, 77%). R_f = 0.34 (40% Et_2O/pentane); ^{1}H NMR (400 MHz, $CDCl_3$) δ 7.38−7.29 (m, 5 H, 5 × Ar−CH), 7.18−7.14 (m, 2 H, 2 × Ar−CH), 6.91−6.87 (m, 2 H, 2 × Ar−CH), 5.11 (s, 2 H, $PhCH_2$), 4.54 (d, *J* = 8.8 Hz, 2 H, C*H*HNC*H*H), 4.31 (d, *J* = 8.8 Hz, 2 H, CH*H*NCH*H*), 3.81 (s, 3 H, Ar-C_qOCH_3), 3.65 (s, 3 H, CO_2CH_3), 2.59 (t, *J* = 7.4 Hz, 2 H, SCH_2), 2.37 (t, *J* = 7.4 Hz, 2 H, C$H_2$$CO_2CH_3$). The observed characterization data (R_f, ^{1}H) were consistent with that previously reported.[14]

Benzyl 3-((3-methoxy-3-oxopropyl)sulfonyl)-3-(4-methoxyphen-yl) azetidine-1-carboxylate (11c)

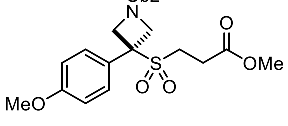

Washed *m*CPBA (3.99 g, 23.1 mmol, 3.0 equiv) was added to a solution of azetidine sulfide **11b** (3.20 mg, 7.7 mmol) in CH_2Cl_2 (400 mL, 0.02 M) in a 1 L round-bottom flask. The reaction mixture was stirred at 25 °C for 3 h and then aq. KOH (3 M, 100 mL) was added and the phases were separated. The aqueous portion was extracted with CH_2Cl_2 (4 × 50 mL). The organic extracts were combined, dried over Na_2SO_4, filtered, and concentrated *in vacuo* using a rotary evaporator to afford azetidine sulfone **11c** as a white solid (3.36 g, 97%). R_f = 0.13 (100% Et_2O); ^{1}H NMR (400 MHz, $CDCl_3$) δ 7.36−7.27 (m, 7 H, 7 × Ar−CH), 6.97 (d, *J* = 8.7 Hz, 2 H, 2 × Ar−CH), 5.12 (s, 2 H, $PhCH_2$), 4.86 (d, *J* = 9.4 Hz, 2 H, C*H*HNC*H*H), 4.48 (d, *J* = 9.4 Hz, 2 H, CH*H*NCH*H*), 3.84 (s, 3 H, Ar-C_qOCH_3), 3.69 (s, 3 H, CO_2CH_3), 3.05−3.01 (m, 2 H, SCH_2), 2.69−2.65 (m, 2 H, $CH_2CO_2CH_3$). The observed characterization data (R_f, ^{1}H) were consistent with that previously reported.[14]

Benzyl 3-(fluorosulfonyl)-3-(4-methoxyphenyl)azetidine-1-carboxylate (11)

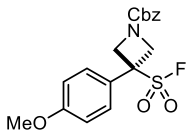

Prepared according to **azetidine sulfinate elimination procedure 1 (ASE-1)** from sulfone **11c** and submitting the sulfinate intermediate directly to the fluorination conditions. Sodium methoxide (5.4 M solution in MeOH, 0.73 mL, 3.90 mmol, 1.0 equiv) was added dropwise to a solution of oxetane sulfone **11c** (1.74 g, 3.90 mmol, 1.0 equiv) in anhydrous THF (9.8 mL, 0.5 M) in a 25 mL round-bottom flask. The azetidine sulfinate salt precipitated immediately after the addition of base to form a white solid. The reaction mixture was stirred at 25 °C for 20 min, then concentrated *in vacuo* using a rotatory evaporator to afford the azetidine sulfinate salt as a white solid, which was subjected directly to the next step without spectroscopical analysis. For the fluorination step, pre-cooled (0 °C) anhydrous MeOH (23.4 mL, 0.25 M) and Selectfluor^{TM} (2.09 g, 5.8 mmol, 1.5 equiv) were added sequentially to the azetidine sulfinate salt at 0 °C, then stirred at 0 °C for 1 h. The reaction mixture was concentrated *in vacuo* using a rotary evaporator. The resulting residue was diluted with Et_2O (120 mL) and filtered through a plug of Celite, eluting with further Et_2O (40 mL). After concentration *in vacuo* using a rotary evaporator, purification by automated flash column chromatography (45% Et_2O/pentane, see conditions below) afforded azetidine sulfonyl fluoride **11** as a white solid (1.05 g, 71%). R_f = 0.23 (45% Et_2O/*n*-hexane); mp = 106–109 °C; IR (film)/cm^{-1} 1717 (C=O st), 1610, 1515, 1454, 1400 (SO_2 st as), 1354, 1258, 1212 (SO_2 st sy), 1184, 1137, 1032, 836, 788; ^{1}H NMR (400 MHz, $CDCl_3$) δ 7.43–7.33 (m, 5 H, 5 × Ar-CH), 7.30–7.26 (m, 2 H, 2 × Ar-CH), 7.03–6.92 (m, 2 H, 2 × Ar-CH), 5.15 (s, 2 H, $PhCH_2$), 4.94–4.92 (d, *J* = 9.9 Hz, 2 H, C*H*HNC*H*H), 4.66–4.64 (d, *J* = 9.9 Hz, 2 H, CH*H*NCH*H*), 3.87 (s, 3 H, OCH_3); ^{13}C (101 MHz, $CDCl_3$) δ 161.1 (Ar-C_q-OCH_3), 155.7 (C_q=O), 135.8 (Ar-C_q-CH_2), 129.9 (2 × Ar-CH), 128.6 (2 × Ar-CH), 128.5 (Ar-CH), 128.2 (2 × Ar-CH), 122.9 (Ar-C_q-C_q), 114.7 (2 × Ar-CH) 67.5 ($PhCH_2$), 63.1 (d, $^2J_{C\text{-}F}$ = 16.2 Hz, C_q), 57.7 and 57.2 (CH_2NCH_2), 55.5 (OCH_3); ^{19}F{^{1}H} NMR (377 MHz, $CDCl_3$) δ 33.5; HRMS (TOF-MS-ES$^+$) *m/z* calcd for $C_{18}H_{19}O_5NFS^+$ $[M+H]^+$: 380.0968, found 380.0974, HRMS (TOF-MS-ES$^+$) *m/z* calcd for $C_{18}H_{18}NO_3^+$ $[M–SO_2F]^+$: 296.1281, found: 296.1275.

Automated Column Conditions: Run on a Biotage® Selekt system. Column type: Biotage® Sfär 50 g. Flow rate: 120 mL/min. Sample mass: 800 mg. Solvent A: pentane, Solvent B: Et_2O. UV wavelength detection: l 200–400 nm. See trace below. From the left: peak 1 (blue, red, green): azetidine sulfonyl fluoride **11**, peak 2 (red): azetidine sulfone **11c**.

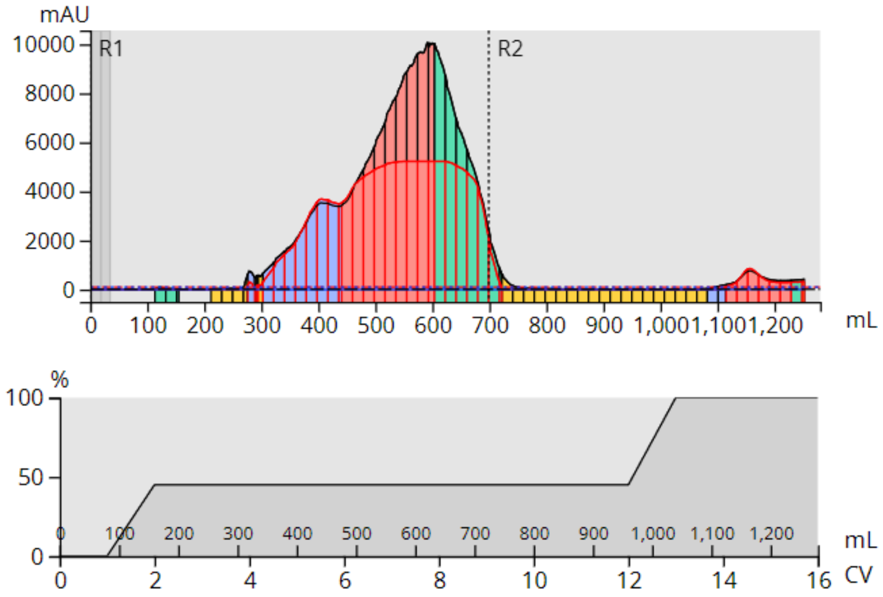

Notes:

It is important to use Et_2O when filtering the crude ASF residue through Celite. *Unknown impurities are commonly observed when this is performed with CH_2Cl_2.*

***11** was further characterized by X-ray crystallography (see Fig. **S7**). Crystals suitable for X-ray analysis were grown by slow evaporation from acetone.*

Azetidine Sulfonyl Fluoride 12

Benzyl 3-hydroxy-3-(4-((triisopropylsilyl)oxy)phenyl)azetidine-1-carboxylate (12a)

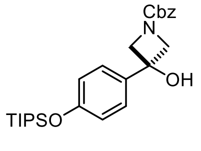

^{i}PrMgCl•LiCl (1.29 M in THF, 47 mL, 60.0 mmol, 1.2 equiv) was added dropwise over 5 min to a solution of (4-iodophenoxy)triisopropylsilane (23.6 g, 65.0 mmol, 1.3 equiv) in THF (170 mL) in a 500 mL round-bottom flask at 0 °C. The reaction mixture was allowed to warm to 25 °C and stirred for 3 h. A solution of 1-Cbz-3-azetidinone (9.91 g, 50 mmol, 1.0 equiv) in THF (24 mL) was added dropwise to the reaction mixture at 0 °C. The reaction mixture was allowed to warm to 25 °C, stirred for 24 h and then cooled to 0 °C and quenched with sat. aq. NH_4Cl (100 mL). The aqueous portion was extracted with Et_2O (3 × 40 mL). The organic extracts were combined, dried over Na_2SO_4, filtered and concentrated under reduced pressure. Purification by flash chromatography (40% Et_2O/pentane) afforded azetidinol **12a** as a white solid (17.7 g, 80%). R_f = 0.37 (30% EtOAc/*n*-hexane); ^{1}H NMR (400 MHz, $CDCl_3$) δ 7.37–7.30 (m, 7 H, 7 × Ar-CH), 6.91–6.87 (m, 2 H, 2 × Ar-CH), 5.12 (s, 2 H, $PhCH_2$), 4.33 (d, *J* = 9.3 Hz, 2 H, C*H*HNC*H*H), 4.23 (d, *J* = 9.3 Hz, 2 H, CH*H*NCH*H*), 3.00 (s, 1 H, OH), 1.31–1.22 (m, 3 H, 3 × SiCH), 1.11 (d, *J* = 7.2 Hz, 18 H, 6 × SiCHC*H*$_3$). The observed characterization data (R_f, ^{1}H) were consistent with that previously reported.[11]

Benzyl 3-((3-methoxy-3-oxopropyl)thio)-3-(4-((triisopropylsilyl)oxy)phenyl)azetidine-1-carboxylate (12b)

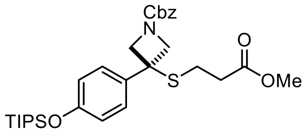

Azetidinol **12a** (17.7 g, 38.9 mmol) was added to a solution of $FeCl_3$ (1.02 g, 2.9 mmol, 7.5 mol%) and methyl 3-mercaptoproprionate (8.86 mL, 77.8 mmol, 2.0 equiv) in anhydrous toluene (78 mL, 0.5 M) in a 250 mL round-bottom flask. The reaction mixture was stirred at 40 °C for 15 h, then quenched with sat. aq. $NaHCO_3$ (100 mL). The layers were separated, and the aqueous portion was extracted with CH_2Cl_2 (3 × 100 mL). The organic extracts were combined, dried over Na_2SO_4, filtered, and concentrated *in vacuo* using a rotary evaporator. Purification by flash column chromatography (20–40% EtOAc/pentane) afforded azetidine sulfide **12b** as a colorless oil which solidified into a white solid in the freezer at –20 °C (14.9 g, 69%). R_f = 0.38 (40% Et_2O/pentane); mp = 90–92 °C; IR (film)/cm^{-1} 2945, 2864, 1710 (C=O st), 1511, 1403, 1355, 1247, 1115, 916, 748, 688; ^{1}H NMR ($CDCl_3$, 400 MHz) δ 7.38 (m, 5 H, 5 × Ar-CH), 7.14–7.06 (m, 2 H, 2 × Ar-CH), 6.92–6.84 (m, 2 H, 2 × Ar-CH), 5.12 (s, 2 H, $PhCH_2$), 4.56 (d, *J* = 8.8 Hz, 2 H, C*H*HNC*H*H), 4.31 (d, *J* = 8.8 Hz, 2 H, CH*H*NCH*H*), 3.66 (s, 3 H, CO_2CH_3), 2.60 (t, *J* = 7.4 Hz, 2 H, SCH_2), 2.34 (t, *J* = 7.4 Hz, 2 H, C*H*$_2$CO$_2$CH$_3$), 1.34–1.20 (m, 3 H, 3 × SiCH), 1.12 (d, *J* = 7.3 Hz, 18 H, 6 × SiCHC*H*$_3$); ^{13}C NMR (101 MHz, $CDCl_3$) δ 172.0 (*C*O$_2$CH$_3$), 156.2 (Ar-C_q-OTIPS), 155.3 (N*C*$_q$-O$_2$CH$_2$), 136.4 (Ar-*C*$_q$-CH$_2$), 134.5 (Ar-*C*$_q$-C$_q$), 128.5 (2 × Ar-CH), 128.1 (Ar-CH), 128.0 (2 × Ar-CH), 127.5 (2 × Ar-CH), 120.0 (2 × Ar-CH), 66.9 ($PhCH_2$), 62.5 (CH_2NCH_2), 51.8 (CO$_2$*C*H$_3$), 47.3 (C_q), 33.8 (*C*H$_2$CO$_2$CH$_3$), 25.2 (SCH_2), 17.9 (6 × SiCH*C*H$_3$), 12.6 (3 × SiCH); HRMS (TOF-MS-ES$^+$) *m/z* calcd for $C_{30}H_{44}NO_5SSi^+$ [M+H]$^+$: 558.2709; found 558.2728.

Benzyl 3-((3-methoxy-3-oxopropyl)sulfonyl)-3-(4-((triisopropylsilyl)oxy)phenyl)azetidine-1-carboxylate (12c)

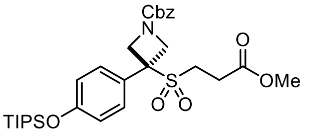

Washed *m*CPBA (1.44 g, 8.32 mmol, 3.0 equiv) was added to a solution of azetidine sulfide **12b** (1.55 gg, 2.77 mmol) in CH_2Cl_2 (140 mL, 0.02 M) in a 500 mL round-bottom flask. The reaction mixture was stirred at 25 °C for 3 h and then aq. KOH (3 M, 60 mL) was added and the phases were separated. The aqueous portion was extracted with CH_2Cl_2 (4 × 60 mL). The organic extracts were combined, dried over Na_2SO_4, filtered, and concentrated *in vacuo* using a rotary evaporator to afford azetidine sulfone **12c** as a white solid (1.63 g, 99%). R_f = 0.54 (40% EtOAc/pentane); mp = 105–109 °C; IR (film)/cm^{-1} 2946, 2866, 1711 (C=O st), 1512, 1415, 1355, 1309, 1271 (SO_2 st as), 1129, 909, 730, 692; ^{1}H NMR (400 MHz, $CDCl_3$) δ 7.38–7.33 (m, 5 H, 5 × Ar-CH), 7.21 (d, *J* = 8.6 Hz, 2 H, 2 × Ar-CH), 6.93 (d, *J* = 8.6 Hz, 2 H, 2 × Ar-CH), 5.11 (s, 2 H, $PhCH_2$), 4.85 (m, 2 H, C*H*HNC*H*H), 4.47 (d, *J* = 9.5 Hz, 2 H, CH*H*NCH*H*), 3.69 (s, 3 H, CO_2CH_3), 3.02 (t, *J* = 7.8 Hz, 2 H, SO_2CH_2), 2.62 (t, *J* = 7.8 Hz, 2 H, $CH_2CO_2CH_3$), 1.35–1.20 (m, 3 H, 3 × SiCH), 1.11 (d, *J* = 7.3 Hz, 18 H, 6 × $SiCHCH_3$); ^{13}C NMR (101 MHz, $CDCl_3$) δ 170.3 (CO_2CH_3), 156.9 (Ar-C_q-OTIPS), 155.4 (NC_q-O_2CH_2), 135.7 (Ar-C_q-CH_2), 129.2 (2 × Ar-CH), 128.1 (2 × Ar-CH), 127.9 (Ar-CH), 127.7 (2 × Ar-CH), 125.1 (Ar-C_q-C_q), 120.1 (2 × Ar-CH), 66.9 ($PhCH_2$), 62.9 (C_q), 56.0 (CH_2NCH_2), 52.0 (CO_2CH_3), 42.7 ($CH_2CO_2CH_3$), 25.5 (SO_2CH_2), 17.5 (6 × $SiCHCH_3$), 12.2 (3 × SiCH); HRMS (TOF-MS-ES$^+$) *m/z* calcd for $C_{30}H_{44}NO_7SSi^+$ $[M+H]^+$: 590.2602; found: 590.2612.

Benzyl 3-(fluorosulfonyl)-3-(4-((triisopropylsilyl)oxy)phenyl)azetidine-1-carboxylate (12)

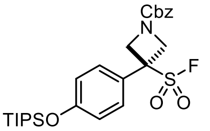

Prepared according to **azetidine sulfinate elimination procedure 2 (ASE-2)** from sulfone **12c** and submitting the sulfinate intermediate directly to the fluorination conditions. Azetidine sulfone **12c** (290 mg, 0.5 mmol, 1.0 equiv) was added to a solution of NaH (60% in mineral oil, 21.3 mg, 0.53 mmol, 1.05 equiv) in THF (2.5 mL, 0.2 M) at 0 °C. The reaction mixture was stirred at 25 °C for 1 h then quenched with anhydrous MeOH (1.0 mL) and concentrated under reduced pressure at 20 °C to afford sulfinate salt as a yellow residue, which was subjected directly to the next step without spectroscopical analysis. For the fluorination step, pre-cooled (0 °C) anhydrous MeOH (3.0 mL, 0.25 M) and Selectfluor™ (270 mg, 0.75 mmol, 1.5 equiv) were added sequentially to the azetidine sulfinate salt at 0 °C, then stirred at 0 °C for 1 h. The reaction mixture was concentrated *in vacuo* using a rotary evaporator. The resulting residue was diluted with Et_2O (20 mL) and filtered through a plug of Celite, eluting with further Et_2O (20 mL). After concentration *in vacuo* using a rotary evaporator, purification by automated flash column chromatography (20% Et_2O/pentane, see conditions below) afforded azetidine sulfonyl fluoride **12** as a viscous colorless oil (160 mg, 64%). R_f = 0.57 (20% Et_2O/pentane); IR (film)/cm^{-1} 2946, 2867, 1716, 1605, 1512, 1460, 1403, 1354 (SO_2 st as), 1273, 1212 (SO_2 st sy), 1138, 909, 732, 692; ^{1}H NMR (400 MHz, $CDCl_3$) δ 7.42–7.30 (m, 5 H, 5 × Ar-CH), 7.19 (d, *J* = 8.2 Hz, 2 H, 2 × Ar-CH), 6.98–6.90 (m, 2 H, 2 × Ar-CH), 5.13 (s, 2 H, $PhCH_2$), 4.90 (d, *J* = 9.8 Hz, 2 H, C*H*HNC*H*H), 4.63 (d, *J* = 9.8 Hz, 2 H, CH*H*NCH*H*), 1.36–1.20 (m, 3 H, 3 × SiCH), 1.11 (d, *J* = 7.4 Hz, 18 H, 6 × $SiCHCH_3$); ^{13}C NMR (101 MHz, $CDCl_3$) δ 157.8 (C_q=O), 155.6 (Ar-C_q-OTIPS), 135.8 (Ar-C_q-CH_2), 129.8 (2 × Ar-CH), 128.6 (2 × Ar-CH), 128.4 (Ar-CH), 128.2 (2 × Ar-CH), 123.1 (Ar-C_q-C_q), 120.5 (2 × Ar-CH), 67.5 ($PhCH_2$), 63.2 (d, $^2J_{C\text{-}F}$= 16.0 Hz, C_q), 57.3 (CH_2NCH_2), 17.8 (3 × SiCH), 12.6 (6 × $SiCHCH_3$); ^{19}F{^{1}H} NMR (377 MHz, $CDCl_3$) δ 33.5; HRMS (TOF-MS-ES$^+$) *m/z* calcd for $C_{26}H_{36}FNO_5SSi^+$ $[M+H]^+$: 522.2140; found: 522.2137.

Automated Column Conditions: Run on a Biotage® Selekt system. Column type: Biotage® Sfär HC 10 g. Flow rate: 40 mL/min. Sample mass: 150 mg. Solvent A: pentane, Solvent B: Et_2O. UV wavelength detection: l 200–400 nm. See trace below. From the left: peak 1 (green): azetidine sulfonyl fluoride **12**, peak 2, 3 (yellow, blue): azetidine fluoride, peak 4 (red): azetidine sulfone **12c**

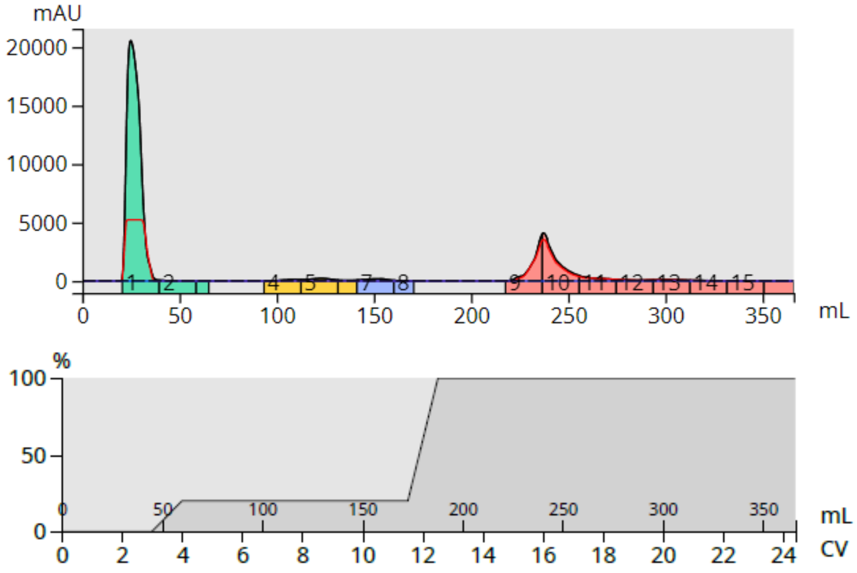

Notes:

It is important to use Et_2O when filtering the crude ASF residue through Celite*. Unknown impurities are commonly observed when this is performed with CH_2Cl_2.*

Azetidine Sulfonyl Fluoride 13

Benzyl 3-hydroxy-3-(2-methoxyphenyl)azetidine-1-carboxylate (13a)

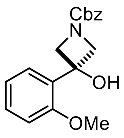

*i*PrMgCl•LiCl (1.14 M in THF, 10.5 mL, 12.0 mmol, 1.2 equiv) was added dropwise over 5 min to a solution of 1-iodo-2-methoxybenzene (1.70 mL, 13.1 mmol, 1.3 equiv) in THF (30 mL) in a 100 mL round-bottom flask at 0 °C. The reaction mixture was allowed to warm to 25 °C and stirred for 3 h. A solution of 1-Cbz-3-azetidinone (2.08 g, 10.2 mmol, 1.0 equiv) in THF (10 mL) was added dropwise to the reaction mixture at 0 °C. The reaction mixture was allowed to warm to 25 °C, stirred for 24 h and then cooled to 0 °C and quenched with sat. aq. NH_4Cl (50 mL). The aqueous portion was extracted with Et_2O (3 × 50 mL). The organic extracts were combined, dried over Na_2SO_4, filtered and concentrated under reduced pressure. Purification by recrystallization in a solvent/anti-solvent (EtOAc/*n*-hexane) afforded azetidinol **13a** as white crystalline solid (2.71 g, 87%). R_f = 0.20 (70% Et_2O/pentane); ^1H NMR (400 MHz, $CDCl_3$) δ 7.37–7.27 (m, 7 H, 7 × Ar-CH), 7.00 (td, *J* = 7.5, 1.1 Hz, 1 H, Ar-CH), 6.94 (dd, *J* = 8.2, 1.1 Hz, 1 H, Ar-CH), 5.12 (s, 2 H, $PhCH_2$), 4.46 (d, *J* = 9.5 Hz, 2 H, C*H*HNC*H*H), 4.24 (d, *J* = 9.5 Hz, 2 H, CH*H*NCH*H*), 3.88 (s, 3 H, OCH_3), 3.40 (s, 1 H, OH). The observed characterization data (R_f, ^1H) were consistent with that previously reported.[11]

Benzyl 3-((3-methoxy-3-oxopropyl)thio)-3-(2-methoxyphenyl)azetidine-1-carboxylate (13b)

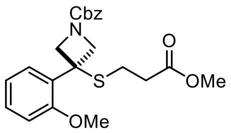

Azetidinol **13a** (2.53 g, 8.08 mmol) was added to a solution of $FeCl_3$ (126 mg, 0.61 mmol, 7.5 mol%) and methyl 3-mercaptoproprionate (1.77 mL, 16.2 mmol, 2.0 equiv) in anhydrous toluene (16 mL, 0.5 M) in a 50 mL round-bottom flask. The reaction mixture was stirred at 40 °C for 15 h, then quenched with sat. aq. $NaHCO_3$ (20 mL). The layers were separated, and the aqueous portion was extracted with CH_2Cl_2 (3 × 50 mL). The organic extracts were combined, dried over Na_2SO_4, filtered, and concentrated *in vacuo* using a rotary evaporator. Purification by flash column chromatography (20% EtOAc/pentane) afforded azetidine sulfide **13b** as a yellow oil (2.30 g, 68%). R_f = 0.23 (40% Et_2O/pentane); ^1H NMR (400 MHz, $CDCl_3$) δ 7.37−7.26 (m, 6 H, 6 × Ar−CH), 7.00−6.93 (m, 2 H, 2 × Ar−CH), 6.90 (d, *J* = 8.2 Hz, 1 H, Ar−CH), 5.11 (s, 2 H, $PhCH_2$), 4.57 (d, *J* = 9.3 Hz, 2 H, C*H*HNC*H*H), 4.28 (d, *J* = 9.3 Hz,

2 H, CH*H*NCH*H*), 3.84 (s, 3 H, OCH_3), 3.64 (s, 3 H, CO_2CH_3), 2.66 (t, *J* = 7.6 Hz, 2 H, SCH_2), 2.35 (t, *J* = 7.6 Hz, 2 H, $CH_2CO_2CH_3$). The observed characterization data (R_f, ^1H) were consistent with that previously reported.[14]

Benzyl 3-((3-methoxy-3-oxopropyl)sulfonyl)-3-(2-methoxyphenyl)azetidine-1-carboxylate (13c)

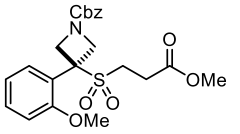

*m*CPBA (≤77%, 3.30 g, 14.5 mmol, 3.0 equiv) was added to a solution of azetidine sulfide **13b** (2.01 g, 4.84 mmol, 1.0 equiv) in CH_2Cl_2 (230 mL, 0.02 M) in a 500 mL round-bottom flask. The reaction mixture was stirred at 25 °C for 3 h then aq. KOH (3 M, 60 mL) was added and the phases were separated. The aqueous portion was extracted with CH_2Cl_2 (4 × 30 mL). The organic extracts were combined, dried over Na_2SO_4, filtered and concentrated *in vacuo* using a rotatory evaporator to afford sulfone **13c** as an amorphous white solid (2.11 g, 97%). R_f = 0.33 (50% EtOAc/pentane); IR (film)/cm^{-1} 2952, 1735 (C=O st), 1705 (C=O st), 1411, 1355, 1306 (SO_2 st as), 1122 (SO_2 st sy), 757; ^1H NMR (400 MHz, $CDCl_3$) δ 7.45–7.38 (m, 1 H, Ar−CH), 7.38–7.28 (m, 5 H, 5 × Ar-CH), 7.17 (dd, *J* = 7.8, 1.7 Hz, 1 H, Ar-CH), 7.06 (t, *J* = 7.5 Hz, 1 H, Ar-CH), 6.96 (d, *J* = 8.4 Hz, 1 H, Ar-CH), 5.10 (s, 2 H, $PhCH_2$), 4.83 (br s, 2 H, C*H*HNC*H*H), 4.55 (br s, 2 H, CH*H*NCH*H*), 3.88 (s, 3 H, OCH_3), 3.68 (s, 3 H, CO_2CH_3), 3.15 (br t, 2 H, SO_2CH_2), 2.70 (t, *J* = 8.0 Hz, 2 H, $CH_2CO_2CH_3$); ^{13}C NMR (101 MHz, $CDCl_3$) δ 170.9 (CO_2CH_3), 157.1 (Ar-C_q-OCH_3), 155.9 (NC_q-O_2CH_2), 136.2 (Ar-C_q-CH_2), 131.4 (Ar-CH), 131.1 (Ar-CH), 128.5 (2 × Ar-CH), 128.2 (Ar-CH), 128.1 (2 × Ar-CH), 121.7 (Ar–CH), 121.4 (Ar-C_q-C_q), 111.5 (Ar-CH), 67.1 ($PhCH_2$), 63.1 (C_q), 56.3 and 55.8 (CH_2NCH_2), 55.5 (OCH_3), 52.4 (CO_2CH_3), 43.7 ($CH_2CO_2CH_3$), 25.9 (SO_2CH_2); HRMS (TOF-MS-ES$^+$) *m/z* calcd for $C_{22}H_{26}NO_7S^+$ $[M+H]^+$: 448.1430, found: 448.1427.

Benzyl 3-(fluorosulfonyl)-3-(2-methoxyphenyl)azetidine-1-carboxylate (13)

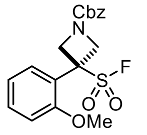

Prepared according to **azetidine sulfinate elimination procedure 2 (ASE-2)** from sulfone **13c** and submitting the sulfinate intermediate directly to the fluorination conditions. A solution of azetidine sulfone **13c** (2.1 g, 4.71 mmol, 1.0 equiv) in anhydrous THF (8.6 mL) was added dropwise to a solution of NaH (60% in mineral oil, 210 mg, 4.71 mmol, 1.0 equiv) in anhydrous THF (15 mL) at 0 °C. The reaction mixture was stirred at 25 °C for 1 h then quenched with anhydrous MeOH (0.75 mL) and concentrated under reduced pressure at 20 °C to afford an orange oil, which was subjected directly to the next step without spectroscopical analysis. For the fluorination step, pre-cooled (0 °C) anhydrous MeOH (32 mL, 0.15 M) and Selectfluor™ (2.56 g, 7.23 mmol, 1.5 equiv) were added sequentially to the azetidine sulfinate salt residue at 0 °C, then stirred at 0 °C for 1 h. The reaction mixture was concentrated *in vacuo* using a rotary evaporator. The resulting residue was diluted with Et_2O (25 mL) and filtered through a plug of Celite, eluting with further Et_2O (40 mL). After concentration *in vacuo* using a rotary evaporator, purification by automated flash column chromatography (20% Et_2O/pentane, see conditions below) afforded azetidine sulfonyl fluoride **13** as a colorless oil (749 mg, 42%). R_f = 0.36 (50% Et_2O/pentane); IR film/cm^{-1} 2956, 1709 (C=O st), 1601, 1497, 1395, 1353 (SO_2 st as), 1210 (SO_2 st sy), 1135, 1025, 755; ^1H NMR (400 MHz, $CDCl_3$) δ 7.44 (m, 1 H, Ar-CH), 7.39–7.30 (m, 5 H, 5 × Ar-CH), 7.15 (d, *J* = 7.5 Hz, 1 H, Ar-CH), 7.04 (t, *J* = 7.5 Hz, 1 H, Ar-CH), 6.97 (d, *J* = 8.4 Hz, 1 H, Ar-CH), 5.12 (s, 2 H, $PhCH_2$), 4.86 (d, *J* = 10.4, Hz, 2 H, C*H*HNC*H*H), 4.69 (d, *J* = 10.4 Hz, 2 H, CH*H*NCH*H*), 3.86 (s, 3 H, OCH_3); ^{13}C NMR (101 MHz, $CDCl_3$) δ 158.3 (Ar-C_q-OCH_3), 156.0 (C_q=O), 136.1 (Ar-C_q-CH_2), 132.3 (Ar-CH), 130.1 (Ar-CH), 128.7 (2 × Ar-CH), 128.5 (Ar-CH), 128.4 (2 × Ar-CH), 121.3 (Ar-CH), 119.3 (Ar-C_q-CH_2), 112.2 (Ar-CH), 67.5 ($PhCH_2$), 63.2 (d, $^2J_{C-F}$ = 16.8 Hz), 57.5 and 57.0 (CH_2NCH_2), 55.9 (OCH_3); $^{19}F\{^1H\}$ NMR (377 MHz, $CDCl_3$) δ 35.5; HRMS (TOF-MS-ES$^+$) *m/z* calcd for $C_{18}H_{19}NO_5FS^+$ $[M+H]^+$: 380.0968, found: 380.0960.

Automated Column Conditions: Run on a Biotage® Selekt system. Column type: Biotage® Sfär HC 50 g. Flow rate: 120 mL/min. Sample mass: 1500 mg. Solvent A: pentane, Solvent B: Et_2O. UV

wavelength detection: λ 200–400 nm. See trace below. From the left: peak 1 (blue): azetidine fluoride, peak 2 (blue, red): azetidine sulfonyl fluoride **13**, peak 3 (green, yellow, red): mixture of azetidinol **13a** and sulfone **13c**.

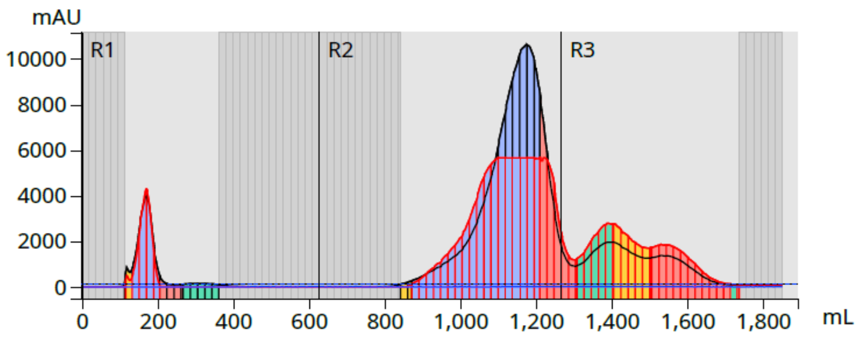

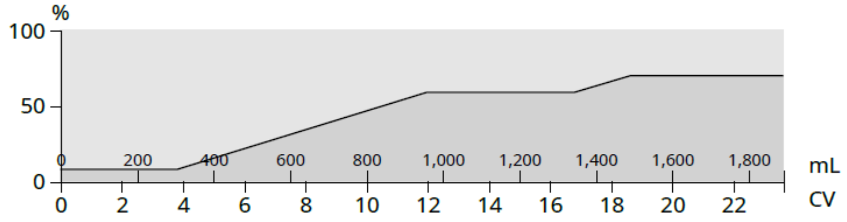

Azetidine Sulfonyl Fluoride 14

Benzyl 3-(benzo[*d*][1,3]dioxol-5-yl)-3-hydroxyazetidine-1-carboxylate (14a)

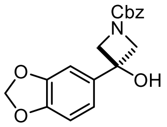

*i*PrMgCl•LiCl (1.20 M in THF, 20.0 mL, 24.0 mmol, 1.2 equiv) was added dropwise over 5 min to a solution of 5-iodobenzo[*d*][1,3]dioxole (3.38 mL, 26.0 mmol, 1.3 equiv) in THF (40 mL) in a 100 mL round-bottom flask at 0 °C. The reaction mixture was allowed to warm to 25 °C and stirred for 5 h. A solution of 1-Cbz-3-azetidinone (4.10 g, 20.0 mmol, 1.0 equiv) in THF (27 mL) was added dropwise to the reaction mixture at 0 °C. The reaction mixture was allowed to warm to 25 °C, stirred for 24 h and then cooled to 0 °C and quenched with sat. aq. NH_4Cl (100 mL). The aqueous portion was extracted with Et_2O (3 × 100 mL). The organic extracts were combined, dried over Na_2SO_4, filtered and concentrated under reduced pressure. Purification by flash column chromatography (25% EtOAc/pentane) afforded azetidinol **14a** as white solid (5.50 g, 84%). R_f = 0.20 (30% EtOAc/*n*-hexane); ^1H NMR (400 MHz, $CDCl_3$) δ 7.36–7.30 (m, 5 H, 5 × Ar-CH), 6.96 (d, *J* = 1.8 Hz, 1 H, Ar-CH), 6.93 (dd, *J* = 8.0, 1.8 Hz, 1 H, Ar-CH), 6.80 (d, *J* = 8.0 Hz, 1 H, Ar-CH), 5.97 (s, 2 H, OCH_2O), 5.12 (s, 2 H, $PhCH_2$), 4.30 (d, *J* = 9.3 Hz, 2 H, C*H*HNC*H*H), 4.21 (d, *J* = 9.3 Hz, 2 H, CH*H*NCH*H*), 2.33 (s, 1 H, OH). The observed characterization data (R_f, ^1H) were consistent with that previously reported.[11]

Benzyl 3-(benzo[*d*][1,3]dioxol-5-yl)-3-((3-methoxy-3-oxopropyl)thio)azetidine-1-carboxylate (14b)

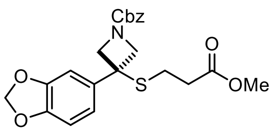

Azetidinol **14a** (2.72 g, 8.3 mmol) was added to a solution of $FeCl_3$ (101 mg, 0.62 mmol, 7.5 mol%) and methyl 3-mercaptoproprionate (1.84 mL, 16.6 mmol, 2.0 equiv) in anhydrous toluene (16.6 mL, 0.5 M) in a 50 mL round-bottom flask. The reaction mixture was stirred at 40 °C for 15 h, then quenched with sat. aq. $NaHCO_3$ (30 mL). The layers were separated, and the aqueous portion was extracted

with CH_2Cl_2 (3 × 50 mL). The organic extracts were combined, dried over Na_2SO_4, filtered, and concentrated *in vacuo* using a rotary evaporator. Purification by flash column chromatography (40% Et_2O/pentane) afforded azetidine sulfide **14b** as a colorless oil (3.02 g, 85%). R_f = 0.32 (30% EtOAc/pentane); IR (film)/cm^{-1} 2950, 2881, 1735 (C=O st), 1702 (C=O st), 1502, 1410, 1351, 1245, 1221, 1124, 1038, 736; ^{1}H NMR (400 MHz, $CDCl_3$) δ 7.41–7.29 (m, 5 H, 5 × Ar-CH), 6.77 (d, *J* = 8.0 Hz, 1 H, Ar-CH), 6.71 (s, 1 H. Ar-CH), 6.67 (dd, *J* = 8.0, 1.7 Hz, 1 H, Ar-CH), 5.97 (s, 2 H, CH_2OCH_2), 5.10 (s, 2 H, $PhCH_2$), 4.49 (d, *J* = 8.8 Hz, 2 H, C*H*HNC*H*H), 4.27 (d, *J* = 8.8 Hz, 2 H, CH*H*NCH*H*), 3.66 (s, 3 H, CO_2CH_3), 2.59 (t, *J* = 7.4 Hz, 2 H, SCH_2), 2.39 (t, *J* = 7.4 Hz, 2 H, $CH_2CO_2CH_3$); ^{13}C NMR (101 MHz, $CDCl_3$) δ 172.0 (CO_2CH_3), 156.2 (NC_q-O_2CH_2), 148.1 (Ar-C_q-O), 146.9 (Ar-C_q-O), 136.4 (Ar-C_q-C_q), 136.1 (Ar-C_q-CH_2), 128.5 (2 × Ar-CH), 128.2 (Ar-CH), 128.1 (2 × Ar-CH), 119.7 (Ar-CH), 107.9 (Ar-CH), 107.1 (Ar-CH), 101.4 (OCH_2O), 67.0 ($PhCH_2$), 62.7 and 62.1 (CH_2NCH_2), 51.9 (CO_2CH_3), 47.8 (C_q), 33.7 ($CH_2CO_2CH_3$), 25.3 (SCH_2); HRMS (TOF-MS-ES$^+$) *m/z* calcd for $C_{22}H_{24}NO_6S^+$ $[M+H]^+$: 430.1324, found: 430.1320.

Benzyl 3-(benzo[*d*][1,3]dioxol-5-yl)-3-((3-methoxy-3-oxopropyl)sulfonyl)azetidine-1-carboxylate (14c)

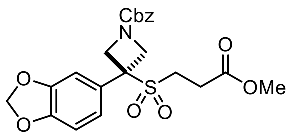

*m*CPBA (≤77%, 4.71 g, 21.0 mmol, 3.0 equiv) was added to a solution of azetidine sulfide **14b** (3.02 g, 7.0 mmol, 1.0 equiv) in CH_2Cl_2 (140 mL, 0.05 M) in a 500 mL round-bottom flask. The reaction mixture was stirred at 25 °C for 3 h then aq. KOH (3 M, 60 mL) was added and the phases were separated. The aqueous portion was extracted with CH_2Cl_2 (4 × 60 mL). The organic extracts were combined, dried over Na_2SO_4, filtered and concentrated *in vacuo* using a rotatory evaporator to afford sulfone **14c** a white solid (2.89 g, 90%). R_f = 0.42 (50% EtOAc/pentane); mp = 132–134 °C. IR (film)/cm^{-1} 2952, 1709 (C=O st), 1491, 1441, 1416, 1355, 1310, 1230, 1128, 1038, 932, 699; ^{1}H NMR (400 MHz, $CDCl_3$) δ 7.40–7.28 (m, 5 H, 5 × Ar-CH), 6.85 (d, *J* = 8.6 Hz, 1 H, Ar-CH), 6.82–6.78 (m, 2 H, 2 × Ar-CH), 6.02 (s, 2 H, CH_2OCH_2), 5.11 (s, 2 H, $PhCH_2$), 4.82 (d, *J* = 9.4 Hz, 2 H, C*H*HNC*H*H), 4.44 (d, *J* = 9.5 Hz, 2 H, CH*H*NCH*H*), 3.70 (s, 3 H, CO_2CH_3), 3.09–3.01 (m, 2 H, SO_2CH_2), 2.70 (t, *J* = 7.7 Hz, 2 H, $CH_2CO_2CH_3$); ^{13}C NMR (101 MHz, $CDCl_3$) δ 170.7 (CO_2CH_3), 155.8 (NC_q-O_2CH_2), 148.8 (Ar-C_q-O), 148.4 (Ar-C_q-O), 136.0 (Ar-C_q-C_q), 128.6 (2 × Ar-CH), 128.3 (Ar-CH), 128.1 (2 × Ar-CH), 127.0 (Ar-C_q-CH_2), 122.3 (Ar-CH), 108.8 (Ar-CH), 108.6 (Ar-CH), 101.8 (CH_2OCH_2), 67.3 ($PhCH_2$), 63.7 (C_q), 56.4 and 55.8 (CH_2NCH_2), 52.5 (CO_2CH_3), 42.9 ($CH_2CO_2CH_3$), 25.8 (SO_2CH_2); HRMS (TOF-MS-ES$^+$) *m/z* calcd for $C_{22}H_{24}NO_8S^+$ $[M+H]^+$: 462.1223, found: 462.1237.

Benzyl 3-(benzo[*d*][1,3]dioxol-5-yl)-3-(fluorosulfonyl)azetidine-1-carboxylate (14)

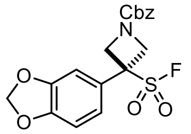

Prepared according to **azetidine sulfinate elimination procedure 2 (ASE-2)** from sulfone **14c** and submitting the sulfinate intermediate directly to the fluorination conditions. Azetidine sulfone **14c** (1.38 g, 3.0 mmol, 1.0 equiv) was added to a solution of NaH (60% in mineral oil, 126 mg, 3.15 mmol, 1.05 equiv) in THF (15 mL, 0.2 M) at 0 °C. The reaction mixture was stirred at 25 °C for 1 h then quenched with anhydrous MeOH (6 mL) and concentrated under reduced pressure at 20 °C to afford sulfinate salt as a pale-yellow solid, which was subjected directly to the next step without spectroscopical analysis. For the fluorination step, pre-cooled (0 °C) anhydrous MeOH (12 mL, 0.25 M) and Selectfluor^{TM} (4.05 g, 4.5 mmol, 1.5 equiv) were added sequentially to the azetidine sulfinate salt at 0 °C, then stirred at 0 °C for 1 h. The reaction mixture was concentrated *in vacuo* using a rotary evaporator. The resulting residue was diluted with Et_2O (50 mL) and filtered through a plug of Celite, eluting with further Et_2O (100 mL). After concentration *in vacuo* using a rotary evaporator, purification by flash column chromatography (40% Et_2O/pentane) afforded azetidine sulfonyl fluoride **14** as a white solid (1.01 g, 86%). R_f = 0.24 (40% Et_2O/pentane); mp = 118–120 °C; IR (film)/cm^{-1} 2924, 1716 (C=O st), 1400, 1231, 1213, 1038, 790, 628; ^{1}H NMR (400 MHz, $CDCl_3$) δ 7.41–7.30 (m, 5 H, 5 × Ar-CH), 6.87 (d, *J* = 8.2 Hz, 1 H, Ar-CH), 6.80 (dd, *J* = 8.1, 2.0 Hz, 1 H, Ar-CH), 6.76 (d, *J* = 2.0 Hz, 1 H, Ar-CH), 6.04 (s,

2 H, OCH_2O), 5.12 (s, 2 H, $PhCH_2$), 4.87 (d, *J* = 9.9 Hz, 2 H, C*H*HN*H*H), 4.59 (d, *J* = 9.9 Hz, 2 H, CH*H*NH*H*); ^{13}C NMR (101 MHz, $CDCl_3$) δ 155.6 (C_q=O), 149.4 (Ar-C_q-O), 148.5 (Ar-C_q-O), 135.7 (Ar-*C*$_q$-C_q), 128.6 (2 × Ar-CH), 128.5 (Ar-CH), 128.2 (2 × Ar-CH), 124.5 (Ar-*C*$_q$-CH_2), 122.9 (Ar-CH), 108.8 (Ar-CH), 108.6 (Ar-CH), 102.0 (OCH_2O), 67.6 ($PhCH_2$), 63.5 (d, $^2J_{C\text{-}F}$ = 16.0 Hz) 57.5 (br, CH_2NCH_2); ^{19}F{^{1}H} NMR (377 MHz, $CDCl_3$) δ 33.7; HRMS (TOF-MS-ES$^+$) *m/z* calcd for $C_{18}H_{16}NO_6FSNa^+$ $[M+Na]^+$: 416.0580, found: 416.0578.

Azetidine Sulfonyl Fluoride 15

Benzyl 3-hydroxy-3-(3,4,5-trimethoxyphenyl)azetidine-1-carboxylate (15a) and benzyl 3-((3-methoxy-3-oxopropyl)thio)-3-(3,4,5-trimethoxyphenyl)azetidine-1-carboxylate (15b)

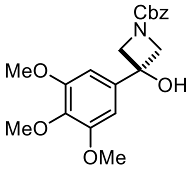

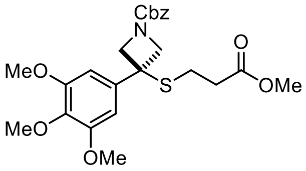

15a and **15b** were prepared according to previously reported procedures.[11,14]

Benzyl 3-((3-methoxy-3-oxopropyl)sulfonyl)-3-(3,4,5-trimethoxyphenyl)azetidine-1-carboxylate (15c)

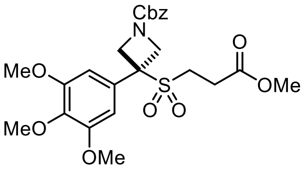

*m*CPBA (≤77%, 700 mg, 3.14 mmol, 3.0 equiv) was added to a solution of azetidine sulfide **15b** (497 mg, 1.04 mmol, 1.0 equiv) in CH_2Cl_2 (21 mL, 0.05 M) in a 50 mL round-bottom flask. The reaction mixture was stirred at 25 °C for 3 h then aq. KOH (3 M, 20 mL) was added and the phases were separated. The aqueous portion was extracted with CH_2Cl_2 (4 × 20 mL). The organic extracts were combined, dried over Na_2SO_4, filtered and concentrated *in vacuo* using a rotatory evaporator to afford sulfone **15c** a white solid (514 mg, 97%). R_f = 0.21 (Et_2O); mp = 160–163 °C; IR (film)/cm^{-1} 2948, 1709 (C=O st), 1588, 1507, 1482, 1415, 1355 (SO_2 st as), 1245, 1122 (SO_2 st as), 768; ^{1}H NMR (400 MHz, $CDCl_3$) δ 7.43–7.31 (m, 5 H, 5 × Ar-CH), 6.56 (s, 2 H, 2 × Ar-CH), 5.13 (s, 2 H, $PhCH_2$), 4.87 (br s, 2 H, C*H*HNC*H*H), 4.52 (d, *J* = 9.5 Hz, 2 H, CH*H*NCH*H*), 3.89 (s, 9 H, 3 × OCH_3), 3.72 (s, 3 H, CO_2CH_3), 3.17–3.06 (m, 2 H, SO_2CH_2), 2.70 (t, *J* = 7.6 Hz, 2 H, C*H*$_2$$CO_2CH_3$); ^{13}C NMR (101 MHz, $CDCl_3$) δ 170.7 (*C*O_2CH_3), 155.8 (N*C*$_q$-O_2CH_2), 153.6 (2 × Ar-*C*$_q$-OCH_3), 139.1 (Ar-*C*$_q$-OCH_3), 136.0 (Ar-*C*$_q$-C_q), 128.8 (Ar-*C*$_q$-CH_2), 128.6 (2 × Ar−CH), 128.3 (Ar-CH), 128.2 (2 × Ar−CH), 105.8 (2 × Ar-CH), 67.4 ($PhCH_2$), 64.0 (C_q), 61.0 (OCH_3), 56.5 (2 × OCH_3), 55.9 (br, CH_2NCH_2), 52.5 (CO_2*C*H_3), 43.3 (*C*$H_2CO_2CH_3$), 26.0 (SO_2CH_2); HRMS (TOF-MS-ES$^+$) *m/z* calcd for $C_{24}H_{33}N_2O_9S^+$ $[M+NH_4]^+$: 525.1907, found: 525.1906.

Benzyl 3-(fluorosulfonyl)-3-(3,4,5-trimethoxyphenyl)azetidine-1-carboxylate (15)

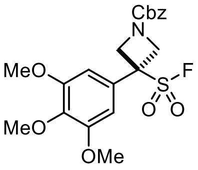

Prepared according to **azetidine sulfinate elimination procedure 2 (ASE-2)** from sulfone **15c** and submitting the sulfinate intermediate directly to the fluorination conditions. Azetidine sulfone **15c** (483 mg, 0.95 mmol, 1.0 equiv) was added to a solution of NaH (60% in mineral oil, 39.9 mg, 1.00 mmol, 1.05 equiv) in THF (4.75 mL, 0.2 M) at 0 °C. The reaction mixture was stirred at 25 °C for 1 h then quenched with anhydrous MeOH (6 mL) and concentrated under reduced pressure at 20 °C to afford sulfinate salt as a pale-yellow residue, which was subjected directly to the next step without spectroscopical analysis. For the fluorination step, pre-cooled (0 °C) anhydrous MeOH (3.8 mL, 0.25 M) and Selectfluor™ (505 mg, 1.43 mmol, 1.5 equiv) were added sequentially to the azetidine

sulfinate salt at 0 °C, then stirred at 0 °C for 1 h. The reaction mixture was concentrated *in vacuo* using a rotary evaporator. The resulting residue was diluted with Et_2O (20 mL) and filtered through a plug of Celite, eluting with further Et_2O (50 mL). After concentration *in vacuo* using a rotary evaporator, purification by flash column chromatography through a short column (Et_2O) afforded azetidine sulfonyl fluoride **15** as a white solid (312 mg, 75%). R_f = 0.28 (60% Et_2O/pentane); mp = 137–140 °C; IR (film)/cm^{-1} 2924, 1716 (C=O st), 1590, 1456, 1400 (SO_2 st as), 1355, 1246, 1207 (SO_2 st sy), 1129; ^{1}H NMR (400 MHz, $CDCl_3$) δ 7.42–7.29 (m, 5 H, 5 × Ar-CH), 6.49 (s, 2 H, 2 × Ar-CH), 5.13 (s, 2 H, $PhCH_2$), 4.90 (d, *J* = 9.9 Hz, 2 H, C*H*HNC*H*H), 4.66 (d, *J* = 9.9 Hz, 2 H, CH*H*NCH*H*), 3.87 (m, 9 H, 3 × OCH_3); ^{13}C NMR (126 MHz, $CDCl_3$) δ 155.6 (C_q=O), 153.6 (2 × Ar-C_q-OCH_3), 139.7 (Ar-C_q-OCH_3), 135.7 (Ar-C_q-C_q), 128.6 (2 × Ar-CH), 128.5 (Ar-CH), 128.3 (2 × Ar-CH), 126.3 (Ar-C_q-CH_2), 105.9 (2 × Ar-CH), 67.6 ($PhCH_2$), 63.7 (d, $^2J_{C\text{-}F}$ = 16.0 Hz, C_q), 60.9 (OCH_3), 57.8 and 57.1 (CH_2NCH_2), 56.4 (2 × OCH_3); ^{19}F{^{1}H} NMR (377 MHz, $CDCl_3$) δ 34.4; HRMS (TOF-MS-ES$^+$) *m/z* calcd for $C_{20}H_{23}NO_7FS^+$ $[M+H]^+$: 440.1179, found: 440.1181.

Divergent access to azetidine sulfonyl fluorides

Azetidine Sulfonyl Fluoride 147

Methyl 3-((3-(4-methoxyphenyl)azetidin-3-yl)thio)propanoate (145)

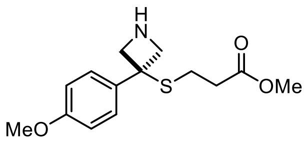

$SiMe_3I$ (24.4 mL, 172 mmol, 8.1 equiv) was added dropwise to a solution of azetidine sulfide **11b** (8.36 g, 20.1 mmol, 1.0 equiv) in MeCN (100 mL, 0.2 M) at –10 °C in a 250 mL round-bottom flask. The reaction mixture was stirred at –10 °C for 4 h then quenched with MeOH (25 mL) and stirred for 10 min. The solvent was removed *in vacuo* using a rotary evaporator. Purification by recrystallization in a solvent/antisolvent ($MeOH/CH_2Cl_2$) system afforded azetidine sulfide **145** as a white solid (5.66 g, quant.). R_f = 0.32 (15% $MeOH/CH_2Cl_2$); mp = 178 °C; IR film/cm^{-1} 2913 (NH st), 2359, 1720 (C=O st), 1511, 1243, 1097, 837; ^{1}H NMR (400 MHz, CD_3OD) δ 7.27 (d, *J* = 9.0 Hz, 2 H, 2 × Ar-CH), 6.99 (d, *J* = 9.0 Hz, 2 H, 2 × Ar-CH), 4.66 (d, *J* = 11.9 Hz, 2 H, C*H*HNC*H*H), 4.27 (d, *J* = 11.9 Hz, 2 H, CH*H*NCH*H*), 3.82 (s, 3 H, OCH_3), 3.63 (s, 3 H, CO_2CH_3), 2.60 (t, *J* = 7.0 Hz, 2 H, SCH_2), 2.34 (t, *J* = 7.0 Hz, 2 H, $CH_2CO_2CH_3$); ^{13}C NMR (101 MHz, CD_3OD) δ 173.7 (CO_2CH_3), 160.9 (Ar-C_q-OCH_3), 134.0 (Ar-C_q-C_q), 128.5 (2 × Ar-CH), 115.3 (2 × Ar-CH), 59.2 (CH_2NCH_2), 55.9 (OCH_3), 52.3 (CO_2CH_3), 51.1 (C_q), 34.5 ($CH_2CO_2CH_3$), 26.4 (SCH_2); HRMS (TOF-MS-ES$^+$) *m/z* calcd for $C_{14}H_{20}NO_3S^+$ $[M+H]^+$: 282.1164, found: 282.1157.

***tert*-Butyl 3-((3-methoxy-3-oxopropyl)thio)-3-(4-methoxyphenyl)azetidine-1-carboxylate (147b)**

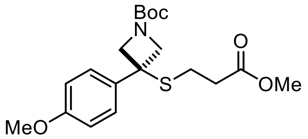

Boc anhydride (1.05 g, 4.80 mmol 1.5 equiv) was added portionwise to a solution of azetidine sulfide **145** (900 mg, 3.20 mmol, 1.0 equiv) and K_2CO_3 (1.14 g, 9.67 mmol, 3.0 equiv) in anhydrous CH_2Cl_2 (15 mL, 0.2 M) in a 50 mL round-bottom flask. The reaction mixture was stirred at 25 °C for 2.5 h then quenched with water (15 mL) and extracted with CH_2Cl_2 (3 × 15 mL). The combined organic extracts were washed with water (3 × 15 mL), dried over Na_2SO_4, filtered and concentrated *in vacuo* using a rotary evaporator. Purification by flash column chromatography (20% EtOAc/pentane) afforded azetidine sulfide **147b** as a colorless oil (1.22 g, quant.). R_f = 0.34 (25% EtOAc/pentane); IR (film)/cm^{-1} 2980, 1703 (C=O st), 1702 (C=O st), 1370, 1113, 1060, 842, 731; ^{1}H NMR (400 MHz, $CDCl_3$) δ 7.32–7.24 (m, 2 H, 2 × Ar-CH), 7.00–6.92 (m, 2 H, 2 × Ar-CH), 4.76 (d, *J* = 9.5 Hz, 2 H, C*H*HNC*H*H), 4.39 (d, *J* = 9.5 Hz, 2 H, CH*H*NCH*H*), 3.84 (s, 3 H, OCH_3), 3.69 (s, 3 H, CO_2CH_3), 3.04 (t, *J* = 7.1 Hz, 2 H, SCH_2), 2.68 (t, *J* = 7.1 Hz, 2 H, $CH_2CO_2CH_3$), 1.44 (s, 9 H, $OC(CH_3)_3$); ^{13}C NMR (101 MHz, CD_3OD) δ 173.8 (CO_2CH_3), 160.3 (NC_q=O), 158.0 (Ar-C_q-OCH_3), 135.8 (Ar-C_q-C_q), 128.9 (2 × Ar-*C*H), 115.0 (2 × Ar-*C*H), 81.5 (O*C*$(CH_3)_3$), 64.0 (br, CH_2NCH_2), 55.8 (OCH_3), 52.2 (CO_2CH_3),

48.2 (C_q), 34.8 ($CH_2CO_2CH_3$), 28.6 ($C(CH_3)_3$), 26.2 (SCH_2); HRMS (TOF-MS-ES$^+$) *m/z* calcd for $C_{19}H_{28}NO_5S^+$ $[M+H]^+$: 382.1688, found: 382.1682.

tert-Butyl 3-((3-methoxy-3-oxopropyl)sulfonyl)-3-(4-methoxyphenyl)azetidine-1-carboxylate (147c)

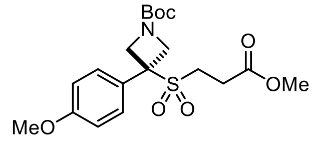

*m*CPBA (≤77%, 878 mg, 3.93 mmol, 3.0 equiv) was added to a solution of azetidine sulfide **147b** (500 mg, 1.31 mmol, 1.0 equiv) in CH_2Cl_2 (50 mL, 0.025 M) in a 100 mL round-bottom flask. The reaction mixture was stirred at 25 °C for 3 h then aq. KOH (3 M, 25 mL) was added and the phases were separated. The aqueous portion was extracted with CH_2Cl_2 (3 × 25 mL). The combined organic extracts were washed with water (3 × 25 mL), dried over Na_2SO_4, filtered and concentrated *in vacuo* using a rotatory evaporator. Purification by flash column chromatography (15% EtOAc/pentane) afforded azetidine sulfone **147c** as a colorless oil (538 mg, 99%); R_f = 0.31 (20% EtOAc/pentane); IR (film)/cm^{-1} 2956, 1703 (2 × C=O st), 1610, 1515, 1392, 1254 (SO_2 st as), 1129 (SO_2 st sy), 1033, 837; ^{1}H NMR (400 MHz, $CDCl_3$) δ 7.27 (d, *J* = 8.6 Hz, 2 H, 2 × Ar-CH), 6.95 (d, *J* = 8.6 Hz, 2 H, 2 × Ar-CH), 4.75 (br d, *J* = 9.5 Hz, 2 H, C*H*HNC*H*H), 4.38 (d, *J* = 9.5 Hz, 2 H, CH*H*NCH*H*), 3.83 (s, 3 H, OCH_3), 3.68 (s, 3 H, CO_2CH_3), 3.03 (br t, *J* = 7.7 Hz, 2 H, SO_2CH_2), 2.67 (t, *J* = 7.7 Hz, 2 H, $CH_2CO_2CH_3$), 1.43 (s, 9 H, $OC(CH_3)_3$); ^{13}C NMR (101 MHz, $CDCl_3$) δ 170.9 (CO_2CH_3) , 160.5 (NC_q=O), 155.7 (Ar-C_q-OCH_3), 129.9 (2 × Ar-*C*H), 125.5 (Ar-C_q-C_q), 114.6 (2 × Ar-*C*H), 80.7 ($OC(CH_3)_3$), 63.1 (C_q), 56.6 (br, CH_2NCH_2), 55.5 (OCH_3), 52.5 (CO_2CH_3), 42.8 ($CH_2CO_2CH_3$), 28.4 ($C(CH_3)_3$), 25.8 (SO_2CH_2); HRMS (TOF-MS-ES$^+$) *m/z* calcd for $C_{19}H_{28}NO_7S^+$ $[M+H]^+$: 414.1586, found: 414.1571.

tert-Butyl 3-(fluorosulfonyl)-3-(4-methoxyphenyl)azetidine-1-carboxylate (147)

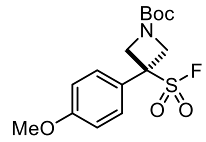

Prepared according to **azetidine sulfinate elimination procedure 2 (ASE-2)** from sulfone **147c** and submitting the sulfinate intermediate directly to the fluorination conditions. A solution of azetidine sulfone **147c** (400 mg, 0.96 mmol, 1.0 equiv) in anhydrous THF (2.5 mL) was added dropwise to a solution of NaH (60% in mineral oil, 46.2 mg, 1.16 mmol, 1.2 equiv) in anhydrous THF (5 mL) at 0 °C. The reaction mixture was stirred at 25 °C for 1 h then quenched with anhydrous MeOH (0.75 mL) and concentrated under reduced pressure at 20 °C to afford the azetidine sulfinate salt as an off-white solid, which was subjected directly to the next step without spectroscopical analysis. For the fluorination step, pre-cooled (0 °C) anhydrous MeOH (8.0 mL, 0.2 M) and Selectfluor™ (510 mg, 1.43 mmol, 1.5 equiv) were added sequentially to the azetidine sulfinate salt residue at 0 °C, then stirred at 0 °C for 1 h. The reaction mixture was concentrated *in vacuo* using a rotary evaporator. The resulting residue was diluted with CH_2Cl_2 (15 mL) and filtered through a plug of Celite, eluting with further CH_2Cl_2 (40 mL). After concentration *in vacuo* using a rotary evaporator, purification by recrystallization at –78 °C from solvent/anti-solvent (CH_2Cl_2/pentane) system removed impurities as a white solid. The mother liquor was concentrated *in vacuo* using a rotary evaporator to afford azetidine sulfonyl fluoride **147** as a colorless oil (205 mg, 62%). R_f = 0.22 (20% CH_2Cl_2/pentane); IR (film)/cm^{-1} 2973, 2931, 1709 (C=O st), 1610, 1515, 1397, 1367, 1256, 1146, 1032, 787; ^{1}H NMR (400 MHz, $CDCl_3$) δ 7.30–7.23 (m, 2 H, 2 × Ar-CH), 7.00–6.93 (m, 2 H, 2 × Ar-CH), 4.82 (d, *J* = 10.0 Hz, 2 H, C*H*HNC*H*H), 4.58–4.50 (m, 2 H, CH*H*NCH*H*), 3.84 (s, 3 H, OCH_3), 1.45 (s, 9 H, $C(CH_3)_3$); ^{13}C NMR (101 MHz, $CDCl_3$) δ 161.0 (C_q=O), 155.5 (Ar-C_q-OCH_3), 130.0 (2 × Ar-CH), 123.3 (Ar-C_q-C_q), 114.7 (2 × Ar-CH), 81.3 ($C(CH_3)_3$), 63.1 (d, $^2J_{C\text{-}F}$ = 15.6 Hz, C_q), 57.0 (br, CH_2NCH_2), 55.6 (OCH_3), 28.3 ($C(CH_3)_3$); ^{19}F{^{1}H} NMR (377 MHz, $CDCl_3$) δ 33.3; HRMS (TOF-MS-ES$^+$) *m/z* calcd for $C_{15}H_{20}NO_3^+$ $[M–SO_2F]^+$: 262.1443, found: 262.1452.

Notes:

*Purification by flash column chromatography using silica is not a viable purification technique as azetidine sulfonyl fluoride **147** is unstable on silica and degradation to the corresponding azetidinol is observed.*

Azetidine Sulfonyl Fluoride 150

Methyl 3-((3-(4-hydroxyphenyl)azetidin-3-yl)thio)propanoate (146)

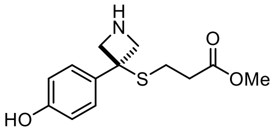

$SiMe_3I$ (1.40 mL, 9.84 mmol, 8.1 equiv) was added dropwise to a solution of azetidine sulfide **12b** (681 mg, 1.22 mmol, 1.0 equiv) in MeCN (6.0 mL, 0.2 M) at –10 °C in a 25 mL round-bottom flask. The reaction mixture was stirred at –10 °C for 4 h then quenched with MeOH (2.5 mL) and stirred for 10 min. The solvent was removed *in vacuo* using a rotary evaporator. Purification by flash column chromatography (10% MeOH/CH_2Cl_2) afforded azetidine sulfide **146** as a brown gum (233 mg, 71%). R_f = 0.29 (15% MeOH/CH_2Cl_2); IR (film)/cm^{-1} 3286 (NH and OH st), 2947, 1731 (C=O st), 1610, 1513, 1437, 1362, 1251, 1176, 835; ^{1}H NMR (400 MHz, CD_3OD) δ 7.13 (d, *J* = 8.6 Hz, 2 H, 2 × Ar-CH), 6.80 (d, *J* = 8.6 Hz, 2 H, 2 × Ar-CH), 5.46 (s, 1 H, NH), 4.61 (d, *J* = 11.7 Hz, 2 H, C*H*HNC*H*H), 4.22 (d, *J* = 11.7 Hz, 2 H, CH*H*NCH*H*), 3.59 (s, 3 H, CO_2CH_3), 2.57 (t, *J* = 7.0 Hz, 2 H, SCH_2), 2.30 (t,z *J* = 7.0 Hz, 2 H, $CH_2CO_2CH_3$); ^{13}C NMR (101 MHz, CD_3OD) δ 173.7 (CO_2CH_3), 158.4 (Ar-C_q-OH), 132.6 (Ar-C_q-C_q), 128.5 (2 × Ar-CH), 116.5 (2 × Ar-CH), 59.4 (CH_2NCH_2), 52.3 (CO_2CH_3), 51.1 (C_q), 34.5 ($CH_2CO_2CH_3$), 26.4 (SCH_2); HRMS (TOF-MS-ES$^+$) *m/z* calcd for $C_{13}H_{18}NO_3S^+$ [M+H]$^+$: 268.1007, found: 268.1003.

tert-Butyl 3-(4-hydroxyphenyl)-3-((3-methoxy-3-oxopropyl)thio)azetidine-1-carboxylate (148)

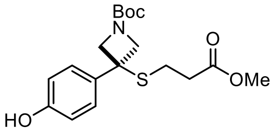

Boc anhydride (41.6 mg, 0.19 mmol 1.0 equiv) was added portionwise to a solution of azetidine sulfide **146** (51.1 mg, 0.19 mmol, 1.0 equiv) and K_2CO_3 (51.1 mg, 0.36 mmol, 1.9 equiv) in anhydrous MeOH (0.40 mL, 0.2 M) in a 10 mL round-bottom flask. The reaction mixture was stirred at 25 °C for 2.5 h and then concentrated *in vacuo* using a rotary evaporator. The crude residue was diluted in 10% MeOH/pentane (10 mL) and filtered through a short plug of silica. Purification by flash column chromatography (25% EtOAc/pentane) afforded azetidine sulfide **148** as an impure pale red oil (26.7 mg, 38%, 90% purity). R_f = 0.19 (25% EtOAc/pentane); IR (film)/cm^{-1} 2978 and 2952 (br OH st), 1737 (C=O st), 1702 (C=O st), 1672, 1420, 1366, 1144; ^{1}H NMR (400 MHz, $CDCl_3$) δ 7.06 (d, *J* = 8.7 Hz, 2 H, 2 × Ar-CH), 6.81 (d, *J* = 8.7 Hz, 2 H, 2 × Ar-CH), 4.44 (d, *J* = 8.1 Hz, 2 H, C*H*HNC*H*H), 4.22 (t, *J* = 8.1 Hz, 2 H, CH*H*NCH*H*), 3.65 (s, 3 H, CO_2CH_3), 2.58 (t, *J* = 7.3 Hz, 2 H, SCH_2), 2.36 (t, *J* = 7.4 Hz, 2 H, $CH_2CO_2CH_3$), 1.44 (s, 9 H, $C(CH_3)_3$); ^{13}C NMR (101 MHz, CD_3OD) δ 172.4 (CO_2CH_3), 156.5 (NC_q=O), 155.7 (Ar-C_q-OH), 133.9 (Ar-C_q-C_q), 127.9 (2 × Ar-CH), 115.6 (2 × Ar-CH), 80.6 ($C_q(CH_3)_3$), 61.9 (CH_2NCH_2), 52.0 (CO_2CH_3), 46.9 (C_q), 33.9 ($CH_2CO_2CH_3$), 28.5 ($C(CH_3)_3$), 25.3 (SCH_2); HRMS (TOF-MS-ES$^+$) *m/z* calcd for $C_{18}H_{26}NO_5S^+$ [M+H]$^+$: 368.1532, found: 368.1532.

Notes:

The observed broad OH stretch in the IR analysis indicates the presence of the free phenol rather than the free azetidine NH.

*If this reaction is performed in CH_2Cl_2, acetone or MeCN then the phenolic OH also reacts with boc-anhydride to yield tert-butyl 3-(4-((tert-butoxycarbonyl)oxy)phenyl)-3-((3-methoxy-3-oxopropyl)thio)azetidine-1-carboxylate **S6** – reported below.*

tert-Butyl 3-(4-((tert-butoxycarbonyl)oxy)phenyl)-3-((3-methoxy-3-oxopropyl)thio)azetidine-1-carboxylate (S6)

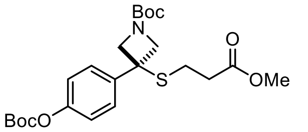

Boc anhydride (120 mg, 0.55 mmol 1.0 equiv) was added portionwise to a solution of azetidine sulfide **146** (147 mg, 0.55 mmol, 1.0 equiv) and K_2CO_3 (165 mg, 1.19 mmol, 2.2 equiv) in anhydrous CH_2Cl_2 (1.50 mL, 0.4 M) in a 50 mL round-bottom flask. The reaction mixture was stirred at 25 °C for 2.5 h then quenched with water (1.5 mL) and extracted with CH_2Cl_2 (3 × 5 mL). The combined organic extracts were washed with water (3 × 5 mL), dried over Na_2SO_4, filtered and concentrated *in vacuo* using a rotary evaporator. Purification by flash column chromatography (10% EtOAc/pentane) afforded azetidine sulfide **S6** as an orange oil (124 mg, 98%). R_f = 0.67 (25% EtOAc/pentane); IR (film)/cm^{-1} 2982, 1802 (C=O st), 1755 (C=O st), 1371, 1211, 1113, 1060, 844; ^{1}H NMR (400 MHz, $CDCl_3$) δ 7.24 (d, *J* = 8.7 Hz, 2 H, 2 × Ar-CH), 7.16 (d, *J* = 8.7 Hz, 2 H, 2 × Ar-CH), 4.43 (d, *J* = 8.8 Hz, 2 H, C*H*HNC*H*H), 4.20 (d, *J* = 8.8 Hz, 2 H, CH*H*NCH*H*), 3.64 (s, 3 H, CO_2CH_3), 2.59 (t, *J* = 7.4 Hz, 2 H, SCH_2), 2.38 (t, *J* = 7.4 Hz, 2 H, C*H*$_2CO_2CH_3$), 1.54 (s, 9 H, $OCOOC(CH_3)_3$), 1.42 (s, 9 H, $NCOOC(CH_3)_3$). ^{13}C NMR (101 MHz, $CDCl_3$) δ 172.0 (*C*O_2CH_3), 156.1 (*C*$O_2C(CH_3)_3$), 151.8 (*C*$O_2C(CH_3)_3$), 150.1 (Ar-*C*$_q$-$OCO_2C(CH_3)_3$), 140.2 (Ar-*C*$_q$-C_q), 127.6 (2 × Ar-CH), 121.6 (2 × Ar-*C*H), 83.9 (O*C*$(CH_3)_3$), 80.1 (O*C*$(CH_3)_3$), 62.8 (br, CH_2NCH_2), 51.9 (CO_2*C*H_3), 46.8 (C_q), 33.7 (*C*$H_2CO_2CH_3$), 28.4 (NCO_2C(*C*$H_3)_3$), 27.8 (OCO_2C(*C*$H_3)_3$), 25.2 (S*C*H_2); HRMS (TOF-MS-ES$^+$) *m/z* calcd for $C_{23}H_{34}NO_7S^+$ $[M+H]^+$: 468.2056, found: 468.2068.

tert-Butyl 3-((3-methoxy-3-oxopropyl)thio)-3-(4-(prop-2-yn-1-yloxy)phenyl)azetidine-1-carboxylate (150b)

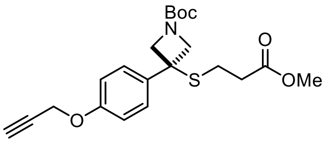

Propargyl bromide (~80% solution in toluene, 0.22 mL, 2.0 mmol, 2.0 equiv) was added dropwise to a solution of phenol sulfide **148** (367 mg, 1.0 mmol, 1.0 equiv) and K_2CO_3 (276 mg, 2.0 mmol, 2.0 equiv) in non-anhydrous acetone (1.0 mL, 0.6 M) in a 25 mL round-bottom flask and the reaction mixture stirred at 40 °C for 20 h. The solvent was removed *in vacuo* using a rotary evaporator and the resulting residue re-dissolved into EtOAc (10 mL). The organic layer was washed with water (3 × 5 mL), dried over Na_2SO_4, filtered and concentrated *in vacuo* using a rotary evaporator. Purification by flash column chromatography (25% EtOAc/pentane) to afford sulfide **150b** as a pale red oil (357 mg, 88%). R_f = 0.21 (20% EtOAc/pentane); IR (film)/cm^{-1} 3299 (≡C-H st), 2952, 1735 (C=O st), 1695 (C=O st), 1509, 1392, 1221, 1140, 910, 729; ^{1}H NMR (400 MHz, $CDCl_3$) δ 7.18 (dd, *J* = 8.5, 1.5 Hz, 2 H, 2 × Ar-CH), 6.96 (dd, *J* = 8.5, 1.5 Hz, 2 H, 2 × Ar-CH), 4.69 (s, 2 H, C*H*$_2$C≡CH), 4.43 (d, *J* = 8.8 Hz, 2 H, C*H*HNC*H*H), 4.20 (d, *J* = 8.8 Hz, 2 H, CH*H*NCH*H*), 3.64 (s, 3 H, CO_2CH_3), 2.59 (t, *J* = 7.3 Hz, 2 H, SCH_2), 2.53 (t, *J* = 2.1 Hz, 1 H, CH_2C≡C*H*), 2.37 (t, *J* = 7.3 Hz, 2 H, C*H*$_2CO_2CH_3$), 1.42 (s, 9 H, $C(CH_3)_3$); ^{13}C NMR (101 MHz, $CDCl_3$) δ 172.1 (*C*$_qO_2CH_3$), 156.7 (*C*$_qO_2C(CH_3)_3$), 156.2 (Ar-*C*$_q$-OCH_2C≡CH), 135.6 (Ar-*C*$_q$-C_q), 127.8 (2 × Ar-CH), 115.1 (2 × Ar-CH), 80.1 (O*C*$(CH_3)_3$), 78.4 (CH_2*C*≡CH), 75.9 (CH_2C≡*C*H), 62.4 (br, CH_2NCH_2), 56.0 (*C*H_2C≡CH), 51.9 (CO_2*C*H_3), 46.9 (C_q), 33.9 (*C*$H_2CO_2CH_3$), 28.5 (NCO_2C(*C*$H_3)_3$), 25.3 (SCH_2); HRMS (TOF-MS-ES$^+$) *m/z* calcd for $C_{21}H_{28}NO_5S^+$ $[M+H]^+$: 406.1688, found: 406.1696.

tert-Butyl 3-((3-methoxy-3-oxopropyl)sulfonyl)-3-(4-(prop-2-yn-1-yloxy)phenyl)azetidine-1-carboxylate (150c)

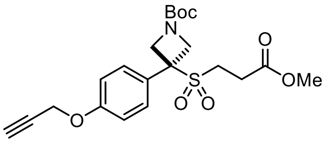

*m*CPBA (≤77%, 2.15 g, 8.74 mmol, 3.0 equiv) was added to a solution of azetidine sulfide **150b** (1.18 g, 2.91 mmol, 1.0 equiv) in CH_2Cl_2 (100 mL, 0.03 M) in a 250 mL round-bottom flask. The reaction mixture was stirred at 25 °C for 4 h then aq. KOH (3 M, 40 mL) was added and the phases were separated. The aqueous portion was extracted with CH_2Cl_2 (3 × 30 mL). The combined organic extracts were washed with water (3 × 30 mL), dried over Na_2SO_4, filtered and concentrated *in vacuo*

using a rotatory evaporator. Purification by flash column chromatography (30% EtOAc/pentane) afforded azetidine sulfone **150c** as a pale yellow gum (1.25 g, 98%); R_f = 0.55 (50% EtOAc/pentane); IR (film)/cm^{-1} 3271, 2976, 1741 (C=O st), 1697 (C=O st), 1511, 1366 (SO_2 st as), 1125 (SO_2 st sy), 1023, 836, 752; ^{1}H NMR (400 MHz, $CDCl_3$) δ 7.30 (d, *J* = 8.4 Hz, 2 H, 2 × Ar-C*H*), 7.04 (d, *J* = 8.4 Hz, 2 H, 2 × Ar-C*H*), 4.80–4.70 (m, 4 H, C*H*HNC*H*H and C*H*$_2$CCH), 4.39 (d, *J* = 9.6 Hz, 2 H, CH*H*NCH*H*), 3.69 (s, 3 H, CO_2CH_3), 3.03 (t, *J* = 7.7 Hz, 2 H, SO_2CH_2), 2.68 (t, *J* = 7.7 Hz, 2 H, CH_2CO_2Me), 2.55 (t, *J* = 2.4 Hz, 1 H, CH_2CC*H*), 1.44 (s, 9 H, $C(CH_3)_3$); ^{13}C NMR (101 MHz, $CDCl_3$) δ 170.9 (CO_2CH_3), 158.4 (N*C*=O), 155.7 (Ar-C_q-OCH_2CCH), 129.9 (2 × Ar-CH), 126.5 (Ar-C_qC_q), 115.5 (2 × Ar-CH), 80.8 (O*C*$(CH_3)_3$), 78.0 (*C*H_2CCH), 76.3 (CH_2*C*CH), 63.1 (CO_2OCH_3), 56.1 (br, C_q), 56.0 (CH_2NCH_2), 52.6 (Ar-C_q-O*C*H_2CCH), 42.9 ($CH_2CO_2CH_3$), 28.4 ($C(CH_3)_3$), 25.8 (SO_2CH_2); HRMS (TOF-MS-ES$^+$) *m/z* calcd for $C_{21}H_{28}NO_7S^+$ [M+H]$^+$: 438.1586, found: 438.1576.

***tert*-Butyl 3-(fluorosulfonyl)-3-(4-(prop-2-yn-1-yloxy)phenyl)azetidine-1-carboxylate (150)**

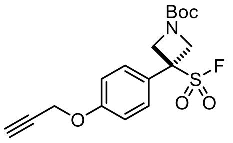

Prepared according to **azetidine sulfinate elimination procedure 2 (ASE-2)** from sulfone **150c** and submitting the sulfinate intermediate directly to the fluorination conditions. A solution of azetidine sulfone **150c** (287 mg, 0.66 mmol, 1.0 equiv) in anhydrous THF (1.60 mL) was added dropwise to a solution of NaH (60% in mineral oil, 27.7 mg, 0.69 mmol, 1.05 equiv) in anhydrous THF (1.70 mL) at 0 °C. The reaction mixture was stirred at 25 °C for 1 h then quenched with anhydrous MeOH (0.60 mL) and concentrated under reduced pressure at 20 °C to afford the azetidine sulfinate salt as a pale-yellow oil, which was subjected directly to the next step without spectroscopical analysis. For the fluorination step, pre-cooled (0 °C) anhydrous MeOH (4.40 mL, 0.15 M) and Selectfluor^{TM} (351 mg, 0.99 mmol, 1.5 equiv) were added sequentially to the azetidine sulfinate salt residue at 0 °C, then stirred at 0 °C for 1 h. The reaction mixture was concentrated *in vacuo* using a rotary evaporator. The resulting residue was diluted with Et_2O (10 mL) and filtered through a plug of Celite, eluting with further Et_2O (30 mL). The filtrate was concentrated *in vacuo* using a rotary evaporator. Purification by flash column chromatography (50% CH_2Cl_2/pentane) afforded azetidine sulfonyl fluoride **150** as a colorless oil (134 mg, 0.36 mmol, 55%). R_f = 0.25 (60% CH_2Cl_2/pentane); IR (film)/cm^{-1} 3288, 2974, 2926, 1701 (C=O st), 1395, 1213, 1146, 835, 787; ^{1}H NMR (400 MHz, $CDCl_3$) δ 7.29 (d, *J* = 8.8 Hz, 2 H, 2 × Ar-CH), 7.06 (d, *J* = 8.8 Hz, 2 H, 2 × Ar-CH), 4.82 (d, *J* = 9.9 Hz, 2 H, C*H*HNC*H*H), 4.73 (d, *J* = 2.4 Hz, 2 H, C*H*$_2$CCH), 4.54 (d, *J* = 9.9 Hz, 2 H, CH*H*NCH*H*), 2.56 (t, *J* = 2.4 Hz, 1 H, CH_2CC*H*), 1.45 (s, 9 H, $C(CH_3)_3$); ^{13}C NMR (101 MHz, $CDCl_3$) δ 158.9 (N*C*=O), 155.5 (Ar-C_q-OCH_2CCH), 130.1 (2 × Ar-CH), 124.3 (Ar-C_q-C_q), 115.6 (2 × Ar-CH), 81.3 (O*C*$(CH_3)_3$), 77.9 (CH_2*C*CH), 76.4 (*C*H_2CCH), 75.9 (CH_2C*C*H), 63.0 (br, C_q), 56.0 (CH_2NCH_2), 28.4 ($OC(CH_3)_3$); ^{19}F{^{1}H} NMR (377 MHz, $CDCl_3$) δ 33.5; HRMS (TOF-MS-ES$^+$) *m/z* calcd for $C_{17}H_{20}NO_3^+$ [M–SO_2F]$^+$: 286.1443, found: 286.1443.

Azetidine Sulfonyl Fluoride 152

Prop-2-yn-1-yl 3-((3-methoxy-3-oxopropyl)thio)-3-(4-methoxyphenyl)azetidine-1-carboxylate (152b)

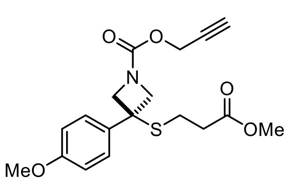

Azetidine sulfide **145** (582 mg, 2.07 mmol, 1.0 equiv) was added to a solution of K_2CO_3 (579 mg, 4.19 mmol, 2.0 equiv) in anhydrous CH_2Cl_2 (10 mL, 0.2 M) in a 25 mL round-bottom flask. The resulting mixture was stirred at 25 °C for 5 min. Propargyl chloroformate (0.31 mL, 3.18 mmol, 1.5 equiv) was added dropwise to the reaction mixture over 10 min. The reaction mixture was stirred at 25 °C for 16 h then quenched with water (15 mL) and extracted with CH_2Cl_2 (3 × 15 mL). The combined organic extracts were washed with water (3 × 15 mL), dried over Na_2SO_4, filtered and concentrated *in vacuo* using a rotary evaporator. Purification by flash column chromatography (15% EtOAc/pentane) afforded azetidine sulfide **152b** as a colorless oil (569 mg, 76%). R_f = 0.37 (20% EtOAc/pentane); IR (film)/cm^{-1} 3284 (≡C-H st), 2950, 1707 (C=O st), 1610, 1511, 1411, 1344, 1243,

1120, 833; ^{1}H NMR (400 MHz, CD_3OD) δ 7.23 (d, *J* = 8.8 Hz, 2 H, 2 × Ar-CH), 6.93 (d, *J* = 8.8 Hz, 2 H, 2 × Ar-CH), 4.68 (d, *J* = 2.5 Hz, 2 H, C*H*$_2$C≡CH), 4.59–4.46 (m, 2 H, C*H*HNC*H*H), 4.31–4.20 (m, 2 H, CH*H*NCH*H*), 3.80 (s, 3 H, OCH_3), 3.63 (s, 3 H, CO_2CH_3), 2.93 (t, *J* = 2.4 Hz, 1 H, CH_2C≡C*H*), 2.59 (t, *J* = 7.1 Hz, 2 H, SCH_2), 2.33 (t, *J* = 7.1 Hz, 2 H, C*H*$_2CO_2CH_3$); ^{13}C NMR (101 MHz, CD_3OD) δ 173.8 (*C*O_2CH_3), 160.4 (NC_q=O), 157.2 (Ar-C_q-OCH_3), 135.5 (Ar-C_q-C_q), 128.9 (2 × Ar-CH), 115.0 (2 × Ar-CH), 79.1 (CH_2*C*≡CH), 76.3 (CH_2C≡*C*H), 64.0 and 63.4 (CH_2NCH_2), 55.8 (OCH_3), 53.8 (*C*H_2C≡CH), 52.2 (CO_2*C*H_3), 48.7 (C_q), 34.7 (*C*$H_2CO_2CH_3$), 26.3 (SCH_2); HRMS (TOF-MS-ES$^+$) *m/z* calcd for $C_{18}H_{22}NO_5S^+$ [M+H]$^+$: 364.1219, found: 364.1213.

Prop-2-yn-1-yl 3-((3-methoxy-3-oxopropyl)sulfonyl)-3-(4-methoxyphenyl)azetidine-1-carboxylate (152c)

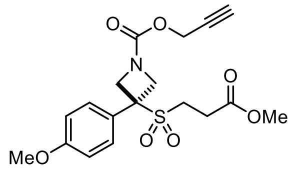

*m*CPBA (≤77%, 1.21 g, 7.04 mmol, 4.3 equiv) was added to a solution of azetidine sulfide **152b** (600 mg, 1.65 mmol, 1.0 equiv) in CH_2Cl_2 (50 mL, 0.03 M) in a 100 mL round-bottom flask. The reaction mixture was stirred at 25 °C for 4 h then aq. KOH (3 M, 25 mL) was added and the phases were separated. The aqueous portion was extracted with CH_2Cl_2 (3 × 25 mL). The combined organic extracts were washed with water (3 × 25 mL), dried over Na_2SO_4, filtered and concentrated *in vacuo* using a rotatory evaporator. Purification by flash column chromatography (40% EtOAc/pentane) afforded azetidine sulfone **152c** as a colorless oil (637 mg, 98%); R_f = 0.28 (50% EtOAc/pentane); IR (film)/cm^{-1} 3274 (≡C-H st), 2952, 1715 (C=O st), 1610, 1515, 1415, 1303 (SO_2 st as), 1251, 1124 (SO_2 st sy), 723; ^{1}H NMR (400 MHz, $CDCl_3$) δ 7.28 (d, *J* = 8.4 Hz, 2 H, 2 × Ar-CH), 6.96 (d, *J* = 8.4 Hz, 2 H, 2 × Ar-CH), 4.84 (br s, 2 H, C*H*HNC*H*H), 4.69 (s, 1 H, C*H*HC≡CH), 4.66 (s, 1 H, CH*H*C≡CH), 4.48 (d, *J* = 9.6 Hz, 2 H, CH*H*NCH*H*), 3.83 (s, 3 H, OCH_3), 3.67 (s, 3 H, CO_2CH_3), 3.02 (t, *J* = 7.6 Hz, 2 H, SO_2CH_2), 2.65 (t, *J* = 7.6 Hz, 2 H, C*H*$_2CO_2CH_3$), 2.48 (t, *J* = 5.5 Hz, 1 H, CH_2C≡C*H*); ^{13}C NMR (101 MHz, $CDCl_3$) δ 170.7 (NC_q=O), 160.5 (*C*O_2CH_3), 154.8 (Ar-C_q-OCH_3), 129.7 (2 × Ar-CH), 125.1 (Ar-C_q-C_q), 114.6 (2 × Ar-CH), 77.9 (CH_2*C*≡CH), 75.1 (CH_2C≡*C*H), 63.3 (C_q), 56.5 and 55.8 (CH_2NCH_2), 55.5 (OCH_3), 53.1 (*C*H_2C≡CH), 52.5 (CO_2*C*H_3), 42.9 (*C*$H_2CO_2CH_3$), 25.8 (SO_2CH_2); HRMS (TOF-MS-ES$^+$) *m/z* calcd for $C_{18}H_{22}NO_7S^+$ [M+H]$^+$: 396.1117, found: 396.1115.

Prop-2-yn-1-yl 3-(fluorosulfonyl)-3-(4-methoxyphenyl)azetidine-1-carboxylate (152)

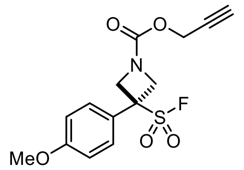

Prepared according to **azetidine sulfinate elimination procedure 2 (ASE-2)** from sulfone **152c** and submitting the sulfinate intermediate directly to the fluorination conditions. A solution of azetidine sulfone **152c** (334 mg, 0.85 mmol, 1.0 equiv) in anhydrous THF (2.25 mL) was added dropwise to a solution of NaH (60% in mineral oil, 35.7 mg, 0.89 mmol, 1.05 equiv) in anhydrous THF (2.0 mL) at 0 °C. The reaction mixture was stirred at 25 °C for 1 h then quenched with anhydrous MeOH (1.0 mL) and concentrated under reduced pressure at 20 °C to afford the azetidine sulfinate salt as a pale-yellow oil, which was subjected directly to the next step without spectroscopical analysis. For the fluorination step, pre-cooled (0 °C) anhydrous MeOH (5.50 mL, 0.15 M) and Selectfluor™ (472 mg, 1.33 mmol, 1.5 equiv) were added sequentially to the azetidine sulfinate salt residue at 0 °C, then stirred at 0 °C for 1 h. The reaction mixture was concentrated *in vacuo* using a rotary evaporator. The resulting residue was diluted with Et_2O (10 mL) and filtered through a plug of Celite, eluting with further Et_2O (30 mL). Concentration of the filtrate *in vacuo* using a rotary evaporator afforded azetidine sulfonyl fluoride **152** as a colorless oil (144 mg, 52%) which could be used without further purification. R_f = 0.30 (CH_2Cl_2); IR (film)/cm^{-1} 3293 (≡C-H st), 2958, 1723 (C=O st), 1517, 1400, 1213 1135, 790; ^{1}H NMR (400 MHz, $CDCl_3$) δ 7.27 (d, *J* = 8.7 Hz, 2 H, 2 × Ar-CH), 7.04–6.92 (m, 2 H, 2 × Ar-CH), 4.91 (d, *J* = 9.8 Hz, 2 H, C*H*HNC*H*H), 4.76–4.52 (m, 4 H, CH*H*NCH*H* and C*H*$_2$CCH), 3.84 (s, 3 H, Ar-C_qOCH_3), 2.49 (t, *J* = 2.4 Hz, 1 H, CH_2CC*H*); ^{13}C NMR (101 MHz, $CDCl_3$) δ 161.1(NC_q=O), 154.7 (Ar-C_q-OCH_3), 129.9 (2 × Ar-CH), 122.8 (Ar-C_q-C_q), 114.8 (2 × Ar-CH), 77.7 (CH_2C≡*C*H), 75.4 (CH_2C≡*C*H), 63.3 (d, $^{2}J_{C\text{-}F}$ = 16.1 Hz, C_q), 57.9 and 53.4 (CH_2NCH_2), 55.6 (OCH_3), 53.4 (O*C*H_2C≡CH); ^{19}F{^{1}H} NMR (377 MHz, $CDCl_3$) δ 33.5; HRMS (TOF-MS-ES$^+$) *m/z* calcd for $C_{14}H_{14}NO_3^+$ [M–SO_2F]$^+$: 244.0974, found: 244.0967.

Notes:

Purification by flash column chromatography using silica is not a viable purification technique as azetidine sulfonyl fluoride ***152*** *is unstable on silica and degradation to the corresponding azetidinol is observedThe same degradation is observed when using Brockman grade I aluminium oxide as the stationary phase. Recrystallisation in CH_2Cl_2/pentane or Et_2O/pentane at −78 °C causes degradation to the corresponding azetidine fluoride with a distinctive shift* of approximately *–150.0 ppm in the ^{19}F NMR spectrum (377 MHz, $CDCl_3$)*

Cyclobutane Sulfonyl Fluorides (16 and 17)

Cyclobutane Sulfonyl Fluoride 16

1-Phenylcyclobutan-1-ol (16a)

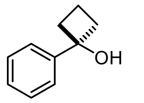

n-BuLi (1.55 M in hexanes, 16.7 mL, 26.0 mmol, 1.3 equiv) was added dropwise over 5 min to a solution of bromobenzene (2.72 mL, 26.0 mmol, 1.3 equiv) in anhydrous THF (94 mL) at –78 °C in a 100 mL round-bottom flask. The reaction mixture was stirred at –78 °C for 10 min. Cyclobutanone (1.49 mL, 20.0 mmol, 1.0 equiv) was added dropwise as a solution in THF (24 mL) to the reaction mixture. After a further 10 min of stirring at –78 °C the reaction mixture was warmed up slowly to 25 °C over ~1 h and quenched with water (100 mL). The aqueous layer was extracted with Et_2O (3 × 100 mL). The organic extracts were combined, washed with brine (100 mL), dried over Na_2SO_4, filtered and concentrated *in vacuo* using a rotary evaporator. Purification by flash column chromatography (20% EtOAc/*n*-hexane) afforded cyclobutanol **16a** as a white solid (2.71 g, 92%). R_f = 0.30 (25% EtOAc/*n*-hexane); mp = 40 °C; IR (film)/cm^{-1} 3303 (br O-H st), 2989, 2940, 2869, 1495, 1446, 1423, 1246, 1185, 1132, 1110, 1074, 1026, 959, 913, 825, 760, 697; ^{1}H NMR (400 MHz, $CDCl_3$) δ 7.54–7.48 (m, 2 H, 2 × Ar-CH), 7.42–7.35 (m, 2 H, 2 × Ar-CH), 7.32–7.28 (m, 1 H, Ar-CH), 2.63–2.52 (m, 2 H, 2 × C_q-C*H*H), 2.43–2.32 (m, 2 H, 2 × C_qCH*H*), 2.09–1.96 (m, 1 H, $C_q$$CH_2$C*H*H), 1.79 (br s, 1 H, OH), 1.68 (dt, *J* = 17.7, 8.8 Hz, 1 H, $C_q$$CH_2$CH*H*). The observed characterization data (^{1}H, R_f) were consistent with that previously reported.[12]

Methyl 3-((1-phenylcyclobutyl)thio)propanoate (16b)

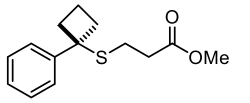

Lithium bis(trifluoromethanesulfonimide) (380 mg, 1.32 mmol, 11 mol%) and tetrabutylammonium hexafluorophosphate (256 mg, 0.66 mmol, 5.5 mol%) were added sequentially to a solution of cyclobutanol **16a** (2.16 g, 12.0 mmol, 1.0 equiv) and methyl 3-mercaptopropionate (2.6 mL, 24.0 mmol, 2.0 equiv) in non-anhydrous $CHCl_3$ (25 mL, 0.5 M). The reaction flask was placed into a pre-heated oil bath at 65 °C. The reaction was stirred for 30 min and then quenched with sat. aq. $NaHCO_3$ (25 mL). The aqueous layer was extracted with CH_2Cl_2 (3 × 25 mL). The combined organic layers were washed with aq. NaOH (1 M, 35 mL), dried over Na_2SO_4, filtered and concentrated *in vacuo* using a rotary evaporator. Purification by flash column chromatography (15% Et_2O/pentane) afforded cyclobutane sulfide **16b** as a white solid (2.19 g, 73%). R_f = 0.50 (15% Et_2O/pentane); mp = 42–43 °C; IR (film)/cm^{-1} 2960, 2921, 1733 (C=O st), 1438, 1358, 1244, 1190, 1167, 779, 743, 705; ^{1}H NMR (400 MHz, $CDCl_3$) δ 7.36–7.28 (m, 2 H, 2 × Ar-CH), 7.25–7.17 (m, 3 H, 3 × Ar-CH), 3.63 (s, 3 H, CO_2CH_3), 2.72–2.59 (m, 2 H, C*H*HC_qC*H*H), 2.49 (t, *J* = 7.7 Hz, 2 H, SCH_2), 2.45–2.32 (m, 3 H, CH*H*C_qCH*H* + CH_2C*H*HCH_2), 2.27 (t, *J* = 7.6 Hz, 2 H, C*H*$_2$CO2CH$_3$), 1.93–1.83 (m, 1 H, CH_2CH*H*CH_2); ^{13}C NMR (101 MHz, $CDCl_3$) δ 172.4 (C=O), 147.3 (Ph-*C*$_q$-C$_q$), 128.1 (2 × Ph-CH), 126.2 (Ph-CH) 125.8 (2 × Ph-CH), 53.1 (C_q), 51.7 (CO_2*C*H$_3$), 34.9 (2 × C_q*C*H$_2$), 34.0 (*C*H$_2$$CO_2CH_3$), 24.9 ($SCH_2$), 16.7 ($CH_2$*C*H$_2$$CH_3$); HRMS (FTMS–NSI$^+$) *m/z* calcd for $C_{14}H_{22}NSO_2^+$ $[M+NH_4]^+$: 268.1366, found: 268.1368.

Methyl 3-((1-phenylcyclobutyl)sulfonyl)propanoate (16c)

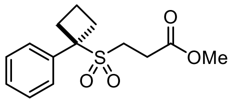

*m*CPBA (≤77%, 5.87 g, 26.3 mmol, 3.0 equiv) was added to a solution of cyclobutane sulfide **16b** (2.19 g, 8.76 mmol, 1.0 equiv) in CH_2Cl_2 (440 mL, 0.05 M) in a 1 L round-bottom flask. The reaction mixture was stirred at 25 °C for 3 h then aq. KOH (3 M, 150 mL) was added and the phases were separated. The aqueous portion was extracted with CH_2Cl_2 (4 × 100 mL). The organic extracts were combined, dried over Na_2SO_4, filtered and concentrated *in vacuo* using a rotatory evaporator to afford sulfone **16c** a white solid (2.48 g, quant.). R_f = 0.15 (10% Et_2O/pentane); mp = 67–71 °C; IR (film)/cm^{-1} 2958, 1743 (C=O st), 1300 (SO_2 st as), 1270, 1242, 1193 (SO_2 st sy), 1176, 1137, 1094, 793, 702; ^{1}H NMR (400 MHz, $CDCl_3$) δ 7.49–7.32 (m, 5 H, 5 × Ar-CH), 3.67 (s, 3 H, CO_2CH_3), 3.23–3.11 (m, 2 H, C*H*HC_qC*H*H), 2.92 (t, *J* = 7.6 Hz, 2 H, SCH_2), 2.76–2.64 (m, 2 H, CH*H*C_qCH*H*), 2.59 (t, *J* = 7.8 Hz, 2 H, $CH_2CO_2CH_3$), 2.38–2.24 (m, 1 H, CH_2C*H*HCH_2), 2.08–1.92 (m, 1 H, CH_2CH*H*CH_2); ^{13}C NMR (101 MHz, $CDCl_3$) δ 171.0 (C=O), 137.5 (Ph-*C*$_q$-C_q), 128.6 (2 × Ph-CH), 128.5 (Ph-CH), 128.3 (2 × Ph-CH), 68.3 (C_q), 52.2 (CO_2CH_3), 41.9 (SO_2CH_2), 29.4 (2 × C_qCH_2), 25.7 ($CH_2CO_2CH_3$), 15.9 (CH_2*C*H$_2CH_2$); HRMS (TOF-MS-ASAP$^+$) *m/z* calcd for $C_{14}H_{22}NSO_4^+$ $[M+NH_4]^+$: 300.1270, found: 300.1267.

Sodium 1-phenylcyclobutane-1-sulfinate (16d)

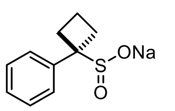

Sulfone **16c** (705 mg, 2.5 mmol, 1.0 equiv) was added to a solution of NaH (60% in mineral oil, 105 mg, 2.63 mmol, 1.05 equiv) in anhydrous THF (15 mL) in a 50 mL round-bottom flask. The reaction mixture was stirred at 25 °C for 1 h then quenched with anhydrous MeOH (15 mL) and concentrated *in vacuo* using a rotary evaporator to afford sulfinate salt **16d** as a white solid (523 mg, 96%). R_f = 0.17 (20% MeOH/CH_2Cl_2); mp = 210–212 °C; IR (film)/cm^{-1} 3294, 2946, 1682, 1598, 1445, 1206, 1034, 1019, 982, 772, 697; ^{1}H NMR (400 MHz, D_2O) δ 7.35–7.30 (m, 2 H, 2 × Ar-CH), 7.25–7.18 (m, 1 H, Ar-CH), 7.14–7.08 (m, 2 H, 2 × Ar-CH), 2.61–2.51 (m, 2 H, C*H*HC_qC*H*H), 2.38–2.25 (m, 2 H, CH*H*C_qCH*H*), 1.92–1.75 (m, 2 H, CH_2C*H*$_2CH_2$); ^{13}C NMR (101 MHz, D_2O) δ 140.5 (Ph-*C*$_q$-C_q), 127.9 (2 × Ph-CH), 127.5 (2 × Ph-CH), 126.6 (Ph-CH), 68.3 (C_q), 27.1 (2 × C_q-*C*H$_2$), 15.2 ($CH_2CH_2CH_2$); HRMS (FTMS–NSI$^+$) *m/z* calcd for $C_{10}H_{12}SO_2Na^+$ $[M+H]^+$: 219.0450, found: 219.0451.

1-Phenylcyclobutane-1-sulfonyl fluoride (16)

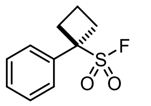

Selectfluor™ (159 mg, 0.45 mmol, 1.5 equiv) was added to a solution of sulfinate salt **16d** (66 mg, 0.3 mmol, 1.0 equiv) in anhydrous MeOH (1.5 mL, 0.2 M) at 0 °C in a 10 mL round-bottom flask. The reaction mixture was stirred at 0 °C for 1 h then concentrated *in vacuo* using a rotary evaporator. The residue was diluted with CH_2Cl_2 (10 mL) and filtered through a short plug of silica, eluting with further CH_2Cl_2 (20 mL). Concentration of the filtrate *in vacuo* using a rotary evaporator afforded cyclobutane sulfonyl fluoride **16** (54 mg, 82%). R_f = 0.78 (100% CH_2Cl_2); mp = 60–62 °C; IR (film)/cm^{-1} 1386 (SO_2 st as), 1192 (SO_2 st sy), 989, 947, 789, 772, 742, 695; ^{1}H NMR (400 MHz, $CDCl_3$) δ 7.49–7.38 (m, 5 H, 5 × Ph-CH), 3.29–3.20 (m, 2 H, C*H*HC_qC*H*H), 2.99–2.87 (m, 2 H, CH*H*C_qCH*H*), 2.55–2.39 (m, 1 H, CH_2C*H*HCH_2), 2.13–2.00 (m, 1 H, CH_2CH*H*CH_2); ^{13}C NMR (101 MHz, $CDCl_3$) δ 135.6 (Ar-*C*$_q$-C_q), 129.1 (Ph-CH), 128.5 (2 × Ph-CH), 128.3 (2 × Ph-CH), 68.3 (d, $^2J_{C\text{-}F}$ = 12.3 Hz, C_q), 31.2 (2 × C_q*C*H$_2$), 15.8 (CH_2*C*H$_2CH_2$); ^{19}F{^{1}H} NMR (377 MHz, $CDCl_3$) δ 32.4; HRMS (FTMS–APCI$^+$) *m/z* calcd for $C_{10}H_{11}^+$ $[M-SO_2F]^+$: 131.0856; found: 131.0855.

Cyclobutane Sulfonyl Fluoride 17

1-(4-Bromophenyl)cyclobutan-1-ol (17a)

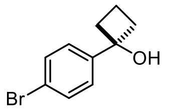

n-BuLi (1.55 M in hexanes, 17.1 mL, 26.0 mmol, 1.3 equiv) was added dropwise over 5 min to a solution of 1-bromo-4-iodobenzene (7.36 g, 26.0 mmol, 1.3 equiv) in anhydrous THF (118 mL) at –78 °C in a 100 mL round-bottom flask. The reaction mixture was stirred at –78 °C for 1 h. Cyclobutanone (1.49 mL, 20.0 mmol, 1.0 equiv) was added dropwise to the reaction mixture. After a further 30 min of stirring at –78 °C the reaction mixture was warmed up slowly to 25 °C over ~2 h and quenched with water (100 mL). The aqueous layer was extracted with Et_2O (3 × 100 mL). The organic extracts were combined, washed with brine (100 mL), dried over Na_2SO_4, filtered and concentrated *in vacuo* using a rotary evaporator. Purification by flash column chromatography (20% Et_2O/pentane) afforded cyclobutanol **17a** as a white solid (4.39 g, 98%). R_f = 0.15 (20% Et_2O/pentane); ^1H NMR (400 MHz, $CDCl_3$) δ 7.49 (d, *J* = 8.4 Hz, 2 H, 2 × Ar-CH), 7.37 (d, *J* = 8.4 Hz, 2 H, 2 × Ar-CH), 2.57–2.46 (m, 2 H, 2 × C_qCHH), 2.42–2.30 (m, 2 H, 2 × C_qCHH), 2.12–1.95 (m, 2 H, C_qCH_2CHH + br s, OH), 1.75–1.63 (m, 1 H, C_qCH_2CHH). The observed characterization data (^1H) were consistent with that previously reported.[21]

Methyl 3-((1-(4-bromophenyl)cyclobutyl)thio)propanoate (17b)

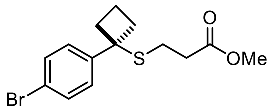

Lithium bis(trifluoromethanesulfonimide) (380 mg, 1.32 mmol, 11 mol%) and tetrabutylammonium hexafluorophosphate (256 mg, 0.66 mmol, 5.5 mol%) were added sequentially to a solution of cyclobutanol **17a** (2.70 g, 12.0 mmol, 1.0 equiv) and methyl 3-mercaptopropionate (2.6 mL, 24.0 mmol, 2.0 equiv) in non-anhydrous $CHCl_3$ (25 mL, 0.5 M). The reaction flask was placed into a preheated oil bath at 65 °C. The reaction was stirred for 25 min and then quenched with sat. aq. $NaHCO_3$ (25 mL). The aqueous layer was extracted with CH_2Cl_2 (3 × 25 mL). The organic layers were washed with aq. NaOH (1 M, 35 mL), dried over Na_2SO_4, filtered and concentrated *in vacuo* using a rotary evaporator. Purification by flash column chromatography (10% Et_2O/pentane) afforded cyclobutane sulfide **17b** as a white solid (3.51 g, 89%). R_f = 0.18 (10% Et_2O/pentane); mp = 45– 46 °C; IR (film)/cm^{-1} 2903, 2949, 1736 (C=O st), 1484, 1434, 1353, 1241, 1170, 1007, 825, 522; ^1H NMR (400 MHz, $CDCl_3$) δ 7.43 (d, *J* = 8.1 Hz, 2 H, 2 × Ar-CH), 7.11 (d, *J* = 8.1 Hz, 2 H, 2 × Ar-CH), 3.64 (s, 3 H, CO_2CH_3), 2.65–2.54 (m, 2 H, C*H*HC_qC*H*H), 2.47 (t, *J* = 7.5 Hz, 2 H, SCH_2), 2.43–2.28 (m, 5 H, CH*H*C_qCH*H* + CH_2C*H*HCH_2 + C*H*$_2CO_2CH_3$), 1.93–1.83 (m, 1 H, CH_2CH*H*CH_2); ^{13}C NMR (101 MHz, $CDCl_3$) δ 172.3 (C=O), 146.5 (Ar-*C*$_q$-C_q), 131.2 (2 × Ar-CH), 127.7 (2 × Ar-CH), 120.1 (Ar-C_qBr), 52.6 (CO_2CH_3), 51.8 (C_q), 34.8 (*C*H$_2CH_2$*C*H$_2$), 33.9 (*C*H$_2CO_2CH_3$), 24.9 (SCH_2), 16.6 (CH_2*C*H$_2CH_2$); HRMS (FTMS–APCI$^+$) *m/z* calcd for $C_{14}H_{18}BrO_2S^+$ $[M+H]^+$: 329.0205, found: 329.0190.

Methyl 3-((1-(4-bromophenyl)cyclobutyl)sulfonyl)propanoate (17c)

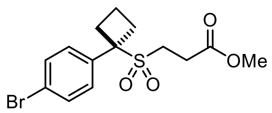

Washed *m*CPBA (5.18 g, 30 mmol, 3.0 equiv) was added to a solution of cyclobutane sulfide **17b** (3.28 g, 10 mmol, 1.0 equiv) in CH_2Cl_2 (500 mL, 0.05 M) in a 1 L round-bottom flask. The reaction mixture was stirred at 25 °C for 3 h then aq. KOH (3 M, 100 mL) was added and the phases were separated. The aqueous portion was extracted with CH_2Cl_2 (4 × 100 mL). The organic extracts were combined, dried over Na_2SO_4, filtered and concentrated *in vacuo* using a rotatory evaporator to afford sulfone **17c** a pale-yellow solid (3.65 g, quant.). R_f = 0.29 (50% Et_2O/pentane); mp = 86–88 °C; IR (film)/cm^{-1} 3000, 2950, 1745 (C=O st), 1485, 1428, 1305 (SO_2 st as), 1272, 1246, 1170, 1136 (SO_2 st sy), 1095, 1008, 826, 781, 705; ^1H NMR (400 MHz, $CDCl_3$) δ 7.58–7.51 (m, 2 H, 2 × Ar-CH), 7.35–7.29 (m, 2 H, 2 × Ar-CH), 3.68 (s, 3 H, CO_2CH_3), 3.22–3.10 (m, 2 H, C*H*HC_qC*H*H), 2.91 (t, *J* = 7.8 Hz, 2 H, SCH_2), 2.69–2.59 (m, 4 H, CH*H*C_qCH*H* + C*H*$_2CO_2CH_3$), 2.40–2.24 (m, 1 H, CH_2C*H*HCH_2), 2.08–1.92 (m, 1 H, CH_2CH*H*CH_2); ^{13}C NMR (101 MHz, $CDCl_3$) δ 171.0 (C=O), 136.7 (Ar-*C*$_q$-C_q), 131.8 (2 × Ar-CH), 130.1 (2 × Ar-CH), 123.0 (Ar-C_q-Br), 67.9 (C_q), 52.4 (OCH_3), 41.7 (SCH_2), 29.5 (*C*H$_2CH_2$*C*H$_2$),

25.6 ($CH_2CO_2CH_3$), 15.9 ($CH_2CH_2CH_2$); HRMS (TOF-MS-ES$^+$) *m/z* calcd for $C_{14}H_{21}NO_4SBr^+$ $[M+NH_4]^+$: 378.0375, found: 378.0368.

1-(4-Bromophenyl)cyclobutane-1-sulfonyl fluoride (17)

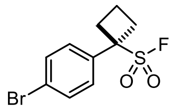

Prepared from sulfone **17c** and the sulfinate salt submitted intermediate directly to the fluorination conditions. Sodium methoxide (5.4 M solution in MeOH, 92 µL, 0.5 mmol, 1.0 equiv) was added dropwise to a solution of cyclobutane sulfone **17c** (180 mg, 0.5 mmol, 1.0 equiv) in anhydrous THF (1.0 mL, 0.5 M) in a 10 mL round bottom flask. The reaction mixture was stirred at 25 °C for 20 min, then concentrated *in vacuo* using a rotatory evaporator to afford the cyclobutane sulfinate salt as a yellow solid, which was subjected directly to the next step without spectroscopical analysis. For the fluorination step, Selectfluor^{TM} (266 mg, 0.75 mmol, 1.5 equiv) was added to anhydrous MeOH (2.5 mL, 0.2 M) at 0 °C in a 10 mL round bottom flask, then stirred for 5 min. The sulfinate salt was added portionwise and the sulfinate flask was rinsed with ice-cold Selectfluor^{TM}/methanol solution and transferred to the reaction vessel. The reaction mixture was stirred for at 0 °C for 1 h, then concentrated *in vacuo* using a rotatory evaporator. The residue was diluted with CH_2Cl_2 (10 mL) and filtered through a plug of Celite, eluting with further CH_2Cl_2 (50 mL). After concentration *in vacuo* using a rotatory evaporator, purification by flash column chromatography through a very short column (CH_2Cl_2) afforded cyclobutane sulfonyl fluoride **17** as a white solid (89.4 mg, 61%). R_f = 0.70 (50% CH_2Cl_2/pentane); mp = 62–64 °C; IR (film)/cm^{-1} 2983, 2944, 1486, 1382 (SO_2 st as), 1190 (SO_2 st sy), 1106, 1009, 832, 783, 747, 664, 572; ^{1}H NMR (400 MHz, $CDCl_3$) δ 7.57 (d, J = 8.6 Hz, 2 H, 2 × Ar-CH), 7.29 (d, J = 8.6 Hz, 2 H, 2 × Ar-CH), 3.29–3.15 (m, 2 H, C*H*HC$_q$C*H*H), 2.96–2.79 (m, 2 H, CH*H*C$_q$CH*H*), 2.54–2.37 (m, 1 H, CH_2C*H*HCH_2), 2.13–1.98 (m, 1 H, CH_2CH*H*CH_2); ^{13}C NMR (101 MHz, $CDCl_3$) δ 134.7 (Ar-*C*$_q$-Cq), 131.9 (2 × Ar-CH), 130.0 (2 × Ar-CH), 123.7 (Ar-C$_q$-Br), 67.8 (d, $^2J_{C\text{-}F}$ = 12.5 Hz, C$_q$), 31.2 ($CH_2CH_2CH_2$), 15.8 ($CH_2CH_2CH_2$); ^{19}F{^{1}H} NMR (377 MHz, $CDCl_3$) δ 32.7; HRMS (FTMS–APCI$^+$) *m/z* calcd for $C_{11}H_{11}BrFO_2^-$ $[M–SO_2+HCOO]^-$: 274.9911, found: 274.9554.

Oxetane analogues of bioactive molecules (18–24, 34, 36, 38, 92, 141)

Synthesis of an oxetane analogue of CDN1163[22]

N-(3-(4-Isopropoxyphenyl)oxetan-3-yl)-2-methylquinolin-8-amine (18)

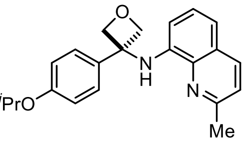

Prepared according to **deFS General Procedure A** under Ar at 60 °C for 2 h with an acidic work-up (see below) and using oxetane sulfonyl fluoride **3** (54.9 mg, 0.2 mmol, 1.0 equiv), K_2CO_3 (35.9 mg, 0.26 mmol, 1.3 equiv), 2-methylquinolin-8-amine (38.0 mg, 0.24 mmol, 1.2 equiv) and anhydrous MeCN (0.67 mL, 0.3 M). Work-up: the reaction mixture was diluted with EtOAc (20 mL) and washed with dilute HCl (0.5 M, 10 mL) to remove the residual amine. The organic layer was dried over Na_2SO_4, filtered and concentrated *in vacuo* using a rotary evaporator. Purification by flash column chromatography (10% EtOAc/pentane) afforded amino-oxetane **18** as an off-white solid (37.6 mg, 54%). R_f = 0.21 (10% EtOAc/pentane); mp = 95–97 °C; IR (film)/cm^{-1} 3383 (NH st), 2974, 2874, 1606, 1571, 1508, 1381, 1245, 1185, 1122, 829; ^{1}H NMR (400 MHz, $CDCl_3$) δ 7.97 (d, *J* = 8.4 Hz, 1 H, Ar-CH), 7.64–7.56 (m, 2 H, 2 × Ar-CH), 7.30 (d, *J* = 8.4 Hz, 1 H, Ar-CH), 7.11–6.99 (m, 3 H, 3 × Ar-CH), 6.90–6.84 (m, 2 H, 2 × Ar-CH), 5.87 (dd, *J* = 7.4, 1.4 Hz, 1 H, Ar-CH), 5.09 (d, *J* = 6.2 Hz, 2 H, C*H*HOC*H*H), 5.04 (d, *J* = 6.2 Hz, 2 H, CH*H*OCH*H*), 4.54 (p, *J* = 6.1 Hz, 1 H, C*H*(CH_3)$_2$), 2.77 (s, 3 H, Ar-CH_3), 1.33 (d, *J* = 6.1 Hz, 6 H, CH(CH_3)$_2$); ^{13}C NMR (126 MHz, $CDCl_3$) δ 157.2 (Ar-*C*$_q$-CH_3) 156.1 (Ar-*C*$_q$-OCH(CH_3)$_2$), 140.7 (Ar-C$_q$-NH), 137.7 (Ar-*C*$_q$-C$_q$), 136.2 (Ar-CH), 133.5 (Ar-C$_q$), 126.7 (2 × Ar-CH + Ar-C$_q$) 126.1 (Ar-CH), 122.3 (Ar-CH), 115.7 (2 × Ar-CH), 114.8 (Ar-CH), 107.3 (Ar-CH), 84.5 (CH_2OCH_2), 69.8 (O*C*H(CH_3)$_2$), 59.6 (C$_q$), 25.2 (Ar-CH_3), 22.1 (OCH(*C*H_3)$_2$); HRMS (TOF-MS-ES$^+$) *m/z* calcd for $C_{22}H_{25}N_2O_2^+$ [M+H]$^+$: 349.1916, found: 349.1915.

Synthesis of an oxetane analogue of MAPK14 inhibitor[23]

3-(4-Phenoxyphenyl)-N-(pyridin-2-ylmethyl)oxetan-3-amine (19)

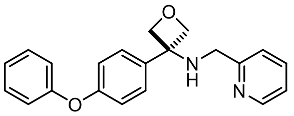

Prepared according to **deFS General Procedure A** under Ar at 60 °C for 5 h with a Celite work-up and using oxetane sulfonyl fluoride **10** (61.7 mg, 0.2 mmol, 1.0 equiv), K_2CO_3 (35.9 mg, 0.24 mmol, 1.3 equiv), 2-picolylamine (24.7 mL, 0.24 mmol, 1.2 equiv) and anhydrous MeCN (0.67 mL, 0.3 M). Purification by flash column chromatography (0–10% MeOH/CH_2Cl_2) afforded amino-oxetane **19** as an orange oil (38.8 mg, 58%). R_f = 0.18 (5% MeOH/CH_2Cl_2). IR (film)/cm^{-1} 3058, 2950, 2868, 1590, 1508, 1489, 1433, 1237, 1169; ^{1}H NMR (400 MHz, $CDCl_3$) δ 8.56 (d, *J* = 4.9 Hz, 1 H, Ar$_{(py)}$-CH), 7.62 (td, *J* = 7.6, 1.8 Hz, 1 H, Ar$_{(py)}$-CH), 7.46–7.40 (m, 2 H, 2 × Ar-CH), 7.35 (t, *J* = 7.7 Hz, 2 H, 2 × Ar-CH), 7.24 (d, *J* = 7.8 Hz, 1 H, Ar$_{(py)}$-CH), 7.17 (dd, *J* = 7.5, 5.0 Hz, 1 H, 1 × Ar-CH), 7.12 (t, *J* = 7.4 Hz, 1 H, Ar$_{(py)}$-CH), 7.04 (d, *J* = 8.2 Hz, 4 H, 4 × Ar-H), 4.98 (d, *J* = 6.4 Hz, 2 H, C*H*HOC*H*H), 4.83 (d, *J* = 6.4 Hz, 2 H, CH*H*OCH*H*), 3.74 (s, 2 H, NHC*H*$_2$), 2.75 (s, 1 H, NH); ^{13}C NMR (101 MHz, $CDCl_3$) δ 159.0 (Ar$_{(py)}$-C$_q$), 156.9 (Ar$_{(Ph)}$-C$_q$-O), 156.5 (Ar-C$_q$-O), 149.2 (Ar$_{(py)}$-CH), 136.8 (Ar-*C*$_q$-C$_q$), 136.4 (Ar$_{(py)}$-CH), 129.7 (2 × Ar-CH), 127.7 (2 × Ar-CH), 123.4 (Ar-CH), 122.3 (Ar$_{(py)}$-CH), 122.0 (Ar$_{(py)}$-CH), 119.0 (2 × Ar-CH), 118.7 (2 × Ar-CH), 82.5 (CH_2OCH_2), 63.1 (C$_q$), 49.1 (NHCH_2); HRMS (TOF-MS-ES$^+$) *m/z* calcd for $C_{21}H_{21}N_2O_2^+$ [M+H]$^+$: 333.1603; found: 333.1606.

Synthesis of an oxetane containing of a-helix mimetic analogue[24]

Methyl 3-isopropoxy-4-(3-isopropoxy-4-((3-(3-isopropoxy-4-methoxyphenyl)oxetan-3-yl)amino)benzamido)benzoate (20)

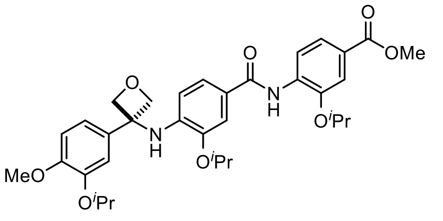

Prepared according to **deFS General Procedure A** under Ar at 60 °C for 2 h with a Celite work-up and using oxetane sulfonyl fluoride **5** (30.4 mg, 0.1 mmol, 1.0 equiv), K_2CO_3 (17.9 mg, 0.13 mmol, 1.3 equiv), aniline **S7** (46.4 mg, 0.12 mmol, 1.2 equiv) and anhydrous MeCN (0.34 mL, 0.3 M). Purification by flash column chromatography (30% EtOAc/pentane) afforded amino-oxetane **20** as a yellow solid (17.0 mg, 27%). R_f = 0.36 (30% EtOAc/pentane); IR (film)/cm^{-1} 3419 (br NH st), 2970, 2871, 1710 (C=O st), 1668 (C=O st), 1596, 1511, 1477, 1436, 1416, 1374, 1340, 1262, 1200, 1175, 1108, 1026, 983, 958, 911, 836, 808, 784, 763, 729, 643; ^{1}H NMR (400 MHz, $CDCl_3$) δ 8.69 (s, 1 H, CONH), 8.61 (d, *J* = 8.5 Hz, 1 H, C_qNHAr-C*H*Ar-CHAr-C_qCO_2Me), 7.69 (dd, *J* = 8.5, 1.8 Hz, 1 H, C_qNHAr-CHAr-C*H*Ar-C_qCO_2Me), 7.56 (d, *J* = 1.8 Hz, 1 H, $C_q{}^i$PrAr-CHAr-C_qCO_2Me), 7.52 (d, *J* = 1.9 Hz, 1 H, $C_q{}^i$PrAr-C*H*Ar-C_qCONH), 7.18–7.16 (m, 2 H, C_qNHAr-C*H*Ar-CHAr-C_qCONH + $C_q{}^i$PrAr-CHAr-C_qAr), 7.07 (dd, *J* = 8.3, 1.9 Hz, 1 H, C_qNHAr-CHAr-C*H*Ar-C_qCONH), 6.93–6.87 (m, 1 H, C_qOMeAr-CHAr-C*H*Ar-C_qAr), 5.81 (d, *J* = 8.2 Hz, 1 H, C_qOMeAr-C*H*Ar-CHAr-C_qAr), 5.50 (s, 1 H, NH), 5.00 (d, *J* = 6.3 Hz, 2 H, C*H*HOC*H*H), 4.96 (d, *J* = 6.3 Hz, 2 H, CH*H*OCH*H*), 4.75 (dhept, *J* = 24.4, 6.1 Hz, 2 H, 2 × C*H*$(CH_3)_2$), 4.47 (hept, *J* = 6.1 Hz, 1 H, C*H*$(CH_3)_2$), 3.89 (s, 3 H, Ar-OCH_3), 3.85 (s, 3 H, CO_2CH_3), 1.47 (d, *J* = 6.0 Hz, 6 H, CH(C*H*$_3$)$_2$), 1.39 (d, *J* = 6.1 Hz, 6 H, CH(C*H*$_3$)$_2$), 1.29 (d, *J* = 6.1 Hz, 6 H, CH(C*H*$_3$)$_2$); ^{13}C NMR (101 MHz, $CDCl_3$) δ 166.9 (CO_2Me), 165.0 (CONH), 149.9 (C_qNHAr-*C*HAr-CHAr-C_qCO_2Me), 147.4 (C_qOMeAr-CHAr-*C*HAr-C_qAr), 145.6 (C_qNHAr-*C*HAr-CHAr-C_qCONH), 145.0 (C_qOMeAr-*C*HAr-CHAr-C_qAr), 139.2 ($C_q{}^i$PrAr-*C*HAr-C_qCONH), 133.6 (Ar-*C*$_q$$CO_2$Me), 133.5 (Ar-*C*$_q$-$C_q$), 124.4 ($C_q$NHAr-*Cq*^{i}PrAr), 123.4 (Ar-C_qOMe), 123.2 (C_qOMeAr-*C*$_q{}^i$PrAr), 119.4 ($C_q{}^i$PrAr-*C*HAr-C_qCO_2Me), 118.4 (Ar-C_qNH), 118.1 ($C_q{}^i$PrAr-*C*HAr-C_qAr), 113.4 (C_qNHAr-CHAr-*C*HAr-C_qO_2Me), 113.0 (C_qNHAr-*C*$_q{}^i$PrAr), 112.0 (C_qNHAr-CHAr-*C*HAr-C_qONH), 111.8 (Ar-*C*$_q$CONH), 110.8 (Ar-C_qNH), 84.3 (CH_2OCH_2), 71.7 (*C*H$(CH_3)_2$), 71.5 (*C*H$(CH_3)_2$), 71.3 (*C*H$(CH_3)_2$), 59.6 (C_q-NH), 56.0 (CO_2*C*H_3), 52.1 (Ar-OCH_3), 22.3 (CH(*C*H_3)$_2$), 22.2 (CH(*C*H_3)$_2$), 22.0 (CH(*C*H_3)$_2$); HRMS (TOF-MS-ES$^+$) *m/z* calcd for $C_{34}H_{43}N_2O_8^+$ $[M+H]^+$: 607.3019, found: 607.3011.

Notes:

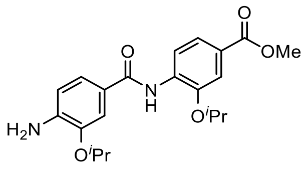

*Aniline **S7** (methyl 4-(4-amino-3-isopropoxybenzamido)-3-isopropoxybenzoate, above) prepared according to literature procedures[24,25] affording an orange solid (462 mg, 74% over 5 steps).*

Synthesis of an oxetane analogue of Fipexide (**S8**, **S9**, **21**)

tert-Butyl 4-(2-(4-chlorophenoxy)acetyl)piperazine-1-carboxylate (S8)

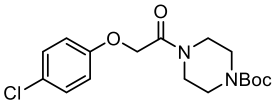

tert-Butyl piperazine-1-carboxylate (558 mg, 3.0 mmol, 1.0 equiv), 4-chlorophenoxyacetic acid (558 mg, 3.0 mmol, 1.0 equiv), *N*,*N'*-dicyclohexylcarbodiimide (618 mg, 3.0 mmol, 1.0 equiv) and 4-dimethylaminopyridine (363 mg, 3.0 mmol, 1.0 equiv) were added to a 100 mL round-bottom flask and dissolved in anhydrous CH_2Cl_2 (25 mL, 0.12 M). The reaction mixture was stirred at 25 °C for 24 h then concentrated *in vacuo* using a rotary evaporator. The resulting precipitate was filtered, washed

with further CH_2Cl_2 (3 × 25 mL) and the filtrate concentrated *in vacuo* using a rotary evaporator. Purification by flash column chromatography (20–50% EtOAc/pentane) afforded amide **S8** as a white solid (558 mg, 52%). R_f = 0.39 (50% EtOAc/*n*-hexane); mp = 107–109 °C; IR (film)/cm^{-1} 2973, 2928, 1675 (C=O st), 1655 (C=O st), 1489, 1459, 1422, 1362, 1234, 1217, 1167, 1012, 824, 643; ^{1}H NMR (400 MHz, $CDCl_3$) δ 7.26–7.22 (m, 2 H, 2 × Ar-CH), 6.90–6.85 (m, 2 H, 2 × Ar-CH), 4.68 (s, 2 H, OCH_2C=O), 3.62–3.50 (m, 4 H, CH_2NCH_2), 3.46–3.35 (m, 4 H, $CH_2N(Boc)CH_2$), 1.46 (s, 9 H, $C(CH_3)_3$); ^{13}C NMR (101 MHz, $CDCl_3$) δ 166.4 (N*C*$_q$=OCH_2), 156.3 (Ar-*C*$_q$-OCH_2), 154.5 (N*C*$O_2C(CH_3)_3$), 129.6 (2 × Ar-CH), 126.8 (Ar-C_q-Cl), 115.9 (2 × Ar-CH), 80.5 (*C*$(CH_3)_3$), 68.0 (O*C*H_2C=O), 45.3 (CH_2NCH_2), 42.0 ($CH_2N(Boc)CH_2$), 28.4 (C(*C*$H_3)_3$). The observed characterization data (^{1}H) were consistent with that previously reported.[26]

2-(4-Chlorophenoxy)-1-(piperazin-1-yl)ethan-1-one (S9)

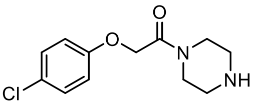

Trifluoroacetic acid (1.24 mL, 16.0 mmol, 16.0 equiv) was added dropwise to a solution of amide **S8** (354 mg, 1.0 mmol, 1.0 equiv) in anhydrous CH_2Cl_2 (50 mL, 0.02 M) at 25 °C in a 100 mL round-bottom flask. After stirring at 25 °C for 48 h the reaction mixture was concentrated *in vacuo* using a rotary evaporator. The resulting precipitate was diluted with aq. NaOH (1 M, 20 mL) and extracted with EtOAc (3 × 40 mL). The combined organic layers were dried over Na_2SO_4, filtered and concentrated *in vacuo* using a rotary evaporator to afford amine **S9** as a white paste (203 mg, 80%). R_f = 0.15 (2% MeOH/CH_2Cl_2); IR (film)/cm^{-1} 3314 (NH st), 2917, 1653 (C=O st), 1489, 1459, 1431, 1217, 1169, 1005, 822, 639; ^{1}H NMR (500 MHz, $CDCl_3$) δ 7.25–7.21 (m, 2 H, 2 × Ar-CH), 6.92–6.83 (m, 2 H, 2 × Ar-CH), 4.67 (s, 2 H, OCH_2C=O), 3.63–3.58 (m, 2 H, C*H*HN(C=O)C*H*H), 3.57–3.52 (m, 2 H, CH*H*N(C=O)CH*H*), 2.88–2.82 (m, 4 H, C*H*$_2$NHC*H*$_2$), 2.52–2.41 (m, 1 H, NH); ^{13}C NMR (126 MHz, $CDCl_3$) δ 166.1 (N*C*$_q$=OCH_2), 156.5 (Ar-*C*$_q$-OCH_2), 129.5 (2 × Ar-CH), 126.7 (Ar-C_q-Cl), 116.0 (2 × Ar-CH), 67.9 (O*C*H_2C=O), 46.5 (*C*$H_2N(C{=}O)CH_2$), 46.3 ($CH_2N(C{=}O)$*C*H_2), 45.8 (*C*H_2NHCH_2), 43.1 (CH_2NH*C*H_2). The observed characterization data (^{1}H) were consistent with that previously reported.[26]

1-(4-(3-(Benzo[*d*][1,3]dioxol-5-yl)oxetan-3-yl)piperazin-1-yl)-2-(4-chlorophenoxy)ethan-1-one (21)

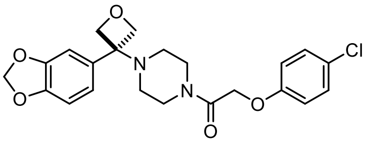

Prepared according to **deFS General Procedure A** under Ar at 60 °C for 4 h with a Celite work-up and using oxetane sulfonyl fluoride **7** (39 mg, 0.15 mmol, 1.0 equiv), K_2CO_3 (27 mg, 0.195 mmol, 1.3 equiv), amine **S9** (45.8 mg, 0.18 mmol, 1.2 equiv) and anhydrous MeCN (0.5 mL, 0.3 M). Purification by flash column chromatography (80–100% EtOAc/pentane) afforded amino-oxetane **21** as a white solid (55.3 mg, 86%). R_f = 0.17 (80% EtOAc/pentane); mp = 174–177 °C; IR (film)/cm^{-1} 2948, 1675 (C=O st), 1491, 1435, 1241, 1223, 1036, 992, 932, 814; ^{1}H NMR (400 MHz, $CDCl_3$) δ 7.19 (d, *J* = 8.9 Hz, 2 H, 2 × Ar-CH), 6.84–6.76 (m, 3 H, 3 × Ar-CH), 6.45 (d, *J* = 8.1 Hz, 2 H, 2 × Ar-CH), 6.00 (s, 2 H, OCH_2O), 4.89–4.80 (m, 4 H, CH_2OCH_2), 4.60 (s, 2 H, OCH_2C=O), 3.63 (br d, *J* = 19.1 Hz, 4 H, $CH_2N(C{=}O)CH_2$), 2.26 (s, 4 H, $CH_2N(C_q)CH_2$); ^{13}C NMR (126 MHz, $CDCl_3$) δ 165.9 (C_q=O), 156.3 (Ar-*C*$_q$-OCH_2C=O), 147.6 (Ar-*C*$_q$-OCH_2O), 147.0 (Ar-*C*$_q$-OCH_2O), 130.5 (Ar-*C*$_q$-C_q), 129.5 (2 × Ar-CH), 126.6 (Ar-C_q-Cl), 120.5 (Ar-CH), 115.9 (2 × Ar-CH), 107.7 (Ar-CH), 107.5 (Ar-CH), 101.3 (OCH_2O), 79.5 (CH_2OCH_2), 67.7 (O*C*H_2C=O), 66.7 (C_q), 46.4 (*C*$H_2N(C{=}O)CH_2$), 45.8 ($CH_2N(C{=}O)$*C*H_2), 45.6 (*C*$H_2N(C_q)CH_2$), 42.3 ($CH_2N(C_q)$*C*H_2); HRMS (TOF-MS-ES$^+$) *m/z* calcd for $C_{22}H_{24}N_2O_5Cl^+$ [M+H]$^+$: 431.1374, found: 431.1346.

Synthesis of an oxetane analogue of Piribedil

2-(4-(3-(Benzo[*d*][1,3]dioxol-5-yl)oxetan-3-yl)piperazin-1-yl)pyrimidine (22)

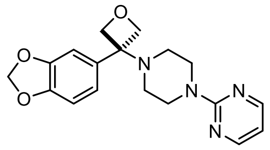

Prepared according to **deFS General Procedure A** under Ar at 60 °C for 4 h with a Celite work-up and using oxetane sulfonyl fluoride **7** (39.0 mg, 0.15 mmol, 1.0 equiv), K_2CO_3 (26.9 mg, 0.195 mmol, 1.3 equiv), 2-(piperazin-1-yl)pyrimidine (30.0 mg, 0.18 mmol, 1.2 equiv) and anhydrous MeCN (0.5 mL, 0.3 M). Purification by flash column chromatography (40–70% EtOAc/pentane) afforded amino-oxetane **22** as a white solid (40.5 mg, 79%). R_f = 0.20 (50% EtOAc/pentane); mp = 103–106 °C; IR (film)/cm^{-1} 2924, 2816, 1582, 1552, 1474, 1443, 1357, 1251, 1036, 977, 798; ^{1}H NMR (400 MHz, $CDCl_3$) δ 8.24 (d, *J* = 4.7 Hz, 2 H, 2 × $Ar_{(py)}$-CH), 6.78 (d, *J* = 7.9 Hz, 1 H, Ar-CH), 6.53–6.45 (m, 2 H, 2 × Ar-CH), 6.43 (t, *J* = 4.7 Hz, 1 H, $Ar_{(py)}$-CH), 5.95 (s, 2 H, OCH_2O), 4.89 (s, 4 H, CH_2OCH_2), 3.87 (t, *J* = 4.9 Hz, 4 H, $CH_2N_{(py)}CH_2$), 2.33 (t, *J* = 4.9 Hz, 4 H, $CH_2N(C_q)CH_2$); ^{13}C NMR (101 MHz, $CDCl_3$) δ 161.3 ($Ar_{(py)}$-C_q), 157.7 (2 × $Ar_{(py)}$-CH), 147.4 (Ar-C_q-OCH_2O), 146.8 (Ar-C_q-OCH_2O), 130.8 (Ar-C_q-C_q), 120.7 (Ar-CH), 109.8 ($Ar_{(py)}$-CH), 107.8 (Ar-CH), 107.6 (Ar-CH), 101.2 (OCH_2O), 79.8 (CH_2OCH_2), 66.8 (C_q), 46.0 ($CH_2N_{(py)}CH_2$), 43.7 ($CH_2N(C_q)CH_2$); HRMS (TOF-MS-ES$^+$) *m/z* calcd for $C_{18}H_{21}N_4O_3^+$ $[M+H]^+$: 341.1614, found: 341.1605.

Synthesis of an oxetane analogue of Fenoverine (**S10**, **S11**, **23**)

2-Chloro-1-(10*H*-phenothiazin-10-yl)ethan-1-one (S10)

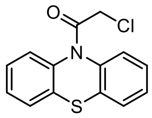

Prepared according to a literature procedure.[27] 2-Chloroacetyl chloride (0.57 mL, 7.5 mmol, 1.5 equiv) was added to a solution of phenothiazine (995 mg, 5.0 mmol, 1.0 equiv) in anhydrous toluene (17.0 mL, 0.3 M) at 0 °C in a 50 mL round-bottom flask. The reaction mixture was stirred at 80 °C for 12 h then cooled to room temperature before quenching with water (20 mL). The phases were separated and the aqueous layer extracted with CH_2Cl_2 (3 × 20 mL). The combined organic layers were dried over Na_2SO_4, filtered and concentrated *in vacuo* to afford amide **S10** as a lime-green solid (1.10 g, 80%). R_f = 0.80 (Et_2O); mp = 117–119 °C; IR (film)/cm^{-1} 1690 (C=O st), 1671, 1459, 1446, 1333, 1236, 1126, 1169, 759; ^{1}H NMR (400 MHz, $CDCl_3$) δ 7.59 (d, *J* = 7.9 Hz, 2 H, 2 × Ar-CH), 7.47 (dd, *J* = 7.7, 1.5 Hz, 2 H, 2 × Ar-CH), 7.36 (td, *J* = 7.7, 1.5 Hz, 2 H, 2 × Ar-CH), 7.27 (td, *J* = 7.6, 1.3 Hz, 2 H, 2 × Ar-CH), 4.19 (s, 2 H, CH_2); ^{13}C NMR (101 MHz, $CDCl_3$) δ 165.5 (C_q=O), 137.9 (2 × Ar-C_q-S), 133.2 (2 × Ar-C_q-N), 128.2 (2 × Ar-CH), 127.5 (2 × Ar-CH), 127.4 (2 × Ar-CH), 126.6 (2 × Ar-CH), 41.9 (CH_2). The observed characterization data (^{1}H, ^{13}C) were consistent with that previously reported.[27]

1-(10*H*-Phenothiazin-10-yl)-2-(piperazin-1-yl)ethan-1-one (S11)

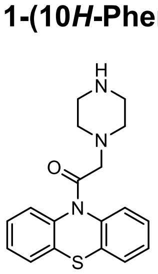

Prepared according to a literature procedure.[28] K_2CO_3 (345 mg, 2.5 mmol, 1.0 equiv) was added portionwise to a solution of amide **S10** (688 mg, 2.5 mmol, 1.0 equiv), KI (415 mg, 2.5 mmol, 1.0 equiv) and piperazine (1.08 g, 12.5 mmol, 5.0 equiv) in anhydrous DMF (35 mL, 0.07 M) at 25 °C in a 100 mL round-bottom flask. The reaction mixture was stirred at 25 °C for 16 h then concentrated *in vacuo* using a rotary evaporator. The resulting residue was diluted with water (50 mL) and extracted with EtOAc (3 × 50 mL). The combined organic layers were washed sequentially with water (50 mL) and brine (50 mL), dried over Na_2SO_4, filtered and concentrated *in vacuo* using a rotary evaporator to afford amine **S11** as a white solid (650 mg, 80%). R_f = 0.10 (10% MeOH/CH_2Cl_2); mp = 145–146 °C; ^{1}H NMR (400 MHz, $CDCl_3$) δ 7.54 (d, *J* = 8.0 Hz, 2 H, 2 × Ar-CH), 7.42 (d, *J* = 7.8 Hz, 2 H, 2 × Ar-CH), 7.29 (t, *J* = 7.7 Hz, 2 H, 2 × Ar-CH), 7.20 (t, *J* = 7.6 Hz, 2 H, 2 × Ar-CH), 3.27 (s, 2 H, $COCH_2$), 2.72 (t, *J* = 4.9 Hz, 4 H, CH_2NHCH_2), 2.35 (t, *J* = 4.8 Hz, 4 H, C*H*$_2$N(CH_2)C*H*$_2$).The observed characterization data (^{1}H, ^{13}C) were consistent with that previously reported.[28]

2-(4-(3-(Benzo[*d*][1,3]dioxol-5-yl)oxetan-3-yl)piperazin-1-yl)-1-(10*H*-phenothiazin-10-yl)ethan-1-one (23)

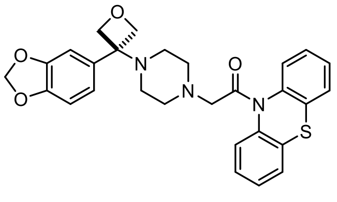

Prepared according to **deFS General Procedure A** under Ar at 60 °C for 4 h with a Celite work-up and using oxetane sulfonyl fluoride **7** (52.0 mg, 0.2 mmol, 1.0 equiv), K_2CO_3 (35.8 mg, 0.26 mmol, 1.3 equiv), amine **S11** (78.1 mg, 0.24 mmol, 1.2 equiv) and anhydrous MeCN (0.67 mL, 0.3 M). Purification by flash column chromatography (60–100% EtOAc/pentane) afforded amino-oxetane **23** as a colorless gum (53.6 mg, 54%). R_f = 0.14 (70% EtOAc/pentane); IR (film)/cm^{-1} 2939, 2821, 2359, 1675 (C=O st), 1487, 1459, 1310, 1249, 1036, 936, 755; ^{1}H NMR (400 MHz, $CDCl_3$) δ 7.48 (d, *J* = 7.9 Hz, 2 H, 2 × Ar-CH), 7.36–7.26 (m, 4 H, 4 × Ar-CH), 7.17 (t, *J* = 7.6 Hz, 2 H, 2 × Ar-CH), 6.83 (d, *J* = 7.9 Hz, 1 H, Ar-CH), 6.48 (d, *J* = 8.1 Hz, 2 H, 2 × Ar-CH), 6.00 (s, 2 H, OCH_2O), 4.85–4.71 (m, 4 H,CH_2OCH_2), 3.26 (s, 2 H, NCH_2C=O), 2.46 (s, 4 H, $CH_2N(C_q)CH_2$), 2.15 (s, 4 H, $CH_2N(CH_2)CH_2$); ^{13}C NMR (126 MHz, $CDCl_3$) δ 168.6 (C_q=O), 147.3 (Ar-C_q-O), 146.6 (Ar-C_q-O), 138.7 (2 × Ar-C_q-S), 132.9 (2 × Ar-C_q-N), 131.1 (Ar-C_q-C_q), 127.8 (2 × Ar-CH), 126.8 (2 × Ar-CH), 126.7 (2 × Ar-CH), 126.6 (2 × Ar-CH), 120.7 (Ar-CH), 107.9 (Ar-CH), 107.5 (Ar-CH), 101.1 (OCH_2O), 79.5 (CH_2OCH_2), 66.5 (C_q), 60.0 (NCH_2C_q=O), 52.7 ($CH_2N(C_q)CH_2$), 45.5 ($CH_2N(CH_2)CH_2$); HRMS (TOF-MS-ES$^+$) *m/z* calcd for $C_{28}H_{28}N_3O_4S^+$ [M+H]$^+$: 502.1801, found: 502.1812.

Synthesis of an oxetane analogue of Medibazine

1-Benzhydryl-4-(3-(benzo[*d*][1,3]dioxol-5-yl)oxetan-3-yl)piperazine (24)

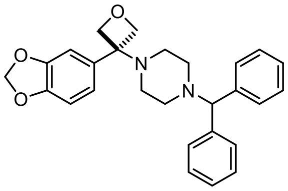

Prepared according to **deFS General Procedure A** under Ar at 60 °C for 4 h with a Celite work-up and using oxetane sulfonyl fluoride **7** (52.0 mg, 0.2 mmol, 1.0 equiv), K_2CO_3 (35.8 mg, 0.26 mmol, 1.3 equiv), 1-benzhydrylpiperazine (60.6 mg, 0.24 mmol, 1.2 equiv) and anhydrous MeCN (0.67 mL, 0.3 M). Purification by flash column chromatography (0–30% EtOAc/pentane) afforded a crude mixture of amino-oxetane **24** and oxetanol **7a**. The crude material was dissolved into Et_2O (20 mL) and washed with aq. KOH (3 M, 3 × 15 mL), dried over Na_2SO_4, filtered and concentrated *in vacuo* using a rotary evaporator to afford amino-oxetane **24** as a colorless gum (55.5 mg, 65%). R_f = 0.17 (50% Et_2O/pentane); IR (film)/cm^{-1} 2942, 2814, 1504, 1485, 1247, 1228, 1036, 908, 729, 706; ^{1}H NMR (400 MHz, $CDCl_3$) δ 7.38–7.32 (m, 4 H, 4 × Ar-CH), 7.24 (t, *J* = 7.5 Hz, 4 H, 4 × Ar-CH), 7.19–7.12 (m, 2 H, 2 × Ar-CH), 6.84 (d, *J* = 7.8 Hz, 1 H, Ar-CH), 6.54–6.46 (m, 2 H, 2 × Ar-CH), 6.01 (s, 2 H, OCH_2O), 4.87–4.76 (m, 4 H, CH_2OCH_2), 4.21 (s, 1 H, NCH), 2.70–2.07 (m, 8 H, $CH_2N(C_q)CH_2$ + $CH_2N(CH)CH_2$); ^{13}C NMR (101 MHz, $CDCl_3$) δ 147.4 (Ar-C_q-O), 146.7 (Ar-C_q-O), 142.6 (2 × Ar-C_q-CHN), 131.3 (Ar-C_q-C_q), 128.4 (4 × Ar-CH), 127.9 (4 × Ar-CH), 126.9 (2 × Ar-CH), 120.8 (Ar-CH), 107.9 (Ar-CH), 107.6 (Ar-CH), 101.2 (OCH_2O), 79.8 (CH_2OCH_2), 76.2 (NCH), 66.5 (C_q), 52.0 ($CH_2N(C_q)CH_2$), 46.0 ($CH_2N(CH)CH_2$); HRMS (TOF-MS-ES$^+$) *m/z* calcd for $C_{27}H_{29}N_2O_3^+$ [M+H]$^+$: 429.2178, found: 429.2169.

Notes:

*The R_f values of **7a** and **24** are identical, therefore the KOH wash was employed to deprotonate the alcohol of **7a** and wash it into the aqueous layer in the liquid-liquid extraction.*

Synthesis of an oxetane analogue of Moclobemide (**33**, **34a**, **34**)

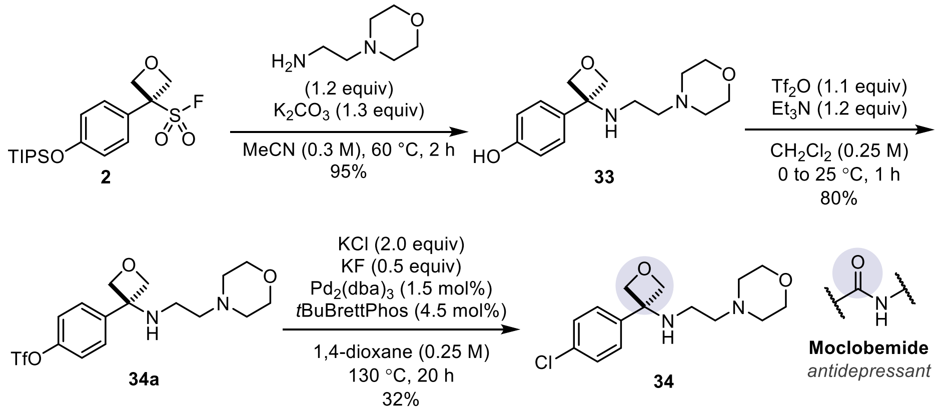

4-(3-((2-Morpholinoethyl)amino)oxetan-3-yl)phenol (33)

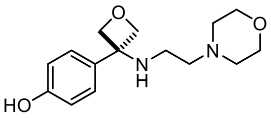

Prepared according to **deFS General Procedure A** under Ar at 60 °C for 2 h with a Celite work-up and using oxetane sulfonyl fluoride **2** (330 mg, 0.85 mmol, 1.0 equiv), K_2CO_3 (155 mg, 1.10 mmol, 1.3 equiv), 4-(2-aminoethyl)morpholine (0.14 mL, 1.02 mmol, 1.2 equiv) and anhydrous MeCN (2.8 mL, 0.3 M). Purification by flash column chromatography (0–25% MeOH/CH_2Cl_2) afforded amino-oxetane **33** as a clear, colorless gum (226 mg, 95%). R_f = 0.09 (5% MeOH/CH_2Cl_2); IR (film)/cm^{-1} 3250 (br O-H), 2947, 2863, 2811, 1612, 1591, 1515, 1450, 1353, 1267, 1246, 1143, 1113, 978, 912, 831, 728, 645, 547; ^{1}H NMR (400 MHz, $CDCl_3$) δ 7.10 (d, *J* = 8.4 Hz, 2 H, 2 × Ar-CH), 6.69 (d, *J* = 8.4 Hz, 2 H, 2 × Ar-CH), 4.97 (d, *J* = 6.4 Hz, 2 H, C*H*HOC*H*H), 4.73 (d, *J* = 6.4 Hz, 2 H, CH*H*OCH*H*), 3.71 (t, *J* = 4.7 Hz, 4 H, CH_2C*H*$_2$OC*H*$_2$$CH_2$), 3.50 (s, 1 H, OH), 2.56–2.46 (m, 4 H, C*H*$_2$C*H*$_2$N), 2.41 (t, *J* = 4.7 Hz, 4 H, CH_2NCH_2); ^{13}C NMR (101 MHz, $CDCl_3$) δ 155.9 (Ar-C_q-OH), 132.9 (Ar-*C*$_q$-C_q), 127.5 (2 × Ar-CH), 115.6 (2 × Ar-CH), 82.3 (CH_2OCH_2), 66.8 (CH_2*C*H_2O*C*H_2CH_2), 62.8 (C_q), 58.0 (N*C*H_2CH_2NH), 53.5 (*C*H_2N*C*H_2), 39.4 (NHCH_2); HRMS (TOF-MS-ES$^+$) *m/z* calcd for $C_{15}H_{23}N_2O_3$ [M+H]: 279.1709; found 279.1707.

4-(3-((2-Morpholinoethyl)amino)oxetan-3-yl)phenyl trifluoromethanesulfonate (34a)

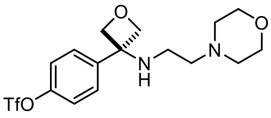

Triethylamine (0.11 mL, 0.78 mmol, 1.2 equiv) and triflic anhydride (0.12 mL, 0.71 mmol, 1.1 equiv) were added sequentially to a solution of amino-oxetane **33** (180 mg, 0.64 mmol, 1.0 equiv) in anhydrous CH_2Cl_2 (2.56 mL, 0.25 M) in a 10 mL round-bottom flask at 0 °C. After warming to 25 °C and stirring for 1 h, the reaction mixture was quenched with sat. aq. $NaHCO_3$ (20 mL). The phases were separated, and the aqueous layer was extracted with CH_2Cl_2 (3 × 25 mL). The combined organic layers were dried over anhydrous Na_2SO_4, filtered, and concentrated *in vacuo* using a rotary evaporator. Purification by flash column chromatography (0–7% MeOH/CH_2Cl_2) afforded oxetane triflate **34a** as a pale pink solid (210 mg, 80%). R_f = 0.30 (10% MeOH/CH_2Cl_2); mp = 68–70 °C; IR (film)/cm^{-1} 2951, 2870, 1500, 1421, 1247, 1209, 1137, 1117, 1029, 982, 887, 845, 756, 636, 610, 516; ^{1}H NMR (400 MHz, $CDCl_3$) δ 7.53 (d, *J* = 8.8 Hz, 2 H, 2 × Ar-CH), 7.30 (d, *J* = 8.8 Hz, 2 H, 2 × Ar-CH), 4.89 (d, *J* = 6.5 Hz, 2 H, C*H*HOC*H*H), 4.75 (d, *J* = 6.5 Hz, 2 H, CH*H*OCH*H*), 3.70 (t, *J* = 4.7 Hz, 4 H, CH_2C*H*$_2$OC*H*$_2$$CH_2$), 2.49–2.36 (m, 8 H, CH_2C*H*$_2$N + C*H*$_2$$CH_2$N + CH_2NCH_2); ^{13}C NMR (101 MHz, $CDCl_3$) δ 148.5 (Ar-C_q-OTf), 143.0 (Ar-*C*$_q$-C_q), 128.1 (2 × Ar-CH), 121.4 (2 × Ar-CH), 118.7 (q, $^1J_{C\text{-}F}$ = 306 Hz, CF_3), 82.3 (CH_2OCH_2), 66.9 (CH_2*C*H_2O*C*H_2CH_2), 62.7 (C_q), 58.1 (N*C*H_2CH_2NH), 53.4 (*C*H_2N*C*H_2), 39.5 (NHCH_2); ^{19}F{^{1}H} NMR (377 MHz, $CDCl_3$) δ –72.9; HRMS (TOF-MS-ES$^+$) *m/z* calcd for $C_{16}H_{22}N_2O_5SF_3^+$ [M+H]$^+$: 411.1202, found 411.1211.

3-(4-Chlorophenyl)-N-(2-morpholinoethyl)oxetan-3-amine (34)

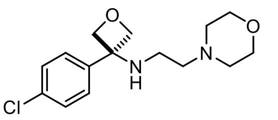

Using chlorination conditions reported by Buchwald,[20] oxetane triflate **34a** (41.0 mg, 0.1 mmol, 1.0 equiv), KCl (14.9 mg, 0.2 mmol, 2.0 equiv), and KF (2.9 mg, 0.05 mmol, 0.5 equiv) were added to a reaction vial (1). The reaction vial (1) was sealed then evacuated and backfilled with Ar three times. $Pd_2(dba)_3$ (1.4 mg, 1.5 µmol, 1.5 mol%) and ^{t}BuBrettPhos (2.2 mg, 4.5 µmol, 4.5 mol%) were added to a separate reaction vial (2). The reaction vial (2) was sealed then evacuated and backfilled with Ar three times. Anhydrous, degassed 1,4-dioxane (0.1 mL) was added to reaction vial (2) and the reaction mixture was stirred at 120 °C for 5 min. After cooling to 25 °C, the contents of reaction vial (2) were transferred to reaction vial (1) by syringe and the reaction mixture was diluted with further 1,4-dioxane (0.3 mL). The reaction mixture was then heated to 130 °C and stirred vigorously for 20 h. After cooling to 25 °C, the reaction mixture was filtered through a plug of Celite and eluted with Et_2O (10 mL). The filtrate was then concentrated *in vacuo* using a rotary evaporator. Purification by flash column chromatography (0–10% $MeOH/CH_2Cl_2$) afforded amino-oxetane **34** as a white crystalline solid (9.7 mg, 32%). R_f = 0.6 (30% $MeOH/CH_2Cl_2$); mp = 64–66 °C; IR (film)/cm^{-1} 3302, 2943, 2859, 2809, 1491, 1453, 1296, 1272, 1116, 1093, 983, 828; ^{1}H NMR (400 MHz, $CDCl_3$) δ 7.41–7.32 (m, 4 H, 4 × Ar-CH), 4.92 (d, *J* = 6.4 Hz, 2 H, C*H*HOC*H*H), 4.74 (d, *J* = 6.4 Hz, 2 H, CH*H*OH*H*), 3.72 (t, *J* = 4.7 Hz, 4 H, $CH_2CH_2OCH_2CH_2$), 2.46 (m, 4 H, CH_2CH_2N), 2.40 (m, 4 H, CH_2NCH_2); ^{13}C NMR (101 MHz, $CDCl_3$) δ 140.9 (Ar-C_q-C_q), 133.2 (Ar-C_q-Cl), 128.7 (2 × Ar-CH), 127.6 (2 × Ar-CH), 82.4 (CH_2OCH_2), 66.9 ($CH_2CH_2OCH_2CH_2$), 62.8 (C_q), 58.2 (NCH_2CH_2NH), 53.6 (CH_2NCH_2), 39.5 ($NHCH_2$); HRMS (TOF-MS-ES$^+$) *m/z* calcd for $C_{15}H_{22}{}^{35}ClO_2{}^+$ $[M+H]^+$: 297.1370; found 297.1361.

Synthesis of an oxetane analogue of Donepezil (**35**, **36a**, **36**)

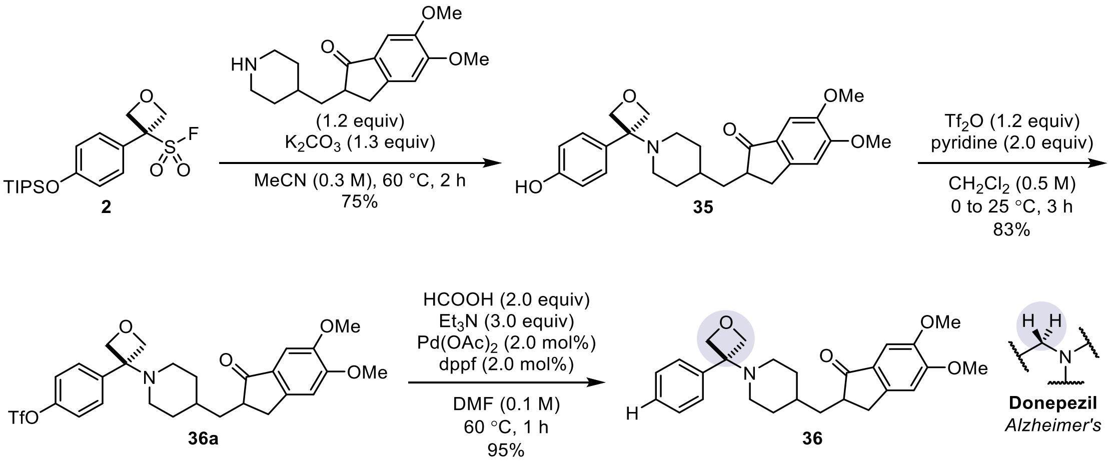

2-((1-(3-(4-Hydroxyphenyl)oxetan-3-yl)piperidin-4-yl)methyl)-5,6-dimethoxy-2,3-dihydro-1H-inden-1-one (35)

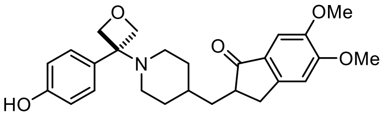

Prepared according to **deFS General Procedure A** under Ar at 60 °C for 2 h with a Celite work-up and using oxetane sulfonyl fluoride **2** (389 mg, 1.0 mmol, 1.0 equiv), K_2CO_3 (179 mg, 1.30 mmol, 1.3 equiv), 5,6-dimethoxy-2-(piperidin-4-ylmethyl)-2,3-dihydro-1H-inden-1-one (376 mg, 1.20 mmol, 1.2 equiv) and anhydrous MeCN (3.0 mL, 0.3 M). Purification by flash column chromatography (EtOAc) afforded amino-oxetane **35** as a white solid (328 mg, 75%). R_f = 0.13 (EtOAc); mp = 195–197 °C; IR (film)/cm^{-1} 3260 (OH st), 2924, 2842, 1690 (C=O st), 1608, 1590, 1500, 1314, 1262, 1223, 911, 729; ^{1}H NMR (400 MHz, $CDCl_3$) δ 7.15 (s, 1 H, Ar-CH), 6.95–6.88 (m, 2 H, 2 × Ar-CH), 6.86–6.79 (m, 3 H, 3 × Ar-CH), 6.46 (br s, 1 H, OH), 4.95–4.82 (m, 4 H, CH_2OCH_2),

3.94 (s, 3 H, OCH_3), 3.87 (s, 3 H, OCH_3), 3.17 (dd, *J* = 16.6, 7.1 Hz, 1 H, C_q=OCH), 2.83–2.54 (m, 4 H, CH_2NCH_2), 1.93–1.56 (m, 5 H, C_q=OCHC*H*$_2$ + C*H*$_2CH_2NCH_2$C*H*H), 1.48–1.19 (m, 4 H, C*H*$_2CH_2NCH_2$CH*H* + C_q=OCHC*H*$_2$CH + C_q=OCHCH$_2$C*H*). ^{13}C NMR (101 MHz, $CDCl_3$) δ 208.2 (C_q=O), 155.6 (Ar-C_q-OH), 155.2 (Ar-*C*$_q$-OCH_3), 149.5 (Ar-*C*$_q$-OCH_3), 149.0 (Ar-C_q), 129.2 (Ar-*C*$_q$-C_q), 128.8 (2 × Ar-CH), 128.7 (Ar-C_q), 114.6 (2 × Ar-CH), 107.4 (Ar-CH), 104.4 (Ar-CH), 80.6 (CH_2OCH_2), 66.4 (C_q), 56.2 (OCH_3), 56.1 (OCH_3), 46.5 (CH_2NCH_2), 45.4 (C_q=O*C*H), 38.7 (C_q=OCH*C*H$_2$CH), 34.3 (*C*H$_2CH_2NCH_2CH_2$), 33.3 ($CH_2CH_2NCH_2$*C*H$_2$), 33.0 (C_q=OCHCH$_2$*C*H$_2$), 31.9 (C_q=OCHCH$_2$*C*H); HRMS (TOF-MS-ES$^+$) *m/z* calcd for $C_{26}H_{32}NO_5^+$ $[M+H]^+$: 438.2280, found: 438.2301.

Notes:

After flushing the Celite *plug with EtOAc, it was found that a significant amount of the phenolic product remained on the* Celite *filter as a solid due to poor solubility in EtAOc. The plug was therefore eluted with further EtOH to dissolve and elute the desired product while still removing the K_2CO_3 as a solid.*

4-(3-(4-((5,6-Dimethoxy-1-oxo-2,3-dihydro-1H-inden-2-yl)methyl)piperidin-1-yl)oxetan-3-yl)phenyl trifluoromethanesulfonate (36a)

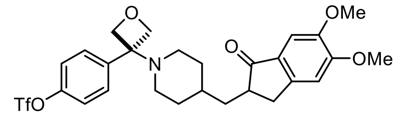

Pyridine (64 mL, 0.8 mmol, 2.0 equiv) and triflic anhydride (80 mL, 0.48 mmol, 1.2 equiv) were added sequentially to a solution of amino-oxetane **35** (175 mg, 0.4 mmol, 1.0 equiv) in anhydrous CH_2Cl_2 (0.8 mL, 0.5 M) in a 10 mL round-bottom flask at 0 °C. After warming to 25 °C and stirring for 3 h, the reaction mixture was quenched with sat. aq. $NaHCO_3$ (15 mL). The phases were separated, and the aqueous layer was extracted with CH_2Cl_2 (3 × 20 mL). The combined organic layers were dried over anhydrous Na_2SO_4, filtered, and concentrated *in vacuo* using a rotary evaporator. Purification by flash column chromatography (70–100% EtOAc/pentane) afforded oxetane triflate **36a** as an off-white solid (190 mg, 83%). R_f = 0.34 (EtOAc); mp = 196–198 °C; IR (film)/cm^{-1} 2920, 1684 (C=O st), 1588, 1416, 1314, 1262, 1213, 1141, 887, 753; ^{1}H NMR (400 MHz, $CDCl_3$) δ 7.35–7.30 (m, 2 H, 2 × Ar-CH), 7.22–7.15 (m, 3 H, 3 × Ar-CH), 6.84 (s, 1 H, Ar-CH), 4.97–4.83 (m, 4 H, CH_2OCH_2), 3.97 (s, 3 H, OCH_3), 3.92 (s, 3 H, OCH_3), 3.20 (dd, *J* = 17.0, 7.6 Hz, 1 H, C_q=OCH), 2.85–2.75 (m, 2 H, C*H*HNC*H*H), 2.73–2.61 (m, 2 H, CH*H*NCH*H*), 1.94–1.83 (m, 1 H, C*H*HCH$_2NCH_2CH_2$), 1.83–1.62 (m, 4 H, C_q=OCHC*H*$_2$ + $CH_2CH_2NCH_2$C*H*$_2$), 1.50–1.25 (m, 4 H, CH*H*CH$_2NCH_2$CH*H* + C_q=OCHC*H*$_2$CH + C_q=OCHCH$_2$C*H*); ^{13}C NMR (126 MHz, $CDCl_3$) δ 207.6 (C_q=O), 155.4 (Ar-*C*$_q$-OCH_3), 149.4 (Ar-*C*$_q$-OTf), 148.6 (Ar-*C*$_q$-OTf), 148.4 (Ar-C_q), 137.8 (Ar-*C*$_q$-C_q), 129.2 (Ar-C_q), 129.1 (2 × Ar-CH), 120.6 (2 × Ar-CH), 118.7 (q, $^1J_{C-F}$ = 322.2 Hz, OCF_3), 107.3 (Ar-CH), 104.3 (Ar-CH), 79.8 (CH_2OCH_2), 66.4 (C_q), 56.2 (OCH_3), 56.1 (OCH_3), 46.5 (CH_2NCH_2), 45.2 (C_q=O*C*H), 38.6 (C_q=OCH*C*H$_2$CH), 34.2 (*C*H$_2CH_2NCH_2CH_2$), 33.2 ($CH_2CH_2NCH_2$*C*H$_2$), 33.0 (C_q=OCHCH$_2$*C*H$_2$), 31.8 (C_q=OCHCH$_2$*C*H); ^{19}F{^{1}H} NMR (377 MHz, $CDCl_3$) δ –72.8; HRMS (TOF-MS-ES$^+$) *m/z* calcd for $C_{27}H_{31}NO_7SF_3^+$ $[M+H]^+$: 570.1773, found: 570.1749.

5,6-Dimethoxy-2-((1-(3-phenyloxetan-3-yl)piperidin-4-yl)methyl)-2,3-dihydro-1H-inden-1-one (36)

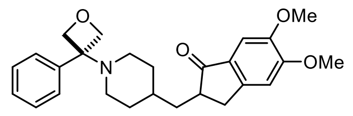

Using reported phenol deoxygenation conditions,[29] triethylamine (83 mL, 0.6 mmol, 3.0 equiv) and formic acid (15.1 µL, 0.4 mmol, 2.0 equiv) were added sequentially to a solution of oxetane triflate **36a** (114 mg, 0.2 mmol, 1.0 equiv), $Pd(OAc)_2$ (0.9 mg, 4 µmol, 2 mol%) and (ferrocene-1,1′-diyl) bis(diphenylphosphane) (2.22 mg, 4 µmol, 2 mol%) in anhydrous DMF (2 mL, 0.1 M) in a 10 mL round-bottom flask. The reaction mixture was warmed to 60 °C and stirred for 1 h. The reaction was cooled down to 25 °C, then brine was added (15 mL). The aqueous layer was extracted with EtOAc (4 × 15 mL). The organic layers were combined, washed with brine (2 × 15 mL), dried over Na_2SO_4, filtered and concentrated *in vacuo* using a rotatory evaporator. Purification by flash column chromatography (50% Et_2O/pentane) afforded amino-oxetane **36** as a pale brown solid (80.0 mg,

95%). R_f = 0.33 (EtOAc); mp = 157–159 °C; IR (film)/cm^{-1} 2927, 1690 (C=O st), 1588, 1497, 1310, 1258, 1221, 1122, 768, 714; ^{1}H NMR (400 MHz, $CDCl_3$) δ 7.42–7.28 (m, 3 H, 3 × Ar-CH), 7.14 (s, 1 H, Ar-CH), 7.05 (d, *J* = 7.5 Hz, 2 H, 2 × Ar-CH), 6.81 (s, 1 H, Ar-CH), 4.93 (d, *J* = 6.0 Hz, 2 H, C*H*HOC*H*H), 4.88 (d, *J* = 6.0 Hz, 2 H, CH*H*OCH*H*), 3.94 (s, 3 H, OCH_3), 3.89 (s, 3 H, OCH_3), 3.15 (dd, *J* = 16.8, 7.3 Hz, 1 H, C_q=OCH), 2.85–2.75 (m, 2 H, C*H*HNC*H*H), 2.69–2.56 (m, 2 H, CH*H*NCH*H*), 1.91–1.81 (m, 1 H, C*H*H$CH_2NCH_2CH_2$), 1.77–1.58 (m, 5 H, (C_q=O)CHCH_2 + $CH_2CH_2NCH_2$CH_2 + H_2O), 1.46–1.25 (m, 4 H, CH*H*CH_2NCH_2CH*H* + (C_q=O)CHCH_2CH + (C_q=O)CHCH_2C*H*); ^{13}C NMR (101 MHz, $CDCl_3$) δ 207.7 (C_q=O), 155.5 (Ar-*C*$_q$-OCH_3), 149.4 (Ar-*C*$_q$-OCH_3), 148.7 (Ar-C_q), 137.0 (Ar-*C*$_q$-C_q), 129.3 (Ar-C_q), 127.6 (2 × Ar-CH), 127.4 (2 × Ar-CH), 127.2 (Ar-CH), 107.3 (Ar-CH), 104.4 (Ar-CH), 80.3 (CH_2OCH_2), 66.9 (C_q), 56.2 (OCH_3), 56.1 (OCH_3), 46.5 (CH_2NCH_2), 45.3 (C_q=O*C*H), 38.7 (C_q=OCH*C*H_2CH), 34.4 (*C*$H_2CH_2NCH_2CH_2$), 33.3 ($CH_2CH_2NCH_2$*C*H_2), 33.1 (C_q=OCHCH_2*C*H_2), 32.0 (C_q=OCHCH_2*C*H); HRMS (TOF-MS-ES$^+$) *m/z* calcd for $C_{26}H_{32}NO_4^+$ $[M+H]^+$: 422.2331, found: 422.2332.

Synthesis of an oxetane analogue of Etobenzanid (**37**, **38**)

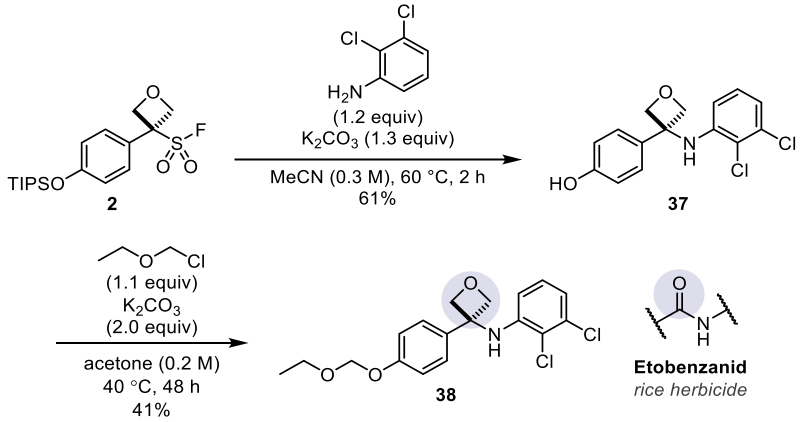

4-(3-((2,3-Dichlorophenyl)amino)oxetan-3-yl)phenol (37)

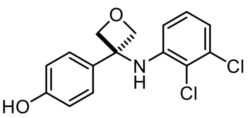

Prepared according to **deFS General Procedure A** under Ar at 60 °C for 2 h with a Celite work-up and using oxetane sulfonyl fluoride **2** (194 mg, 0.5 mmol, 1.0 equiv), K_2CO_3 (89.7 mg, 0.65 mmol, 1.3 equiv), 1,2-dichloroaniline (97.2 mg, 0.6 mmol, 1.2 equiv) and anhydrous MeCN (1.5 mL, 0.3 M). Purification by flash column chromatography (0–30%EtOAc/pentane) afforded amino-oxetane **37** as a white solid (94 mg, 61%). R_f = 0.35 (30% EtOAc/pentane); mp = 190–192 °C; IR (film)/cm^{-1} 3414 (NH st), 3226 (OH st), 2879, 1584, 1517, 1446, 1320, 1273, 1232, 1174, 971, 919, 835, 770; ^{1}H NMR (500 MHz, $CDCl_3$) δ 7.48–7.43 (m, 2 H, 2 × Ar-CH), 6.87–6.78 (m, 4 H, 4 × Ar-CH), 5.70 (dd, *J* = 7.5, 2.2 Hz, 1 H, Ar-CH), 5.31 (s, 1 H, NH), 4.96 (d, *J* = 6.7 Hz, 3 H, C*H*HOC*H*H + OH), 4.92 (d, *J* = 6.4 Hz, 2 H, CH*H*OCH*H*); ^{13}C NMR (126 MHz, $CDCl_3$) δ 155.1 (Ar-C_q-OH), 142.2 (Ar-C_q-NH), 133.3 (Ar-*C*$_q$-C_q), 133.0 (Ar-C_q-Cl), 127.4 (Ar-CH), 126.7 (2 × Ar-CH), 118.9 (Ar-CH), 117.8 (Ar-C_q-Cl), 115.7 (2 × Ar-CH), 110.9 (Ar-CH), 84.2 (CH_2OCH_2), 59.9 (C_q); HRMS (FTMS–ES$^+$) *m/z* calcd for $C_{15}H_{12}Cl_2NO_2^-$ $[M–H]^-$: 308.0251, found: 308.0254.

N-(2,3-Dichlorophenyl)-3-(4-(ethoxymethoxy)phenyl)oxetan-3-amine (38)

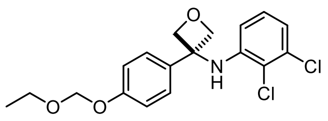

(Chloromethoxy)ethane (10 mL, 0.11 mmol, 1.1 equiv) was added to a solution of oxetane phenol **37** (31.0 mg, 0.1 mmol, 1.0 equiv) and K_2CO_3 (27.6 mg, 0.2 mmol, 2.0 equiv) in non-anhydrous acetone (0.5 mL, 0.2 M) in a 10 mL round-bottom flask. The reaction mixture was stirred at 40 °C for 48 h then diluted with EtOAc (15 mL). The organic layer was washed with water (3 × 10 mL) and brine (10 mL), dried with Na_2SO_4, filtered and concentrated *in vacuo* using a rotatory evaporator. Purification by flash column chromatography (0–20% EtOAc/pentane) afforded amino-oxetane **38** as a colorless oil (15.2 mg, 41%). R_f = 0.48 (20% EtOAc/pentane); IR (film)/cm^{-1} 3412 (NH st), 2952, 2878, 1588, 1510, 1452, 1230, 1180, 1107, 993, 764; ^{1}H NMR (400 MHz, $CDCl_3$) δ 7.54–7.46 (m, 2 H, 2 × Ar-CH), 7.09–7.01 (m, 2 H, 2 × Ar-CH), 6.87–6.76 (m, 2 H, 2 × Ar-CH), 5.71 (dd, *J* = 7.4, 2.2 Hz, 1 H, Ar-CH), 5.31 (s, 1 H, NH), 5.22 (s, 2 H, OCH_2O), 4.97 (d, *J* = 6.3 Hz, 2 H, C*H*HOC*H*H), 4.92 (d, *J* = 6.3 Hz, 2 H, CH*H*OCH*H*), 3.78–3.68 (m, 2 H, OC*H*$_2$CH$_3$), 1.22 (t, *J* = 7.1 Hz, 3 H, OCH$_2$C*H*$_3$); ^{13}C NMR (101 MHz, $CDCl_3$) δ 157.0 (Ar-C_q-OCH$_2$), 142.3 (Ar-C_q-NH), 133.9 (Ar-*C*$_q$-C_q), 133.3 (Ar-C_q-Cl), 127.4 (Ar-CH), 126.4 (2 × Ar-CH), 118.9 (Ar-CH), 117.8 (Ar-C_q-Cl), 116.6 (2 × Ar-CH), 111.0 (Ar-CH), 93.2 (OCH_2O), 84.1 (CH_2OCH_2), 64.3 (O*C*H$_2$CH$_3$), 59.9 (C_q), 15.1 (OCH$_2$*C*H$_3$); HRMS (FTMS–ES$^+$) *m/z* calcd for $C_{18}H_{18}Cl_2NO_3^-$ [M–H]$^-$: 366.0669, found: 366.0670. The observed characterization data were consistent with that previously reported.[30]

Synthesis of an oxetane analogue of Chlormidazole (**91**, **92a**, **92**)

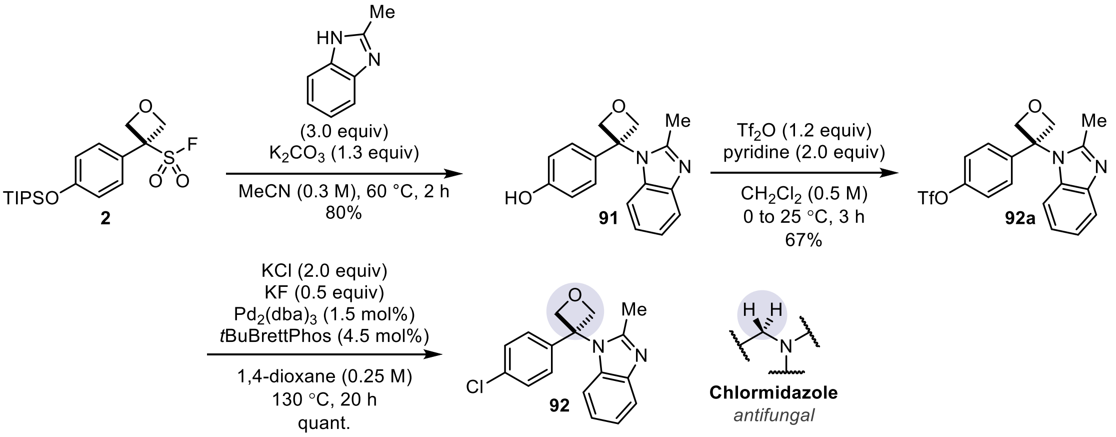

4-(3-(2-Methyl-1H-benzo[*d*]imidazol-1-yl)oxetan-3-yl)phenol (91)

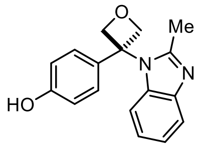

Prepared according to **deFS General Procedure A** under Ar at 70 °C for 2 h with a Celite work-up and using oxetane sulfonyl fluoride **2** (77.6 mg, 0.2 mmol, 1.0 equiv), K_2CO_3 (35.8 mg, 0.26 mmol, 1.3 equiv), 2-methyl-1H-benzo[*d*]imidazole (79.2 mg, 0.6 mmol, 3.0 equiv) and anhydrous MeCN (0.67 mL, 0.3 M). Purification by flash column chromatography (5% EtOH/CH_2Cl_2) afforded oxetano-imidazole **91** as a white solid (44.8 mg, 80%). R_f = 0.23 (5% EtOH/CH_2Cl_2); mp = 198–201 °C; IR (film)/cm^{-1} 3054 (OH st), 2883, 2607, 2361, 1590, 1511 ,1454, 1396, 1280, 1254, 1238, 990, 833, 744; ^{1}H NMR (400 MHz, DMSO-d_6) δ 9.59 (s, 1 H, OH), 7.61 (d, *J* = 7.8 Hz, 1 H, $Ar_{(imidazole)}$-CH), 7.18 (td, *J* = 7.6, 1.2 Hz, 1 H, $Ar_{(imidazole)}$-CH), 7.11 (ddd, *J* = 8.4, 7.2, 1.3 Hz, 1 H, $Ar_{(imidazole)}$-CH), 7.01–6.95 (m, 1 H, $Ar_{(imidazole)}$-CH), 6.92–6.85 (m, 2 H, 2 × Ar-CH), 6.79–6.2 (m, 2 H, 2 × Ar-CH), 5.58 (d, *J* = 6.4 Hz, 2 H, C*H*HOC*H*H), 5.04 (d, *J* = 6.7 Hz, 2 H, CH*H*OCH*H*), 2.24 (s, 3 H, CH_3); ^{13}C NMR (101 MHz, DMSO-d_6) δ 157.3 (Ar-C_q-OH), 151.2 (NC_qN), 142.4 ($Ar_{(imidazole)}$-C_q) 134.5 ($Ar_{(imidazole)}$-C_q), 131.5 (Ar-*C*$_q$-C_q), 125.1 (2 × Ar-CH), 122.1 ($Ar_{(imidazole)}$-CH), 121.6 ($Ar_{(imidazole)}$-CH), 118.8 ($Ar_{(imidazole)}$-CH), 115.7 (2 × Ar-CH), 110.4

($Ar_{(imidazole)}$-CH), 82.1 (CH_2OCH_2), 61.0 (C_q), 14.6 (CH_3); HRMS (FTMS–$APCI^+$) *m/z* calcd for $C_{17}H_{17}N_2O_2^+$ $[M+H]^+$: 281.1285, found: 281.1280.

4-(3-(2-Methyl-1H-benzo[*d*]imidazol-1-yl)oxetan-3-yl)phenyl trifluoromethanesulfonate (92a)

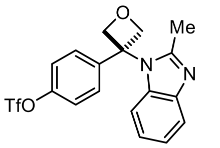

Pyridine (49 mL, 0.6 mmol, 2.0 equiv) and triflic anhydride (60 mL, 0.36 mmol, 1.2 equiv) were added sequentially to a solution of oxetano-imidazole **91** (84.1 mg, 0.3 mmol, 1.0 equiv) in anhydrous CH_2Cl_2 (0.6 mL, 0.5 M) in a 10 mL round-bottom flask at 0 °C. After warming to 25 °C and stirring for 3 h, the reaction mixture was quenched with sat. aq. $NaHCO_3$ (10 mL). The phases were separated, and the aqueous layer was extracted with CH_2Cl_2 (3 × 15 mL). The combined organic layers were dried over anhydrous Na_2SO_4, filtered, and concentrated *in vacuo* using a rotary evaporator. Purification by flash column chromatography (10–50% EtOAc/pentane) afforded oxetane triflate **92a** as an orange gum (83 mg, 67%). R_f = 0.33 (EtOAc); IR (film)/cm^{-1} 2967, 2883, 2358, 2341, 1502, 1426, 1213, 1141, 889, 744; ^1H NMR (400 MHz, CD_3OD) δ 7.67 (d, *J* = 8.0 Hz, 1 H, $Ar_{(imidazole)}$-CH), 7.42 (d, *J* = 9.0 Hz, 2 H, 2 × Ar-CH), 7.33 (d, *J* = 9.0 Hz, 2 H, 2 × Ar-CH), 7.31–7.26 (m, 1 H, $Ar_{(imidazole)}$-CH), 7.21 (td, *J* = 7.7, 1.2 Hz, 1 H, $Ar_{(imidazole)}$-CH), 6.96 (d, *J* = 8.1 Hz, 1 H, $Ar_{(imidazole)}$-CH), 5.75 (d, *J* = 6.9 Hz, 2 H, C*H*HOC*H*H), 5.14 (d, *J* = 6.8 Hz, 2 H, CH*H*OCH*H*), 2.33 (s, 3 H, CH_3); ^{13}C NMR (126 MHz, CD_3OD) δ 153.0 (Ar-C_q-OTf), 151.0 (NC_qN), 143.3 ($Ar_{(imidazole)}$-C_q), 143.1 ($Ar_{(imidazole)}$-C_q), 135.6 (Ar-*C*$_q$-C_q), 127.5 (2 × Ar-CH), 124.4 ($Ar_{(imidazole)}$-CH), 124.0 ($Ar_{(imidazole)}$-CH), 123.5 (2 × Ar-CH), 120.1 (q, $^1J_{C\text{-}F}$ = 320.4 Hz, CF_3), 119.9 ($Ar_{(imidazole)}$-CH), 111.7 ($Ar_{(imidazole)}$-CH), 83.7 (CH_2OCH_2), 63.1 (C_q), 14.7 (CH_3); $^{19}F\{^1H\}$ NMR (377 MHz, CD_3OD) δ –75.0; HRMS (FTMS–$APCI^+$) *m/z* calcd for $C_{18}H_{16}F_3N_2O_4S^+$ $[M+H]^+$: 413.0777, found: 413.0781.

1-(3-(4-Chlorophenyl)oxetan-3-yl)-2-methyl-1H-benzo[*d*]imidazole (92)

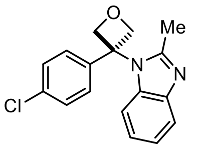

Using chlorination conditions reported by Buchwald,[20] oxetane triflate **92b** (82.5 mg, 0.2 mmol, 1.0 equiv), KCl (29.8 mg, 0.4 mmol, 2.0 equiv), and KF (5.8 mg, 0.1 mmol, 0.5 equiv) were added to a reaction vial (1). The reaction vial (1) was sealed then evacuated and backfilled with Ar three times. $Pd_2(dba)_3$ (2.75 mg, 3 μmol, 1.5 mol%) and ^{t}BuBrettPhos (4.36 mg, 9 μmol, 4.5 mol%) were added to a separate reaction vial (2). The reaction vial (2) was sealed then evacuated and backfilled with Ar three times. Anhydrous, degassed 1,4-dioxane (0.2 mL) was added to reaction vial (2) and the reaction mixture was stirred at 120 °C for 5 min. After cooling to 25 °C, the contents of reaction vial (2) were transferred to reaction vial (1) by syringe and the reaction mixture was diluted with further 1,4-dioxane (0.6 mL). The reaction mixture was then heated to 130 °C and stirred vigorously for 20 h. After cooling to 25 °C, the reaction mixture was filtered through a plug of Celite and eluted with Et_2O (20 mL). The filtrate was then concentrated *in vacuo* using a rotary evaporator. Purification by flash column chromatography (50–100% EtOAc/pentane) afforded oxetano-imidazole **92** as a pale-yellow solid (60.0 mg, quant.). R_f = 0.33 (EtOAc); mp = 165–168 °C; IR (film)/cm^{-1} 3954, 2960, 2879, 2359, 1520, 1493, 1457, 1388, 1368, 1265, 1094 (C-Cl st), 1012, 828, 740, 531; ^1H NMR (400 MHz, CD_3CN) δ 7.65 (d, *J* = 8.0 Hz, 1 H, $Ar_{(imidazole)}$-CH), 7.37 (d, *J* = 8.2 Hz, 2 H, 2 × Ar-CH), 7.23 (t, *J* = 7.6 Hz, 1 H, $Ar_{(imidazole)}$-CH), 7.16–7.02 (m, 3 H, $Ar_{(imidazole)}$-CH + 2 × Ar-CH), 6.86 (d, *J* = 8.1 Hz, 1 H, $Ar_{(imidazole)}$-CH), 5.67 (d, *J* = 6.0 Hz, 2 H, C*H*HOC*H*H), 5.03 (d, *J* = 6.0 Hz, 2 H, CH*H*OCH*H*), 2.24 (s, 3 H, CH_3); ^{13}C NMR (101 MHz, CD_3CN) δ 152.5 (NC_qN), 143.9 ($Ar_{(imidazole)}$-C_q), 141.5 ($Ar_{(imidazole)}$-C_q), 135.8 (Ar-*C*$_q$-C_q), 134.6 (Ar-C_q-Cl), 130.1 (2 × Ar-CH), 126.8 (2 × Ar-CH), 123.4 ($Ar_{(imidazole)}$-CH), 123.0 ($Ar_{(imidazole)}$-CH), 120.2 ($Ar_{(imidazole)}$-CH), 111.2 ($Ar_{(imidazole)}$-CH), 83.3 (CH_2OCH_2), 62.4 (C_q), 15.2 (CH_3); HRMS (FTMS–$APCI^+$) *m/z* calcd for $C_{17}H_{16}ClN_2O^+$ $[M+H]^+$: 299.0946, found: 299.0945.

Synthesis of an oxetane analogue of Safinamide

(*S*)-2-((3-(4-((3-Fluorobenzyl)oxy)phenyl)oxetan-3-yl)amino)propenamide (141)

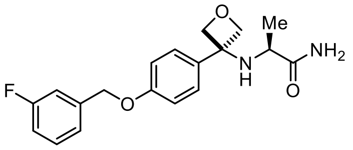

Prepared according to **deFS General Procedure A** under Ar at 60 °C for 2 h with a Celite work-up and using oxetane sulfonyl fluoride **133** (68.1 mg, 0.2 mmol, 1.0 equiv), K_2CO_3 (71.7 mg, 0.52 mmol, 2.6 equiv), Ala-NH_2 hydrochloride (29.9 mg, 0.24 mmol, 1.2 equiv) and anhydrous MeCN (0.67 mL, 0.3 M). Purification by flash column chromatography (0–10% MeOH/CH_2Cl_2) afforded amino-oxetane **141** as pale-yellow crystals (23 mg, 34%). $[\alpha]_D^{21}$ = –6.57 (c 0.5, $CHCl_3$); R_f = 0.09 (5% MeOH/CH_2Cl_2); mp = 137–140 °C; IR (film)/cm^{-1} 3427 (NH_2 st), 3297 (NH_2 st), 3200 (NH st), 2958, 2874, 1675 (C=O st), 1610, 1513, 1450, 1243, 1181, 978, 831, 784; ^{1}H NMR (400 MHz, $CDCl_3$) δ 7.40–7.32 (m, 1 H, $Ar_{(Ph-F)}$-CH), 7.22–7.12 (m, 4 H, 2 × $Ar_{(Ph-F)}$-CH + 2 × Ar-CH), 7.06–6.98 (m, 1 H, $Ar_{(Ph-F)}$-CH), 6.95 (d, *J* = 8.7 Hz, 2 H, 2 × Ar-CH), 6.82 (s, 1 H, N*H*H), 5.27 (s, 1 H, NH*H*), 5.06 (s, 2 H, Ar-C_q-OCH_2), 4.97 (dd, *J* = 14.1, 6.4 Hz, 2 H, C*H*HOC*H*H), 4.72 (dd, *J* = 14.7, 6.4 Hz, 2 H, CH*H*OCH*H*), 3.17 (q, *J* = 7.1 Hz, 1 H, NHC*H*C_q=O), 2.15 (s, 1 H, N*H*CHC_q=O), 1.28 (d, *J* = 7.1 Hz, 3 H, CH_3); ^{13}C NMR (126 MHz, $CDCl_3$) δ 178.0 (C_q=O), 163.0 (d, $^1J_{C\text{-}F}$ = 246.4 Hz, $Ar_{(Ph-F)}$-C_q-F), 157.9 (Ar-*C*$_q$-OCH_2), 139.3 (d, $^3J_{C\text{-}F}$ = 7.3 Hz, $Ar_{(Ph-F)}$-C_q), 134.5 (Ar-*C*$_q$-C_q), 130.2 (d, $^3J_{C\text{-}F}$ = 8.3 Hz, $Ar_{(Ph-F)}$-CH), 127.4 (2 × Ar-CH), 122.7 (d, $^4J_{C\text{-}F}$ = 3.0 Hz, $Ar_{(Ph-F)}$-CH), 115.0 (2 × Ar-CH), 114.9 (d, $^2J_{C\text{-}F}$ = 21.0 Hz, $Ar_{(Ph-F)}$-CH), 114.2 (d, $^2J_{C\text{-}F}$ = 22.1 Hz, $Ar_{(Ph-F)}$-CH), 82.3 (*C*H_2OCH_2), 82.0 (CH_2O*C*H_2), 69.2 (Ar-C_q-O*C*H_2), 63.2 (C_q), 53.2 (NH*C*HC_q=O), 20.9 (CH_3); ^{19}F{^{1}H} NMR (377 MHz, $CDCl_3$) δ –112.7; HRMS (TOF-MS-ES$^+$) *m/z* calcd for $C_{19}H_{22}N_2O_3F^+$ $[M+H]^+$: 345.1614, found: 345.1600.

Notes:

***141** was further characterized by X-ray crystallography (see Fig. **S28–S30**). Crystals suitable for X-ray analysis were grown by slow evaporation from $CDCl_3$.*

Post-deFS functionalization from phenol 25 (26–32)

4-(3-Morpholinooxetan-3-yl)phenol (25)

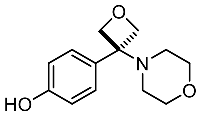

Prepared according to **deFS General Procedure A** under Ar at 60 °C for 2 h with a Celite work-up and using oxetane sulfonyl fluoride **2** (440 mg, 1.1 mmol, 1.0 equiv), K_2CO_3 (223 mg, 1.4 mmol, 1.3 equiv), morpholine (0.11 mL, 1.3 mmol, 1.2 equiv) and anhydrous MeCN (3.3 mL, 0.3 M). Purification by flash column chromatography (80–100% EtOAc/*n*-hexane) afforded amino-oxetane **25** as a white solid (263 mg, 98%). R_f = 0.20 (80% EtOAc/*n*-hexane); ^{1}H NMR (400 MHz, $CDCl_3$) δ 6.93 (d, *J* = 8.1 Hz, 2 H, 2 × Ar-CH), 6.84 (d, *J* = 8.1 Hz, 2 H, 2 × Ar-CH), 5.09 (s, 1 H, OH), 4.90 (d, *J* = 9.1 Hz, 2 H, C*H*HOC*H*H), 4.89 (d, *J* = 9.1 Hz, 2 H, CH*H*OCH*H*), 3.75 (t, *J* = 4.6 Hz, 4 H, CH_2C*H*$_2$OC*H*$_2CH_2$), 2.33 (s, 4 H, CH_2NCH_2). The observed characterization data (R_f, ^{1}H) were consistent with that previously reported.[6]

4-(3-(4-(Pyridin-2-yloxy)phenyl)oxetan-3-yl)morpholine (26)

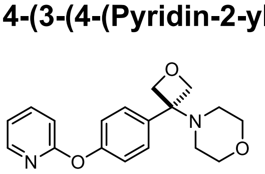

Using literature reported conditions,[31] amino-oxetane **25** (47.1 mg, 0.2 mmol, 1.0 equiv), picolinic acid (4.9 mg, 0.04 mmol, 20 mol%), CuI (3.8 mg, 0.02 mmol, 10 mol%), and K_3PO_4 (93.4 mg, 0.44 mmol, 2.2 equiv) were added to a reaction vial. The reaction vial was sealed then evacuated and backfilled with Ar three times. 2-Iodopyridine (28 μL, 0.26 mmol, 1.3 equiv) and anhydrous, degassed DMSO (0.44 mL, 0.45 M) were added sequentially by syringe to the reaction vial. The reaction mixture was heated to 110 °C and stirred for 24 h. After cooling to 25 °C, water (15 mL) followed by EtOAc (15 mL) were added.

The phases were separated, and the aqueous layer was extracted with EtOAc (2 × 15 mL). The combined organic layers were washed with brine (15 mL), dried over Na_2SO_4, filtered, and concentrated *in vacuo* using a rotary evaporator. Purification by flash column chromatography (80–100% EtOAc/*n*-hexane) afforded amino-oxetane **26** as a pale-yellow solid (39 mg, 60%). R_f = 0.13 (80% EtOAc/*n*-hexane); mp = decomposition observed before melting at 200 °C; IR (film)/cm^{-1} 2948, 2874, 2853, 2822, 1671, 1590, 1507, 1465, 1427, 1289, 1264, 1242, 1211, 1168, 1144, 1115, 1065, 989, 931, 884, 853, 776, 732, 546; ^{1}H NMR (400 MHz, $CDCl_3$) δ 8.22 (dd, *J* = 5.1, 2.0 Hz, 1 H, $Ar_{(py)}$-CH), 7.71 (td, *J* = 7.7, 2.0 Hz, 1 H, $Ar_{(py)}$-CH), 7.17 (d, *J* = 8.5 Hz, 2 H, 2 × Ar-CH), 7.13–7.06 (m, 2 H, 2 × Ar-CH), 7.02 (dd, *J* = 7.2, 5.0 Hz, 1 H, $Ar_{(py)}$-CH), 6.94 (d, *J* = 8.3 Hz, 1 H, $Ar_{(py)}$-CH), 4.93 (d, *J* = 6.0 Hz, 2 H, C*H*HOC*H*H), 4.89 (d, *J* = 6.0 Hz, 2 H, CH*H*OCH*H*), 3.75 (t, *J* = 4.6 Hz, 4 H, 2 × OCH_2CH_2), 2.36 (t, *J* = 4.6 Hz, 4 H, 2 × NCH_2); ^{13}C NMR (101 MHz, $CDCl_3$) δ 163.5 ($Ar_{(py)}$-C_q-O), 153.4 (Ar-C_q-O), 147.8 ($Ar_{(py)}$-CH), 139.6 ($Ar_{(py)}$-CH), 133.4 (Ar-C_q-C_q), 128.5 (2 × Ar-CH), 120.6 (2 × Ar-CH), 118.7 ($Ar_{(py)}$-CH), 111.7 ($Ar_{(py)}$-CH), 79.2 (CH_2OCH_2), 67.2 (2 × OCH_2CH_2), 66.5 (C_q), 46.4 (2 × NCH_2); HRMS (TOF-MS-ES$^+$) *m/z* calcd for $C_{18}H_{21}N_2O_3$ [M+H]: 313.1552; found 313.1561.

***tert*-Butyl 3-(4-(3-morpholinooxetan-3-yl)phenoxy)azetidine-1-carboxylate (27)**

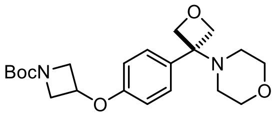

A solution of amino-oxetane **25** (94.2 mg, 0.4 mmol, 1.0 equiv) in DMF (1.3 mL) was added to a solution of NaH (60% in mineral oil, 19 mg, 0.48 mmol, 1.2 equiv) in anhydrous DMF (0.2 mL) at 0 °C. The reaction mixture was stirred at 0 °C for 30 min then a solution of *tert*-butyl-3-bromoazetidine-1-carboxylate (0.14 mL, 0.8 mmol, 2.0 equiv) in anhydrous DMF (0.4 mL) was added by syringe. After stirring at 80 °C for 24 h, the reaction mixture was cooled to 25 °C and water (20 mL) followed by EtOAc (20 mL) were added. The phases were separated, and the aqueous layer was extracted with EtOAc (3 × 15 mL). The combined organic layers were dried over anhydrous Na_2SO_4, filtered, and concentrated *in vacuo* using a rotary evaporator. Purification by flash column chromatography (80–100% EtOAc/*n*-hexane) afforded amino-oxetane **27** as white solid (108 mg, 69%). R_f = 0.22 (100% EtOAc); mp = 113–115 °C; IR (film)/cm^{-1} 2950, 2877, 2822, 1694 (C=O st), 1608, 1510, 1453, 1393, 1366, 1293, 1177, 1143, 1113, 1039, 989, 910, 856, 831, 771, 727, 674, 646, 555; ^{1}H NMR (400 MHz, $CDCl_3$) δ 6.97 (d, *J* = 8.4 Hz, 2 H, 2 × Ar-CH), 6.74 (d, *J* = 8.4 Hz, 2 H, 2 × Ar-CH), 4.91–4.80 (m, 5 H, CH_2C*H*CH_2, CH_2OCH_2), 4.30 (dd, *J* = 9.7, 6.4 Hz, 2 H, NC*H*$_2$CH), 4.01 (dd, *J* = 9.7, 4.1 Hz, 2 H, NC*H*$_2$CH), 3.72 (t, *J* = 4.9 Hz, 4 H, 2 × OC*H*$_2$CH$_2$), 2.29 (t, *J* = 4.9 Hz, 4 H, 2 × NC*H*$_2$CH$_2$), 1.45 (s, 9 H, 3 × CH_3); ^{13}C NMR (101 MHz, $CDCl_3$) δ 156.1 (C_q=O), 155.8 (Ar-*C*$_q$-OCH), 130.4 (Ar-*C*$_q$-C_q), 128.7 (2 × Ar-CH), 114.0 (2 × Ar-CH), 79.9 (*C*$_q$(CH_3)$_3$), 79.2 (2 × OCH_2), 67.1 (2 × OCH_2CH_2), 66.4 (C_q), 65.7 (OCH), 56.4 (br, 2 × NCH_2CH), 46.3 (2 × NCH_2CH_2), 28.4 (3 × CH_3); HRMS (TOF-MS-ES$^+$) *m/z* calcd for $C_{21}H_{31}N_2O_5$ [M+H]: 391.2233; found 391.2229.

4-(3-(4-(Azetidin-3-yloxy)phenyl)oxetan-3-yl)morpholine (28)

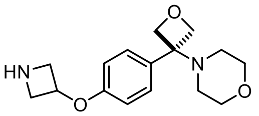

Trifluoroacetic acid (0.18 mL, 2.3 mmol, 8.5 equiv) was added dropwise to a solution of amino-oxetane **27** (106 mg, 0.27 mmol, 1.0 equiv) in anhydrous CH_2Cl_2 (1.22 mL, 0.25 M) at 0 °C. After stirring at 0 °C for 1 h, the reaction mixture was warmed to 25 °C and stirred for a further 19 h. The reaction mixture was quenched with aq. NaOH (1 M, 10 mL), stirred for 5 min, and diluted with CH_2Cl_2 (10 mL). The phases were separated, and the aqueous layer was extracted with CH_2Cl_2 (3 × 10 mL). The combined organic layers were dried over Na_2SO_4, filtered, and concentrated *in vacuo* using a rotary evaporator to afford amino-oxetane **28** as a white solid (51 mg, 67%). R_f = 0.09 (MeOH); mp = 113–115 °C; IR (film)/cm^{-1} 3325, 2947, 2858, 2823, 1607, 1579, 1511, 1453, 1363, 1292, 1241, 1180, 1114, 930, 834, 767, 730, 672, 556; ^{1}H NMR (400 MHz, $CDCl_3$) δ 6.96 (d, *J* = 8.6 Hz, 2 H, 2 × Ar-CH), 6.76 (d, *J* = 8.6 Hz, 2 H, 2 × Ar-CH), 5.01 (p, *J* = 6.1 Hz, 1 H, CH_2C*H*CH_2), 4.88 (d, *J* = 6.0 Hz, 2 H, C*H*HOC*H*H), 4.86 (d, *J* = 6.0 Hz, 2 H, CH*H*OCH*H*), 3.94 (t, *J* = 7.2 Hz, 2 H, NC*H*$_2$CH), 3.82 (t, *J* = 7.2 Hz, 2 H, NC*H*$_2$CH), 3.74 (t, *J* = 4.6 Hz, 4 H, 2 × OC*H*$_2$CH2), 2.30 (t, *J* = 4.6 Hz, 4 H, 2 × NC*H*$_2$CH$_2$), 1.75 (s, 1 H, NH); ^{13}C

NMR (101 MHz, $CDCl_3$) δ 156.2 (Ar-C_q-OCH), 129.8 (Ar-C_q-C_q), 128.6 (2 × Ar-CH), 114.0 (2 × Ar-CH), 79.3 (2 × OCH_2), 70.6 (OCH), 67.1 (2 × OCH_2CH_2), 66.4 (C_q), 54.8 (2 × NCH_2CH), 46.3 (2 × NCH_2CH_2); HRMS (TOF-MS-ES$^+$) *m/z* calcd for $C_{16}H_{23}N_2O_3$ [M+H]: 291.1709; found 291.1713.

4-(3-Morpholinooxetan-3-yl)phenyl trifluoromethanesulfonate (29)

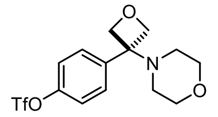

Triflic anhydride (106 μL, 0.63 mmol, 1.1 equiv) was added dropwise to a solution of pyridine (91 μL, 1.15 mmol, 2.0 equiv) and amino-oxetane **25** (135 mg, 0.57 mmol, 1.0 equiv) in anhydrous CH_2Cl_2 (1.15 mL, 0.5 M). After stirring at 25 °C for 3 h, the reaction was quenched with sat. aq. $NaHCO_3$ (10 mL). The phases were separated, and the aqueous layer was extracted with CH_2Cl_2 (3 × 10 mL). The combined organic layers were dried over anhydrous Na_2SO_4, filtered, and concentrated *in vacuo*. Purification by flash column chromatography (80% EtOAc/*n*-hexane) afforded oxetane triflate **29** as red gum (141 mg, 96%). R_f = 0.37 (80% EtOAc/*n*-hexane); ^{1}H NMR (400 MHz, $CDCl_3$) δ 7.32 (d, *J* = 8.7 Hz, 2 H, 2 × Ar-CH), 7.19 (d, *J* = 8.7 Hz, 2 H, 2 × Ar-CH), 4.92 (d, *J* = 6.2 Hz, 2 H, C*H*HOC*H*H), 4.87 (d, *J* = 6.2 Hz, 2 H, CH*H*OCH*H*), 3.75 (t, *J* = 4.6 Hz, 4 H, CH_2C*H*$_2$OC*H*$_2CH_2$), 2.32 (s, 4 H, CH_2NCH_2). ^{19}F{^{1}H} NMR (377 MHz, $CDCl_3$) δ –72.7. The observed characterization data (R_f, ^{1}H, ^{19}F) were consistent with that previously reported.[6]

4-(4-(3-Morpholinooxetan-3-yl)phenyl)morpholine (30)

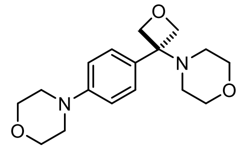

Using conditions developed by Buchwald,[32] oxetane triflate **29** (73.5 mg, 0.2 mmol, 1.0 equiv), $Pd(OAc)_2$ (2.2 mg, 0.01 mmol, 5 mol%), JohnPhos (6.0 mg, 0.02 mmol, 10 mol%), and K_3PO_4 (63.6 mg, 0.3 mmol, 1.5 equiv) were added to a reaction vial. The reaction vial was sealed then evacuated and backfilled with Ar three times. Morpholine (29 μL, 0.24 mmol, 1.2 equiv) followed by anhydrous, degassed THF (0.4 mL, 0.5 M) were added by syringe and the reaction mixture was stirred at 65 °C for 24 h. After cooling to 25 °C, the reaction mixture was diluted with Et_2O (10 mL) and filtered through a plug of Celite, eluting with further Et_2O (3 × 10 mL). The solvent was then removed *in vacuo* using a rotary evaporator. Purification by flash column chromatography (80% EtOAc/*n*-hexane) afforded amino-oxetane **30** as a pale-yellow solid (19 mg, 32%). R_f = 0.2 (80% EtOAc/*n*-hexane); mp = 174–176 °C; IR (film)/cm^{-1} 2952, 2852, 2822, 1609, 1517, 1451, 1379, 1265, 1230, 1116, 988, 930, 818, 731, 676, 563; ^{1}H NMR (400 MHz, $CDCl_3$) δ 6.96 (d, *J* = 8.5 Hz, 2 H, 2 × Ar-CH), 6.91 (d, *J* = 8.5 Hz, 2 H, 2 × Ar-CH), 4.89 (d, *J* = 6.0 Hz, 2 H, C*H*HOC*H*H), 4.86 (d, *J* = 6.0 Hz, 2 H, CH*H*OCH*H*), 3.88 (t, *J* = 4.8 Hz, 4 H, CH_2C*H*$_2$OC*H*$_2CH_2$), 3.73 (t, *J* = 4.6 Hz, 4 H, CH_2C*H*$_2$OC*H*$_2CH_2$), 3.18 (t, *J* = 4.8 Hz, 4 H, CH_2NCH_2), 2.31 (t, *J* = 4.6 Hz, 4 H, CH_2NCH_2); ^{13}C NMR (101 MHz, $CDCl_3$) δ 150.4 (Ar-C_q-N), 128.5 (Ar-C_q-C_q), 128.2 (2 × Ar-CH), 114.8 (2 × Ar-CH), 79.4 (CH_2OCH_2), 67.2 (2 × OCH_2), 66.9 (2 × OCH_2), 66.4 (C_q), 49.1 (2 × NCH_2), 46.4 (2 × NCH_2); HRMS (TOF-MS-ES$^+$) *m/z* calcd for $C_{17}H_{25}N_2O_3$ [M+H]: 305.1865; found 305.1805.

4-(3-(4-(5-(Trifluoromethyl)pyrimidin-2-yl)phenyl)oxetan-3-yl)morpholine (31)

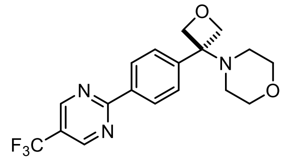

Using literature reported conditions,[33] oxetane triflate **29** (36.8 mg, 0.1 mmol, 1.0 equiv), $Pd(PPh_3)_4$ (3.5 mg, 0.003 mmol, 3 mol%), Cs_2CO_3 (65 mg, 0.2 mmol, 2.0 equiv), and 2-(4,4,5,5-tetramethyl-1,3,2-dioxaborolan-2-yl)-5-(trifluoromethyl)pyrimidine (41 mg, 0.15 mmol, 1.5 equiv) were added to a reaction vial. The reaction vial was sealed then evacuated and backfilled with Ar three times. Anhydrous, degassed 1,4-dioxane:water (7:3, 0.5 mL, 0.2 M) was added by syringe and the reaction mixture was stirred at 110 °C for 20 h. After cooling to 25 °C, the reaction mixture was diluted with Et_2O (10 mL) and filtered through a plug of Celite, eluting with further Et_2O (3 × 10 mL). The solvent was then removed *in vacuo* using a rotary evaporator. Purification by flash column chromatography (60–80% EtOAc/*n*-hexane) afforded amino-oxetane **31** as a white solid (35 mg, 93%). R_f = 0.15 (80% EtOAc/*n*-hexane); mp = 160–162 °C; IR (film)/cm^{-1} 2953, 2876, 2824, 1571,

1550, 1454, 1353, 1299, 1267, 1192, 1147, 1117, 991, 933, 838, 818, 572; ^{1}H NMR (400 MHz, $CDCl_3$) δ 9.11 (s, 2 H, 2 × $Ar_{(py)}$-CH), 7.66 (d, *J* = 8.0 Hz, 2 H, 2 × Ar-CH), 7.29 (d, *J* = 8.0 Hz, 2 H, 2 × Ar-CH), 4.96 (d, *J* = 6.2 Hz, 2 H, C*H*HOC*H*H), 4.94 (d, *J* = 6.2 Hz, 2 H, CH*H*OCH*H*), 3.77 (t, *J* = 4.6 Hz, 4 H, $CH_2CH_2OCH_2CH_2$), 2.38 (d, *J* = 4.6 Hz, 4 H, CH_2NCH_2); ^{13}C NMR (101 MHz, $CDCl_3$) δ 155.8 (q, $^2J_{C–F}$ = 31.4 Hz, $Ar_{(py)}$-C_q-CF_3), 155.7 (2 × $Ar_{(py)}$-CH), 139.4 (Ar-C_q-C_q), 135.6 (Ar-C_q-$Ar_{(py)}$-C_q), 132.1 ($Ar_{(py)}$-C_q-Ar-C_q), 128.5 (2 × Ar-CH), 127.0 (2 × Ar-CH), 119.7 (d, $^1J_{C-F}$ = 274.9 Hz, CF_3), 78.8 (CH_2OCH_2), 67.1 (2 × OCH_2), 66.7 (C_q), 46.5 (2 × NCH_2); ^{19}F{^{1}H} NMR (377 MHz, $CDCl_3$) δ –70.1; HRMS (TOF-MS-ES$^+$) *m/z* calcd for $C_{18}H_{19}N_3O_2F_3$ [M+H]: 366.1429; found 366.1414.

4-(3-(4-(Phenylsulfonyl)phenyl)oxetan-3-yl)morpholine (32)

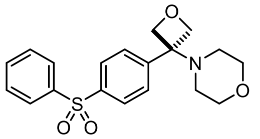

Using literature reported conditions,[34] phenyllithium (1.82 M, 0.11 mL, 0.2 mmol, 2.0 equiv) was added dropwise to a solution of DABSO (24 mg, 0.1 mmol, 1.0 equiv) in degassed 1,4-dioxane (0.77 mL, 0.13 M). The reaction was stirred at 25 °C for 2 h. Oxetane triflate **29** (49 mg, 0.13 mmol, 1.3 equiv), $Pd(OAc)_2$ (3.0 mg, 0.013 mmol, 10 mol%), Cs_2CO_3 (65 mg, 0.2 mmol, 2.0 equiv) and Xantphos (7.7 mg, 0.013 mmol, 10 mol%) were added to a separate microwave vial. The reaction vial was evacuated and backfilled with nitrogen three times. The 1,4-dioxane suspension was then added via syringe. The reaction was stirred at 110 ºC for 16 h then cooled to room temperature. CH_2Cl_2 (10 mL) was added and the reaction was filtered through Celite and then concentrated *in vacuo* using a rotary evaporator. Purification by flash column chromatography (50% EtOAc/CH_2Cl_2) afforded amino-oxetane **32** as a colorless oil (21 mg, 58%). R_f = 0.24 (50% EtOAc/CH_2Cl_2); IR (film)/cm^{-1} 2957, 2856, 1447, 1319 (S=O as st), 1309, 1290, 1266, 1157 (S=O sy st), 1110, 990, 932, 912, 729; ^{1}H NMR (400 MHz, $CDCl_3$) δ 8.01–7.94 (m, 4 H, 4 × Ph-CH), 7.64–7.58 (m, 1 H, Ph-CH), 7.58–7.51 (m, 2 H, 2 × Ar-CH), 7.25–7.18 (m, 2 H, 2 × Ar-CH), 4.90 (d, *J* = 6.4 Hz, 2 H, C*H*HOC*H*H), 4.83 (d, *J* = 6.4 Hz, 2 H, CH*H*OCH*H*), 3.72 (t, *J* = 4.5 Hz, 4 H, $CH_2CH_2OCH_2CH_2$), 2.29 (t, *J* = 4.5 Hz, 4 H, CH_2NCH_2); ^{13}C NMR (101 MHz, $CDCl_3$) δ 143.1 (Ar-C_q-SO_2), 141.3 (Ar-C_q-SO_2), 140.8 (Ar-C_q-C_q), 133.4 (Ph-CH), 129.4 (2 × Ar-CH), 128.0 (2 × Ar-CH), 127.7 (2 × Ph-CH), 127.4 (2 × Ph-CH), 78.4 (CH_2OCH_2), 67.0 (2 × OCH_2), 66.7 (C_q), 46.3 (2 × NCH_2); HRMS (FTMS–NSI$^+$) *m/z* calcd for $C_{19}H_{22}NO_4S$ [M+H]$^+$: 360.1262, found: 360.1262.

Oxetane deFS with NH-azole nucleophiles (39–63)

1-(3-(4-Methoxyphenyl)oxetan-3-yl)-1*H*-pyrazole (39)

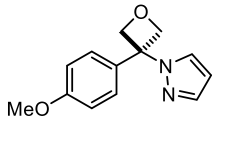

Prepared according to **deFS General Procedure B** under Ar at 60 °C for 2 h with an aqueous work-up and using oxetane sulfonyl fluoride **1** (49.2 mg, 0.2 mmol, 1.0 equiv), K_2CO_3 (35.8 mg, 0.26 mmol, 1.3 equiv), 1*H*-pyrazole (16.3 mg, 0.24 mmol, 1.2 equiv) and anhydrous MeCN (0.67 mL, 0.3 M). Purification by flash column chromatography (50% Et_2O/petroleum spirits) afforded oxetano-pyrazole **39** as a colorless oil which solidified into a white solid in the freezer at –20 °C (34.0 mg, 74%). R_f = 0.19 (30% EtOAc/ *n*-hexane); mp = 39–40 °C; IR (film)/cm^{-1} 2958, 2886, 2839, 1612, 1514, 1252, 1183, 1030, 987; ^{1}H NMR (400 MHz, $CDCl_3$) δ 7.69 (d, *J* = 1.8 Hz, 1 H, $Ar_{(pyrazole)}$-CH), 7.23 (d, *J* = 2.4 Hz, 1 H, $Ar_{(pyrazole)}$-CH), 7.13–7.05 (m, 2 H, 2 × Ar-CH), 6.98–6.89 (m, 2 H, 2 × Ar-CH), 6.34 (dd, *J* = 2.4, 1.8 Hz, 1 H, $Ar_{(pyrazole)}$-CH), 5.53 (d, *J* = 6.5 Hz, 2 H, C*H*HOC*H*H), 5.20 (d, *J* = 6.5 Hz, 2 H, C*H*HOC*H*H), 3.84 (s, 3 H, OCH_3); ^{13}C NMR (101 MHz, $CDCl_3$) δ 159.7 (Ar-C_q-OCH_3), 140.1 ($Ar_{(pyrazole)}$-CH), 133.2 (Ar-C_q-C_q), 128.5 ($Ar_{(pyrazole)}$-CH), 127.1 (2 × Ar-CH), 114.4 (2 × Ar-CH), 106.3 ($Ar_{(pyrazole)}$-CH), 82.6 (CH_2OCH_2), 66.3 (C_q), 55.5 (OCH_3); HRMS (TOF-MS-ES$^+$) *m/z* calcd for $C_{13}H_{15}N_2O_2^+$ $[M+H]^+$: 231.1134, found: 231.1138.

1-(3-(4-Methoxyphenyl)oxetan-3-yl)-4-methyl-1*H*-pyrazole (40)

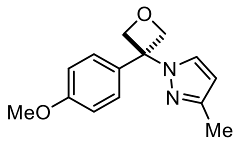

Prepared according to **deFS General Procedure B** under Ar at 60 °C for 2 h with an aqueous work-up and using oxetane sulfonyl fluoride **1** (49.2 mg, 0.2 mmol, 1.0 equiv), K_2CO_3 (35.9 mg, 0.26 mmol, 1.3 equiv), 4-methylpyrazole (19.8 mL, 0.24 mmol, 1.2 equiv) and anhydrous MeCN (0.67 mL, 0.3 M). Purification by flash column chromatography (50% Et_2O/petroleum spirits) afforded oxetano-pyrazole **40** as a colorless oil (37.6 mg, 77%). R_f = 0.32 (50% Et_2O/pentane); IR (film)/cm^{-1} 2954, 2883, 2837, 1611, 1580, 1512, 1460, 1416, 1347, 1300, 1247, 1180, 998, 828; ^{1}H NMR (400 MHz, $CDCl_3$) δ 7.47 (s, 1 H, $Ar_{(pyrazole)}$-CH), 7.12–7.04 (m, 2 H, 2 × Ar-CH), 6.98 (s, 1 H, $Ar_{(pyrazole)}$-CH), 6.96–6.87 (m, 2 H, 2 × Ar-CH), 5.48 (d, *J* = 6.4 Hz, 2 H, C*H*HOC*H*H), 5.15 (d, *J* = 6.4 Hz, 2 H, CH*H*OCH*H*), 3.82 (s, 3 H, OCH_3), 2.08 (s, 3 H, C_q-CH_3); ^{13}C NMR (101 MHz, $CDCl_3$) δ 159.4 (Ar-C_q-OCH_3), 140.3 ($Ar_{(pyrazole)}$-CH), 133.2 (Ar-C_q-C_q), 127.1 ($Ar_{(pyrazole)}$-CH), 126.9 (2 × Ar-CH), 116.7 ($Ar_{(pyrazole)}$-CH), 114.2 (2 × Ar-CH), 82.4 (CH_2OCH_2), 65.9 (C_q), 55.3 (OCH_3), 8.9 (C_q-CH_3); HRMS (TOF-MS-ES$^+$) *m/z* calcd for $C_{14}H_{17}N_2O_2^+$ $[M+H]^+$: 245.1290, found: 245.1279.

4-Bromo-1-(3-(4-methoxyphenyl)oxetan-3-yl)-1*H*-pyrazole (41)

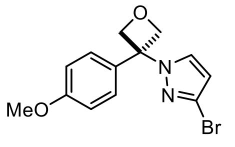

Prepared according to **deFS General Procedure B** under Ar at 60 °C for 2 h with an aqueous work-up and using oxetane sulfonyl fluoride **1** (49.2 mg, 0.2 mmol, 1.0 equiv), K_2CO_3 (35.8 mg, 0.26 mmol, 1.3 equiv), 4-bromopyrazole (35.3 mg, 0.24 mmol, 1.2 equiv) and anhydrous MeCN (0.67 mL, 0.3 M). Purification by flash column chromatography (1% Et_2O/CH_2Cl_2) afforded oxetano-pyrazole **41** as a yellow oil which solidified into a pale-yellow solid in the freezer at –20 °C (37.7 mg, 61%). R_f = 0.19 (1% Et_2O/CH_2Cl_2); mp = 50–51 °C; IR (film)/cm^{-1} 3118, 2956, 2884, 2865, 1611, 1512, 1323, 1301, 1248, 1178, 987, 972, 949, 827, 731, 610, 578, 550; ^{1}H (400 MHz, $CDCl_3$) δ 7.62 (s, 1 H, $Ar_{(pyrazole)}$-CH), 7.16 (s, 1 H, $Ar_{(pyrazole)}$-CH), 7.14–7.07 (m, 2 H, 2 × Ar-CH), 6.98–6.90 (m, 2 H, 2 × Ar-CH), 5.45 (d, *J* = 6.5 Hz, 2 H, C*H*HOC*H*H), 5.18 (d, *J* = 6.5 Hz, 2 H, CH*H*OCH*H*), 3.83 (s, 3 H, OCH_3); ^{13}C NMR (101 MHz, $CDCl_3$) δ 159.9 (Ar-C_q-OCH_3), 140.6 ($Ar_{(pyrazole)}$-CH), 131.9 (Ar-C_q-C_q), 128.8 ($Ar_{(pyrazole)}$-CH), 127.4 (2 × Ar-CH), 114.5 (2 × Ar-CH), 93.9 (C_q-Br), 82.1 (CH_2OCH_2), 67.0 (C_q), 55.5 (OCH_3); HRMS (TOF-MS-ES$^+$) *m/z* calcd for $C_{13}H_{14}N_2O_2{}^{79}Br^+$ $[M+H]^+$: 309.0239, found: 309.0239.

1-(3-(4-Methoxyphenyl)oxetan-3-yl)-4-(4,4,5,5-tetramethyl-1,3,2-dioxaborolan-2-yl)-1*H*-pyrazole (42)

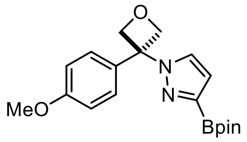

Prepared according to **deFS General Procedure B** under Ar at 60 °C for 2 h with an aqueous work-up and using oxetane sulfonyl fluoride **1** (49.2 mg, 0.2 mmol, 1.0 equiv), K_2CO_3 (35.8 mg, 0.26 mmol, 1.3 equiv), 4-(4,4,5,5-tetramethyl-1,3,2-dioxaborolan-2-yl)-1H-pyrazole (46.6 mg, 0.24 mmol, 1.2 equiv) and anhydrous MeCN (0.67 mL, 0.3 M). Purification by automated flash column chromatography (0–60% Et_2O/petroleum spirits, see conditions below) afforded oxetano-pyrazole **42** as a white solid (35.7 mg, 50%). R_f = 0.28 (50% Et_2O/pentane); mp = 91–92 °C; IR (film)/cm^{-1} 2941, 1610, 1552, 1515, 1368, 1297, 1264, 1243, 987, 827; ^{1}H NMR (400 MHz, $CDCl_3$) δ 7.95 (s, 1 H, $Ar_{(pyrazole)}$-CH), 7.51 (s, 1 H, $Ar_{(pyrazole)}$-CH), 7.14–7.06 (m, 2 H, 2 × Ar-CH), 6.96–6.88 (m, 2 H, 2 × Ar-CH), 5.50 (d, *J* = 6.6 Hz, 2 H, C*H*HOC*H*H), 5.18 (d, *J* = 6.5 Hz, 2 H, CH*H*OCH*H*), 3.83 (s, 3 H, OCH_3), 1.31 (s, 12 H, 4 × C-CH_3); ^{13}C NMR (126 MHz, $CDCl_3$) δ 159.72 (Ar-C_q-OCH_3), 146.21 ($Ar_{(pyrazole)}$-CH), 135.46 ($Ar_{(pyrazole)}$-CH), 132.61 (Ar-C_q-C_q), 127.33 (2 × Ar-CH), 114.47 (2 × Ar-CH), 83.57 (2 × B-O-*C*(CH_3)$_2$), 82.52 (CH_2OCH_2), 66.35 (C_q), 55.51 (OCH_3), 24.91 (4 × B-O-C(*C*H_3)$_2$); ^{11}B NMR (128 MHz, $CDCl_3$) δ 29.88; HRMS (TOF-MS-ES$^+$) *m/z* calcd for $C_{19}H_{26}N_2O_4B^+$ [M+H]$^+$: 357.1986, found: 357.1988.

Notes:

The quaternary carbon directly attached to the boron atom was not observed in the ^{13}C NMR of ***42****, likely due to quadrupolar relaxation.*[35]

Automated Column Conditions: Run on a Biotage® Selekt system. Column type: Biotage® Sfär 10 g. Flow rate: 40 mL/min. Sample mass: 100 mg. Solvent A: petroleum spirits, Solvent B: Et_2O. UV wavelength detection: l 200–400 nm. See trace below. From the left: peak 1 (yellow): oxetano-pyrazole **42**.

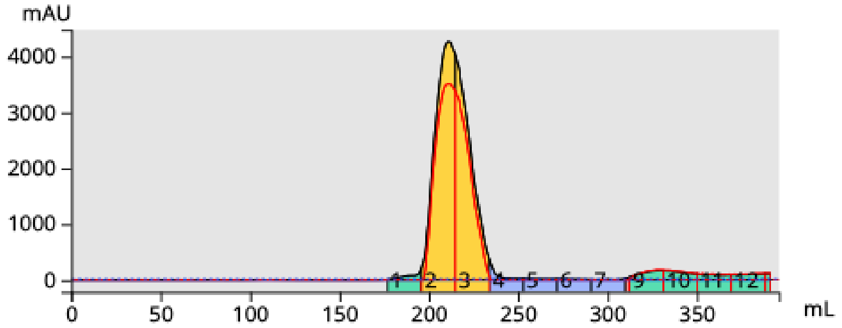

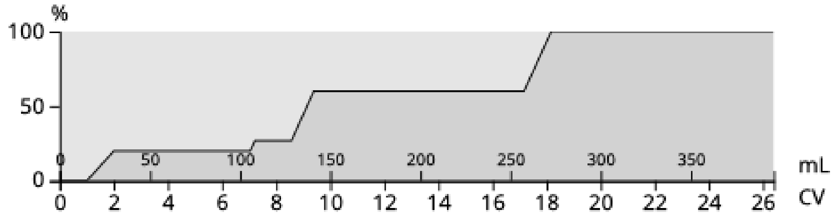

1-(3-(4-Methoxyphenyl)oxetan-3-yl)-3-methyl-1*H*-pyrazole (43) and 1-(3-(4-methoxyphenyl)oxetan-3-yl)-5-methyl-1*H*-pyrazole (43')

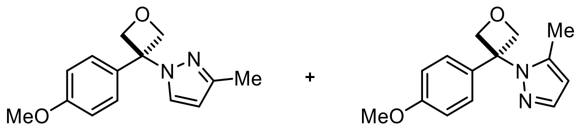

major 88:12 minor

Prepared according to **deFS General Procedure B** under Ar at 60 °C for 2 h with an aqueous work-up and using oxetane sulfonyl fluoride **1** (49.2 mg, 0.2 mmol, 1.0 equiv), K_2CO_3 (35.8 mg, 0.26 mmol, 1.3 equiv), 3/5-methyl-1*H*-pyrazole (19.3 mL, 0.24 mmol, 1.2 equiv) and anhydrous MeCN (0.67 mL, 0.3 M). Purification by flash column chromatography (40% Et_2O/petroleum spirits) afforded an 88:12 regioisomeric mixture of oxetano-pyrazoles **43** and **43'** as a colorless oil (45.8 mg, 94%). Further purification of a small quantity by preparatory TLC (40% Et_2O/petroleum spirits × 2) and subsequent selective nOe experiments identified the major and minor regioisomers as 3-methyl (**43**) and 5-methyl (**43'**).

Major regioisomer (**43**): R_f = 0.21 (40% Et_2O/pentane); IR (film)/cm^{-1} 2958, 1612, 1515, 1252, 1183, 986, 830, 757; ^{1}H NMR (400 MHz, $CDCl_3$) δ 7.10 (d, *J* = 2.3 Hz, 1 H, $Ar_{(pyrazole)}$-C_5H), 7.09–7.03 (m, 2 H, 2 × Ar-CH), 6.95–6.87 (m, 2 H, 2 × Ar-CH), 6.10 (dd, *J* = 2.3, 0.6 Hz, 1 H, $Ar_{(pyrazole)}$-C_4H), 5.50 (dd, *J* = 6.3, 0.6 Hz, 2 H, C*H*HOC*H*H), 5.14 (dd, J = 6.3, 0.6 Hz, 2 H, CH*H*OCH*H*), 3.82 (s, 3 H, OCH_3), 2.35 (s, 3 H, C_q-CH_3); ^{13}C NMR (101 MHz, $CDCl_3$) δ 159.6 (Ar-C_q-OCH_3), 149.4 ($Ar_{(pyrazole)}$-C_q), 133.6 (Ar-C_q-C_q), 129.3 ($Ar_{(pyrazole)}$-C_5H), 127.0 (2 × Ar-CH), 114.3 (2 × Ar-CH), 105.9 ($Ar_{(pyrazole)}$-C_4H), 82.7 (CH_2OCH_2), 65.9 (C_q), 55.5 (OCH_3), 13.9 (C_q-CH_3); HRMS (TOF-MS-ES$^+$) *m/z* calcd for $C_{14}H_{17}N_2O_2^+$ $[M+H]^+$: 245.1290, found: 245.1285.

Minor regioisomer (**43'**): R_f = 0.26 (40% Et_2O/pentane); IR (film)/cm^{-1} 2959, 1611, 1513, 1388, 1252, 1183, 1031, 987, 831; ^{1}H NMR (400 MHz, $CDCl_3$) δ 7.53 (d, *J* = 1.8 Hz, 1 H, $Ar_{(pyrazole)}$-C_3H), 6.95 (d, *J* = 9.1 Hz, 2 H, 2 × Ar-CH), 6.87 (d, *J* = 8.9 Hz, 2 H, 2 × Ar-CH), 6.16 (dd, *J* = 1.8, 0.8 Hz, 1 H, $Ar_{(pyrazole)}$-C_4H), 5.62 (d, *J* = 6.9 Hz, 2 H, C*H*HOC*H*H), 4.96 (d, *J* = 6.9 Hz, 2 H, CH*H*OCH*H*), 3.80 (s, 3 H, OCH_3), 1.94 (s, 3 H, C_q-CH_3); ^{13}C NMR (101 MHz, $CDCl_3$) δ 159.4 (Ar-C_q-OCH_3), 138.8 ($Ar_{(pyrazole)}$-C_3H), 136.7 ($Ar_{(pyrazole)}$-C_q), 134.4 (Ar-C_q-C_q), 125.6 (2 × Ar-CH), 114.4 (2 × Ar-CH), 107.1 ($Ar_{(pyrazole)}$-C_4H), 82.6 (CH_2OCH_2), 64.9 (C_q), 55.4 (OCH_3), 11.5 (C_q-CH_3).

Notes:

Regioisomers assigned from selective nOe spectra (see pages S299, S300 and S302, S303 for full details and spectra):

- *nOe between the oxetane CH_2 and pyrazole NC-H is visible for the major product (**43**) but not the minor product (**43'**).*
- *Weaker pyrazole CH_3/oxetane CH_2 and pyrazole CH_3/PMP Ar-CH nOe correlations were observed for the major product (**43**) versus the minor product (**43'**).*

4-(3-(3-Methyl-1*H*-pyrazol-1-yl)oxetan-3-yl)phenol (44) and 4-(3-(5-methyl-1*H*-pyrazol-1-yl)oxetan-3-yl)phenol (44')

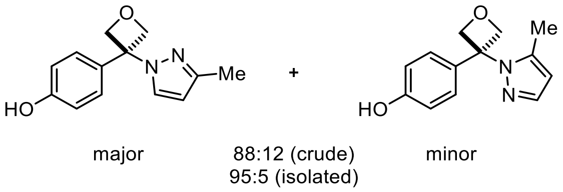

Prepared according to **deFS General Procedure B** under Ar at 60 °C for 2 h with a Celite work-up and using oxetane sulfonyl fluoride **2** (77.7 mg, 0.2 mmol, 1.0 equiv), K_2CO_3 (35.8 mg, 0.26 mmol, 1.3 equiv), 3/5-methyl-1*H*-pyrazole (19.3 mL, 0.24 mmol, 1.2 equiv) and anhydrous MeCN (0.67 mL, 0.3 M). Purification by flash column chromatography (40% EtOAc/pentane) afforded a 95:5 regioisomeric mixture of oxetano-pyrazoles **44** and **44'** as a white solid (45 mg, 98%). R_f = 0.24 (40% EtOAc/pentane); mp = 201–203 °C; ^{1}H NMR (400 MHz, $CDCl_3$) δ 7.53 (s, 0.05 H, $Ar_{(pyrazole)}$-C_3H, **44'**), 7.16 (d, *J* = 2.3 Hz, 1 H, $Ar_{(pyrazole)}$-C_5H, **44**), 6.94 (d, *J* = 8.5 Hz, 2 H, 2 × Ar-CH, **44**), 6.86 (d, *J* = 8.8 Hz, 0.1 H, 2 × Ar-CH, **44'**), 6.75 (d, *J* = 8.4 Hz, 2 H, 2 × Ar-CH, **44**), 6.15 (s, 0.05 H, $Ar_{(pyrazole)}$-C_4H, **44'**), 6.11 (d, *J* = 2.4 Hz, 1 H, $Ar_{(pyrazole)}$-C_4H, **44**), 5.60 (d, *J* = 6.5 Hz, 0.15 H, C*H*HOC*H*H + br s, OH, **44'**) 5.46 (d, *J* = 6.5 Hz, 2 H, C*H*HOC*H*H, **44**), 5.11 (d, *J* = 6.5 Hz, 2 H, CH*H*OCH*H*, **44**), 4.94 (d, *J* = 6.4 Hz, 0.10 H, CH*H*OCH*H*, **44'**) 2.35 (s, 3 H, CH_3, **44**); 1.94 (s, 0.15 H, CH_3, **44'**); ^{13}C NMR (126 MHz, $CDCl_3$) δ 155.6 (Ar-C_q-OH, **44**), 149.3 ($Ar_{(pyrazole)}$-C_q, **44**), 133.2 (Ar-C_q-C_q, **44**), 129.4 ($Ar_{(pyrazole)}$-C_5H, **44**), 127.3 (2 × Ar-CH, **44'**), 126.9 (2 × Ar-CH, **44**), 115.9 (2 × Ar-CH, **44'**), 115.8 (2 × Ar-CH, **44**), 105.9 ($Ar_{(pyrazole)}$-C_4H, **44**), 82.5 (CH_2OCH_2, **44**), 82.0 (CH_2OCH_2, **44'**), 65.9 (C_q, **44**), 13.7 (CH_3, **44**); HRMS (TOF-MS-ES$^+$) *m/z* calcd for $C_{13}H_{15}N_2O_2^+$ [M+H]$^+$: 231.1134, found: 231.1130.

Notes:

*The 88:12 regiomeric ratio was determined by relative ^{1}H NMR integrations of the crude reaction mixture. However, after purification by column chromatoghraphy, a regiomeric ratio of 95:5 was observed. Presumably some of amino-oxetane **44'** was lost during this purification.*

*Regioisomers assigned by analogy of **43** and **43**' assignment:*

- *Oxetane quaternary carbons have distinct ^{13}C NMR chemical shifts for each regioisomer:*
  - *PMP 3-methyl **43** = 65.9 ppm; phenol 3-methyl **44** = 65.9 ppm; PMP 5-methyl **43'** = 64.9 ppm. C_q was not observed for phenol 5-methyl **44'** due to the very low quantity from the reaction.*
- *Oxetane CH_2 protons have distinct ^{1}H NMR chemical shifts for each regioisomer in both cases:*
  - *PMP 3-methyl **43** = 5.50 and 5.14 ppm; phenol 3-methyl **44** = 5.46 and 5.11 ppm; PMP 5-methyl **43'** = 5.62 and 4.96 ppm; phenol 5-methyl **44'** = 5.60 and 4.94 ppm*
- *Pyrazole Ar-CH protons have distinct ^{1}H NMR chemical shifts for each regioisomer in both cases:*
  - *PMP 5-methyl **43** = 7.10 and 6.10 ppm; phenol 5-methyl **44** = 7.16 and 6.15 ppm; PMP 3-methyl **43'** = 7.53 and 6.16 ppm; phenol 3-methyl **44'** = 7.53 and 7.15 ppm*

1-(3-(4-(Benzyloxy)phenyl)oxetan-3-yl)-1*H*-pyrazole (45)

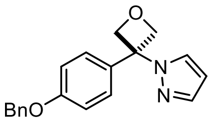

Prepared according to **deFS General Procedure B** under Ar at 70 °C for 2 h with a Celite work-up and using oxetane sulfonyl fluoride **132** (64.5 mg, 0.2 mmol, 1.0 equiv), K_2CO_3 (35.8 mg, 0.26 mmol, 1.3 equiv), 1*H*-pyrazole (16.3 mg, 0.24 mmol, 1.2 equiv) and anhydrous MeCN (0.67 mL, 0.3 M). Purification by flash column chromatography (20–30% EtOAc/*n*-hexane) afforded oxetano-pyrazole **45** as a white solid

(52.3 mg, 85%). R_f = 0.28 (30% EtOAc/*n*-hexane); mp = 109–111 °C; IR (film)/cm^{-1} 2954, 2883, 1608, 1511, 1454, 1394, 1247; ^{1}H NMR (400 MHz, $CDCl_3$) δ 7.68 (d, *J* = 1.8 Hz, 1 H, $Ar_{(pyrazole)}$-CH), 7.46–7.31 (m, 5 H, 5 × Ph-CH), 7.23 (d, *J* = 2.4 Hz, 1 H, $Ar_{(pyrazole)}$-CH), 7.09–7.04 (m, 2 H, 2 × Ar-CH), 7.02–6.95 (m, 2 H, 2 × Ar-CH), 6.34 (t, *J* = 2.1 Hz, 1 H, $Ar_{(pyrazole)}$-CH), 5.51 (d, *J* = 6.5 Hz, 2 H, C*H*HOC*H*H), 5.18 (d, *J* = 6.5 Hz, 2 H, CH*H*OCH*H*), 5.08 (s, 2 H, $PhCH_2$); ^{13}C NMR (101 MHz, $CDCl_3$) δ 158.7 (Ar-C_q-OBn), 140.0 ($Ar_{(pyrazole)}$-CH), 136.7 (Ar-C_q-CH_2O), 133.4 (Ar-C_q-C_q), 128.7 (Ar-CH), 128.5 (2 × Ar-CH), 128.1 (2 × Ar-CH), 127.5 ($Ar_{(pyrazole)}$-CH), 127.0 (2 × Ar-CH), 115.2 (2 × Ar-CH), 106.2 ($Ar_{(pyrazole)}$-CH), 82.5 (CH_2OCH_2), 70.1 ($PhCH_2$), 66.1 (C_q); HRMS (TOF-MS-ES^+) *m/z* calcd for $C_{19}H_{19}N_2O_2^+$ $[M+H]^+$: 307.1447; found: 307.1452.

Notes:

***45** was further characterized by X-ray crystallography (see Fig. **S8**). Crystals suitable for X-ray analysis were grown by slow evaporation from $CDCl_3$.*

4-(3-(2*H*-Indazol-2-yl)oxetan-3-yl)phenol (46)

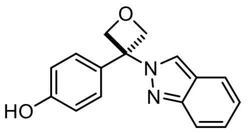

Prepared according to **deFS General Procedure B** under Ar at 60 °C for 2 h with a Celite work-up and using oxetane sulfonyl fluoride **2** (77.7 mg, 0.2 mmol, 1.0 equiv), K_2CO_3 (35.8 mg, 0.26 mmol, 1.3 equiv), 1/2*H*-indazole (28.3 mg, 0.24 mmol, 1.2 equiv) and anhydrous MeCN (0.67 mL, 0.3 M). Purification by flash column chromatography (20–40% EtOAc/pentane) afforded oxetano-indazole **46** as a white solid (42 mg, 78%). R_f = 0.26 (40% EtOAc/pentane); mp = 205–206 °C; IR (film)/cm^{-1} 3066 (OH st), 2956, 1612, 1515, 1457, 1260, 980.3, 792, 731; ^{1}H NMR (400 MHz, CD_3OD) δ 8.12 (s, 1 H, $Ar_{(indazole)}$-CH), 7.67 (dd, *J* = 12.5, 8.6 Hz, 2 H, 2 × $Ar_{(indazole)}$-CH), 7.32 (ddd, *J* = 8.8, 6.6, 1.1 Hz, 1 H, $Ar_{(indazole)}$-CH), 7.09 (dd, *J* = 8.3, 6.8 Hz, 1 H, $Ar_{(indazole)}$-CH), 7.06–7.01 (m, 2 H, 2 × Ar-CH), 6.81 (d, *J* = 8.7 Hz, 2 H, 2 × Ar-CH), 5.59 (d, *J* = 6.8 Hz, 2 H, C*H*HOC*H*H), 5.28 (d, *J* = 6.8 Hz, 2 H, CH*H*OCH*H*); ^{13}C NMR (101 MHz, CD_3OD) δ 159.0 (Ar-C_q-OH), 150.2 ($Ar_{(indazole)}$-C_q), 132.6 (Ar-C_q-C_q), 128.1 (2 × Ar-CH), 127.9 ($Ar_{(indazole)}$-CH), 124.7 ($Ar_{(indazole)}$-CH), 123.2 ($Ar_{(indazole)}$-C_q), 123.1 ($Ar_{(indazole)}$-CH), 121.8 ($Ar_{(indazole)}$-CH), 117.8 ($Ar_{(indazole)}$-CH), 116.6 (2 × Ar-CH), 83.6 (CH_2OCH_2), 69.2 (C_q); HRMS (TOF-MS-ES^+) *m/z* calcd for $C_{16}H_{15}N_2O_2^+$ $[M+H]^+$: 267.1134, found: 267.1148.

Notes:

***46** was further characterized by X-ray crystallography (see Fig. **S9**). Crystals suitable for X-ray analysis were grown by slow evaporation from CD_3OD.*

1-(3-(4-Methoxyphenyl)oxetan-3-yl)-1*H*-imidazole (47)

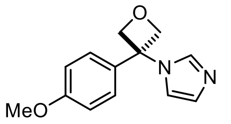

Prepared according to **deFS General Procedure A** under Ar at 70 °C for 2 h with a Celite work-up and using oxetane sulfonyl fluoride **1** (49.2 mg, 0.2 mmol, 1.0 equiv), K_2CO_3 (35.8 mg, 0.26 mmol, 1.3 equiv), imidazole (40.8 mg, 0.60 mmol, 3.0 equiv) and anhydrous MeCN (0.67 mL, 0.3 M). Purification by flash column chromatography (EtOAc) afforded oxetano-imidazole **47** as a colorless oil (31.0 mg, 67%). R_f = 0.21 (EtOAc); ^{1}H NMR (400 MHz, $CDCl_3$) δ 7.42 (s, 1 H, $Ar_{(imidazole)}$-CH), 7.18 (s, 1 H, $Ar_{(imidazole)}$-CH), 7.11–7.05 (m, 3 H, $Ar_{(imidazole)}$-CH + 2 × Ar-CH), 6.97–6.91 (m, 2 H, 2 × Ar-CH), 5.23 (s, 4 H, CH_2OCH_2), 3.83 (s, 3 H, OCH_3); The observed characterization data (R_f, ^{1}H) were consistent with that previously reported.[6]

4-(3-(1*H*-imidazol-1-yl)oxetan-3-yl)phenol (48)

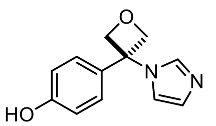

Prepared according to **deFS General Procedure A** under Ar at 70 °C for 2 h with a Celite work-up and using oxetane sulfonyl fluoride **2** (77.7 mg, 0.2 mmol, 1.0 equiv), K_2CO_3 (35.8 mg, 0.26 mmol, 1.3 equiv), imidazole (40.8 mg, 0.60 mmol, 3.0 equiv) and anhydrous MeCN (0.67 mL, 0.3 M). Purification by flash column

chromatography (5–10% MeOH/CH_2Cl_2) afforded oxetano-imidazole **48** as a white solid (38.4 mg, 89%). R_f = 0.35 (10% MeOH/CH_2Cl_2); mp = 166–169 °C; IR (film)/cm^{-1} 3125 (OH st), 2883, 1605, 1504, 1457, 1277, 1254, 1178, 988, 926, 844, 822, 747, 660, 561; ^{1}H NMR (400 MHz, CD_3OD) δ 7.68 (s, 1 H, $Ar_{(imidazole)}$-CH), 7.28 (s, 1 H, $Ar_{(imidazole)}$-CH), 7.12–7.03 (m, 3 H, $Ar_{(imidazole)}$-CH + 2 × Ar-CH), 6.87–6.79 (m, 2 H, 2 × Ar-CH), 5.23 (s, 4 H, CH_2OCH_2); ^{13}C NMR (101 MHz, CD_3OD) δ 159.1 (Ar-C_q-OH), 137.5 ($Ar_{(imidazole)}$-CH), 132.3 (Ar-C_q-C_q), 129.6 ($Ar_{(imidazole)}$-CH), 128.1 (2 × Ar-CH), 119.5 ($Ar_{(imidazole)}$-CH), 116.8 (2 × Ar-CH), 84.1 (CH_2OCH_2), 65.0 (C_q); HRMS (TOF-MS-ES$^+$) *m/z* calcd for $C_{12}H_{13}N_2O_2^+$ [M+H]$^+$: 217.0977, found: 217.0968.

Ethyl 1-(3-(4-hydroxyphenyl)oxetan-3-yl)-1*H*-imidazole-4-carboxylate (49) and ethyl 1-(3-(4-methoxyphenyl)oxetan-3-yl)-1*H*-imidazole-5-carboxylate (49')

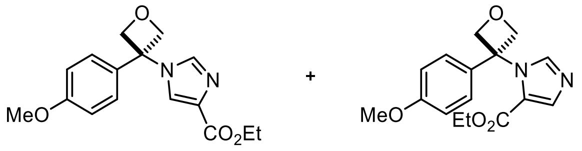

major 53:47 minor

Prepared according to **deFS General Procedure A** under Ar at 70 °C for 2 h with a Celite work-up and using oxetane sulfonyl fluoride **1** (49.2 mg, 0.2 mmol, 1.0 equiv), K_2CO_3 (35.8 mg, 0.26 mmol, 1.3 equiv), ethyl 1*H*-imidazole-4/5-carboxylate (84.1 mg, 0.60 mmol, 3.0 equiv) and anhydrous MeCN (0.67 mL, 0.3 M). Purification by flash column chromatography (80–100% EtOAc/pentane) afforded oxetano-imidazole **49'** as a colorless gum (16.5 mg, 27%) followed by oxetano-imidazole **49** as a colorless gum (20.1 mg, 33%).

Major regioisomer (**49**): R_f = 0.15 (EtOAc); IR (film)/cm^{-1} 2962, 2840, 1724 (C=O st), 1203, 1252, 1183, 1142, 1026, 834; ^{1}H NMR (400 MHz, $CDCl_3$) δ 7.71 (s, 1 H, $Ar_{(imidazole)}$-CH), 7.50 (s, 1 H, $Ar_{(imidazole)}$-CH), 7.12–7.04 (m, 2 H, 2 × Ar-CH), 7.00–6.92 (m, 2 H, 2 × Ar-CH), 5.28 (d, *J* = 6.9 Hz, 2 H, C*H*HOC*H*H), 5.18 (d, *J* = 6.9 Hz, 2 H, CH*H*OCH*H*), 4.38 (q, *J* = 7.1 Hz, 2 H, OC*H*$_2$CH$_3$), 3.84 (s, 3 H, OCH_3), 1.39 (t, *J* = 7.1 Hz, 3 H, OCH_2CH_3); ^{13}C NMR (126 MHz, $CDCl_3$) δ 162.7 (C_q=O), 160.1 (Ar-C_q-OCH_3), 136.7 ($Ar_{(imidazole)}$-CH), 134.6 (Ar-C_q-C_q) 130.5 ($Ar_{(imidazole)}$-C_q), 127.1 (2 × Ar-CH), 123.7 ($Ar_{(imidazole)}$-CH), 114.7 (2 × Ar-CH), 83.2 (CH_2OCH_2), 64.1 (C_q), 60.7 (OCH_2CH_3), 55.4 (OCH_3), 14.4 (OCH_2CH_3); HRMS (TOF-MS-ES$^+$) *m/z* calcd for $C_{16}H_{19}N_2O_4^+$ [M+H]$^+$: 303.1345, found: 303.1347.

Minor regioisomer (**49'**): R_f = 0.25 (EtOAc); IR (film)/cm^{-1} 2958, 2837, 1712 (C=O st), 1512, 1376, 1249, 1142, 829; ^{1}H NMR (400 MHz, $CDCl_3$) δ 7.89 (s, 1 H, $Ar_{(imidazole)}$-CH), 7.76–7.69 (m, 1 H, $Ar_{(imidazole)}$-CH), 7.05 (d, *J* = 8.9 Hz, 2 H, 2 × Ar-CH), 6.89 (d, *J* = 8.9 Hz, 1 H, 2 × Ar-CH), 5.36 (d, *J* = 7.1 Hz, 2 H, C*H*HOC*H*H), 5.10 (d, *J* = 7.2 Hz, 2 H, CH*H*OCH*H*), 4.16 (q, *J* = 7.1 Hz, 2 H, OC*H*$_2$CH$_3$), 1.25 (t, *J* = 7.1 Hz, 3 H, OCH_2CH_3); ^{13}C NMR (101 MHz, $CDCl_3$) δ 159.3 (C_q=O), 159.3 (Ar-C_q-OCH_3), 140.2 ($Ar_{(imidazole)}$-CH), 138.7 ($Ar_{(imidazole)}$-CH), 133.8 (Ar-C_q-C_q), 124.9 (2 × Ar-CH), 123.2 ($Ar_{(imidazole)}$-C_q), 114.2 (2 × Ar-CH), 83.3 (CH_2OCH_2), 63.7 (C_q), 60.7 (OCH_2CH_3), 55.3 (OCH_3), 14.1 (OCH_2CH_3); HRMS (TOF-MS-ES$^+$) *m/z* calcd for $C_{16}H_{19}N_2O_4^+$ [M+H]$^+$: 303.1345, found: 303.1339.

Regioisomers assigned from NOESY experiments (see pages S310 and S312 for spectra):

- *In the NOESY experiment for **49**, a nOe is observed between both imidazole CH environments (7.71 and 7.50 ppm) and a para-methoxy aromatic 2 × CH environment (7.09 ppm). Conversely in the NOESY experiment for **49'**, a nOe is only observed between one imidazole CH environment (7.69 ppm) and the corresponding para-methoxy 2 × CH environment (7.03 ppm), suggesting the other imidazole CH environment is too far away from the para-methoxy environment.*
- *As is common with other N-heterocycle amino-oxetanes described in this work, the more sterically hindered regioisomer exhibits a wider range in the chemical shift of the oxetane CH_2 environments, presumably due to the steric clash between these environments and the N-heterocycle substituent. In this case, the major, less sterically hindered regioisomer **49** has a*

narrower chemical shift range of the oxetane CH_2 environments (5.28 and 5.18 ppm) compared to the minor, more sterically hindered ***49'*** *(5.36 and 5.10 ppm).*

Ethyl 1-(3-(4-hydroxyphenyl)oxetan-3-yl)-1*H*-imidazole-4-carboxylate (50) and ethyl 1-(3-(4-hydroxyphenyl)oxetan-3-yl)-1*H*-imidazole-5-carboxylate (50')

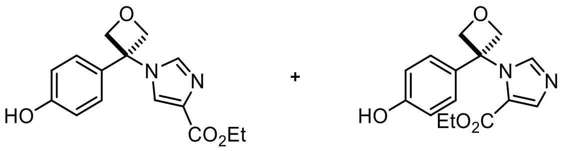

major 90:10 minor

Prepared according to **deFS General Procedure A** under Ar at 70 °C for 2 h with a Celite work-up and using oxetane sulfonyl fluoride **2** (77.7 mg, 0.2 mmol, 1.0 equiv), K_2CO_3 (35.8 mg, 0.26 mmol, 1.3 equiv), ethyl 1*H*-imidazole-4/5-carboxylate (84.1 mg, 0.60 mmol, 3.0 equiv) and anhydrous MeCN (0.67 mL, 0.3 M). Purification by flash column chromatography afforded a 90:10 regioisomeric mixture of oxetano-imidazoles **50** and **50'** as a white solid (47 mg, 82%). R_f = 0.30 (EtOH/CH_2Cl_2); mp = 173–175 °C; IR (film)/cm^{-1} 3066 (OH st), 2956, 1612, 1515, 1457, 1260, 980.3, 792, 731; ^{1}H NMR (400 MHz, $CDCl_3$) δ 7.88 (d, *J* = 1.4 Hz, 1 H, $Ar_{(imidazole)}$-CH, **50**), 7.72 (s, 0.1 H, $Ar_{(imidazole)}$-CH, **50'**), 7.37 (d, *J* = 1.5 Hz, 0.14 H, $Ar_{(imidazole)}$-CH, **50'**), 7.35 (d, *J* = 1.6 Hz, 1 H, $Ar_{(imidazole)}$-CH, **50**), 6.98 (d, *J* = 8.7 Hz, 2 H, 2 × Ar-CH, **50**), 6.92 (d, *J* = 8.8 Hz, 2 H, 2 × Ar-CH, **50**), 6.80 (d, *J* = 8.6 Hz, 0.2 H, 2 × Ar-CH, **50'**), 5.34 (d, *J* = 7.3 Hz, 0.2 H, C*H*HOC*H*H, **50'**) 5.30 (d, *J* = 7.0 Hz, 2 H, C*H*HOC*H*H, **50**), 5.14 (d, *J* = 7.1 Hz, 2 H, CH*H*OCH*H*, **50**), 5.07 (d, *J* = 7.0 Hz, 0.2 H, CH*H*OCH*H*, **50'**), 4.37 (q, *J* = 7.1 Hz, 2 H, OCH_2CH$_3$, **50**), 4.15 (q, *J* = 7.1 Hz, 0.2 H, OCH_2CH$_3$, **50'**), 3.90 (s, 1 H, OH, **50**) 1.36 (t, *J* = 7.1 Hz, 3 H, OCH$_2$CH_3, **50**), 1.24 (q, *J* = 6.9 Hz, 0.3 H, OCH$_2$CH_3, **50'**); ^{13}C NMR (101 MHz, $CDCl_3$) δ 162.6 (C_q=O), 157.5 (Ar-C_q-OH), 137.0 ($Ar_{(imidazole)}$-CH), 134.4 (Ar-C_q-C_q), 129.2 ($Ar_{(imidazole)}$-C_q), 127.3 (2 × Ar-CH), 123.6, 116.6 (2 × Ar-CH), 83.1 (CH_2OCH_2), 64.5 (C_q), 60.9 (OCH$_2$CH$_3$), 14.4 (OCH$_2$$CH_3$); HRMS (TOF-MS-ES$^+$) *m/z* calcd for $C_{15}H_{17}N_2O_4^+$ [M+H]$^+$: 289.1188, found: 289.1184.

Notes:

The OH signal observed for ***50*** *is of reduced intensity (integration = 0.5) presumably due to exchange with H_2O.*

Regioisomers assigned by analogy of ***49*** *and* ***49'*** *assignment:*

- *The OC**H**$_2$CH$_3$ ^{1}H NMR signal is very similar between major product* ***49*** *(4.38 ppm) and* ***50*** *(4.37 ppm), while the OCH$_2$C**H**$_3$ ^{1}H NMR signal is in good agreement between minor product* ***49'*** *(1.25 ppm) and* ***50'*** *(1.24 ppm)*

1-(3-(4-Methoxyphenyl)oxetan-3-yl)-1*H*-benzo[*d*]imidazole (51)

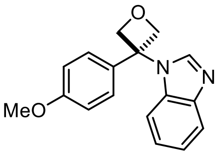

Prepared according to **deFS General Procedure A** under Ar at 70 °C for 2 h with a Celite work-up and using oxetane sulfonyl fluoride **1** (49.2 mg, 0.2 mmol, 1.0 equiv), K_2CO_3 (35.8 mg, 0.26 mmol, 1.3 equiv), benzimidazole (70.9 mg, 0.60 mmol, 3.0 equiv) and anhydrous MeCN (0.67 mL, 0.3 M). Purification by flash column chromatography (10–40% EtOAc/*n*-hexane) afforded oxetano-imidazole **51** as a colorless gum (28.5 mg, 51%). R_f = 0.07 (20% EtOAc/*n*-hexane); IR (film)/cm^{-1} 2957, 1610, 1512, 1484, 1457, 1250, 1183, 991, 743; ^{1}H NMR (400 MHz, $CDCl_3$) δ 7.88 (d, *J* = 8.1 Hz, 1 H, $Ar_{(imidazole)}$-CH), 7.85 (s, 1 H, $Ar_{(imidazole)}$-CH), 7.36–7.27 (m, 1 H, $Ar_{(imidazole)}$-CH), 7.25–7.20 (m, 1 H, $Ar_{(imidazole)}$-CH), 7.12–7.08 (m, 2 H, 2 × Ar-CH), 7.05 (d, *J* = 8.1 Hz, 1 H, $Ar_{(imidazole)}$-CH), 6.92–6.84 (m, 2 H, 2 × Ar-CH), 5.58 (d, *J* = 6.6 Hz, 2 H, C*H*HOC*H*H), 5.25 (d, *J* = 6.6 Hz, 2 H, CH*H*OCH*H*), 3.79 (s, 3 H, OCH_3); ^{13}C NMR (101 MHz, $CDCl_3$) δ 159.7 (Ar-C_q-OCH_3), 144.0 ($Ar_{(imidazole)}$-C_q), 141.2 ($Ar_{(imidazole)}$-CH), 132.8 (Ar-C_q-C_q), 132.1 ($Ar_{(imidazole)}$-C_q), 126.2 (2 × Ar-CH), 123.5 ($Ar_{(imidazole)}$-CH),

122.7 ($Ar_{(imidazole)}$-CH), 121.0 ($Ar_{(imidazole)}$-CH), 114.5 (2 × Ar-CH), 111.0 ($Ar_{(imidazole)}$-CH), 82.7 (CH_2OCH_2), 62.4 (C_q), 55.4 (OCH_3); HRMS (TOF-MS-ES$^+$) *m/z* calcd for $C_{17}H_{17}N_2O_2^+$ $[M+H]^+$: 281.1290, found: 281.1287.

7-(3-(4-Methoxyphenyl)oxetan-3-yl)-1,3-dimethyl-3,7-dihydro-1*H*-purine-2,6-dione (52) and 8-(3-(4-methoxyphenyl)oxetan-3-yl)-1,3-dimethyl-3,7-dihydro-1*H*-purine-2,6-dione (52')

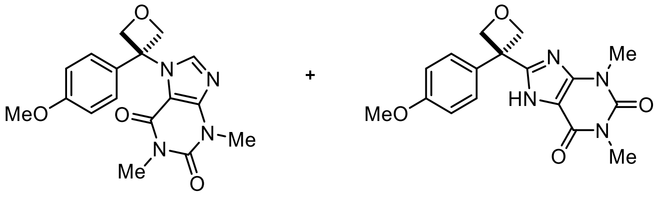

Prepared according to **deFS General Procedure A** under Ar at 70 °C for 2 h with a Celite work-up and using oxetane sulfonyl fluoride **1** (49.2 mg, 0.2 mmol, 1.0 equiv), K_2CO_3 (35.8 mg, 0.26 mmol, 1.3 equiv), theophylline (108.1 mg, 0.60 mmol, 3.0 equiv) and anhydrous MeCN (0.67 mL, 0.3 M). Purification by column chromatography (60–90% EtOAc/*n*-hexane) afforded diaryl-oxetane **52'** as an off-white solid (10.6 mg, 15%) followed by oxetano-imidazole **52** as a white solid (11.0 mg, 16%).

Major regioisomer (**52**): R_f = 0.14 (80% EtOAc/*n*-hexane); mp = 204–206 °C; IR (film)/cm^{-1} 2955, 1702 (C=O st), 1658 (C=O st), 1540, 1514, 1446, 1252, 1184, 986; ^{1}H NMR (400 MHz, $CDCl_3$) δ 7.70 (s, 1 H, $Ar_{(imidazole)}$-CH), 7.15 (d, *J* = 8.9 Hz, 2 H, 2 × Ar-CH), 6.88 (d, *J* = 8.9 Hz, 2 H, 2 × Ar-CH), 5.37 (d, *J* = 7.3 Hz, 2 H, C*H*HOC*H*H), 5.21 (d, *J* = 7.4 Hz, 2 H, CH*H*OCH*H*), 3.79 (s, 3 H, OCH_3), 3.63 (s, 3 H, NCH_3), 3.32 (s, 3 H, NCH_3); ^{13}C NMR (101 MHz, $CDCl_3$) δ 159.6 (Ar-C_q-OCH_3), 154.3 ($N(CH_3)C_q$=O), 151.6 ($N(CH_3)C_q$=O), 149.5 ($Ar_{(imidazole)}$-C_q), 139.1 ($Ar_{(imidazole)}$-CH), 132.5 (Ar-C_q-C_q), 125.5 (2 × Ar-CH), 114.5 (2 × Ar-CH), 107.1 ($Ar_{(imidazole)}$-C_q), 82.4 (CH_2OCH_2), 63.8 (C_q), 55.3 (OCH_3), 30.0 (NCH_3), 28.2 (NCH_3); HRMS (TOF-MS-ES$^+$) *m/z* calcd for $C_{17}H_{19}N_4O_4^+$ $[M+H]^+$: 343.1406, found: 343.1395.

Minor regioisomer (**52'**): R_f = 0.23 (80% EtOAc/*n*-hexane); mp = 260–263 °C; IR (film)/cm^{-1} 2924, 2361, 1702 (C=O st), 1649 (C=O st), 1510, 1250, 985; ^{1}H NMR (400 MHz, $CDCl_3$) δ 11.66 (s, 1 H, NH), 7.21 (d, *J* = 8.7 Hz, 2 H, 2 × Ar-CH), 6.89 (d, *J* = 8.7 Hz, 2 H, 2 × Ar-CH), 5.47 (d, *J* = 5.9 Hz, 2 H, C*H*HOC*H*H), 5.22 (d, *J* = 6.0 Hz, 2 H, CH*H*OCH*H*), 3.80 (s, 3 H, OCH_3), 3.66 (s, 3 H, NCH_3), 3.38 (s, 3 H, NCH_3); ^{13}C NMR (101 MHz, $CDCl_3$) δ 159.1 (Ar-C_q-OCH_3), 157.4 ($Ar_{(imidazole)}$-C_q-C_q), 155.7 ($N(CH_3)C_q$=O), 151.5 ($N(CH_3)C_q$=O), 149.1 ($Ar_{(imidazole)}$-C_q) 133.1 (Ar-C_q-C_q), 127.2 (2 × Ar-CH), 114.5 (2 × Ar-CH), 107.2 ($Ar_{(imidazole)}$-C_q), 81.6 (CH_2OCH_2), 55.4 (OCH_3), 48.0 (C_q), 30.4 (NCH_3), 28.4 (NCH_3); HRMS (TOF-MS-ES$^+$) *m/z* calcd for $C_{17}H_{19}N_4O_4^+$ $[M+H]^+$: 343.1406, found: 343.1401.

Notes:

*Major regioisomer (**52**) assumed to have alkylated on the N^7 due to overwhelming established literature precedent of theophylline alkylation occurring on this nitrogen.*

*Minor regioisomer (**52'**) assigned as a diaryl oxetane due to distinct oxetane quaternary carbon ^{13}C NMR shift (48.0 ppm) sitting within the typical range for diaryl oxetanes (46.0–51.0 ppm).*

1-(3-(4-Methoxyphenyl)oxetan-3-yl)-1*H*-1,2,4-triazole (53) and 4-(3-(4-methoxyphenyl)oxetan-3-yl)-4*H*-1,2,4-triazole (53')

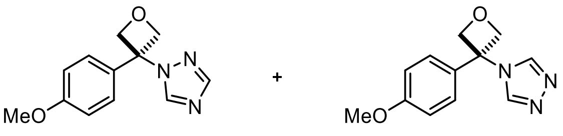

major 55:45 minor

Prepared according to **deFS General Procedure A** under Ar at 70 °C for 2 h with a Celite work-up and using oxetane sulfonyl fluoride **1** (49.2 mg, 0.2 mmol, 1.0 equiv), K_2CO_3 (35.8 mg, 0.26 mmol, 1.3 equiv), 1/4*H*-1,2,4-triazole (41.4 mg, 0.60 mmol, 3.0 equiv) and anhydrous MeCN (0.67 mL, 0.3 M). Purification by flash column chromatography (0–10% MeOH/EtOAc) afforded oxetano-triazole **53** as a colorless gum (20.3 mg, 44%) followed by oxetano-triazole **53'** as pale-yellow crystals (16.4 mg, 35%).

Major regioisomer (**53**): R_f = 0.24 (EtOAc); IR (film)/cm^{-1} 2959, 2887, 1612, 1514, 1251, 1183, 1026, 991, 831; ^{1}H NMR (400 MHz, $CDCl_3$) δ 8.07 (s, 1 H, $Ar_{(triazole)}$-CH), 7.70 (s, 1 H, $Ar_{(triazole)}$-CH), 7.20–7.12 (m, 2 H, 2 × Ar-CH), 7.01–6.93 (m, 2 H, 2 × Ar-CH), 5.44 (d, *J* = 6.7 Hz, 2 H, C*H*HOC*H*H), 5.25 (d, *J* = 6.7 Hz, 2 H, CH*H*OCH*H*), 3.84 (s, 3 H, OCH_3); ^{13}C NMR (101 MHz, $CDCl_3$) δ 160.1 (Ar-C_q-OCH_3), 152.4 ($Ar_{(triazole)}$-CH), 142.6 ($Ar_{(triazole)}$-CH), 130.2 (Ar-C_q-C_q), 127.6 (2 × Ar-CH), 114.7 (2 × Ar-CH), 82.0 (CH_2OCH_2), 65.4 (C_q), 55.4 (OCH_3); HRMS (TOF-MS-ES$^+$) *m/z* calcd for $C_{12}H_{14}N_3O_2^+$ [M+H]$^+$: 232.1086, found: 232.1088.

Minor regioisomer (**53'**): 0.29 (10% MeOH/EtOAc); IR (film) cm^{-1} 2957, 2933, 1614, 1518, 1461, 1257, 1185, 1019, 974, 822, 646; ^{1}H NMR (400 MHz, $CDCl_3$) δ 8.21 (s, 2 H, 2 × $Ar_{(triazole)}$-CH), 7.14–7.06 (m, 2 H, 2 × Ar-CH), 7.03–6.94 (m, 2 H, 2 × Ar-CH), 5.38–5.32 (m, 2 H, C*H*HOC*H*H), 5.13–5.07 (m, 2 H, CH*H*OCH*H*), 3.85 (s, 3 H, OCH_3); ^{13}C NMR (101 MHz, $CDCl_3$) δ 160.4 (Ar-C_q-OCH_3), 141.4 (2 × $Ar_{(triazole)}$-CH), 129.2 (Ar-C_q-C_q), 127.3 (2 × Ar-CH), 115.0 (2 × Ar-CH), 83.1 (CH_2OCH_2), 63.4 (C_q), 55.5 (OCH_3); HRMS (TOF-MS-ES$^+$) *m/z* calcd for $C_{12}H_{14}N_3O_2^+$ [M+H]$^+$: 232.1086, found: 232.1090.

4-(3-(1*H*-1,2,4-Triazol-1-yl)oxetan-3-yl)phenol (54) and 4-(3-(4*H*-1,2,4-Triazol-4-yl)oxetan-3-yl)phenol (54')

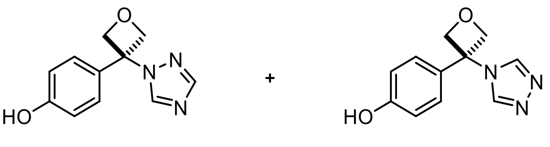

major 90:10 minor

Prepared according to **deFS General Procedure A** under Ar at 70 °C for 2 h with a Celite work-up and using oxetane sulfonyl fluoride **2** (77.7 mg, 0.2 mmol, 1.0 equiv), K_2CO_3 (35.8 mg, 0.26 mmol, 1.3 equiv), 1/4*H*-1,2,4-triazole (41.4 mg, 0.60 mmol, 3.0 equiv) and anhydrous MeCN (0.67 mL, 0.3 M). Purification by flash column chromatography (EtOAc) afforded oxetano-triazole **54** as a white solid (35.7 mg, 82%). R_f = 0.26 (70% EtOAc/pentane); mp = 138–142 °C; IR (film)/cm^{-1} 3111 (OH st), 2889, 1508, 1482, 1379, 1271, 1146, 885, 826, 734, 649; ^{1}H NMR (400 MHz, $CDCl_3$) δ 8.24 (s, 1 H, OH), 8.13 (s, 1 H, $Ar_{(triazole)}$-CH), 7.69 (s, 1 H, $Ar_{(triazole)}$-CH), 7.13–7.05 (m, 2 H, 2 × Ar-CH), 6.99–6.89 (m, 2 H, 2 × Ar-CH), 5.45 (d, *J* = 6.7 Hz, 2 H, C*H*HOC*H*H), 5.32–5.24 (m, 2 H, CH*H*OCH*H*); ^{13}C NMR (101 MHz, $CDCl_3$) δ 157.7 (Ar-C_q-OH), 152.0 ($Ar_{(triazole)}$-CH), 142.6 ($Ar_{(triazole)}$-CH), 128.9 (Ar-C_q-C_q), 128.0 (2 × Ar-CH), 116.5 (2 × Ar-CH), 82.0 (CH_2OCH_2), 65.7 (C_q); HRMS (TOF-MS-ES$^+$) *m/z* calcd for $C_{11}H_{12}N_3O_2^+$ [M+H]$^+$: 218.0937, found: 218.0947.

Notes:

*Although both products were observed in the crude ^{1}H NMR sample, only major regioisomer **54** was afforded after purification. The observed signals for minor regioisomer **54'** in the crude sample are provided below:*

^{1}H NMR (400 MHz, $CDCl_3$) δ 8.28 (s, 2 H, 2 × $Ar_{(triazole)}$-CH), 7.00 (d, *J* = 8.7 Hz, 2 H, 2 × Ar-CH), 6.94 (d, *J* = 8.7 Hz, 2 H, 2 × Ar-CH), 5.38 (d, *J* = 7.4 Hz, 2 H, C*H*HOC*H*H), 5.06 (d, *J* = 7.7 Hz, 1 H, CH*H*OCH*H*).

1-(3-(4-Methoxyphenyl)oxetan-3-yl)-1*H*-1,2,3-triazole (55) and 2-(3-(4-methoxyphenyl)oxetan-3-yl)-2*H*-1,2,3-triazole (55')

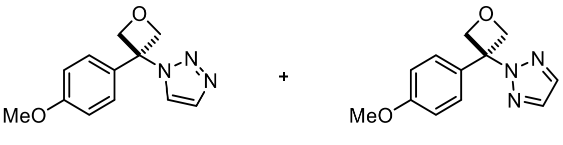

Prepared according to **deFS General Procedure A** under Ar at 70 °C for 2 h with a Celite work-up and using oxetane sulfonyl fluoride **1** (49.2 mg, 0.2 mmol, 1.0 equiv), K_2CO_3 (35.8 mg, 0.26 mmol, 1.3 equiv), 1/2*H*-1,2,3-triazole (41.4 mg, 0.60 mmol, 3.0 equiv) and anhydrous MeCN (0.67 mL, 0.3 M). Purification by flash column chromatography (40–100% EtOAc/pentane) afforded oxetano-triazole **55'** as a white solid (14.6 mg, 32%) followed by oxetano-triazole **55** as a white solid (24.0 mg, 52%).

Major regioisomer (**55**): R_f = 0.53 (EtOAc); mp = 99–101 °C; IR (film)/cm^{-1} 2958, 2887, 1610, 1513, 1301, 1250, 1182, 1026, 985, 829; ^{1}H NMR (400 MHz, $CDCl_3$) δ 7.75 (d, *J* = 1.1 Hz, 1 H, $Ar_{(triazole)}$-CH), 7.33 (d, *J* = 1.1 Hz, 1 H, $Ar_{(triazole)}$-CH), 7.16–7.08 (m, 2 H, 2 × Ar-CH), 6.97–6.89 (m, 2 H, 2 × Ar-CH), 5.57 (d, *J* = 6.9 Hz, 2 H, C*H*HOC*H*H), 5.26 (d, *J* = 7.2 Hz, 2 H, CH*H*OCH*H*), 3.82 (s, 3 H, OCH_3); ^{13}C NMR (101 MHz, $CDCl_3$) δ 160.0 (Ar-C_q-OCH_3), 134.1 ($Ar_{(triazole)}$-CH), 131.2 (Ar-C_q-C_q), 127.0 (2 × Ar-CH), 122.7 ($Ar_{(triazole)}$-CH), 114.6 (2 × Ar-CH), 82.0 (CH_2OCH_2), 66.0 (C_q), 55.4 (OCH_3); HRMS (TOF-MS-ES$^+$) *m/z* calcd for $C_{12}H_{14}N_3O_2^+$ $[M+H]^+$: 232.1086, found: 232.1076.

Minor regioisomer (**55'**): R_f = 0.43 (40% EtOAc/*n*-hexane); mp = 62–65 °C; IR (film)/cm^{-1} 2960, 2889, 1610, 1512, 1248, 1181, 1028, 983, 960, 824; ^{1}H NMR (400 MHz, $CDCl_3$) δ 7.72 (s, 2 H, 2 × $Ar_{(triazole)}$-CH), 7.09–7.01 (m, 2 H, 2 × Ar-CH), 6.91–6.83 (m, 2 H, 2 × Ar-CH), 5.60 (d, *J* = 6.9 Hz, 2 H, C*H*HOC*H*H), 5.26 (d, *J* = 6.9 Hz, 2 H, CH*H*OCH*H*), 3.79 (s, 3 H, OCH_3); ^{13}C NMR (101 MHz, $CDCl_3$) δ 159.5 (Ar-C_q-OCH_3), 134.7 (2 × $Ar_{(triazole)}$-CH), 132.3 (Ar-C_q-C_q), 126.6 (2 × Ar-CH), 114.1 (2 × Ar-CH), 81.3 (CH_2OCH_2), 69.6 (C_q), 55.3 (OCH_3); HRMS (TOF-MS-ES$^+$) *m/z* calcd for $C_{12}H_{14}N_3O_2^+$ $[M+H]^+$: 232.1086, found: 232.1076.

4-(3-(1*H*-1,2,3-Triazol-1-yl)oxetan-3-yl)phenol (56) and 4-(3-(2*H*-1,2,3-triazol-2-yl)oxetan-3-yl)phenol (56')

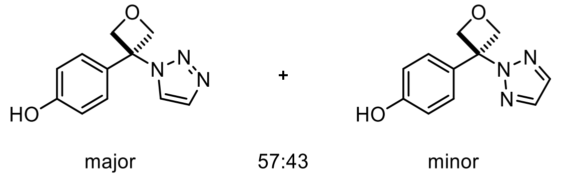

Prepared according to **deFS General Procedure A** under Ar at 70 °C for 2 h with a Celite work-up and using oxetane sulfonyl fluoride **2** (77.7 mg, 0.2 mmol, 1.0 equiv), K_2CO_3 (35.8 mg, 0.26 mmol, 1.3 equiv), 1/2*H*-1,2,3-triazole (41.4 mg, 0.60 mmol, 3.0 equiv) and anhydrous MeCN (0.67 mL, 0.3 M). Purification by flash column chromatography (50–100% Et_2O/pentane) afforded oxetano-

triazole **56'** as a white solid (16.3 mg, 38%) followed by oxetano-triazole **56** as a white solid (21.7 mg, 50%).

Major regioisomer (**56**): R$_f$ = 0.21 (Et_2O); mp = 202–203 °C; IR (film)/cm^{-1} 3338 (OH st), 3036, 2246, 1605, 1519, 1277, 1228, 1072, 993, 800, 781; ^{1}H NMR (400 MHz, CD_3OD) δ 7.87 (d, *J* = 1.2 Hz, 1 H, Ar$_{(triazole)}$-CH), 7.78 (d, *J* = 1.2 Hz, 1 H, Ar$_{(triazole)}$-CH), 7.11 (d, *J* = 8.7 Hz, 2 H, 2 × Ar-CH), 6.83 (d, *J* = 8.7 Hz, 2 H, 2 × Ar-CH), 5.49 (d, *J* = 7.0 Hz, 2 H, C*H*HOC*H*H), 5.30 (d, *J* = 7.0 Hz, 2 H, CH*H*OCH*H*); ^{13}C NMR (101 MHz, CD_3OD) δ 159.3 (Ar-C$_q$-OH), 134.7 (Ar$_{(triazole)}$-CH), 131.4 (Ar-*C*$_q$-C$_q$), 128.2 (2 × Ar-CH), 125.1 (Ar$_{(triazole)}$-CH), 116.8 (2 × Ar-CH), 82.9 (CH_2OCH_2), 68.0 (C$_q$); HRMS (TOF-MS-ES$^+$) *m/z* calcd for $C_{11}H_{12}N_3O_2^+$ [M+H]$^+$: 218.0930, found: 218.0921.

Minor regioisomer (**56'**): R$_f$ = 0.27 (50% Et_2O/pentane); mp = 150–151 °C; IR (film)/cm^{-1} 3362 (OH st), 1608, 1595, 1515, 1353, 1262, 1234, 1169, 969, 811; ^{1}H NMR (400 MHz, $CDCl_3$) δ 7.74 (s, 2 H, 2 × Ar$_{(triazole)}$-CH), 6.95 (d, *J* = 8.7 Hz, 2 H, 2 × Ar-CH), 6.73 (d, *J* = 8.8 Hz, 2 H, 2 × Ar-CH), 5.66–5.57 (m, 3 H, C*H*HOC*H*H + OH), 5.25 (d, *J* = 7.0 Hz, 2 H, CH*H*OCH*H*); ^{13}C NMR (101 MHz, $CDCl_3$) δ 155.8 (Ar-C$_q$-OH), 134.7 (2 × Ar$_{(triazole)}$-CH), 132.0 (Ar-*C*$_q$-C$_q$), 126.7 (2 × Ar-CH), 115.7 (2 × Ar-CH), 81.3 (CH_2OCH_2), 69.6 (C$_q$); HRMS (TOF-MS-ES$^+$) *m/z* calcd for $C_{11}H_{12}N_3O_2^+$ [M+H]$^+$: 218.0930, found: 218.0932.

1-(3-(4-Methoxyphenyl)oxetan-3-yl)-1*H*-benzo[*d*][1,2,3]triazole (57) and 2-(3-(4-methoxyphenyl)oxetan-3-yl)-2*H*-benzo[*d*][1,2,3]triazole (57')

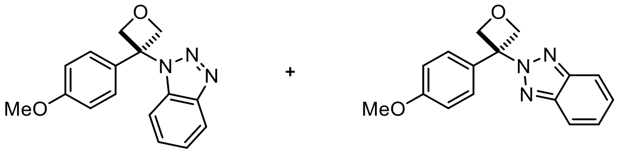

Prepared according to **deFS General Procedure A** under Ar at 70 °C for 2 h with a Celite work-up and using oxetane sulfonyl fluoride **1** (49.2 mg, 0.2 mmol, 1.0 equiv), K_2CO_3 (35.8 mg, 0.26 mmol, 1.3 equiv), 1/2*H*-benzo[*d*][1,2,3]triazole (71.5 mg, 0.60 mmol, 3.0 equiv) and anhydrous MeCN (0.67 mL, 0.3 M). Purification by flash column chromatography (20–40% EtOAc/*n*-hexane) afforded oxetano-triazole **57'** as a white solid (13.6 mg, 24%) followed by oxetano-triazole **57** as a white solid (29.5 mg, 52%).

Major regioisomer (**57**): R$_f$ = 0.14 (30% EtOAc/*n*-hexane); mp = 130–133 °C; IR (film)/cm^{-1} 2959, 2888, 1611, 1514, 1252, 1183, 1028, 830, 747; ^{1}H NMR (400 MHz, $CDCl_3$) δ 8.18–8.09 (m, 1 H, Ar$_{(triazole)}$-CH), 7.42–7.33 (m, 2 H, 2 × Ar$_{(triazole)}$-CH), 7.13–7.05 (m, 2 H, 2 × Ar-CH), 7.03–6.94 (m, 1 H, Ar$_{(triazole)}$-CH), 6.91–6.82 (m, 2 H, 2 × Ar-CH), 5.79 (d, *J* = 6.8 Hz, 2 H, C*H*HOC*H*H), 5.34 (d, *J* = 6.8 Hz, 2 H, CH*H*OCH*H*), 3.78 (s, 3 H, OCH_3); ^{13}C NMR (101 MHz, $CDCl_3$) δ 159.7 (Ar-C$_q$-OCH_3), 146.5 (Ar$_{(triazole)}$-C$_q$), 131.9 (Ar$_{(triazole)}$-C$_q$), 131.3 (Ar-*C*$_q$-C$_q$), 127.7 (Ar$_{(triazole)}$-CH), 126.3 (2 × Ar-CH), 124.2 (Ar$_{(triazole)}$-CH), 120.5 (Ar$_{(triazole)}$-CH), 114.5 (2 × Ar-CH), 110.0 (Ar$_{(triazole)}$-CH), 81.6 (CH_2OCH_2), 65.5 (C$_q$), 55.3 (OCH_3); HRMS (TOF-MS-ES$^+$) *m/z* calcd for $C_{16}H_{16}N_3O_2^+$ [M+H]$^+$: 282.1243, found: 282.1247.

Minor regioisomer (**57'**): R$_f$ = 0.32 (30% EtOAc/*n*-hexane); mp = 98–100 °C; IR (film)/cm^{-1} 2960, 2891, 1612, 1514, 1251, 1183, 1030, 982, 828, 743; ^{1}H NMR (400 MHz, $CDCl_3$) δ 7.89 (dd, *J* = 6.6, 3.1 Hz, 2 H, 2 × Ar$_{(triazole)}$-CH), 7.42 (dd, *J* = 6.6, 3.1 Hz, 2 H, Ar$_{(triazole)}$-CH), 7.15 (d, *J* = 8.8 Hz, 2 H, 2 × Ar-CH), 6.87 (d, *J* = 8.9 Hz, 2 H, 2 × Ar-CH), 5.79 (d, *J* = 7.0 Hz, 2 H, C*H*HOC*H*H), 5.39 (d, *J* = 7.0 Hz, 2 H, CH*H*OCH*H*), 3.77 (s, 3 H, OCH_3); ^{13}C NMR (101 MHz, $CDCl_3$) δ 159.7 (Ar-C$_q$-OCH_3), 144.4 (2 × Ar$_{(triazole)}$-C$_q$), 131.9 (Ar-*C*$_q$-C$_q$), 126.8 (2 × Ar-CH), 126.7 (2 × Ar$_{(triazole)}$-CH), 118.3 (2 × Ar$_{(triazole)}$-CH), 114.2 (2 × Ar-CH), 81.4 (CH_2OCH_2), 71.1 (C$_q$), 55.3 (OCH_3); HRMS (TOF-MS-ES$^+$) *m/z* calcd for $C_{16}H_{16}N_3O_2^+$ [M+H]$^+$: 282.1243, found: 282.1234.

4-(3-(1*H*-Benzo[*d*][1,2,3]triazol-1-yl)oxetan-3-yl)phenol (58) and 4-(3-(2*H*-benzo[*d*][1,2,3]triazol-2-yl)oxetan-3-yl)phenol (58')

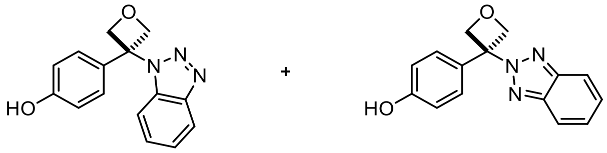

major 67:33 minor

Prepared according to **deFS General Procedure A** under Ar at 70 °C for 2 h with a Celite work-up and using oxetane sulfonyl fluoride **2** (77.7 mg, 0.2 mmol, 1.0 equiv), K_2CO_3 (35.8 mg, 0.26 mmol, 1.3 equiv), 1/2*H*-benzo[*d*][1,2,3]triazole (71.5 mg, 0.60 mmol, 3.0 equiv) and anhydrous MeCN (0.67 mL, 0.3 M). Purification by flash column chromatography (40–60% Et_2O/pentane) afforded oxetano-triazole **58'** as a white solid (14.5 mg, 27%) followed by oxetano-triazole **58** as a white solid (29.0 mg, 54%).

Major regioisomer (**58**): R_f = 0.11 (50% Et_2O/pentane); mp = 199–201 °C; IR (film)/cm^{-1} 3068 (OH st), 2342, 1560, 1517, 1448, 1239, 1150, 1046, 984, 746; ^{1}H NMR (400 MHz, CD_3OD) δ 8.07–8.02 (m, 1 H, $Ar_{(triazole)}$-CH), 7.48–7.39 (m, 2 H, 2 × $Ar_{(triazole)}$-CH), 7.25–7.17 (m, 1 H, $Ar_{(triazole)}$-CH), 7.07 (d, *J* = 8.7 Hz, 2 H, 2 × Ar-CH), 6.78 (d, *J* = 8.7 Hz, 2 H, 2 × Ar-CH), 5.72 (d, *J* = 6.9 Hz, 2 H, C*H*HOC*H*H), 5.39 (d, *J* = 6.9 Hz, 2 H, CH*H*OCH*H*); ^{13}C NMR (101 MHz, CD_3OD) δ 159.1 (Ar-C_q-OH), 147.3 ($Ar_{(triazole)}$-C_q), 133.2 ($Ar_{(triazole)}$-C_q), 131.1 (Ar-C_q-C_q), 129.1 ($Ar_{(triazole)}$-CH), 127.7 (2 × Ar-CH), 125.9 ($Ar_{(triazole)}$-CH), 120.5 ($Ar_{(triazole)}$-CH), 116.8 (2 × Ar-CH), 112.0 ($Ar_{(triazole)}$-CH), 82.2 (CH_2OCH_2), 67.5 (C_q); HRMS (TOF-MS-ES$^+$) *m/z* calcd for $C_{15}H_{14}N_3O_2^+$ [M+H]$^+$: 268.1086, found: 268.1076.

Minor regioisomer (**58'**): R_f = 0.32 (50% Et_2O/pentane); mp = 176–179 °C; IR (film)/cm^{-1} 3226 (OH st), 3038, 2402, 1584, 1511, 1448, 1265, 1228, 972, 744; ^{1}H NMR (400 MHz, CD_3OD) δ 7.90 (dd, *J* = 6.6, 3.1 Hz, 2 H, 2 × $Ar_{(triazole)}$-CH), 7.46 (dd, *J* = 6.6, 3.1 Hz, 2 H, 2 × $Ar_{(triazole)}$-CH), 7.13–7.04 (m, 2 H, 2 × Ar-CH), 6.82–6.72 (m, 2 H, 2 × Ar-CH), 5.72 (d, *J* = 7.0 Hz, 2 H, C*H*HOC*H*H), 5.40 (d, *J* = 7.1 Hz, 2 H, CH*H*OCH*H*); ^{13}C NMR (101 MHz, CD_3OD) δ 157.6 (Ar-C_q-OH), 144.2 (2 × $Ar_{(triazole)}$-C_q), 130.4 (Ar-C_q-C_q), 126.7 (2 × Ar-CH), 126.5 (2 × $Ar_{(triazole)}$-CH), 117.7 (2 × $Ar_{(triazole)}$-CH), 115.1 (2 × Ar-CH), 80.9 (C_q), 71.4 (C_q); HRMS (TOF-MS-ES$^+$) *m/z* calcd for $C_{15}H_{14}N_3O_2^+$ [M+H]$^+$: 268.1086, found: 268.1081.

3-Azido-3-(4-methoxyphenyl)oxetane (59)

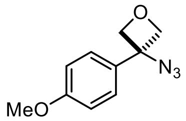

Oxetane sulfonyl fluoride **1** (49 mg, 0.2 mmol, 1.0 equiv) was added to a flame-dried vial and sealed. Anhydrous MeCN (0.67 mL, 0.3 M) was added followed by $TMSN_3$ (32 µL, 0.24 mmol, 1.2 equiv) in a fast dropwise manner. The reaction mixture was stirred at 70 °C for 2 h. After cooling to 25 °C, the reaction mixture was quenched with aq. 1 M NaOH (10 mL) and stirred for 5 min. The reaction mixture was diluted with CH_2Cl_2 (10 mL), the phases separated, and the aqueous layer was extracted with CH_2Cl_2 (3 × 10 mL). The combined organic layers were dried over anhydrous Na_2SO_4, filtered, and concentrated *in vacuo* to afford oxetane azide **59** as a clear, colorless gum (35 mg, 85%). R_f = 0.39 (20% EtOAc/*n*-hexane); IR (film)/cm^{-1} 2955, 2878, 2840, 2104 (NºNºN st), 1611, 1514, 1301, 1249, 1181, 1028, 985, 631, 548; ^{1}H NMR (400 MHz, $CDCl_3$) δ 7.40–7.29 (m, 2 H, 2 × Ar-CH), 6.96 (d, *J* = 8.5 Hz, 2 H, 2 × Ar-CH), 5.00 (d, *J* = 6.8 Hz, 2 H, C*H*HOC*H*H), 4.95 (d, *J* = 6.8 Hz, 2 H, C*H*HOC*H*H), 3.83 (s, 3 H, OCH_3); ^{13}C NMR (101 MHz, $CDCl_3$) δ 159.6 (Ar-C_q-OCH_3), 130.6 (Ar-C_q-C_q), 126.9 (2 × Ar-CH), 114.3 (2 × Ar-CH), 82.0 (CH_2OCH_2), 66.3 (C_q), 55.4 (OCH_3); HRMS (FTMS–APCI$^+$) *m/z* calcd for $C_{10}H_{11}O_2^+$ [M–N_3]$^+$: 163.0754; found 163.0753.

2-(3-(4-Methoxyphenyl)oxetan-3-yl)-5-methyl-2*H*-tetrazole (60) and 1-(3-(4-methoxyphenyl)oxetan-3-yl)-5-methyl-1*H*-tetrazole (60')

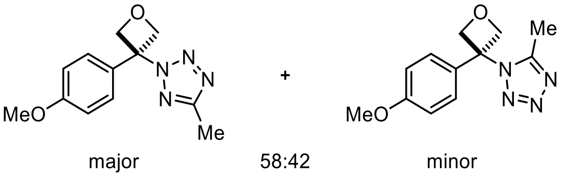

Prepared according to **deFS General Procedure A** under Ar at 70 °C for 2 h with a Celite work-up and using oxetane sulfonyl fluoride **1** (49.2 mg, 0.2 mmol, 1.0 equiv), K_2CO_3 (35.8 mg, 0.26 mmol, 1.3 equiv), 5-methyl-2/1*H*-tetrazole (50.5 mg, 0.60 mmol, 3.0 equiv) and anhydrous MeCN (0.67 mL, 0.3 M). Purification by flash column chromatography (40–60% EtOAc/*n*-hexane) afforded oxetano-tetrazole **60** as a white solid (23.8 mg, 48%) followed by oxetano-tetrazole **60'** as a white solid (19.3 mg, 39%).

Major regioisomer (**60**): R_f = 0.29 (40% EtOAc/*n*-hexane); IR (film)/cm^{-1} 2962, 2838, 1613, 1514, 1303, 1252, 1182, 1027, 986, 831; ^{1}H NMR (400 MHz, $CDCl_3$) δ 7.14 (d, *J* = 8.8 Hz, 2 H, 2 × Ar-CH), 6.90 (d, *J* = 8.8 Hz, 2 H, 2 × Ar-CH), 5.59 (d, *J* = 7.1 Hz, 2 H, C*H*HOC*H*H), 5.33 (d, *J* = 7.0 Hz, 2 H, CH*H*OCH*H*), 3.80 (s, 3 H, OCH_3), 2.56 (s, 3 H, C_q-CH_3); ^{13}C NMR (101 MHz, $CDCl_3$) δ 163.3 ($Ar_{(tetrazole)}$-C_q), 159.9 (Ar-C_q-OCH_3), 130.3 (Ar-C_q-C_q), 126.8 (2 × Ar-CH), 114.4 (2 × Ar-CH), 80.7 (CH_2OCH_2), 69.4 (C_q), 55.4 (OCH_3), 11.0 (C_q-CH_3); HRMS (TOF-MS-ES$^+$) *m/z* calcd for $C_{12}H_{15}N_4O_2^+$ $[M+H]^+$: 247.1195, found: 247.1205.

Minor regioisomer (**60'**): R_f = 0.063 (40% EtOAc/*n*-hexane); IR (film)/cm^{-1} 2961, 2891, 2361, 1611, 1515, 1254, 1185, 1026, 989, 831; ^{1}H NMR (400 MHz, $CDCl_3$) δ 7.07–7.00 (m, 2 H, 2 × Ar-CH), 6.95–6.89 (m, 2 H, 2 × Ar-CH), 5.56 (d, *J* = 6.5 Hz, 2 H, C*H*HOC*H*H), 5.15 (d, *J* = 6.5 Hz, 2 H, CH*H*OCH*H*), 3.81 (s, 3 H, OCH_3), 2.23 (s, 3 H, C_q-CH_3); ^{13}C NMR (101 MHz, $CDCl_3$) δ 160.1 (Ar-C_q-OCH_3), 151.4 ($Ar_{(tetrazole)}$-C_q), 129.8 (Ar-C_q-C_q), 125.8 (2 × Ar-CH), 114.8 (2 × Ar-CH), 81.1 (CH_2OCH_2), 64.2 (C_q), 55.4 (OCH_3), 9.5 (C_q-CH_3); HRMS (TOF-MS-ES$^+$) *m/z* calcd for $C_{12}H_{15}N_4O_2^+$ $[M+H]^+$: 247.1195, found: 247.1189.

Notes:

Melting points were not measured for any of the tetrazole products due to nitrogen content.

60' *was further characterized by X-ray crystallography (see Fig.* ***S10****). Crystals suitable for X-ray analysis were grown by slow evaporation from $CDCl_3$.*

4-(3-(5-Methyl-2*H*-tetrazol-2-yl)oxetan-3-yl)phenol (61) and 4-(3-(5-Methyl-1*H*-tetrazol-1-yl)oxetan-3-yl)phenol (61')

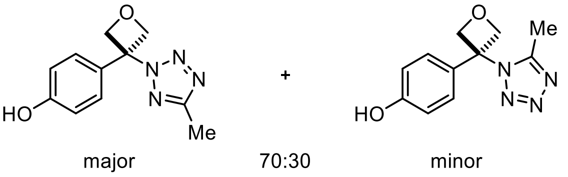

Prepared according to **deFS General Procedure A** under Ar at 70 °C for 2 h with a Celite work-up and using oxetane sulfonyl fluoride **2** (77.7 mg, 0.2 mmol, 1.0 equiv), K_2CO_3 (35.8 mg, 0.26 mmol, 1.3 equiv), 5-methyl-2/1*H*-tetrazole (50.5 mg, 0.60 mmol, 3.0 equiv) and anhydrous MeCN (0.67 mL, 0.3 M). Purification by flash column chromatography (30–70% EtOAc/pentane) afforded oxetano-tetrazole **61** as a white solid (30.0 mg, 65%) followed by oxetano-tetrazole **61'** as a white solid (12.5 mg, 27%).

Major regioisomer (**61**): R_f = 0.58 (70% EtOAc/pentane); IR (film)/cm^{-1} 3176 (OH st), 2889, 1610, 1523, 1284, 1232, 1180, 982, 831, 744; ^{1}H NMR (400 MHz, $CDCl_3$) δ 7.09 (d, *J* = 8.7 Hz, 2 H, 2 × Ar-CH), 6.83 (d, *J* = 8.7 Hz, 2 H, 2 × Ar-CH), 5.58 (d, *J* = 7.2 Hz, 2 H, C*H*HOC*H*H), 5.33 (d, *J* = 6.8 Hz, 3 H, CH*H*OCH*H* + OH), 2.57 (s, 3 H, C_q-CH_3); ^{13}C NMR (101 MHz, $CDCl_3$) δ 163.3 ($Ar_{(tetrazole)}$-C_q), 156.2 (Ar-C_q-OH), 130.3 (Ar-C_q-C_q), 127.1 (2 × Ar-CH), 115.9 (2 × Ar-CH), 80.7 (CH_2OCH_2), 69.4 (C_q), 11.0 (C_q-CH_3); HRMS (TOF-MS-ES$^+$) *m/z* calcd for $C_{11}H_{13}N_4O_2^+$ $[M+H]^+$: 233.1039, found: 233.1035.

Minor regioisomer (**61'**): R_f = 0.25 (70% EtOAc/pentane); IR (film)/cm^{-1} 3116 (OH st), 2883, 1592, 1517, 1362, 1277, 1222, 1064, 986, 807, 695; ^{1}H NMR (400 MHz, $CDCl_3$) δ 6.98 (d, *J* = 8.7 Hz, 2 H, 2 × Ar-CH), 6.88 (d, *J* = 8.7 Hz, 2 H, 2 × Ar-CH), 5.56 (d, *J* = 6.9 Hz, 2 H, C*H*HOC*H*H), 5.15 (d, *J* = 6.9 Hz, 2 H, CH*H*OCH*H*), 2.24 (s, 3 H, C_q-CH_3); ^{13}C NMR (101 MHz, $CDCl_3$) δ 156.5 (Ar-C_q-OH), 151.5 ($Ar_{(tetrazole)}$-C_q), 129.8 (Ar-C_q-C_q), 126.0 (2 × Ar-CH), 116.4 (2 × Ar-CH), 81.0 (CH_2OCH_2), 64.2 (C_q), 9.5 (C_q-CH_3); HRMS (TOF-MS-ES$^+$) *m/z* calcd for $C_{11}H_{13}N_4O_2^+$ $[M+H]^+$: 233.1039, found: 233.1039.

Notes:

*Regioisomers assigned by analogy of **60** and **60'** assignment:*

- *Oxetane quaternary carbon ^{13}C NMR shift in good agreement between **60, 61** (69.4 ppm) and **60', 61'** (64.2 ppm)*

2-(3-(4-Methoxyphenyl)oxetan-3-yl)-5-phenyl-2*H*-tetrazole (62) and 1-(3-(4-Methoxyphenyl)oxetan-3-yl)-5-phenyl-1*H*-tetrazole (62')

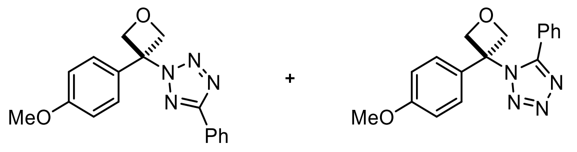

Prepared according to **deFS General Procedure A** under Ar at 70 °C for 2 h with a Celite work-up and using oxetane sulfonyl fluoride **1** (49.2 mg, 0.2 mmol, 1.0 equiv), K_2CO_3 (35.8 mg, 0.26 mmol, 1.3 equiv), 5-phenyl-2/1*H*-tetrazole (87.7 mg, 0.60 mmol, 3.0 equiv) and anhydrous MeCN (0.67 mL, 0.3 M). Purification by flash column chromatography (10–40% EtOAc/*n*-hexane) afforded oxetano-tetrazole oxetane **62** as a colorless gum (43.8 mg, 71%) followed by oxetano-tetrazole **62'** as a colorless gum (14.5 mg, 24%).

Major regioisomer (**62**): R_f = 0.29 (30% EtOAc/*n*-hexane); IR (film)/cm^{-1} 2890, 2960, 1611, 1514, 1450, 1252, 1183, 1026, 986, 829; ^{1}H NMR (400 MHz, $CDCl_3$) δ 8.20–8.12 (m, 2 H, 2 × Ph-CH), 7.54–7.43 (m, 3 H, 3 × Ph-CH), 7.24–7.15 (m, 2 H, 2 × Ar-CH), 6.97–6.87 (m, 2 H, 2 × Ar-CH), 5.68 (d, *J* = 7.1 Hz, 2 H, C*H*HOC*H*H), 5.39 (d, *J* = 7.2 Hz, 2 H, CH*H*OCH*H*), 3.80 (s, 3 H, OCH_3); ^{13}C NMR (101 MHz, $CDCl_3$) δ 165.4 ($Ar_{(tetrazole)}$-C_q), 159.9 (Ar-C_q-OCH_3), 130.5 (Ph-CH), 130.4 (Ar-C_q-C_q), 128.9 (2 × Ph-CH), 127.2 (Ph-C_q), 127.0 (2 × Ph-CH), 126.8 (2 × Ar-CH), 114.4 (2 × Ar-CH), 80.9 (CH_2OCH_2), 69.7 (C_q), 55.4 (OCH_3); HRMS (TOF-MS-ES$^+$) *m/z* calcd for $C_{17}H_{17}N_4O_2^+$ $[M+H]^+$: 309.1352, found: 309.1358.

Minor regioisomer (**62'**): R_f = 0.14 (30% EtOAc/*n*-hexane); IR (film)/cm^{-1} 2959, 2889, 1608, 1513, 1460, 1255, 1184, 1028, 830, 732, 695; ^{1}H NMR (400 MHz, $CDCl_3$) δ 7.56–7.46 (m, 1 H, Ph-CH), 7.43–7.32 (m, 4 H, 4 × Ph-CH), 7.20–7.12 (m, 2 H, 2 × Ar-CH), 7.02–6.93 (m, 2 H, 2 × Ar-CH), 5.23 (d, *J* = 7.3 Hz, 2 H, C*H*HOC*H*H), 4.91 (d, *J* = 7.3 Hz, 2 H, CH*H*OCH*H*), 3.85 (s, 3 H, OCH_3); ^{13}C NMR (101 MHz, $CDCl_3$) δ 160.1 (Ar-C_q-OCH_3), 154.1 ($Ar_{(tetrazole)}$-C_q), 131.9 (Ar-C_q-C_q), 131.6 (Ph-CH), 129.2 (2 × Ph-CH), 128.4 (2 × Ph-CH), 125.4 (2 × Ar-CH), 123.7 (Ph-C_q), 114.9 (2 × Ar-CH), 82.0 (CH_2OCH_2), 65.0 (C_q), 55.4 (OCH_3); HRMS (TOF-MS-ES$^+$) *m/z* calcd for $C_{17}H_{17}N_4O_2^+$ $[M+H]^+$: 309.1352, found: 309.1339.

Notes:

*Regioisomers assigned by analogy of **60** and **60'** assignment:*

- *Oxetane quaternary carbon ^{13}C NMR shift in good agreement between **60, 62** (69.4 ppm; 69.7 ppm) and **60', 62'** (64.2 ppm; 65.0 ppm).*

5-(Ethylthio)-2-(3-(4-methoxyphenyl)oxetan-3-yl)-2*H*-tetrazole (63) and 5-(ethylthio)-1-(3-(4-methoxyphenyl)oxetan-3-yl)-1*H*-tetrazole (63')

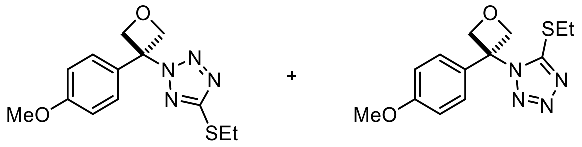

major 65:35 minor

Prepared according to **deFS General Procedure A** under Ar at 70 °C for 2 h with a Celite work-up and using oxetane sulfonyl fluoride **1** (49.2 mg, 0.2 mmol, 1.0 equiv), K_2CO_3 (35.8 mg, 0.26 mmol, 1.3 equiv), 5-(ethylthio)-2/1*H*-tetrazole (78.1 mg, 0.60 mmol, 3.0 equiv) and anhydrous MeCN (0.67 mL, 0.3 M). Purification by flash column chromatography (10–30% EtOAc/*n*-hexane) afforded oxetano-tetrazole **63** as a colorless gum (28.7 mg, 49%) followed by oxetano-tetrazole **63'** as a colorless gum (15.9 mg, 27%).

Major regioisomer (**63**): R_f = 0.25 (20% EtOAc/*n*-hexane); IR (film)/cm^{-1} 2963, 2891, 1612, 1515, 1393, 1303, 1252, 1180, 1032, 987, 830; ^{1}H NMR (400 MHz, $CDCl_3$) δ 7.15 (d, *J* = 8.7 Hz, 2 H, 2 × Ar-CH), 6.90 (d, *J* = 8.8 Hz, 2 H, 2 × Ar-CH), 5.58 (d, *J* = 7.2 Hz, 2 H, C*H*HOC*H*H), 5.33 (d, *J* = 7.2 Hz, 2 H, CH*H*OCH*H*), 3.80 (s, 3 H, OCH_3), 3.22 (q, *J* = 7.3 Hz, 2 H, SCH_2CH$_3$), 1.42 (t, *J* = 7.3 Hz, 3 H, SCH$_2$CH_3); ^{13}C NMR (101 MHz, $CDCl_3$) δ 164.8 ($Ar_{(tetrazole)}$-C_q), 160.0 (Ar-C_q-OCH_3), 130.0 (Ar-C_q-C_q), 126.9 (2 × Ar-CH), 114.4 (2 × Ar-CH), 80.6 (CH_2OCH_2), 69.9 (C_q), 55.4 (OCH_3), 26.5 (SCH_2CH$_3$), 14.9 (SCH$_2$$CH_3$); HRMS (TOF-MS-ES$^+$) *m/z* calcd for $C_{13}H_{17}N_4O_2S^+$ [M+H]$^+$: 293.1072, found: 293.1068.

Minor regioisomer (**63'**): R_f = 0.13 (20% EtOAc/*n*-hexane); IR (film)/cm^{-1} 2964, 2889, 1611, 1514, 1392, 1254, 1184, 1028, 990, 830; ^{1}H NMR (400 MHz, $CDCl_3$) δ 7.09 (d, *J* = 8.8 Hz, 2 H, 2 × Ar-CH), 6.91 (d, *J* = 8.8 Hz, 2 H, 2 × Ar-CH), 5.55 (d, *J* = 7.1 Hz, 2 H, C*H*HOC*H*H), 5.14 (d, *J* = 7.1 Hz, 2 H, CH*H*OCH*H*), 3.81 (s, 3 H, OCH_3), 3.30 (q, *J* = 7.3 Hz, 2 H, SCH_2CH$_3$), 1.40 (t, J = 7.3 Hz, 3 H, SCH$_2$CH_3); ^{13}C NMR (101 MHz, $CDCl_3$) δ 160.0 (Ar-C_q-OCH_3), 153.7 ($Ar_{(tetrazole)}$-C_q), 129.4 (Ar-C_q-C_q), 126.3 (2 × Ar-CH), 114.5 (2 × Ar-CH), 80.5 (CH_2OCH_2), 64.6 (C_q), 55.4 (OCH_3), 28.1 (SCH_2CH$_3$), 14.6 (SCH$_2$$CH_3$); HRMS (TOF-MS-ES$^+$) *m/z* calcd for $C_{13}H_{17}N_4O_2S^+$ [M+H]$^+$: 293.1072, found: 293.1063.

Notes:

*Regioisomers assigned by analogy of **60** and **60'** assignment:*

- *Oxetane quaternary carbon ^{13}C NMR shift in good agreement between **60, 63** (69.4 ppm; 69.9 ppm) and **60', 63'** (64.2 ppm; 64.6 ppm).*

Oxetane deFS with S=NH nucleophiles (64–77)

***tert*-Butyl((3-(4-methoxyphenyl)oxetan-3-yl)imino)(methyl)-λ^6-sulfanone (64)**

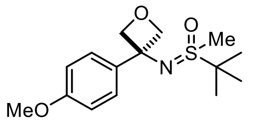

Prepared according to **deFS General Procedure A** under Ar at 60 °C for 2 h with a Celite work-up and using oxetane sulfonyl fluoride **1** (49.2 mg, 0.2 mmol, 1.0 equiv), K_2CO_3 (35.8 mg, 0.26 mmol, 1.3 equiv), *tert*-butyl(imino)(methyl)-λ^6-sulfanone **S12** (81.0 mg, 0.60 mmol, 3.0 equiv) and anhydrous MeCN (0.67 mL, 0.3 M). Purification by filtration through a silica plug afforded oxetane sulfoximine **64** as an off-white

solid (50.5 mg, 85%). R_f = 0.33 (EtOAc); mp = 87–89 °C; IR (film)/cm^{-1} 2950, 2871, 2846, 1608, 1510, 1460, 11239, 1176, 1147, 1027, 977, 950, 830, 755, 713, 552; ^{1}H NMR (400 MHz, $CDCl_3$) δ 7.47 (d, *J* = 8.6 Hz, 2 H, 2 × Ar-CH), 6.92 (d, *J* = 8.6 Hz, 2 H, 2 × Ar-CH), 5.18 (d, *J* = 6.1 Hz, 1 H, C*H*HOCHH), 5.09 (d, *J* = 6.1 Hz, 1 H, CHHOC*H*H), 5.06 (d, *J* = 5.3 Hz, 1 H, CH*H*OCHH), 4.79 (d, *J* = 5.3 Hz, 1 H, CHHOCH*H*), 3.83 (s, 3 H, OCH_3), 2.20 (s, 3 H, SCH_3), 1.44 (s, 9 H, $C(CH_3)_3$); ^{13}C NMR (101 MHz, $CDCl_3$) δ 158.7 (Ar-C_q-OCH_3), 137.7 (Ar-C_q-C_q), 127.3 (2 × Ar-CH), 113.8 (2 × Ar-CH), 87.9 (CH_2OCH_2), 84.3 (CH_2OCH_2), 61.8 (C_q), 60.0 ($C(CH_3)_3$), 55.3 (OCH_3), 34.8 (SCH_3), 23.5 ($C(CH_3)_3$); HRMS (TOF-MS-ES$^+$) *m/z* calcd for $C_{15}H_{24}NO_3S^+$ $[M+H]^+$: 298.1477; found 298.1482.

1-((3-(4-Methoxyphenyl)oxetan-3-yl)imino)tetrahydro-1*H*-1λ^6-thiophene 1-oxide (65)

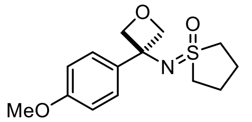

Prepared according to **deFS General Procedure A** under Ar at 60 °C for 2 h with a Celite work-up and using oxetane sulfonyl fluoride **1** (49.2 mg, 0.2 mmol, 1.0 equiv), K_2CO_3 (35.8 mg, 0.26 mmol, 1.3 equiv), 1-iminotetrahydro-1*H*-1λ^6-thiophene 1-oxide **S13** (72.0 mg, 0.60 mmol, 3.0 equiv) and anhydrous MeCN (0.67 mL, 0.3 M). Purification flash column chromatography (80–90% EtOAc/*n*-hexane) afforded oxetane sulfoximine **65** as a white solid (34.8 mg, 62%). R_f = 0.10 (80% EtOAc/*n*-hexane); mp = 98–100 °C; IR (film)/cm^{-1} 3500, 2948, 2875, 1611, 1511, 1460, 1243, 1170, 1075, 979, 835; ^{1}H NMR (400 MHz, $CDCl_3$) δ 7.35 (d, *J* = 8.7 Hz, 2 H, 2 × Ar-CH), 6.92 (d, *J* = 8.7 Hz, 2 H, 2 × Ar-CH), 5.08 (m, 4 H, CH_2OCH_2), 3.83 (s, 3 H, OCH_3), 2.67 (dt, *J* = 13.3, 6.7 Hz, 2 H, C*H*$_2$$SCH_2$), 2.56 (dt, *J* = 13.3, 6.7 Hz, 2 H, CH_2SCH_2), 2.08–1.90 (m, 4 H, $CH_2CH_2SCH_2CH_2$); ^{13}C NMR (101 MHz, $CDCl_3$) δ 159.0 (Ar-C_q-OCH_3), 135.4 (Ar-C_q-C_q), 127.7 (2 × Ar-CH), 114.0 (2 × Ar-CH), 85.5 (CH_2OCH_2), 62.8 (C_q), 55.3 (OCH_3), 53.7(CH_2SCH_2), 23.1 ($CH_2CH_2SCH_2CH_2$); HRMS (TOF-MS-ES$^+$) *m/z* calcd for $C_{14}H_{20}NO_3S^+$ $[M+H]^+$: 282.1164; found 282.1161.

3-(4-Methoxyphenyl)oxetan-3-yl)imino)(methyl)(*p*-tolyl)-λ^6-sulfanone (66)

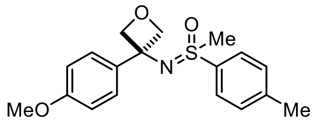

Prepared according to **deFS General Procedure A** under Ar at 60 °C for 2 h with a Celite work-up and using oxetane sulfonyl fluoride **1** (49.2 mg, 0.2 mmol, 1.0 equiv), K_2CO_3 (35.8 mg, 0.26 mmol, 1.3 equiv), imino(methyl)(*p*-tolyl)-λ^6-sulfanone **S14** (102.0 mg, 0.60 mmol, 3.0 equiv) and anhydrous MeCN (0.67 mL, 0.3 M). Purification by flash column chromatography (60% EtOAc/*n*-hexane) afforded oxetane sulfoximine **66** as a white solid (36.7 mg, 55%). R_f = 0.37 (80% EtOAc/*n*-hexane); mp = 112–113 °C; IR (film)/cm^{-1} 2948, 2876, 1608, 1511, 1409, 1241, 1169, 1097, 1031, 976, 814, 760, 628, 520; ^{1}H NMR (400 MHz, $CDCl_3$) δ 7.59 (d, *J* = 8.0 Hz, 2 H, 2 × Ar-CH), 7.36 (d, *J* = 8.7 Hz, 2 H, 2 × Ar-CH), 7.18 (d, *J* = 8.0 Hz, 2 H, 2 × Ar-CH), 6.74 (d, *J* = 8.7 Hz, 2 H, 2 × Ar-CH), 5.12 (m, 2 H, C*H*$_2$OCHH), 4.89 (d, *J* = 5.8 Hz, 1 H, CH_2OC*H*H), 4.83 (d, *J* = 5.8 Hz, 1 H, CH_2OCH*H*), 3.76 (s, 3 H, OCH_3), 2.97 (s, 3 H, SCH_3), 2.39 (s, 3 H, C_q-CH_3); ^{13}C NMR (101 MHz, $CDCl_3$) δ 158.5 (Ar-C_q-OCH_3), 143.0 (Ar-C_q-S), 139.3 (Ar-C_q-CH_3), 136.7 (Ar-C_q-C_q), 129.6 (2 × Ar-CH), 127.6 (2 × Ar-CH), 127.2 (2 × Ar-CH), 113.5 (2 × Ar-CH), 86.3 (CH_2OCH_2), 85.1 (CH_2OCH_2), 62.1 (C_q), 55.2 (OCH_3), 47.2 (SCH_3), 21.4 (Ar-C_q-CH_3); HRMS (TOF-MS-ES$^+$) *m/z* calcd for $C_{18}H_{22}NO_3S^+$ $[M+H]^+$: 332.1320; found 332.1320.

((3-(4-Methoxyphenyl)oxetan-3-yl)imino)diphenyl-λ^6-sulfanone (67)

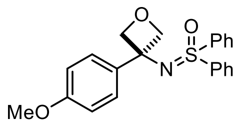

Prepared according to **deFS General Procedure A** under Ar at 60 °C for 2 h with a Celite work-up and using oxetane sulfonyl fluoride **1** (49.2 mg, 0.2 mmol, 1.0 equiv), K_2CO_3 (35.8 mg, 0.26 mmol, 1.3 equiv), *S*,*S*-diphenyl sulfoximine **S15** (130.0 mg, 0.6 mmol, 3.0 equiv) and anhydrous MeCN (0.67 mL, 0.3 M). The residue was diluted with EtOAc (10 mL) and washed with aq. HCl (1 M, 3 × 10 mL). The organic layer was separated, dried over Na_2SO_4, filtered, and concentrated *in vacuo* using a rotary evaporator. Purification by filtration through a plug of silica (40% EtOAc/*n*-hexane) afforded oxetane sulfoximine

67 as a clear, colorless gum (63.6 mg, 84%). R_f = 0.43 (40% EtOAc/*n*-hexane); IR (film)/cm^{-1} 2950, 2872, 1608, 1509, 1443, 241, 1171, 1087, 1025, 976, 722, 686, 631, 587, 547, 523; ^{1}H NMR (400 MHz, $CDCl_3$) δ 7.93–7.81 (m, 4 H, 4 × Ar-CH), 7.53–7.33 (m, 8 H, 8 × Ar-CH), 6.77 (d, *J* = 8.7 Hz, 2 H, 2 × Ar-CH), 5.24 (d, *J* = 6.1 Hz, 2 H, CH*H*OCH*H*), 4.86 (d, *J* = 6.1 Hz, 2 H, C*H*HOC*H*H), 3.78 (s, 3 H, OCH_3); ^{13}C NMR (101 MHz, $CDCl_3$) δ 158.4 (Ar-*C*$_q$-OCH_3), 143.0 (Ar-C$_q$-S), 137.1 (Ar-*C*$_q$-C$_q$), 132.1 (2 × Ar-CH), 129.0 (4 × Ar-CH), 127.8 (4 × Ar-CH), 127.1 (2 × Ar-CH), 113.5 (2 × Ar-CH), 86.0 (CH_2OCH_2), 61.9 (C$_q$), 55.3 (OCH_3); HRMS (TOF-MS-ES$^+$) *m/z* calcd for $C_{22}H_{22}NO_3S^+$ $[M+H]^+$: 380.1320; found 380.1320.

((3-(4-Methoxyphenyl)oxetan-3-yl)imino)(tetrahydro-2*H*-pyran-4-yl)(*p*-tolyl)-λ^6-sulfanone (68)

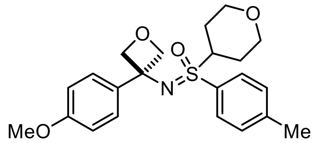

Prepared according to **deFS General Procedure A** under Ar at 60 °C for 2 h with a Celite work-up and using oxetane sulfonyl fluoride **1** (17.2 mg, 0.07 mmol, 1.0 equiv), K_2CO_3 (13.0 mg, 0.091 mmol, 1.3 equiv), imino(tetrahydro-2*H*-pyran-4-yl)(*p*-tolyl)-λ^6-sulfanone **S16** (54.0 mg, 0.21 mmol, 3.0 equiv) and anhydrous MeCN (0.23 mL, 0.3 M). Purification by flash column chromatography (80% EtOAc/*n*-hexane) afforded oxetane sulfoximine **68** as a white solid (26.0 mg, 94%). R_f = 0.40 (EtOAc); mp = 101–103 °C; IR (film)/cm^{-1} 2954, 2846, 1608, 1511, 1385, 1229, 1169, 1025, 808, 731. 658, 532; ^{1}H NMR (400 MHz, $CDCl_3$) δ 7.47 (dd, *J* = 8.7, 8.0 Hz, 4 H, 4 × Ar-CH), 7.18 (d, *J* = 8.0 Hz, 2 H, 2 × Ar-CH), 6.76 (d, *J* = 8.7 Hz, 2 H, 2 × Ar-CH), 5.09 (m, 2 H, C*H*HOCH*H*), 4.82 (d, *J* = 5.6 Hz, 1 H, CH*H*OCHH), 4.75 (d, *J* = 6.0 Hz, 1 H, CHHOC*H*H), 4.09 (ddd, *J* = 11.6, 4.9, 1.8 Hz, 1 H, OCH*H*), 4.01 (dt, *J* = 11.5, 3.7 Hz, 1 H, OC*H*H), 3.77 (s, 3 H, OCH_3), 3.40–3.21 (m, 2 H, OCH*H* + CH), 3.21–3.08 (m, 1 H, OCH*H*), 2.39 (s, 3 H, CH_3), 2.16 (ddt, *J* = 13.2, 4.0, 1.9 Hz, 1 H, CHC*H*H), 1.85–1.70 (m, 3 H, C*H*$_2$CHCH*H*); ^{13}C NMR (101 MHz, $CDCl_3$) δ 158.4 (Ar-*C*$_q$-OCH_3), 143.2 (Ar-*C*$_q$-S), 137.7 (Ar-*C*$_q$-CH_3), 135.8 (Ar-*C*$_q$-C$_q$), 129.6 (2 × Ar-CH), 129.3 (2 × Ar-CH), 126.9 (2 × Ar-CH), 113.4 (2 × Ar-CH), 87.4 (*C*H_2OCH_2), 84.9 (CH_2O*C*H_2), 66.9 (CH_2*C*$H_2OCH_2CH_2$), 66.6 (CH_2CH_2O*C*H_2CH_2), 62.2 (CH), 61.5 (C$_q$), 55.2 (OCH_3), 25.9 (*C*$H_2CH_2OCH_2CH_2$), 25.5 ($CH_2CH_2OCH_2$*C*H_2), 21.4 (CH_3); HRMS (TOF-MS-ES$^+$) *m/z* calcd for $C_{22}H_{28}NO_4S^+$ $[M+H]^+$: 402.1739; found 402.1732.

***tert*-Butyl 4-(*N*-(3-(4-methoxyphenyl)oxetan-3-yl)-4-methylphenylsulfonimidoyl)piperidine-1-carboxylate (69)**

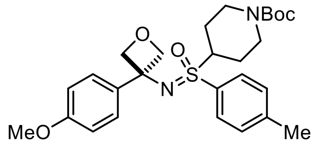

Prepared according to **deFS General Procedure A** under Ar at 60 °C for 2 h with a Celite work-up and using oxetane sulfonyl fluoride **1** (17.2 mg, 0.07 mmol, 1.0 equiv), K_2CO_3 (13.0 mg, 0.091 mmol, 1.3 equiv), *tert*-butyl 4-(4-methylphenylsulfonimidoyl)piperidine-1-carboxylate **S17** (74 mg, 0.21 mmol, 3.0 equiv) and anhydrous MeCN (0.23 mL, 0.3 M). Purification by flash column chromatography (40% EtOAc/*n*-hexane) afforded oxetane sulfoximine **69** as a clear colorless gum (29.1 mg, 83%). R_f = 0.32 (50% EtOAc/*n*-hexane); IR (film)/cm^{-1} 2960, 2870, 1692 (C=O st), 1614, 1511, 1421, 1322, 1247, 1169, 1115, 1012, 832, 737, 664, 532; ^{1}H NMR (400 MHz, $CDCl_3$) δ 7.46 (dd, *J* = 16.6, 8.1 Hz, 4 H, 4 × Ar-CH), 7.18 (d, *J* = 8.0 Hz, 2 H, 2 × Ar-CH), 6.76 (d, *J* = 8.7 Hz, 2 H, 2 × Ar-CH), 5.08 (dd, *J* = 5.8, 3.0 Hz, 2 H, C*H*HOC*H*H), 4.81 (d, *J* = 5.6 Hz, 1 H, CH*H*OCHH), 4.75 (d, *J* = 6.1 Hz, 1 H, CHHOCH*H*), 4.22 (br, 2 H, C*H*$_2$NCH$_2$), 3.78 (s, 3 H, OCH_3), 3.09–2.97 (m, 1 H, CH), 2.59 (br, 2 H, CH$_2$NC*H*$_2$), 2.39 (s, 3 H, C$_q$-CH_3), 2.23 (br, 1 H, CH*H*), 1.80 (br, 1 H, CH*H*), 1.67–1.51 (m, 2 H, CH*H*CHCH*H*), 1.44 (s, 9 H, $C(CH_3)_3$); ^{13}C NMR (101 MHz, $CDCl_3$) δ 158.4 (C$_q$=O), 154.4 (Ar-*C*$_q$-OCH_3), 143.3 (Ar-C$_q$-S), 137.6 (Ar-C$_q$-CH_3), 135.9 (Ar-*C*$_q$-C$_q$), 129.6 (2 × Ar-CH), 129.3 (2 × Ar-CH), 126.9 (2 × Ar-CH), 113.4 (2 × Ar-CH), 87.3 (*C*$(CH_3)_3$), 84.9 (*C*H_2OCH_2), 80.0 (CH_2O*C*H_2), 63.4

(C_q), 61.6 (CH), 55.3 (OCH_3), 42.8 (CH_2NCH_2), 28.4 ($C(CH_3)_3$), 25.3 (CH_2), 24.9 (CH_2), 21.4 (C_q-CH_3); HRMS (TOF-MS-ES+) *m/z* calcd for $C_{27}H_{37}N_2O_5S$ [M+H]: 501.2423; found 501.2422.

((3-(4-Methoxyphenyl)oxetan-3-yl)imino)(methyl)(pyridin-2-yl)-λ^6-sulfanone (70)

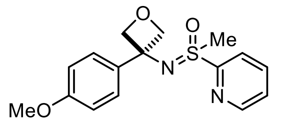

Prepared according to **deFS General Procedure A** under Ar at 60 °C for 2 h with a Celite work-up and using oxetane sulfonyl fluoride **1** (49.2 mg, 0.2 mmol, 1.0 equiv), K_2CO_3 (35.8 mg, 0.26 mmol, 1.3 equiv), imino(methyl)(pyridine-2-yl)-λ^6-sulfanone **S18** (94.0 mg, 0.6 mmol, 3.0 equiv) and anhydrous MeCN (0.67 mL, 0.3 M). Purification by flash column chromatography (EtOAc) afforded oxetane sulfoximine **70** as a white gum (38.8 mg, 61%). R_f = 0.25 (EtOAc); IR (film)/cm^{-1} 3483, 2948, 2876, 1608, 1511, 1421, 1229, 1169, 1025, 976, 832, 730, 616, 520; ^{1}H NMR (400 MHz, $CDCl_3$) δ 8.52 (ddd, *J* = 4.7, 1.7, 1.0 Hz, 1 H, $Ar_{(py)}$-CH), 7.60 (td, *J* = 7.7, 1.7 Hz, 1 H, $Ar_{(py)}$-CH), 7.52 (dt, *J* = 7.7, 1.0 Hz, 1 H, $Ar_{(py)}$-CH), 7.35–7.25 (m, 1 H, $Ar_{(py)}$-CH), 7.22–7.06 (m, 2 H, 2 × Ar-CH), 6.61–6.51 (m, 2 H, 2 × Ar-CH), 5.09 (m, 3 H, C*HH*OC*H*H), 4.81 (d, *J* = 5.7 Hz, 1 H, CHHOCH*H*), 3.70 (s, 3 H, OCH_3), 3.14 (s, 3 H, SCH_3); ^{13}C NMR (101 MHz, $CDCl_3$) δ 159.5 ($Ar_{(py)}$-C_q-S), 158.3 (Ar-C_q-OCH_3), 149.5 ($Ar_{(py)}$-CH), 137.3 ($Ar_{(py)}$-CH), 135.1 (Ar-C_q-C_q), 127.8 (2 × Ar-CH), 125.5 ($Ar_{(py)}$-CH), 122.3 ($Ar_{(py)}$-CH), 113.2 (2 × Ar-CH), 86.5 (*C*H_2OCH_2), 84.8 (CH_2O*C*H_2), 62.4 (C_q), 55.2 (OCH_3), 42.9 (SCH_3); HRMS (TOF-MS-ES$^+$) *m/z* calcd for $C_{16}H_{19}N_2O_3S^+$ $[M+H]^+$: 319.1116; found 319.1117.

(Chloromethyl)((3-(4-methoxyphenyl)oxetan-3-yl)imino)(*p*-tolyl)-λ^6-sulfanone (71)

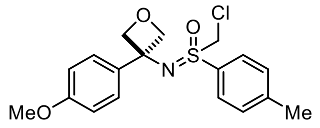

Prepared according to **deFS General Procedure A** under Ar at 60 °C for 2 h with a Celite work-up and using oxetane sulfonyl fluoride **1** (17.2 mg, 0.07 mmol, 1.0 equiv), K_2CO_3 (13.0 mg, 0.091 mmol, 1.3 equiv), (chloromethyl)(imino)(*p*-tolyl)-λ^6 sulfanone **S19** (45.0 mg, 0.22 mmol, 3.0 equiv) and anhydrous MeCN (0.23 mL, 0.3 M). The residue was diluted with EtOAc (10 mL) and washed with aq. HCl (1 M, 3 × 10 mL). The organic layer was dried over Na_2SO_4, filtered, and concentrated *in vacuo* using a rotary evaporator. Purification by flash column chromatography (30–70% EtOAc/*n*-hexane) afforded oxetane sulfoximine **71** as a white solid (17.4 mg, 68%). R_f = 0.18 (30% EtOAc/*n*-hexane); mp = 70–72 °C; IR (film)/cm^{-1} 2950, 2875, 1609, 1512, 1284, 1247, 1176, 1029, 980, 830, 524; ^{1}H NMR (400 MHz, $CDCl_3$) δ 7.74 (d, *J* = 8.3 Hz, 2 H, 2 × Ar-CH), 7.45 (d, *J* = 8.8 Hz, 2 H, 2 × Ar-CH), 7.28 (d, *J* = 8.3 Hz, 2 H, 2 × Ar-CH), 6.86 (d, *J* = 8.8 Hz, 2 H, 2 × Ar-CH), 5.14 (d, J = 5.9 Hz, 1 H, CHHOCH*H*), 5.12–5.05 (m, 2 H, C*H*HOC*H*H), 4.89 (d, *J* = 5.9 Hz, 1 H, CH*H*OCHH), 4.21 (d, *J* = 12.0 Hz, 1 H, C*H*H-Cl), 3.97 (d, J = 12.0 Hz, 1 H, CH*H*-Cl), 3.80 (s, 3 H, OCH_3), 2.42 (s, 3 H, C_q-CH_3); ^{13}C NMR (101 MHz, $CDCl_3$) δ 158.9 (Ar-C_q-OCH_3), 144.3 (Ar-C_q-S), 136.0 (Ar-C_q-CH_3), 134.5 (Ar-C_q-C_q), 129.6 (2 × Ar-CH), 128.9 (2 × Ar-CH), 127.4 (2 × Ar-CH), 113.8 (2 × Ar-CH), 86.0 (*C*H_2OCH_2), 85.6 (CH_2O*C*H_2), 62.2 (C_q), 59.5 (SCH_2), 55.3 (OCH_3), 21.6 (C_q-CH_3); HRMS (TOF-MS-ES$^+$) *m/z* calcd for $C_{18}H_{21}NO_3S^{35}Cl^+$ $[M+H]^+$: 366.0931; found 366.0934.

Bicyclo[1.1.0]butan-1-yl((3-(4-methoxyphenyl)oxetan-3-yl)imino)(*p*-tolyl)-λ^6-sulfanone (72)

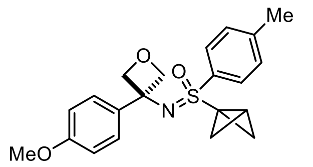

Prepared according to **deFS General Procedure A** under Ar at 60 °C for 2 h with a Celite work-up and using oxetane sulfonyl fluoride **1** (49.2 mg, 0.2 mmol, 1.0 equiv), K_2CO_3 (35.8 mg, 0.26 mmol, 1.3 equiv), bicyclo[1.1.0]butan-1-yl(imino)(*p*-tolyl)-λ^6-sulfanone-methane **S20** (124.0 mg, 0.6 mmol, 3.0 equiv) and anhydrous MeCN (0.67 mL, 0.3 M). Purification by flash column chromatography (30–40% EtOAc/*n*-hexane) afforded oxetane sulfoximine **72** as a clear, colorless gum (53.3 mg, 72%). R_f = 0.29 (40% EtOAc/*n*-hexane); IR (film)/cm^{-1} 2950, 2871, 1608, 1509, 1459, 1332, 1241, 1170, 1105, 1028, 978, 808, 752, 667, 622, 542; ^{1}H NMR (400 MHz, $CDCl_3$) δ 7.63 (d, *J* = 8.2 Hz, 2 H, 2 × Ar-CH), 7.47 (d, *J* = 8.8 Hz, 2 H, 2 ×

Ar-CH), 7.20 (d, *J* = 8.2 Hz, 2 H, 2 × Ar-CH), 6.80 (d, *J* = 8.8 Hz, 2 H, 2 × Ar-CH), 5.15 (d, *J* = 5.8 Hz, 2 H, C*H*$_2$OCHH), 4.83 (d, *J* = 5.6 Hz, 1 H, CH$_2$OC*H*H), 4.76 (d, *J* = 5.8 Hz, 1 H, CH$_2$OCH*H*), 3.79 (s, 3 H, OCH$_3$), 2.62–2.48 (m, 1 H, CH), 2.40 (s, 3 H, C$_q$-CH$_3$), 2.18–2.09 (m, 2 H, 2 × CH*H*), 1.33–1.28 (m, 1 H, C*H*H), 1.20–1.10 (m, 1 H, C*H*H); ^{13}C NMR (101 MHz, CDCl$_3$) δ 158.4 (Ar-*C*$_q$-OCH$_3$), 142.8 (Ar-C$_q$-S), 138.8 (Ar-*C*$_q$-CH$_3$), 137.7 (Ar-*C*$_q$-C$_q$), 129.5 (2 × Ar-CH), 127.9 (2 × Ar-CH), 127.0 (2 × Ar-CH), 113.4 (2 × Ar-CH), 86.7 (*C*H$_2$OCH$_2$), 85.6 (CH$_2$O*C*H$_2$), 61.7 (C$_q$), 55.2 (OCH$_3$), 38.3 (CH$_2$), 36.3 (CH$_2$), 26.0 (S-C$_q$), 21.4 (C$_q$-*C*H$_3$), 12.6 (CH); HRMS (TOF-MS-ES$^+$) *m/z* calcd $C_{21}H_{24}NO_3S^+$ [M+H]$^+$: 370.1477; found 370.1478.

(4-Bromophenyl)((3-(4-methoxyphenyl)oxetan-3-yl)imino)(methyl)-λ^6-sulfanone (73)

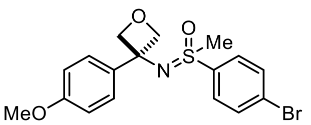

Prepared according to **deFS General Procedure A** under Ar at 60 °C for 2 h with a Celite work-up and using oxetane sulfonyl fluoride **1** (246.0 mg, 1.0 mmol, 1.0 equiv), K$_2$CO$_3$ (180.0 mg, 1.3 mmol, 1.3 equiv), (4-bromophenyl)(imino)(methyl)-λ^6-sulfanone **S21** (702.0 mg, 3.0 mmol, 3.0 equiv) and anhydrous MeCN (3.33 mL, 0.3 M). The residue was diluted with EtOAc (20 mL) and washed with aq. HCl (1 M, 3 × 20 mL). The organic layer was separated, dried over Na$_2$SO$_4$, filtered, and concentrated *in vacuo* using a rotary evaporator. Purification by filtration through a plug of silica (90% EtOAc/*n*-hexane) afforded oxetane sulfoximine **73** as a white solid (313.9 mg, 85%). R$_f$ = 0.23 (80% EtOAc/*n*-hexane); mp = 126–128 °C; IR (film)/cm^{-1} 2951, 2974, 2835, 1610, 1571, 1511, 1466, 1386, 1244, 1176, 1067, 1030, 978, 827, 770, 571, 553, 525, 512; ^{1}H NMR (400 MHz, CDCl$_3$) δ 7.44 (m, 4 H, 4 × Ar-CH), 7.18 (d, *J* = 8.8 Hz, 2 H, 2 × Ar-CH), 6.65 (d, *J* = 8.8 Hz, 2 H, 2 × Ar-CH), 5.13 (d, *J* = 5.9 Hz, 1 H, C*H*HOCHH), 5.08 (d, *J* = 5.9 Hz, 1 H, CHHOC*H*H), 4.96 (d, *J* = 5.9 Hz, 1 H, CH*H*OCHH), 4.92 (d, *J* = 5.9 Hz, 1 H, CHHOCH*H*), 3.74 (s, 3 H, OCH$_3$), 2.99 (s, 3 H, SCH$_3$); ^{13}C NMR (101 MHz, CDCl$_3$) δ 158.6 (Ar-*C*$_q$-OCH$_3$), 141.0 (Ar-*C*$_q$-S), 135.5 (Ar-*C*$_q$-C$_q$), 132.0 (2 × Ar-CH), 129.2 (2 × Ar-CH), 127.5 (2 × Ar-CH), 127.1 (Ar-*C*$_q$-Br), 113.4 (2 × Ar-CH), 86.3 (*C*H$_2$OCH$_2$), 84.6 (CH$_2$O*C*H$_2$), 62.3 (C$_q$), 55.3 (OCH$_3$), 47.0 (SCH$_3$); HRMS (TOF-MS-ES$^+$) *m/z* calcd for $C_{17}H_{19}NO_3S^{79}Br^+$ [M+H]$^+$: 396.0269; found 396.0258.

((3-(Benzo[*d*][1,3]dioxol-5-yl)oxetan-3-yl)imino)(4-bromophenyl)(methyl)-λ^6-sulfanone (74)

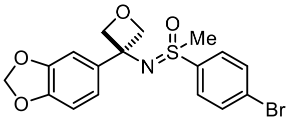

Prepared according to **deFS General Procedure A** under Ar at 60 °C for 4 h with a Celite work-up and using oxetane sulfonyl fluoride **7** (52.0 mg, 0.2 mmol, 1.0 equiv), K$_2$CO$_3$ (35.8 mg, 0.26 mmol, 1.3 equiv), (4-bromophenyl)(imino)(methyl)-λ^6-sulfanone **S21** (141.0 mg, 0.6 mmol, 3.0 equiv) and anhydrous MeCN (0.67 mL, 0.3 M). The residue was diluted with EtOAc (10 mL) and washed with aq. HCl (1 M, 3 × 10 mL). The organic layer was separated, dried over Na$_2$SO$_4$, filtered, and concentrated *in vacuo* using a rotary evaporator. Purification by filtration through a plug of silica (40% EtOAc/*n*-hexane) afforded oxetane sulfoximine **74** as a white solid (63.4 mg, 77%). R$_f$ = 0.28 (80% EtOAc/*n*-hexane); mp = 159–162 °C; IR (film)/cm^{-1} 2950, 2875, 2245, 1571, 1504, 1485, 1435, 1386, 1220, 1153, 1064, 1034, 974, 905, 831, 810, 766, 726, 568, 531, 503; ^{1}H NMR (400 MHz, CDCl$_3$) δ 7.51–7.41 (m, 4 H, 4 × Ar-CH), 6.77 (dd, *J* = 8.0, 1.9 Hz, 1 H, Ar-CH), 6.63 (d, *J* = 1.8 Hz, 1 H, Ar-CH), 6.58 (d, *J* = 8.0 Hz, 1 H, Ar-CH), 5.88 (s, 2 H, OCH$_2$O), 5.10 (d, *J* = 6.1 Hz, 1 H, C*H*HOCHH), 5.04 (d, *J* = 5.7 Hz, 1 H, CH*H*OCHH), 4.96 (d, *J* = 6.1 Hz, 1 H, CHHOC*H*H), 4.87 (d, *J* = 5.7 Hz, 1 H, CHHOCH*H*), 3.04 (s, 3 H, SCH$_3$); ^{13}C NMR (101 MHz, CDCl$_3$) δ 147.3 (Ar-*C*$_q$-OCH$_2$), 146.4 (Ar-*C*$_q$-OCH$_2$), 141.0 (Ar-*C*$_q$-S), 137.1 (Ar-*C*$_q$-C$_q$), 131.9 (2 × Ar-CH), 129.2 (2 × Ar-CH), 127.0 (Ar-*C*$_q$-Br), 119.5 (Ar-CH), 107.7 (Ar-CH), 107.4 (Ar-CH), 101.1 (OCH$_2$O), 86.3 (*C*H$_2$OCH$_2$), 84.4 (CH$_2$O*C*H$_2$), 62.6 (C$_q$), 46.9 (SCH$_3$); HRMS (TOF-MS-ES$^+$) *m/z* calcd for $C_{17}H_{17}NO_4S^{79}Br^+$ [M+H]$^+$: 410.0062; found 410.0058.

((3-(4-(Benzyloxy)phenyl)oxetan-3-yl)imino)(4-bromophenyl)(methyl)-λ^6-sulfanone (75)

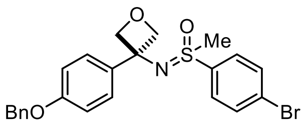

Prepared according to **deFS General Procedure A** under Ar at 60 °C for 2 h with a Celite work-up and using oxetane sulfonyl fluoride **132** (64.5 mg, 0.2 mmol, 1.0 equiv), K_2CO_3 (35.8 mg, 0.26 mmol, 1.3 equiv), (4-bromophenyl)(imino)(methyl)-λ^6-sulfanone **S21** (141.0 mg, 0.6 mmol, 3.0 equiv) and anhydrous MeCN (0.67 mL, 0.3 M). The residue was diluted with EtOAc (10 mL) and washed with aq. HCl (1 M, 3 × 10 mL). The organic layer was separated, dried over Na_2SO_4, filtered, and concentrated *in vacuo* using a rotary evaporator. Purification by flash column chromatography (80% EtOAc/*n*-hexane) afforded oxetane sulfoximine **75** as a white solid (88.6 mg, 94%). R_f = 0.31 (80% EtOAc/*n*-hexane); mp = 157–160 °C; IR (film)/cm^{-1} 3030, 2948, 2872, 1608, 1571, 1510, 1469, 1454, 1385, 1295, 1234, 1178; ^{1}H NMR (400 MHz, $CDCl_3$) δ 7.49–7.32 (m, 9 H, 5 × Ph-CH + 4 × Ar-CH), 7.20–7.15 (m, 2 H, 2 × Ar-CH), 6.76–6.69 (m, 2 H, 2 × Ar-CH), 5.11 (dd, *J* = 21.7, 5.9 Hz, 2 H, C*H*HOC*H*H), 5.03 (dd, *J* = 21.7, 5.9 Hz, 2 H, CH*H*OCH*H*), 4.98 (s, 2 H, $PhCH_2$), 2.98 (s, 3 H, CH_3); ^{13}C NMR (101 MHz, $CDCl_3$) δ 157.8 (Ar-C_q-OBn), 141.0 (Ar-C_q-S), 136.8 (Ar-C_q-CH_2O), 135.7 (Ar-C_q-C_q), 131.9 (2 × Ar-CH), 129.1 (2 × Ar-CH), 128.5 (2 × Ar-CH), 127.9 (2 × Ar-CH), 127.4 (2 × Ar-CH), 126.9 (Ar-C_q-Br), 114.2 (2 × Ar-CH), 86.3 (CH_2OCH_2), 84.5 (CH_2OCH_2), 70.0 ($PhCH_2$), 62.2 (C_q), 46.9 (SCH_3); HRMS (TOF-MS-ES$^+$) *m/z* calcd for $C_{23}H_{24}NO_3S^{79}Br^+$ [M+H]$^+$: 472.0582; found 472.0571.

***75** was further characterized by X-ray crystallography (see Fig. **S11**). Crystals suitable for X-ray analysis were grown by slow evaporation from $CDCl_3$.*

4-(*N*-(3-(4-Methoxyphenyl)oxetan-3-yl)-4-methylphenylsulfonimidoyl)morpholine (76)

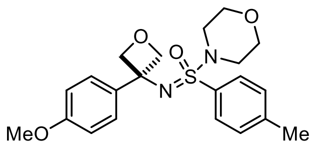

Prepared according to **deFS General Procedure A** under Ar at 60 °C for 2 h with a Celite work-up and using oxetane sulfonyl fluoride **1** (49.2 mg, 0.2 mmol, 1.0 equiv), K_2CO_3 (35.8 mg, 0.26 mmol, 1.3 equiv), 4-(4-methylphenylsulfonimidoyl)morpholine **S22** (144.0 mg, 0.6 mmol, 3.0 equiv) and anhydrous MeCN (0.67 mL, 0.3 M). Purification by flash column chromatography (50% EtOAc/*n*-hexane) afforded oxetane sulfonimidamide **76** as a white foam (61 mg, 76%). R_f = 0.26 (60% EtOAc/*n*-hexane); IR (film)/cm^{-1} 2954, 2853, 1611, 1511, 1455, 1337, 1287, 1242, 1175, 1105, 1069, 979, 812, 711; ^{1}H NMR (400 MHz, $CDCl_3$) δ 7.74 (d, *J* = 8.1 Hz, 2 H, 2 × Ar-CH), 7.62 (d, *J* = 8.8 Hz, 2 H, 2 × Ar-CH), 7.32 (d, *J* = 8.1 Hz, 2 H, 2 × Ar-CH), 6.94 (d, *J* = 8.8 Hz, 2 H, 2 × Ar-CH), 5.47 (d, *J* = 6.0 Hz, 1 H, C*H*HOCHH), 5.31 (d, *J* = 5.6 Hz, 1 H, CH*H*OCHH), 5.06 (d, *J* = 6.0 Hz, 1 H, CHHOC*H*H), 4.85 (d, *J* = 5.6 Hz, 1 H, CHHOCH*H*), 3.83 (s, 3 H, OCH_3), 3.48 (ddd, *J* = 11.4, 6.3, 3.1 Hz, 2 H, $CH_2CH_2OCH_2CH_2$), 3.40 (ddd, *J* = 11.4, 6.3, 3.1 Hz, 2 H, $CH_2CH_2OCH_2CH_2$), 2.83 (ddd, *J* = 11.9, 6.3, 3.0 Hz, 2 H, CH_2NCH_2), 2.72 (ddd, *J* = 11.6, 6.4, 3.1 Hz, 2 H, CH_2NCH_2), 2.43 (s, 3 H, C_q-CH_3); ^{13}C NMR (101 MHz, $CDCl_3$) δ 158.6 (Ar-C_q-OCH_3), 142.9 (Ar-C_q-S), 137.9 (Ar-C_q-C_q), 132.9 (Ar-C_q-CH_3), 129.4 (2 × Ar-CH), 127.4 (2 × Ar-CH), 127.0 (2 × Ar-CH), 113.6 (2 × Ar-CH), 87.1 (CH_2OCH_2), 84.7 (CH_2OCH_2), 66.2 ($CH_2CH_2OCH_2CH_2$), 61.4 (C_q), 55.4 (OCH_3), 46.9 (CH_2NCH_2), 21.4 (C_q-CH_3); HRMS (TOF-MS-ES$^+$) *m/z* calcd for $C_{21}H_{27}N_2O_4S^+$ [M+H]$^+$: 403.1692; found 403.1691.

***N*-(3-(4-Methoxyphenyl)oxetan-3-yl)-1,1-diphenyl-l^4-sulfanimine (77)**

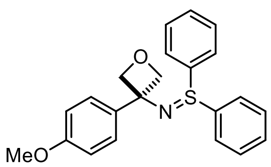

Prepared according to **deFS General Procedure A** under Ar at 60 °C for 2 h with a Celite work-up and using oxetane sulfonyl fluoride **1** (49.2 mg, 0.2 mmol, 1.0 equiv), K_2CO_3 (35.8 mg, 0.26 mmol,1.3 equiv), *S*,*S*-diphenyl sulfilimine monohydrate (132.0 mg, 0.6 mmol, 3.0 equiv) and anhydrous MeCN (0.67 mL, 0.3 M). Purification by filtration through a plug of silica (60% EtOAc/*n*-hexane) afforded oxetane sulfilimine **77** as a white solid (51.7 mg, 71%). R_f = 0.21 (60% EtOAc/*n*-hexane); mp

= 152–154 °C; IR (film)/cm^{-1} 3500, 3057, 2950, 2835, 2880, 2247, 1610, 1511, 1471, 1443, 1280, 1247, 1131, 1097, 831, 685, 619, 513; ^{1}H NMR (400 MHz, $CDCl_3$) δ 7.63–7.41 (m, 10 H, 10 × Ar-CH), 6.99 (d, *J* = 8.8 Hz, 2 H, 2 × Ar-CH), 6.62 (d, *J* = 8.8 Hz, 2 H, 2 × Ar-CH), 5.48 (d, *J* = 6.7 Hz, 2 H, C*H*HOC*H*H), 5.02 (d, *J* = 6.7 Hz, 2 H, CH*H*OCH*H*), 3.77 (s, 3 H, OCH_3); ^{13}C NMR (101 MHz, $CDCl_3$) δ 159.3 (Ar-C_q-OCH_3), 137.5 (Ar-C_q-S), 132.3 (2 × Ar-CH), 129.8 (4 × Ar-CH), 129.7 (2 × Ar-CH), 128.5 (Ar-C_q-C_q), 126.9 (4 × Ar-CH), 113.5 (2 × Ar-CH), 78.4 (CH_2OCH_2), 68.0 (C_q), 55.3 (OCH_3); HRMS (TOF-MS-ES$^+$) *m/z* calcd for $C_{22}H_{22}NO_2S^+$ [M+H]$^+$: 364.1371; found 364.1385.

Oxetane deFS with phosphorous nucleophiles (78–87)

Dimethyl (3-(4-methoxyphenyl)oxetan-3-yl)phosphonate (78)

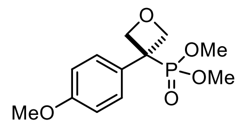

Prepared according to **deFS General Procedure A** under Ar at 60 °C for 2 h with a Celite work-up and using oxetane sulfonyl fluoride **1** (24.6 mg, 0.1 mmol, 1.0 equiv), K_2CO_3 (18.0 mg, 0.13 mmol, 1.3 equiv), trimethyl phosphite (14 µL, 0.12 mmol, 1.2 equiv) and anhydrous MeCN (0.34 mL, 0.3 M). Purification by flash column chromatography (5% MeOH/CH_2Cl_2) afforded oxetane phosphonate **78** as a white solid (11.2 mg, 41%). R_f = 0.26 (5% MeOH/CH_2Cl_2); mp = 93–94 °C; IR (film)/cm^{-1} 2954, 2844, 1609, 1510, 1460, 1243 (P=O st), 1010, 928, 817, 779, 608, 562; ^{1}H NMR (400 MHz, $CDCl_3$) δ 7.07 (dd, $^3J_{H\text{-}H}$ = 8.9, $^4J_{H\text{-}P}$ = 2.7 Hz, 2 H, 2 × Ar-CH), 6.92 (d, *J* = 8.6 Hz, 2 H, 2 × Ar-CH), 5.22 (dd, $^3J_{H\text{-}P}$ = 16.1, $^2J_{H\text{-}H}$ = 6.0 Hz, 2 H, C*H*HOC*H*H), 5.02 (dd, $^3J_{H\text{-}P}$ = 18.3, $^2J_{H\text{-}H}$ = 6.0 Hz, 2 H, CH*H*OCH*H*), 3.81 (s, 3 H, Ar-OCH_3), 3.69 (d, $^3J_{H\text{-}P}$ = 10.5 Hz, 6 H, $P(OCH_3)_2$); ^{13}C NMR (101 MHz, $CDCl_3$) δ 158.9 (Ar-C_q-OCH_3), 130.0 (d, $^2J_{C\text{-}P}$ = 2.8 Hz, Ar-C_q-C_q), 128.4 (d, $^3JC_{\text{-}P}$ = 4.6 Hz, 2 × Ar-CH), 114.1 (d, $^4J_{C\text{-}P}$ = 3.1 Hz, 2 × Ar-CH), 78.3 (d, $^2J_{C\text{-}P}$ = 5.2 Hz, CH_2OCH_2), 55.3 (OCH_3), 53.9 (d, $^2J_{C\text{-}P}$ = 7.2 Hz, $P(OCH_3)_2$), 45.6 (d, $^1J_{C\text{-}P}$ = 146.3 Hz, C_q); ^{31}P{^{1}H} NMR (162 MHz, $CDCl_3$) δ 27.4 (s, $P(OCH_3)_2$); HRMS (TOF-MS-ES$^+$) *m/z* calcd for $C_{12}H_{18}O_5P^+$ [M+H]$^+$: 273.0892, found: 273.0883.

Diisopropyl (3-(4-methoxyphenyl)oxetan-3-yl)phosphonate (79)

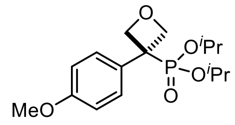

Prepared according to **deFS General Procedure A** under Ar at 60 °C for 2 h with a Celite work-up and using oxetane sulfonyl fluoride **1** (24.6 mg, 0.1 mmol, 1.0 equiv), triisopropyl phosphite (74 µL, 0.3 mmol, 3.0 equiv) and anhydrous MeCN (0.34 mL, 0.3 M). Purification by flash column chromatography (80% EtOAc/pentane) afforded oxetane phosphonate **79** as a colorless oil (8.2 mg, 25%). R_f = 0.27 (80% EtOAc/pentane); IR (film)/cm^{-1} 2978, 2879, 1513, 1247 (P=O st), 999 (P-O-C al st), 981 (P-O-C al st); ^{1}H NMR (400 MHz, $CDCl_3$) δ 7.12–7.04 (m, 2 H, 2 × Ar-CH), 6.93–6.86 (m, 2 H, 2 × Ar-CH), 5.21 (dd, $^3J_{H\text{-}P}$ = 15.9, $^2J_{H\text{-}H}$ = 5.9 Hz, 2 H), 4.99 (dd, $^3J_{H\text{-}P}$ = 17.3, $^2J_{H\text{-}H}$ = 5.9, 2 H), 4.58 (h, *J* = 6.4 Hz, 2 H, P(OC*H*(CH_3)$_2$), 3.81 (s, 3 H, OCH_3), 1.30 (d, *J* = 6.7 Hz, 6 H, P(OCH(C*H*$_3$)$_2$), 1.19 (d, *J* = 6.1 Hz, 6 H, P(OCH(C*H*$_3$)$_2$); ^{13}C NMR (101 MHz, $CDCl_3$) δ 158.6 (d, $^5J_{C\text{–}P}$ = 3.3 Hz, Ar-C_q-OCH_3), 130.8 (d, $^2J_{C\text{–}P}$ = 3.2 Hz, Ar-C_q-C_q), 128.6 (d, $^3J_{C\text{–}P}$ = 4.7 Hz, 2 × Ar-CH), 113.7 (d, $^4J_{C\text{–}P}$ = 3.2 Hz, 2 × Ar-CH), 78.4 (d, $^2J_{C\text{–}P}$ = 5.1 Hz, CH_2OCH_2), 71.4 (d, $^2J_{C\text{–}P}$ = 7.4 Hz, P(O*C*H(CH_3)$_2$), 55.3 (OCH_3), 45.6 (d, $^1J_{C\text{–}P}$ = 148.3 Hz, C_q), 24.2 (d, $^3J_{C\text{–}P}$ = 3.1 Hz, P(OCH(*C*H$_3$)$_2$), 23.7 (d, $^3J_{C\text{–}P}$ = 5.7 Hz, P(OCH(*C*H$_3$)$_2$); ^{31}P{^{1}H} NMR (162 MHz, $CDCl_3$) δ 23.0 (s, P(O^{i}Pr)$_2$; HRMS (TOF-MS-ES$^+$) *m/z* calcd for $C_{16}H_{26}O_5P^+$ [M+H]$^+$: 329.1518, found: 329.1528.

Diethyl (3-(4-methoxyphenyl)oxetan-3-yl)phosphonate (80)

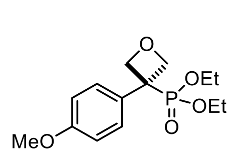

Prepared according to **deFS General Procedure A** under Ar at 60 °C for 2 h with a Celite work-up and using oxetane sulfonyl fluoride **1** (49.2 mg, 0.1 mmol, 1.0 equiv), triethyl phosphite (51.4 µL, 0.3 mmol, 3.0 equiv) and anhydrous MeCN

(0.34 mL, 0.3 M). Purification by flash column chromatography (EtOAc) afforded oxetane phosphonate **80** as a white solid (20.9 mg, 70%). R_f = 0.32 (5% MeOH/CH_2Cl_2); mp = 80–83 °C; IR (film)/cm^{-1} 2959, 2885, 1609, 1512, 1243 (P=O st), 1048, 1016, 961, 930, 837, 608, 555; ^{1}H NMR (400 MHz, $CDCl_3$) δ 7.11–7.03 (m, 2 H, 2 × Ar-CH), 6.97–6.87 (m, 2 H, 2 × Ar-CH), 5.28–5.18 (dd, 2 H, $^3J_{H\text{-}P}$ = 16.0, $^2J_{H\text{-}H}$ = 5.9 Hz, C*H*HOC*H*H), 5.06–4.95 (dd, 2 H, $^3J_{H\text{-}P}$ = 17.9, $^2J_{H\text{-}H}$ = 5.9 Hz, CH*H*OCH*H*), 4.12–3.90 (m, 4 H, P(OCH_2CH_3)$_2$), 3.81 (s, 3 H, OCH$_3$), 1.26 (t, *J* = 6.9 Hz, 6 H, P(OCH$_2$CH_3)$_2$); ^{13}C NMR (101 MHz, $CDCl_3$) δ 158.8 (d, $^5J_{C\text{–}P}$ = 3.3 Hz, Ar-C_q-OCH$_3$), 130.5 (d, $^2J_{C\text{–}P}$ = 3.1 Hz, Ar-C_q-C$_q$), 128.5 (d, $^3J_{C\text{–}P}$ = 4.6 Hz, 2 × Ar-CH), 113.9 (d, $^4J_{C\text{–}P}$ = 2.9 Hz, 2 × Ar-CH), 78.4 (d, $^2J_{C\text{–}P}$ = 5.1 Hz, CH$_2$OCH$_2$), 63.0 (d, $^2J_{C\text{–}P}$ = 7.2 Hz, P(OCH$_2$CH$_3$)$_2$), 55.3 (OCH$_3$), 45.6 (d, $^1J_{C\text{–}P}$ = 146.2 Hz, C$_q$), 16.5 (d, $^3J_{C\text{–}P}$ = 5.6 Hz, P(OCH$_2$$CH_3$)$_2$); ^{31}P{^{1}H} NMR (162 MHz, $CDCl_3$) δ 25.0 (s, P(OCH$_2$CH$_3$)$_2$); HRMS (TOF-MS-ES$^+$) *m/z* calcd for $C_{14}H_{12}O_5P^+$ [M+H]$^+$: 301.1205, found: 301.1204.

Notes:

***80** was further characterized by X-ray crystallography (see Fig. **S12–S14**). Crystals suitable for X-ray analysis were grown by slow evaporation from CDCl$_3$.*

Ethyl (3-(4-methoxyphenyl)oxetan-3-yl)(methyl)phosphinate (81)

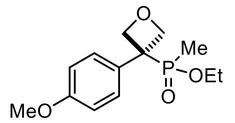

Prepared according to **deFS General Procedure A** under Ar at 60 °C for 2 h with a Celite work-up and using oxetane sulfonyl fluoride **1** (24.6 mg, 0.1 mmol, 1.0 equiv), diethyl methylphosphonite (45 µL, 0.3 mmol, 3.0 equiv) and anhydrous MeCN (0.34 mL, 0.3 M). Purification by flash column chromatography (5–10% MeOH/CH_2Cl_2) afforded oxetane phosphinate **81** as a white solid (14.9 mg, 55%). R_f = 0.21 (5% MeOH/CH_2Cl_2); mp = 91–93 °C; IR (film)/cm^{-1} 2949, 2880, 1515, 1300, 1257, 1205 (P=O st), 1042 (P-O-C al st), 1021 (P-O-C al st), 994, 957, 884, 780, 431; ^{1}H NMR (400 MHz, $CDCl_3$) δ 7.05–6.98 (m, 2 H, 2 × Ar-CH), 6.94–6.88 (m, 2 H, 2 × Ar-CH), 5.23–5.12 (m, 2 H, C*H*HOC*H*H), 5.09–5.01 (m, 2 H, CH*H*OCH*H*), 4.12–3.91 (m, 2 H, POCH_2CH$_3$), 3.81 (s, 3 H, OCH$_3$), 1.47 (d, $^2J_{H\text{–}P}$ = 13.4 Hz, 3 H, PCH$_3$), 1.27 (t, *J* = 7.0 Hz, 3 H, POCH$_2$CH_3); ^{13}C NMR (126 MHz, $CDCl_3$) δ 158.8 (d, $^5J_{C\text{–}P}$ = 3.1 Hz, Ar-C_q-OCH$_3$), 130.2 (Ar-C_q-C$_q$), 128.3 (d, $^3J_{C\text{–}P}$ = 4.0 Hz, 2 × Ar-CH), 114.0 (d, $^4J_{C\text{–}P}$ = 2.8 Hz, 2 × Ar-CH), 77.8 (CH$_2$OCH$_2$), 61.4 (d, $^2J_{C\text{–}P}$ = 6.9 Hz, POCH$_2$CH$_3$), 55.3 (OCH$_3$), 47.9 (d, $^1J_{C\text{–}P}$ = 92.7 Hz, C$_q$), 16.7 (d, $^3J_{C\text{–}P}$ = 5.5 Hz, POCH$_2$$CH_3$), 10.4 (d, $^1J_{C\text{–}P}$ = 93.5 Hz, PCH$_3$); ^{31}P{^{1}H} NMR (162 MHz, $CDCl_3$) δ 51.0 (s, P(CH$_3$)OCH$_2$CH$_3$); HRMS (TOF-MS-ES$^+$) *m/z* calcd for $C_{13}H_{20}O_4P^+$ [M+H]$^+$: 271.1099, found: 271.1090.

Methyl (3-(4-methoxyphenyl)oxetan-3-yl)(phenyl)phosphinate (82)

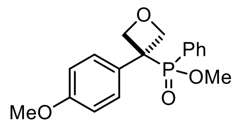

Prepared according to **deFS General Procedure A** under Ar at 60 °C for 2 h with a Celite work-up and using oxetane sulfonyl fluoride **1** (37.0 mg, 0.15 mmol, 1.0 equiv), dimethyl phenylphosphonite (72 µL, 0.45 mmol, 3.0 equiv) and anhydrous MeCN (0.51 mL, 0.3 M). Purification by flash column chromatography (5–10% MeOH/CH_2Cl_2) afforded oxetane phosphinate **82** as a white solid (26.6 mg, 56%). R_f = 0.26 (5% MeOH/CH_2Cl_2); mp = 102–105 °C; IR (film)/cm^{-1} 2949, 2877, 1609, 1511, 1439, 1296, 1248 (P=O st), 1224 (P=O st), 1180, 1116, 1028 (P-O-C al st), 724, 553; ^{1}H NMR (400 MHz, $CDCl_3$) δ 7.52 (t, *J* = 7.3 Hz, 1 H, Ph-CH), 7.45–7.29 (m, 4 H, 4 × Ph-CH), 6.81–6.67 (m, 4 H, 4 × Ar-CH), 5.40–5.31 (m, 2 H, C*H*HOC*H*H), 5.02–4.92 (m, 2 H, CH*H*OCH*H*), 3.81–3.74 (m, 6 H, C$_q$-OCH$_3$ + POCH$_3$); ^{13}C NMR (126 MHz, $CDCl_3$) δ 158.6 (d, $^5J_{C\text{–}P}$ = 3.2 Hz, Ar-C_q-OCH$_3$), 132.8 (d, $^2J_{C\text{–}P}$ = 9.0 Hz, 2 × Ph-CH), 132.7 (d, $^4J_{C\text{–}P}$ = 2.9 Hz, Ph-CH), 130.3 (Ar-C_q-C$_q$), 128.8 (d, $^3J_{C\text{–}P}$ = 4.2 Hz, 2 × Ph-CH), 128.3 (d, $^3J_{C\text{–}P}$ = 12.3 Hz, 2 × Ar-CH), 126.8 (d, $^1J_{C\text{–}P}$ = 125.5 Hz, Ph-C$_q$-P), 113.5 (d, $^4J_{C\text{–}P}$ = 2.8 Hz, 2 × Ar-CH), 77.8 (CH$_2$OCH$_2$), 55.3 (C$_q$-OCH$_3$), 52.3 (d, $^2J_{C\text{–}P}$ = 6.9 Hz, POCH$_3$), 47.8 (d, $^1J_{C\text{–}P}$ = 99.3 Hz, C$_q$); ^{31}P{^{1}H} NMR (162 MHz, $CDCl_3$) δ 41.1 (s, P(Ph)OCH$_3$); HRMS (TOF-MS-ES$^+$) *m/z* calcd for $C_{17}H_{20}O_4P^+$ [M+H]$^+$: 319.1099, found: 319.1105.

(3-(4-Methoxyphenyl)oxetan-3-yl)diphenylphosphine oxide (83)

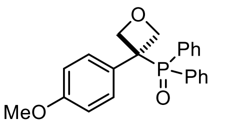

Prepared according to **deFS General Procedure A** under Ar at 60 °C for 2 h with a Celite work-up and using oxetane sulfonyl fluoride **1** (24.6 mg, 0.1 mmol, 1.0 equiv), dimethyl methoxydiphenylphosphine (60 µL, 0.3 mmol, 3.0 equiv) and anhydrous MeCN (0.34 mL, 0.3 M). Purification by flash column chromatography (EtOAc) afforded oxetane phosphine oxide **83** as a white solid (18.9 mg, 52%). R_f = 0.22 (EtOAc); mp = 96–98 °C; IR (film)/cm^{-1} 2931, 2877, 1610, 1512, 1437, 1250 (P=O st), 1183, 1115, 999, 930, 834, 721, 698, 543; ^{1}H NMR (400 MHz, $CDCl_3$) δ 7.74–7.65 (m, 4 H, 4 × Ph-CH), 7.58–7.47 (m, 2 H, 2 × 4 × Ph-CH), 7.43 (m, 4 H, 4 × Ph-CH), 6.79–6.69 (m, 4 H, 4 × Ar-CH), 5.46 (dd, $^3J_{H\text{-}P}$ = 13.7, $^2J_{H\text{-}H}$ = 6.1 Hz, 2 H, C*H*HOC*H*H), 5.04 (dd, $^3J_{H\text{-}P}$ = 14.5, $^2J_{H\text{-}H}$ = 6.1 Hz, 2 H, CH*H*OCH*H*), 3.78 (s, 3 H, OCH_3); ^{13}C NMR (126 MHz, $CDCl_3$) δ 158.6 (d, $^5J_{C\text{–}P}$ = 2.8 Hz, Ar-C_q-OCH_3), 132.3 (d, $^2J_{C\text{–}P}$ = 8.3 Hz, 4 × Ph-CH), 132.2 (d, $^4J_{C\text{–}P}$ = 2.8 Hz, 2 × Ar-CH), 130.5 (Ar-C_q-C_q), 129.5 (d, $^4J_{C\text{–}P}$ = 3.9 Hz, 2 × Ph-CH), 129.4 (d, $^1J_{C\text{–}P}$ = 97.1 Hz, 2 × Ph-C_q-P), 128.3 (d, $^3J_{C\text{–}P}$ = 11.4 Hz, 4 × Ph-CH), 113.4 (2 × Ar-CH), 78.1 (CH_2OCH_2), 55.3 (OCH_3), 48.8 (d, $^1J_{C\text{–}P}$ = 66.5 Hz, C_q); ^{31}P{^{1}H} NMR (162 MHz, $CDCl_3$) δ 29.3 (s, $PO(Ph)_2$); HRMS (TOF-MS-ES$^+$) *m/z* calcd for $C_{22}H_{22}O_3P^+$ $[M+H]^+$: 365.1307, found: 365.1299.

Diethyl (3-(4-(benzyloxy)phenyl)oxetan-3-yl)phosphonate (84)

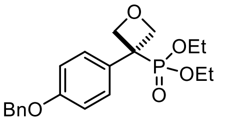

Prepared according to **deFS General Procedure A** under Ar at 70 °C for 2 h with a Celite work-up and using oxetane sulfonyl fluoride **132** (64.5 mg, 0.2 mmol, 1.0 equiv), triethyl phosphite (102.8 µL, 0.6 mmol, 3.0 equiv) and anhydrous MeCN (0.67 mL, 0.3 M). Purification by automated column chromatography (0–100% EtOAc/pentane, see conditions below) afforded oxetane phosphonate **84** as a white solid (46.0 mg, 61%). R_f = 0.27 (EtOAc); mp = 92.9–94.4 °C. IR (film)/cm^{-1} 2978, 2879, 1610, 1511, 1456, 1241 (P=O st), 1049, 1012, 960. ^{1}H NMR (400 MHz, $CDCl_3$) δ 7.49–7.30 (m, 5 H, 5 × Ph-CH), 7.11–7.05 (m, 2 H, 2 × Ar-CH), 7.02–6.95 (m, 2 H, 2 × Ar-CH), 5.24 (dd, 2 H, $^3J_{H\text{–}P}$ = 15.9, $^2J_{H\text{–}H}$ = 6.0 Hz, C*H*HOC*H*H), 5.08 (s, 2 H, $PhCH_2$), 5.02 (dd, 2 H, $^3J_{H\text{–}P}$ = 17.7, $^2J_{H\text{–}H}$ = 6.0 Hz, CH*H*OCH*H*), 4.16–3.91 (m, 4 H, 2 × $P(OCH_2CH_3)_2$), 1.27 (t, *J* = 7.1 Hz, 6 H, $P(OCH_2CH_3)_2$). ^{13}C NMR (101 MHz, $CDCl_3$) δ 157.9 (d, $^5J_{C\text{–}P}$ = 3.0 Hz, Ar-C_q-OBn), 136.7 (Ar-C_q-CH_2O), 130.7 (d, $^2J_{C\text{–}P}$ = 3.0 Hz, Ar-C_q-C_q), 128.6 (Ar-CH), 128.5 (2 × Ar-CH), 128.0 (2 × Ar-CH), 127.4 (2 × Ar-CH), 114.8 (2 × Ar-CH), 78.3 (d, $^2J_{C\text{–}P}$ = 6.1 Hz, CH_2OCH_2), 70.0 ($PhCH_2$), 63.0 (d, $^2J_{C\text{–}P}$ = 8.1 Hz, $P(OCH_2CH_3)_2$), 45.5 (d, $^1J_{C\text{–}P}$ = 146.1 Hz, C_q), 16.4 (d, $^3J_{C\text{–}P}$ = 6.1 Hz, $P(OCH_2CH_3)_2$); ^{31}P{^{1}H} NMR (162 MHz, $CDCl_3$) δ 24.9 (s, $P(OCH_2CH_3)_2$); HRMS (TOF-MS-ES$^+$) *m/z* calcd for $C_{20}H_{25}O_5P^+$ $[M+Na]^+$: 399.1337; found: 399.1346.

Automated Column Conditions: Run on a Biotage® Selekt system. Column type: Biotage® Sfar® HC 5 g. Flow rate: 18 mL/min. Sample mass: 97 mg. Solvent A: pentane, Solvent B: EtOAc. UV wavelength detection: l 200–400 nm. See trace and gradient below. From the left: peak 1 (green): oxetane fluoride; peak 2 (yellow): oxetane phosphonate **84**.

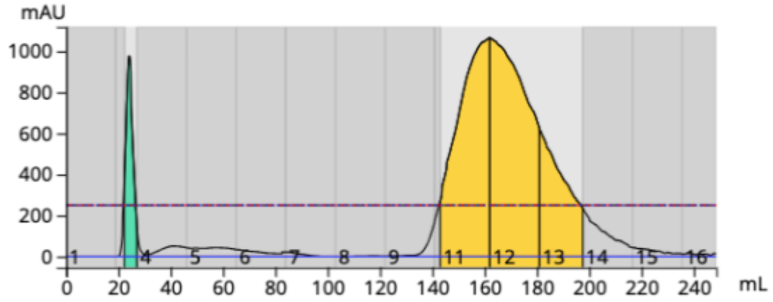

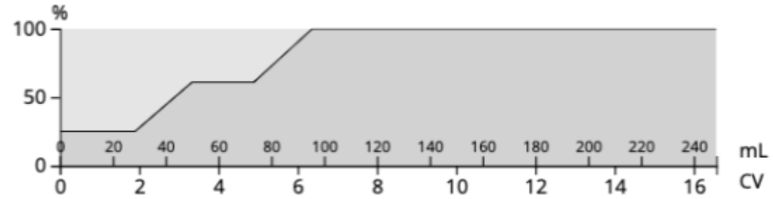

Notes:

***84** was further characterized by X-ray crystallography (see Fig. **S15**). Crystals suitable for X-ray analysis were grown by slow evaporation from $CDCl_3$.*

Diethyl (3-(benzo[*d*][1,3]dioxol-5-yl)oxetan-3-yl)phosphonate (85)

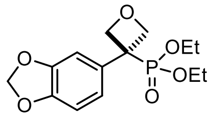

Prepared according to **deFS General Procedure A** under Ar at 60 °C for 4 h with a Celite work-up and using oxetane sulfonyl fluoride **7** (52.0 mg, 0.2 mmol, 1.0 equiv), triethyl phosphite (102.8 µL, 0.6 mmol, 3.0 equiv) and anhydrous MeCN (0.67 mL, 0.3 M). Purification by flash column chromatography (70–100% EtOAc/pentane) afforded oxetane phosphonate **85** as white crystals (45.0 mg, 72%). R_f = 0.25 (EtOAc); mp = 100–102 °C; IR (film)/cm^{-1} 2980, 2879, 1504, 1485, 1243 (P=O st), 1243, 1222, 1028, 1012 (P-O-C st), 943 (P-O-C st), 895, 813, 628; ^{1}H NMR (400 MHz, $CDCl_3$) δ 6.80 (d, *J* = 8.0 Hz, 1 H, Ar-CH), 6.65–6.55 (m, 2 H, 2 × Ar-CH), 5.96 (s, 2 H, OCH_2O), 5.19 (dd, $^3J_{H\text{-}P}$ = 15.9, $^2J_{H\text{-}H}$ = 6.0 Hz, 2 H, C*H*HOC*H*H), 4.97 (dd, $^3J_{H\text{-}P}$ = 17.8, $^2J_{H\text{-}H}$ = 6.0 Hz, 2 H), 4.14–3.93 (m, 4 H, P(OC*H*$_2$CH$_3$)$_2$), 1.28 (t, *J* = 7.0 Hz, 6 H, P(OCH$_2$C*H*$_3$)$_2$); ^{13}C NMR (101 MHz, $CDCl_3$) δ 147.7 (d, $^5J_{C\text{–}P}$ = 3.1 Hz, Ar-*C*$_q$-OCH$_2$), 146.8 (d, $^5J_{C\text{–}P}$ = 3.0 Hz, Ar-*C*$_q$-OCH$_2$), 132.3 (d, $^2J_{C\text{–}P}$ = 3.5 Hz, Ar-*C*$_q$-C$_q$), 120.7 (d, $^3J_{C\text{–}P}$ = 5.6 Hz, Ar-CH), 108.4–107.9 (m, 3 × Ar-CH), 101.2 (OCH_2O), 78.3 (d, $^2J_{C\text{–}P}$ = 5.2 Hz, CH_2OCH_2), 63.1 (d, $^2J_{C\text{–}P}$ = 7.1 Hz, P(O*C*H$_2$CH$_3$)$_2$), 46.1 (d, $^1J_{C\text{–}P}$ = 146.4 Hz, C$_q$), 16.5 (d, $^3J_{C\text{–}P}$ = 5.6 Hz, P(OCH$_2$*C*H$_3$)$_2$); ^{31}P{^{1}H} NMR (162 MHz, $CDCl_3$) δ 24.7 (s, P(OCH$_2$CH$_3$)$_2$); HRMS (TOF-MS-ES$^+$) *m/z* calcd for $C_{14}H_{20}O_6P^+$ [M+H]$^+$: 315.0998, found: 315.0996.

3-(4-Methoxyphenyl)oxetan-3-yl diphenylphosphinate (86)

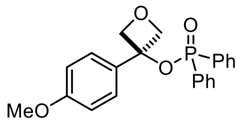

Prepared according to **deFS General Procedure A** under Ar at 60 °C for 2 h with a Celite work-up and using oxetane sulfonyl fluoride **1** (24.6 mg, 0.1 mmol, 1.0 equiv), K_2CO_3 (41.4 mg, 0.3 mmol, 3.0 equiv), diphenylphosphine oxide (61.0 mg, 0.3 mmol, 3.0 equiv) and anhydrous MeCN (0.34 mL, 0.3 M). Purification by flash column chromatography (40–100% EtOAc/pentane) afforded oxetane phosphinate **86** as a white solid (22.4 mg, 59%). R_f = 0.26 (50% EtOAc/pentane); mp = 104–106 °C; IR (film)/cm^{-1} 2922, 2879, 1612, 1514, 1438, 1249, 1218 (P=O st), 1101, 966, 916, 724, 690, 534; ^{1}H NMR (400 MHz, $CDCl_3$) δ 7.68–7.58 (m, 4 H, 4 × Ph-CH), 7.48–7.40 (m, 2 H, 2 × Ph-CH), 7.39–7.28 (m, 6 H, 4 × Ph-CH + 2 × Ar-CH), 6.70 (d, *J* = 8.7 Hz, 2 H, 2 × Ar-CH), 5.37 (d, *J* = 7.3 Hz, 2 H, C*H*HOC*H*H), 5.01 (d, *J* = 7.2 Hz, 2 H, CH*H*OCH*H*), 3.75 (s, 3 H, OCH_3); ^{13}C NMR (101 MHz, $CDCl_3$) δ 159.5 (Ar-*C*$_q$-OCH$_3$), 132.6 (d, $^1J_{C\text{–}P}$

= 137.0 Hz, 2 × Ph-C_q-P), 131.8 (d, $^4J_{C-P}$ = 2.9 Hz, 2 × Ph-CH),131.3 (d, $^3J_{C-P}$ = 10.3 Hz, 4 × Ph-CH + Ar-C_q-C_q), 128.3 (d, $^2J_{C-P}$ = 13.5 Hz, 4 × Ph-CH), 128.0 (2 × Ar-CH), 113.6 (2 × Ar-CH) 83.2 (d, $^3J_{C-P}$ = 6.6 Hz, CH_2OCH_2), 81.7 (d, $^2J_{C-P}$ = 7.8 Hz, C_q), 55.3 (OCH_3); $^{31}P\{^1H\}$ NMR (162 MHz, $CDCl_3$) δ 30.8 (s, $OP(O)(Ph)_2$); HRMS (TOF-MS-ES$^+$) *m/z* calcd for $C_{22}H_{22}O_4P^+$ $[M+H]^+$: 381.1256, found: 381.1273.

3-(4-Methoxyphenyl)oxetan-3-yl dimethylphosphinate (87)

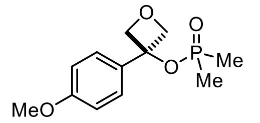

Prepared according to **deFS General Procedure A** under Ar at 60 °C for 2 h with a Celite work-up and using oxetane sulfonyl fluoride **1** (24.6 mg, 0.1 mmol, 1.0 equiv), K_2CO_3 (41.4 mg, 0.3 mmol, 3.0 equiv), dimethylphosphine oxide (23.4 mL, 0.3 mmol, 3.0 equiv) and anhydrous MeCN (0.34 mL, 0.3 M). Purification by flash column chromatography (50% EtOAc/*n*-hexane then 2% MeOH/EtOAc) afforded oxetane phosphinate **87** as a white crystalline solid (7.1 mg, 28%). R_f = 0.33 (5% MeOH/CH_2Cl_2); mp = 115–117 °C; IR (film)/cm^{-1} 2919, 2848, 1611, 1515, 1463, 1294, 1255, 1195 (P=O st), 1171, 1117, 1028, 940, 878, 808, 615, 530; ^{1}H NMR (400 MHz, $CDCl_3$) δ 7.43 (d, *J* = 8.8 Hz, 2 H, 2 × Ar-CH), 6.94 (d, *J* = 8.8 Hz, 2 H, 2 × Ar-CH), 5.23 (d, *J* = 7.2 Hz, 2 H, C*H*HOC*H*H), 5.05 (d, *J* = 7.2 Hz, 2 H, CH*H*OCH*H*), 3.83 (s, 3 H, OCH_3), 1.19 (d, $^2J_{H-P}$ = 14.0 Hz, 6 H, 2 × PCH_3); ^{13}C NMR (101 MHz, $CDCl_3$) δ 159.9 (Ar-C_q-OCH_3), 131.4 (Ar-C_q-C_q), 128.4 (2 × Ar-CH), 114.1 (2 × Ar-CH), 83.0 (d, $^3J_{C-P}$ = 7.2 Hz, CH_2OCH_2), 80.5 (d, $^2J_{C-P}$ = 7.6 Hz, C_q), 55.4 (OCH_3), 17.6 (d, $^1J_{C-P}$ = 94.1 Hz, 2 × PCH_3); $^{31}P\{^1H\}$ NMR (162 MHz, $CDCl_3$) δ 53.6 (s, $OP(O)(Me)_2$); HRMS (FTMS–APCI$^+$) *m/z* calcd for $C_{10}H_{11}O_2^+$ $[M-C_2H_6O_2P]^+$: 163.0754, found: 163.0755.

Notes:

***87** was further characterized by X-ray crystallography (see Fig. **S16–S18**). Crystals suitable for X-ray analysis were grown by slow evaporation from $CDCl_3$.*

Oxetane deFS with amines (142, S23, 144, S24, S26–28)

Methyl (3-(4-(prop-2-yn-1-yloxy)phenyl)oxetan-3-yl)-*L*-serinate (142)

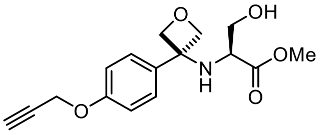

Prepared according to **General Procedure A** under Ar at 60 °C for 2 h with a Celite work-up and using oxetane sulfonyl fluoride **134** (67.6 mg, 0.25 mmol, 1.0 equiv), K_2CO_3 (90 mg, 0.65 mmol, 1.3 equiv), *L*-serine methyl ester hydrochloride (46.7 mg, 0.3 mmol, 1.2 equiv) and anhydrous MeCN (0.83 mL, 0.3 M). Purification by flash column chromatography (70% EtOAc/pentane) afforded amino-oxetane **142** as a colorless gum (48.3 mg, 63%). $[\alpha]_D^{21}$ = –22.4 (c 1.0, $CHCl_3$); R_f = 0.33 (80% EtOAc/pentane); IR (film)/cm^{-1} 3416 (NH st), 3284 (OH st), 2950, 2876, 1731 (C=O st), 1610, 1513, 1221, 1180, 1053, 1023, 978, 833; ^{1}H NMR (400 MHz, $CDCl_3$) δ 7.25–7.16 (m, 2 H, 2 × Ar-CH), 7.02–6.94 (m, 2 H, 2 × Ar-CH), 5.03 (d, *J* = 6.5 Hz, 1 H, C*H*HOCH_2), 4.91 (d, *J* = 6.4 Hz, 1 H, CH*H*OCH_2), 4.75 (d, *J* = 6.5 Hz, 1 H, CH_2OC*H*H), 4.73–4.69 (m, 3 H, CH_2OCH*H* and C*H*$_2$CCH), 3.63 (s, 3 H, OCH_3), 3.49 (d, *J* = 5.6 Hz, 2 H, C*H*$_2$OH), 3.36 (t, *J* = 5.5 Hz, 1 H, N-CH-C_q=O), 2.79 (br s, 1 H, NH), 2.60 (br s, 1 H, OH), 2.53 (t, *J* = 2.4 Hz, 1 H, CH_2CC*H*); ^{13}C NMR (101 MHz, $CDCl_3$) δ 173.9 (C_q=O), 157.0 (Ar-C_q-OCH_2CCH), 134.9 (Ar-C_q-C_q), 127.6 (2 × Ar-CH), 115.1 (2 × Ar-CH), 82.8 (*C*H_2OCH_2), 81.9 (CH_2O*C*H_2), 78.4 (CH_2*C*CH), 75.7 (CH_2C*C*H), 63.3 (CH_2OH), 62.8 (C_q), 57.8 (N-*C*H-C_q=O), 55.8 (*C*H_2CCH), 52.4 (CO_2*C*H_3); HRMS (TOF-MS-ES$^+$) *m/z* calcd for $C_{16}H_{20}NO_5^+$ $[M+H]^+$: 306.1341, found: 306.1341.

5'-O-*tert*-Butyldimethylsilyl-2'-3'-O-isopropylideneadenosine (S23)

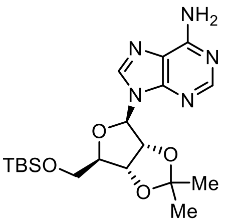

Prepared according to a literature procedure.[36] Imidazole (255 mg, 3.75 mmol, 2.5 equiv) was added to a solution of 2'-3'-O-isopropylideneadenosine (461.0 mg, 1.5 mmol, 1.0 equiv) in DMF (5.0 mL, 0.3 M) at 0 °C in a 25 mL round-bottom flask. *tert*-Butyldimethylsilyl chloride (272 mg, 1.8 mmol, 1.2 equiv) was added and the resulting reaction mixture stirred at 0 °C for 2 h, then allowed to warm to room temperature and stirred for 16 h. The reaction mixture was concentrated *in vacuo* using a rotary evaporator. The resulting residue was diluted with 2:1 EtOAc/water (100 mL) and the layers separated. The organic layer was washed with water (3 × 50 mL) and brine (40 mL), dried over Na_2SO_4, filtered and concentrated *in vacuo* to afford aniline **S23** as a white solid (632 mg, quant.). R_f = 0.18 (60% EtOAc/pentane); ^{1}H NMR (400 MHz, $CDCl_3$) δ 8.39 (s, 1 H, NCHN), 8.07 (s, 1 H, Ar-CH), 6.18 (d, *J* = 2.5 Hz, 1 H, NCHO), 5.63 (s, 2 H, NH_2), 5.28 (dd, *J* = 6.2, 2.5 Hz, 1 H, NCHC*H*O), 4.96 (dd, *J* = 6.2, 2.5 Hz, 1 H, $TBSOCH_2CHCHO$), 4.44 (td, *J* = 3.9, 2.3 Hz, 1 H, $TBSOCH_2CH$), 3.89 (dd, *J* = 11.2, 3.9 Hz, 1 H, TBSOC*H*H), 3.77 (dd, *J* = 11.2, 4.2 Hz, 1 H, TBSOCH*H*), 1.64 (s, 3 H, $C(CH_3)(CH_3)$), 1.41 (s, 3 H, $C(CH_3)(CH_3)$), 0.85 (s, 9 H, $SiC(CH_3)_3$), 0.02 (d, *J* = 2.6 Hz, 6 H, $Si(CH_3)_2$). The observed characterization data (^{1}H) were consistent with that previously reported.[36]

N-(3-(4-(allyloxy)phenyl)oxetan-3-yl)-9-((3aS,4S,6S,6aS)-6-(((*tert*-butyldimethylsilyl)oxy)methyl)-2,2-dimethyltetrahydrofuro[3,4-*d*][1,3]dioxol-4-yl)-9*H*-purin-6-amine (144)

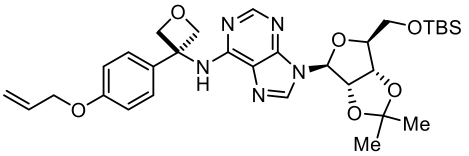

Prepared according to **deFS General Procedure A** under Ar at 60 °C for 18 h with a Celite work-up and using oxetane sulfonyl fluoride **135** (27.2 mg, 0.10 mmol, 1.0 equiv), K_2CO_3 (18.0 mg, 0.13 mmol, 1.3 equiv), aniline **S23** (63.2 mg, 0.15 mmol, 0.15 equiv) and anhydrous MeCN (0.34 mL, 0.3 M). Purification by flash column chromatography (30% EtOAc/pentane) afforded amino-oxetane **144** as a colorless gum (18.5 mg, 30%). R_f = 0.56 (50% EtOAc/pentane); IR (film)/cm^{-1} 2952, 2857, 1614 (C=C st), 1511, 1472, 1373, 1295, 1249, 1215, 1088, 837; ^{1}H NMR (400 MHz, $CDCl_3$) δ 8.29 (s, 1 H, NCHN), 8.07 (s, 1 H, $Ar_{(adenosine)}$-CH), 7.51 (d, *J* = 8.8 Hz, 2 H, 2 × Ar-CH), 6.89 (d, *J* = 8.8 Hz, 2 H, 2 × Ar-CH), 6.17 (d, *J* = 2.4 Hz, 1 H, NCHO), 6.04 (ddt, *J* = 16.2, 10.5, 5.3 Hz, 1 H, $CH_2CH{=}CH_2$), 5.40 (dd, *J* = 17.3, 1.6 Hz, 1 H, $CH_2CH{=}CHH$), 5.31–5.24 (m, 2 H, $CH_2CH{=}CHH$ and NCHC*H*O), 5.18 (m, 2 H, CH_2OCH_2), 5.04 (dd, *J* = 6.7, 3.0 Hz, 2 H, CH_2OCH_2), 4.94 (dd, *J* = 6.2, 2.4 Hz, 1 H, $TBSOCH_2CHCHO$), 4.52 (d, *J* = 5.4 Hz, 2 H, $OCH_2CH{=}CH_2$), 4.45 (q, *J* = 3.5 Hz, 1 H, $TBSOCH_2CH$), 3.89 (dd, *J* = 11.3, 3.7 Hz, 1 H, TBSOC*H*H), 3.78 (dd, *J* = 11.2, 4.1 Hz, 1 H, TBSOCH*H*), 1.63 (s, 3 H, $C(CH_3)(CH_3)$), 1.40 (s, 3 H, $C(CH_3)(CH_3)$), 0.84 (s, 9 H, $SiC(CH_3)_3$), 0.02 (d, *J* = 3.3 Hz, 6 H, $Si(CH_3)_2$); ^{13}C NMR (101 MHz, $CDCl_3$) δ 157.9 (Ar-C_q-OCH_2), 153.1 (N-C_q-NH), 152.9 (N-CH-N), 152.2 (N-C_q-N), 139.0 (N-*C*H-NCH), 134.4 (Ar-C_q-C_q), 133.2 ($CH_2CH{=}CH_2$), 126.3 (2 × Ar-CH), 120.4 ($Ar_{(adenosine)}$-C_q), 117.7 ($CH_2CH{=}CH_2$), 114.7 (2 × Ar-CH), 114.1 ($C_q(CH_3)_2$), 91.5 (NCHO), 87.4 (NCH*C*HO), 85.0 ($TBSOCH_2CH$), 83.7 (CH_2OCH_2), 81.4 ($TBSOCH_2CHCHO$), 68.9 ($OCH_2CH{=}CH_2$), 63.6 (CH_2OTBS), 59.5 (C_q), 27.2 ($C_q(CH_3)(CH_3)$), 25.9 ($SiC(CH_3)_3$), 25.4 ($C_q(CH_3)(CH_3)$), 18.3 ($SiC(CH_3)_3$), -5.4 ($Si(CH_3)$), -5.5 ($Si(CH_3)$); HRMS (TOF-MS-ES+) *m/z* calcd for $C_{31}H_{44}N_5O_6Si^+$ $[M+H]^+$: 610.3061, found: 610.3091.

1-((2*R*,4*S*,5*S*)-4-Amino-5-(((*tert*-butyldimethylsilyl)oxy)methyl)tetrahydrofuran-2-yl)-5-methylpyrimidine-2,4(1*H*,3*H*)-dione (S24)

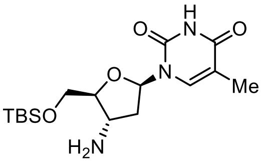

Prepared according to a patent procedure.[37] Imidazole (680 mg, 10.0 mmol, 5.0 equiv) was added to a solution of 1-((2*R*,4*S*,5*S*)-4-amino-5-(((*tert*-butyldimethylsilyl)oxy)methyl)tetrahydrofuran-2-yl)-5-methylpyrimidine-2,4(1*H*,3*H*)-dione (482.0 mg, 2.0 mmol, 1.0 equiv) in DMF (4.0 mL, 0.5 M). The reaction mixture was stirred at 60 °C for 5 min. *tert*-Butyldimethylsilyl chloride (362.0 mg, 6.0 mmol, 1.2 equiv) was added and the resulting reaction mixture stirred at 60 °C for 1 h. The reaction was quenched with sat. aq. $NaHCO_3$ (40 mL) and the aqueous layer extracted with EtOAc (4 × 50 mL). The combined organic fractions were washed with water (3 × 40 mL), brine (30 mL), dried over Na_2SO_4, filtered and concentrated *in vacuo* using a rotary evaporator. Purification by flash column chromatography (15–20% MeOH/EtOAc) afforded amine **S24** as a white solid (356 mg, 50%). R_f = 0.15 (20% MeOH/EtOAc); ^{1}H NMR (400 MHz, $CDCl_3$) δ 7.52 (s, 1 H, C_q=CH), 6.26 (t, *J* = 6.1 Hz, 1 H, OC*H*N), 3.94 (dd, *J* = 11.4, 3.0 Hz, 1 H, TBSOC*H*H), 3.85 (dd, *J* = 11.4, 2.7 Hz, 1 H, TBSOCH*H*), 3.73 (m, 1 H, C*H*NH_2), 3.69–3.60 (m, 1 H, TBSOCH_2C*H*), 2.26–2.15 (m, 2 H, NH_2CHCH_2), 1.93 (s, 3 H, C=$C_q$$CH_3$), 0.94 (s, 9 H, SiC$(CH_3)_3$), 0.13 (s, 3 H, Si$CH_3$), 0.12 (s, 3 H, Si$CH_3$). The observed characterization data (^{1}H) were consistent with that previously reported.[37]

1-(4-((3-(benzo[*d*][1,3]dioxol-5-yl)oxetan-3-yl-*tert*-butyldimethylsilylthymidine (S26)

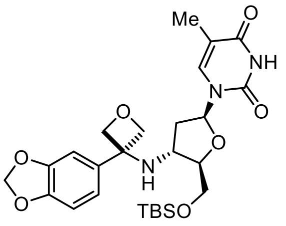

Prepared according to **deFS General Procedure A** under Ar at 60 °C for 4 h with a Celite work-up and using oxetane sulfonyl fluoride **7** (55.7 mg, 0.18 mmol, 1.0 equiv), K_2CO_3 (31.7 mg, 0.23 mmol, 1.3 equiv), amine **S24** (98.0 mg, 0.28 mmol, 1.5 equiv) and anhydrous MeCN (0.67 mL, 0.3 M). Purification by flash column chromatography (50% EtOAc/pentane) afforded amino-oxetane **S26** as a white solid (59.3 mg, 61%). R_f = 0.26 (60% EtOAc/pentane); mp = 83 °C; IR (film)/cm^{-1} 2952, 1683 (C=O st), 1230, 908, 725; ^{1}H NMR (400 MHz, $CDCl_3$) δ 8.66 (s, 1 H, N*H*$(CO)_2$), 7.29–7.24 (m, 2 H, 2 × Ar-CH), 6.84–6.75 (m, 1 H, NC*H*CCH_3), 6.75–6.69 (m, 2 H, 2 × Ar-CH), 6.23 (t, *J* = 6.4 Hz, 1 H, OCHN), 5.98 (s, 2 H, C$H_2$$(O)_2$Ar), 5.00 (d, *J* = 6.4 Hz, 1 H, C*H*HOCHH), 4.95 (d, *J* = 6.3 Hz, 1 H, CH*H*OCHH), 4.68 (dd, *J* = 6.3, 4.6 Hz, 2 H, CHHOC*HH*), 3.77 (dd, *J* = 11.1, 2.8 Hz, 1 H, C*H*HOTBS), 3.65 (dt, *J* = 5.4, 2.7 Hz, 1 H, C*H*CH_2OTBS), 3.58 (dd, *J* = 11.1, 2.6 Hz, 1 H, CH*H*OTBS), 3.24 (dt, *J* = 7.6, 5.2 Hz, 1 H, NHC*H*), 2.26 (s, 1 H, C_qN*H*), 2.06–1.93 (m, 1 H, NHCHC*H*HCH), 1.87 (s, 3 H, CCH_3), 1.85–1.76 (m, 1 H, NHCHCH*H*CH), 0.87 (s, 9 H, SiC$(CH_3)_3$), 0.03 (d, *J* = 8.1 Hz, 6 H, Si$(CH_3)_2$); ^{13}C NMR (101 MHz, $CDCl_3$) δ 163.7 (N*C*(O)C(CH_3)CH), 150.4 (NC(O)N), 148.4 (Ar-C_q-OCH_2O), 147.3 (Ar-C_q-OCH_2O), 136.2 (C(CH_3)*C*HN), 135.4 (Ar-C_q-C_q), 119.8 (Ar-CH), 111.0 (*C*(CH_3)CHN), 108.3 (Ar-CH), 107.1 (Ar-CH), 101.5 (*C*$H_2$$(O)_2$Ar), 85.9 (OCHN), 84.6 (*C*HCH_2OTBS), 83.2 (*C*H_2OCH_2), 82.8 (CH_2O*C*H_2), 63.6 (C_q), 62.9 (CH_2OTBS), 53.9 (NHCH), 40.6 (NHCH*C*H_2CH), 26.0 (SiC$(CH_3)_3$), 18.5 (Si*C*$(CH_3)_3$), 12.7 (CHCCH_3), –5.3 (Si$(CH_3)_2$); HRMS (TOF-MS-ES$^+$) *m/z* calcd for $C_{26}H_{38}N_3O_7Si^+$ $[M+H]^+$: 532.2479, found: 532.2463.

Methyl 3-isopropoxy-4-((3-(4-methoxyphenyl)oxetan-3-yl)amino)benzoate (S27)

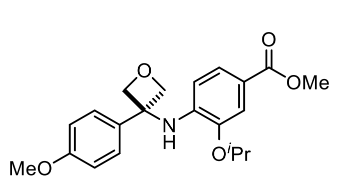

Prepared according to **deFS General Procedure A** under air at 60 °C for 2 h with a Celite work-up and using oxetane sulfonyl fluoride **5** (49.2 mg, 0.2 mmol, 1.0 equiv), K_2CO_3 (35.8 mg, 0.26 mmol, 1.3 equiv), aniline **S25** (50.0 mg, 0.24 mmol, 1.2 equiv) and anhydrous MeCN (0.67 mL, 0.3 M). Purification by flash column chromatography (10% EtOAc/pentane) afforded amino-oxetane **S27** as a white solid (47.2 mg, 64%). R_f = 0.14; mp = 121–124 °C; IR (film)/cm^{-1} 3418 (br NH st), 2948, 2872, 1700 (C=O st), 1600, 1512, 1437, 1358, 1276, 1203, 1179, 1109, 992, 908, 829, 730; ^{1}H NMR (400 MHz, $CDCl_3$) δ 7.52–7.49 (m, 3 H, 2 × $Ar_{(PMP)}$-CH + C_qiPrAr-C*H*Ar-$C_q$$CO_2$$CH_3$), 7.36 (dd, *J* = 8.3, 1.7 Hz, 1 H, C_qNHAr-CHAr-C*H*Ar-$C_q$$CO_2$$CH_3$), 6.91 (d, *J* = 8.1 Hz, 2 H, 2 × $Ar_{(PMP)}$-CH), 5.72 (d, *J* = 8.3 Hz, 1 H, C_qNHAr-C*H*Ar-CHAr-$C_q$$CO_2$$CH_3$), 5.54 (s, 1 H, NH),

4.98 (d, J = 6.3 Hz, 2 H, CHHOCHH), 4.94 (d, J = 6.3 Hz, 2 H, CHHOCHH), 4.73 (hept, J = 5.9 Hz, 1 H, CH(CH_3)$_2$), 3.82 (s, 3 H, CO_2CH_3), 3.80 (s, 3 H, Ar-OCH_3), 1.45 (d, J = 6.1 Hz, 6 H, CH(CH_3)$_2$); ^{13}C NMR (101 MHz, $CDCl_3$) δ 167.3 (CO_2CH_3), 158.9 (Ar-C_q-OCH_3), 144.2 (Ar-C_q-O(CH_3)$_2$), 139.8 (Ar-C_q-NH), 133.1 (Ar-C_q-C_q), 126.4 (2 × $Ar_{(PMP)}$-CH), 123.6 (C_qNHAr-CHAr-*C*HAr-$C_qCO_2CH_3$), 118.4 (Ar-C_q-CO_2CH_3), 114.1 (2 × $Ar_{(PMP)}$-CH), 113.0 (C_q^{i}PrAr-*C*HAr-$C_qCO_2CH_3$), 110.4 (C_qNHAr-*C*HAr-CHAr-$C_qCO_2CH_3$), 84.3 (CH_2OCH_2), 71.2 (*C*H(CH_3)$_2$), 59.4 (C_q-NH), 55.2 (Ar-OCH_3), 51.6 (CO_2*C*H_3), 22.2 (CH(*C*H_3)$_2$); HRMS (TOF-MS-ES$^+$) m/z calcd for $C_{21}H_{26}NO_5$ [M+H]: 372.1811, found: 372.1805.

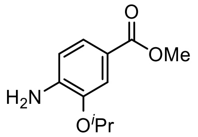

*Aniline **S25** (methyl 4-amino-3-isopropoxybenzoate, above) prepared according to literature procedures[24,25] affording an orange oil (361 mg, 92% over 3 steps).*

Methyl 3-isopropoxy-4-((3-(3-isopropoxy-4-methoxyphenyl)oxetan-3-yl)amino)benzoate (S28)

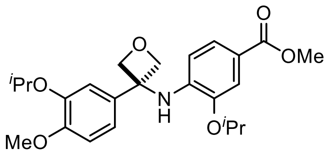

Prepared according to **deFS General Procedure A** under air at 60 °C for 2 h with a Celite work-up and using oxetane sulfonyl fluoride **5** (30.0 mg, 0.1 mmol, 1.0 equiv), K_2CO_3 (18.0 mg, 0.13 mmol, 1.3 equiv), aniline **S25** (28.0 mg, 0.12 mmol, 1.2 equiv) and anhydrous MeCN (0.34 mL, 0.3 M). Purification by flash chromatography (10% EtOAc/pentane) afforded amino-oxetane **S28** (31.0 mg, 72%) as a colorless oil. R_f = 0.08; IR (film)/cm^{-1} 3418 (br NH st), 2972, 2872, 2833, 2246, 1699 (C=O st), 1598, 1511, 1437, 1355, 1213, 1177, 1138, 1108, 1026, 991, 957, 907, 880, 810, 764, 727, 646, 610; ^{1}H NMR (400 MHz, $CDCl_3$) δ 7.49 (d, J = 1.8 Hz, 1 H, C_qOCH_3Ar-C_q^{i}PrAr-CHAr-C_q), 7.35 (dd, J = 8.3, 1.8 Hz, 1 H, C_qNHAr-CHAr-CHAr-$C_qCO_2CH_3$), 7.14– 7.11(m, 2 H, C_qOCH_3Ar-CHAr-CHAr-C_qAr + C_q^{i}PrAr-CHAr-$C_qCO_2CH_3$), 6.86 (d, J = 9.0 Hz, 1 H, C_qOCH_3Ar-CHAr-CHAr-C_q), 5.74 (d, J = 8.3 Hz, 1 H, C_qNHAr-CHAr-CHAr-$C_qCO_2CH_3$), 5.51 (s, 1 H, NH), 4.97 (d, J = 6.3 Hz, 2 H, CHHOCHH), 4.93 (d, J = 6.3 Hz, 2 H, CHHOCHH), 4.73 (hept, J = 6.1 Hz, 1 H, CH(CH_3)$_2$), 4.45 (hept, J = 6.1 Hz, 1 H, CH(CH_3)$_2$), 3.84 (s, 3 H, CO_2CH_3), 3.82 (s, 3 H, OCH_3), 1.44 (d, J = 6.1 Hz, 6 H, CH(CH_3)$_2$), 1.27 (d, J = 6.0 Hz, 6 H, CH(CH_3)$_2$); ^{13}C NMR (101 MHz, $CDCl_3$) δ 167.3 (CO_2CH_3), 149.7 (Ar-C_q-OCH_3), 147.3 (C_qOCH_3Ar-C_q^{i}PrAr), 144.1 (C_qNHAr-C_q^{i}PrAr), 139.9 (Ar-C_q-NH), 133.5 (Ar-C_q-C_q), 123.6 (C_qNHAr-CHAr-*C*HAr-$C_qCO_2CH_3$), 118.5 (Ar-C_q-CO_2CH_3), 118.0 (C_q^{i}PrAr-*C*HAr-$C_qCO_2CH_3$), 113.2 (C_qOCH_3Ar-*C*HAr-CHAr-C_qAr), 113.1 (C_q^{i}PrAr-*C*HAr-C_qAr), 111.9 (C_qOCH_3Ar-*C*HAr-CHAr-C_qAr), 110.6 (C_qNHAr-*C*HAr-CHAr-$C_qCO_2CH_3$), 84.3 (CH_2OCH_2), 71.4 (*C*H(CH_3)$_2$), 71.2 (*C*H(CH_3)$_2$), 59.5 (C_q-NH), 55.9 (Ar-OCH_3), 51.6 (CO_2*C*H_3), 22.2 (CH(*C*H_3)$_2$), 21.9 (CH(*C*H_3)$_2$); HRMS (TOF-MS-ES$^+$) m/z calcd for $C_{24}H_{32}NO_6$ [M+H]: 430.2230, found: 430.2233.

Further derivatization of oxetane deFS products (88–90, 143)

1-(3-(4-Methoxyphenyl)oxetan-3-yl)-4-(*p*-tolyl)-1*H*-1,2,3-triazole (88)

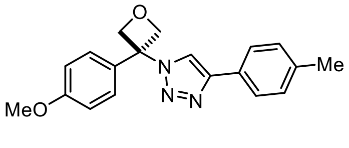

$Cu(OAc)_2$ (2.2 mg, 0.012 mmol, 10 mol%), a solution of oxetane azide **59** (25 mg, 0.12 mmol, 1.0 equiv) in MeCN (0.6 mL, 0.2 M), 4-ethynyltoluene (20 µL, 0.156 mmol, 1.3 equiv), and a solution of sodium ascorbate (71 mg, 0.36 mmol, 3.0 equiv) in H_2O (0.6 mL, 0.2 M) were added sequentially to a 10 mL round-bottom flask. After stirring at 25 °C for 24 h, sat. aq. $NaHCO_3$ (10 mL) and EtOAc (10 mL) were added sequentially. The phases were separated, and the organic layer was washed with sat. aq. $NaHCO_3$ (4 × 10 mL). The organic layer was dried over Na_2SO_4, filtered, and concentrated *in vacuo* using a rotary evaporator. Purification by flash column

chromatography (50% EtOAc/*n*-hexane) afforded oxetano-triazole **88** as a white solid (21 mg, 53%). R_f = 0.40 (50% EtOAc/*n*-hexane); mp = 172–174 °C; IR (film)/cm^{-1} 3132, 2957, 2887, 2837, 1612, 1581, 1515, 1459, 1437, 1252, 1229, 1183, 1026, 989, 931, 826, 732, 575, 551, 521; ^{1}H NMR (400 MHz, $CDCl_3$) δ 7.70 (d, *J* = 8.1 Hz, 2 H, 2 × Ar-CH), 7.49 (s, 1 H, NCH), 7.24–7.17 (m, 4 H, 4 × Ar-CH), 6.99–6.92 (m, 2 H, 2 × Ar-CH), 5.63 (d, *J* = 6.7 Hz, 2 H, C*H*HOC*H*H), 5.28 (d, *J* = 6.7 Hz, 2 H, C*H*HOC*H*H), 3.84 (s, 3 H, OCH_3), 2.38 (s, 3 H, CH_3); ^{13}C NMR (101 MHz, $CDCl_3$) δ 160.0 (Ar-C_q-OCH_3), 148.2 (Ar-C_q-Cq), 138.2 (N-C_q-CH), 131.3 (Ar-C_q-CH_3), 129.5 (2 × Ar-CH), 127.5 (Ar-C_q-C_q-N), 127.0 (2 × Ar-CH), 125.7 (2 × Ar-CH), 118.4 (NCH), 114.6 (2 × Ar-CH), 81.9 (CH_2OCH_2), 66.2 (C_q), 55.4 (OCH_3), 21.3 (CH_3); HRMS (TOF-MS-ES$^+$) *m/z* calcd for $C_{19}H_{20}N_3O_2$ [M+H]: 322.1556; found 322.1552.

(4-Ethynylphenyl)((3-(4-methoxyphenyl)oxetan-3-yl)imino)(methyl)-λ^6-sulfanone (89)

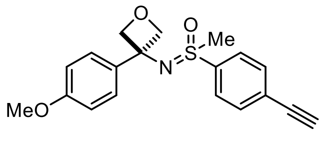

CuI (1.9 mg, 10 µmol, 5 mol%), $Pd(PPh_3)_4$ (5.8 mg, 5 µmol, 2.5 mol%), and oxetane sulfoximine **73** (79 mg, 0.2 mmol, 1.0 equiv) were added sequentially to a reaction vial. The reaction vial was sealed then evacuated and refilled with Ar three times. Anhydrous, degassed triethylamine (0.67 mL, 0.3 M) and TMS acetylene (0.42 mL, 0.3 mmol, 3.0 equiv) were added sequentially by syringe. After stirring at 90 °C for 20 h, the reaction mixture was cooled to room temperature and diluted with CH_2Cl_2 (10 mL), aq. HCl (1 M, 10 mL), and brine (10 mL). The layers were separated, and the aqueous layer was extracted with CH_2Cl_2 (3 × 10 mL). The combined organic layers were dried over Na_2SO_4, filtered, and concentrated *in vacuo* using a rotary evaporator. Purification by flash column chromatography (40–50% EtOAc/*n*-hexane) afforded TMS-protected oxetane sulfoximine **89a** as an impure beige solid which was carried onto the next step with no further purification.

K_2CO_3 (2.1 mg, 15 µmol, 10 mol%) was added to a solution of oxetane sulfoximine **89a** (62 mg, 0.15 mmol, 1.0 equiv) in MeOH (1.5 mL, 0.1 M). After stirring for 2 h at room temperature, the reaction mixture was concentrated *in vacuo* using a rotary evaporator. The residue was diluted with CH_2Cl_2 (10 mL) and aq. HCl (1M, 10 mL). The layers were separated, and the aqueous layer was extracted with CH_2Cl_2 (3 × 10 mL). The combined organic layers were dried over Na_2SO_4, filtered, and concentrated *in vacuo* using a rotary evaporator. Purification by column chromatography (50% EtOAc/*n*-hexane) afforded oxetane sulfoximine **89** as a white solid (47 mg, 92%, 74% over two steps). R_f = 0.23 (50% EtOAc/*n*-hexane); mp = 157–159 °C; IR (film)/cm^{-1} 3255 (≡C-H st), 2954, 2101, 1608, 1512, 1223, 1169, 1091, 1025, 976, 832, 761, 640, 544, 484; ^{1}H NMR (400 MHz, $CDCl_3$) δ 7.56 (d, *J* = 8.5 Hz, 2 H, 2 × Ar-CH), 7.42 (d, *J* = 8.5 Hz, 2 H, 2 × Ar-CH), 7.20 (d, *J* = 8.7 Hz, 2 H, 2 × Ar-CH), 6.66 (d, *J* = 8.7 Hz, 2 H, 2 × Ar-CH), 5.14 (d, *J* = 6.0 Hz, 1 H, C*H*HOCHH), 5.09 (d, *J* = 5.7 Hz, 1 H, CH*H*OCHH), 4.95 (d, *J* = 6.0 Hz, 1 H, CHHOC*H*H), 4.92 (d, *J* = 5.7 Hz, 1 H, CHHOCH*H*), 3.74 (s, 3 H, OCH_3), 3.24 (s, 1 H, CH), 3.00 (s, 3 H, SCH_3); ^{13}C NMR (101 MHz, $CDCl_3$) δ 158.6 (Ar-C_q-OCH_3), 142.1 (Ar-C_q-S), 135.6 (Ar-C_q-C_q), 132.4 (2 × Ar-CH), 127.5 (2 × Ar-CH), 127.5 (2 × Ar-CH), 126.0 (Ar-C_q-C), 113.4 (2 × Ar-CH), 86.4 (OCH_2), 84.7 (OCH_2), 82.0 (*C*°CH), 80.4 (C°*C*H), 62.2 (C_q), 55.2 (OCH_3), 46.9 (SCH_3); HRMS (TOF-MS-ES$^+$) *m/z* calcd for $C_{19}H_{20}NO_3S$ [M+H]: 342.1164; found 342.1164.

((3-(4-Methoxyphenyl)oxetan-3-yl)imino)(methyl)(4-morpholinophenyl)-λ^6-sulfanone (90)

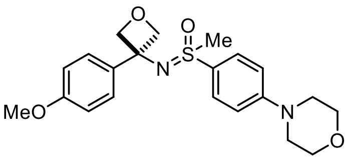

Using conditions developed by Buchwald,[32] $Pd(OAc)_2$ (2.2 mg, 10 μmol, 5 mol%), JohnPhos (6.0 mg, 20 μmol, 10 mol%), oxetane sulfoximine **73** (79 mg, 0.2 mmol, 1.0 equiv), and K_3PO_4 (85 mg, 0.4 mmol, 2.0 equiv) were added sequentially to a reaction vial. The reaction vial was sealed then evacuated and refilled with Ar three times. Anhydrous, degassed morpholine (26 μL, 0.3 mmol, 1.5 equiv) and THF (0.4 mL, 0.5 M) were added sequentially by syringe. After stirring at 65 °C for 23 h, the reaction mixture was cooled to room temperature and diluted with EtOAc (10 mL) and filtered through a plug of Celite, eluting with further EtOAc (3 × 10 mL). The solvent was then removed *in vacuo* using a rotary evaporator. Purification by filtration through a plug of silica (EtOAc) afforded oxetane sulfoximine **90** as a white solid (68 mg, 85%). R_f = 0.22 (EtOAc); mp = 140–142 °C; IR (film)/cm^{-1} 2953, 2866, 2833, 158, 1505, 1449, 1238, 1171, 1094, 1027, 974, 923, 823, 775, 726, 576, 529; ^{1}H NMR (400 MHz, $CDCl_3$) δ 7.55 (d, *J* = 8.8 Hz, 2 H, 2 × Ar-CH), 7.39 (d, *J* = 8.8 Hz, 2 H, 2 × Ar-CH), 6.76 (m, 4 H, 4 × Ar-CH), 5.14 (d, *J* = 5.9 Hz, 1 H, CH*H*OCHH), 5.11 (d, *J* = 5.7 Hz, 1 H, C*H*HOCHH), 4.85 (d, *J* = 5.7 Hz, 1 H, CHHOC*H*H), 4.78 (d, *J* = 5.9 Hz, 1 H, CHHOCH*H*), 3.89–3.80 (m, 4 H, $CH_2C\mathit{H}_2OC\mathit{H}_2CH_2$), 3.76 (s, 3 H, OCH_3), 3.30–3.17 (m, 4 H, CH_2NCH_2), 2.97 (s, 3 H, SCH_3); ^{13}C NMR (101 MHz, $CDCl_3$) δ 158.3 (Ar-C_q-OCH_3), 153.6 (Ar-C_q-N), 137.1 (Ar-C_q-S), 130.8 (Ar-C_q-C_q), 129.2 (2 × Ar-CH), 127.2 (2 × Ar-CH), 114.0 (2 × Ar-CH), 113.4 (2 × Ar-CH), 86.5 ($\mathit{C}H_2OCH_2$), 85.0 ($CH_2O\mathit{C}H_2$), 66.5 ($CH_2\mathit{C}H_2O\mathit{C}H_2CH_2$), 62.0 ($C_q$), 55.2 ($OCH_3$), 47.7 ($CH_2NCH_2$), 47.5 ($SCH_3$); HRMS (TOF-MS-ES$^+$) *m/z* calcd for $C_{21}H_{27}N_2O_4S$ [M+H]: 403.1692; found 403.1686.

Methyl (3-(4-((1-benzyl-1*H*-1,2,3-triazol-4-yl)methoxy)phenyl)oxetan-3-yl)-*L*-serinate (143)

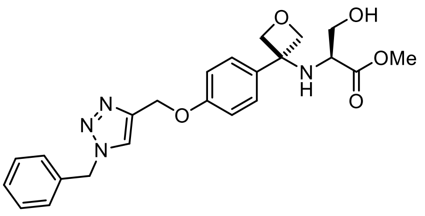

Benzyl azide (15 mg, 0.12 mmol, 1.2 equiv), sodium ascorbate (4 mg, 0.02 mmol, 20 mol%) and amino-oxetane **142** were added sequentially to a flame-dried 10 mL round-bottom flask (1). The flask (1) was evacuated and backfilled with Ar three times. A second 10 mL round-bottom flask (2) was charged with $CuSO_4$ (1 mg, 0.005 mmol, 5 mol%) and dissolved into ^{t}BuOH (0.33 mL) and water (0.17 mL). The $CuSO_4$ solution (2) was degassed, backfilled with Ar three times and transferred to reaction vessel (1) by syringe. The resulting solution was stirred at room temperature for 24 h. The reaction mixture was quenched with NH_4Cl (1 mL), the phases were separated and the aqueous portion was extracted with EtOAc (3 × 5 mL). The combined organic layers were dried over Na_2SO_4, filtered and concentrated *in vacuo* using a rotary evaporator. Purification by flash column chromatography (EtOAc) afforded amino-oxetane **143** as a colorless gum (37.6 mg, 86%). $[\alpha]_D^{21}$ = –24.0 (c 1.0, $CHCl_3$); R_f = 0.29 (EtOAc); IR (film)/cm^{-1} 3422 (br N-H and O-H st), 2950, 2876, 1733 (C=O st), 1608, 1513, 1241, 1181, 1051, 1008, 980, 835, 721; ^{1}H NMR (400 MHz, $CDCl_3$) δ 7.56 (s, 1 H, $Ar_{(triazole)}$-CH), 7.45–7.35 (m, 3 H, 3 × Ph-CH), 7.33–7.26 (m, 2 H, 2 × Ph-CH), 7.24–7.16 (m, 2 H, 2 × Ar-CH), 7.03–6.95 (m, 2 H, 2 × Ar-CH), 5.56 (s, 2 H, $PhCH_2$), 5.20 (s, 2 H, $Ar_{(triazole)}CH_2$), 5.04 (d, *J* = 6.5 Hz, 1 H, $C\mathit{H}HOCH_2$), 4.91 (d, *J* = 6.4 Hz, 1 H, $CH\mathit{H}OCH_2$), 4.76 (d, *J* = 6.5 Hz, 1 H, $CH_2OC\mathit{H}H$), 4.73 (d, *J* = 6.5 Hz, 1 H, $CH_2OCH\mathit{H}$), 3.63 (s, 3 H, OCH_3), 3.51 (d, *J* = 5.4 Hz, 2 H, $C\mathit{H}_2OH$), 3.36 (t, *J* = 5.4 Hz, 1 H, NH-C*H*-C_q=O), 2.75 (br s, 2 H, NH + OH); ^{13}C NMR (101 MHz, $CDCl_3$) δ 173.9 (C_q=O), 157.6 (Ar-C_q-$OCH_2Ar_{(triazole)}$), 144.4 ($Ar_{(triazole)}$-C_q), 134.6 (Ar-C_q-C_q), 134.4 (Ph-C_q), 129.2 (2 × PhCH), 128.9 (PhCH), 128.2 (2 × PhCH), 127.7 (2 × Ar-CH), 122.7 ($Ar_{(triazole)}$-CH), 115.0 (2 × Ar-CH), 82.9 ($\mathit{C}H_2OCH_2$), 81.9 ($CH_2O\mathit{C}H_2$), 63.3 (CH_2OH), 62.7 (C_q), 62.1 (O-CH_2-$Ar_{(triazole)}$), 57.8 (N-*C*H-C_q=O), 54.3 (CH_2CCH), 52.4 ($CO_2\mathit{C}H_3$); HRMS (TOF-MS-ES$^+$) *m/z* calcd for $C_{23}H_{27}N_4O_5^+$ [M+H]$^+$: 439.1981, found: 439.1988.

Oxetane SuFEx products (93–105, S29–31)

3-(4-Methoxyphenyl)-3-(methylsulfonyl)oxetane (93)

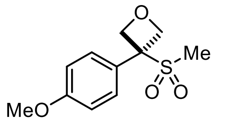

Methyllithium (1.6 M in Et_2O, 75.0 µL, 0.12 mmol, 1.2 equiv) was added dropwise to a solution of oxetane sulfonyl fluoride **1** (24.6 mg, 0.1 mmol, 1.0 equiv) in anhydrous THF (1.0 mL, 0.1 M) at –78 °C. Following the addition of methyllithium, the reaction vial was transferred from an acetone/dry-ice bath at –78 °C to a water/ice bath at 0 °C and stirred for 1 h. The reaction was quenched with sat. aq. NH_4Cl (5 mL), layers separated, and the aqueous portion extracted with EtOAc (3 × 5 mL). The organic fractions were combined, dried over Na_2SO_4, filtered and concentrated *in vacuo* using a rotary evaporator. Purification by flash column chromatography (80% Et_2O/pentane) afforded oxetane sulfone **93** as a white solid (16.9 mg, 70%). R_f = 0.14 (80% Et_2O/pentane); mp = 162–164 °C; IR (film)/cm^{-1} 2951, 2925, 1609, 1514, 1286 (SO_2 st as), 1245, 1184, 1135, 1024 (SO_2 st sy), 991, 927, 839, 518; ^{1}H NMR (400 MHz, $CDCl_3$) δ 7.17 (d, *J* = 8.7 Hz, 2 H, 2 × Ar-CH), 6.96 (d, *J* = 8.8 Hz, 2 H, 2 × Ar-CH), 5.36 (d, *J* = 7.2 Hz, 2 H, C*H*HOC*H*H), 5.14 (d, *J* = 7.2 Hz, 2 H, CH*H*OCH*H*), 3.83 (s, 3 H, C_q-OCH_3), 2.72 (s, 3 H, SO_2CH_3); ^{13}C NMR (101 MHz, $CDCl_3$) δ 160.3 (Ar-*C*$_q$-OCH_3), 129.4 (2 × Ar-CH), 125.7 (Ar-*C*$_q$-C_q), 114.4 (2 × Ar-CH), 76.4 (CH_2OCH_2), 68.3 (C_q), 55.4 (C_q-O*C*H_3), 35.4 (SO_2CH_3); HRMS (FTMS–APCI$^+$) *m/z* calcd for $C_{10}H_{11}O_2^+$ $[M-C_3H_3SO_2]^+$: 163.0754, found: 163.0732.

Notes:

93 *was further characterized by X-ray crystallography (see Fig.* ***S19–S21****). Crystals suitable for X-ray analysis were grown by slow evaporation from* $CDCl_3$*.*

3-(4-Methoxyphenyl)-3-(phenylsulfonyl)oxetane (94)

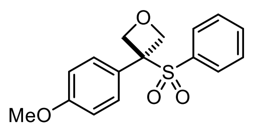

Phenyllithium (1.9 M in dibutylether, 63.0 µL, 0.12 mmol, 1.2 equiv) was added dropwise to a solution of oxetane sulfonyl fluoride **1** (24.6 mg, 0.1 mmol, 1.0 equiv) in anhydrous THF (1.0 mL, 0.1 M) at –78 °C. Following the addition of phenyllithium, the reaction vial was transferred from an acetone/dry-ice bath at –78 °C to a water/ice bath at 0 °C and stirred for 1 h. The reaction was quenched with sat. aq. NH_4Cl (5 mL), layers separated, and the aqueous portion extracted with EtOAc (3 × 5 mL). The organic fractions were combined, dried over Na_2SO_4, filtered and concentrated *in vacuo* using a rotary evaporator. Purification by flash column chromatography (30% EtOAc/pentane) afforded oxetane sulfone **94** as a white solid (17.2 mg, 59%). R_f = 0.14 (30% EtOAc/pentane); mp = 145–146 °C; IR (film)/cm^{-1} 2883, 1608, 1515, 1448, 1302 (SO_2 st as), 1247, 1148 (SO_2 st sy), 1019, 997, 835, 725, 554; ^{1}H NMR (400 MHz, $CDCl_3$) δ 7.58 (tt, *J* = 7.0, 1.8 Hz, 1 H, Ph-CH), 7.46–7.34 (m, 4 H, 4 × Ph-CH), 6.82–6.71 (m, 4 H, 4 × Ar-CH), 5.49 (d, *J* = 7.2 Hz, 2 H, C*H*HOC*H*H), 5.02 (d, *J* = 7.0 Hz, 2 H, CH*H*OCH*H*), 3.80 (s, 3 H, OCH_3). The observed characterization data (R_f, mp, ^{1}H) were consistent with that previously reported.[13]

3-(4-Methoxyphenyl)-3-((trifluoromethyl)sulfonyl)oxetane (95)

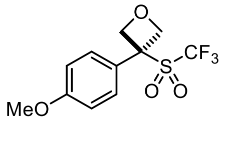

Oxetane sulfonyl fluoride **1** (49.2 mg, 0.2 mmol, 1.0 equiv) and trimethyl(trifluoromethyl)silane (88.0 µL, 0.6 mmol, 3.0 equiv) were added to a flame-dried microwave vial under Ar. The reaction vessel was sealed and anhydrous MeCN (0.67 mL, 0.3 M) was added. The reaction mixture was stirred at 60 ºC for 3 h. The reaction mixture was allowed to cool to 25 °C, EtOAc (5 mL) was added and the mixture was filtered through a plug of Celite, eluting with further EtOAc (3 × 10 mL). Purification by flash column chromatography (20% Et_2O/pentane) oxetane sulfone **95** as an sticky, amorphous white

solid (39.7 mg, 67%). R_f = 0.21 (20% Et_2O/pentane); IR (film)/cm^{-1} 2957, 2843, 1610, 1514, 1357 (SO_2 st as), 1256, 1207 (C–F st), 1123 (SO_2 st sy), 1022, 836, 623; ^{1}H NMR (400 MHz, CD_3CN) δ 7.34–7.26 (m, 2 H, 2 × Ar-CH), 7.08–6.99 (m, 2 H, 2 × Ar-CH), 5.41 (d, *J* = 8.0 Hz, 2 H, C*H*HOC*H*H), 5.22 (d, *J* = 8.1 Hz, 2 H, CH*H*OCH*H*), 3.83 (s, 3 H, OCH_3); ^{13}C NMR (101 MHz, CD_3CN) δ 161.9 (Ar-C_q-OCH_3), 131.3 (2 × Ar-CH), 123.4 (Ar-C_q-C_q), 121.1 (q, $^1J_{C-F}$ = 329.8 Hz, CF_3), 115.3 (2 × Ar-CH), 77.0 (CH_2OCH_2), 70.4 (C_q), 56.2 (OCH_3); ^{19}F{^{1}H} NMR (377 MHz, CD_3CN) δ –70.7.

Notes:

***95** did not ionize very well and a tractable mass ion was not found.*

3-(Butylsulfonyl)-3-(4-methoxyphenyl)oxetane (S29)

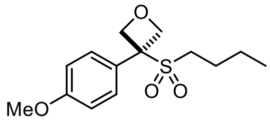

n-BuLi (1.52 M in hexanes, 79 µL, 0.12 mmol, 1.2 equiv) was added dropwise to a solution of sulfonyl fluoride **1** (24.6 mg, 0.1 mmol, 1.0 equiv) in anhydrous THF (1.0 mL, 0.1 M) at –78 °C. After stirring for 15 min at –78 °C, the reaction was quenched with NH_4Cl (5 mL). The layers were separated and the aqueous portion extracted with EtOAc (3 × 5 mL). The organic fractions were dried over Na_2SO_4, filtered and concentrated *in vacuo* using a rotary evaporator. Purification by flash column chromatography (50% Et_2O/pentane) afforded oxetane sulfone **S29** as a colorless gum (6.9 mg, 24%). R_f = 0.15 (50% Et_2O/pentane); IR (film)/cm^{-1} 2956, 2877, 1609, 1512, 1460, 1297 (SO_2 st as), 1250, 1181, 1135 (SO_2 st sy), 1096, 998, 835; ^{1}H NMR (400 MHz, $CDCl_3$) δ 7.20 (d, *J* = 8.3 Hz, 2 H, 2 × Ar-CH), 6.96 (d, *J* = 8.3 Hz, 2 H, 2 × Ar-CH), 5.41 (d, *J* = 7.0 Hz, 2 H, C*H*HOC*H*H), 5.10 (d, *J* = 7.0 Hz, 2 H, CH*H*OCH*H*), 3.84 (s, 3 H, OCH_3), 2.72–2.64 (m, 2 H, SO_2CH_2), 1.70 (p, *J* = 7.8 Hz, 2 H, $SO_2CH_2CH_2$), 1.36 (h, *J* = 7.5 Hz, 2 H, CH_2CH_3), 0.88 (t, *J* = 7.3 Hz, 3 H, CH_2CH_3); ^{13}C NMR (101 MHz, $CDCl_3$) δ 160.2 (Ar-C_q-OCH_3), 129.3 (2 × Ar-CH), 126.2 (Ar-C_q-C_q), 114.4 (2 × Ar-CH), 76.5 (CH_2OCH_2), 67.9 (C_q), 55.4 (OCH_3), 47.5 (SO_2CH_2), 23.0 ($SO_2CH_2CH_2$), 21.8 (CH_2CH_3), 13.5 (CH_2CH_3); HRMS (TOF-MS-ES$^+$) *m/z* calcd for $C_{14}H_{24}NO_4S^+$ $[M+NH_4]^+$: 302.1426, found: 302.1412.

4-((3-(4-Methoxyphenyl)oxetan-3-yl)sulfonyl)morpholine (96)

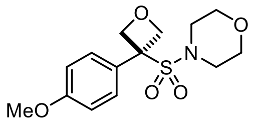

n-BuLi (1.55 M in hexanes, 0.26 mL, 0.4 mmol, 2.0 equiv) was added dropwise to a solution of morpholine (35.0 µL, 0.4 mmol, 2.0 equiv) in anhydrous THF (1.0 mL) at –78 °C. After stirring for 15 min, a solution of oxetane sulfonyl fluoride **1** (49.2 mg, 0.2 mmol, 1.0 equiv) in anhydrous THF (1.0 mL) was added dropwise to the reaction mixture. Following the addition of oxetane sulfonyl fluoride, the reaction vial was transferred from an acetone/dry-ice bath at –78 °C to a water/ice bath at 0 °C and stirred for 1 h. The reaction was then quenched with MeOH (2.0 mL) and concentrated *in vacuo* using a rotary evaporator. Purification by flash column chromatography (80% Et_2O/pentane) afforded oxetane sulfonamide **96** as a white solid (49.9 mg, 80%). R_f = 0.21 (80% Et_2O/pentane); mp = 149 °C; IR (film)/cm^{-1} 2886, 2857, 1610, 1514, 1455, 1322 (SO_2 st as), 1252, 1149 (SO_2 st sy), 1112, 954; ^{1}H NMR (400 MHz, $CDCl_3$) δ 7.23–7.15 (m, 2 H, 2 × Ar-CH), 6.99–6.89 (m, 2 H, 2 × Ar-CH), 5.34 (d, *J* = 6.8 Hz, 2 H, C*H*HOC*H*H), 5.08 (d, *J* = 6.9 Hz, 2 H, CH*H*OCH*H*), 3.83 (s, 3 H, OCH_3), 3.54 (t, *J* = 4.7 Hz, 4 H, $CH_2CH_2OCH_2CH_2$), 2.96 (d, *J* = 4.6 Hz, 4 H, CH_2NCH_2). ^{13}C NMR (101 MHz, $CDCl_3$) δ 160.1 (Ar-C_q-OCH_3), 129.4 (2 × Ar-CH), 126.9 (Ar-C_q-Cq), 114.1 (2 × Ar-CH), 78.0 (CH_2OCH_2) 68.5 (C_q), 67.0 ($CH_2CH_2OCH_2CH_2$), 55.4 (OCH_3), 47.0 (CH_2NCH_2). HRMS (FTMS–APCI$^+$) *m/z* calcd for $C_{14}H_{19}NO_5S^{35}Cl^-$ $[M+Cl]^-$: 348.0678, found: 348.0670.

Notes:

***96** was further characterized by X-ray crystallography (see Fig. **S22**). Crystals suitable for X-ray analysis were grown by slow evaporation from acetone.*

N-Benzyl-3-(4-methoxyphenyl)oxetane-3-sulfonamide (97)

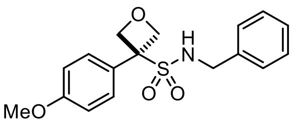

n-BuLi (1.55 M in hexanes, 0.13 mL, 0.2 mmol, 2.0 equiv) was added dropwise to a solution of benzylamine (22.0 µL, 0.2 mmol, 2.0 equiv) in anhydrous THF (0.5 mL) at –78 °C. After stirring for 30 min, a solution of oxetane sulfonyl fluoride **1** (24.6 mg, 0.1 mmol, 1.0 equiv) in anhydrous THF (0.5 mL) at 0 °C was added dropwise to the reaction mixture. Following the addition of oxetane sulfonyl fluoride, the reaction vial was transferred from an acetone/dry-ice bath at –78 °C to a water/ice bath at 0 °C and stirred for 1 h. The reaction was then quenched with sat. aq. NH_4Cl (5 mL). The aqueous layer was extracted with EtOAc (3 × 10 mL) and the organic layers dried over Na_2SO_4, filtered and concentrated *in vacuo* using a rotary evaporator. Purification by flash column chromatography (80% Et_2O/pentane) afforded oxetane sulfonamide **97** as a white solid (23.7 mg, 71%). R_f = 0.28 (80% Et_2O/*n*-hexane); mp = 130–133 °C; IR (film)/cm^{-1} 3158, 2954, 2882, 1610, 1514, 1317 (SO_2 st as), 1253, 1144 (SO_2 st sy), 1091, 992, 826, 620, 555; ^{1}H NMR (400 MHz, $CDCl_3$) δ 7.36–7.27 (m, 3 H, 3 × Ph-CH), 7.25–7.14 (m, 4 H, 2 × Ar-CH + 2 × Ph-CH), 6.96–6.90 (m, 2 H, 2 × Ar-CH), 5.31 (d, *J* = 7.0 Hz, 2 H, C*H*HOC*H*H), 5.07 (d, *J* = 7.0 Hz, 2 H, CH*H*OCH*H*), 4.46 (t, *J* = 5.9 Hz, 1 H, NH), 3.93 (d, *J* = 5.8 Hz, 2 H, NCH_2), 3.83 (s, 3 H, OCH_3); ^{13}C NMR (101 MHz, $CDCl_3$) δ 160.0 (Ar-C_q-OCH_3), 137.0 (Ar-C_q-CH_2), 129.6 (2 × Ar-CH), 128.8 (2 × Ph-CH), 128.1 (Ph-CH), 127.9 (2 × Ph-CH), 127.0 (Ar-C_q-C_q), 114.1 (2 × Ar-CH), 77.7 (CH_2OCH_2), 68.2 (C_q), 55.4 (OCH_3), 48.6 (NCH_2); HRMS (TOF-MS-ES$^+$) *m/z* calcd for $C_{17}H_{23}N_2O_4S^+$ [M+NH$_4$]$^+$: 351.1379, found: 351.1373.

3-(4-Methoxyphenyl)-N-phenyloxetane-3-sulfonamide (98)

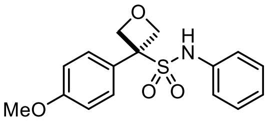

n-BuLi (1.55 M in hexanes, 0.13 mL, 0.2 mmol, 2.0 equiv) was added dropwise to a solution of aniline (18.3 µL, 0.2 mmol, 2.0 equiv) in anhydrous THF (0.5 mL) at –78 °C. After stirring for 1 h, a solution of oxetane sulfonyl fluoride **1** (24.6 mg, 0.1 mmol, 1.0 equiv) in anhydrous THF (0.5 mL) at 0 °C was added dropwise to the reaction mixture. Following the addition of the oxetane sulfonyl fluoride, the reaction vial was transferred from an acetone/dry-ice bath at –78 °C to a water/ice bath at 0 °C and stirred for 1 h. The reaction was then quenched with sat. aq. NH_4Cl (5 mL). The aqueous layer was extracted with EtOAc (3 × 10 mL) and the organic layers dried over Na_2SO_4, filtered and concentrated *in vacuo* using a rotary evaporator. Purification by flash column chromatography (30% EtOAc/pentane) afforded oxetane sulfonamide **98** as a white solid (14.2 mg, 45%). R_f = 0.21 (30% EtOAc/*n*-hexane); mp = 120–123 °C; IR (film)/cm^{-1} 3253 (NH st), 2958, 2889, 1610, 1513, 1491, 1337 (SO_2 st as), 1303, 1252, 1149 (SO_2 st sy), 1027, 929; ^{1}H NMR (400 MHz, $CDCl_3$) δ 7.30–7.21 (m, 2 H, 2 × Ph-CH), 7.17–7.05 (m, 5 H, 3 × Ph-CH + 2 × Ar-CH), 6.94– 6.85 (m, 2 H, 2 × Ar-CH), 6.36 (s, 1 H, NH), 5.30 (dd, *J* = 7.3, 1.3 Hz, 2 H, C*H*HOC*H*H), 5.02 (dd, *J* = 7.2, 1.3 Hz, 2 H, CH*H*OCH*H*), 3.82 (s, 3 H, OCH_3); ^{13}C NMR (101 MHz, $CDCl_3$) δ 160.2 (Ar-C_q-OCH_3), 137.1 (Ar-C_q-C_q), 129.8 (2 × Ph-CH), 129.4 (2 × Ar-CH), 126.5 (Ph-C_q-N), 125.2 (Ph-CH), 120.6 (2 × Ph-CH), 114.2 (2 × Ar-CH), 77.8 (CH_2OCH_2), 69.5 (C_q), 55.5 (OCH_3); HRMS (FTMS–ES$^+$) *m/z* calcd for $C_{16}H_{16}NO_4S^+$ [M+H]$^+$: 318.0806, found: 318.0803.

N-Phenyl-3-(4-((triisopropylsilyl)oxy)phenyl)oxetane-3-sulfonamide (99)

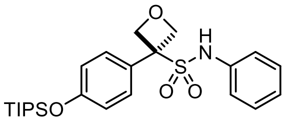

n-BuLi (1.55 M in hexanes, 0.13 mL, 0.2 mmol, 2.0 equiv) was added dropwise to a solution of aniline (36.6 µL, 0.4 mmol, 2.0 equiv) in anhydrous THF (1.0 mL) at –78 °C. After stirring for 1 h, a solution of oxetane sulfonyl fluoride **2** (77.7 mg, 0.2 mmol, 1.0 equiv) in anhydrous THF (1.0 mL) at 0 °C was added dropwise to the reaction mixture. Following the addition of the oxetane sulfonyl fluoride, the reaction vial was transferred from an acetone/dry-ice bath at –78 °C to a water/ice bath at 0 °C and stirred for 1 h. The reaction was then quenched with sat. aq. NH_4Cl (5 mL). The aqueous layer was extracted with EtOAc (3 × 10 mL) and the organic layers dried over Na_2SO_4, filtered and concentrated *in vacuo* using a rotary evaporator. Purification by flash column chromatography (20–

70% Et_2O/pentane) afforded oxetane sulfonamide **99** as a pale-yellow gum (48.3 mg, 52%). R_f = 0.13 (30% Et_2O/pentane); IR (film)/cm^{-1} 3235 (NH st), 2947, 2868, 1344 (SO_2 st as), 1510, 1491, 1428, 1286, 1265, 1146 (SO_2 st sy), 1103, 995, 924, 882, 831, 664; ^{1}H NMR (400 MHz, $CDCl_3$) δ 7.26–7.21 (m, 2 H, 2 × Ph-CH), 7.13–7.07 (m, 1 H, Ph-CH), 7.07–7.02 (m, 4 H, 2 × Ph-CH + 2 × Ar-CH), 6.90–6.82 (m, 2 H, 2 × Ar-CH), 6.50 (s, 1 H, NH), 5.32 (d, *J* = 7.1 Hz, 2 H, C*H*HOC*H*H), 5.03 (d, *J* = 7.2 Hz, 2 H, CH*H*OCH*H*), 1.35–1.20 (m, 3 H, 3 × SiCH), 1.11 (d, *J* = 7.3 Hz, 18 H, 6 × SiCHC*H*$_3$); ^{13}C NMR (101 MHz, $CDCl_3$) δ 156.9 (Ar-*C*$_q$-OCH_3), 137.1 (Ar-*C*$_q$-C_q), 129.8 (2 × Ph-CH), 129.3 (2 × Ar-CH), 126.8 (Ph-C_q-N), 125.1 (Ph-CH), 120.6 (2 × Ph-CH), 119.9 (2 × Ar-CH), 77.8 (CH_2OCH_2), 69.5 (C_q), 17.9 (6 × SiCH*C*H$_3$), 12.7 (3 × Si*C*HCH$_3$); HRMS (TOF-MS-ES$^+$) *m/z* calcd for $C_{24}H_{35}NO_4NaSiS^+$ [M+Na]$^+$: 484.1954, found: 484.1965.

1-((3-(4-Methoxyphenyl)oxetan-3-yl)sulfonyl)-1*H*-imidazole (100)

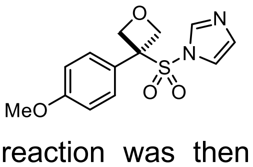

NaH (60% in mineral oil, 8.0 mg, 0.2 mmol, 2.0 equiv) was added to a solution of imidazole (13.6 mg, 0.2 mmol, 2.0 equiv) in anhydrous THF (1.0 mL, 0.1 M) at 0 °C. After stirring for 15 min, oxetane sulfonyl fluoride **1** (24.6 mg, 0.1 mmol, 1.0 equiv) was added and the reaction mixture was stirred at 0 °C for 1 h. The reaction was then quenched with MeOH (1.0 mL) and concentrated *in vacuo* using a rotary evaporator. Purification by flash column chromatography (80% Et_2O/pentane) afforded oxetane sulfonamide **100** as a white solid (25.8 mg, 88%). R_f = 0.15 (80% Et_2O/pentane); mp = 117–118 °C; IR (film)/cm^{-1} 3087, 2929, 1607, 1513, 1460, 1366 (SO_2 st as), 1247, 1184, 1149 (SO_2 st sy), 1020, 988, 846, 822; ^{1}H NMR (400 MHz, CD_3CN) δ 7.38–7.33 (m, 1 H, NCHN), 7.04–7.03 (m, 1 H, $Ar_{(imidazole)}$-CH), 7.01–6.99 (m, 1 H, $Ar_{(imidazole)}$-CH), 6.93–6.83 (m, 4 H, 4 × Ar-CH), 5.37 (d, *J* = 8.0 Hz, 2 H, C*H*HOC*H*H), 5.13 (d, *J* = 8.0 Hz, 2 H, CH*H*OCH*H*), 3.80 (s, 3 H, OCH_3); ^{13}C NMR (101 MHz, CD_3CN) δ 161.7 (Ar-*C*$_q$-OCH_3), 138.7 (NCHN), 131.5 ($Ar_{(imidazole)}$-CH), 130.4 (2 × Ar-CH), 125.2 (Ar-*C*$_q$-C_q), 120.1 ($Ar_{(imidazole)}$-CH), 115.1 (2 × Ar-CH), 77.4 (CH_2OCH_2), 71.7 (C_q), 56.2 (OCH_3). HRMS (FTMS–APCI$^+$) *m/z* calcd for $C_{14}H_{15}N_2O_4^-$ [M–SO_2+HCOO]$^-$: 275.1037, found: 275.1039, HRMS (TOF-MS-ES$^+$) *m/z* calcd for $C_{13}H_{14}N_2O_2^+$ [M+H]$^+$: 231.1055, found: 231.1102.

Notes:

***100** was further characterized by X-ray crystallography (see Fig. **S23**). Crystals suitable for X-ray analysis were grown by slow evaporation from CD_3CN.*

1-((3-(4-Methoxyphenyl)oxetan-3-yl)sulfonyl)-2-methyl-1*H*-imidazole (S30)

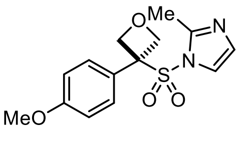

NaH (60% in mineral oil, 16.0 mg, 0.4 mmol, 1.2 equiv) was added to a solution of 2-methyl-1*H*-imidazole (32.8 mg, 0.4 mmol, 2.0 equiv) in anhydrous THF (1 mL, 0.1 M) at 0 °C. After stirring for 15 min, oxetane sulfonyl fluoride **1** (49.2 mg, 0.2 mmol, 1.0 equiv) was added to the reaction mixture. The reaction mixture was allowed to warm to rt over 3 h. The reaction was then quenched with MeOH (2.0 mL) and concentrated *in vacuo* using a rotary evaporator. Purification by flash column chromatography (80% Et_2O/pentane) afforded oxetane sulfonamide **S30** as a white solid (30.1 mg, 48%). R_f = 0.10 (80% Et_2O/pentane); mp = 129–130 °C; IR (film)/cm^{-1} 3114, 2885, 1607, 1512, 1360 (SO_2 st as), 1251, 1175, 1147 (SO_2 st sy), 1029, 973, 844, 561; ^{1}H NMR (400 MHz, $CDCl_3$) δ 7.11 (d, *J* = 1.7 Hz, 1 H, SNCH), 6.91–6.83 (m, 3 H, SNCHC*H* + 2 × Ar-CH), 6.78–6.70 (m, 2 H, 2 × Ar-CH), 5.51 (d, *J* = 7.2 Hz, 2 H, C*H*HOC*H*H), 5.10 (d, *J* = 7.3 Hz, 2 H, CH*H*OCH*H*), 3.81 (s, 3 H, OCH_3), 1.65 (s, 3 H, $Ar_{(imidazole)}$-CH_3); ^{13}C NMR (101 MHz, $CDCl_3$) δ 161.0 (Ar-*C*$_q$-OCH_3), 148.4 (NC_qN), 129.4 (2 × Ar-CH), 128.4 (SNCH), 124.5 (Ar-*C*$_q$-C_q), 119.3 (SNCH*C*H), 114.6 (2 × Ar-CH), 70.5 (C_q), 55.5 (OCH_3), 13.9 ($Ar_{(imidazole)}$-CH_3); HRMS (TOF-MS-ES$^+$) *m/z* calcd for $C_{14}H_{17}N_2O_2^+$ [M–SO_2+H]$^+$: 245.1290, found: 245.1203.

3-(4-Methoxyphenyl)oxetane-3-sulfonyl azide (101)

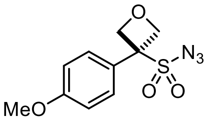

Oxetane sulfonyl fluoride **1** (49 mg, 0.2 mmol, 1.0 equiv) and NaN_3 (39 mg, 0.6 mmol, 3.0 equiv) were added to a flame-dried vial under Ar and sealed. 15-Crown-5 ether (0.12 mL, 0.6 mmol, 3.0 equiv) and anhydrous THF (0.67 mL, 0.3 M) were added sequentially. After stirring at ambient temperature for 24 h, the reaction mixture was quenched with aq. NaOH (1 M, 10 mL) and stirred for 5 min. The reaction mixture was diluted with CH_2Cl_2 (10 mL), the phases separated, and the aqueous layer extracted with CH_2Cl_2 (3 × 10 mL). The combined organic layers were dried over Na_2SO_4, filtered, and concentrated *in vacuo* using a rotary evaporator. Purification by automated flash column chromatography (0–50% EtOAc/*n*-hexane, see conditions below) afforded oxetane sulfonyl azide **101** as a crystalline white solid (49.0 mg, 90%). R_f = 0.53 (50% EtOAc/*n*-hexane); IR (film)/cm^{-1} 2956, 2917, 2891, 2848, 2136 (N°N°N st), 1609, 1514, 1462, 1359 (SO_2 st as), 1304, 1254, 1180, 1154 (SO_2 st as), 1025, 999, 837, 752, 622, 554; ^{1}H NMR (400 MHz, $CDCl_3$) δ 7.26 (d, *J* = 8.5 Hz, 2 H, 2 × Ar-CH), 7.02 (d, *J* = 8.5 Hz, 2 H, 2 × Ar-CH), 5.40 (d, *J* = 7.4 Hz, 2 H, C*H*HOC*H*H), 5.21 (d, *J* = 7.4 Hz, 2 H, CH*H*OCH*H*), 3.87 (s, 3 H, OCH_3); ^{13}C NMR (101 MHz, $CDCl_3$) δ 160.9 (Ar-*C*$_q$-OCH_3), 129.8 (2 × Ar-CH), 124.4 (Ar-*C*$_q$-C_q), 114.6 (2 × Ar-CH), 77.0 (CH_2OCH_2), 71.6 (C_q), 55.5 (OCH_3).

Notes:

101 *did not ionize very well and a tractable mass ion was not found.*

Automated Column Conditions: Run on a Biotage® Selekt system. Column type: Biotage® Sfär® HC 10 g. Flow rate: 40 mL/min. Sample mass: 100 mg. Solvent A: *n*-hexane, Solvent B: EtOAc. UV wavelength detection: l 200–400 nm. See trace and gradient below. From the left: peak 1 (blue): 15-crown-5 ether + oxetane fluoride, peak 2 (red): oxetane sulfonyl azide **101**.

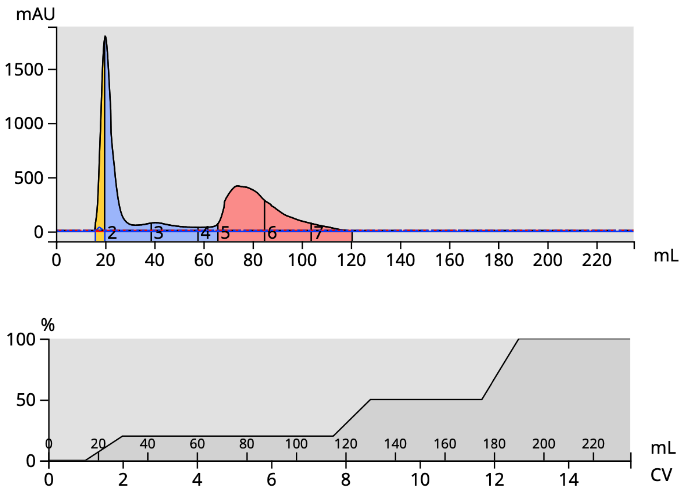

4-((1-Phenylcyclobutyl)sulfonyl)morpholine (102)

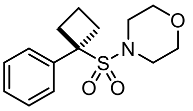

n-BuLi (1.55 M in hexanes, 0.13 mL, 0.2 mmol, 2.0 equiv) was added dropwise to a solution of morpholine (18.0 µL, 0.2 mmol, 2.0 equiv) in anhydrous THF (0.5 mL) at –78 °C. After stirring for 15 min, a solution of cyclobutane sulfonyl fluoride **16** (21.4 mg, 0.1 mmol, 1.0 equiv) in anhydrous THF (0.5 mL) was added dropwise to the reaction mixture. Following the addition of cyclobutane sulfonyl fluoride, the reaction vial was transferred from an acetone/dry-ice bath at –78 °C to a water/ice bath at 0 °C and stirred for 1 h. The reaction was then quenched with MeOH (2.0 mL) and concentrated *in vacuo* using a rotary evaporator. Purification by flash column chromatography (30% Et_2O/pentane) afforded cyclobutane sulfonamide **102** as a white solid (18.4 mg, 65%). R_f = 0.15 (30% Et_2O/pentane); mp = 101–103 °C;

IR (film)/cm^{-1} 2855, 1445, 1307 (SO_2 st as), 1258, 1144 (SO_2 st sy), 1115, 1069, 948, 787, 706, 561; ^{1}H NMR (400 MHz, $CDCl_3$) δ 7.49–7.30 (m, 5 H, 5 × Ph-CH), 3.50 (d, *J* = 4.7 Hz, 4 H, CH_2OCH_2), 3.15–3.03 (m, 2 H, 2 × C_qC*H*H), 2.87 (t, *J* = 4.6 Hz, 4 H, CH_2NCH_2), 2.75–2.63 (m, 2 H, 2 × C_qCH*H*), 2.41–2.25 (m, 1 H, C_qCH_2C*H*H), 2.00–1.85 (m, 1 H, C_qCH_2CH*H*); ^{13}C NMR (101 MHz, $CDCl_3$) δ 138.3 (Ar-*C*$_q$-C_q), 128.5 (2 × Ar-CH), 128.3 (2 × Ar-CH), 128.2 (Ar-CH), 69.2 (C_q), 67.0 (CH_2OCH_2), 47.0 (CH_2NCH_2), 31.4 (*C*H_2CH_2*C*H_2), 16.1 (CH_2*C*H_2CH_2); HRMS (TOF-MS-ES$^+$) *m/z* calcd for $C_{14}H_{20}NO^+$ [M–SO_2+H]$^+$: 218.1545, found: 218.1532.

Phenyl 3-(4-chlorophenyl)oxetane-3-sulfonate (103)

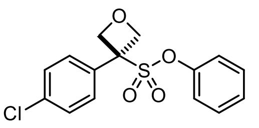

Oven-dried Cs_2CO_3 (26.0 mg, 0.08 mmol, 1.3 equiv), phenol (6.6 mg, 0.07 mmol, 1.2 equiv), and oxetane sulfonyl fluoride **140** (15.0 mg, 0.06 mmol, 1.0 equiv) were added sequentially to a reaction vial and sealed. Anhydrous MeCN (0.2 mL, 0.3 M) was added and the reaction mixture was stirred at 60 °C for 2 h. After cooling to 25 °C, the reaction mixture was diluted with EtOAc (10 mL) and filtered through a plug of Celite, eluting with further EtOAc (3 × 10 mL). The solvent was then removed *in vacuo* using a rotary evaporator. Purification by preparative thin-layer chromatography (20% Et_2O/pentane) afforded oxetane sulfinate ester **103** as a clear, colorless gum (18.8 mg, 96%). R_f = 0.18 (20% Et_2O/pentane); IR (film)/cm^{-1} 2958, 2889, 1587, 1487, 1353 (SO_2 st as), 1199, 1166, 1141 (SO_2 st sy), 1096, 1000, 861, 772, 733, 689, 625, 547; ^{1}H NMR (400 MHz, $CDCl_3$) δ 7.49–7.41 (m, 2 H, 2 × Ar-CH), 7.38–7.31 (m, 2 H, 2 × Ph-CH), 7.31–7.23 (m, 3 H, 3 × Ph-CH), 7.12–7.05 (m, 2 H, 2 × Ar-CH), 5.51 (d, *J* = 7.4 Hz, 2 H, C*H*HOC*H*H), 5.20 (d, *J* = 7.4 Hz, 2 H, CH*H*OCH*H*); ^{13}C NMR (101 MHz, $CDCl_3$) δ 148.9 (Ph-C_q-OSO_2), 135.8 (Ar-*C*$_q$-C_q), 132.4 (Ar-*C*$_q$-Cl), 129.9 (2 × Ph-CH), 129.9 (2 × Ar-CH), 129.1 (2 × Ar-CH), 127.3 (Ph-CH), 121.7 (2 × Ph-CH), 77.2 (CH_2OCH_2), 67.7 (C_q).

Notes:

***103** did not ionize very well and a tractable mass ion was not found.*

4-Nitrophenyl 3-(4-methoxyphenyl)oxetane-3-sulfonate (104)

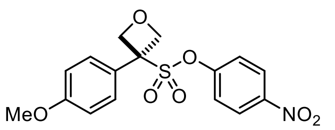

NaH (60% in mineral oil, 16.0 mg, 0.4 mmol, 2.0 equiv) was added to a solution of 4-nitrophenol (55.6 mg, 0.4 mmol, 2.0 equiv) in anhydrous THF (0.68 mL, 0.3 M) at 0 °C. The reaction was allowed to warm to 25 °C over 30 min. Oxetane sulfonyl fluoride **1** (49.2 mg, 0.2 mmol, 1.0 equiv) was added and the reaction stirred at 25 °C for 48 h. EtOAc (5 mL) was then added and the mixture was filtered through a plug of Celite, eluting with further EtOAc (3 × 10 mL). The solvent was then removed *in vacuo* using a rotary evaporator. Purification by flash column chromatography (40–50% Et_2O/pentane) afforded oxetane sulfinate ester **104** as an off-white solid (41.3 mg, 57%). R_f = 0.29 (50% Et_2O/pentane); mp = 112–114 °C; IR (film)/cm^{-1} 2957, 2888, 1610, 1586, 1525 (NO_2 st as), 1484 (N=O st), 1369 (NO_2 st sy), 1348 (NO_2 st sy), 1248 (SO_2 st as), 1177 (C-N st), 1145 (C-N st), 867, 834, 732, 616; ^{1}H NMR (400 MHz, $CDCl_3$) δ 8.26–8.18 (m, 2 H, 2 × Ar-CH), 7.26–7.19 (m, 4 H, 4 × Ar-CH), 7.03–6.95 (m, 2 H, 2 × Ar-CH), 5.49 (d, *J* = 7.4 Hz, 2 H, C*H*HOC*H*H), 5.26 (d, *J* = 7.5 Hz, 2 H, CH*H*OCH*H*), 3.86 (s, 3 H, OCH_3); ^{13}C NMR (101 MHz, $CDCl_3$) δ 160.6 (Ar-*C*$_q$-OCH_3), 153.6 (Ar-C_q-OSO_2), 146.1 (Ar-C_q-NO_2), 129.8 (2 × Ar-CH), 125.5 (2 × Ar-CH), 124.6 (Ar-*C*$_q$-C_q), 122.5 (2 × Ar-CH), 114.4 (2 × Ar-CH), 77.2 (CH_2OCH_2), 68.8 (C_q), 55.5 (OCH_3); HRMS (FTMS–APCI$^+$) *m/z* calcd for $C_{10}H_{11}O_2^+$ [M–$C_6H_4NO_5S$]$^+$: 163.0754, found: 163.0753.

2,2,2-Trifluoroethyl 3-(4-methoxyphenyl)oxetane-3-sulfonate (105)

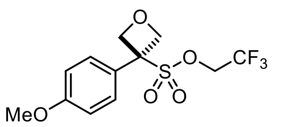

NaH (60% in mineral oil, 8.0 mg, 0.2 mmol, 2.0 equiv) was added to a solution of 2,2,2-trifluoroethan-1-ol (14.5 µL, 0.2 mmol, 2.0 equiv) in anhydrous THF (0.34 mL, 0.3 M) at 0 °C. The reaction was allowed to warm to 25 °C over 30 min. Oxetane sulfonyl fluoride **1** (24.6 mg, 0.1 mmol, 1.0 equiv) was added and

the reaction stirred at 25 °C for 19 h. EtOAc (5 mL) was then added and the mixture was filtered through a plug of Celite, eluting with further EtOAc (3 × 10 mL). The solvent was then removed *in vacuo* using a rotary evaporator. Purification by flash column chromatography (20% Et_2O/pentane) afforded oxetane sulfinate ester **105** as a white solid (24.7 mg, 76%). R_f = 0.13 (20% Et_2O/pentane); mp = 52–55 °C; IR (film)/cm^{-1} 2963, 1612, 1515, 1364 (SO_2 st as), 1282, 1254, 1165 (C-F st), 1025 (SO_2 st sy), 838, 559; ^{1}H NMR (400 MHz, $CDCl_3$) δ 7.22–7.16 (m, 2 H, 2 × Ar–CH), 7.00–6.95 (m, 2 H, 2 × Ar–CH), 5.41 (d, *J* = 7.2 Hz, 2 H, C*H*HOC*H*H), 5.17 (d, *J* = 7.2 Hz, 2 H, CH*H*OCH*H*), 4.08 (q, *J* = 7.8 Hz, 2 H, CH_2CF_3), 3.85 (s, 3 H, OCH_3); ^{13}C NMR (101 MHz, $CDCl_3$) δ 160.5 (Ar-C_q-OCH_3), 129.6 (2 × Ar–CH), 124.7 (Ar-C_q-C_q), 121.7 (q, $^1J_{C-F}$ = 277.8 Hz, CF_3), 114.4 (2 × Ar–CH), 77.3 (CH_2OCH_2), 68.0 (C_q), 65.4 (q, $^2J_{C-F}$ = 38.2 Hz, CF_3), 55.4 (OCH_3); ^{19}F{^{1}H} NMR (377 MHz, $CDCl_3$) δ –74.2; HRMS (TOF-MS-ES$^+$) *m/z* calcd for $C_{12}H_{12}F_3O_5S^-$ [M-H]$^-$: 325.0363, found: 325.1844.

4-Cyanophenyl 3-(4-methoxyphenyl)oxetane-3-sulfonate (S31)

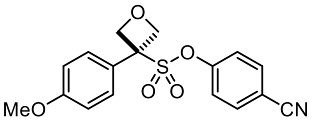

NaH (60% in mineral oil, 16.0 mg, 0.4 mmol, 2.0 equiv) was added to a solution of 4-cyanophenol (48.0 mg, 0.4 mmol, 2.0 equiv) in anhydrous THF (0.68 mL, 0.3 M) at 0 °C. The reaction was allowed to warm to 25 °C over 30 min. Oxetane sulfonyl fluoride **1** (49.2 mg, 0.2 mmol, 1.0 equiv) was added and the reaction stirred at 25 °C for 6 h. A 50% Et_2O/pentane solvent system (5 mL) was then added and the mixture filtered through a plug of silica, eluting with further 50% Et_2O/pentane (3 × 10 mL). Concentration of the filtrate *in vacuo* using a rotary evaporator afforded oxetane sulfinate ester **S31** as an off-white solid (30.3 mg, 44%). %). R_f = 0.21 (50% Et_2O/pentane); mp = 108–110 °C; IR (film)/cm^{-1} 2957, 2925, 2230 (C≡N st), 1607, 1514, 1496, 1355, 1253 (SO_2 st as), 1149, 1024 (SO_2 st sy), 858, 781, 554; ^{1}H NMR (400 MHz, $CDCl_3$) δ 7.65 (d, *J* = 8.8 Hz, 2 H, 2 × Ar-CH), 7.23 (d, *J* = 8.8 Hz, 2 H, 2 × Ar-CH), 7.17 (d, *J* = 8.8 Hz, 2 H, 2 × Ar-CH), 6.98 (d, *J* = 8.9 Hz, 2 H, 2 × Ar-CH), 5.48 (d, *J* = 7.4 Hz, 2 H, C*H*HOC*H*H), 5.25 (d, *J* = 7.4 Hz, 2 H, CH*H*OCH*H*), 3.85 (s, 3 H, OCH_3); ^{13}C NMR (101 MHz, $CDCl_3$) δ 160.6 (Ar-C_q-OCH_3), 152.2 (Ar-C_q-OSO_2), 134.0 (2 × Ar-CH), 129.8 (2 × Ar-CH), 124.7 (Ar-C_q-C_q), 122.8 (2 × Ar-CH), 117.7 (C≡N), 114.4 (2 × Ar-CH), 111.1 (C_q-CN), 77.2 (CH_2OCH_2), 68.7 (C_q), 55.5 (OCH_3); HRMS (FTMS-MS-ES$^+$) *m/z* calcd for $C_{17}H_{14}NO_5S^-$ [M-H]$^-$: 344.0598, found: 344.1776.

Phenyl 3-(4-chlorophenyl)oxetane-3-sulfonate (S32)

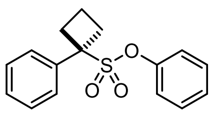

Oven-dried Cs_2CO_3 (49.6 mg, 0.152 mmol, 1.3 equiv), phenol (13.3 mg, 0.141 mmol, 1.2 equiv), and cyclobutane sulfonyl fluoride **16** (25.1 mg, 0.117 mmol, 1.0 equiv) were added sequentially to a reaction vial and sealed. Anhydrous MeCN (0.39 mL, 0.3 M) was added and the reaction mixture was stirred at 60 °C for 2 h. After cooling to 25 °C, the reaction mixture was diluted with EtOAc (10 mL) and filtered through a plug of Celite, eluting with further EtOAc (3 × 10 mL). The solvent was then removed *in vacuo* using a rotary evaporator. Purification by flash column chromatography (10% Et_2O/pentane) afforded cyclobutane sulfinate ester **S32** as a white solid (29.4 mg, 87%). R_f = 0.30 (10% Et_2O/pentane); mp = 100–102 °C; IR (film)/cm^{-1} 3060, 3030, 3002, 2953, 1586, 1485, 1341 (SO_2 st as), 1189, 1144 (SO_2 st sy), 853, 769, 723, 698, 547; ^{1}H NMR (400 MHz, $CDCl_3$) δ 7.59–7.52 (m, 2 H, 2 × Ph-CH), 7.52–7.37 (m, 3 H, 3 × Ph-CH), 7.30 (dd, *J* = 8.6, 6.9 Hz, 2 H, 2 × Ph-CH), 7.27–7.18 (m, 1 H, Ph-CH), 6.97–6.91 (m, 2 H, 2 × Ph-CH), 3.40–3.28 (m, 2 H, CH_2), 3.00–2.85 (m, 2 H, CH_2), 2.50 (dtt, *J* = 11.5, 9.8, 7.2 Hz, 1 H, C*H*H), 2.07 (dtt, *J* = 11.5, 9.8, 5.7 Hz, 1 H, CH*H*); ^{13}C NMR (101 MHz, $CDCl_3$) δ 149.0 (Ph-C_q-OSO_2), 137.5 (Ar-C_q-C_q), 129.6 (2 × Ph-CH), 128.8 (2 × Ph-CH), 128.5 (Ph-CH), 128.3 (2 × Ph-CH), 126.7 (Ph-CH), 121.9 (2 × Ph-CH), 68.0 (C_q), 31.1 (2 × C_qCH_2), 16.0 ($CH_2CH_2CH_3$).

Notes:

***S32** did not ionize very well and a tractable mass ion was not found*

Azetidine deFS products (106–129, 149, 151, 153)

Benzyl 3-(4-methoxyphenyl)-3-morpholinoazetidine-1-carboxylate (106)

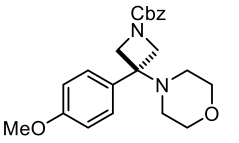

Prepared according to **deFS General Procedure A** under Ar at 60 °C for 2 h with a Celite work-up and using azetidine sulfonyl fluoride **11** (75.8 mg, 0.2 mmol, 1.0 equiv), K_2CO_3 (35.8 mg, 0.26 mmol, 1.3 equiv), morpholine (21 mL, 0.24 mmol, 1.2 equiv) and anhydrous MeCN (0.67 mL, 0.3 M). Purification by flash column chromatography (70% EtOAc/pentane) afforded amino-azetidine **106** as a white solid (65.7 mg, 84%). R_f = 0.27 (70% EtOAc/pentane); mp = 127–129 °C; IR (film)/cm^{-1} 2953, 2853, 1709 (C=O st), 1603, 1513, 1451, 1418, 1356, 1296, 1249, 1179, 1117, 1025, 949, 833, 759, 699, 561; ^{1}H NMR (400 MHz, $CDCl_3$) δ 7.38–7.32 (m, 5 H, 5 × $Ar_{(Cbz)}$-CH), 7.06–7.02 (m, 2 H, 2 × Ar-CH), 6.94–6.91 (m, 2 H, 2 × Ar-CH), 5.12 (s, 2 H, CH_2Ph), 4.27 (d, *J* = 8.3 Hz, 2 H, C*H*HNC*H*H), 4.24 (d, *J* = 8.3, 2 H, CH*H*NCH*H*), 3.85 (3 H, s, OCH_3), 3.71 (t, *J* = 4.0 Hz, 4 H, CH_2OCH_2), 2.29 (br s, 4 H, $CH_2N_{(morpholine)}CH_2$); ^{13}C NMR (101 MHz, $CDCl_3$) δ 158.9 (Ar-C_qOMe), 156.5 (C=O), 136.5 (Ar-C_qCH$_2$), 128.7 (2 × Ar-CH), 128.6 (Ar-C_qC$_q$), 128.5 (Ar-CH), 128.1 (2 × Ar-CH), 128.0 (2 × Ar-CH), 113.3 (2 × Ar-CH), 67.0 (CH_2OCH_2), 66.8 (CH_2Ph), 62.4 (C_q), 58.5 (br, $CH_2N_{(Cbz)}CH_2$), 57.6 (br, $CH_2N_{(Cbz)}CH_2$), 55.4 (OCH_3), 46.4 ($CH_2N_{(morpholine)}CH_2$); HRMS (TOF-MS-ES$^+$) *m/z* calcd for $C_{22}H_{26}N_2O_4$ [M+H]: 383.1971, found: 383.1967.

4-(3-(4-Methoxyphenyl)azetidin-3-yl)morpholine (107)

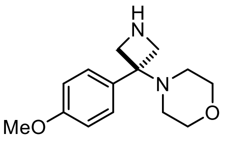

$SiMe_3I$ (0.21 mL, 1.5 mmol, 8.0 equiv) was added dropwise to a solution of amino-azetidine **106** (76.5 mg, 0.2 mmol, 1.0 equiv) in MeCN (1.2 mL, 0.17 M) at –10 °C. After stirring for 4 h at –10 °C, MeOH (81 µL, 2.0 mmol, 10 equiv) was added dropwise. After stirring for a further 30 min at –10 °C, the reaction mixture was concentrated *in vacuo* using a rotary evaporator. The residue was re-dissolved in CH_2Cl_2 (10 mL) and washed with sat. aq. $NaHCO_3$ (10 mL) followed by sat. aq. $Na_2S_2O_3$ (10 mL). The organic layer was dried over Na_2SO_4, filtered, and concentrated *in vacuo* using a rotary evaporator. Purification by automated flash column chromatography (0–10% MeOH/CH_2Cl_2) afforded amino-azetidine **107** as a white gum (38.9 mg, 78%). R_f = 0.11 (10% MeOH/CH_2Cl_2); IR (film)/cm^{-1} 3355 (br NH st), 295, 2830, 1655, 1608, 1512, 1453, 1327, 1296, 1244, 1177, 1107, 1022, 982, 834, 609; ^{1}H NMR (400 MHz, CD_3OD) δ 7.15 (d, *J* = 8.7 Hz, 2 H, 2 × Ar-CH), 7.01 (d, *J* = 8.7 Hz, 2 H, 2 × Ar-CH), 4.34 (br s, 4 H, C*H*$_2$NHC*H*$_2$), 3.84 (s, 3 H, OCH_3), 3.73 (t, *J* = 4.7 Hz, 4 H, 2 × OCH_2), 2.34 (s, 4 H, 2 × NCH_2); ^{13}C NMR (101 MHz, CD_3OD) δ 159.5 (Ar-C_q-OCH_3), 128.2 (2 × Ar-CH), 126.1 (Ar-C_q-C_q), 113.2 (2 × Ar-CH), 66.8 (2 × OCH_2), 64.4 (C_q), 54.7 ($NHCH_2$), 54.7 ($NHCH_2$), 54.5 (OCH_3), 46.1 (2 × NCH_2); HRMS (TOF-MS-ES$^+$) *m/z* calcd for $C_{14}H_{21}N_2O_2$ [M+H]: 249.1603, found: 249.1593.

Benzyl 3-(benzylamino)-3-(4-methoxyphenyl)azetidine-1-carboxylate (108)

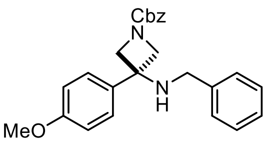

Prepared according to **deFS General Procedure A** under Ar at 60 °C for 2 h with a Celite work-up and using azetidine sulfonyl fluoride **11** (75.8 mg, 0.2 mmol, 1.0 equiv), K_2CO_3 (35.8 mg, 0.26 mmol, 1.3 equiv), benzylamine (26 mL, 0.24 mmol, 1.2 equiv) and anhydrous MeCN (0.67 mL, 0.3 M). Purification by flash column chromatography (70% EtOAc/pentane) afforded amino-azetidine **108** as a white gum (70.2 mg, 87%). R_f = 0.31 (70% EtOAc/pentane); IR (film)/cm^{-1} 3059 (NH st), 3028, 2951, 2878, 2833, 1700 (C=O st), 1607, 1580, 1512, 1449, 1412, 1351, 1289, 1176, 1105, 1027, 829, 731, 696, 611, 553, 458; ^{1}H NMR (400 MHz, $CDCl_3$) δ 7.41-7.27 (m, 12 H, 5 × $Ar_{(Cbz)}$-CH, 5 × Ar- CH, 2 × Ar-CH), 6.98 (m, 2 H, 2 × Ar-CH), 5.16 (s, 2 H, $COCH_2$), 4.38 (d, *J* = 8.8 Hz, 2 H, C*H*HNC*H*H), 4.17 (d, *J* = 8.8 Hz, 2 H, CH*H*NCH*H*), 3.87 (s, 3 H, OCH_3), 3.54 (s, 2 H, CH_2NH), 1.90 (br s, 1 H, NH); ^{13}C NMR (101 MHz, $CDCl_3$) δ 158.8 (Ar-C_qOMe), 156.6 (C=O), 139.7 (Ar-C_qCH$_2$NH), 136.5 (Ar-C_qCH2), 134.2 (Ar-C_qC$_q$), 128.4 (2 × $Ar_{(Cbz)}$-CH), 128.1 (2 × $Ar_{(Cbz)}$-CH), 128.0 (Ar(Cbz)-CH), 127.9 (2 × Ar-CH), 127.4 (2 × Ar-CH), 127.1 (Ar-CH), 113.9 (2 × $Ar_{(PMP)}$-CH), 66.7 (C_q), 61.4 (br, CH_2N), 60.5 (br, CH_2N),

58.5 (OCH_2Ph), 55.2 (OCH_3), 47.8 (NCH_2Ph); HRMS (TOF-MS-ES$^+$) *m/z* calcd for $C_{25}H_{27}N_2O_3$ [M+H]: 403.2022, found: 403.2034.

Benzyl 3-((1-benzhydrylazetidin-3-yl)amino)-3-(4-methoxyphenyl)azetidine-1-carboxylate (109)

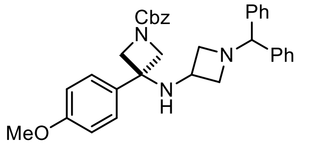

Prepared according to **deFS General Procedure A** under Ar at 60 °C for 2 h with a Celite work-up and using azetidine sulfonyl fluoride **11** (75.8 mg, 0.2 mmol, 1.0 equiv), K_2CO_3 (35.8 mg, 0.26 mmol, 1.3 equiv), 1-benzhydrylazetidin-3-amine (57.2 mg, 0.24 mmol, 1.2 equiv) and anhydrous MeCN (0.67 mL, 0.3 M). Purification by flash column chromatography (40% EtOAc/pentane) afforded amino-azetidine **109** as a white gum (56.8 mg, 77%). R_f = 0.25 (40% EtOAc/pentane); IR (film)/cm^{-1} 3059 (NH st), 3027, 2951, 2830, 1705 (C=O st), 1610, 1514, 1451, 1416, 1354, 1304, 1248, 1210, 1179, 1118, 1028, 831, 746, 701, 636, 610; ^{1}H NMR (400 MHz, $CDCl_3$) δ 7.43–7.33 (m, 9 H, 9 × Ar-CH), 7.35–7.32 (m, 4 H, 4 × Ar-CH), 7.22–7.15 (m, 4 H, 4 × Ar-CH), 7.20 (m, 4 H, 4 × Ar-CH), 6.88 (m, 2 H, 2 × Ar-CH), 5.11 (s, 2 H, 2 × CH_2Ph), 4.31 (d, *J* = 8.7 Hz, 2 H, C*H*HNC*H*H), 4.26 (s, 1 H, C*H*Ph_2), 4.03 (d, *J* = 8.7 Hz, 2 H, CH*H*NCH*H*), 3.81 (s, 3 H, OCH_3), 3.39 (m, 3 H, NHC*H* + N(C*H*H)2CH), 2.59 (m, 2 H, N(CH*H*)$_2$CH), 1.96 (br s, 1 H, NH); ^{13}C NMR (101 MHz, $CDCl_3$) δ 158.9 (Ar-C_qOMe), 156.6 (C=O), 142.0 (2 × Ar-C_q), 136.5 (Ar-$C_q$$CH_2$) 134.5 (Ar-$C_q$Cq), 128.52 (2 × Ar-CH), 128.47 (4 × Ar-CH), 128.12 (Ar-CH), 128.08 (2 × Ar-CH), 127.4 (5 × Ar-CH), 127.3 (2 × Ar-CH), 127.2 (Ar-CH), 114.0 (2 × Ar-CH), 78.4 ($CHPh_2$), 66.8 (CH_2Ph), 63.1 (CH_2NCH_2), 61.7 (br, $N_{(Cbz)}CH_2$), 61.1 (br, $N_{(Cbz)}CH_2$), 58.1 (C_q), 55.3 (OCH_3), 44.5 (NHCH); HRMS (TOF-MS-ES$^+$) *m/z* calcd for $C_{34}H_{36}N_3O_3$ [M+H]: 534.2757, found: 534.2757.

Benzyl (*S*)-3-((3-hydroxy-1-methoxy-1-oxopropan-2-yl)amino)-3-(4-methoxyphenyl)azetidine-1-carboxylate (110)

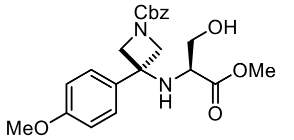

Prepared according to **deFS General Procedure A** under Ar at 60 °C for 2 h with a Celite work-up and using azetidine sulfonyl fluoride **11** (75.8 mg, 0.2 mmol, 1.0 equiv), K_2CO_3 (72.0 mg, 0.52 mmol, 2.6 equiv), *L*-serine methyl ester hydrochloride (37.3 mg, 0.24 mmol, 1.2 equiv) and anhydrous MeCN (0.67 mL, 0.3 M). Purification by flash column chromatography (100% CH_2Cl_2) afforded amino-azetidine **110** as a colorless oil (61.3 mg, 74%). $[\alpha]_D^{23}$ = –87.6 (c 1.0, $CDCl_3$); R_f = 0.23 (100% CH_2Cl_2); IR (film)/cm^{-1} 3400 (OH st), 3300 (NH st), 1700 (C=O st), 1610, 1582, 1513, 1450, 1417, 1354, 1246, 1178, 1115, 1028, 969, 833, 735, 699, 611, 564; ^{1}H NMR (400 MHz, $CDCl_3$) δ 7.37–7.30 (m, 5 H, 5 × Ar-CH), 7.21 (d, *J* = 8.5 Hz, 2 H, 2 × Ar-CH), 6.90 (d, *J* = 8.5 Hz, 2 H, 2 × Ar-CH), 5.10 (s, 2 H, CH_2Ph), 4.41 (d, *J* = 8.9 Hz, 1 H, C*H*HNCH_2), 4.33 (d, *J* = 8.9 Hz, 1 H, CH_2NC*H*H), 4.16 (d, *J* = 8.9 Hz, 1 H, CH*H*NCH_2), 4.14 (d, *J* = 8.9 Hz, 1 H, CH2NCH*H*), 3.82 (s, 3 H, OCH_3), 3.59 (s, 3 H, CO_2CH_3), 3.50 (d, *J* = 5.4 Hz, 2 H, C*H*2OH), 3.30 (t, *J* = 5.4 Hz, 1 H, C*H*CH2), 2.90 (br, OH, NH); ^{13}C NMR (101 MHz, $CDCl_3$) δ 173.6 (C=$O_{(serine)}$), 159.1 (Ar- C_qOMe), 156.6 (C=$O_{(Cbz)}$), 136.5 (Ar-$C_q$$CH_2$), 133.6 (Ar-$C_q$$C_q$), 128.5 (2 × Ar-$C_{(Cbz)}$H), 128.1 (Ar-CH), 128.0 (2 × Ar-CH), 127.8 (2 × Ar-CH), 114.1 (2 × Ar-CH), 66.9 (CH_2Ph), 63.3 (C_q), 61.6 (br, N*C*H2), 61.0 (br, N*C*H2), 58.3 (CHHC), 58.1 (CH_2OH), 55.3 (OCH_3), 52.4 (CO_2*C*H_3); HRMS (TOF-MS-ES$^+$) *m/z* calcd for $C_{22}H_{27}N_2O_6$ [M+H]: 415.1869, found: 415.1866.

Benzyl 3-(diethylamino)-3-(4-methoxyphenyl)azetidine-1-carboxylate (111)

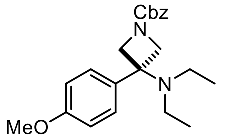

Prepared according to **deFS General Procedure A** under Ar at 60 °C for 2 h with a Celite work-up and using azetidine sulfonyl fluoride **11** (75.8 mg, 0.2 mmol, 1.0 equiv), K_2CO_3 (72.0 mg, 0.52 mmol, 2.6 equiv), diethylamine hydrochloride (26.3 mg, 0.24 mmol, 1.2 equiv) and anhydrous MeCN (0.67 mL, 0.3 M). Purification by flash column chromatography (70% Et_2O/pentane) afforded amino-azetidine **111** as a white gum (53.0 mg, 72%). R_f = 0.18 (70% Et_2O/pentane); IR (film)/cm^{-1} 2964, 1709 (C=O st), 1608, 1511, 1449, 1416, 1354, 1295, 1247, 1181, 1140, 1097, 1029, 832, 698, 614; ^{1}H NMR (400 MHz, $CDCl_3$) δ 7.38–

7.31 (m, 5 H, 5 × $Ar_{(Cbz)}$-CH), 7.22–7.18 (m, 2 H, 2 × Ar-CH), 6.93–6.89 (m, 2 H, 2 × Ar-CH), 5.11 (s, 2 H, CH_2Ph), 4.29 (d, *J* = 8.4 Hz, 2 H, C*H*HNC*H*H), 4.24 (d, *J* = 8.4, 2 H, CH*H*NCH*H*), 3.84 (s, 3 H, OCH_3), 2.39 (q, *J* = 7.2 Hz, 4 H, 2 × NC*H*2CH3), 1.06 (t, *J* = 7.2 Hz, 6 H, 2 × NCH_2CH_3); ^{13}C NMR (101 MHz, $CDCl_3$) δ 158.6 (Ar-C_qOMe), 156.4 (C=O), 136.7 (Ar-$C_q$$CH_2$), 132.3 (Ar-$C_qC_q$), 128.5 (Ar-CH), 128.3 (2 × Ar-$C_{(Cbz)}$H), 128.0 (2 × $Ar_{(Cbz)}$-CH + 2 × $Ar_{(PMP)}$-CH), 113.3 (2 × Ar-CH), 66.7 (CH_2Ph), 63.1 (C_q), 60.4 (br, N*C*H2), 59.5 (br, N*C*H2), 55.3 (OCH_3), 43.9 (N$CH_2$$CH_3$), 15.7 ($NCH_2CH_3$); HRMS (TOF-MS-ES$^+$) *m/z* calcd for $C_{22}H_{29}N_2O_3$ [M+H]: 369.2178, found: 369.2173.

Benzyl-(4,4-difluoropiperidin-1-yl)-3-(4-methoxyphenyl)azetidine-1-carboxylate (112)

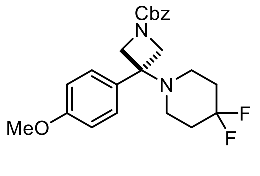

Prepared according to **deFS General Procedure A** under Ar at 60 °C for 2 h with a Celite work-up and using azetidine sulfonyl fluoride **11** (75.8 mg, 0.2 mmol, 1.0 equiv), K_2CO_3 (35.8 mg, 0.26 mmol, 1.3 equiv), 4,4'-difluoropiperidine (29.1 mg, 0.24 mmol, 1.2 equiv) and anhydrous MeCN (0.67 mL, 0.3 M). Purification by flash column chromatography (20–40% EtOAc/pentane) afforded amino-azetidine **112** as a colorless oil (77.0 mg, 92%). R_f = 0.28 (40% EtOAc/pentane); IR (film)/cm^{-1} 3032, 2946, 2835, 1703 (C=O st), 1609, 1512, 1415, 1355, 1179, 1134, 1100, 1047, 990, 957, 834, 736, 698, 541; ^{1}H NMR (400 MHz, $CDCl_3$) δ 7.41–7.29 (m, 5 H, 5 × Ar-CH), 7.04 (d, *J* = 8.8 Hz, 2 H, 2 × Ar-CH), 6.92 (d, *J* = 8.8 Hz, 2 H, 2 × Ar-CH), 5.12 (s, 2 H, $PhCH_2$), 4.31 (d, *J* = 8.3 Hz, 2 H, C*H*H$N_{(Cbz)}$C*H*H), 4.21 (d, *J* = 8.3 Hz, 2 H, CH*H*$N_{(Cbz)}$CH*H*), 3.85 (s, 3 H, OCH_3), 2.34 (br, 4 H, $C_qN(CH_2)_2$), 2.06–1.94 (m, 4 H, $(CH_2)_2CF_2$); ^{13}C NMR (101 MHz, $CDCl_3$) δ 158.9 (Ar-C_qOMe), 156.4 (C=O), 136.5 (Ar-$C_q$$CH_2$), 128.5 (2 × $Ar_{(Cbz)}$-CH), 128.4 (Ar-CH), 128.1 (Ar-C_qC_q), 128.0 (2 × Ar-CH), 121.5 (t, *J* = 242.6 Hz, CF_2), 113.3 (2 × Ar-CH), 66.8 ($PhCH_2$), 62.2 (C_q), 59.3 (br, CH_2N), 58.5 (br, CH_2N), 55.3 (OCH_3), 43.1 (t, *J* = 5.6 Hz, $N(CH_2)_2$), 34.2 (t, *J* = 23.1 Hz, $(CH_2)_2CF_2$); ^{19}F{^{1}H} NMR (377 MHz, $CDCl_3$) δ –97.69; HRMS (TOF-MS-ES$^+$) *m/z* calcd for $C_{23}H_{27}N_2O_3F_2$ [M+H]: 417.1990, found: 417.1995.

Benzyl 3-(4-(2-(4-methoxyphenoxy)ethyl)piperazin-1-yl)-3-(4-methoxyphenyl)azetidine-1-carboxylate (113)

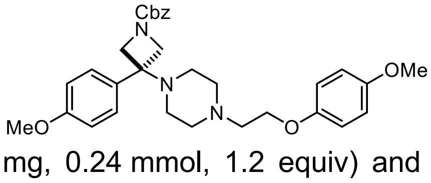

Prepared according to **deFS General Procedure A** under Ar at 60 °C for 2 h with a Celite work-up and using azetidine sulfonyl fluoride **11** (75.8 mg, 0.2 mmol, 1.0 equiv), K_2CO_3 (35.8 mg, 0.26 mmol, 1.3 equiv), 1-(2-(4-methoxyphenoxy)ethyl)piperazine (47.2 mg, 0.24 mmol, 1.2 equiv) and anhydrous MeCN (0.67 mL, 0.3 M). Purification by flash column chromatography (0–20% Et_2O/CH_2Cl_2) afforded amino-azetidine **113** as a white gum (74.1 mg, 70%). R_f = 0.35 (20% Et_2O/CH_2Cl_2); IR (film)/cm^{-1} 2940, 2829, 1706 (C=O st), 1609, 1508, 1455, 1417, 1297, 1230, 1179, 1118, 1034, 981, 820, 737, 699, 580; ^{1}H NMR (400 MHz, $CDCl_3$) δ 7.37-7.31 (m, 5 H, 5 × Ar-CH), 7.02 (d, *J* = 8.4 Hz, 2 H, 2 × Ar-CH), 6.89 (d, *J* = 8.4 Hz, 2 H, 2 × Ar-CH), 6.82 (s, 4 H, 4 × Ar-CH), 5.11 (s, 2 H, CH_2Ph), 4.28 (d, *J* = 8.4 Hz, 2 H, C*H*HNC*H*H), 4.23 (d, *J* = 8.4 Hz, 2 H, CH*H*NCH*H*), 4.01 (t, *J* = 5.7 Hz, 2 H, O$CH_2$$CH_2$), 3.82 (s, 3 H, OCH_3), 3.77 (s, 3 H, OCH_3), 2.77 (t, *J* = 5.7 Hz, 2 H, OCH_2CH_2), 2.64 (br, 4 H, CH_2NCH_2), 2.35 (br, 4 H, CH_2NCH_2); ^{13}C NMR (101 MHz, $CDCl_3$) δ 158.9 (Ar-C_qOMe), 156.4 (C=O), 153.9 (Ar-C_qOMe), 152.8 (Ar-C_qOCH_2), 136.6 (Ar-$C_q$$CH_2$), 128.7 (2 × Ar-CH), 128.53 (Ar-C_qC_q), 128.48 (Ar-CH), 128.0 (4 × Ar-CH), 115.5 (2 × Ar-CH), 114.6 (2 × Ar-CH), 113.2 (2 × Ar-CH), 66.7 ($PhCH_2$), 66.3 (OCH_2CH_2), 62.2 (C_q), 58.9 (br, CH_2N), 58.1 (br, CH_2N), 57.2 (OCH_2CH_2), 55.7 (OCH_3), 55.4 (OCH_3), 53.6 (CH_2NCH_2), 45.6 (CH_2NCH_2); HRMS (TOF-MS-ES$^+$) *m/z* calcd for $C_{31}H_{38}N_3O_5$ [M+H]: 532.2811, found: 532.2800.

3-(4-Methoxyphenyl)-3-((1*S*,5*R*)-8-oxo-1,5,6,8-tetrahydro-2*H*-1,5-methanopyrido[1,2-*a*][1,5]diazocin-3(4*H*)-yl)azetidine-1-carboxylate (114)

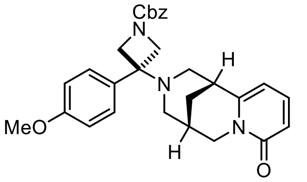

Anhydrous K_2CO_3 (18 mg, 0.13 mmol, 1.3 equiv), cytisine (19.2 mg, 0.1 mmol, 1.0 equiv), and azetidine sulfonyl fluoride **11** (45.5 mg, 0.12 mmol, 1.2 equiv) were added sequentially to a flame-dried reaction tube under argon. The reaction vessel was sealed and anhydrous MeCN (0.33 mL, 0.3 M) was added by syringe. After stirring at 60 °C for 2 h, the reaction mixture was left to cool to 25 °C, EtOAc was added and the mixture was filtered through a plug of Celite, eluting with further EtOAc (3 × 10 mL). The solvent was then removed in vacuo using a rotatory evaporator. Purification by flash column chromatography (100% EtOAc) afforded amino-azetidine **114** as a yellow gum (78.7 mg, 81%). $[\alpha]_D^{23}$ = –12.6 (c 1.0, $CDCl_3$), R_f = 0.15 (100% EtOAc); IR (film)/cm^{-1} 2940, 2809, 1708 (C=O st), 1653 (C=O st), 1609, 1573, 1546, 1512, 1418, 1356, 1296, 1249, 1179, 1152, 1122, 1026, 800, 735, 567; ^{1}H NMR (400 MHz, $CDCl_3$) δ 7.36-7.29 (m, 6 H, 5 × Ar-CH + C*H*CHC=O), 6.98 (d, *J* = 8.1 Hz, 2 H, 2 × Ar-CH), 6.88 (d, *J* = 8.1 Hz, 2 H, 2 × Ar-CH), 6.52 (d, *J* = 8.8 Hz, 1 H, CHC=O), 5.98 (br, 1 H, C*H*CHCHC=O), 5.04 (m, 2 H, CH_2Ph), 4.26–3.90 (m, 5 H, 2 × $N_{(azetidine)}CH_2$ + C*H*HNC=O), 3.82 (s, 3 H, OCH_3), 3.77 (br, 1 H, CH*H*NC=O), 2.93 (br, 1 H, CHC_qN), 2.81 (br, 2 H, NC*H*HCHCH$_2$NC=O + NC*H*HCHC$_q$), 2.44 (br, 1 H, CHCH$_2$C=O), 2.03-2.01 (m, 2 H, CH*H*NCH*H*), 1.83 (d, *J* = 12.5 Hz, 1 H, $C_{(bridge)}$*H*H), 1.59 (d, *J* = 12.5 Hz, 1 H, $C_{(bridge)}$H*H*); ^{13}C NMR (101 MHz, $CDCl_3$) δ 163.6 (C=O), 158.9 (Ar-*C$_q$*OMe), 156.2 (C=O), 151.0 (*C$_q$*NC=O), 138.9 (*C*HCHC=O), 136.5 (Ar-*C$_q$*CH$_2$), 128.5 (2 × Ar-CH), 128.3 (Ar-*C$_q$*C$_q$), 128.1 (Ar-CH), 128.0 (2 × Ar-CH), 116.9 (*C*HC=O), 113.3 (2 × Ar-CH), 104.8 (*C*HCHCHC=O), 66.8 (PhCH$_2$), 62.0 (C_q), 58.7 (br, CH_2N), 57.8 (br, CH_2N), 55.3 (OCH_3), 53.8 (N*C*H$_2$CHC$_q$), 52.9 (N*C*H$_2$CHCH$_2$NC$_q$=O), 49.8 (*C*H$_2$NC=O), 35.1 (*C*HC$_q$N), 27.7 (*C*HCH$_2$NC=O), 25.7 ($C_{(bridge)}H_2$); HRMS (TOF-MS-ES$^+$) *m/z* calcd for $C_{29}H_{31}N_3O_4$ [M+H]: 486.2393, found: 486.2389.

Benzyl 3-(4-methoxyphenyl)-3-((4-methoxyphenyl)(methyl)amino)azetidine-1-carboxylate (115)

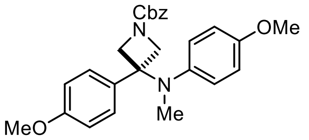

Prepared according to **deFS General Procedure A** under Ar at 60 °C for 2 h with a Celite work-up and using azetidine sulfonyl fluoride **11** (75.8 mg, 0.2 mmol, 1.0 equiv), K_2CO_3 (35.8 mg, 0.26 mmol, 1.3 equiv), 4-methoxy-*N*-aniline (32.9 mg, 0.24 mmol, 1.2 equiv) and anhydrous MeCN (0.67 mL, 0.3 M). Purification by flash column chromatography (30% Et_2O/pentane) afforded amino-azetidine **115** as an off-white gum (66.7 mg, 77%). R_f = 0.21 (30% Et_2O/pentane); IR (film)/cm^{-1} 2951, 2889, 2831, 1705 (C=O st), 1608, 1579, 1508, 1447, 1409, 1349, 1294, 1243, 1213, 1179, 1109, 1081, 1032, 966, 823, 760, 733, 700, 611, 550; ^{1}H NMR (400 MHz, $CDCl_3$) δ 7.45–7.33 (m, 5 H, 5 × Ar-CH), 7.26 (d, *J* = 8.4 Hz, 2 H, 2 × Ar-CH), 6.91 (d, *J* = 8.8 Hz, 2 H, 2 × Ar-CH), 6.80 (d, *J* = 8.8 Hz, 2 H, 2 × Ar-CH), 6.41 (d, *J* = 8.4 Hz, 2 H, 2 × Ar-CH), 5.18 (s, 2 H, CH_2Ph), 4.45 (d, *J* = 8.5 Hz, 2 H, C*H*HNC*H*H), 4.17 (d, *J* = 8.5 Hz, 2 H, CH*H*NCH*H*), 3.83 (s, 3 H, OCH_3), 3.77 (s, 3 H, OCH_3), 3.05 (s, 3 H, NCH_3); ^{13}C NMR (101 MHz, $CDCl_3$) δ 158.9 (Ar-*C$_q$*OMe), 156.6 (C=O), 152.2 (Ar-*C$_q$*OMe), 141.3 (Ar-*C$_q$*NMe), 136.5 (Ar-*C$_q$*CH$_2$), 134.4 (Ar-*C$_q$*C$_q$), 128.6 (2 × Ar-CH), 128.2 (4 × Ar-CH), 127.1 (Ar-CH), 116.2 (2 × Ar-CH), 114.5 (2 × Ar-CH), 114.0 (2 × Ar-CH), 67.0 (PhCH$_2$), 62.5 (br, CH_2N), 61.7 (br, CH_2N), 60.7 (C_q), 55.7 (OCH_3), 55.3 (OCH_3), 36.8 (NCH_3); HRMS (TOF-MS-ES$^+$) *m/z* calcd for $C_{26}H_{29}N_2O_4$ [M+H]: 433.2127, found: 433.2115.

Benzyl 3-(4-methoxyphenyl)-3-((6-methoxypyridin-3-yl)amino)azetidine-1-carboxylate (116)

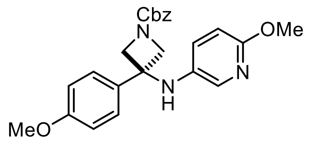

Prepared according to **deFS General Procedure A** under Ar at 60 °C for 2 h with a Celite work-up and using azetidine sulfonyl fluoride **11** (75.8 mg, 0.2 mmol, 1.0 equiv), K_2CO_3 (35.8 mg, 0.26 mmol, 1.3 equiv), 5-amino-2-methoxypyridine (29.8 mg, 0.24 mmol, 1.2 equiv) and anhydrous MeCN (0.67 mL, 0.3 M). Purification by flash column chromatography (20–80% EtOAc/pentane) afforded amino-azetidine **116** as an orange/red oil (71.1 mg, 85%). R_f = 0.36 (40% EtOAc/pentane); IR (film)/cm^{-1} 3341 (NH st), 3059, 2951, 2885, 2837, 1695 (C=O st), 1490, 1416, 1352, 1246, 1178, 1027, 906, 826, 726, 696, 544; ^{1}H NMR (400 MHz, $CDCl_3$) δ 7.45–7.32 (m, 7 H, 7 × Ar-CH), 7.26 (d, *J* = 2.9 Hz, 1 H, Ar-CH), 6.89 (d, *J* = 8.7 Hz, 2 H, 2 × Ar-CH), 6.76 (dd, *J* = 8.8, 2.9 Hz, 1 H, Ar-CH), 6.57 (d, *J* = 8.8 Hz, 1 H, Ar-CH), 5.16 (s, 2 H, CH_2Ph), 4.37 (d, *J* = 8.9 Hz, 2 H, C*H*HNC*H*H), 4.19 (d, *J* = 8.9 Hz, 2 H, CH*H*NCH*H*), 3.83 (s, 3 H, OCH_3), 3.82 (s, 3 H, OCH_3); ^{13}C NMR (101 MHz, $CDCl_3$) δ 159.0 (Ar-C_qOMe), 157.7 (Ar-C_qOMe), 156.5 (C=O), 136.4 (Ar-C_qCH$_2$), 135.1 (Ar-C_qNH), 133.6 (Ar-C_qC$_q$), 131.9 (Ar-CH), 128.6 (2 × Ar-CH), 128.2 (Ar-CH), 128.1 (2 × Ar-CH), 127.4 (Ar-CH), 126.7 (2 × Ar-CH), 114.3 (2 × Ar-CH), 111.0 (Ar-CH), 67.0 (PhCH$_2$), 63.0 (C$_q$), 55.7 (CH_2NCH_2), 55.3 (OCH_3), 53.4 (OCH_3); HRMS (TOF-MS-ES$^+$) *m/z* calcd for $C_{24}H_{26}N_3O_4$ [M+H]: 420.1923, found: 420.1934.

Benzyl 3-((4-bromophenyl)amino)-3-(4-methoxyphenyl)azetidine-1-carboxylate (117)

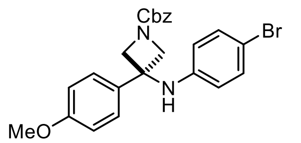

Prepared according to **deFS General Procedure A** under Ar at 60 °C for 2 h with a Celite work-up and using azetidine sulfonyl fluoride **11** (75.8 mg, 0.2 mmol, 1.0 equiv), K_2CO_3 (35.8 mg, 0.26 mmol, 1.3 equiv), *p*-bromoaniline (41.3 mg, 0.24 mmol, 1.2 equiv) and anhydrous MeCN (0.67 mL, 0.3 M). Purification by flash column chromatography (50% Et_2O/*n*-hexane) afforded amino-azetidine **117** as a white solid (84.1 mg, 90%). R_f = 0.10 (40% Et_2O/*n*-hexane); IR (film)/cm^{-1} 3355 (NH st), 2853, 2884, 2834, 1692 (C=O st), 1591, 1510, 1491, 1450, 1416, 1353, 1297, 1247, 1178, 1118, 1026, 962, 812, 761, 696, 606, 507, 459; ^{1}H NMR (400 MHz, $CDCl_3$) δ 7.43–7.32 (m, 7 H, 7 × Ar-CH), 7.19 (d, *J* = 8.6 Hz, 2 H, 2 × Ar-CH), 6.90 (d, *J* = 8.6 Hz, 2 H, 2 × Ar-CH), 6.22 (d, *J* = 8.6 Hz, 2 H, 2 × Ar-CH), 5.17 (s, 2 H, CH_2Ph), 4.64 (br, 1 H, NH), 4.36 (d, *J* = 8.9 Hz, 2 H, C*H*HNC*H*H), 4.21 (d, *J* = 8.9 Hz, 2 H, m, CH*H*NCH*H*), 3.82 (s, 3 H, OCH_3); ^{13}C NMR (101 MHz, $CDCl_3$) δ 159.0 (Ar-C_qOMe), 156.5 (C=O), 143.4 (Ar-C_qNH), 136.3 (Ar-C_qCH$_2$), 133.7 (Ar-C_qC$_q$), 132.0 (2 × Ar-CH), 128.6 (2 × Ar-CH), 128.2 (Ar-CH), 128.1 (2 × Ar-CH), 126.6 (2 × Ar-CH), 115.9 (2 × Ar-CH), 114.2 (2 × Ar-CH), 110.2 (Ar-C_qBr), 67.1 (PhCH$_2$), 63.4 (CH_2NCH_2), 55.3 (OCH_3), 55.2 (C$_q$); HRMS (TOF-MS-ES$^+$) *m/z* calcd for $C_{24}H_{23}N_2O_3{}^{79}Br$ [M+H]: 467.0970, found: 467.0972.

Benzyl 3-(4-hydroxyphenyl)-3-morpholinoazetidine-1-carboxylate (118)

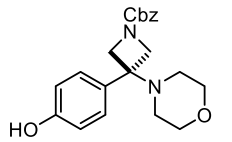

Prepared according to **deFS General Procedure A** under Ar at 60 °C for 2 h with a Celite work-up and using azetidine sulfonyl fluoride **12** (260 mg, 0.49 mmol, 1.0 equiv), K_2CO_3 (88.0 mg, 0.64 mmol, 1.3 equiv), morpholine (50.8 μL, 0.59 mmol, 1.2 equiv) and anhydrous MeCN (1.65 mL, 0.3 M). Purification by flash column chromatography (50–100% EtOAc/*n*-hexane) afforded amino-azetidine **118** as a white solid (150 mg, 85%). R_f = 0.57 (100% EtOAc); mp = 184–186 °C; IR (film)/cm^{-1} 3302 (OH st), 2959, 2854, 2815, 1686 (C=O st), 1445, 1416, 1365, 1267, 1209, 1135, 1111, 1039, 841, 741, 560; ^{1}H NMR (400 MHz, $CDCl_3$) δ 7.37 (m, 5 H, 4 × Ar-CH), 6.97–6.88 (m, 2 H, 2 × Ar-CH), 6.86–6.78 (m, 2 H, 2 × Ar-CH), 6.05 (s, 1 H, OH), 5.13 (s, 2 H, CH_2Ph), 4.24 (m, 4 H, C*HH*NC*HH*), 3.71 (t, *J* = 4.6 Hz, 4 H, CH_2OCH_2), 2.31–2.25 (br, 4 H, $CH_2N_{(morpholine)}CH_2$); ^{13}C NMR (101 MHz, $CDCl_3$) δ 156.6 (C=O st), 155.3 (Ar-C_qOH), 136.4 (Ar-C_qCH$_2$), 128.8 (2 × Ar-CH), 128.5 (2 × Ar-CH), 128.3 (Ar-C_qC$_q$), 128.1(Ar-CH), 128.0 (2 × Ar-CH), 114.7 (2 × Ar-CH), 67.0 (CH_2OCH_2), 66.9(CH_2Ph), 62.3 (C$_q$), 58.5 (br, N*C*H2), 57.7 (br,

N*C*H2), 46.3 ($CH_2N_{(morpholine)}CH_2$); HRMS (TOF-MS-ES$^+$) *m/z* calcd for $C_{21}H_{25}N_2O_4^+$ [M+H]$^+$: 369.1814, found: 369.1802.

Benzyl 3-(*tert*-butylamino)-3-(2-methoxyphenyl)azetidine-1-carboxylate (119)

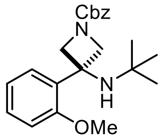

Prepared according to **deFS General Procedure A** under Ar at 60 °C for 4.5 h with a Celite work-up and using azetidine sulfonyl fluoride **13** (76.6 mg, 0.2 mmol, 1.0 equiv), K_2CO_3 (35.8 mg, 0.26 mmol, 1.3 equiv), 2-methylpropan-2-amine (25.2 µL, 0.24 mmol, 1.2 equiv) and anhydrous MeCN (0.67 mL, 0.3 M). Purification by flash column chromatography (30–50% EtOAc/pentane) afforded amino-azetidine **119** as a colorless oil (23.6 mg, 32%). R_f = 0.15 (30% EtOAc/pentane); IR (film)/cm^{-1} 2954, 1709 (C=O st) 1418, 1357, 1239, 1115, 1027, 792; ^{1}H NMR (400 MHz, $CDCl_3$) δ 7.38–7.23 (m, 7 H, 7 × Ar-CH), 6.95 (t, *J* = 7.5 Hz, 1 H, Ar-CH), 6.84 (d, *J* = 8.2 Hz, 1 H, Ar-CH), 5.06 (d, *J* = 3.7 Hz, 2 H, $PhCH_2$), 4.46–4.29 (m, 2 H, C*H*HNC*H*H), 4.12 (d, *J* = 8.7 Hz, 2 H, CH*H*NCH*H*), 3.84 (s, 3 H, OCH_3), 0.82 (s, 9 H, $NC(CH_3)_3$); ^{13}C NMR (101 MHz, $CDCl_3$) δ 158.0 (Ar-C_q-OCH_3), 156.7 (C_q=O), 136.9 (Ar-C_q-C_q), 132.3 (Ar-C_q-CH_2), 128.9 (Ph-CH), 128.5 (2 × Ph-CH), 128.1 (2 × Ph-CH), 128.0 (Ar-CH), 127.5 (Ar-CH), 120.4 (Ar-CH), 110.9 (Ar-CH), 66.6 ($PhCH_2$), 63.2 (CH_2NCH_2), 61.8 (CH_2NCH_2), 56.1 (C_q), 55.2 (OCH_3), 52.4 (N$C_q(CH_3)_3$), 30.5 ($NC(CH_3)_3$); HRMS (TOF-MS-ES$^+$) *m/z* calcd for $C_{22}H_{29}N_2O_3^+$ [M+H]$^+$: 369.2178, found: 369.2184.

***tert*-Butyl 2-((3-(benzo[*d*][1,3]dioxol-5-yl)-1-((benzyloxy)carbonyl)azetidin-3-yl)amino)-6,7-dihydrothiazolo[5,4-*c*]pyridine-5(4*H*)-carboxylate (120)**

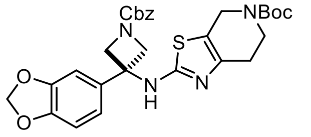

Prepared according to **deFS General Procedure A** under Ar at 60 °C for 4 h with a Celite work-up and using azetidine sulfonyl fluoride **14** (78.7 mg, 0.2 mmol, 1.0 equiv), K_2CO_3 (35.8 mg, 0.26 mmol, 1.3 equiv), *tert*-butyl 2-amino-6,7-dihydrothiazolo[5,4-*c*]pyridine-5(4*H*)-carboxylate (61.3 mg, 0.24 mmol, 1.2 equiv) and anhydrous MeCN (0.67 mL, 0.3 M). Purification by flash column chromatography (20–50% EtOAc/pentane) afforded amino-azetidine **120** as a colorless gum (73.4 mg, 65%). R_f = 0.25 (40% EtOAc/pentane); IR (film)/cm^{-1} 3295, 2974, 1687 (C=O st), 1530, 1418, 1355, 1230, 1154, 1115, 907, 731; ^{1}H NMR (400 MHz, $CDCl_3$) δ 7.42–7.30 (m, 5 H, 5 × Ph-CH), 7.03–6.93 (m, 2 H, 2 × Ar-CH), 6.81 (d, *J* = 8.7 Hz, 1 H, Ar-CH), 5.99 (s, 2 H, OCH_2O), 5.14 (s, 2 H, $PhCH_2$), 4.43–4.27 (m, 6 H, $CH_2N_{(azetidine)}CH_2$ + Ar-CH_2-$N_{(piperidine)}$), 3.71–3.55 (br s, 2 H, C*H*2CH2), 2.68–2.49 (br s, 2 H, CH2C*H*2), 1.96 (br s, 1 H, NH), 1.46 (s, 9 H, $C(CH_3)_3$); ^{13}C NMR (101 MHz, $CDCl_3$) δ 164.9 (C_q=O), 156.5 (C_q=O), 154.6 ($Ar_{(thiazole)}$-C_q-N), 148.4 (Ar-C_q-OCH_2), 147.4 (Ar-C_q-OCH_2), 144.4 ($Ar_{(thiazole)}$-C_q), 136.3 (Ar-C_q-C_q), 134.4 (Ar-C_q-CH_2), 128.6 (2 × Ph-CH_2), 128.2 (Ph-CH), 128.1 (2 × Ph-CH_2), 119.4 (Ar-CH), 114.6 ($Ar_{(thiazole)}$-C_q), 108.3 (Ar-CH), 106.5 (Ar-CH), 101.4 (OCH_2O), 80.2 ($C(CH_3)_3$), 67.1 ($PhCH_2$), 62.0 ($CH_2N_{(azetidine)}CH_2$), 56.8 (C_q), 41.8, 41.3 and 40.7 ($CH_2N_{(piperidine)}CH_2$ rotamers), 28.4 ($C(CH_3)_3$), 26.8 (NCH_2CH_2); HRMS (TOF-MS-ES$^+$) *m/z* calcd for $C_{29}H_{33}N_4O_6S^+$ [M+H]$^+$: 565.2121, found: 565.2105.

Benzyl 3-(benzo[*d*][1,3]dioxol-5-yl)-3-(methyl(3-phenyl-3-(4-(trifluoromethyl)phenoxy)-propyl)amino)azetidine-1-carboxylate (121)

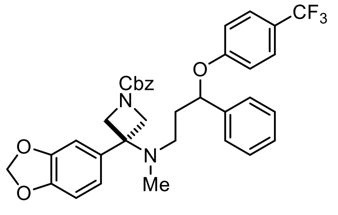

Prepared according to **deFS General Procedure A** under Ar at 60 °C for 4 h with a Celite work-up and using azetidine sulfonyl fluoride **14** (78.7 mg, 0.2 mmol, 1.0 equiv), K_2CO_3 (72.0 mg, 0.52 mmol, 2.6 equiv), fluoxetine hydrochloride (83.0 mg, 0.24 mmol, 1.2 equiv) and anhydrous MeCN (0.67 mL, 0.3 M). Purification by flash column chromatography (10–20% acetone/pentane) afforded amino-azetidine **121** as a colorless gum (80.0 mg, 65%). R_f = 0.28 (20% acetone/pentane); IR (film)/cm^{-1} 2952, 2883, 1701 (C=O st), 1612, 1489, 1323, 1244, 1109, 1068, 808, 729; ^{1}H NMR (400 MHz, DMSO-d_6) δ 7.52–7.48 (m, 2 H, 2 × $Ar_{(CF3)}$-CH), 7.40–7.21 (m, 10 H, 10 × Ph-CH), 7.08–7.03 (m, 2 H, 2 × $Ar_{(CF3)}$-CH), 6.84–6.77 (m, 2 H, 2 × Ar-CH), 6.69 (dd, *J* = 8.0, 1.9 Hz, 1 H, Ar-CH), 5.96 (q, *J* = 1.1 Hz, 2 H, OCH_2O), 5.46 (dd, *J* = 8.0,

4.8 Hz, 1 H, Ph-CH-O), 5.00 (q, *J* = 12.7 Hz, 2 H, $PhCH_2$), 4.12–4.05 (m, 2 H, CH_2NCH_2), 4.04–3.98 (m, 2 H, CH_2NCH_2), 2.40 (dt, *J* = 12.4, 7.3 Hz, 1 H, $CH_3NCHHCH_2$), 2.21 (ddd, *J* = 12.5, 7.7, 5.1 Hz, 1 H, $CH_3NCHHCH_2$), 2.13–2.01 (m, 4 H, CH_3NCH_2CHH and CH_3), 1.94 (ddd, *J* = 14.2, 7.1, 4.8 Hz, 1 H, CH_3NCH_2CHH); ^{13}C NMR (126 MHz, DMSO-d_6) δ 160.2 (Ar-C_q-OCH), 155.4 (C_q=O), 146.6 (Ar-C_q-OCH_2), 145.8 (Ar-C_q-OCH_2), 140.5 (Ph-C_q-CH), 136.5 (Ph-C_q), 131.5 (Ar-C_q-C_q), 127.9 (2 × Ph-CH), 127.7 (2 × Ph-CH), 127.2 (Ph-CH), 127.1 (Ph-CH), 126.9 (2 × Ph-CH), 126.1 (q, $^3J_{C-F}$ = 3.8 Hz, $Ar_{(CF3)}$-CH), 125.6 (2 × Ph-CH), 123.9 (q, $^1J_{C-F}$ = 271.2 Hz, CF_3), 121.0 (q, $^2J_{C-F}$ = 32.0 Hz, Ar-C_q-CF_3), 120.2 (Ar-CH), 115.9 (2 × $Ar_{(CF3)}$-CH), 107.4 (Ar-CH), 106.7 (Ar-CH), 100.4 (OCH_2O), 77.4 (Ph-CH-O), 65.3 ($PhCH_2$), 63.0 (NC_q), 58.3 and 58.3 (CH_2NCH_2), 46.1 ($CH_3NCH_2CH_2$), 35.6 ($CH_3NCH_2CH_2$), 34.0 (CH_3); $^{19}F\{^1H\}$ NMR (377 MHz, DMSO-d_6) δ –59.9; HRMS (TOF-MS-ES$^+$) *m/z* calcd for $C_{35}H_{34}N_2O_5F_3^+$ $[M+H]^+$: 619.2420, found: 619.2419.

Notes:

*The ^{1}H and ^{13}C NMR spectra for **121** were measured at 373 K to resolve poor definition of signals caused by slow rotation of the sterically hindered Cbz group.*

Only the two central peaks of the quartet ^{13}C signals at 125.0 and 122.9 ppm are clearly observed due to the low intensity of these quaternary signals. The two outer peaks of the quartet at 126.1 ppm are seen as shoulders of the central peaks.

3-Ethyl 5-methyl 2-((2-((1-((benzyloxy)carbonyl)-3-(3,4,5-trimethoxyphenyl)azetidin-3-yl)amino)ethoxy)methyl)-4-(2-chlorophenyl)-6-methyl-1,4-dihydropyridine-3,5-dicarboxylate (122)

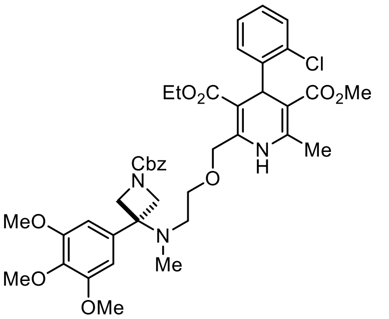

Prepared according to **deFS General Procedure A** under Ar at 60 °C for 24 h with a Celite work-up and using azetidine sulfonyl fluoride **15** (87.9 mg, 0.2 mmol, 1.0 equiv), K_2CO_3 (72.0 mg, 0.52 mmol, 2.6 equiv), amlodipine besylate (136.0 mg, 0.24 mmol, 1.2 equiv) and anhydrous MeCN (0.67 mL, 0.3 M). Purification by flash column chromatography (10–20% acetone/pentane) afforded amino-azetidine **122** as an amorphous white solid (100.0 mg, 65%). R_f = 0.57 (40% acetone/pentane); IR (film)/cm^{-1} 3312 (NH st), 2947, 1687 (C=O st), 1588, 1467, 1415, 1206, 1096, 1037, 910, 729; ^{1}H NMR (400 MHz, $CDCl_3$) δ 7.39–7.31 (m, 6 H, 5 × Ph-CH + $Ar_{(Cl)}$-CH), 7.26–7.21 (m, 2 H, $Ar_{(Cl)}$-CH + C_qNHC_q), 7.13 (td, *J* = 7.5, 1.4 Hz, 1 H, $Ar_{(Cl)}$-CH), 7.05 (td, *J* = 7.6, 1.8 Hz, 1 H $Ar_{(Cl)}$-CH), 6.58 (s, 2 H, 2 × $Ar_{(OMe)}$-CH), 5.40 (s, 1 H, CH), 5.12 (s, 2 H, $PhCH_2$), 4.76 (d, *J* = 16.5 Hz, 1 H, $OCHHC_qNHC_q$), 4.67 (d, *J* = 16.4 Hz, 1 H, $OCHHC_qNHC_q$), 4.33 (d, *J* = 8.9 Hz, 2 H, CHHNCHH), 4.14–4.08 (m, 2 H, CHHNCHH), 4.03 (m, 2 H, OCH_2CH_3), 3.89–3.82 (s, 9 H, 3 × C_q-OCH_3), 3.62 (m, 5 H, CO_2CH_3 + OCH_2CH_2), 2.66 (t, *J* = 5.1 Hz, 2 H, OCH_2CH_2), 2.32 (s, 3 H, C_q-CH_3), 1.90 (br s, 1 H, CH_2NH), 1.18 (t, *J* = 7.1 Hz, 3 H, OCH_2CH_3); ^{13}C NMR (101 MHz, $CDCl_3$) δ 168.0 (CO_2CH_3), 167.2 ($CO_2CH_2CH_3$), 156.8 (NC_q=O), 153.6 (2 × $Ar_{(OMe)}$-C_q-OCH_3), 145.7 ($Ar_{(Cl)}$-C_q-CH), 145.3 (OCH_2-C_q-NH-C_q), 143.9 (C_q-CH_3), 137.7 ($Ar_{(OMe)}$-C_q-OCH_3), 137.5 (Ar-C_q-C_q), 136.5 ($Ar_{(Ph)}$-C_q-CH_2), 132.5 ($Ar_{(Cl)}$-C_q-Cl), 131.6 (CHAr-C_q-Ar-CH), 129.4 (ClAr-C_qAr-CH), 128.6 (2 × Ph-CH), 128.3 (Ph-CH), 128.2 (2 × Ph-CH), 127.5 (ClAr-C_qAr-CHAr-CH), 126.9 (CHAr-C_qAr-CHAr-CH), 104.1 ($C_qCO_2CH_3$), 103.4 (2 × $Ar_{(OMe)}$-CH), 101.7 ($C_qCO_2CH_2CH_3$), 71.4 (OCH_2CH_2), 68.0 ($OCH_2C_qNHC_q$), 67.1 ($PhCH_2$), 61.0 (CH_2NCH_2), 59.9 (OCH_2CH_3), 59.0 ($Ar_{(OMe)}$-C_q-C_q), 56.4 (3 × C_q-OCH_3), 50.9 (CO_2CH_3), 43.1 (OCH_2CH_2), 37.5 (Ar-C_q-CH), 19.6 (C_q-CH_3), 14.4 (OCH_2CH_3); HRMS (TOF-MS-ES$^+$) *m/z* calcd for $C_{40}H_{47}N_3O_{10}{}^{35}Cl^+$ $[M+H]^+$: 764.2950, found: 764.2985.

Benzyl 3-(4-iodo-1*H*-pyrazol-1-yl)-3-(4-methoxyphenyl)azetidine-1-carboxylate (123)

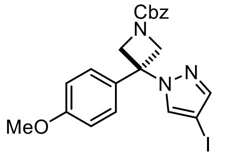

Prepared according to **deFS General Procedure A** under Ar at 60 °C for 2 h with a Celite work-up and using azetidine sulfonyl fluoride **11** (75.9 mg, 0.2 mmol, 1.0 equiv), K_2CO_3 (35.8 mg, 0.26 mmol, 1.3 equiv), 4-iodo-1*H*-pyrazole (46.6 mg, 0.24 mmol, 1.2 equiv) and anhydrous MeCN (0.67 mL, 0.3 M). Purification by flash column chromatography (20% EtOAc/pentane) afforded pyrazole-azetidine **123** as a colorless gum (83.2 mg, 85%). R_f = 0.64 (40% EtOAc/pentane); IR (film)/cm^{-1} 2954, 1702 (C=O st), 1610, 1515, 1418, 1353, 1252, 1176, 1116, 939; ^{1}H NMR (400 MHz, $CDCl_3$) δ 7.65 (s, 1 H, $Ar_{(pyrazole)}$-CH), 7.41–7.31 (m, 5 H, 5 × Ph-CH), 7.15–7.07 (m, 3 H, $Ar_{(pyrazole)}$-CH + 2 × Ar-CH), 6.94 (d, *J* = 8.8 Hz, 2 H, 2 × Ar-CH), 5.14 (s, 2 H, $PhCH_2$), 4.96–4.78 (m, 2 H, C*H*HNC*H*H), 4.64 (d, *J* = 9.1 Hz, 2 H, CH*H*NCH*H*), 3.84 (s, 3 H, OCH_3); ^{13}C NMR (101 MHz, $CDCl_3$) δ 159.9 (Ar-C_q-OCH_3), 156.4 (C_q=O), 145.2 ($Ar_{(pyrazole)}$-CH), 136.3 (Ar-C_q-C_q), 133.3 ($Ar_{(pyrazole)}$-CH), 131.3 (Ar-C_q-CH_2), 128.5 (2 × Ph-CH), 128.2 (Ph-CH), 128.1 (2 × Ph-CH), 127.8 (2 × Ar-CH), 114.5 (2 × Ar-CH), 67.1 ($PhCH_2$), 62.2 (C_q), 61.8 (CH_2NCH_2), 57.0 ($Ar_{(pyrazole)}$-C_q-I), 55.4 (OCH_3); HRMS (TOF-MS-ES$^+$) *m/z* calcd for $C_{21}H_{21}N_3O_3I^+$ $[M+H]^+$: 490.0628, found: 490.0625.

Benzyl 3-(1*H*-imidazol-1-yl)-3-(4-methoxyphenyl)azetidine-1-carboxylate (124)

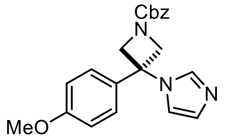

Prepared according to **deFS General Procedure A** under Ar at 70 °C for 2 h with a Celite work-up and using azetidine sulfonyl fluoride **11** (75.9 mg, 0.2 mmol, 1.0 equiv), K_2CO_3 (35.8 mg, 0.26 mmol, 1.3 equiv), 1*H*-imidazole (40.8 mg, 0.60 mmol, 3.0 equiv) and anhydrous MeCN (0.67 mL, 0.3 M). Purification by automated flash column chromatography (EtOAc, see conditions below) afforded imidazole-azetidine **124** as a colorless gum (58 mg, 80%). R_f = 0.28 (EtOAc); IR (film)/cm^{-1} 3111, 2958, 2891, 1708 (C=O st), 1610, 1514, 1452, 1415, 1354, 1301, 1252, 1183, 1137, 1072, 1026, 832, 738, 699, 663; ^{1}H NMR (400 MHz, $CDCl_3$) δ 7.42–7.31 (m, 6 H, $Ar_{(imidazole)}$-CH + 5 × Ar-CH), 7.16 (s, 1 H, $Ar_{(imidazole)}$-CH), 7.07–7.00 (m, 3 H, $Ar_{(imidazole)}$-CH + 2 × Ar-CH), 6.93 (d, *J* = 8.6 Hz, 2 H, 2 × Ar-CH), 5.16 (s, 2 H, $PhCH_2$), 4.68 (d, *J* = 9.2 Hz, 2 H, C*H*HNC*H*H), 4.64 (d, *J* = 9.2 Hz, 2 H, CH*H*NCH*H*), 3.83 (s, 3 H, OCH_3); ^{13}C NMR (101 MHz, $CDCl_3$) δ 159.9 (Ar-C_q-OMe), 156.3 (C=O), 136.3 ($Ar_{(imidazole)}$-CH), 136.0 (Ar-C_q-CH_2), 132.2 (Ar-C_q-C_q), 130.3 ($Ar_{(imidazole)}$-CH), 128.6 (2 × Ar-CH), 128.4 (Ar-CH), 128.2 (2 × Ar-CH), 127.0 (2 × Ar-CH), 117.7 ($Ar_{(imidazole)}$-CH), 114.5 (2 × Ar-CH), 67.4 ($PhCH_2$), 63.3 (br, CH_2NCH_2), 58.2 (C_q), 55.4 (OCH_3); HRMS (TOF-MS-ES$^+$) *m/z* calcd for $C_{21}H_{21}N_3O_3$ [M+H]: 364.1668, found: 364.1661.

Automated Column Conditions: Run on a Biotage® Selekt system. Column type: Biotage® Sfär® HC Duo 10 g. Flow rate: 40 mL/min. Sample mass: 200 mg. Solvent A: *n*-hexane, Solvent B: EtOAc, Solvent C: MeOH. UV wavelength detection: l 200–400 nm. See trace and gradient below. From the left: peak 1 (blue): azetidine fluoride, peak 2 (green): azetidine imidazole **124**, peak 3 (flat, green): imidazole, peak 4 (yellow): azetidine sulfonyl imidazole.

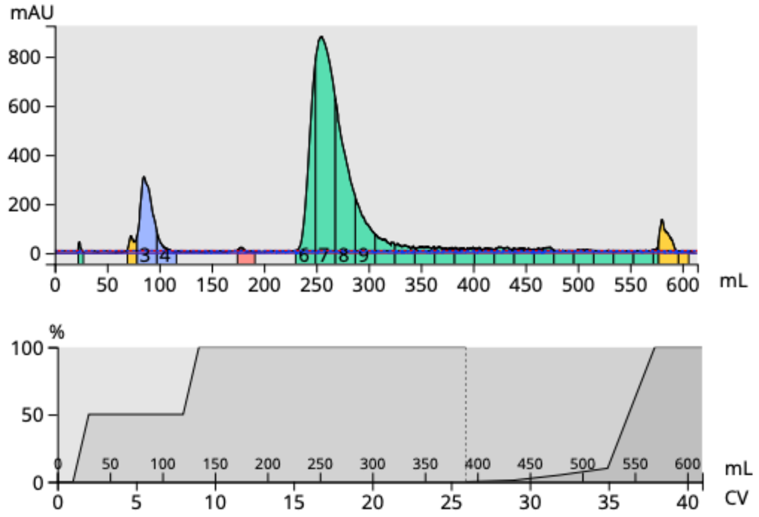

Benzyl 3-(2-methoxyphenyl)-3-(1*H*-1,2,4-triazol-1-yl)azetidine-1-carboxylate (125)

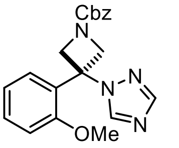

Prepared according to **deFS General Procedure A** under Ar at 60 °C for 4.5 h with a Celite work-up and using azetidine sulfonyl fluoride **13** (64.5 mg, 0.17 mmol, 1.0 equiv), K_2CO_3 (30.5 mg, 0.22 mmol, 1.3 equiv), 1/4*H*-1,2,4-triazole (35.2 mg, 0.51 mmol, 3.0 equiv) and anhydrous MeCN (0.57 mL, 0.3 M). Purification by flash column chromatography (20–40% EtOAc/pentane) afforded triazole-azetidine **125** as a colorless oil (55.2 mg, 88%). R_f = 0.29 (35% EtOAc/pentane); IR (film)/cm^{-1} 2948, 1709 (C=O st), 1495, 1415, 1355, 1251, 1135, 1027, 753; ^{1}H NMR (400 MHz, $CDCl_3$) δ 7.63–7.60 (m, 1 H, Ar-CH), 7.44–7.28 (m, 5 H, 5 × Ph-CH), 7.19 (dd, *J* = 7.7, 1.7 Hz, 1 H, Ar-CH), 7.08 (d, *J* = 2.4 Hz, 1 H, Ar-CH), 7.03 (t, *J* = 7.5 Hz, 1 H, Ar-CH), 6.94 (d, *J* = 8.3 Hz, 1 H, Ar-CH), 6.16 (t, *J* = 2.1 Hz, 1 H, Ar-CH), 5.12 (s, 2 H, $PhCH_2$), 4.99–4.68 (m, 4 H, CH_2NCH_2), 3.76 (s, 3 H, Ar-C_q-OCH_3); ^{13}C NMR (101 MHz, $CDCl_3$) δ 157.5 (Ar-C_q-OCH_3), 156.7 (C_q=O), 140.2 ($Ar_{(triazole)}$-CH), 136.7 (Ph-C_q-CH_2), 130.8 ($Ar_{(triazole)}$-CH), 128.6 (2 × Ph-CH), 128.5 (Ph-CH), 128.2 (2 × Ph-CH), 128.2 (Ar-CH), 126.8 (Ar-C_q-C_q), 121.0 (Ar-CH), 111.6 (Ar-CH), 105.4 (Ar-CH), 67.0 ($PhCH_2$), 61.8 (CH_2NCH_2), 61.1 (C_q), 55.5 (OCH_3); HRMS (TOF-MS-ES$^+$) *m/z* calcd for $C_{22}H_{29}N_2O_3^+$ [M+H]$^+$: 369.2178, found: 369.2184.

Benzyl 3-(((4-bromophenyl)(methyl)(oxo)-λ6-sulfaneylidene)amino)-3-(4-methoxyphenyl)-azetidine-1-carboxylate (126)

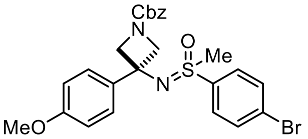

Prepared according to **deFS General Procedure A** under Ar at 60 °C for 2 h with a Celite work-up and using azetidine sulfonyl fluoride **11** (75.9 mg, 0.2 mmol, 1.0 equiv), (4-bromophenyl)(imino)(methyl)-λ^6-sulfanone **S21** (140.5 mg, 0.60 mmol, 3.0 equiv), K_2CO_3 (37.5 mg, 0.26 mmol, 1.3 equiv), and anhydrous MeCN (0.34 mL, 0.3 M). The residue was diluted with EtOAc (20 mL) and washed with aq. HCl (1 M, 3 × 20 mL). The organic layer was separated, dried over Na_2SO_4, filtered, and concentrated *in vacuo* using a rotary evaporator. Purification by flash column chromatography (20–100% EtOAc/*n*-hexane) afforded azetidine sulfoximine **126** as a white solid (86 mg, 82%). R_f = 0.27 (50% EtOAc/*n*-hexane); mp = 158–159 °C; IR (film)/cm^{-1} 2952, 2885, 1703 (C=O st), 1513, 1416 (S=O st), 1356, 1244, 1177, 1118, 767; ^{1}H NMR (400 MHz, $CDCl_3$) δ 7.41 (br, 4 H, 4 × Ar-CH), 7.36–7.28 (m, 5 H, 5 × Ar-CH), 7.10 (br, 2 H, 2 × Ar-CH), 6.60 (d, *J* = 8.3 Hz, 2 H, 2 × Ar-CH), 5.08 (s, 2 H, $PhCH_2$), 4.50 (d, *J* = 8.7 Hz, 1 H, CH*H*NCHH), 4.46–4.34 (m, 3 H, C*H*HNC*HH*), 3.72 (s, 3 H, OCH_3), 2.94 (s, 3 H, SCH_3); ^{13}C NMR (101 MHz, $CDCl_3$) δ 158.6 (Ar-C_q-OMe), 156.3 (C=O), 140.7 (Ar-C_q-S), 136.6 (Ar-C_q-CH_2), 135.0 (Ar-C_q-C_q), 131.9 (2 × Ar-CH), 129.1 (2 × Ar-CH), 128.3 (2 × Ar-CH), 127.9 (Ar-CH), 127.8 (2 × Ar-CH), 127.5 (2 × Ar-CH), 126.9 (Ar-C_q-Br), 113.3 (2 × Ar-CH), 66.5 ($PhCH_2$), 65.5 (br, CH*H*NCHH), 64.9 (br, CHHNC*H*H), 63.6 (br, C*H*HNCHH), 63.1 (br, CHHNCH*H*), 57.8 (C_q),

55.2 (OCH_3), 46.9 (SCH_3); HRMS (TOF-MS-ES$^+$) *m/z* calcd for $C_{25}H_{26}N_2O_4S^{79}Br^+$ $[M+H]^+$: 529.0797; found 529.0795.

Benzyl 3-(diethoxyphosphoryl)-3-(4-methoxyphenyl)azetidine-1-carboxylate (127)

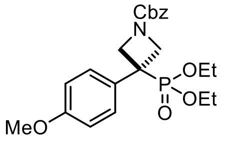

Prepared according to **deFS General Procedure A** under Ar at 60 °C for 2 h with a Celite work-up and using azetidine sulfonyl fluoride **11** (38.0 mg, 0.1 mmol, 1.0 equiv) and triethyl phosphite (51.4 µL, 0.3 mmol, 3.0 equiv) and anhydrous MeCN (0.34 mL, 0.3 M). Purification by flash column chromatography (70% EtOAc/pentane) afforded azetidine phosphonate **127** as a white solid (24.9 mg, 57%). R_f = 0.17 (70% EtOAc/pentane); mp = 92–94 °C; IR (film)/cm^{-1} 2979, 1711 (C=O st), 1513, 1413, 1250 (P=O st), 1050 (P-O-C al st), 1027 (P-O-C al st), 964, 557; $^1H\{^{31}P\}$ NMR (400 MHz, $CDCl_3$) δ 7.38–7.29 (m, 5 H, 5 × Ph-CH), 7.15 (d, *J* = 8.7 Hz, 2 H, 2 × Ar-CH), 6.91 (d, *J* = 8.6 Hz, 2 H, 2 × Ar-CH), 5.11 (s, 2 H, $PhCH_2$), 4.67 (d, *J* = 8.6 Hz, 2 H, C*H*HNC*H*H), 4.34 (d, *J* = 8.6 Hz, 2 H, CH*H*NCH*H*), 4.05–3.89 (m, 4 H, $P(OCH_2CH_3)_2$), 3.81 (s, 3 H, OCH_3), 1.22 (t, *J* = 7.0 Hz, 6 H, $P(OCH_2CH_3)_2$); ^{13}C NMR (101 MHz, $CDCl_3$) δ 159.1 (Ar-C_q-OCH_3), 156.1 (C_q=O), 136.7 (Ph-C_q-CH_2), 130.0 (Ar-C_q-C_q), 128.8 (d, $^3J_{C–P}$ = 3.5 Hz, 2 × Ar-CH), 128.5 (2 × Ph-CH), 128.0 (Ph-CH), 128.0 (2 × Ph-CH), 114.0 (2 × Ar-CH), 66.8 ($PhCH_2$), 63.1 (d, $^2J_{C–P}$ = 6.1 Hz, $P(OCH_2CH_3)_2$), 57.4 (CH_2NCH_2), 55.3 (OCH_3), 40.7 (d, $^1J_{C–P}$ = 150.0 Hz, C_q), 16.3 (d, $^3J_{C–P}$ = 4.3 Hz, $P(OCH_2CH_3)_2$); $^{31}P\{^1H\}$ NMR (162 MHz, $CDCl_3$) δ 25.3 (s, $P(OCH_2CH_3)_2$); HRMS (TOF-MS-ES$^+$) *m/z* calcd for $C_{22}H_{29}NO_6P^+$ $[M+H]^+$: 434.1733, found: 434.1733.

Notes:

*The ^1H and ^{13}C NMR spectra for **127** were measured at 328 K to resolve poor definition of signals caused by slow rotation of the sterically hindered $PO(OEt)_2$ group.*

Benzyl 3-((diphenylphosphoryl)oxy)-3-(4-methoxyphenyl)azetidine-1-carboxylate (128)

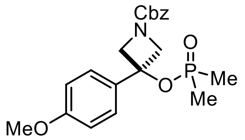

Prepared according to **deFS General Procedure A** under Ar at 60 °C for 2 h with a Celite work-up and using azetidine sulfonyl fluoride **11** (38.0 mg, 0.1 mmol, 1.0 equiv), K_2CO_3 (41.4 mg, 0.3 mmol, 3.0 equiv) and diphenylphosphine oxide (61.0 mg, 0.3 mmol, 3.0 equiv) and anhydrous MeCN (0.34 mL, 0.3 M). Purification by flash column chromatography (20–40% EtOAc/pentane) afforded azetidine phosphinate **128** as a white gum (11.3 mg, 22%). R_f = 0.51 (80% EtOAc/pentane); IR (film)/cm^{-1} 2950, 1709 (C=O st), 1601, 1515, 1439, 1420, 1355, 1250 (P=O st), 1179, 1131, 1027 (P-O-C al st), 956, 729, 695; ^1H NMR (400 MHz, $CDCl_3$) δ 7.66–7.56 (m, 4 H, 4 × Ar-CH), 7.46–7.38 (m, 2 H, 2 × Ar-CH), 7.36–7.25 (m, 11 H, 11 × Ar-CH), 6.72–6.64 (m, 2 H, 2 × Ar-CH), 5.08 (s, 2 H, $PhCH_2$), 4.80 (d, *J* = 9.8 Hz, 2 H, C*H*HNC*H*H), 4.51 (d, *J* = 9.8 Hz, 2 H, CH*H*NCH*H*), 3.74 (s, 3 H, OCH_3); ^{13}C NMR (101 MHz, $CDCl_3$) δ 159.6 (Ar-C_q-OCH_3), 156.3 (C_q=O), 136.4 (Ph-C_q-CH_2), 133.1 (Ph-C_q-P), 131.8 (2 × Ph-CH + Ph-C_q-P), 131.2 (d, $^3J_{C–P}$ = 10.4 Hz, 4 × Ph-CH), 131.0 (d, $^3J_{C–P}$ = 2.2 Hz, Ar-C_q-C_q), 128.4 (2 × Ph-CH), 128.2 (2 × Ar-CH), 128.2 (d, $^2J_{C–P}$ = 13.4 Hz, 4 × Ph-CH), 128.0 (Ph-CH), 127.9 (2 × Ph-CH), 113.6 (2 × Ar-CH), 78.3 (d, $^3J_{C–P}$ = 7.9 Hz, C_q), 66.8 ($PhCH_2$), 62.7 (CH_2NCH_2), 55.2 (OCH_3); $^{31}P\{^1H\}$ NMR (162 MHz, $CDCl_3$) δ 30.6 (s, $OP(O)(Ph)_2$); HRMS (TOF-MS-ES$^+$) *m/z* calcd for $C_{30}H_{28}NO_5PNa^+$ $[M+Na]^+$: 536.1603, found: 536.1580.

Notes:

*The ^1H NMR spectrum for **128** was measured at 328 K to resolve poor definition of signals caused by slow rotation of the sterically hindered $OPO(Ph)_2$ group.*

The other Ph-C_q-P signal from the ^{31}P splitting in the ^{13}C NMR cannot be observed due to overlap the Ph-CH signal at 131.8 ppm.

Benzyl 3-(diethoxyphosphoryl)-3-(2-methoxyphenyl)azetidine-1-carboxylate (129)

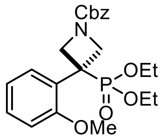

Prepared according to **deFS General Procedure A** under Ar at 60 °C for 4.5 h with a Celite work-up and using azetidine sulfonyl fluoride **13** (64.5 mg, 0.17 mmol, 1.0 equiv) and triethyl phosphite (51.4 μL, 0.3 mmol, 3.0 equiv) and anhydrous MeCN (0.57 mL, 0.3 M). Purification by flash column chromatography (30% EtOAc/pentane) afforded azetidine phosphonate **129** as a colorless oil (27.0 mg, 36%). R_f = 0.15 (30% EtOAc/pentane) IR (film)/cm^{-1} 2980, 1713 (C=O st), 1497, 1415, 1249, 1027, 964, 755; $^1H\{^{31}P\}$ NMR (400 MHz, $CDCl_3$) δ 7.38–7.23 (m, 6 H, 5 × Ph-CH + Ar-CH), 7.05–6.99 (m, 1 H, Ar-CH), 6.96 (t, *J* = 7.4 Hz, 1 H, Ar-CH), 6.88 (d, *J* = 8.3 Hz, 1 H, Ar-CH), 5.09 (d, *J* = 3.4 Hz, 2 H, $PhCH_2$), 4.64 (dd, *J* = 17.2, 9.2 Hz, 2 H, C*H*HNC*H*H), 4.42 (dd, *J* = 18.1, 9.2 Hz, 2 H, CH*H*NCH*H*), 4.06–3.88 (m, 4 H, $P(OCH_2CH_3)_2$), 3.81 (s, 3 H, OCH_3), 1.21 (t, *J* = 7.0 Hz, 6 H, $P(OCH_2CH_3)_2$); ^{13}C NMR (101 MHz, $CDCl_3$) δ 157.6 (Ar-C_q-OCH_3), 156.2 (C_q=O), 136.7 (Ph-C_q-CH_2), 129.4 (d, $^3J_{C-P}$ = 4.4 Hz, Ar-CH), 129.2 (d, $^4J_{C-P}$ = 2.6 Hz, Ar-CH), 128.6 (2 × Ph-CH), 128.1 (3 × Ph-CH), 125.7 (Ar-C_q-C_q), 120.7 (Ar-CH), 111.3 (Ar-CH), 66.8 ($PhCH_2$), 63.0 ($P(OCH_2CH_3)_2$), 57.1 (d, $^2J_{C-P}$ = 71.6 Hz, CH_2NCH_2), 55.5 (OCH_3), 39.9 (d, $^1J_{C-P}$ = 147.8 Hz, C_q), 16.5 (d, $^3J_{C-P}$ = 5.2 Hz, $P(OCH_2CH_3)_2$); $^{31}P\{^1H\}$ NMR (162 MHz, $CDCl_3$) δ 25.6; HRMS (TOF-MS-ES$^+$) *m/z* calcd for $C_{22}H_{29}NO_6P^+$ [M+H]$^+$: 434.1733, found: 434.1721.

***tert*-Butyl 3-(4-methoxyphenyl)-3-morpholinoazetidine-1-carboxylate (149)**

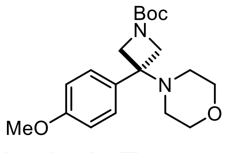

A microwave vial (1) was charged with K_2CO_3 (34.2 mg, 0.25 mmol, 1.2 equiv) and flame-dried under Ar. The microwave vial (1) was sealed and morpholine (21.0 μL, 0.24 mmol, 1.2 equiv) and anhydrous MeCN (0.50 mL, 0.4 M) were added sequentially by syringe. The microwave vial (1) was cooled to 0 °C in an ice bath. To a separate flame-dried microwave vial (2) charged with Ar and cooled to 0 °C was added azetidine sulfonyl fluoride **147** (70.1 mg, 0.20 mmol, 1.0 equiv) and anhydrous MeCN (0.20 mL). The solution of **147** (0.20 mL) was added to the morpholine solution (1) at 0 °C. Once the addition was complete, the reaction mixture was warmed to room temperature and stirred for 30 min before heating to 40 °C. After 4 h at 40 °C, the reaction was diluted with EtOAc (5 mL) and filtered through a plug of Celite, eluting with further EtOAc (3 × 5 mL). The solvent was removed *in vacuo* using a rotary evaporator. Purification by flash column chromatography (50% EtOAc/pentane) afforded amino-azetidine **149** as a colorless oil (48.8 mg, 70%). R_f = 0.21 (50% EtOAc/pentane); IR (film)/cm^{-1} 2961, 1700 (C=O st), 1513, 1403, 1249, 1116, 833; ^1H NMR (400 MHz, $CDCl_3$) δ 7.05 (d, *J* = 8.2 Hz, 2 H, 2 × Ar-CH), 6.92 (d, *J* = 8.2 Hz, 2 H, 2 × Ar-CH), 4.15 (t, *J* = 5.5 Hz, 4 H, $CH_2N_{(azetidine)}CH_2$), 3.84 (s, 3 H, OCH_3), 3.71 (t, *J* = 4.6 Hz, 4 H, CH_2OCH_2), 2.30 (d, *J* = 5.5 Hz, 4 H, $CH_2N_{(morpholine)}CH_2$), 1.44 (s, 9 H, $C(CH_3)_3$).; ^{13}C NMR (101 MHz, $CDCl_3$) δ 159.0 (NC=O), 156.4 (Ar-C_q-OCH_3), 129.2 (Ar-C_q-C_q), 128.8 (2 × Ar-CH), 113.3 (2 × Ar-CH), 79.7 ($OC(CH_3)_3$), 67.2 (CH_2OCH_2), 61.9 (CH_2NCH_2), 57.8 (br, C_q), 55.5 (OCH_3), 46.5 ($CH_2N_{(morpholine)}CH_2$), 28.5 ($C(CH_3)_3$). HRMS (TOF-MS-ES$^+$) *m/z* calcd for $C_{19}H_{29}N_2O_4^+$ [M+H]$^+$: 349.2127, found: 349.2118.

Notes:

If the reaction is performed according to ***deFS General Procedure A*** *(i.e. immediately heated to 60 °C) the yield is significantly lower (37%).*

***tert*-Butyl 3-morpholino-3-(4-(prop-2-yn-1-yloxy)phenyl)azetidine-1-carboxylate (151)**

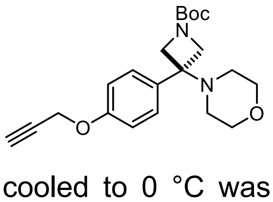

A microwave vial (1) was charged with K_2CO_3 (34.2 mg, 0.25 mmol, 1.3 equiv) and flame-dried under Ar. The microwave vial (1) was sealed and morpholine (20.0 μL, 0.23 mmol, 1.2 equiv) and anhydrous MeCN (0.40 mL, 0.38 M) were added sequentially by syringe. The microwave vial (1) was cooled to 0 °C in an ice bath. To a separate flame-dried microwave vial (2) charged with Ar and cooled to 0 °C was added azetidine sulfonyl fluoride **150** (69.9 mg, 0.19 mmol, 1.0 equiv) and anhydrous MeCN (0.20 mL). The solution of **150** (0.20 mL) was added to the morpholine solution (1)

at 0 °C. Once the addition was complete, the reaction mixture was warmed to room temperature and stirred for 30 min before being heated to 40 °C for 4 h and then 60 °C. After 16 h at 60 °C, the reaction was diluted with EtOAc (5 mL) and filtered through a plug of Celite, eluting further with EtOAc (3 × 5 mL). The solvent was removed *in vacuo* using a rotary evaporator. Purification by flash column chromatography (40% EtOAc/pentane) afforded amino-azetidine **151** as a colorless oil (36.6 mg, 52%). R_f = 0.35 (50% EtOAc/pentane); IR (film)/cm^{-1} 3289, 2969, 1692 (C=O st), 1511, 1402, 1170, 1116, 1019, 732; ^{1}H NMR (400 MHz, $CDCl_3$) δ 7.03 (d, *J* = 8.4 Hz, 2 H, 2 × Ar-CH), 6.97 (d, *J* = 8.4 Hz, 2 H, 2 × Ar-CH), 4.70 (d, *J* = 2.4 Hz, 2 H, C*H*$_2$CCH), 4.17–4.10 (m, 4 H, $CH_2N_{(azetidine)}CH_2$), 3.68 (t, *J* = 4.6 Hz, 4 H, CH_2OCH_2), 2.53 (t, *J* = 2.9 Hz, 1 H, CH_2CC*H*), 2.32–2.24 (m, 4 H, $CH_2N_{(morpholine)}CH_2$), 1.42 (s, 9 H, C(C*H*$_3$)$_3$); ^{13}C NMR (101 MHz, $CDCl_3$) δ 156.9 (NC=O), 156.4 (Ar-*C*$_q$-OCH$_2$CCH), 130.1 (Ar-*C*$_q$-C$_q$), 128.8 (2 × Ar-CH), 114.3 (2 × Ar-CH), 79.7 (O*C*(CH$_3$)$_3$), 78.5 (CH$_2$*C*CH), 75.9 (CH$_2$C*C*H), 67.1 (CH_2OCH_2), 61.9 ($CH_2N_{(azetidine)}CH_2$), 57.8 (br, C$_q$), 55.9 (*C*H$_2$CCH), 46.5 ($CH_2N_{(morpholine)}CH_2$), 28.5 (C(*C*H$_3$)$_3$); HRMS (TOF-MS-ES$^+$) *m/z* calcd for $C_{21}H_{29}N_2O_4^+$ [M+H]$^+$: 373.2127, found: 373.2133.

Prop-2-yn-1-yl 3-(4-methoxyphenyl)-3-morpholinoazetidine-1-carboxylate (153)

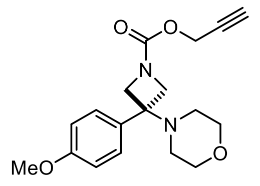

A microwave vial (1) was charged with K_2CO_3 (33.0 mg, 0.24 mmol, 1.2 equiv) and flame-dried under Ar. The microwave vial (1) was sealed and morpholine (21.0 µL, 0.24 mmol, 1.2 equiv) and anhydrous MeCN (0.50 mL) were added sequentially by syringe. The microwave vial (1) was cooled to 0 °C in an ice bath. To a separate flame-dried microwave vial (2) charged with Ar and cooled to 0 °C was added azetidine sulfonyl fluoride **152** (65.1 mg, 0.20 mmol, 1.0 equiv) and anhydrous MeCN (0.20 mL). The solution of **152** (0.20 mL) was added to the morpholine solution (1) at 0 °C. Once the addition was complete, the reaction mixture was warmed to room temperature and stirred for 30 min before being heated to 40 °C. After 4 h at 40 °C, the reaction was diluted with EtOAc (5 mL) and filtered through a plug of Celite, eluting with further EtOAc (3 × 5 mL). The solvent was removed *in vacuo* using a rotary evaporator. Purification by flash column chromatography (50% EtOAc/pentane) afforded amino-azetidine **153** as a colorless oil (42.3 mg, 64%). R_f = 0.20 (50% EtOAc/pentane); IR (film)/cm^{-1} 3284, 2950, 1713 (C=O st), 1608, 1513, 1419, 1247, 1115, 1023, 833; ^{1}H NMR (400 MHz, $CDCl_3$) δ 7.00 (d, *J* = 8.6 Hz, 2 H, 2 × Ar-CH), 6.89 (d, *J* = 8.6 Hz, 2 H, 2 × Ar-CH), 4.66 (d, *J* = 2.5 Hz, 2 H, C*H*$_2$CCH), 4.26–4.19 (m, 4 H, $CH_2N_{(azetidine)}CH_2$), 3.81 (s, 3 H, OCH_3), 3.67 (t, *J* = 4.6 Hz, 4 H, CH_2OCH_2), 2.45 (t, *J* = 2.5 Hz, 1 H, CH_2CC*H*), 2.26 (t, *J* = 4.6 Hz, 4 H, $CH_2N_{(morpholine)}CH_2$); ^{13}C NMR (101 MHz, $CDCl_3$) δ 159.0 (NC=O), 155.5 (Ar-*C*$_q$-OCH$_3$), 128.7 (2 × Ar-CH), 128.6 (Ar-*C*$_q$-C$_q$), 113.4 (2 × Ar-CH), 78.4 (CH$_2$*C*CH), 74.7 (CH$_2$C*C*H), 67.1 (CH_2OCH_2), 62.5 ($CH_2N_{(azetidine)}CH_2$), 58.1 (br, C$_q$), 55.4 (OCH_3), 52.7 (*C*H$_2$CCH), 46.4 ($CH_2N_{(morpholine)}CH_2$); HRMS (TOF-MS-ES$^+$) *m/z* calcd for $C_{18}H_{23}N_2O_4^+$ [M+H]$^+$: 331.1658, found: 331.1653.

***tert*-Butyl-3-*tert*-butyldimethylsilylthymidine-3-(4-(prop-2-yn-1-yloxy)phenyl)azetidine-1-carboxylate (154)**

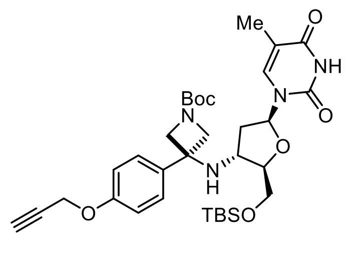

A microwave vial was charged with K_2CO_3 (11.8 mg, 0.09 mmol, 1.3 equiv) and flame-dried under Ar. Amine **S24** (23.8 mg, 0.07 mmol, 1.0 equiv) and anhydrous MeCN (0.23 mL, 0.3 M) were added to the microwave vial and the resulting reaction mixture was cooled to 0 °C. Azetidine sulfonyl fluoride **150** (26.8 mg, 0.07 mmol, 1.0 equiv) was added to the reaction mixture and the microwave vial was sealed. The reaction mixture was warmed to 40 °C for 4 h and then to 60 °C for 2 h. The reaction was diluted with EtOAc (5 mL) and filtered through a plug of Celite, eluting with further EtOAc (3 × 5 mL). The solvent was then removed in vacuo using a rotatory evaporator. Purification by flash column chromatography (50% EtOAc/pentane) afforded amino-azetidine **154** as a white solid (26.7 mg, 63%). R_f = 0.35 (60% EtOAc/pentane); mp = 89 °C; IR (film)/cm^{-1} 3310, 2928, 1690 (C=O st), 1403, 1258, 1120, 833; ^{1}H NMR (400 MHz, $CDCl_3$) δ 8.34 (s, 1 H, N*H*(CO)$_2$), 7.24–7.19 (m, 3 H,

2 × Ar-CH and NC*H*CCH$_3$), 6.98 (d, *J* = 7.9 Hz, 2 H, 2 × Ar-C*H*), 6.13 (t, *J* = 6.4 Hz, 1 H, OC*H*N), 4.69 (d, *J* = 2.4 Hz, 2 H, *C*H$_2$CCH), 4.28 (dd, *J* = 8.7, 4.3 Hz, 2 H, C*H$_2$*NCH$_2$), 3.97 (d, *J* = 8.6 Hz, 2 H, CH$_2$NC*H$_2$*), 3.81–3.70 (m, 1 H, C*H*CH$_2$OTBS), 3.60 (d, *J* = 10.2 Hz, 2 H, C*H$_2$*OTBS), 3.23 (d, *J* = 6.4 Hz, 1 H, NHC*H*), 2.52 (t, *J* = 2.4 Hz, 1 H, CH$_2$CC*H*), 1.87 (s, 3 H, CCH$_3$), 1.85–1.69 (m, 2 H, NHCHC*H$_2$*CH), 1.64 (s, 1 H, C$_q$N*H*) 1.43 (s, 9 H, C(CH$_3$)$_3$), 0.89 (s, 9 H, SiC(CH$_3$)$_3$), 0.05 (s, 3 H, Si(CH$_3$)$_2$), 0.04 (s, 3 H, Si(C*H$_3$*)$_2$); ^{13}C NMR (101 MHz, CDCl$_3$) δ 163.5 (N*C*(O)C(CH$_3$)CH), 160.7 (N*C*O$_2$C(CH$_3$)$_3$), 157.2 (N*C*(O)N), 156.5 (Ar-*C$_q$*-OCH$_2$CCH), 150.2 (Ar-*C*$_q$-C$_q$), 135.5 (C(CH$_3$)*C*HN), 127.9 (2 × Ar-CH), 115.3 (2 × Ar-CH and *C*(CH$_3$)CHN), 85.7 (OCHN), 84.6 (*C*HCH$_2$OTBS), 80.0 (O*C*(CH$_3$)$_3$), 78.4 (CH$_2$C*C*H), 75.9 (CH$_2$*C*CH), 62.8 (CH$_2$OTBS), 60.9 (br, *C*$_q$), 58.0 (*C*H$_2$CCH), 56.0 (*C*H$_2$NCH$_2$), 54.1 (NH*C*H), 40.6 (NHCH*C*H$_2$CH), 28.5 (OC(*C*H$_3$)$_3$), 26.1 (SiC(*C*H$_3$)$_3$), 18.5 (Si*C*(CH$_3$)$_3$), 12.7 (CHC*C*H$_3$), –5.3 (Si(CH$_3$)$_2$); HRMS (TOF-MS-ES$^+$) *m/z* calcd for $C_{33}H_{49}N_4O_7Si^+$ [M+H]$^+$: 641.3371, found: 641.3362.

Azetidine SuFEx product (130)

Benzyl 3-((((3*R*,8*R*,9*R*,10*S*,13*S*,14*R*,17*S*)-10,13-dimethyl-17-((*S*)-6-methylheptan-2-yl)-2,3,4,7,8,9,10,11,12,13,14,15,16,17-tetradecahydro-1*H*-cyclopenta[*a*]phenanthren-3-yl)oxy)sulfonyl)-3-(3,4,5-trimethoxyphenyl)azetidine-1-carboxylate (130)

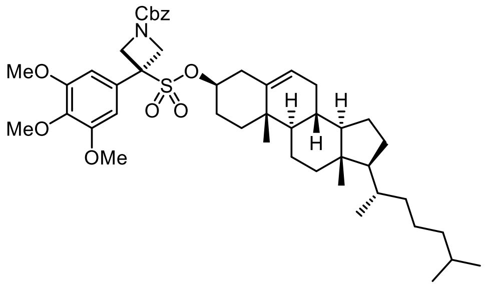

NaH (60% in mineral oil, 12.0 mg, 0.3 mmol,1.5 equiv) was added to a solution of cholesterol (116 mg, 0.3 mmol, 1.5 equiv) in anhydrous THF (0.67 mL, 0.3 M) at 0 °C. The reaction was allowed to warm to rt over 30 min. Azetidine sulfonyl fluoride **15** (87.9 mg, 0.2 mmol, 1.0 equiv) was added and the reaction stirred at rt for 20 h. EtOAc (10 mL) was then added and the mixture was filtered through a plug of Celite, eluting with further EtOAc (3 × 10 mL). Purification by automated flash column chromatography (50% Et$_2$O/pentane, see conditions below) afforded azetidine sulfinate ester **130** as a white solid (81 mg, 50%). R_f = 0.22 (50% Et$_2$O/pentane); mp = 127–129 °C; IR (film)/cm^{-1} 2939, 1716 (C=O st), 1589, 1509, 1455, 1416, 1353 (SO$_2$ st as), 1246, 1129 (SO$_2$ st sy), 920, 732, 666, 652; ^{1}H NMR (400 MHz, CDCl$_3$) δ 7.37 (s, 5 H, 5 × Ar-CH), 6.52 (s, 2 H, 2 × Ar-CH), 5.38–5.32 (m, 1 H, CH=C), 5.14 (s, 2 H, OCH$_2$), 4.84 (d, *J* = 9.5 Hz, 2 H, 2 × NCH*H*), 4.55 (dd, *J* = 9.5, 3.0 Hz, 2 H, 2 × NC*H*H), 4.36 (dt, *J* = 11.5, 6.2 Hz, 1 H, OCH), 3.88 (s, 9 H, 3 × OCH$_3$), 2.44 (t, *J* = 13.2 Hz, 1 H, CH), 2.34 (dd, *J* = 13.7, 4.9 Hz, 1 H, CH), 2.02 (dt, *J* = 13.3, 3.6 Hz, 2 H, 2 × CH), 1.97–1.78 (m, 4 H, 2 × CH$_2$), 1.67–1.06 (m, 20 H, 20 × CH), 0.99 (s, 3 H, CH$_3$), 0.93 (d, *J* = 6.4 Hz, 3 H, CH$_3$), 0.88 (dd, *J* = 6.6, 1.8 Hz, 6 H, 2 × CH$_3$), 0.68 (s, 3 H, CH$_3$); ^{13}C NMR (101 MHz, CDCl$_3$) δ 155.8 (C=O), 153.1 (2 × Ar-*C*$_q$OCH$_3$), 138.9 (Ar-*C*$_q$OCH$_3$) , 138.4 (CH=*C*), 136.0 (Ar-*C*$_q$C$_q$), 129.0 (Ar-*C*$_q$CH$_2$), 128.6 (2 × Ar-CH), 128.3 (Ar-CH), 128.2 (2 × Ar-CH), 124.0 (CH=*C*), 106.2 (2 × Ar-CH), 84.4 (OCH), 67.3 (CH$_2$), 62.6 (C$_q$SO$_2$), 61.0 (OCH$_3$), 58.1 (NCH$_2$), 57.3 (NCH$_2$), 56.6 (CH), 56.4 (2 × OCH$_3$), 56.1 (CH), 49.8 (CH), 42.3 (*C*$_q$CH$_3$), 39.6 (*C*$_q$CH$_2$), 39.5 (CH*C*H$_2$CH$_2$), 39.1 (OCH*C*H$_2$), 36.8 (CH$_2$*C*H$_2$), 36.3 (*C*H$_2$CH$_2$), 36.2 (CH*C*H$_2$CH$_2$), 35.8 (*C*HCH$_3$), 31.9 (*C*$_q$CH$_3$), 31.7 (OCH*C*H$_2$), 28.2 (CH*C*H$_2$CH$_2$), 28.0 (*C*H(CH$_3$)$_2$), 24.3 (CH*C*H$_2$CH$_2$), 23.8 (CH$_2$*C*H$_2$CH$_2$), 22.8 (*C*H$_3$CH), 22.6 (*C*H$_3$CH), 21.0 (CH$_2$), 19.2 (*C*H$_3$CH), 18.7 (*C*H$_3$C$_q$), 11.8 (CH$_3$); HRMS (TOF-MS-ES$^+$) *m/z* calcd for $C_{47}H_{68}NO_8S^+$ [M+H]$^+$: 806.4666, found: 806.4681.

Automated Column Conditions: Run on a Biotage® Selekt system. Column type: Biotage® Sfär® HC Duo 10 g. Flow rate: 40 mL/min. Sample mass: 200 mg. Solvent A: *n*-hexane, Solvent B: Et$_2$O. UV wavelength detection: l 200–400 nm. See trace and gradient below. From the left: peak 1 (yellow): azetidine fluoride, peak 2 (red): azetidine sulfonyl fluoride **15**, peak 3 (yellow): cholesterol, peak 4 (red to blue): azetidine sulfinate ester **130**.

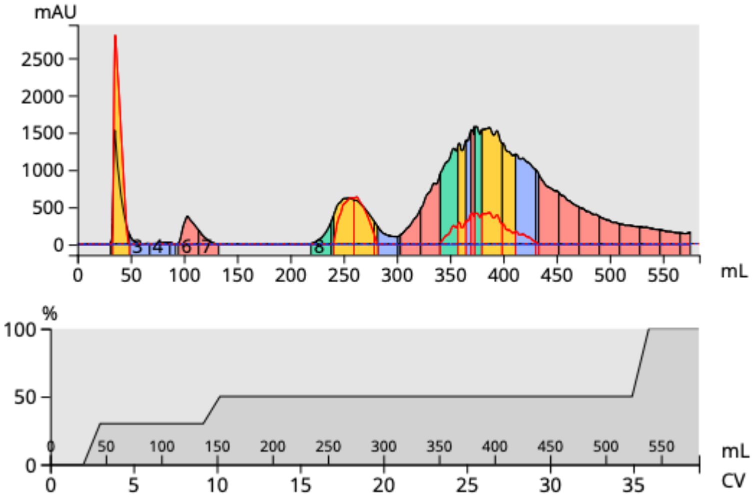
mAU
2500
2000
1500
1000
500
0
0 50 100 150 200 250 300 350 400 450 500 550
mL
3 4 6 7
8
%
100
50
0
0 50 100 150 200 250 300 350 400 450 500 550
mL
0 5 10 15 20 25 30 35
CV

Linker and Molecular Glue-type deFS products (156–162)

4-((3-(Benzo[*d*][1,3]dioxol-5-yl)oxetan-3-yl)amino)-2-(2,6-dioxopiperidin-3-yl)isoindoline-1,3-dione (156)

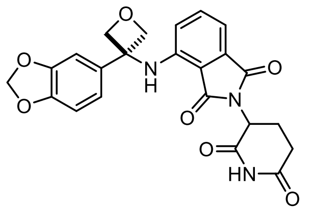

Prepared according to **deFS General Procedure C** under Ar at 80 °C for 4 h with a Celite work-up and using oxetane sulfonyl fluoride **7** (176 mg, 0.68 mmol, 3.4 equiv), K_2CO_3 (88.1 mg, 0.64 mmol, 3.2 equiv), pomalidomide (54.6 mg, 0.20 mmol, 1.0 equiv) and anhydrous MeCN (4.0 mL, 0.05 M). The reaction mixture was cooled to 0 °C, diluted with EtOAc (5 mL) and filtered through a plug of Celite, eluting with further chilled (0 °C) EtOAc (3 × 5 mL). The filtrate solvent was removed *in vacuo* using a rotary evaporator. Purification by flash column chromatography (50% EtOAc/pentane) afforded amino-oxetane **156** as a yellow solid (69.1 mg, 76%). R_f = 0.19 (50% EtOAc/pentane); mp = 220–221 °C; IR (film)/cm^{-1} 1697 (C=O st), 1504, 1485, 1405, 1359, 1258, 1178, 1036; ^{1}H NMR (400 MHz, $CDCl_3$) δ 8.26 (s, 1 H, CONH), 7.31 (dd, *J* = 8.4, 7.2 Hz, 1 H, Ar-CH), 7.19–7.10 (m, 2 H, 2 × Ar-CH), 7.10–7.03 (m, 2 H, Ar-CH and C_qNH), 6.81 (d, *J* = 8.2 Hz, 1 H, Ar-CH), 6.08 (d, *J* = 8.4 Hz, 1 H, Ar-CH), 5.98 (s, 2 H, $CH_2(O)_2$Ar), 5.01–4.91 (m, 5 H, CH_2OCH_2 and NCHCO), 3.00–2.69 (m, 3 H, NCHC*H*$_2$CHH and NCHCH$_2$C*H*H), 2.24–2.12 (m, 1 H, NCHCH$_2$CH*H*); ^{13}C NMR (101 MHz, $CDCl_3$) δ 171.1 (CH*C*ONH), 169.6 (CH$_2$*C*ONH), 168.4 (N*C*OAr), 167.4 (N*C*OAr), 148.7 (Ar-*C*$_q$-OCH$_2$O), 147.5 (Ar-*C*$_q$-OCH$_2$O), 143.9 (Ar-*C*$_q$-NH), 136.0 (Ar-*C*$_q$-C$_q$), 134.6, (Ar-CH) 132.8 (Ar-*C*$_q$-CO), 118.7 (Ar-*C*$_q$-CO), 118.4 (Ar-CH), 113.0 (Ar-CH), 111.7 (Ar-CH), 108.6 (Ar-CH), 105.9 (Ar-CH), 101.6 ($CH_2(O)_2$Ar), 83.9 (*C*H$_2$OCH$_2$), 83.8 (CH$_2$O*C*H$_2$), 59.9 (C$_q$), 49.2 (N*C*HCH$_2$), 31.6 (NCH*C*H$_2$), 22.9 (NHCO*C*H$_2$); HRMS (FTMS–ES $^-$) *m/z* calcd for $C_{23}H_{18}N_3O_7{}^-$ [M−H] $^-$: 448.1150, found: 448.115.

Notes:

156 *strongly fluoresces in CH_2Cl_2, $CDCl_3$ and EtOAc solution with irradiation from daylight.*

To provide a preliminary assessment of stability of these product types in buffer solution, a solution of ***156*** *was examined using LCMS: 0.2 mg of* ***156*** *was dissolved into 0.33 mL of anhydrous DMSO (1.4 µM). 100 µL of this solution was added to a HPLC vial along with 20 µL of 21 µM benzoic acid solution in anhydrous DMSO and 890 µL of aqueous ammonium carbonate buffer (50 mM, pH 7.8). This sample was then monitored by LCMS to assess the aqueous stability of the compound, using the benzoic acid as a reference. t = 0 was measured 1 hour after sample preparation. After t = 2.8 days, the ratio of absorbance between the benzoic acid standard and the peak corresponding to the* ***156*** *was the within error of the observed ratio at t = 0 and there was no evidence of additional peaks that would correspond to a hydrolysis product.*

Benzyl 3-(benzo[*d*][1,3]dioxol-5-yl)-3-((2-(2,6-dioxopiperidin-3-yl)-1,3-dioxoisoindolin-4-yl)amino)azetidine-1-carboxylate (157)

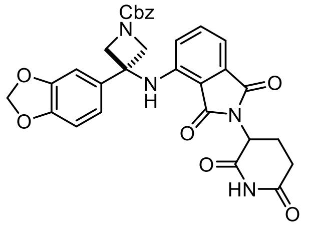

Prepared according to **deFS General Procedure C** under Ar at 80 °C for 4 h with a Celite work-up and using azetidine sulfonyl fluoride **14** (119 mg, 0.30 mmol, 3.0 equiv), K_2CO_3 (49.1 mg, 0.36 mmol, 3.6 equiv), pomalidomide (27.4 mg, 0.10 mmol, 1.0 equiv) and anhydrous MeCN (2.0 mL, 0.05 M). The reaction mixture was cooled to 0 °C, diluted with EtOAc (5 mL) and filtered through a plug of Celite, eluting with further chilled (0 °C) EtOAc (3 × 5 mL). The filtrate solvent was removed *in vacuo* using a rotary evaporator. Purification by flash column chromatography (50% EtOAc/pentane) afforded amino-azetidine **157** as a yellow solid (49.1 mg, 85%). R_f = 0.26 (50% EtOAc/pentane); mp = 176–176 °C; IR (film)/cm^{-1} 2359, 1698 (C=O st), 1621 (C=O st), 1258, 1405, 1359, 1197, 1178, 747; ^{1}H NMR (400 MHz, $CDCl_3$) δ 8.62 (s, 1 H, CONH), 7.37–7.27 (m, 6 H, 6 × Ar-CH), 7.13 (d, *J* = 7.2 Hz, 1 H, Ar-CH), 7.02 (s, 1 H, C_qNH), 7.00–6.91 (m, 2 H, 2 × Ar-CH), 6.76 (d, *J* = 8.1 Hz, 1 H, Ar-CH), 6.16 (d, *J*

= 8.4 Hz, 1 H, Ar-CH), 5.95 (s, 2 H, C*H*$_2$(O)$_2$Ar), 5.13 (s, 2 H, PhCH$_2$), 4.96 (dd, *J* = 12.0, 5.3 Hz, 1 H, NCHCO), 4.37 (d, *J* = 9.0 Hz, 2 H, C*H*$_2$NCH$_2$), 4.28 (d, *J* = 9.0 Hz, 2 H, CH$_2$NC*H*$_2$), 2.95–2.65 (m, 3 H, NCHC*H*$_2$CHH and NCHCH$_2$C*H*H), 2.19–2.06 (m, 1 H, NCHC*H*$_2$CH*H*); ^{13}C NMR (101 MHz, CDCl$_3$) δ 171.4 (CH*C*ONH), 169.5 (CH$_2$*C*ONH), 168.6 (NCOAr), 167.4 (NCOAr), 156.4 (N*C*O$_2$CH$_2$Ph), 148.7 (Ar-*C*$_q$-OCH$_2$O), 147.4 (Ar-*C*$_q$-OCH$_2$O), 143.6 (Ar-*C*$_q$-NH), 136.3 (Ar-*C*$_q$CH$_2$), 135.8 (Ar-*C*$_q$-C$_q$), 135.0, (Ar-CH) 132.7 (Ar-*C*$_q$-CO), 128.6 (2 × Ar$_{(Cbz)}$-CH), 128.3 (Ar$_{(Cbz)}$-CH), 128.2 (2 × Ar$_{(Cbz)}$-CH), 118.6 (Ar-*C*$_q$-CO), 113.1 (Ar-CH), 112.0 (Ar-CH), 108.6 (Ar-CH), 105.8 (Ar-CH), 101.5 (CH$_2$(O)$_2$Ar), 67.3 (C$_q$), 63.3 (br, (CH$_2$)$_2$N), 55.2, (PhCH$_2$), 49.1(N*C*HCH$_2$), 31.5 (NCH*C*H$_2$), 22.9 (NHCO*C*H$_2$); HRMS (TOF-MS-ES$^+$) *m/z* calcd for $C_{31}H_{27}N_4O_8^+$ [M+H]$^+$: 583.1829, found: 583.1855.

Notes:

***157** strongly fluoresces in CH$_2$Cl$_2$, CDCl$_3$ and EtOAc solution with irradiation from daylight.*

2-(2,6-Dioxopiperidin-3-yl)-4-((3-(4-(prop-2-yn-1-yloxy)phenyl)oxetan-3-yl)amino)isoindoline-1,3-dione (158)

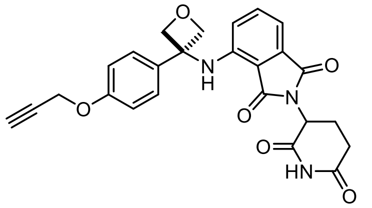

Prepared according to **deFS General Procedure C** under Ar at 80 °C for 20 h with a Celite work-up and using oxetane sulfonyl fluoride **134** (91.7 mg, 0.34 mmol, 3.4 equiv), K$_2$CO$_3$ (45.5 mg, 0.33 mmol, 3.3 equiv), pomalidomide (27.4 mg, 0.10 mmol, 1.0 equiv) and anhydrous MeCN (2.0 mL, 0.05 M). The reaction mixture was cooled to 0 °C, diluted with EtOAc (5 mL) and filtered through a plug of Celite, eluting with further chilled (0 °C) EtOAc (3 × 5 mL). The filtrate solvent was removed *in vacuo* using a rotary evaporator. Purification by flash column chromatography (50% EtOAc/pentane) afforded amino-oxetane **158** as a yellow solid (31.1 mg, 68%). R$_f$ = 0.20 (50% EtOAc/pentane); mp = 131–132 °C; IR (film)/cm^{-1} 3280, 2956, 1698, 1621, 1508, 1359, 1260, 1176, 1027, 747; ^{1}H NMR (400 MHz, CDCl$_3$) δ 8.16 (s, 1 H, CON*H*), 7.61–7.51 (m, 2 H, 2 × Ar-CH), 7.30 (t, *J* = 7.8 Hz, 1 H, Ar-CH), 7.14 (d, *J* = 7.1 Hz, 1 H, Ar-CH), 7.08 (s, 1 H, C$_q$NH), 7.05–6.97 (m, 2 H, 2 × Ar-CH), 6.05 (d, *J* = 8.4 Hz, 1 H, Ar-CH), 5.03–4.92 (m, 5 H, CH$_2$OCH$_2$ and NCHCO), 4.70 (d, *J* = 2.4 Hz, 2 H, C*H*$_2$CCH), 2.98–2.70 (m, 3 H, NCHC*H*$_2$CHH and NCHCH$_2$C*H*H), 2.53 (t, *J* = 2.4 Hz, 1 H, CH$_2$CC*H*), 2.24–2.13 (m, 1 H, NCHC*H*$_2$CH*H*); ^{13}C NMR (101 MHz, CDCl$_3$) δ 171.2 (CH*C*ONH), 169.6 (CH$_2$*C*ONH), 168.4 (N*C*OAr), 167.5 (N*C*OAr), 157.4 (Ar-*C*$_q$-OCH$_2$), 144.0 (Ar-*C*$_q$-NH), 136.0 (Ar-*C*$_q$-C$_q$), 133.5, (Ar-CH) 132.9 (Ar-*C*$_q$-CO), 126.6 (2 × Ar-CH), 118.5 (Ar-*C*$_q$-CO), 115.5 (2 × Ar-CH), 113.0 (Ar-CH), 111.7 (Ar-CH), 84.0 (CH$_2$*O*CH$_2$), 78.4 (CH$_2$*C*CH), 75.9 (CH$_2$C*C*H), 59.7 (C$_q$), 56.0 (*C*H$_2$CCH), 49.2 (N*C*HCH$_2$), 31.6 (NCH*C*H$_2$), 23.0 (NHCO*C*H$_2$); HRMS (TOF-MS-ES$^+$) *m/z* calcd for $C_{25}H_{22}N_3O_6^+$ [M+H]$^+$: 460.1509, found: 460.1509.

Notes:

***158** strongly fluoresces in CH$_2$Cl$_2$, CDCl$_3$ and EtOAc solution with irradiation from daylight.*

Ethyl 2-(4-(3-((2-(2,6-dioxopiperidin-3-yl)-1,3-dioxoisoindolin-4-yl)amino)oxetan-3-yl)phenoxy)acetate (159)

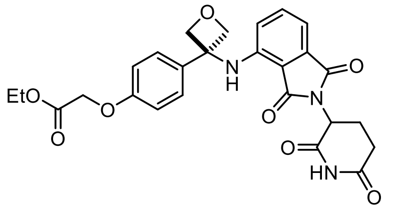

Prepared according to **deFS General Procedure C** under Ar at 80 °C for 18 h with a Celite work-up and using oxetane sulfonyl fluoride **136** (88.6 mg, 0.28 mmol, 2.8 equiv), K$_2$CO$_3$ (47.1 mg, 0.34 mmol, 3.4 equiv), pomalidomide (27.4 mg, 0.10 mmol, 1.0 equiv) and anhydrous MeCN (2.0 mL, 0.05 M). The reaction mixture was cooled to 0 °C, diluted with EtOAc (5 mL) and filtered through a plug of Celite, eluting with further chilled (0 °C) EtOAc (3 × 5 mL). The filtrate solvent was removed *in vacuo* using a rotary evaporator. Purification by flash column chromatography (60% EtOAc/pentane) afforded amino-oxetane **159** as a yellow solid (44.8 mg, 88%). *R*$_f$ = 0.15 (50% EtOAc/pentane); mp = 136–137 °C; IR (film)/cm^{-1} 2956, 2874, 1757 (C=O st), 1698 (C=O st), 1621, 1511, 1405, 1200, 1180, 747; ^{1}H NMR (400 MHz, CDCl$_3$) δ 8.41 (s, 1 H, CONH), 7.57–7.50 (m, 2 H,

2 × Ar-CH), 7.32–7.26 (m, 1 H, Ar-CH), 7.13 (d, *J* = 7.1 Hz, 1 H, Ar-CH), 7.08 (s, 1 H, C_qNH), 6.95–6.89 (m, 2 H, 2 × Ar-CH), 6.03 (d, *J* = 8.4 Hz, 1 H, Ar-CH), 4.96 (d, *J* = 2.5 Hz, 5 H, CH_2OCH_2 and NCHCO), 4.62 (s, 2 H, OCH_2CO_2Et), 4.26 (q, *J* = 7.1 Hz, 2 H, $COCH_2CH_3$), 2.97–2.70 (m, 3 H, NCHCH_2CHH and $NCHCH_2C$*H*H), 2.22–2.11 (m, 1 H, NCHCH_2CH*H*), 1.29 (t, *J* = 7.1 Hz, 3 H, $COCH_2CH_3$); ^{13}C NMR (101 MHz, $CDCl_3$) δ 171.2 (CH*C*ONH), 169.6 (CH_2*C*ONH), 168.9 (NCOAr), 168.5 (CO_2Et), 167.4 (NCOAr), 157.6 (Ar-C_q-OCH_2), 144.0 (Ar-C_q-NH), 135.9 (Ar-C_q-C_q), 133.6, (Ar-CH) 132.8 (Ar-C_q-CO), 126.6 (2 × Ar-CH), 118.5 (Ar-C_q-CO), 115.2 (2 × Ar-CH), 112.9 (Ar-CH), 111.7 (Ar-CH), 84.0 (CH_2OCH_2), 83.9 (CH_2OCH_2), 65.4 (CH_2CO_2Et), 61.6 ($CO_2CH_2CH_3$), 59.6 (C_q), 49.1 (N*C*HCH$_2$), 31.5 ($NCHCH_2$), 22.9 ($NHCOCH_2$), 14.3 ($COCH_2CH_3$); HRMS (TOF-MS-ES$^+$) *m/z* calcd for $C_{26}H_{29}N_4O_8^+$ $[M+NH_4]^+$: 525.1985, found: 525.1985.
Notes:

***159** strongly fluoresces in CH_2Cl_2, $CDCl_3$ and EtOAc solution with irradiation from daylight.*

Prop-2-yn-1-yl 3-((2-(2,6-dioxopiperidin-3-yl)-1,3-dioxoisoindolin-4-yl)amino)-3-(4-methoxyphenyl)azetidine-1-carboxylate (160)

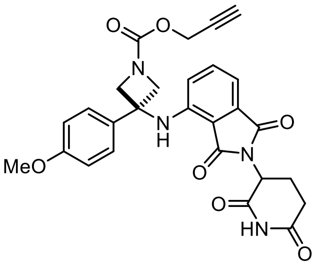

A microwave vial was charged with K_2CO_3 (42.0 mg, 0.30 mmol, 3.0 equiv.) and flame-dried under Ar. Pomalidomide (27.5 mg, 0.10 mmol, 1.0 equiv) and anhydrous MeCN (2.00 mL, 0.05 M) were added to the microwave vial and the resulting mixture was stirred at 70 °C for 5 mins before being cooled to room temperature. Azetidine sulfonyl fluoride **152** (56.9 mg, 0.17 mmol, 1.7 equiv.) was added to the reaction mixture in one portion. Once the addition was complete, the reaction mixture was warmed to 40 °C. After 1.5 h, the reaction mixture was heated to 60 °C. After 1 h at 60 °C the reaction mixture was cooled to 0 °C, diluted with EtOAc (5 mL) and filtered through a plug of Celite, further eluting with chilled (0 °C) EtOAc (3 x 5 mL). The solvent was removed from the filtrate *in vacuo* using a rotary evaporator. Purification by flash column chromatography (50% EtOAc/pentane) afforded **160** as a yellow solid (35.9 mg, 69%). R_f = 0.21 (50% EtOAc/pentane); mp = 139 °C; IR (film)/cm^{-1} 3375, 3267 (≡C-H st), 2955, 1694 (C=O st), 1405, 1252, 1116, 1026, 730; ^1H NMR (400 MHz, $CDCl_3$) δ 8.50 (s, 1H, CON*H*), 7.39 (d, *J* = 8.5 Hz, 2 H, 2 × Ar-CH), 7.29 (t, *J* = 7.9 Hz, 1 H, Ar-CH), 7.14 (d, *J* = 7.2 Hz, 1 H, Ar-CH), 7.03 (s, 1 H, C_qNH), 6.89 (d, *J* = 8.6 Hz, 2 H, 2 × Ar-CH), 6.15 (d, *J* = 8.4 Hz, 1 H, Ar-CH), 5.00–4.93 (m, 1 H, NC*H*CO), 4.71 (d, *J* = 2.5 Hz, 2 H, CH_2CCH), 4.41 (d, *J* = 9.0 Hz, 2 H, CH_2NCH_2), 4.31 (d, *J* = 9.1 Hz, 2 H, CH_2NCH_2), 3.79 (s, 3 H, Ar-OCH_3), 2.99–2.70 (m, 3 H, $NCHCH_2CH_2$ + $NCHCH_2C$*H*H), 2.49 (t, *J* = 2.5 Hz, 1 H, CH_2CCH), 2.23–2.11 (m, 1 H, $NCHCH_2CH$*H*); ^{13}C NMR (101 MHz, $CDCl_3$) δ 171.3 (CH*C*ONH), 169.6 (CH_2*C*ONH), 168.5 (NCOAr), 167.4 (NCOAr), 159.4 (N*C*O_2CH_2CCH), 155.4 (Ar-C_q-OCH_3), 143.7 (Ar-CH), 135.9 (Ar-C_q-C_q), 132.7, (Ar-C_q-CO) 126.5 (2 × Ar-CH), 118.7 (Ar-C_q-CO), 114.5 (2 × Ar-CH), 113.1 (Ar-CH), 111.9 (Ar-CH), 78.1 (CH_2CCH), 75.1 (CH_2CCH), 55.4 (C_q), 55.0 (CH_2CCH), 53.1 (CH_2NCH_2), 49.1 (N*C*HCH_2), 31.5 ($NCHCH_2$), 22.9 ($NHCOCH_2$); HRMS (TOF-MS-ES$^+$) *m/z* calcd for $C_{27}H_{28}N_5O_7Na^+$ $[M+Na]^+$: 539.1543, found: 539.1536.

Notes:

***160** strongly fluoresces in CH_2Cl_2, $CDCl_3$ and EtOAc solution with irradiation from daylight.*

*To provide a preliminary assessment of stability in buffer solution a solution of **160** was examined using LCMS: 0.7 mg of **160** was dissolved into 1.00 mL of anhydrous DMSO (1.4 μM). 100 μL of this solution was added to a HPLC vial along with 8.3 μL of 27 μM benzoic acid solution in anhydrous DMSO and 892 μL of aqueous ammonium carbonate buffer (50 mM, pH 7.8). This sample was then monitored by LCMS to assess the aqueous stability of the compound, using the benzoic acid as a reference. t = 0 was measured 4 hours after sample preparation. After t = 0.9 days, the ratio of absorbance between the benzoic acid standard and the peak corresponding to the **160** was 81% of the observed ratio at t = 0. The main degradation product observed had a mass of +18 compared to **160**, consistent with hydrolysis of the glutarimide ring.*[38]

3-((3-(Benzo[*d*][1,3]dioxol-5-yl)oxetan-3-yl)amino)piperidine-2,6-dione (161)

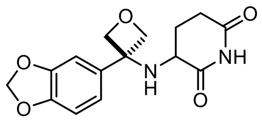

A microwave vial was flame-dried under Ar and charged with 3-aminopiperidine-2,6-dione hydrochloride (16.5 mg, 0.1 mmol, 1.0 equiv), triethylamine (28 µL, 0.2 mmol, 2.0 equiv) and anhydrous MeCN (2 mL, 0.05 M). The reaction mixture was stirred at 80 °C for 10 min, followed by addition of oxetane sulfonyl fluoride **7** (39 mg, 0.15 mmol, 1.50 equiv). The microwave vial was sealed and the reaction was stirred at 80 °C for 2 h. The reaction was cooled to room temperature and concentrated *in vacuo* using a rotary evaporator. Purification by flash column chromatography (10% MeOH/CH_2Cl_2) afforded amino-oxetane **161** as a pale blue gum (15.5 mg, 51%). R_f = 0.26 (10% MeOH/CH_2Cl_2); IR (film)/cm^{-1} 3299, 2958, 2878, 2361, 1701 (C=O st), 1485, 1435, 1353, 1191, 1232, 1034, 910, 729; ^{1}H NMR (400 MHz, $CDCl_3$) δ 8.00 (s, 1 H, CONH), 6.83 (m, 3 H, 3 × Ar-CH), 6.00 (d, *J* = 1.7 Hz, 2 H, OCH_2O), 5.07 (d, *J* = 6.5 Hz, 1 H, C*H*HOCHH), 4.89 (d, *J* = 6.4 Hz, 1 H, CH*H*OCHH), 4.78 (d, *J* = 6.5 Hz, 1 H, CHHOC*H*H), 4.73 (d, *J* = 6.4 Hz, 1 H, CHHOCH*H*), 3.23 (dd, *J* = 11.5, 5.6 Hz, 1 H, C_q-NH-C*H*), 3.08 (s, 1 H, C_q-N*H*-CH), 2.65 (m, 1 H, $NCHCH_2C$*H*H), 2.39 (m, 1 H, $NCHCH_2CH$*H*), 1.82–1.65 (m, 2 H, NCHCH_2CH$_2$); ^{13}C NMR (101 MHz, $CDCl_3$) δ 174.1 (CH*C*ONH), 171.5 (CH_2*C*ONH), 148.3 (Ar-C_q-OCH_2), 147.2 (Ar-C_q-OCH_2), 135.7 (Ar-C_q-C_q), 119.8 (Ar-CH), 108.2 (Ar-CH), 107.1 (Ar-CH), 101.3 (OCH_2O), 83.4 (*C*H_2OCH_2), 82.1 (CH_2O*C*H_2), 63.3 (C_q), 54.4 (N*C*HCH_2), 31.1 (NCH*C*H_2), 26.9 (NHCO*C*H_2); HRMS (FTMS–ES$^+$) *m/z* calcd for $C_{15}H_{15}N_2O_5^-$ [M–H]$^-$: 303.0986, found: 303.0990.

Notes:

Triethylamine is used as a base rather than K_2CO_3 to facilitate solubility of 3-aminopiperidine-2,6-dione hydrochloride.

3-((3-(4-Hydroxyphenyl)oxetan-3-yl)amino)piperidine-2,6-dione (162)

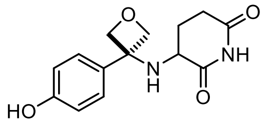

A microwave vial was flame-dried under Ar and charged with 3-aminopiperidine-2,6-dione hydrochloride (16.5 mg, 0.1 mmol, 1.0 equiv), triethylamine (28.0 µL, 0.2 mmol, 2.0 equiv) and anhydrous MeCN (2.0 mL, 0.05 M). The reaction mixture was stirred at 80 °C for 10 min, followed by addition of oxetane sulfonyl fluoride **2** (58.3 mg, 0.15 mmol, 1.5 equiv). The microwave vial was sealed and the reaction was stirred at 80 °C for 2 h. The reaction was cooled to room temperature and concentrated *in vacuo* using a rotary evaporator. Purification by flash column chromatography (10% MeOH/CH_2Cl_2) afforded amino-oxetane **162** as a white solid (20.7 mg, 75%). R_f = 0.20 (10% MeOH/CH_2Cl_2); mp = 187–190 °C; IR (film)/cm^{-1} 3215 (br OH st), 2924, 2853, 1700 (C=O st), 1612, 1517, 1359, 1245, 1193, 972, 837; ^{1}H NMR (400 MHz, $(CD_3)_2CO$ with TMS) δ 9.56 (s, 1 H, CONH), 8.44 (s, 1 H, OH), 7.32 (d, *J* = 8.6 Hz, 2 H, 2 × Ar-CH), 6.88 (d, *J* = 8.6 Hz, 2 H, 2 × Ar-CH), 4.99 (d, *J* = 6.3 Hz, 1 H, C*H*HOCH_2), 4.82–4.70 (m, 3 H, CH*H*OCH_2 and CH_2OC*H_2*), 3.34–3.26 (m, 2 H, C_q-N*H*-CH and C_q-NH-C*H*), 2.57–2.40 (m, 2 H, $NCHCH_2C$*H_2*), 1.74–1.61 (m, 2 H, NCHC*H_2*CH_2); ^{13}C NMR (101 MHz, $(CD_3)_2CO$ with TMS) δ 174.9 (CH*C*ONH), 171.9 (CH_2*C*ONH), 156.6 (Ar-C_q-OH), 133.8 (Ar-C_q-C_q), 128.0 (2 × Ar-CH), 115.2 (2 × Ar-CH), 83.2 (*C*H_2OCH_2), 81.8 (CH_2O*C*H_2), 62.7 (C_q), 53.9 (N*C*HCH_2), 30.5 (NCH*C*H_2), 26.6 (NHCO*C*H_2); HRMS (FTMS–ES$^+$) *m/z* calcd for $C_{14}H_{15}N_2O_4^-$ [M–H]$^-$: 275.1037, found: 275.1039.

Notes:

Triethylamine is used as a base rather than K_2CO_3 to facilitate solubility of 3-aminopiperidine-2,6-dione hydrochloride.

References

(1) Love, B. E.; Jones, E. G. The Use of Salicylaldehyde Phenylhydrazone as an Indicator for the Titration of Organometallic Reagents. *J. Org. Chem.* **1999**, *64* (10), 3755–3756.

(2) (a) Zenzola, M.; Doran, R.; Luisi, R.; Bull, J. A. Synthesis of Sulfoximine Carbamates by Rhodium-Catalyzed Nitrene Transfer of Carbamates to Sulfoxides. *J. Org. Chem.* **2015**, *80* (12), 6391–6399; (b) Zenzola, M.; Doran, R.; Degennaro, L.; Luisi, R.; Bull, J. A. Transfer of Electrophilic NH Using Convenient Sources of Ammonia: Direct Synthesis of NH Sulfoximines from Sulfoxides. *Angew. Chem., Int. Ed.* **2016**, *55* (25), 7203–7207; (c) Tota, A.; Zenzola, M.; Chawner, S. J.; John-Campbell, S. S.; Carlucci, C.; Romanazzi, G.; Degennaro, L.; Bull, J. A.; Luisi, R. Synthesis of NH-Sulfoximines from Sulfides by Chemoselective One-Pot N- and O-Transfers. *Chem. Commun.* **2017**, *53* (2), 348–351; (d) Briggs, E. L.; Tota, A.; Colella, M.; Degennaro, L.; Luisi, R.; Bull, J. A. Synthesis of Sulfonimidamides from Sulfenamides via an Alkoxy-Amino-Λ6-Sulfanenitrile Intermediate. *Angew. Chem., Int. Ed.* **2019**, *58* (40), 14303–14310.

(3) Jamir, L.; Ali, A. R.; Ghosh, H.; Chipem, F. A. S.; Patel, B. K. The Thiocarbonyl "S" Is Softer than Thiolate "S": A Catalyst-Free One-Pot Synthesis of

(4) Galli, C. "Cesium Ion Effect" and Macrocyclization. A Critical Review. *Org. Prep. Proced. Int.* **1992**, *24* (3), 285–307.

(5) Bordwell, F. G. Equilibrium Acidities in Dimethyl Sulfoxide Solution. *Acc. Chem. Res.* **1988**, *21* (12), 456–463.

(6) Rojas, J. J.; Croft, R. A.; Sterling, A. J.; Briggs, E. L.; Antermite, D.; Schmitt, D. C.; Blagojevic, L.; Haycock, P.; White, A. J. P.; Duarte, F.; Choi, C.; Mousseau, J. J.; Bull, J. A. Amino-Oxetanes as Amide Isosteres by an Alternative Defluorosulfonylative Coupling of Sulfonyl Fluorides. *Nat. Chem.* **2022**, *14* (2), 160–169.

(7) R Core Team. *R: A language and environment for statistical computing*, version 3.2.2; R Foundation for Statistical Computing: Vienna, Austria, **2015**.

(8) Burés, J. Variable Time Normalization Analysis: General Graphical Elucidation of Reaction Orders from Concentration Profiles. *Angew. Chem., Int. Ed.* **2016**, *55* (52), 16084–16087.

(9) Press, W. H.; Flannery, B. P.; Teukolsky, S. A.; Vetterling, W. T. In *Numerical Recipes in Pascal, The Art of Scientific Computing*; Cambridge University Press, Cambridge, **1989**.

(10) Croft, R. A.; Mousseau, J. J.; Choi, C.; Bull, J. A. Structurally Divergent Lithium Catalyzed Friedel–Crafts Reactions on Oxetan-3-ols: Synthesis of 3,3-Diaryloxetanes and 2,3-Dihydrobenzofurans. *Chem. Eur. J.* **2016**, *22*, 16271–16276.

(11) Denis, C.; Dubois, M. A. J.; Voisin-Chiret, A. S.; Bureau, R.; Choi, C.; Mousseau, J. J.; Bull, J. A. Synthesis of 3,3-Diarylazetidines by Calcium(II)-Catalyzed Friedel-Crafts Reaction of Azetidinols with Unexpected Cbz Enhanced Reactivity. *Org. Lett.* **2019**, *21*, 300–304.

(12) Croft, R. A.; Dubois, M. A. J.; Boddy, A. J.; Denis, C.; Lazaridou, A.; Voisin-Chiret, A. S.; Bureau, R.; Choi, C.; Mousseau, J. J.; Bull, J. A. Catalytic Friedel-Crafts Reactions on Saturated Heterocycles and Small Rings for sp^3-sp^2 Coupling of Medicinally Relevant Fragments. *Eur. J. Org. Chem.* **2019**, *2019*, 5385–5395.

(13) Croft, R. A.; Mousseau, J. J.; Choi, C.; Bull, J. A. Lithium-Catalyzed Thiol Alkylation with Tertiary and Secondary Alcohols: Synthesis of 3-Sulfanyl-Oxetanes as Bioisosteres. *Chem. Eur. J.* **2018**, *24* (4), 818–821.

(14) Dubois, M. A. J.; Lazaridou, A.; Choi, C.; Mousseau, J. J.; Bull, J. A. Synthesis of 3-Aryl-3-Sulfanyl Azetidines by Iron-Catalyzed Thiol Alkylation with N-Cbz Azetidinols. *J. Org. Chem.* **2019**, *84*, 5943–5956.

(15) Saejong, P.; Rojas, J. J.; Denis, C.; White, A. J. P.; Voisin-Chiret, A. S.; Choi, C.; Bull, J. A. Synthesis of Oxetane and Azetidine Ethers as Ester Isosteres by Brønsted Acid Catalysed Alkylation

of Alcohols with 3-Aryl-Oxetanols and 3-Aryl-Azetidinols. *Org. Biomol. Chem.* **2023**, *21* (27), 5553–5559.

(16) Rojas, J. J.; Torrisi, E.; Dubois, M. A. J.; Hossain, R.; White, A. J. P.; Zappia, G.; Mousseau, J. J.; Choi, C.; Bull, J. A. Oxetan-3-Ols as 1,2-Bis-Electrophiles in a Brønsted-Acid-Catalyzed Synthesis of 1,4-Dioxanes. *Org. Lett.* **2022**, *24* (12), 2365–2370.

(17) (a) Dolomanov, O. V.; Bourhis, L. J.; Gildea, R. J.; Howard, J. A. K.; Puschmann, H. OLEX2: A Complete Structure Solution, Refinement and Analysis Program. *J. Appl. Crystallogr.* **2009**, *42* (2), 339–341. (b) A.L. Spek (2003, 2009) PLATON, A Multipurpose Crystallographic Tool, Utrecht University, Utrecht, The Netherlands. See also A.L. Spek, *Acta. Cryst.*, **2015**, C71, 9–18.

(18) SHELXTL v5.1, Bruker AXS, Madison, WI, 1998.

(19) Sheldrick, G. M. Crystal Structure Refinement with SHELXL. *Acta Crystallogr. Sect. C Struct. Chem.* **2015**, *71* (1), 3–8.

(20) Pan, J.; Wang, X.; Zhang, Y.; Buchwald, S. L. An Improved Palladium-Catalyzed Conversion of Aryl and Vinyl Triflates to Bromides and Chlorides. *Org. Lett.* **2011**, *13*, 4974–4976.

(21) Allen, B. D. W.; Hareram, M. D.; Seastram, A. C.; McBride, T.; Wirth, T.; Browne, D. L.; Morrill, L. C. Manganese-Catalyzed Electrochemical Deconstructive Chlorination of Cycloalkanols via Alkoxy Radicals. *Org. Lett.* **2019**, *21*, 9241–9246.

(22) (a) Mengeste, A. M.; Lund, J.; Katare, P.; Ghobadi, R.; Bakke, H. G.; Lunde, P. K.; Eide, L.; Mahony, G. O.; Göpel, S.; Peng, X. R.; Kase, E. T.; Thoresen, G. H.; Rustan, A. C. The Small Molecule SERCA Activator CDN1163 Increases Energy Metabolism in Human Skeletal Muscle Cells. *Curr. Res. Pharmacol. Drug Discov.* **2021**, *2*, 100060; (b) Nguyen, H. T.; Noriega Polo, C.; Wiederkehr, A.; Wollheim, C. B.; Park, K. S. CDN1163, an Activator of Sarco/Endoplasmic Reticulum Ca^{2+} ATPase, up-Regulates Mitochondrial Functions and Protects against Lipotoxicity in Pancreatic β-Cells. *Br. J. Pharmacol.* **2023**, *180*, 2762–2776.

(23) Michelotti, E. L.; Moffett, K. K.; Nguyen, D.; Kelly, M. J.; Shetty, R.; Chai, X.; Northrop, K.; Namboodiri, V.; Campbell, B.; Flynn, G. A.; Fujimoto, T.; Hollinger, F. P.; Bukhtiyarova, M.; Springman, E. B.; Karpusas, M. Two Classes of P38α MAP Kinase Inhibitors Having a Common Diphenylether Core but Exhibiting Divergent Binding Modes. *Bioorganic Med. Chem. Lett.* **2005**, *15*, 5274–5279.

(24) Yap, J. L.; Cao, X.; Vanommeslaeghe, K.; Jung, K. Y.; Peddaboina, C.; Wilder, P. T.; Nan, A.; MacKerell, A. D.; Smythe, W. R.; Fletcher, S. Relaxation of the Rigid Backbone of an Oligoamide-Foldamer-Based α-Helix Mimetic: Identification of Potent Bcl-x L Inhibitors. *Org. Biomol. Chem.* **2012**, *10*, 2928–2933.

(25) Flack, T.; Romain, C.; White, A. J. P.; Haycock, P. R.; Barnard, A. Design, Synthesis, and Conformational Analysis of Oligobenzanilides as Multifacial α-Helix Mimetics. *Org. Lett.* **2019**, *21*, 4433–4438.

(26) Jang, M. Y.; Lin, Y.; De Jonghe, S.; Gao, L. J.; Vanderhoydonck, B.; Froeyen, M.; Rozenski, J.; Herman, J.; Louat, T.; Van Belle, K.; Waer, M.; Herdewijn, P. Discovery of 7-N-Piperazinylthiazolo[5,4-d] Pyrimidine Analogues as a Novel Class of Immunosuppressive Agents with in Vivo Biological Activity. *J. Med. Chem.* **2011**, *54*, 655–668.

(27) Ramprasad, J.; Nayak, N.; Dalimba, U. Design of New Phenothiazine-Thiadiazole Hybrids via Molecular Hybridization Approach for the Development of Potent Antitubercular Agents. *Eur. J. Med. Chem.* **2015**, *106*, 75–84.

(28) Hui, A. L.; Chen, Y.; Zhu, S. J.; Gan, C. S.; Pan, J.; Zhou, A. Design and Synthesis of Tacrine-Phenothiazine Hybrids as Multitarget Drugs for Alzheimer's Disease. *Med. Chem. Res.* **2014**, *23*, 3546–3557.

(29) Cacchi, S.; Ciattini, P. G.; Morera, E.; Ortar, G. Palladium-Catalyzed Triethylammonium Formate Reduction of Aryl Triflates. A Selective Method for the Deoxygenation of Phenols. *Tetrahedron Lett.* **1986**, *27*, 5541–5544.

(30) Tian, D.; Chen, G.; Wang, X.; Zhang, H. J. Modular Access to Functionalized Oxetanes as Benzoyl Bioisosteres. *J. Am. Chem. Soc.* **2024**, *146*, 18011–18018.

(31) Maiti, D.; Buchwald, S. L. Cu-Catalyzed Arylation of Phenols: Synthesis of Sterically Hindered and Heteroaryl Diaryl Ethers. *J. Org. Chem.* **2010**, *75*, 1791–1794.
(32) Wolfe, J. P.; Tomori, H.; Sadighi, J. P.; Yin, J.; Buchwald, S. L. Simple, Efficient Catalyst System for the Palladium-Catalyzed Amination of Aryl Chlorides, Bromides, and Triflates. *J. Org. Chem.* **2000**, *65*, 1158–1174.
(33) Chen, H.; Volgraf, M.; Do, S.; Kolesnikov, A.; Shore, D. G.; Verma, V. A.; Villemure, E.; Wang, L.; Chen, Y.; Hu, B.; Lu, A. J.; Wu, G.; Xu, X.; Yuen, P. W.; Zhang, Y.; Erickson, S. D.; Dahl, M.; Brotherton-Pleiss, C.; Tay, S.; Ly, J. Q.; Murray, L. J.; Chen, J.; Amm, D.; Lange, W.; Hackos, D. H.; Reese, R. M.; Shields, S. D.; Lyssikatos, J. P.; Safina, B. S.; Estrada, A. A. Discovery of a Potent (4 R,5 S)-4-Fluoro-5-Methylproline Sulfonamide Transient Receptor Potential Ankyrin 1 Antagonist and Its Methylene Phosphate Prodrug Guided by Molecular Modeling. *J. Med. Chem.* **2018**, *61*, 3641–3659.
(34) Emmett, E. J.; Hayter, B. R.; Willis, M. C. Palladium-Catalyzed Three-Component Diaryl Sulfone Synthesis Exploiting the Sulfur Dioxide Surrogate DABSO. *Angew. Chem., Int. Ed.* **2013**, *52*, 12679–12683.
(35) Wrackmeyer, B. Carbon-13 NMR Spectroscopy of Boron Compounds. *Prog. Nucl. Magn. Reson. Spectrosc.* **1979**, *12*, 227–259.
(36) Smit, C.; Blümer, J.; Eerland, M. F.; Albers, M. F.; Müller, M. P.; Goody, R. S.; Itzen, A.; Hedberg, C. Efficient Synthesis and Applications of Peptides Containing Adenylylated Tyrosine Residues. *Angew. Chem., Int. Ed.* **2011**, *50*, 9200–9204.
(37) Ramiya, P. H. OLIGONUCLEOTIDE COMPOSITIONS AND METHODS OF MAKING THE SAME. US Patent WO 2015168310, November 5, 2015.
(38) (a) Ferreira de Melo T. R.; Dulmovits B. M.; dos Santos Fernandes G. F.; de Souza C. M.; Lanaro C.; He M.; Al Abed Y.; Chung M. C.; Blanc L.; Costa F. F.; dos Santos J. L. Synthesis and pharmacological evaluation of pomalidomide derivatives useful for sickle cell disease treatment. *Bioorganic Chemistry* **2021**, *114*, 105077. (b) Hoffmann M.; Kasserra C.; Reyes J.; Schafer P.; Kosek J.; Capone L.; Parton A.; Kim-Kang H.; Surapaneni S.; Kumar G.; Absorption, metabolism and excretion of [^{14}C]pomalidomide in humans following oral administration. *Cancer Chemother. Pharmacol.* **2012** *71*(2), 489–501.
