# Supplementary material for: Harnessing Oxetane and Azetidine Sulfonyl Fluorides for Opportunities in Drug Discovery
Source: J Am Chem Soc. 2024 Dec 12;146(51):35377–89. doi: 10.1021/jacs.4c14164 (PMC11673132; doi:10.1021/jacs.4c14164)
Supplement: Supplementary file 2 — ja4c14164_si_002.pdf [file ja4c14164_si_002.pdf]

^{1}H, ^{13}C, ^{19}F, ^{31}P and ^{11}B NMR Spectra for Selected Compounds

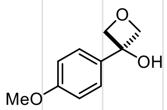

1a

^{1}H NMR (400 MHz), $CDCl_3$

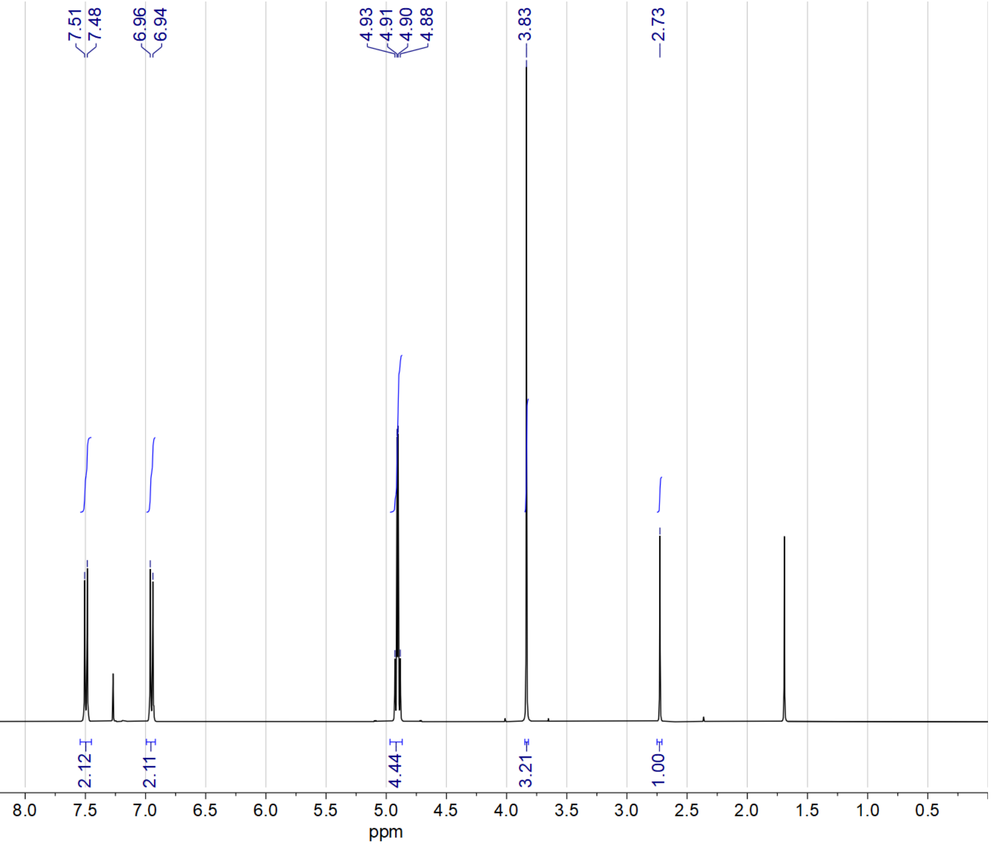

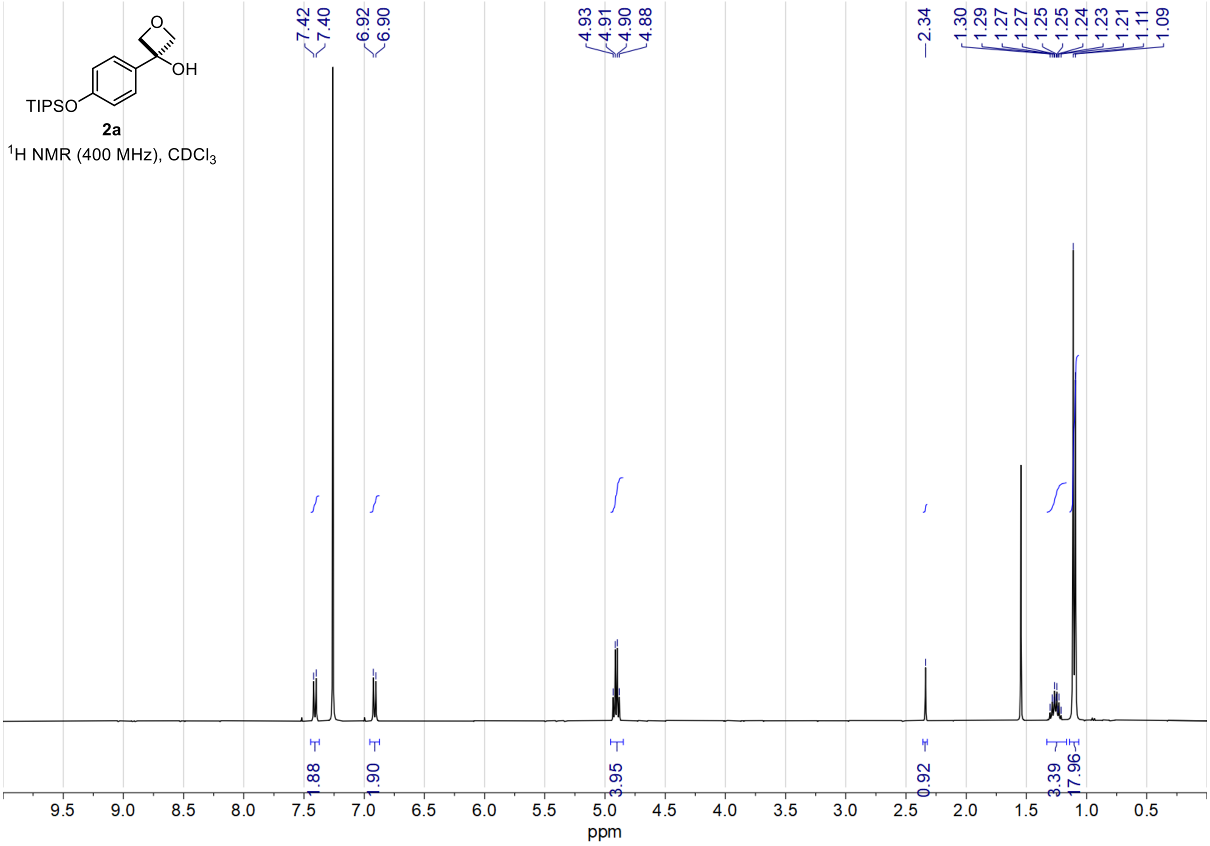
TIPSO
OH
O
2a
1H NMR (400 MHz), CDCl3
7.42
7.40
6.92
6.90
4.93
4.91
4.90
4.88
2.34
1.30
1.29
1.27
1.27
1.25
1.25
1.24
1.23
1.21
1.11
1.09
1.88
1.90
3.95
0.92
3.39
17.96
ppm

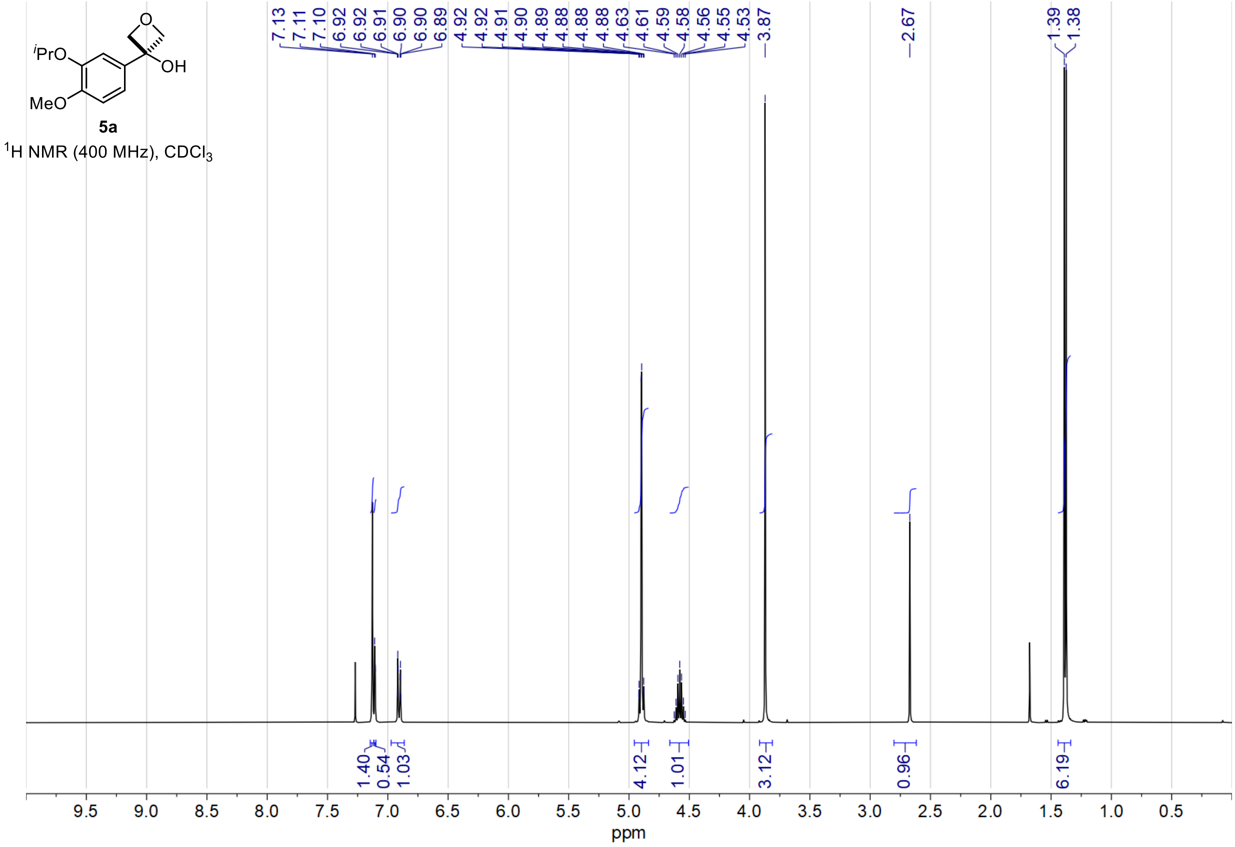

7.13
7.11
7.10
6.92
6.92
6.91
6.90
6.90
6.89
4.92
4.92
4.91
4.90
4.89
4.88
4.88
4.88
4.63
4.61
4.59
4.58
4.56
4.55
4.53
3.87
2.67
1.39
1.38
1.40
0.54
1.03
4.12
1.01
3.12
0.96
6.19
ppm

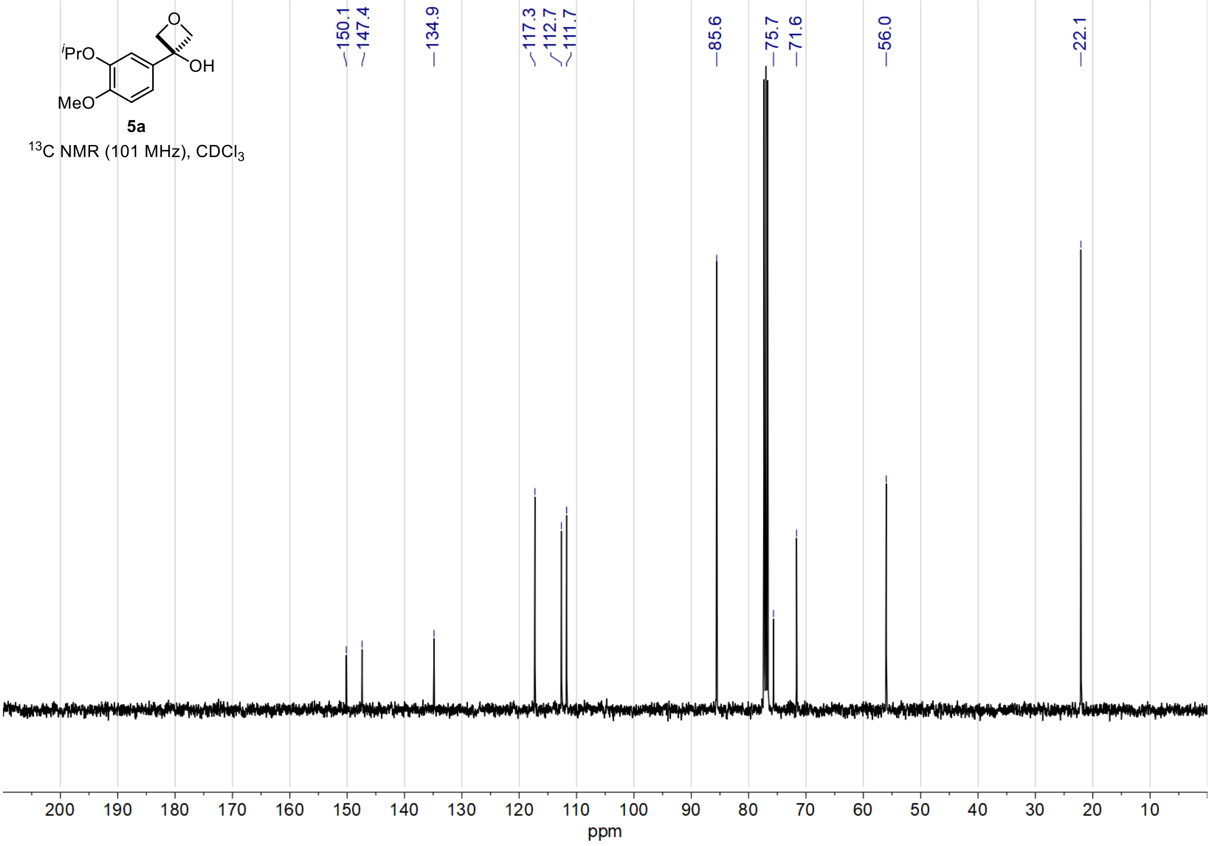

150.1
147.4
134.9
117.3
112.7
111.7
85.6
75.7
71.6
56.0
22.1
ppm

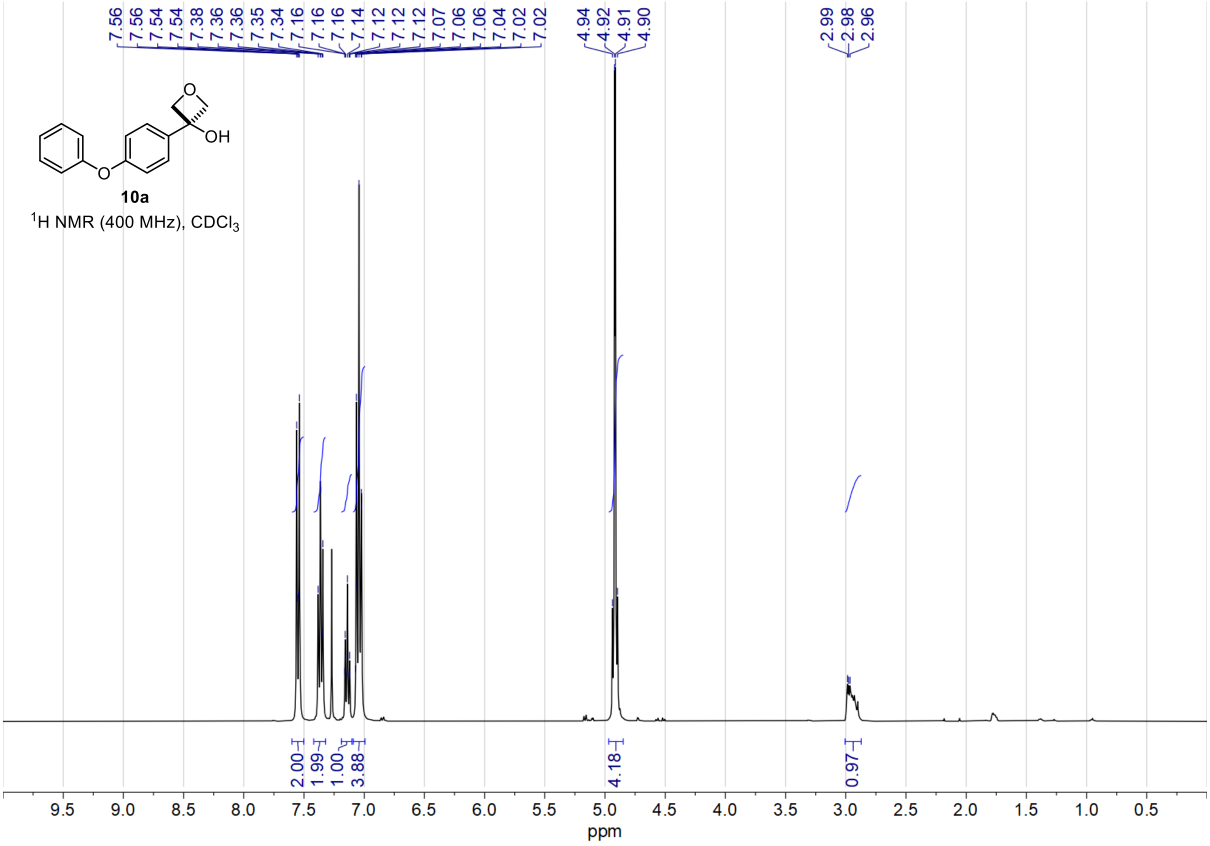
7.56
7.56
7.54
7.54
7.38
7.36
7.36
7.35
7.34
7.16
7.16
7.16
7.14
7.12
7.12
7.12
7.07
7.06
7.06
7.04
7.02
7.02
4.94
4.92
4.91
4.90
2.99
2.98
2.96
OH
O
O
10a
^{1}H NMR (400 MHz), $CDCl_3$
2.00
1.99
1.00
3.88
4.18
0.97
9.5 9.0 8.5 8.0 7.5 7.0 6.5 6.0 5.5 5.0 4.5 4.0 3.5 3.0 2.5 2.0 1.5 1.0 0.5
ppm

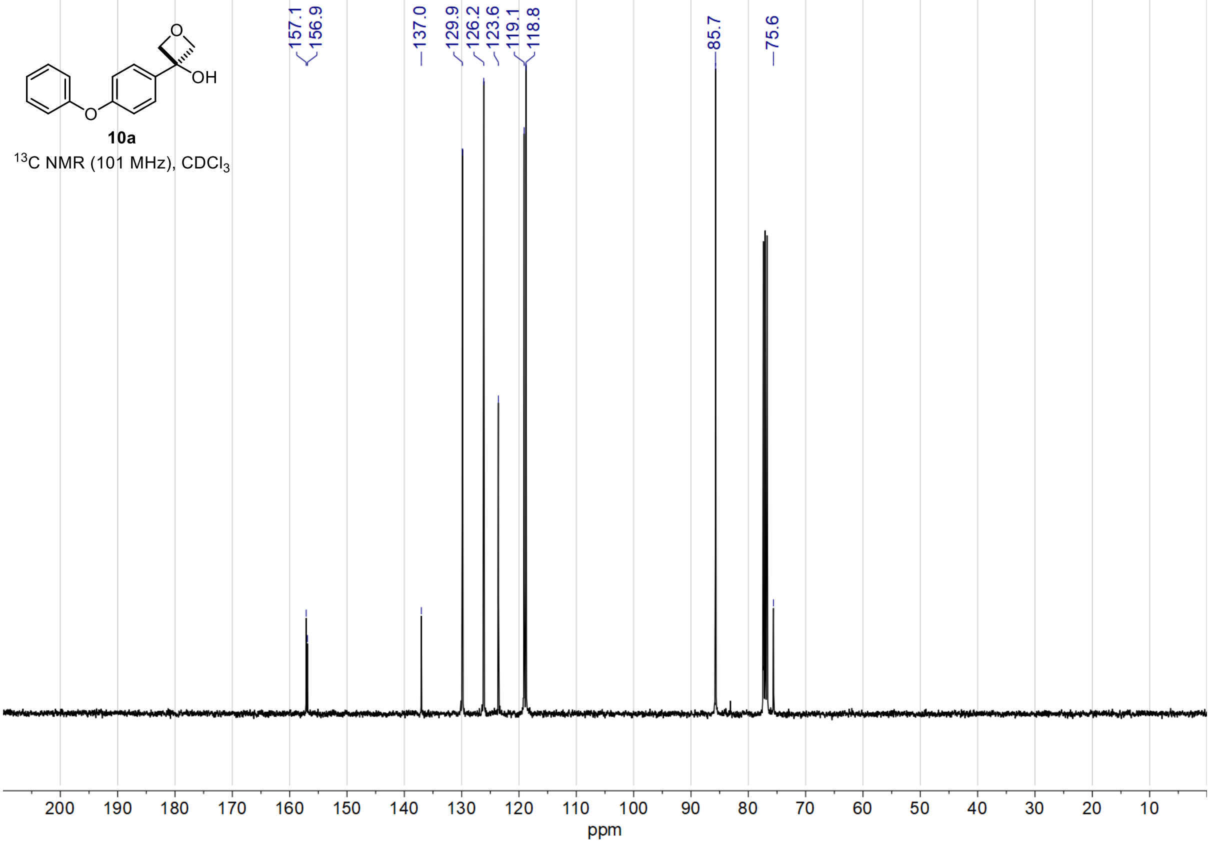
157.1
156.9
137.0
129.9
126.2
123.6
119.1
118.8
85.7
75.6
OH
O
O
10a
^{13}C NMR (101 MHz), $CDCl_3$
200 190 180 170 160 150 140 130 120 110 100 90 80 70 60 50 40 30 20 10
ppm

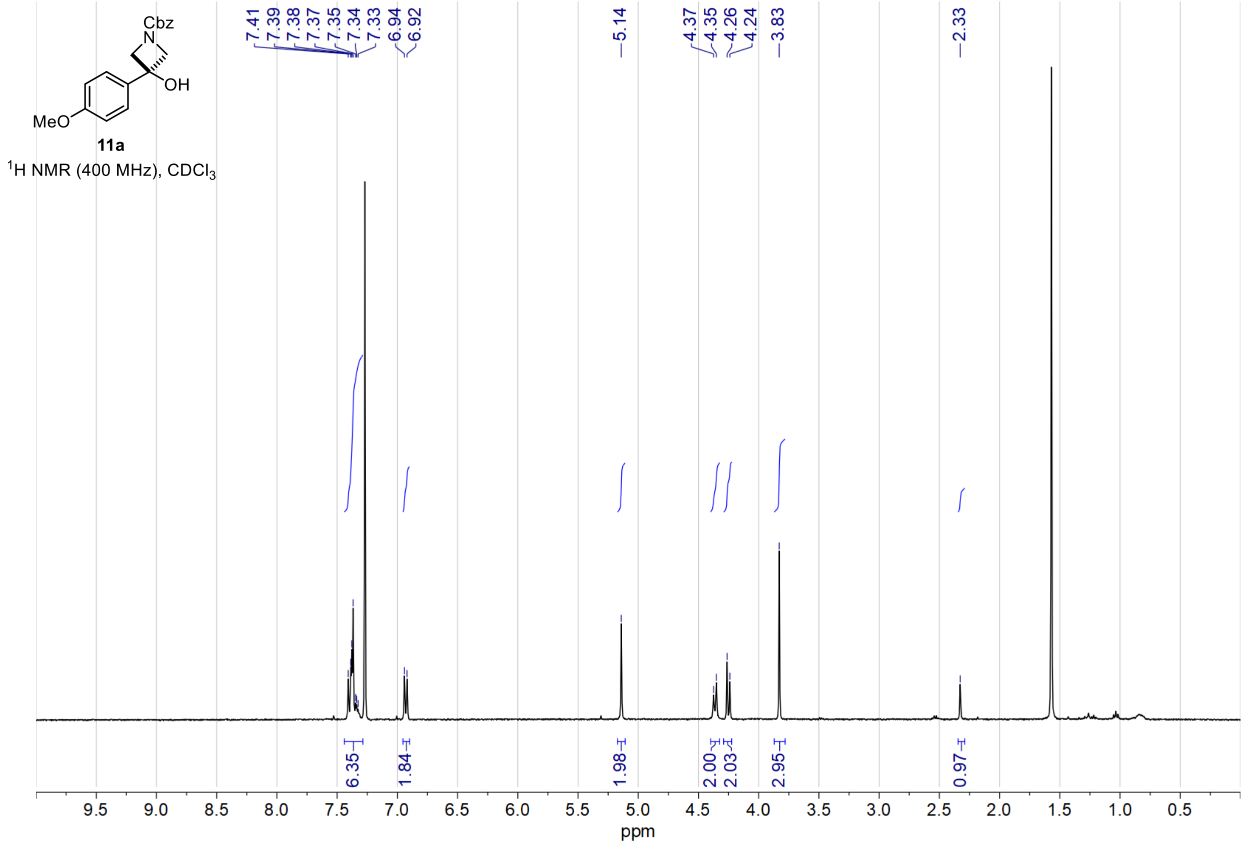
Cbz
N
OH
MeO
11a
^{1}H NMR (400 MHz), CDCl$_3$
7.41
7.39
7.38
7.37
7.35
7.34
7.33
6.94
6.92
5.14
4.37
4.35
4.26
4.24
3.83
2.33
6.35
1.84
1.98
2.00
2.03
2.95
0.97
9.5
9.0
8.5
8.0
7.5
7.0
6.5
6.0
5.5
5.0
4.5
4.0
3.5
3.0
2.5
2.0
1.5
1.0
0.5
ppm

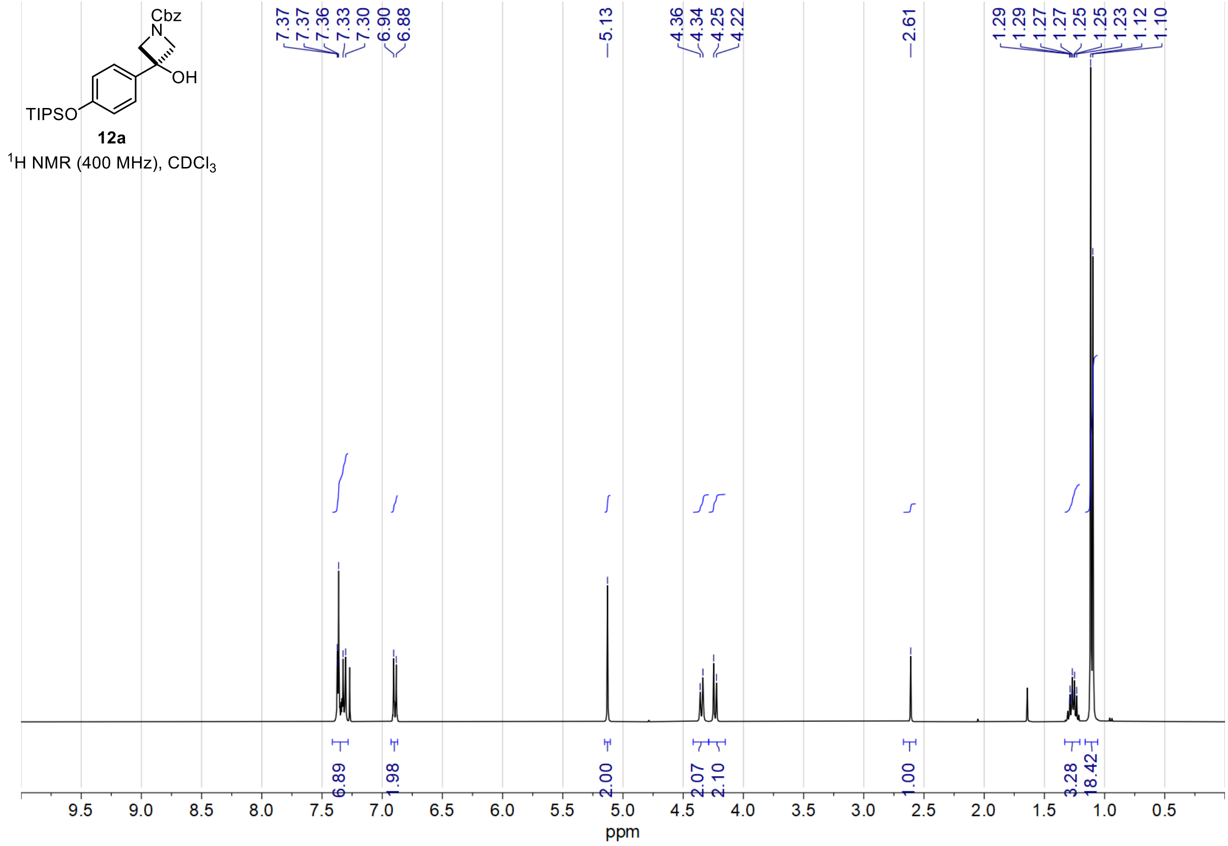
Cbz
N
OH
TIPSO
12a
1H NMR (400 MHz), CDCl3
7.37
7.37
7.36
7.33
7.30
6.90
6.88
5.13
4.36
4.34
4.25
4.22
2.61
1.29
1.29
1.27
1.27
1.25
1.25
1.23
1.12
1.10
6.89
1.98
2.00
2.07
2.10
1.00
3.28
18.42
9.5
9.0
8.5
8.0
7.5
7.0
6.5
6.0
5.5
5.0
4.5
4.0
3.5
3.0
2.5
2.0
1.5
1.0
0.5
ppm

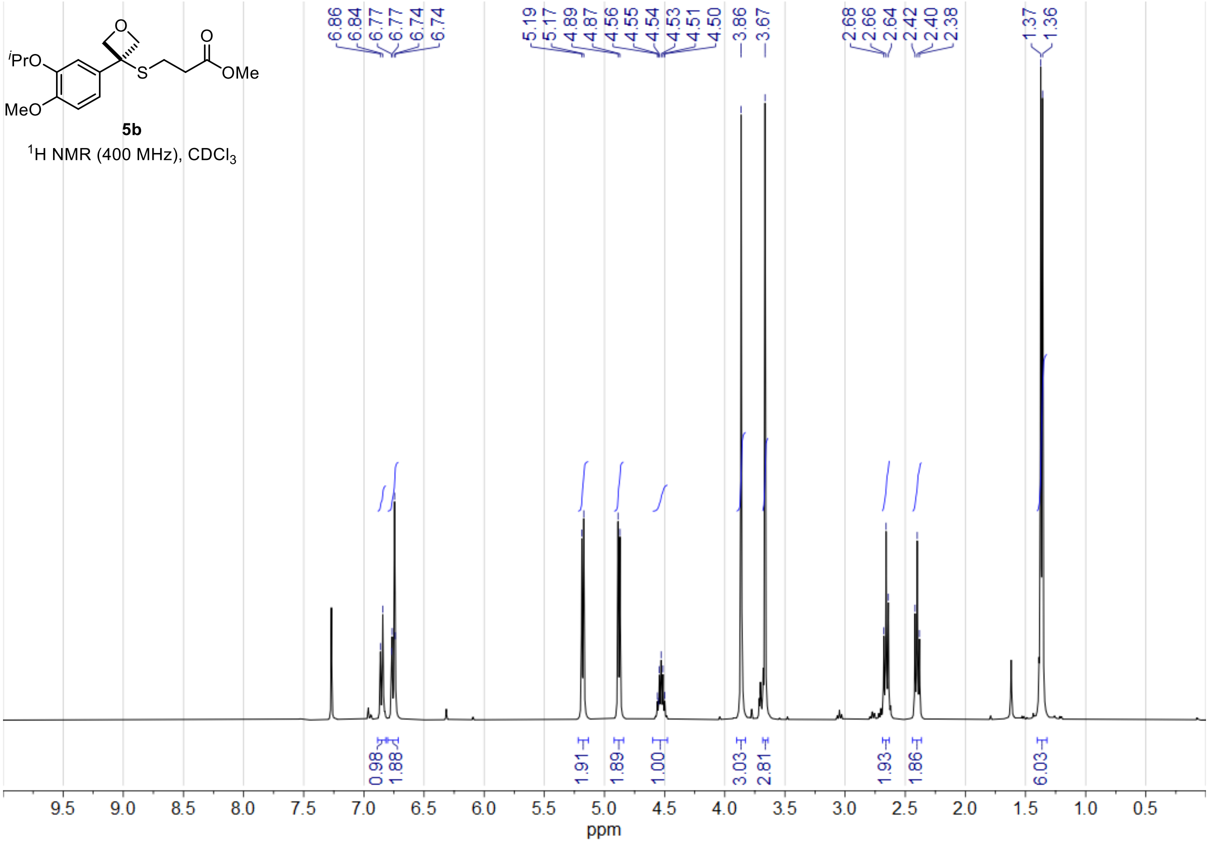

iPrO
MeO
OMe
O
S
5b
1H NMR (400 MHz), CDCl3
6.86 6.84 6.77 6.77 6.74 6.74
5.19 5.17 4.89 4.87 4.56 4.55 4.54 4.53 4.51 4.50
3.86
3.67
2.68 2.66 2.64 2.42 2.40 2.38
1.37 1.36
0.98 1.88 1.91 1.89 1.00 3.03 2.81 1.93 1.86 6.03
9.5 9.0 8.5 8.0 7.5 7.0 6.5 6.0 5.5 5.0 4.5 4.0 3.5 3.0 2.5 2.0 1.5 1.0 0.5
ppm

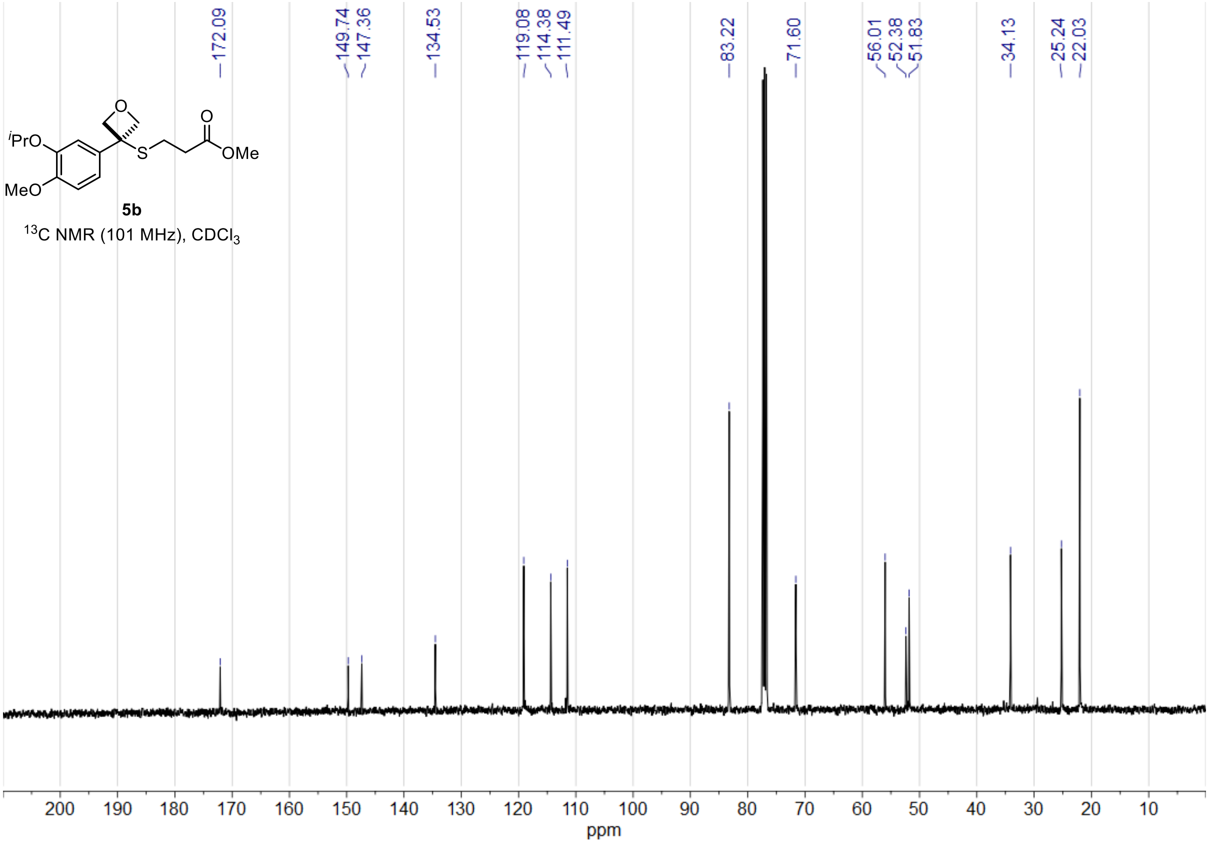

iPrO
MeO
OMe
O
S
5b
13C NMR (101 MHz), CDCl3
172.09
149.74 147.36
134.53
119.08 114.38 111.49
83.22
71.60
56.01 52.38 51.83
34.13
25.24 22.03
200 190 180 170 160 150 140 130 120 110 100 90 80 70 60 50 40 30 20 10
ppm

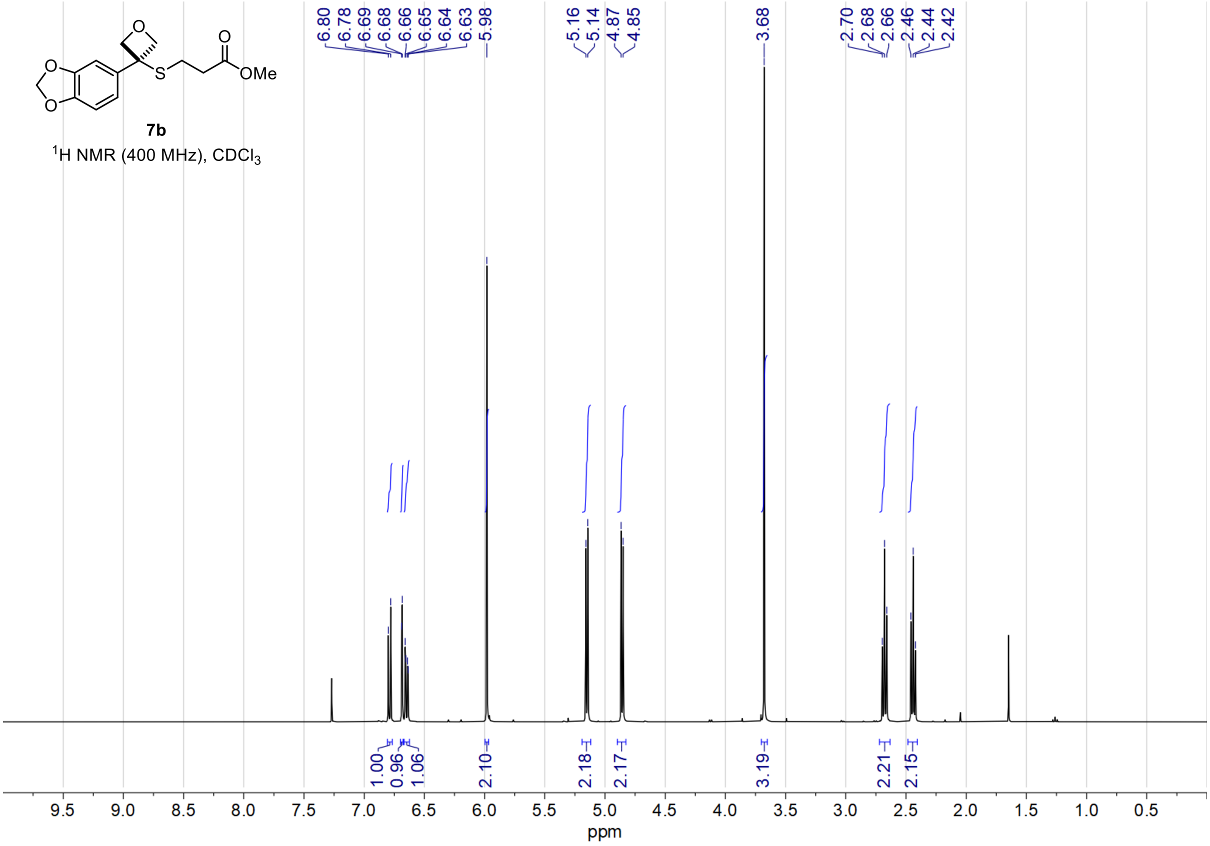

6.80
6.78
6.69
6.68
6.66
6.65
6.64
6.63
5.98
5.16
5.14
4.87
4.85
3.68
2.70
2.68
2.66
2.46
2.44
2.42
O
S
O
OMe
O
O
7b
^{1}H NMR (400 MHz), $CDCl_3$
1.00
0.96
1.06
2.10
2.18
2.17
3.19
2.21
2.15
9.5
9.0
8.5
8.0
7.5
7.0
6.5
6.0
5.5
5.0
4.5
4.0
3.5
3.0
2.5
2.0
1.5
1.0
0.5
ppm

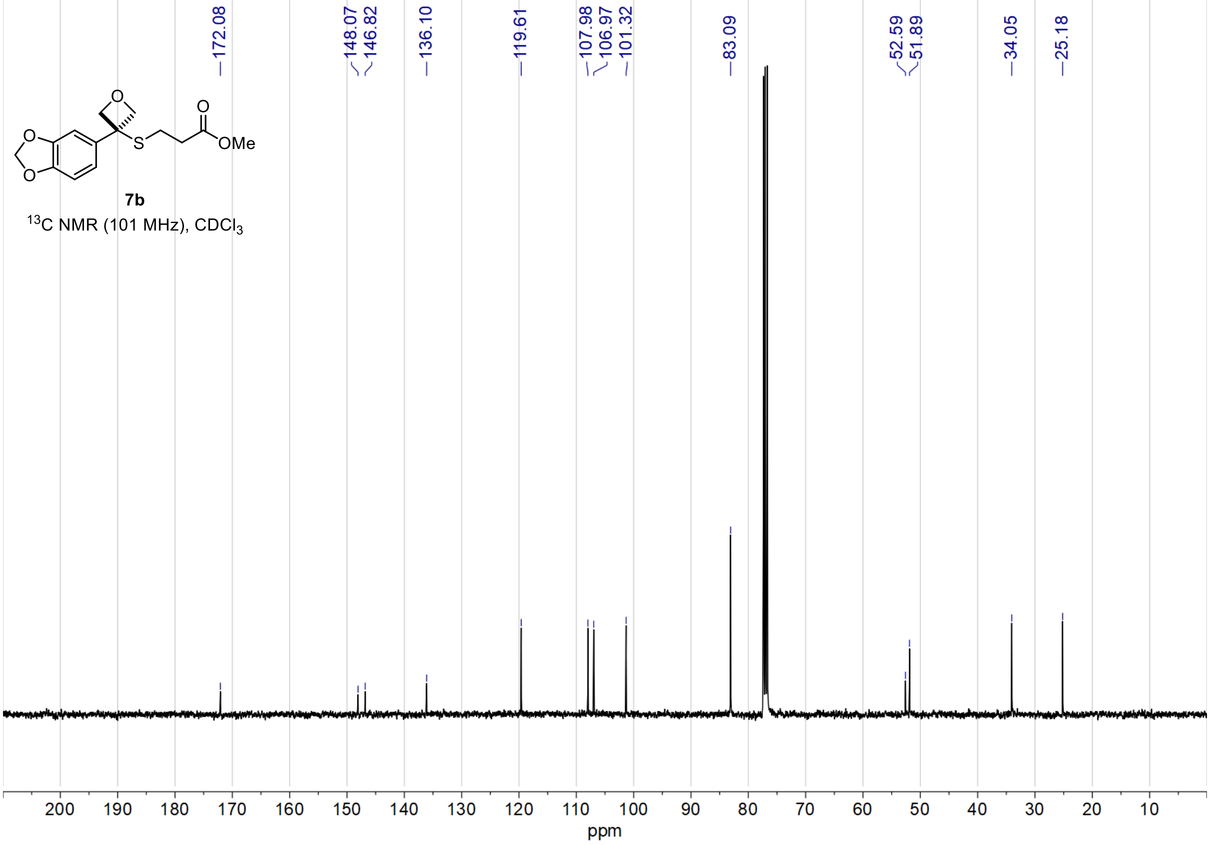

172.08
148.07
146.82
136.10
119.61
107.98
106.97
101.32
83.09
52.59
51.89
34.05
25.18
O
S
O
OMe
O
O
7b
^{13}C NMR (101 MHz), $CDCl_3$
200
190
180
170
160
150
140
130
120
110
100
90
80
70
60
50
40
30
20
10
ppm

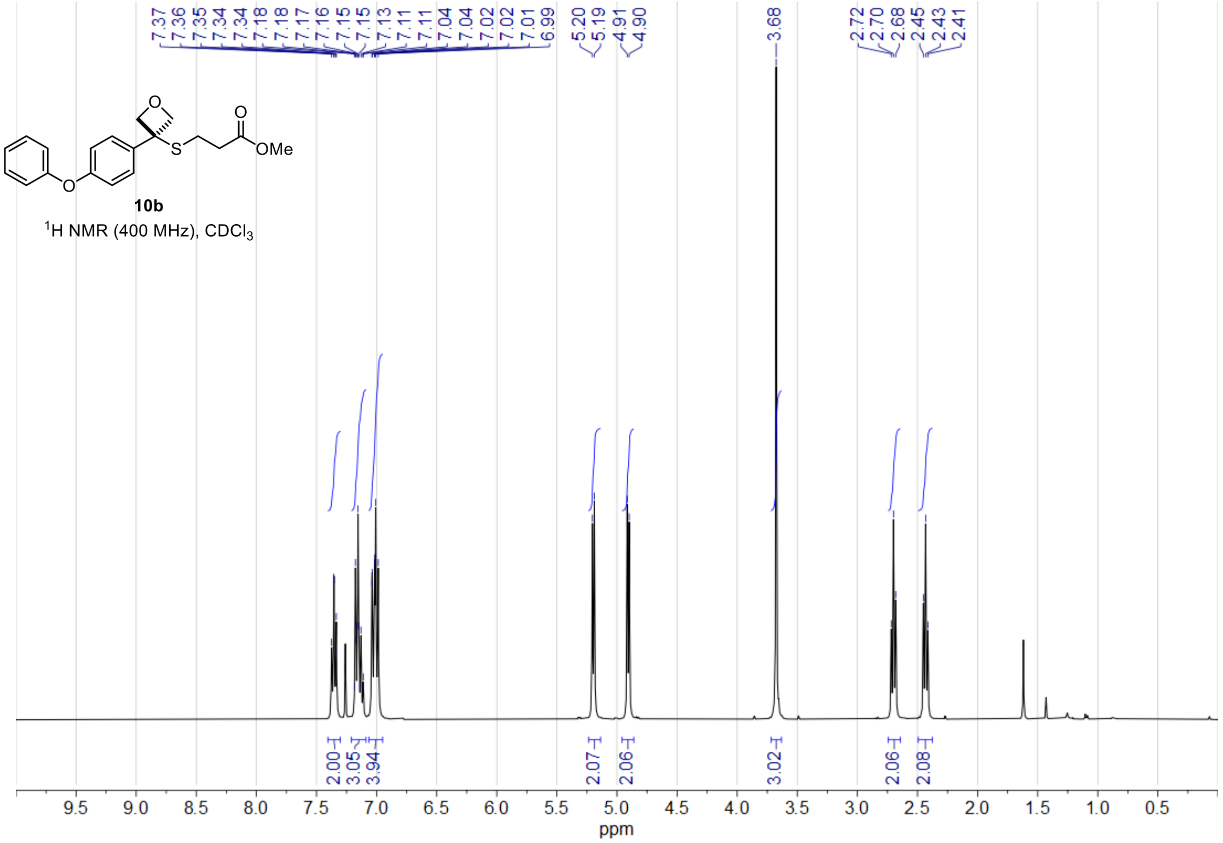
7.37
7.36
7.35
7.34
7.34
7.18
7.17
7.16
7.15
7.15
7.13
7.11
7.11
7.04
7.04
7.02
7.02
7.01
6.99
5.20
5.19
4.91
4.90
3.68
2.72
2.70
2.68
2.45
2.43
2.41
OMe
10b
^{1}H NMR (400 MHz), CDCl$_3$
2.00
3.05
3.94
2.07
2.06
3.02
2.06
2.08
ppm

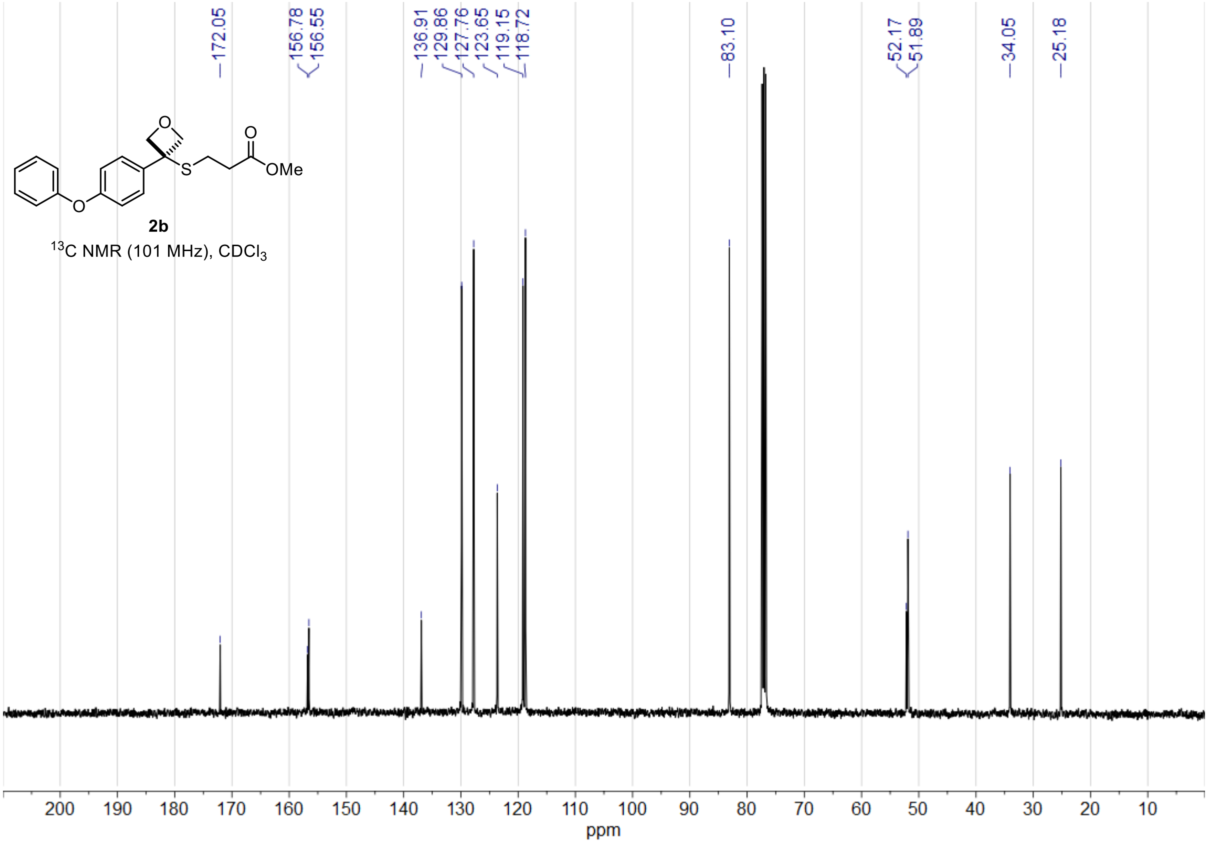
172.05
156.78
156.55
136.91
129.86
127.76
123.65
119.15
118.72
83.10
52.17
51.89
34.05
25.18
OMe
2b
^{13}C NMR (101 MHz), CDCl$_3$
ppm

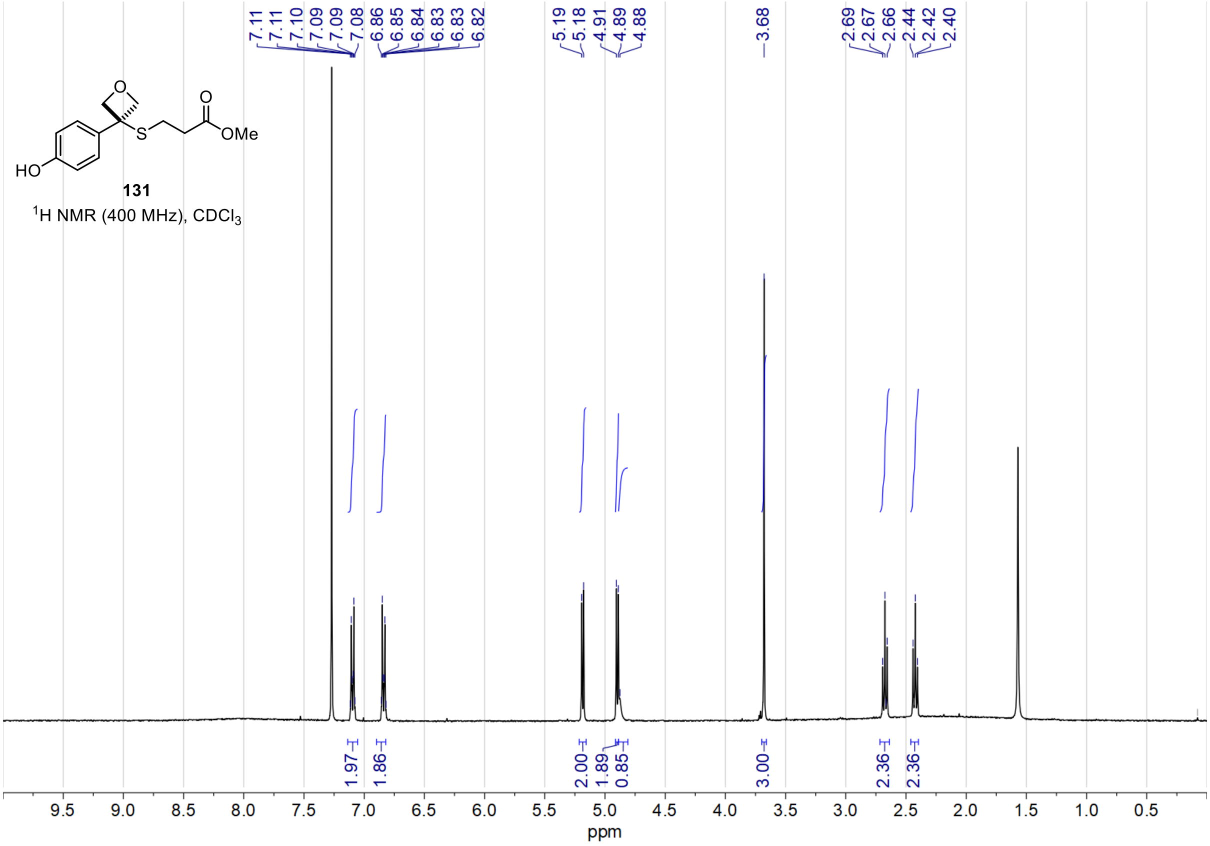

7.11
7.11
7.10
7.09
7.09
7.08
6.86
6.85
6.84
6.83
6.83
6.82
5.19
5.18
4.91
4.89
4.88
3.68
2.69
2.67
2.66
2.44
2.42
2.40
O
O
S
OMe
HO
131
1H NMR (400 MHz), CDCl3
1.97
1.86
2.00
1.89
0.85
3.00
2.36
2.36
9.5
9.0
8.5
8.0
7.5
7.0
6.5
6.0
5.5
5.0
4.5
4.0
3.5
3.0
2.5
2.0
1.5
1.0
0.5
ppm

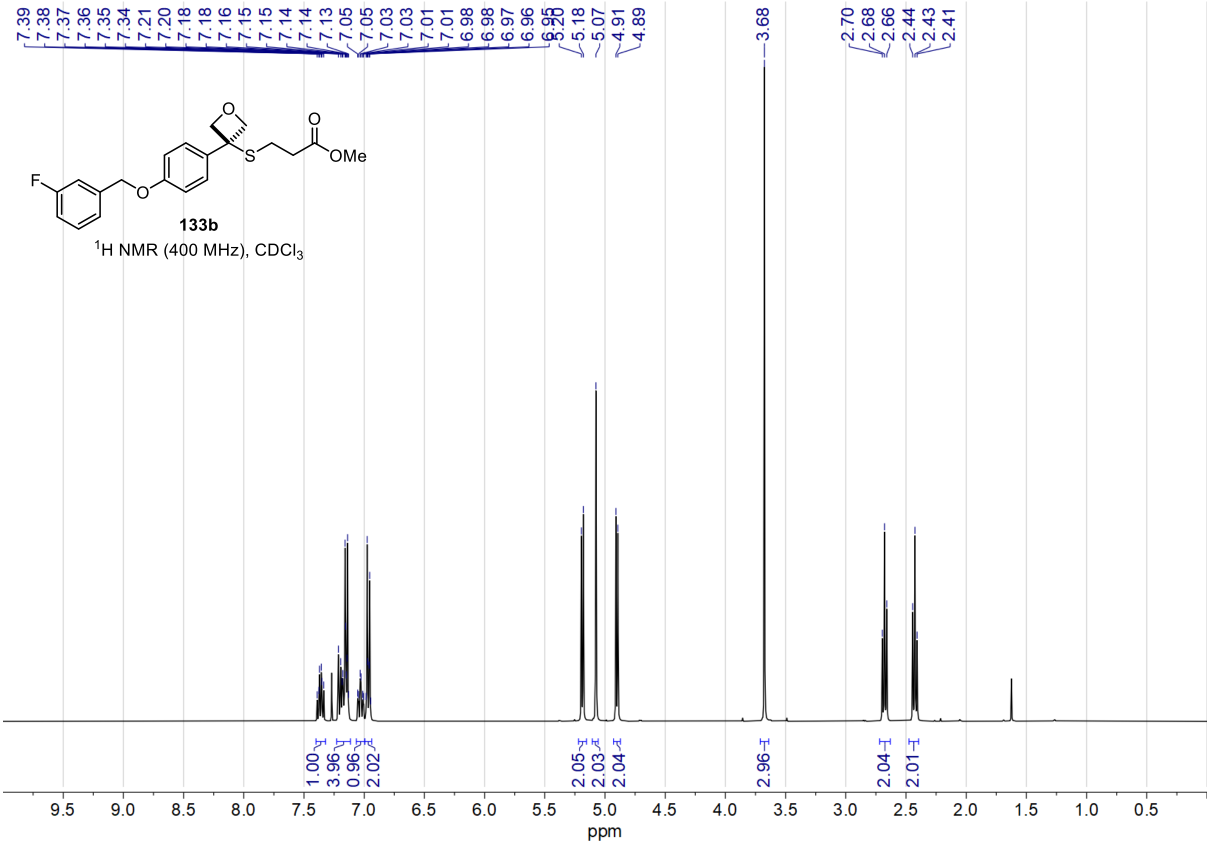
7.39 7.38 7.37 7.36 7.35 7.34 7.21 7.20 7.18 7.18 7.16 7.15 7.15 7.14 7.14 7.13 7.05 7.05 7.03 7.03 7.01 7.01 6.98 6.97 6.96 6.95 5.20 5.18 5.07 4.91 4.89
3.68
2.70 2.68 2.66 2.44 2.43 2.41
F
O
O
S
O
OMe
133b
^{1}H NMR (400 MHz), CDCl$_3$
1.00 3.96 0.96 2.02 2.05 2.03 2.04 2.96 2.04 2.01
9.5 9.0 8.5 8.0 7.5 7.0 6.5 6.0 5.5 5.0 4.5 4.0 3.5 3.0 2.5 2.0 1.5 1.0 0.5
ppm

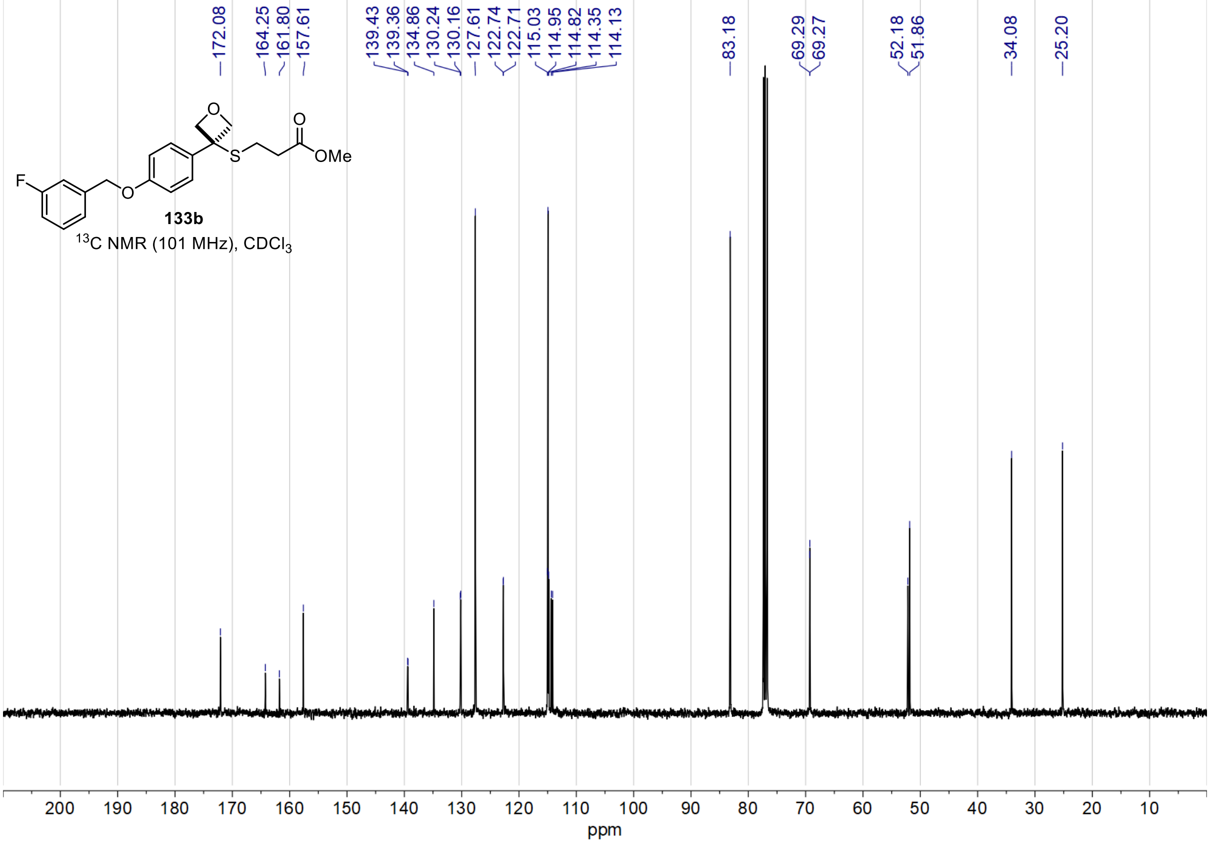
172.08
164.25 161.80 157.61
139.43 139.36 134.86 130.24 130.16 127.61 122.74 122.71 115.03 114.95 114.82 114.35 114.13
83.18
69.29 69.27
52.18 51.86
34.08
25.20
F
O
O
S
O
OMe
133b
^{13}C NMR (101 MHz), CDCl$_3$
200 190 180 170 160 150 140 130 120 110 100 90 80 70 60 50 40 30 20 10
ppm

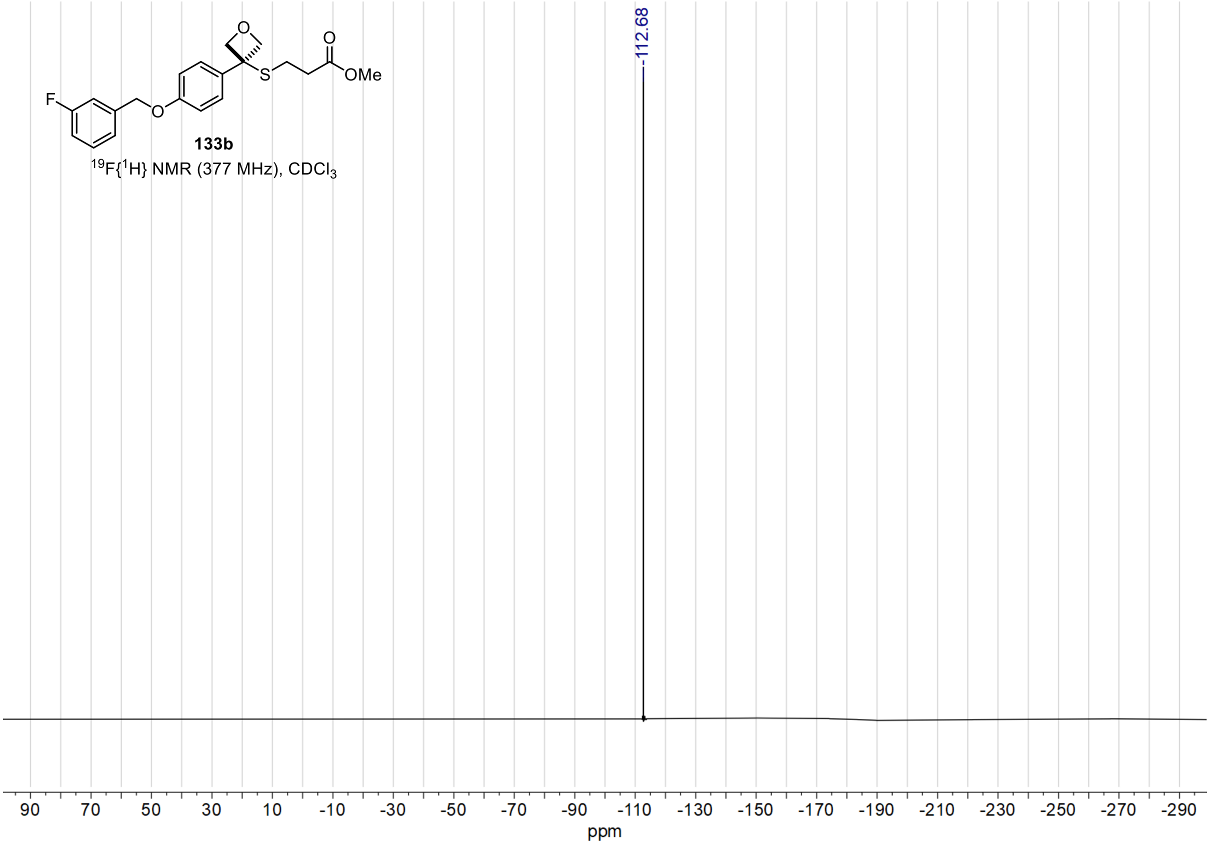

F
O
O
S
O
OMe
133b
$^{19}F\{^{1}H\}$ NMR (377 MHz), $CDCl_3$
-112.68
90 70 50 30 10 -10 -30 -50 -70 -90 -110 -130 -150 -170 -190 -210 -230 -250 -270 -290
ppm

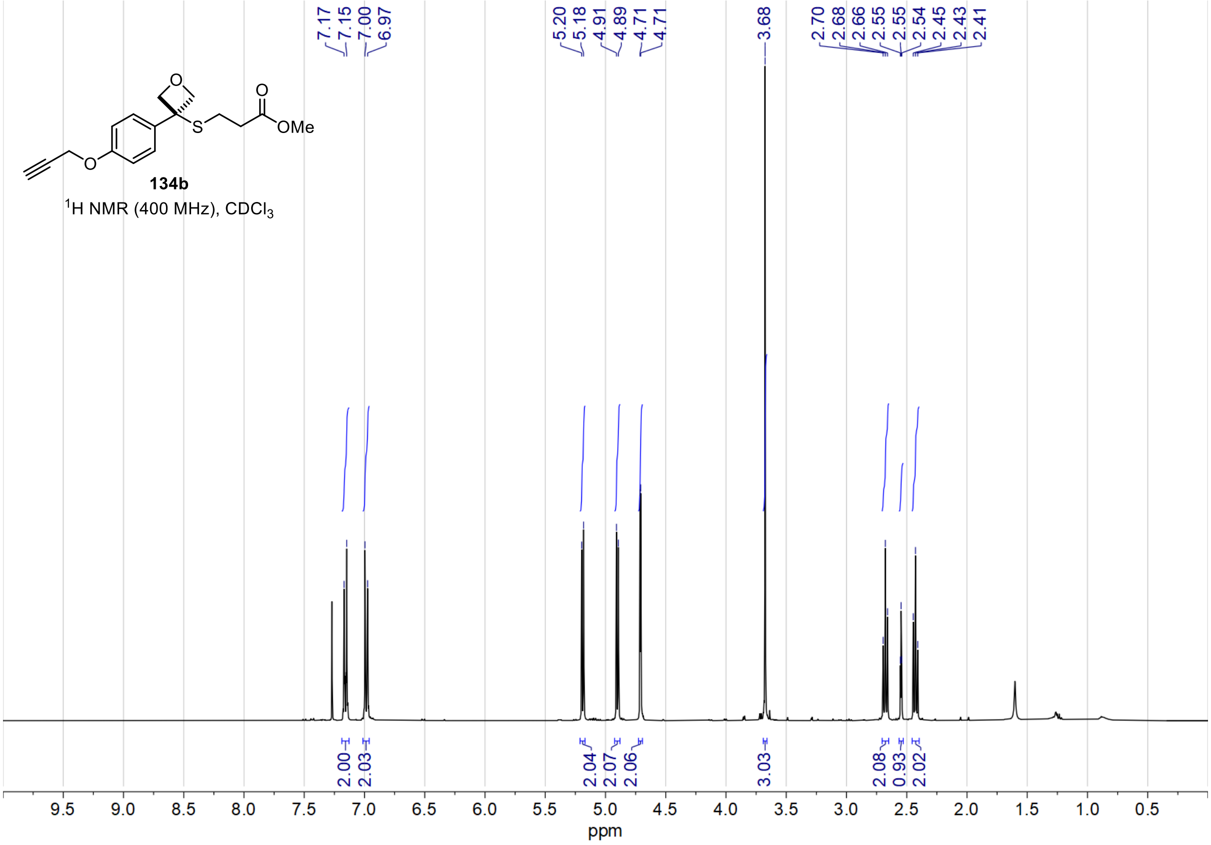
7.17
7.15
7.00
6.97
5.20
5.18
4.91
4.89
4.71
4.71
3.68
2.70
2.68
2.66
2.55
2.55
2.54
2.45
2.43
2.41
134b
^{1}H NMR (400 MHz), CDCl$_3$
2.00
2.03
2.04
2.07
2.06
3.03
2.08
0.93
2.02
ppm

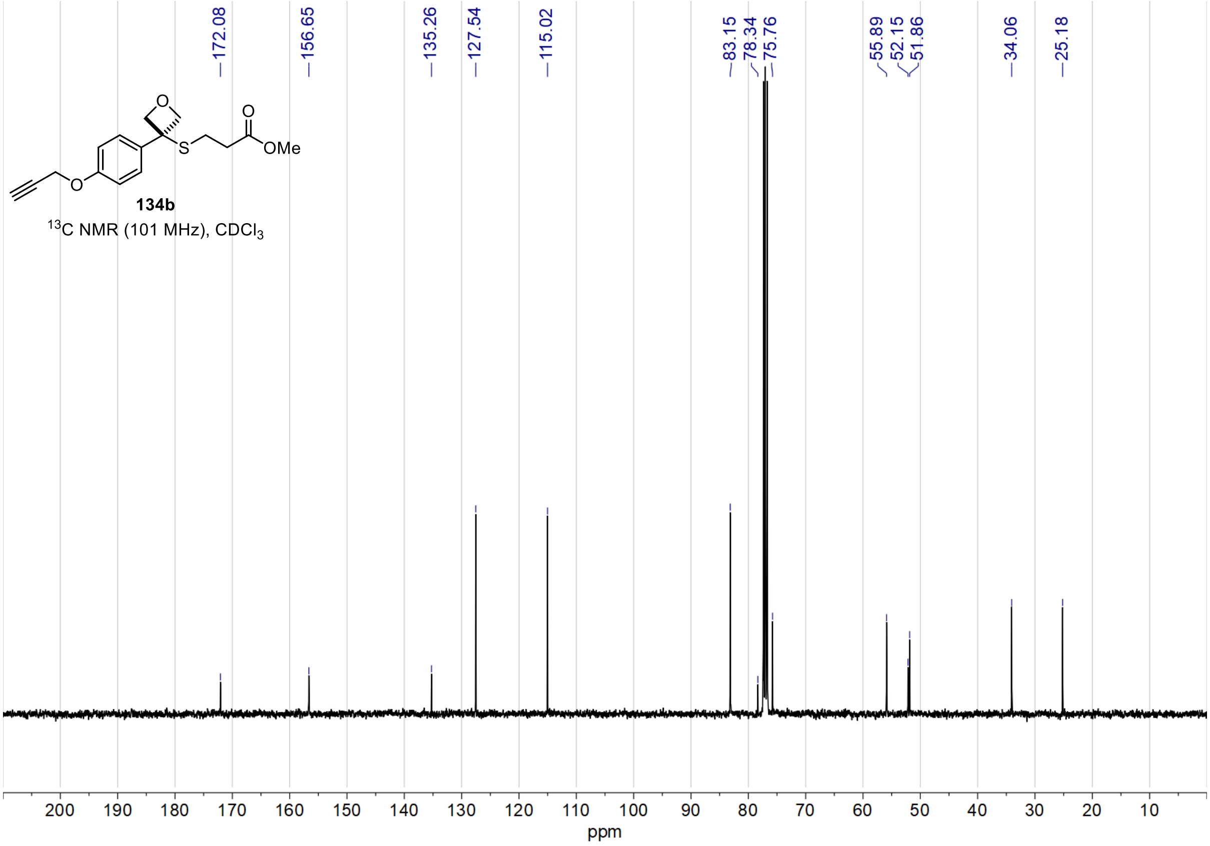
172.08
156.65
135.26
127.54
115.02
83.15
78.34
75.76
55.89
52.15
51.86
34.06
25.18
134b
^{13}C NMR (101 MHz), CDCl$_3$
ppm

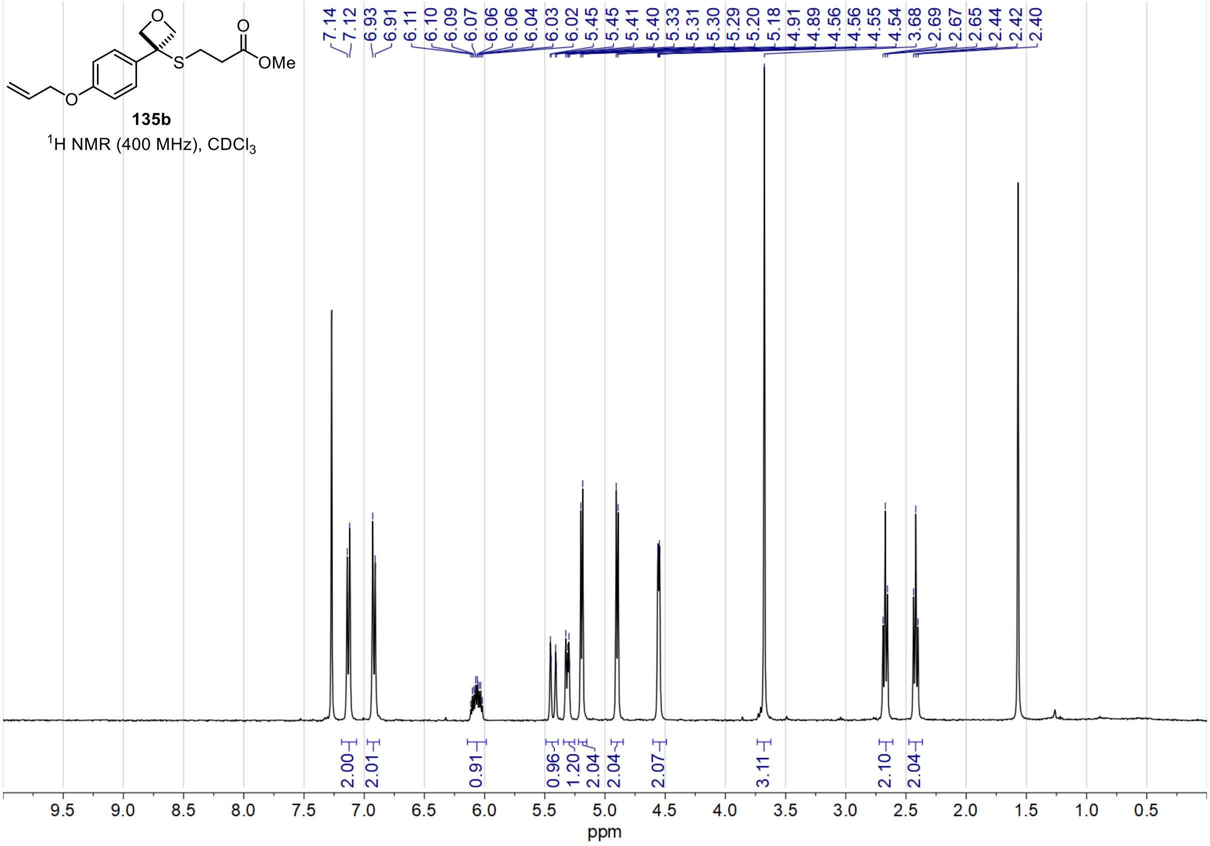
OMe
135b
^{1}H NMR (400 MHz), $CDCl_3$
ppm

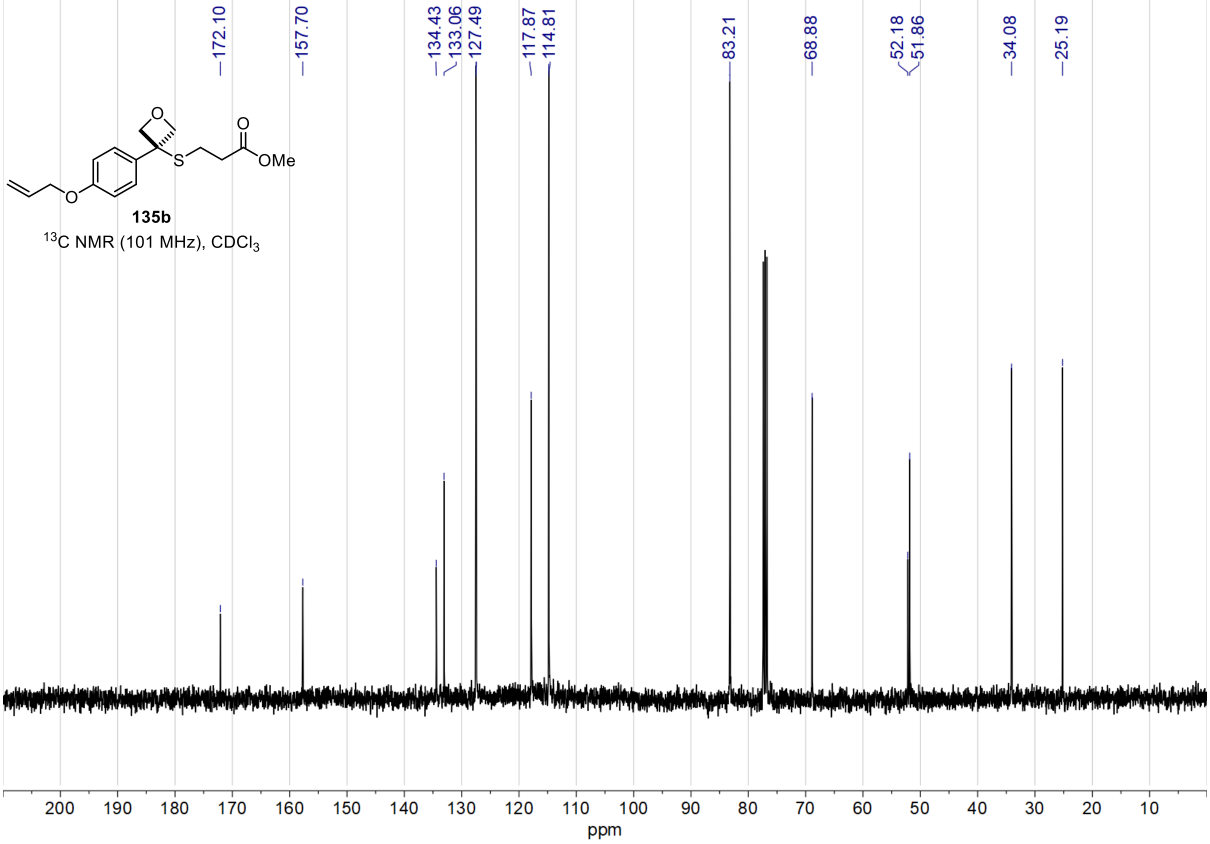
OMe
135b
^{13}C NMR (101 MHz), $CDCl_3$
ppm

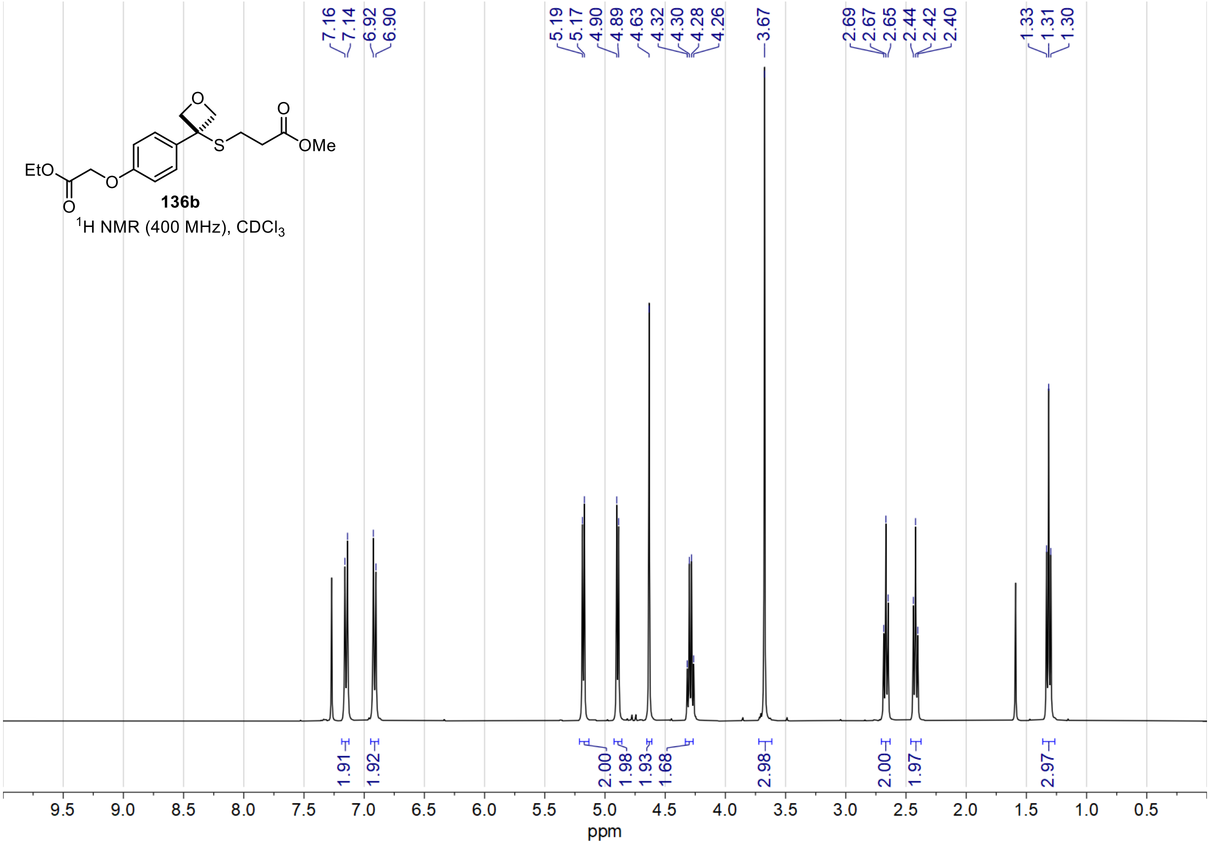
7.16
7.14
6.92
6.90
5.19
5.17
4.90
4.89
4.63
4.32
4.30
4.28
4.26
3.67
2.69
2.67
2.65
2.44
2.42
2.40
1.33
1.31
1.30
EtO
O
O
O
S
O
OMe
136b
1H NMR (400 MHz), CDCl3
1.91
1.92
2.00
1.98
1.93
1.68
2.98
2.00
1.97
2.97
ppm

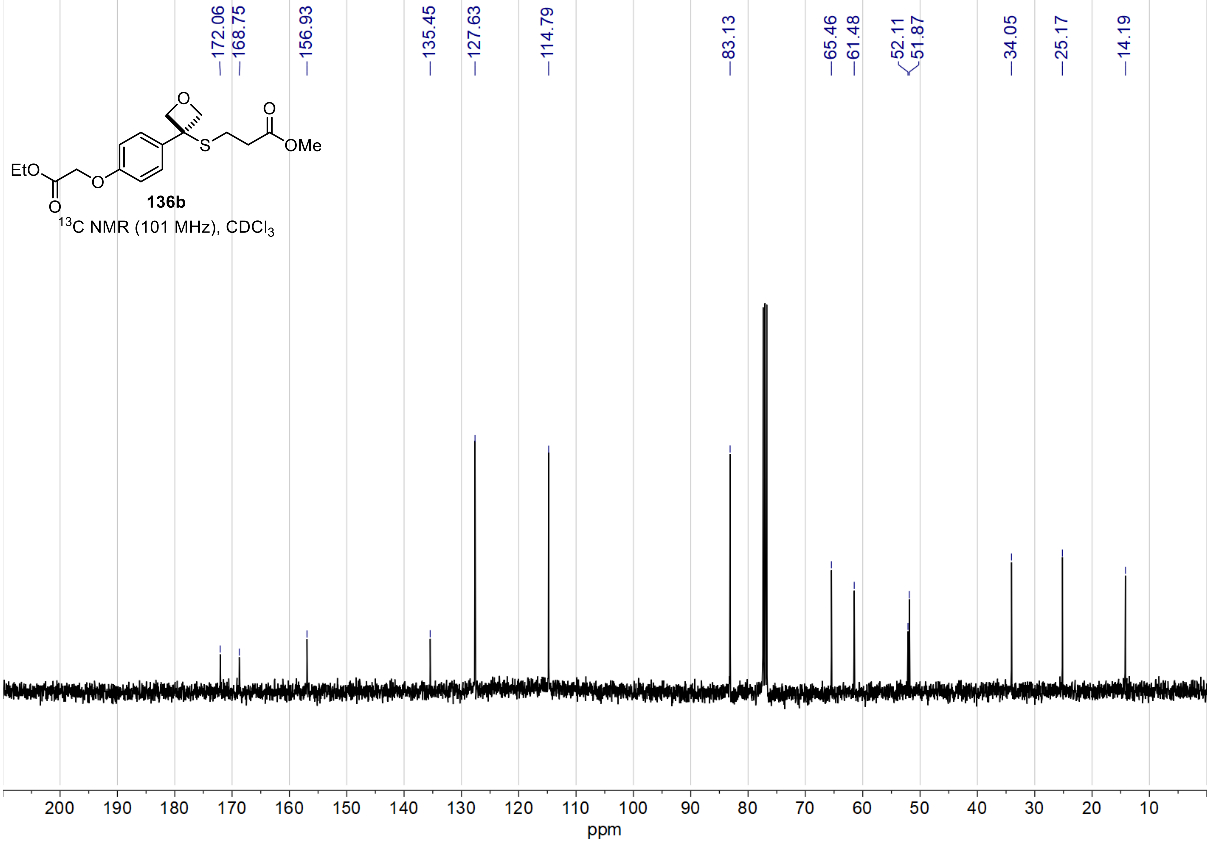
172.06
168.75
156.93
135.45
127.63
114.79
83.13
65.46
61.48
52.11
51.87
34.05
25.17
14.19
EtO
O
O
O
S
O
OMe
136b
13C NMR (101 MHz), CDCl3
ppm

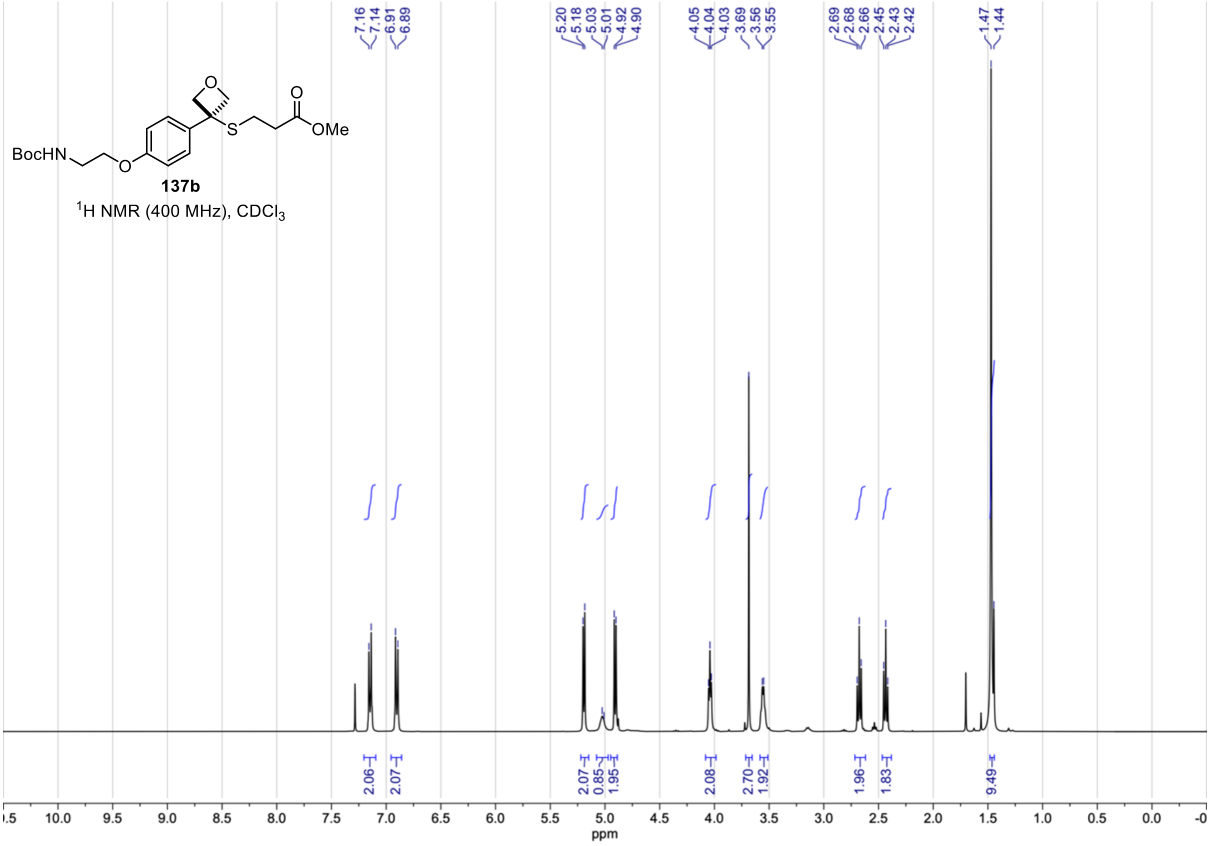
BocHN
O
S
OMe
137b
1H NMR (400 MHz), CDCl3
7.16 7.14 6.91 6.89
5.20 5.18 5.03 5.01 4.92 4.90
4.05 4.04 4.03 3.69 3.56 3.55
2.69 2.68 2.66 2.45 2.43 2.42
1.47 1.44
2.06 2.07 2.07 0.85 1.95 2.08 2.70 1.92 1.96 1.83 9.49
ppm

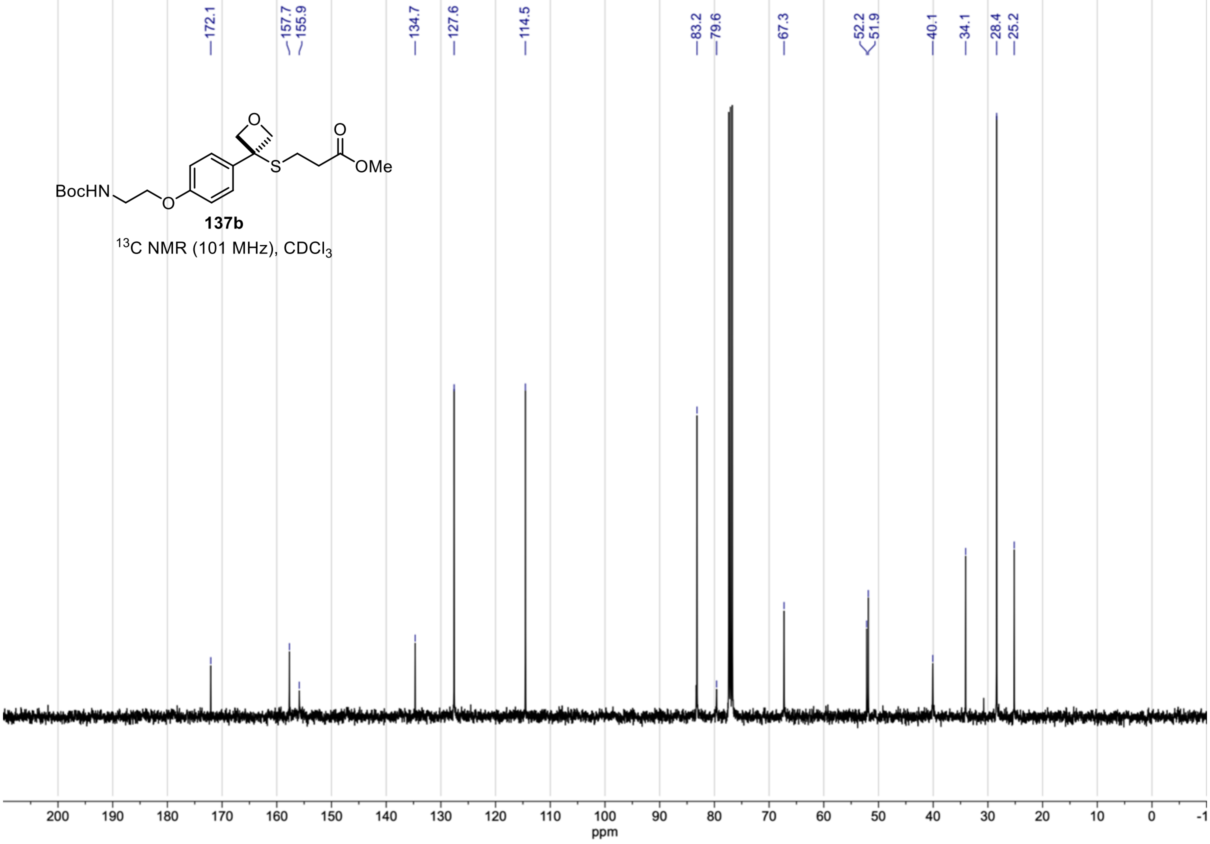
BocHN
O
S
OMe
137b
13C NMR (101 MHz), CDCl3
172.1
157.7 155.9
134.7
127.6
114.5
83.2
79.6
67.3
52.2 51.9
40.1
34.1
28.4 25.2
ppm

138

^{1}H NMR (400 MHz), $CDCl_3$

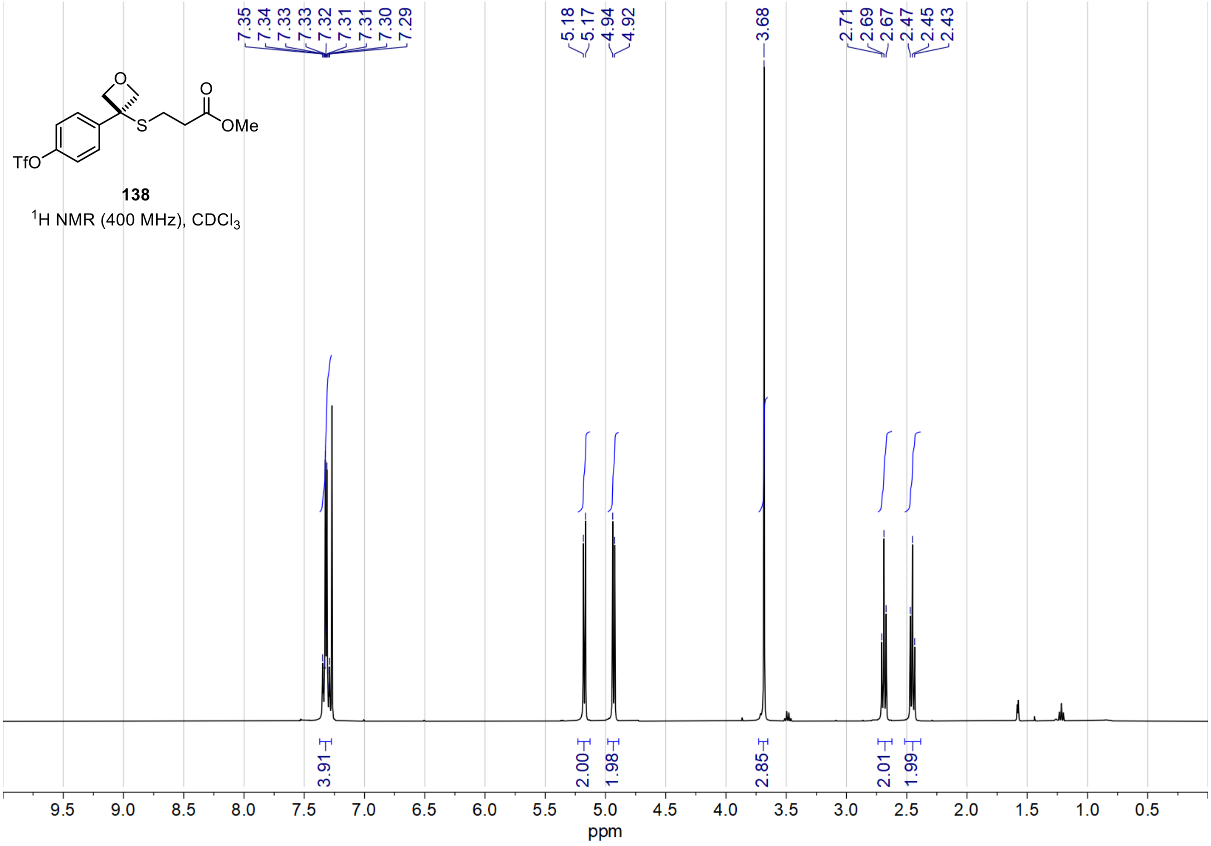

138

$^{19}F\{^1H\}$ NMR (377 MHz), $CDCl_3$

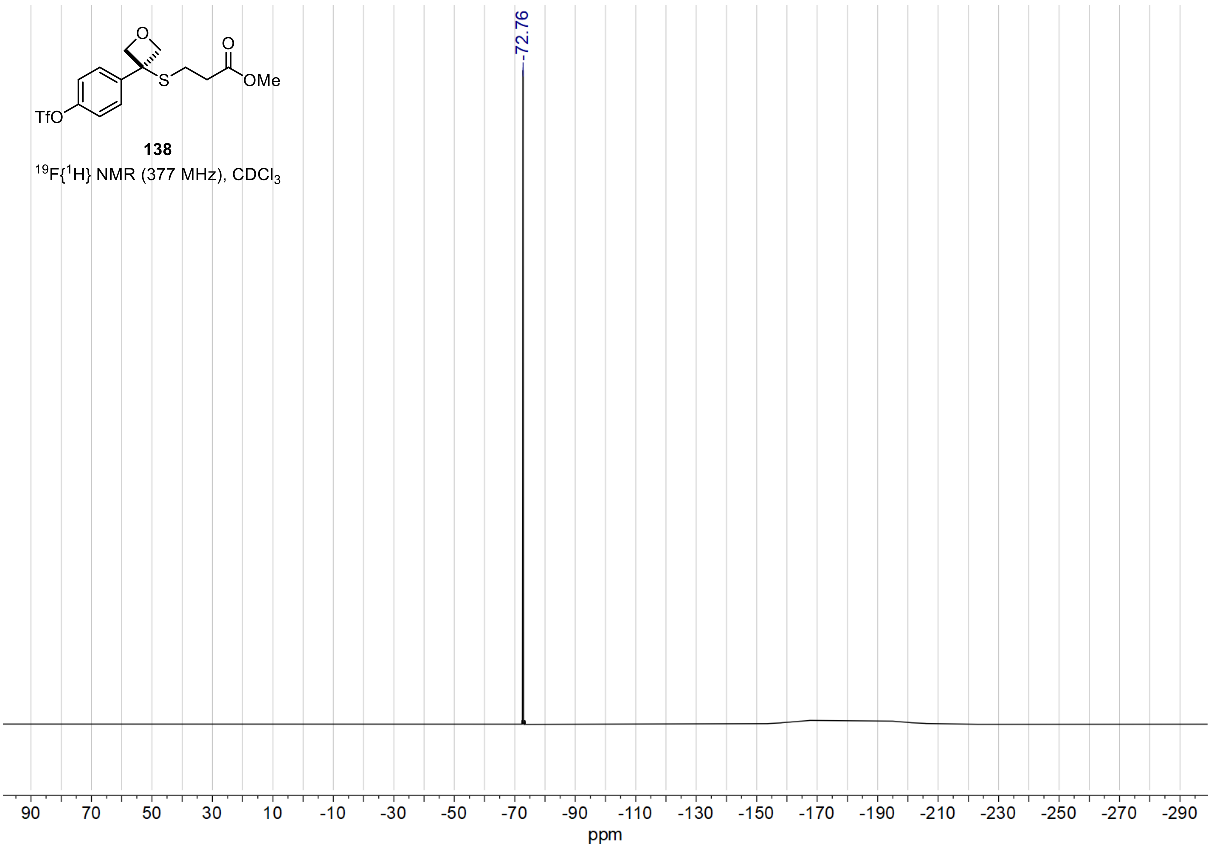

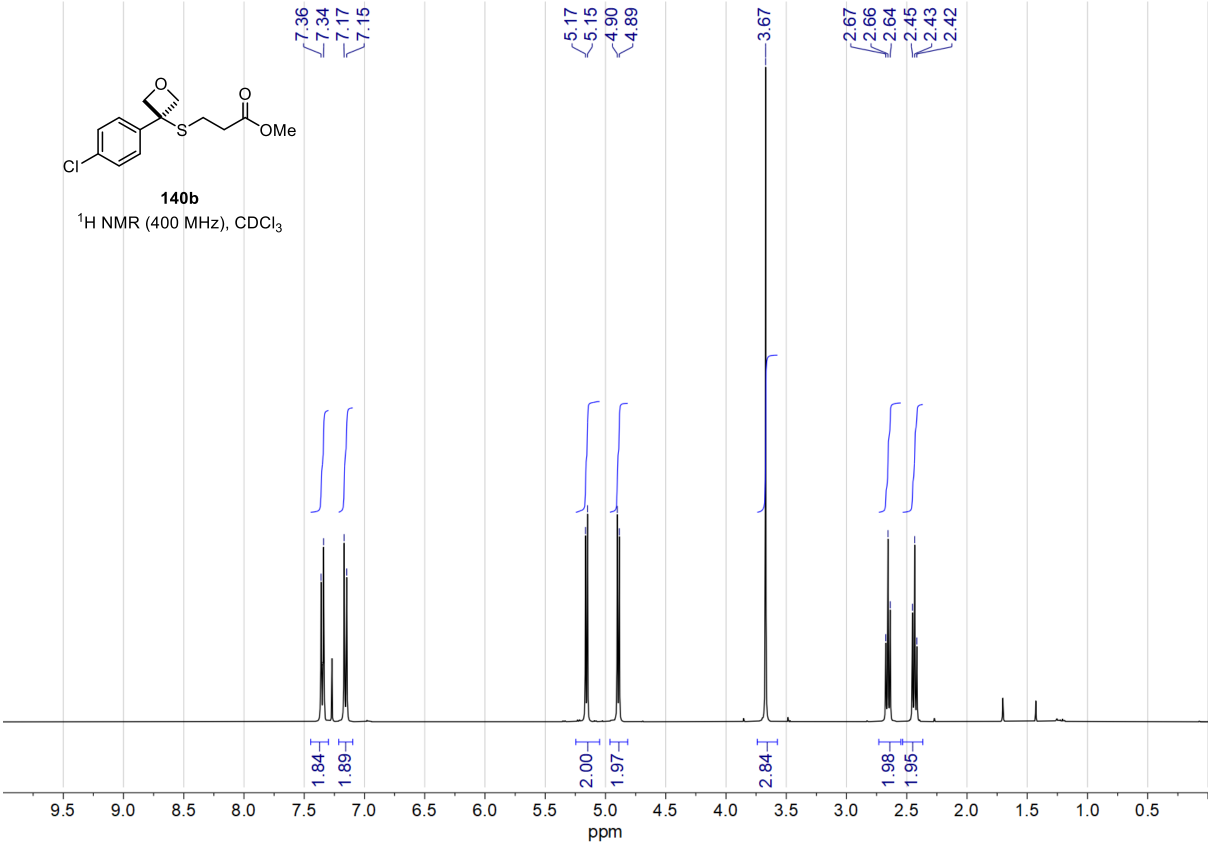
7.36
7.34
7.17
7.15
5.17
5.15
4.90
4.89
3.67
2.67
2.66
2.64
2.45
2.43
2.42
O
O
S
OMe
Cl
140b
1H NMR (400 MHz), CDCl3
1.84
1.89
2.00
1.97
2.84
1.98
1.95
9.5
9.0
8.5
8.0
7.5
7.0
6.5
6.0
5.5
5.0
4.5
4.0
3.5
3.0
2.5
2.0
1.5
1.0
0.5
ppm

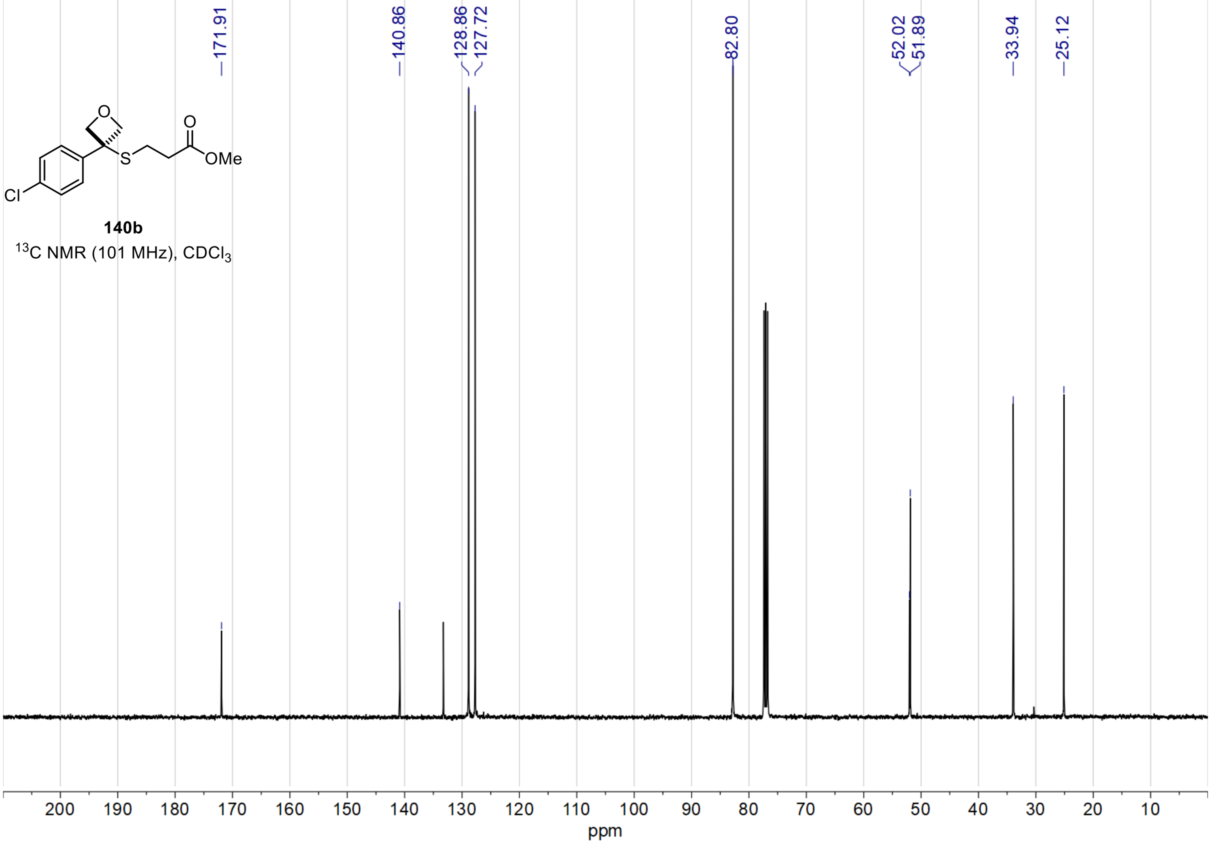
171.91
140.86
128.86
127.72
82.80
52.02
51.89
33.94
25.12
O
O
S
OMe
Cl
140b
13C NMR (101 MHz), CDCl3
200
190
180
170
160
150
140
130
120
110
100
90
80
70
60
50
40
30
20
10
ppm

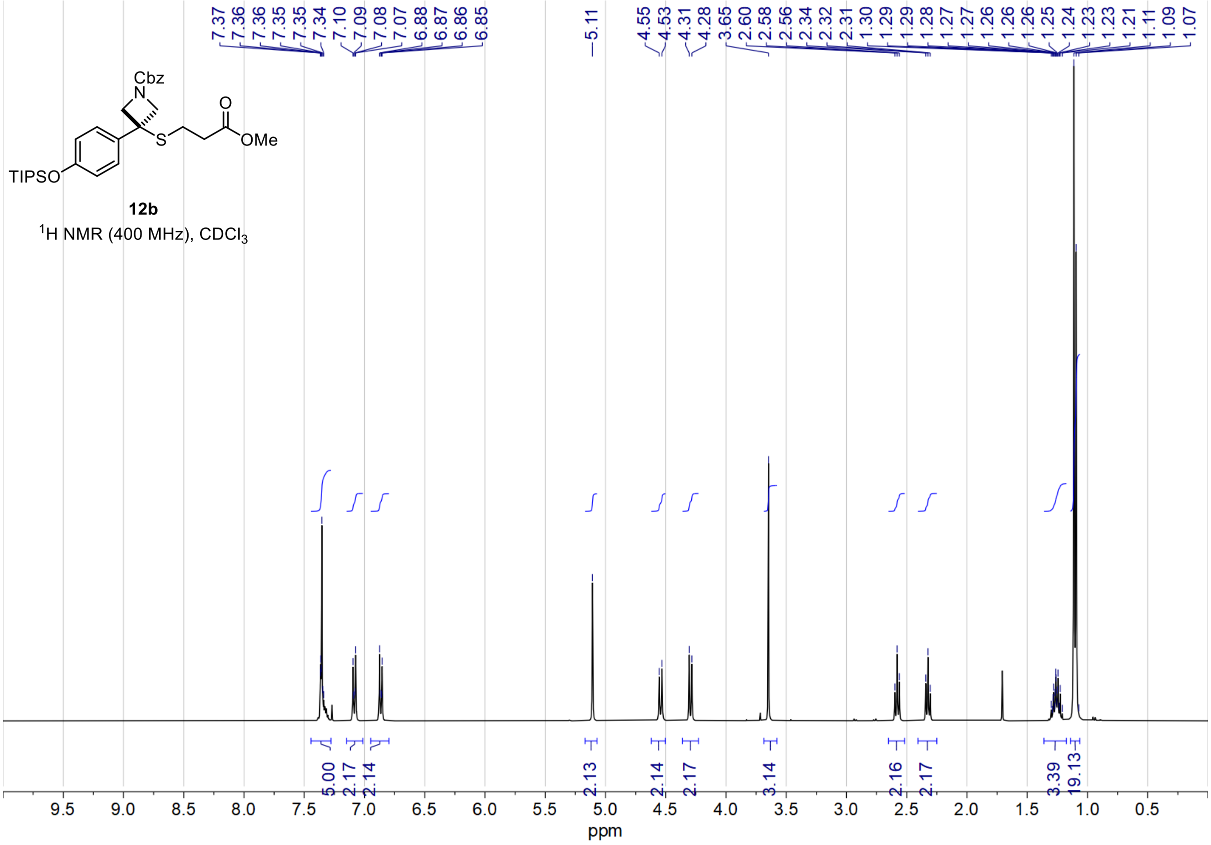

12b

^{1}H NMR (400 MHz), $CDCl_3$

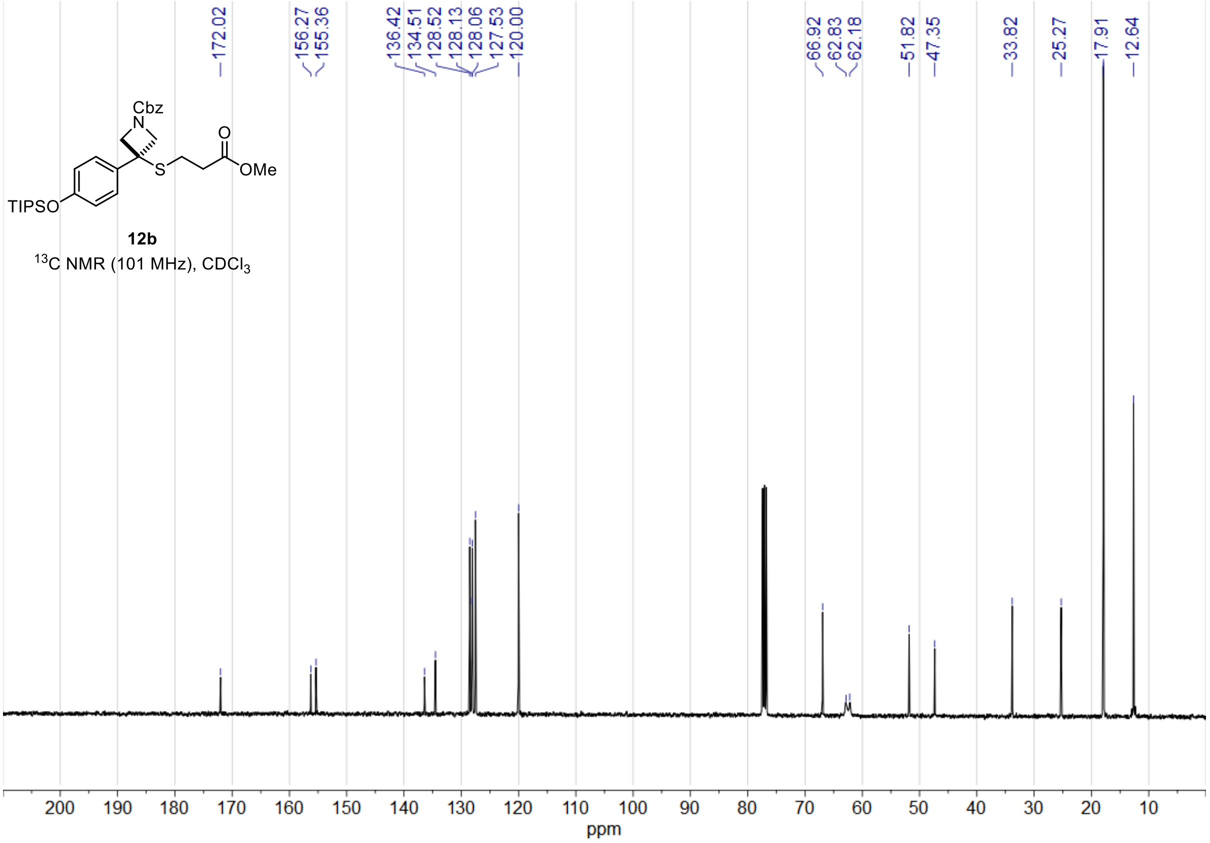

12b

^{13}C NMR (101 MHz), $CDCl_3$

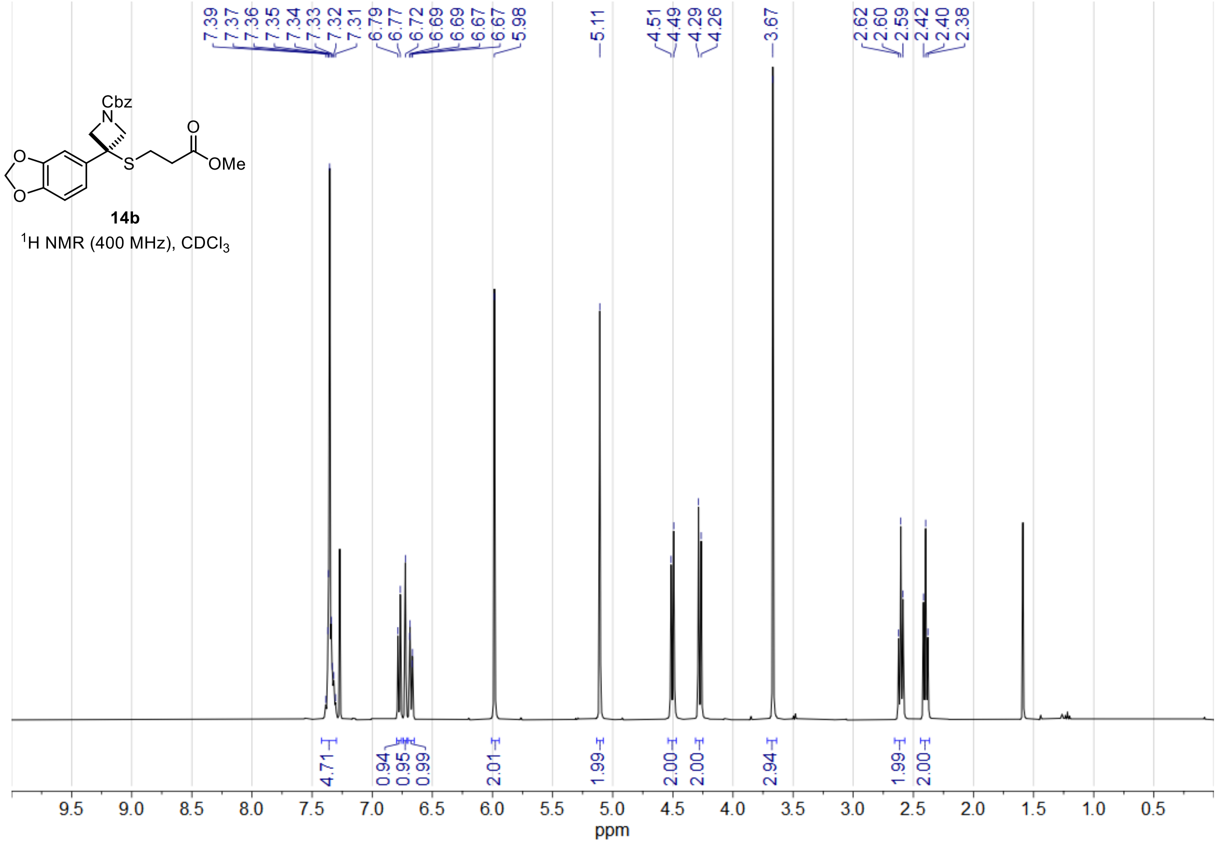

14b

^{1}H NMR (400 MHz), $CDCl_3$

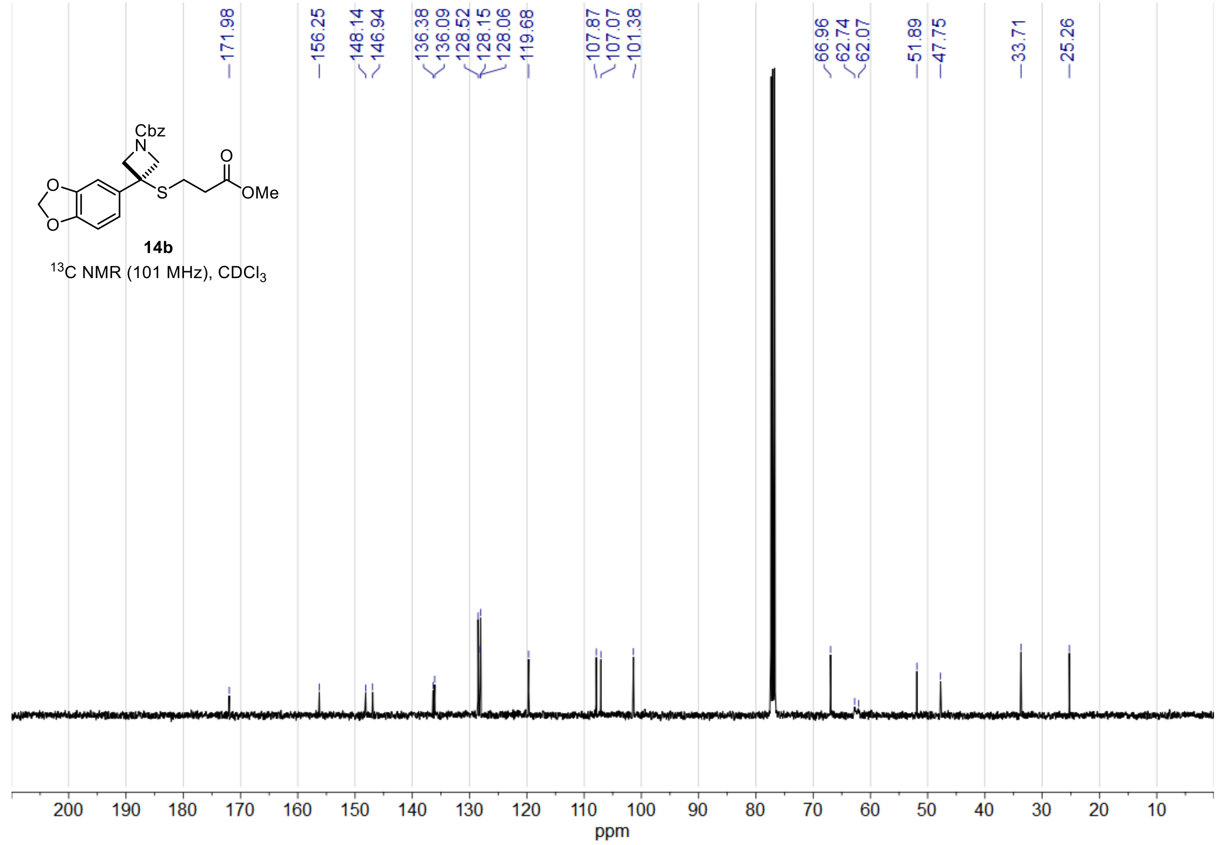

14b

^{13}C NMR (101 MHz), $CDCl_3$

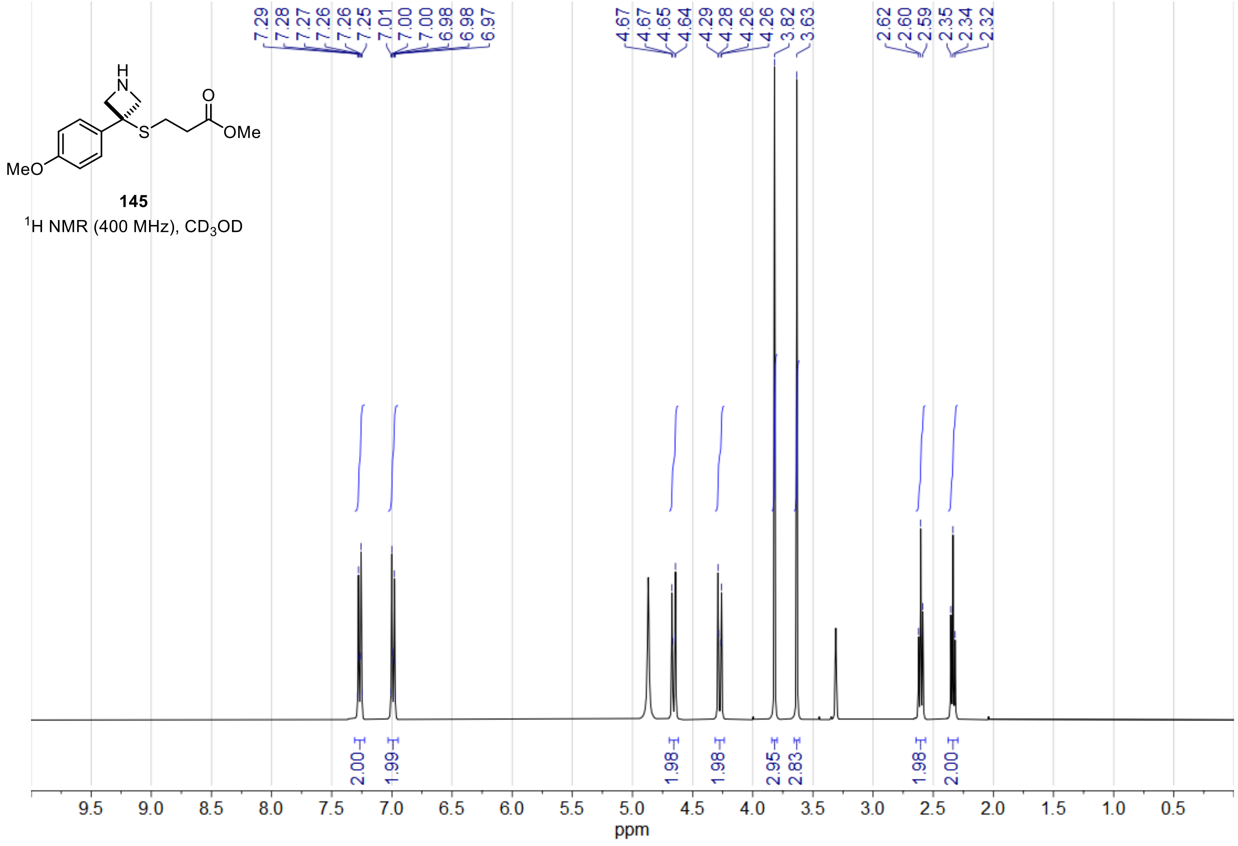
145
1H NMR (400 MHz), CD3OD
7.29 7.28 7.27 7.26 7.26 7.25 7.01 7.00 7.00 6.98 6.98 6.97
4.67 4.67 4.65 4.64 4.29 4.28 4.26 4.26 3.82 3.63
2.62 2.60 2.59 2.35 2.34 2.32
2.00 1.99 1.98 1.98 2.95 2.83 1.98 2.00
ppm

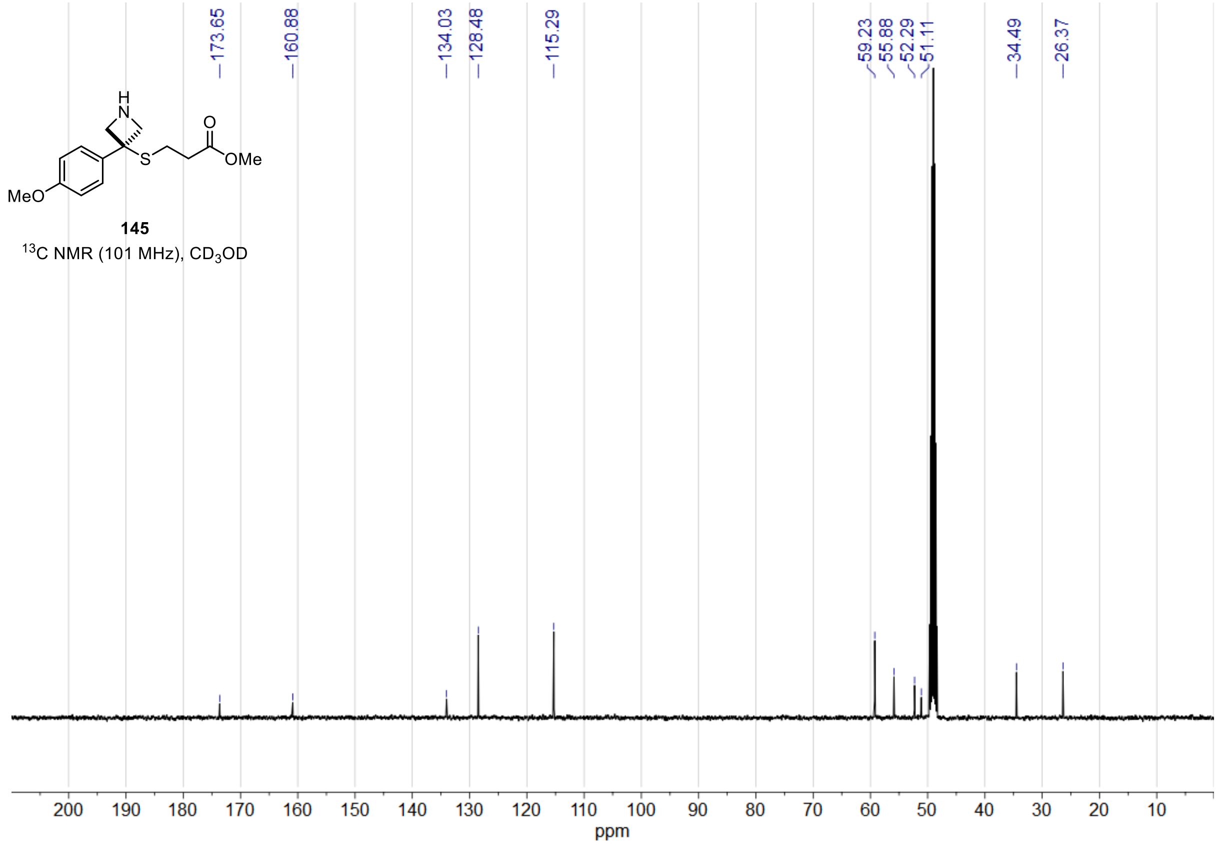
145
13C NMR (101 MHz), CD3OD
173.65 160.88 134.03 128.48 115.29 59.23 55.88 52.29 51.11 34.49 26.37
ppm

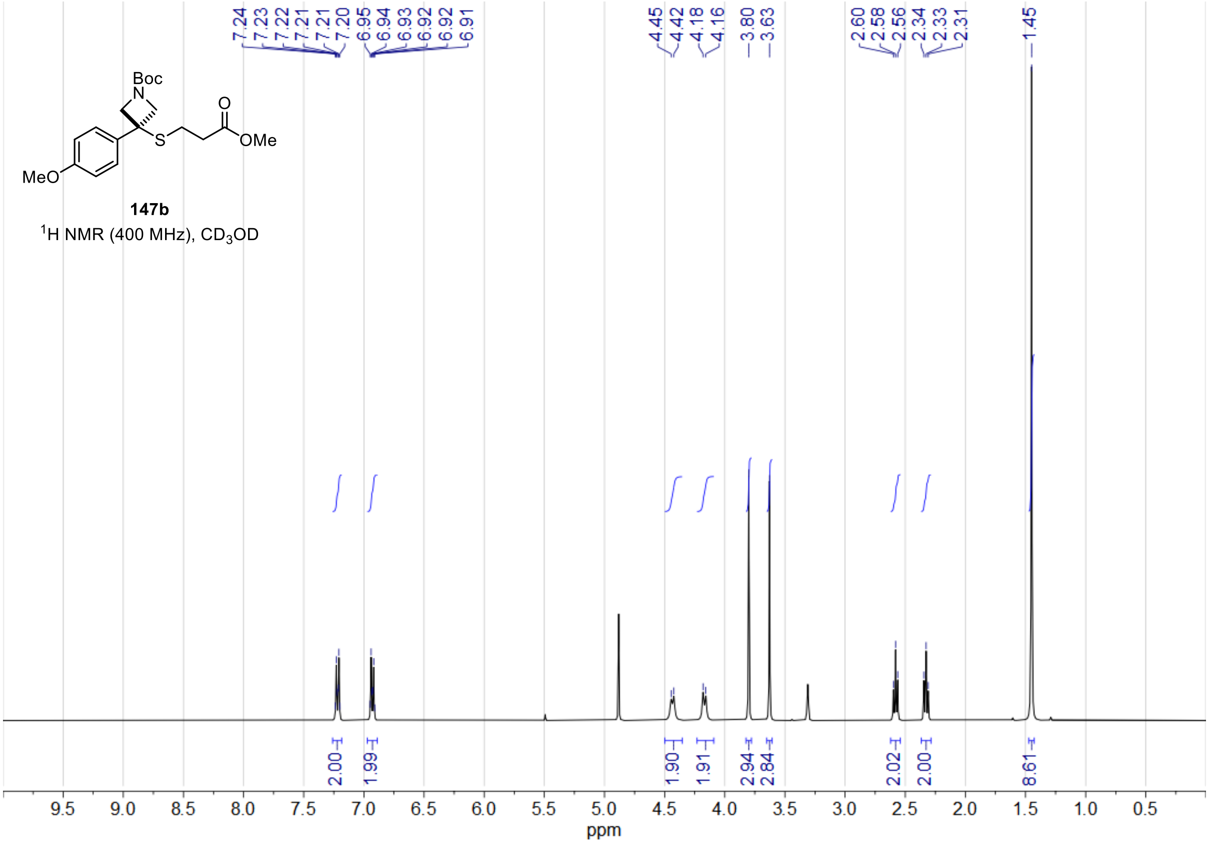
Boc
N
O
S
OMe
MeO
147b
1H NMR (400 MHz), CD3OD
7.24
7.23
7.22
7.21
7.21
7.20
6.95
6.94
6.93
6.92
6.92
6.91
4.45
4.42
4.18
4.16
3.80
3.63
2.60
2.58
2.56
2.34
2.33
2.31
1.45
2.00
1.99
1.90
1.91
2.94
2.84
2.02
2.00
8.61
ppm

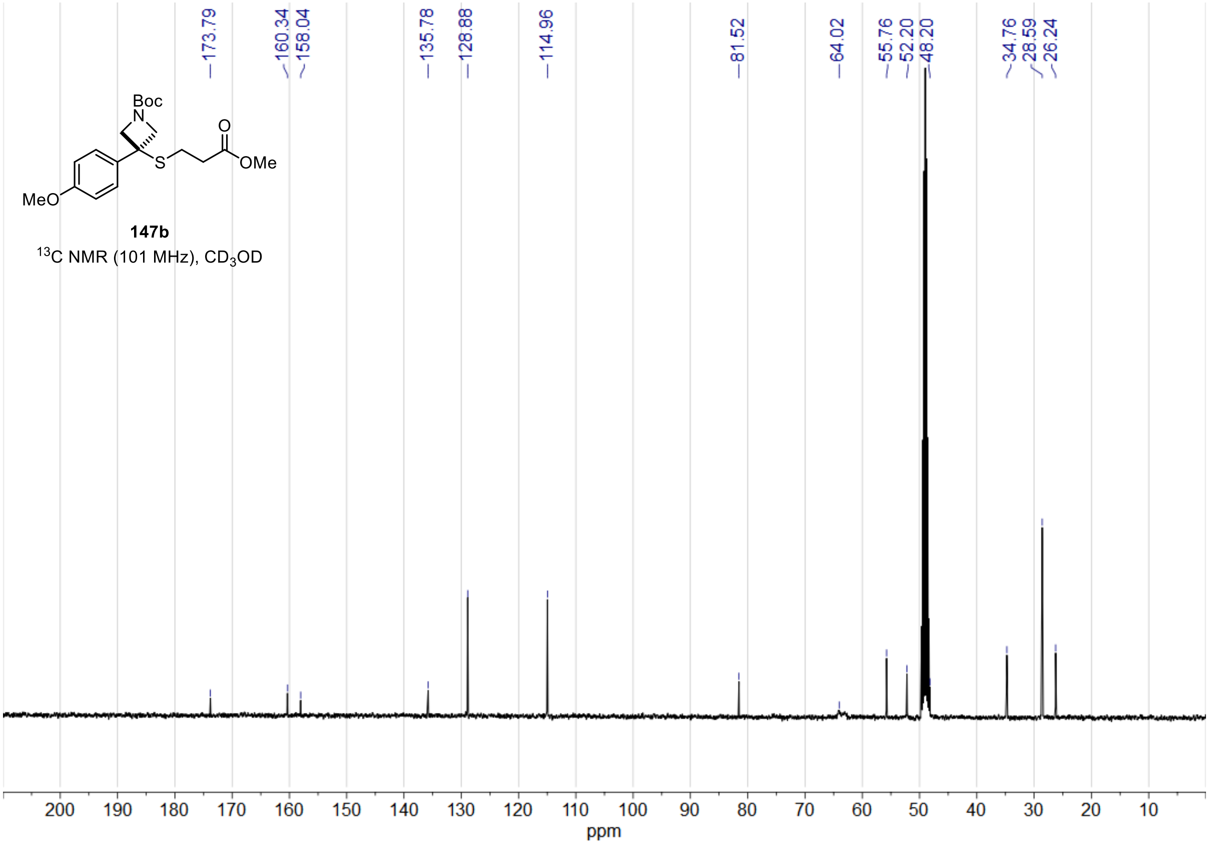
Boc
N
O
S
OMe
MeO
147b
13C NMR (101 MHz), CD3OD
173.79
160.34
158.04
135.78
128.88
114.96
81.52
64.02
55.76
52.20
48.20
34.76
28.59
26.24
ppm

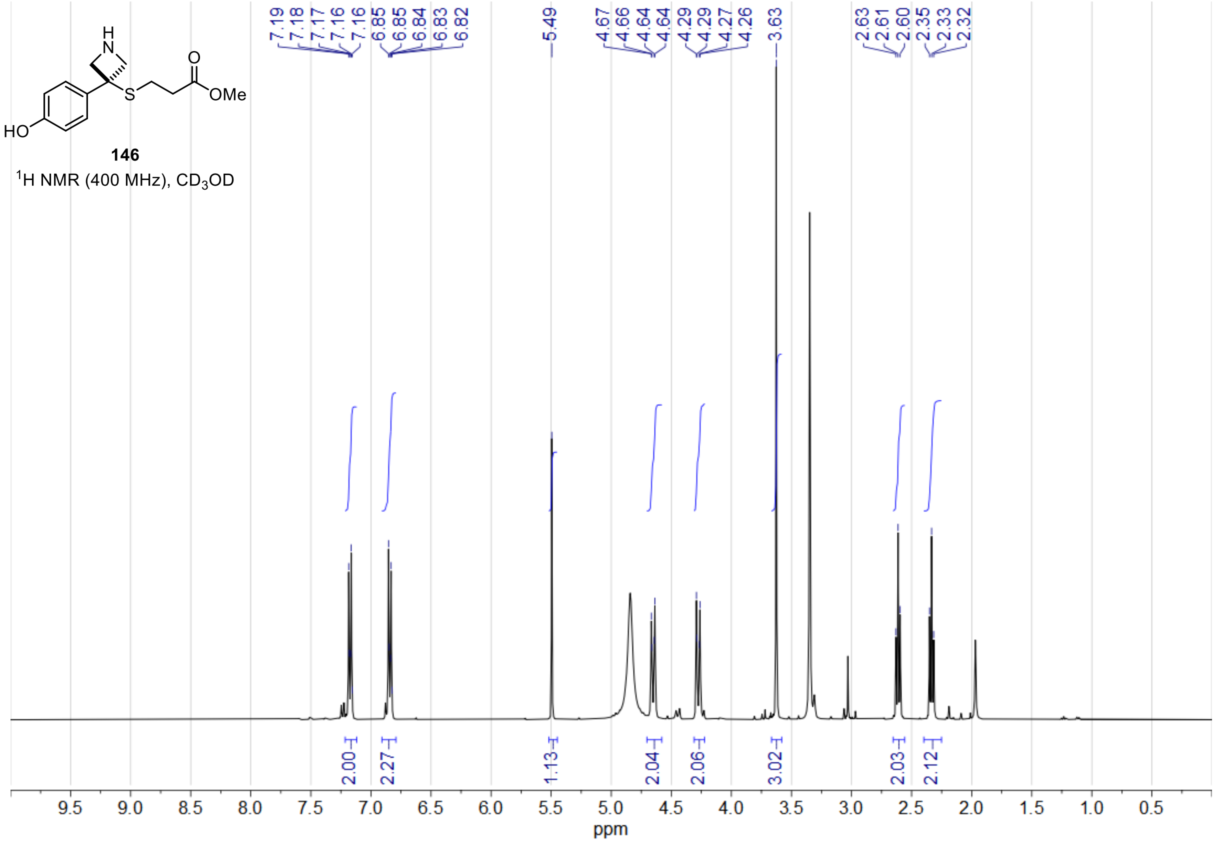

146

^{1}H NMR (400 MHz), CD_3OD

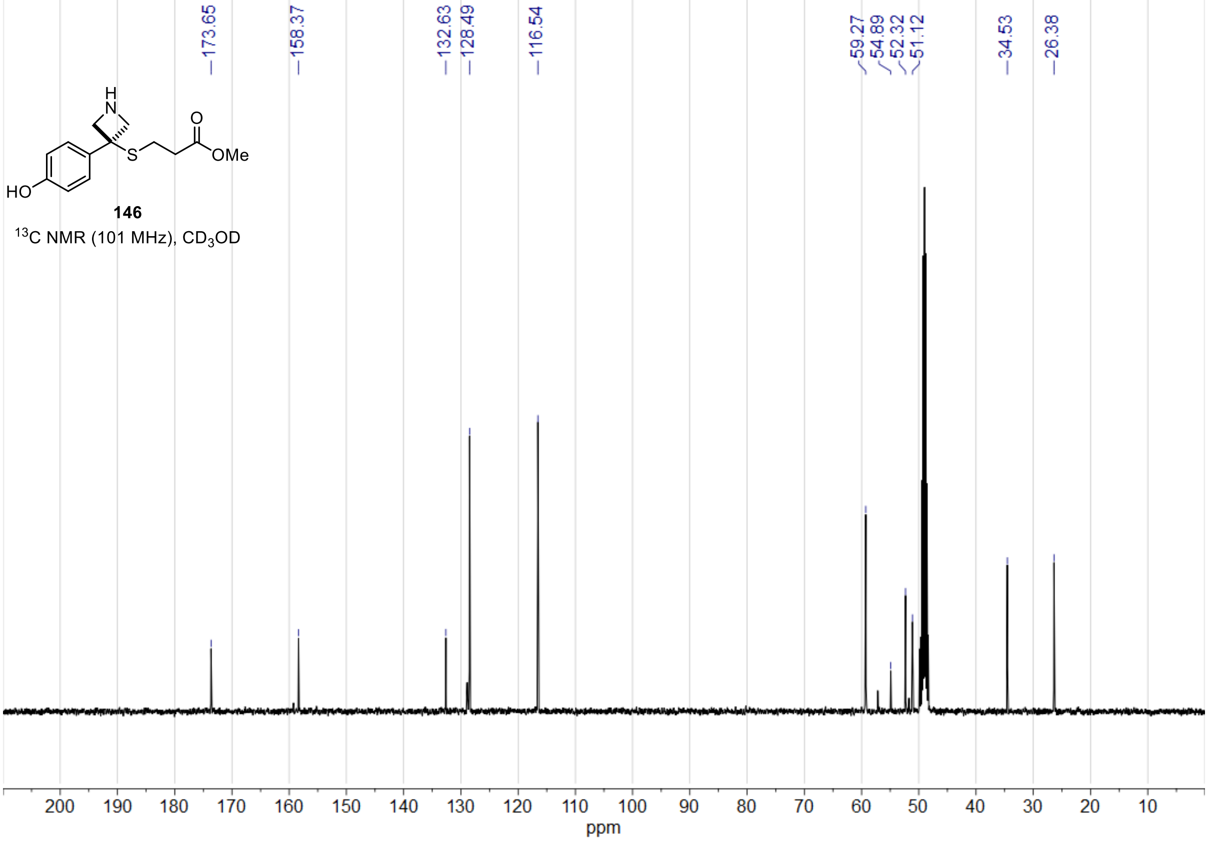

146

^{13}C NMR (101 MHz), CD_3OD

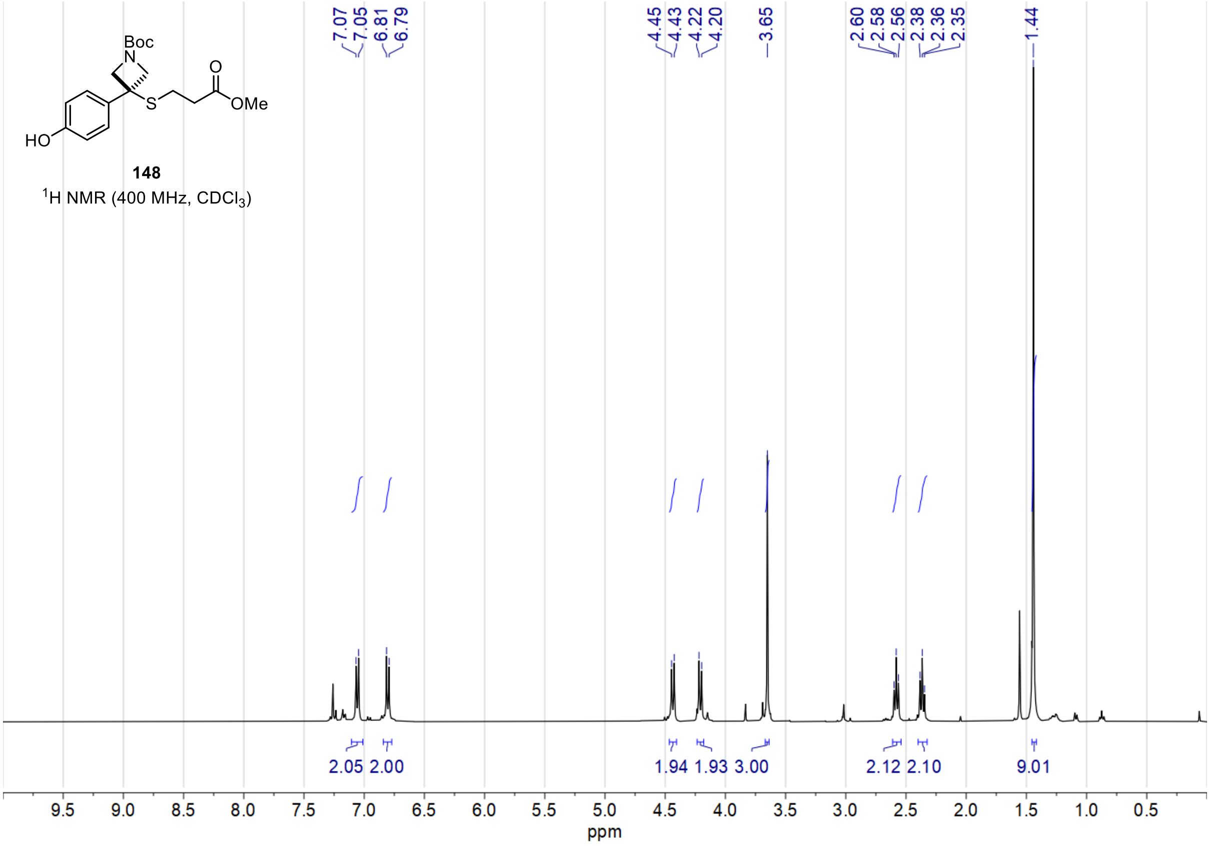
Boc
N
S
O
OMe
HO
148
1H NMR (400 MHz, CDCl3)
7.07
7.05
6.81
6.79
4.45
4.43
4.22
4.20
3.65
2.60
2.58
2.56
2.38
2.36
2.35
1.44
2.05
2.00
1.94
1.93
3.00
2.12
2.10
9.01
9.5 9.0 8.5 8.0 7.5 7.0 6.5 6.0 5.5 5.0 4.5 4.0 3.5 3.0 2.5 2.0 1.5 1.0 0.5
ppm

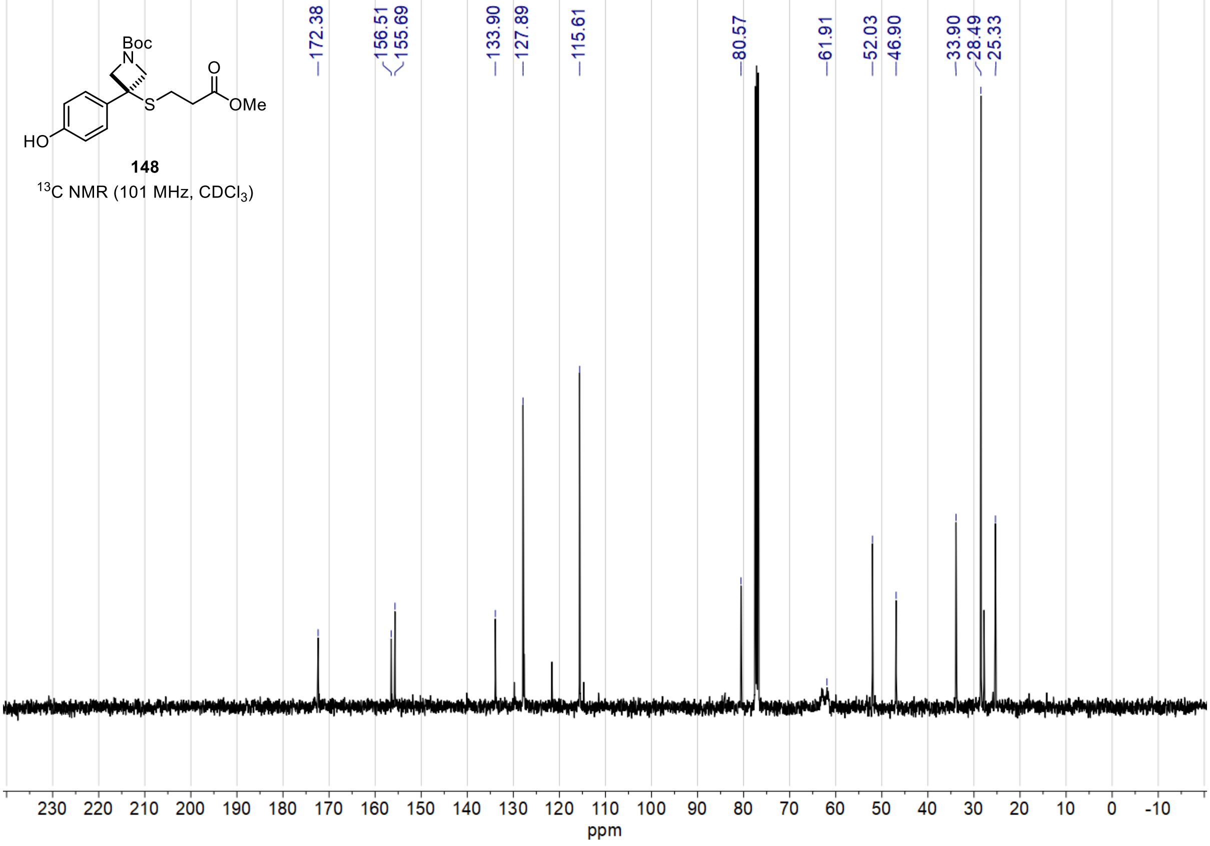
Boc
N
S
O
OMe
HO
148
13C NMR (101 MHz, CDCl3)
172.38
156.51
155.69
133.90
127.89
115.61
80.57
61.91
52.03
46.90
33.90
28.49
25.33
230 220 210 200 190 180 170 160 150 140 130 120 110 100 90 80 70 60 50 40 30 20 10 0 -10
ppm

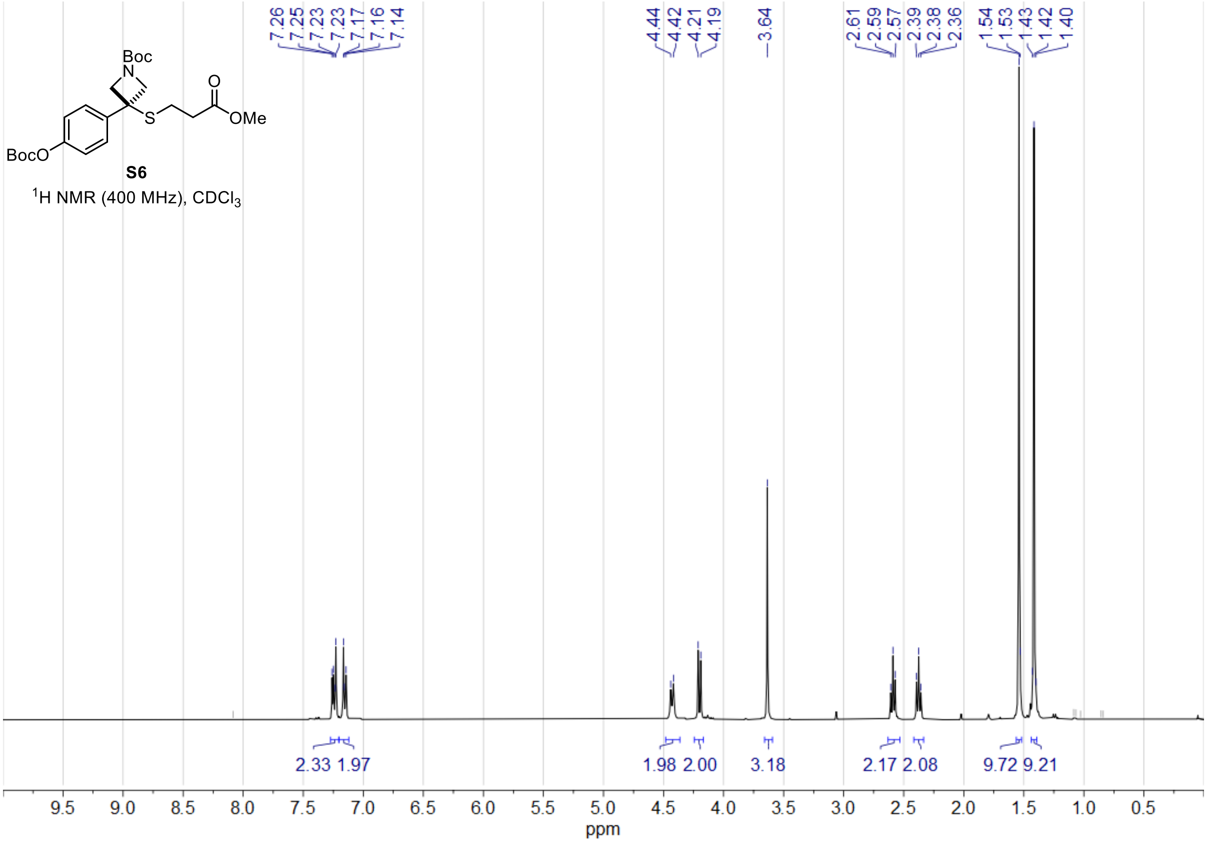
Boc
N
S
O
OMe
BocO
S6
^{1}H NMR (400 MHz), $CDCl_3$
7.26
7.25
7.23
7.23
7.17
7.16
7.14
4.44
4.42
4.21
4.19
3.64
2.61
2.59
2.57
2.39
2.38
2.36
1.54
1.53
1.43
1.42
1.40
2.33 1.97
1.98 2.00
3.18
2.17 2.08
9.72 9.21
9.5 9.0 8.5 8.0 7.5 7.0 6.5 6.0 5.5 5.0 4.5 4.0 3.5 3.0 2.5 2.0 1.5 1.0 0.5
ppm

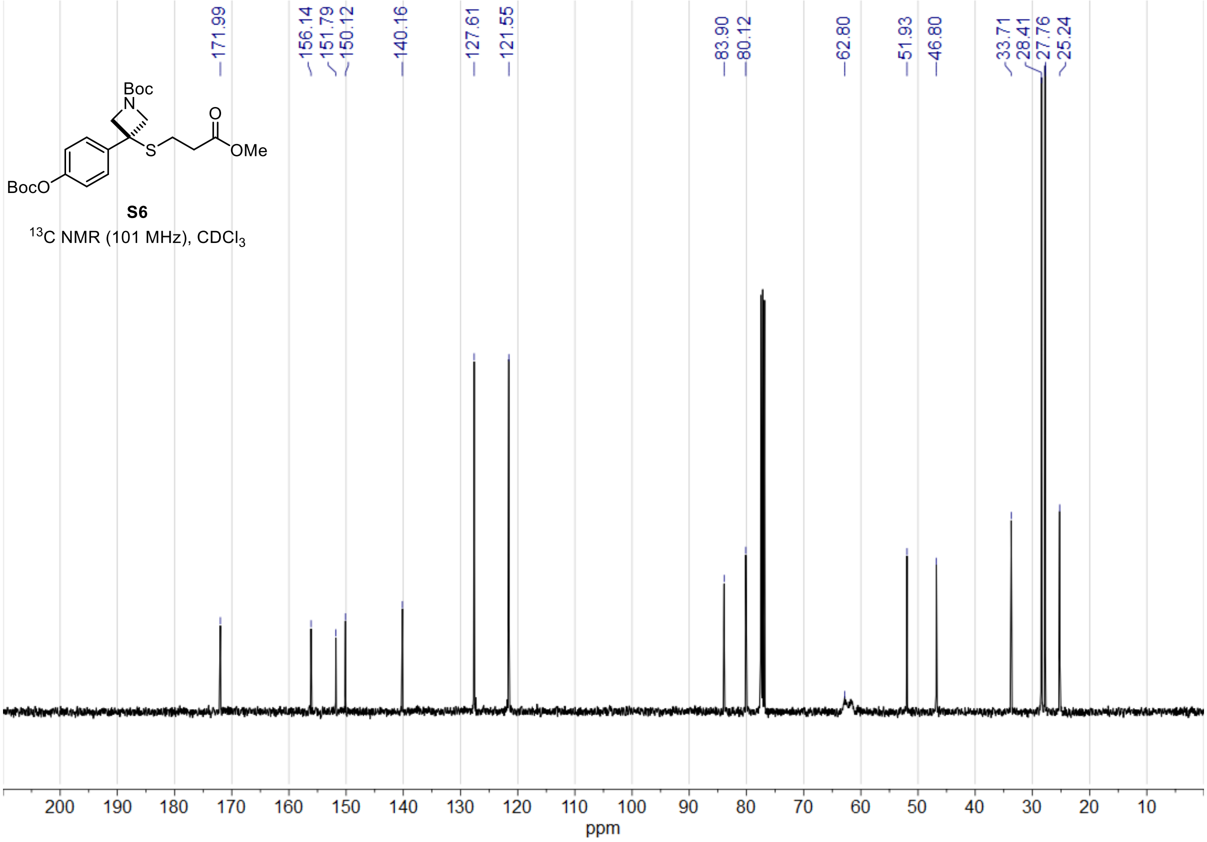
171.99
156.14
151.79
150.12
140.16
127.61
121.55
83.90
80.12
62.80
51.93
46.80
33.71
28.41
27.76
25.24
Boc
N
S
O
OMe
BocO
S6
^{13}C NMR (101 MHz), $CDCl_3$
200 190 180 170 160 150 140 130 120 110 100 90 80 70 60 50 40 30 20 10
ppm

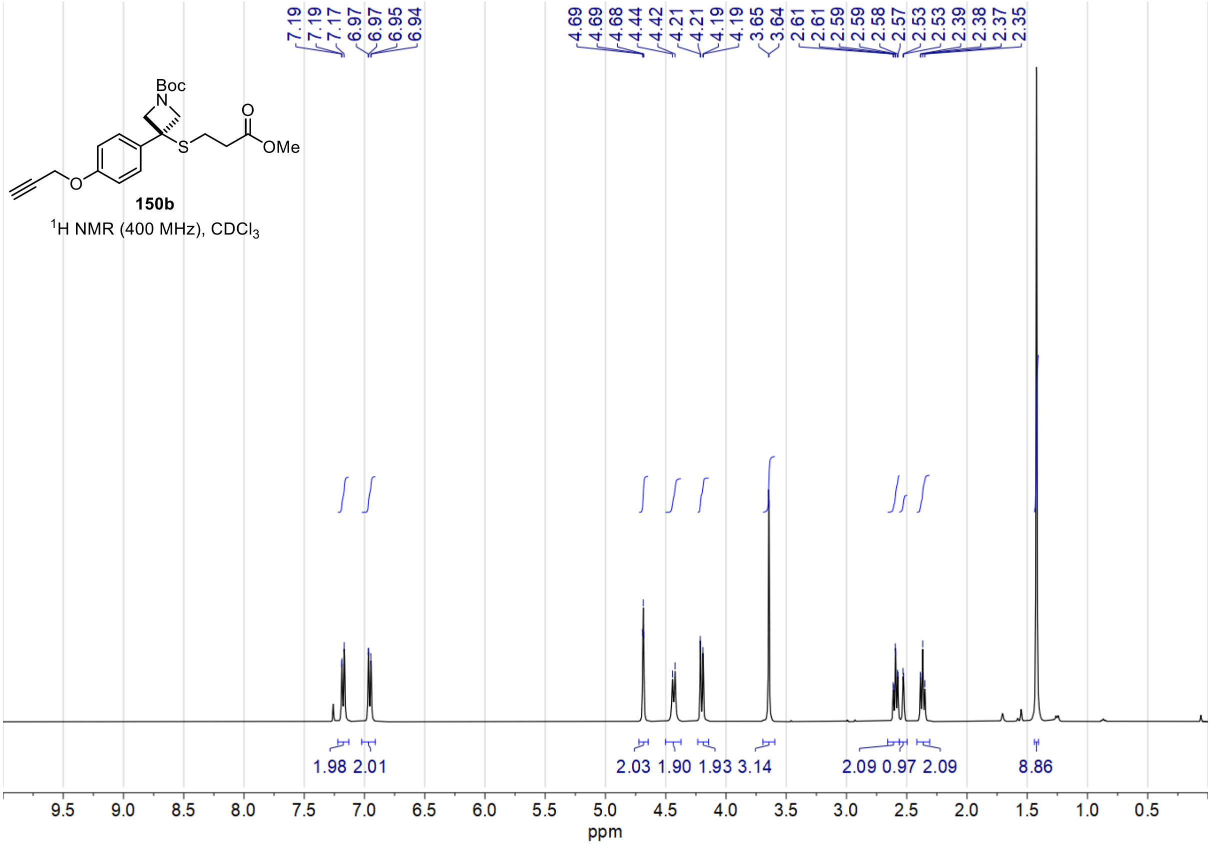
Boc
N
S
O
OMe
O
150b
^{1}H NMR (400 MHz), CDCl$_3$
7.19 7.19 7.17 6.97 6.97 6.95 6.94
4.69 4.69 4.68 4.44 4.42 4.21 4.21 4.19 4.19 3.65 3.64 2.61 2.61 2.59 2.59 2.58 2.57 2.53 2.53 2.39 2.38 2.37 2.35
1.98 2.01 2.03 1.90 1.93 3.14 2.09 0.97 2.09 8.86
9.5 9.0 8.5 8.0 7.5 7.0 6.5 6.0 5.5 5.0 4.5 4.0 3.5 3.0 2.5 2.0 1.5 1.0 0.5
ppm

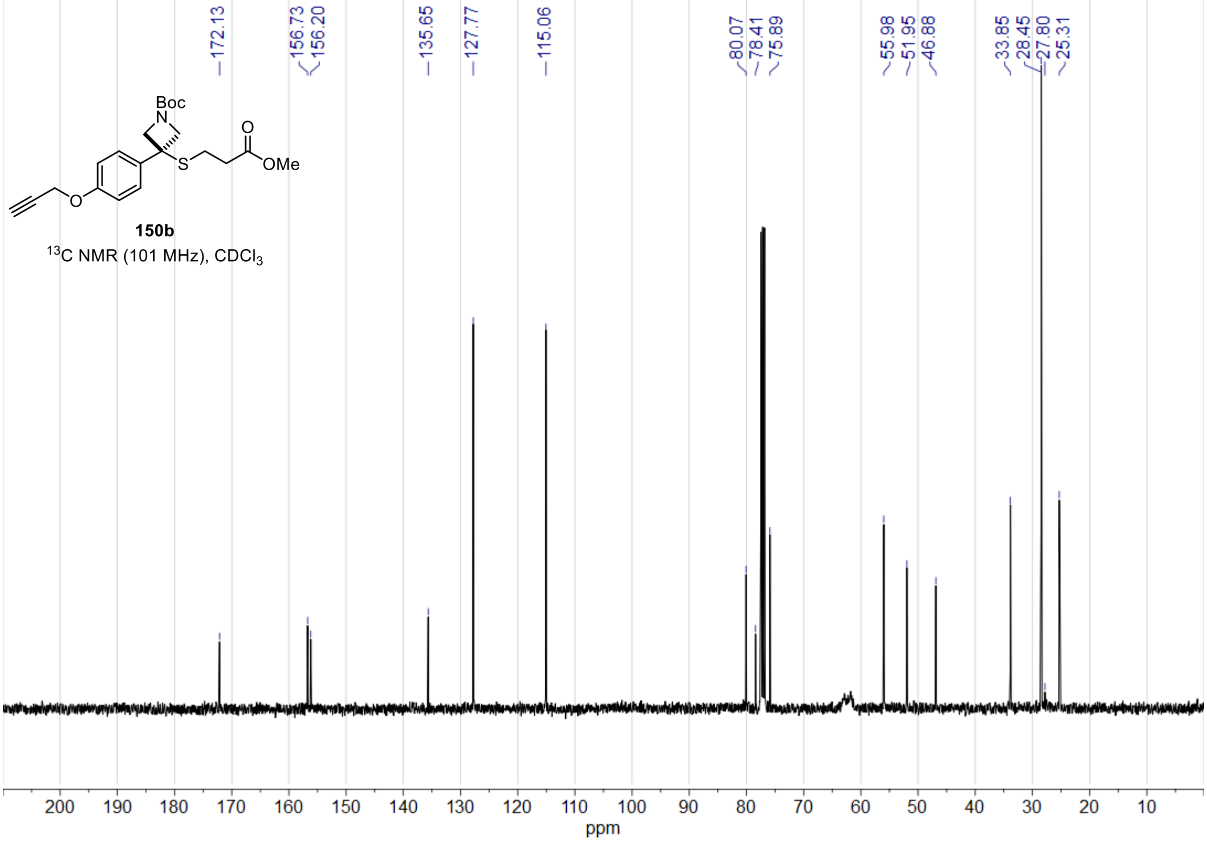
Boc
N
S
O
OMe
O
150b
^{13}C NMR (101 MHz), CDCl$_3$
172.13 156.73 156.20 135.65 127.77 115.06 80.07 78.41 75.89 55.98 51.95 46.88 33.85 28.45 27.80 25.31
200 190 180 170 160 150 140 130 120 110 100 90 80 70 60 50 40 30 20 10
ppm

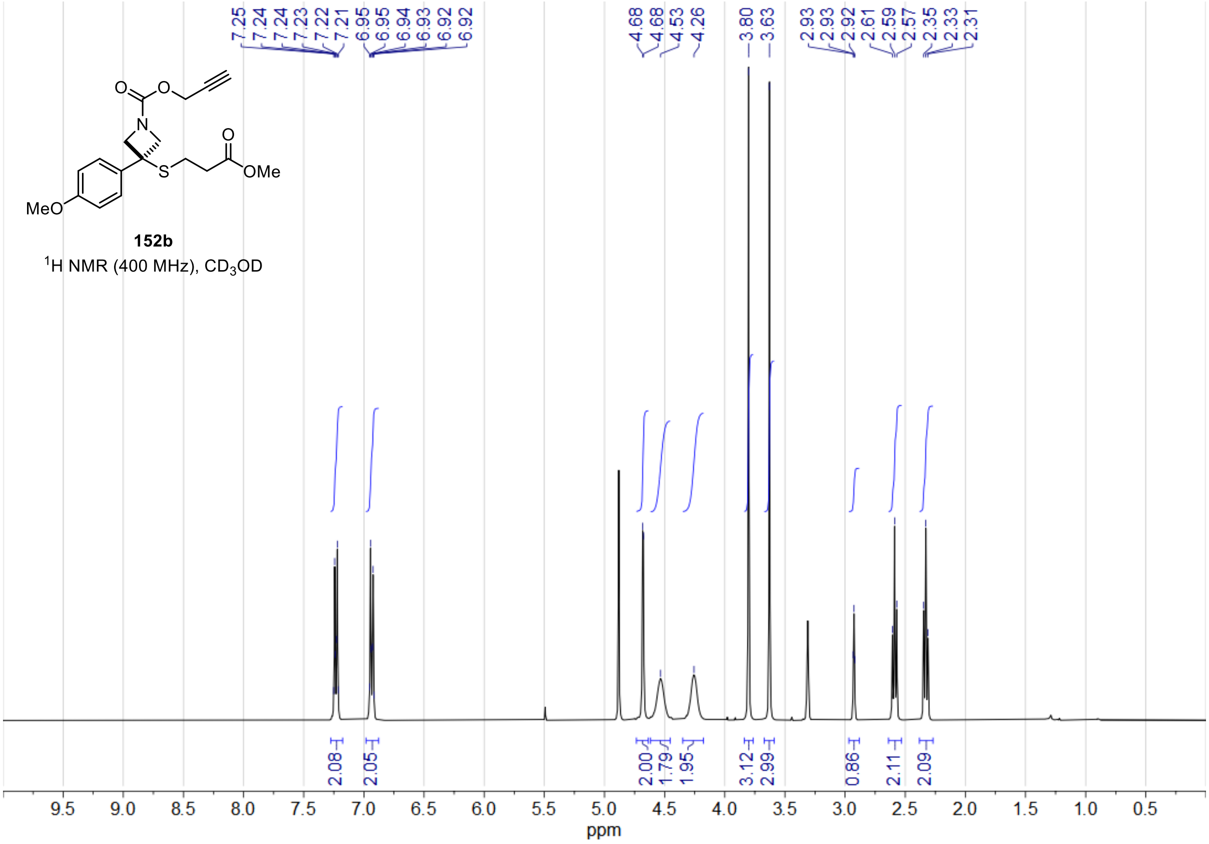

152b

^{1}H NMR (400 MHz), CD_3OD

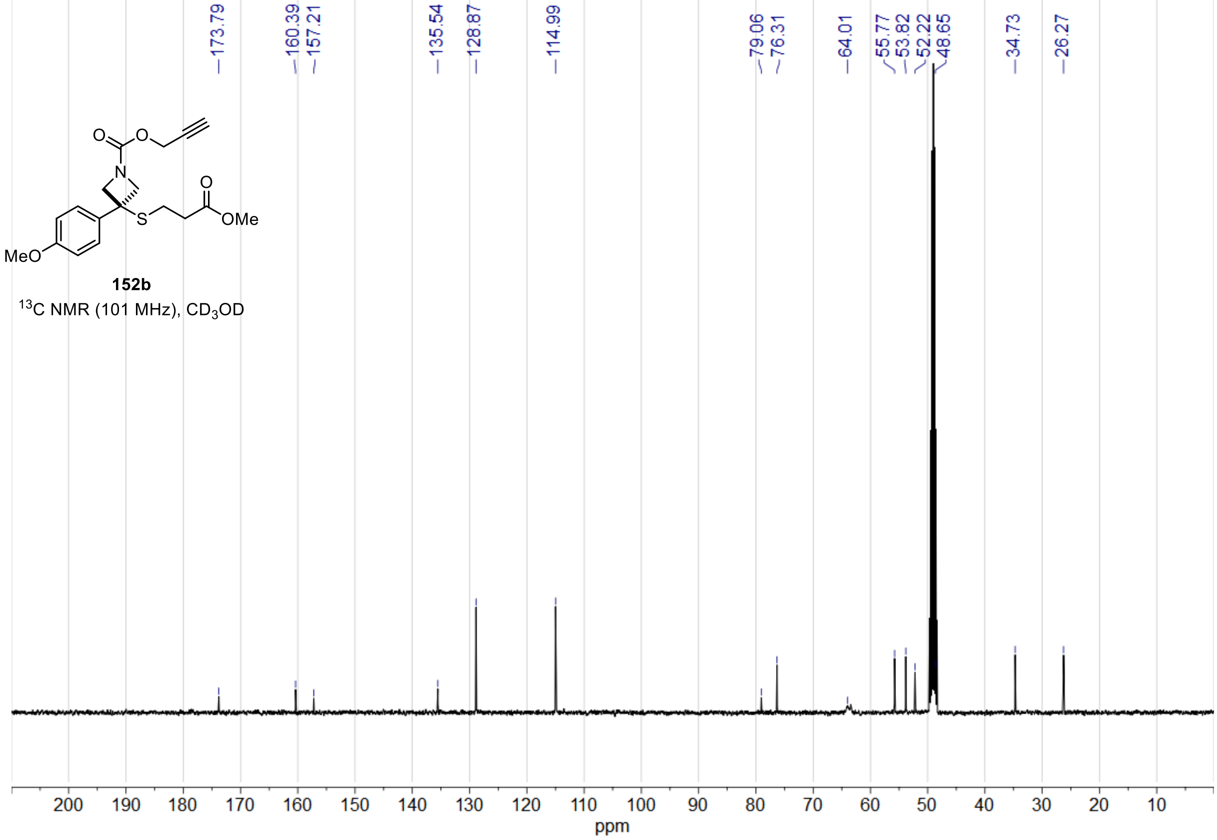

152b

^{13}C NMR (101 MHz), CD_3OD

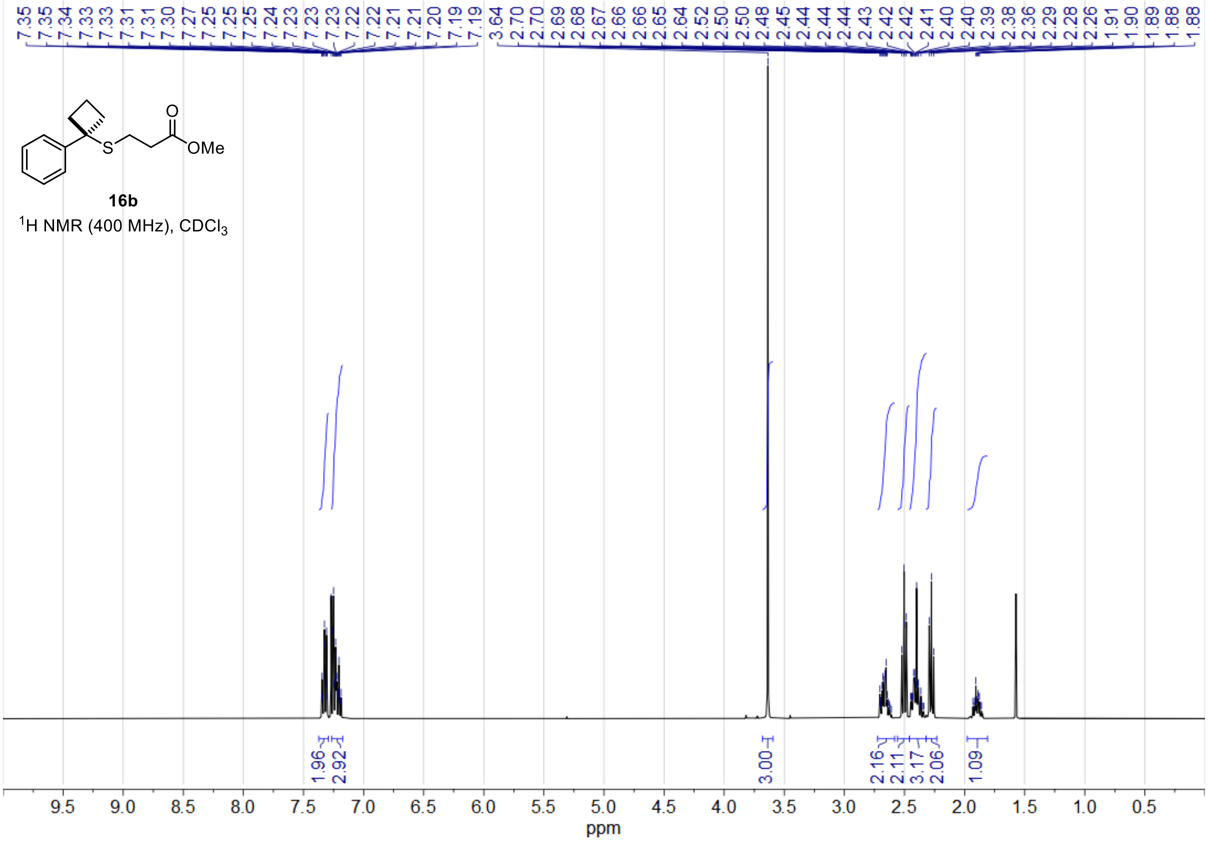
7.35 7.35 7.34 7.33 7.33 7.31 7.31 7.30 7.27 7.25 7.25 7.25 7.24 7.23 7.23 7.23 7.22 7.22 7.21 7.21 7.20 7.19 3.64 2.70 2.70 2.69 2.68 2.67 2.66 2.66 2.65 2.64 2.52 2.50 2.50 2.48 2.45 2.44 2.44 2.44 2.43 2.42 2.42 2.41 2.40 2.40 2.39 2.38 2.36 2.29 2.28 2.26 1.91 1.90 1.89 1.88 1.88
O
S
OMe
16b
^{1}H NMR (400 MHz), $CDCl_3$
1.96
2.92
3.00
2.16
2.11
3.17
2.06
1.09
9.5 9.0 8.5 8.0 7.5 7.0 6.5 6.0 5.5 5.0 4.5 4.0 3.5 3.0 2.5 2.0 1.5 1.0 0.5
ppm

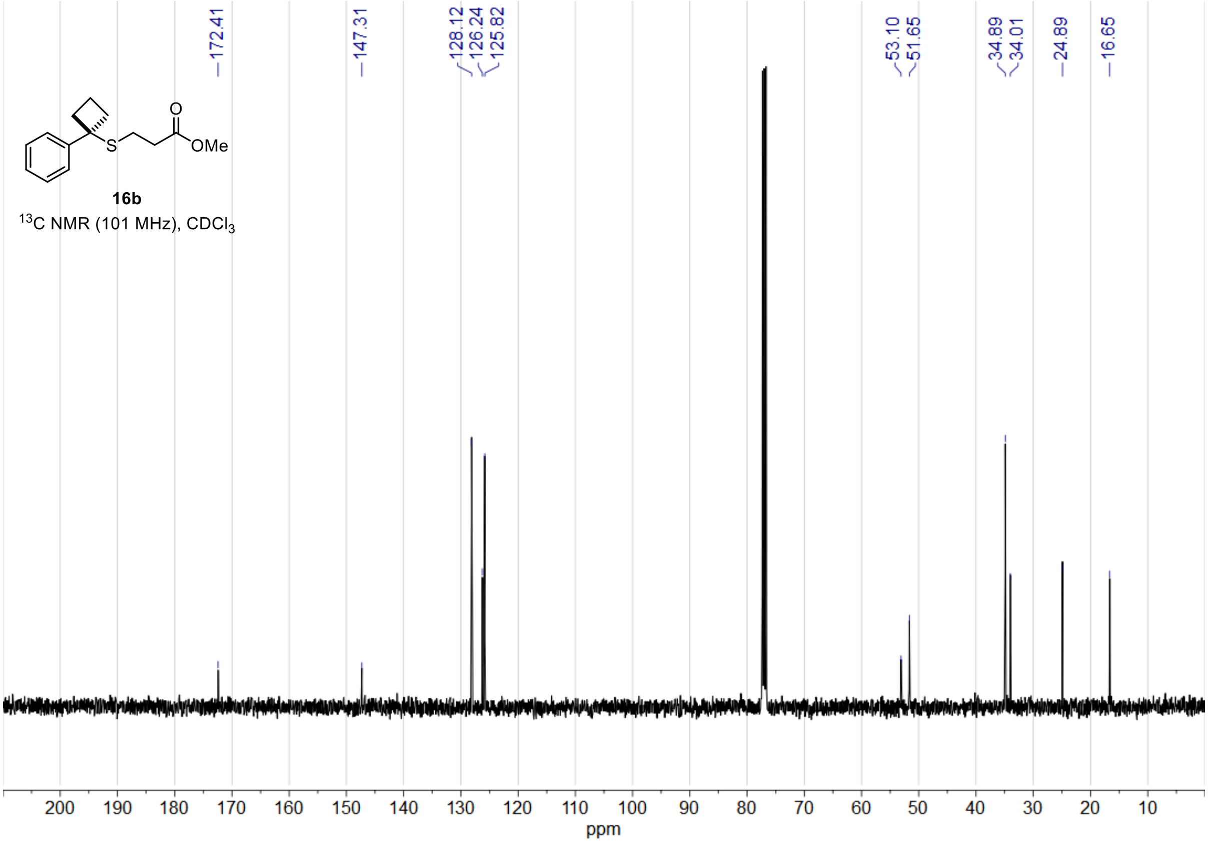
172.41
147.31
128.12 126.24 125.82
53.10 51.65
34.89 34.01
24.89
16.65
O
S
OMe
16b
^{13}C NMR (101 MHz), $CDCl_3$
200 190 180 170 160 150 140 130 120 110 100 90 80 70 60 50 40 30 20 10
ppm

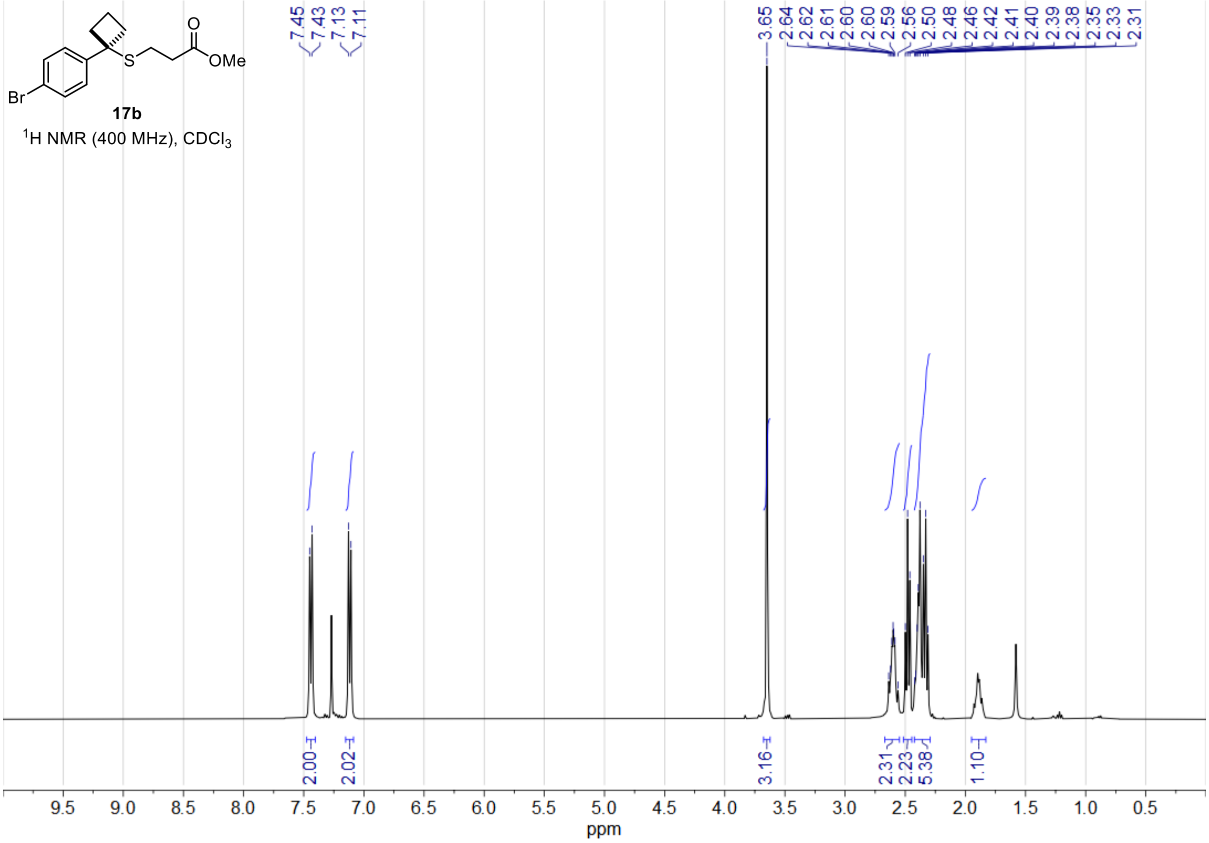
Br
S
O
OMe
17b
^{1}H NMR (400 MHz), CDCl$_3$
7.45
7.43
7.13
7.11
3.65
2.64
2.62
2.61
2.60
2.60
2.59
2.56
2.50
2.48
2.46
2.42
2.41
2.40
2.39
2.38
2.35
2.33
2.31
2.00
2.02
3.16
2.31
2.23
5.38
1.10
9.5 9.0 8.5 8.0 7.5 7.0 6.5 6.0 5.5 5.0 4.5 4.0 3.5 3.0 2.5 2.0 1.5 1.0 0.5
ppm

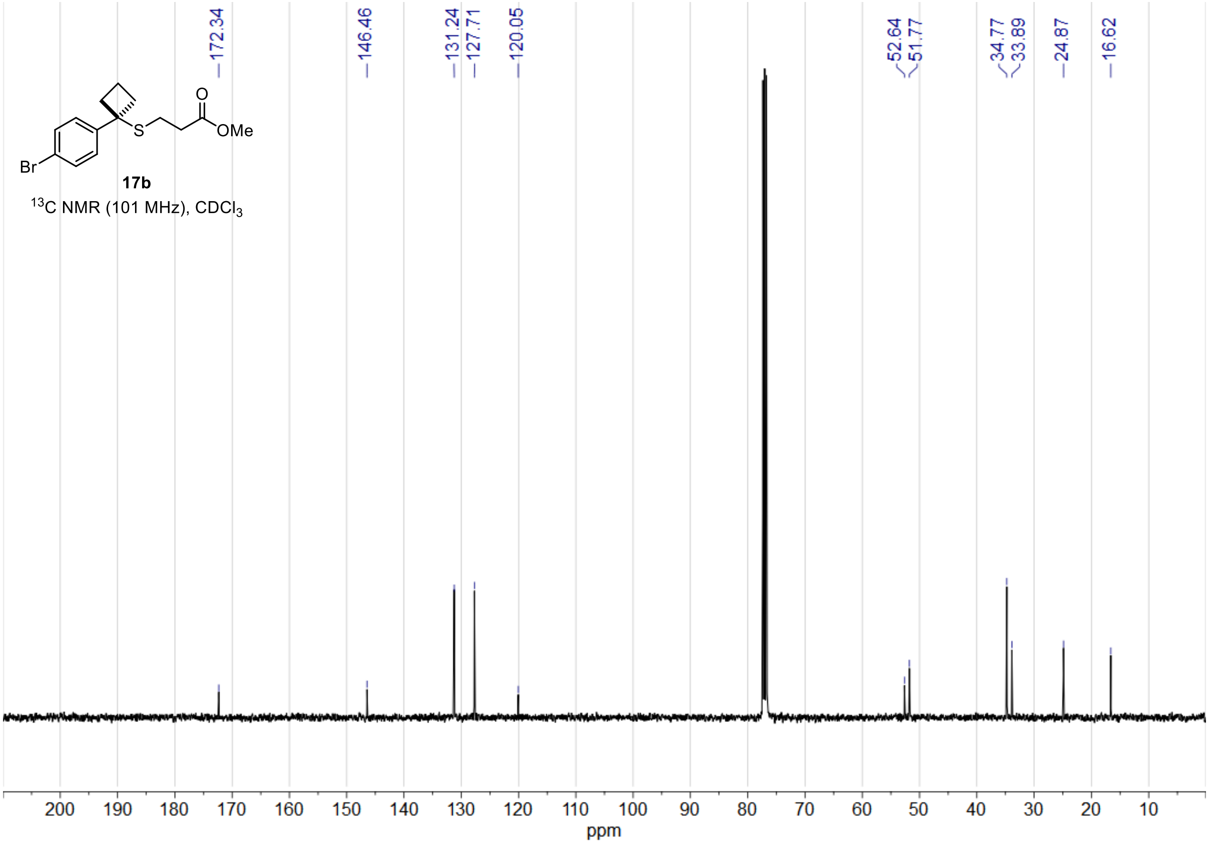
Br
S
O
OMe
17b
^{13}C NMR (101 MHz), CDCl$_3$
172.34
146.46
131.24
127.71
120.05
52.64
51.77
34.77
33.89
24.87
16.62
200 190 180 170 160 150 140 130 120 110 100 90 80 70 60 50 40 30 20 10
ppm

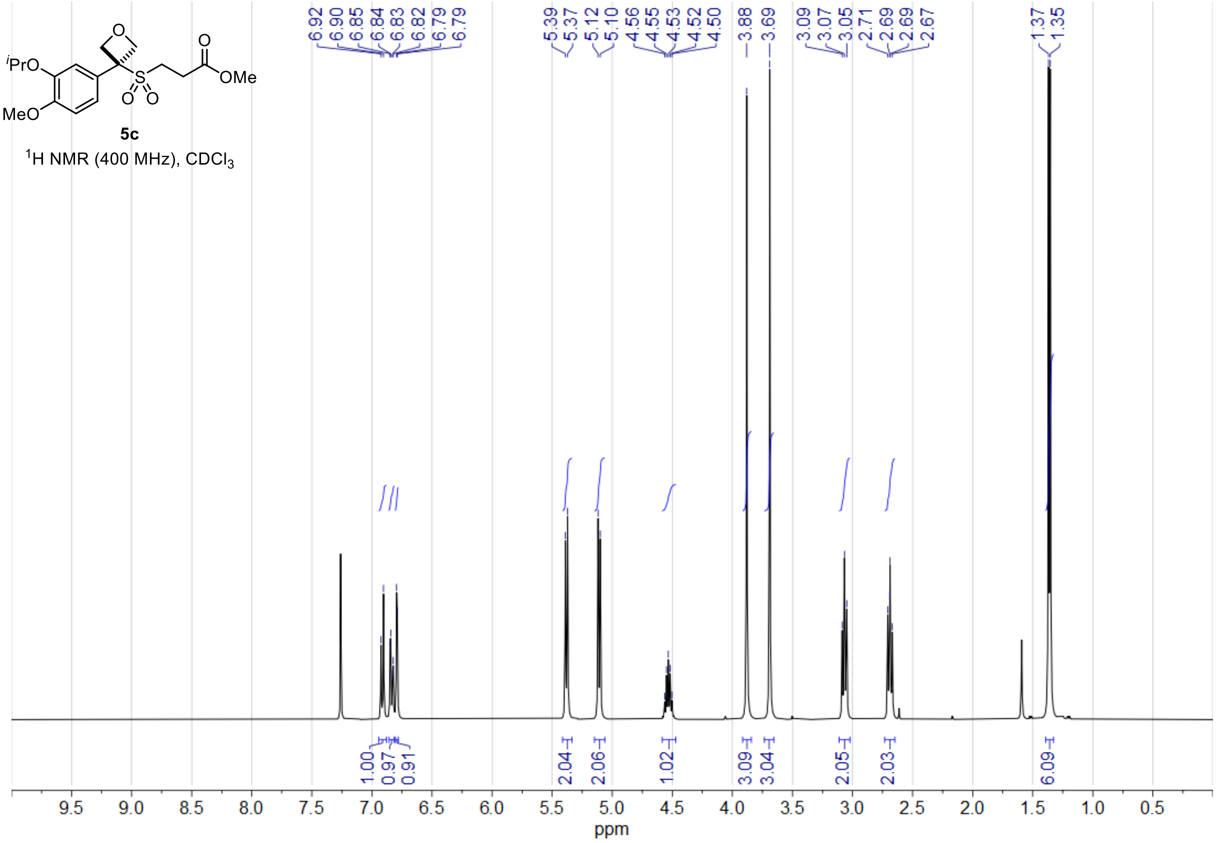

iPrO
MeO
O
OMe
5c
1H NMR (400 MHz), CDCl3
6.92 6.90 6.85 6.84 6.83 6.82 6.79 6.79
5.39 5.37 5.12 5.10
4.56 4.55 4.53 4.52 4.50
3.88
3.69
3.09 3.07 3.05 2.71 2.69 2.69 2.67
1.37 1.35
1.00 0.97 0.91 2.04 2.06 1.02 3.09 3.04 2.05 2.03 6.09
9.5 9.0 8.5 8.0 7.5 7.0 6.5 6.0 5.5 5.0 4.5 4.0 3.5 3.0 2.5 2.0 1.5 1.0 0.5
ppm

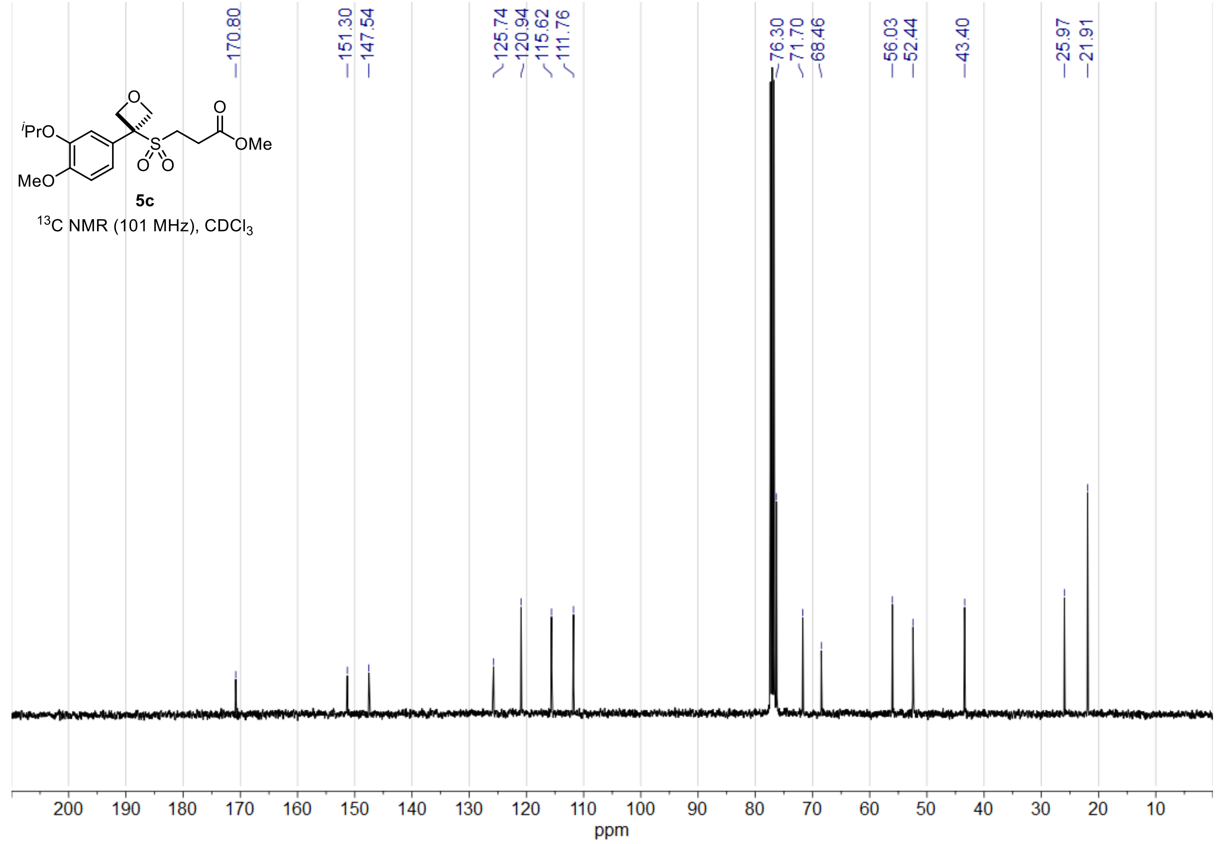

iPrO
MeO
O
OMe
5c
13C NMR (101 MHz), CDCl3
170.80
151.30 147.54
125.74 120.94 115.62 111.76
76.30 71.70 68.46
56.03 52.44
43.40
25.97 21.91
200 190 180 170 160 150 140 130 120 110 100 90 80 70 60 50 40 30 20 10
ppm

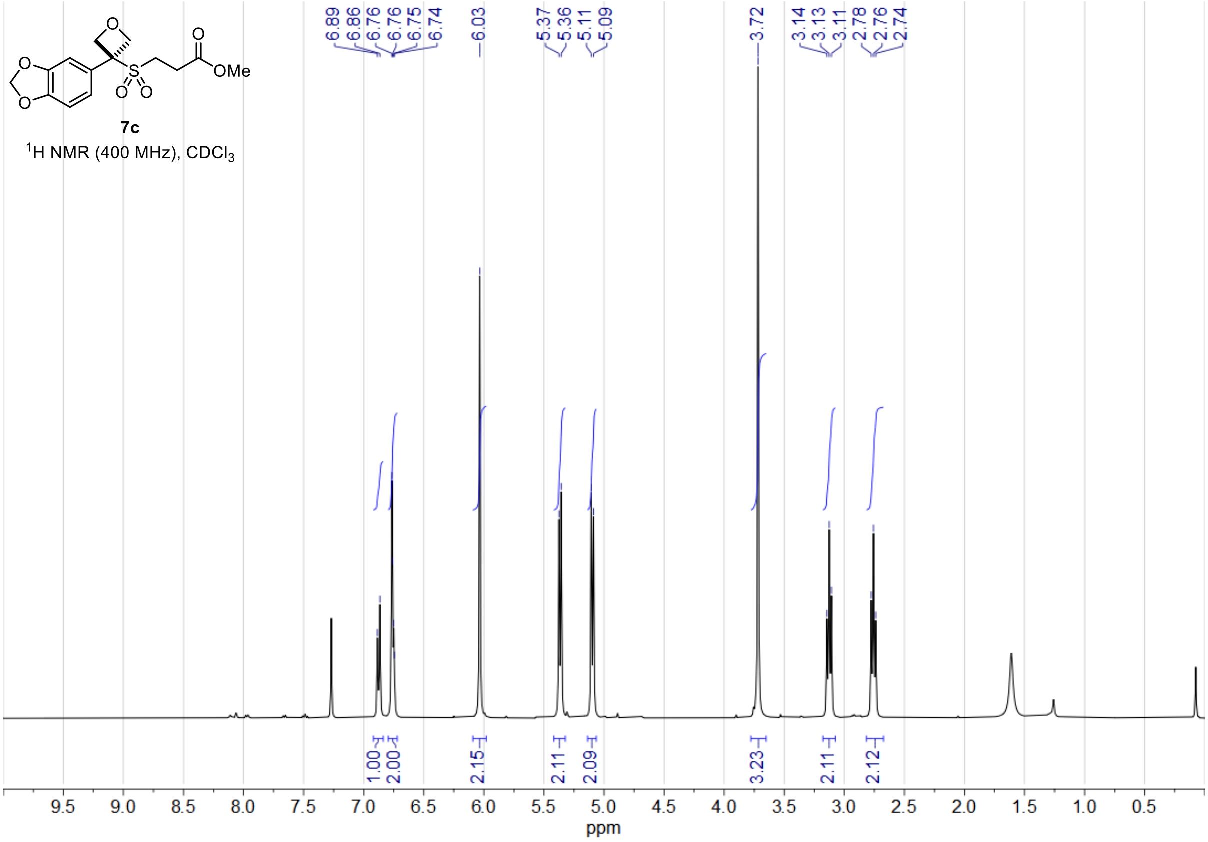
6.89
6.86
6.76
6.76
6.75
6.74
6.03
5.37
5.36
5.11
5.09
3.72
3.14
3.13
3.11
2.78
2.76
2.74
OMe
7c
1H NMR (400 MHz), CDCl3
1.00
2.00
2.15
2.11
2.09
3.23
2.11
2.12
9.5 9.0 8.5 8.0 7.5 7.0 6.5 6.0 5.5 5.0 4.5 4.0 3.5 3.0 2.5 2.0 1.5 1.0 0.5
ppm

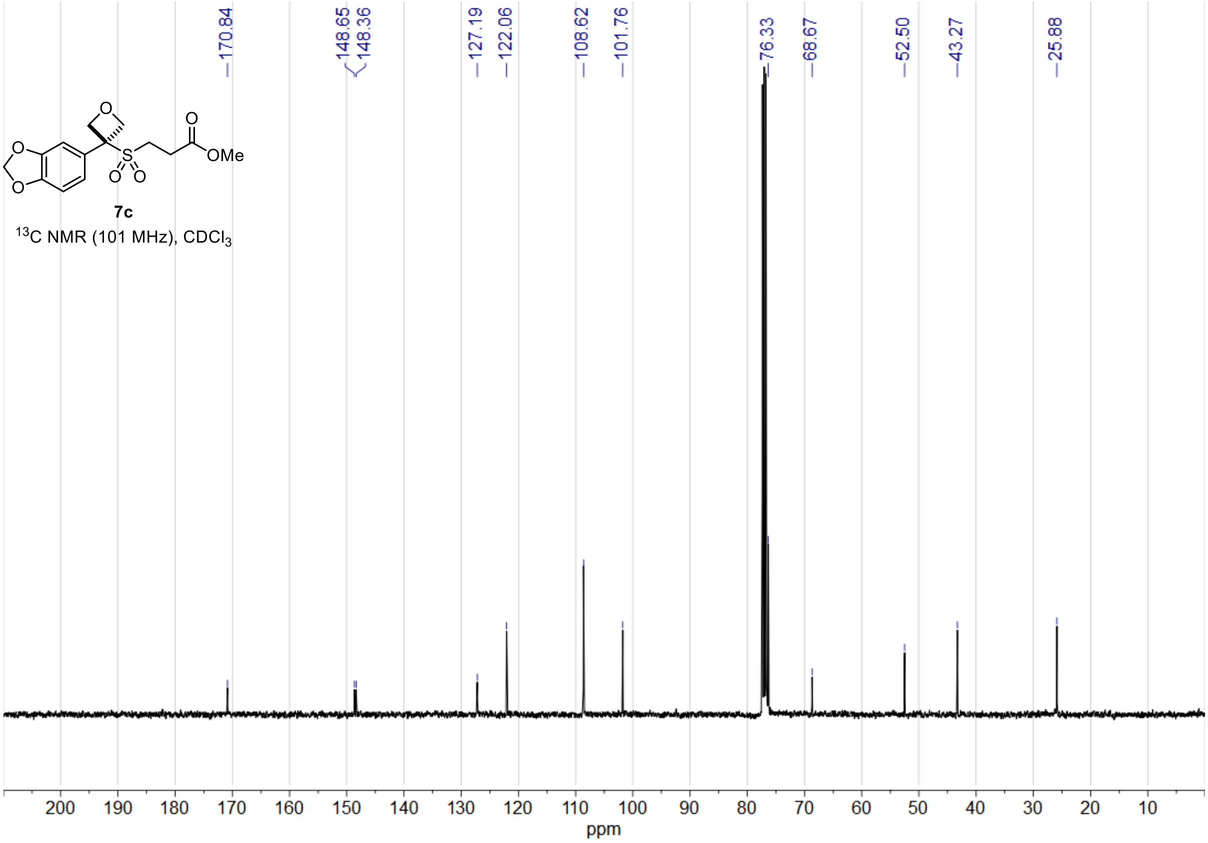
170.84
148.65
148.36
127.19
122.06
108.62
101.76
76.33
68.67
52.50
43.27
25.88
OMe
7c
13C NMR (101 MHz), CDCl3
200 190 180 170 160 150 140 130 120 110 100 90 80 70 60 50 40 30 20 10
ppm

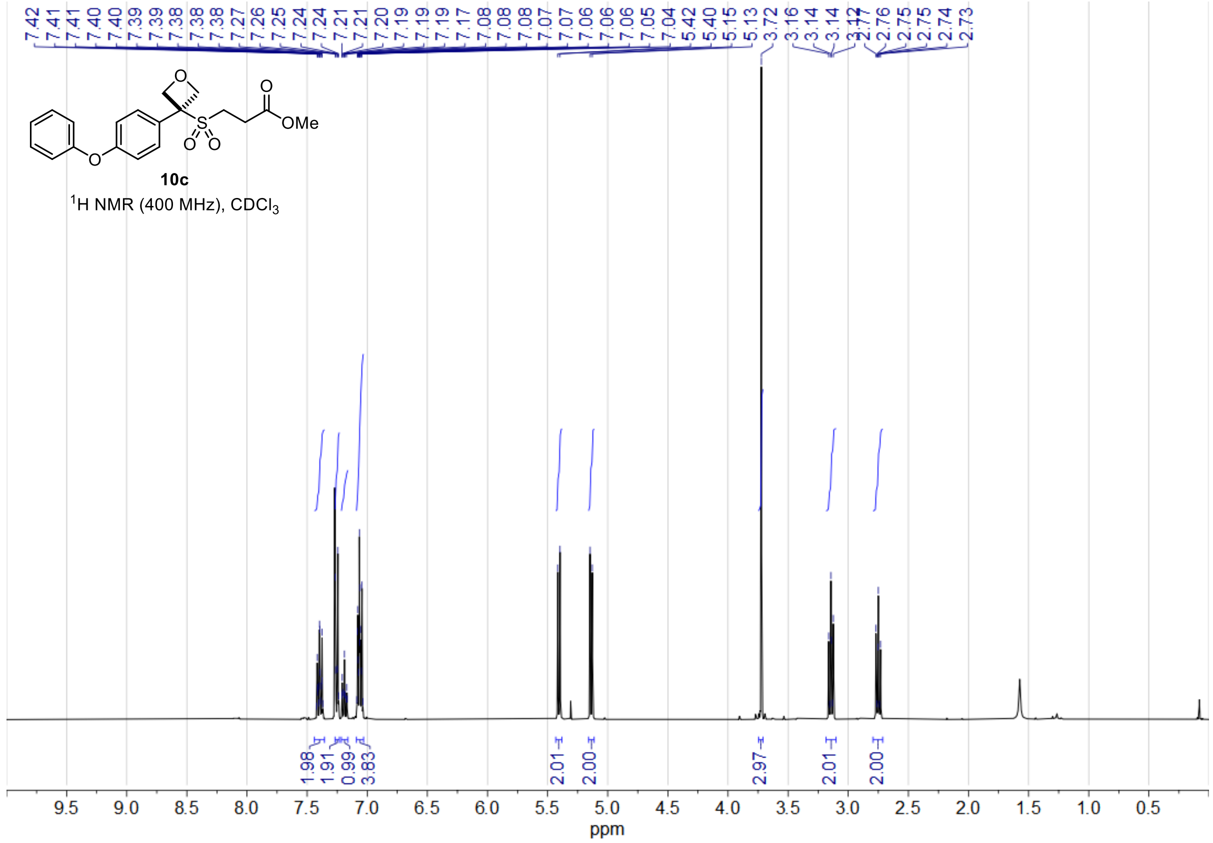
7.42
7.41
7.41
7.40
7.40
7.39
7.39
7.38
7.38
7.38
7.27
7.26
7.25
7.24
7.24
7.21
7.21
7.20
7.19
7.19
7.19
7.17
7.17
7.08
7.08
7.08
7.07
7.07
7.06
7.06
7.06
7.05
7.04
5.42
5.40
5.15
5.13
3.72
3.16
3.14
3.14
3.12
2.77
2.76
2.75
2.75
2.74
2.73
OMe
O
O
S
O
O
O
10c
1H NMR (400 MHz), CDCl3
1.98
1.91
0.99
3.83
2.01
2.00
2.97
2.01
2.00
9.5
9.0
8.5
8.0
7.5
7.0
6.5
6.0
5.5
5.0
4.5
4.0
3.5
3.0
2.5
2.0
1.5
1.0
0.5
ppm

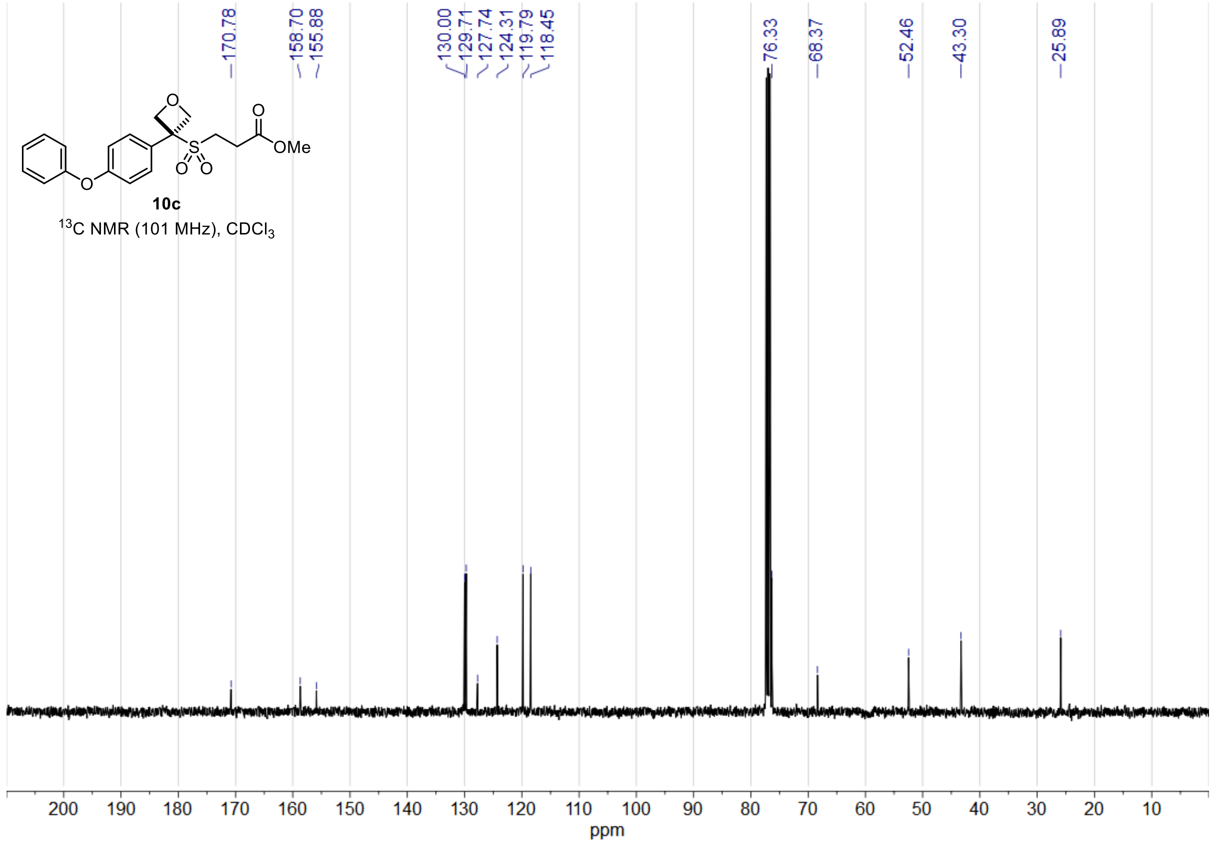
170.78
158.70
155.88
130.00
129.71
127.74
124.31
119.79
118.45
76.33
68.37
52.46
43.30
25.89
OMe
10c
13C NMR (101 MHz), CDCl3
200
190
180
170
160
150
140
130
120
110
100
90
80
70
60
50
40
30
20
10
ppm

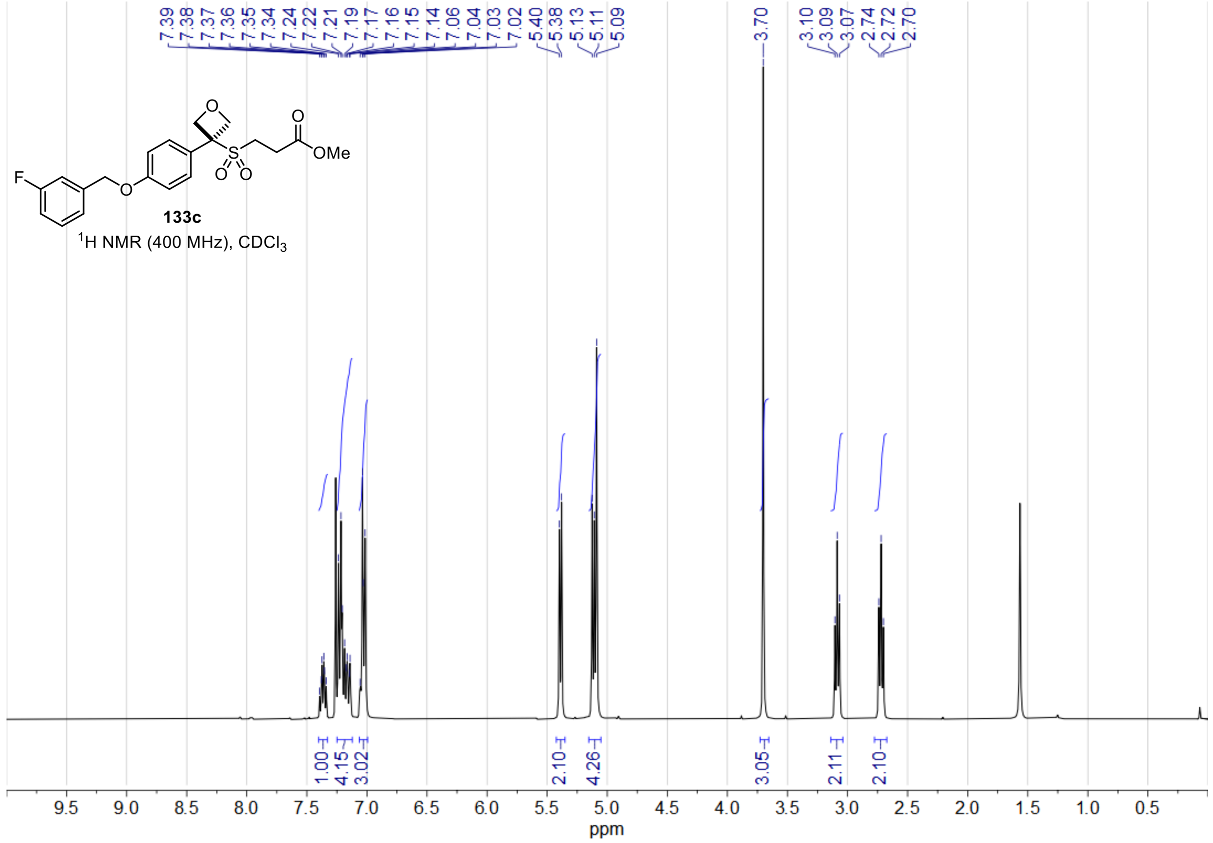
7.39 7.38 7.37 7.36 7.35 7.34 7.24 7.22 7.21 7.19 7.17 7.16 7.15 7.14 7.06 7.04 7.03 7.02 5.40 5.38 5.13 5.11 5.09
3.70
3.10 3.09 3.07 2.74 2.72 2.70
F
O
OMe
S
133c
^{1}H NMR (400 MHz), $CDCl_3$
1.00 4.15 3.02 2.10 4.26 3.05 2.11 2.10
9.5 9.0 8.5 8.0 7.5 7.0 6.5 6.0 5.5 5.0 4.5 4.0 3.5 3.0 2.5 2.0 1.5 1.0 0.5
ppm

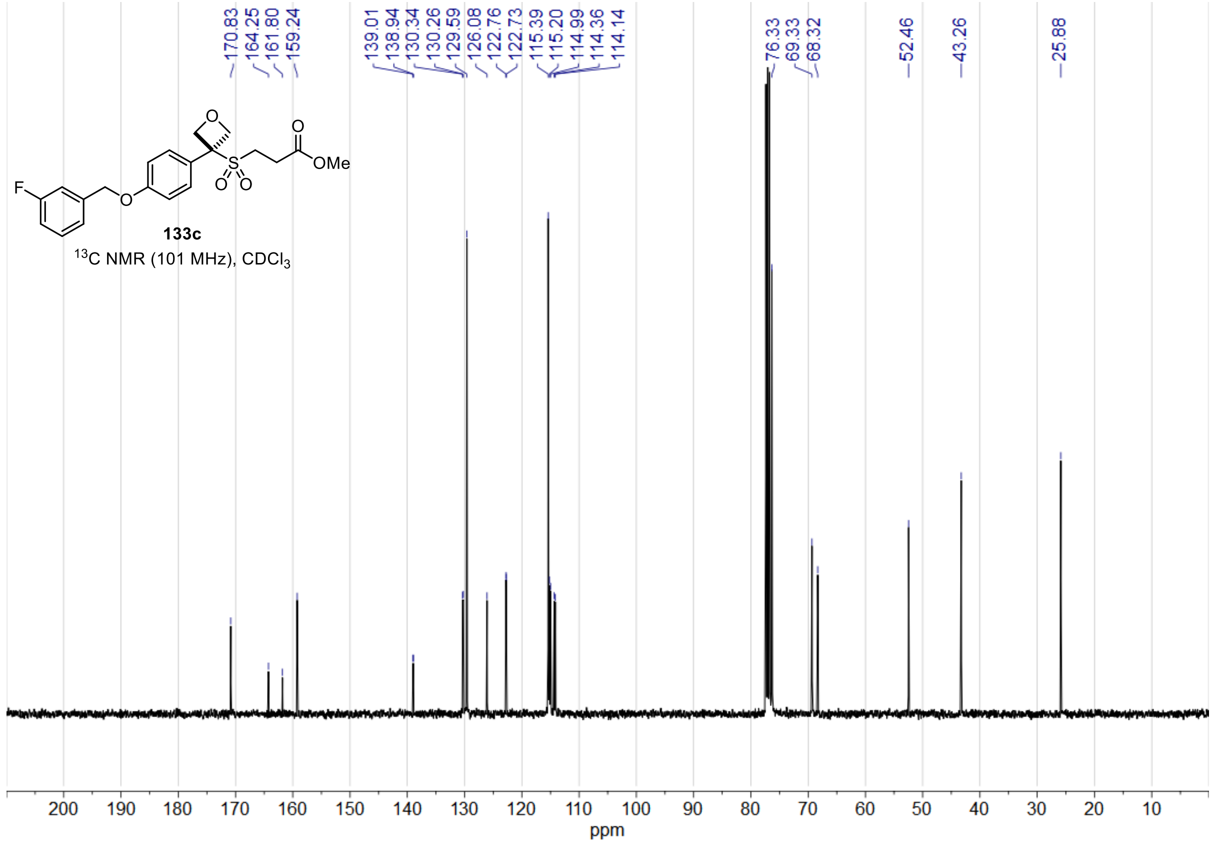
170.83 164.25 161.80 159.24
139.01 138.94 130.34 130.26 129.59 126.08 122.76 122.73 115.39 115.20 114.99 114.36 114.14
76.33 69.33 68.32
52.46
43.26
25.88
F
O
OMe
S
133c
^{13}C NMR (101 MHz), $CDCl_3$
200 190 180 170 160 150 140 130 120 110 100 90 80 70 60 50 40 30 20 10
ppm

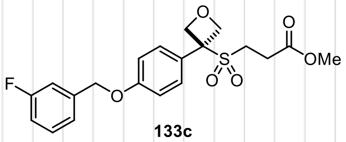

133c

$^{19}F\{^{1}H\}$ NMR (377 MHz), $CDCl_3$

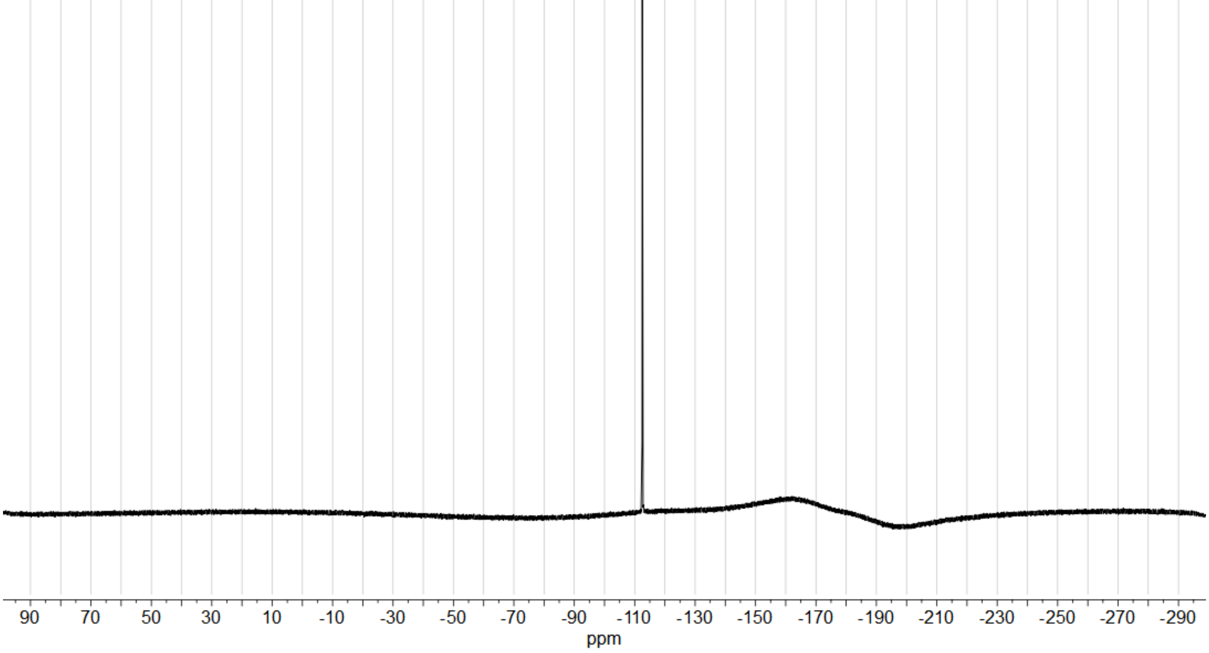

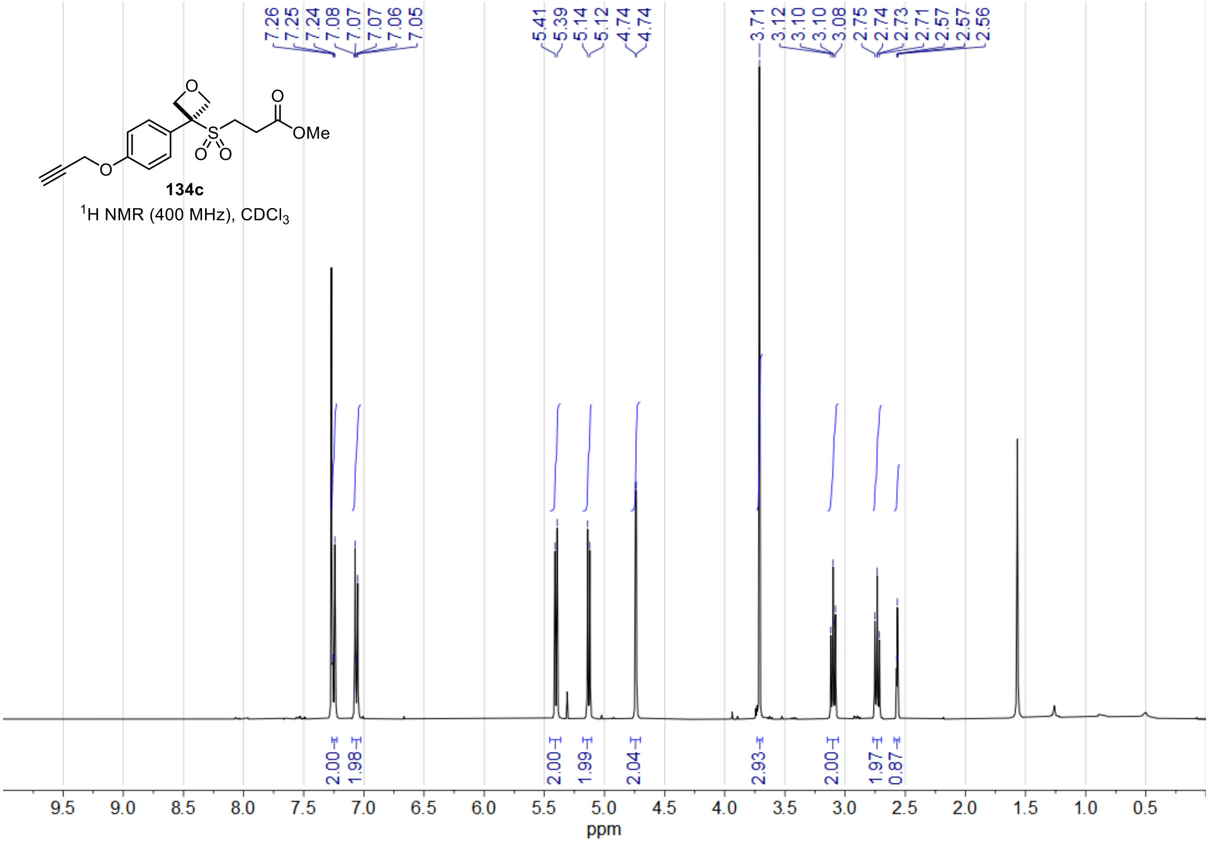
7.26
7.25
7.24
7.08
7.07
7.07
7.06
7.05
5.41
5.39
5.14
5.12
4.74
4.74
3.71
3.12
3.10
3.10
3.08
2.75
2.74
2.73
2.71
2.57
2.57
2.56
O
O
OMe
S
O O
O
134c
^{1}H NMR (400 MHz), $CDCl_3$
2.00
1.98
2.00
1.99
2.04
2.93
2.00
1.97
0.87
9.5
9.0
8.5
8.0
7.5
7.0
6.5
6.0
5.5
5.0
4.5
4.0
3.5
3.0
2.5
2.0
1.5
1.0
0.5
ppm

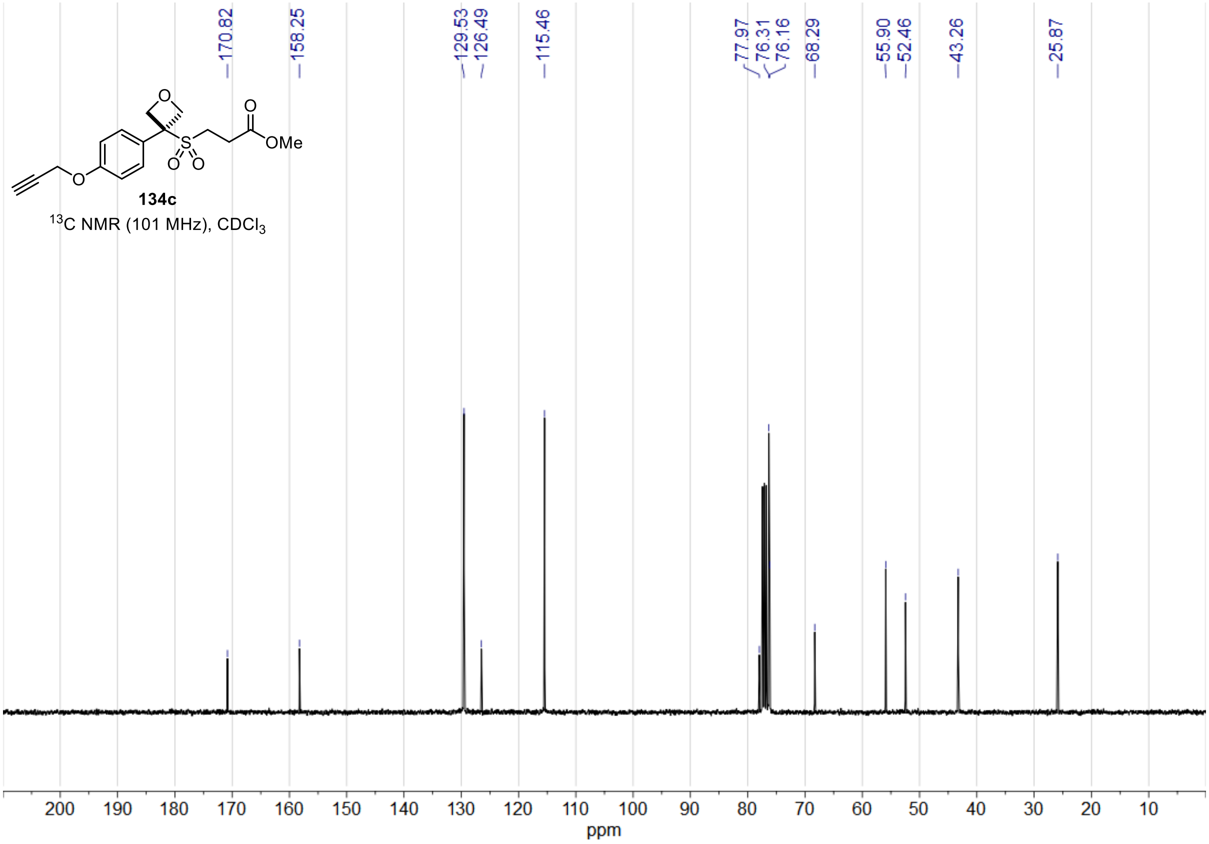
170.82
158.25
129.53
126.49
115.46
77.97
76.31
76.16
68.29
55.90
52.46
43.26
25.87
O
O
OMe
S
O O
O
134c
^{13}C NMR (101 MHz), $CDCl_3$
200
190
180
170
160
150
140
130
120
110
100
90
80
70
60
50
40
30
20
10
ppm

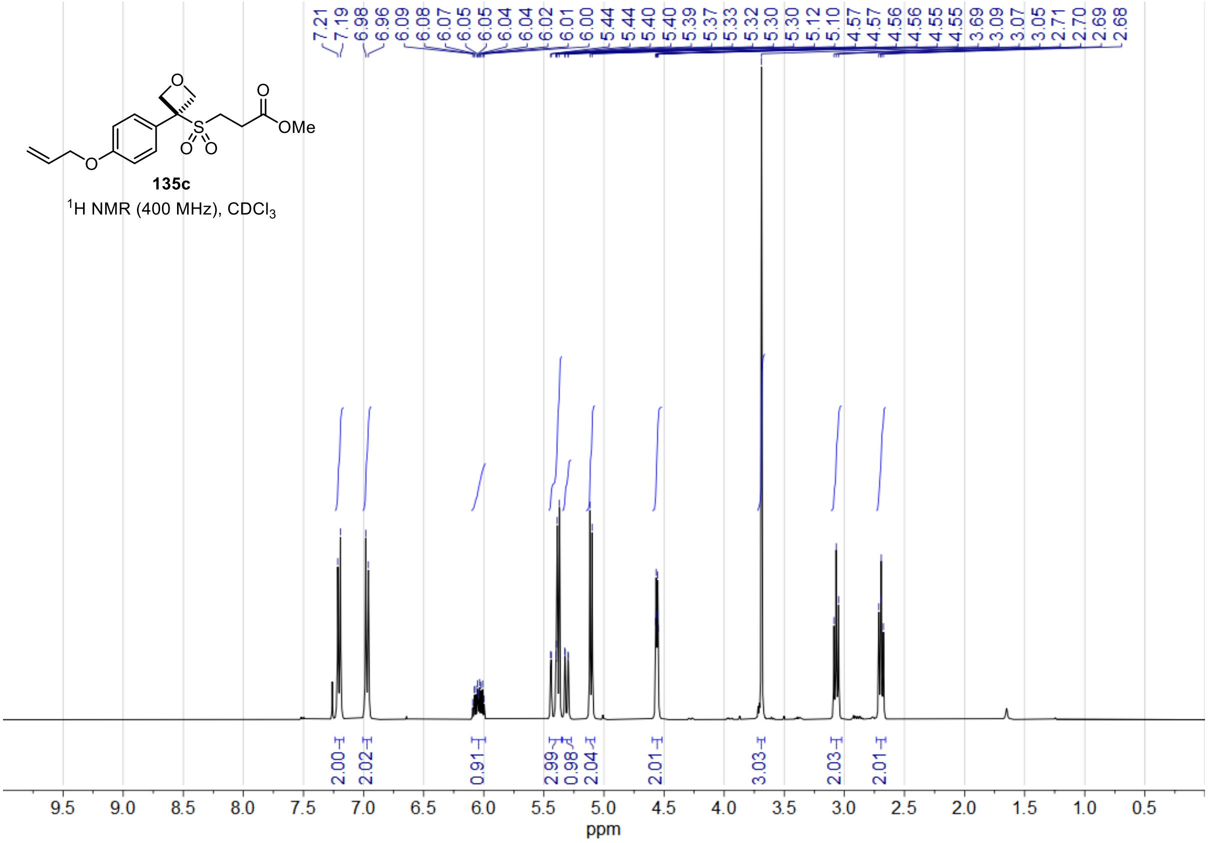

OMe
135c
¹H NMR (400 MHz), CDCl₃
7.21 7.19 6.98 6.96 6.09 6.08 6.07 6.05 6.05 6.04 6.04 6.02 6.01 6.00 5.44 5.44 5.40 5.40 5.39 5.37 5.33 5.32 5.30 5.12 5.10 4.57 4.57 4.56 4.56 4.55 4.55 3.69 3.09 3.07 3.05 2.71 2.70 2.69 2.68
2.00 2.02 0.91 2.99 0.98 2.04 2.01 3.03 2.03 2.01
9.5 9.0 8.5 8.0 7.5 7.0 6.5 6.0 5.5 5.0 4.5 4.0 3.5 3.0 2.5 2.0 1.5 1.0 0.5
ppm

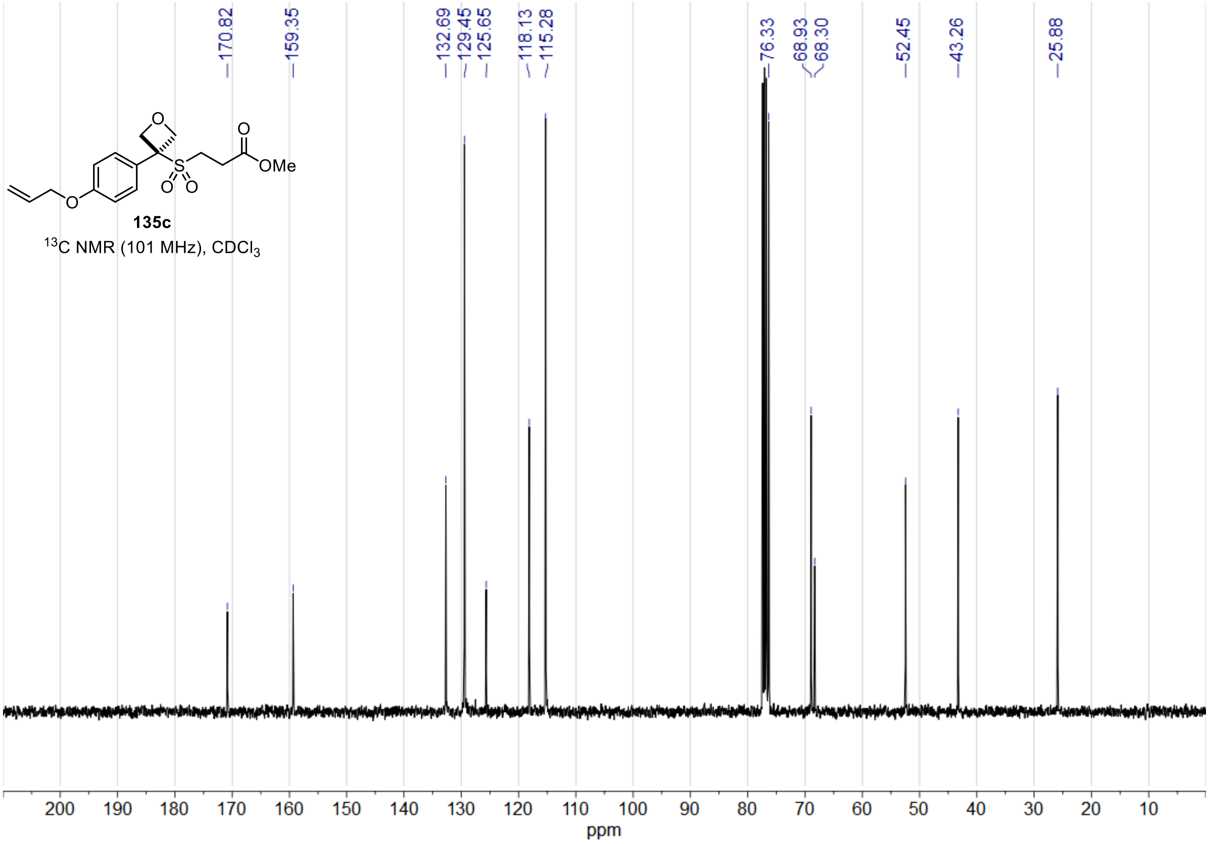

OMe
135c
¹³C NMR (101 MHz), CDCl₃
170.82 159.35 132.69 129.45 125.65 118.13 115.28 76.33 68.93 68.30 52.45 43.26 25.88
200 190 180 170 160 150 140 130 120 110 100 90 80 70 60 50 40 30 20 10
ppm

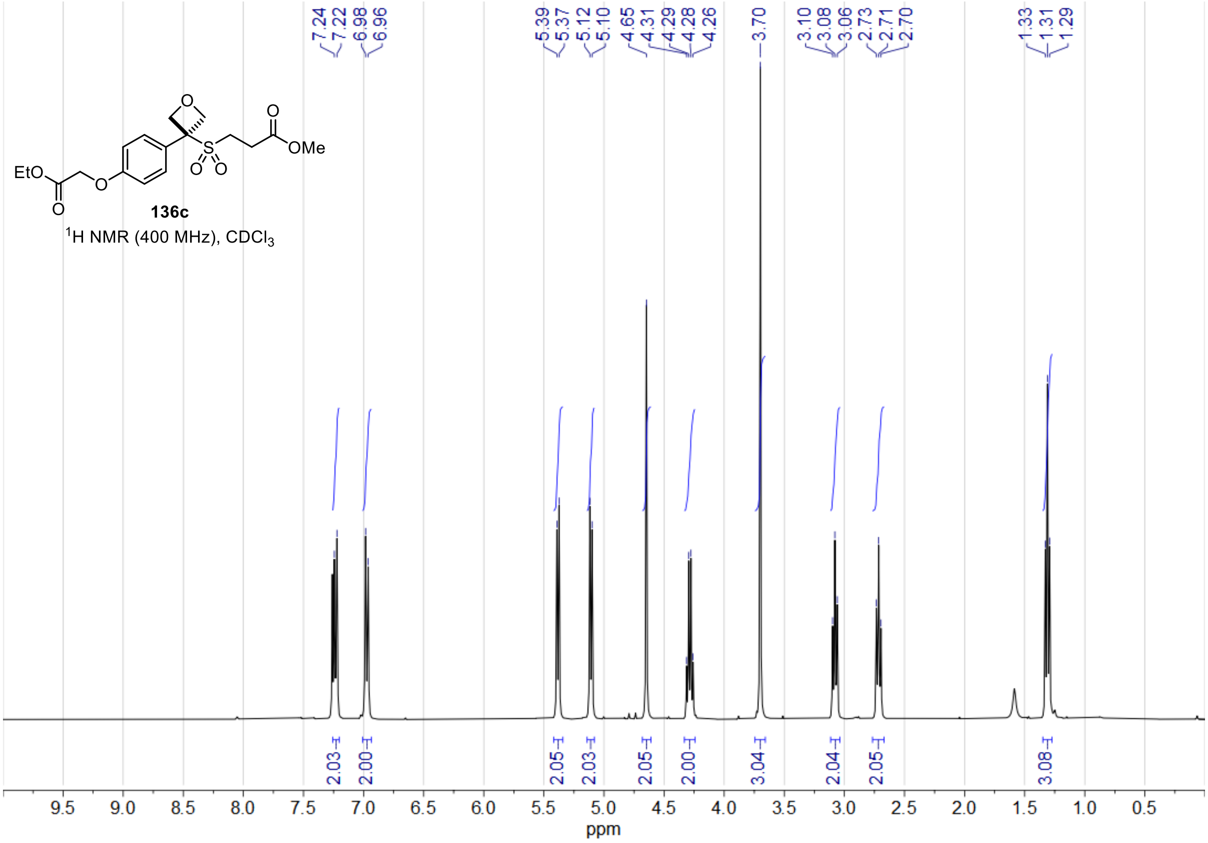
7.24
7.22
6.98
6.96
5.39
5.37
5.12
5.10
4.65
4.31
4.29
4.28
4.26
3.70
3.10
3.08
3.06
2.73
2.71
2.70
1.33
1.31
1.29
EtO
O
O
O
S
O
O
O
OMe
136c
^{1}H NMR (400 MHz), CDCl$_3$
2.03
2.00
2.05
2.03
2.05
2.00
3.04
2.04
2.05
3.08
9.5 9.0 8.5 8.0 7.5 7.0 6.5 6.0 5.5 5.0 4.5 4.0 3.5 3.0 2.5 2.0 1.5 1.0 0.5
ppm

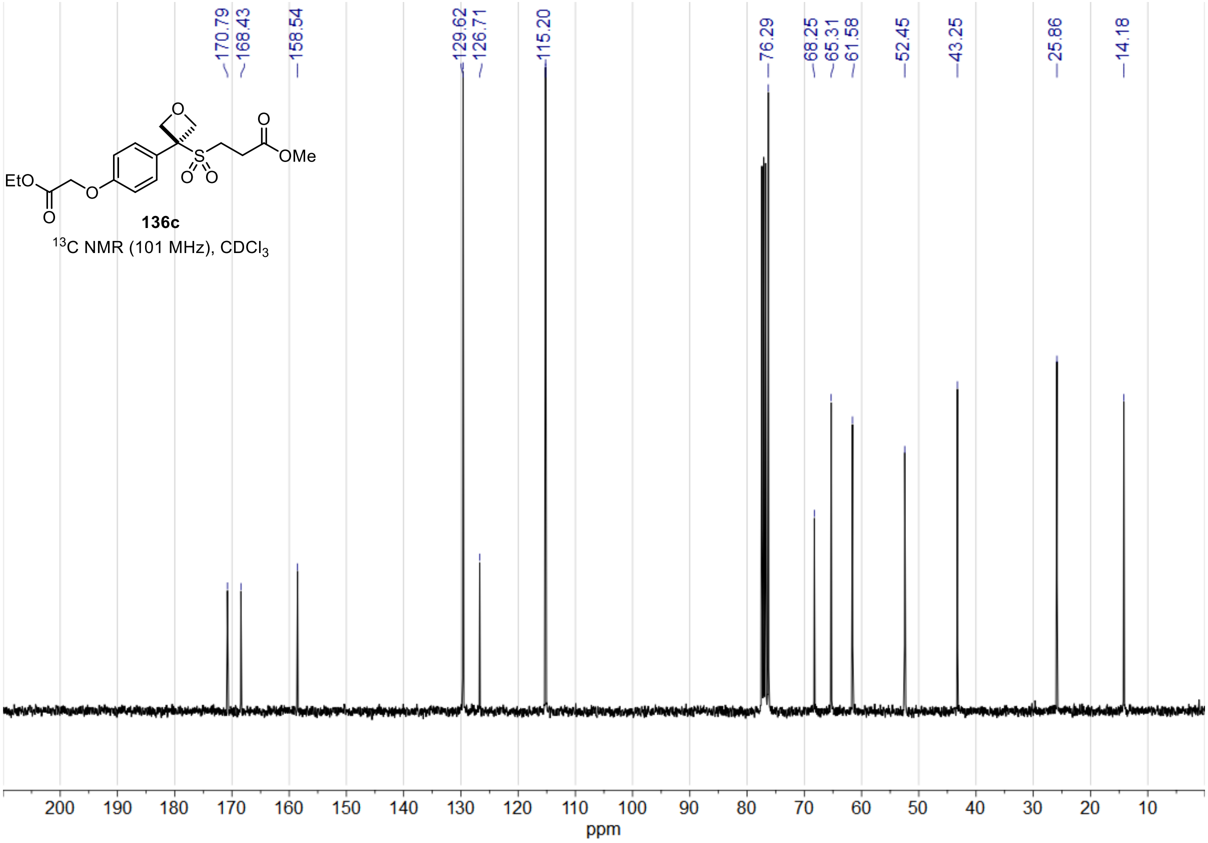
170.79
168.43
158.54
129.62
126.71
115.20
76.29
68.25
65.31
61.58
52.45
43.25
25.86
14.18
EtO
O
O
O
S
O
O
O
OMe
136c
^{13}C NMR (101 MHz), CDCl$_3$
200 190 180 170 160 150 140 130 120 110 100 90 80 70 60 50 40 30 20 10
ppm

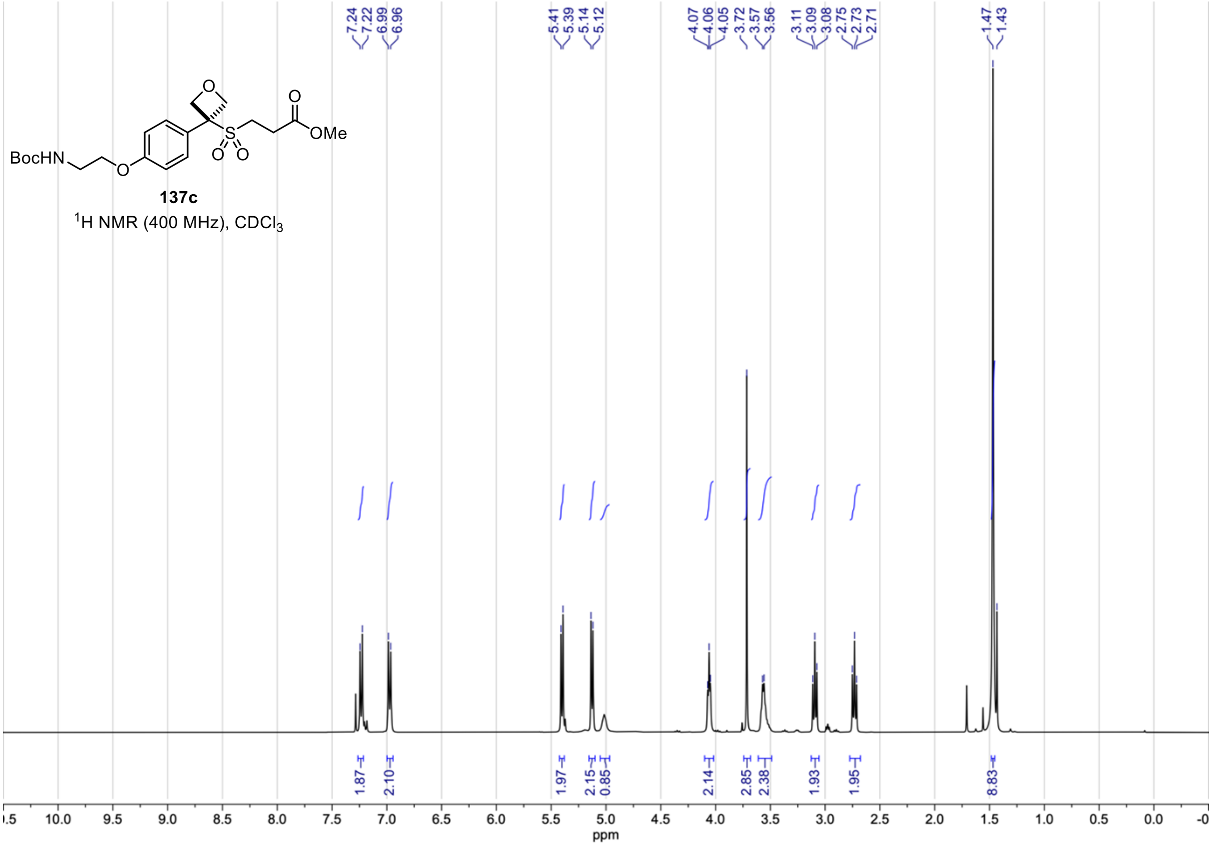
BocHN
O
O
S
O
O
OMe
O
137c
^{1}H NMR (400 MHz), CDCl$_3$
7.24
7.22
6.99
6.96
5.41
5.39
5.14
5.12
4.07
4.06
4.05
3.72
3.57
3.56
3.11
3.09
3.08
2.75
2.73
2.71
1.47
1.43
1.87
2.10
1.97
2.15
0.85
2.14
2.85
2.38
1.93
1.95
8.83
ppm

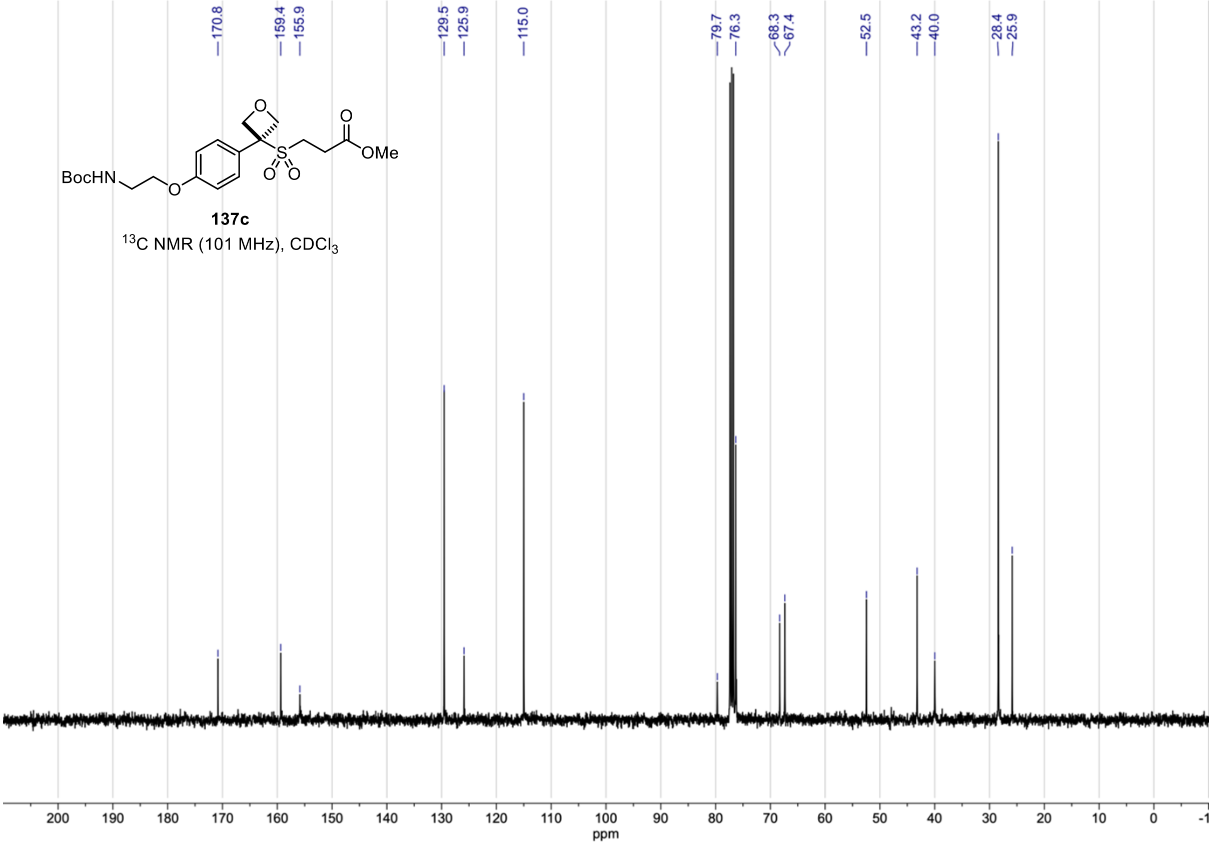
BocHN
O
O
S
O
O
OMe
O
137c
^{13}C NMR (101 MHz), CDCl$_3$
170.8
159.4
155.9
129.5
125.9
115.0
79.7
76.3
68.3
67.4
52.5
43.2
40.0
28.4
25.9
ppm

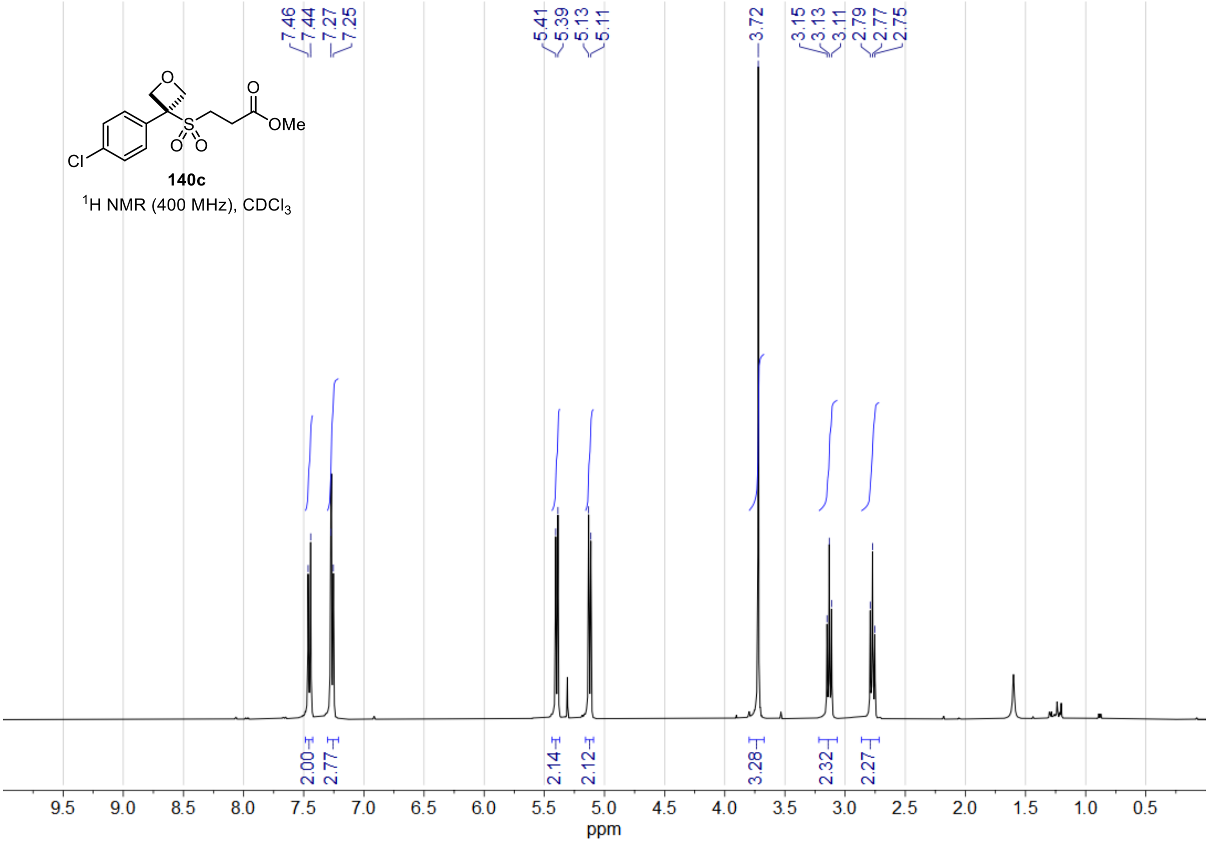
7.46
7.44
7.27
7.25
5.41
5.39
5.13
5.11
3.72
3.15
3.13
3.11
2.79
2.77
2.75
O
OMe
S
O O
Cl
140c
1H NMR (400 MHz), CDCl3
2.00
2.77
2.14
2.12
3.28
2.32
2.27
9.5 9.0 8.5 8.0 7.5 7.0 6.5 6.0 5.5 5.0 4.5 4.0 3.5 3.0 2.5 2.0 1.5 1.0 0.5
ppm

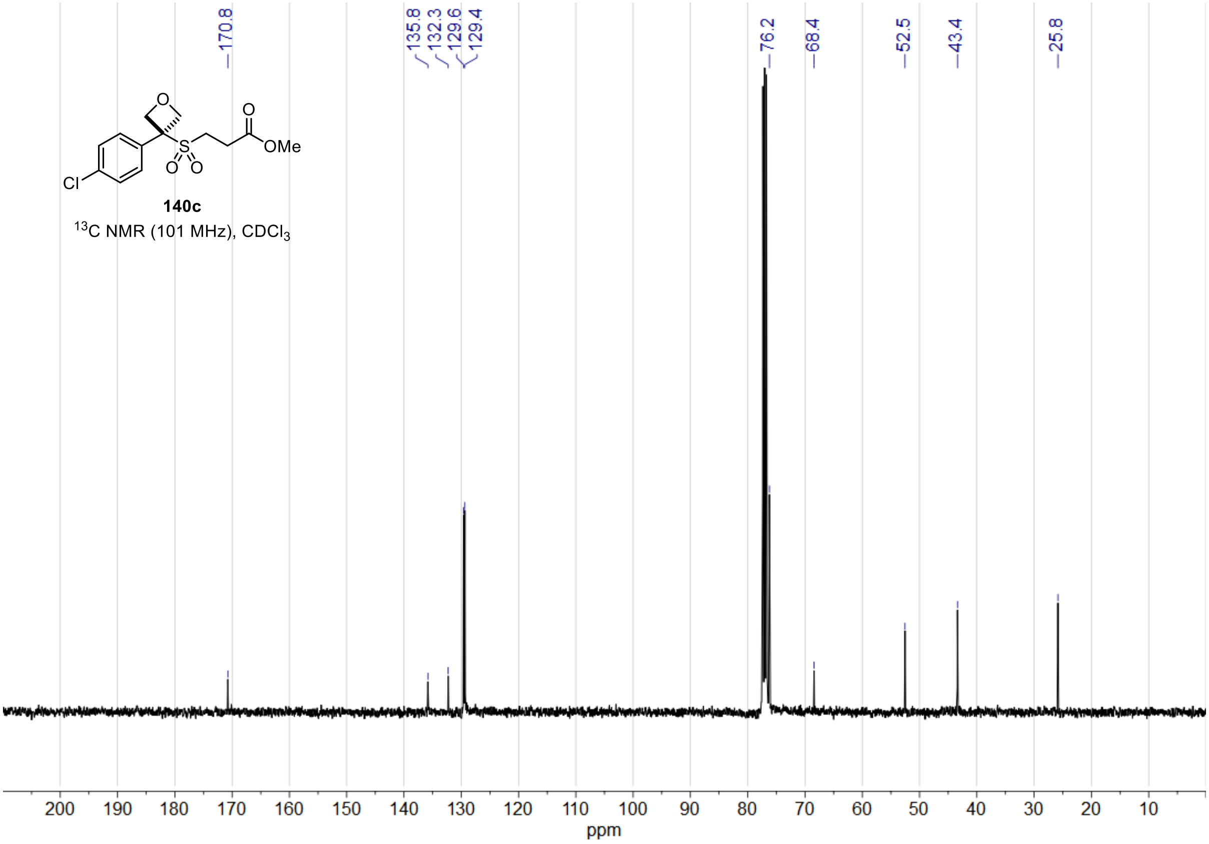
170.8
135.8
132.3
129.6
129.4
76.2
68.4
52.5
43.4
25.8
O
OMe
S
O O
Cl
140c
13C NMR (101 MHz), CDCl3
200 190 180 170 160 150 140 130 120 110 100 90 80 70 60 50 40 30 20 10
ppm

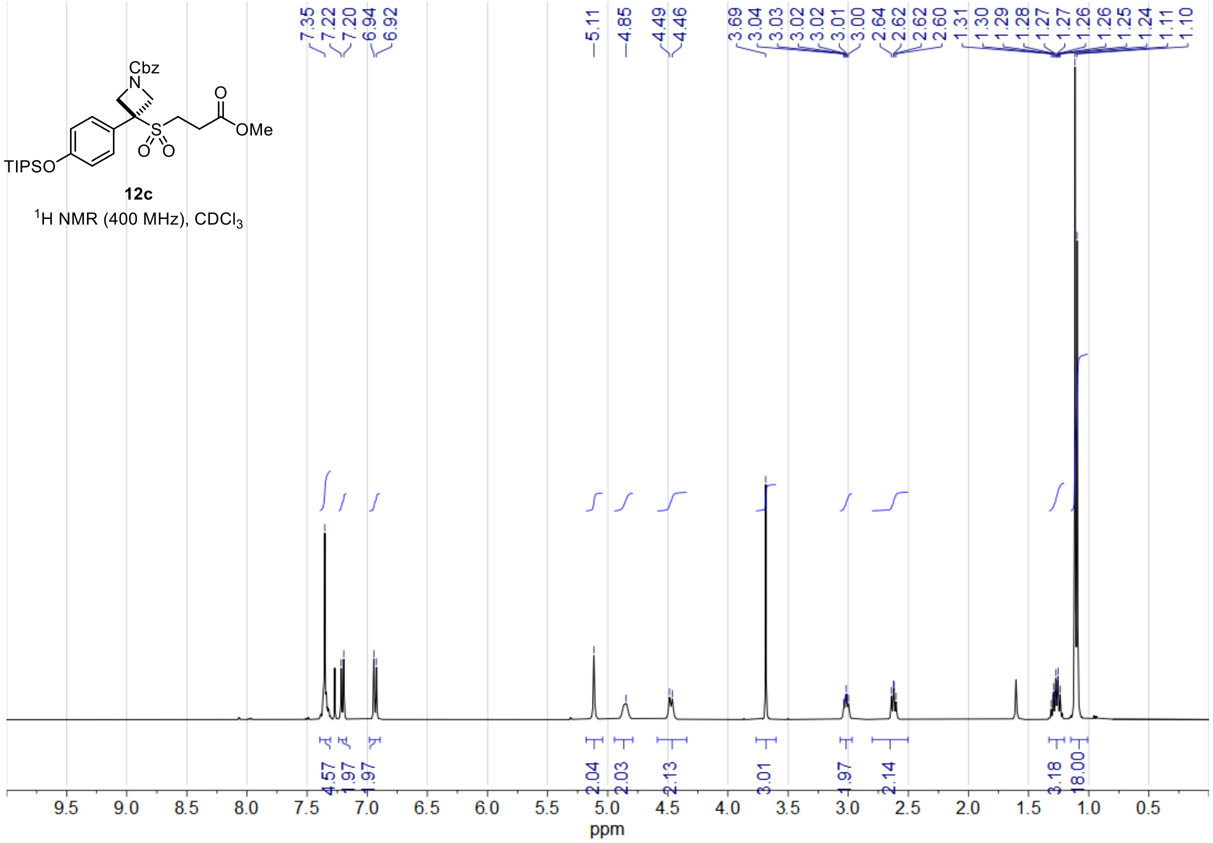
7.35
7.22
7.20
6.94
6.92
5.11
4.85
4.49
4.46
3.69
3.04
3.03
3.02
3.02
3.01
3.00
2.64
2.62
2.62
2.60
1.31
1.30
1.29
1.28
1.27
1.27
1.26
1.26
1.25
1.24
1.11
1.10
Cbz
N
S
O
O
O
OMe
TIPSO
12c
^{1}H NMR (400 MHz), $CDCl_3$
4.57
1.97
1.97
2.04
2.03
2.13
3.01
1.97
2.14
3.18
18.00
9.5
9.0
8.5
8.0
7.5
7.0
6.5
6.0
5.5
5.0
4.5
4.0
3.5
3.0
2.5
2.0
1.5
1.0
0.5
ppm

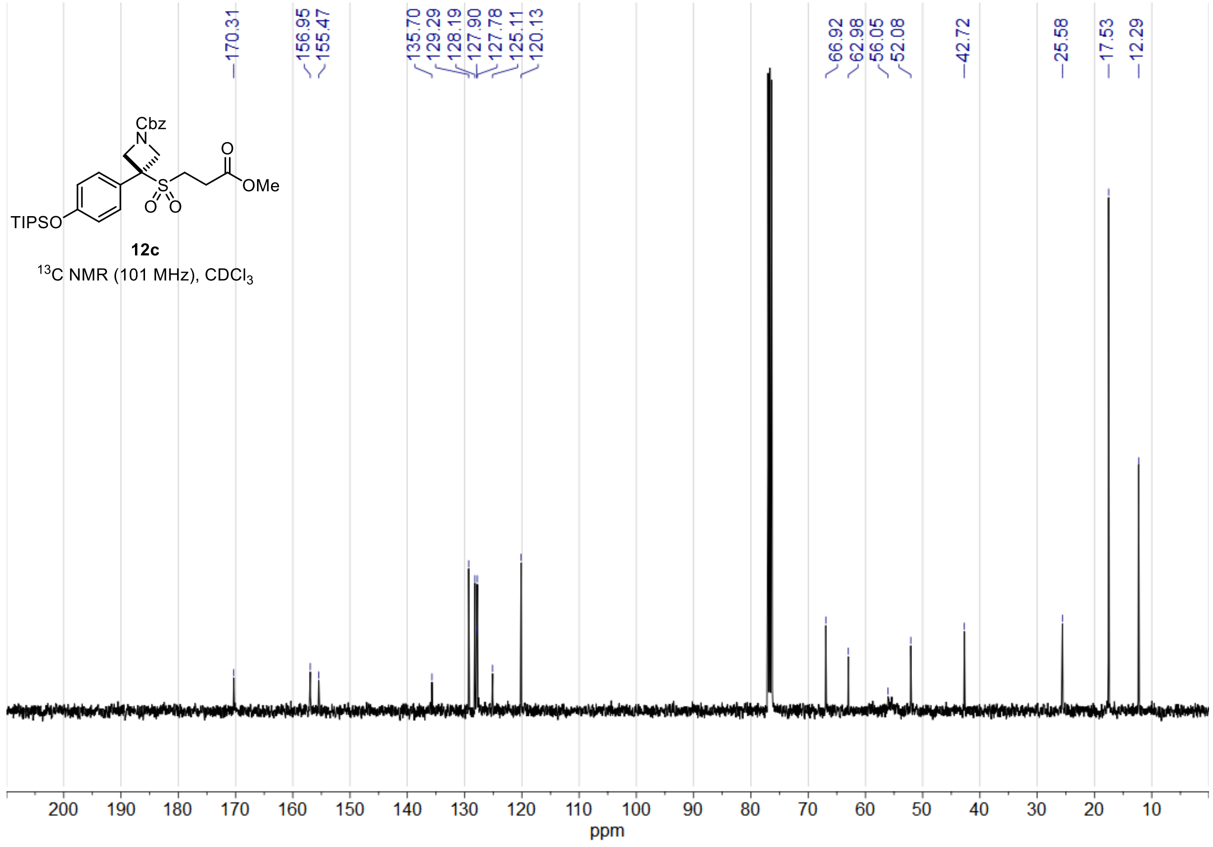
170.31
156.95
155.47
135.70
129.29
128.19
127.90
127.78
125.11
120.13
66.92
62.98
56.05
52.08
42.72
25.58
17.53
12.29
Cbz
N
S
O
O
O
OMe
TIPSO
12c
^{13}C NMR (101 MHz), $CDCl_3$
200
190
180
170
160
150
140
130
120
110
100
90
80
70
60
50
40
30
20
10
ppm

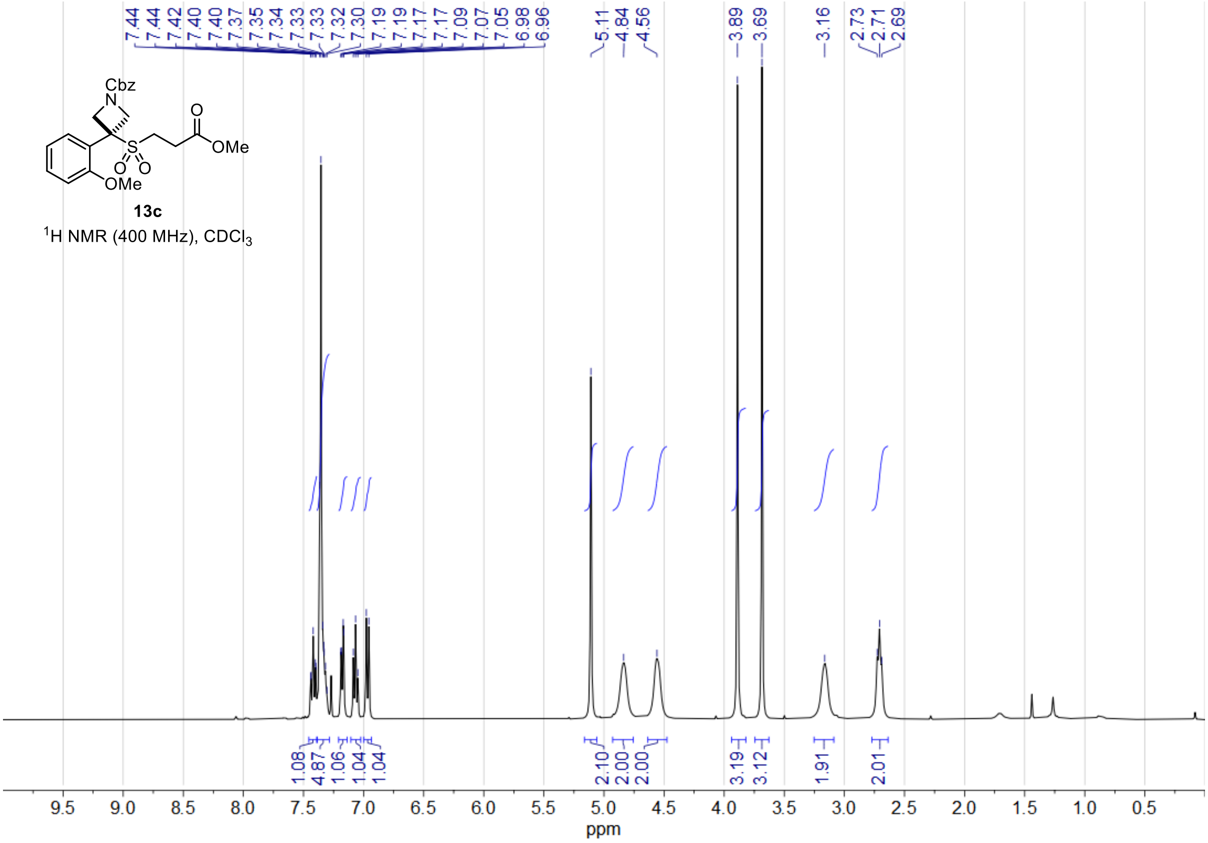
7.44 7.44 7.42 7.40 7.40 7.37 7.35 7.34 7.33 7.33 7.32 7.30 7.19 7.19 7.17 7.17 7.09 7.07 7.05 6.98 6.96
5.11 4.84 4.56
3.89 3.69
3.16
2.73 2.71 2.69
Cbz
N
O
S
OMe
O O
OMe
13c
^{1}H NMR (400 MHz), CDCl$_3$
1.08 4.87 1.06 1.04 1.04
2.10 2.00 2.00
3.19 3.12
1.91
2.01
9.5 9.0 8.5 8.0 7.5 7.0 6.5 6.0 5.5 5.0 4.5 4.0 3.5 3.0 2.5 2.0 1.5 1.0 0.5
ppm

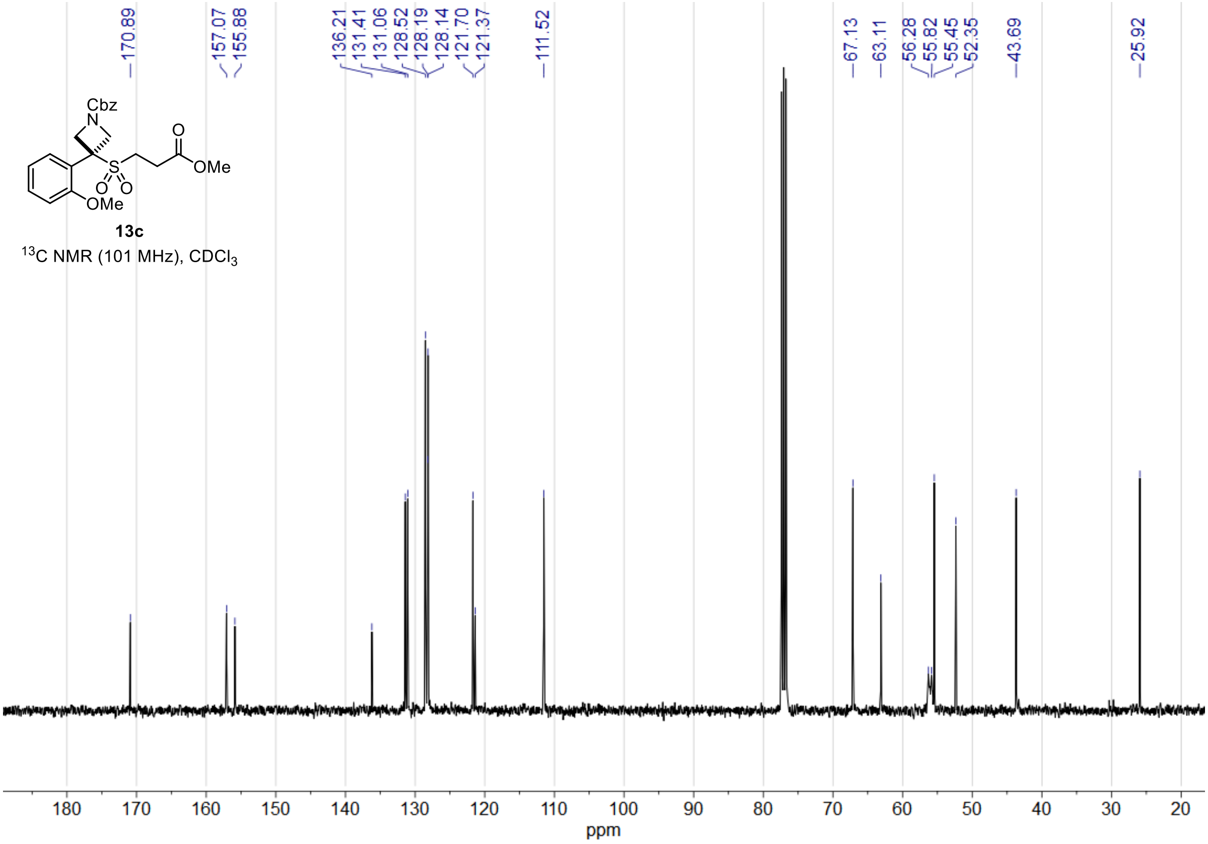
170.89
157.07 155.88
136.21 131.41 131.06 128.52 128.19 128.14 121.70 121.37
111.52
67.13
63.11
56.28 55.82 55.45 52.35
43.69
25.92
Cbz
N
O
S
OMe
O O
OMe
13c
^{13}C NMR (101 MHz), CDCl$_3$
180 170 160 150 140 130 120 110 100 90 80 70 60 50 40 30 20
ppm

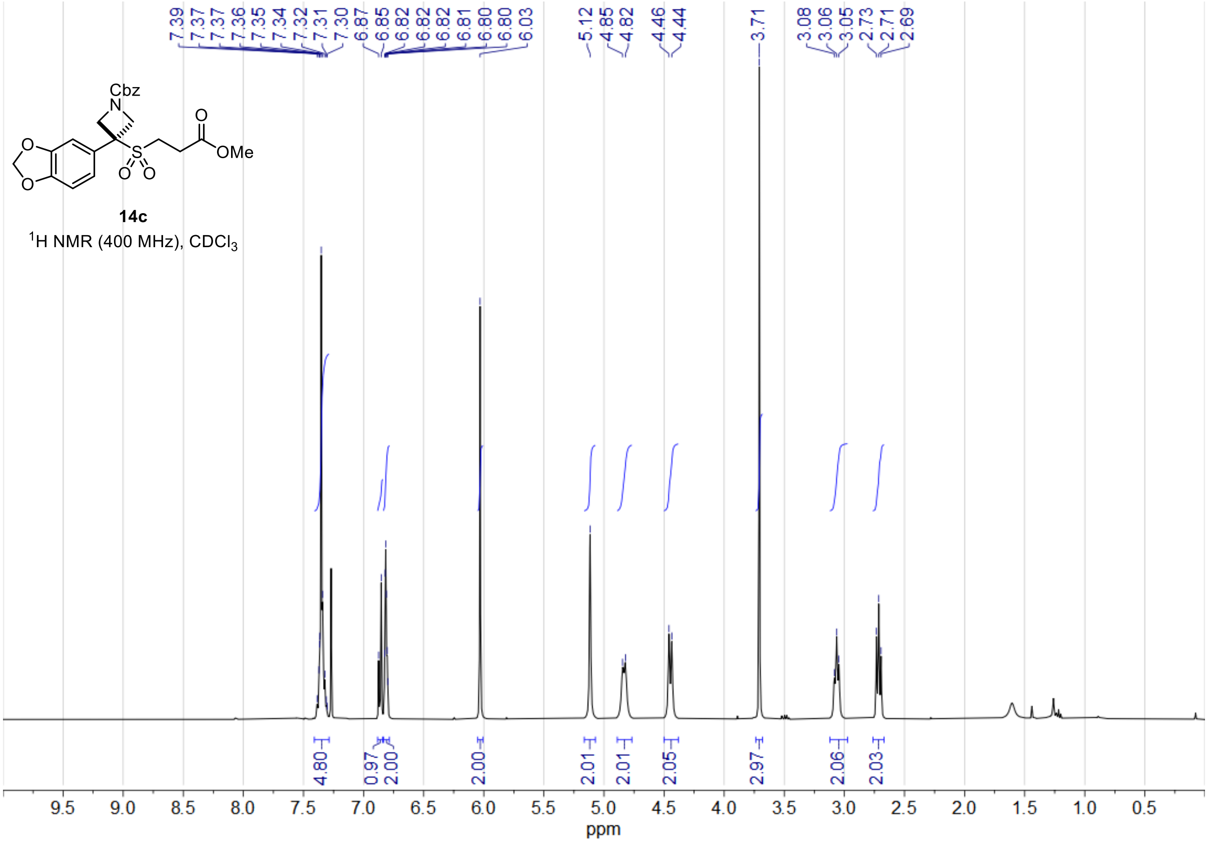
7.39
7.37
7.37
7.36
7.35
7.34
7.32
7.31
7.30
6.87
6.85
6.82
6.82
6.82
6.81
6.80
6.80
6.03
5.12
4.85
4.82
4.46
4.44
3.71
3.08
3.06
3.05
2.73
2.71
2.69
Cbz
N
O
OMe
S
O
O
14c
^{1}H NMR (400 MHz), CDCl$_3$
4.80
0.97
2.00
2.00
2.01
2.01
2.05
2.97
2.06
2.03
ppm

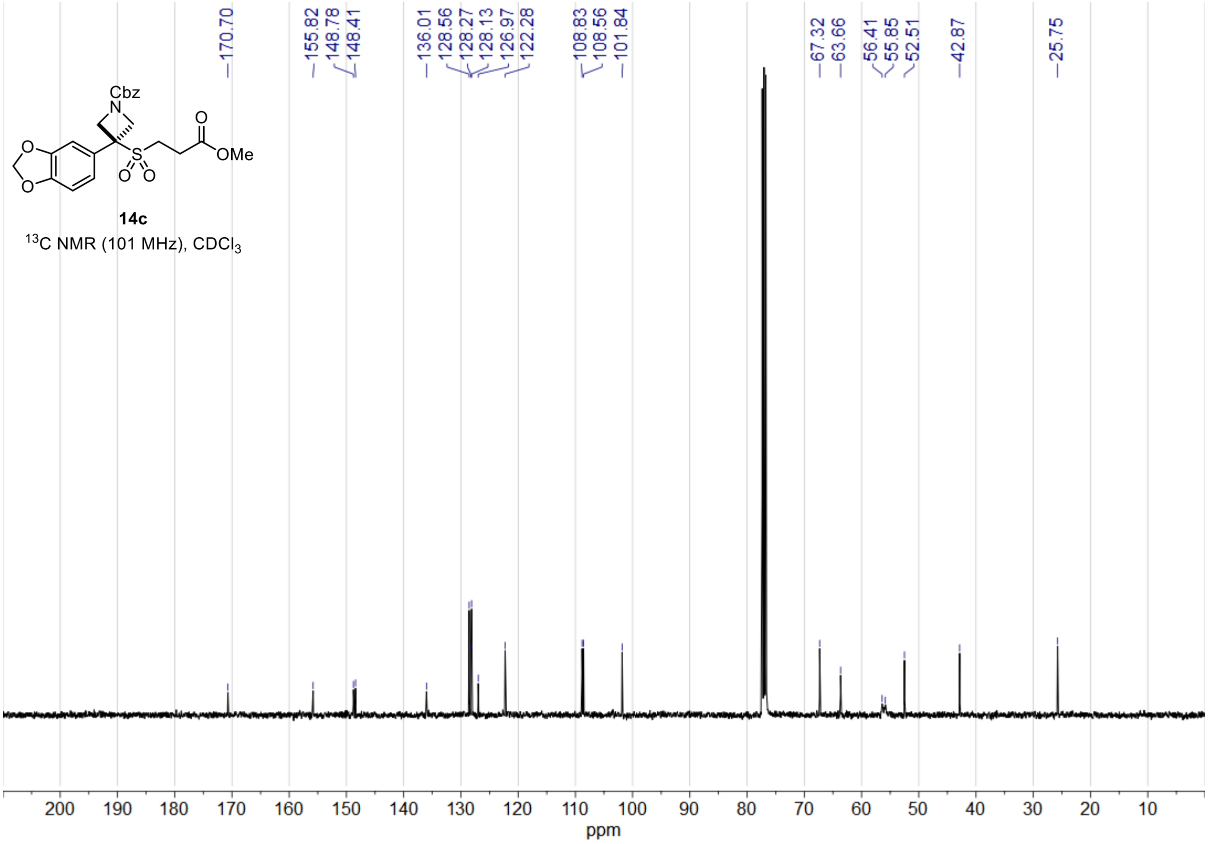
170.70
155.82
148.78
148.41
136.01
128.56
128.27
128.13
126.97
122.28
108.83
108.56
101.84
67.32
63.66
56.41
55.85
52.51
42.87
25.75
Cbz
N
O
OMe
S
O
O
14c
^{13}C NMR (101 MHz), CDCl$_3$
ppm

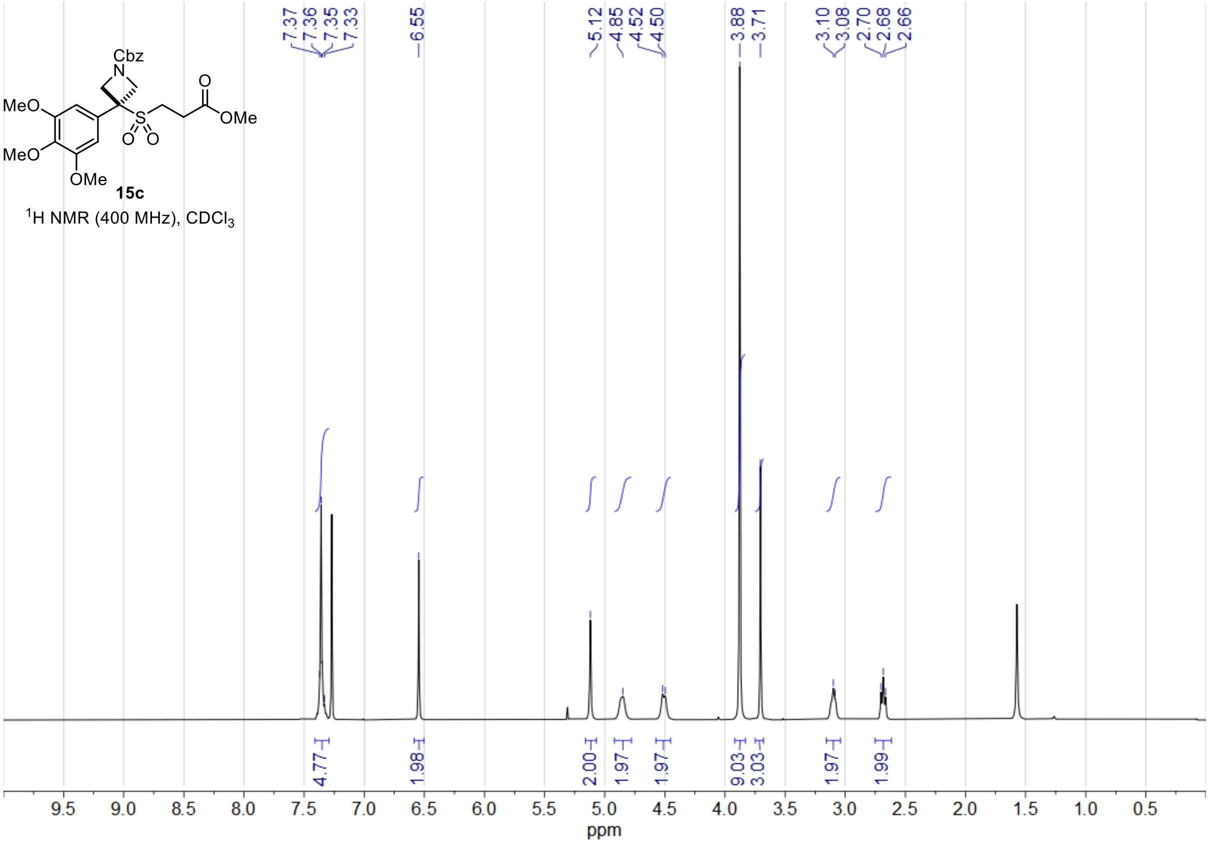

15c

^{1}H NMR (400 MHz), $CDCl_3$

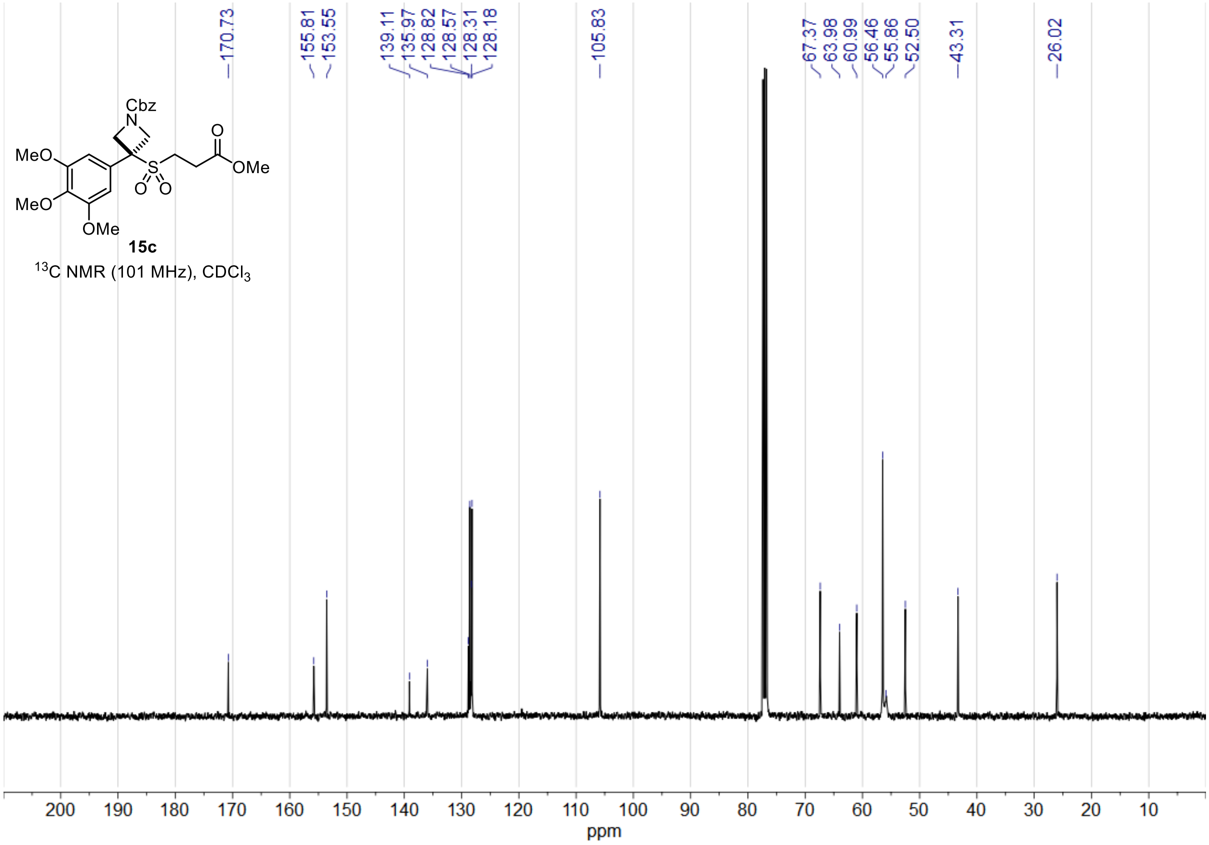

15c

^{13}C NMR (101 MHz), $CDCl_3$

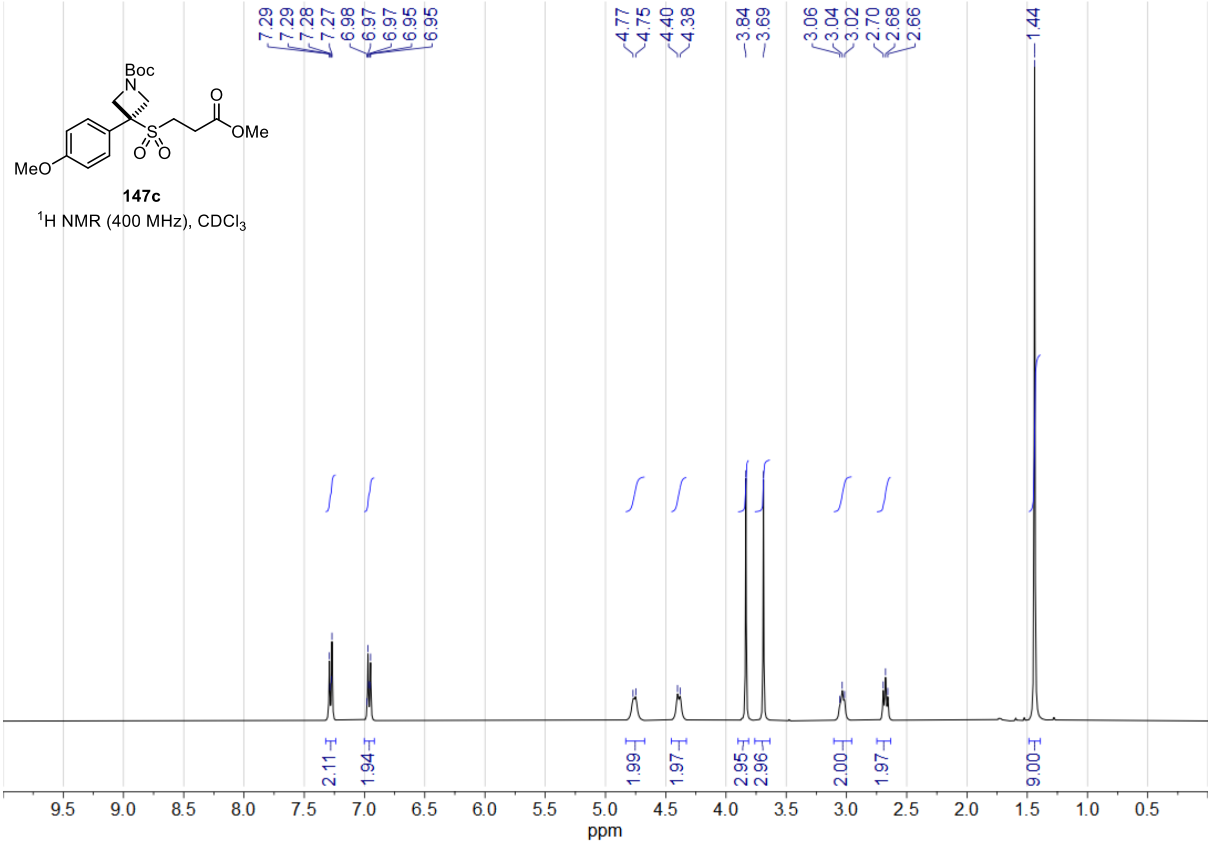

147c

^{1}H NMR (400 MHz), $CDCl_3$

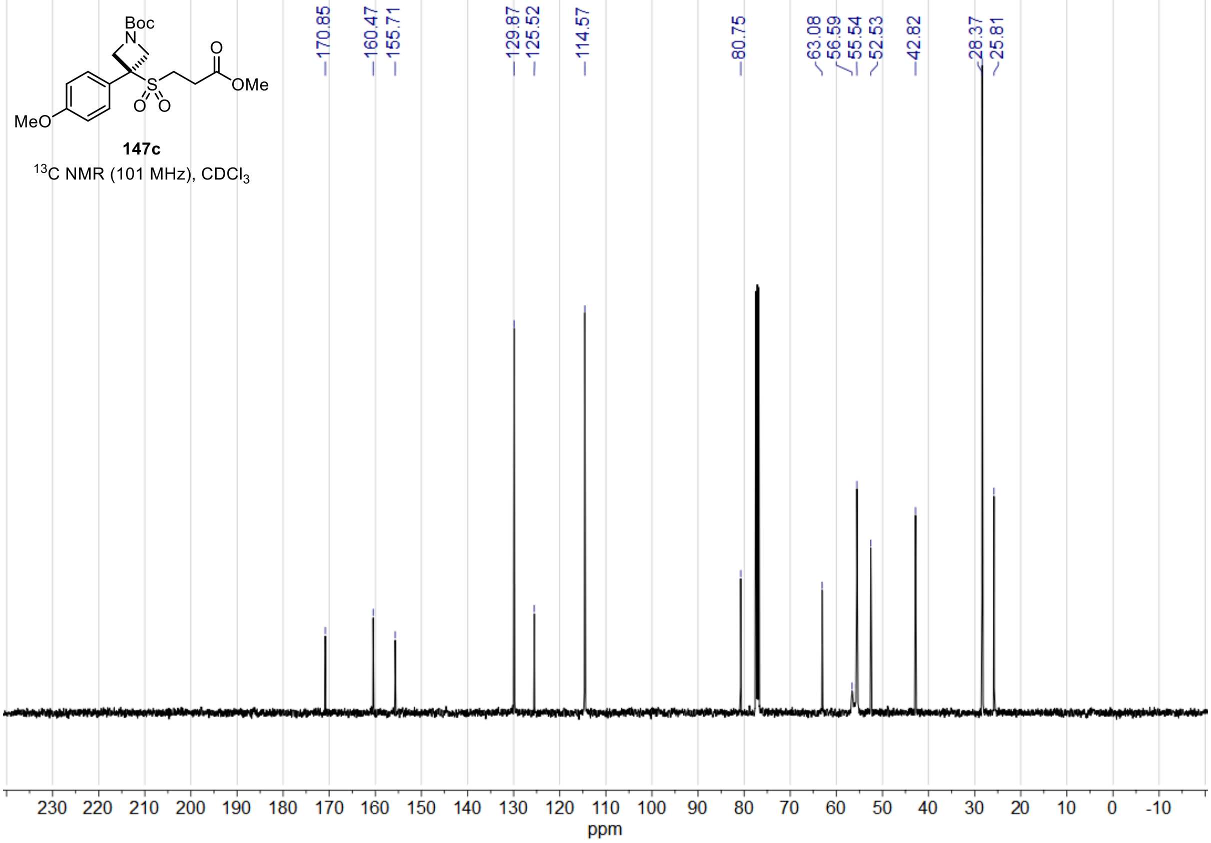

147c

^{13}C NMR (101 MHz), $CDCl_3$

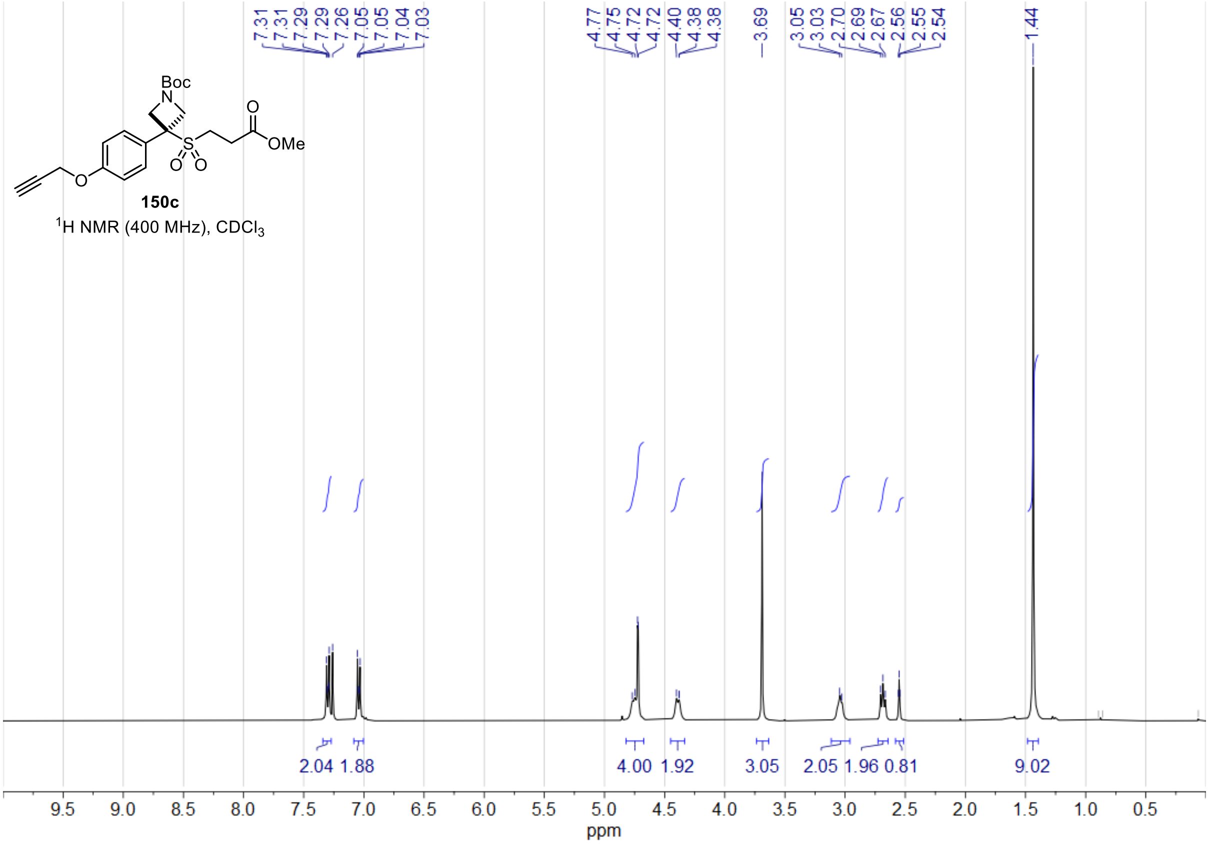
Boc
N
S
O O
O
OMe
O
150c
^{1}H NMR (400 MHz), CDCl$_3$
7.31 7.31 7.29 7.26 7.05 7.05 7.04 7.03
4.77 4.75 4.72 4.72 4.40 4.38 4.38
3.69
3.05 3.03 2.70 2.69 2.67 2.56 2.55 2.54
1.44
2.04 1.88 4.00 1.92 3.05 2.05 1.96 0.81 9.02
9.5 9.0 8.5 8.0 7.5 7.0 6.5 6.0 5.5 5.0 4.5 4.0 3.5 3.0 2.5 2.0 1.5 1.0 0.5
ppm

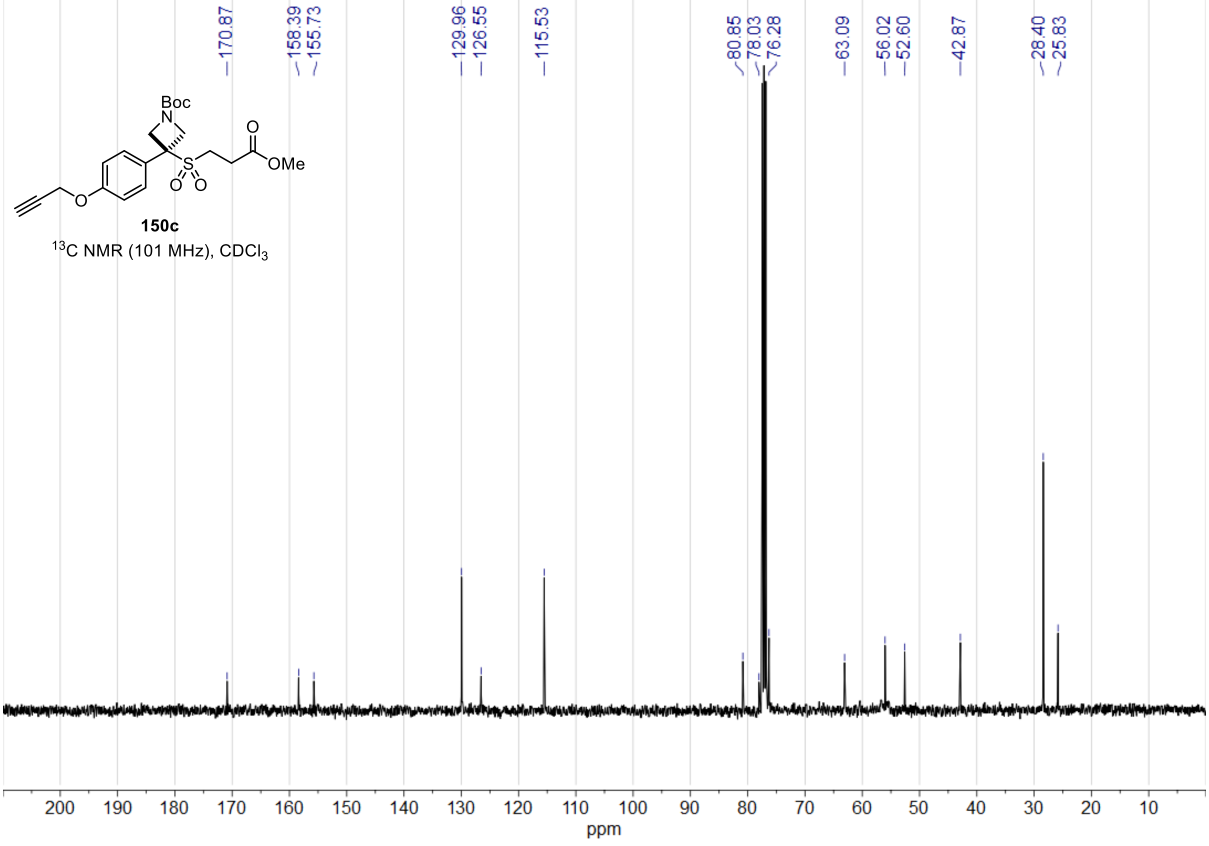
Boc
N
S
O O
O
OMe
O
150c
^{13}C NMR (101 MHz), CDCl$_3$
170.87
158.39 155.73
129.96 126.55
115.53
80.85 78.03 76.28
63.09
56.02 52.60
42.87
28.40 25.83
200 190 180 170 160 150 140 130 120 110 100 90 80 70 60 50 40 30 20 10
ppm

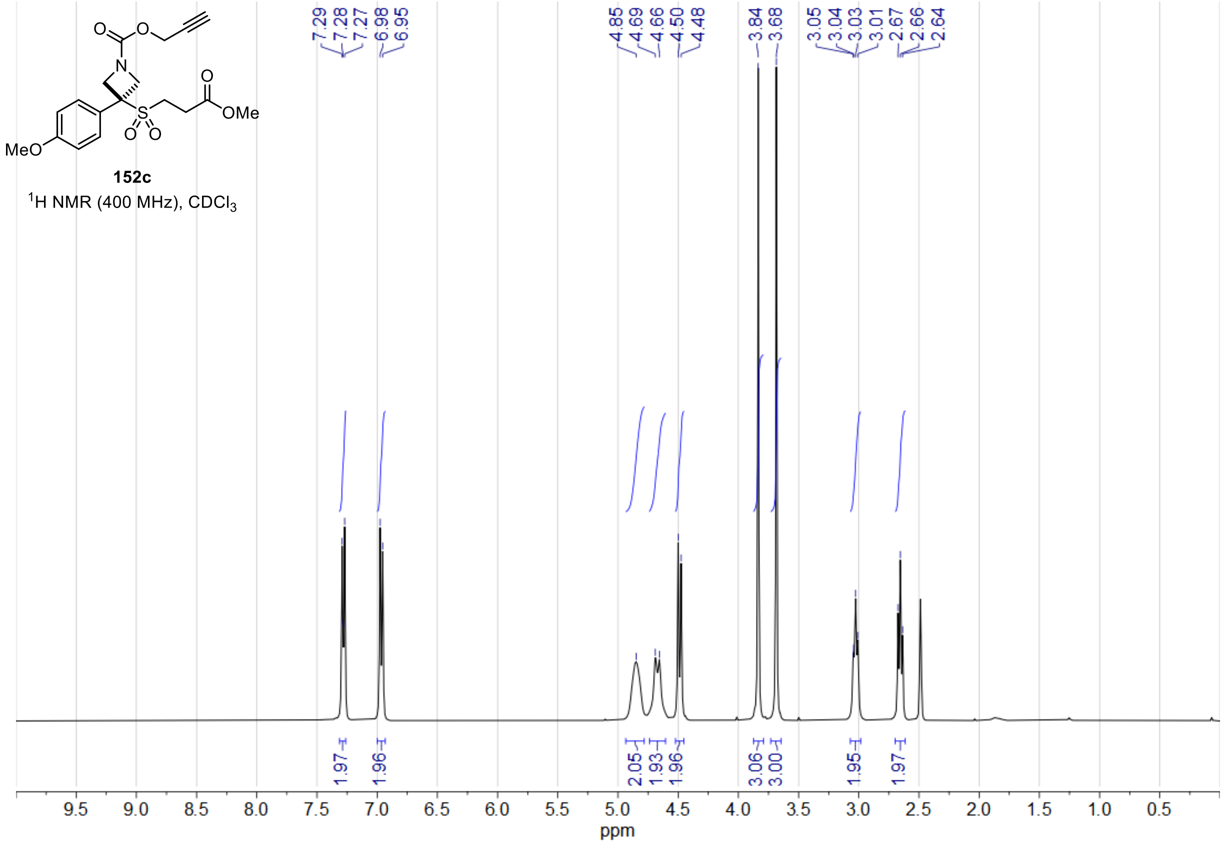
152c
1H NMR (400 MHz), CDCl3
7.29
7.28
7.27
6.98
6.95
4.85
4.69
4.66
4.50
4.48
3.84
3.68
3.05
3.04
3.03
3.01
2.67
2.66
2.64
1.97
1.96
2.05
1.93
1.96
3.06
3.00
1.95
1.97
ppm

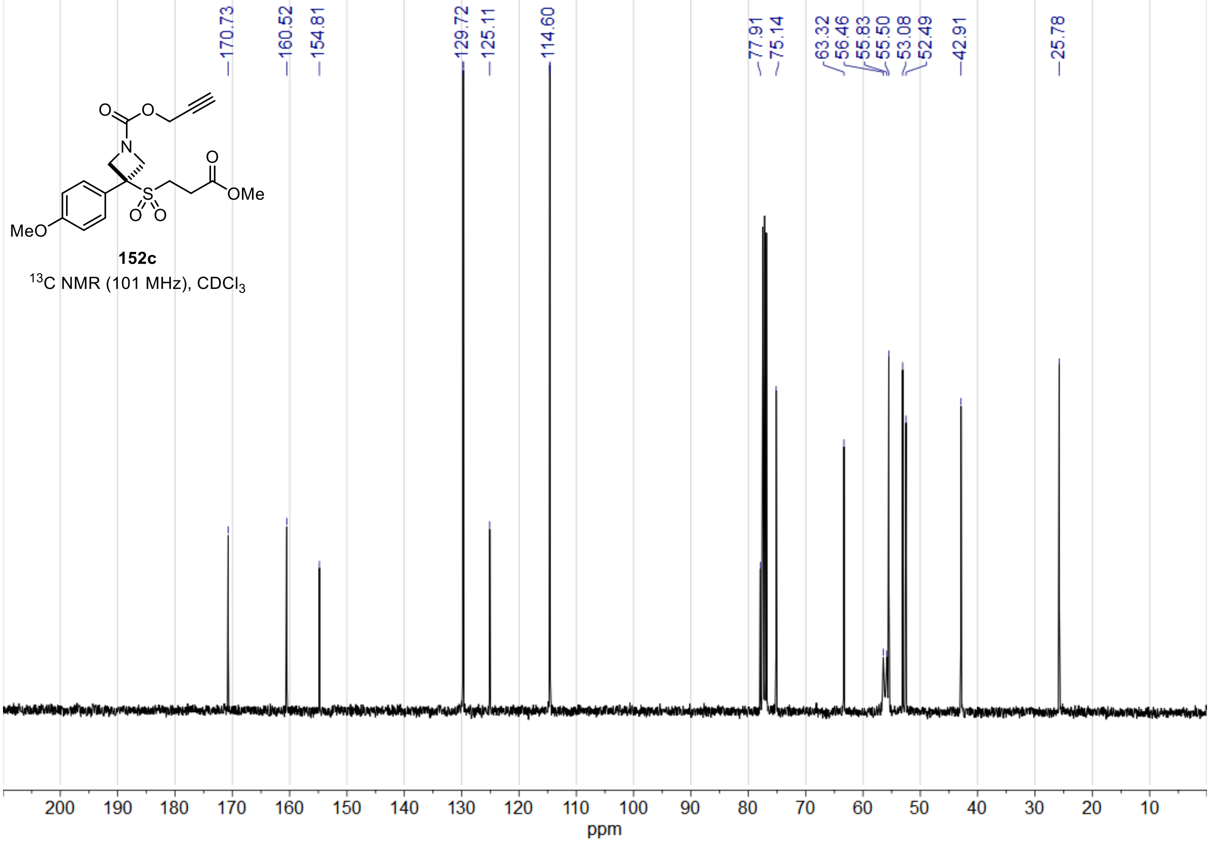
152c
13C NMR (101 MHz), CDCl3
170.73
160.52
154.81
129.72
125.11
114.60
77.91
75.14
63.32
56.46
55.83
55.50
53.08
52.49
42.91
25.78
ppm

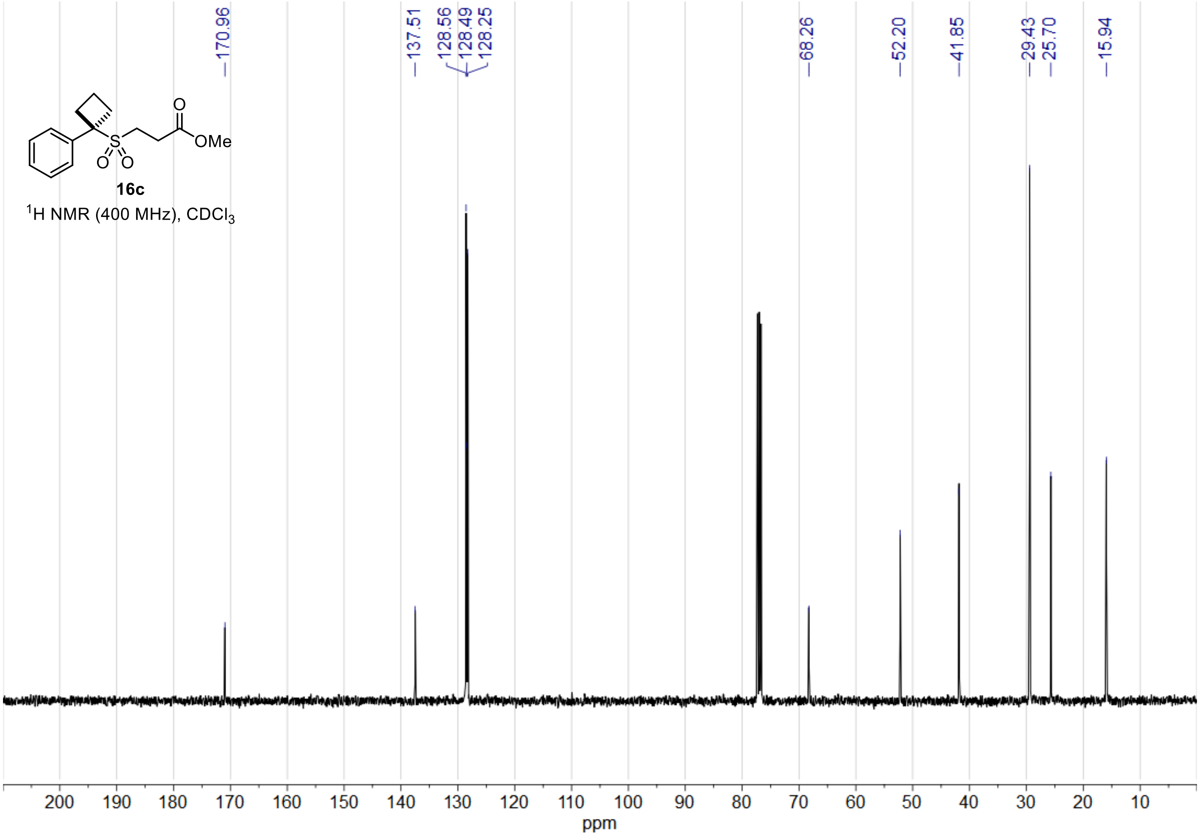
170.96
137.51
128.56
128.49
128.25
68.26
52.20
41.85
29.43
25.70
15.94
O
OMe
S
O O
16c
^{1}H NMR (400 MHz), CDCl$_3$
200 190 180 170 160 150 140 130 120 110 100 90 80 70 60 50 40 30 20 10
ppm

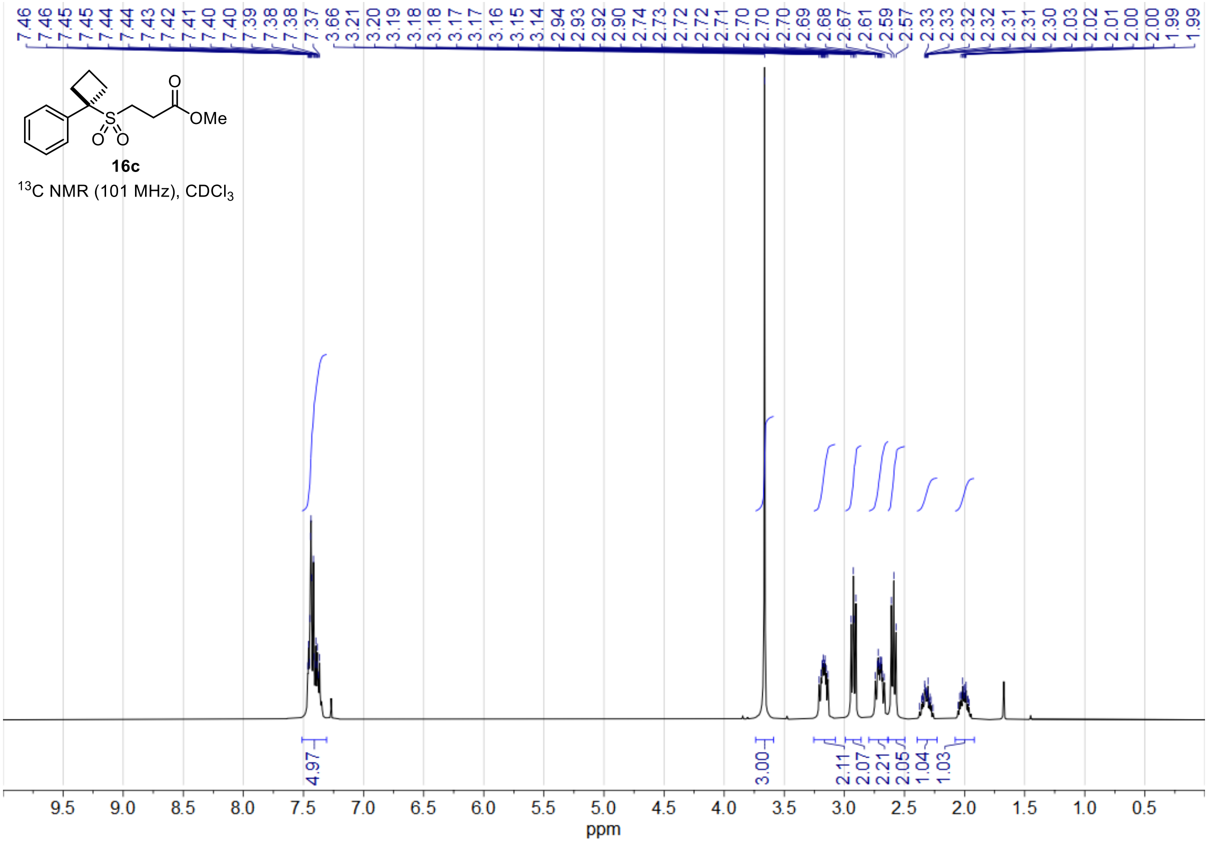
7.46 7.46 7.45 7.45 7.44 7.43 7.42 7.41 7.40 7.40 7.39 7.38 7.38 7.37 3.66 3.21 3.20 3.19 3.18 3.18 3.17 3.17 3.16 3.15 3.14 2.94 2.93 2.92 2.90 2.74 2.73 2.72 2.72 2.71 2.70 2.70 2.70 2.69 2.68 2.67 2.61 2.59 2.57 2.33 2.33 2.32 2.32 2.31 2.31 2.30 2.03 2.02 2.01 2.00 2.00 1.99 1.99
O
OMe
S
O O
16c
^{13}C NMR (101 MHz), CDCl$_3$
4.97
3.00
2.11
2.07
2.21
2.05
1.04
1.03
9.5 9.0 8.5 8.0 7.5 7.0 6.5 6.0 5.5 5.0 4.5 4.0 3.5 3.0 2.5 2.0 1.5 1.0 0.5
ppm

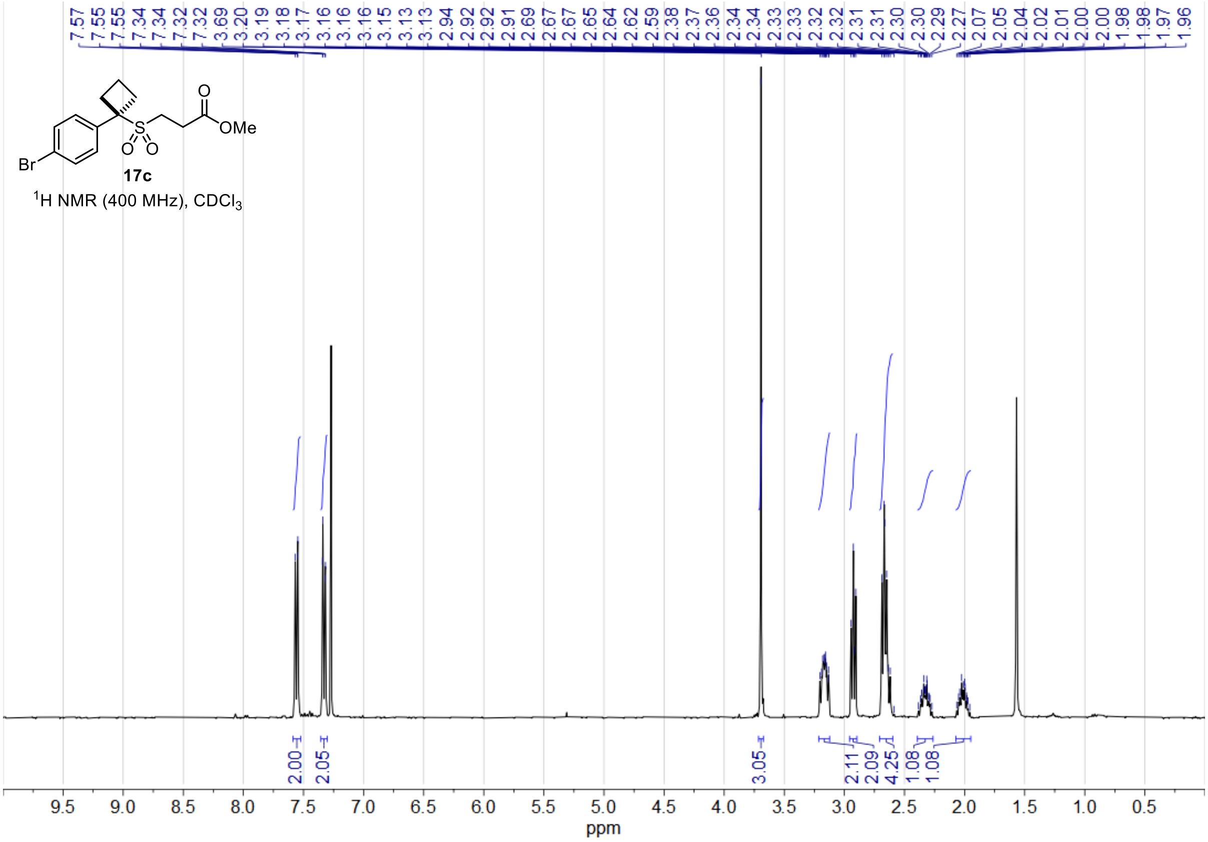

17c

^{1}H NMR (400 MHz), $CDCl_3$

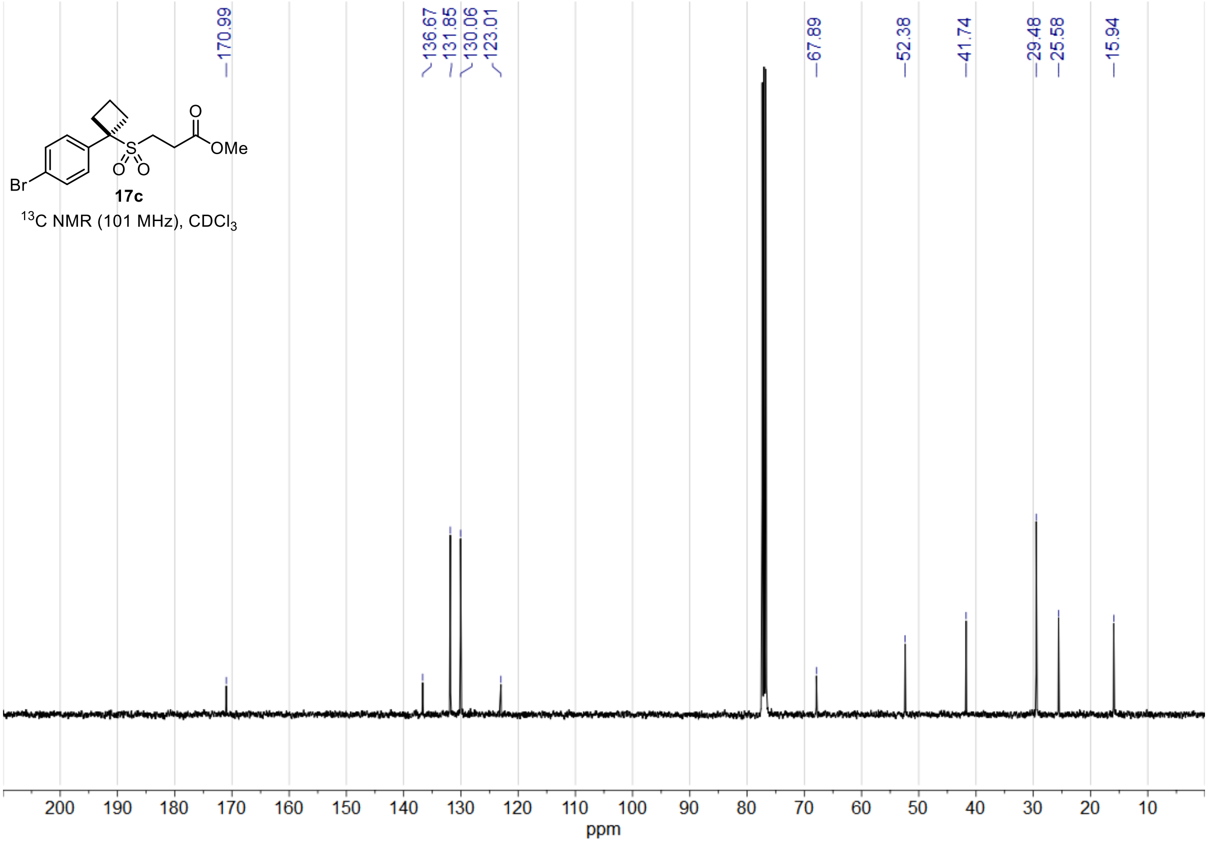

17c

^{13}C NMR (101 MHz), $CDCl_3$

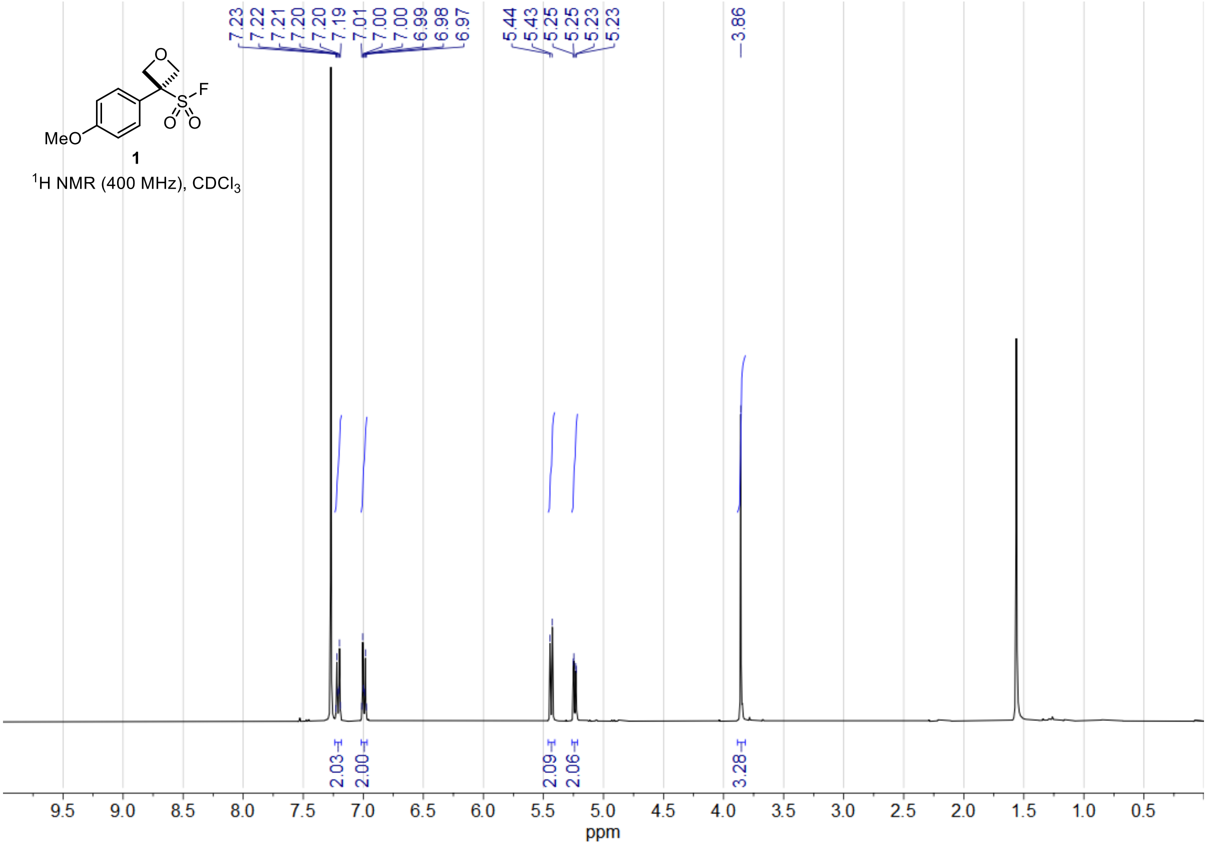

7.23
7.22
7.21
7.20
7.20
7.19
7.01
7.00
7.00
6.99
6.98
6.97
5.44
5.43
5.25
5.25
5.23
5.23
3.86
MeO
O
S
F
O
O
1
^{1}H NMR (400 MHz), CDCl$_3$
2.03
2.00
2.09
2.06
3.28
9.5 9.0 8.5 8.0 7.5 7.0 6.5 6.0 5.5 5.0 4.5 4.0 3.5 3.0 2.5 2.0 1.5 1.0 0.5
ppm

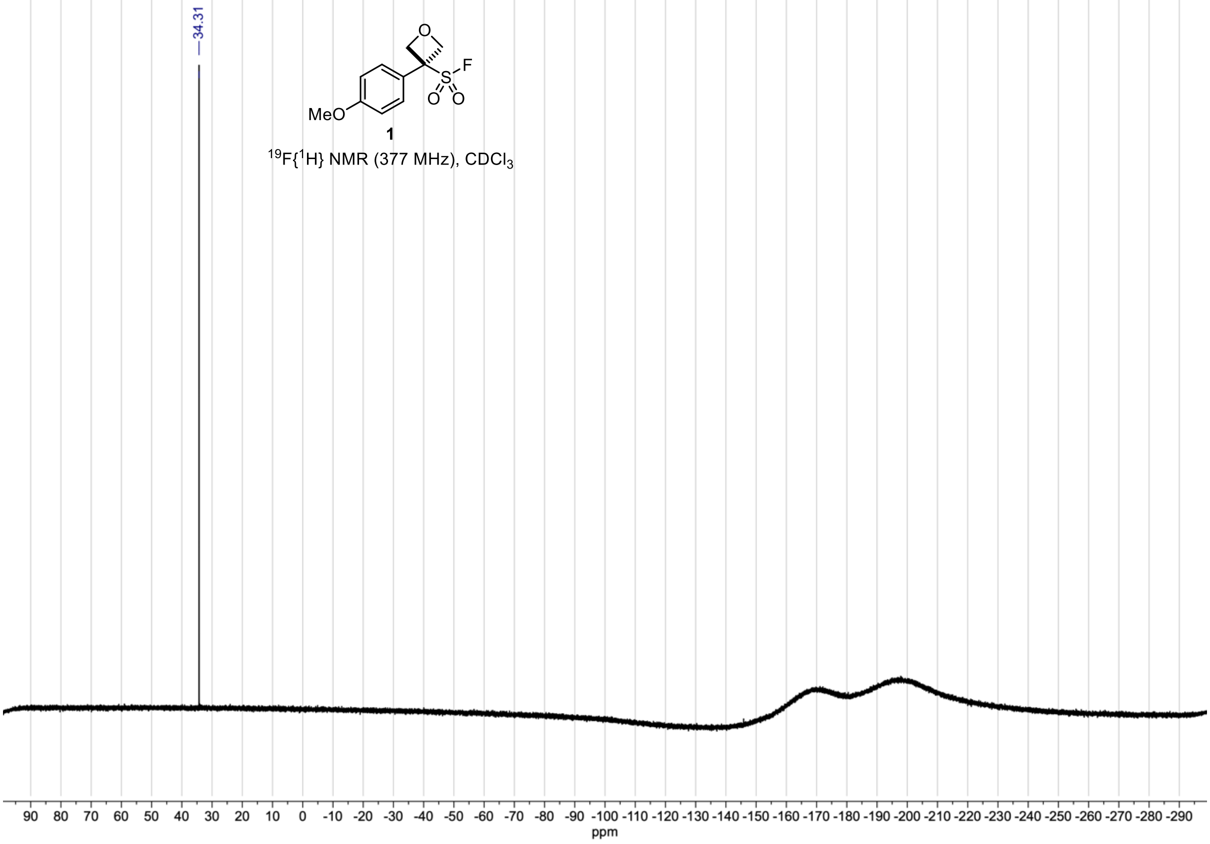

34.31
MeO
O
S
F
O
O
1
^{19}F{^{1}H} NMR (377 MHz), CDCl$_3$
90 80 70 60 50 40 30 20 10 0 -10 -20 -30 -40 -50 -60 -70 -80 -90 -100 -110 -120 -130 -140 -150 -160 -170 -180 -190 -200 -210 -220 -230 -240 -250 -260 -270 -280 -290
ppm

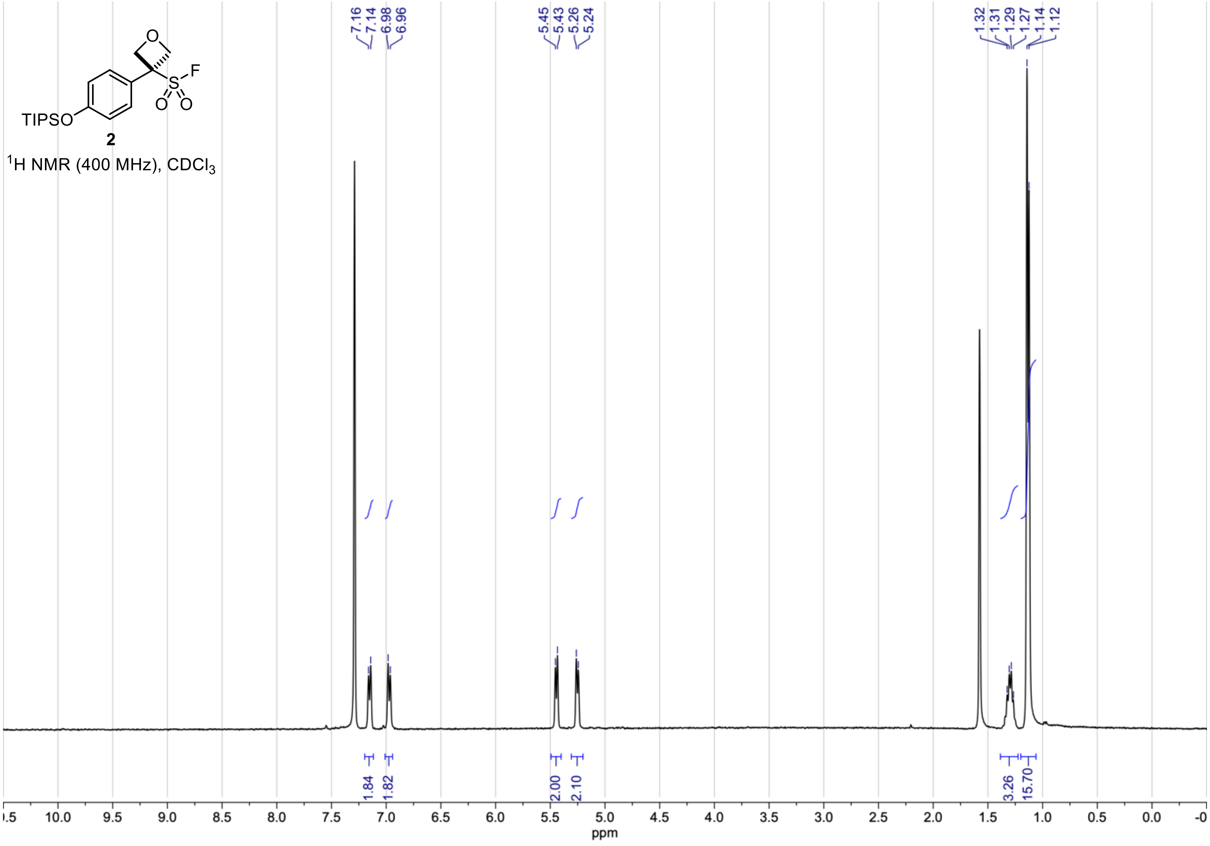
TIPSO
2
^{1}H NMR (400 MHz), CDCl3
7.16
7.14
6.98
6.96
5.45
5.43
5.26
5.24
1.32
1.31
1.29
1.27
1.14
1.12
1.84
1.82
2.00
2.10
3.26
15.70
ppm

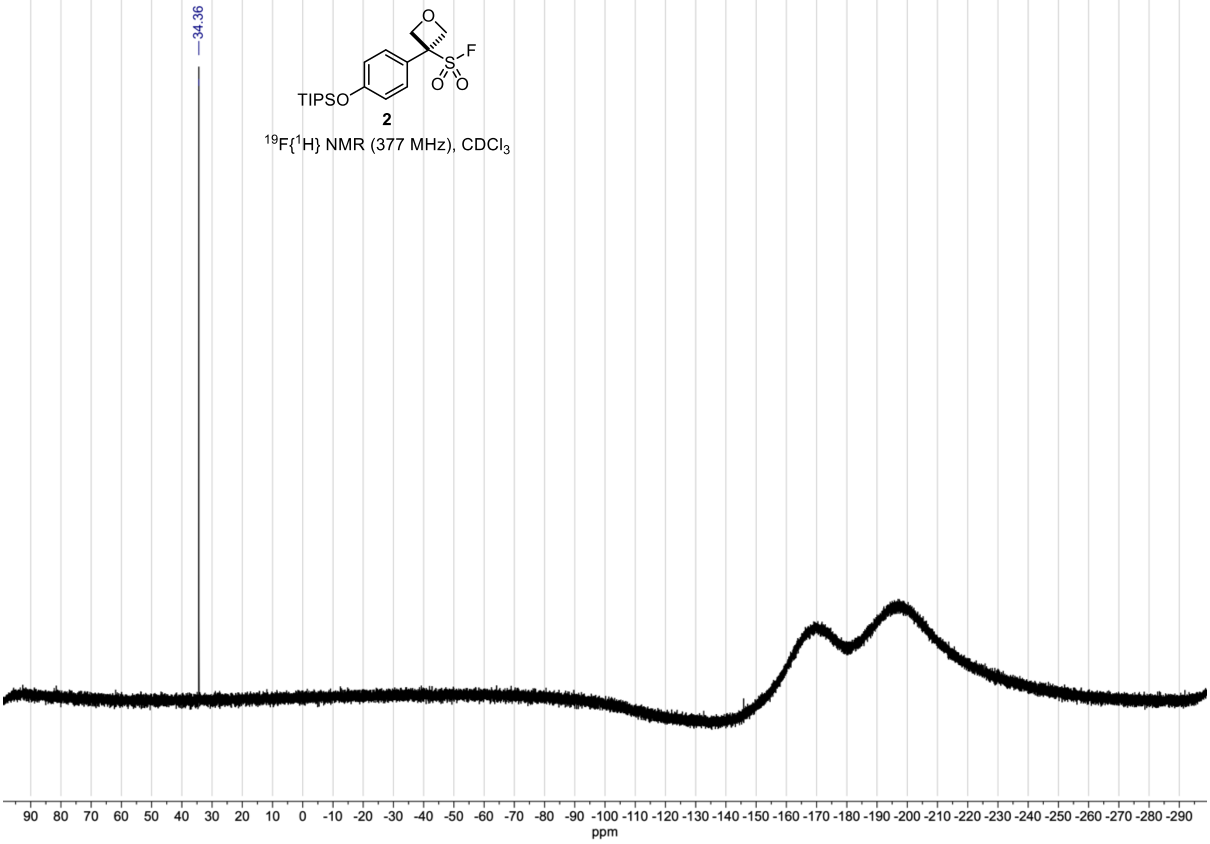
34.36
TIPSO
2
^{19}F{^{1}H} NMR (377 MHz), CDCl3
ppm

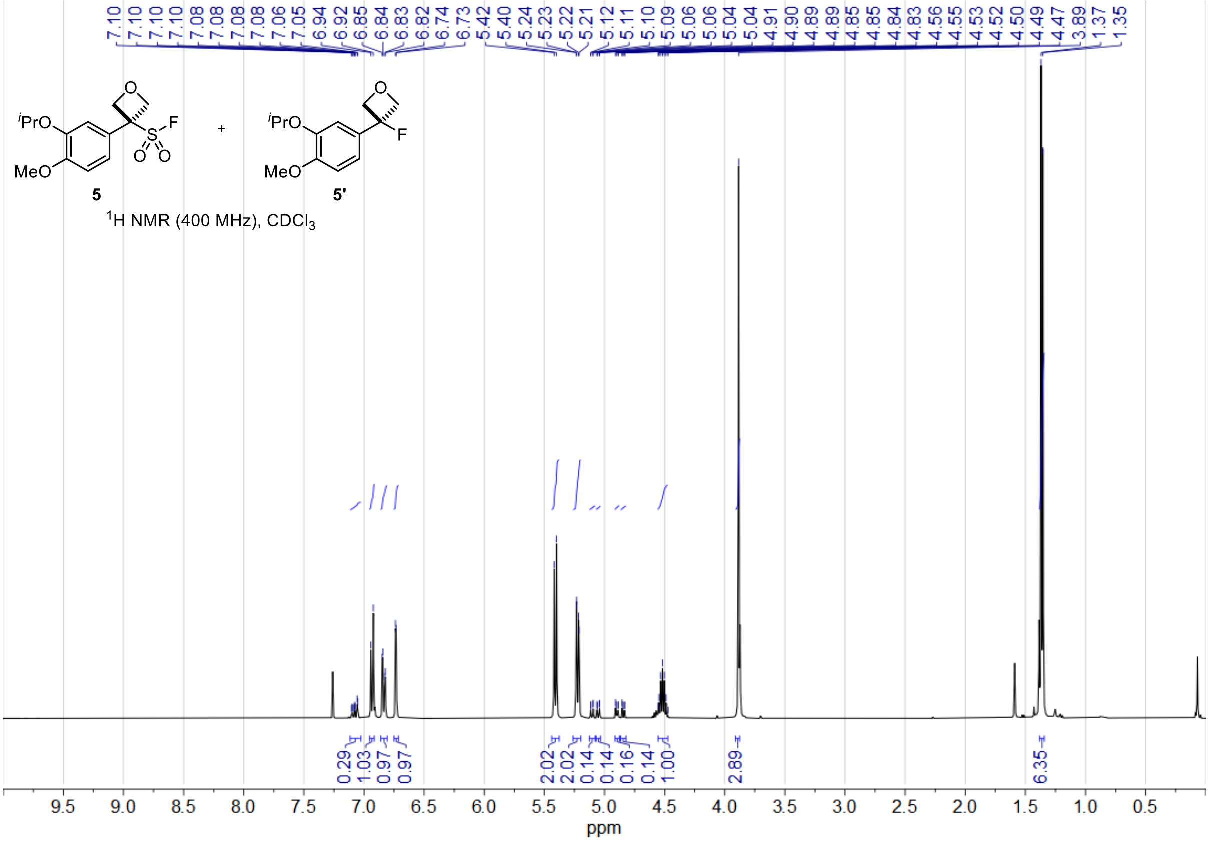
7.10 7.10 7.10 7.10 7.08 7.08 7.08 7.06 7.05 6.94 6.92 6.85 6.84 6.83 6.82 6.74 6.73 5.42 5.40 5.24 5.23 5.22 5.21 5.12 5.11 5.10 5.09 5.06 5.04 5.04 4.91 4.90 4.89 4.89 4.85 4.85 4.84 4.83 4.56 4.55 4.53 4.52 4.50 4.49 4.47 3.89 1.37 1.35
^{i}PrO
MeO
F
O
S
O O
5
+
^{i}PrO
MeO
O
F
5'
^{1}H NMR (400 MHz), CDCl$_3$
0.29 1.03 0.97 0.97 2.02 2.02 0.14 0.14 0.16 0.14 1.00 2.89 6.35
9.5 9.0 8.5 8.0 7.5 7.0 6.5 6.0 5.5 5.0 4.5 4.0 3.5 3.0 2.5 2.0 1.5 1.0 0.5
ppm

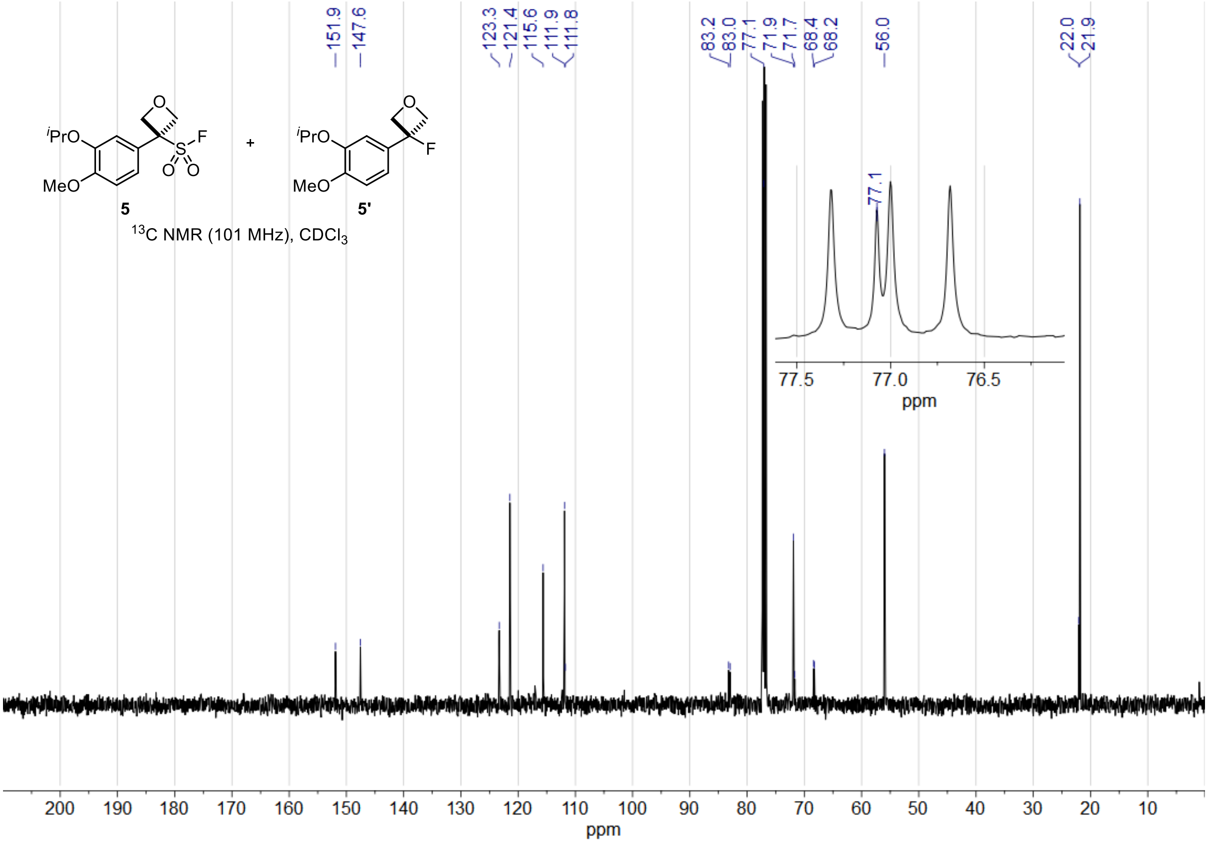
151.9 147.6 123.3 121.4 115.6 111.9 111.8 83.2 83.0 77.1 71.9 71.7 68.4 68.2 56.0 22.0 21.9
^{i}PrO
MeO
F
O
S
O O
5
+
^{i}PrO
MeO
O
F
5'
^{13}C NMR (101 MHz), CDCl$_3$
77.1
77.5 77.0 76.5
ppm
200 190 180 170 160 150 140 130 120 110 100 90 80 70 60 50 40 30 20 10
ppm

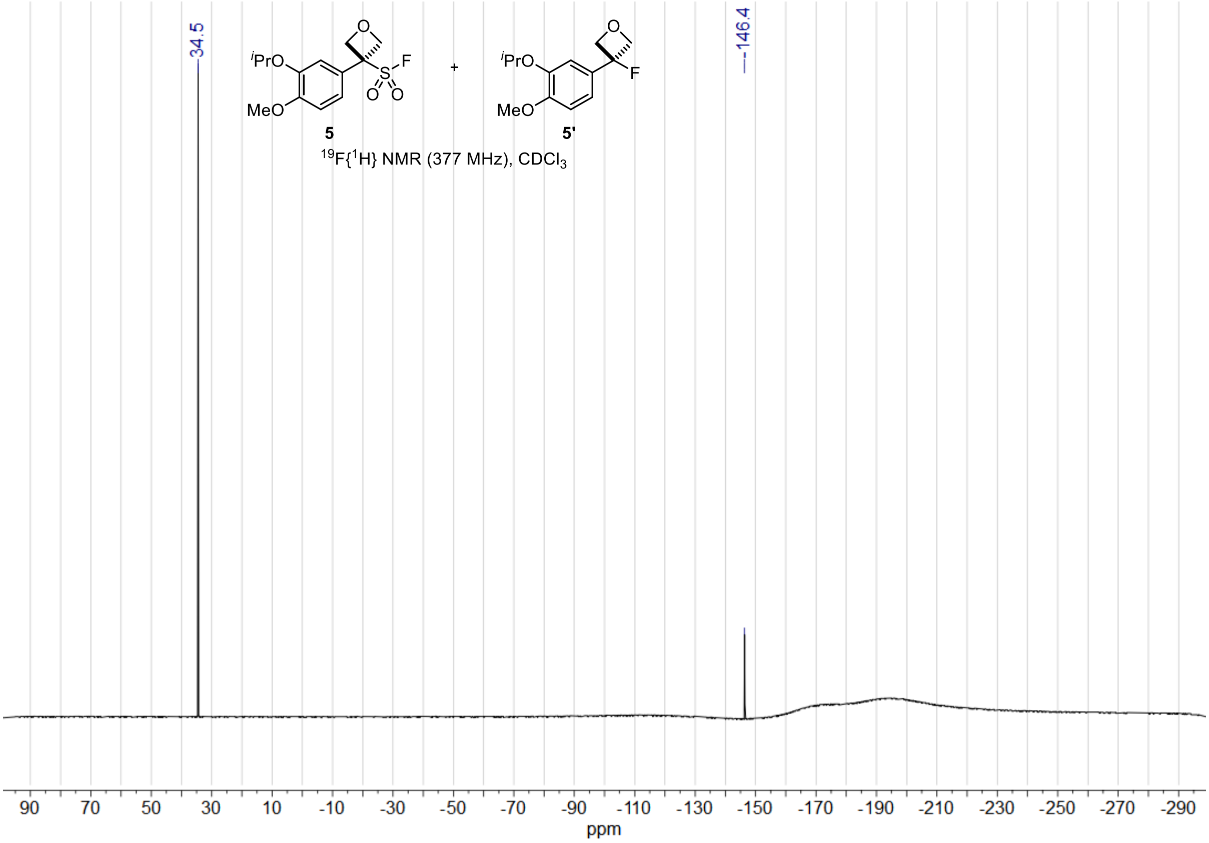
34.5
-146.4
iPrO
MeO
5
+
5'
19F{1H} NMR (377 MHz), CDCl3
90
70
50
30
10
-10
-30
-50
-70
-90
-110
-130
-150
-170
-190
-210
-230
-250
-270
-290
ppm

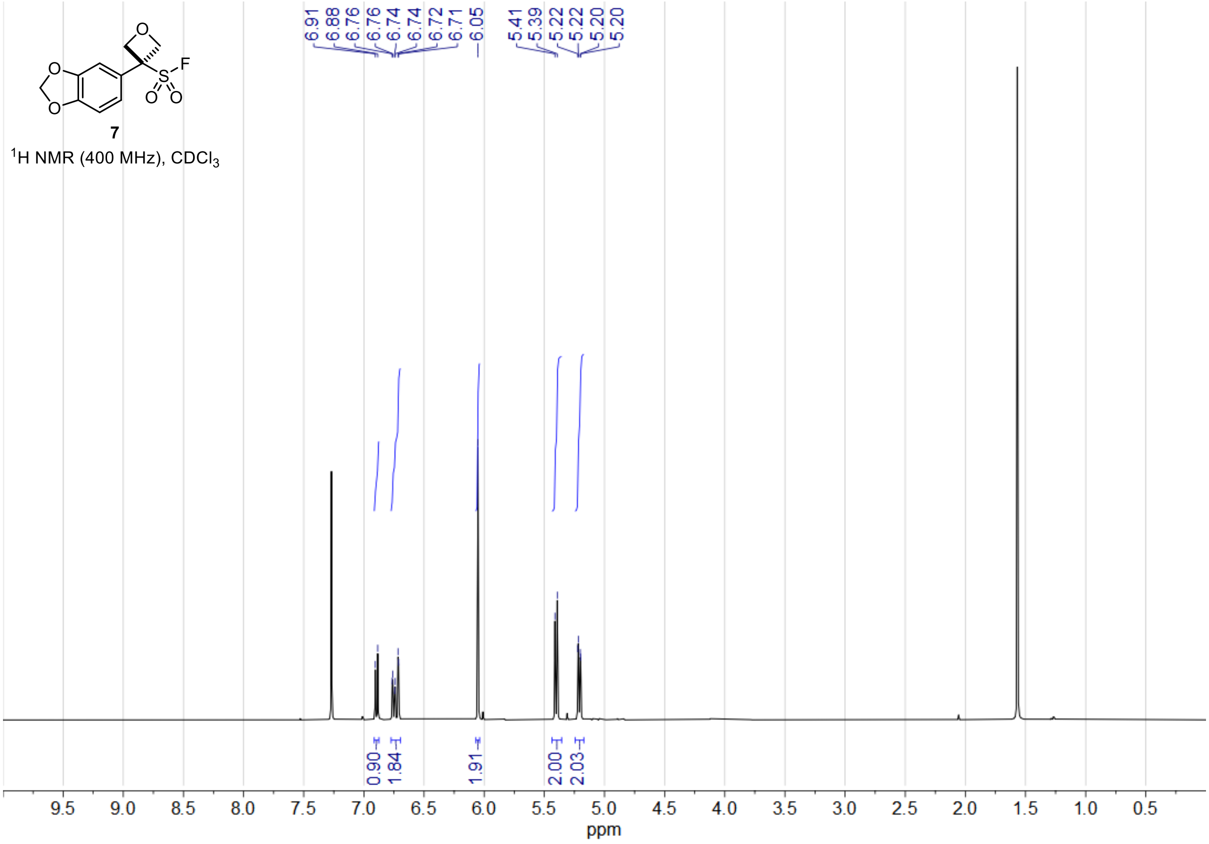
7
1H NMR (400 MHz), CDCl3
6.91
6.88
6.76
6.76
6.74
6.74
6.72
6.71
6.05
5.41
5.39
5.22
5.22
5.20
5.20
0.90
1.84
1.91
2.00
2.03
ppm

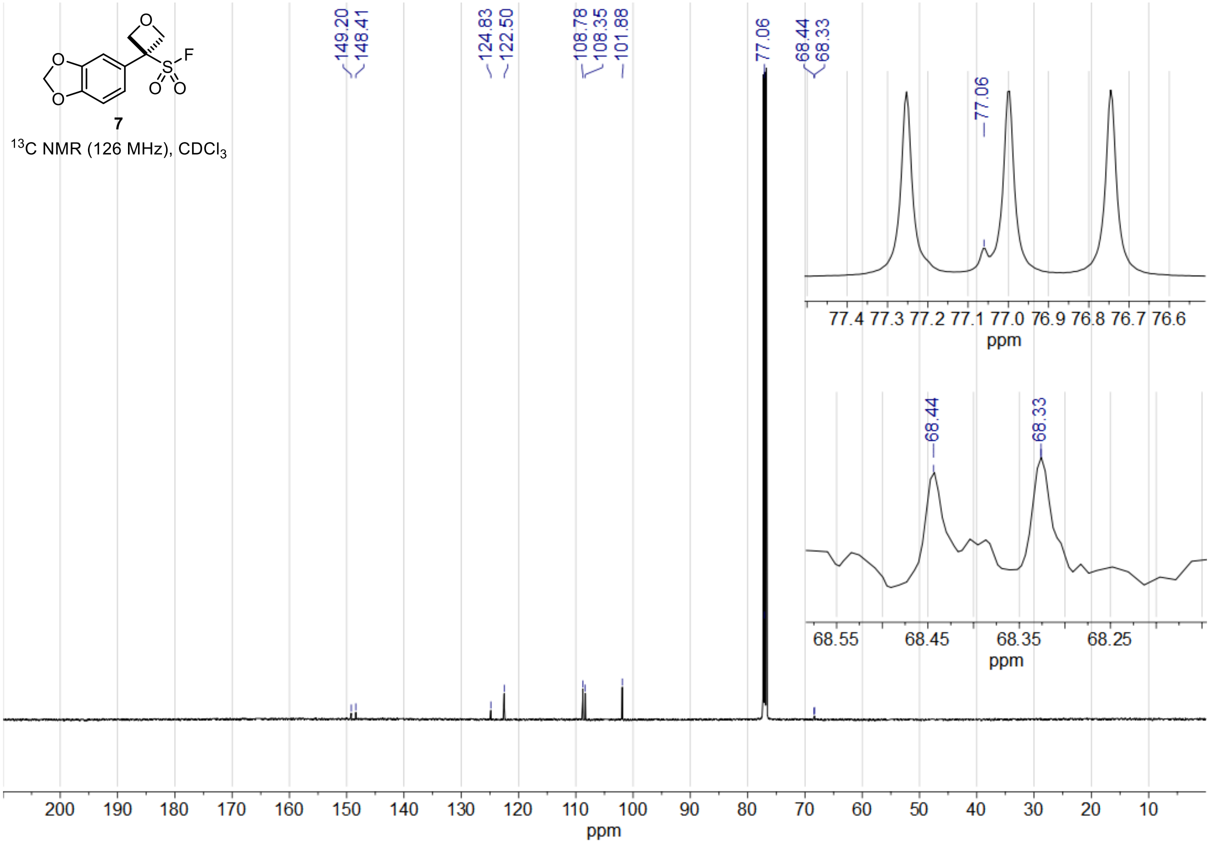
7
13C NMR (126 MHz), CDCl3
149.20
148.41
124.83
122.50
108.78
108.35
101.88
77.06
68.44
68.33
ppm

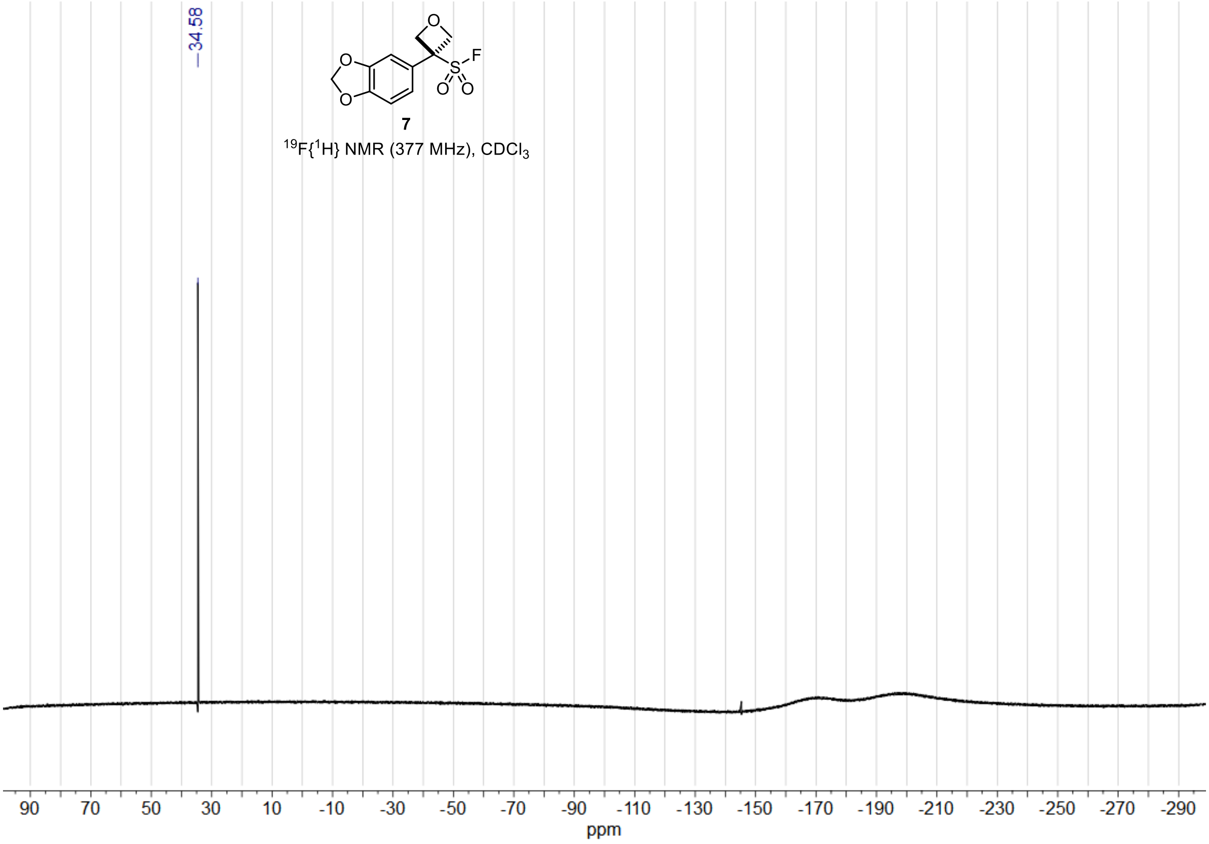

34.58
7
$^{19}F\{^1H\}$ NMR (377 MHz), $CDCl_3$
90
70
50
30
10
-10
-30
-50
-70
-90
-110
-130
-150
-170
-190
-210
-230
-250
-270
-290
ppm

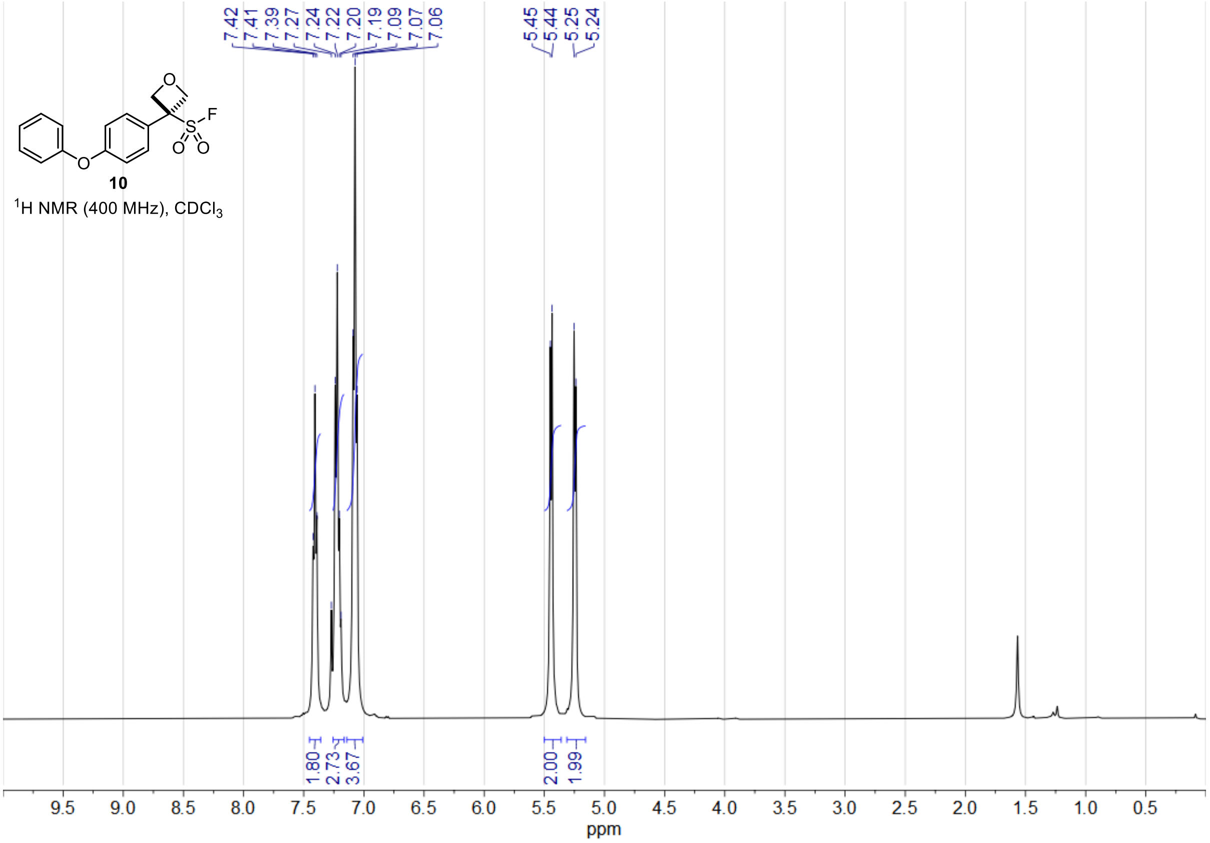
7.42 7.41 7.39 7.27 7.24 7.22 7.20 7.19 7.09 7.07 7.06
5.45 5.44 5.25 5.24
O
S
F
O
O
O
10
1H NMR (400 MHz), CDCl3
1.80 2.73 3.67 2.00 1.99
9.5 9.0 8.5 8.0 7.5 7.0 6.5 6.0 5.5 5.0 4.5 4.0 3.5 3.0 2.5 2.0 1.5 1.0 0.5
ppm

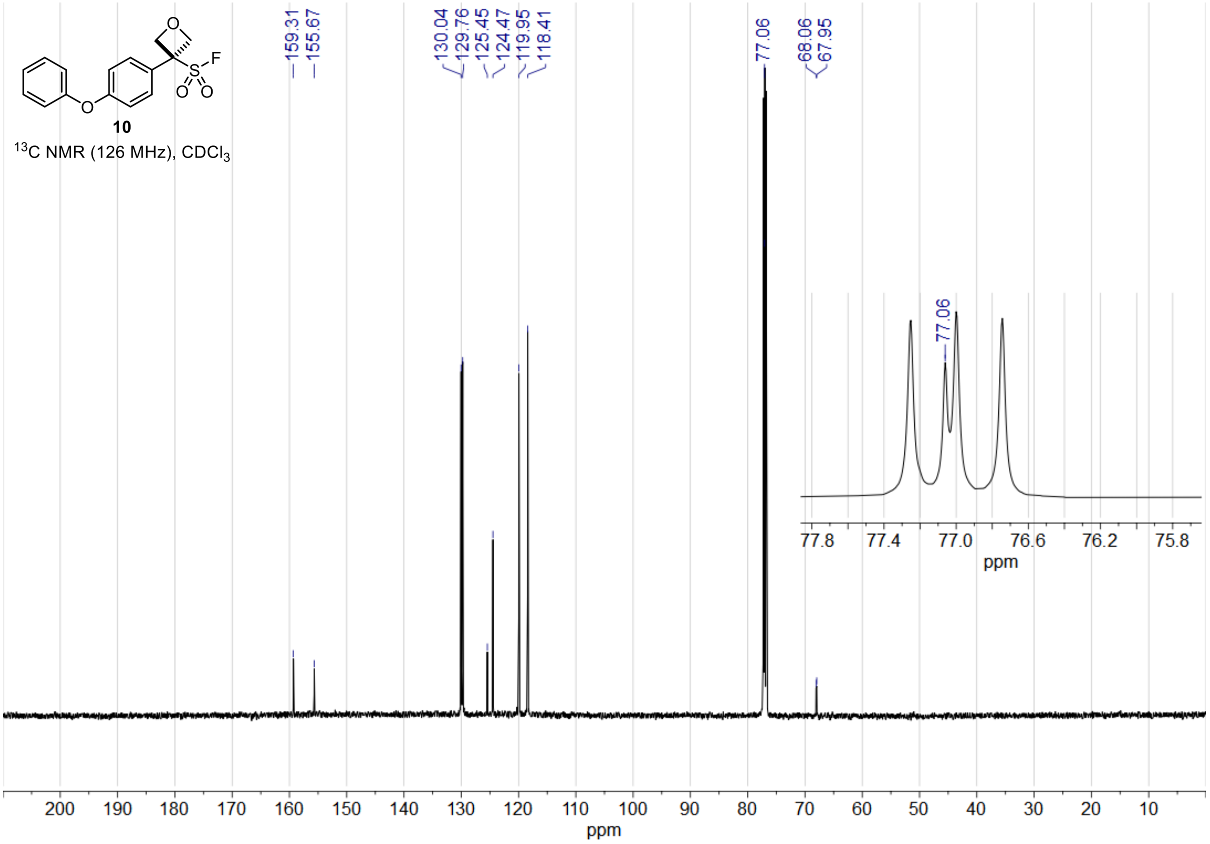
159.31 155.67
130.04 129.76 125.45 124.47 119.95 118.41
77.06
68.06 67.95
O
S
F
O
O
O
10
13C NMR (126 MHz), CDCl3
77.06
77.8 77.4 77.0 76.6 76.2 75.8
ppm
200 190 180 170 160 150 140 130 120 110 100 90 80 70 60 50 40 30 20 10
ppm

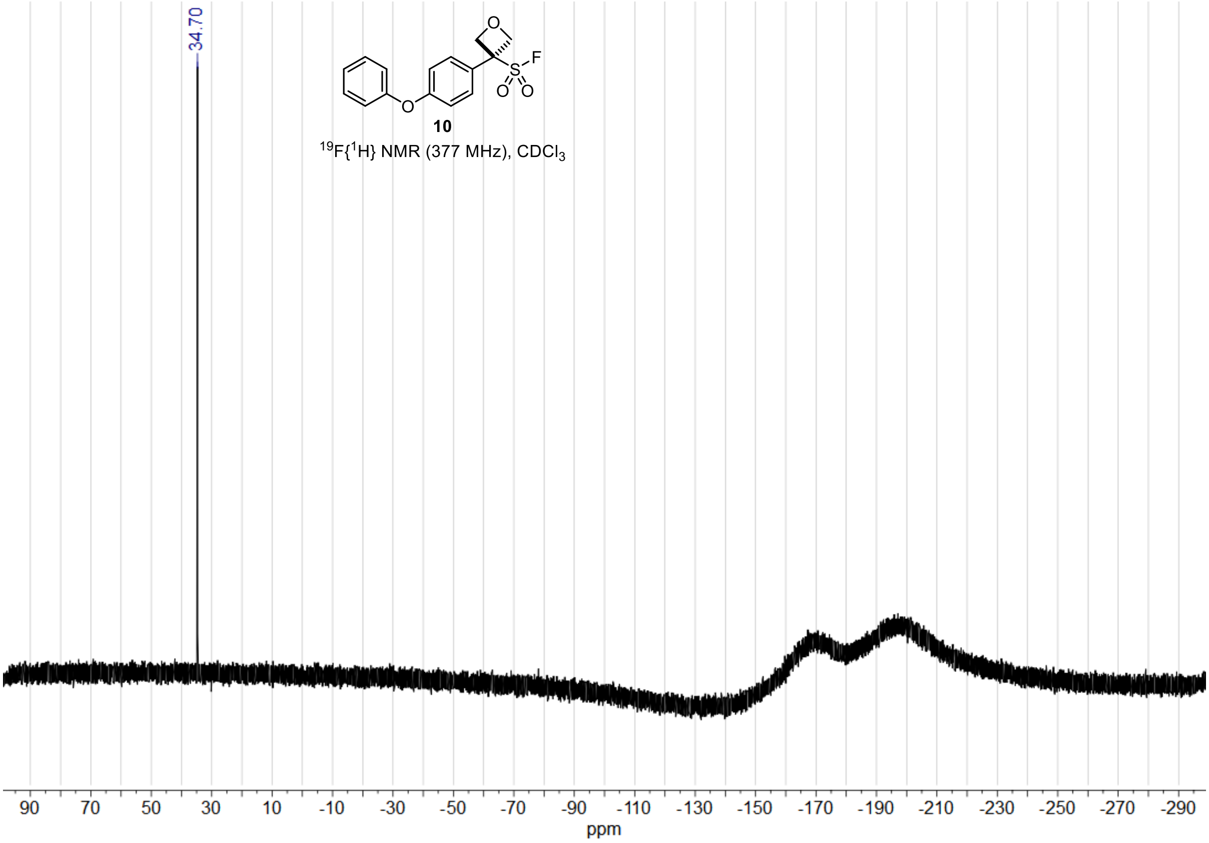
34.70
10
$^{19}F\{^1H\}$ NMR (377 MHz), $CDCl_3$
90
70
50
30
10
-10
-30
-50
-70
-90
-110
-130
-150
-170
-190
-210
-230
-250
-270
-290
ppm

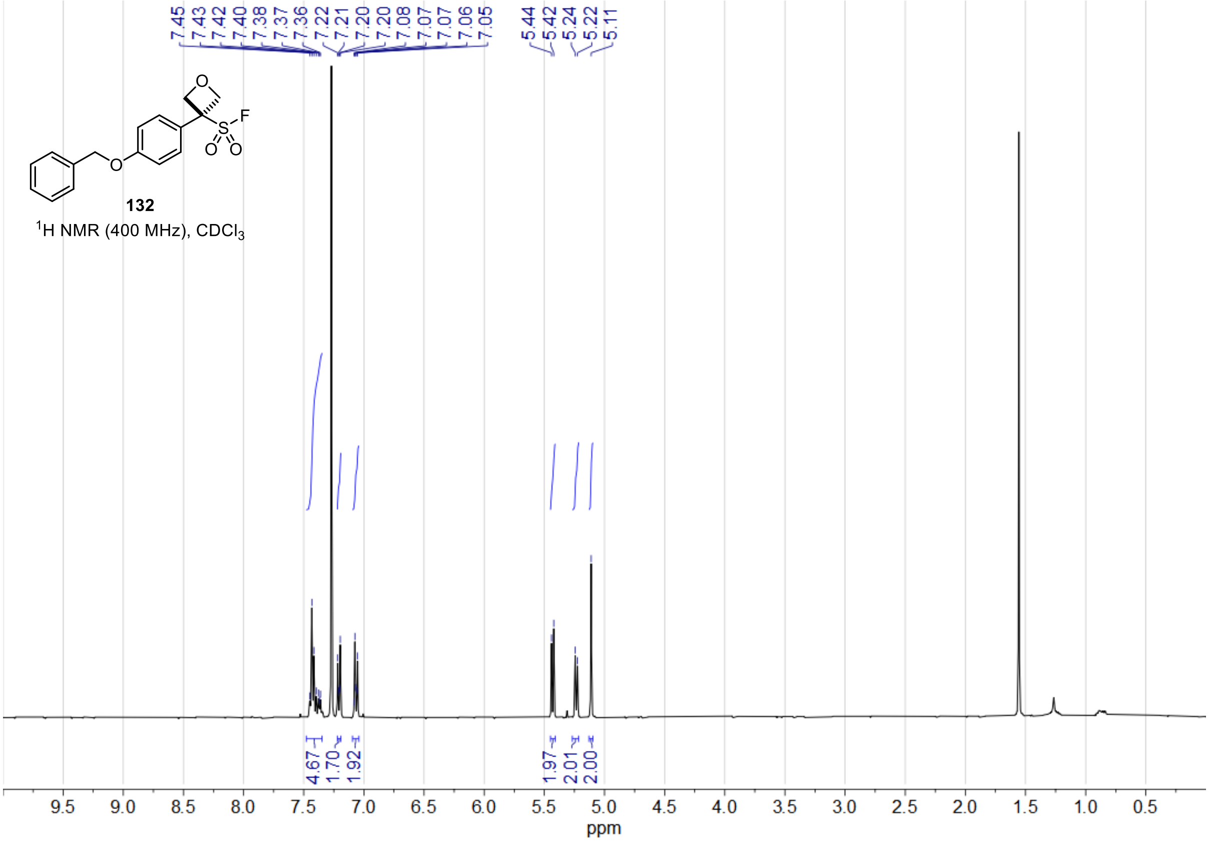
7.45
7.43
7.42
7.40
7.38
7.37
7.36
7.22
7.21
7.20
7.20
7.08
7.07
7.07
7.06
7.05
5.44
5.42
5.24
5.22
5.11
O
S
F
O
O
O
132
^{1}H NMR (400 MHz), $CDCl_3$
4.67
1.70
1.92
1.97
2.01
2.00
9.5
9.0
8.5
8.0
7.5
7.0
6.5
6.0
5.5
5.0
4.5
4.0
3.5
3.0
2.5
2.0
1.5
1.0
0.5
ppm

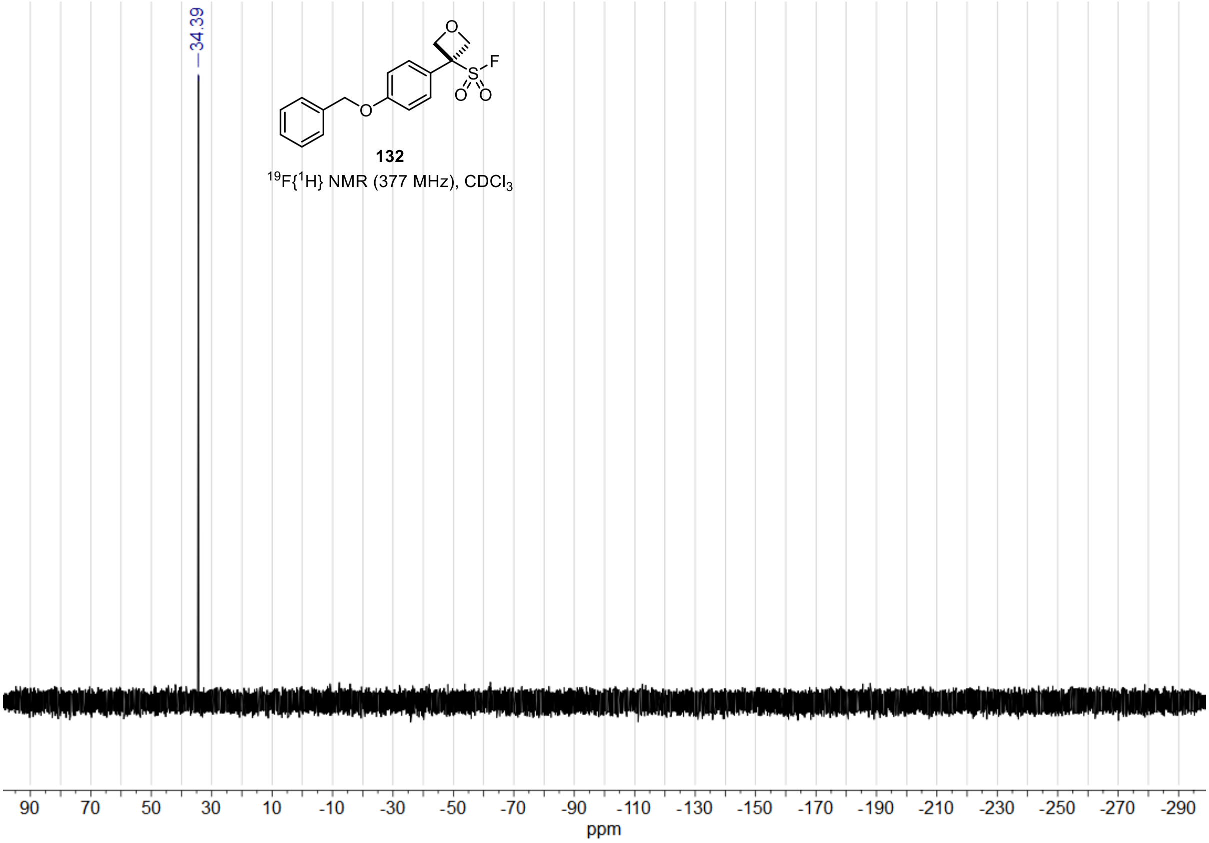
—34.39
O
S
F
O
O
O
132
^{19}F{^{1}H} NMR (377 MHz), $CDCl_3$
90
70
50
30
10
-10
-30
-50
-70
-90
-110
-130
-150
-170
-190
-210
-230
-250
-270
-290
ppm

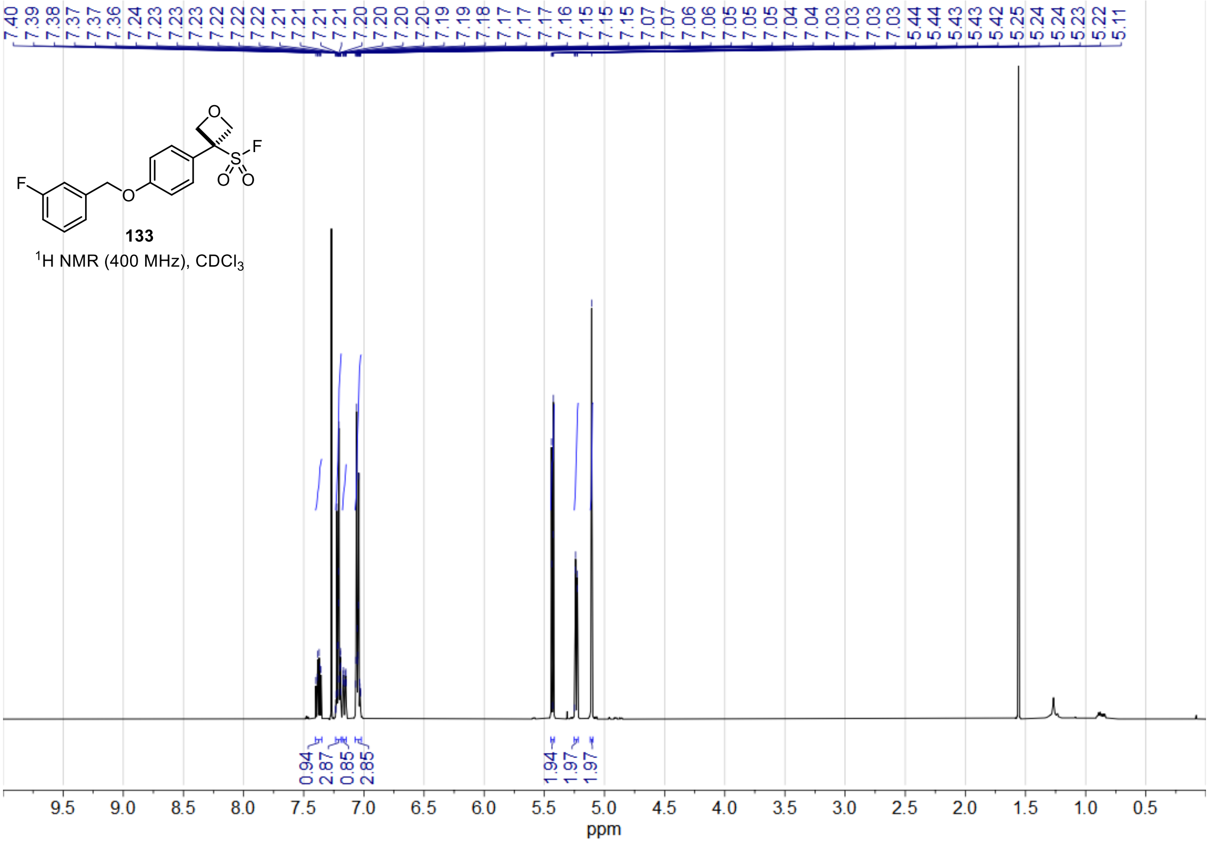
133
1H NMR (400 MHz), CDCl3
7.40 7.39 7.38 7.37 7.37 7.36 7.24 7.23 7.23 7.23 7.22 7.22 7.22 7.22 7.21 7.21 7.21 7.21 7.20 7.20 7.20 7.20 7.19 7.19 7.18 7.17 7.17 7.17 7.16 7.15 7.15 7.15 7.07 7.07 7.06 7.06 7.05 7.05 7.05 7.04 7.04 7.03 7.03 7.03 7.03 5.44 5.44 5.43 5.43 5.42 5.25 5.24 5.24 5.23 5.22 5.11
0.94 2.87 0.85 2.85 1.94 1.97 1.97
ppm

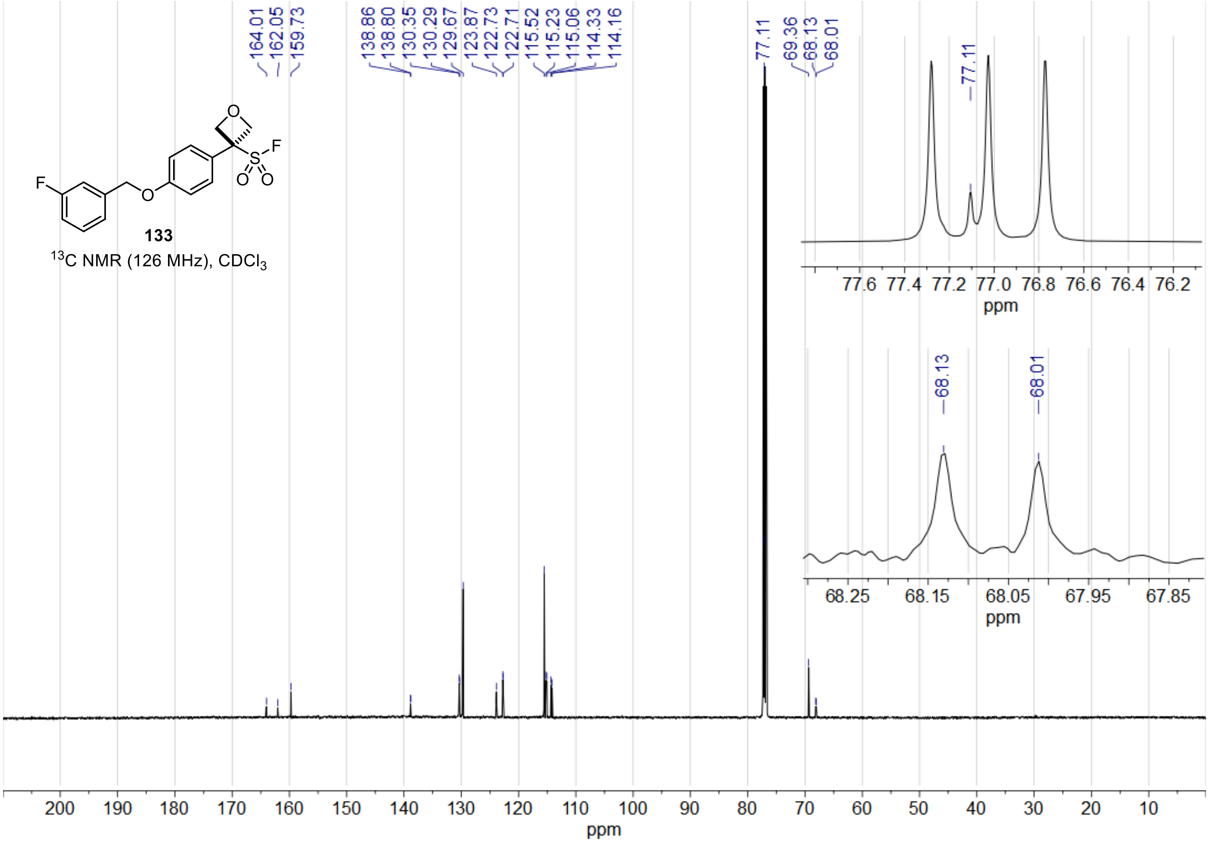
133
13C NMR (126 MHz), CDCl3
164.01 162.05 159.73 138.86 138.80 130.35 130.29 129.67 123.87 122.73 122.71 115.52 115.23 115.06 114.33 114.16 77.11 69.36 68.13 68.01
77.11
68.13 68.01
ppm

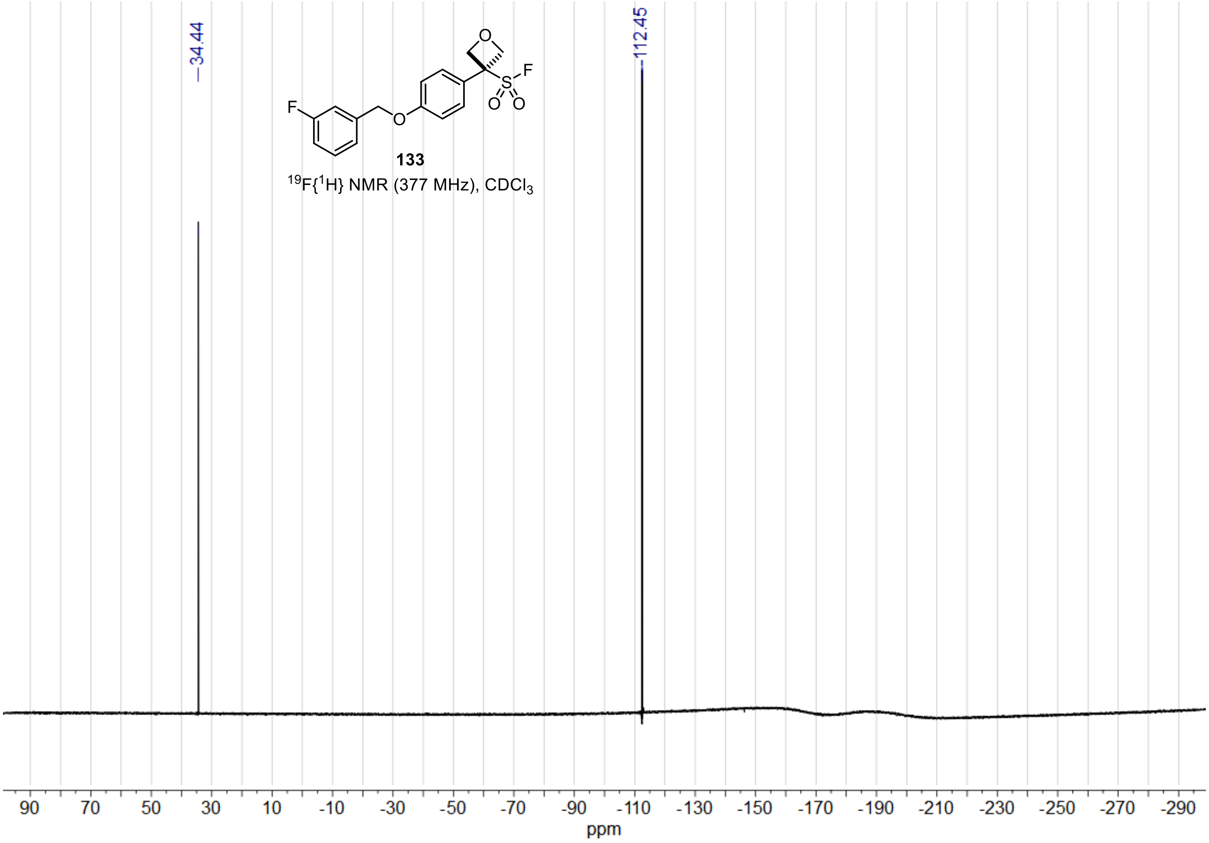

F
O
O
S
F
O O
133
^{19}F{^{1}H} NMR (377 MHz), CDCl$_3$
–34.44
–112.45
90
70
50
30
10
-10
-30
-50
-70
-90
-110
-130
-150
-170
-190
-210
-230
-250
-270
-290
ppm

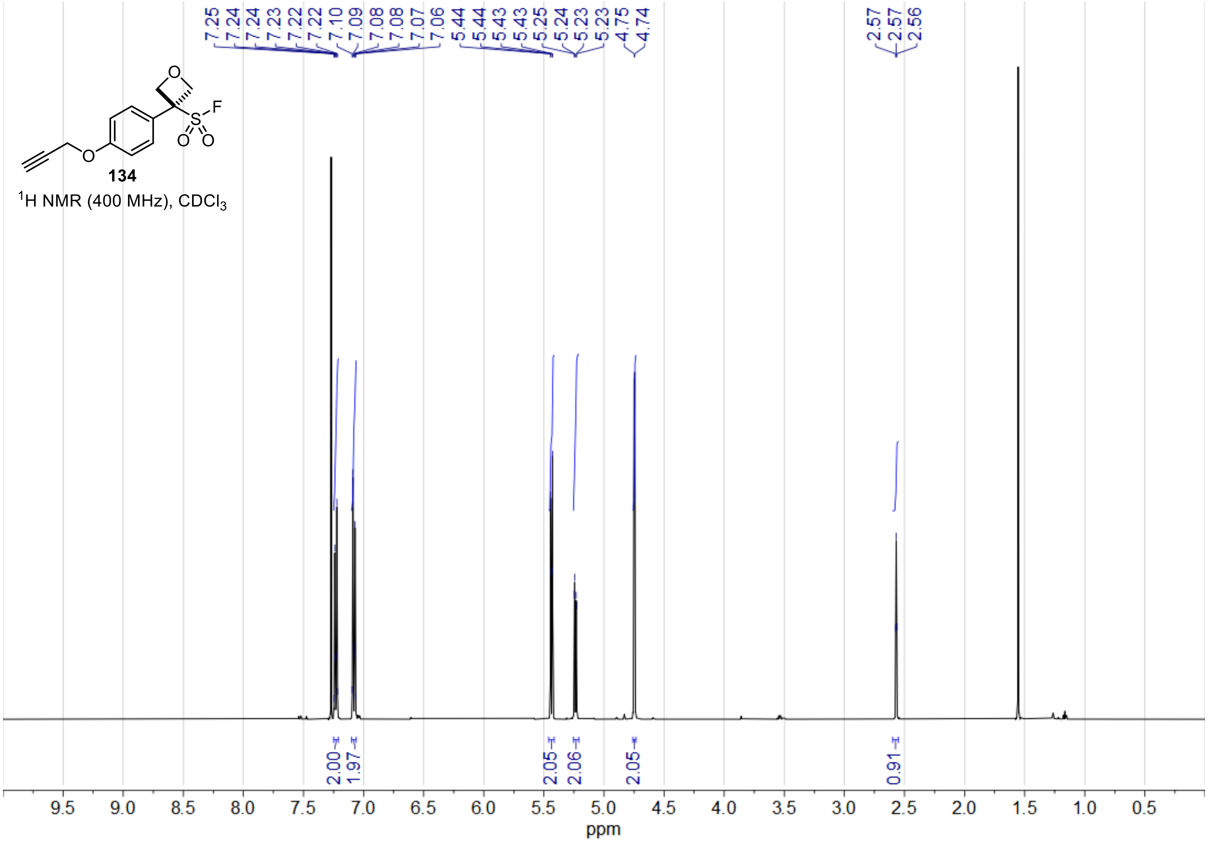
134
1H NMR (400 MHz), CDCl3
7.25
7.24
7.24
7.23
7.22
7.22
7.10
7.09
7.08
7.08
7.07
7.06
5.44
5.44
5.43
5.43
5.25
5.24
5.23
5.23
4.75
4.74
2.57
2.57
2.56
2.00
1.97
2.05
2.06
2.05
0.91
ppm

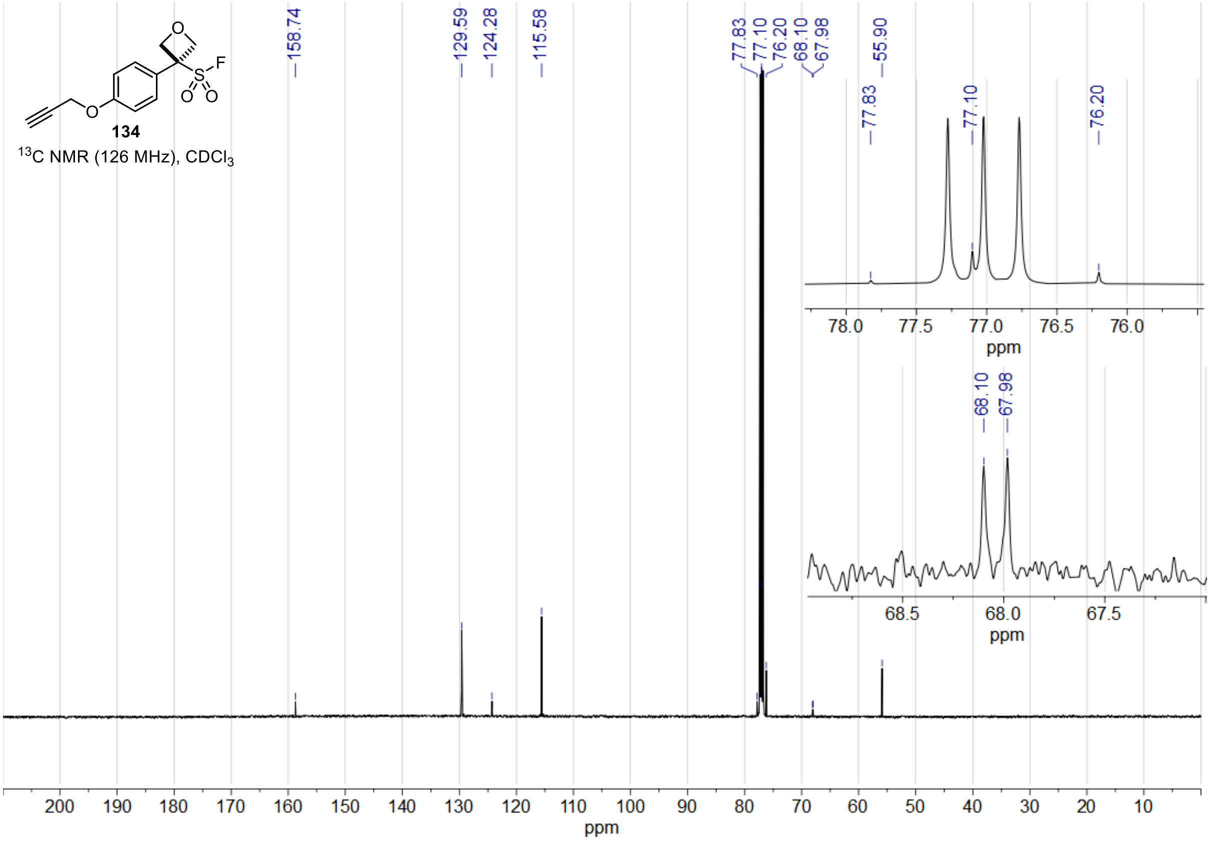
134
13C NMR (126 MHz), CDCl3
158.74
129.59
124.28
115.58
77.83
77.10
76.20
68.10
67.98
55.90
ppm

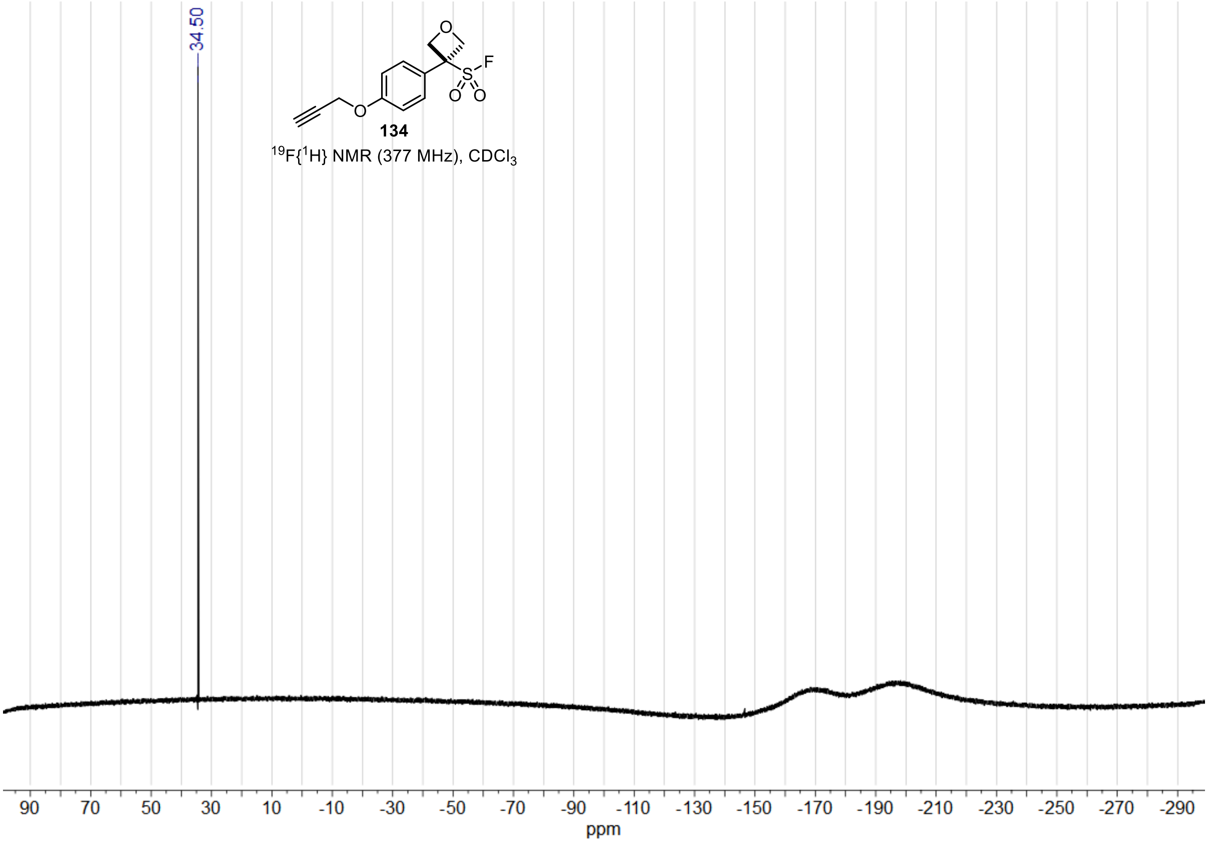

134

$^{19}F\{^{1}H\}$ NMR (377 MHz), $CDCl_3$

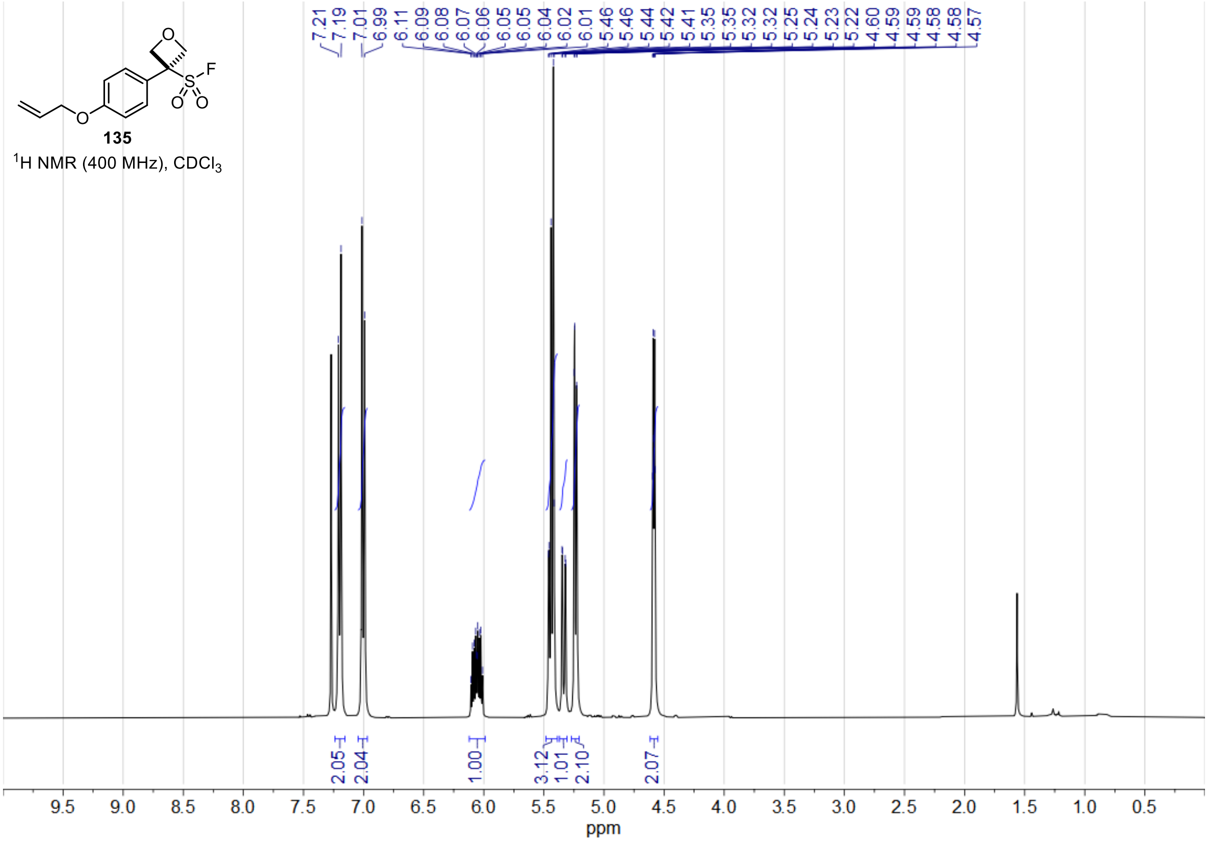
135
¹H NMR (400 MHz), CDCl₃
7.21
7.19
7.01
6.99
6.11
6.09
6.08
6.07
6.06
6.05
6.05
6.04
6.02
6.01
5.46
5.46
5.44
5.42
5.41
5.35
5.35
5.32
5.32
5.25
5.24
5.23
5.22
4.60
4.59
4.59
4.58
4.58
4.57
2.05
2.04
1.00
3.12
1.01
2.10
2.07
ppm

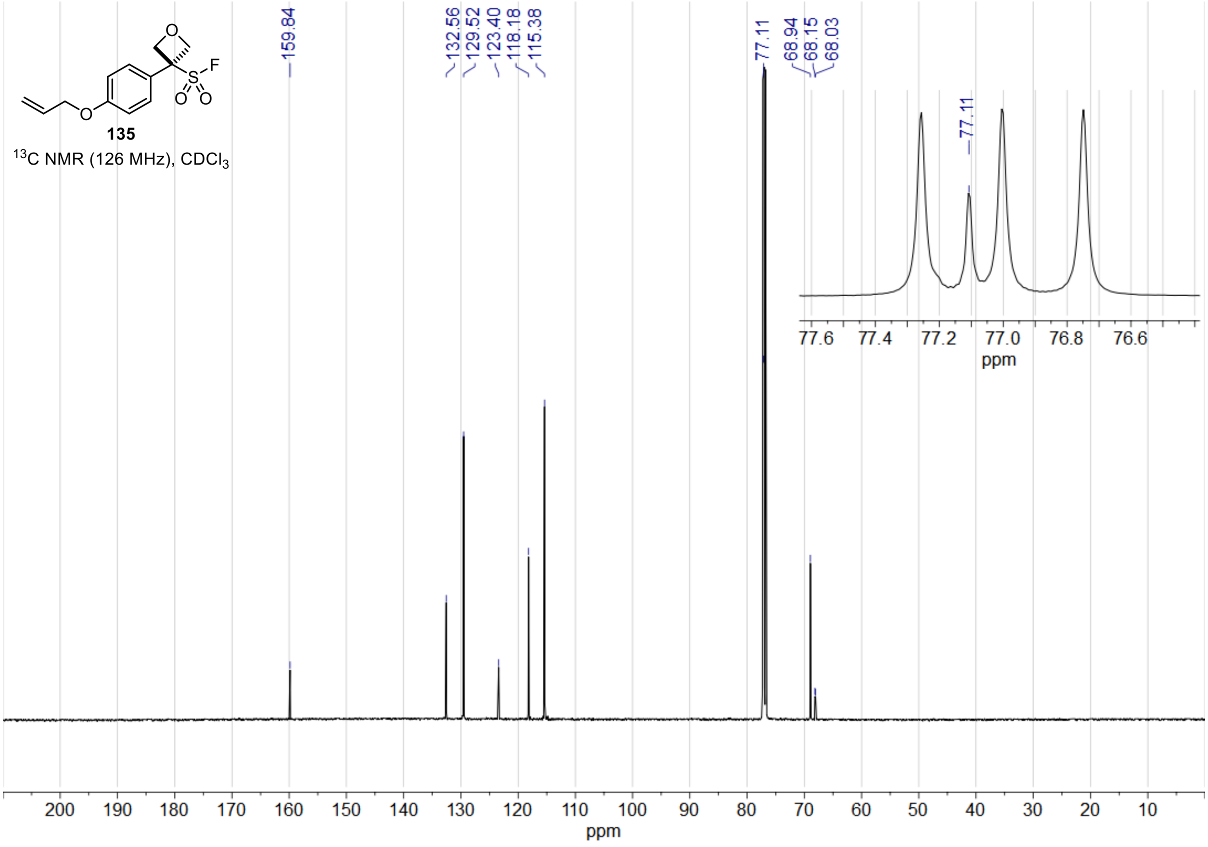
135
¹³C NMR (126 MHz), CDCl₃
159.84
132.56
129.52
123.40
118.18
115.38
77.11
68.94
68.15
68.03
77.11
ppm
ppm

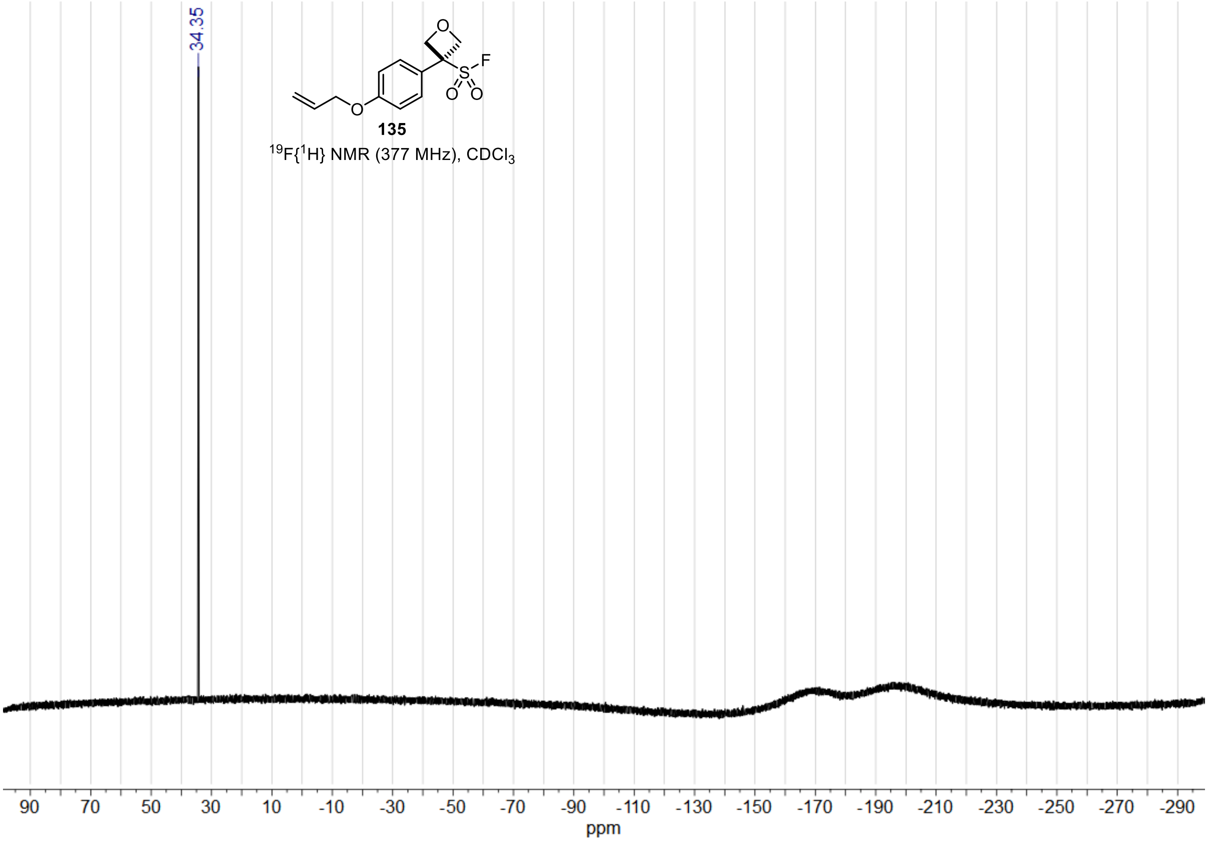

34.35
135
19F{1H} NMR (377 MHz), CDCl3
90
70
50
30
10
-10
-30
-50
-70
-90
-110
-130
-150
-170
-190
-210
-230
-250
-270
-290
ppm

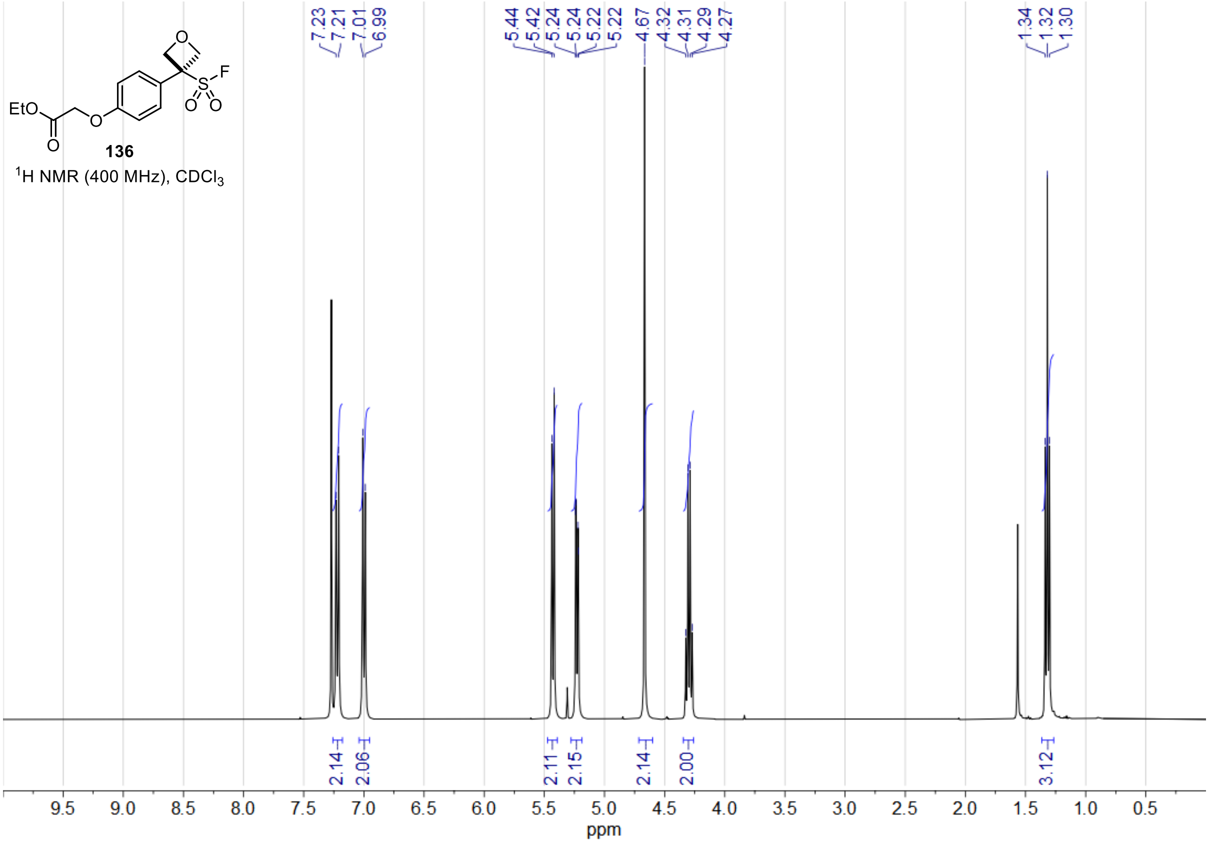
7.23
7.21
7.01
6.99
5.44
5.42
5.24
5.24
5.22
5.22
4.67
4.32
4.31
4.29
4.27
1.34
1.32
1.30
EtO
O
O
O
S
F
O O
136
1H NMR (400 MHz), CDCl3
2.14
2.06
2.11
2.15
2.14
2.00
3.12
9.5 9.0 8.5 8.0 7.5 7.0 6.5 6.0 5.5 5.0 4.5 4.0 3.5 3.0 2.5 2.0 1.5 1.0 0.5
ppm

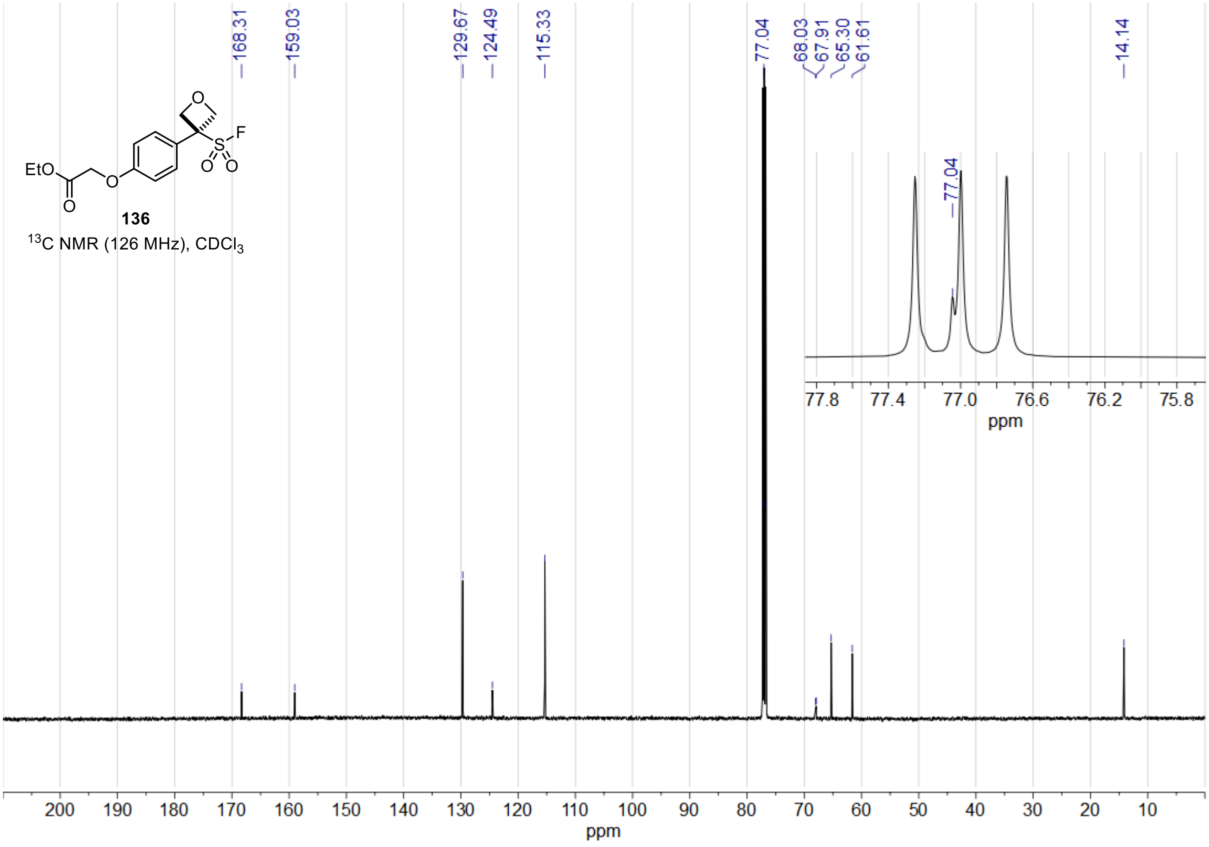
168.31
159.03
129.67
124.49
115.33
77.04
68.03
67.91
65.30
61.61
14.14
EtO
O
O
O
S
F
O O
136
13C NMR (126 MHz), CDCl3
77.04
77.8 77.4 77.0 76.6 76.2 75.8
ppm
200 190 180 170 160 150 140 130 120 110 100 90 80 70 60 50 40 30 20 10
ppm

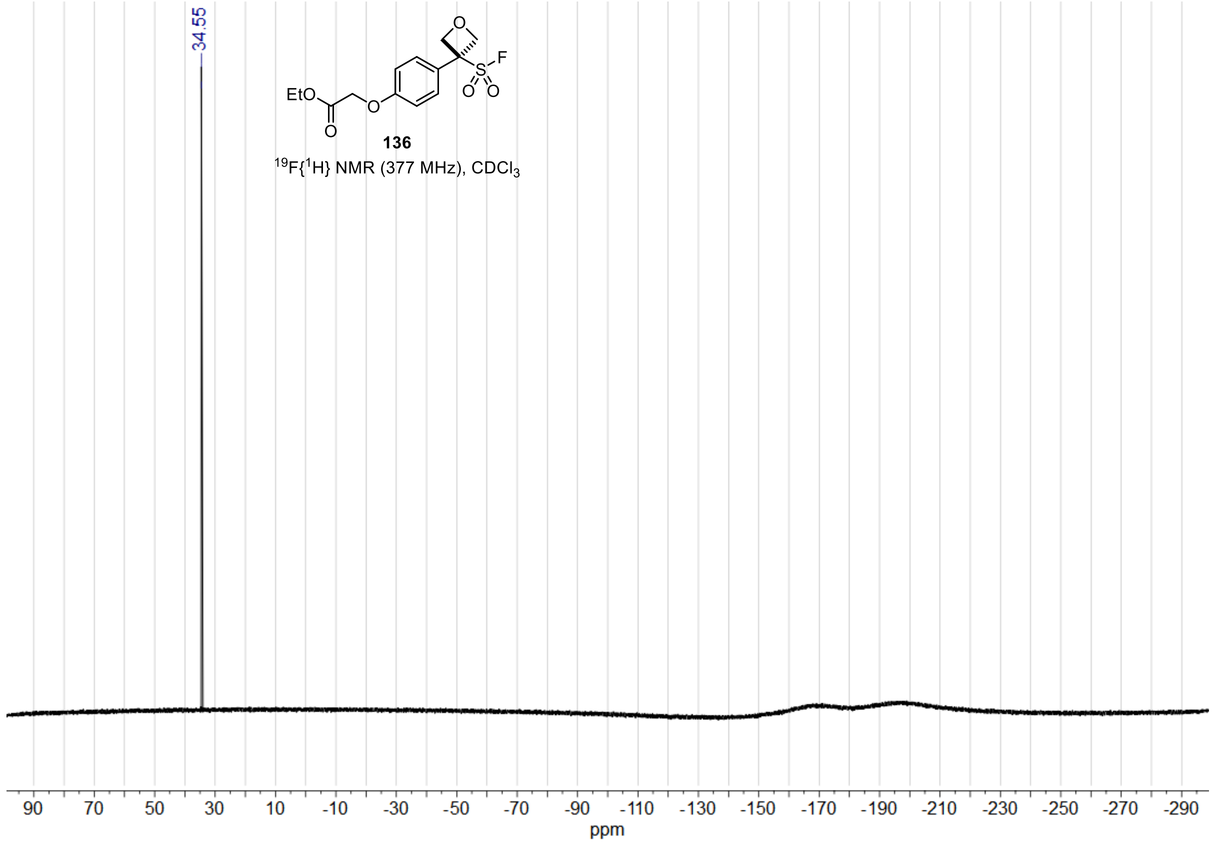
34.55
EtO
O
O
O
S
F
O
O
136
$^{19}F\{^1H\}$ NMR (377 MHz), CDCl$_3$
90
70
50
30
10
-10
-30
-50
-70
-90
-110
-130
-150
-170
-190
-210
-230
-250
-270
-290
ppm

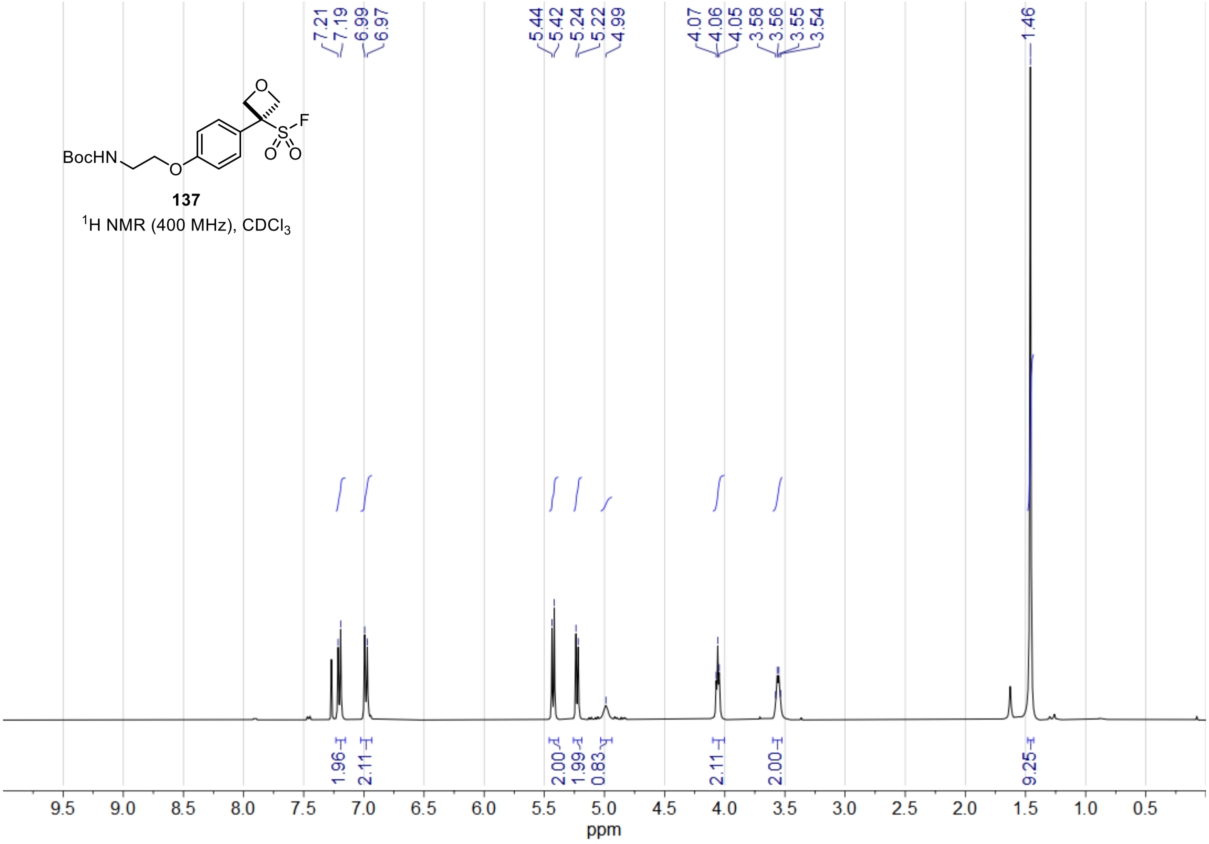
BocHN
O
O
S
F
O O
137
^{1}H NMR (400 MHz), $CDCl_3$
7.21
7.19
6.99
6.97
5.44
5.42
5.24
5.22
4.99
4.07
4.06
4.05
3.58
3.56
3.55
3.54
1.46
1.96
2.11
2.00
1.99
0.83
2.11
2.00
9.25
9.5 9.0 8.5 8.0 7.5 7.0 6.5 6.0 5.5 5.0 4.5 4.0 3.5 3.0 2.5 2.0 1.5 1.0 0.5
ppm

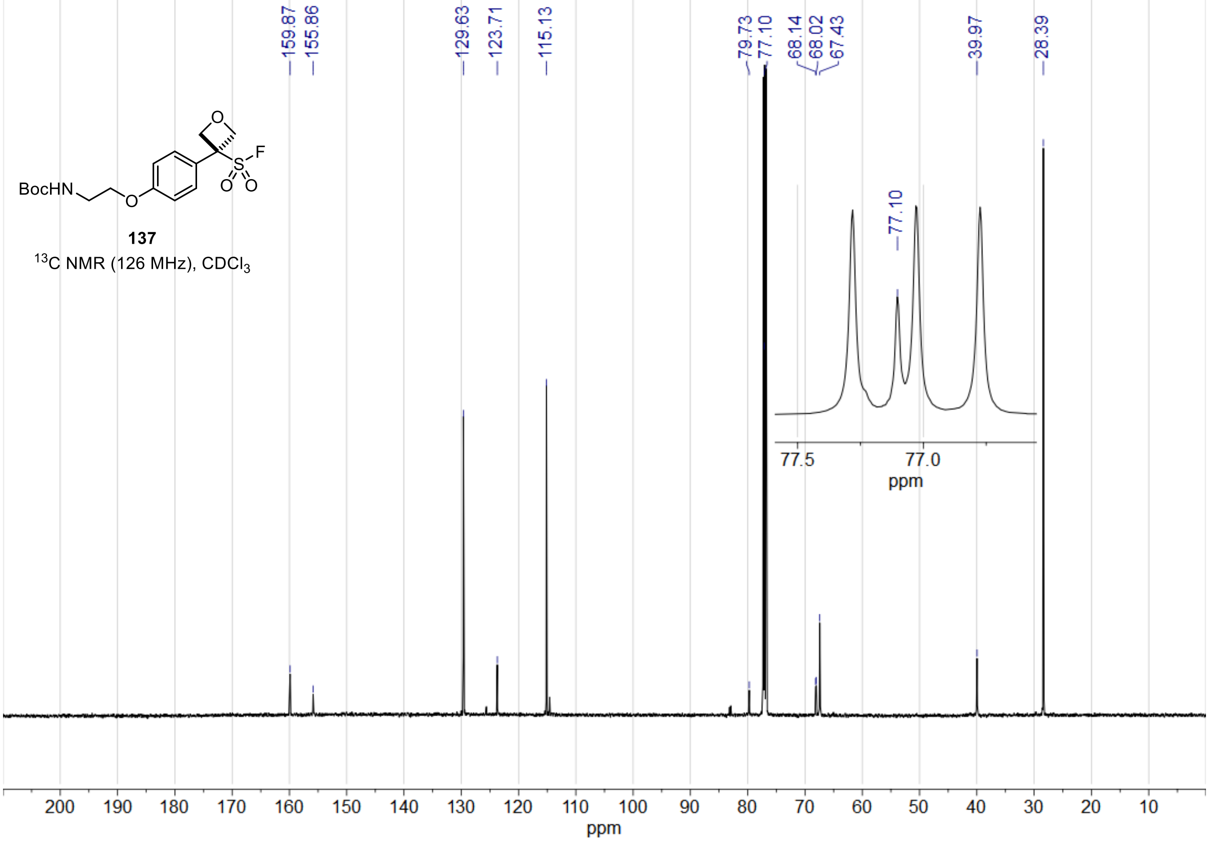
BocHN
O
O
S
F
O O
137
^{13}C NMR (126 MHz), $CDCl_3$
159.87
155.86
129.63
123.71
115.13
79.73
77.10
68.14
68.02
67.43
39.97
28.39
77.10
77.5 77.0
ppm
200 190 180 170 160 150 140 130 120 110 100 90 80 70 60 50 40 30 20 10
ppm

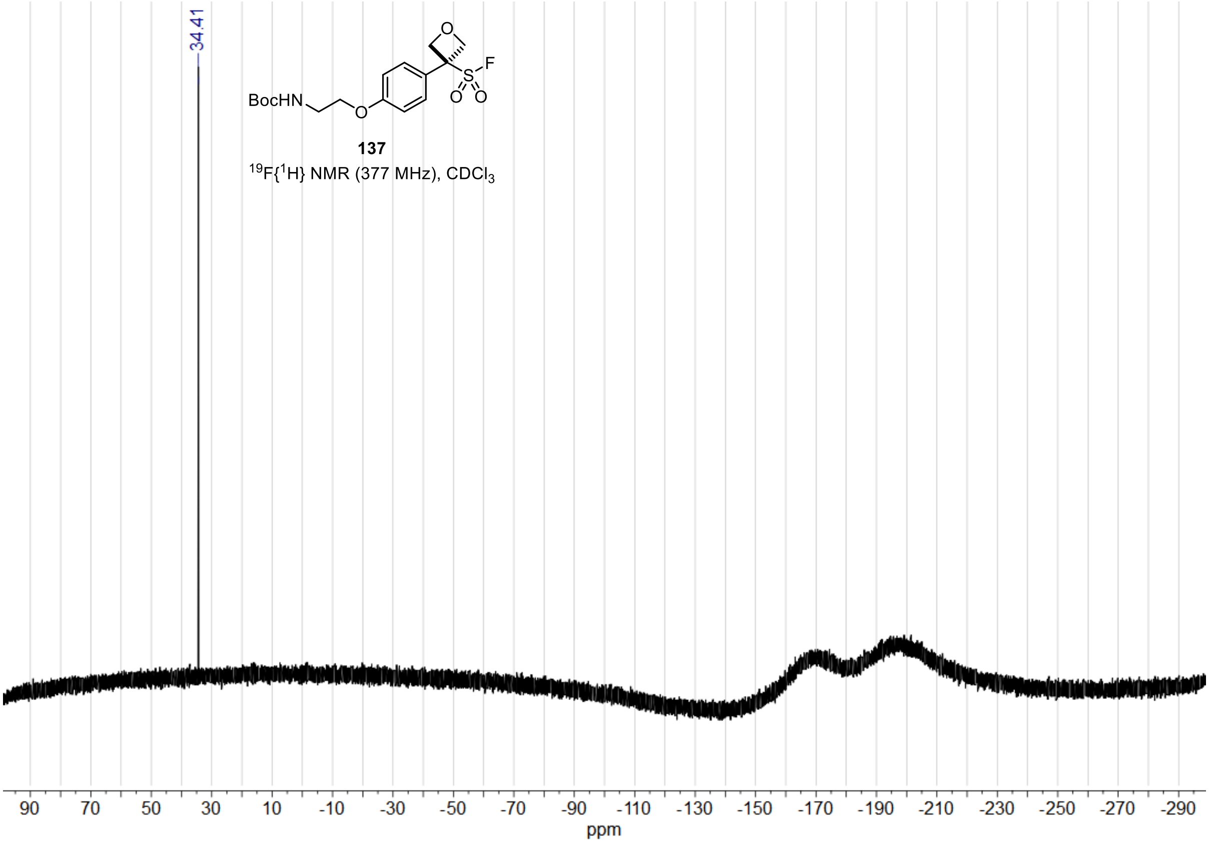
34.41
BocHN
O
O
S
F
O O
137
19F{1H} NMR (377 MHz), CDCl3
90 70 50 30 10 -10 -30 -50 -70 -90 -110 -130 -150 -170 -190 -210 -230 -250 -270 -290
ppm

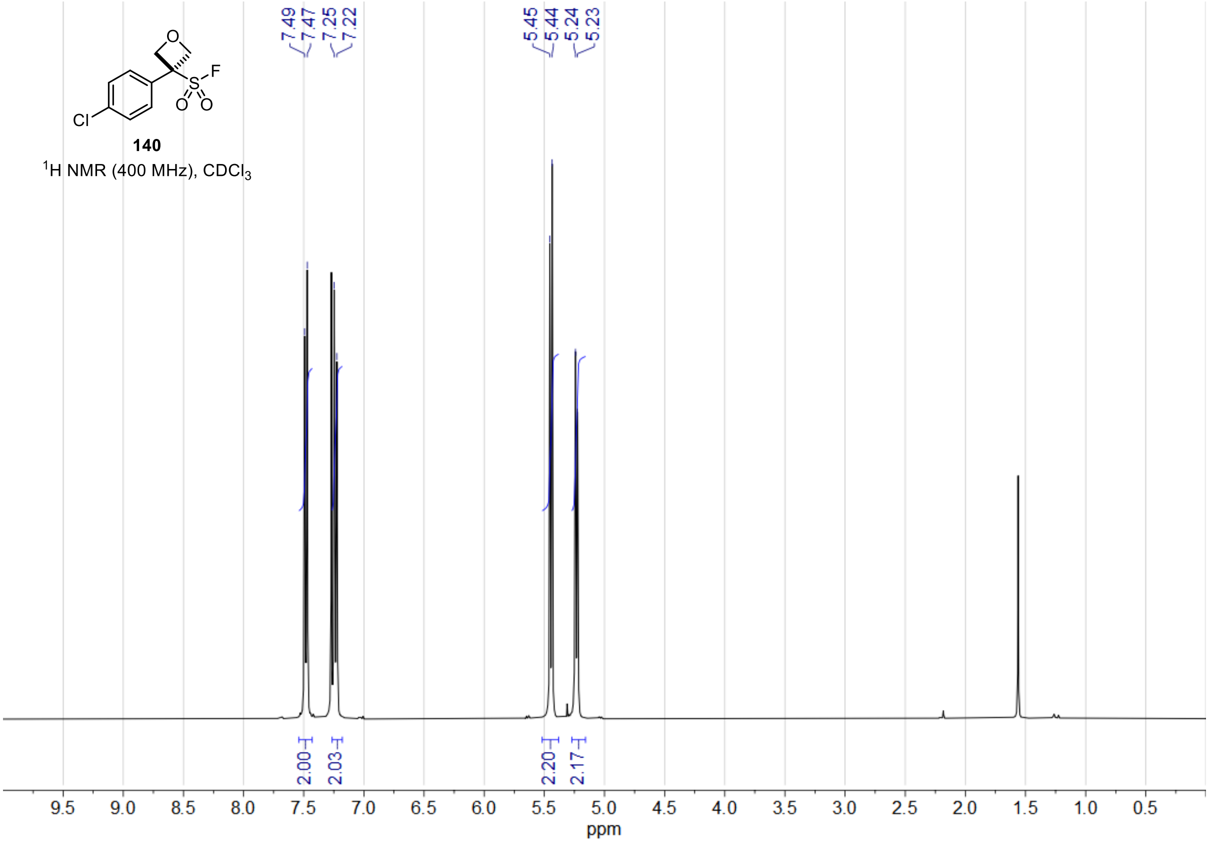

140

^{1}H NMR (400 MHz), $CDCl_3$

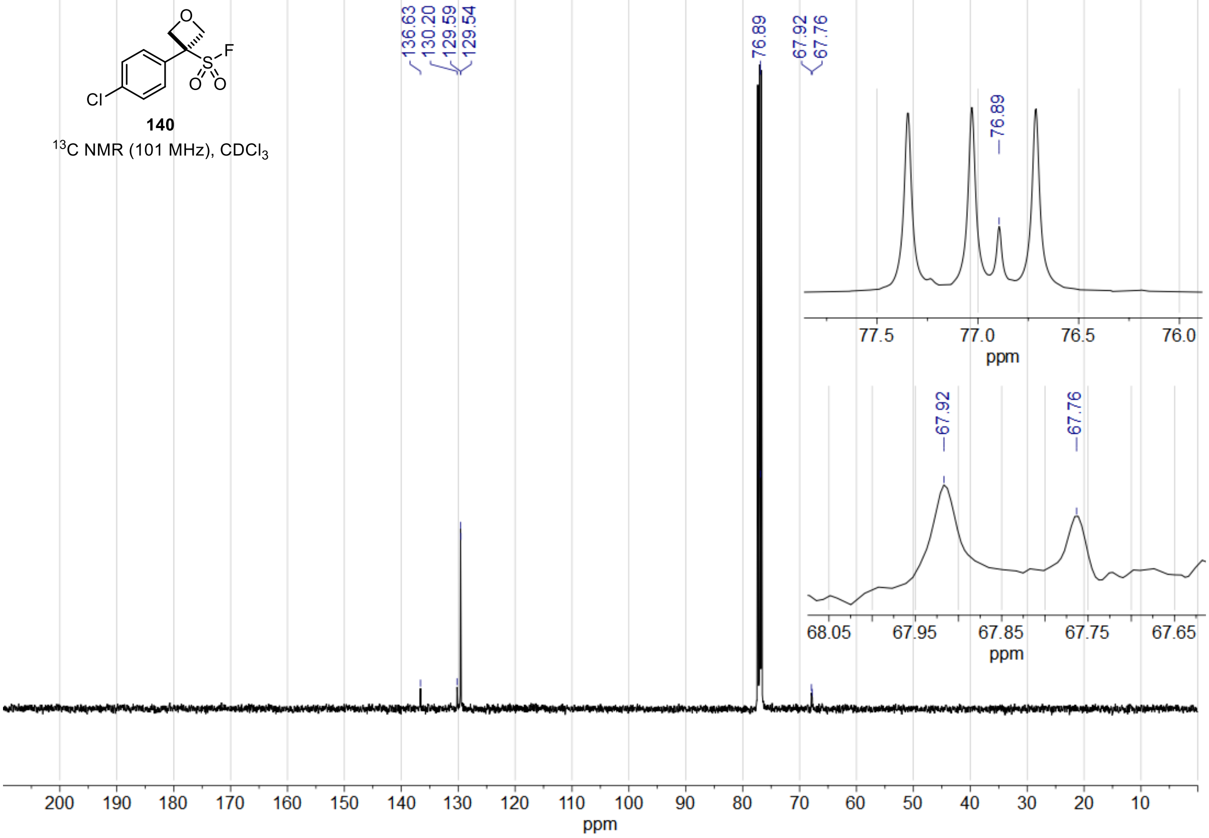

140

^{13}C NMR (101 MHz), $CDCl_3$

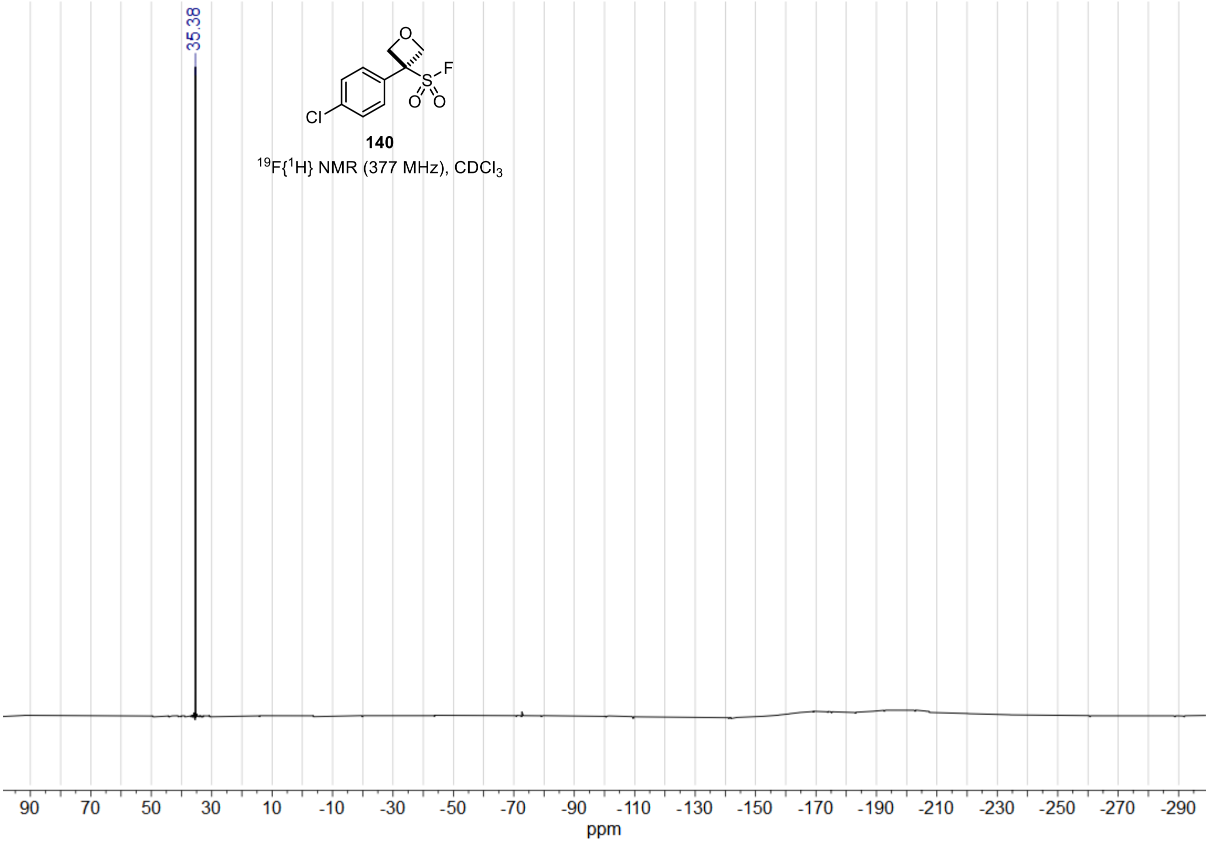

140

$^{19}F\{^{1}H\}$ NMR (377 MHz), $CDCl_3$

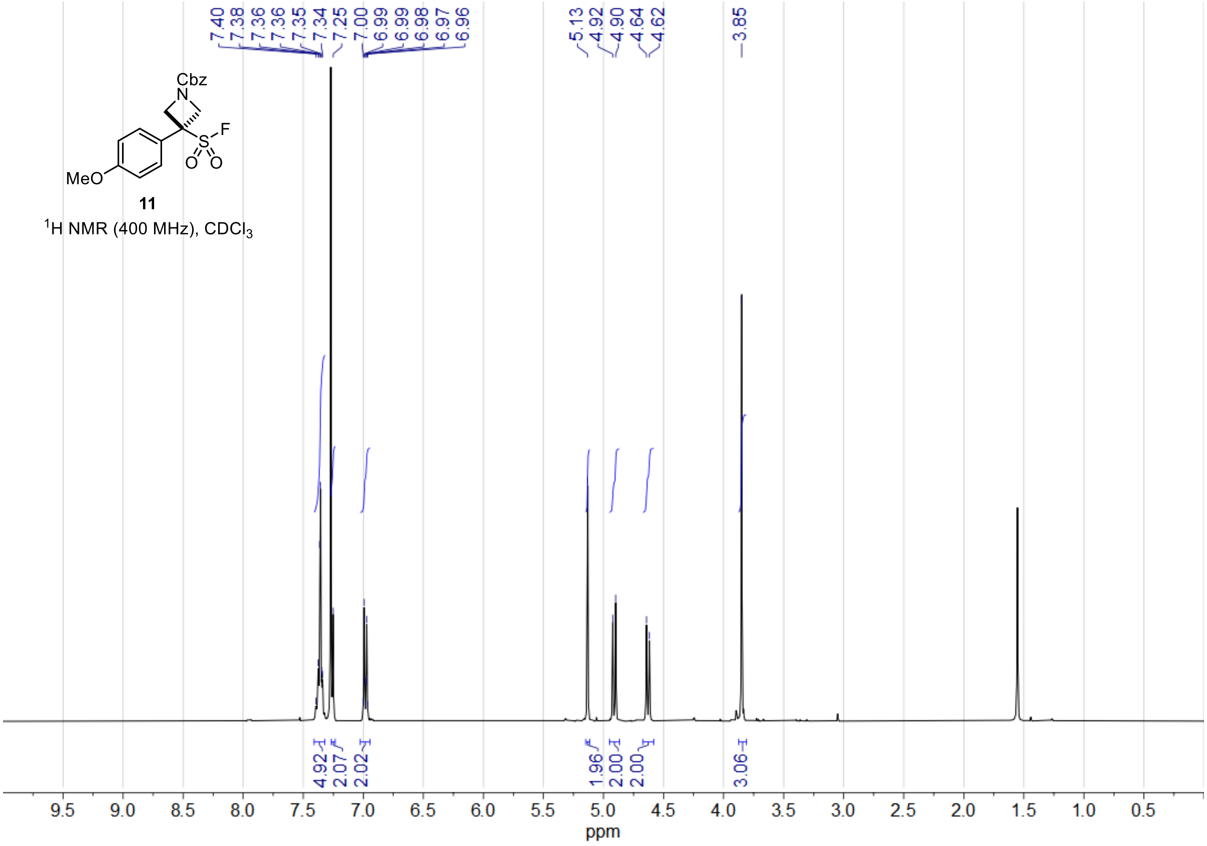
Cbz
N
S
F
O
O
MeO
11
1H NMR (400 MHz), CDCl3
7.40
7.38
7.36
7.36
7.35
7.34
7.25
7.00
6.99
6.99
6.98
6.97
6.96
5.13
4.92
4.90
4.64
4.62
3.85
4.92
2.07
2.02
1.96
2.00
2.00
3.06
9.5
9.0
8.5
8.0
7.5
7.0
6.5
6.0
5.5
5.0
4.5
4.0
3.5
3.0
2.5
2.0
1.5
1.0
0.5
ppm

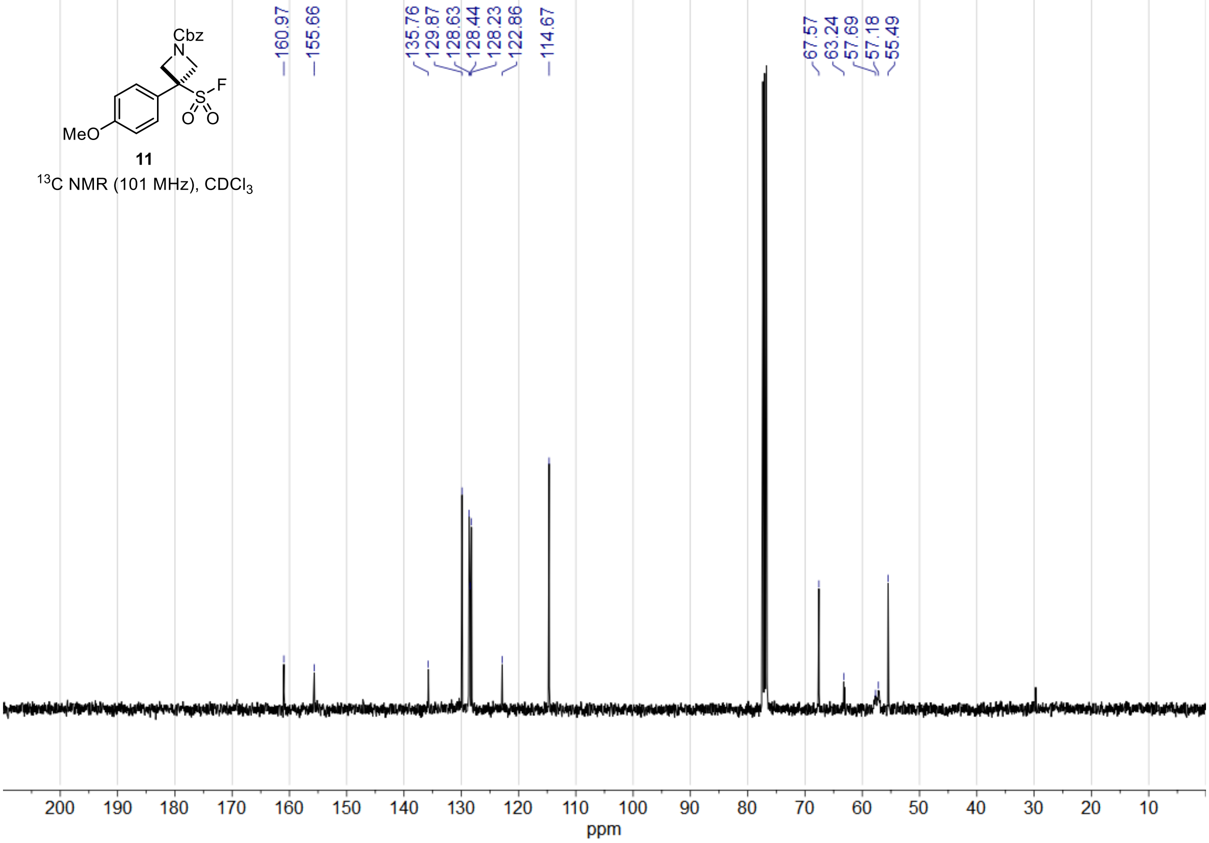
Cbz
N
S
F
O
O
MeO
11
13C NMR (101 MHz), CDCl3
160.97
155.66
135.76
129.87
128.63
128.44
128.23
122.86
114.67
67.57
63.24
57.69
57.18
55.49
200
190
180
170
160
150
140
130
120
110
100
90
80
70
60
50
40
30
20
10
ppm

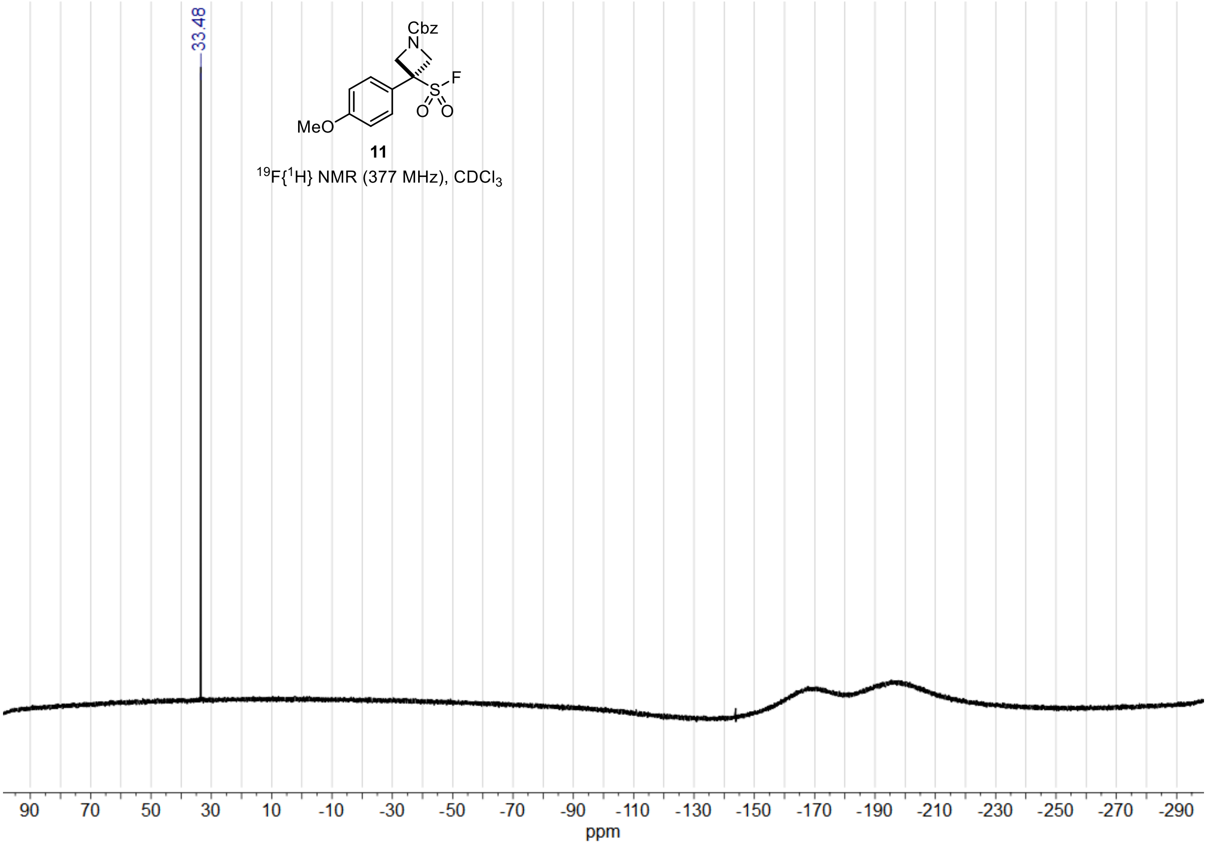
33.48
Cbz
N
F
S
O O
MeO
11
19F{1H} NMR (377 MHz), CDCl3
90 70 50 30 10 -10 -30 -50 -70 -90 -110 -130 -150 -170 -190 -210 -230 -250 -270 -290
ppm

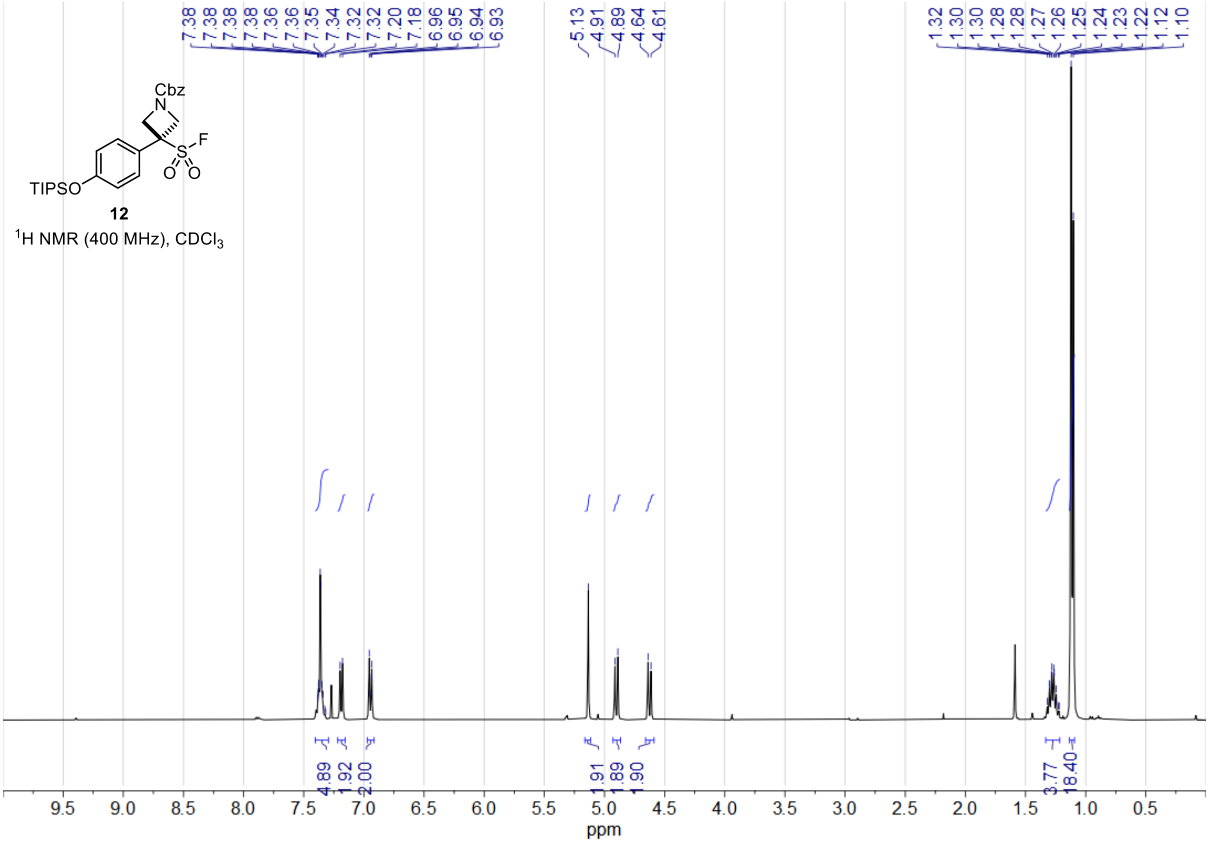
7.38
7.38
7.38
7.38
7.36
7.36
7.35
7.34
7.32
7.32
7.20
7.18
6.96
6.95
6.94
6.93
5.13
4.91
4.89
4.64
4.61
1.32
1.30
1.30
1.28
1.28
1.27
1.26
1.25
1.24
1.23
1.22
1.12
1.10
Cbz
N
F
S
O
O
TIPSO
12
^{1}H NMR (400 MHz), $CDCl_3$
4.89
1.92
2.00
1.91
1.89
1.90
3.77
18.40
9.5
9.0
8.5
8.0
7.5
7.0
6.5
6.0
5.5
5.0
4.5
4.0
3.5
3.0
2.5
2.0
1.5
1.0
0.5
ppm

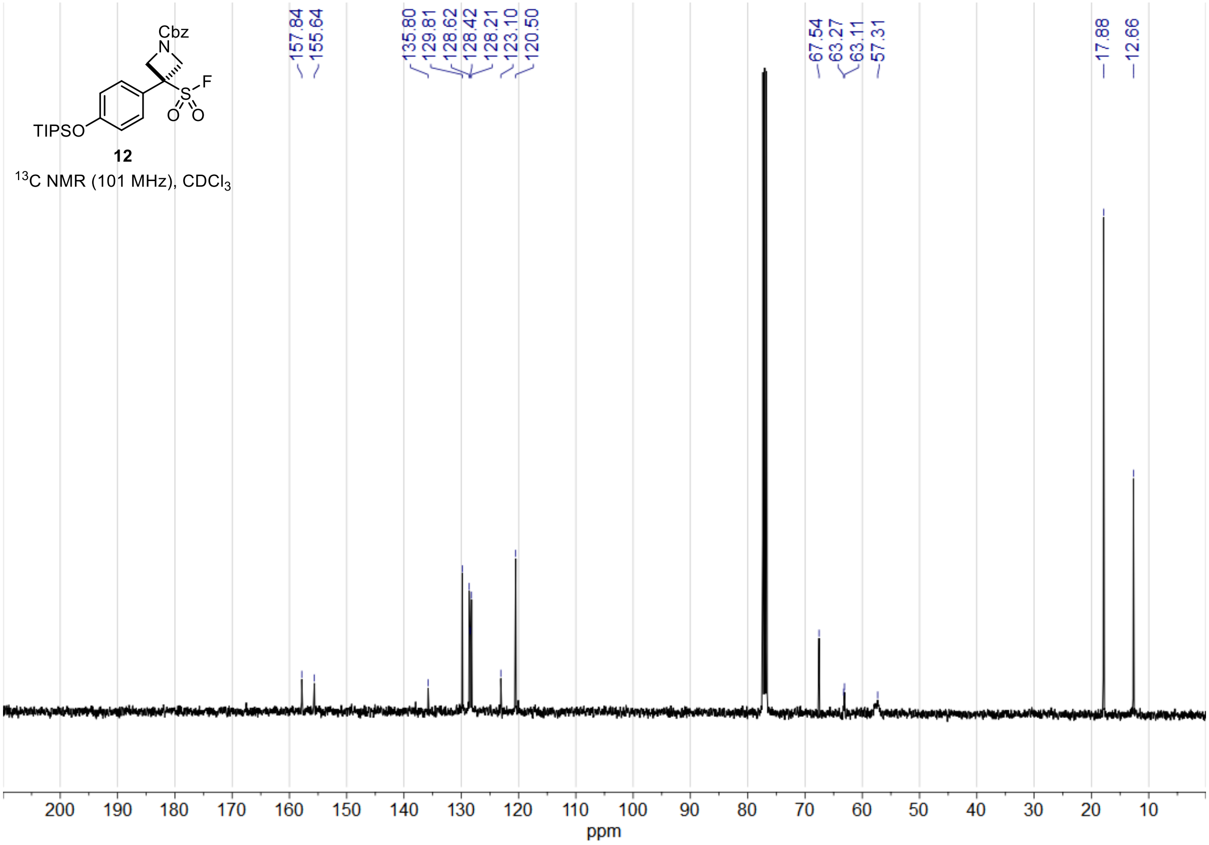
157.84
155.64
135.80
129.81
128.62
128.42
128.21
123.10
120.50
67.54
63.27
63.11
57.31
17.88
12.66
Cbz
N
F
S
O
O
TIPSO
12
^{13}C NMR (101 MHz), $CDCl_3$
200
190
180
170
160
150
140
130
120
110
100
90
80
70
60
50
40
30
20
10
ppm

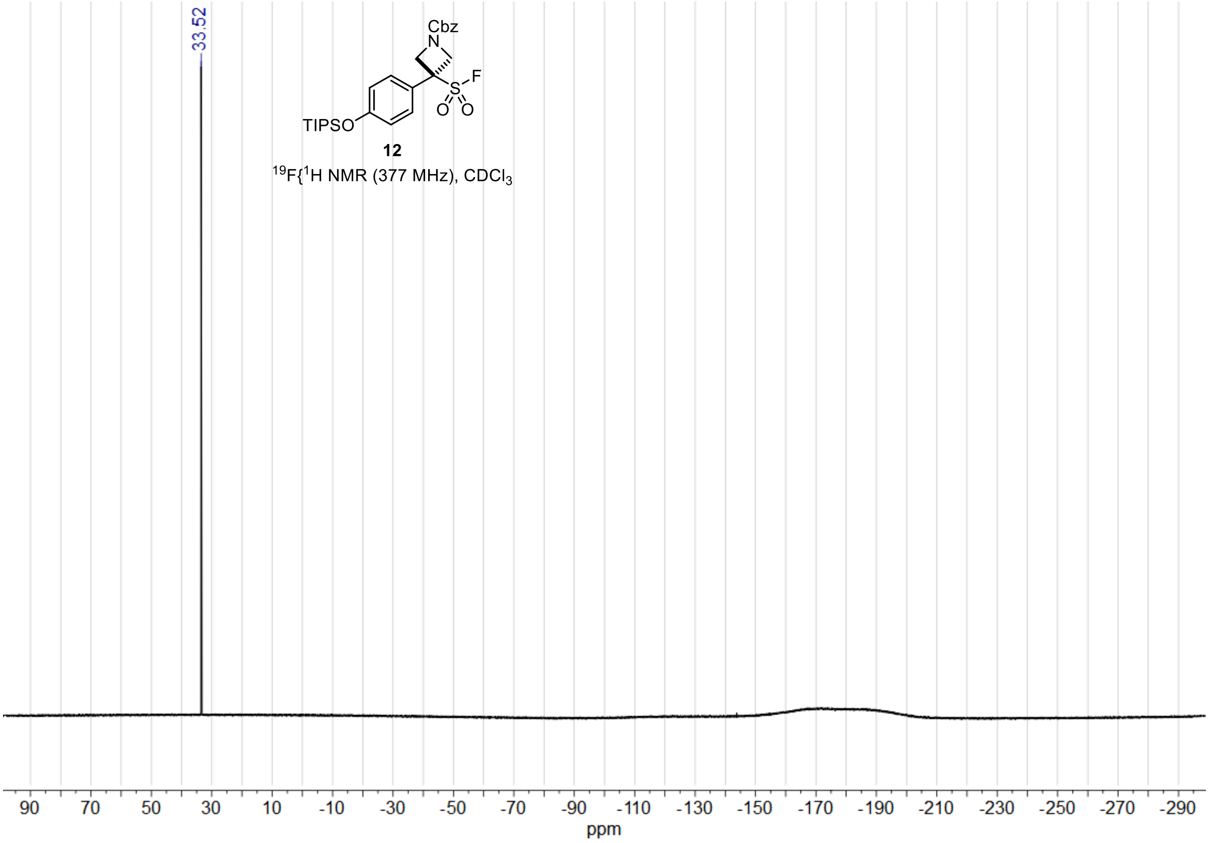
33.52
Cbz
N
F
S
O
O
TIPSO
12
19F{1H NMR (377 MHz), CDCl3
90
70
50
30
10
-10
-30
-50
-70
-90
-110
-130
-150
-170
-190
-210
-230
-250
-270
-290
ppm

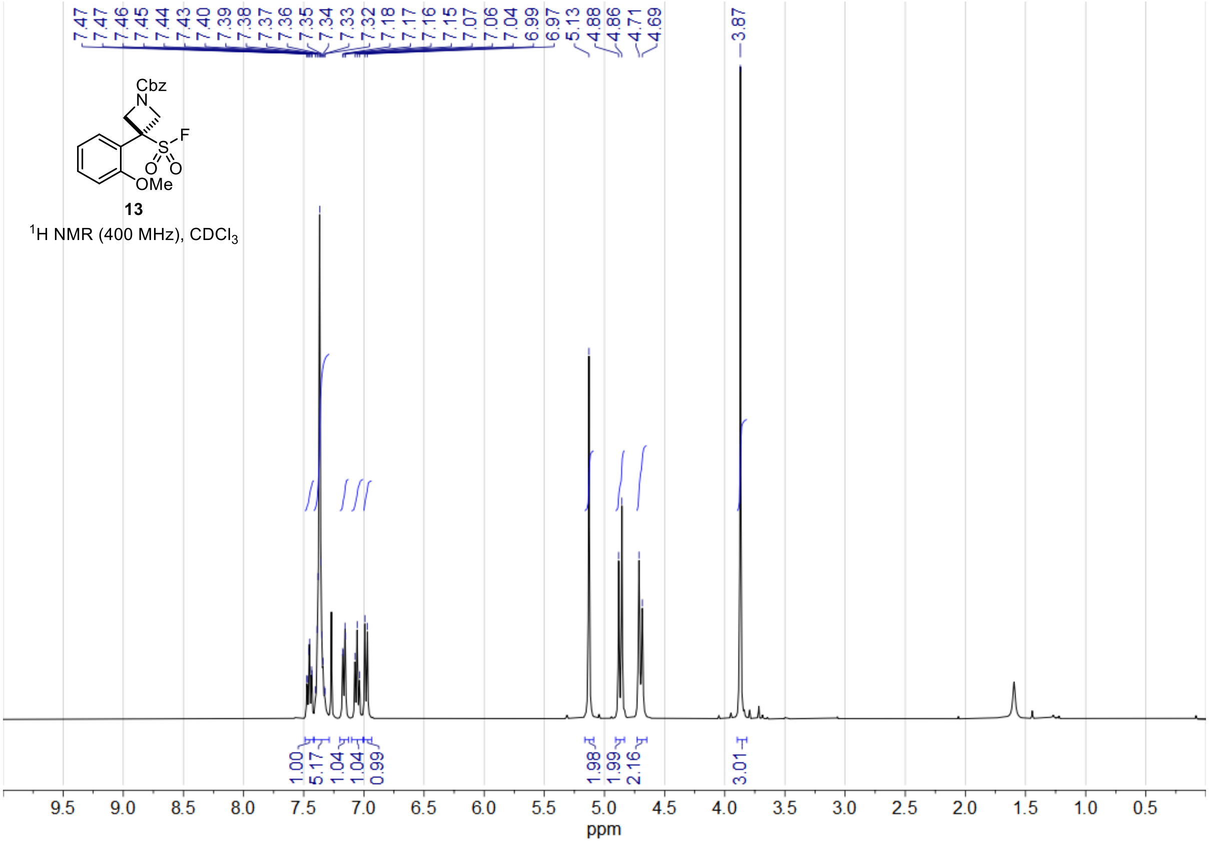
7.47
7.47
7.46
7.45
7.44
7.43
7.40
7.39
7.38
7.37
7.36
7.35
7.34
7.33
7.32
7.18
7.17
7.16
7.15
7.07
7.06
7.04
6.99
6.97
5.13
4.88
4.86
4.71
4.69
3.87
Cbz
N
S
F
O
O
OMe
13
¹H NMR (400 MHz), CDCl₃
1.00
5.17
1.04
1.04
0.99
1.98
1.99
2.16
3.01
9.5
9.0
8.5
8.0
7.5
7.0
6.5
6.0
5.5
5.0
4.5
4.0
3.5
3.0
2.5
2.0
1.5
1.0
0.5
ppm

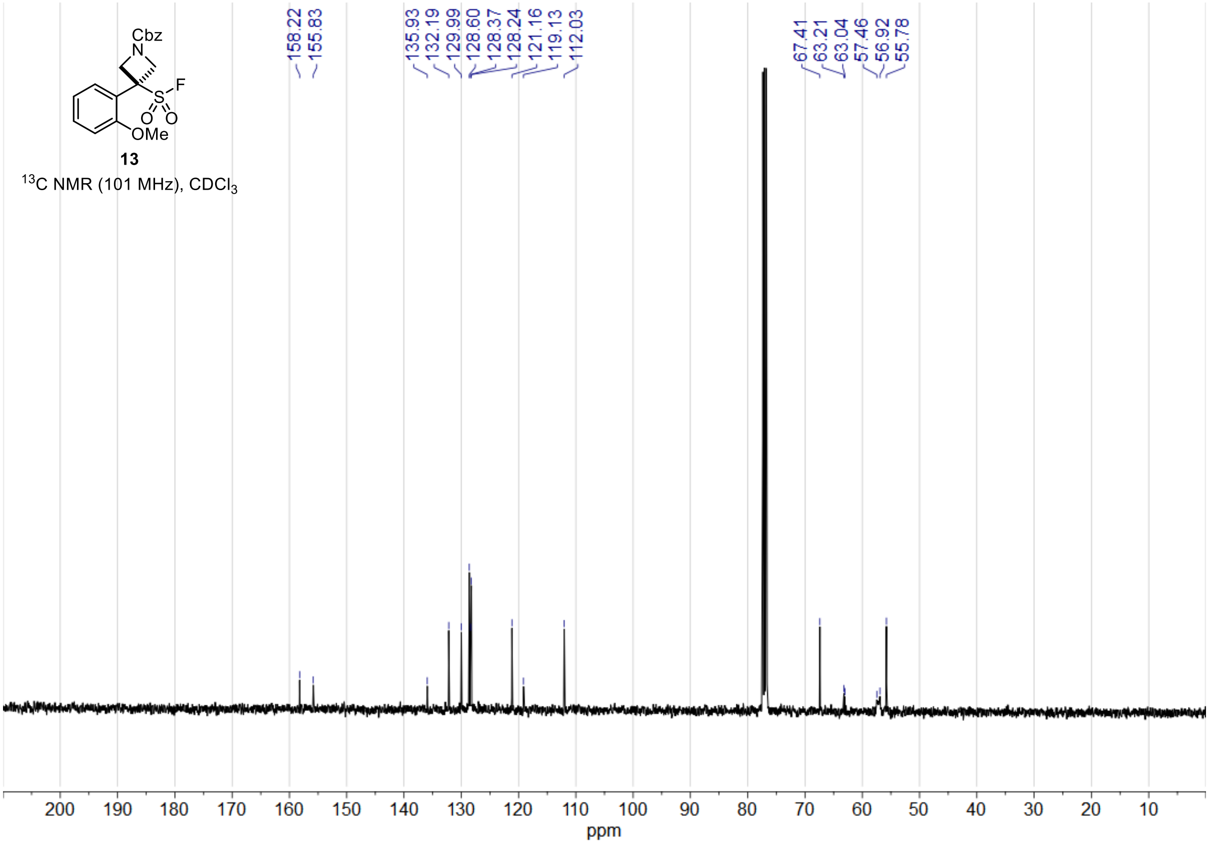
158.22
155.83
135.93
132.19
129.99
128.60
128.37
128.24
121.16
119.13
112.03
67.41
63.21
63.04
57.46
56.92
55.78
Cbz
N
S
F
O
O
OMe
13
¹³C NMR (101 MHz), CDCl₃
200
190
180
170
160
150
140
130
120
110
100
90
80
70
60
50
40
30
20
10
ppm

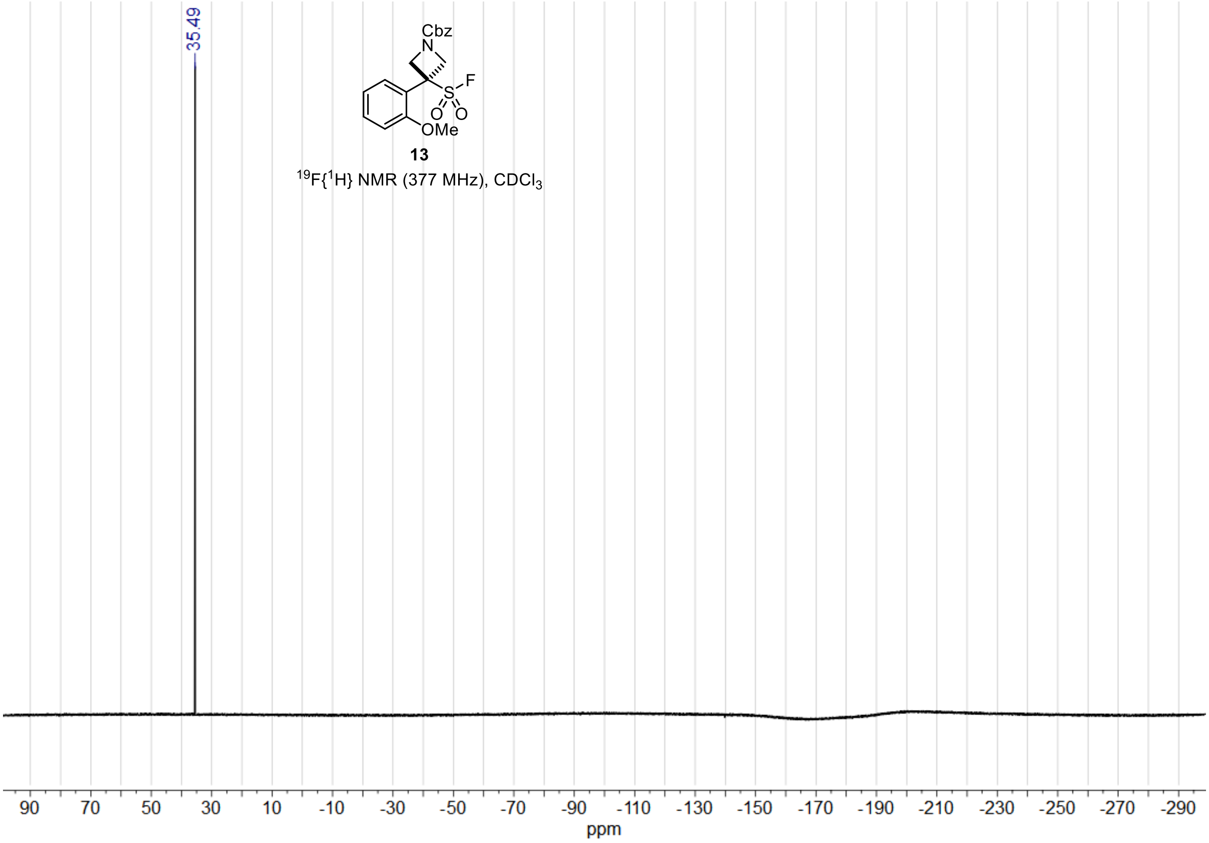

13

$^{19}F\{^{1}H\}$ NMR (377 MHz), $CDCl_3$

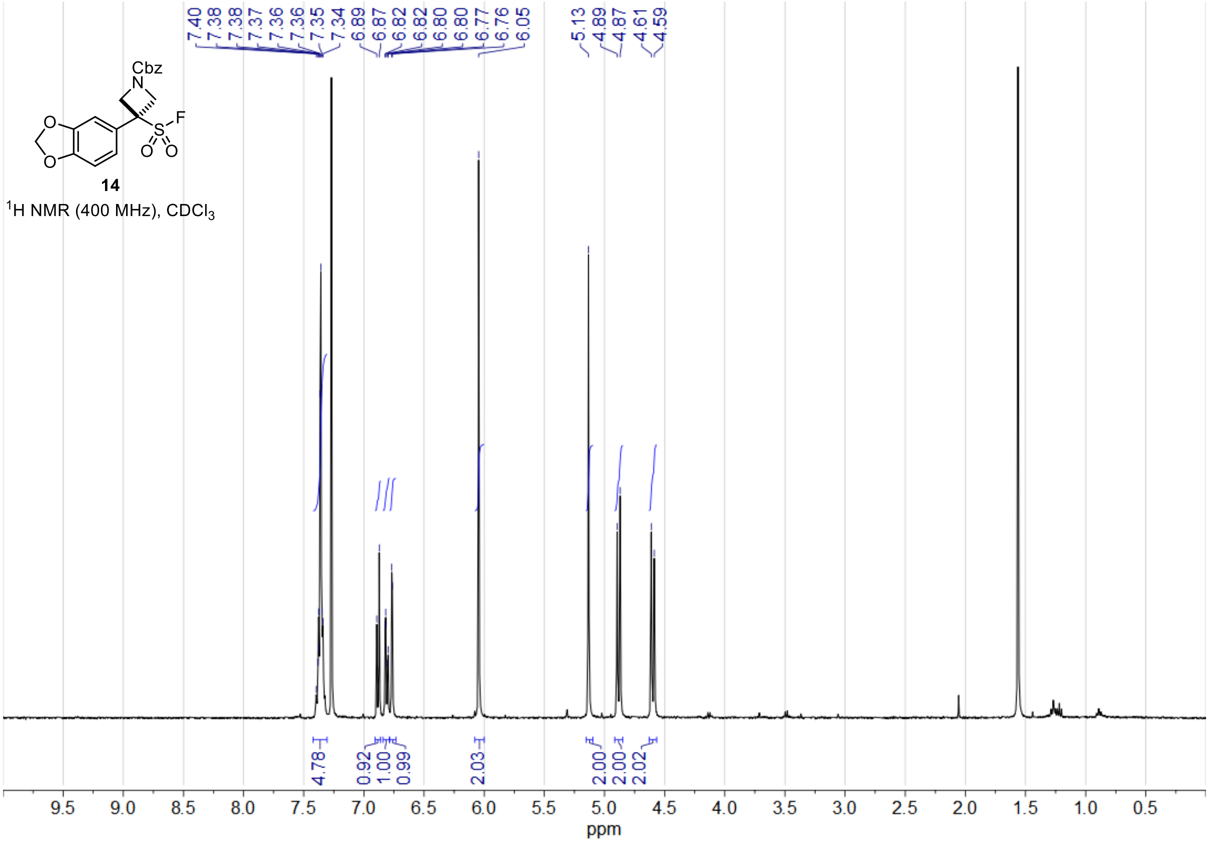
7.40 7.38 7.38 7.37 7.36 7.36 7.35 7.34 6.89 6.87 6.82 6.82 6.80 6.77 6.76 6.05
5.13 4.89 4.87 4.61 4.59
Cbz
N
S
F
O
O
O
O
14
^{1}H NMR (400 MHz), CDCl$_3$
4.78 0.92 1.00 0.99 2.03 2.00 2.00 2.02
9.5 9.0 8.5 8.0 7.5 7.0 6.5 6.0 5.5 5.0 4.5 4.0 3.5 3.0 2.5 2.0 1.5 1.0 0.5
ppm

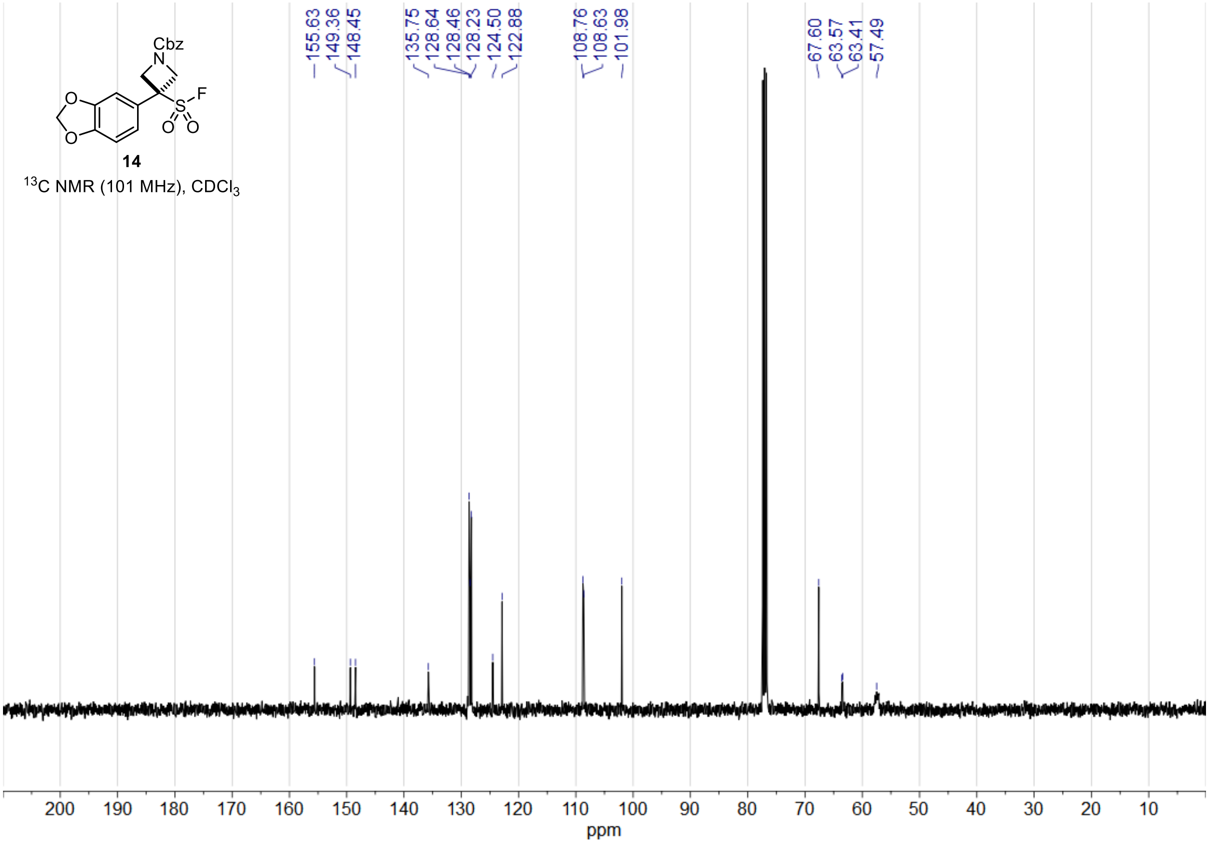
155.63 149.36 148.45 135.75 128.64 128.46 128.23 124.50 122.88 108.76 108.63 101.98
67.60 63.57 63.41 57.49
Cbz
N
S
F
O
O
O
O
14
^{13}C NMR (101 MHz), CDCl$_3$
200 190 180 170 160 150 140 130 120 110 100 90 80 70 60 50 40 30 20 10
ppm

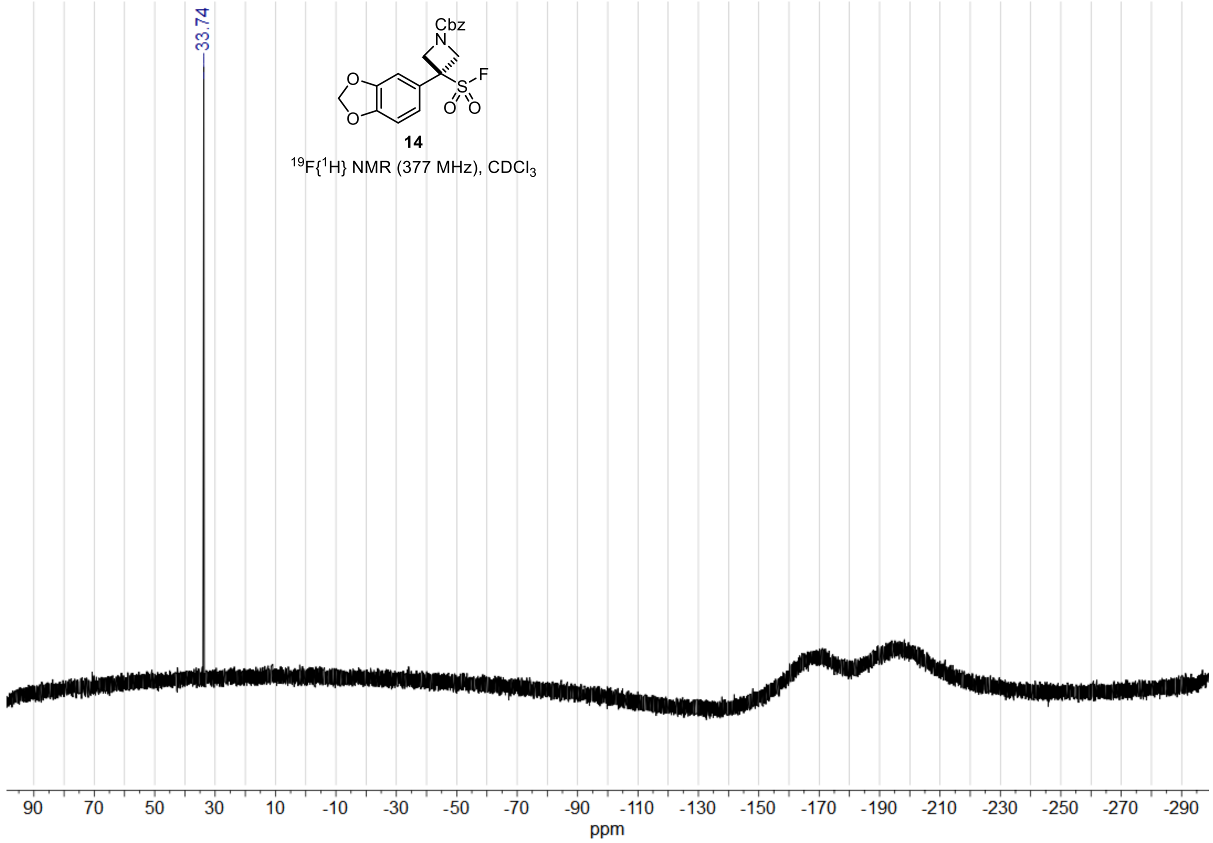
33.74
Cbz
N
F
S
O
O
O
O
14
19F{1H} NMR (377 MHz), CDCl3
90
70
50
30
10
-10
-30
-50
-70
-90
-110
-130
-150
-170
-190
-210
-230
-250
-270
-290
ppm

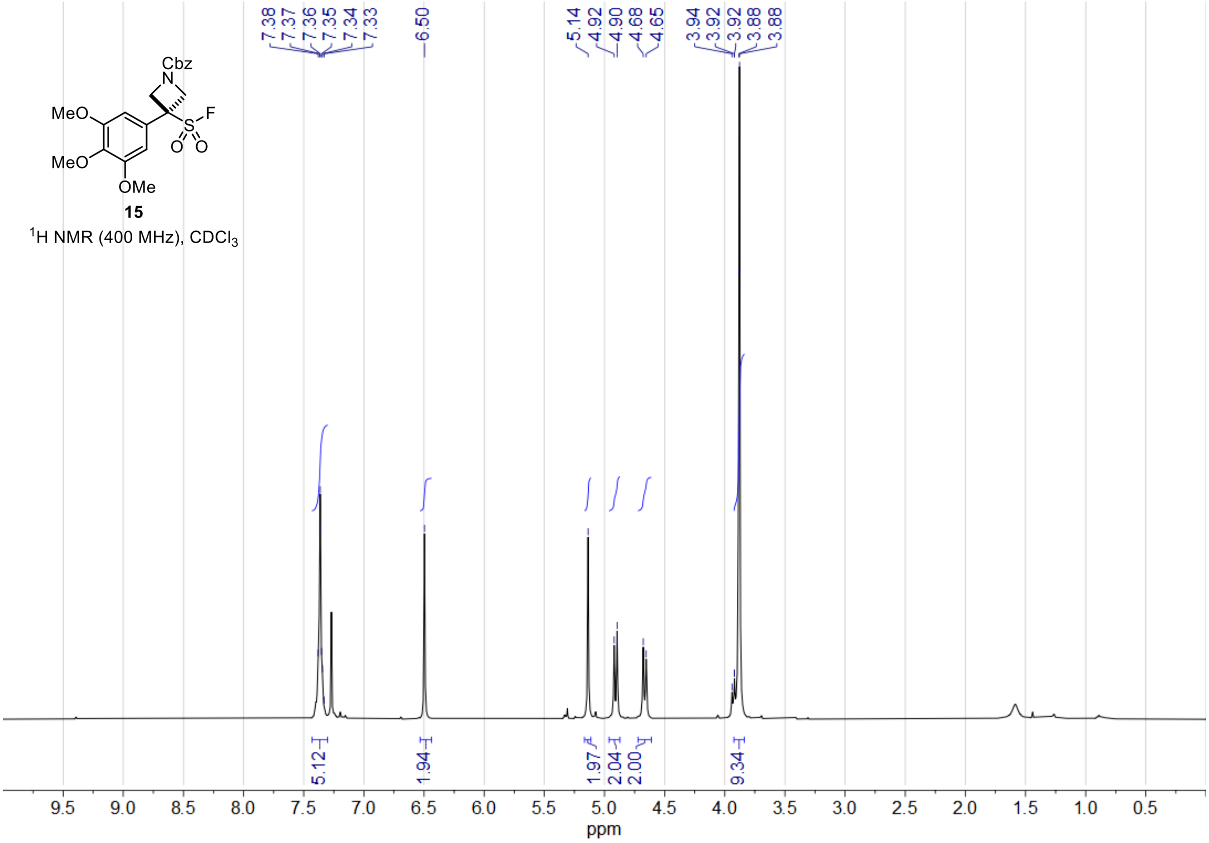
7.38
7.37
7.36
7.35
7.34
7.33
6.50
5.14
4.92
4.90
4.68
4.65
3.94
3.92
3.92
3.88
3.88
Cbz
N
MeO
MeO
OMe
S
F
O
O
15
1H NMR (400 MHz), CDCl3
5.12
1.94
1.97
2.04
2.00
9.34
9.5
9.0
8.5
8.0
7.5
7.0
6.5
6.0
5.5
5.0
4.5
4.0
3.5
3.0
2.5
2.0
1.5
1.0
0.5
ppm

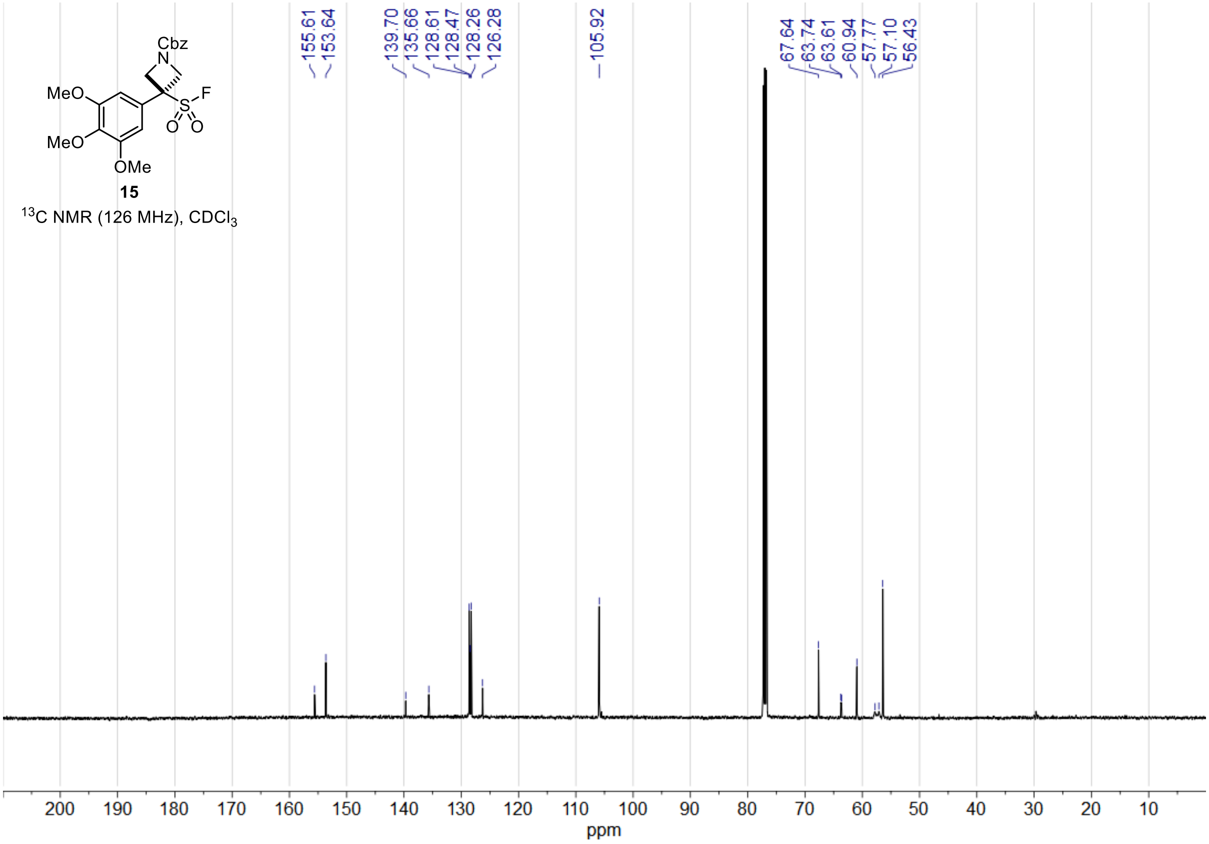
155.61
153.64
139.70
135.66
128.61
128.47
128.26
126.28
105.92
67.64
63.74
63.61
60.94
57.77
57.10
56.43
Cbz
N
MeO
MeO
OMe
S
F
O
O
15
13C NMR (126 MHz), CDCl3
200
190
180
170
160
150
140
130
120
110
100
90
80
70
60
50
40
30
20
10
ppm

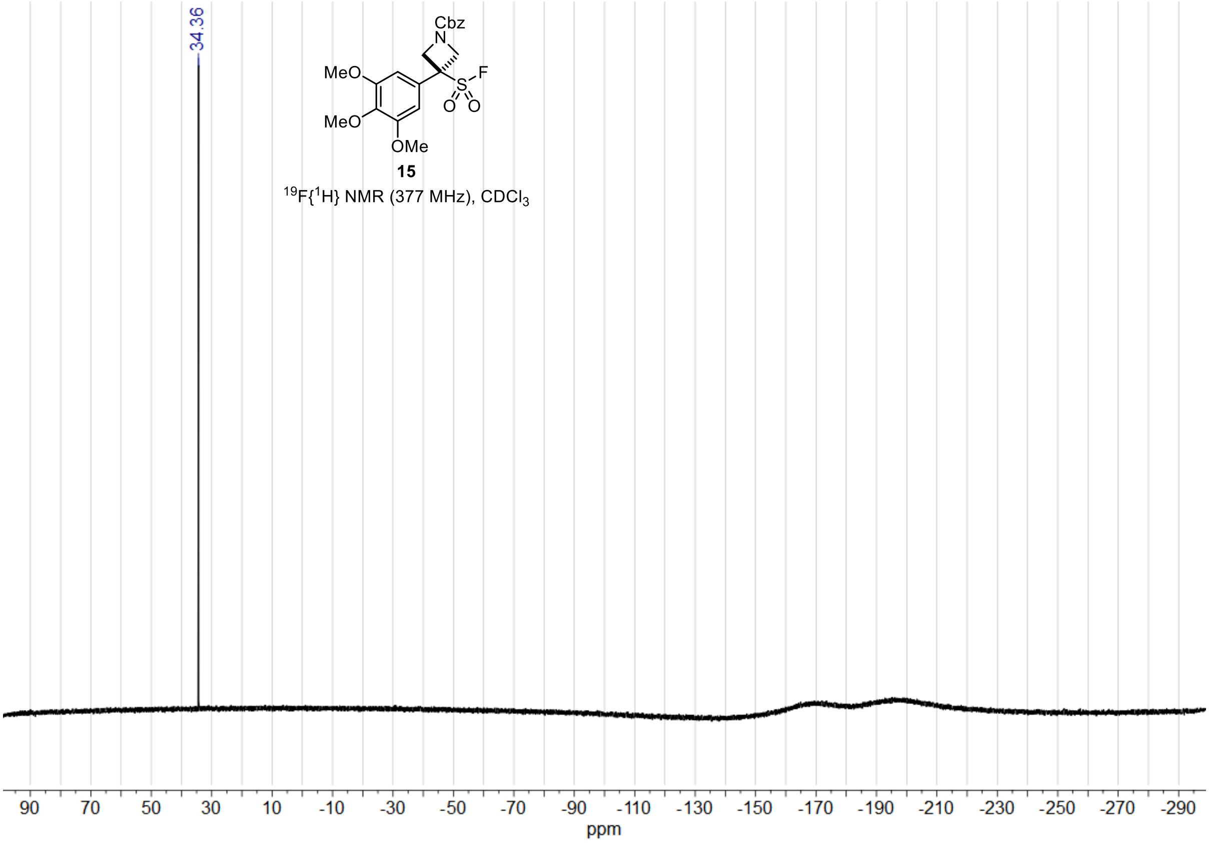

15

$^{19}F\{^{1}H\}$ NMR (377 MHz), $CDCl_3$

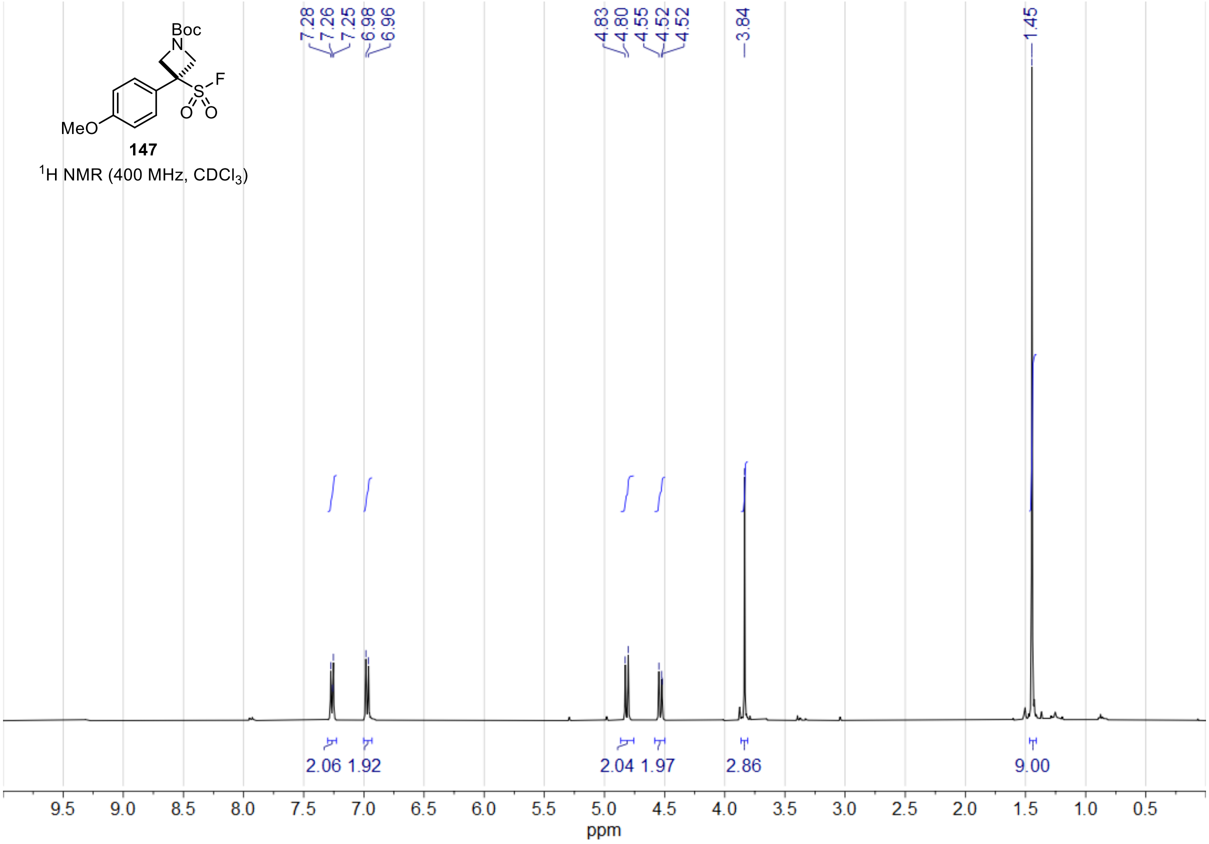
Boc
N
S
F
O
O
MeO
147
1H NMR (400 MHz, CDCl3)
7.28
7.26
7.25
6.98
6.96
4.83
4.80
4.55
4.52
4.52
3.84
1.45
2.06
1.92
2.04
1.97
2.86
9.00
ppm

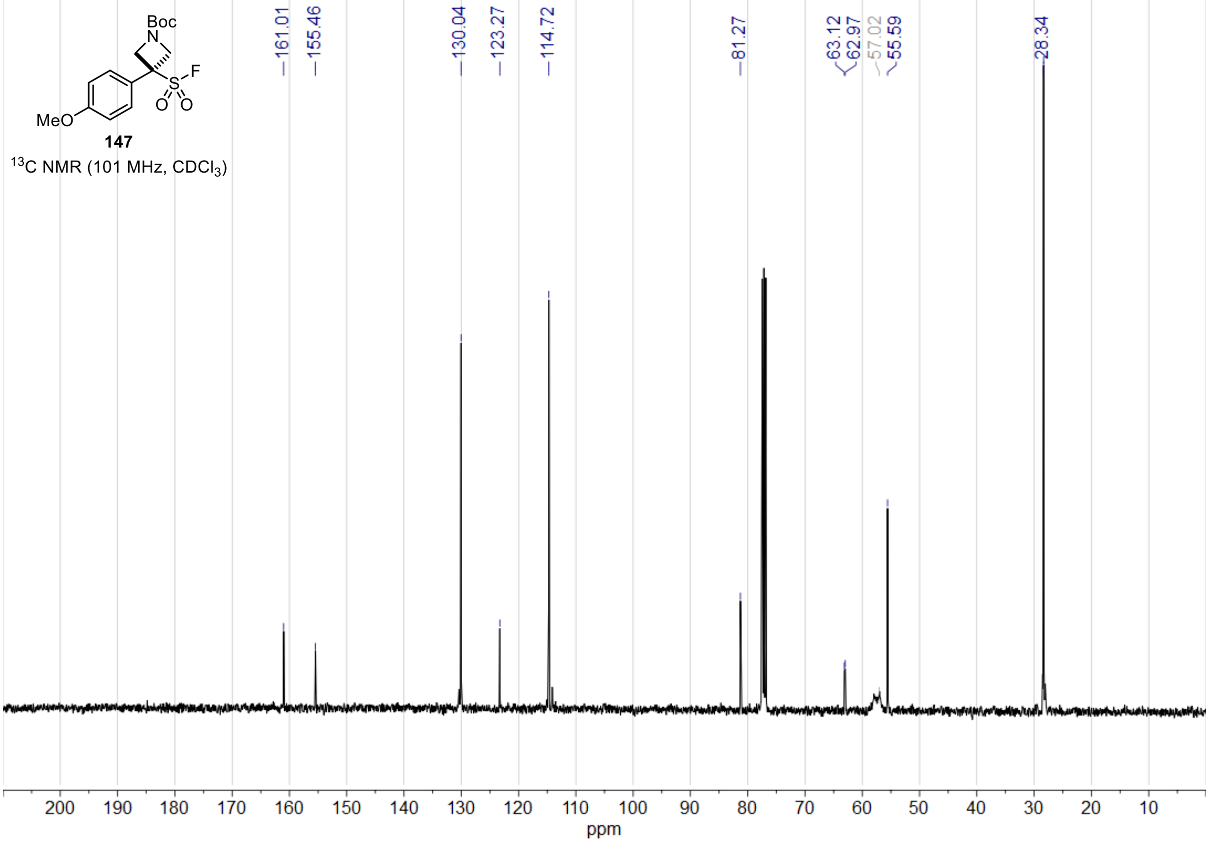
Boc
N
S
F
O
O
MeO
147
13C NMR (101 MHz, CDCl3)
161.01
155.46
130.04
123.27
114.72
81.27
63.12
62.97
57.02
55.59
28.34
ppm

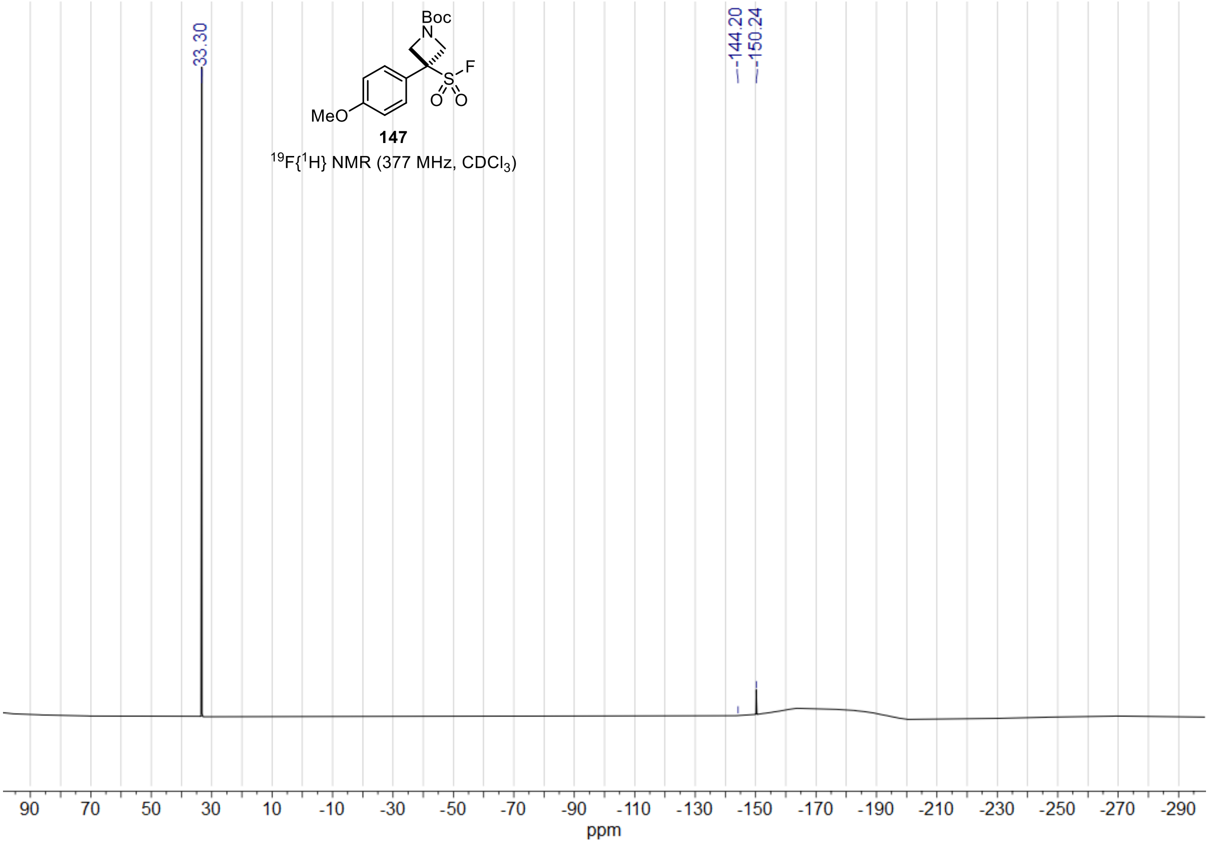
Boc
N
F
S
O O
MeO
147
$^{19}F\{^1H\}$ NMR (377 MHz, CDCl$_3$)
33.30
-144.20
-150.24
90 70 50 30 10 -10 -30 -50 -70 -90 -110 -130 -150 -170 -190 -210 -230 -250 -270 -290
ppm

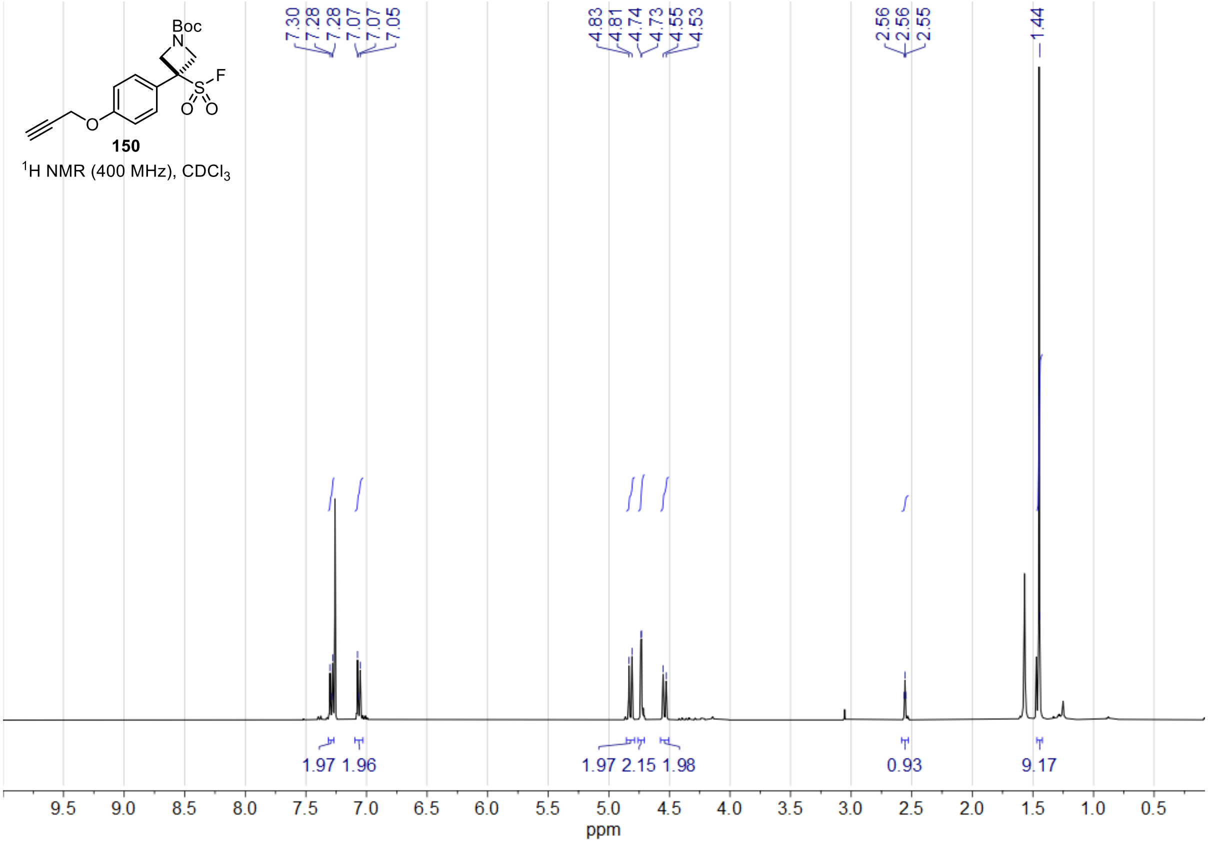
Boc
N
S
F
O
O
O
150
1H NMR (400 MHz), CDCl3
7.30
7.28
7.28
7.07
7.07
7.05
4.83
4.81
4.74
4.73
4.55
4.53
2.56
2.56
2.55
1.44
1.97 1.96
1.97 2.15 1.98
0.93
9.17
9.5 9.0 8.5 8.0 7.5 7.0 6.5 6.0 5.5 5.0 4.5 4.0 3.5 3.0 2.5 2.0 1.5 1.0 0.5
ppm

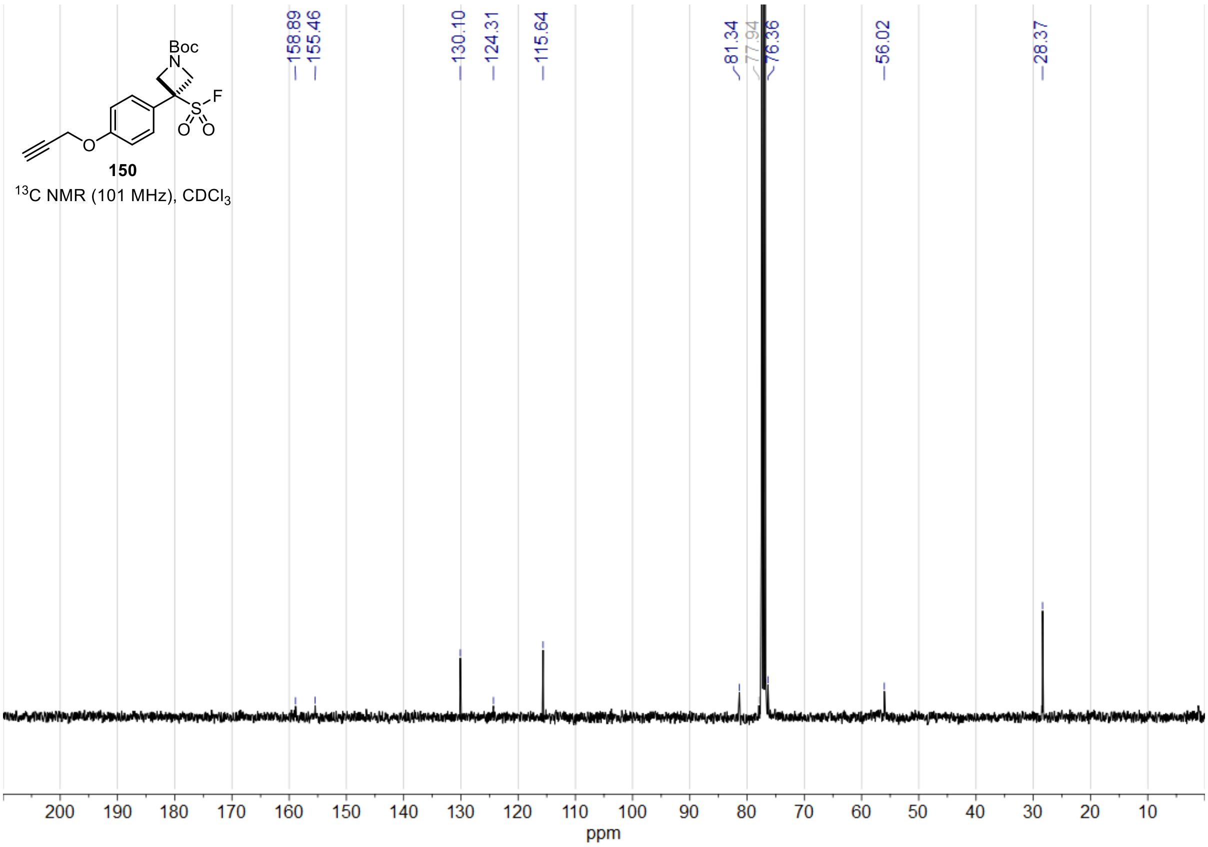
Boc
N
S
F
O
O
O
150
13C NMR (101 MHz), CDCl3
158.89
155.46
130.10
124.31
115.64
81.34
77.94
76.36
56.02
28.37
200 190 180 170 160 150 140 130 120 110 100 90 80 70 60 50 40 30 20 10
ppm

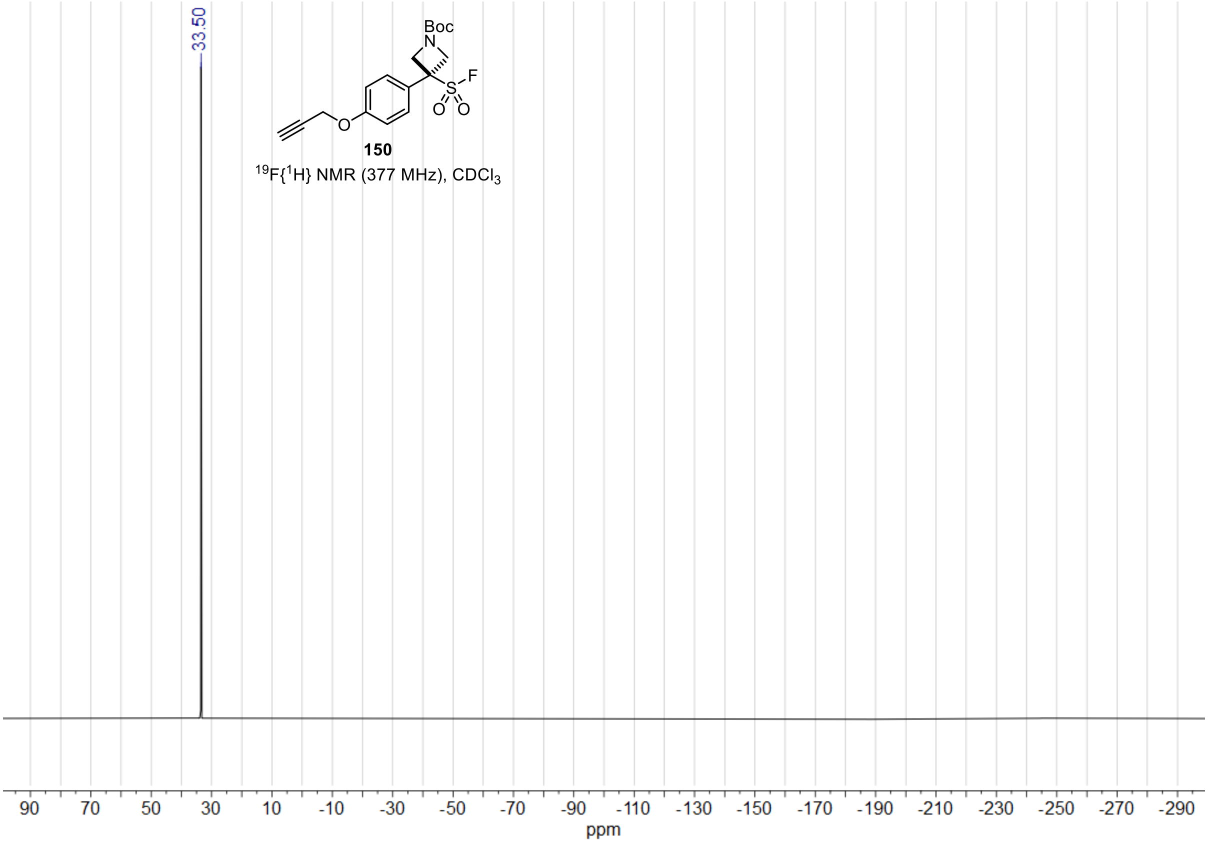
33.50
Boc
N
S
F
O
O
O
150
19F{1H} NMR (377 MHz), CDCl3
90
70
50
30
10
-10
-30
-50
-70
-90
-110
-130
-150
-170
-190
-210
-230
-250
-270
-290
ppm

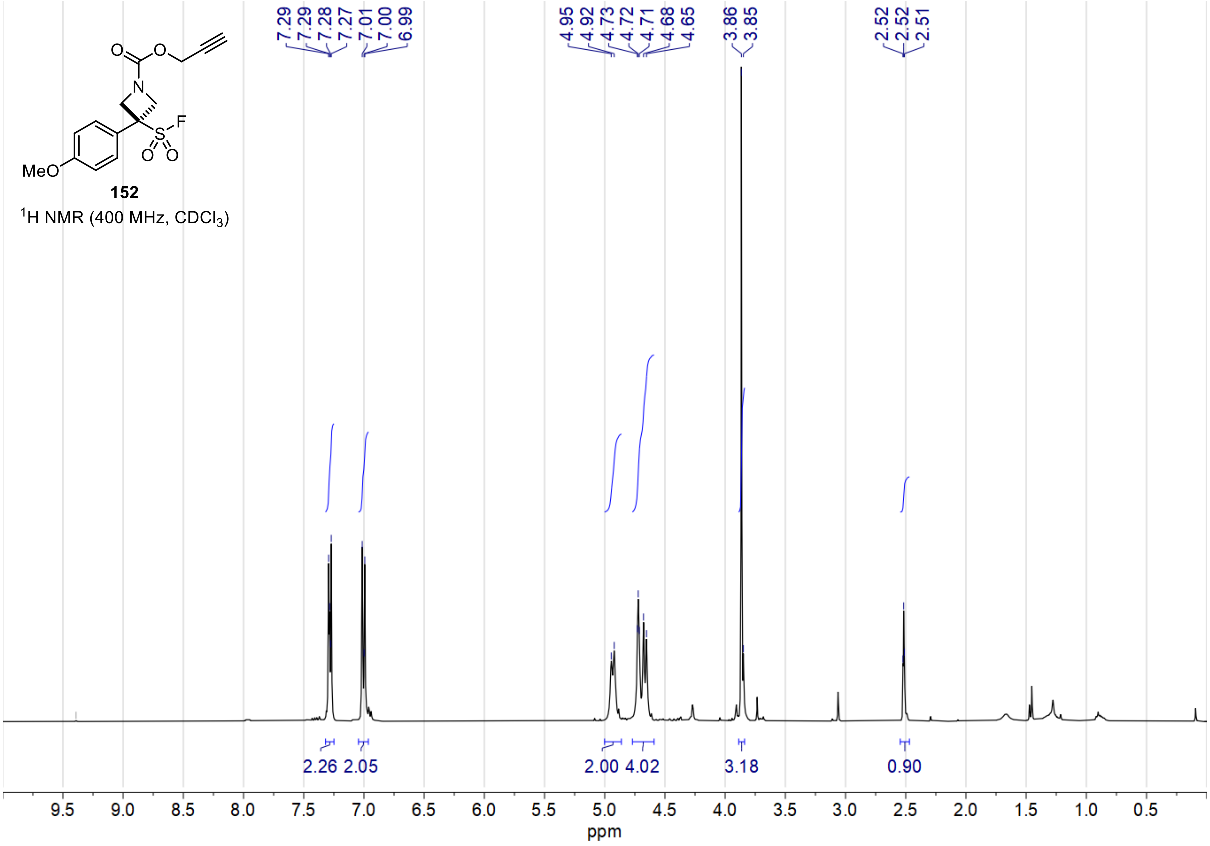

7.29 7.29 7.28 7.27 7.01 7.00 6.99
4.95 4.92 4.73 4.72 4.71 4.68 4.65
3.86 3.85
2.52 2.52 2.51
MeO
O
N
S
F
152
^{1}H NMR (400 MHz, $CDCl_3$)
2.26 2.05
2.00 4.02
3.18
0.90
9.5 9.0 8.5 8.0 7.5 7.0 6.5 6.0 5.5 5.0 4.5 4.0 3.5 3.0 2.5 2.0 1.5 1.0 0.5
ppm

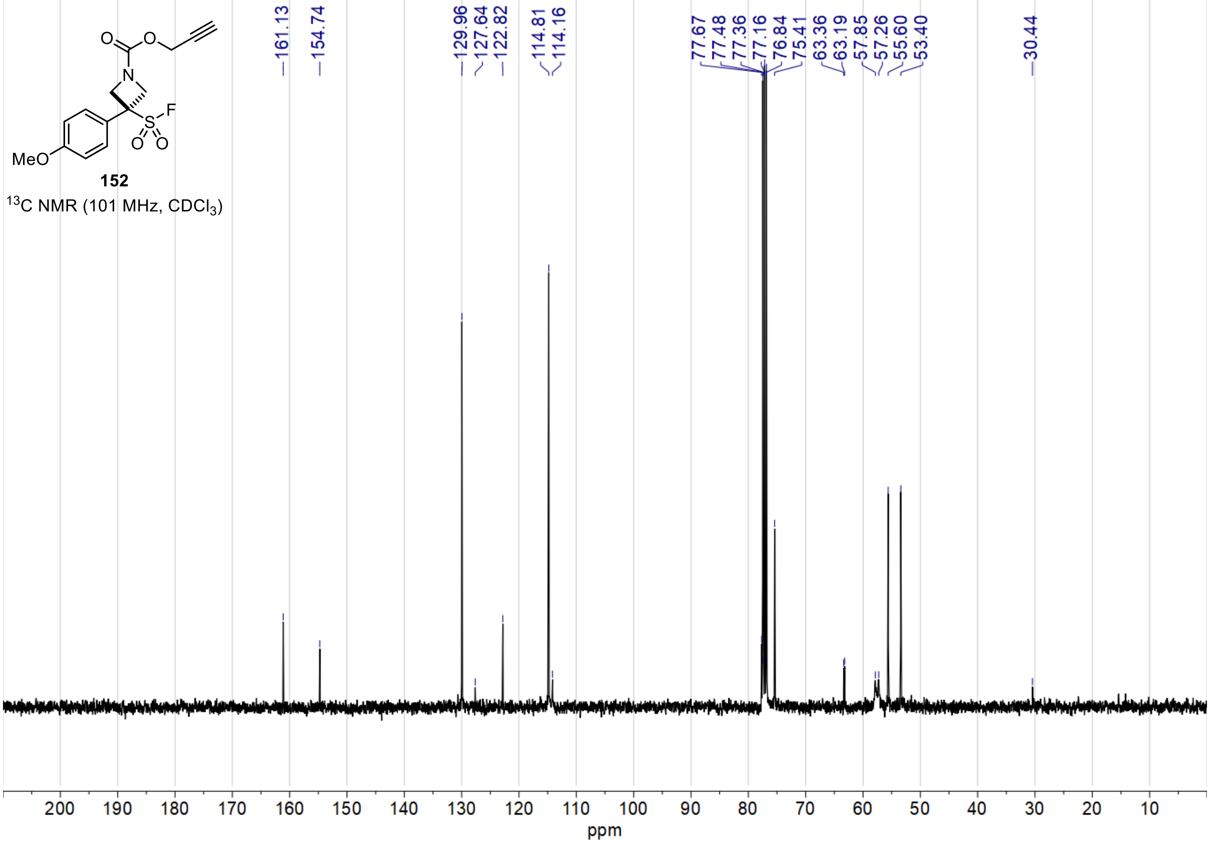

161.13
154.74
129.96 127.64 122.82
114.81 114.16
77.67 77.48 77.36 77.16 76.84 75.41 63.36 63.19 57.85 57.26 55.60 53.40
30.44
MeO
O
N
S
F
152
^{13}C NMR (101 MHz, $CDCl_3$)
200 190 180 170 160 150 140 130 120 110 100 90 80 70 60 50 40 30 20 10
ppm

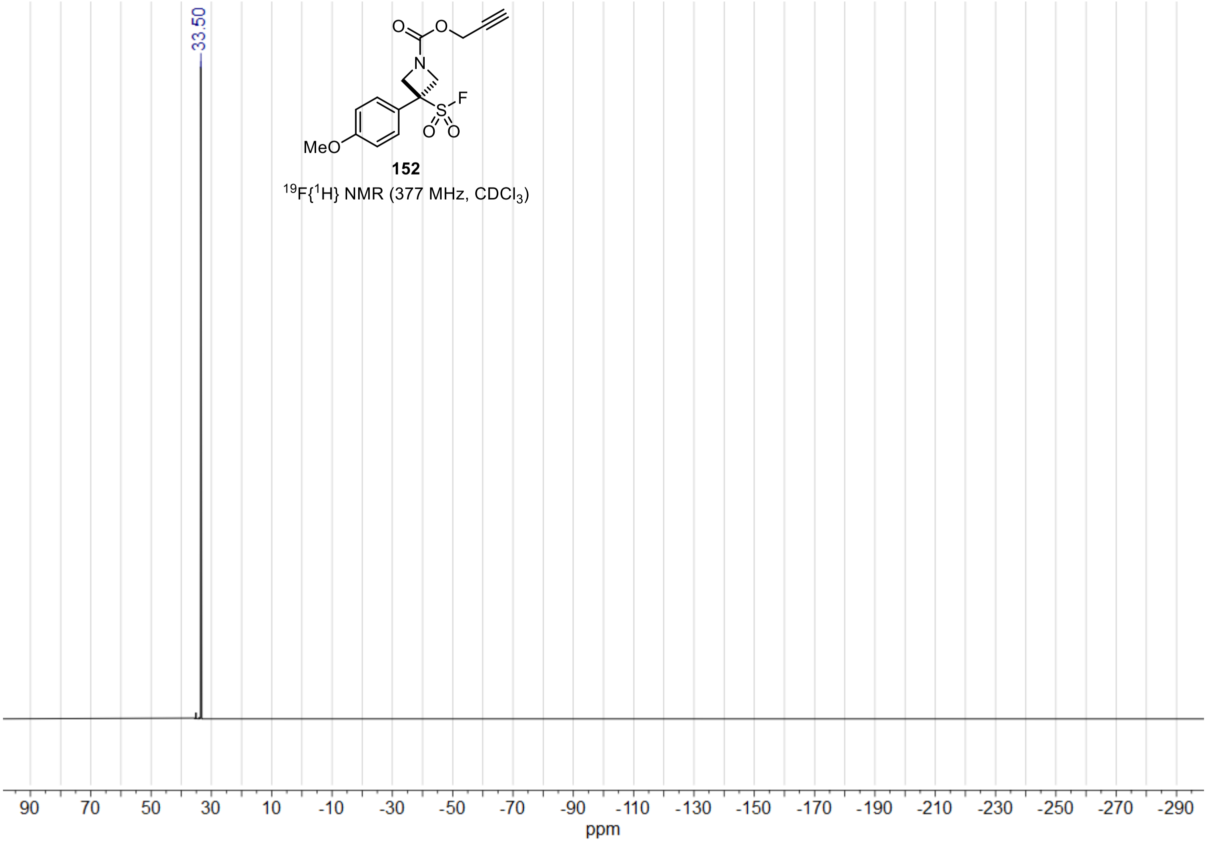

152

$^{19}F\{^{1}H\}$ NMR (377 MHz, $CDCl_3$)

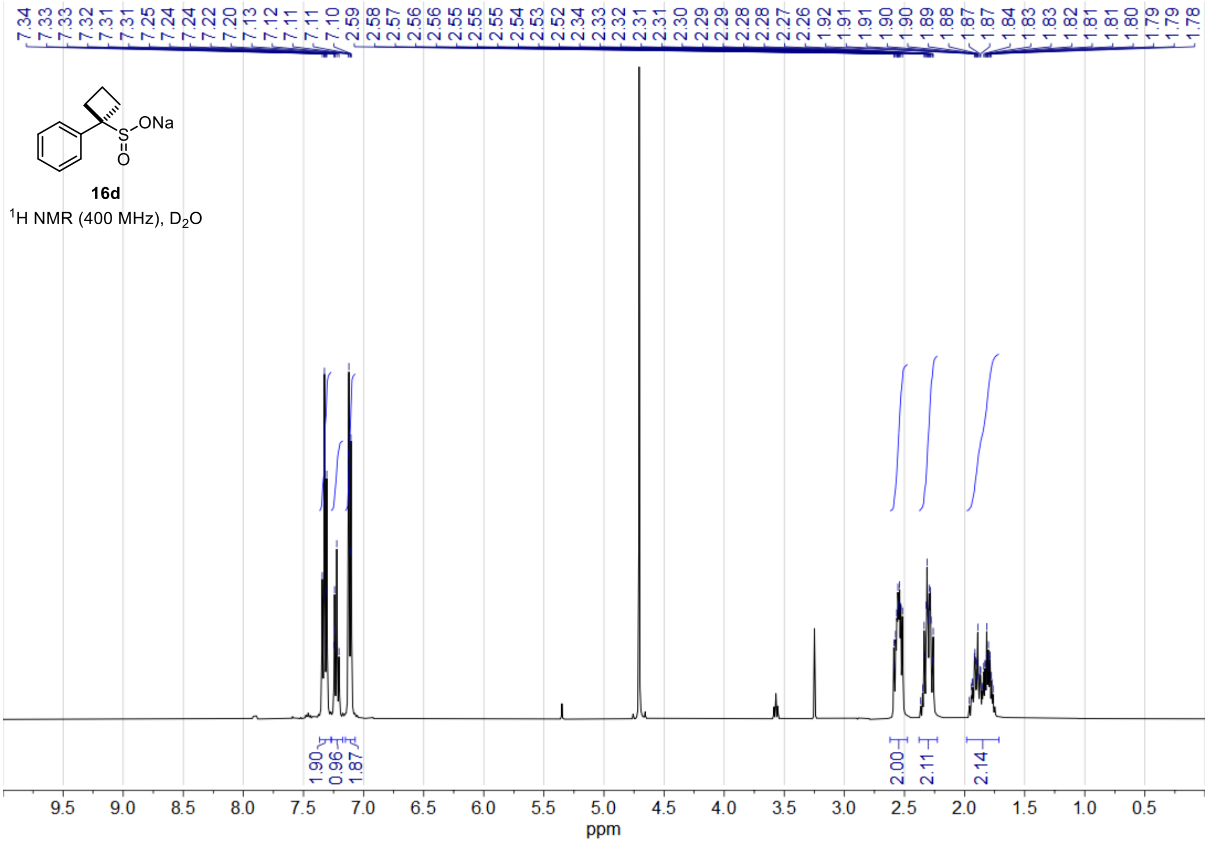
16d
^{1}H NMR (400 MHz), D_2O
7.34 7.33 7.33 7.32 7.31 7.31 7.25 7.24 7.24 7.22 7.22 7.20 7.13 7.12 7.11 7.11 7.10 2.59 2.58 2.57 2.56 2.55 2.55 2.55 2.54 2.53 2.52 2.34 2.33 2.32 2.31 2.31 2.30 2.29 2.29 2.28 2.28 2.27 2.26 1.92 1.91 1.91 1.90 1.90 1.89 1.88 1.87 1.87 1.84 1.83 1.83 1.82 1.81 1.81 1.80 1.79 1.79 1.78
1.90 0.96 1.87 2.00 2.11 2.14
ppm

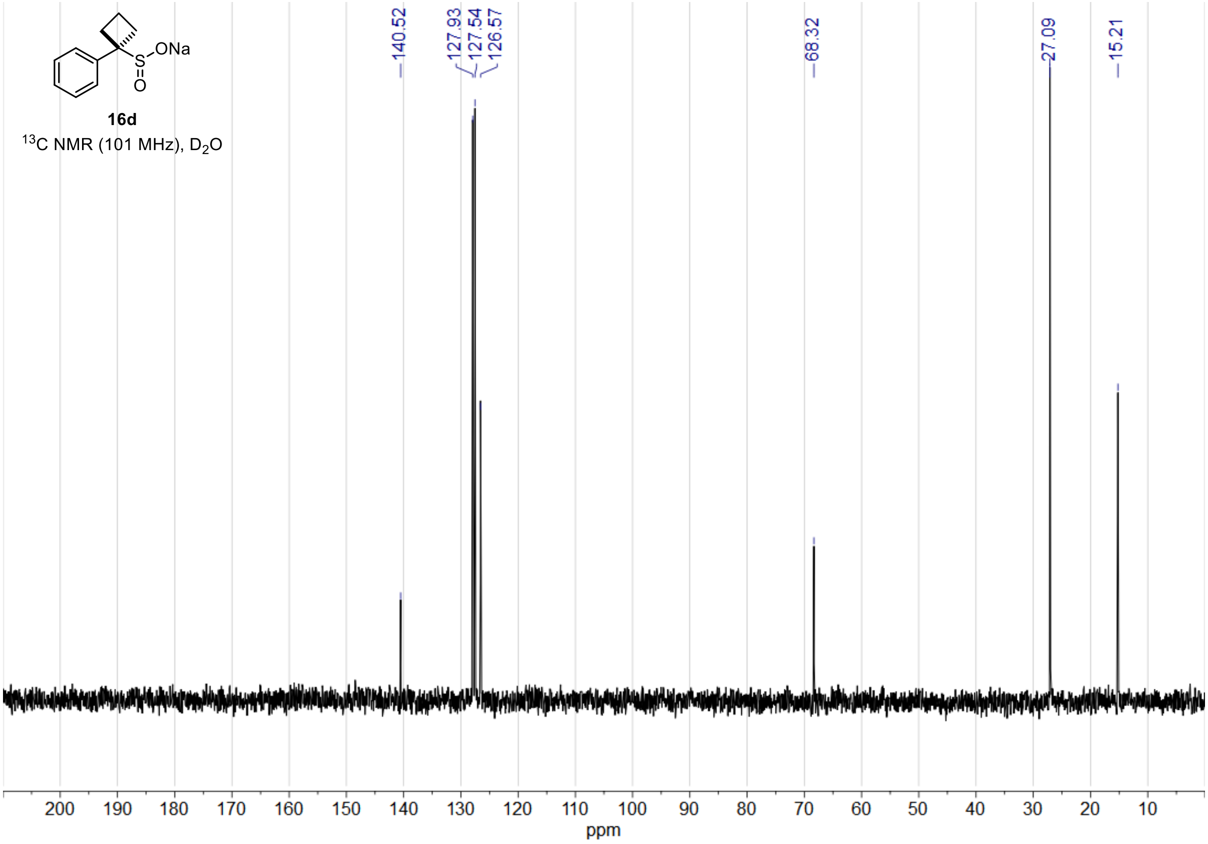
16d
^{13}C NMR (101 MHz), D_2O
140.52 127.93 127.54 126.57 68.32 27.09 15.21
ppm

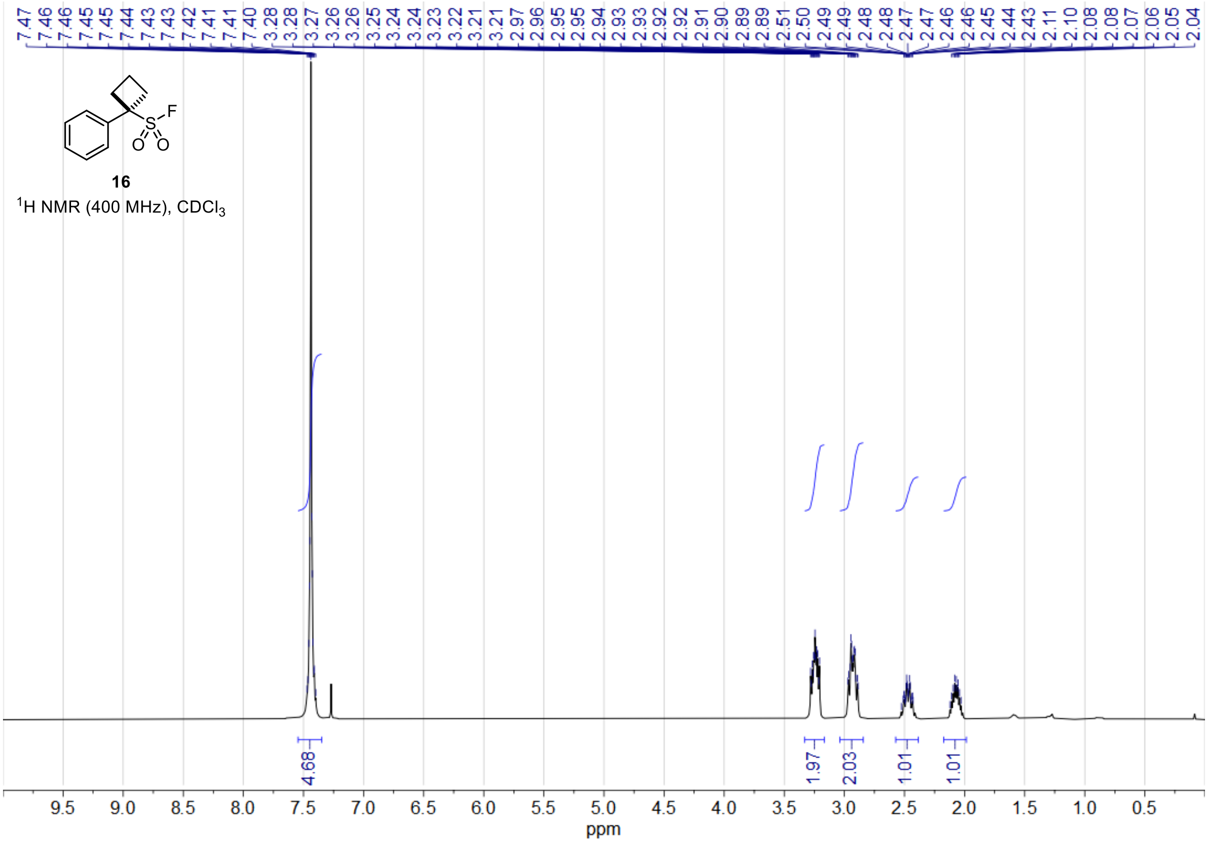
7.47
7.46
7.46
7.45
7.45
7.44
7.43
7.43
7.42
7.41
7.41
7.40
3.28
3.28
3.27
3.26
3.26
3.25
3.24
3.24
3.23
3.22
3.21
3.21
2.97
2.96
2.95
2.95
2.94
2.93
2.93
2.92
2.92
2.91
2.90
2.89
2.89
2.51
2.50
2.49
2.49
2.48
2.48
2.47
2.47
2.46
2.46
2.45
2.44
2.43
2.11
2.10
2.08
2.08
2.07
2.06
2.05
2.04
16
^{1}H NMR (400 MHz), CDCl$_3$
4.68
1.97
2.03
1.01
1.01
ppm

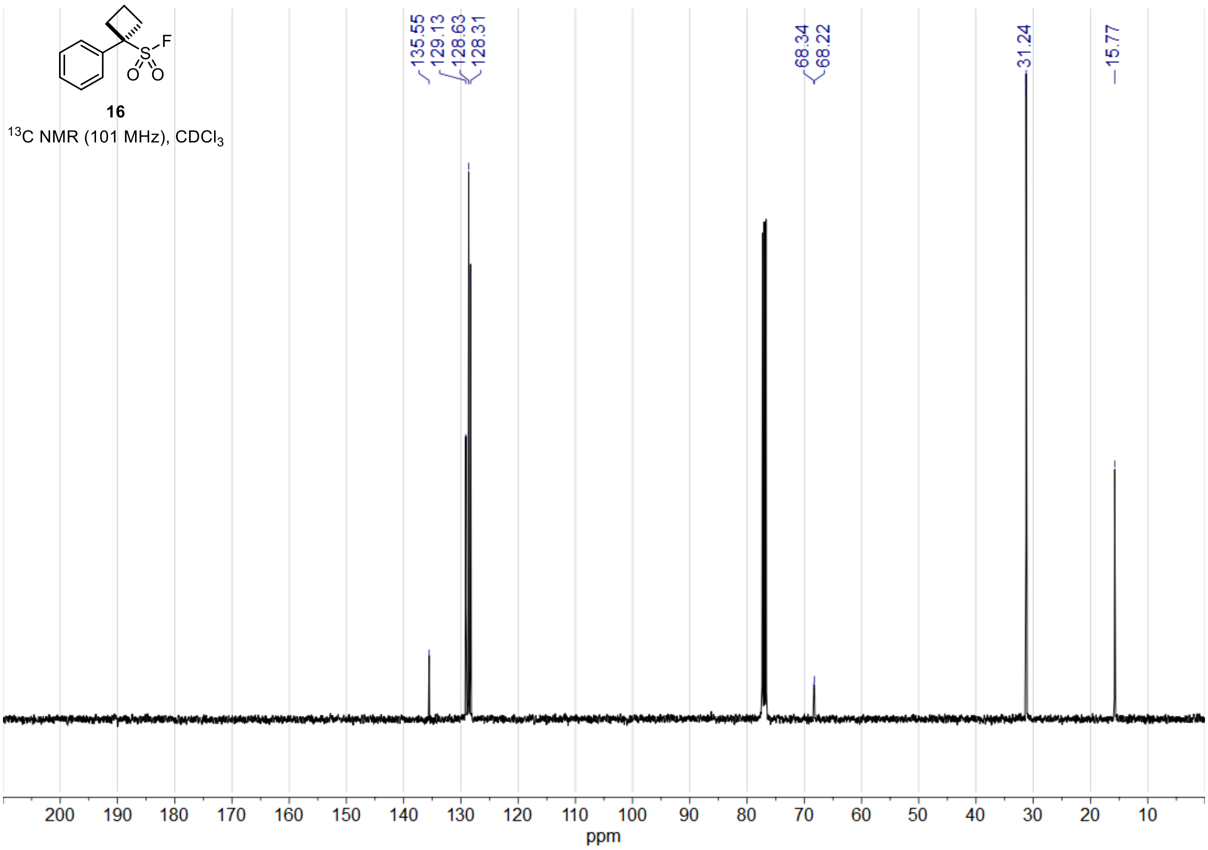
16
^{13}C NMR (101 MHz), CDCl$_3$
135.55
129.13
128.63
128.31
68.34
68.22
31.24
15.77
ppm

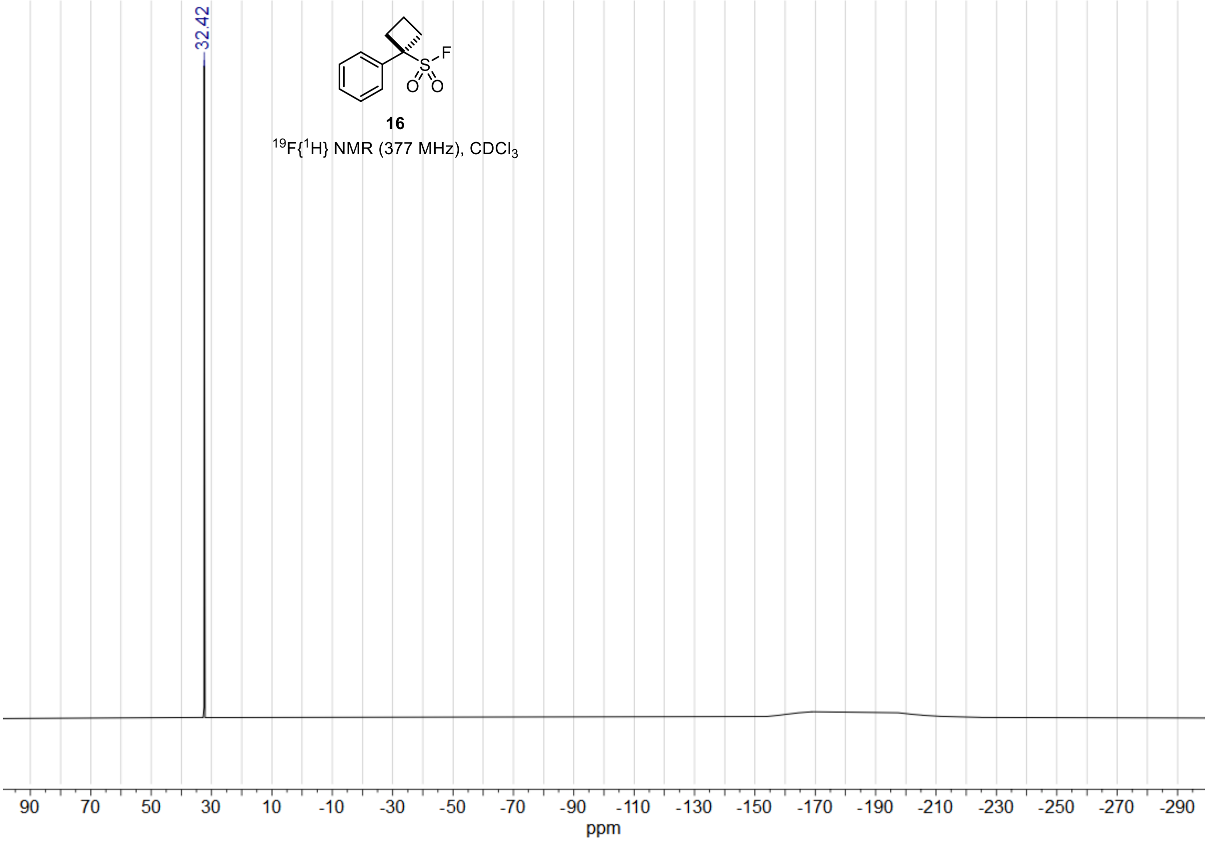

16

$^{19}F\{^{1}H\}$ NMR (377 MHz), $CDCl_3$

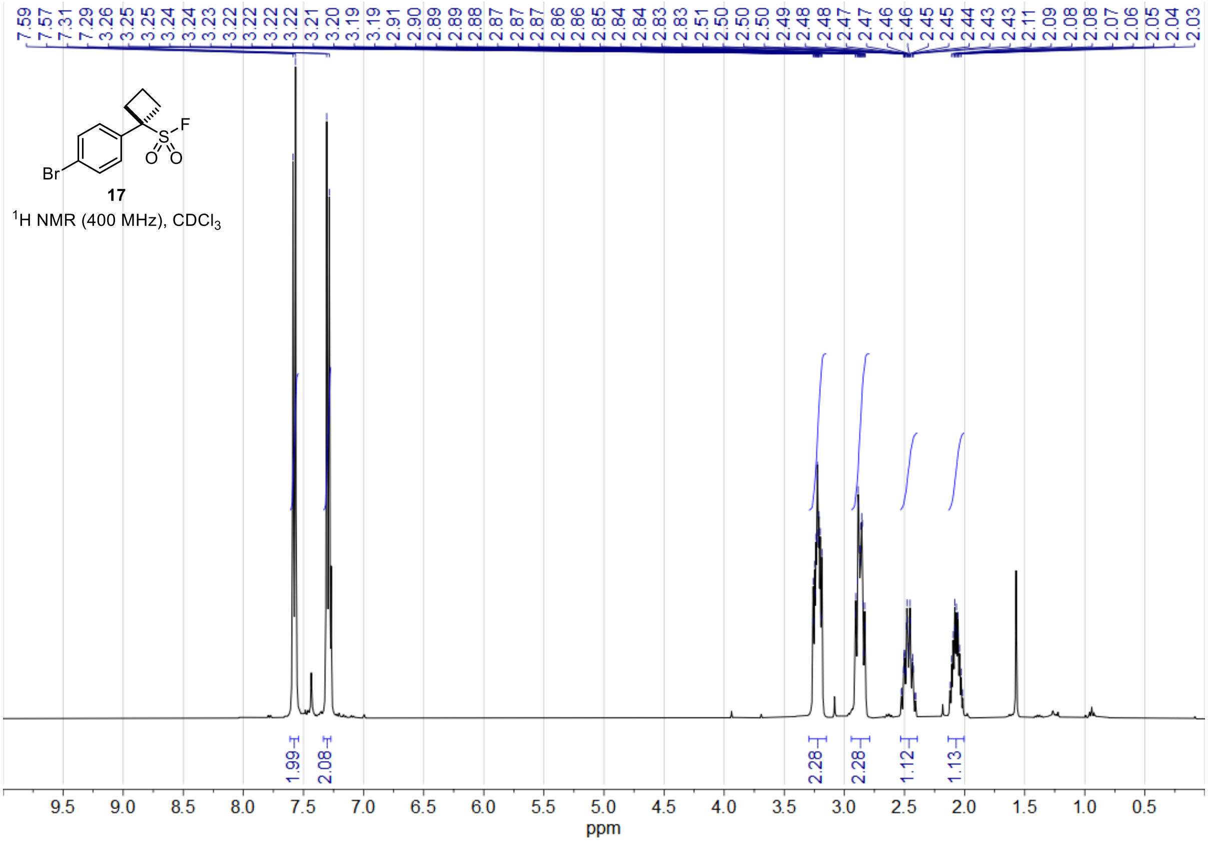
7.59
7.57
7.31
7.29
3.26
3.25
3.25
3.24
3.24
3.23
3.22
3.22
3.22
3.22
3.21
3.20
3.19
2.91
2.90
2.89
2.89
2.88
2.87
2.87
2.87
2.86
2.86
2.85
2.84
2.84
2.83
2.83
2.51
2.50
2.50
2.50
2.49
2.48
2.48
2.47
2.47
2.46
2.46
2.45
2.45
2.44
2.43
2.43
2.11
2.09
2.08
2.08
2.07
2.06
2.05
2.04
2.03
Br
S
F
O
O
17
^{1}H NMR (400 MHz), CDCl$_3$
1.99
2.08
2.28
2.28
1.12
1.13
ppm

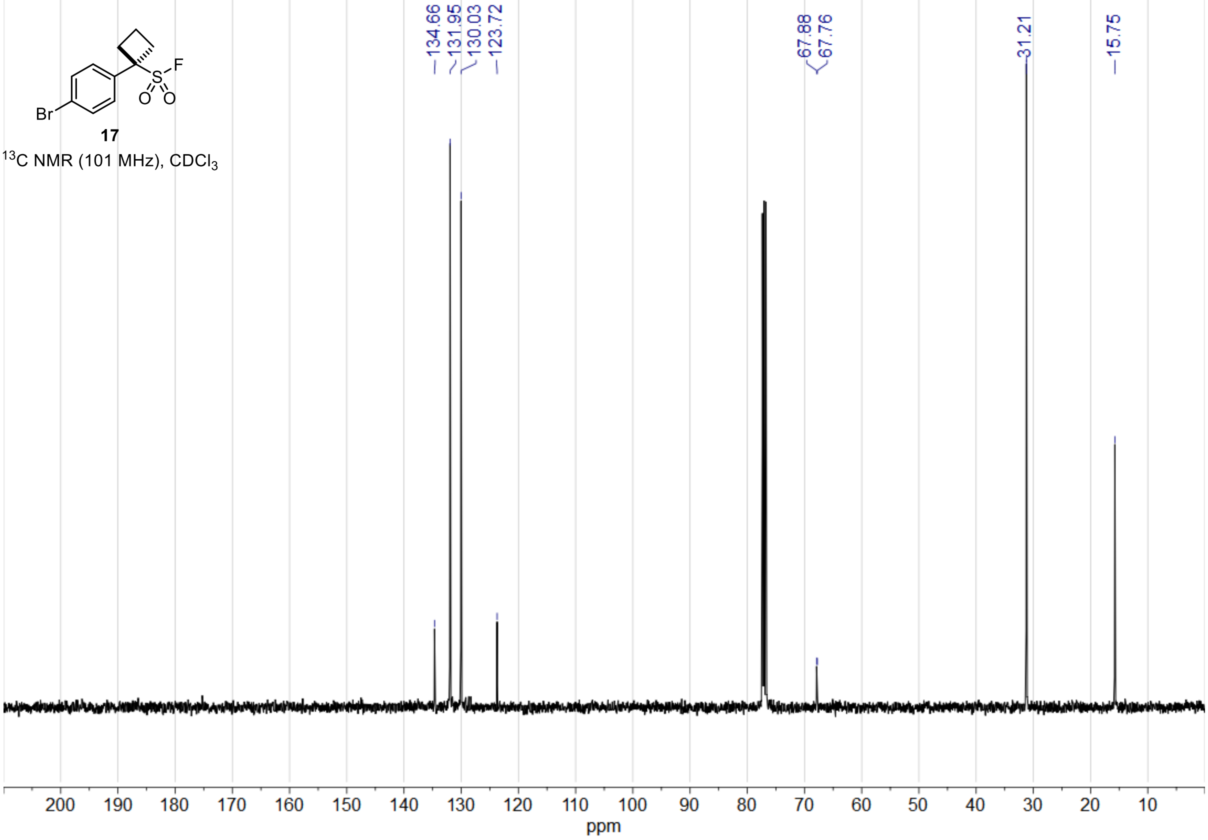
134.66
131.95
130.03
123.72
67.88
67.76
31.21
15.75
Br
S
F
O
O
17
^{13}C NMR (101 MHz), CDCl$_3$
ppm

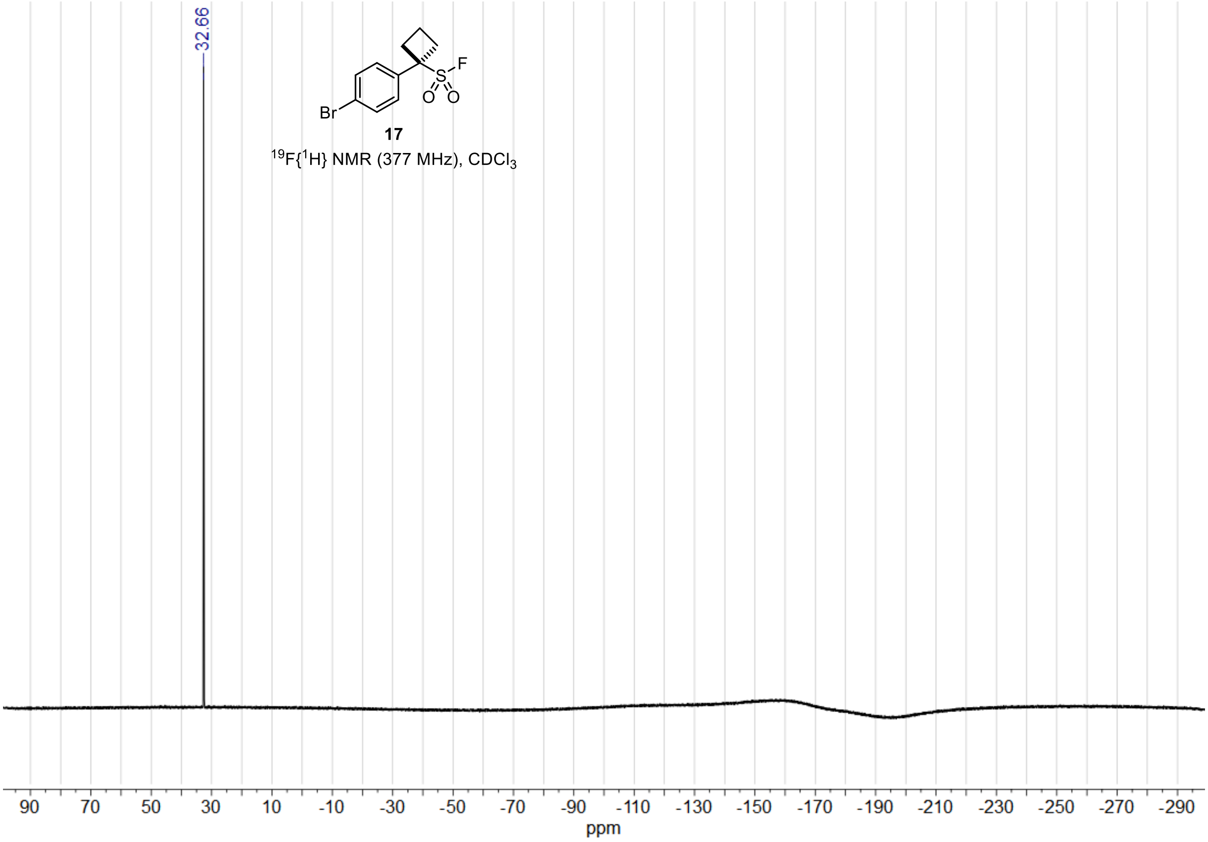
32.66
Br
F
S
O
O
17
$^{19}F\{^1H\}$ NMR (377 MHz), $CDCl_3$
90
70
50
30
10
-10
-30
-50
-70
-90
-110
-130
-150
-170
-190
-210
-230
-250
-270
-290
ppm

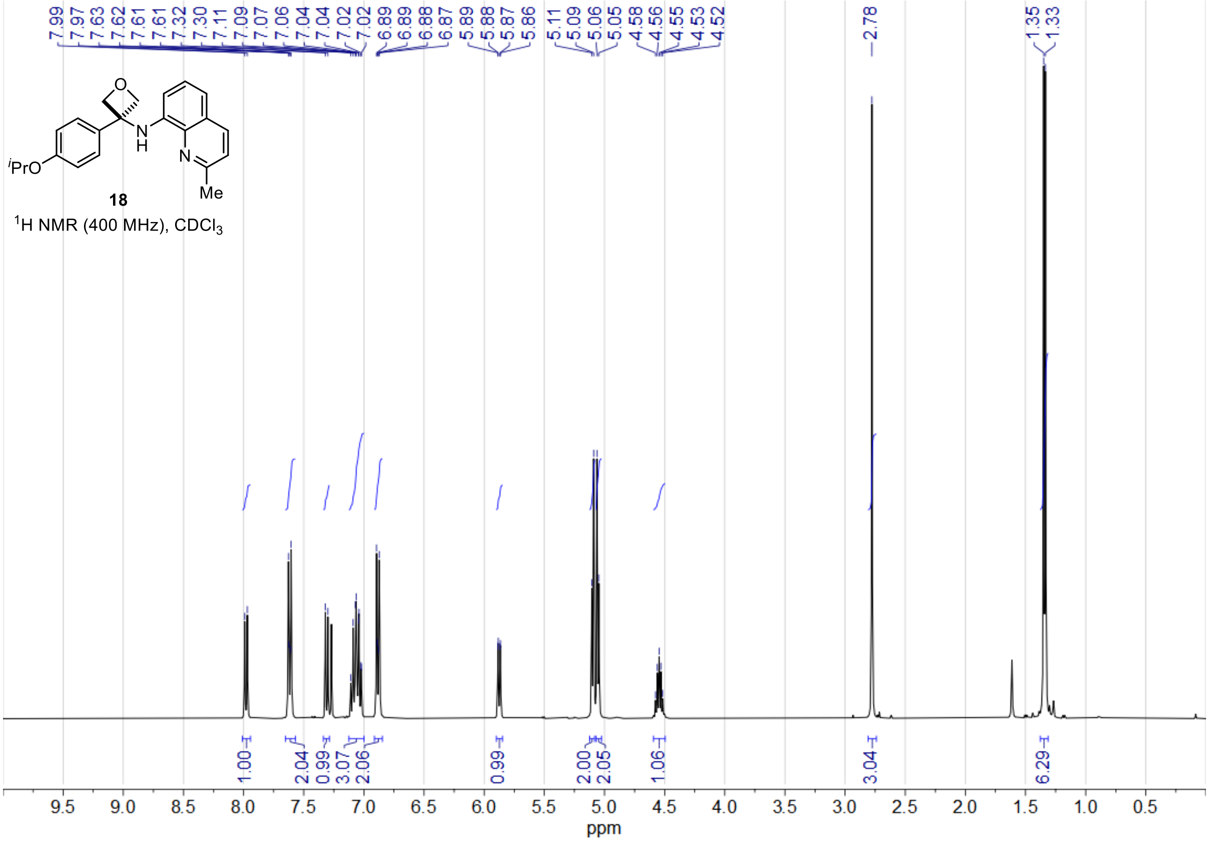
7.99
7.97
7.63
7.62
7.61
7.61
7.32
7.30
7.11
7.09
7.07
7.06
7.04
7.04
7.02
7.02
6.89
6.89
6.88
6.87
5.89
5.88
5.87
5.86
5.11
5.09
5.06
5.05
4.58
4.56
4.55
4.53
4.52
2.78
1.35
1.33
iPrO
O
N
H
N
Me
18
1H NMR (400 MHz), CDCl3
1.00
2.04
0.99
3.07
2.06
0.99
2.00
2.05
1.06
3.04
6.29
9.5
9.0
8.5
8.0
7.5
7.0
6.5
6.0
5.5
5.0
4.5
4.0
3.5
3.0
2.5
2.0
1.5
1.0
0.5
ppm

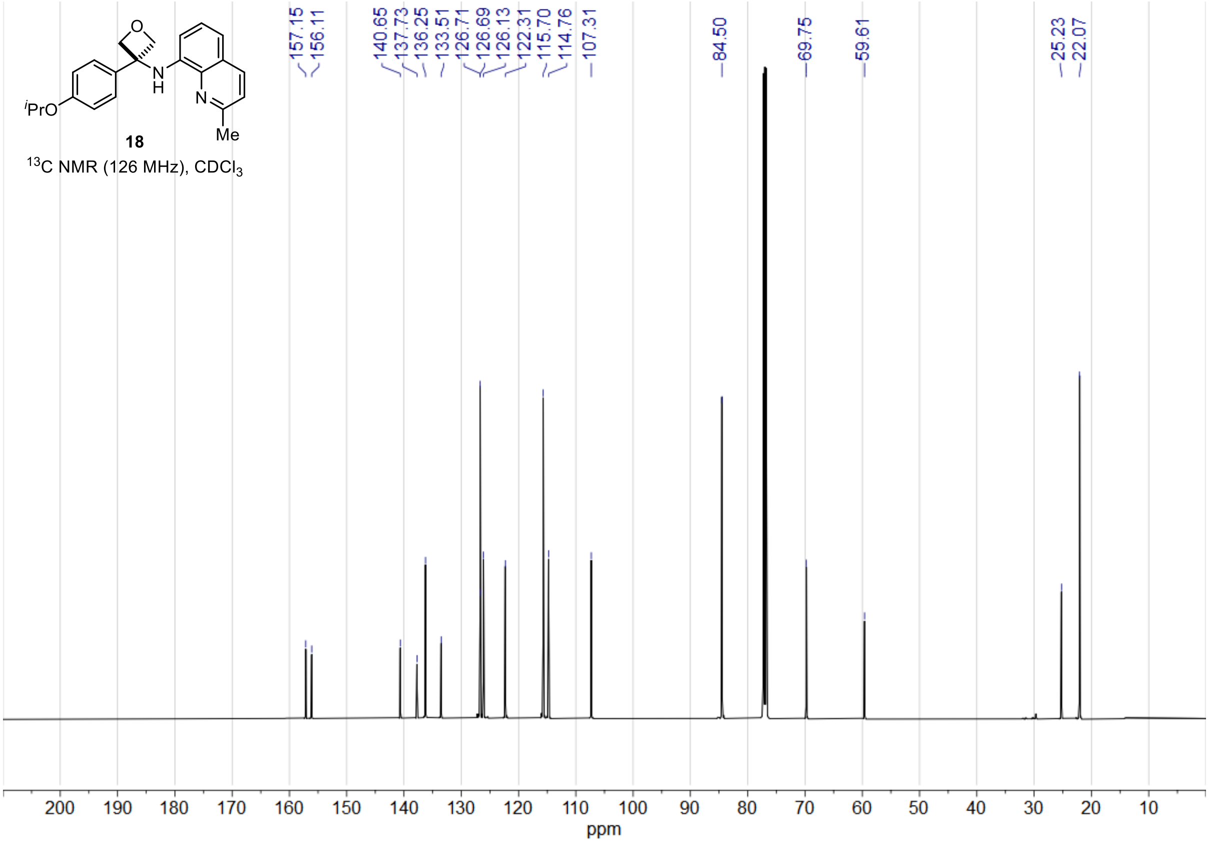
157.15
156.11
140.65
137.73
136.25
133.51
126.71
126.69
126.13
122.31
115.70
114.76
107.31
84.50
69.75
59.61
25.23
22.07
iPrO
O
N
H
N
Me
18
13C NMR (126 MHz), CDCl3
200
190
180
170
160
150
140
130
120
110
100
90
80
70
60
50
40
30
20
10
ppm

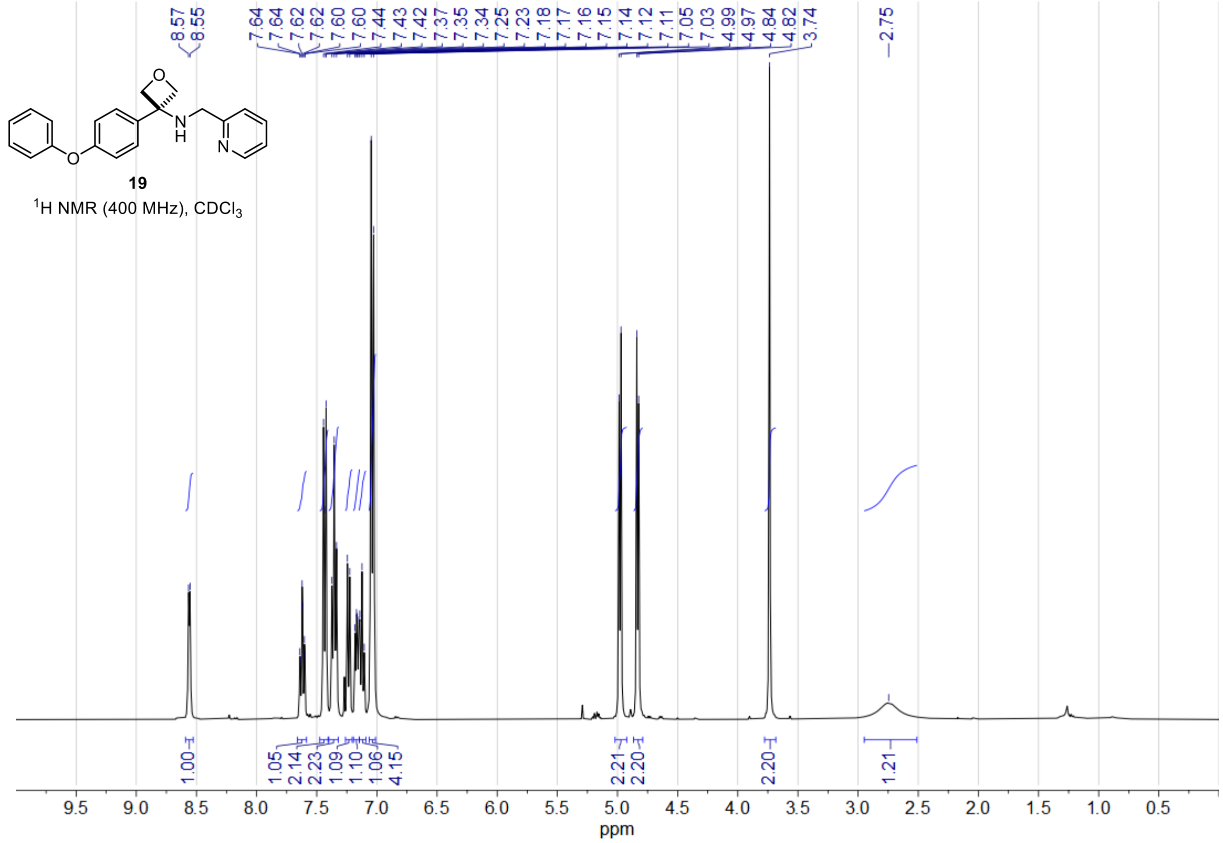
19
1H NMR (400 MHz), CDCl3
8.57
8.55
7.64
7.64
7.62
7.62
7.60
7.44
7.43
7.42
7.37
7.35
7.34
7.25
7.23
7.18
7.17
7.16
7.15
7.14
7.12
7.11
7.05
7.03
4.99
4.97
4.84
4.82
3.74
2.75
1.00
1.05
2.14
2.23
1.09
1.10
1.06
4.15
2.21
2.20
2.20
1.21
ppm

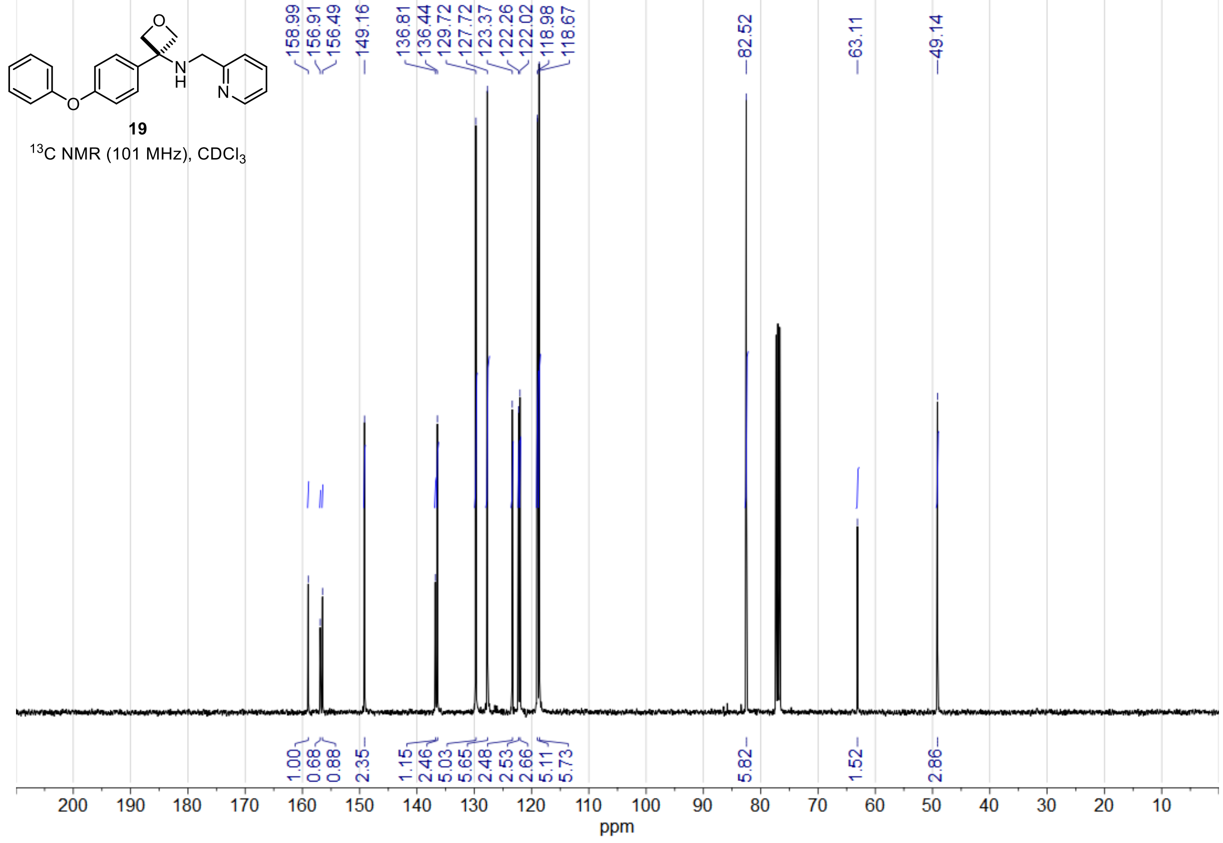
19
13C NMR (101 MHz), CDCl3
158.99
156.91
156.49
149.16
136.81
136.44
129.72
127.72
123.37
122.26
122.02
118.98
118.67
82.52
63.11
49.14
1.00
0.68
0.88
2.35
1.15
2.46
5.03
5.65
2.48
2.53
2.66
5.11
5.73
5.82
1.52
2.86
ppm

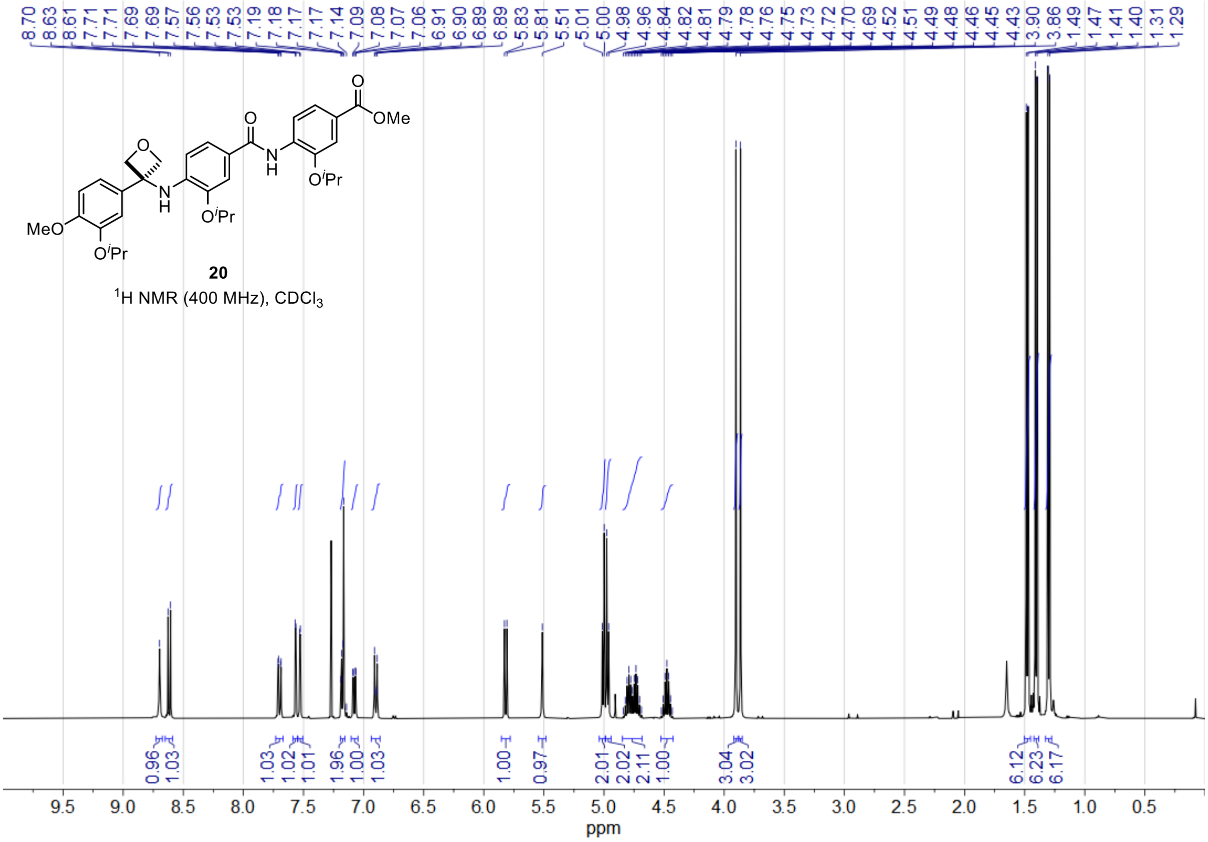
OMe
OⁱPr
MeO
20
¹H NMR (400 MHz), CDCl₃
8.70 8.63 8.61 7.71 7.71 7.69 7.69 7.57 7.56 7.53 7.53 7.19 7.18 7.17 7.17 7.14 7.09 7.08 7.07 7.06 6.91 6.90 6.89 6.89 5.83 5.81 5.51 5.01 5.00 4.98 4.96 4.84 4.82 4.81 4.79 4.78 4.76 4.75 4.73 4.72 4.70 4.69 4.52 4.51 4.49 4.48 4.46 4.45 4.43 3.90 3.86 1.49 1.47 1.41 1.40 1.31 1.29
0.96 1.03 1.03 1.02 1.01 1.96 1.00 1.03 1.00 0.97 2.01 2.02 2.11 1.00 3.04 3.02 6.12 6.23 6.17
ppm

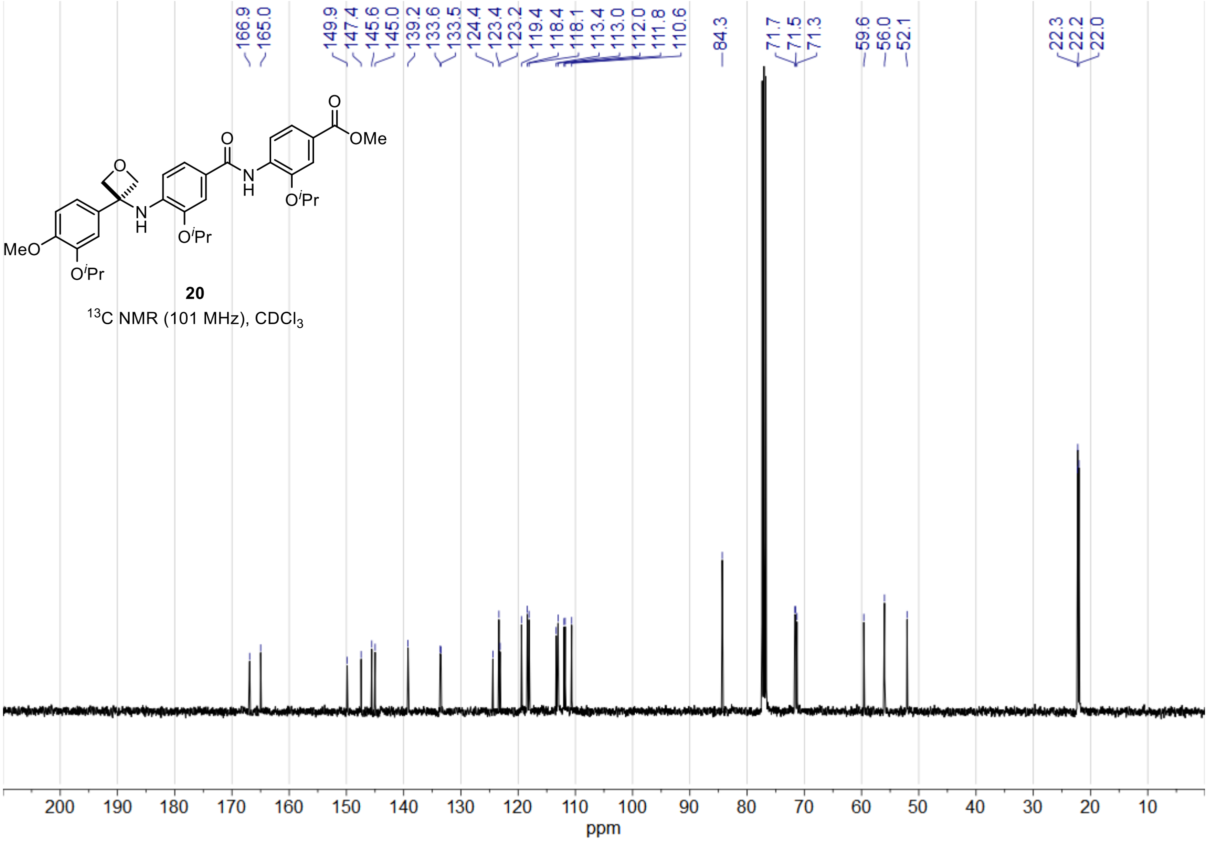
OMe
MeO
20
¹³C NMR (101 MHz), CDCl₃
166.9 165.0 149.9 147.4 145.6 145.0 139.2 133.6 133.5 124.4 123.4 123.2 119.4 118.4 118.1 113.4 113.0 112.0 111.8 110.6 84.3 71.7 71.5 71.3 59.6 56.0 52.1 22.3 22.2 22.0
ppm

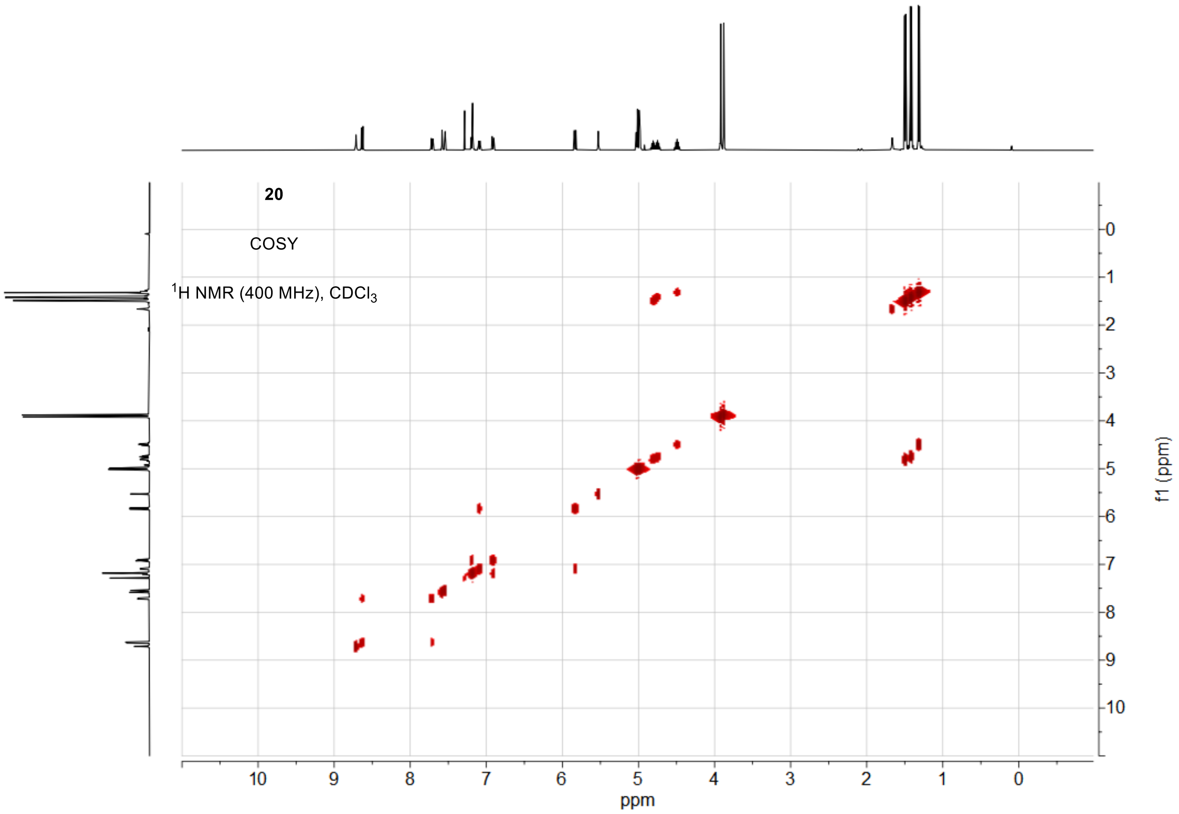
20
COSY
¹H NMR (400 MHz), CDCl₃
f1 (ppm)
ppm

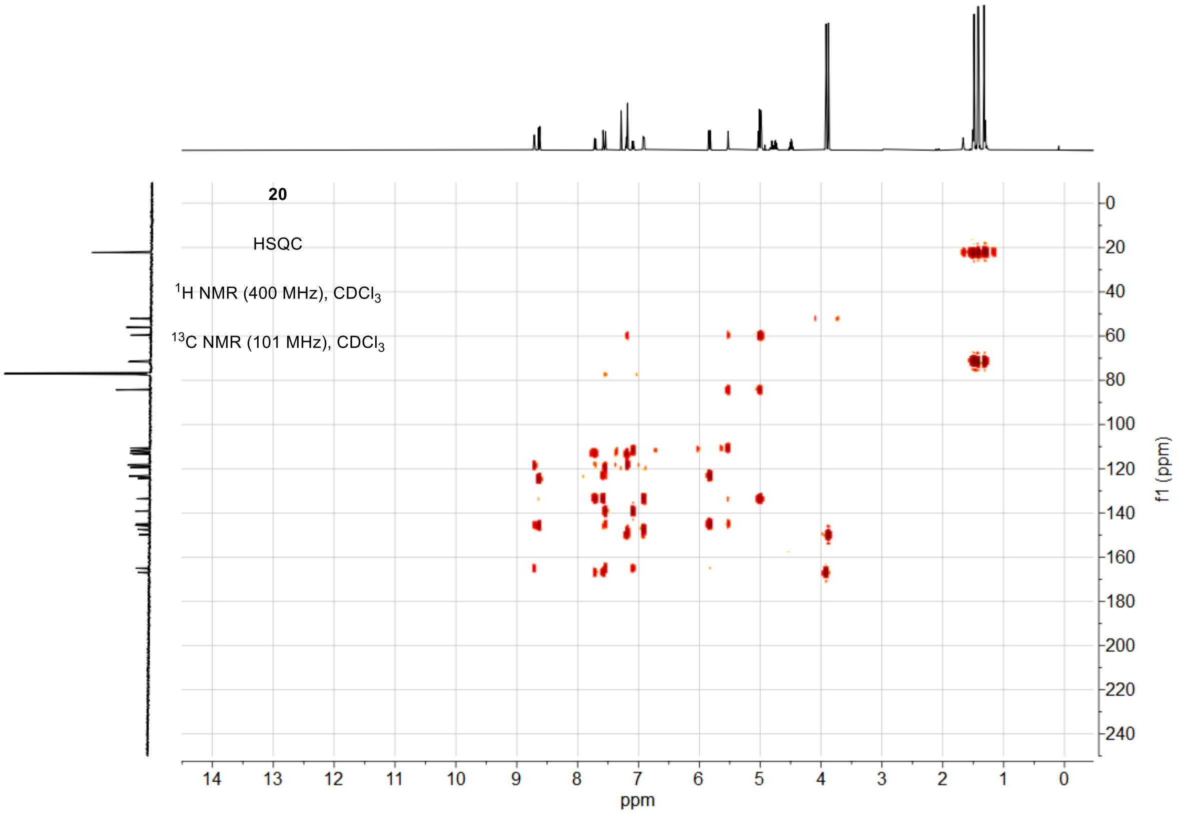
20
HSQC
¹H NMR (400 MHz), CDCl₃
¹³C NMR (101 MHz), CDCl₃
f1 (ppm)
ppm

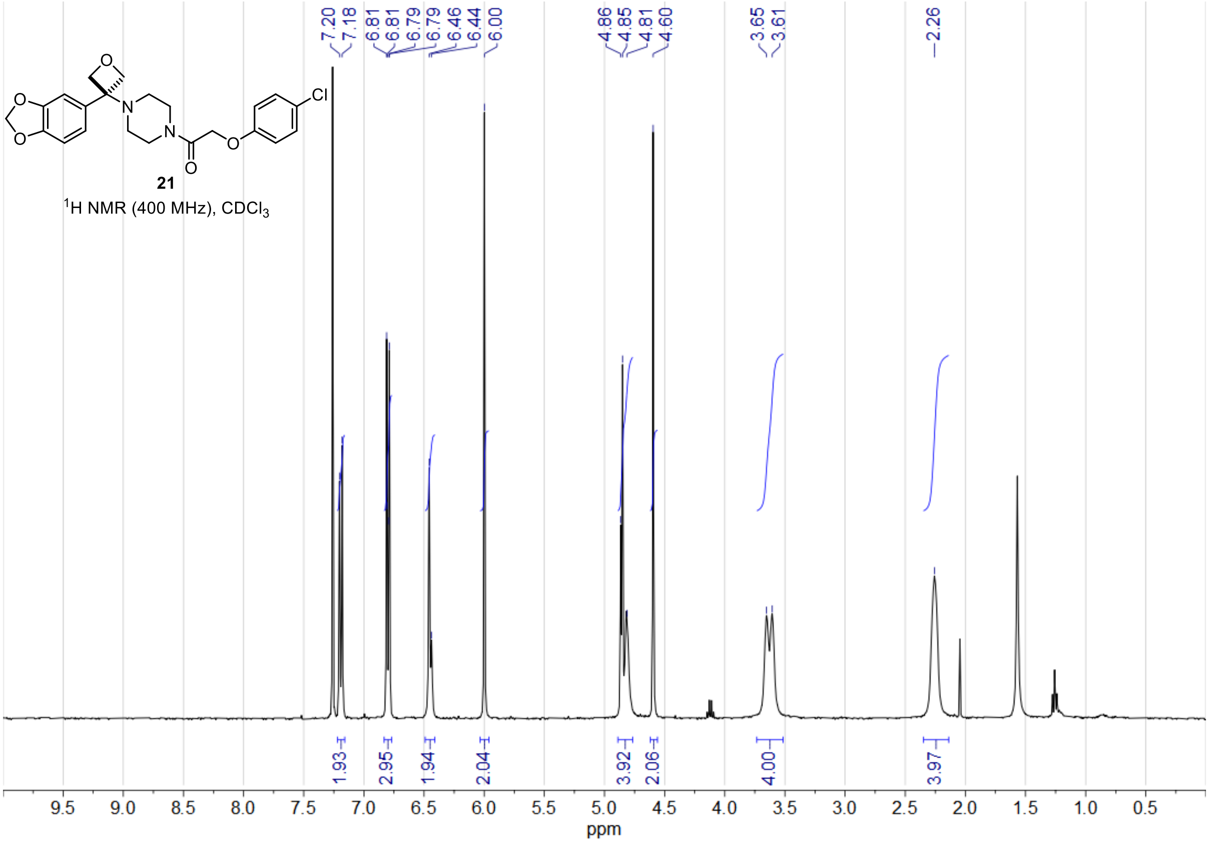
7.20
7.18
6.81
6.81
6.79
6.79
6.46
6.44
6.00
4.86
4.85
4.81
4.60
3.65
3.61
2.26
Cl
O
O
O
N
N
O
O
21
^{1}H NMR (400 MHz), CDCl$_3$
1.93
2.95
1.94
2.04
3.92
2.06
4.00
3.97
9.5 9.0 8.5 8.0 7.5 7.0 6.5 6.0 5.5 5.0 4.5 4.0 3.5 3.0 2.5 2.0 1.5 1.0 0.5
ppm

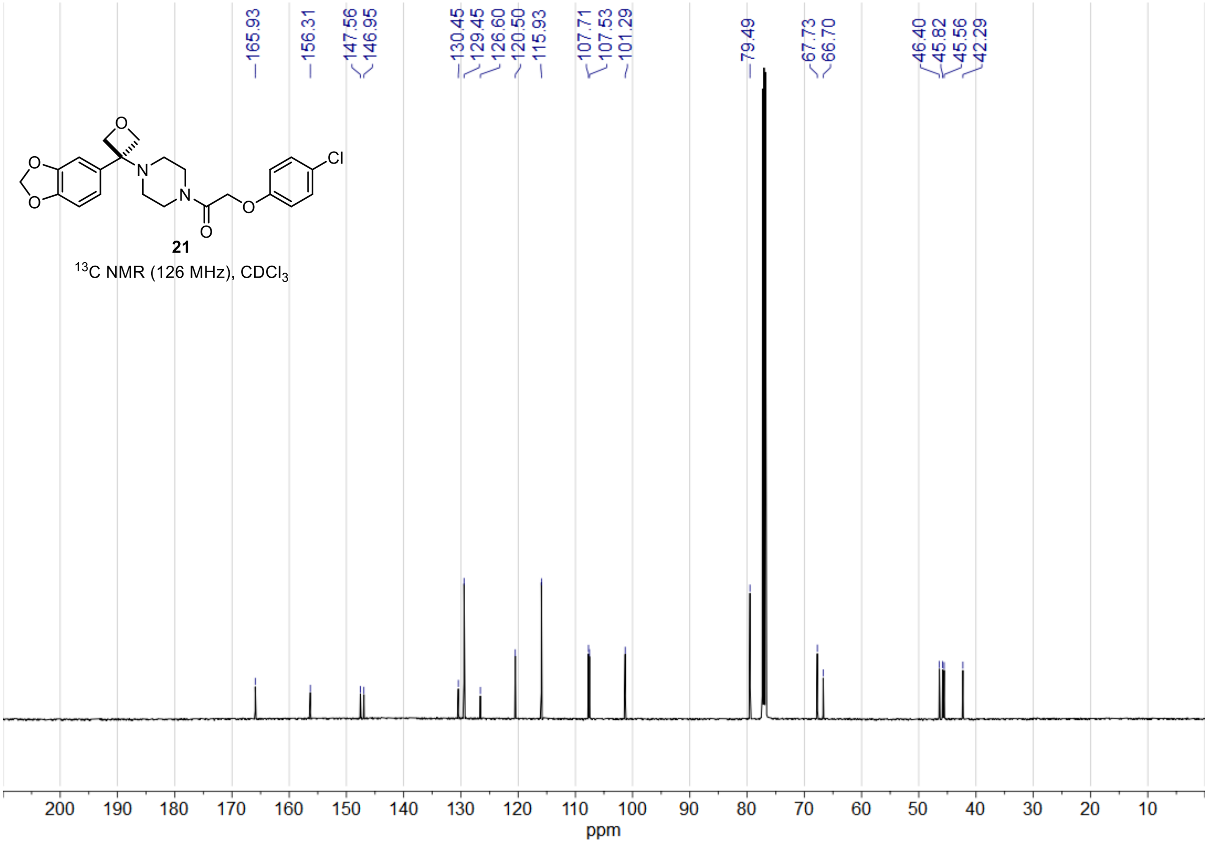
165.93
156.31
147.56
146.95
130.45
129.45
126.60
120.50
115.93
107.71
107.53
101.29
79.49
67.73
66.70
46.40
45.82
45.56
42.29
Cl
O
O
O
N
N
O
O
21
^{13}C NMR (126 MHz), CDCl$_3$
200 190 180 170 160 150 140 130 120 110 100 90 80 70 60 50 40 30 20 10
ppm

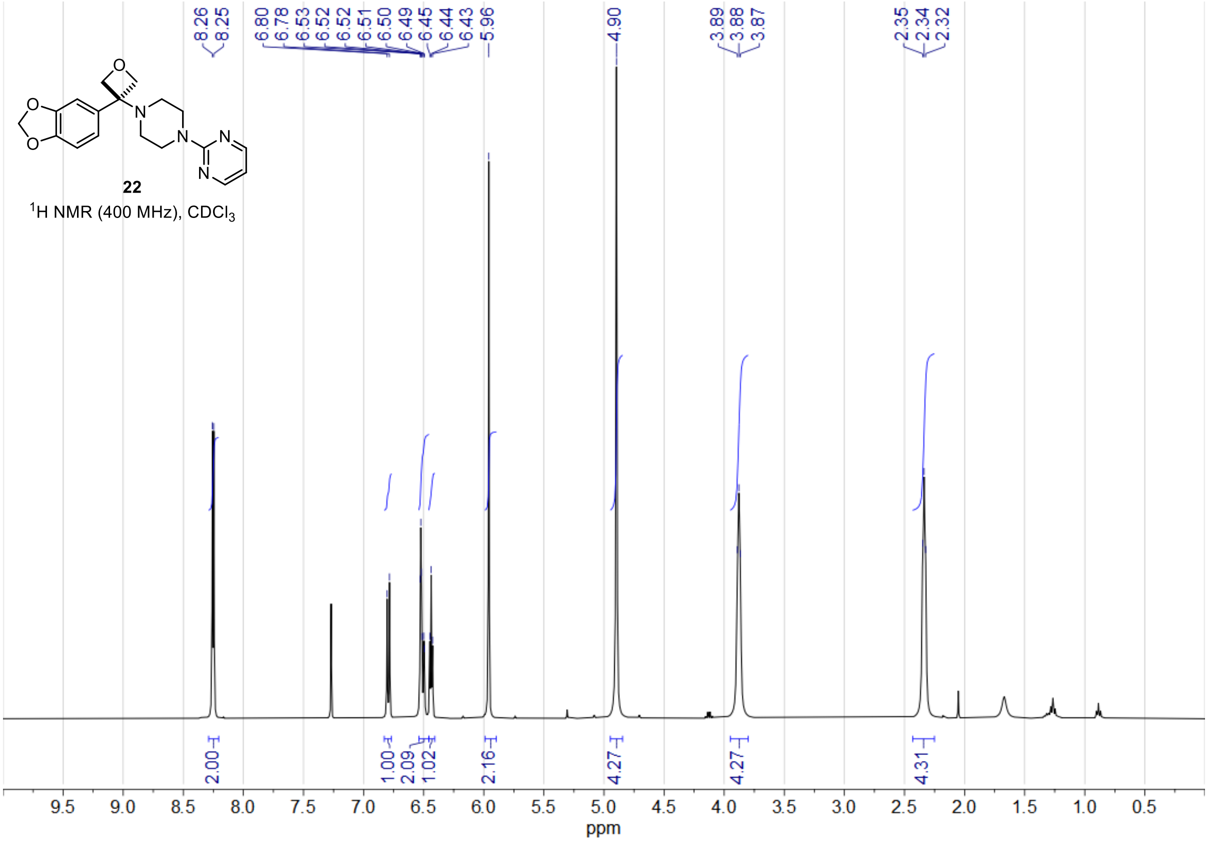
8.26
8.25
6.80
6.78
6.53
6.52
6.52
6.51
6.50
6.49
6.45
6.44
6.43
5.96
4.90
3.89
3.88
3.87
2.35
2.34
2.32
22
^{1}H NMR (400 MHz), CDCl$_3$
2.00
1.00
2.09
1.02
2.16
4.27
4.27
4.31
ppm

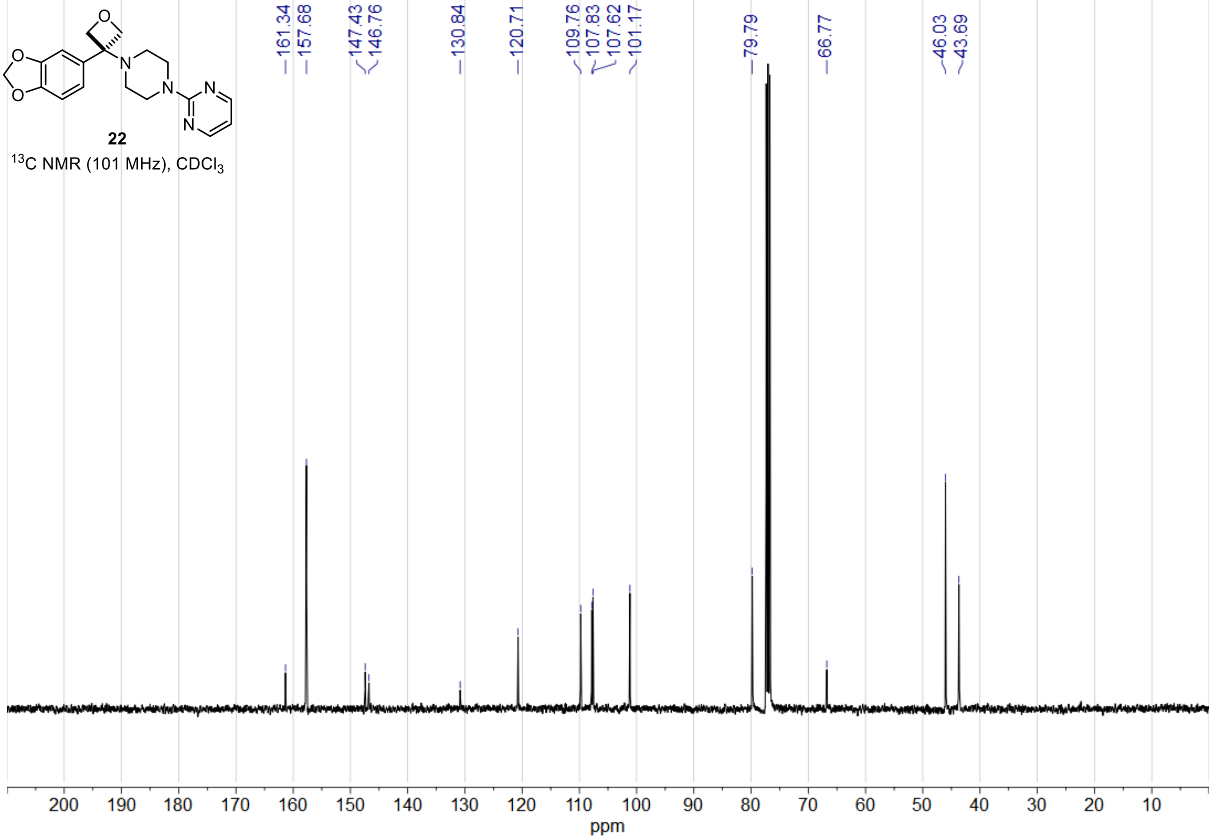
161.34
157.68
147.43
146.76
130.84
120.71
109.76
107.83
107.62
101.17
79.79
66.77
46.03
43.69
22
^{13}C NMR (101 MHz), CDCl$_3$
ppm

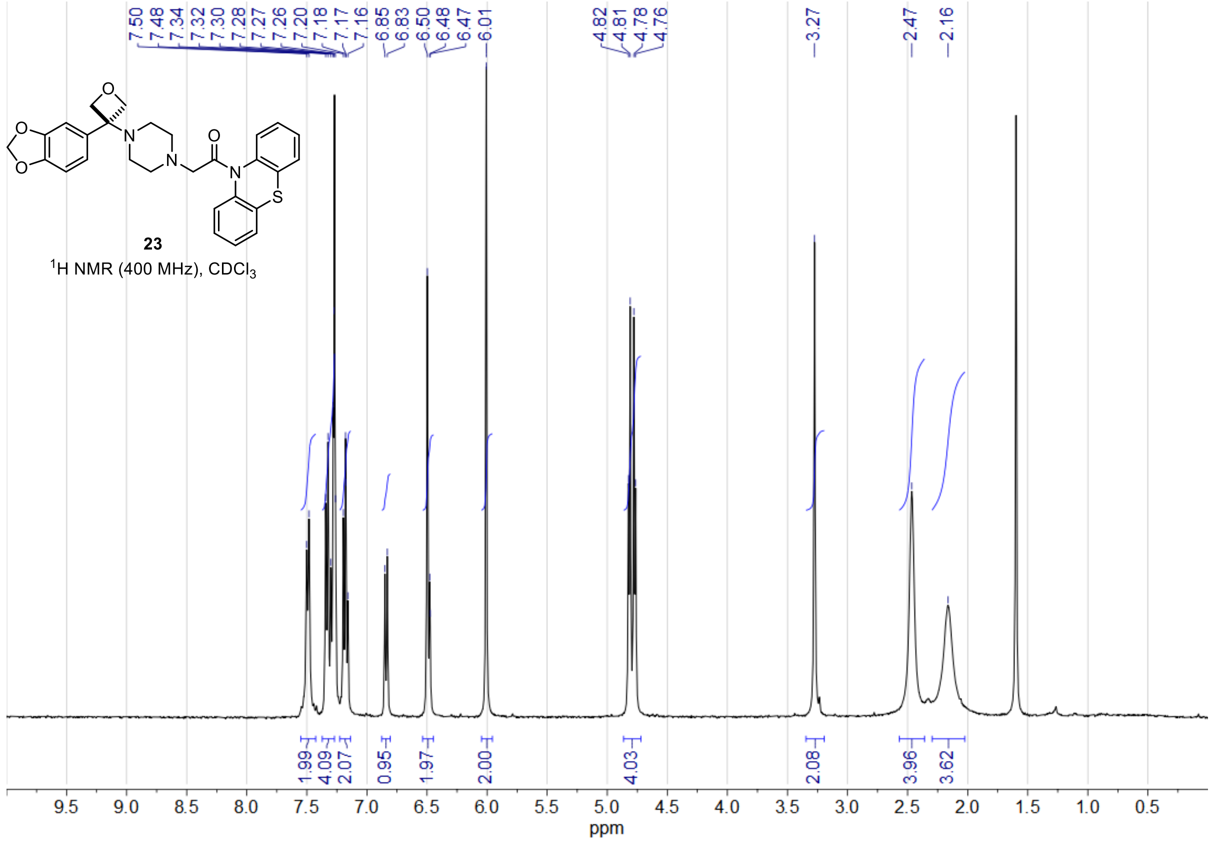
23
1H NMR (400 MHz), CDCl3
7.50 7.48 7.34 7.32 7.30 7.28 7.27 7.26 7.20 7.18 7.17 7.16 6.85 6.83 6.50 6.48 6.47 6.01 4.82 4.81 4.78 4.76 3.27 2.47 2.16
1.99 4.09 2.07 0.95 1.97 2.00 4.03 2.08 3.96 3.62
ppm

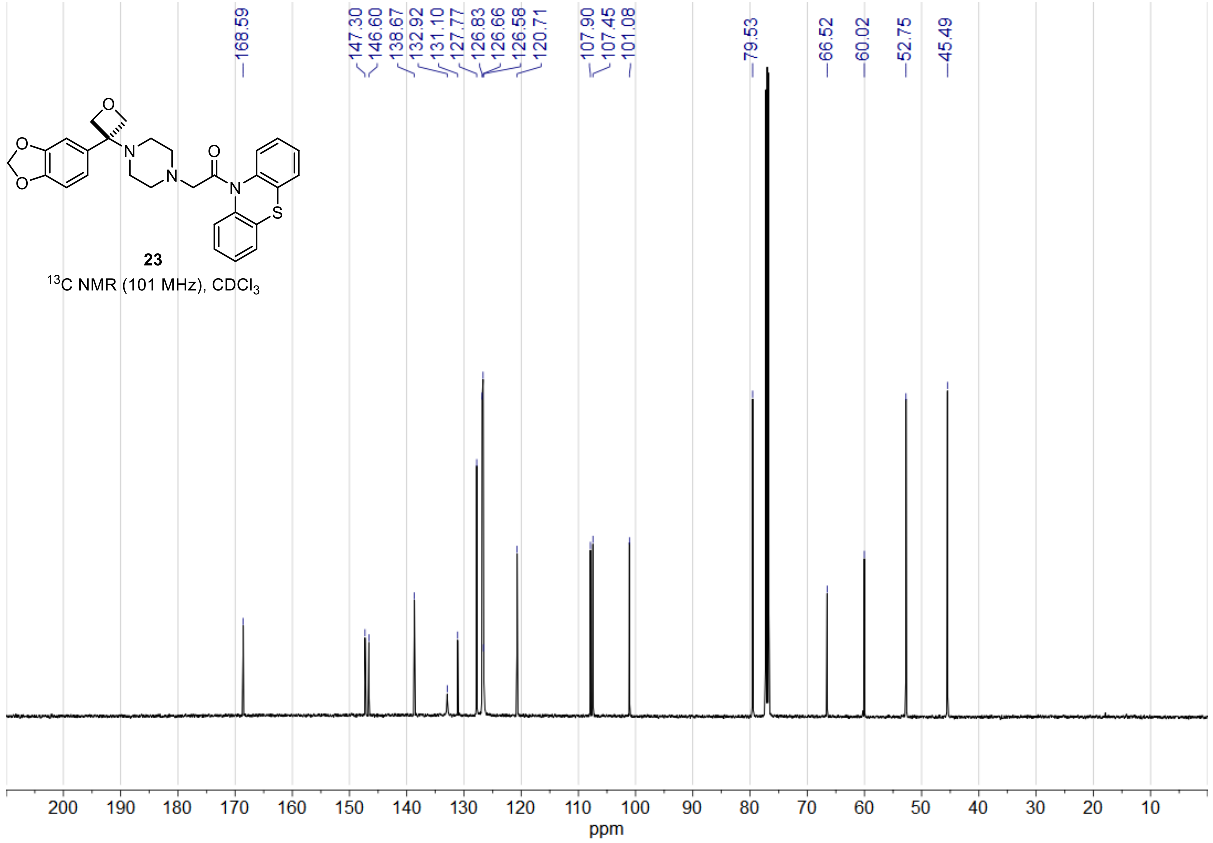
23
13C NMR (101 MHz), CDCl3
168.59 147.30 146.60 138.67 132.92 131.10 127.77 126.83 126.66 126.58 120.71 107.90 107.45 101.08 79.53 66.52 60.02 52.75 45.49
ppm

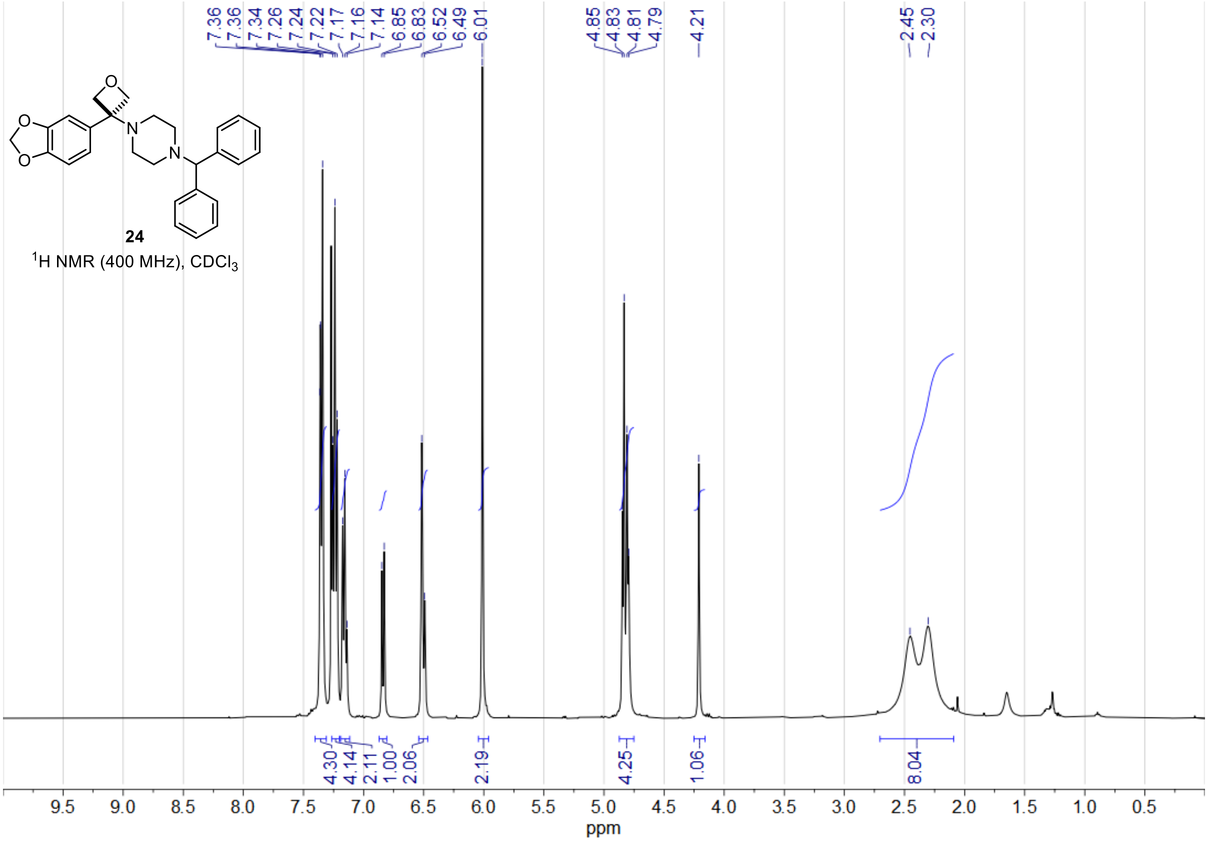
24
^{1}H NMR (400 MHz), $CDCl_3$
7.36
7.36
7.34
7.26
7.24
7.22
7.17
7.16
7.14
6.85
6.83
6.52
6.49
6.01
4.85
4.83
4.81
4.79
4.21
2.45
2.30
4.30
4.14
2.11
1.00
2.06
2.19
4.25
1.06
8.04
9.5
9.0
8.5
8.0
7.5
7.0
6.5
6.0
5.5
5.0
4.5
4.0
3.5
3.0
2.5
2.0
1.5
1.0
0.5
ppm

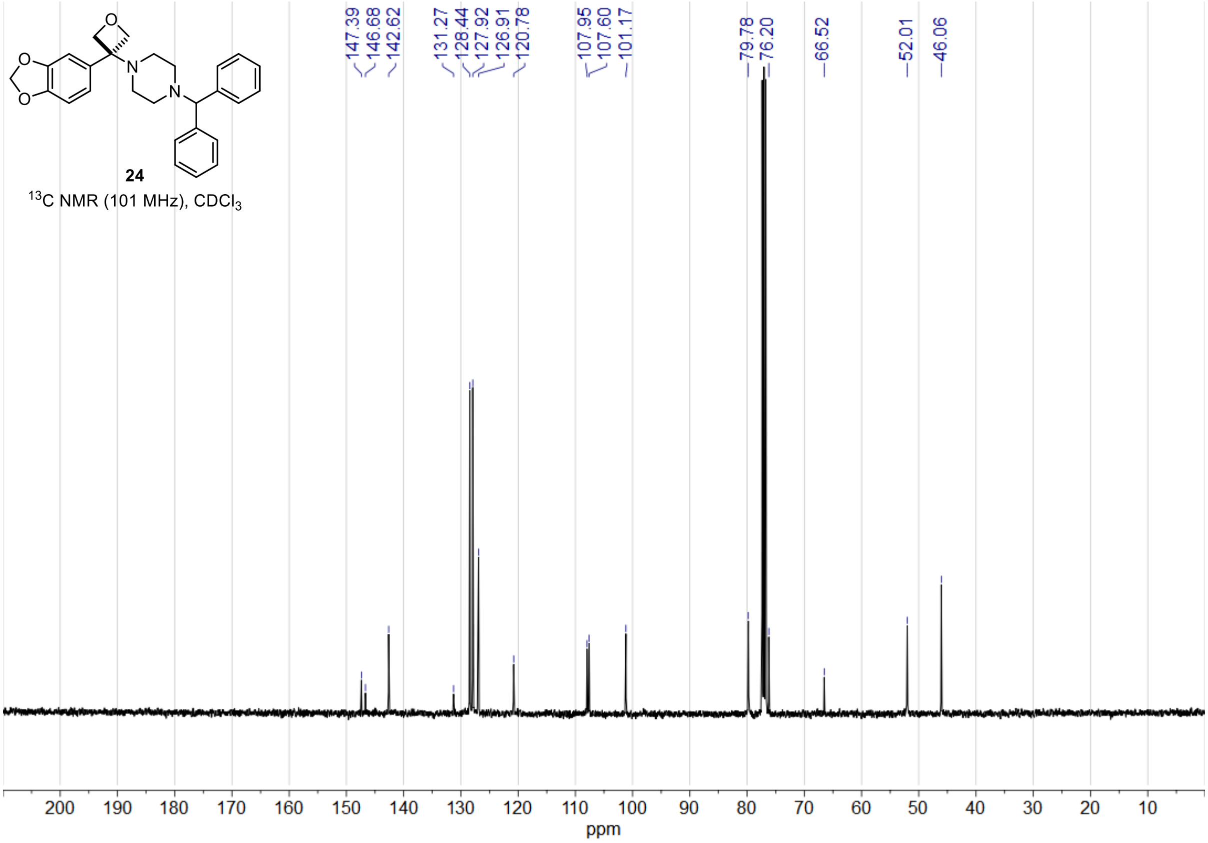
24
^{13}C NMR (101 MHz), $CDCl_3$
147.39
146.68
142.62
131.27
128.44
127.92
126.91
120.78
107.95
107.60
101.17
79.78
76.20
66.52
52.01
46.06
200
190
180
170
160
150
140
130
120
110
100
90
80
70
60
50
40
30
20
10
ppm

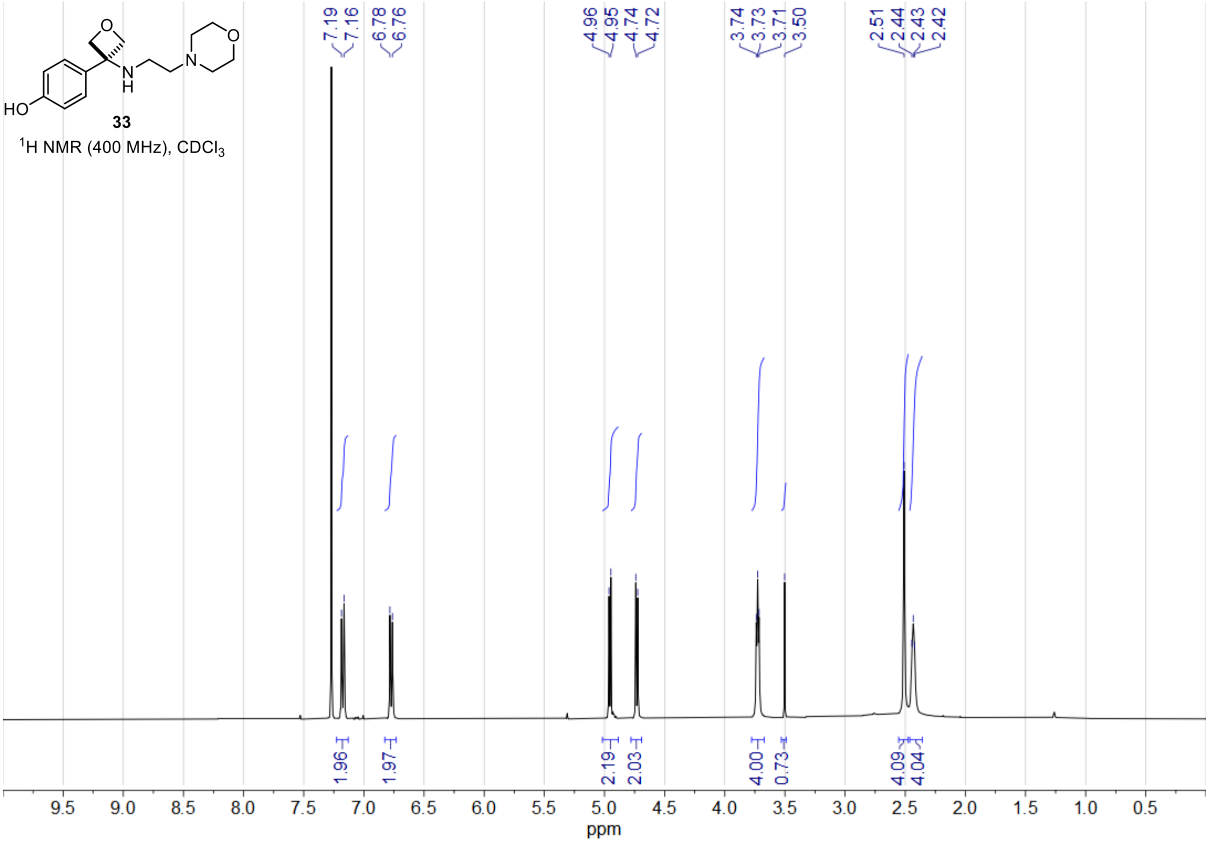
HO
O
N
H
N
O
33
^{1}H NMR (400 MHz), CDCl$_3$
7.19
7.16
6.78
6.76
4.96
4.95
4.74
4.72
3.74
3.73
3.71
3.50
2.51
2.44
2.43
2.42
1.96
1.97
2.19
2.03
4.00
0.73
4.09
4.04
9.5 9.0 8.5 8.0 7.5 7.0 6.5 6.0 5.5 5.0 4.5 4.0 3.5 3.0 2.5 2.0 1.5 1.0 0.5
ppm

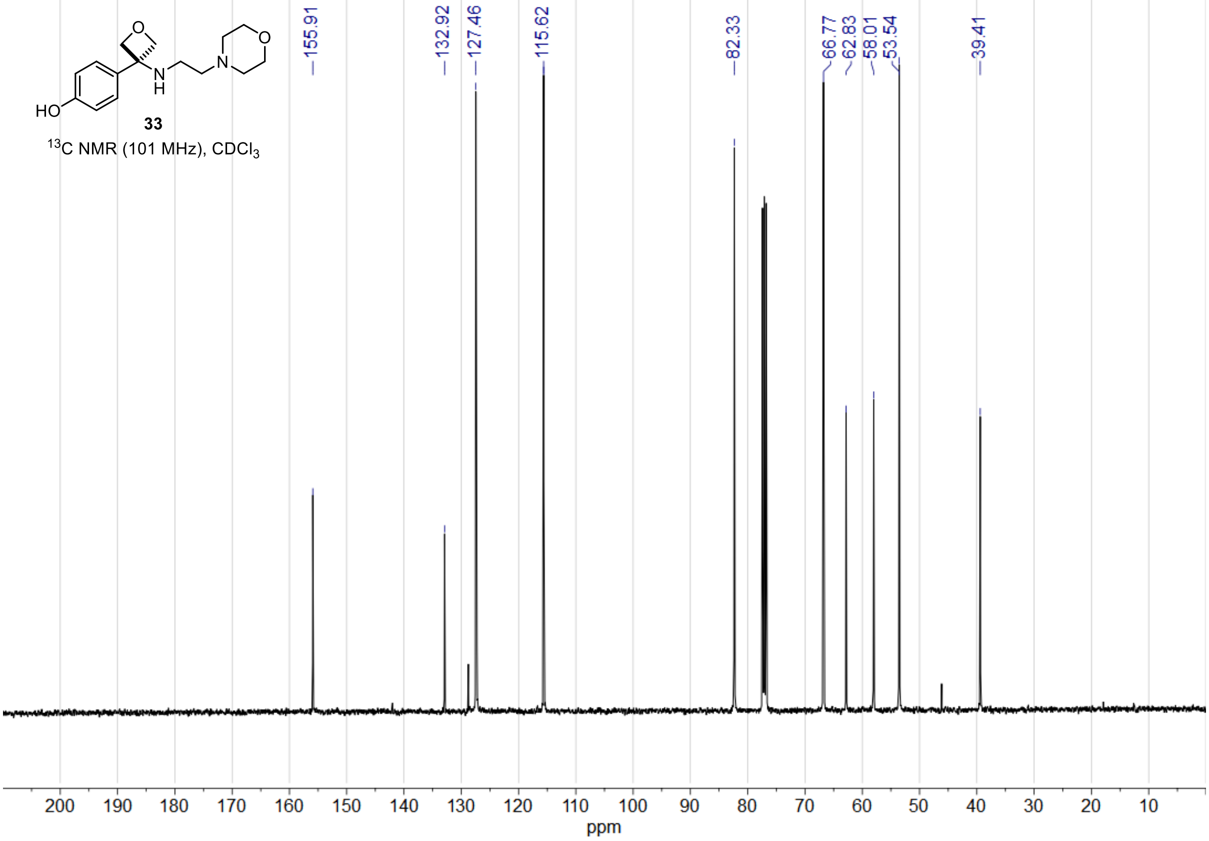
HO
O
N
H
N
O
33
^{13}C NMR (101 MHz), CDCl$_3$
155.91
132.92
127.46
115.62
82.33
66.77
62.83
58.01
53.54
39.41
200 190 180 170 160 150 140 130 120 110 100 90 80 70 60 50 40 30 20 10
ppm

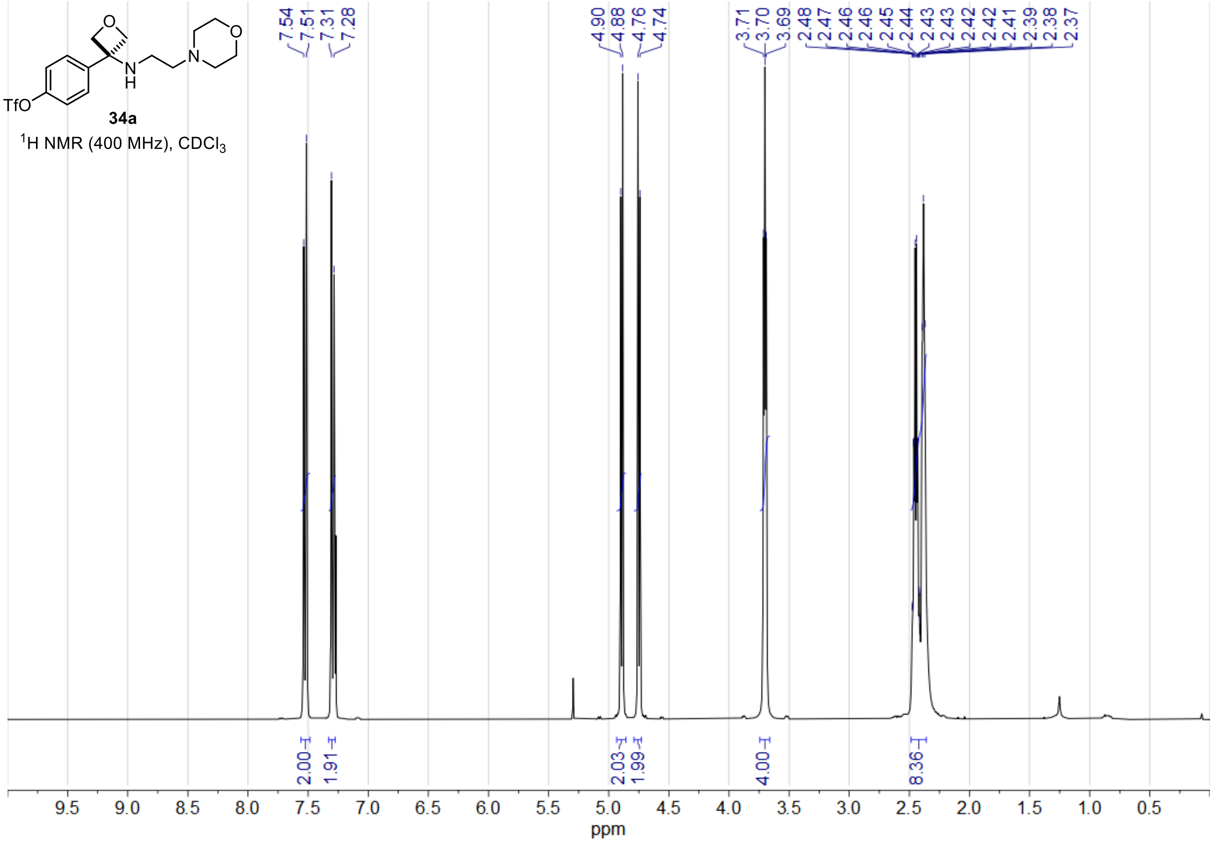
TfO
34a
¹H NMR (400 MHz), CDCl₃
7.54
7.51
7.31
7.28
4.90
4.88
4.76
4.74
3.71
3.70
3.69
2.48
2.47
2.46
2.46
2.45
2.44
2.43
2.43
2.42
2.42
2.41
2.39
2.38
2.37
2.00
1.91
2.03
1.99
4.00
8.36
ppm

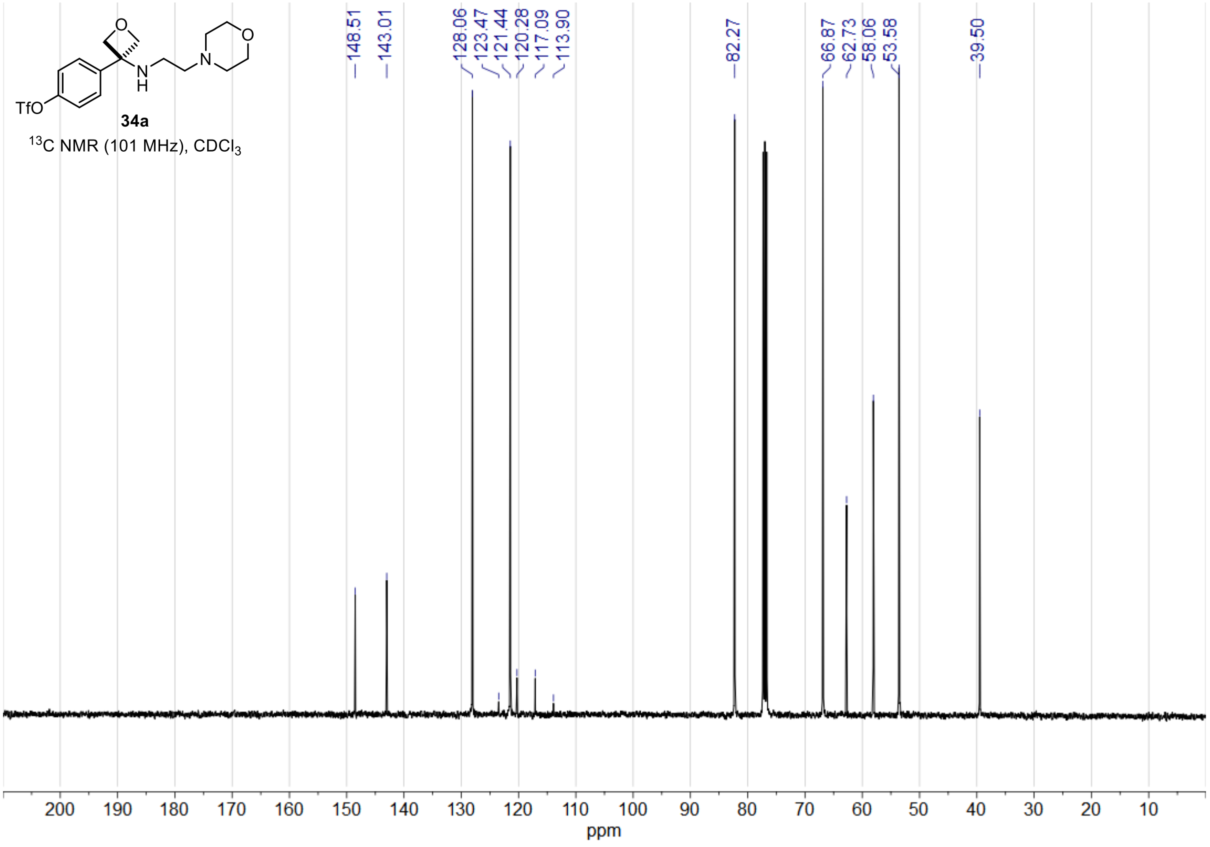
TfO
34a
¹³C NMR (101 MHz), CDCl₃
148.51
143.01
128.06
123.47
121.44
120.28
117.09
113.90
82.27
66.87
62.73
58.06
53.58
39.50
ppm

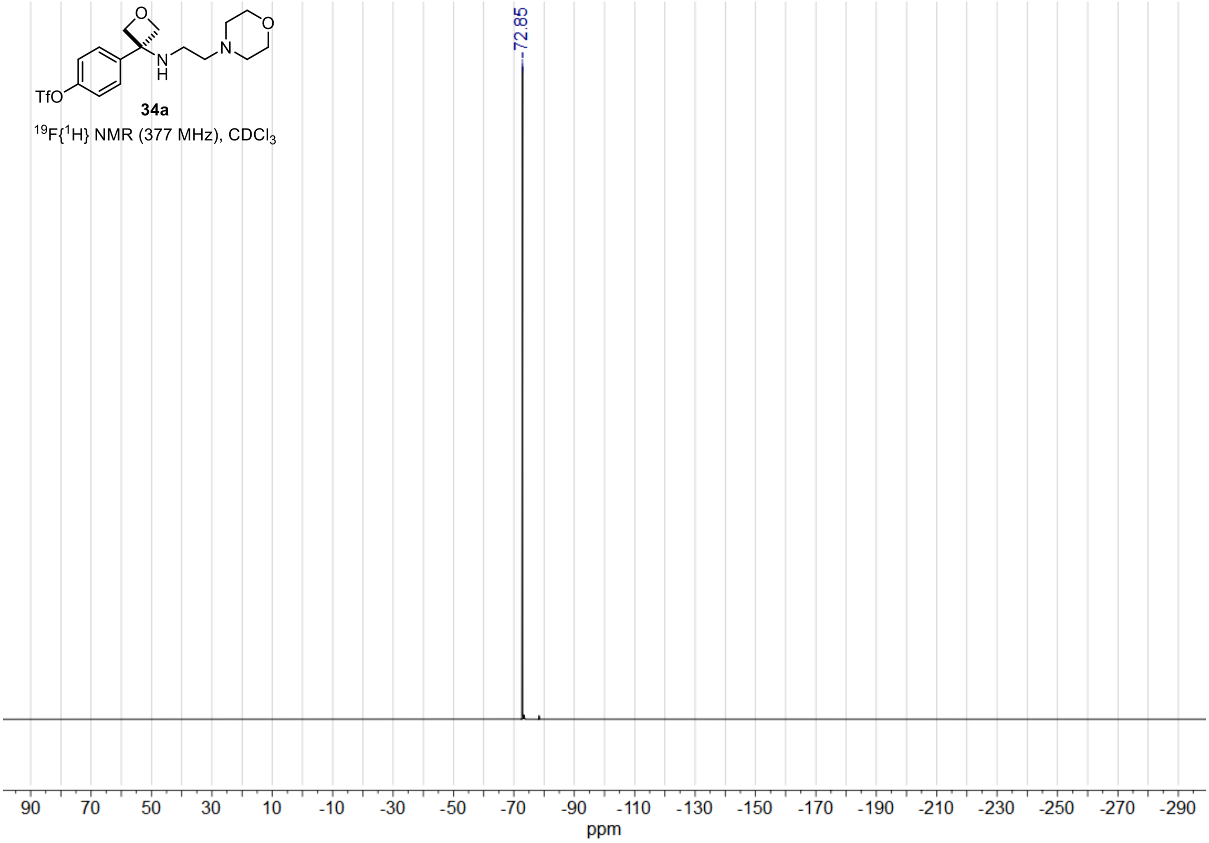
TfO
O
N
H
N
O
34a
$^{19}F\{^{1}H\}$ NMR (377 MHz), $CDCl_3$
-72.85
90
70
50
30
10
-10
-30
-50
-70
-90
-110
-130
-150
-170
-190
-210
-230
-250
-270
-290
ppm

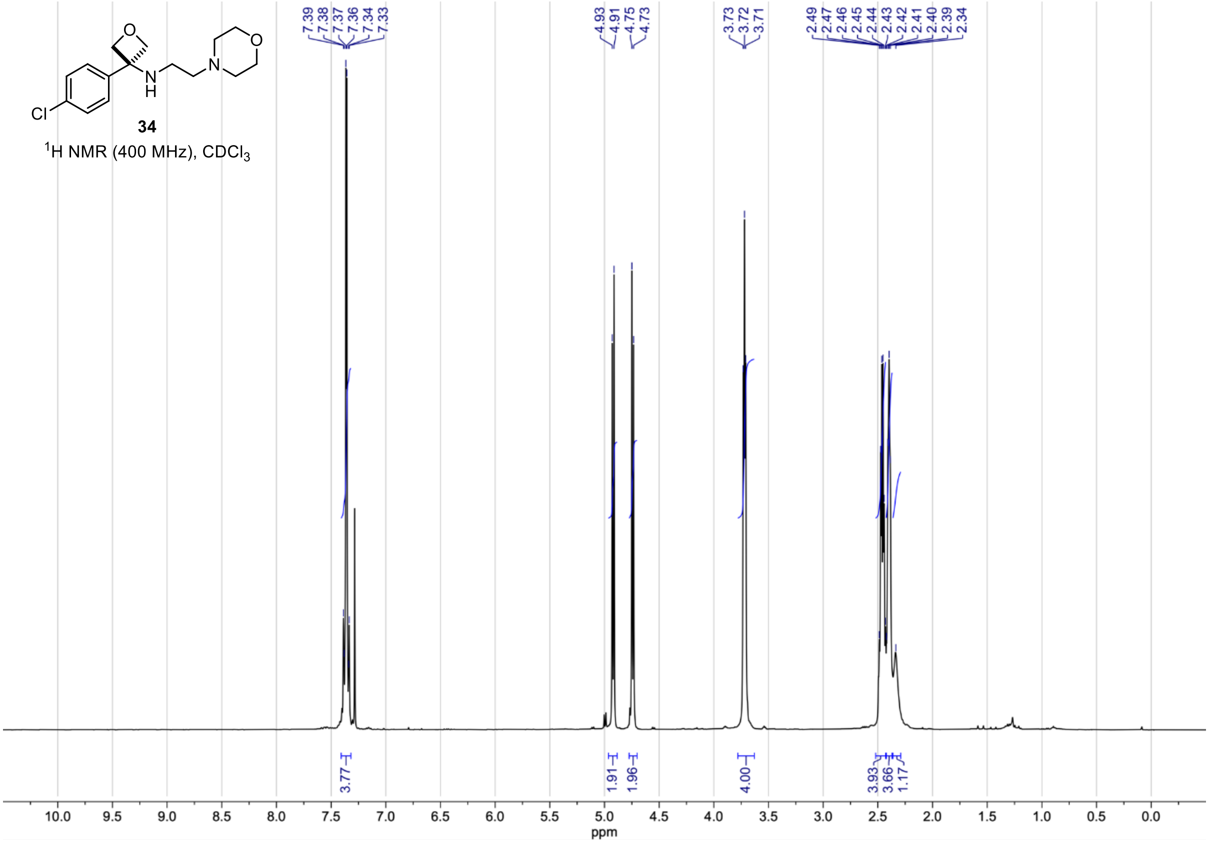

34

^{1}H NMR (400 MHz), $CDCl_3$

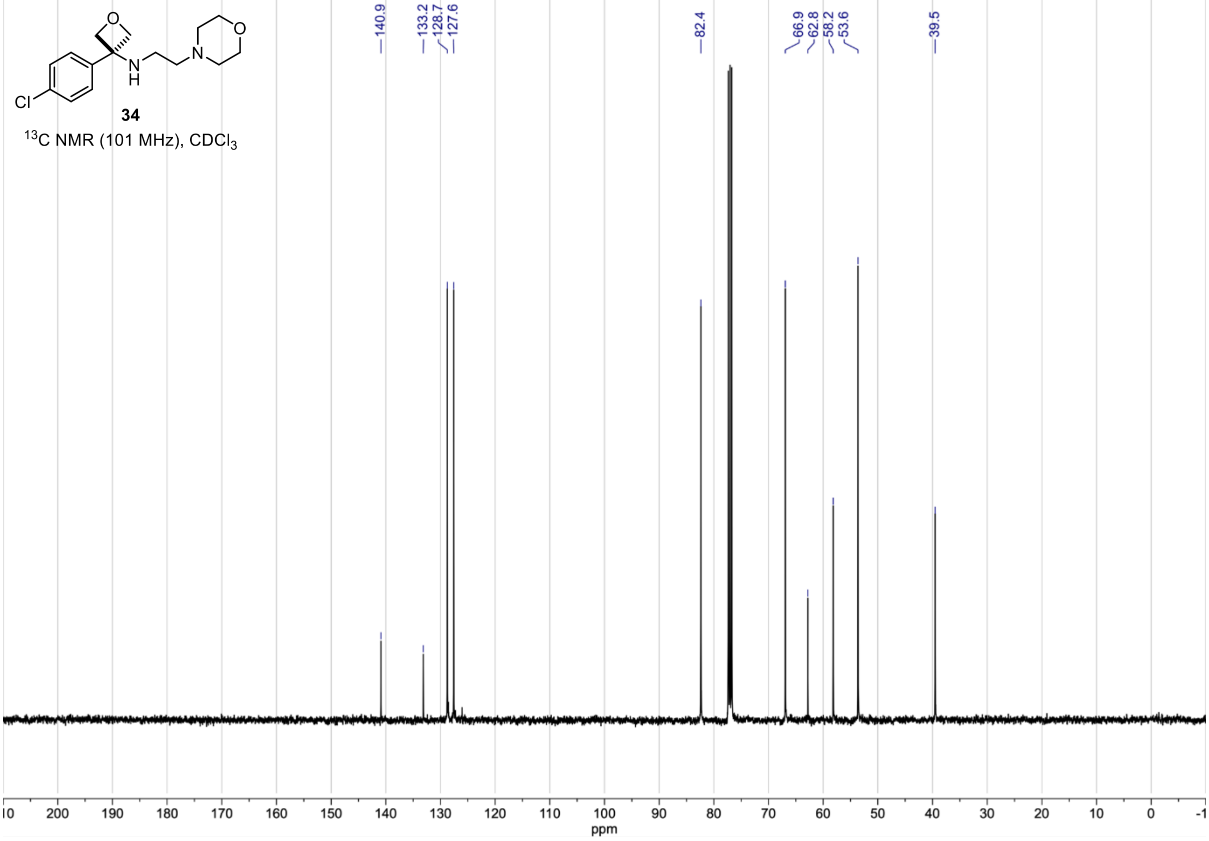

34

^{13}C NMR (101 MHz), $CDCl_3$

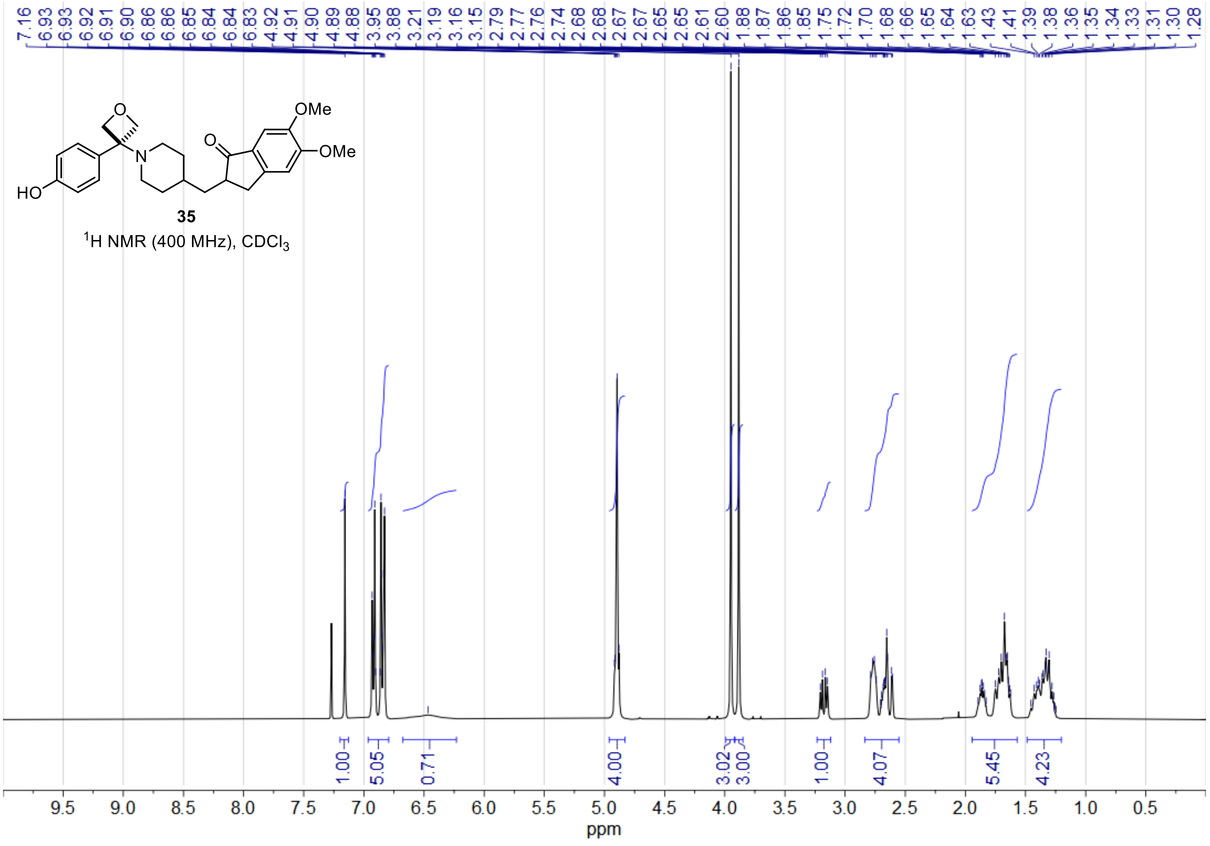
7.16 6.93 6.93 6.92 6.91 6.90 6.86 6.86 6.85 6.84 6.84 6.83 4.92 4.91 4.90 4.89 4.88 3.95 3.88 3.21 3.19 3.16 3.15 2.79 2.77 2.76 2.74 2.68 2.68 2.67 2.67 2.65 2.61 2.60 1.88 1.87 1.86 1.85 1.75 1.72 1.70 1.68 1.66 1.65 1.64 1.63 1.43 1.41 1.39 1.38 1.36 1.35 1.34 1.33 1.31 1.30 1.28
OMe
OMe
O
N
HO
35
^{1}H NMR (400 MHz), $CDCl_3$
1.00 5.05 0.71 4.00 3.02 3.00 1.00 4.07 5.45 4.23
9.5 9.0 8.5 8.0 7.5 7.0 6.5 6.0 5.5 5.0 4.5 4.0 3.5 3.0 2.5 2.0 1.5 1.0 0.5
ppm

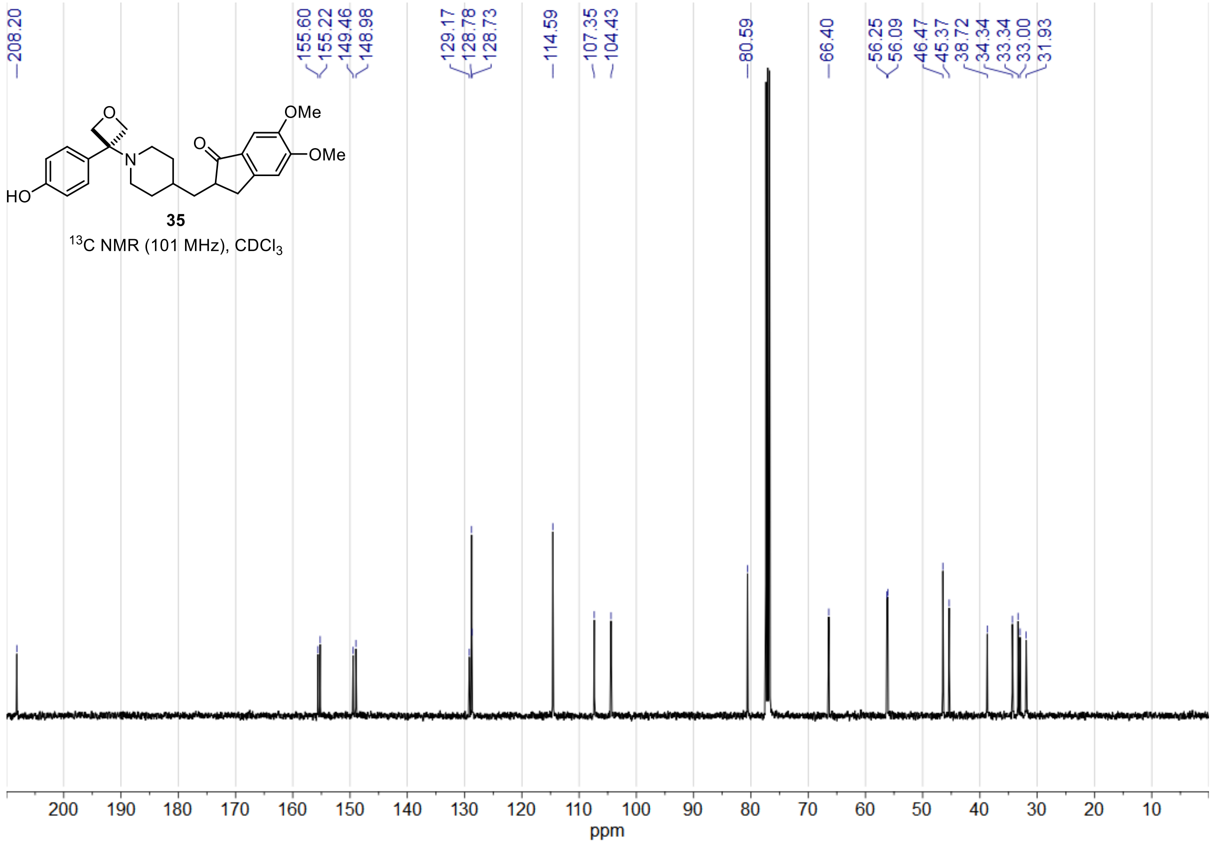
208.20 155.60 155.22 149.46 148.98 129.17 128.78 128.73 114.59 107.35 104.43 80.59 66.40 56.25 56.09 46.47 45.37 38.72 34.34 33.34 33.00 31.93
OMe
OMe
O
N
HO
35
^{13}C NMR (101 MHz), $CDCl_3$
200 190 180 170 160 150 140 130 120 110 100 90 80 70 60 50 40 30 20 10
ppm

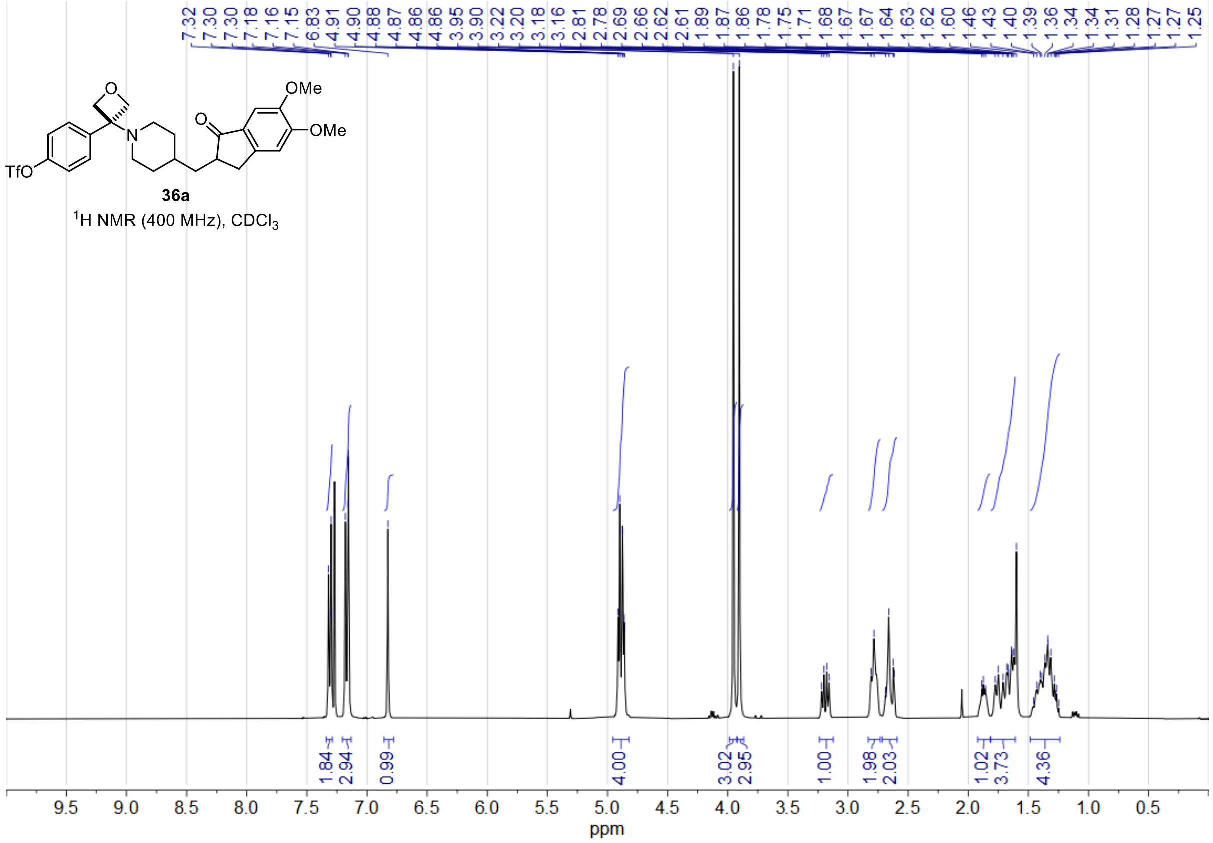

36a
^{1}H NMR (400 MHz), CDCl$_3$
7.32 7.30 7.30 7.18 7.16 7.15 6.83 4.91 4.90 4.88 4.87 4.86 4.86 3.95 3.90 3.22 3.20 3.18 3.16 2.81 2.78 2.69 2.66 2.62 2.61 1.89 1.87 1.86 1.78 1.75 1.71 1.68 1.67 1.67 1.64 1.63 1.62 1.60 1.46 1.43 1.40 1.39 1.36 1.34 1.31 1.28 1.27 1.27 1.25
1.84 2.94 0.99 4.00 3.02 2.95 1.00 1.98 2.03 1.02 3.73 4.36
ppm

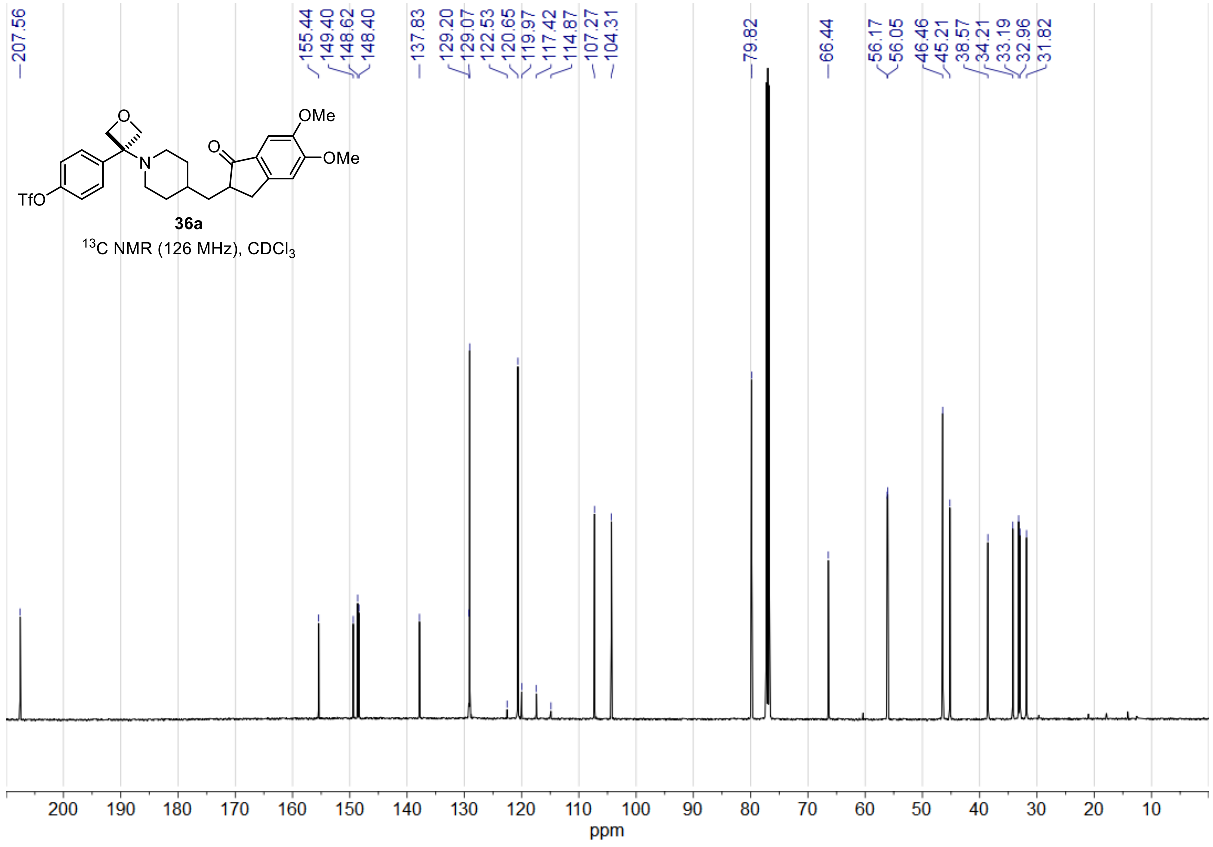

36a
^{13}C NMR (126 MHz), CDCl$_3$
207.56 155.44 149.40 148.62 148.40 137.83 129.20 129.07 122.53 120.65 119.97 117.42 114.87 107.27 104.31 79.82 66.44 56.17 56.05 46.46 45.21 38.57 34.21 33.19 32.96 31.82
ppm

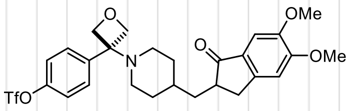

36a

$^{19}F\{^1H\}$ NMR (377 MHz), $CDCl_3$

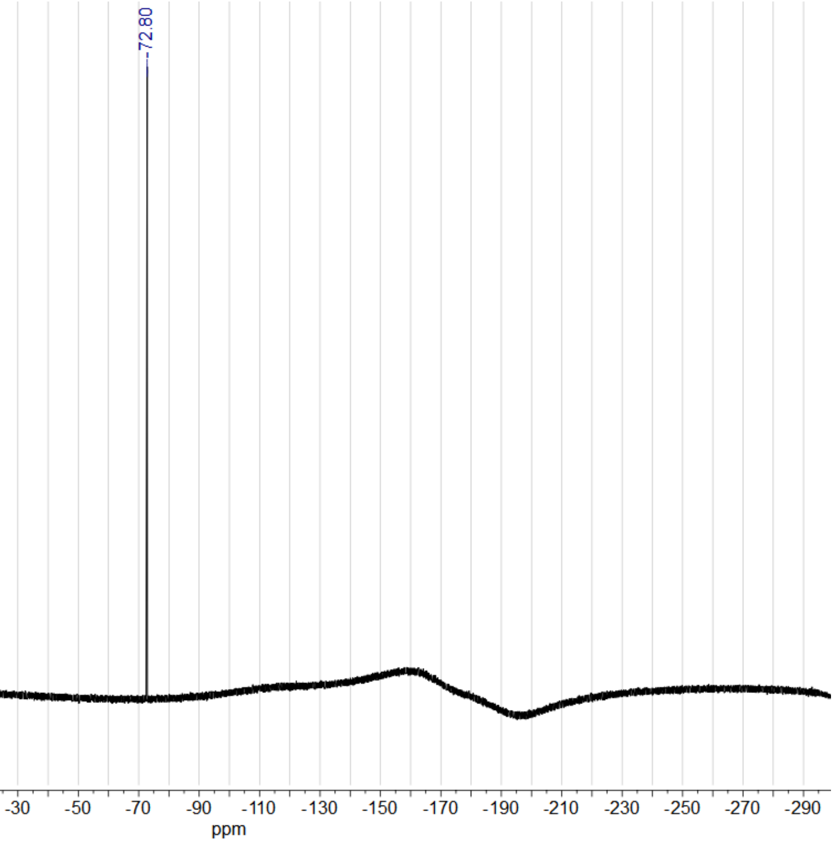

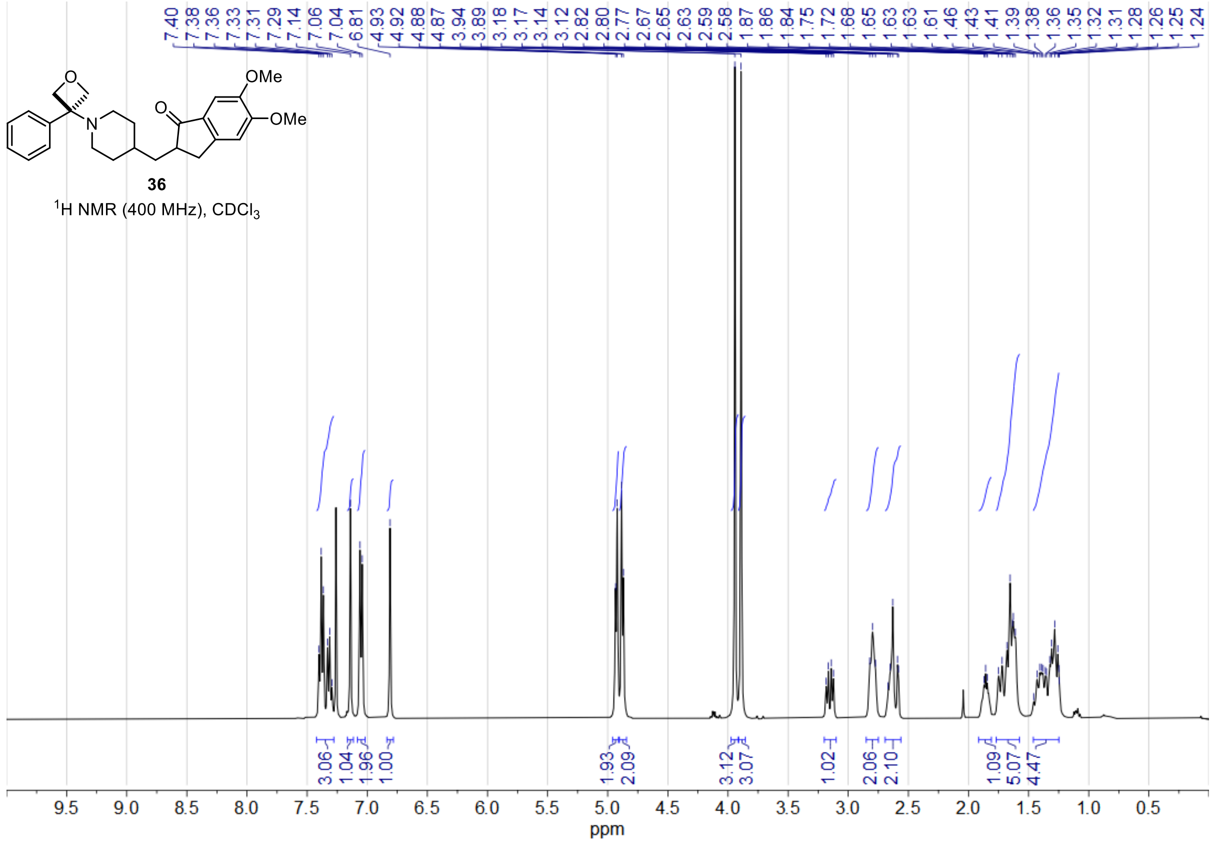

36

^{1}H NMR (400 MHz), $CDCl_3$

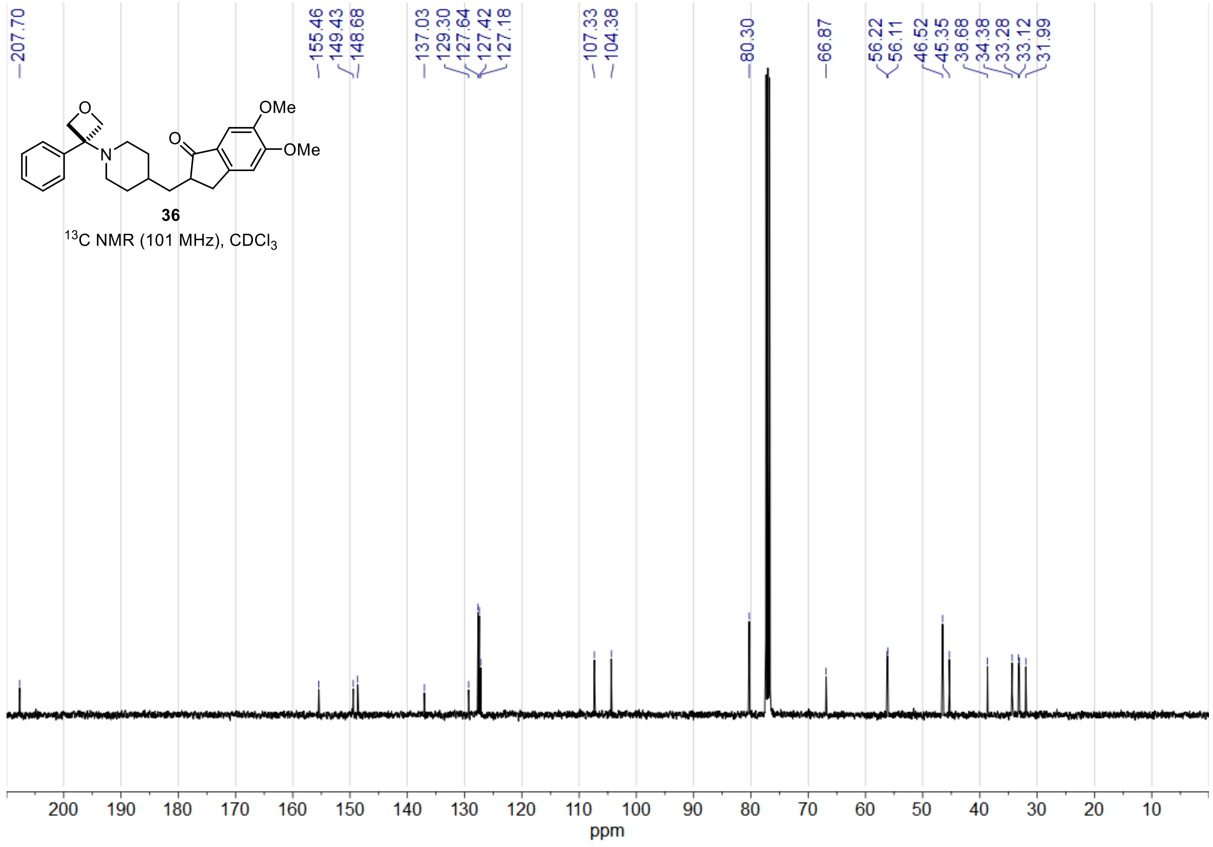

36

^{13}C NMR (101 MHz), $CDCl_3$

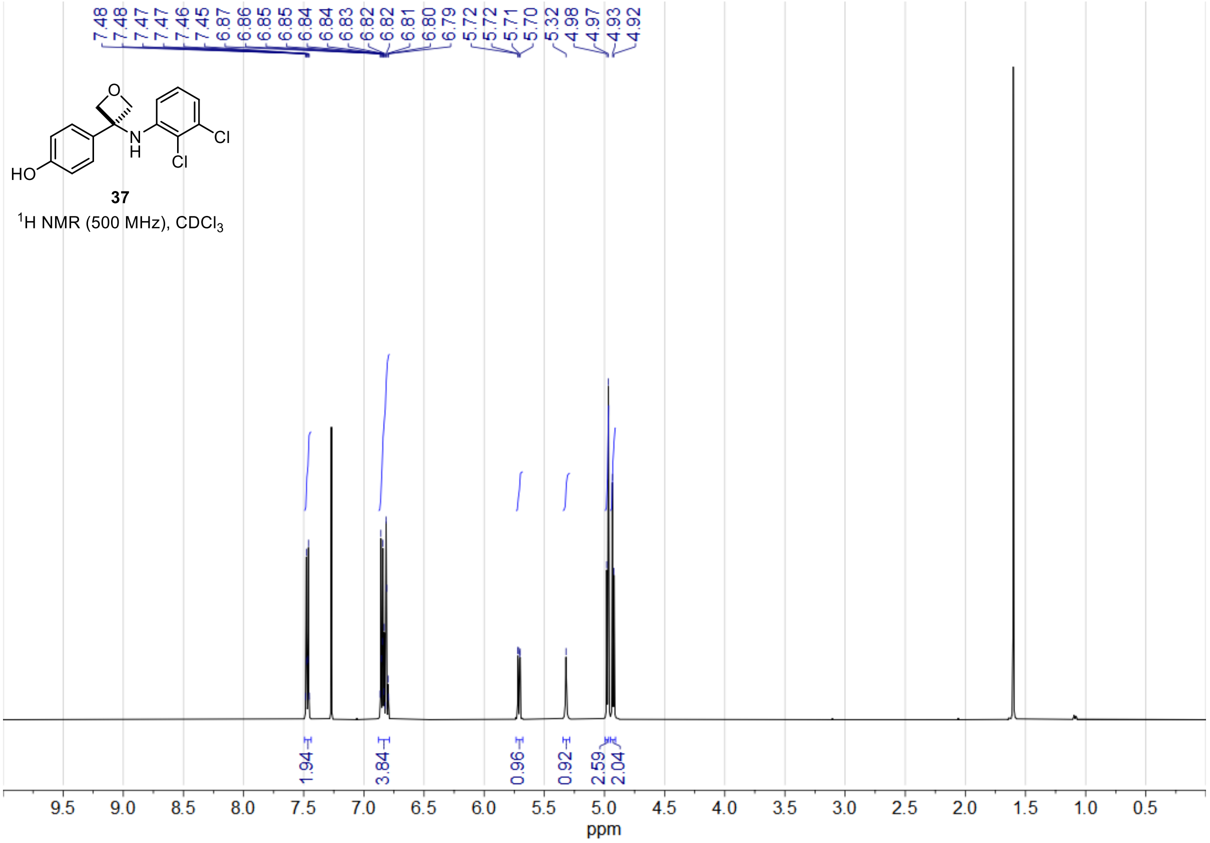
7.48 7.48 7.47 7.47 7.46 7.45 6.87 6.86 6.85 6.85 6.84 6.84 6.83 6.82 6.82 6.81 6.80 6.79 5.72 5.72 5.71 5.70 5.32 4.98 4.97 4.93 4.92
HO
N
H
O
Cl
Cl
37
1H NMR (500 MHz), CDCl3
1.94
3.84
0.96
0.92
2.59
2.04
9.5 9.0 8.5 8.0 7.5 7.0 6.5 6.0 5.5 5.0 4.5 4.0 3.5 3.0 2.5 2.0 1.5 1.0 0.5
ppm

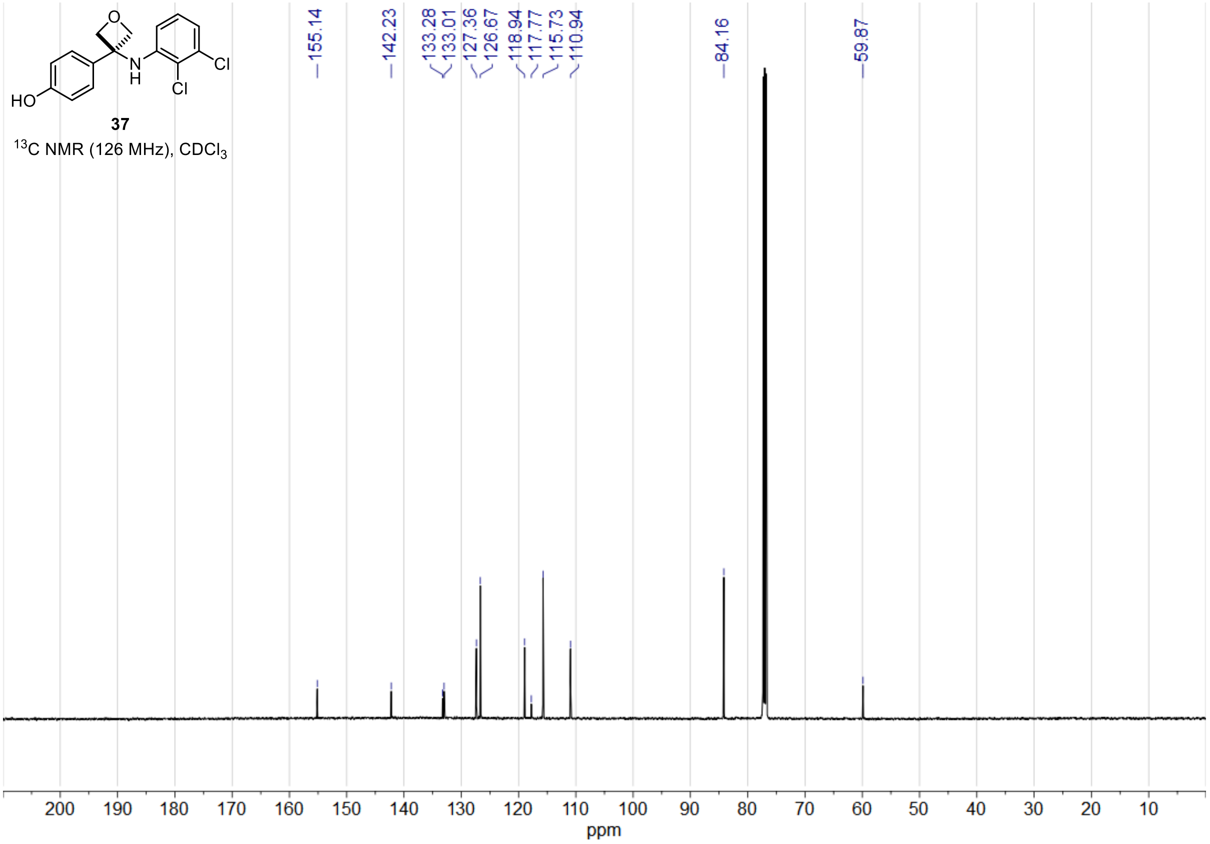
155.14
142.23
133.28 133.01 127.36 126.67 118.94 117.77 115.73 110.94
84.16
59.87
HO
N
H
O
Cl
Cl
37
13C NMR (126 MHz), CDCl3
200 190 180 170 160 150 140 130 120 110 100 90 80 70 60 50 40 30 20 10
ppm

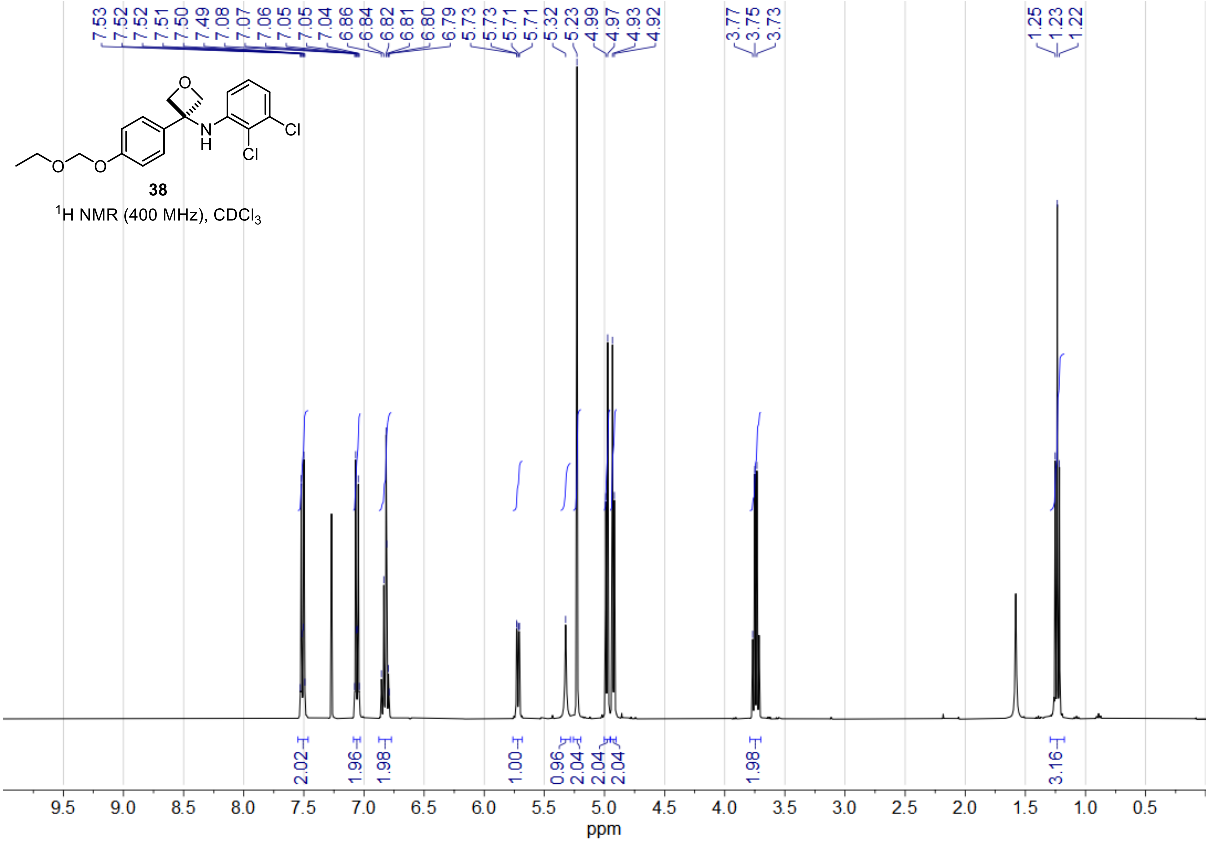
7.53
7.52
7.52
7.51
7.50
7.49
7.08
7.07
7.06
7.05
7.05
7.04
6.86
6.84
6.82
6.81
6.80
6.79
5.73
5.73
5.71
5.71
5.32
5.23
4.99
4.97
4.93
4.92
3.77
3.75
3.73
1.25
1.23
1.22
Cl
Cl
N
H
O
O
O
38
^{1}H NMR (400 MHz), CDCl$_3$
2.02
1.96
1.98
1.00
0.96
2.04
2.04
2.04
1.98
3.16
9.5
9.0
8.5
8.0
7.5
7.0
6.5
6.0
5.5
5.0
4.5
4.0
3.5
3.0
2.5
2.0
1.5
1.0
0.5
ppm

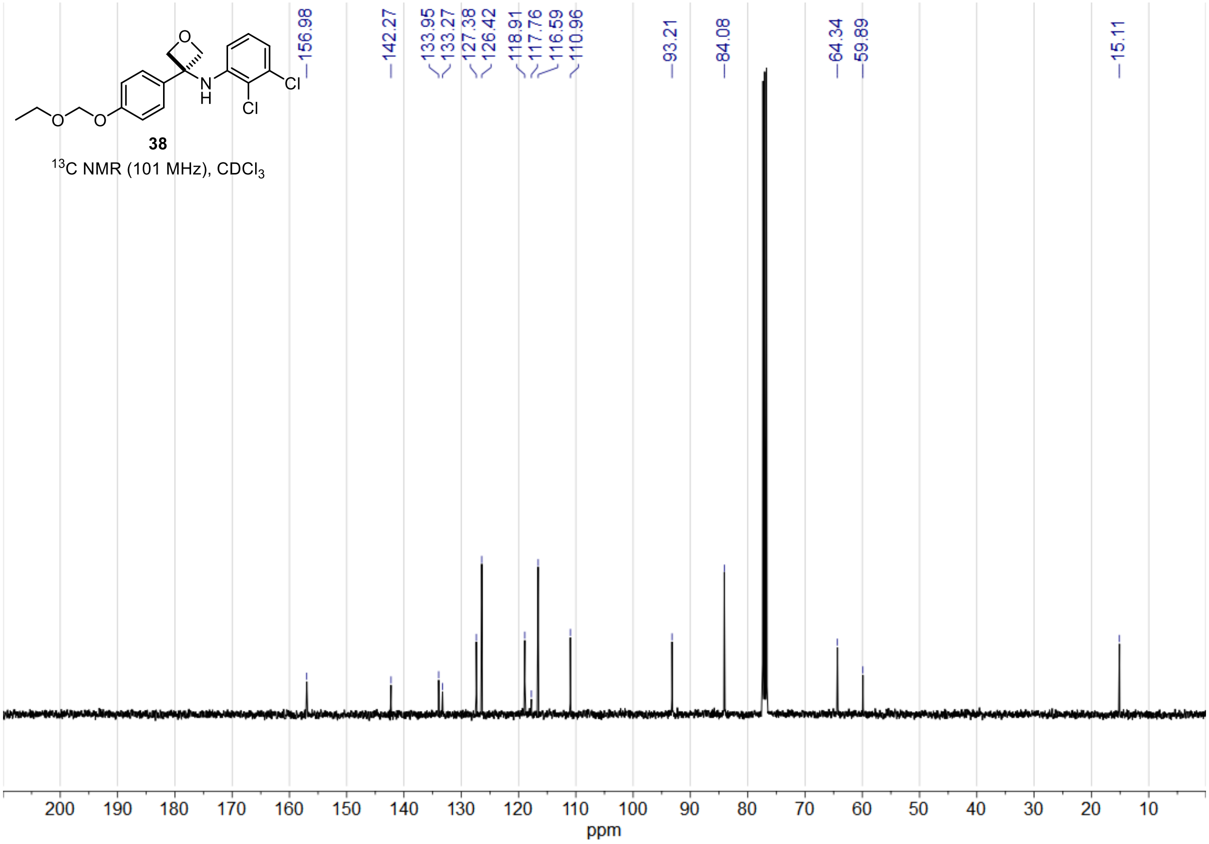
156.98
142.27
133.95
133.27
127.38
126.42
118.91
117.76
116.59
110.96
93.21
84.08
64.34
59.89
15.11
Cl
Cl
N
H
O
O
O
38
^{13}C NMR (101 MHz), CDCl$_3$
200
190
180
170
160
150
140
130
120
110
100
90
80
70
60
50
40
30
20
10
ppm

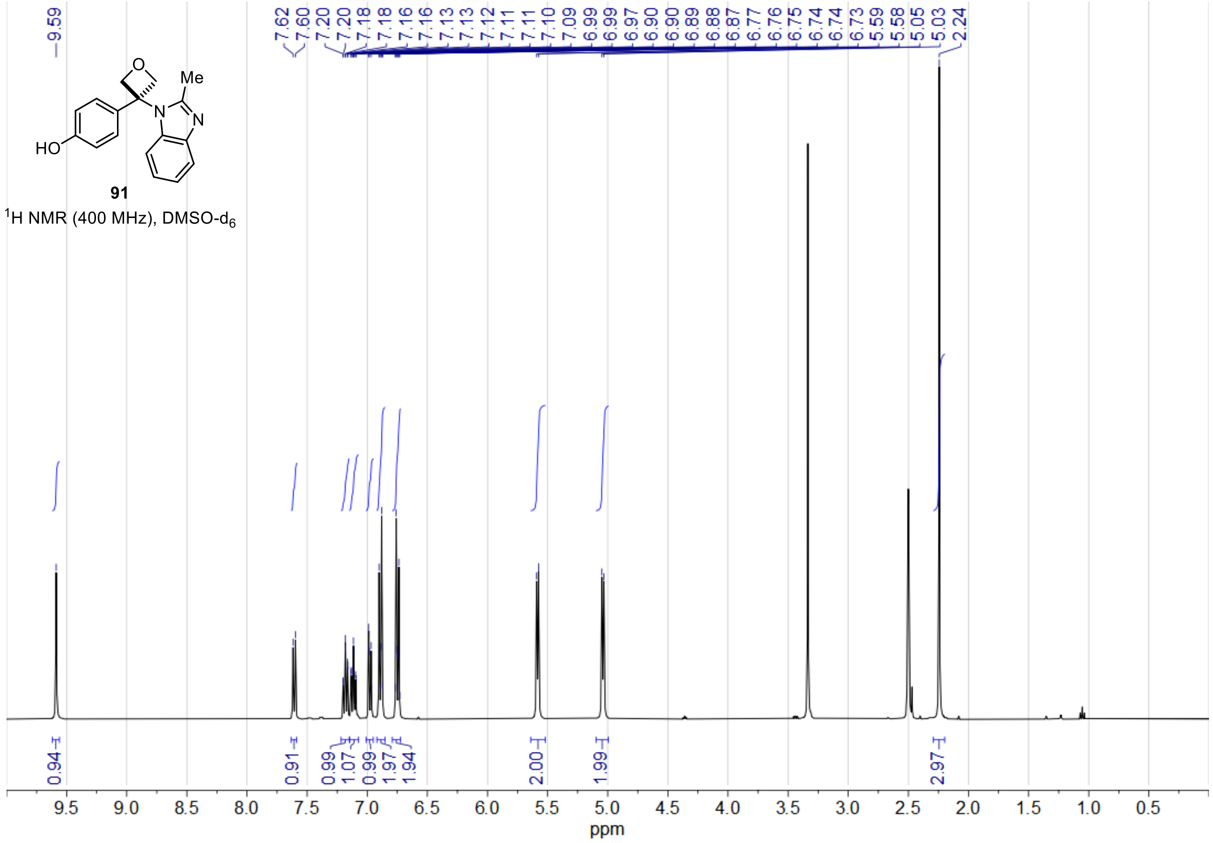

91

^{1}H NMR (400 MHz), DMSO-d_6

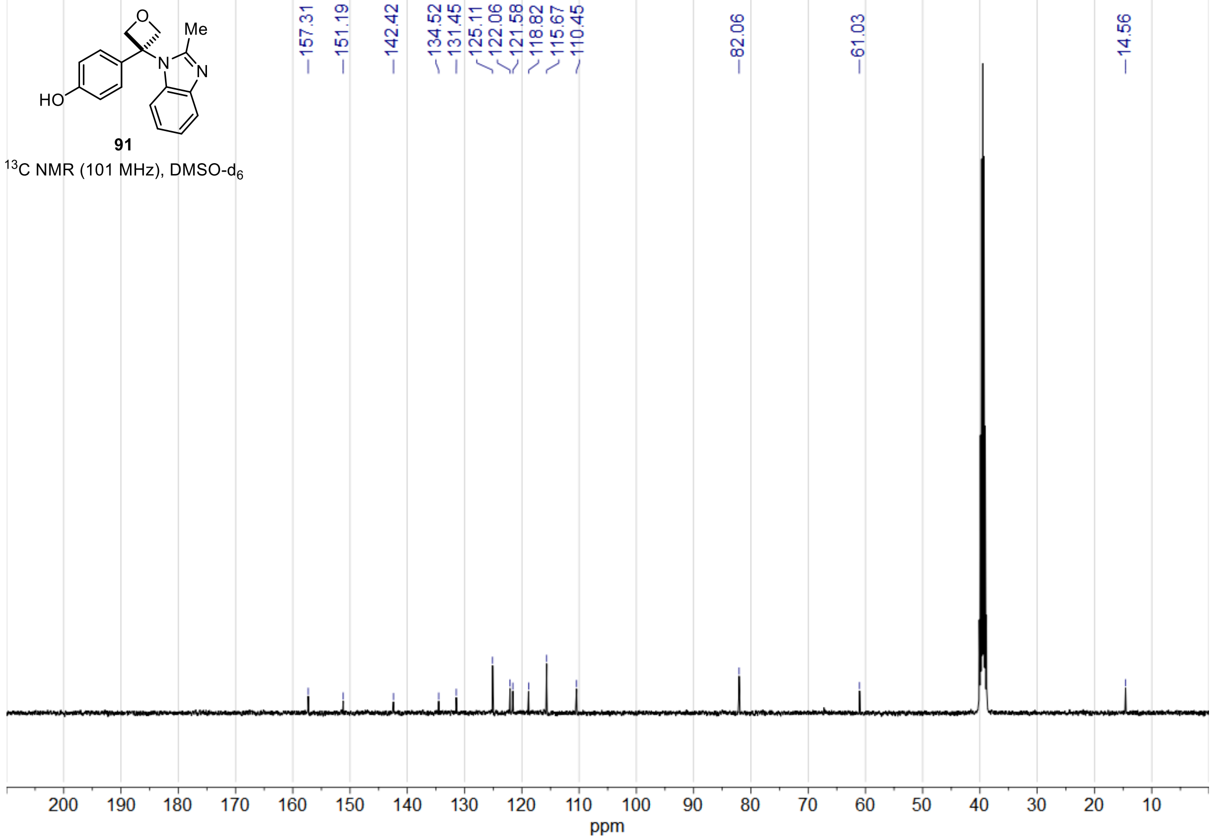

91

^{13}C NMR (101 MHz), DMSO-d_6

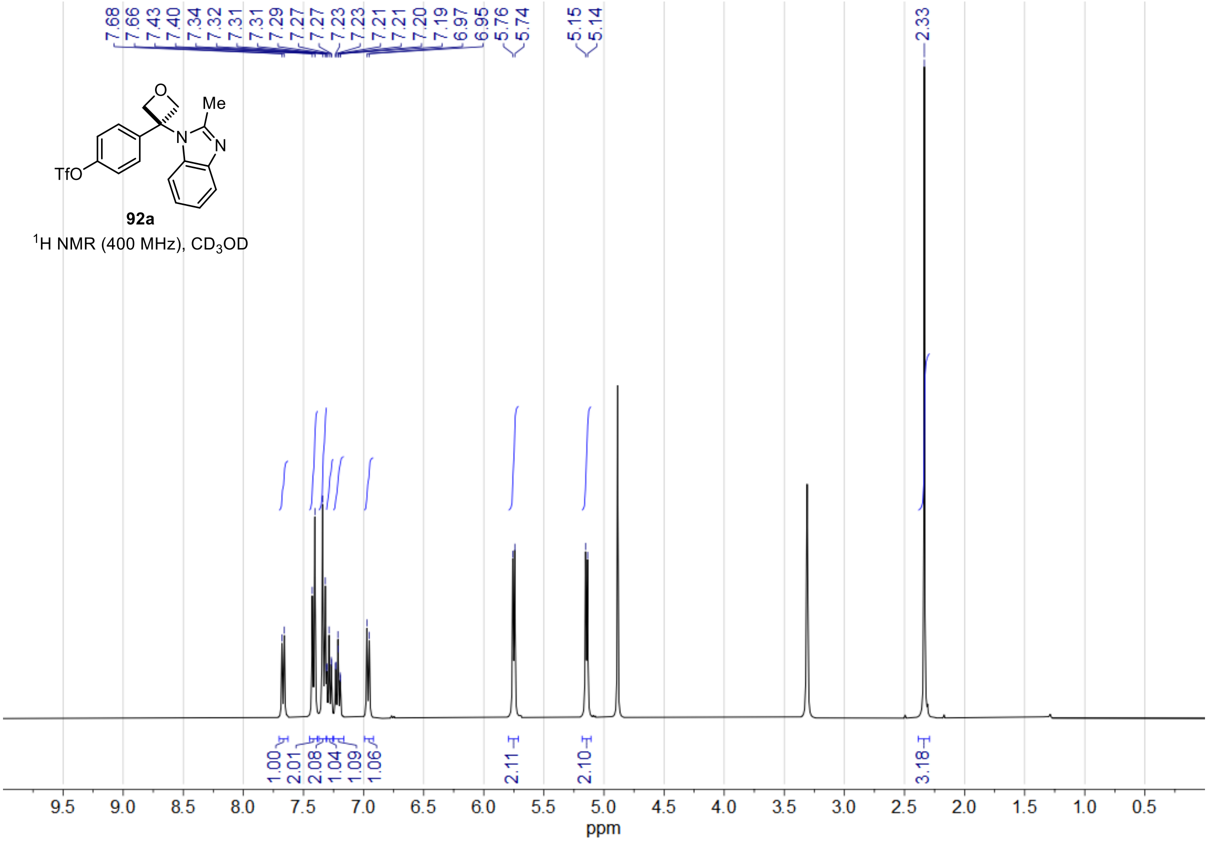
7.68
7.66
7.43
7.40
7.34
7.32
7.31
7.31
7.29
7.27
7.27
7.23
7.23
7.21
7.21
7.20
7.19
6.97
6.95
5.76
5.74
5.15
5.14
2.33
Me
O
N
N
TfO
92a
^{1}H NMR (400 MHz), CD_3OD
1.00
2.01
2.08
1.04
1.09
1.06
2.11
2.10
3.18
ppm

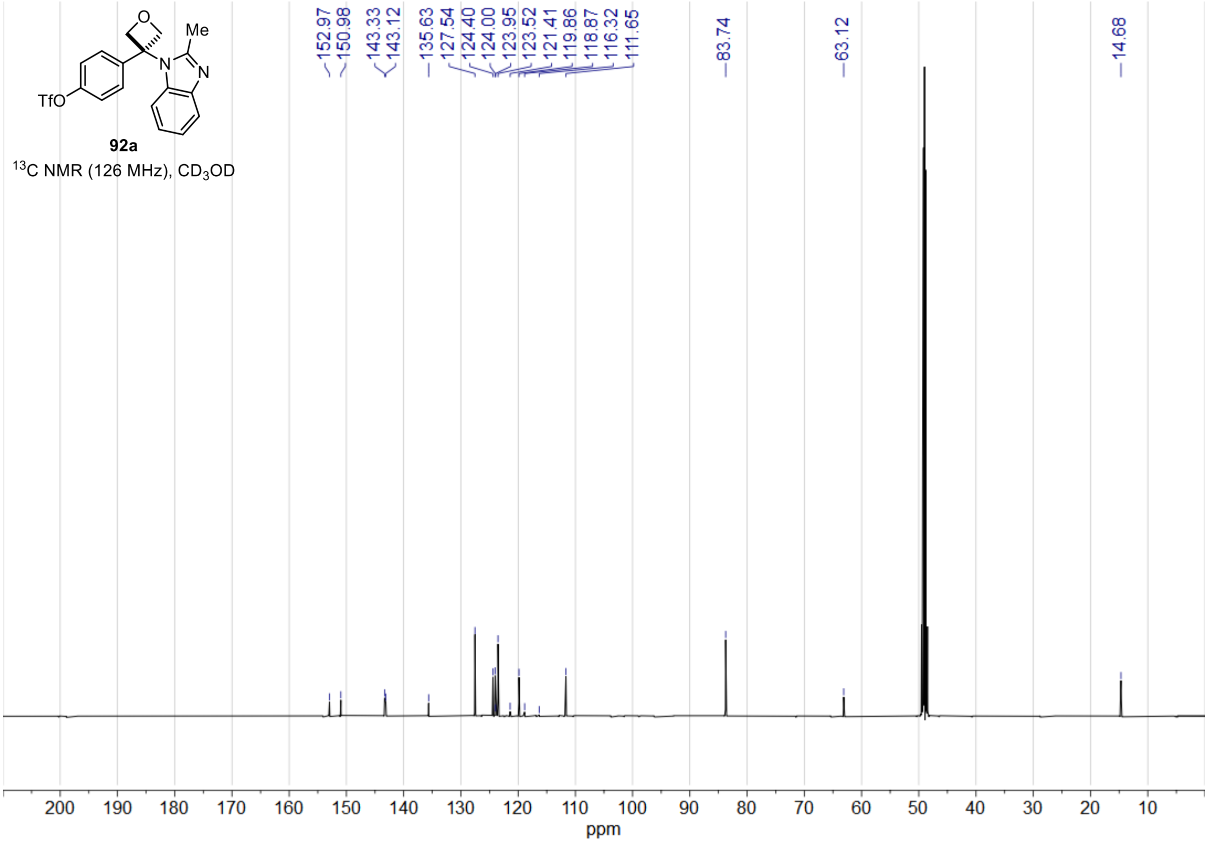
152.97
150.98
143.33
143.12
135.63
127.54
124.40
124.00
123.95
123.52
121.41
119.86
118.87
116.32
111.65
83.74
63.12
14.68
Me
O
N
N
TfO
92a
^{13}C NMR (126 MHz), CD_3OD
ppm

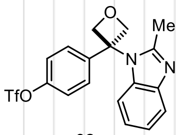

92a

$^{19}F\{^1H\}$ NMR (377 MHz), CD_3OD

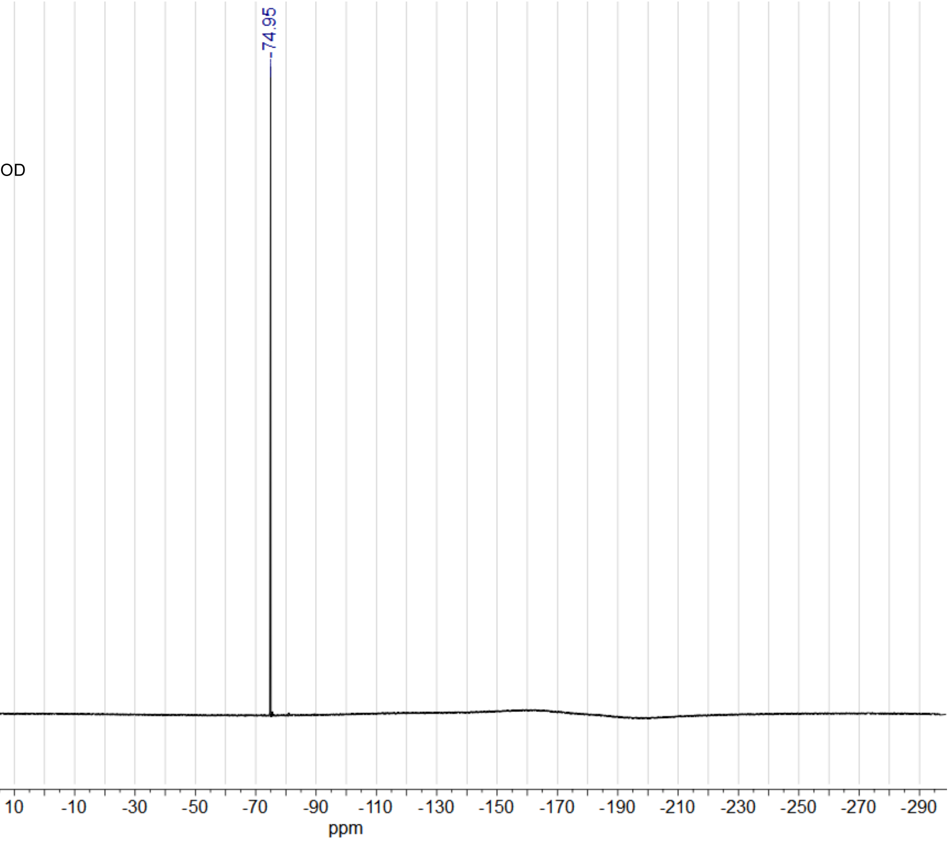

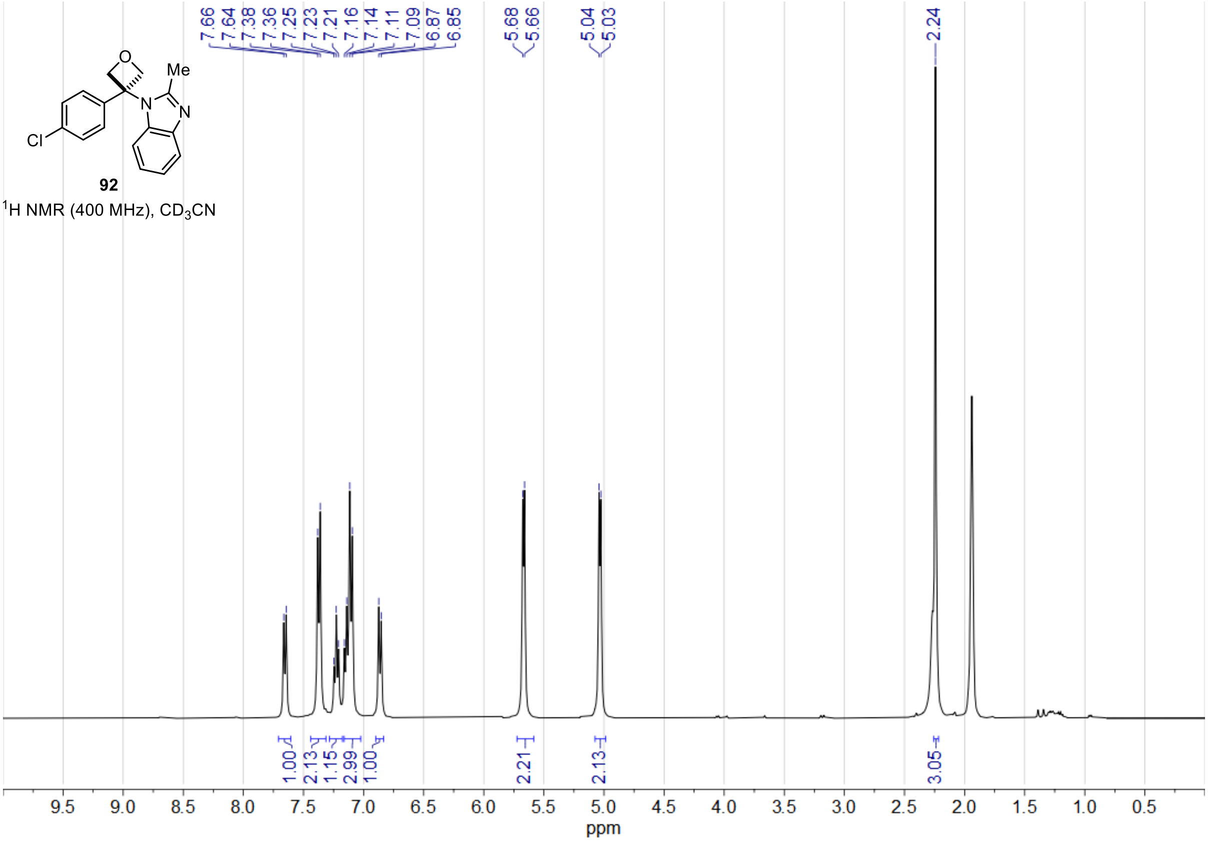

92

^1H NMR (400 MHz), CD_3CN

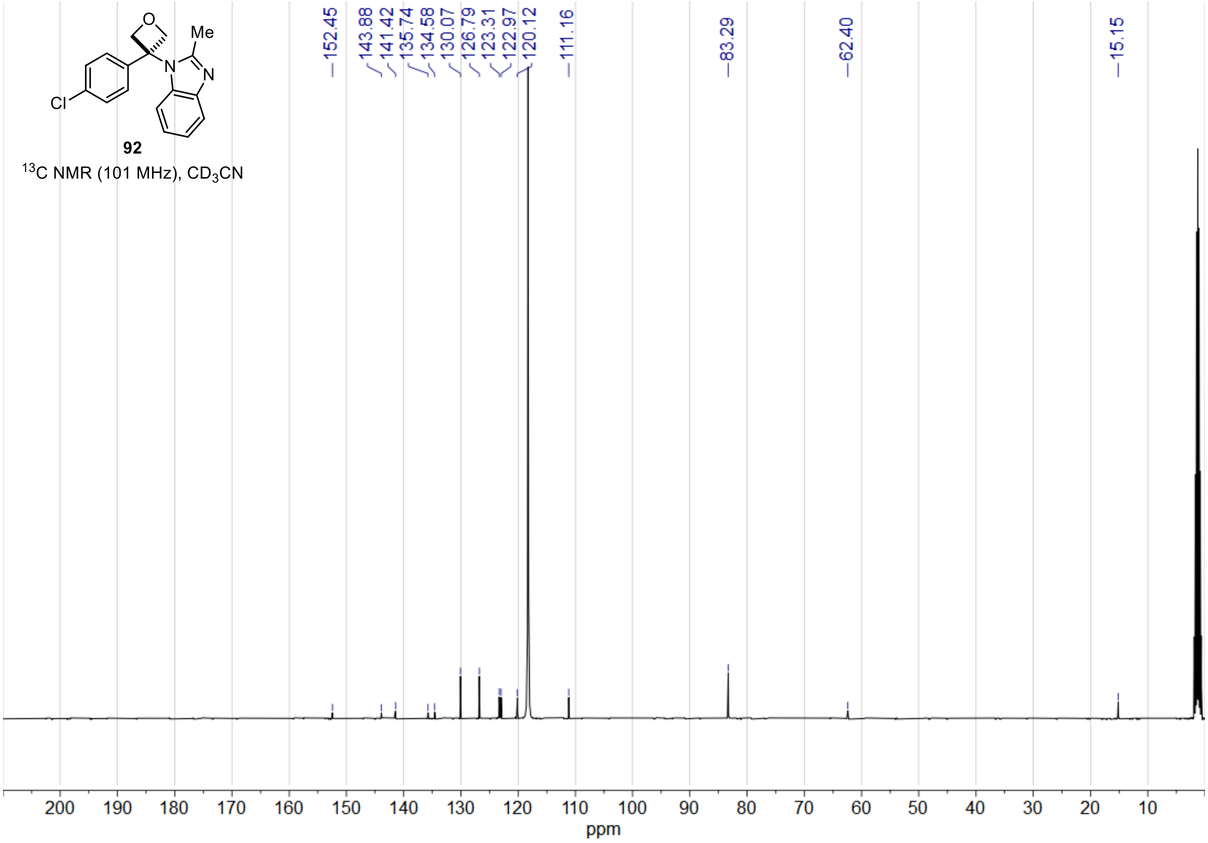

92

^{13}C NMR (101 MHz), CD_3CN

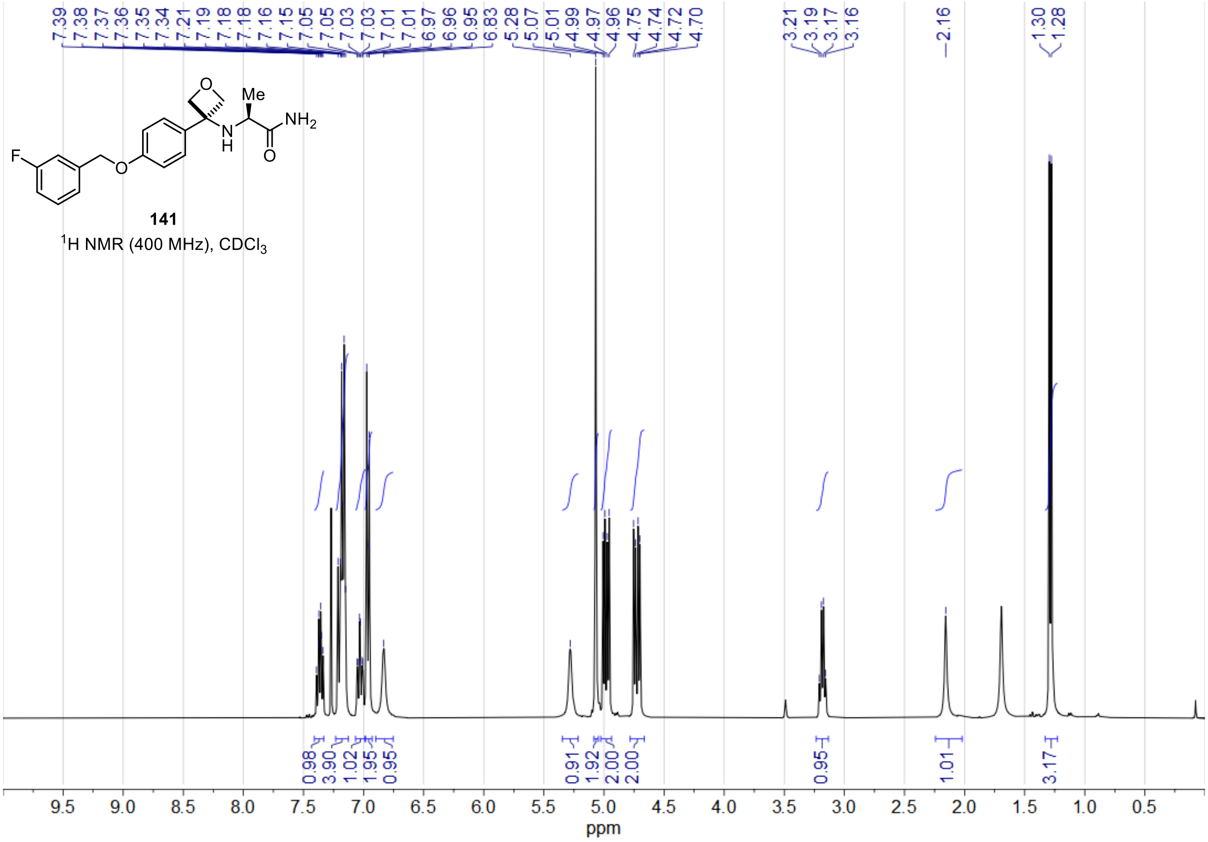
7.39 7.38 7.37 7.36 7.35 7.34 7.21 7.19 7.18 7.16 7.15 7.05 7.03 7.03 7.01 6.97 6.96 6.95 6.83 5.28 5.07 5.01 4.99 4.97 4.96 4.75 4.74 4.72 4.70 3.21 3.19 3.17 3.16 2.16 1.30 1.28
Me
NH2
O
F
N
H
141
^{1}H NMR (400 MHz), CDCl$_3$
0.98 3.90 1.02 1.95 0.95 0.91 1.92 2.00 2.00 0.95 1.01 3.17
ppm

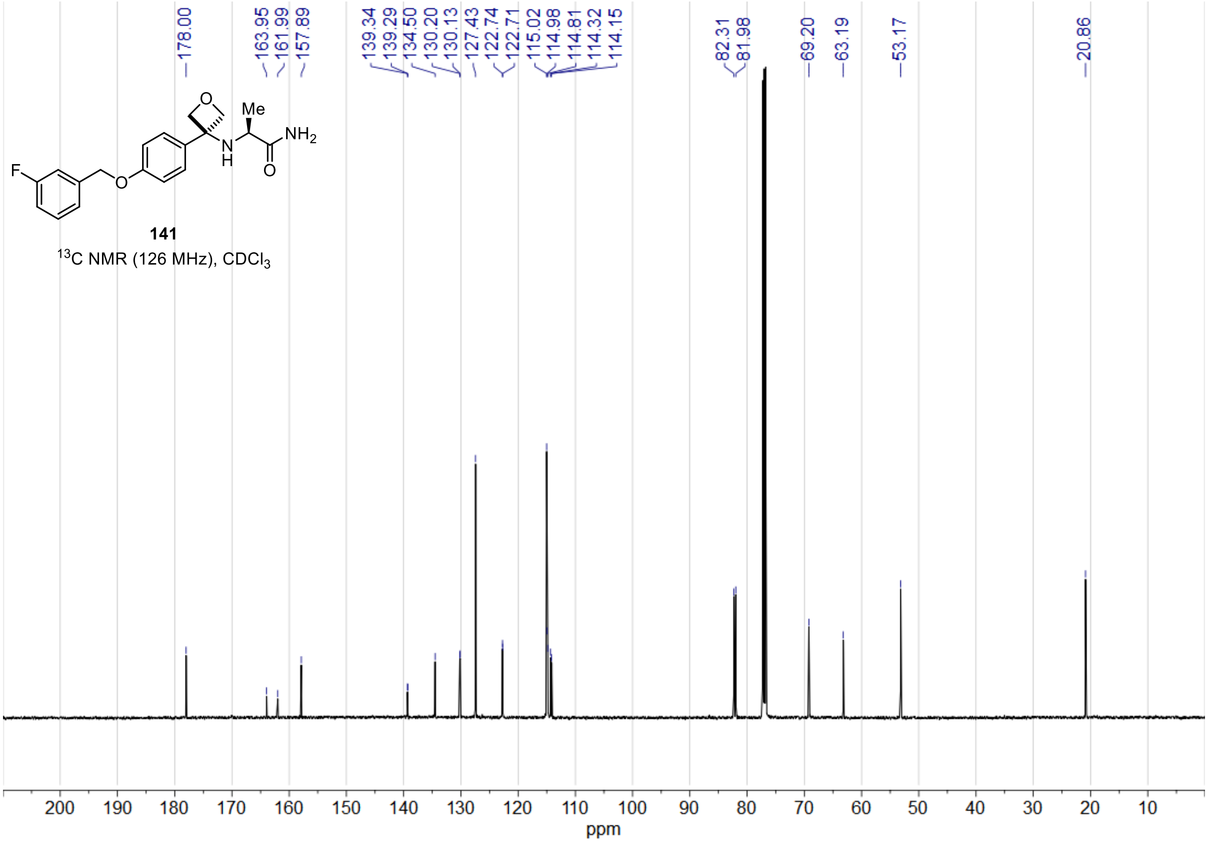
178.00 163.95 161.99 157.89 139.34 139.29 134.50 130.20 130.13 127.43 122.74 122.71 115.02 114.98 114.81 114.32 114.15 82.31 81.98 69.20 63.19 53.17 20.86
Me
NH2
O
F
N
H
141
^{13}C NMR (126 MHz), CDCl$_3$
ppm

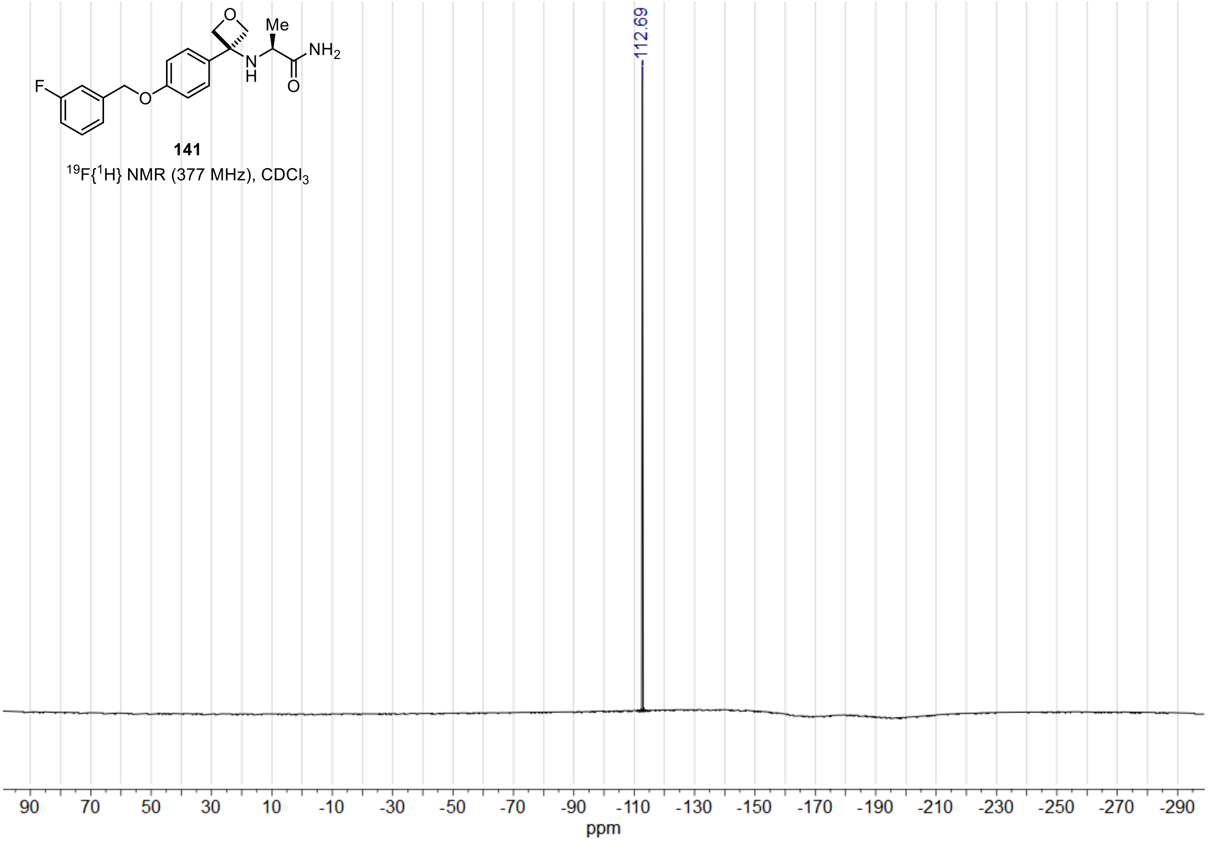
F
O
O
Me
N
H
NH2
O
141
19F{1H} NMR (377 MHz), CDCl3
-112.69
90 70 50 30 10 -10 -30 -50 -70 -90 -110 -130 -150 -170 -190 -210 -230 -250 -270 -290
ppm

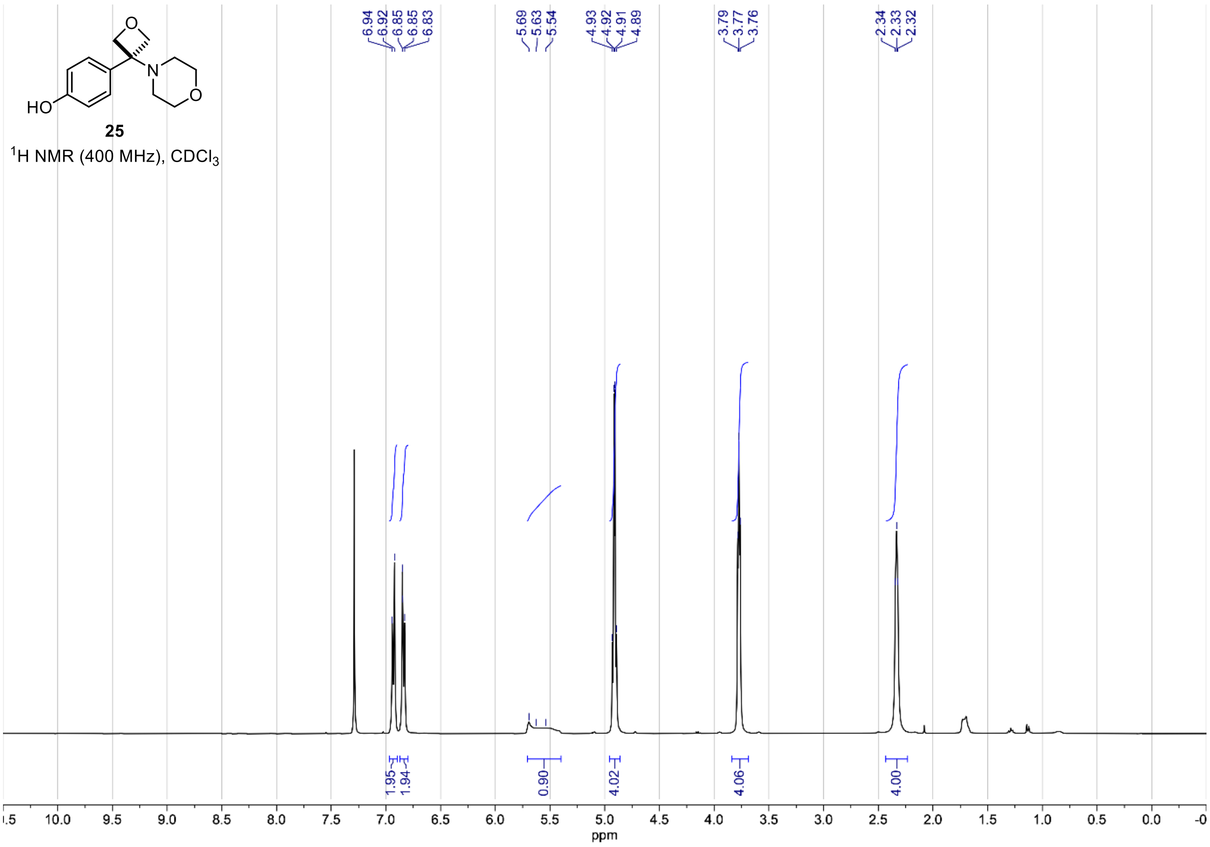

25

^{1}H NMR (400 MHz), $CDCl_3$

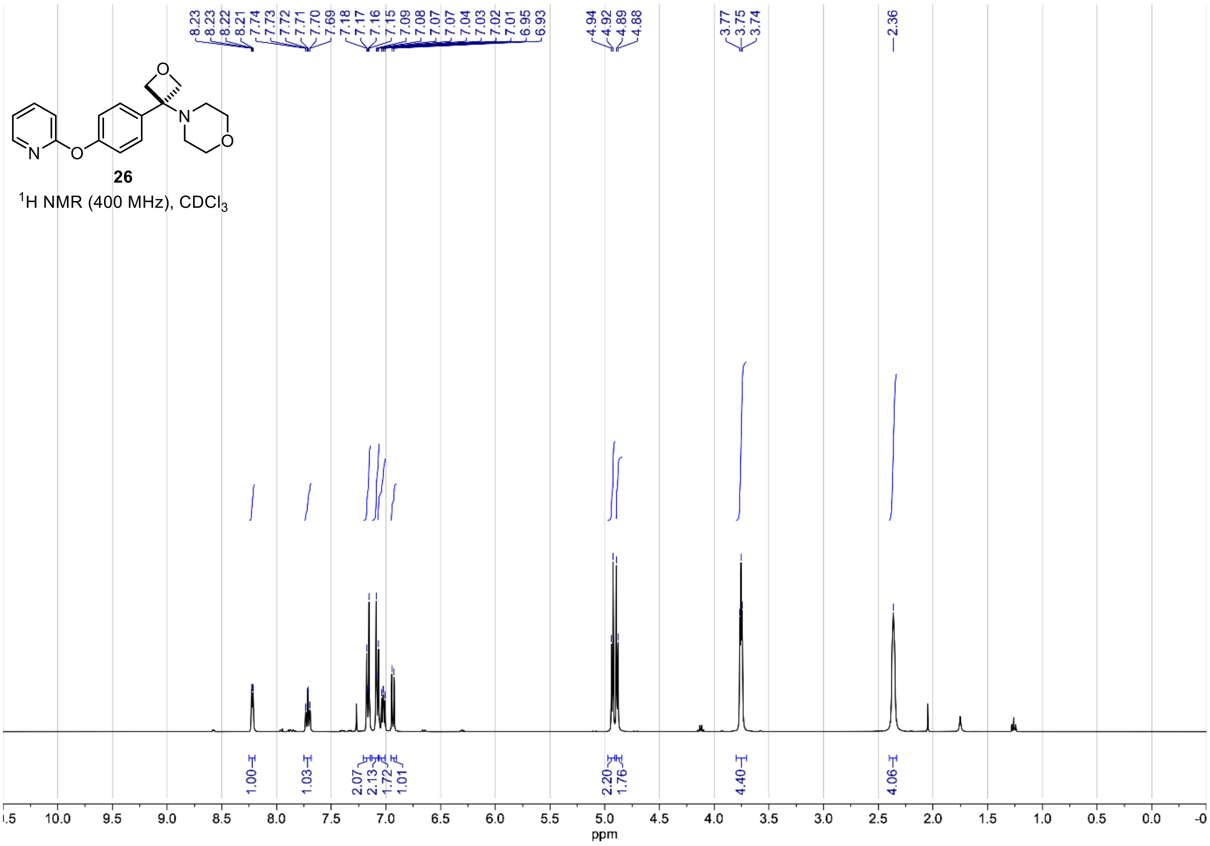
26
^{1}H NMR (400 MHz), $CDCl_3$
ppm

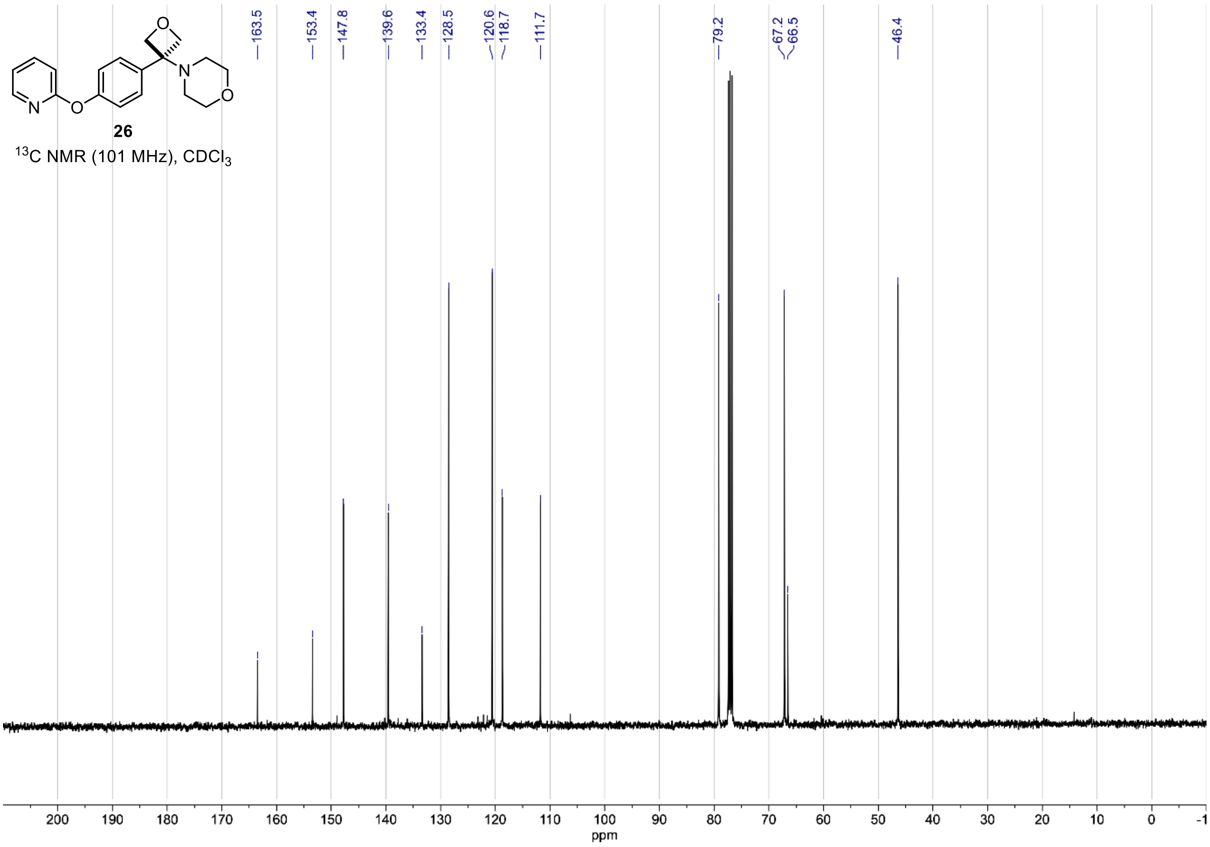
26
^{13}C NMR (101 MHz), $CDCl_3$
ppm

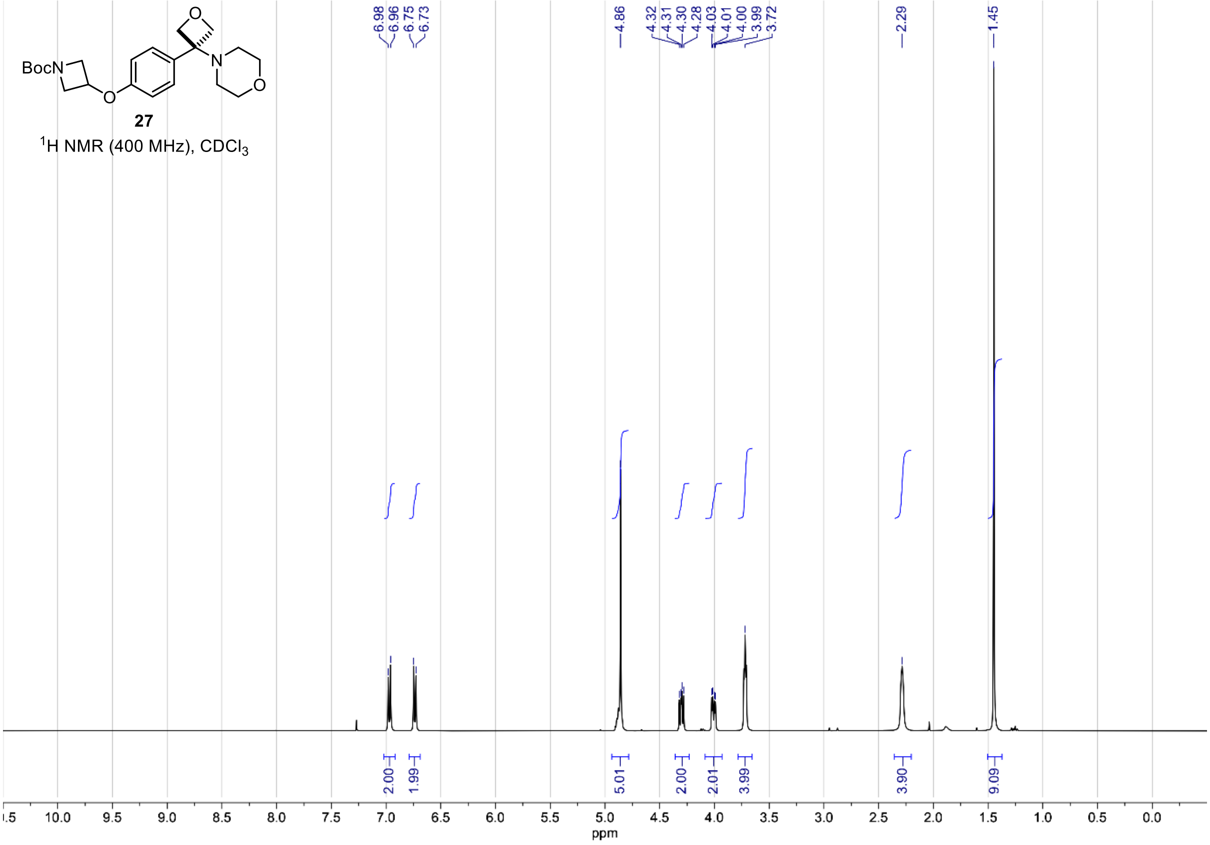
BocN
O
N
O
O
27
1H NMR (400 MHz), CDCl3
6.98
6.96
6.75
6.73
4.86
4.32
4.31
4.30
4.28
4.03
4.01
4.00
3.99
3.72
2.29
1.45
2.00
1.99
5.01
2.00
2.01
3.99
3.90
9.09
ppm

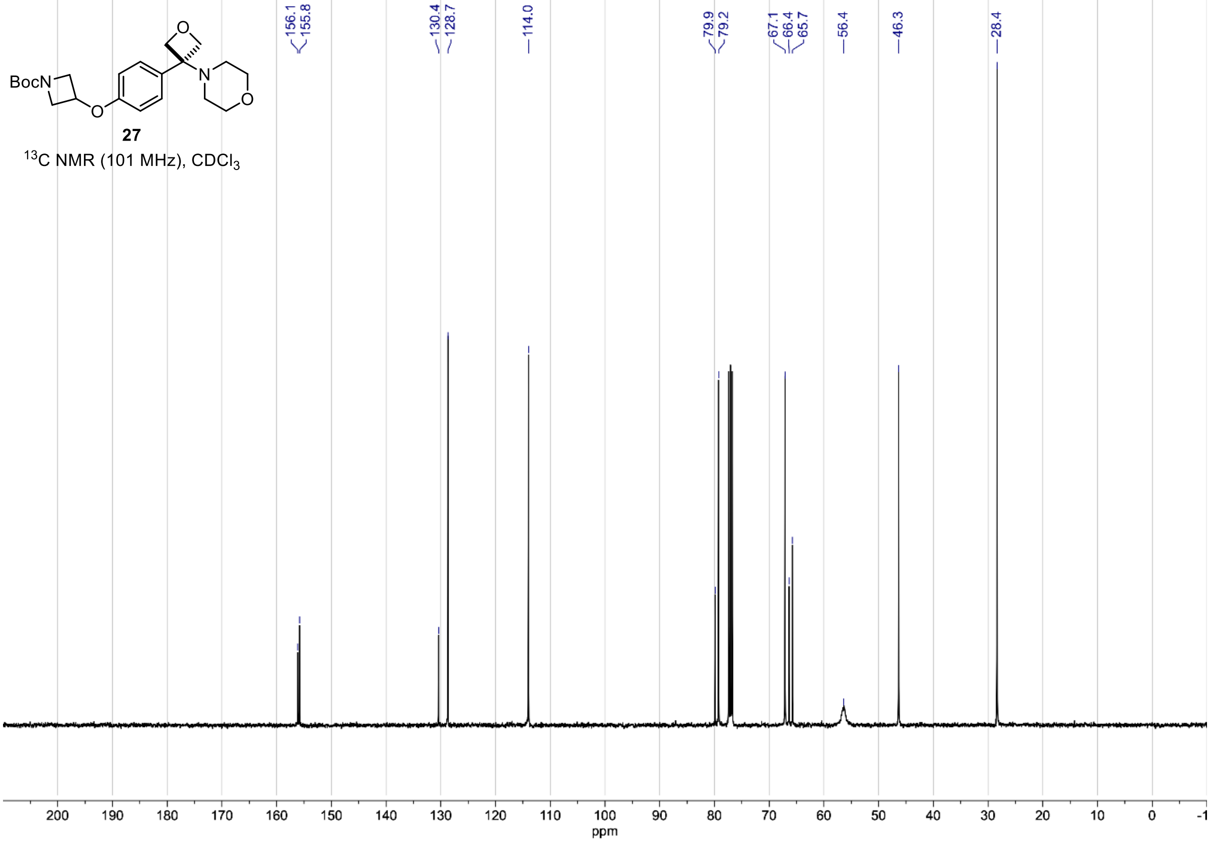
BocN
O
N
O
O
27
13C NMR (101 MHz), CDCl3
156.1
155.8
130.4
128.7
114.0
79.9
79.2
67.1
66.4
65.7
56.4
46.3
28.4
ppm

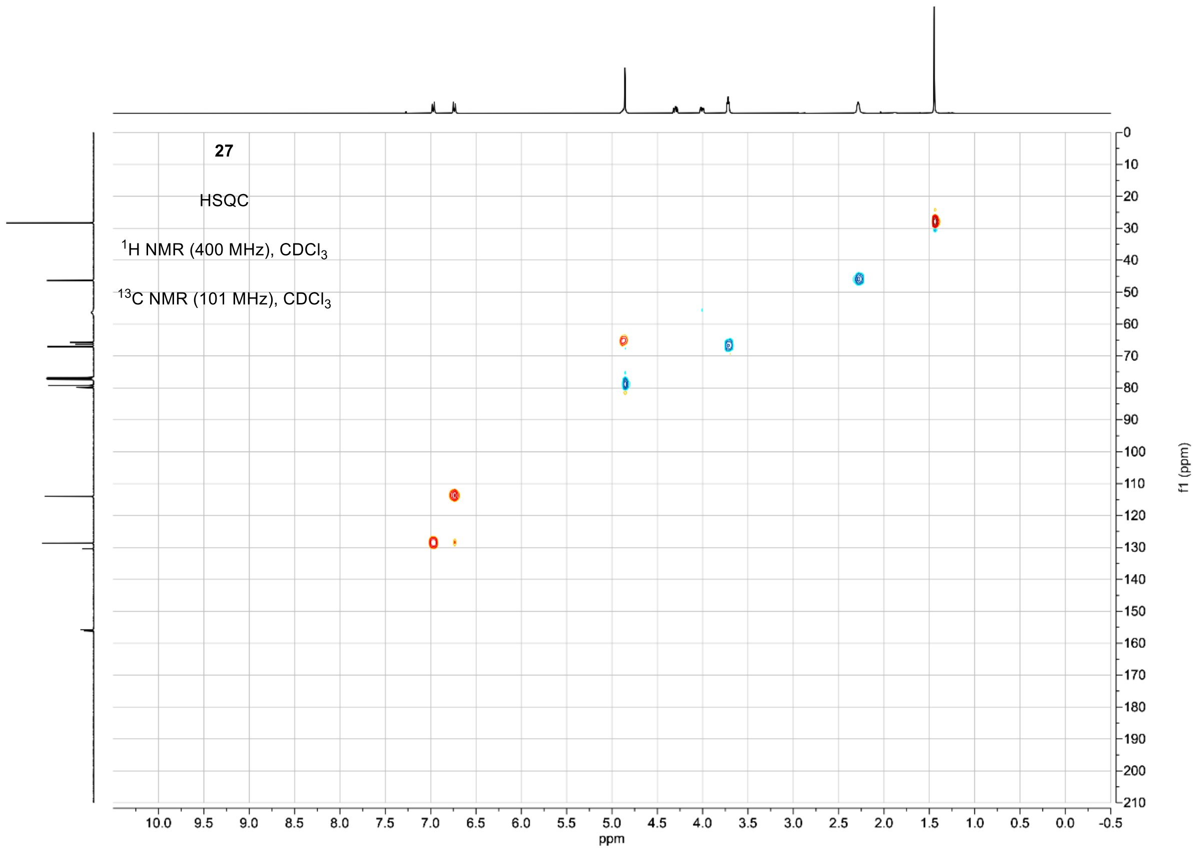
27
HSQC
1H NMR (400 MHz), CDCl3
13C NMR (101 MHz), CDCl3
ppm
f1 (ppm)

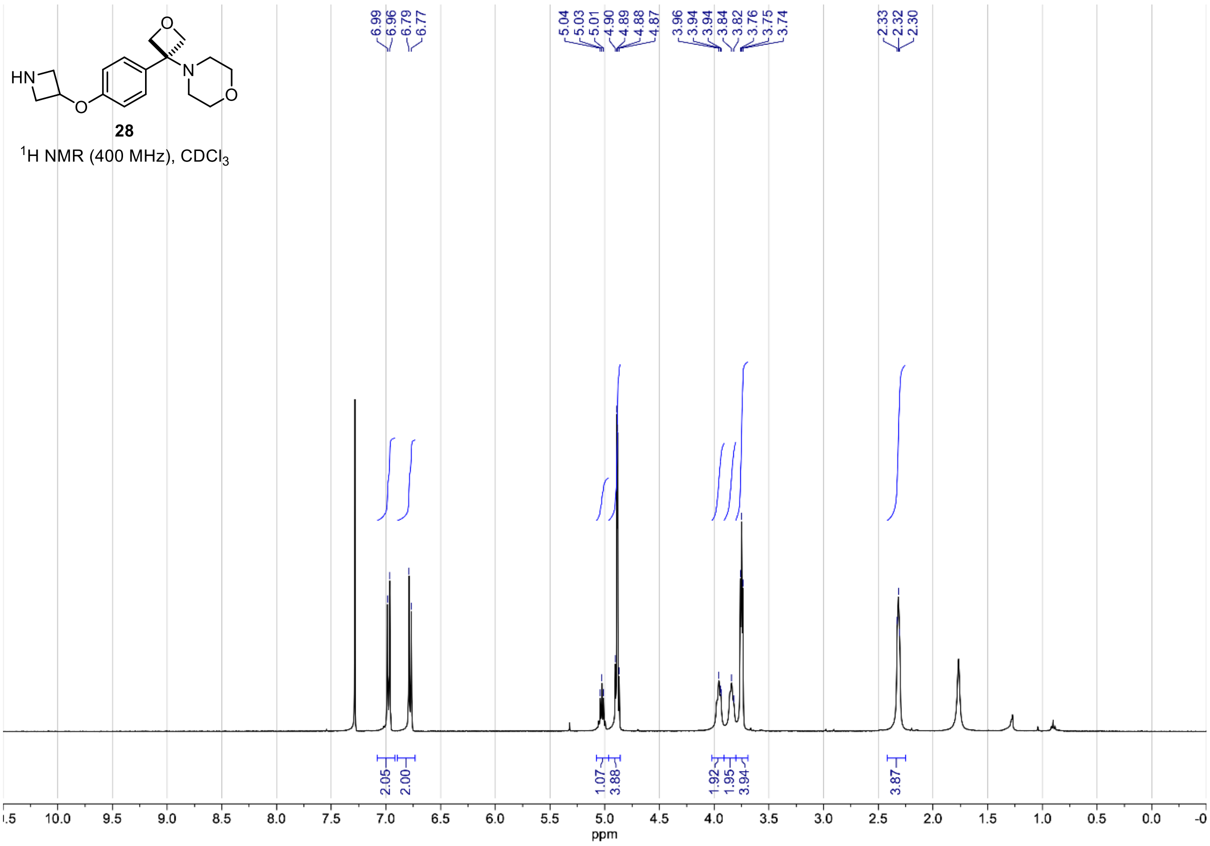
28
1H NMR (400 MHz), CDCl3
6.99
6.96
6.79
6.77
5.04
5.03
5.01
4.90
4.89
4.88
4.87
3.96
3.94
3.94
3.84
3.82
3.76
3.75
3.74
2.33
2.32
2.30
2.05
2.00
1.07
3.88
1.92
1.95
3.94
3.87
ppm

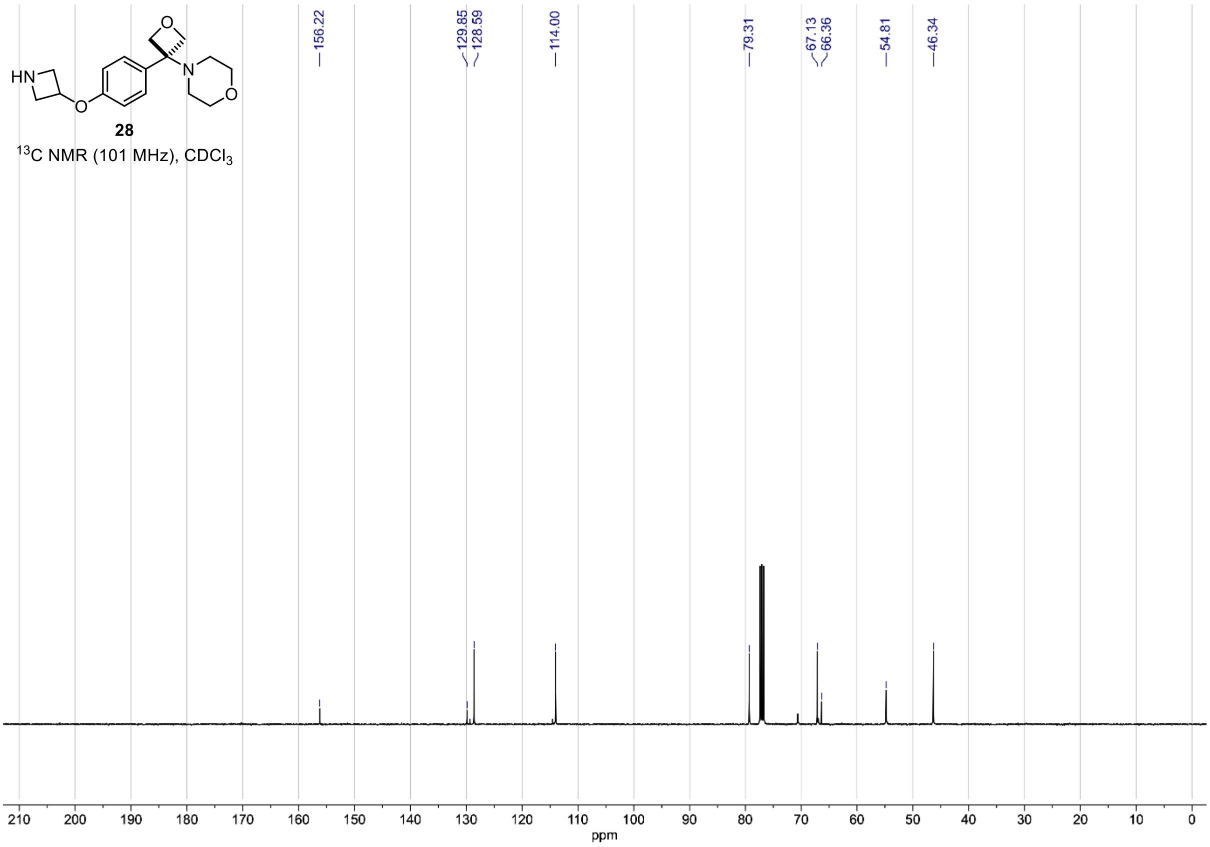
28
13C NMR (101 MHz), CDCl3
156.22
129.85
128.59
114.00
79.31
67.13
66.36
54.81
46.34
ppm

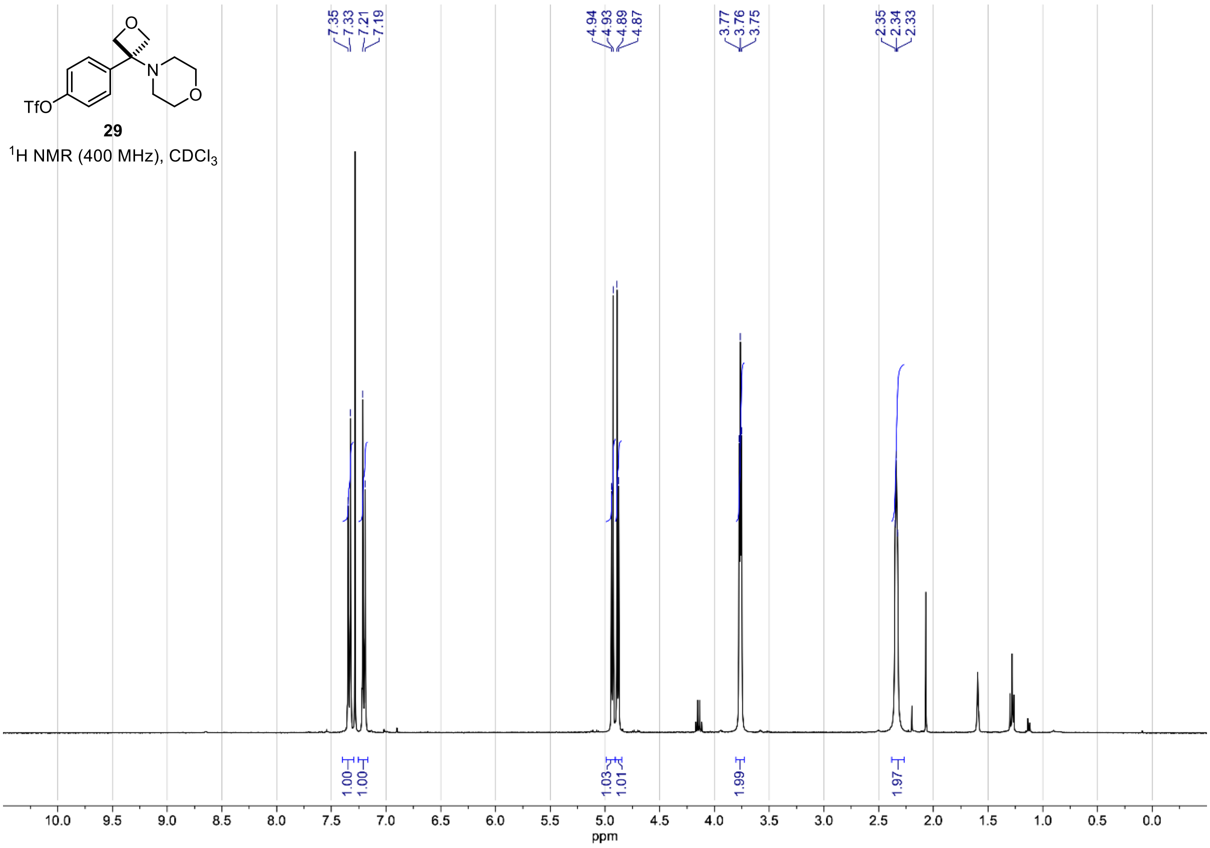

29

^{1}H NMR (400 MHz), $CDCl_3$

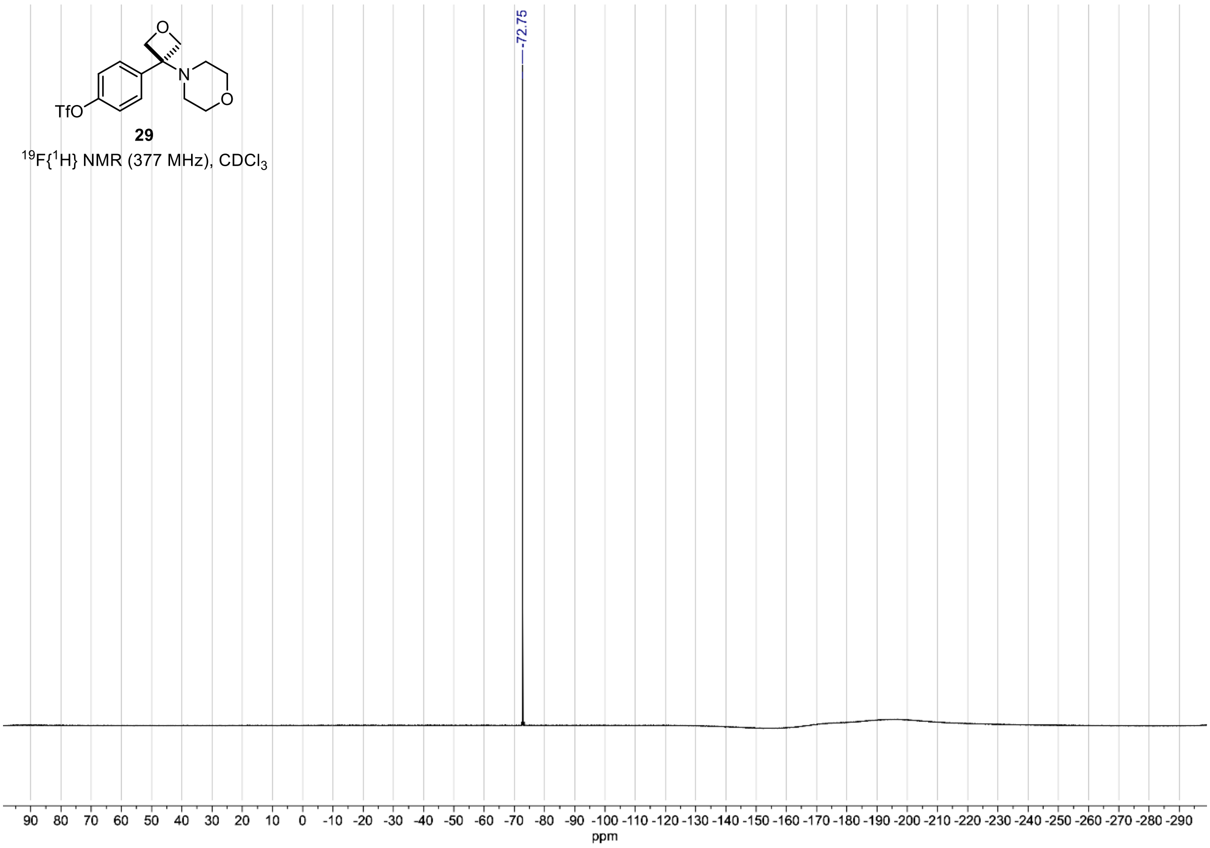

29

$^{19}F\{^{1}H\}$ NMR (377 MHz), $CDCl_3$

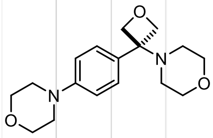

30

^{1}H NMR (400 MHz), $CDCl_3$

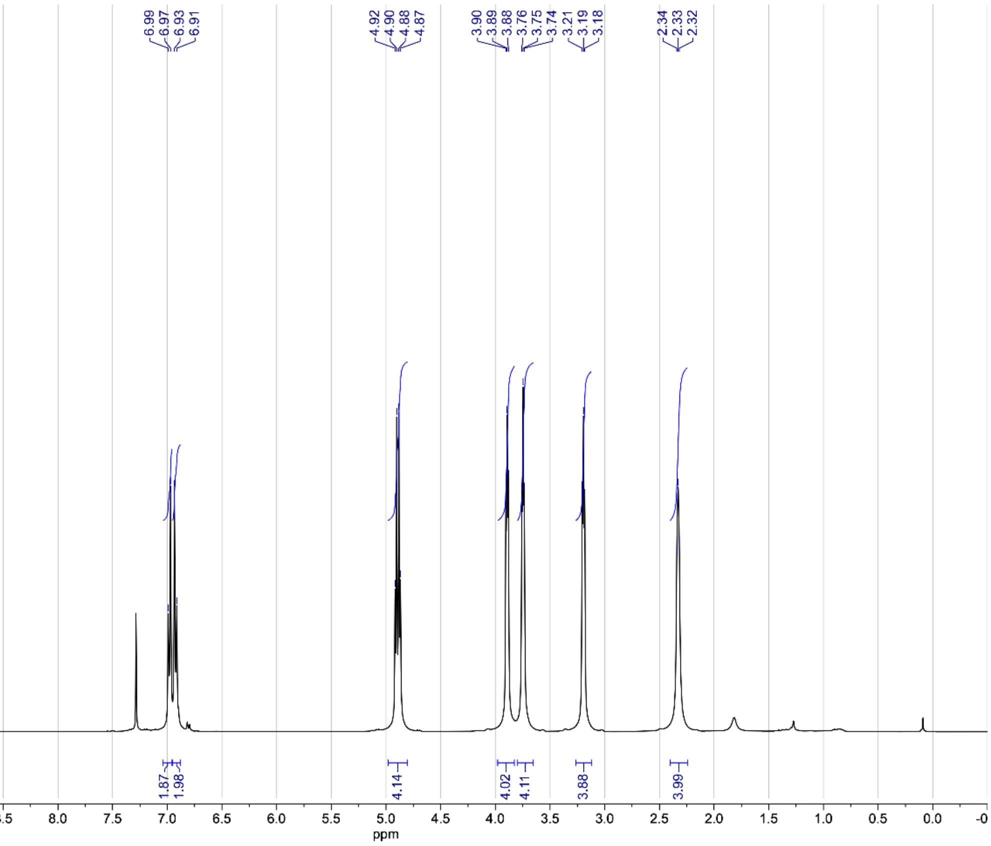

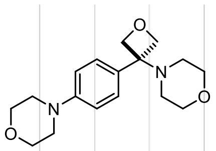

30

^{13}C NMR (101 MHz), $CDCl_3$

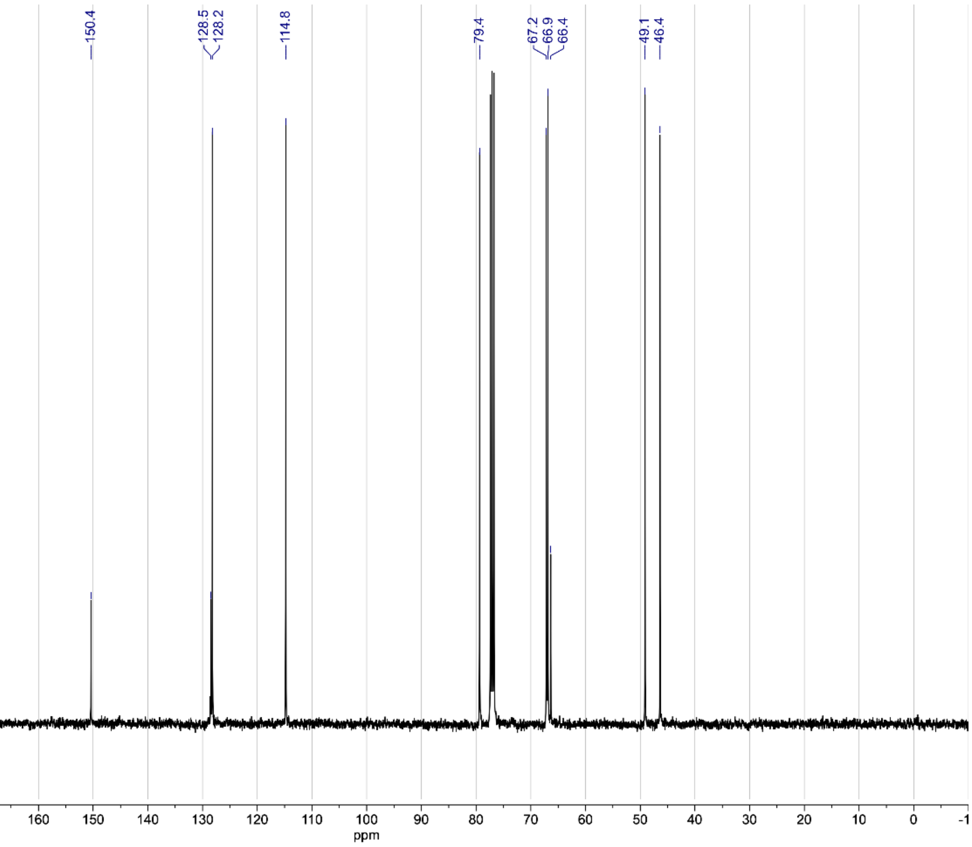

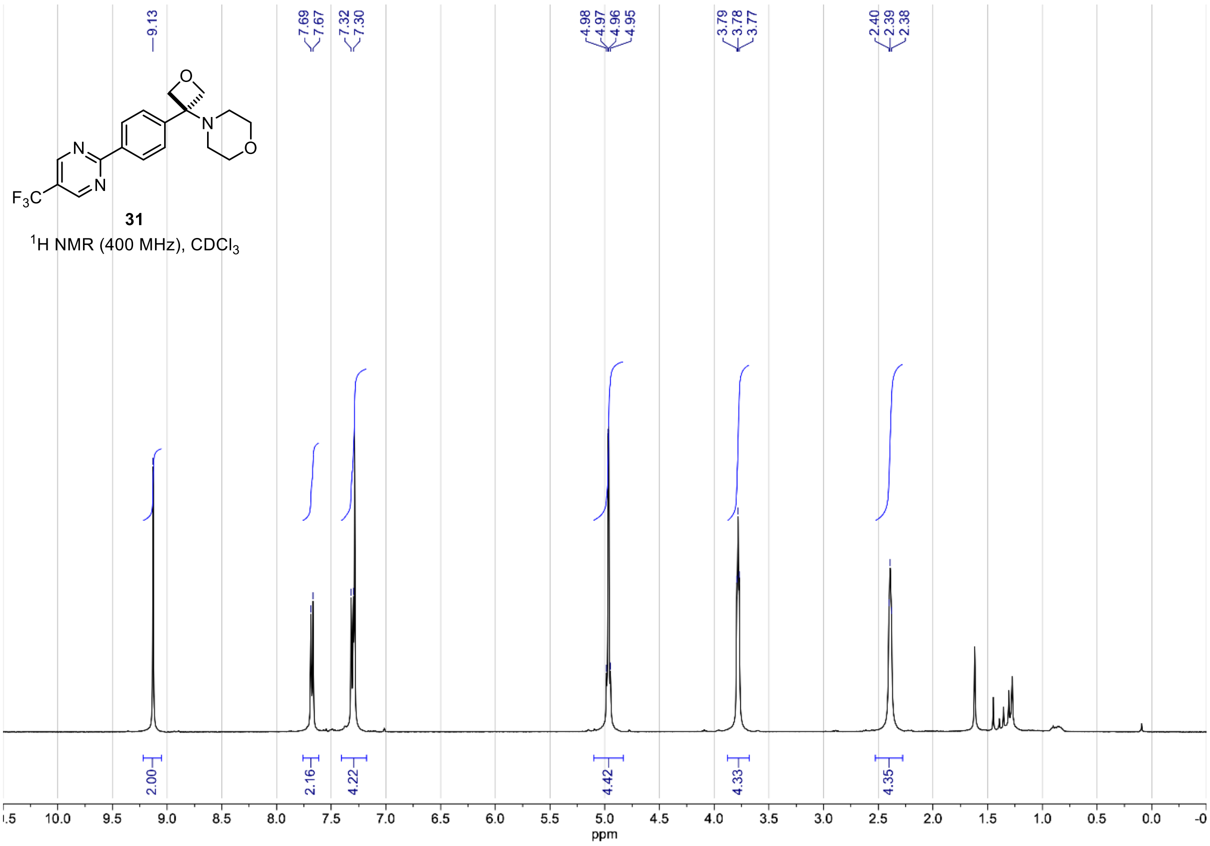

31

^{1}H NMR (400 MHz), $CDCl_3$

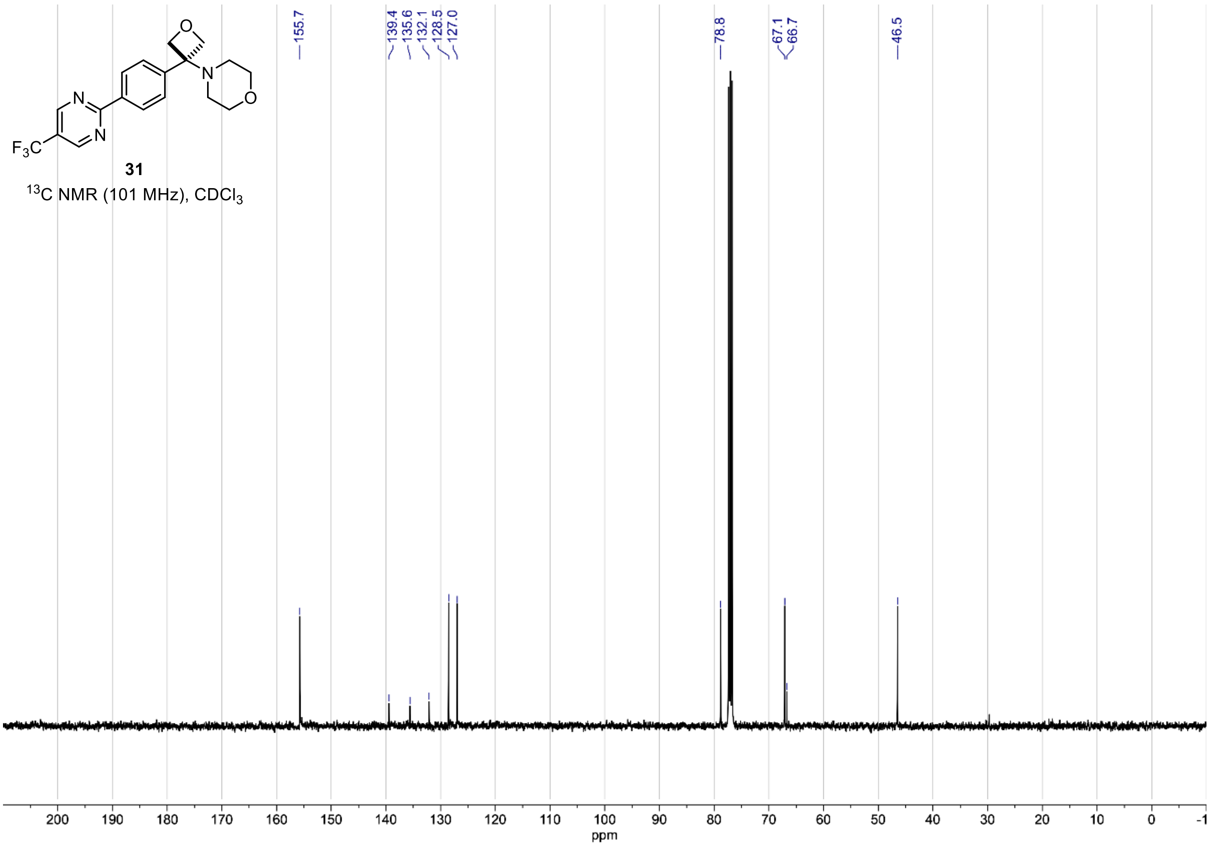

31

^{13}C NMR (101 MHz), $CDCl_3$

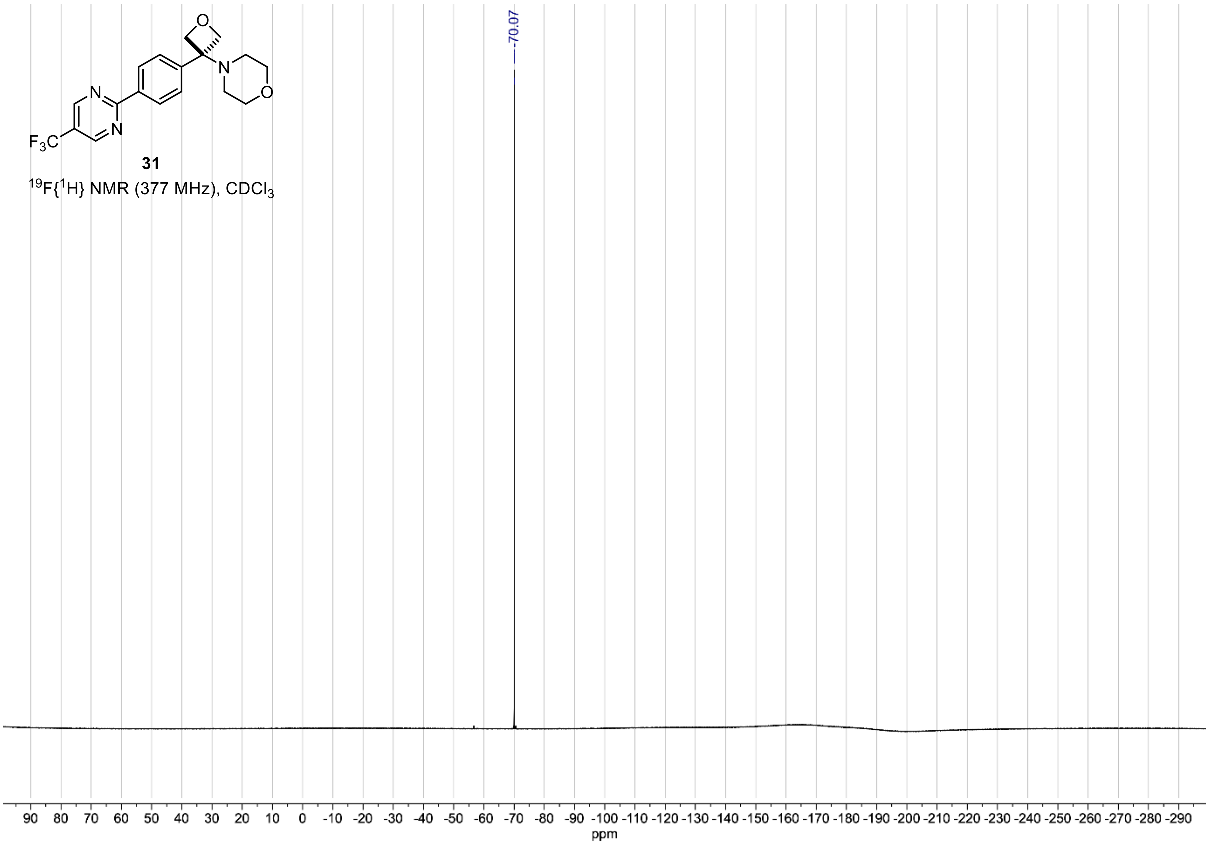

-70.07
F3C
N
N
O
N
O
31
19F{1H} NMR (377 MHz), CDCl3
90 80 70 60 50 40 30 20 10 0 -10 -20 -30 -40 -50 -60 -70 -80 -90 -100 -110 -120 -130 -140 -150 -160 -170 -180 -190 -200 -210 -220 -230 -240 -250 -260 -270 -280 -290
ppm

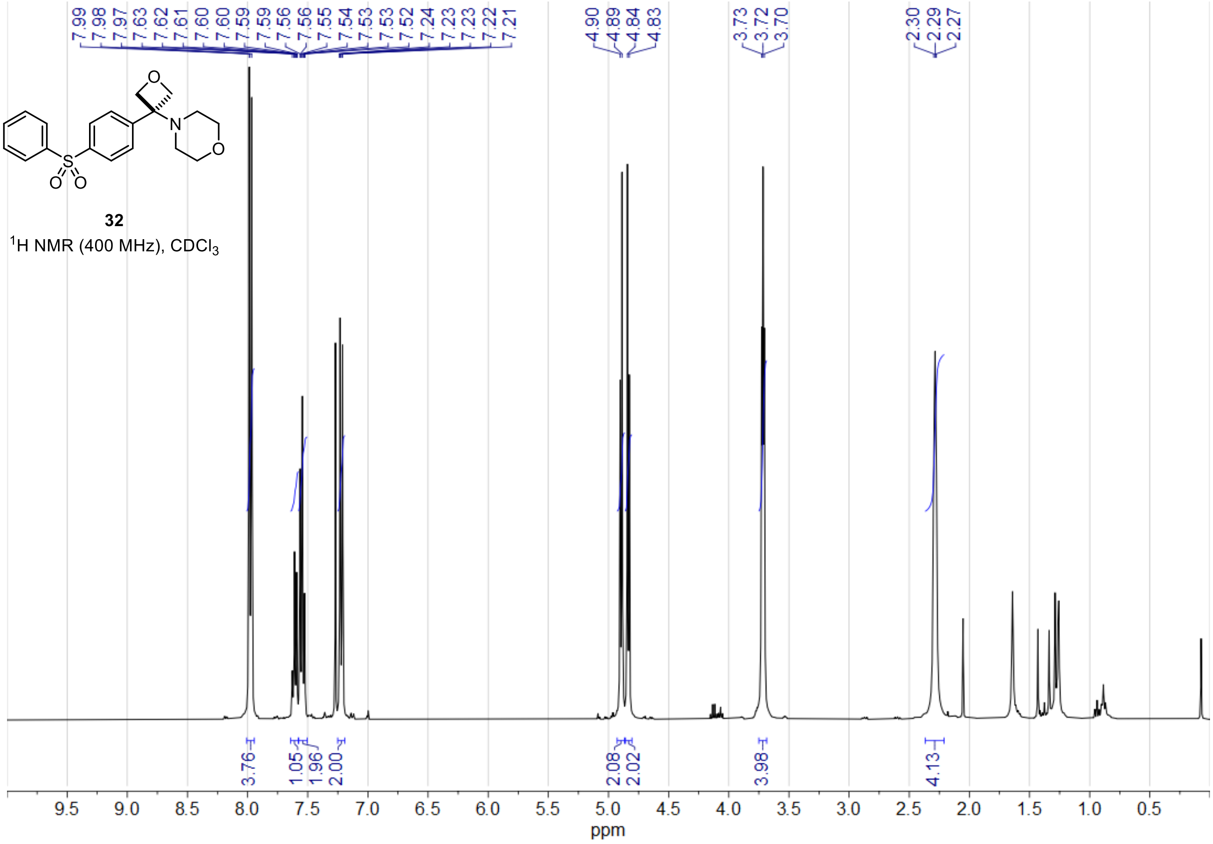
7.99 7.98 7.97 7.63 7.62 7.61 7.60 7.60 7.59 7.59 7.59 7.56 7.56 7.55 7.54 7.53 7.53 7.52 7.24 7.23 7.23 7.22 7.21
4.90 4.89 4.84 4.83
3.73 3.72 3.70
2.30 2.29 2.27
32
^{1}H NMR (400 MHz), CDCl$_3$
3.76 1.05 1.96 2.00 2.08 2.02 3.98 4.13
9.5 9.0 8.5 8.0 7.5 7.0 6.5 6.0 5.5 5.0 4.5 4.0 3.5 3.0 2.5 2.0 1.5 1.0 0.5
ppm

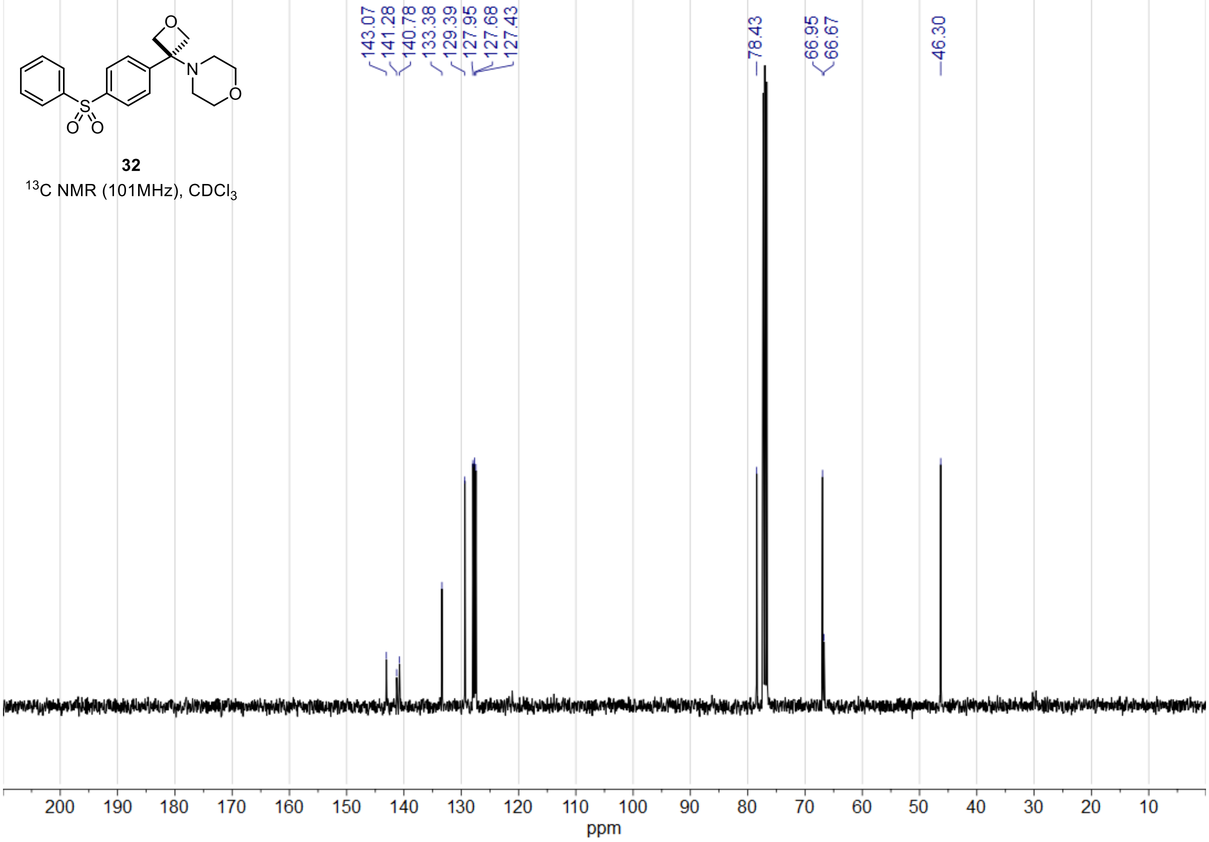
143.07 141.28 140.78 133.38 129.39 127.95 127.68 127.43
78.43
66.95 66.67
46.30
32
^{13}C NMR (101MHz), CDCl$_3$
200 190 180 170 160 150 140 130 120 110 100 90 80 70 60 50 40 30 20 10
ppm

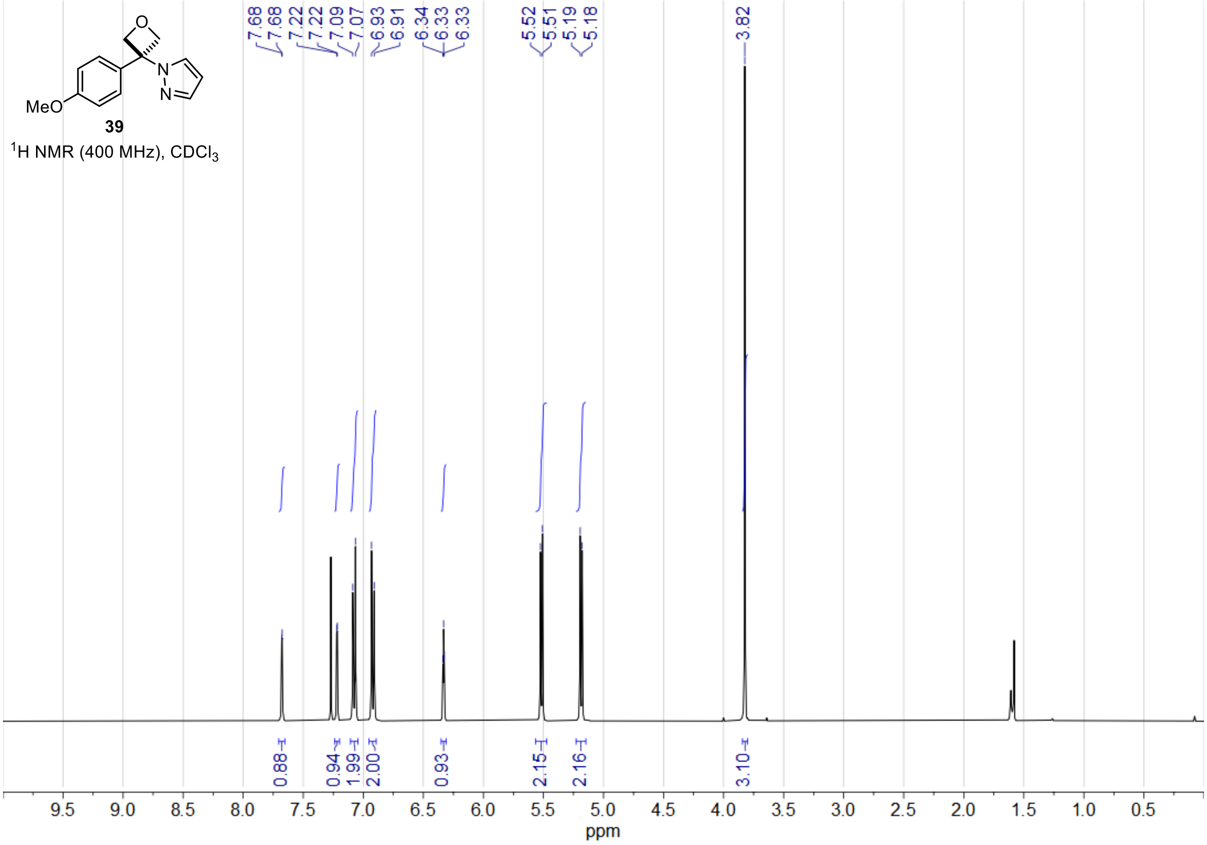

39

^{1}H NMR (400 MHz), $CDCl_3$

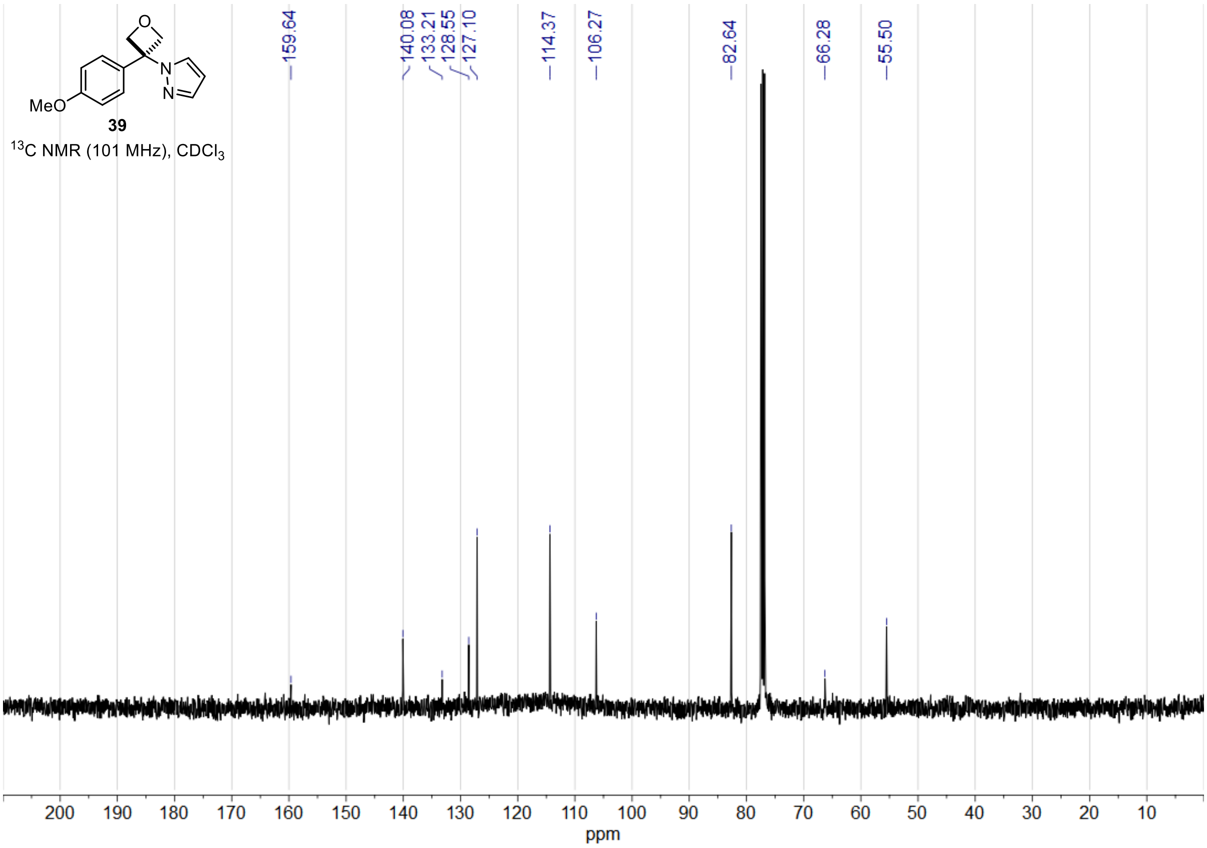

39

^{13}C NMR (101 MHz), $CDCl_3$

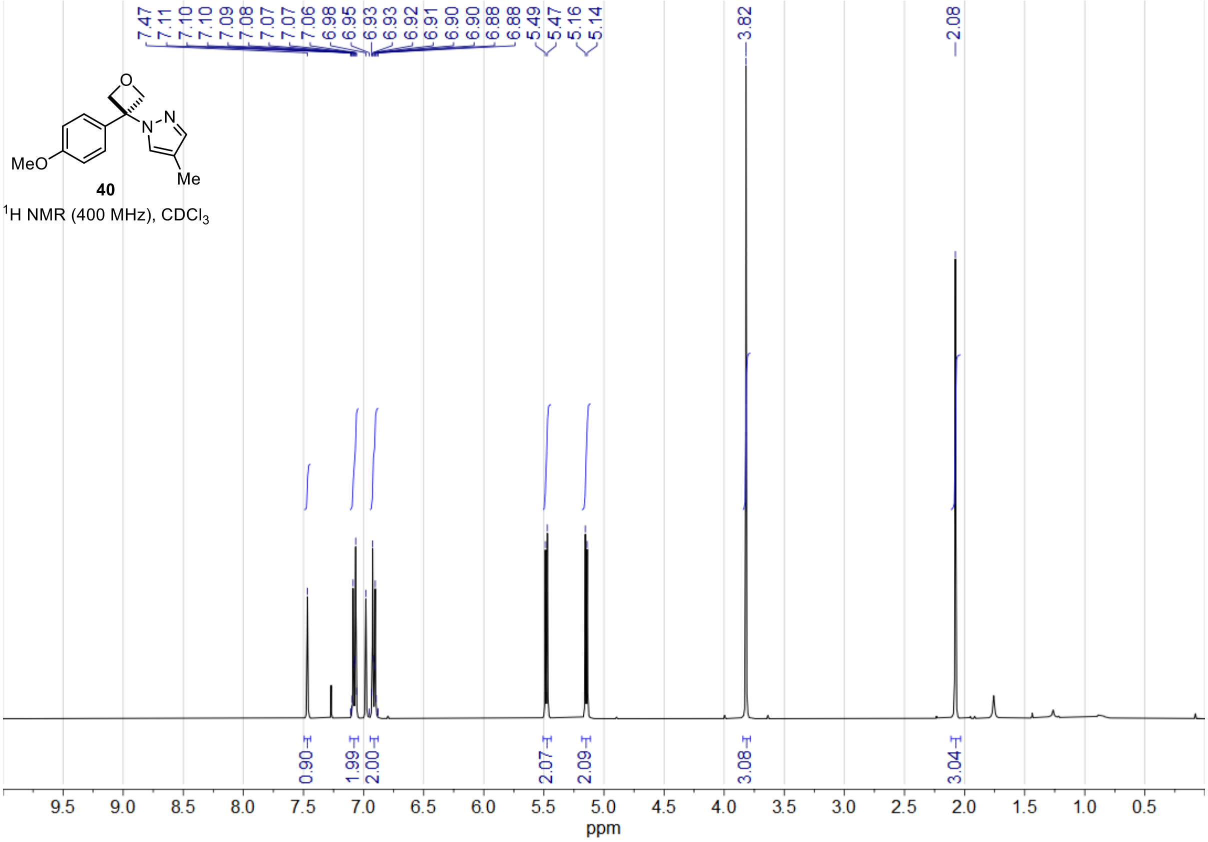
7.47
7.11
7.10
7.10
7.09
7.08
7.07
7.07
7.06
6.98
6.95
6.93
6.93
6.92
6.91
6.90
6.90
6.88
5.49
5.47
5.16
5.14
3.82
2.08
MeO
N
N
O
Me
40
^{1}H NMR (400 MHz), $CDCl_3$
0.90
1.99
2.00
2.07
2.09
3.08
3.04
9.5
9.0
8.5
8.0
7.5
7.0
6.5
6.0
5.5
5.0
4.5
4.0
3.5
3.0
2.5
2.0
1.5
1.0
0.5
ppm

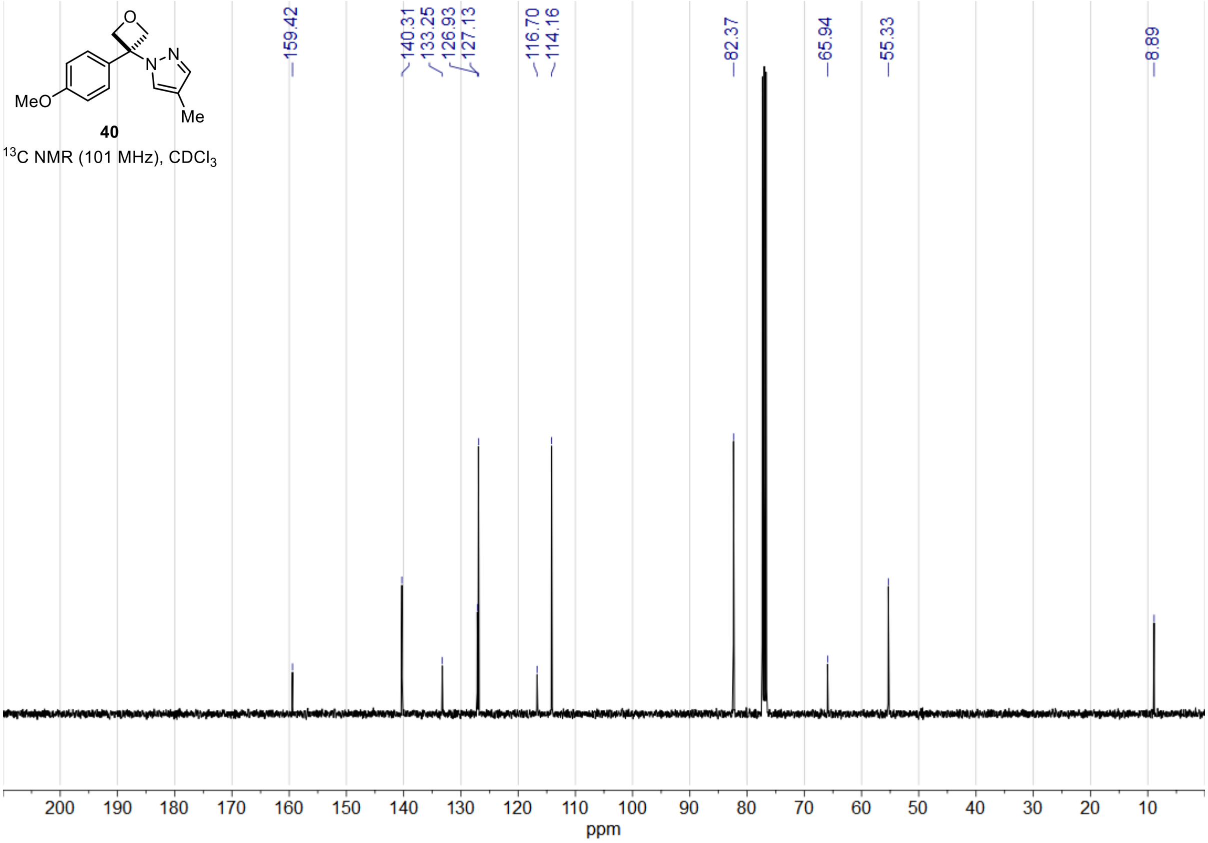
159.42
140.31
133.25
126.93
127.13
116.70
114.16
82.37
65.94
55.33
8.89
MeO
N
N
O
Me
40
^{13}C NMR (101 MHz), $CDCl_3$
200
190
180
170
160
150
140
130
120
110
100
90
80
70
60
50
40
30
20
10
ppm

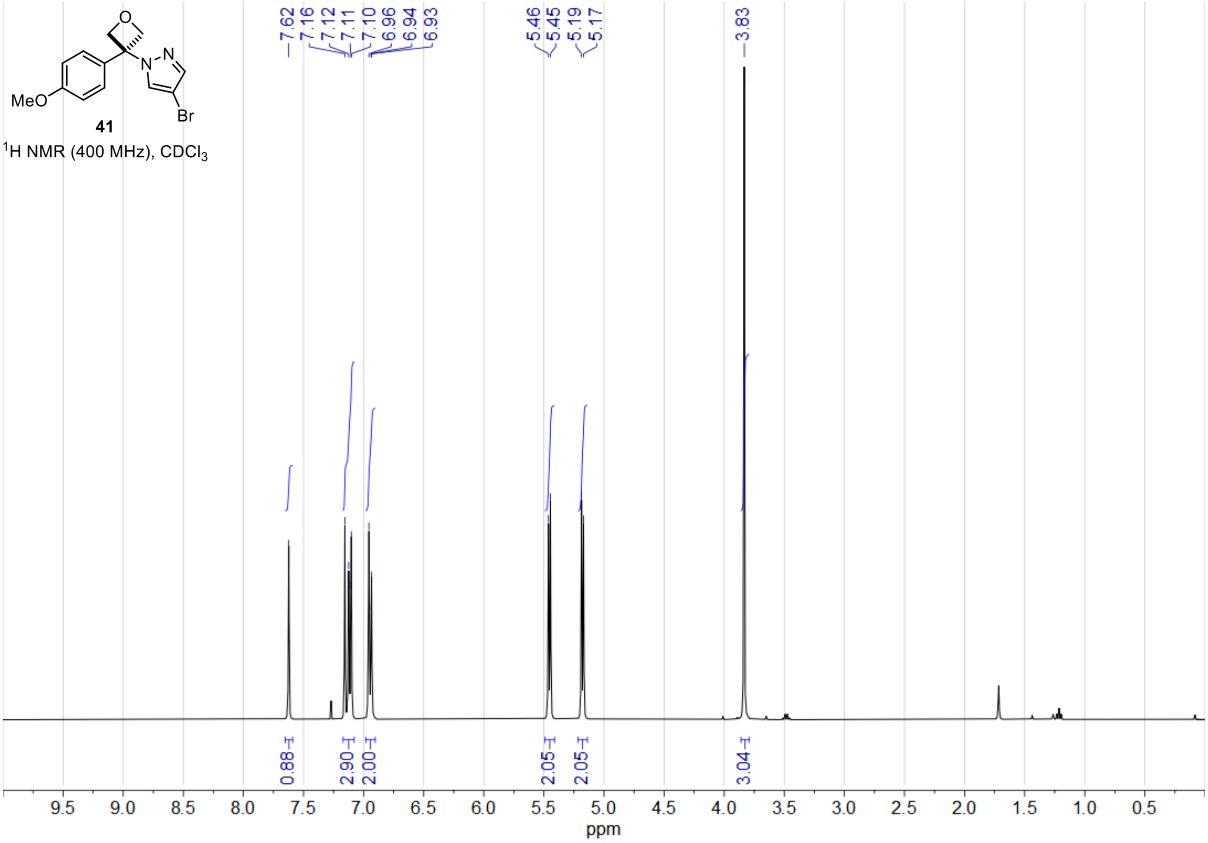
MeO
41
Br
N
O
^{1}H NMR (400 MHz), CDCl$_3$
7.62
7.16
7.12
7.11
7.10
6.96
6.94
6.93
5.46
5.45
5.19
5.17
3.83
0.88
2.90
2.00
2.05
2.05
3.04
ppm

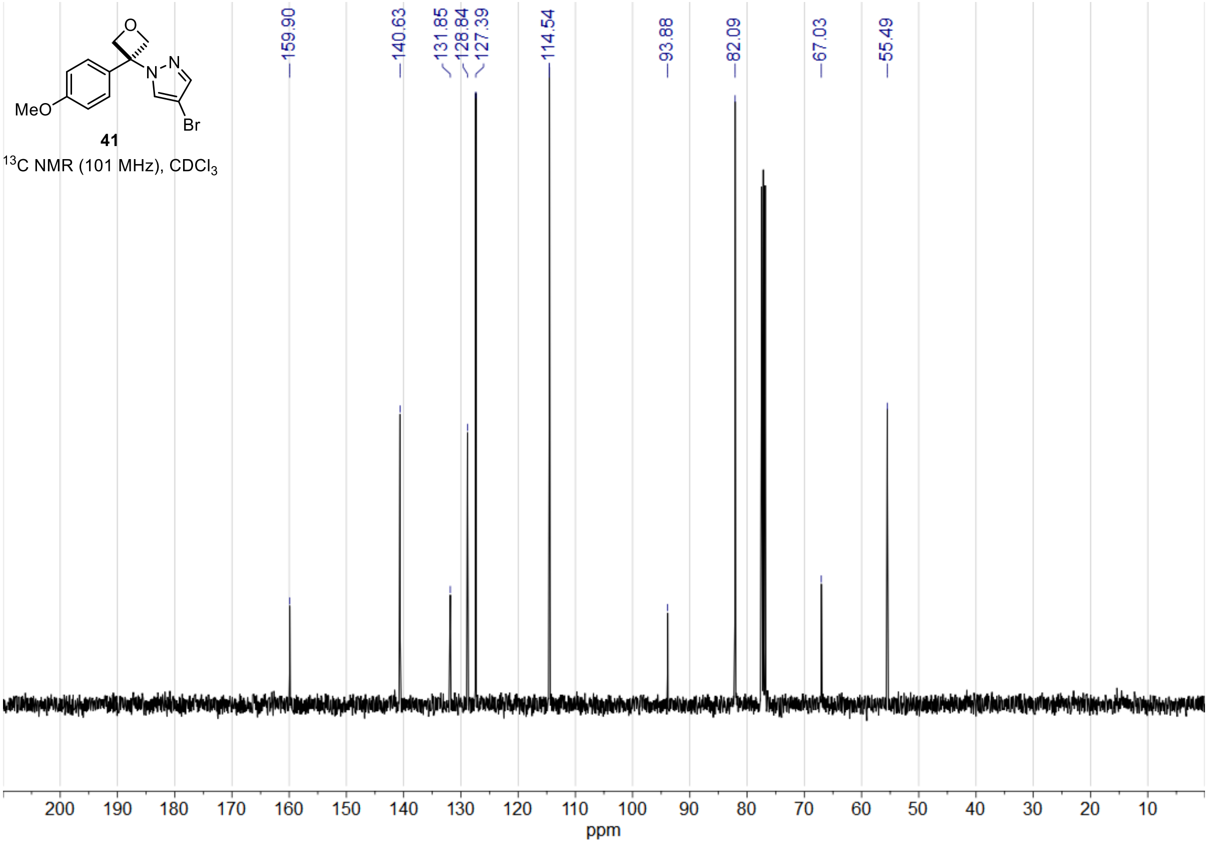
MeO
41
Br
N
O
^{13}C NMR (101 MHz), CDCl$_3$
159.90
140.63
131.85
128.84
127.39
114.54
93.88
82.09
67.03
55.49
ppm

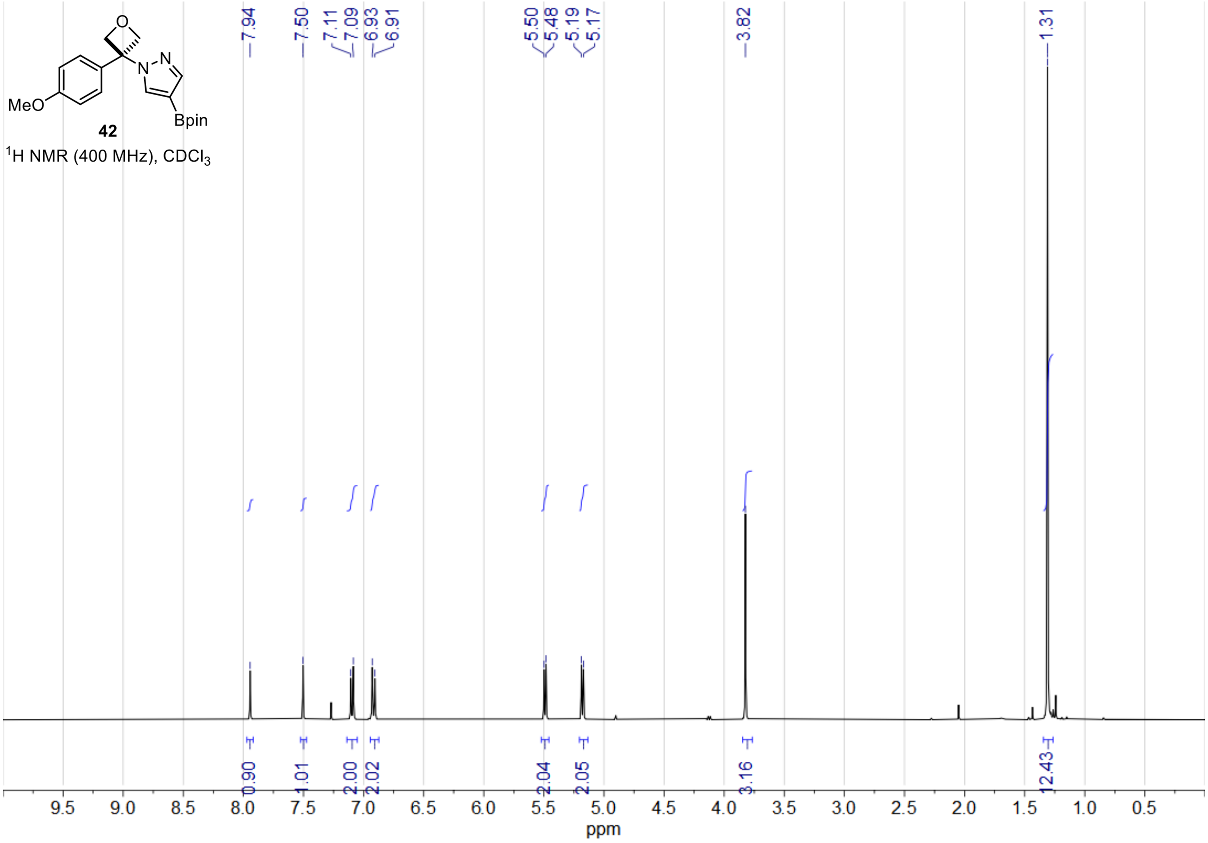
MeO
Bpin
42
1H NMR (400 MHz), CDCl3
7.94
7.50
7.11
7.09
6.93
6.91
5.50
5.48
5.19
5.17
3.82
1.31
0.90
1.01
2.00
2.02
2.04
2.05
3.16
12.43
9.5 9.0 8.5 8.0 7.5 7.0 6.5 6.0 5.5 5.0 4.5 4.0 3.5 3.0 2.5 2.0 1.5 1.0 0.5
ppm

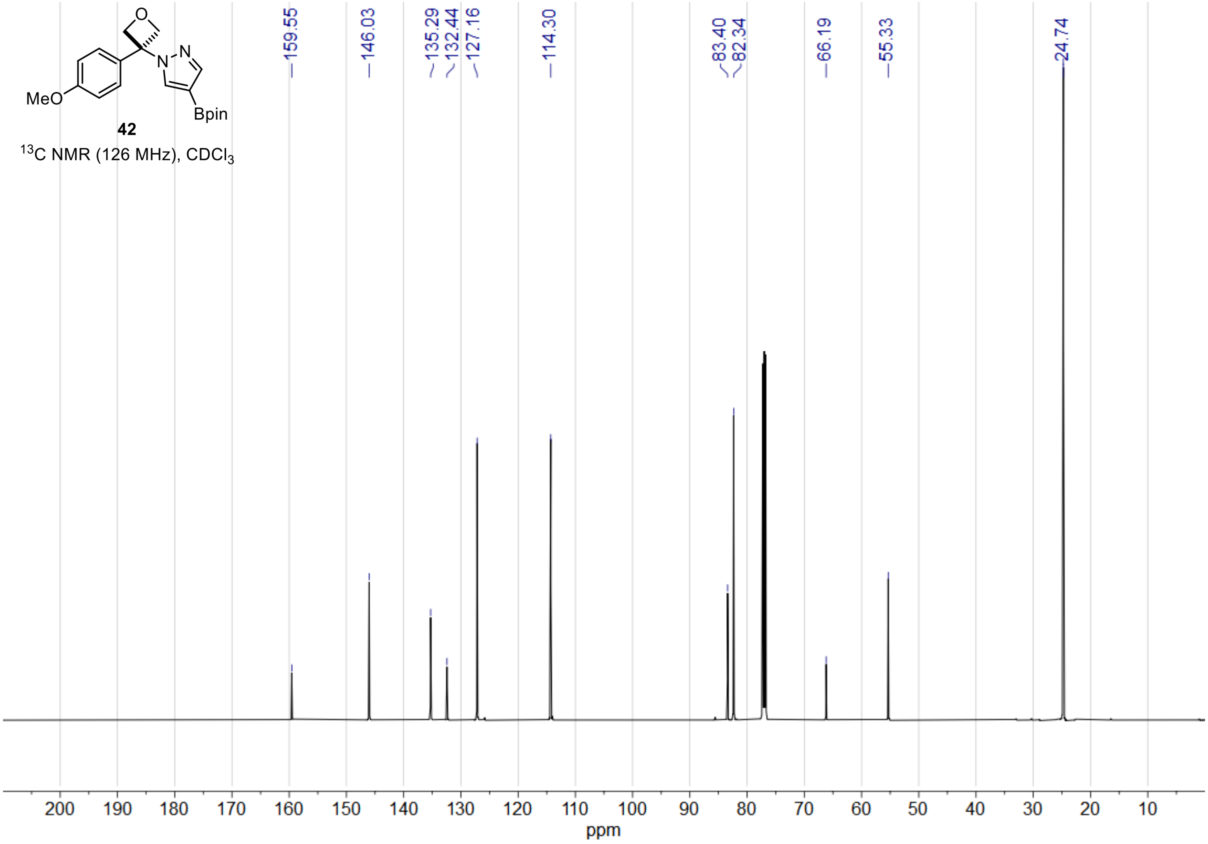
MeO
Bpin
42
13C NMR (126 MHz), CDCl3
159.55
146.03
135.29
132.44
127.16
114.30
83.40
82.34
66.19
55.33
24.74
200 190 180 170 160 150 140 130 120 110 100 90 80 70 60 50 40 30 20 10
ppm

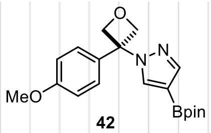

42

^{11}B NMR (128 MHz), $CDCl_3$

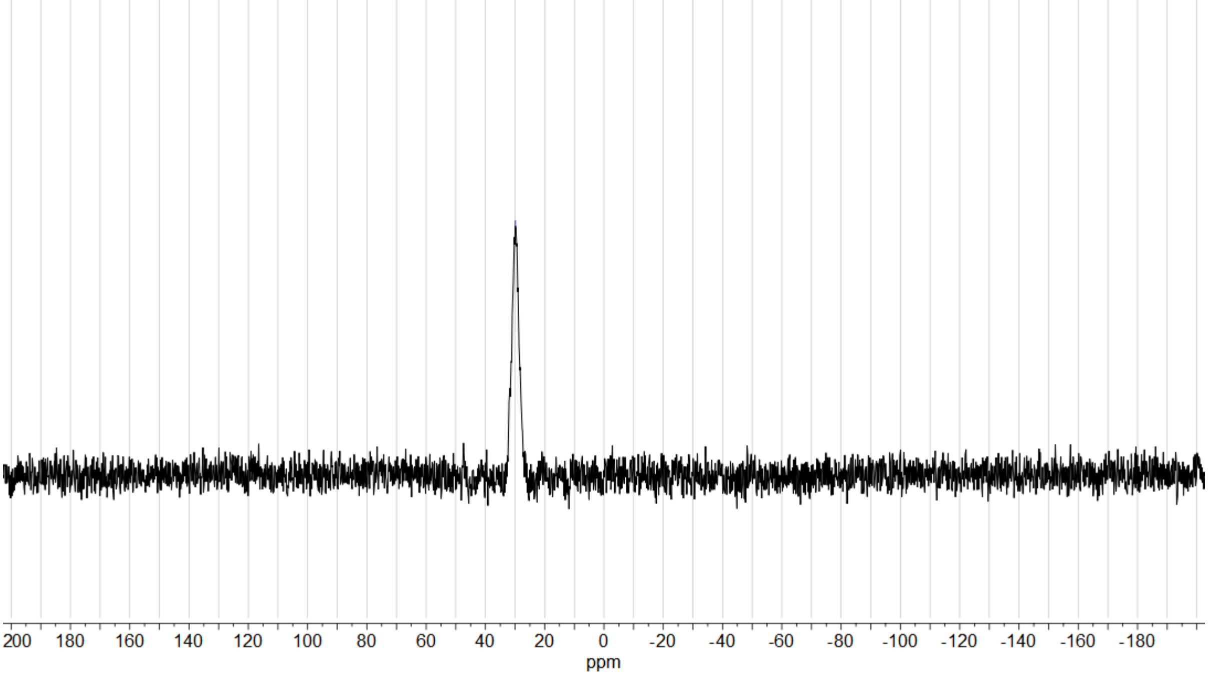

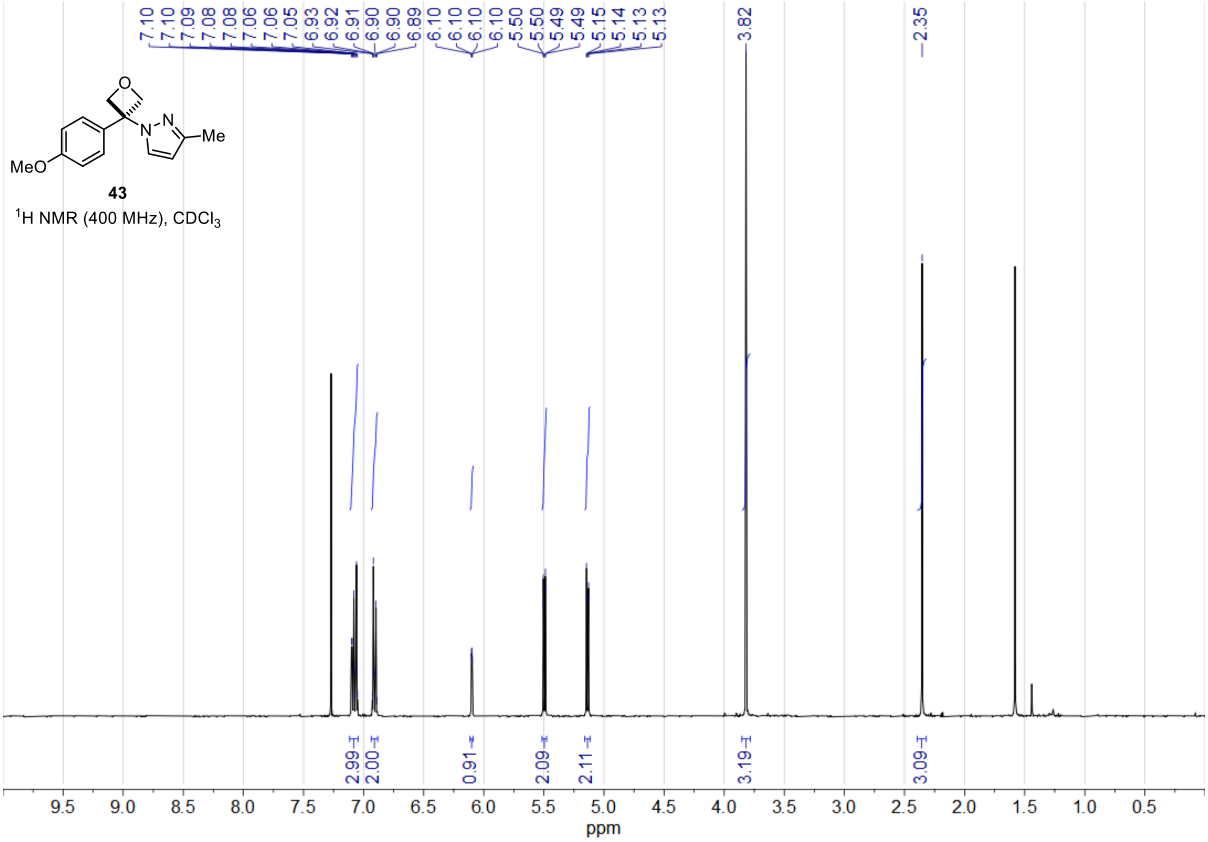

43

^{1}H NMR (400 MHz), $CDCl_3$

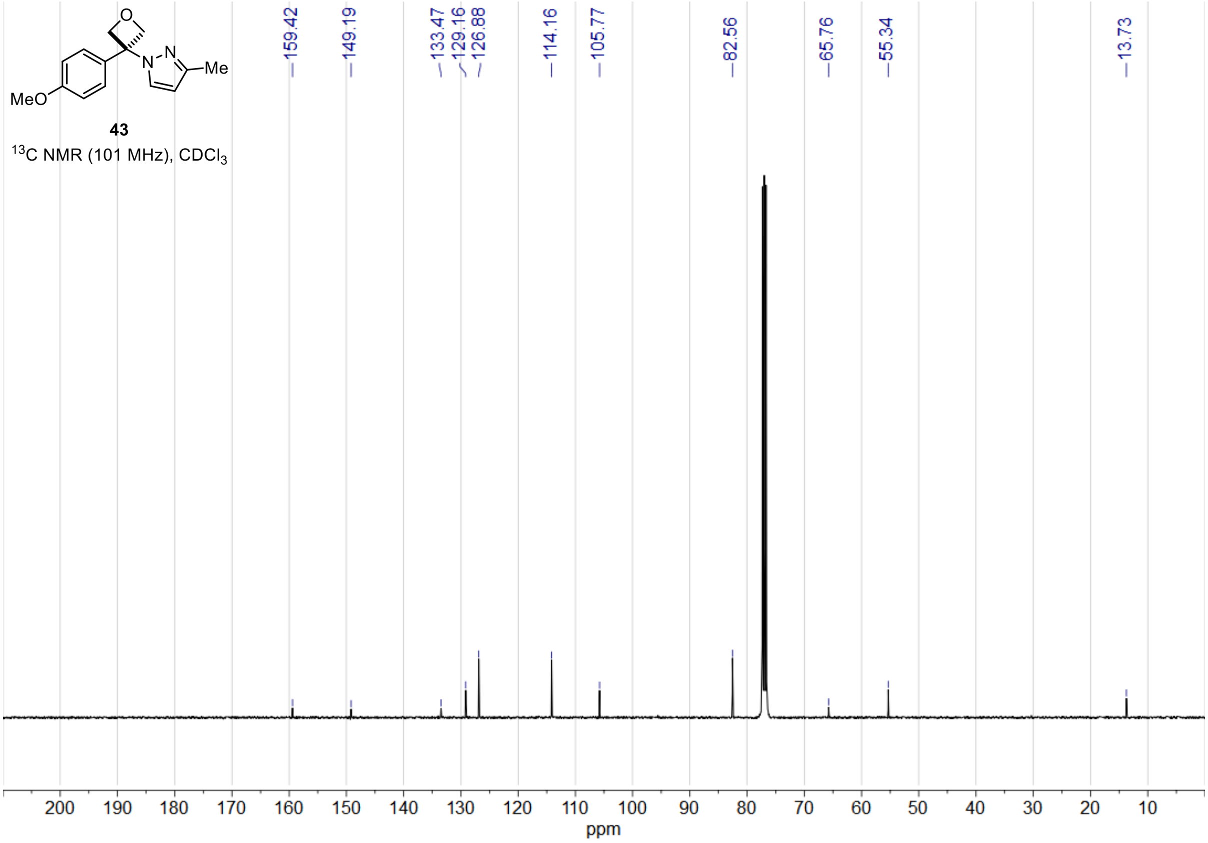

43

^{13}C NMR (101 MHz), $CDCl_3$

Selective nOe of **43** – irradiation at 5.48 ppm:

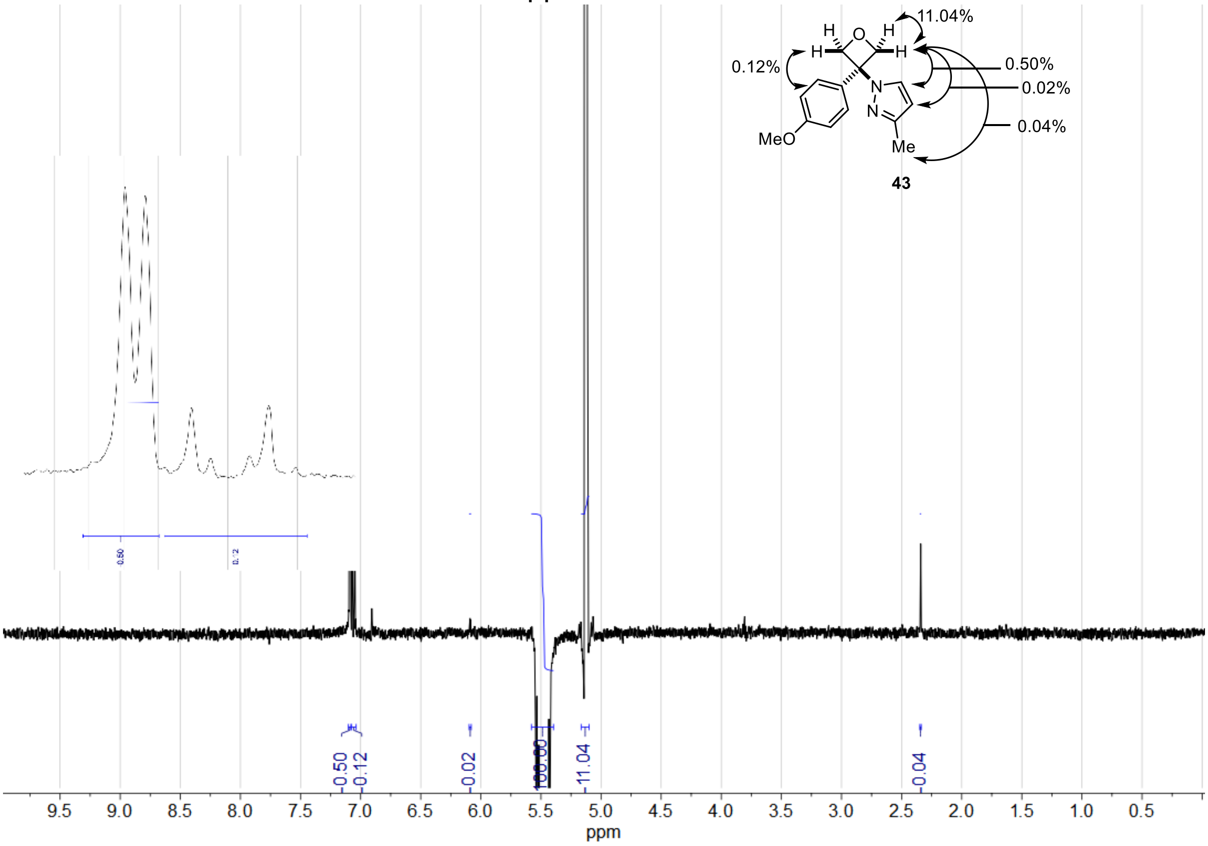

Selective nOe of **43** – irradiation at 5.13 ppm:

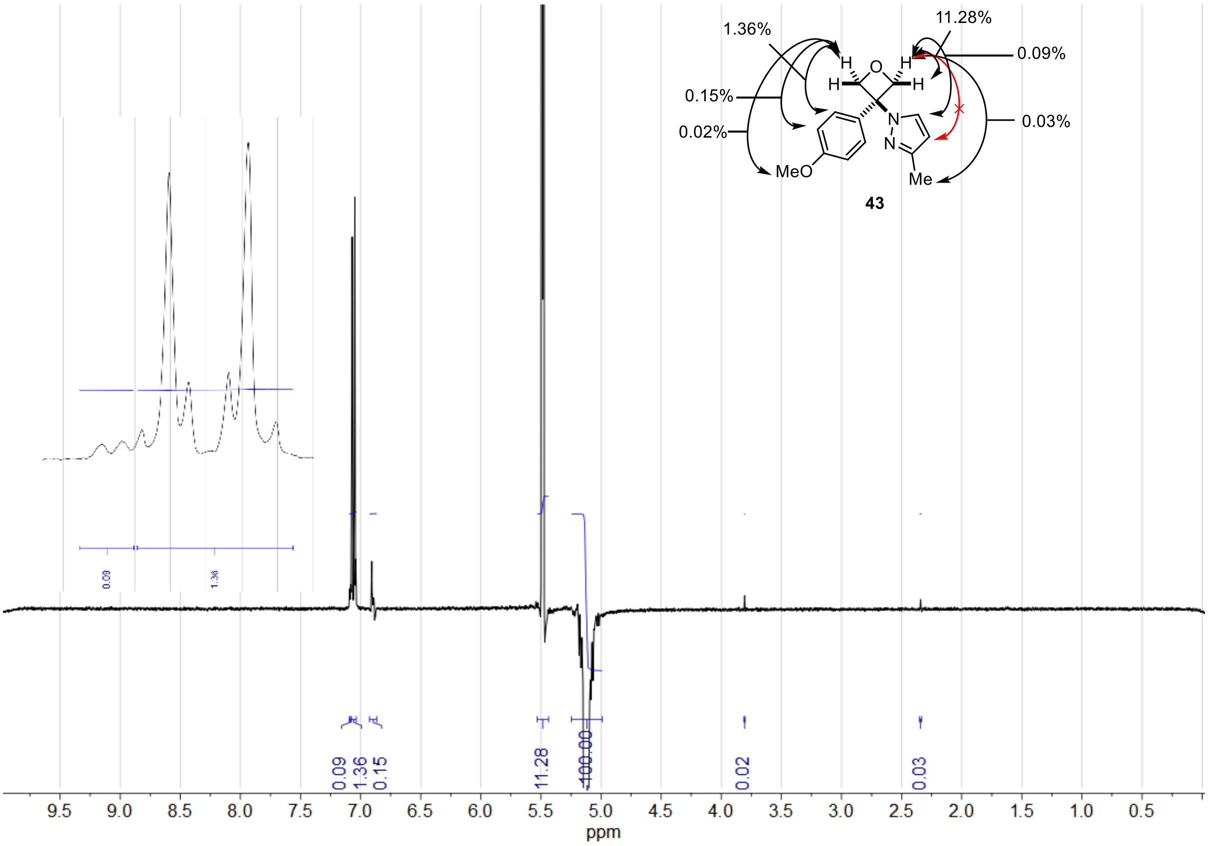

Selective nOe of **43** – irradiation at 2.34 ppm:

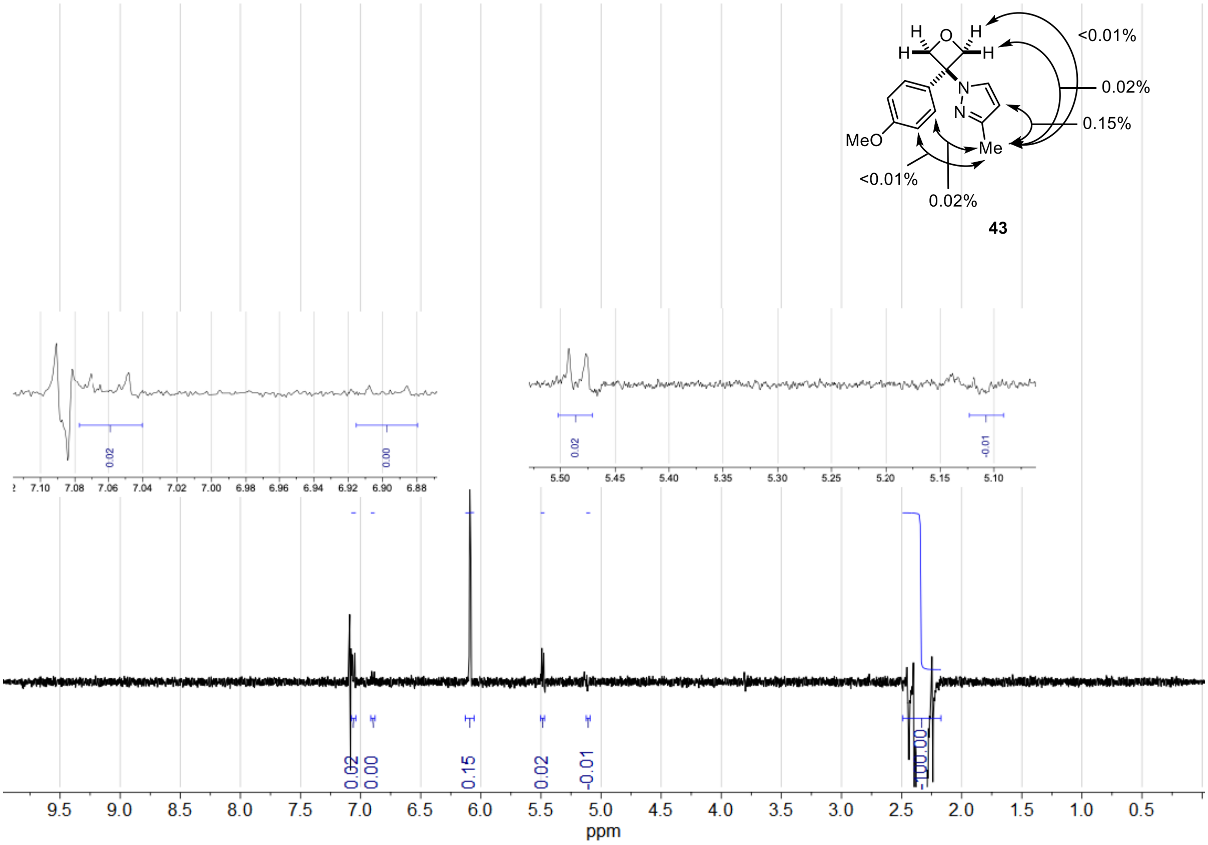

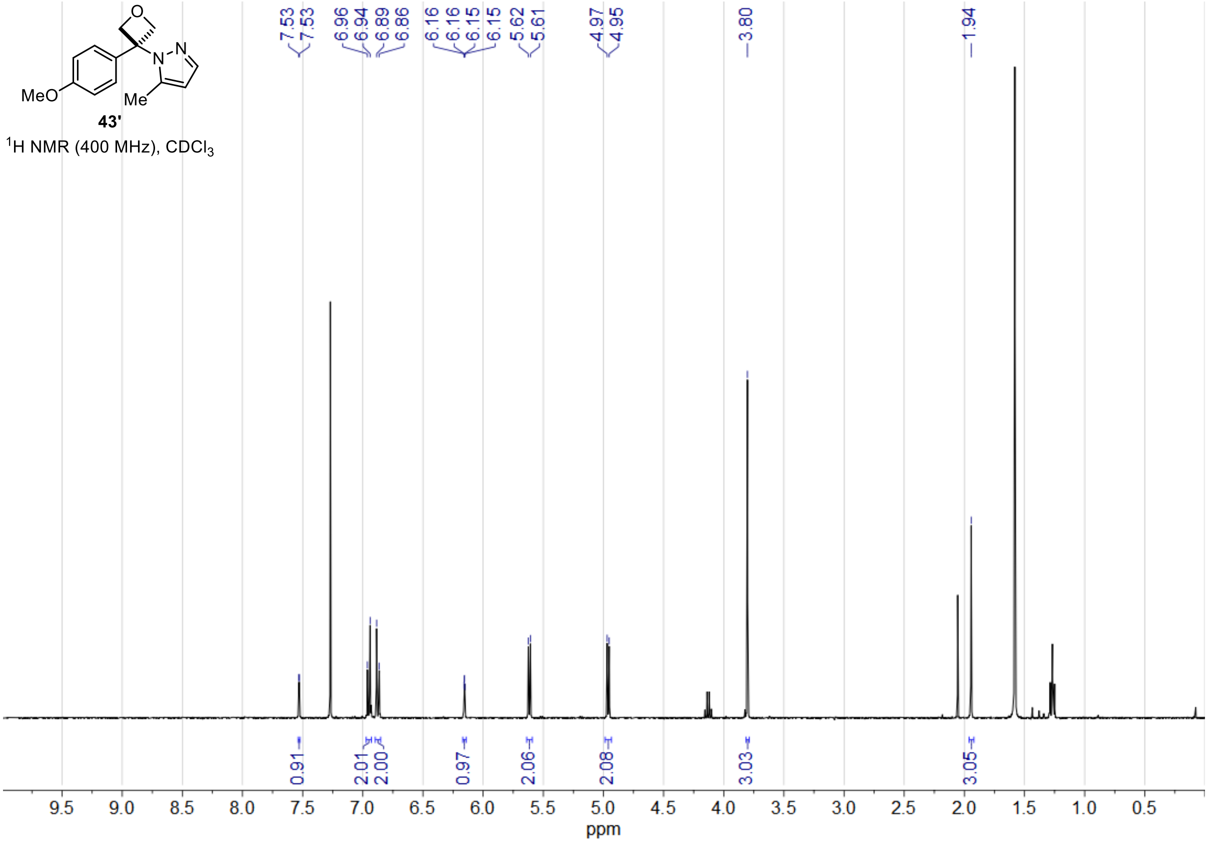
MeO
Me
43'
^{1}H NMR (400 MHz), CDCl$_3$
7.53
7.53
6.96
6.94
6.89
6.86
6.16
6.16
6.15
6.15
5.62
5.61
4.97
4.95
3.80
1.94
0.91
2.01
2.00
0.97
2.06
2.08
3.03
3.05
9.5 9.0 8.5 8.0 7.5 7.0 6.5 6.0 5.5 5.0 4.5 4.0 3.5 3.0 2.5 2.0 1.5 1.0 0.5
ppm

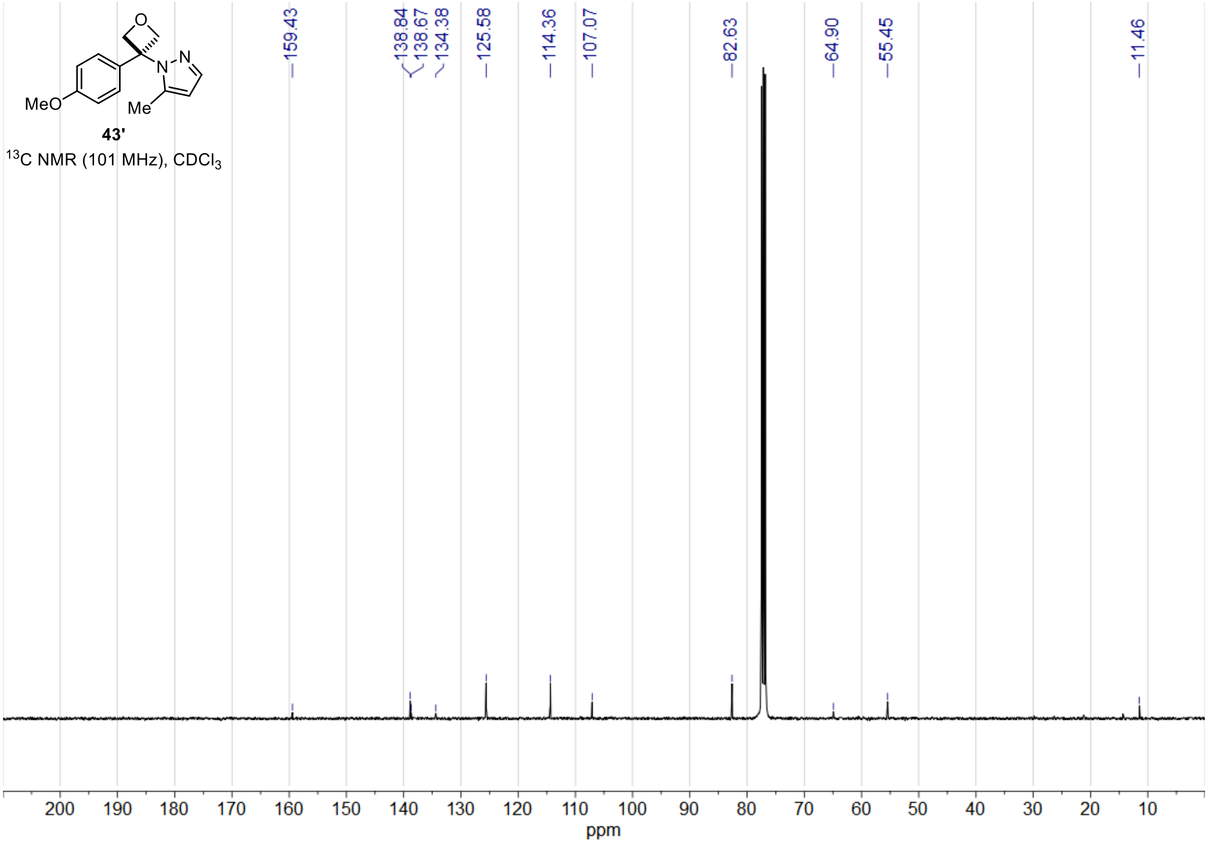
MeO
Me
43'
^{13}C NMR (101 MHz), CDCl$_3$
159.43
138.84
138.67
134.38
125.58
114.36
107.07
82.63
64.90
55.45
11.46
200 190 180 170 160 150 140 130 120 110 100 90 80 70 60 50 40 30 20 10
ppm

Selective nOe of **43’** – irradiation at 5.60 ppm

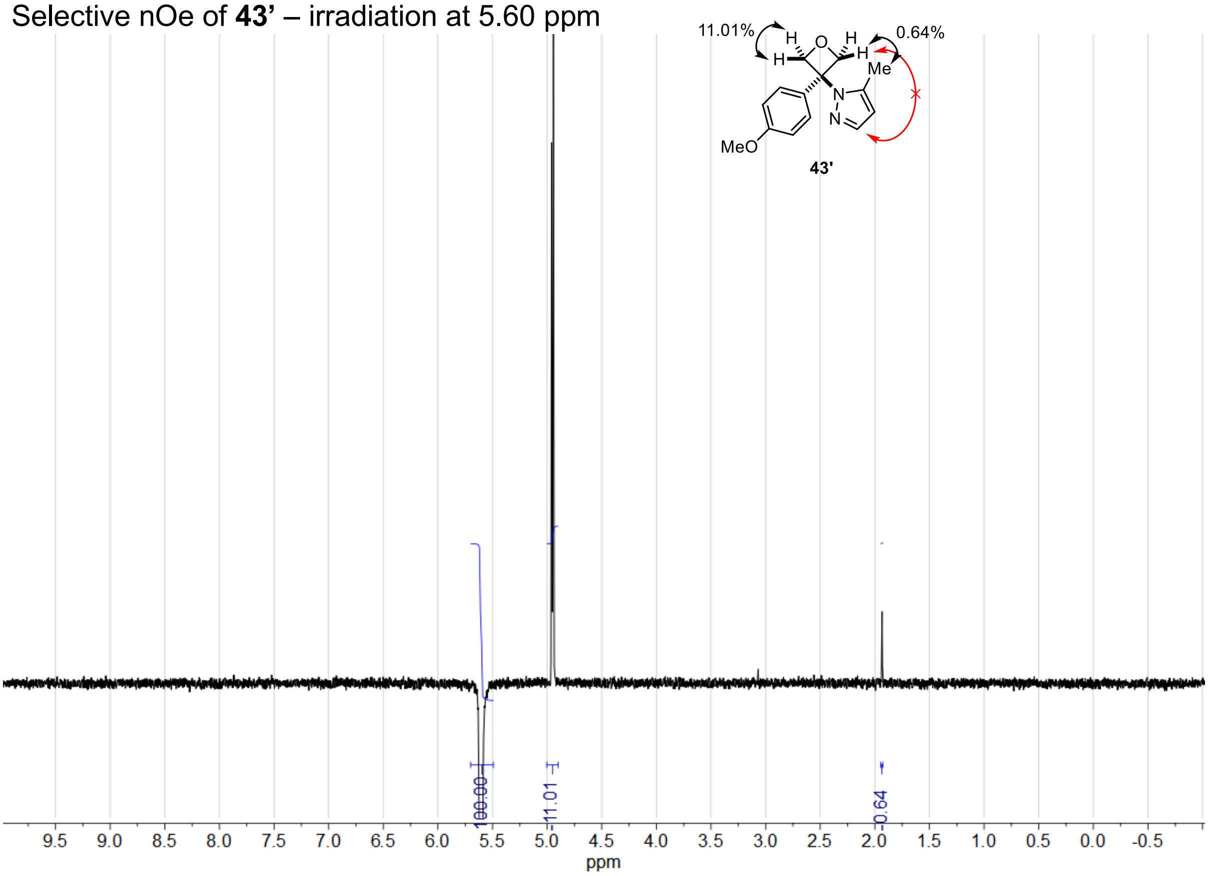

Selective nOe of **43’** – irradiation at 4.95 ppm

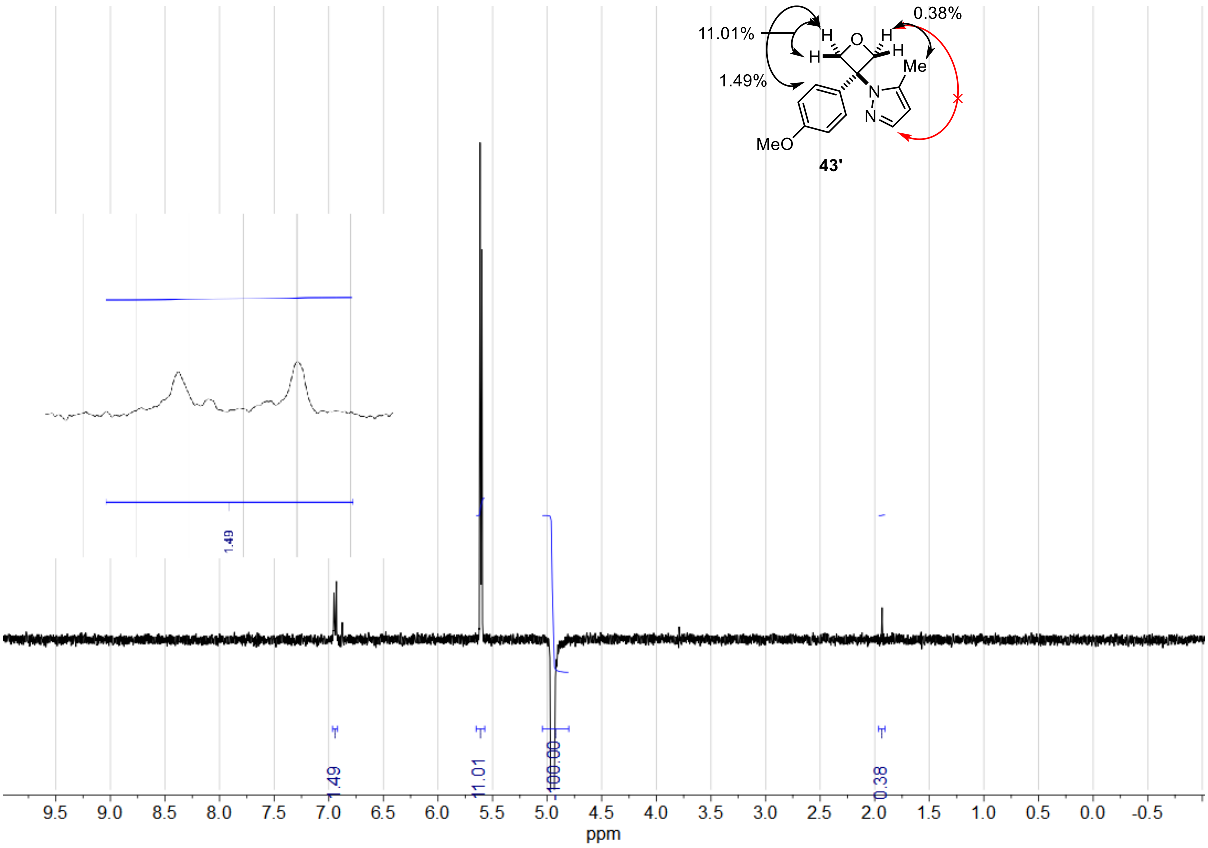

Selective nOe of **43'** – irradiation at 1.93 ppm

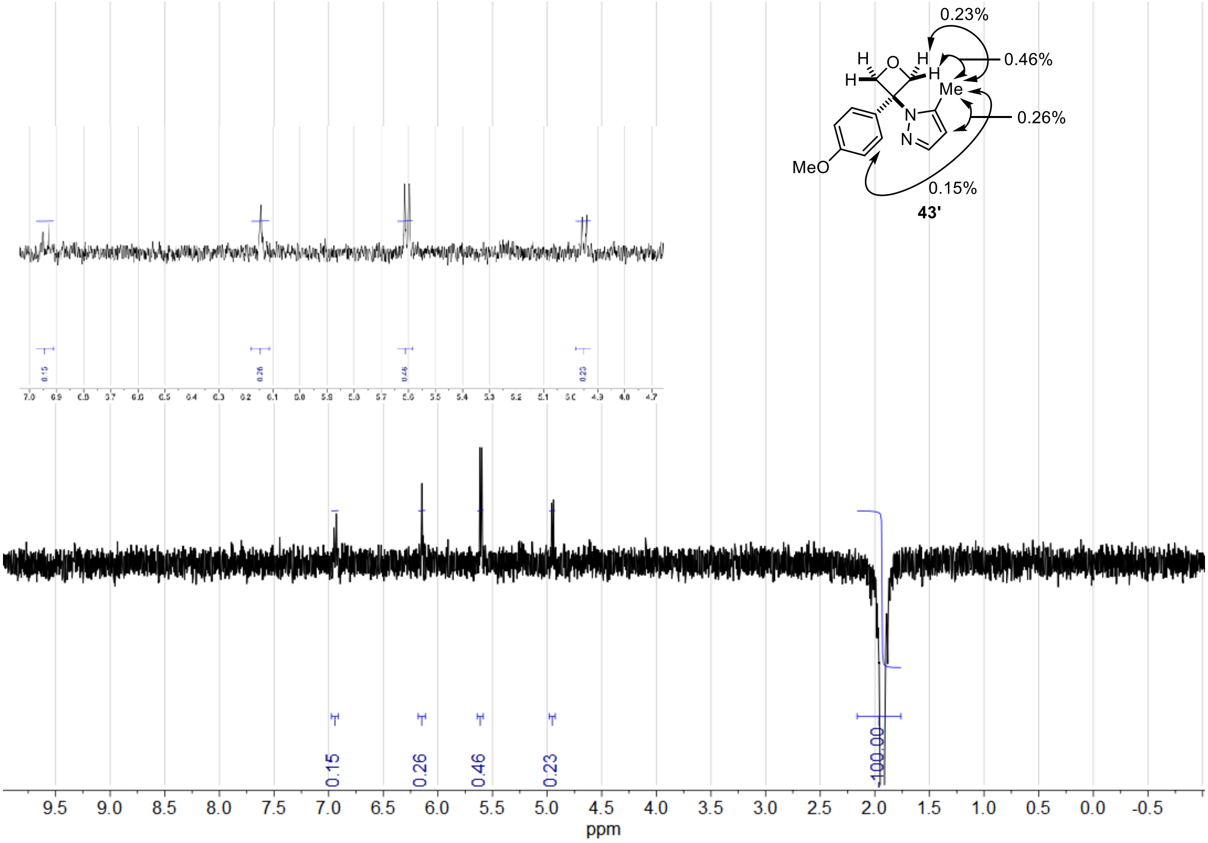

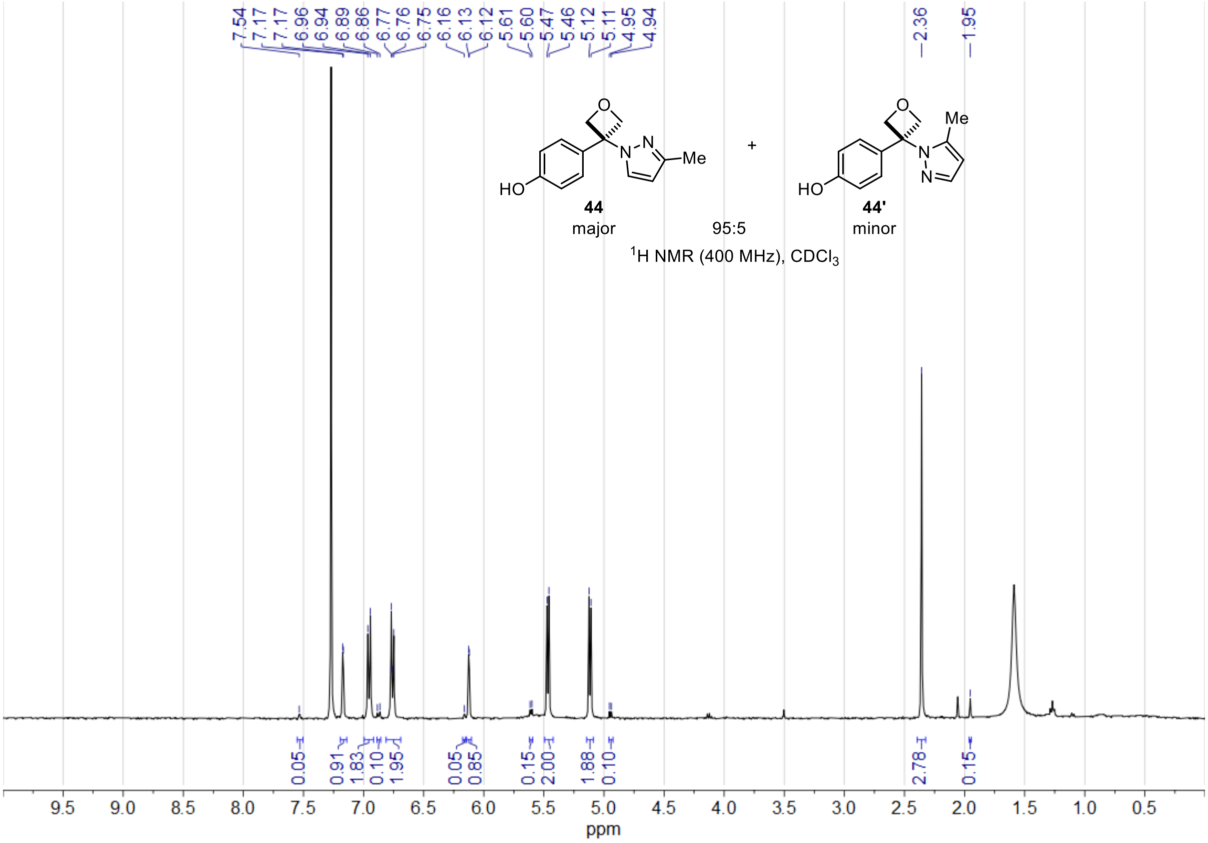

7.54 7.17 7.17 6.96 6.94 6.89 6.86 6.77 6.76 6.75 6.16 6.13 6.12 5.61 5.60 5.47 5.46 5.12 5.11 4.95 4.94
2.36
1.95
HO
O
N
N
Me
+
Me
44
major
95:5
44'
minor
¹H NMR (400 MHz), CDCl3
0.05 0.91 1.83 0.10 1.95 0.05 0.85 0.15 2.00 1.88 0.10 2.78 0.15
9.5 9.0 8.5 8.0 7.5 7.0 6.5 6.0 5.5 5.0 4.5 4.0 3.5 3.0 2.5 2.0 1.5 1.0 0.5
ppm

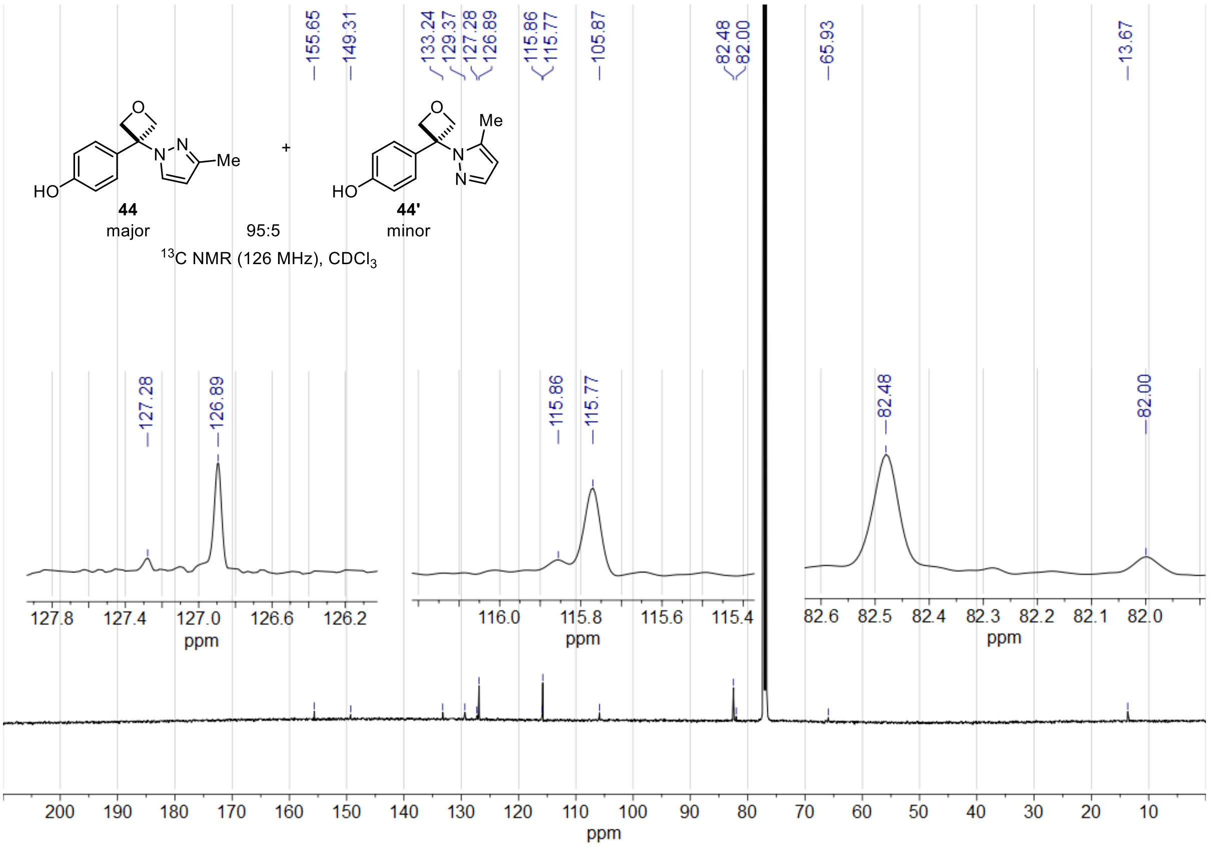

155.65
149.31
133.24 129.37 127.28 126.89
115.86 115.77
105.87
82.48 82.00
65.93
13.67
HO
O
N
N
Me
+
44
major
95:5
44'
minor
¹³C NMR (126 MHz), CDCl3
127.28 126.89
127.8 127.4 127.0 126.6 126.2
ppm
115.86 115.77
116.0 115.8 115.6 115.4
ppm
82.48 82.00
82.6 82.5 82.4 82.3 82.2 82.1 82.0
ppm
200 190 180 170 160 150 140 130 120 110 100 90 80 70 60 50 40 30 20 10
ppm

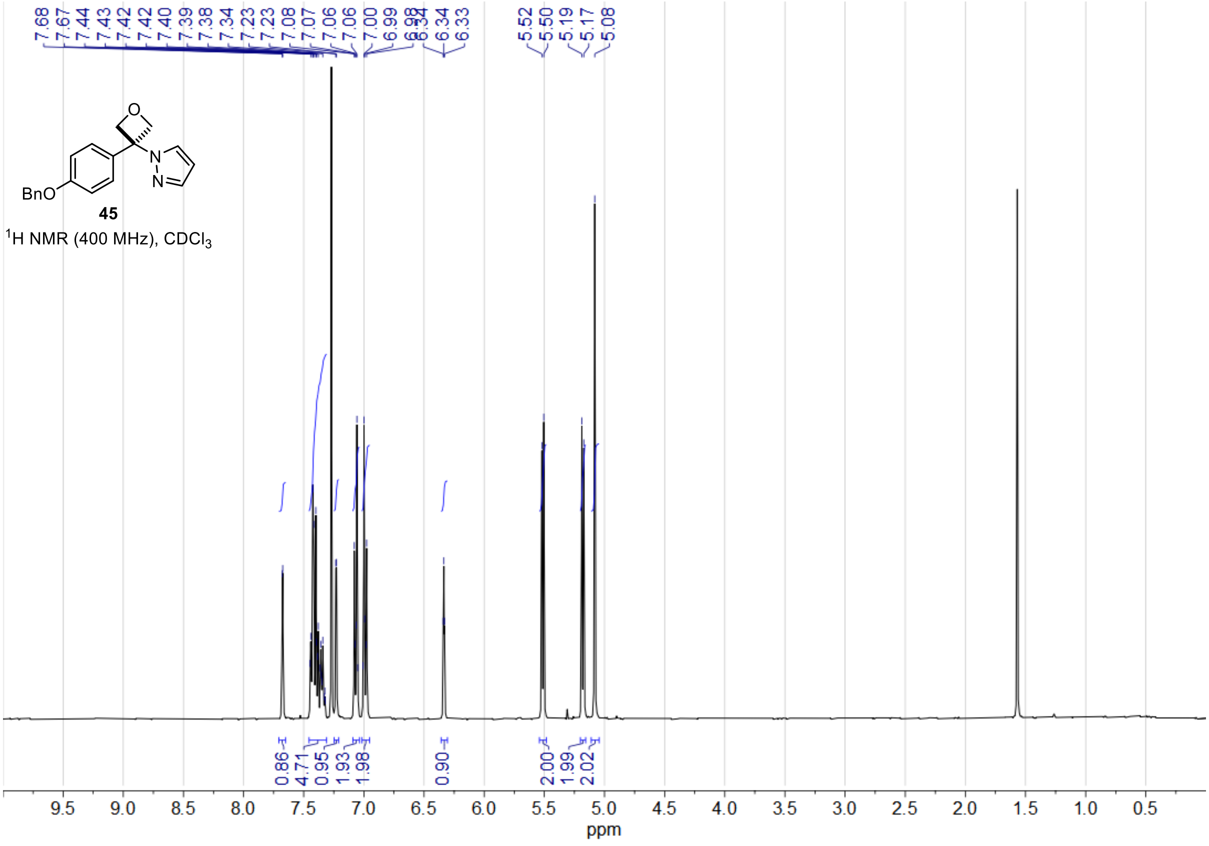
7.68
7.67
7.44
7.43
7.42
7.42
7.40
7.39
7.38
7.34
7.23
7.23
7.08
7.07
7.06
7.06
7.00
6.99
6.98
6.34
6.34
6.33
5.52
5.50
5.19
5.17
5.08
BnO
O
N
N
45
^{1}H NMR (400 MHz), CDCl$_3$
0.86
4.71
0.95
1.93
1.98
0.90
2.00
1.99
2.02
9.5
9.0
8.5
8.0
7.5
7.0
6.5
6.0
5.5
5.0
4.5
4.0
3.5
3.0
2.5
2.0
1.5
1.0
0.5
ppm

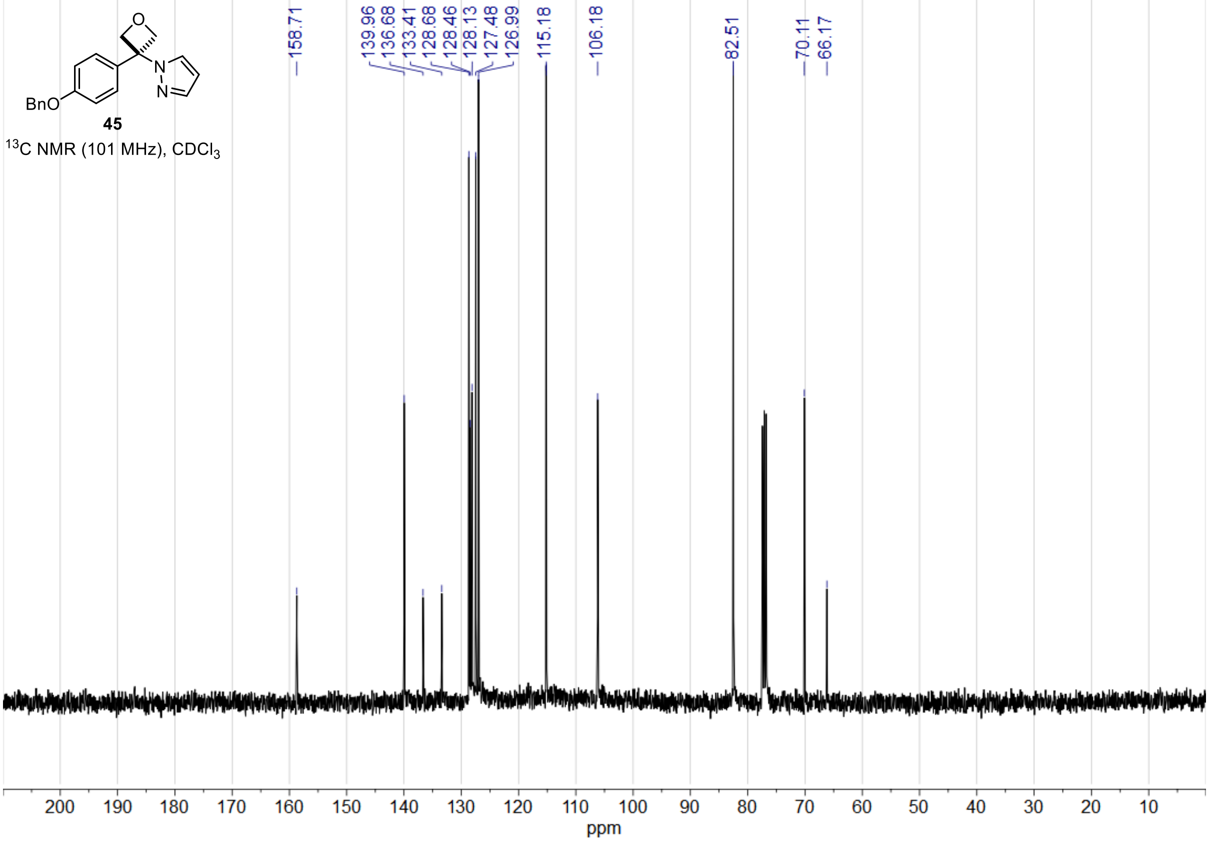
158.71
139.96
136.68
133.41
128.68
128.46
128.13
127.48
126.99
115.18
106.18
82.51
70.11
66.17
BnO
O
N
N
45
^{13}C NMR (101 MHz), CDCl$_3$
200
190
180
170
160
150
140
130
120
110
100
90
80
70
60
50
40
30
20
10
ppm

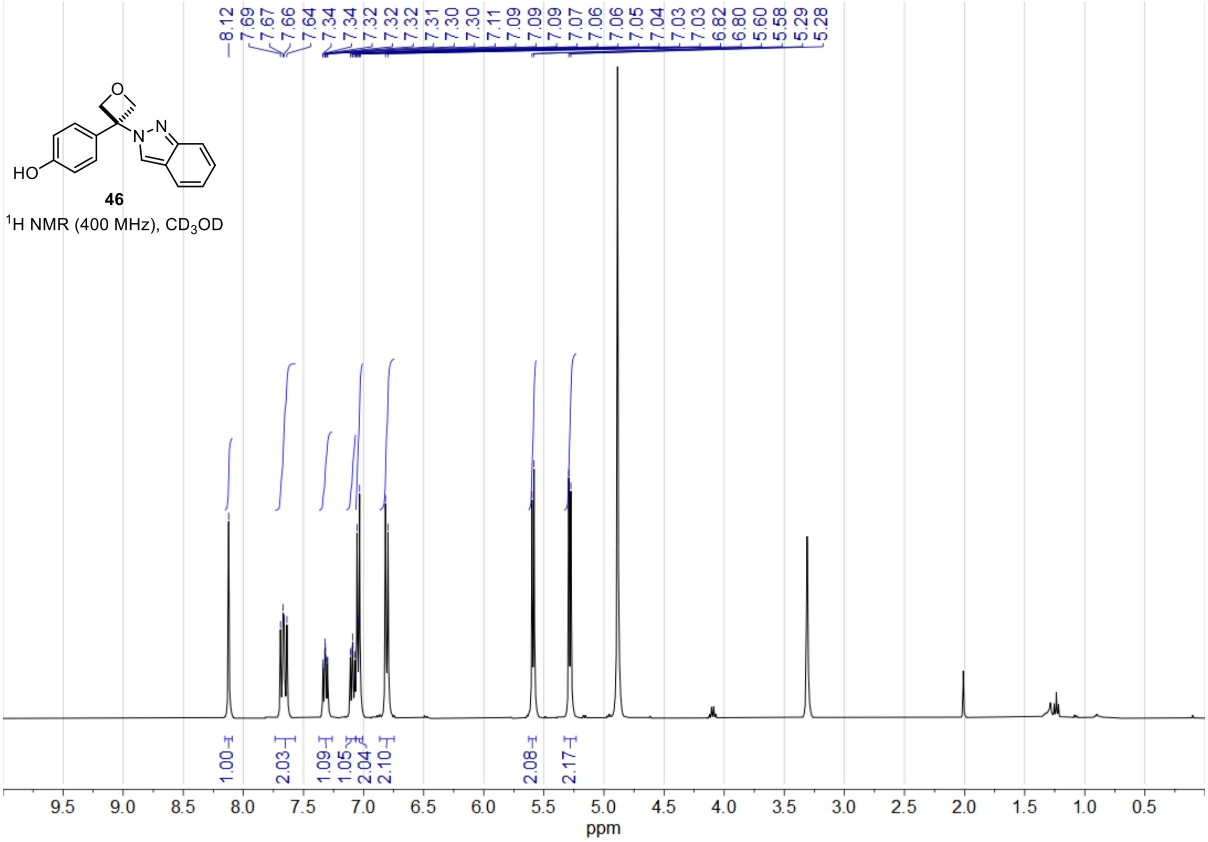

46

^{1}H NMR (400 MHz), CD_3OD

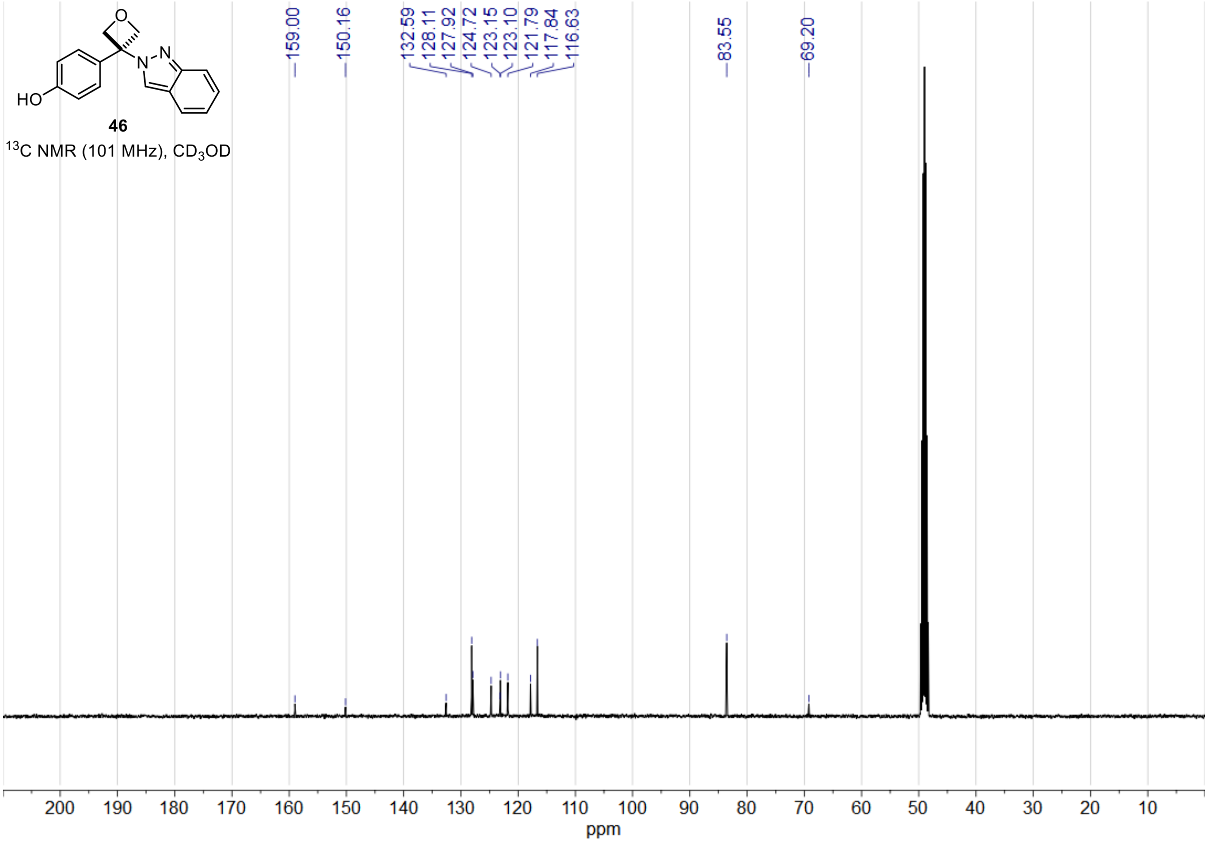

46

^{13}C NMR (101 MHz), CD_3OD

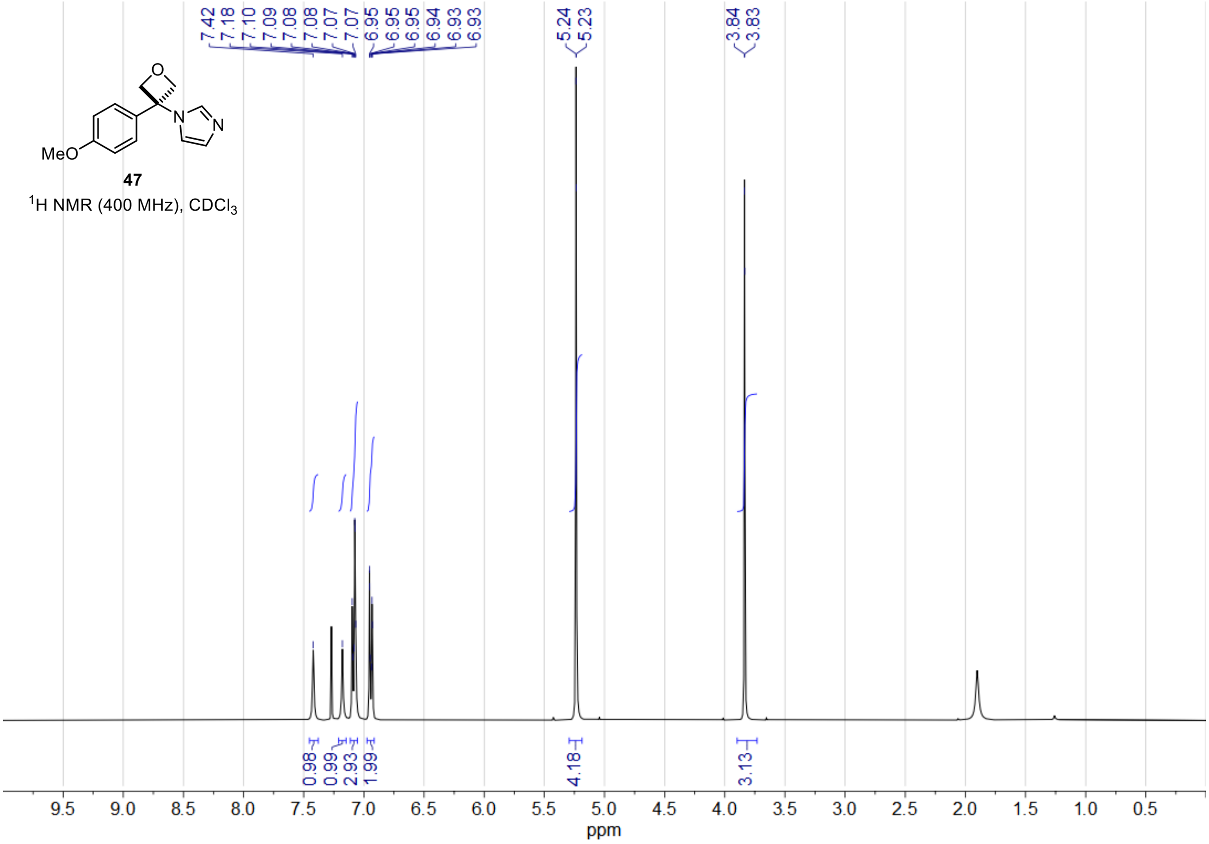

7.42
7.18
7.10
7.09
7.08
7.08
7.07
7.07
6.95
6.95
6.95
6.94
6.93
6.93
5.24
5.23
3.84
3.83
MeO
O
N
N
47
^{1}H NMR (400 MHz), $CDCl_3$
0.98
0.99
2.93
1.99
4.18
3.13
9.5
9.0
8.5
8.0
7.5
7.0
6.5
6.0
5.5
5.0
4.5
4.0
3.5
3.0
2.5
2.0
1.5
1.0
0.5
ppm

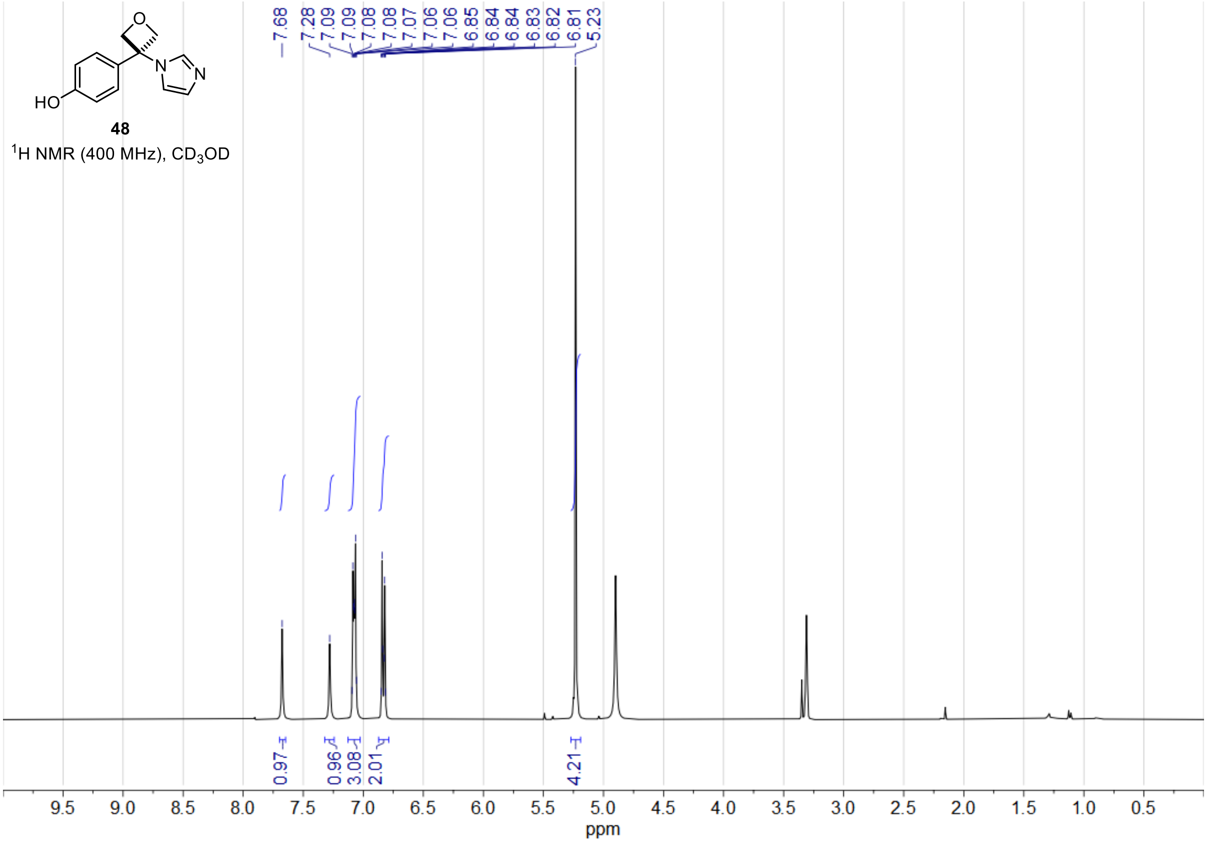

48

^{1}H NMR (400 MHz), CD_3OD

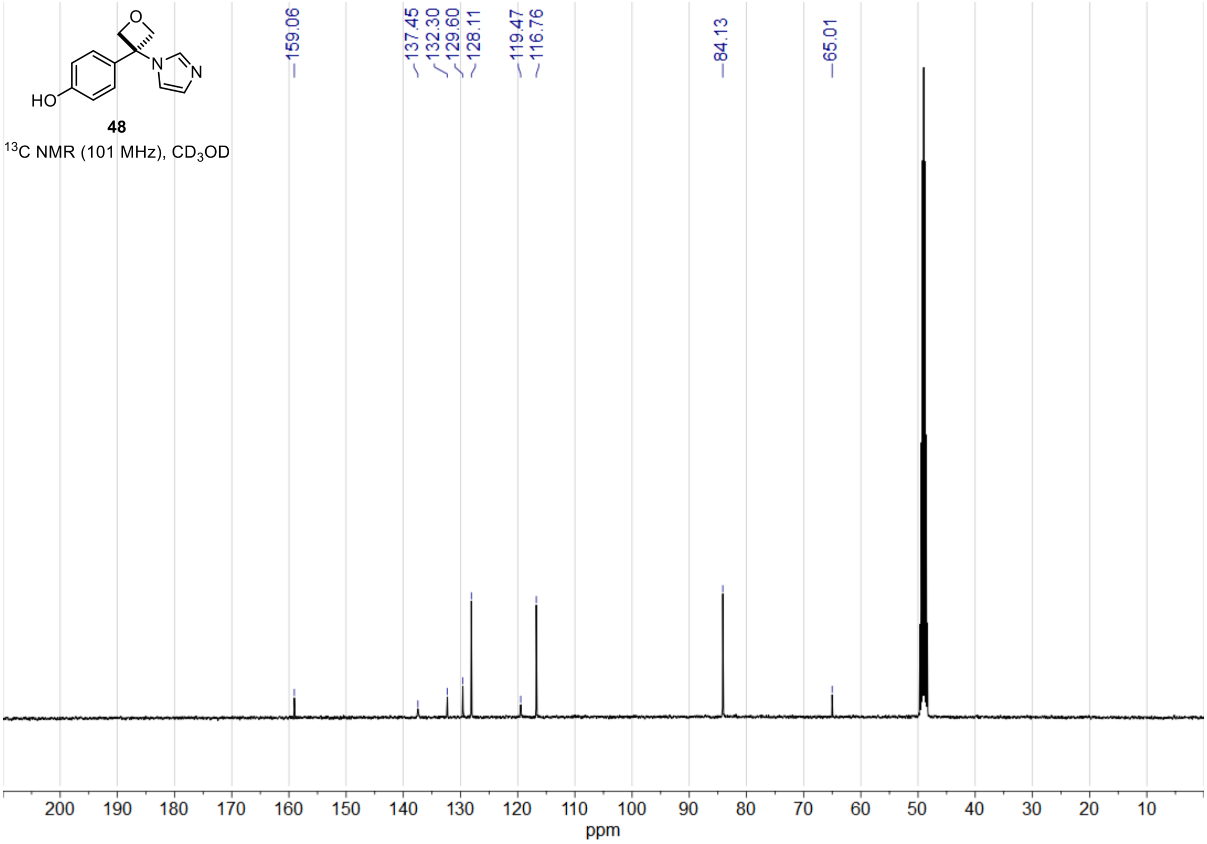

48

^{13}C NMR (101 MHz), CD_3OD

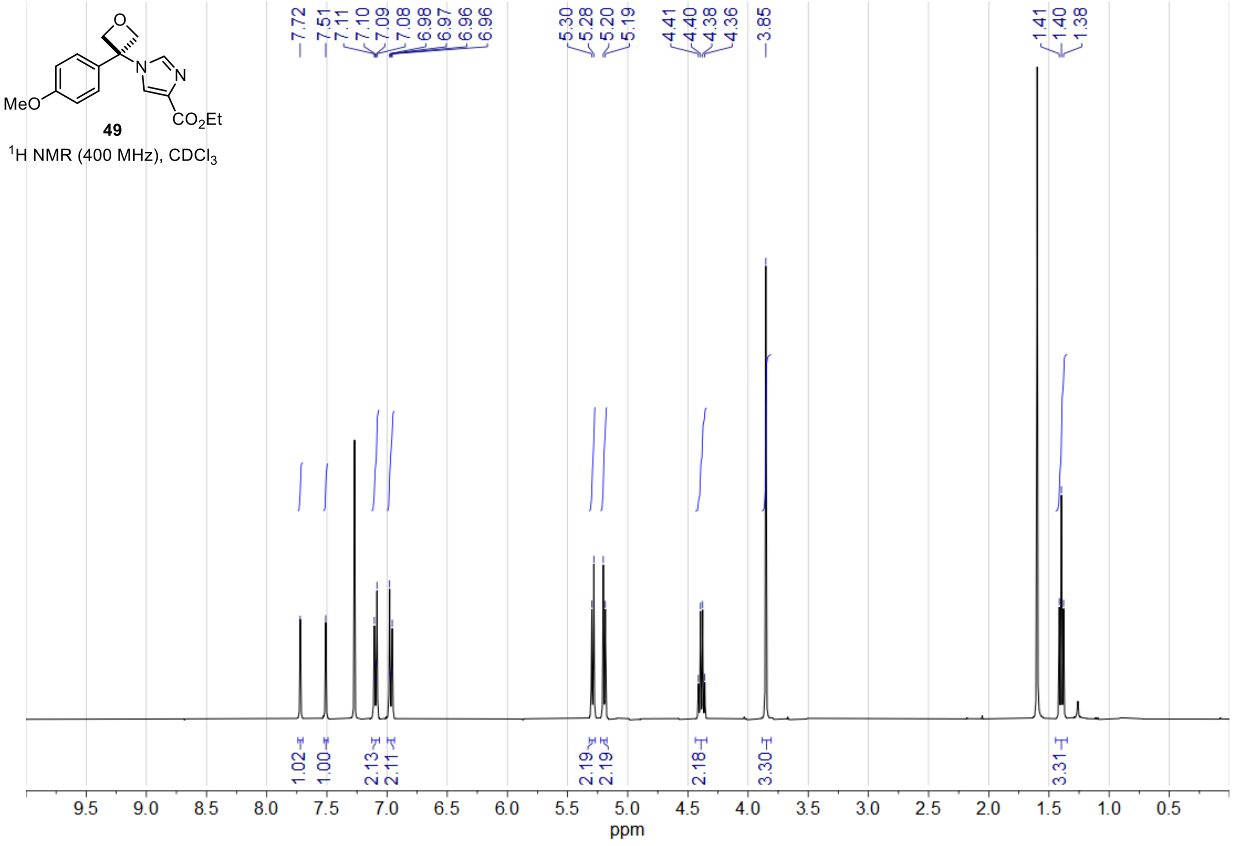
MeO
CO2Et
49
1H NMR (400 MHz), CDCl3
7.72
7.51
7.11
7.10
7.09
7.08
6.98
6.97
6.96
6.96
5.30
5.28
5.20
5.19
4.41
4.40
4.38
4.36
3.85
1.41
1.40
1.38
1.02
1.00
2.13
2.11
2.19
2.19
2.18
3.30
3.31
ppm

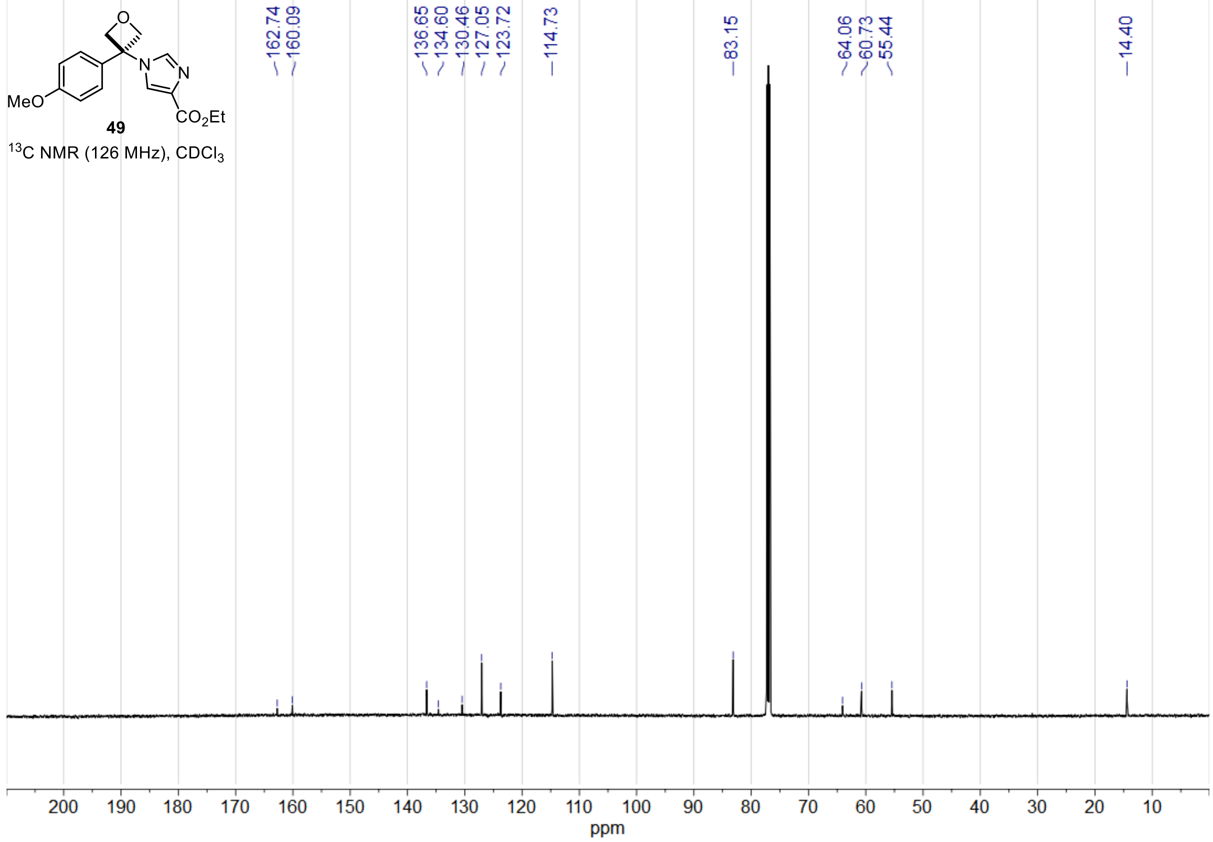
MeO
CO2Et
49
13C NMR (126 MHz), CDCl3
162.74
160.09
136.65
134.60
130.46
127.05
123.72
114.73
83.15
64.06
60.73
55.44
14.40
ppm

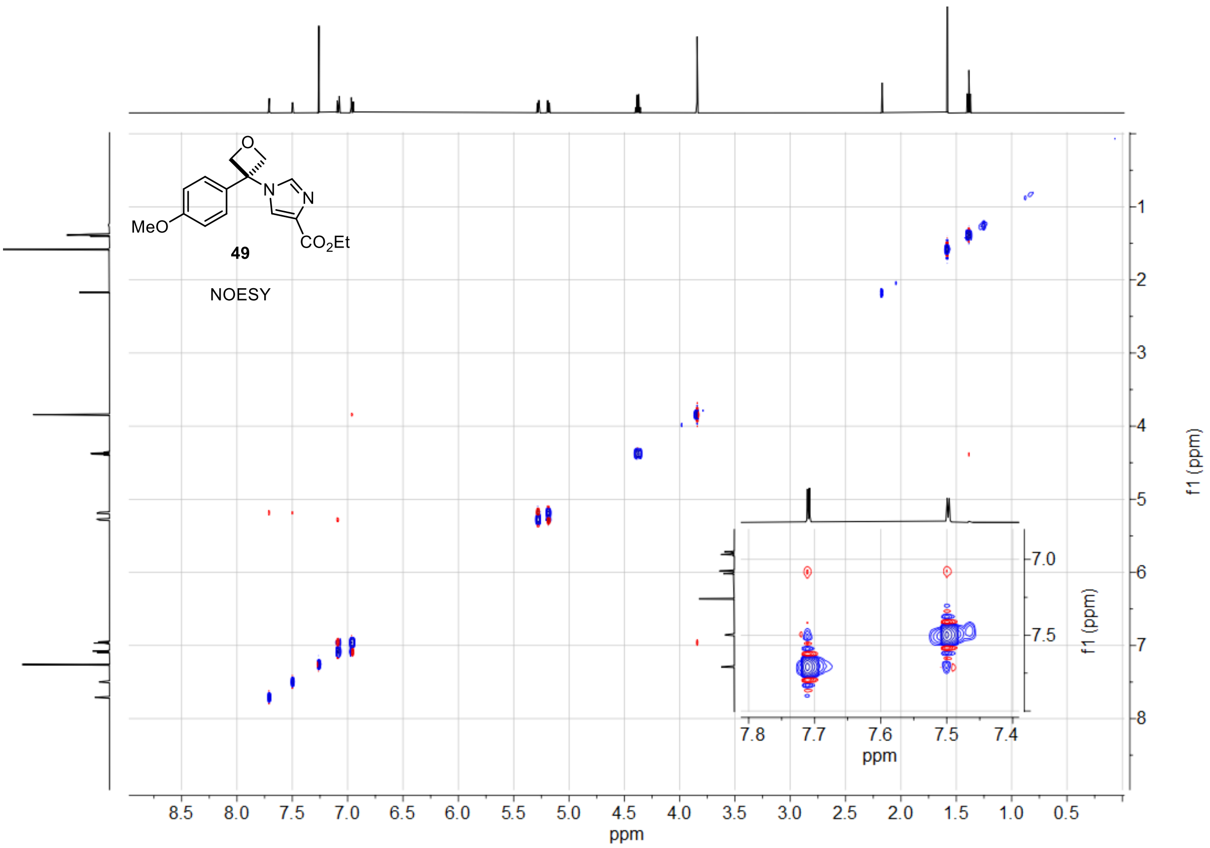
MeO
N
N
O
CO2Et
49
NOESY
8.5
8.0
7.5
7.0
6.5
6.0
5.5
5.0
4.5
4.0
3.5
3.0
2.5
2.0
1.5
1.0
0.5
ppm
f1 (ppm)
7.8
7.7
7.6
7.5
7.4
7.0
7.5

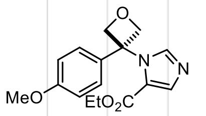

49'

^{1}H NMR (400 MHz), $CDCl_3$

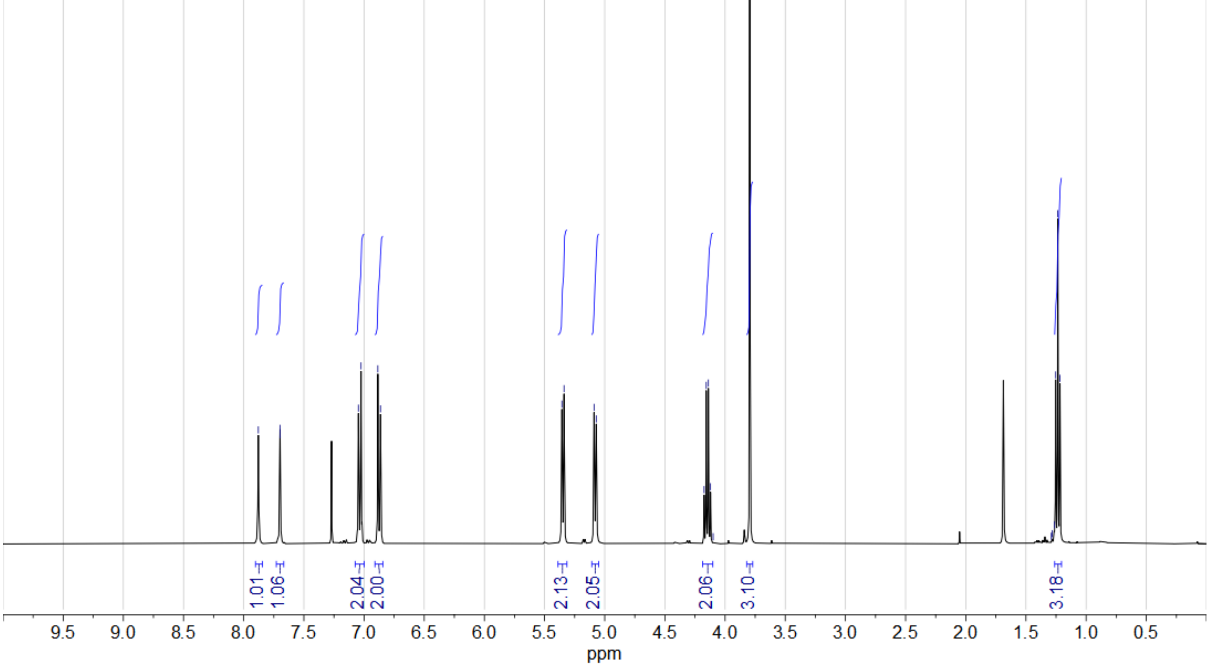

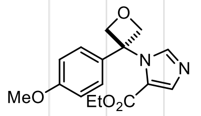

49'

^{13}C NMR (101 MHz), $CDCl_3$

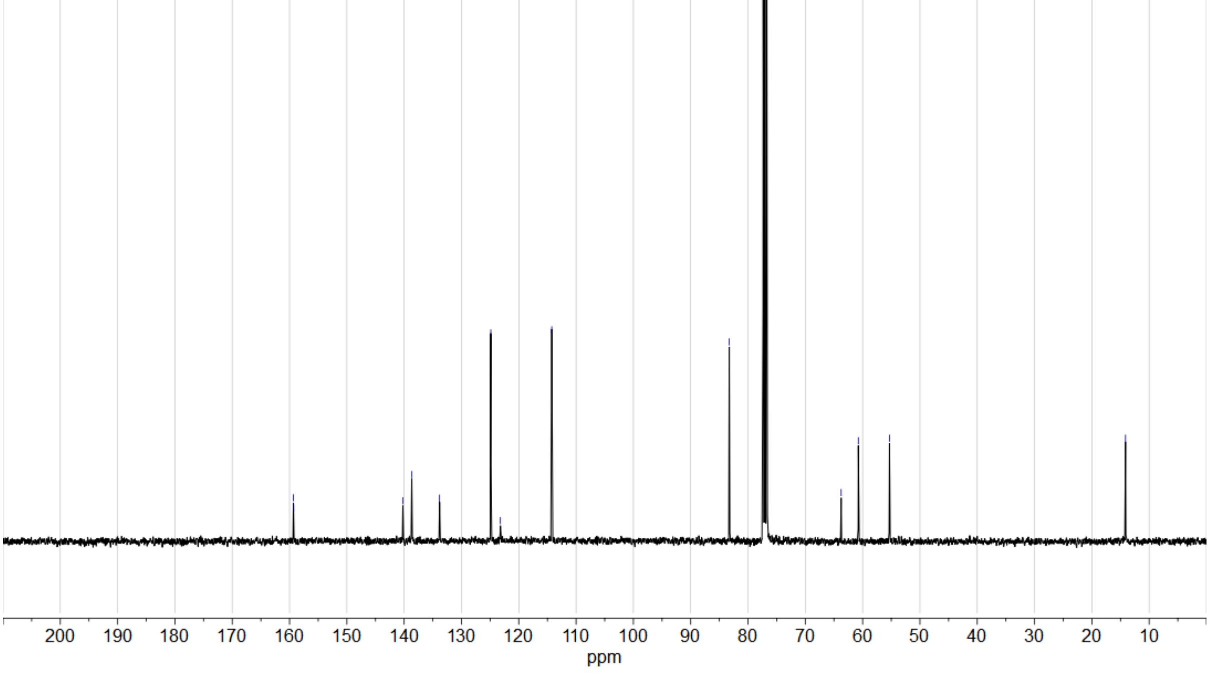

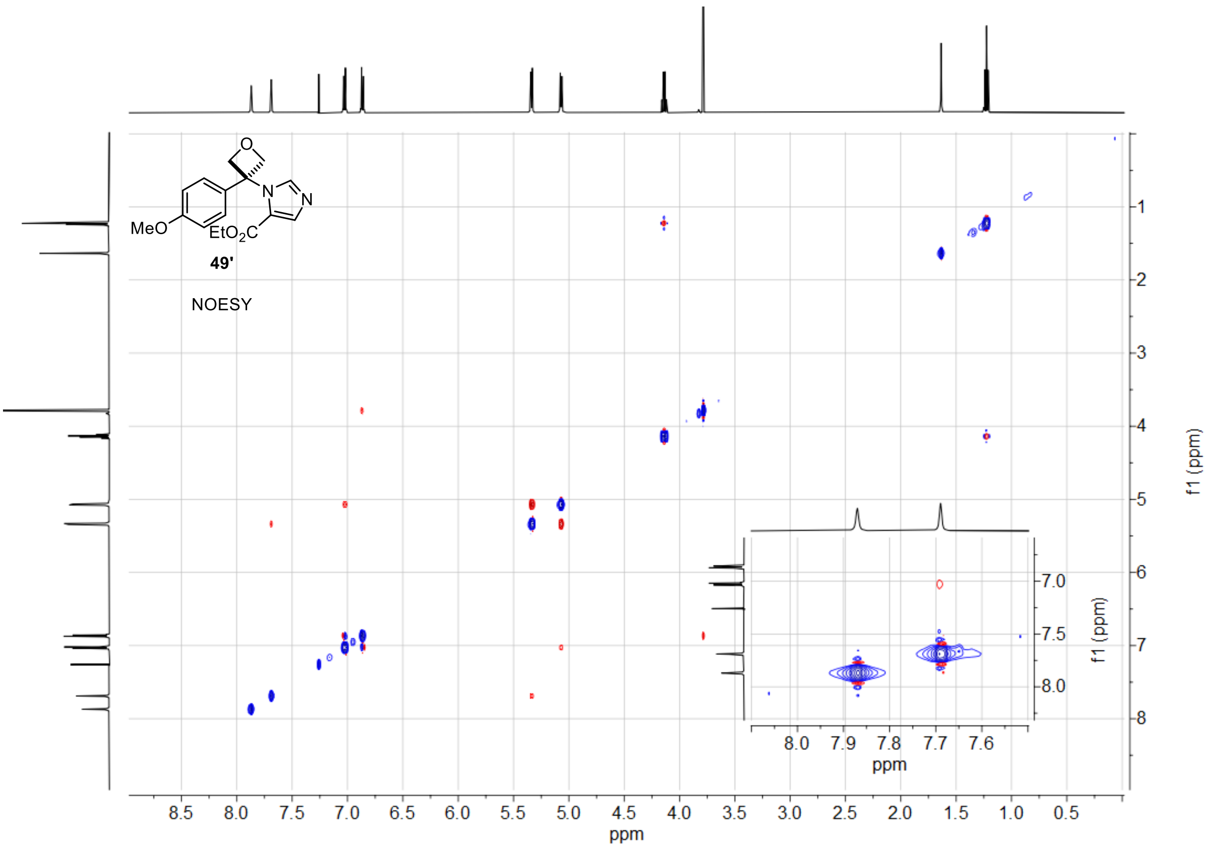
MeO
EtO2C
N
N
O
49'
NOESY
ppm
f1 (ppm)

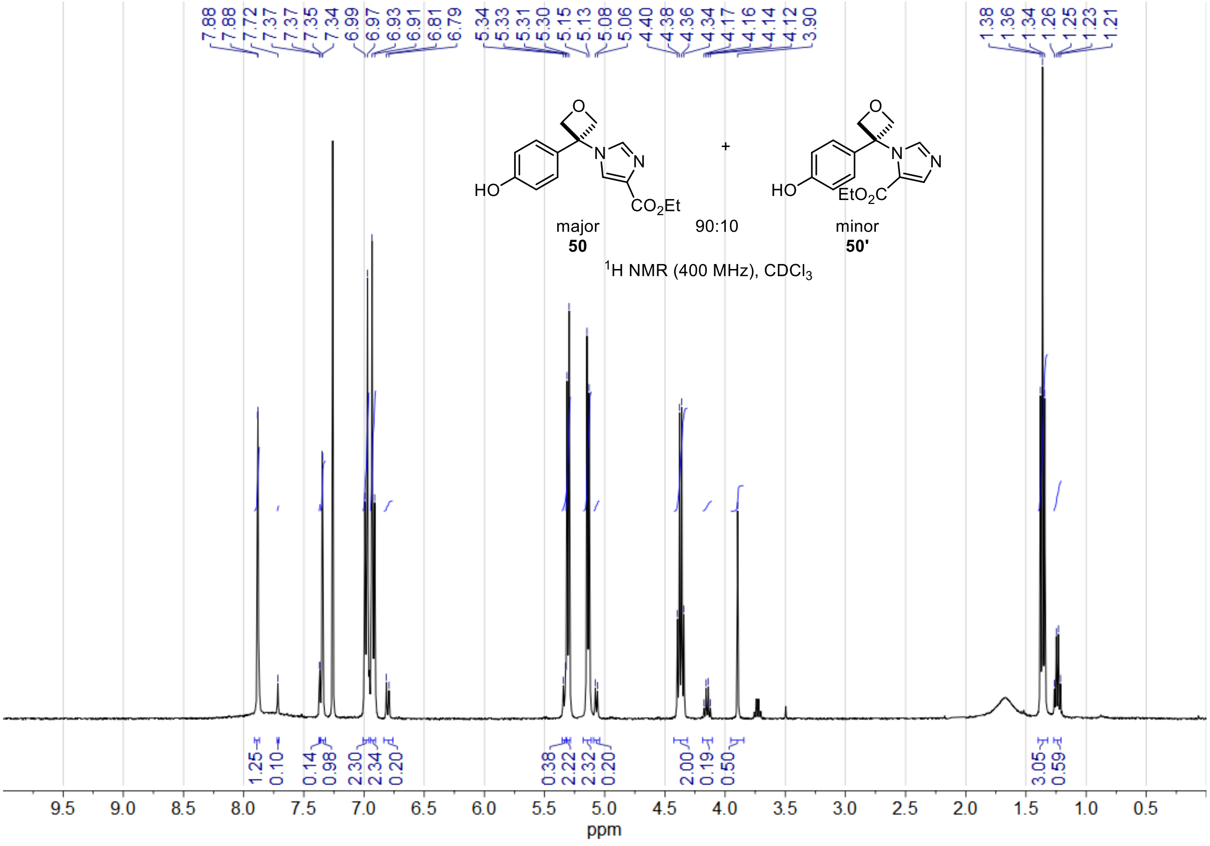

7.88 7.88 7.72 7.37 7.37 7.35 7.34 6.99 6.97 6.93 6.91 6.81 6.79 5.34 5.33 5.31 5.30 5.15 5.13 5.08 5.06 4.40 4.38 4.36 4.34 4.17 4.16 4.14 4.12 3.90 1.38 1.36 1.34 1.26 1.25 1.23 1.21
HO
N
N
O
CO2Et
major
50
+
90:10
EtO2C
minor
50'
1H NMR (400 MHz), CDCl3
1.25 0.10 0.14 0.98 2.30 2.34 0.20 0.38 2.22 2.32 0.20 2.00 0.19 0.50 3.05 0.59
9.5 9.0 8.5 8.0 7.5 7.0 6.5 6.0 5.5 5.0 4.5 4.0 3.5 3.0 2.5 2.0 1.5 1.0 0.5
ppm

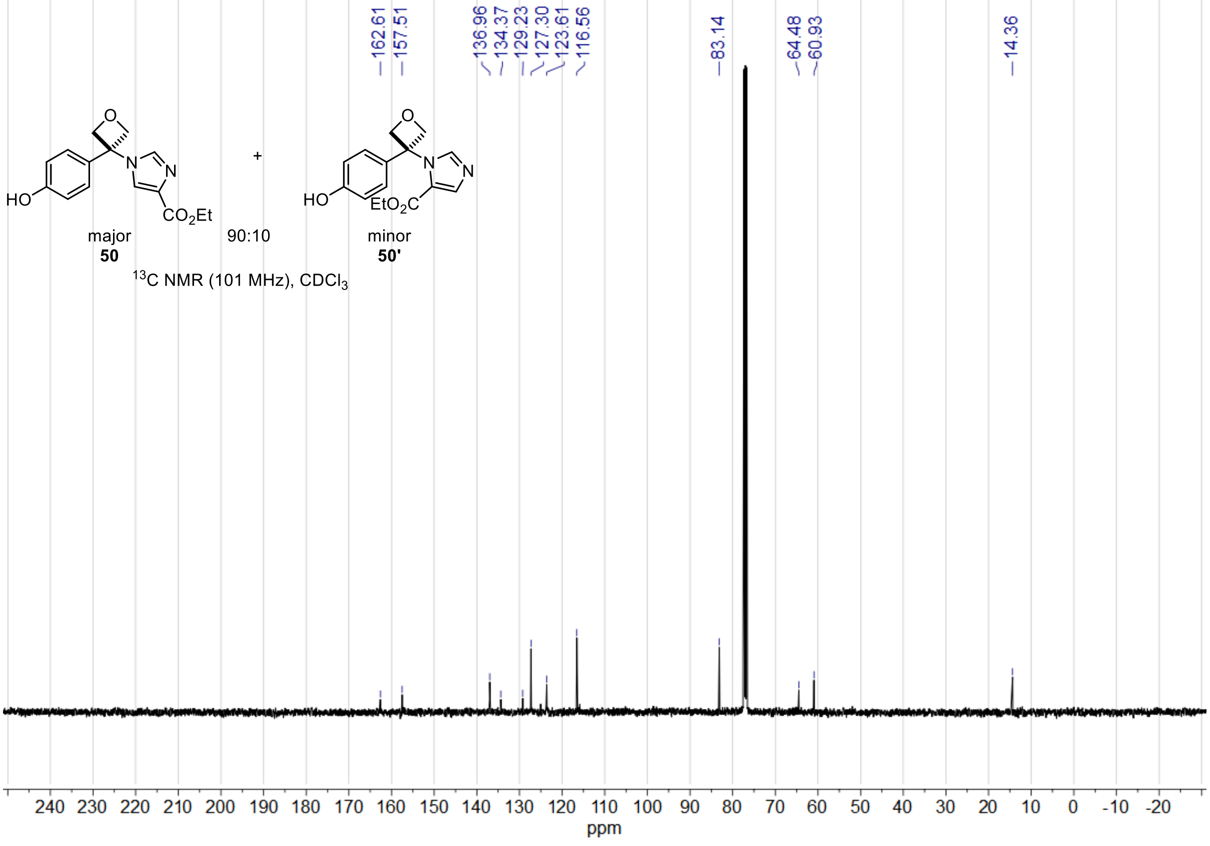

162.61 157.51 136.96 134.37 129.23 127.30 123.61 116.56 83.14 64.48 60.93 14.36
HO
N
N
O
CO2Et
major
50
+
90:10
EtO2C
minor
50'
13C NMR (101 MHz), CDCl3
240 230 220 210 200 190 180 170 160 150 140 130 120 110 100 90 80 70 60 50 40 30 20 10 0 -10 -20
ppm

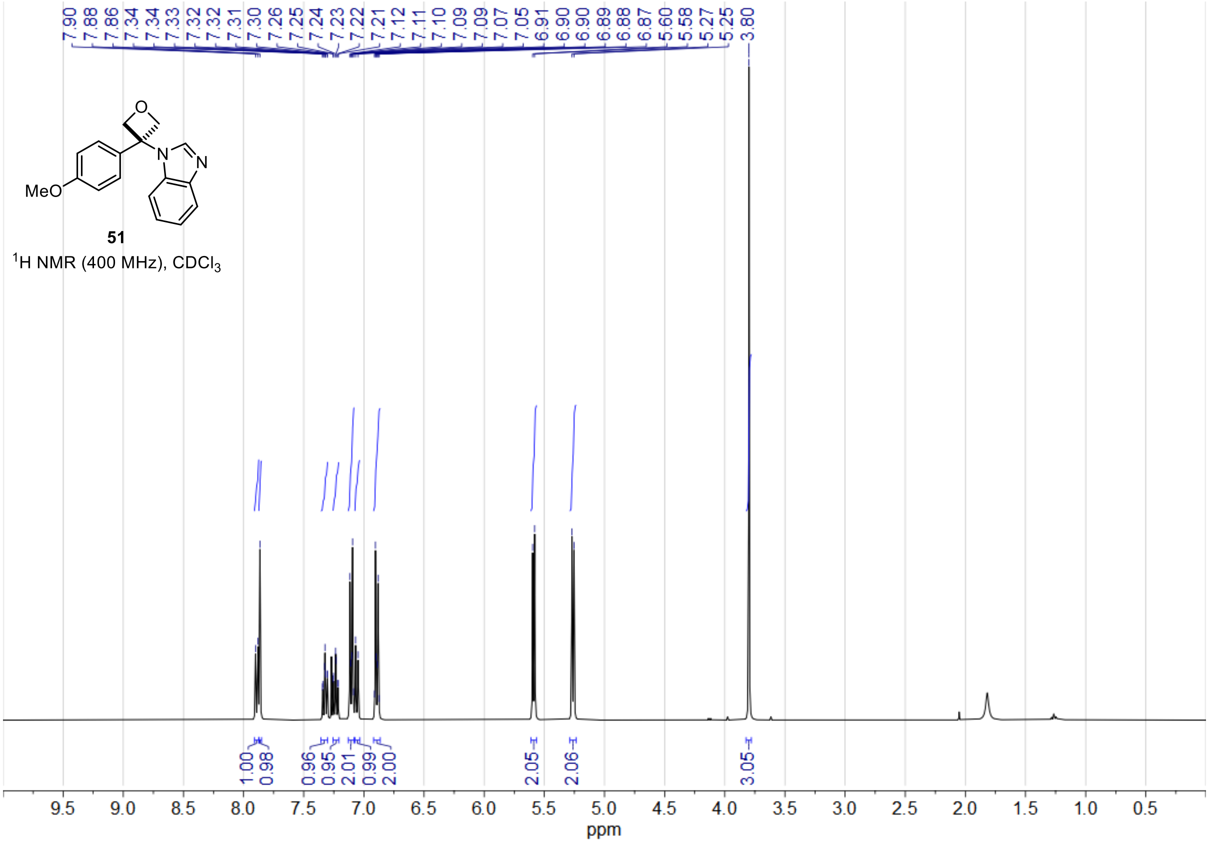

51

^{1}H NMR (400 MHz), $CDCl_3$

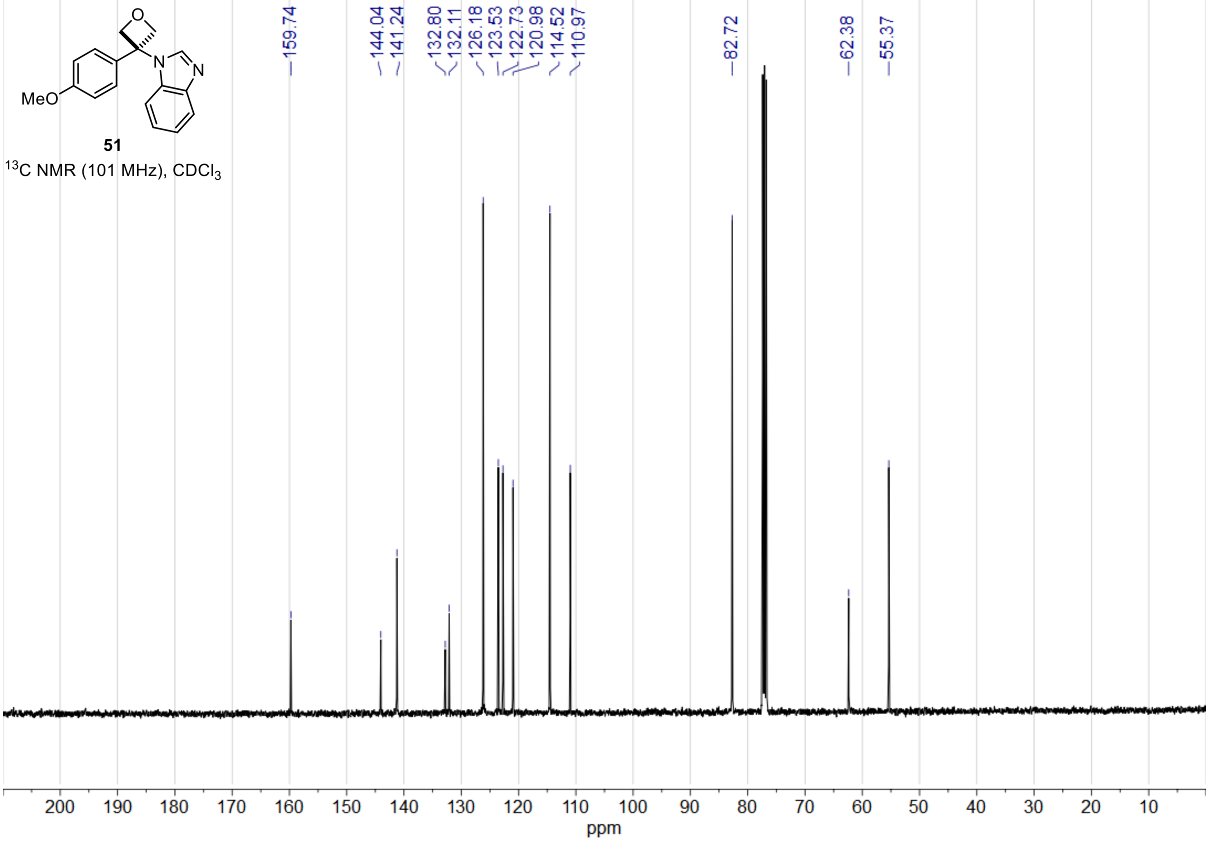

51

^{13}C NMR (101 MHz), $CDCl_3$

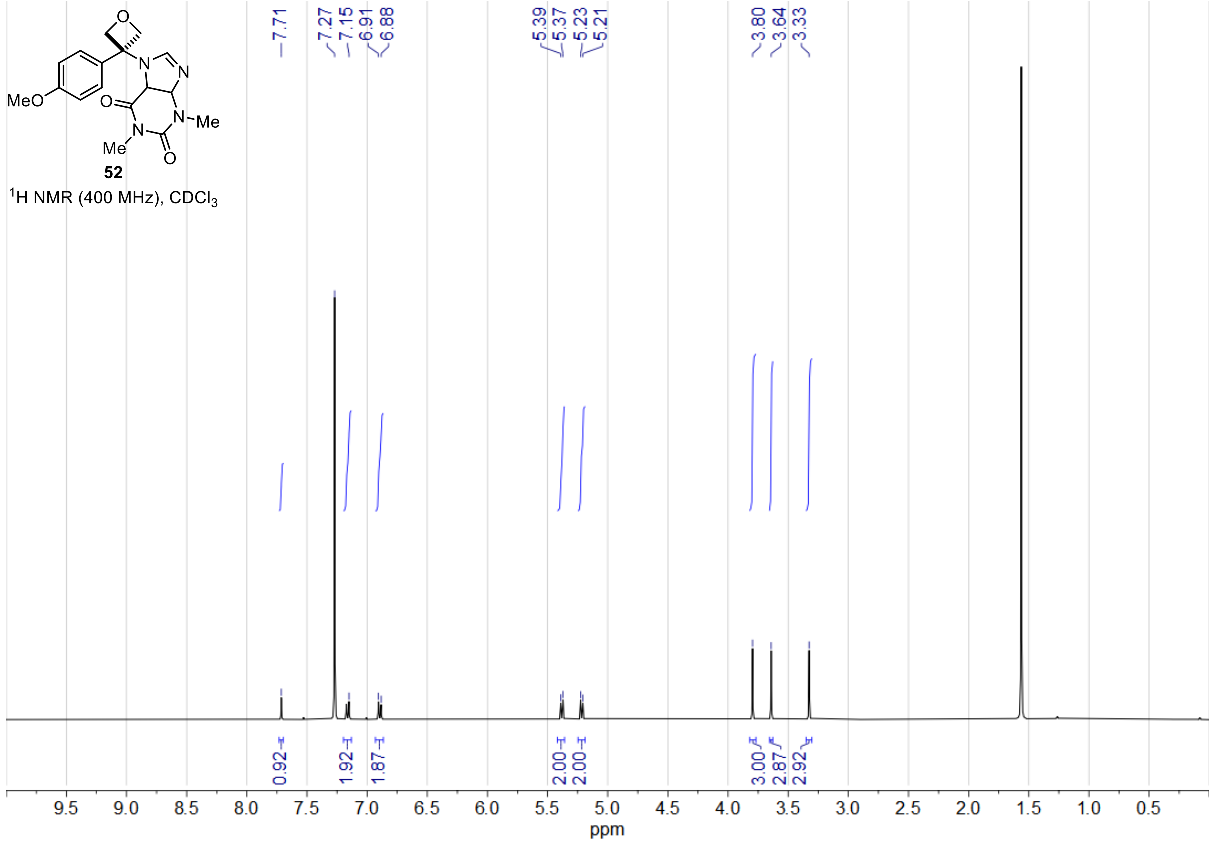

52

^{1}H NMR (400 MHz), $CDCl_3$

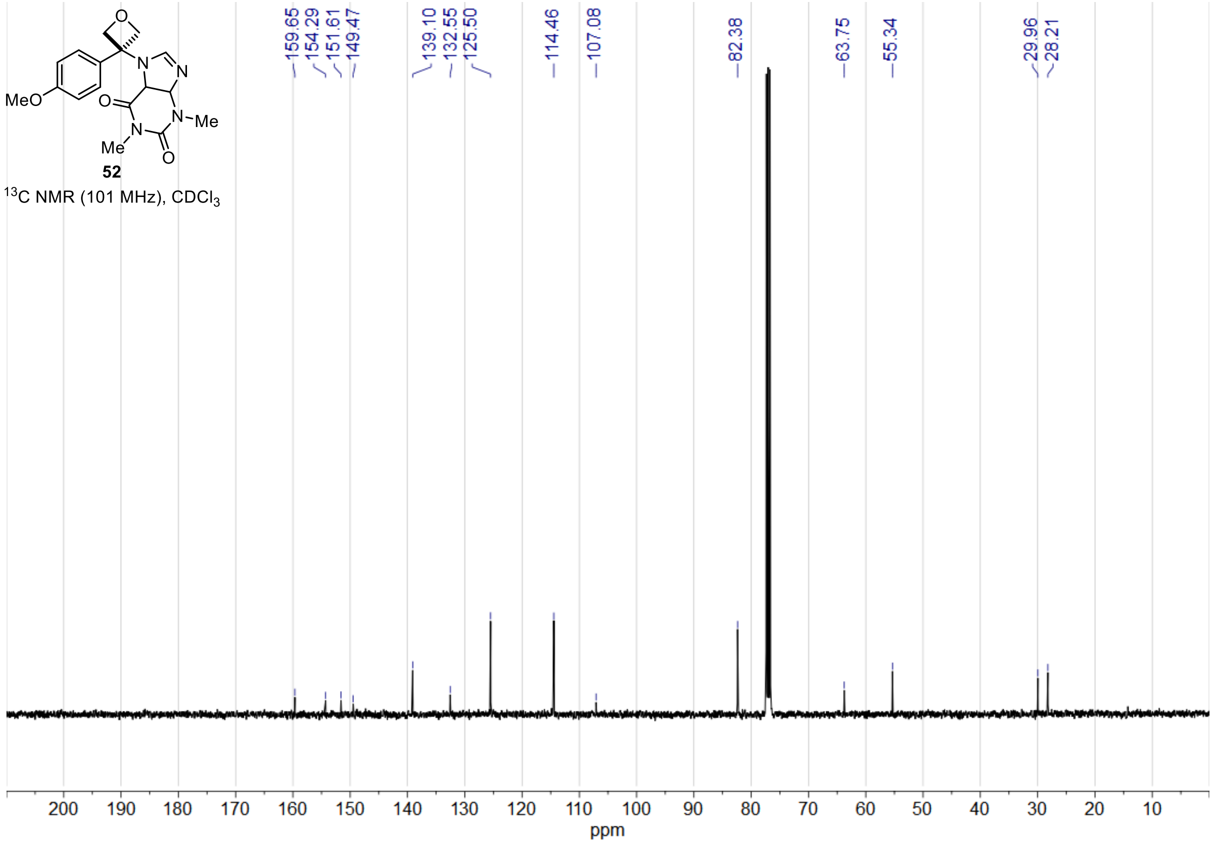

52

^{13}C NMR (101 MHz), $CDCl_3$

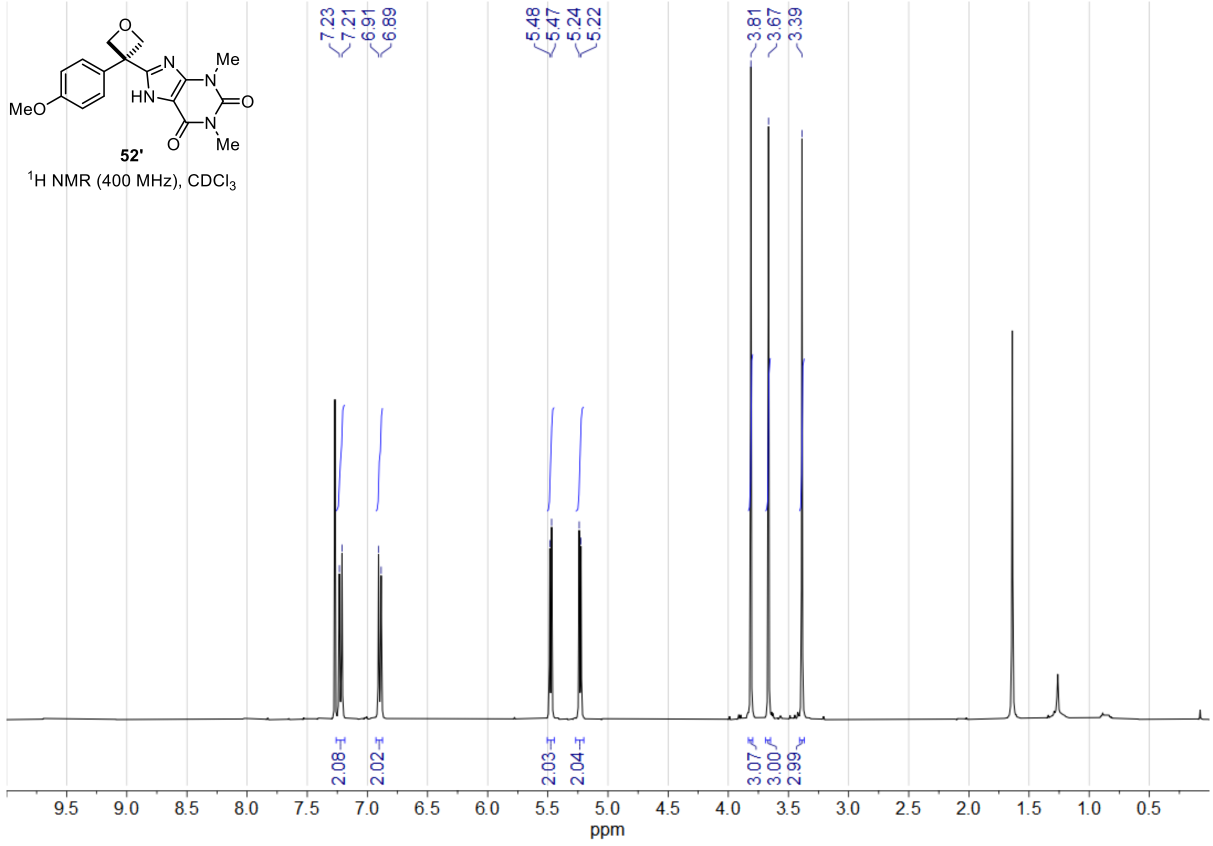
MeO
HN
N
N
Me
O
N
Me
O
O
52'
^{1}H NMR (400 MHz), CDCl3
7.23
7.21
6.91
6.89
5.48
5.47
5.24
5.22
3.81
3.67
3.39
2.08
2.02
2.03
2.04
3.07
3.00
2.99
9.5 9.0 8.5 8.0 7.5 7.0 6.5 6.0 5.5 5.0 4.5 4.0 3.5 3.0 2.5 2.0 1.5 1.0 0.5
ppm

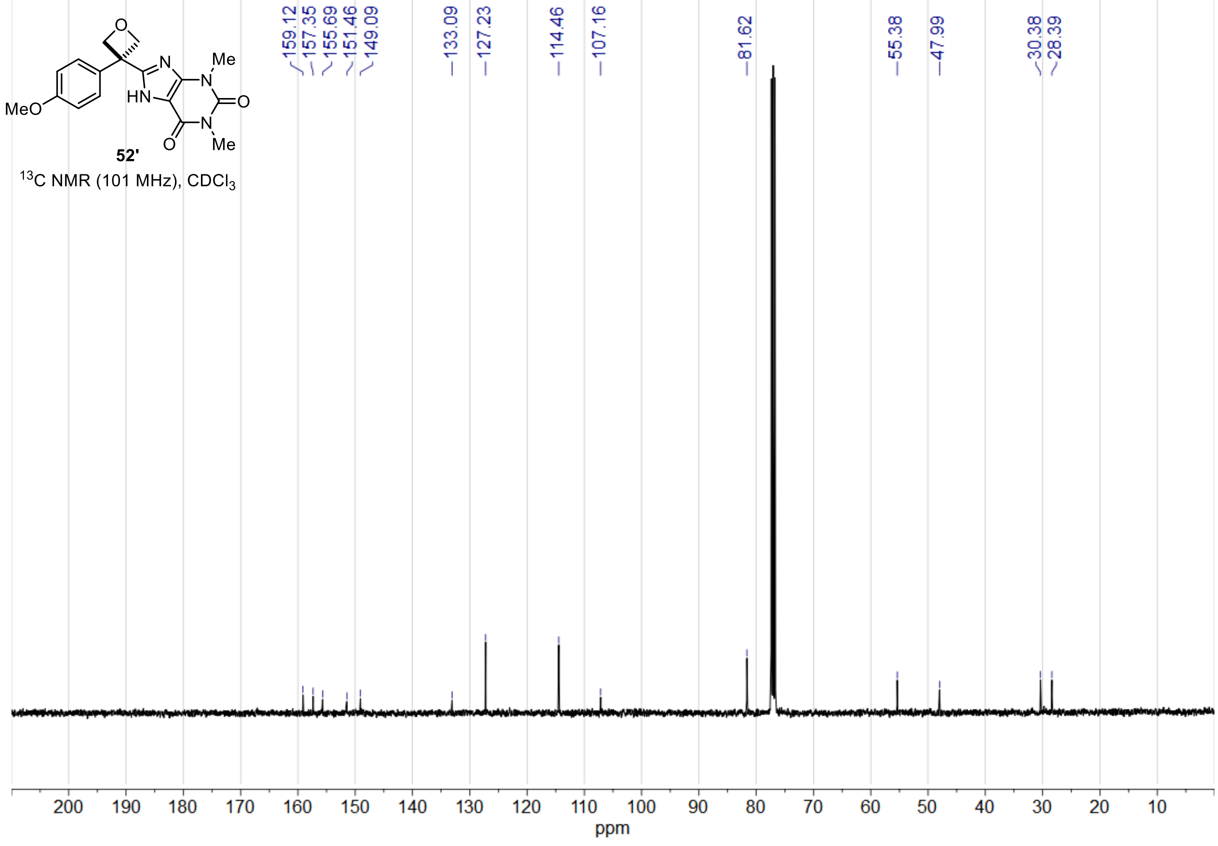
MeO
HN
N
N
Me
O
N
Me
O
O
52'
^{13}C NMR (101 MHz), CDCl3
159.12
157.35
155.69
151.46
149.09
133.09
127.23
114.46
107.16
81.62
55.38
47.99
30.38
28.39
200 190 180 170 160 150 140 130 120 110 100 90 80 70 60 50 40 30 20 10
ppm

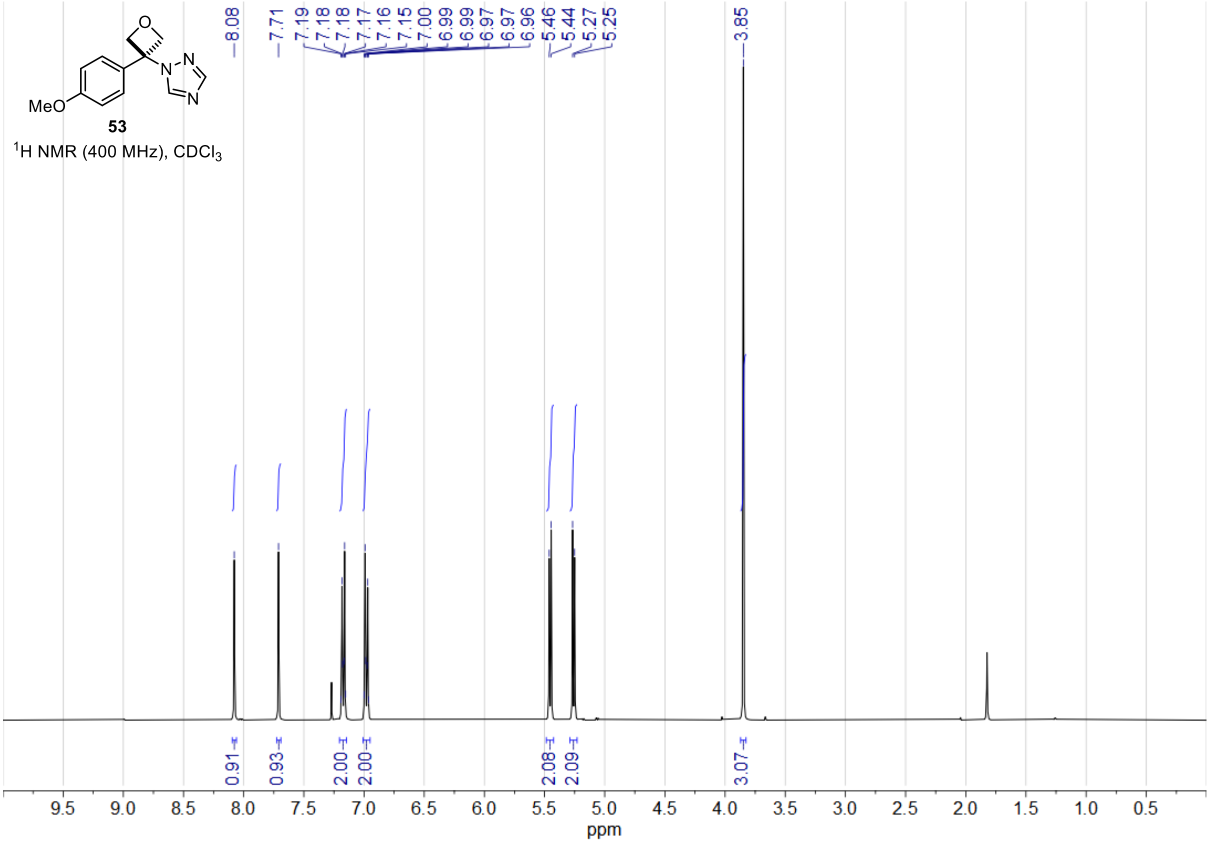

53

^{1}H NMR (400 MHz), $CDCl_3$

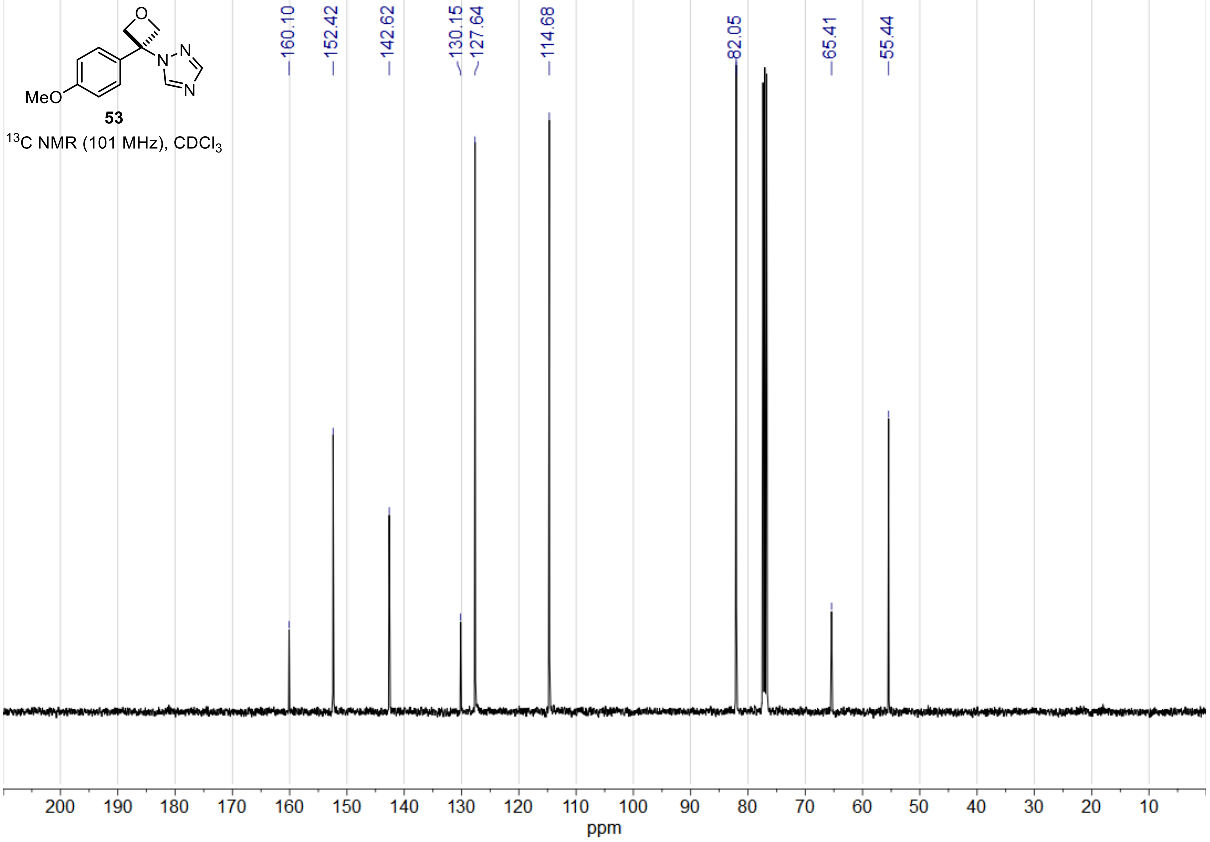

53

^{13}C NMR (101 MHz), $CDCl_3$

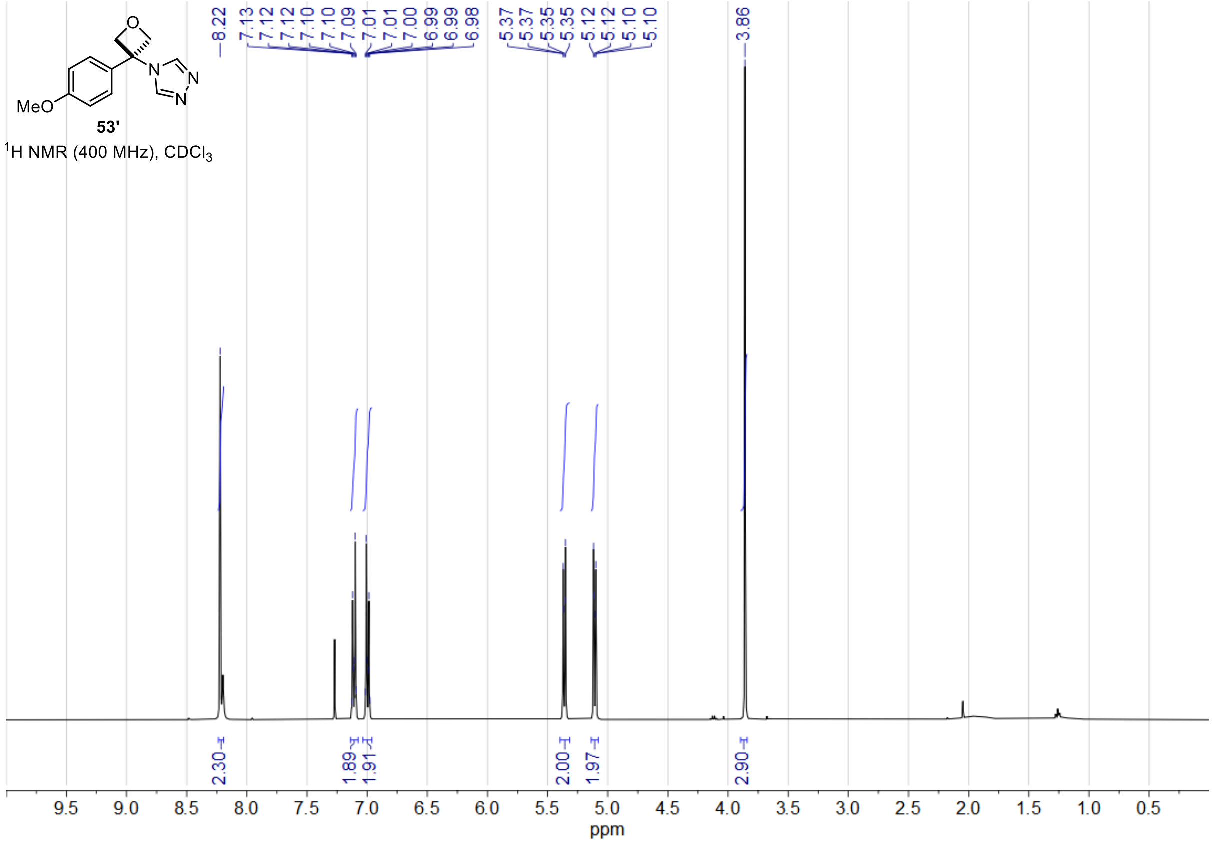
MeO
N
N
N
O
53'
1H NMR (400 MHz), CDCl3
8.22
7.13
7.12
7.12
7.10
7.10
7.09
7.01
7.01
7.00
6.99
6.99
6.98
5.37
5.37
5.35
5.35
5.12
5.12
5.10
5.10
3.86
2.30
1.89
1.91
2.00
1.97
2.90
9.5 9.0 8.5 8.0 7.5 7.0 6.5 6.0 5.5 5.0 4.5 4.0 3.5 3.0 2.5 2.0 1.5 1.0 0.5
ppm

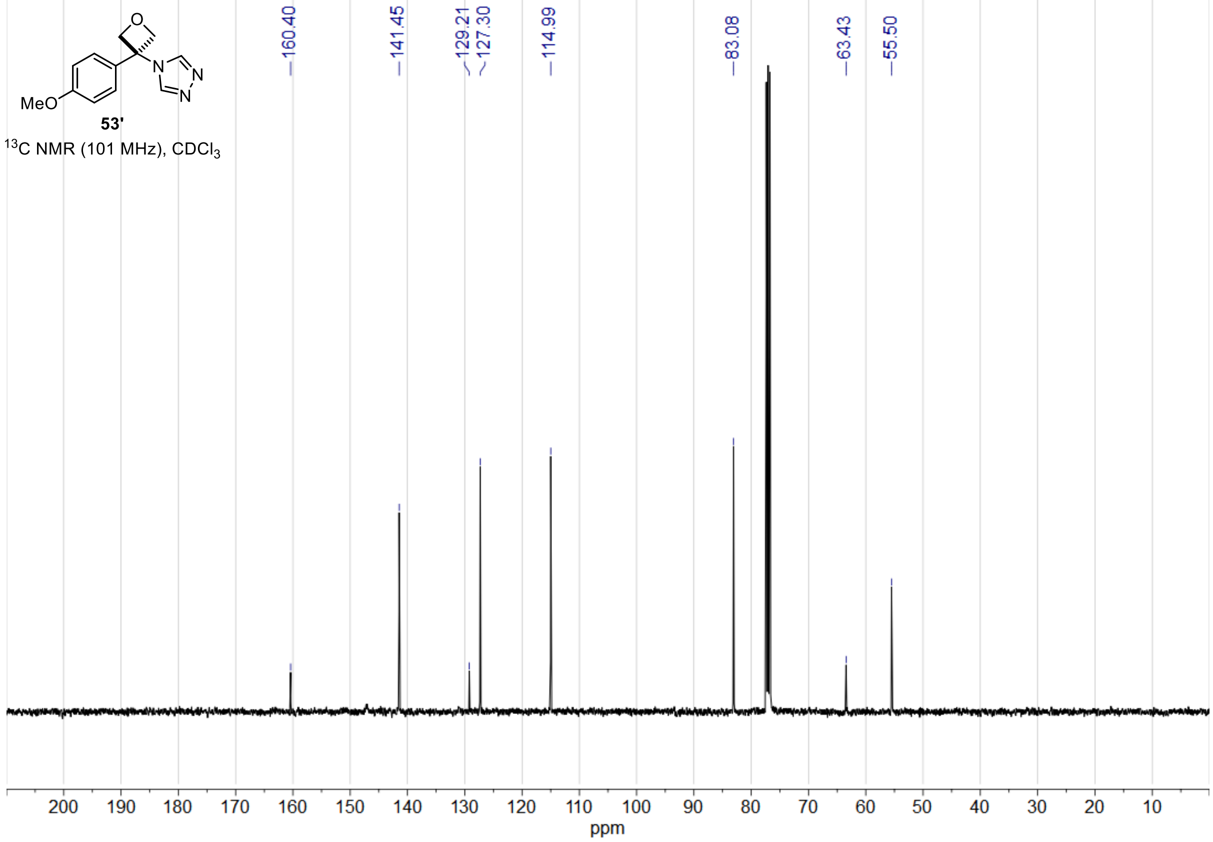
MeO
N
N
N
O
53'
13C NMR (101 MHz), CDCl3
160.40
141.45
129.21
127.30
114.99
83.08
63.43
55.50
200 190 180 170 160 150 140 130 120 110 100 90 80 70 60 50 40 30 20 10
ppm

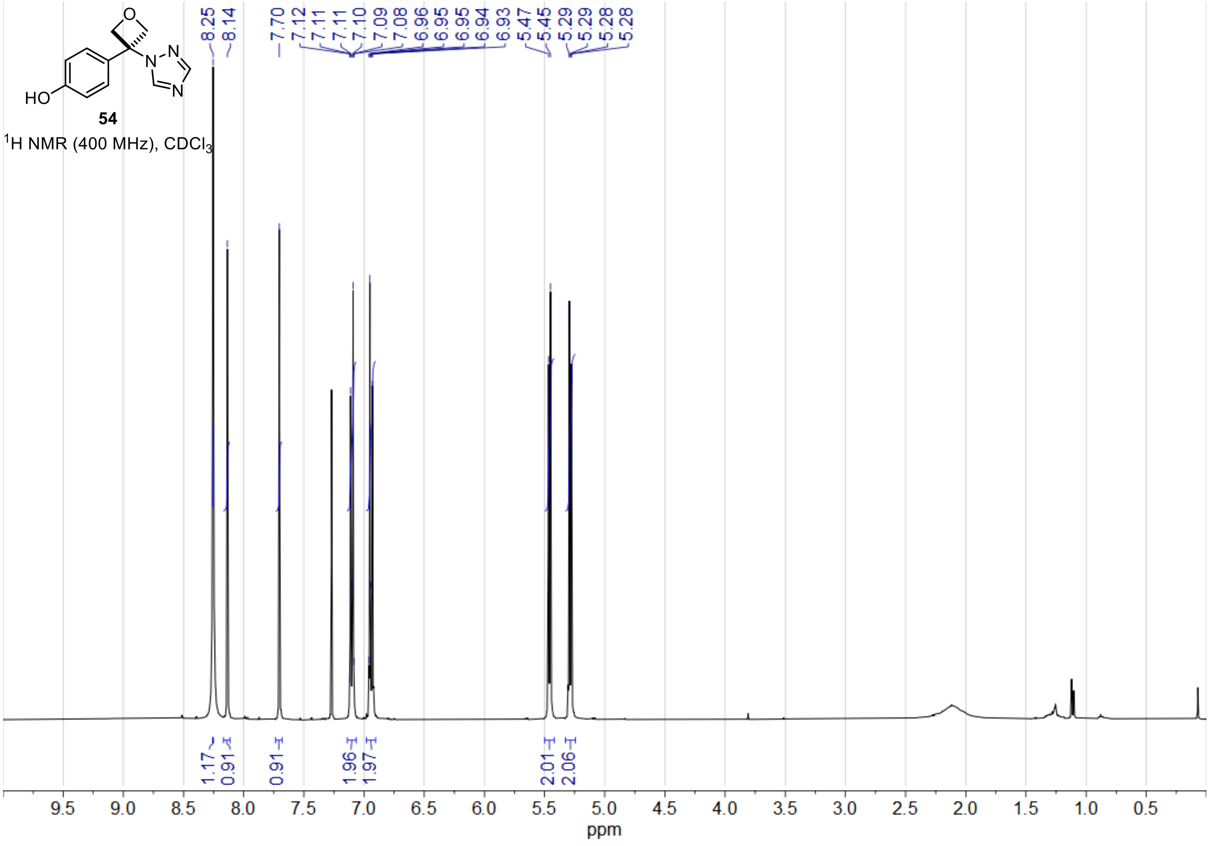
54
1H NMR (400 MHz), CDCl3
8.25
8.14
7.70
7.12
7.11
7.11
7.10
7.09
7.08
6.96
6.95
6.95
6.94
6.93
5.47
5.45
5.29
5.29
5.28
5.28
1.17
0.91
0.91
1.96
1.97
2.01
2.06
9.5 9.0 8.5 8.0 7.5 7.0 6.5 6.0 5.5 5.0 4.5 4.0 3.5 3.0 2.5 2.0 1.5 1.0 0.5
ppm

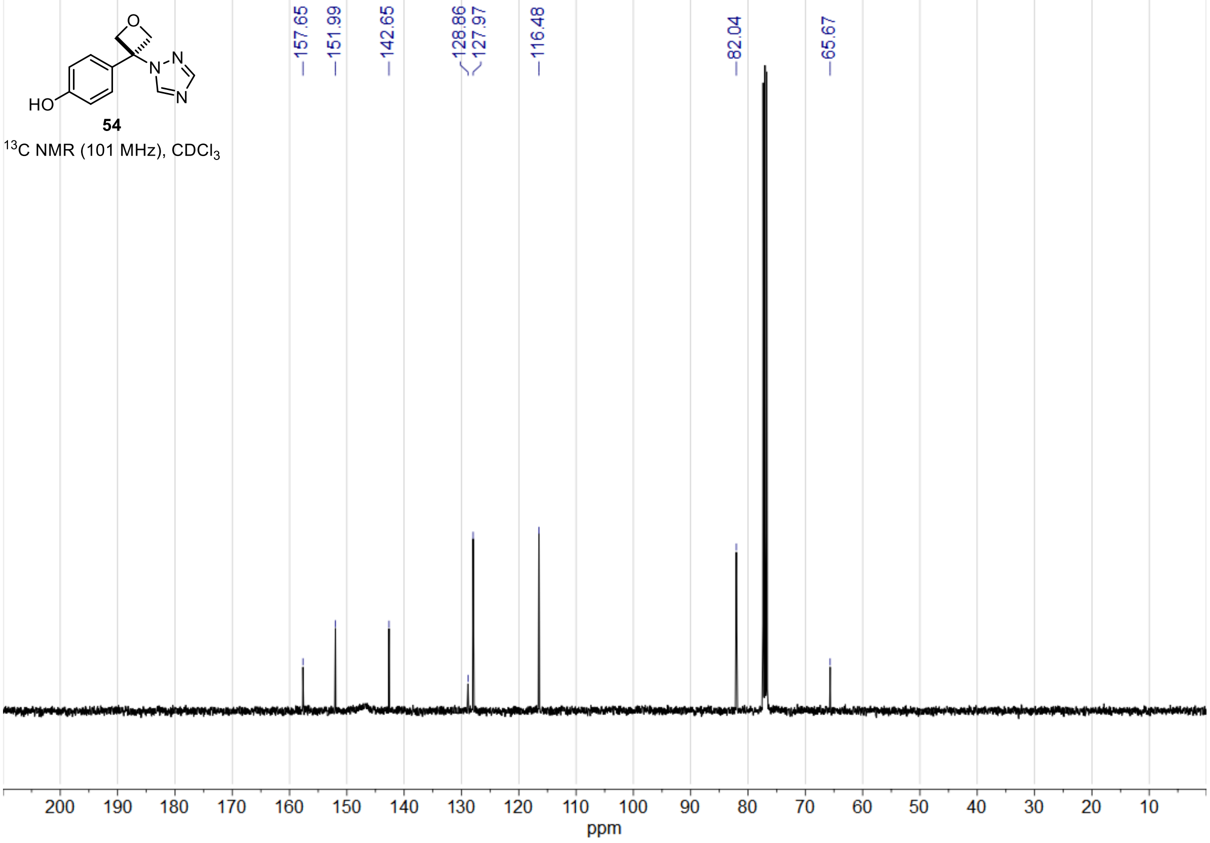
54
13C NMR (101 MHz), CDCl3
157.65
151.99
142.65
128.86
127.97
116.48
82.04
65.67
200 190 180 170 160 150 140 130 120 110 100 90 80 70 60 50 40 30 20 10
ppm

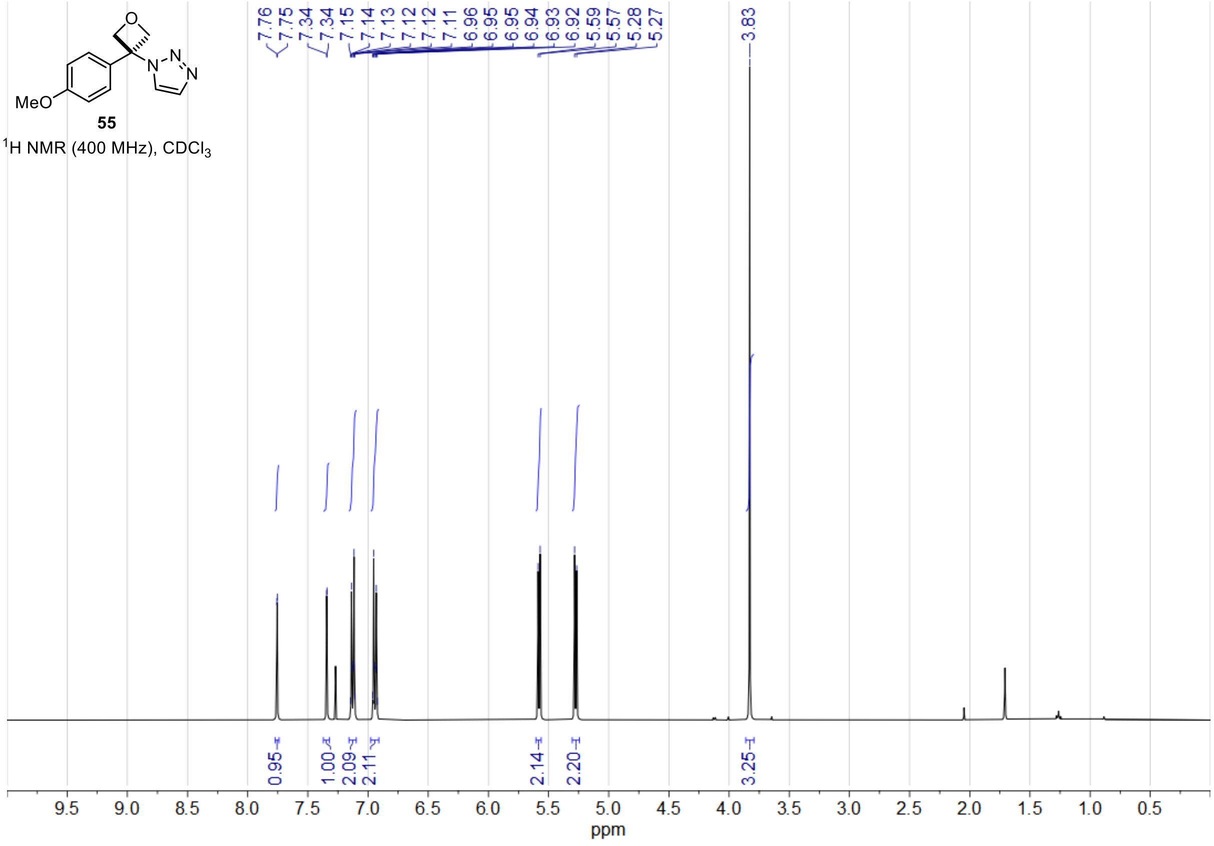

55
^{1}H NMR (400 MHz), CDCl$_3$

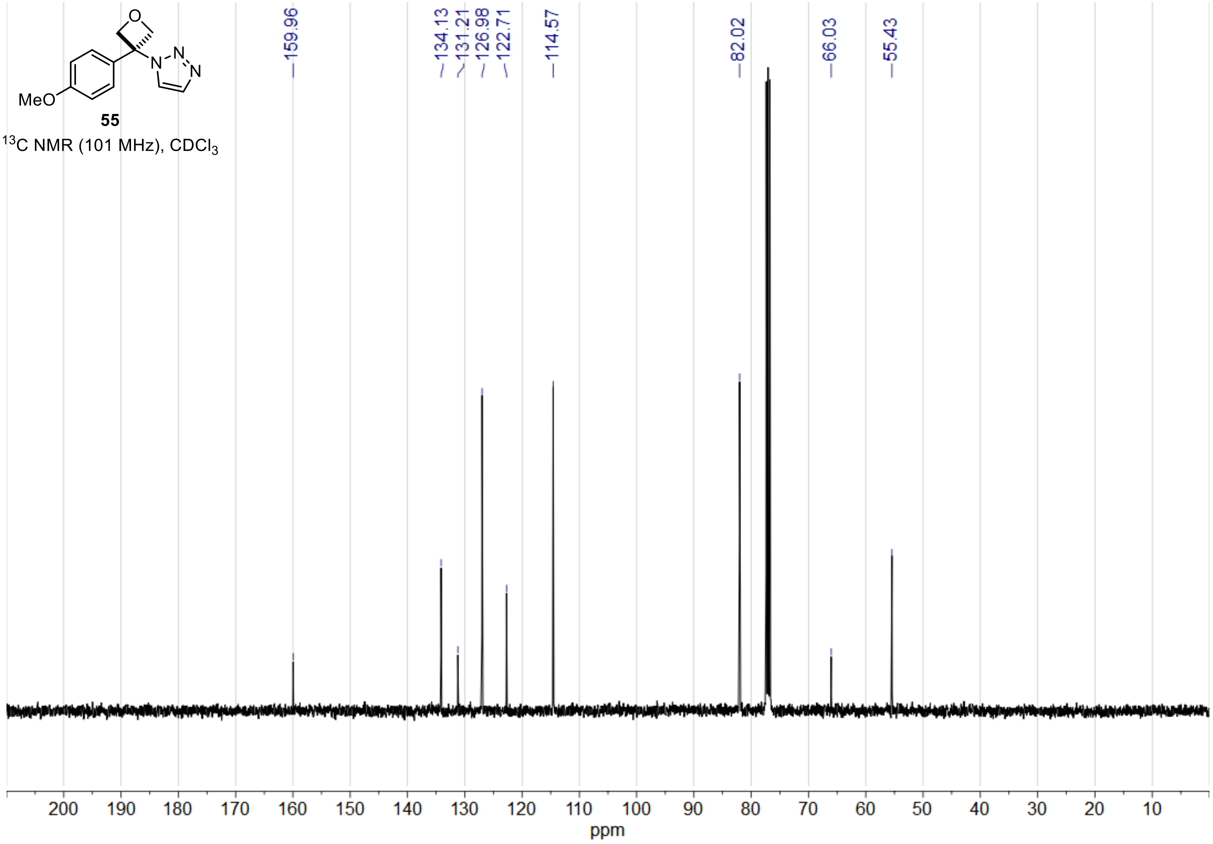

55
^{13}C NMR (101 MHz), CDCl$_3$

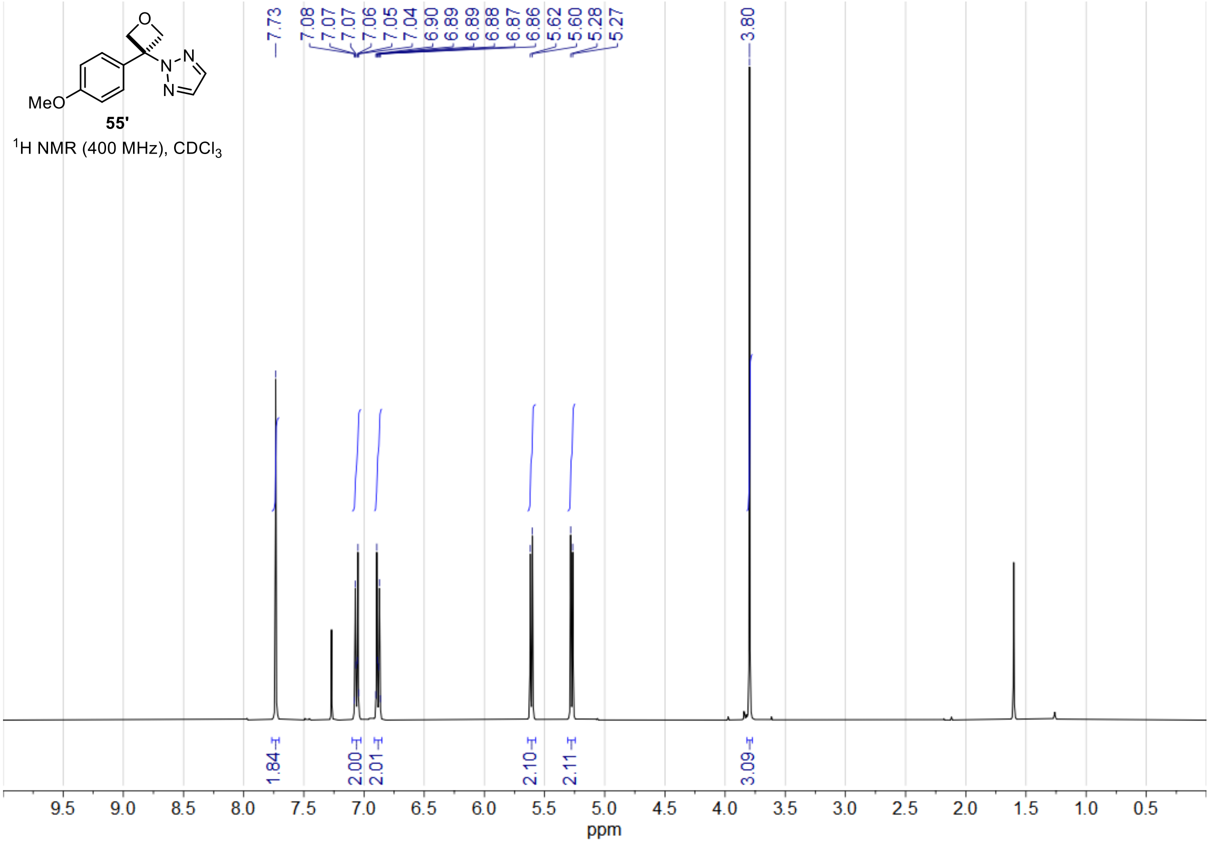

^{1}H NMR (400 MHz), $CDCl_3$

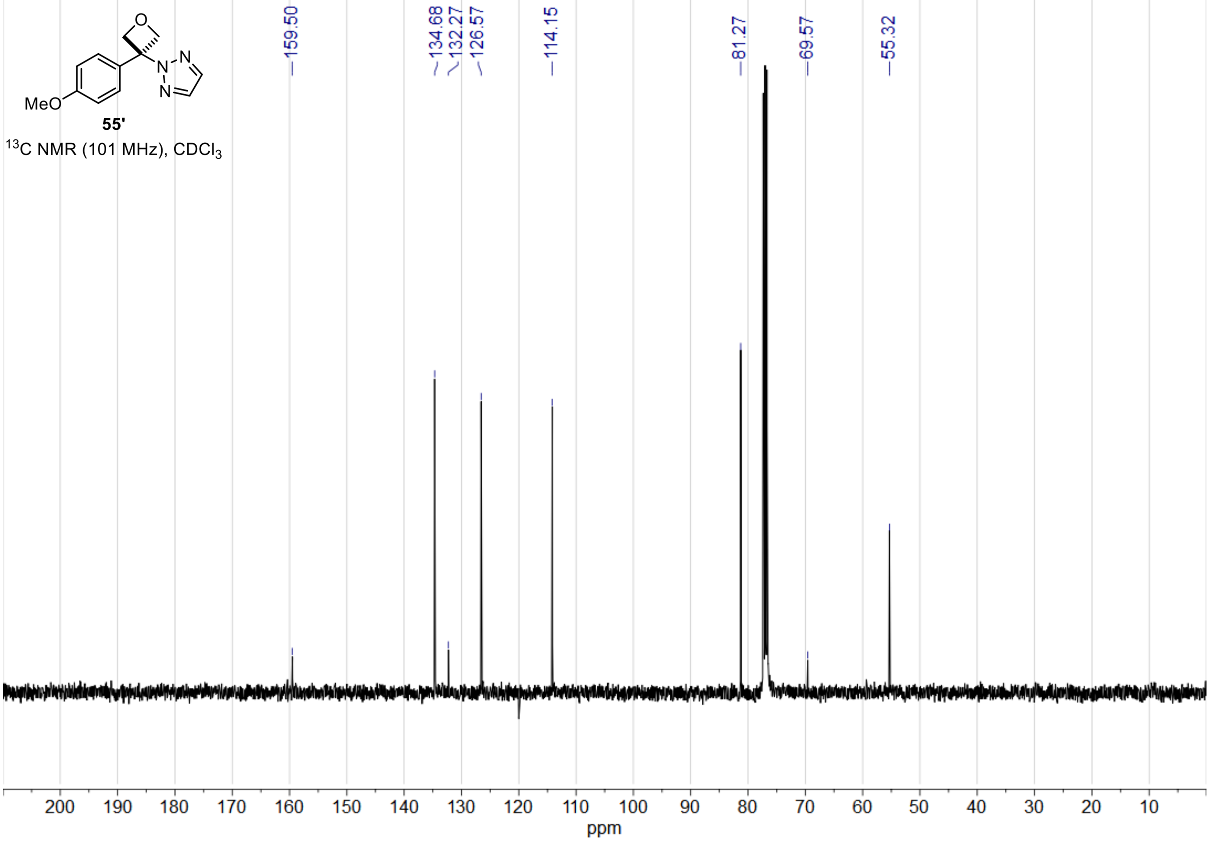

^{13}C NMR (101 MHz), $CDCl_3$

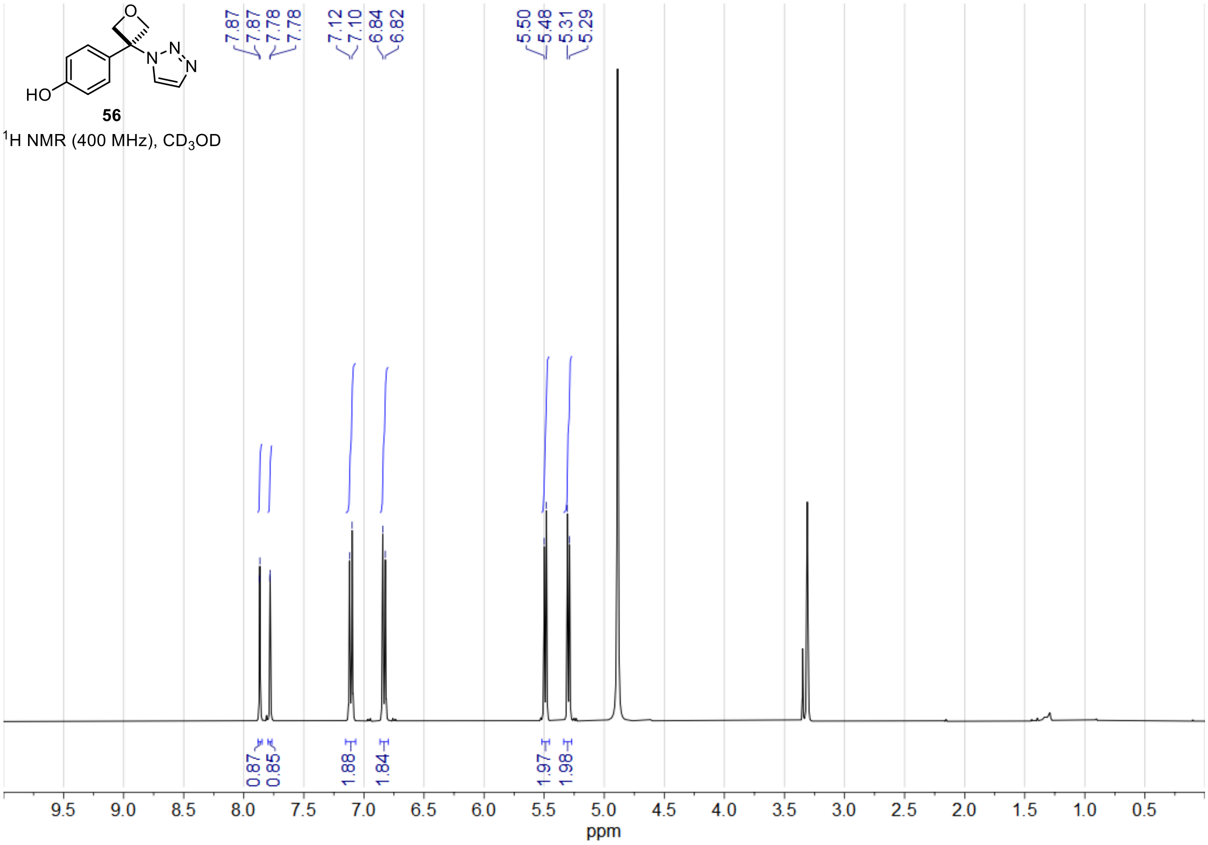

56

^{1}H NMR (400 MHz), CD_3OD

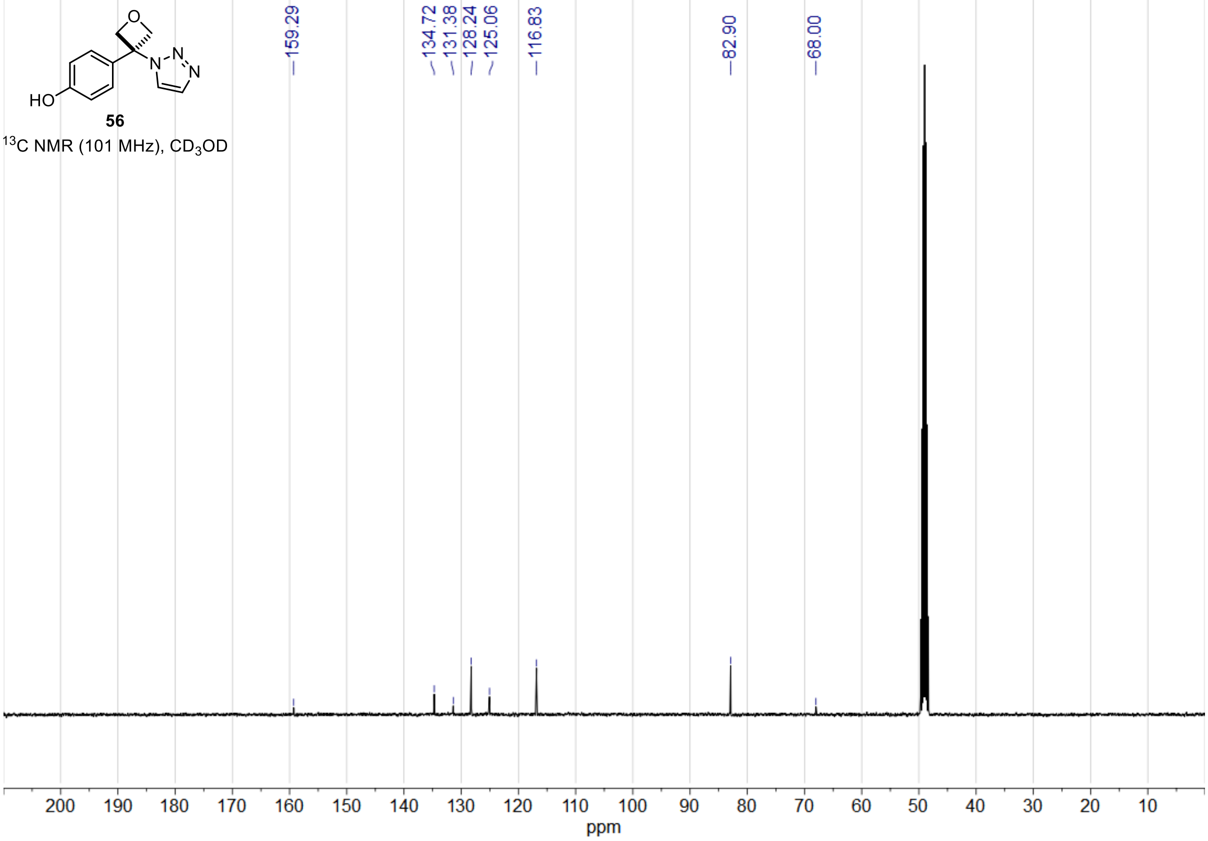

56

^{13}C NMR (101 MHz), CD_3OD

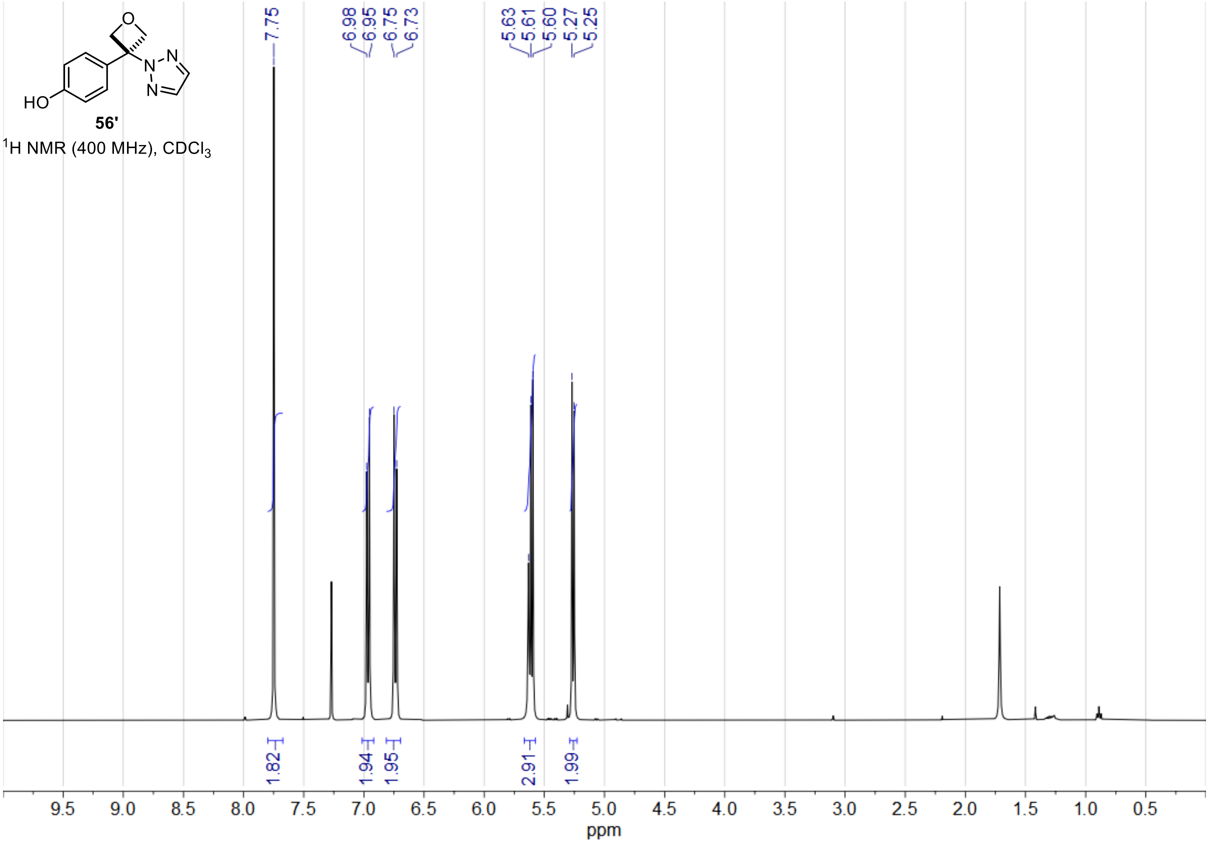
HO
O
N
N
N
56'
^{1}H NMR (400 MHz), CDCl$_3$
7.75
6.98
6.95
6.75
6.73
5.63
5.61
5.60
5.27
5.25
1.82
1.94
1.95
2.91
1.99
9.5 9.0 8.5 8.0 7.5 7.0 6.5 6.0 5.5 5.0 4.5 4.0 3.5 3.0 2.5 2.0 1.5 1.0 0.5
ppm

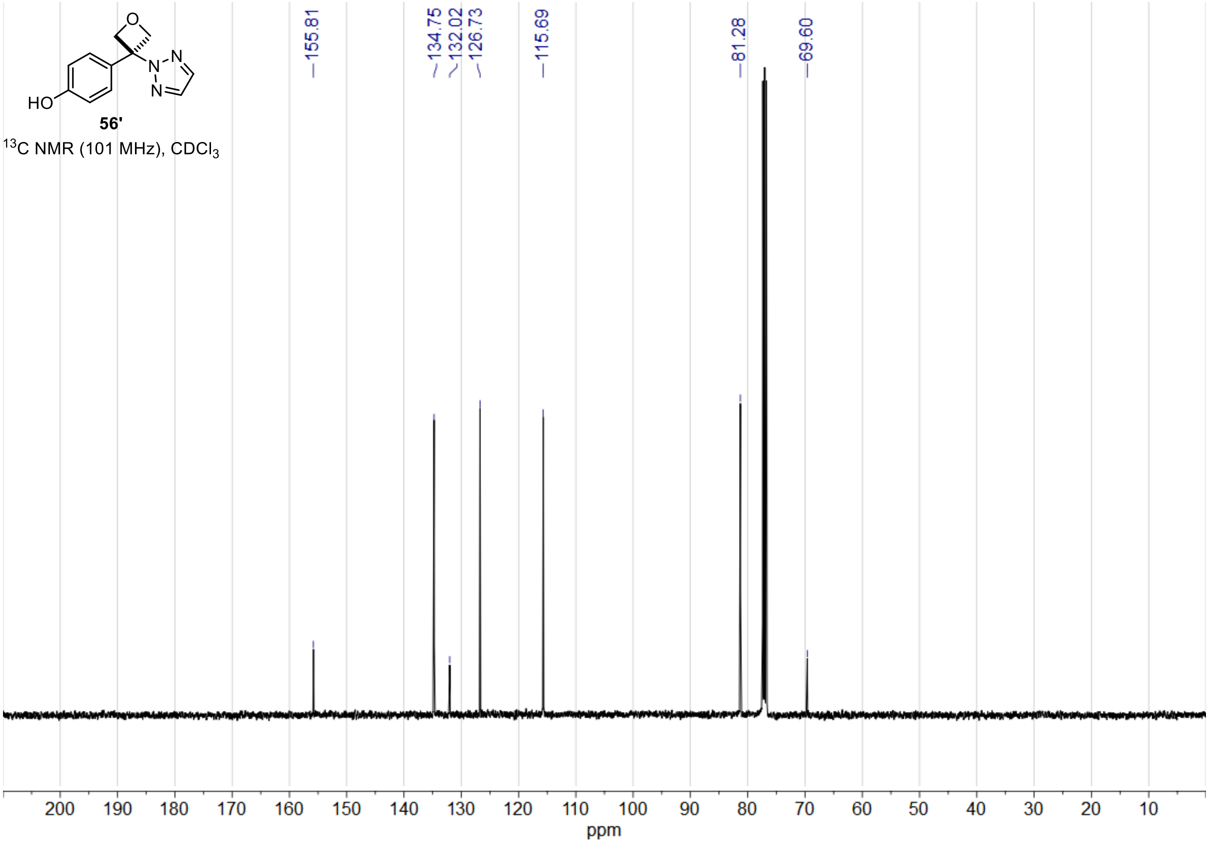
HO
O
N
N
N
56'
^{13}C NMR (101 MHz), CDCl$_3$
155.81
134.75
132.02
126.73
115.69
81.28
69.60
200 190 180 170 160 150 140 130 120 110 100 90 80 70 60 50 40 30 20 10
ppm

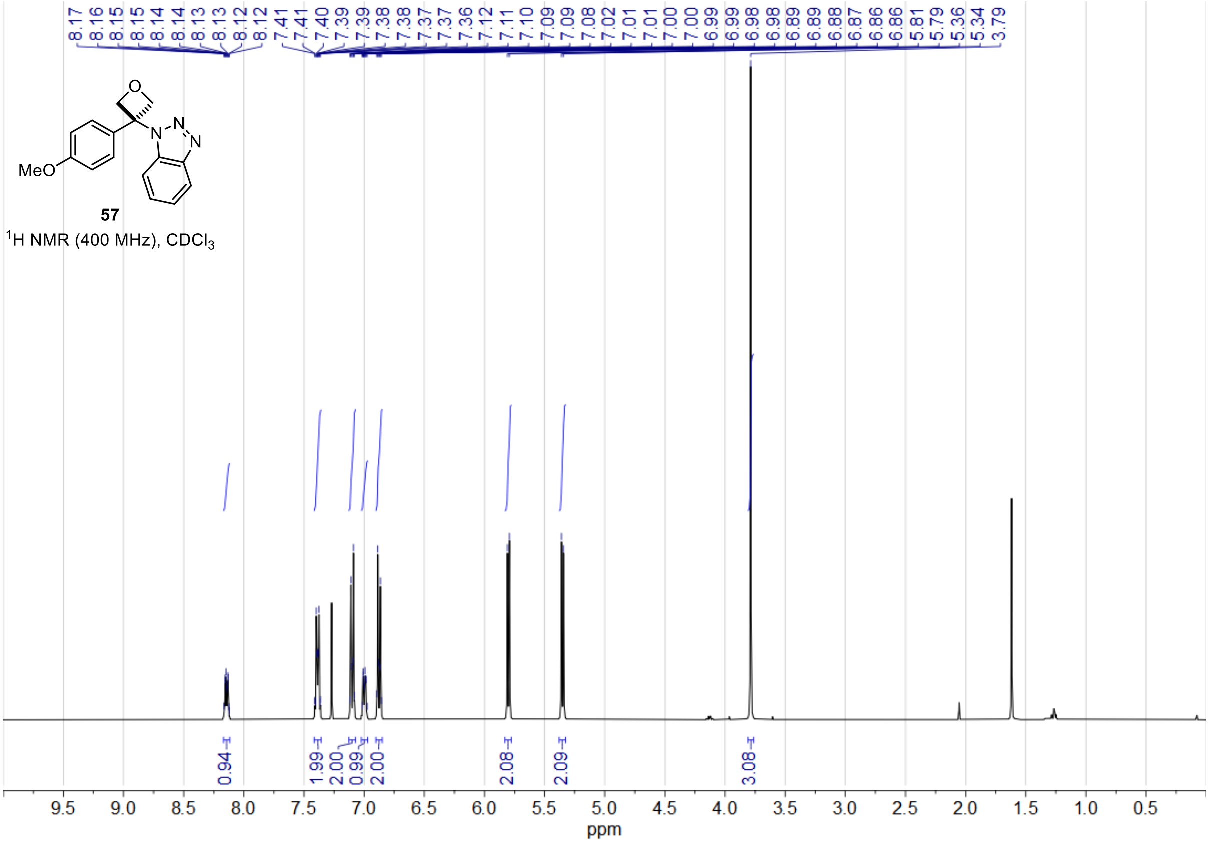

57

^{1}H NMR (400 MHz), $CDCl_3$

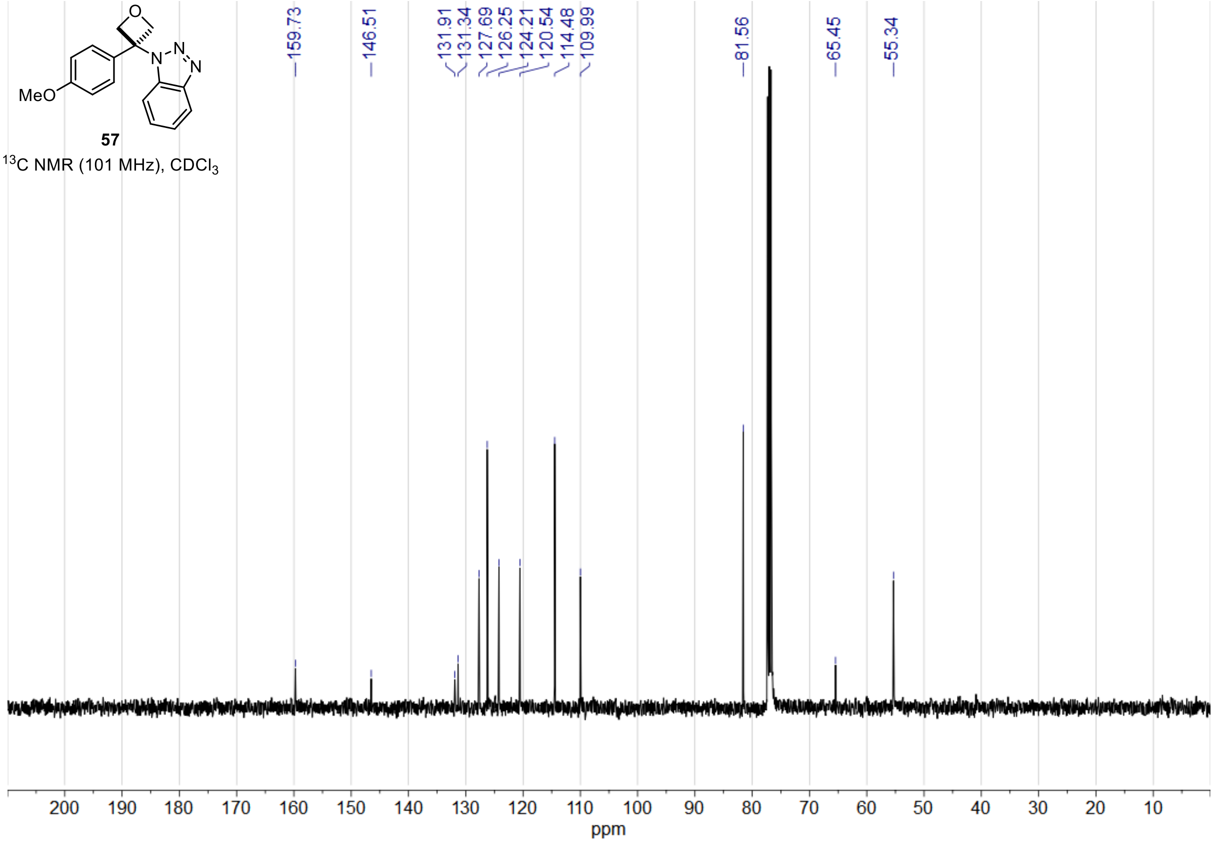

57

^{13}C NMR (101 MHz), $CDCl_3$

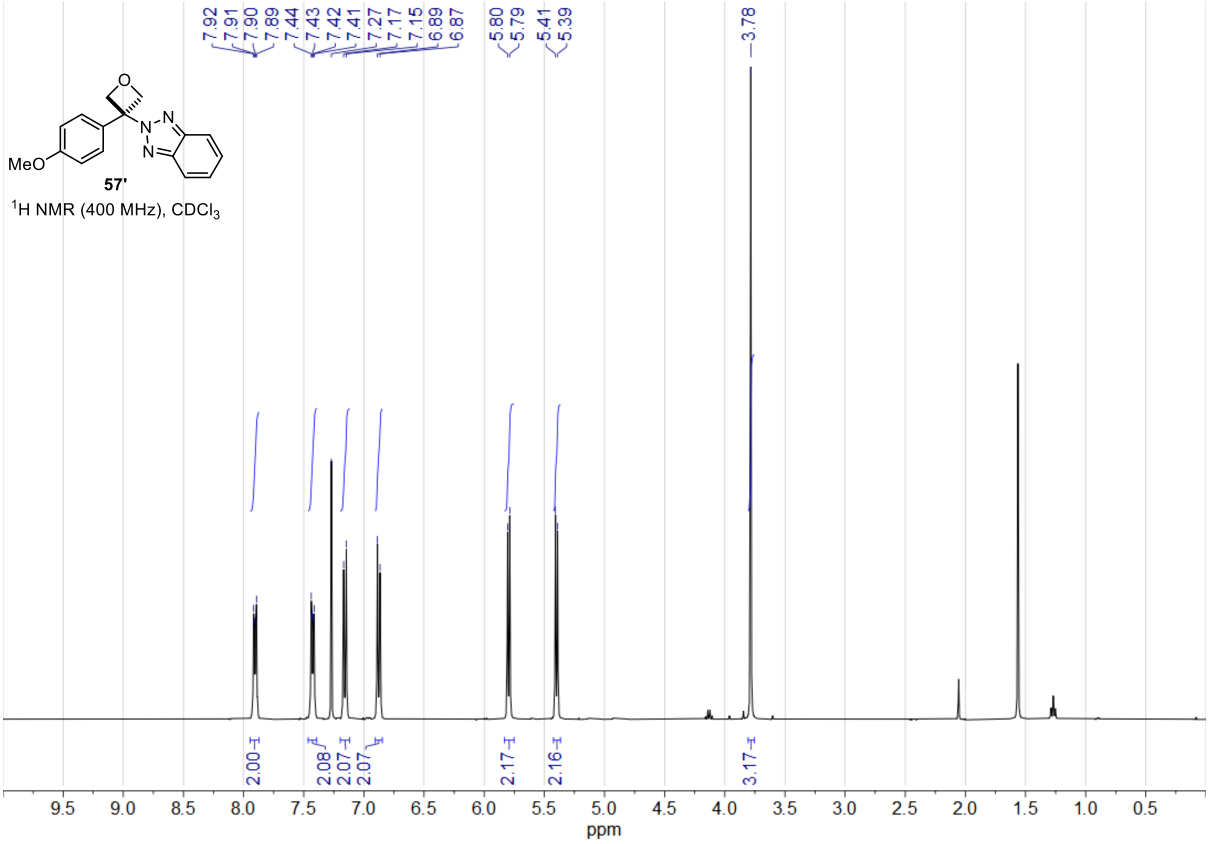
7.92
7.91
7.90
7.89
7.44
7.43
7.42
7.41
7.27
7.17
7.15
6.89
6.87
5.80
5.79
5.41
5.39
3.78
MeO
N
N
N
O
57'
1H NMR (400 MHz), CDCl3
2.00
2.08
2.07
2.07
2.17
2.16
3.17
ppm

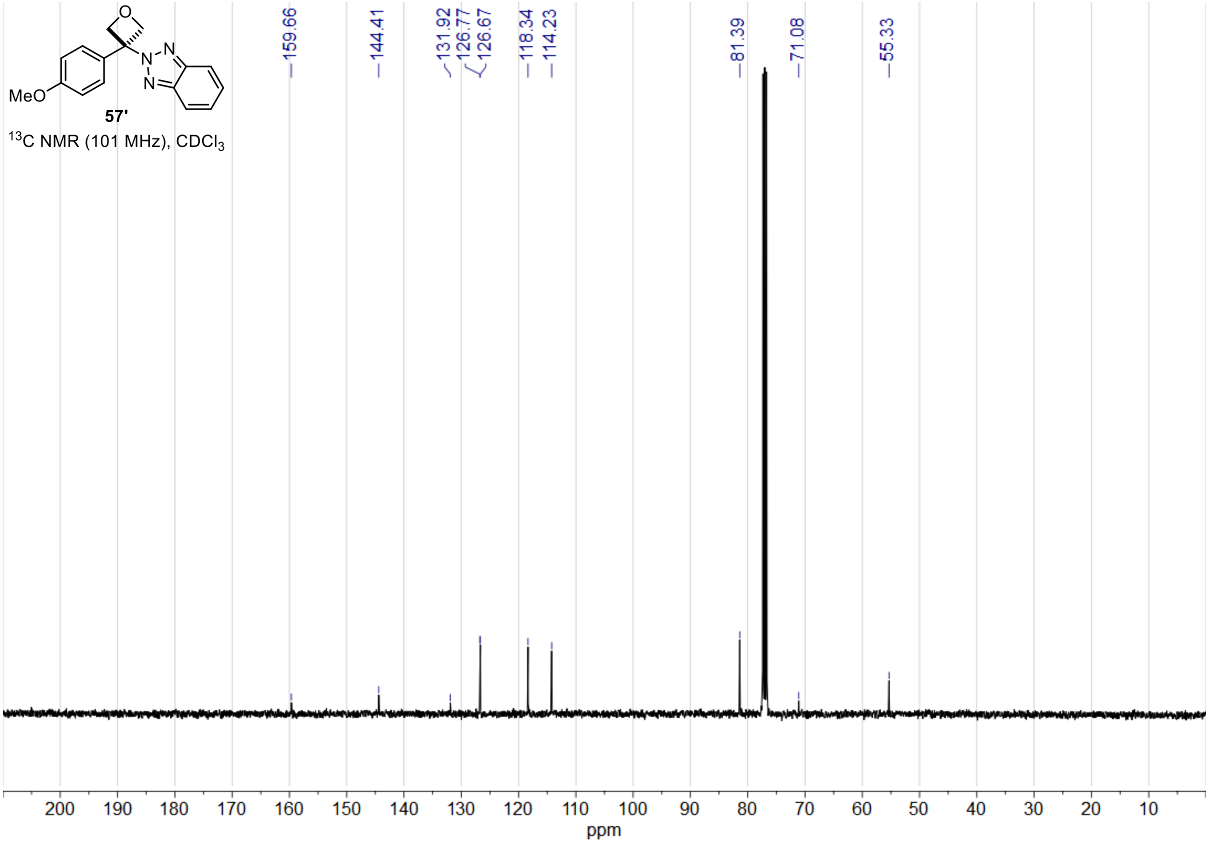
159.66
144.41
131.92
126.77
126.67
118.34
114.23
81.39
71.08
55.33
MeO
N
N
N
O
57'
13C NMR (101 MHz), CDCl3
ppm

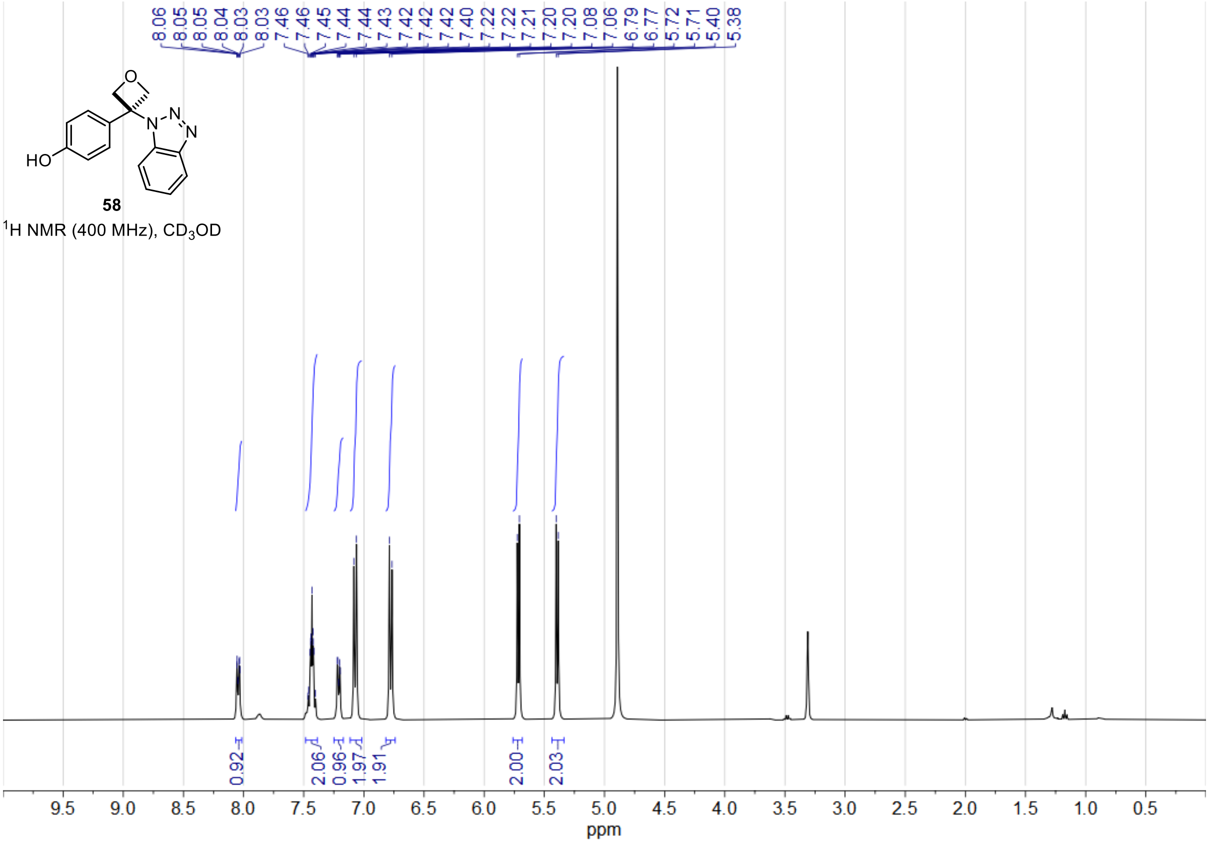
8.06
8.05
8.05
8.04
8.03
8.03
7.46
7.45
7.44
7.44
7.43
7.42
7.42
7.42
7.40
7.22
7.22
7.21
7.20
7.20
7.08
7.06
6.79
6.77
5.72
5.71
5.40
5.38
HO
O
N
N
N
58
^{1}H NMR (400 MHz), CD_3OD
0.92
2.06
0.96
1.97
1.91
2.00
2.03
ppm

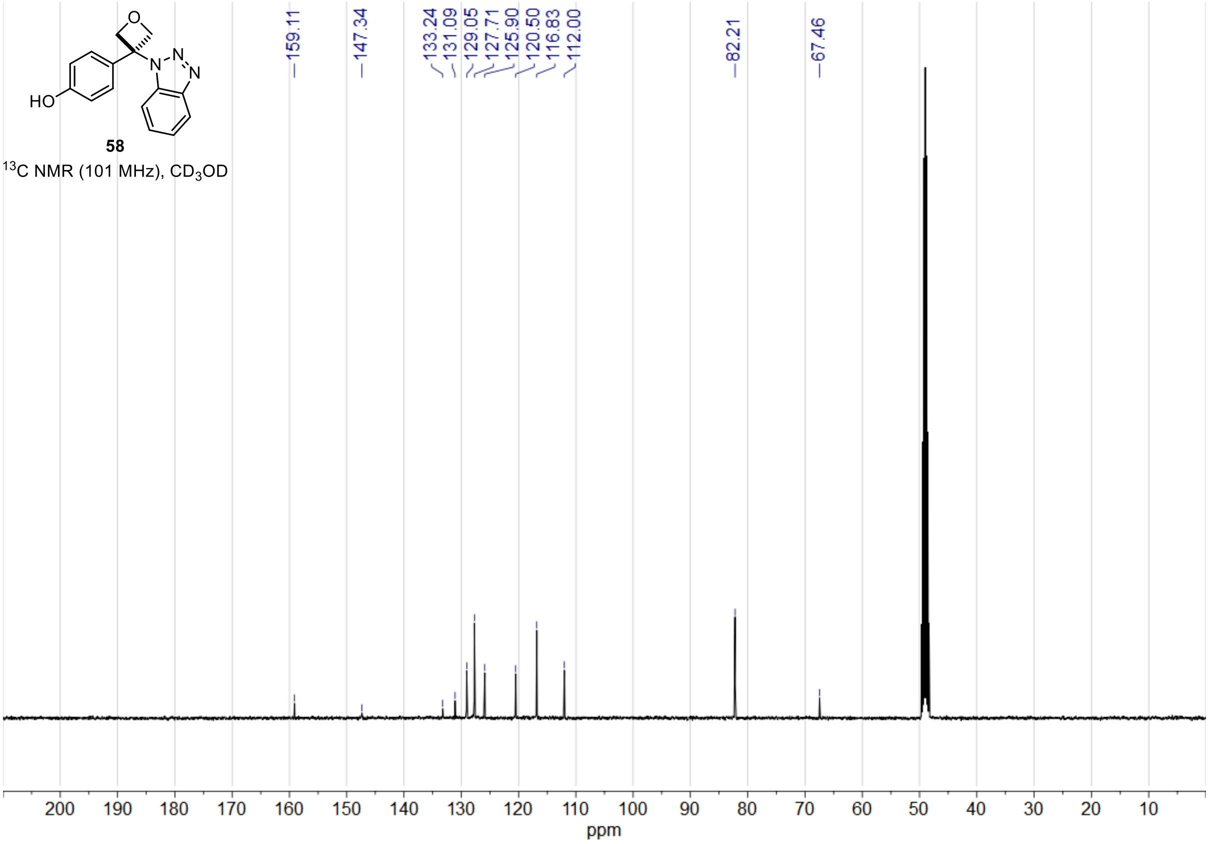
159.11
147.34
133.24
131.09
129.05
127.71
125.90
120.50
116.83
112.00
82.21
67.46
HO
O
N
N
N
58
^{13}C NMR (101 MHz), CD_3OD
ppm

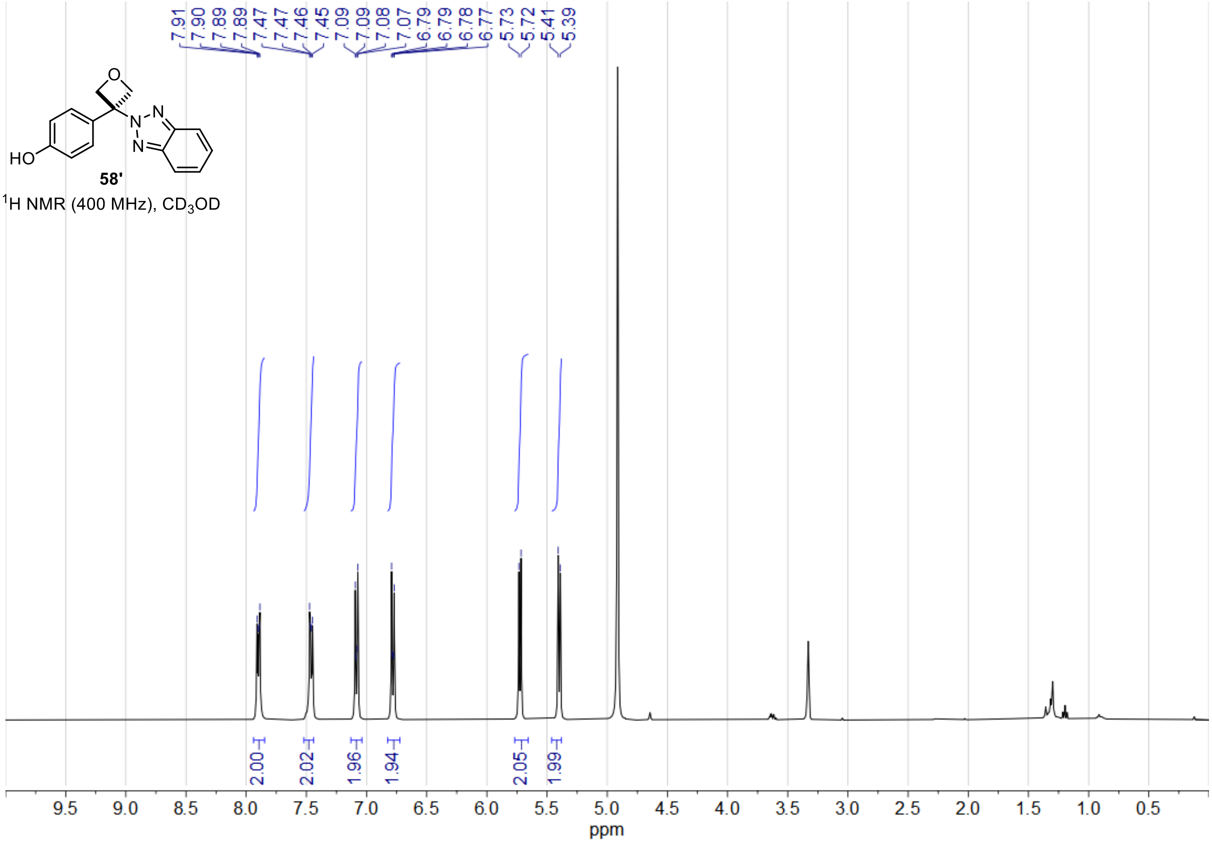

58'

^{1}H NMR (400 MHz), CD_3OD

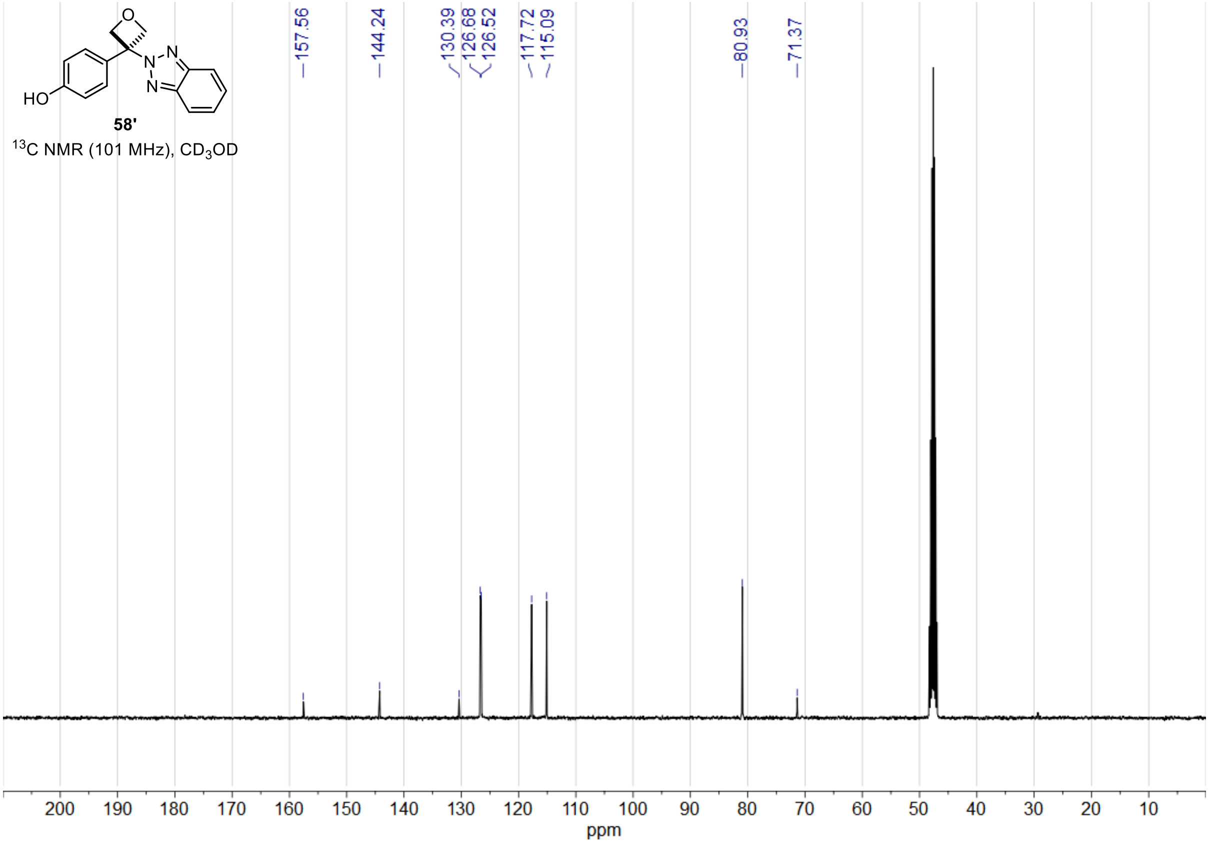

58'

^{13}C NMR (101 MHz), CD_3OD

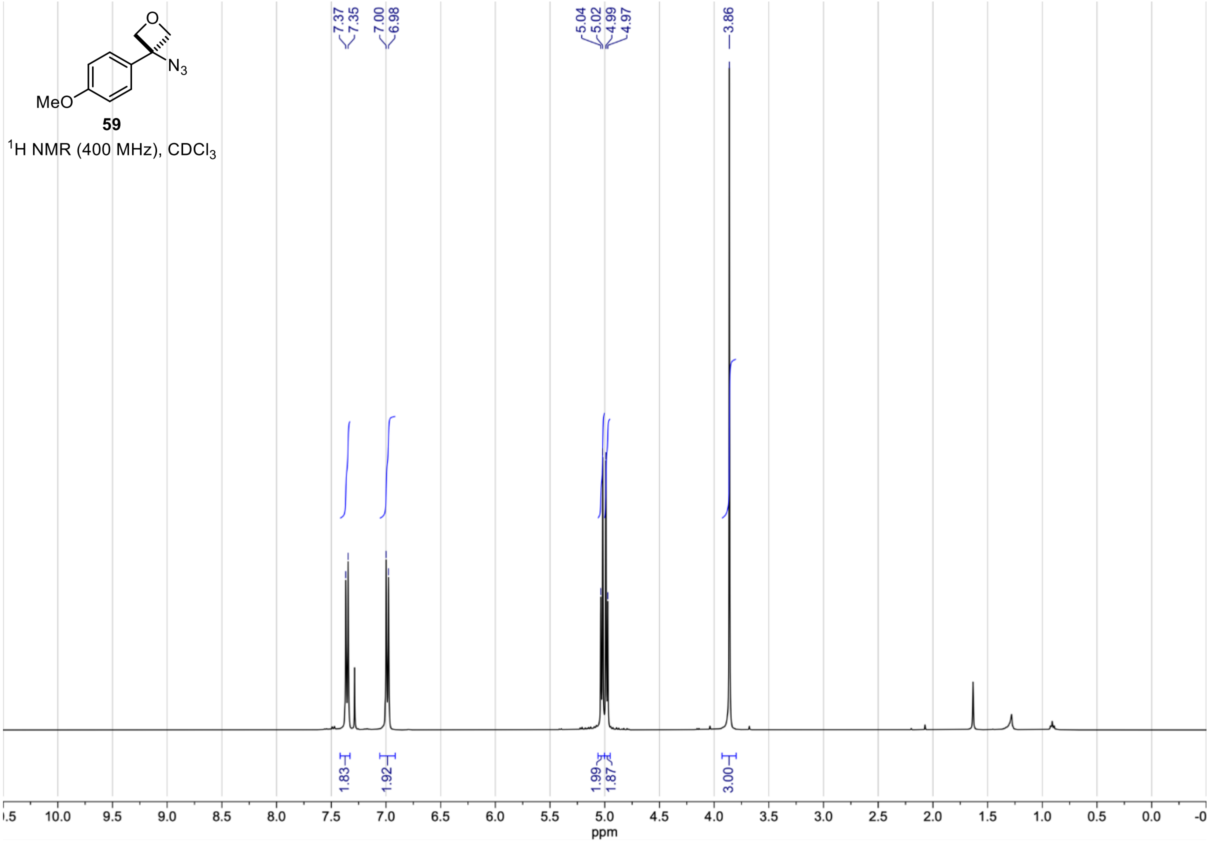
MeO
N3
59
1H NMR (400 MHz), CDCl3
7.37
7.35
7.00
6.98
5.04
5.02
4.99
4.97
3.86
1.83
1.92
1.99
1.87
3.00
10.0 9.5 9.0 8.5 8.0 7.5 7.0 6.5 6.0 5.5 5.0 4.5 4.0 3.5 3.0 2.5 2.0 1.5 1.0 0.5 0.0
ppm

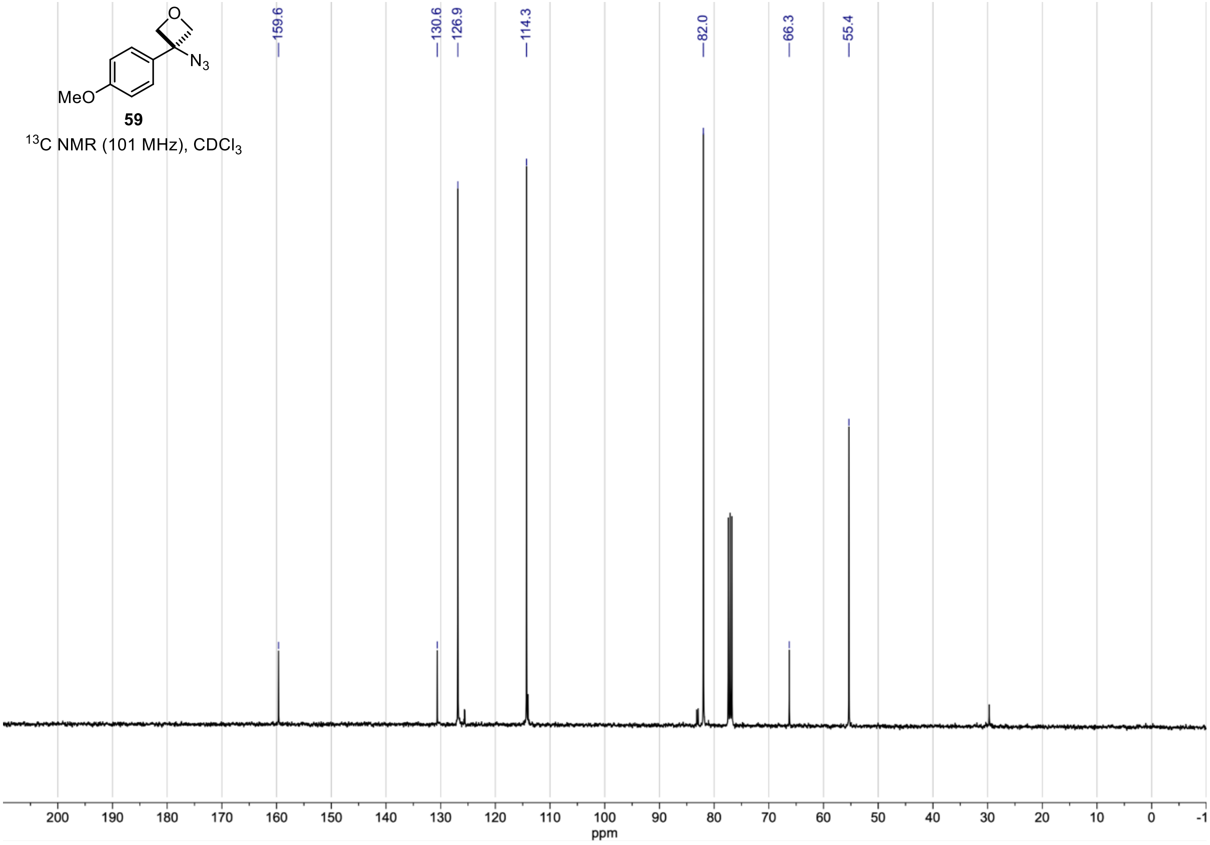
MeO
N3
59
13C NMR (101 MHz), CDCl3
159.6
130.6
126.9
114.3
82.0
66.3
55.4
200 190 180 170 160 150 140 130 120 110 100 90 80 70 60 50 40 30 20 10 0
ppm

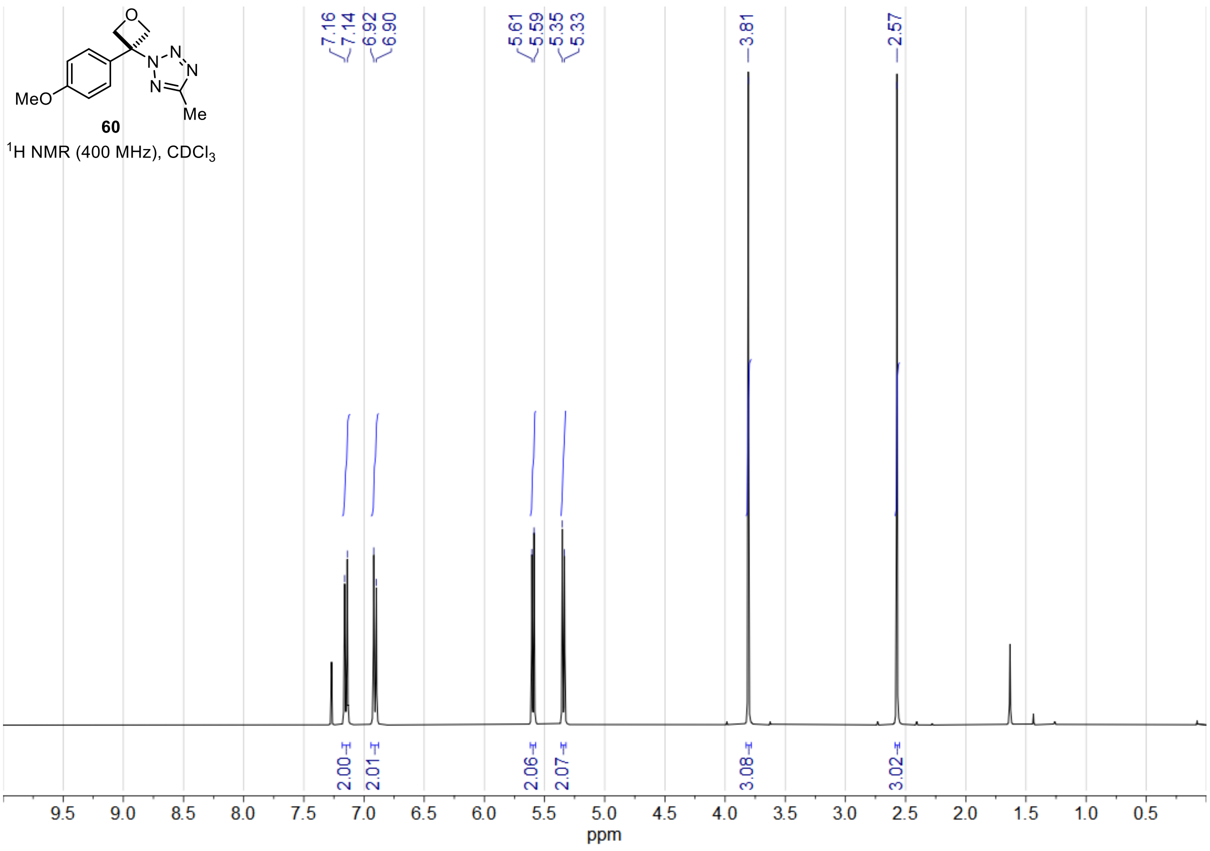
MeO
Me
60
^{1}H NMR (400 MHz), CDCl3
7.16
7.14
6.92
6.90
5.61
5.59
5.35
5.33
3.81
2.57
2.00
2.01
2.06
2.07
3.08
3.02
9.5
9.0
8.5
8.0
7.5
7.0
6.5
6.0
5.5
5.0
4.5
4.0
3.5
3.0
2.5
2.0
1.5
1.0
0.5
ppm

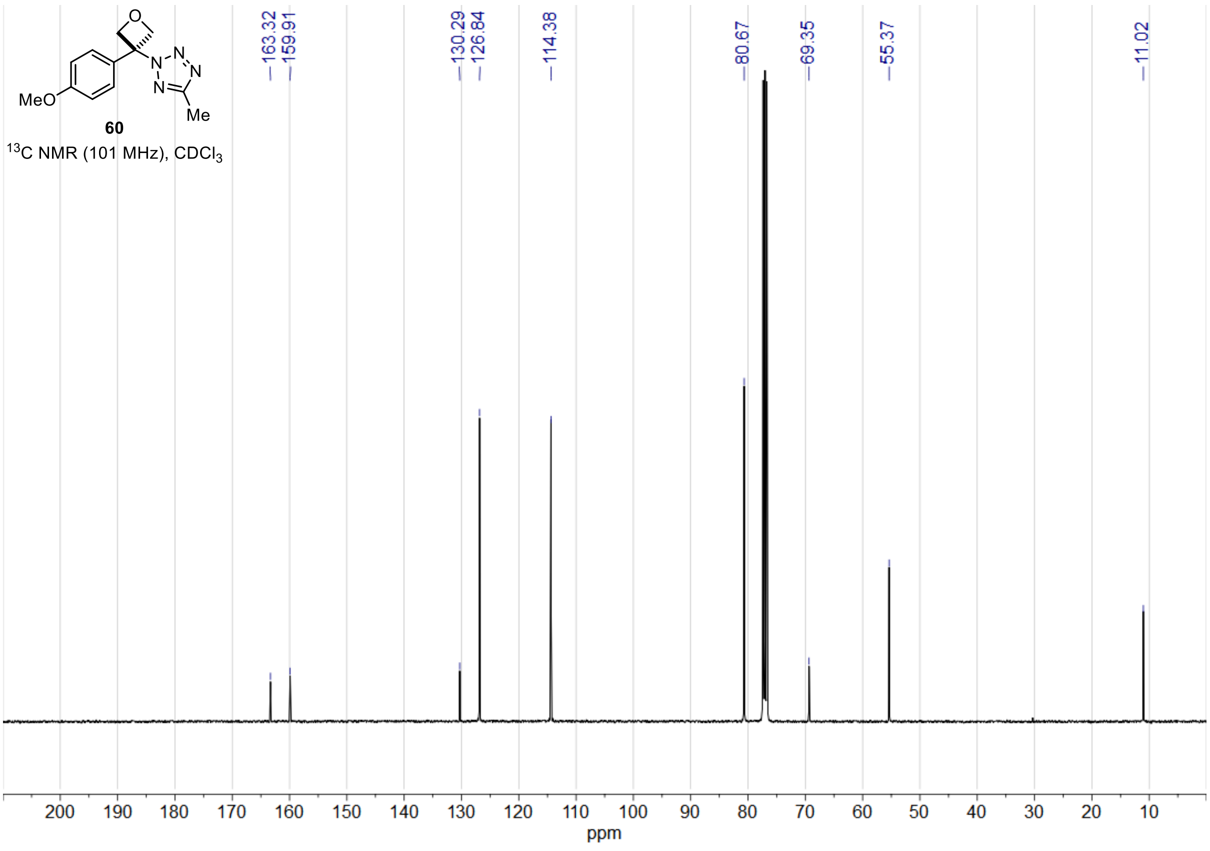
MeO
Me
60
^{13}C NMR (101 MHz), CDCl3
163.32
159.91
130.29
126.84
114.38
80.67
69.35
55.37
11.02
200
190
180
170
160
150
140
130
120
110
100
90
80
70
60
50
40
30
20
10
ppm

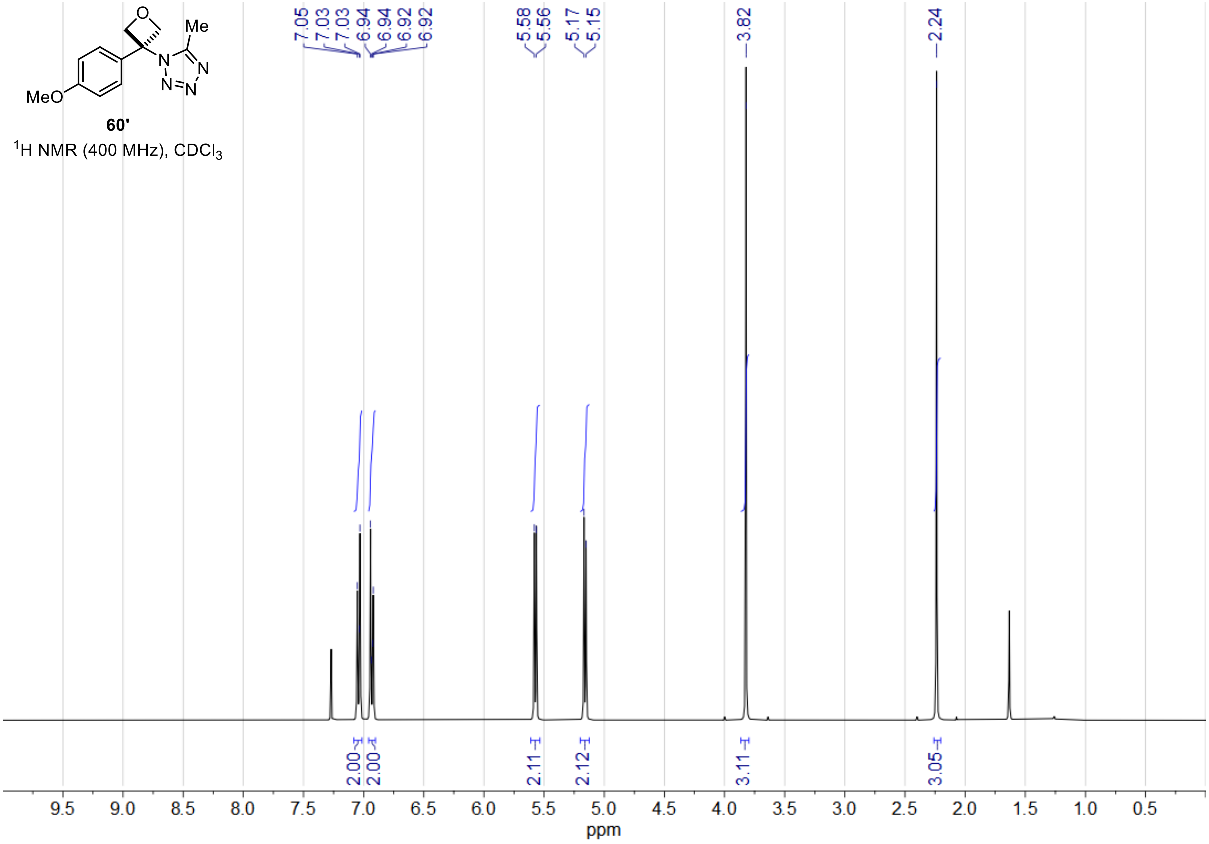
MeO
Me
60'
¹H NMR (400 MHz), CDCl3
7.05
7.03
7.03
6.94
6.94
6.92
6.92
5.58
5.56
5.17
5.15
3.82
2.24
2.00
2.00
2.11
2.12
3.11
3.05
ppm

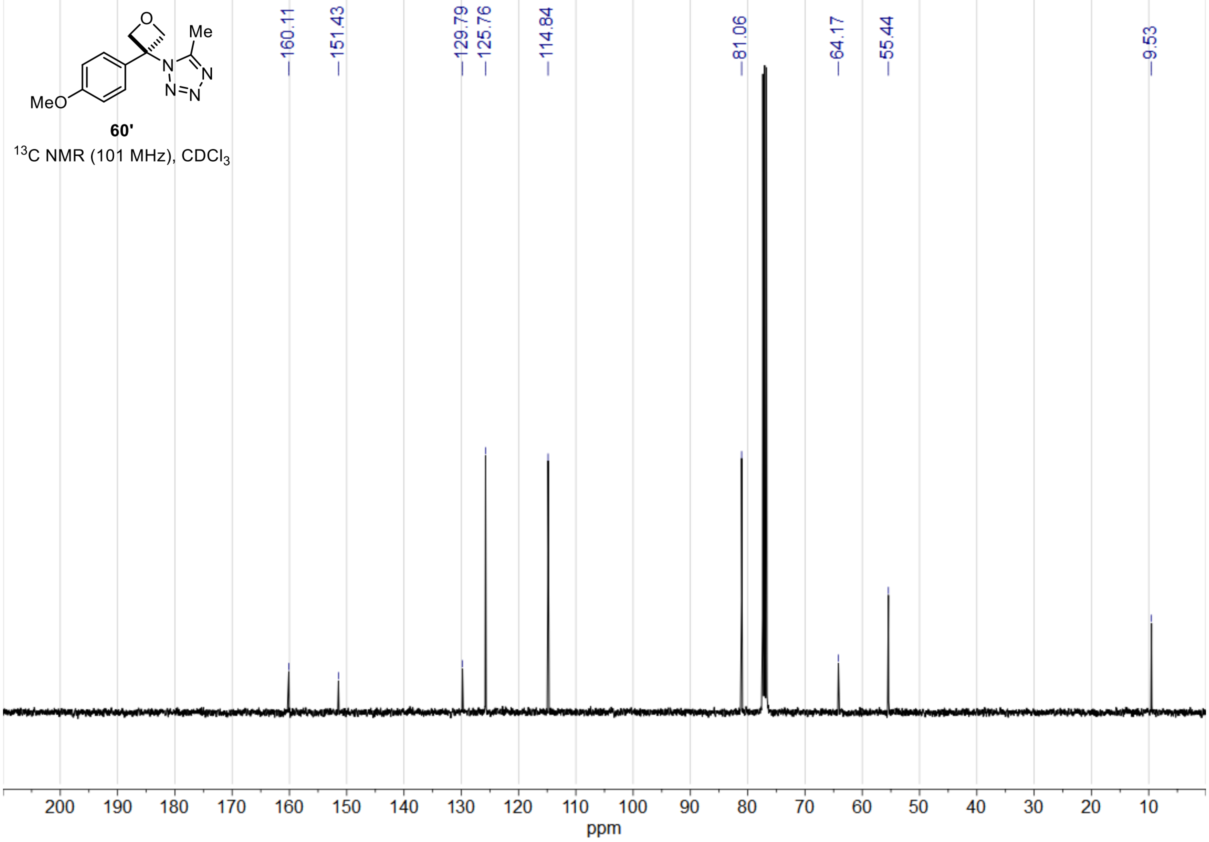
MeO
Me
60'
¹³C NMR (101 MHz), CDCl3
160.11
151.43
129.79
125.76
114.84
81.06
64.17
55.44
9.53
ppm

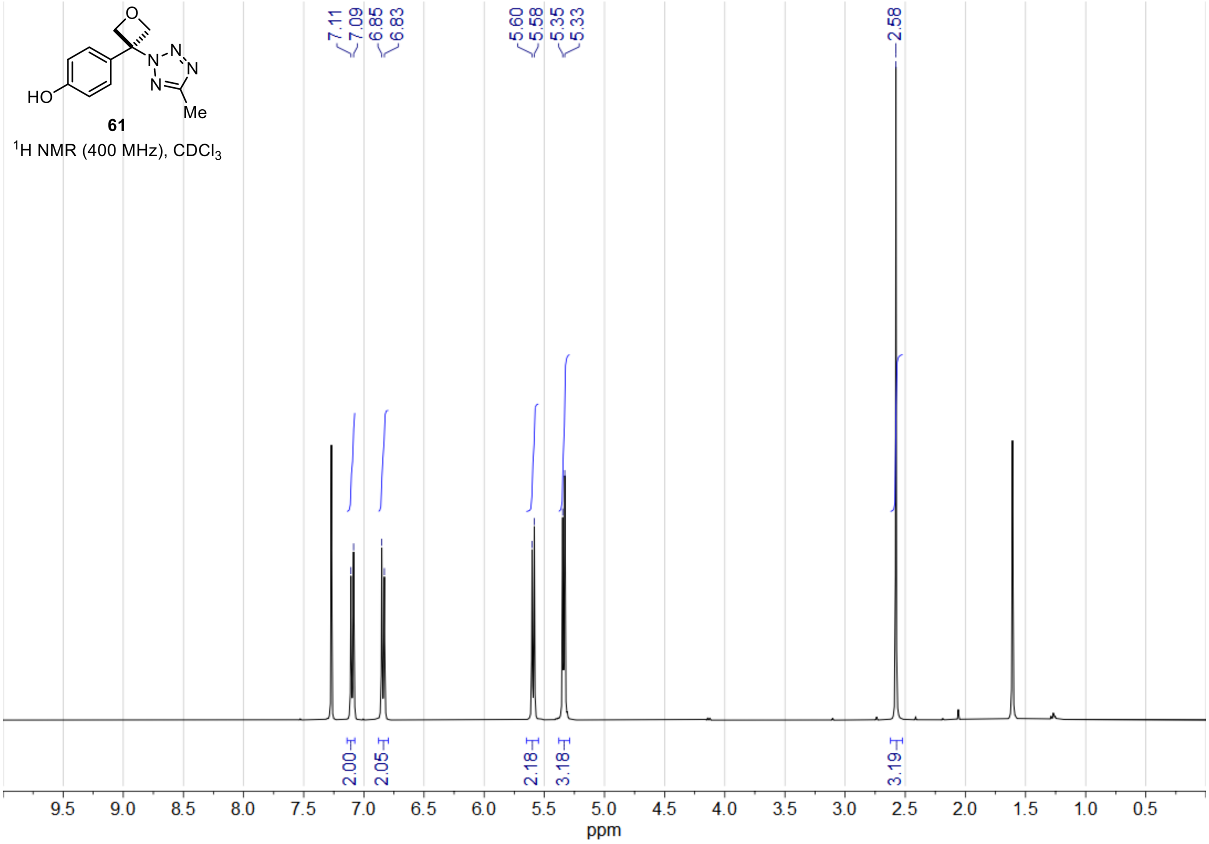
HO
Me
61
^{1}H NMR (400 MHz), CDCl$_3$
7.11
7.09
6.85
6.83
5.60
5.58
5.35
5.33
2.58
2.00
2.05
2.18
3.18
3.19
9.5
9.0
8.5
8.0
7.5
7.0
6.5
6.0
5.5
5.0
4.5
4.0
3.5
3.0
2.5
2.0
1.5
1.0
0.5
ppm

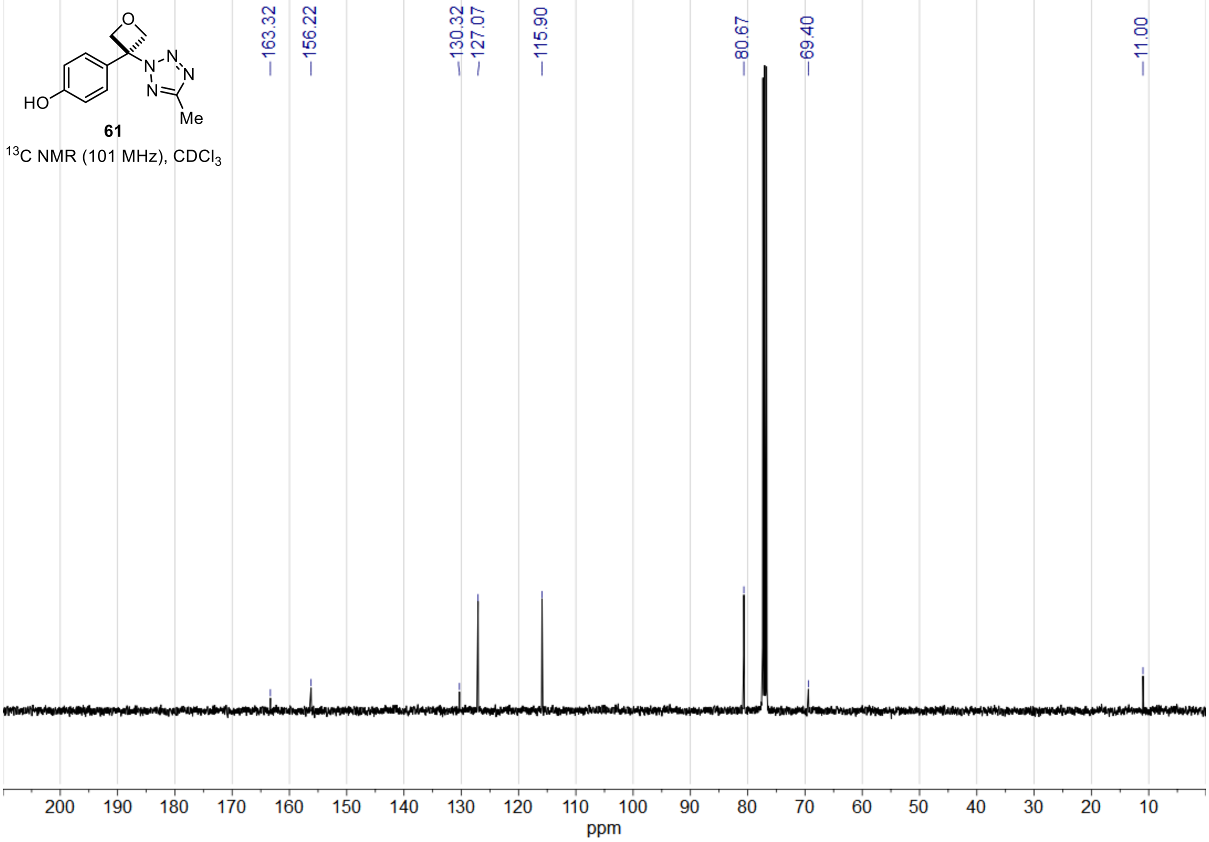
HO
Me
61
^{13}C NMR (101 MHz), CDCl$_3$
163.32
156.22
130.32
127.07
115.90
80.67
69.40
11.00
200
190
180
170
160
150
140
130
120
110
100
90
80
70
60
50
40
30
20
10
ppm

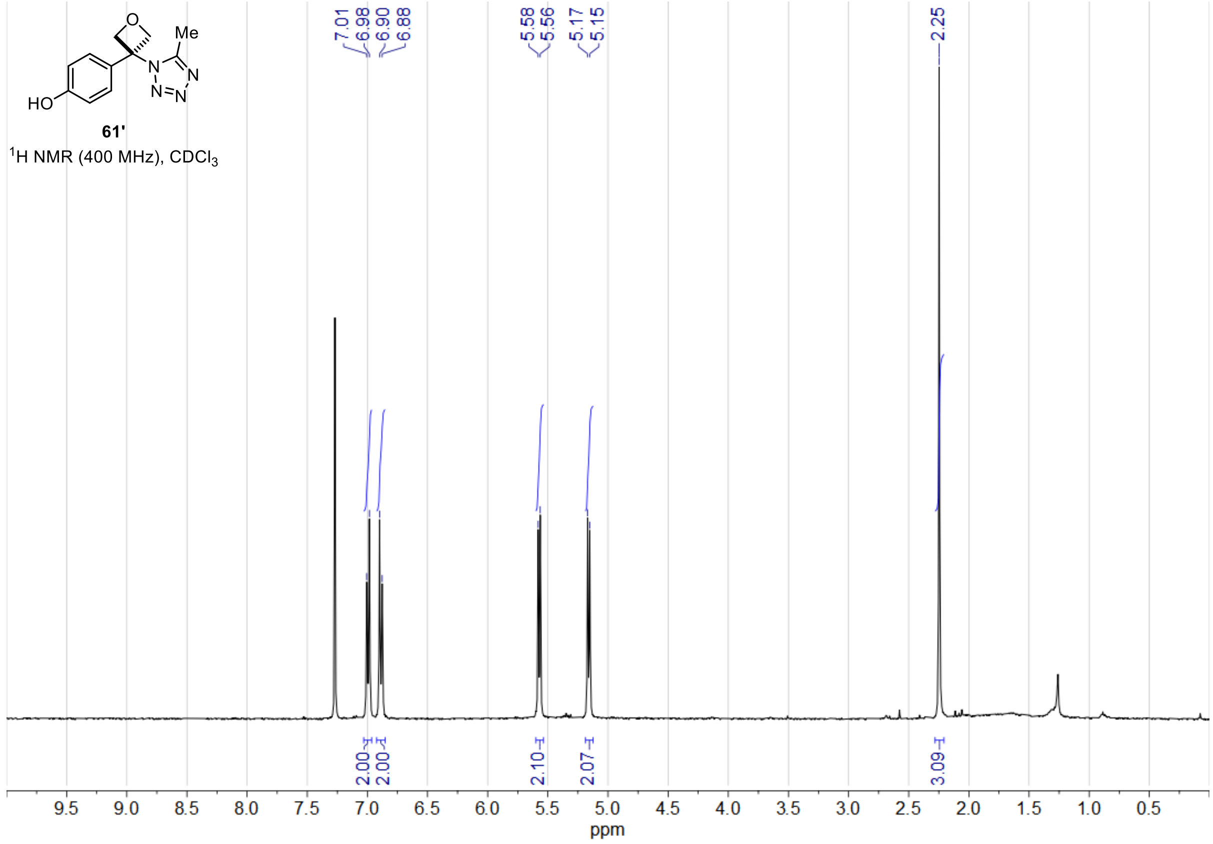
Me
HO
61'
1H NMR (400 MHz), CDCl3
7.01
6.98
6.90
6.88
5.58
5.56
5.17
5.15
2.25
2.00
2.00
2.10
2.07
3.09
ppm

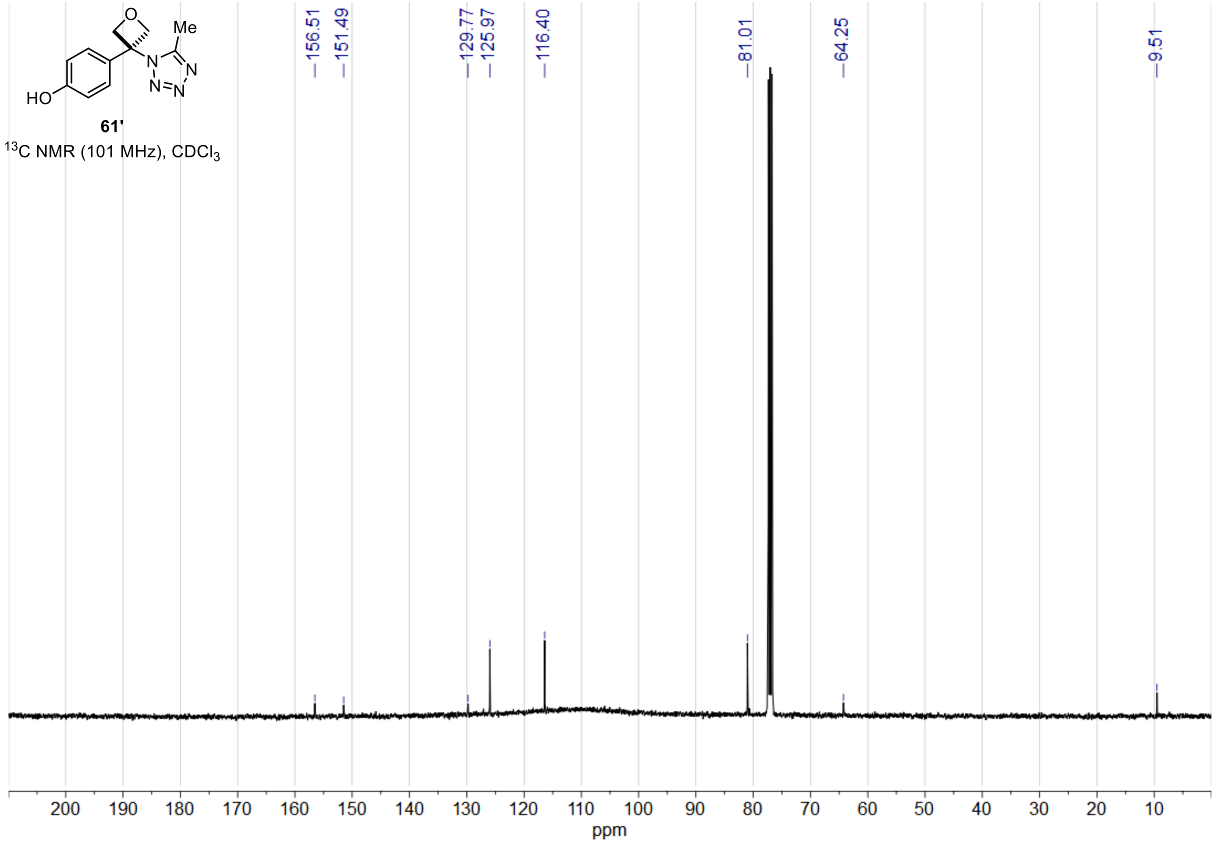
Me
HO
61'
13C NMR (101 MHz), CDCl3
156.51
151.49
129.77
125.97
116.40
81.01
64.25
9.51
ppm

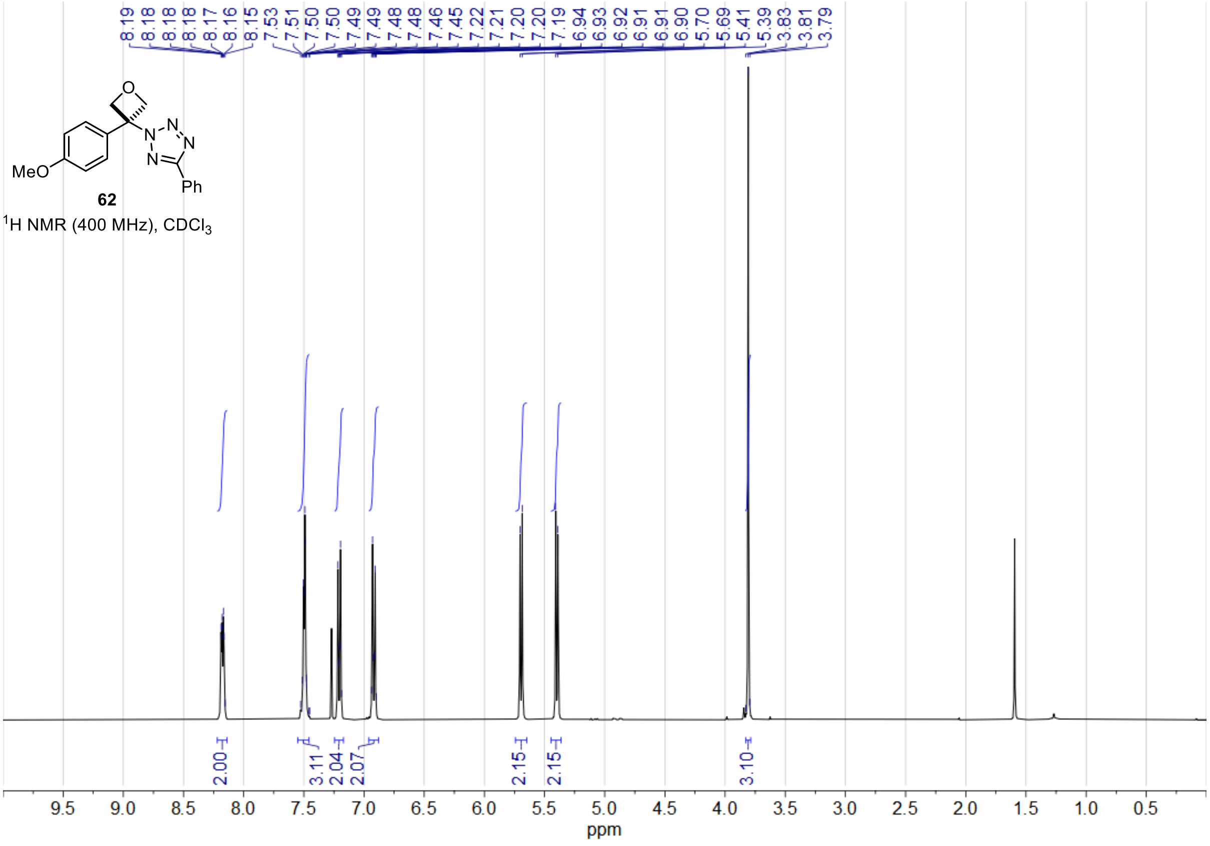

62

^{1}H NMR (400 MHz), $CDCl_3$

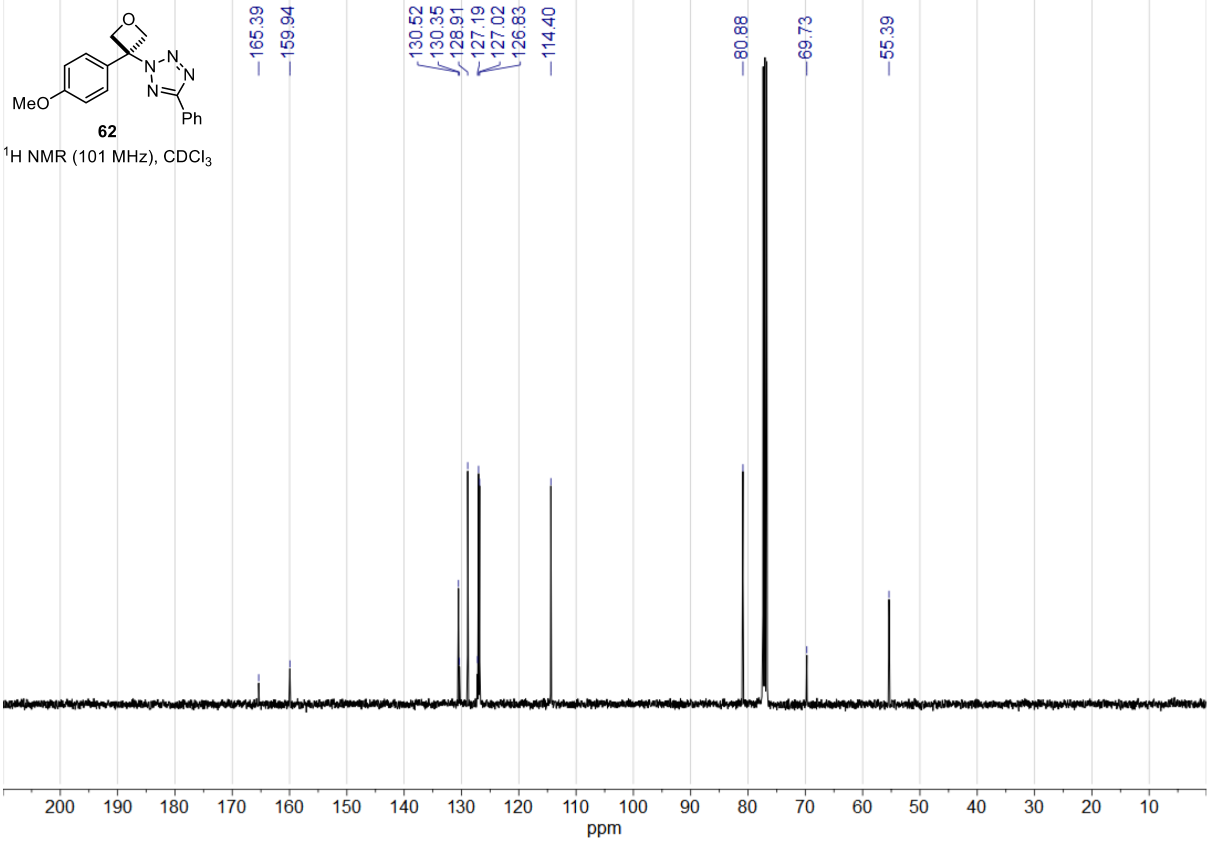

62

^{1}H NMR (101 MHz), $CDCl_3$

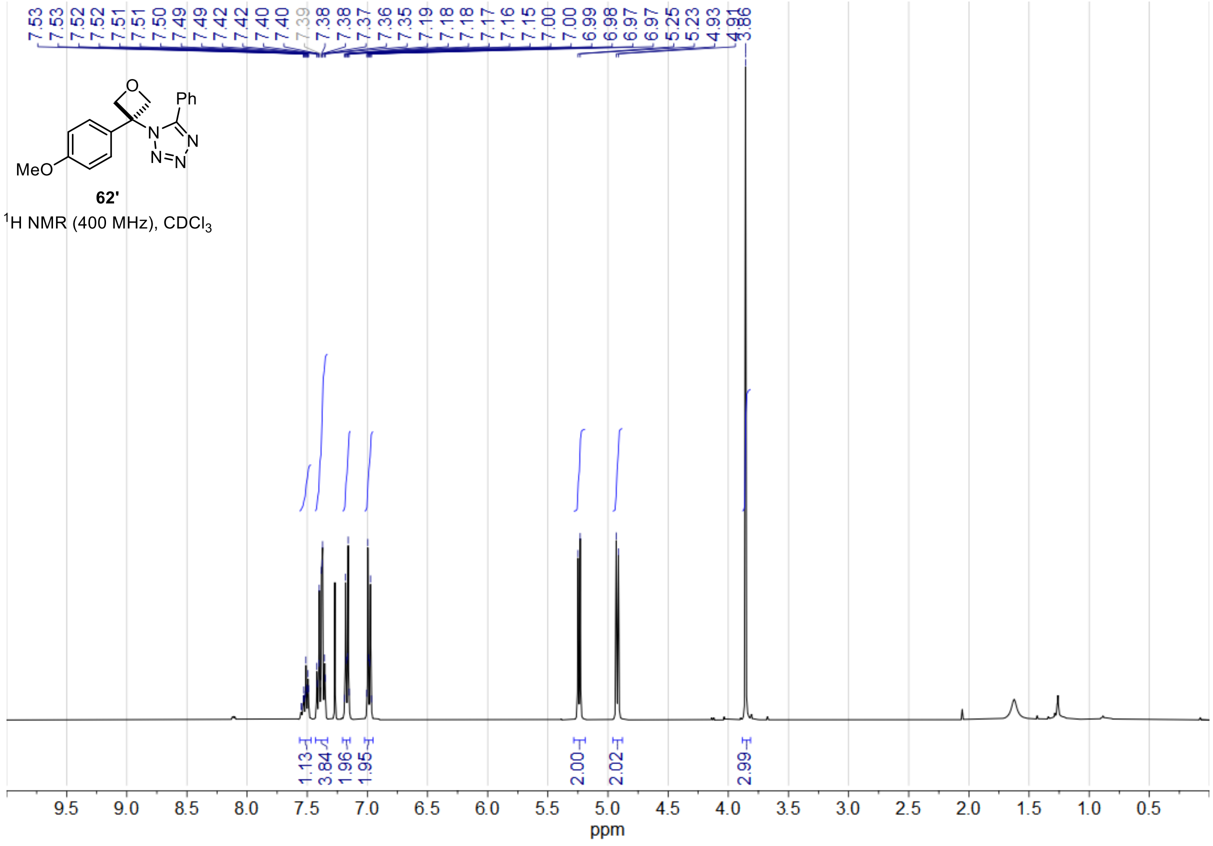

62'

^{1}H NMR (400 MHz), $CDCl_3$

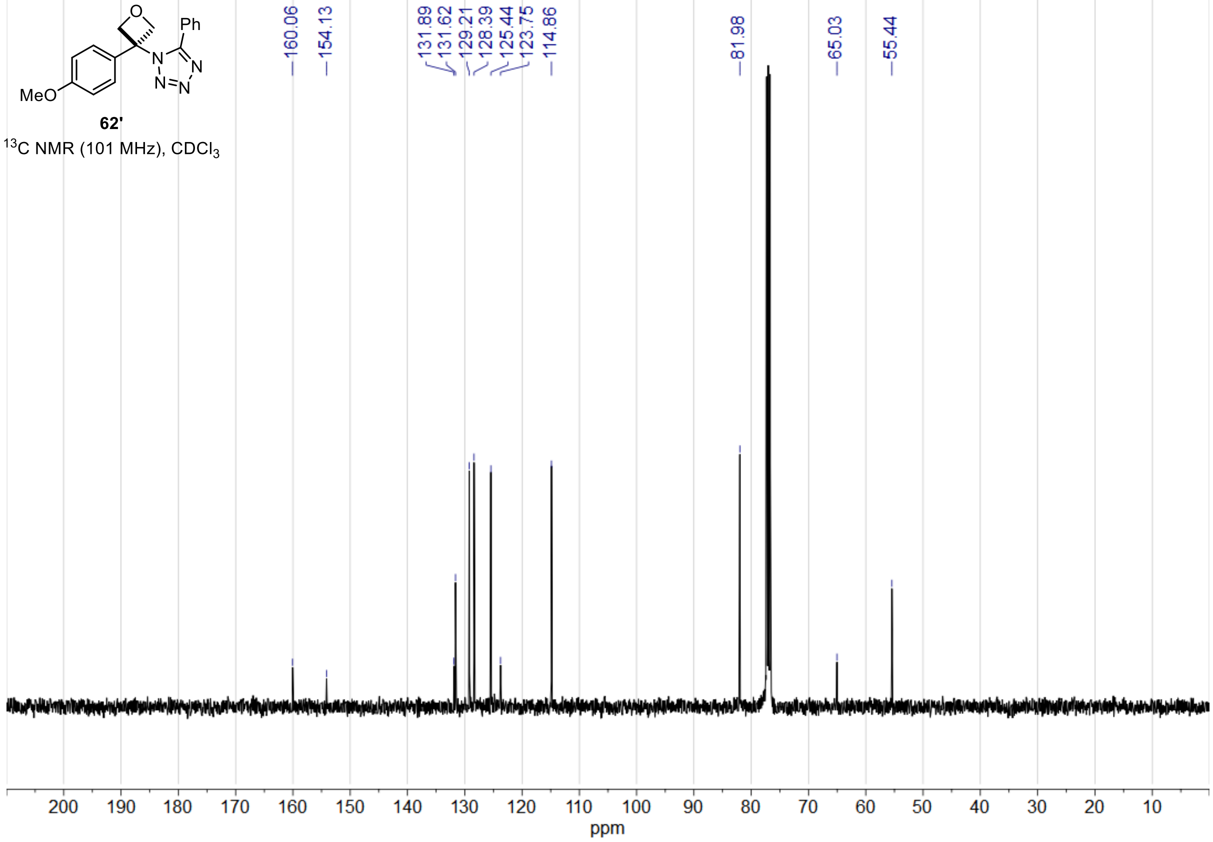

62'

^{13}C NMR (101 MHz), $CDCl_3$

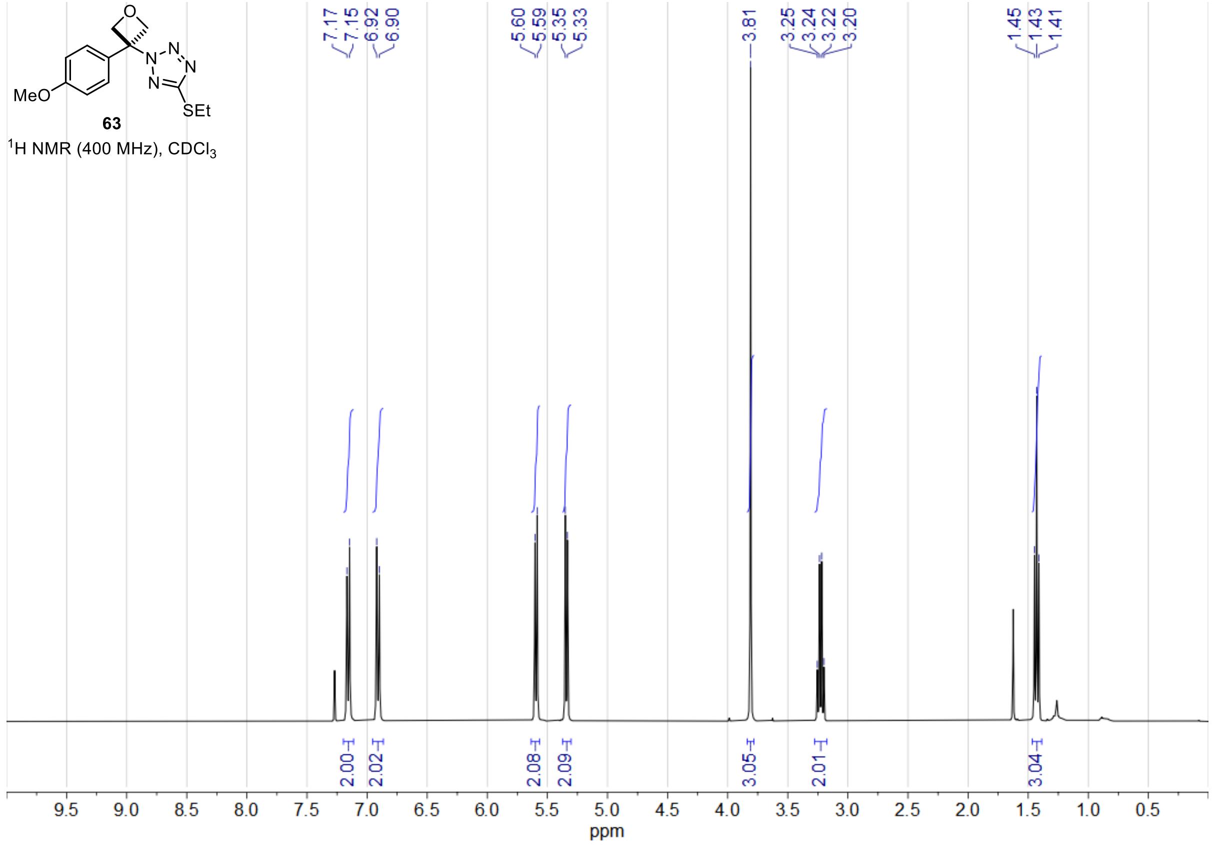
MeO
63
SEt
^{1}H NMR (400 MHz), CDCl3
7.17
7.15
6.92
6.90
5.60
5.59
5.35
5.33
3.81
3.25
3.24
3.22
3.20
1.45
1.43
1.41
2.00
2.02
2.08
2.09
3.05
2.01
3.04
ppm

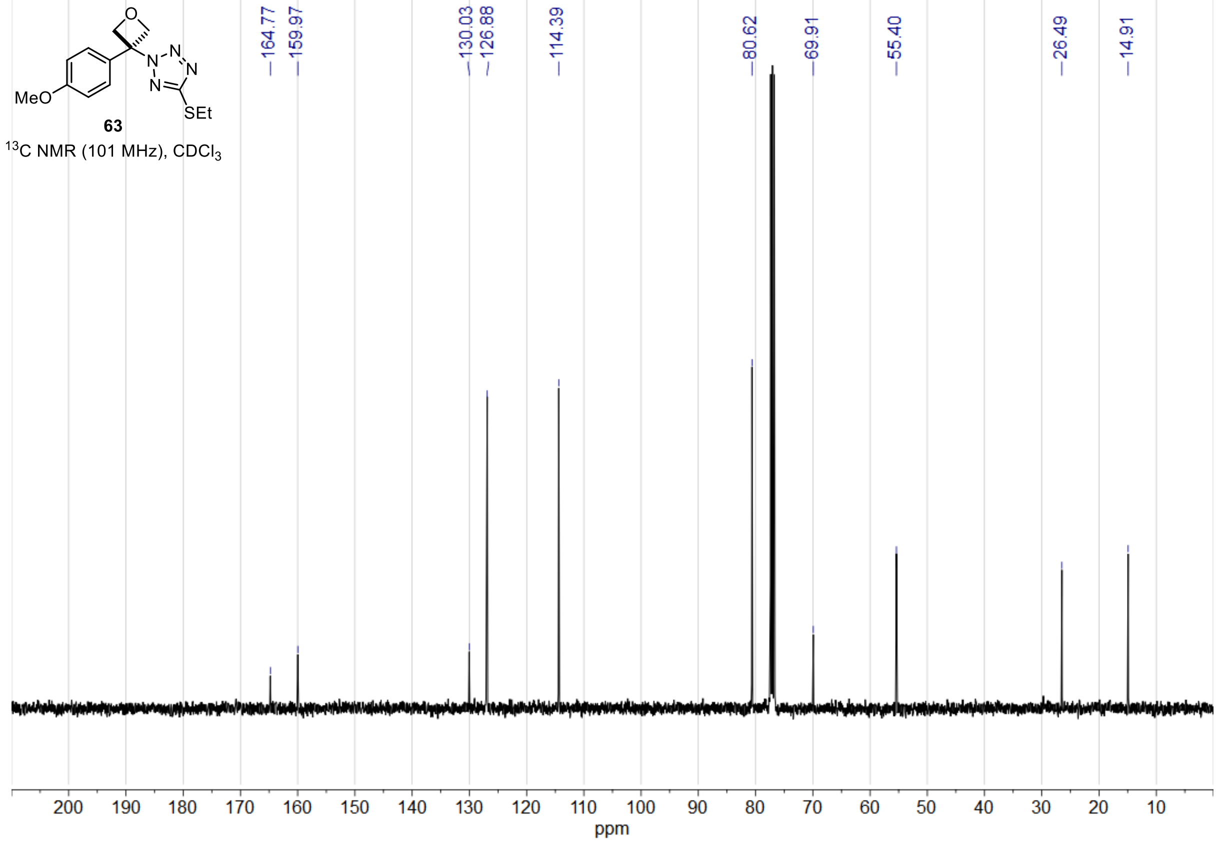
MeO
63
SEt
^{13}C NMR (101 MHz), CDCl3
164.77
159.97
130.03
126.88
114.39
80.62
69.91
55.40
26.49
14.91
ppm

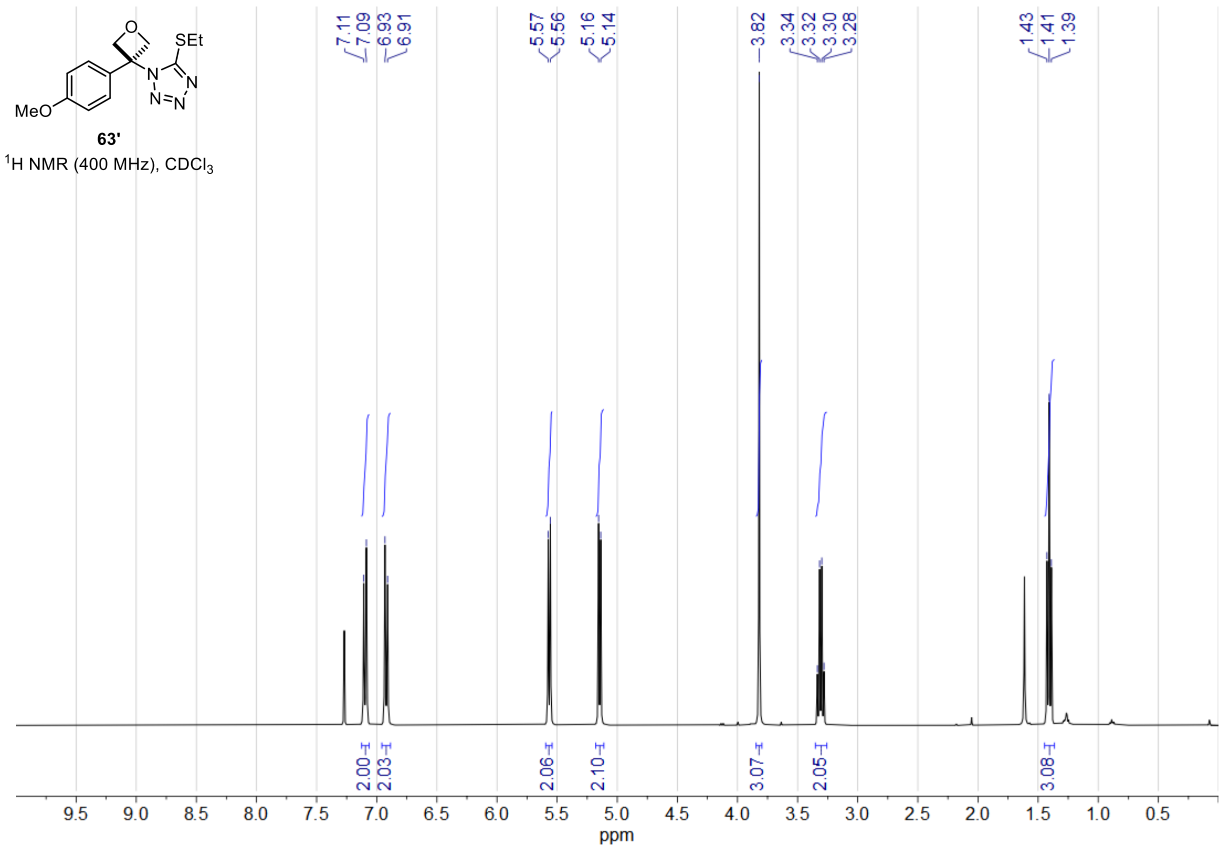
SEt
MeO
63'
^{1}H NMR (400 MHz), CDCl$_3$
7.11
7.09
6.93
6.91
5.57
5.56
5.16
5.14
3.82
3.34
3.32
3.30
3.28
1.43
1.41
1.39
2.00
2.03
2.06
2.10
3.07
2.05
3.08
ppm

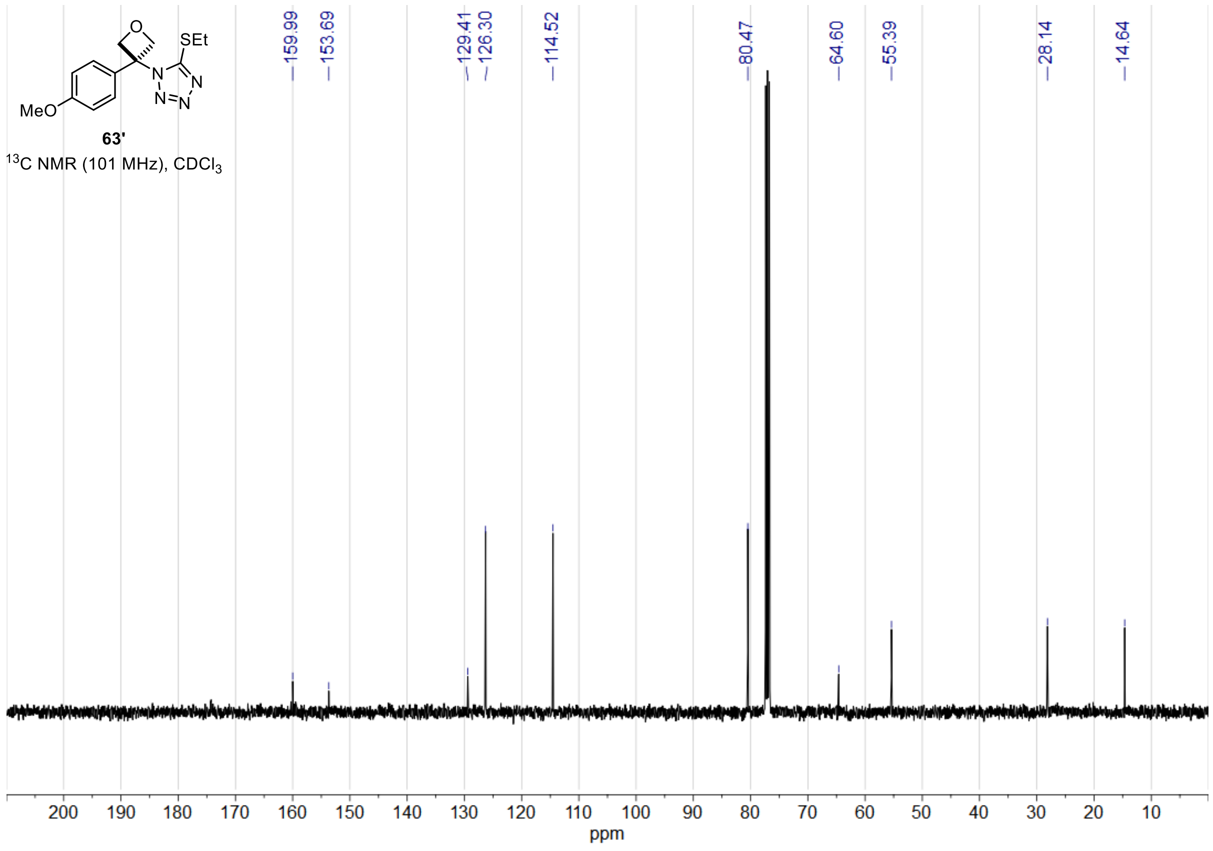
SEt
MeO
63'
^{13}C NMR (101 MHz), CDCl$_3$
159.99
153.69
129.41
126.30
114.52
80.47
64.60
55.39
28.14
14.64
ppm

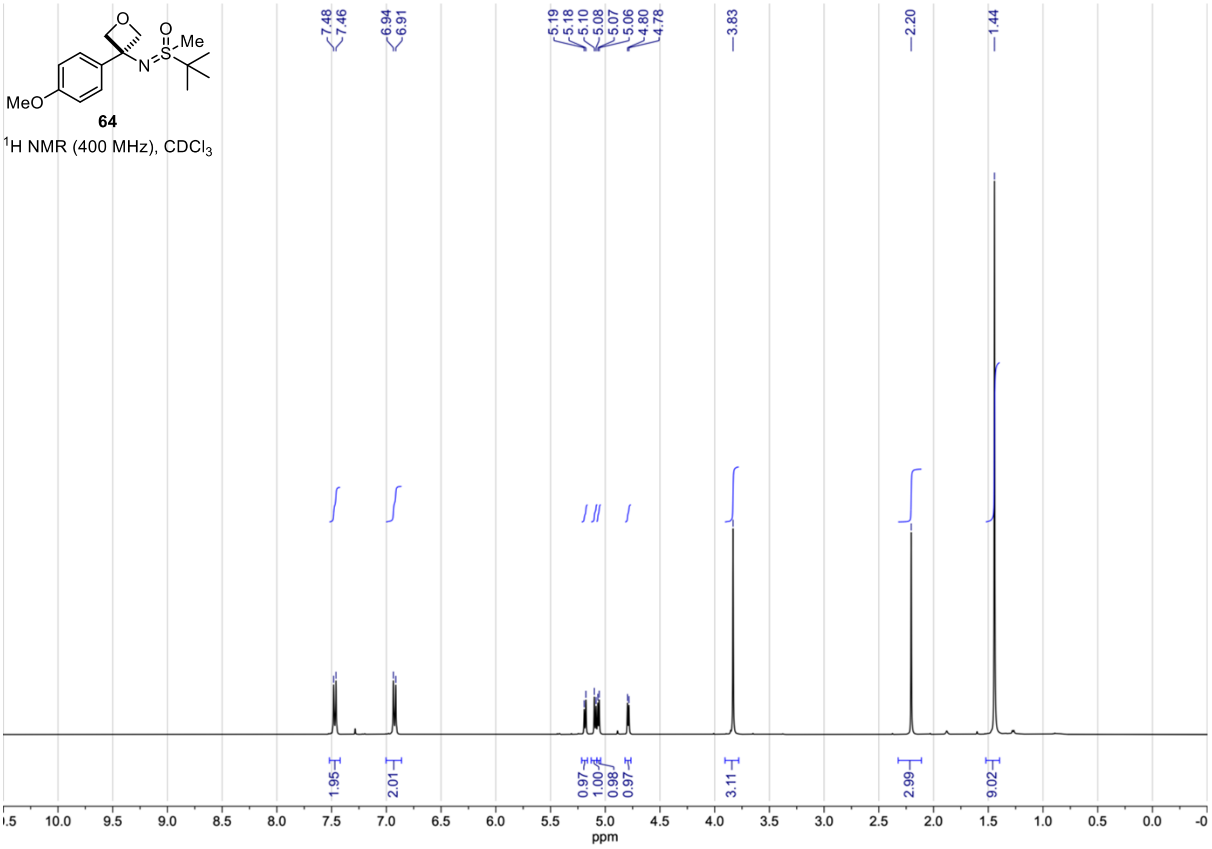
64
1H NMR (400 MHz), CDCl3
7.48
7.46
6.94
6.91
5.19
5.18
5.10
5.08
5.07
5.06
4.80
4.78
3.83
2.20
1.44
1.95
2.01
0.97
1.00
0.98
0.97
3.11
2.99
9.02
ppm

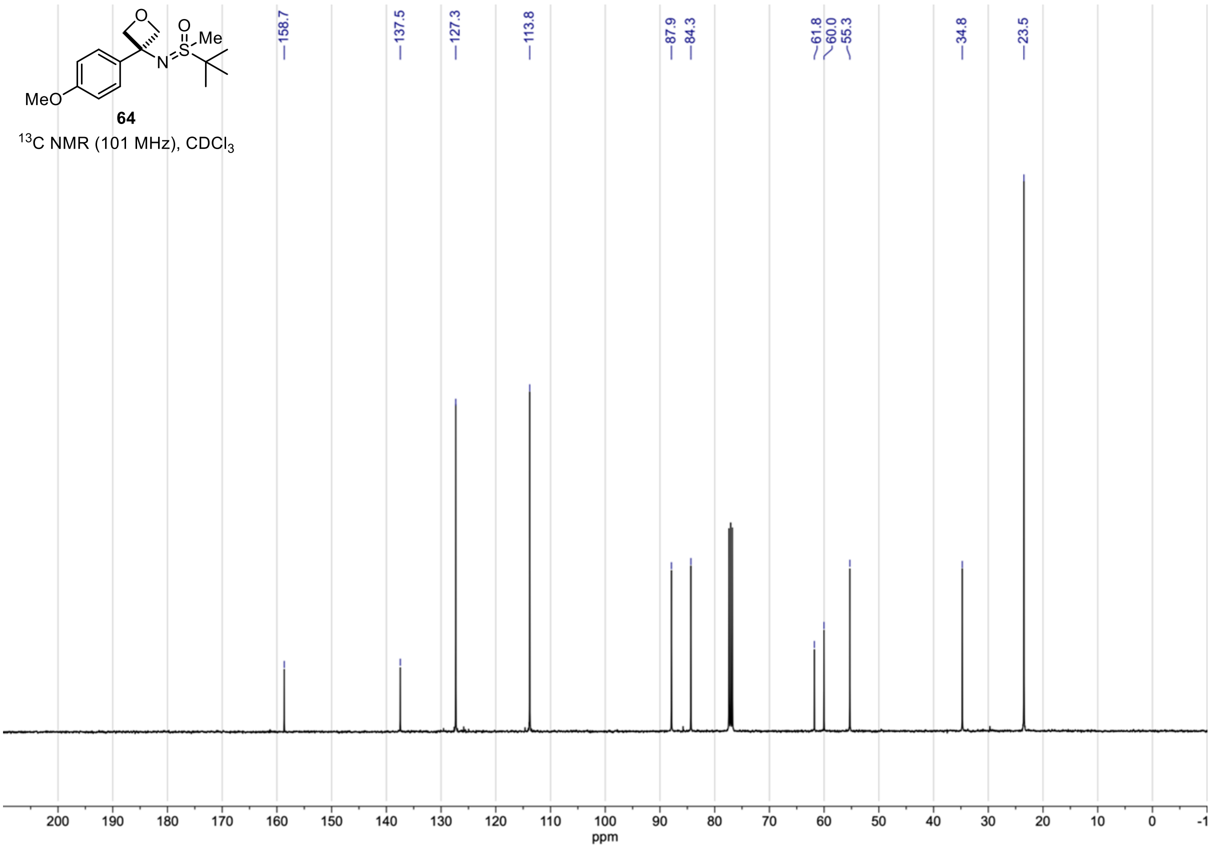
64
13C NMR (101 MHz), CDCl3
158.7
137.5
127.3
113.8
87.9
84.3
61.8
60.0
55.3
34.8
23.5
ppm

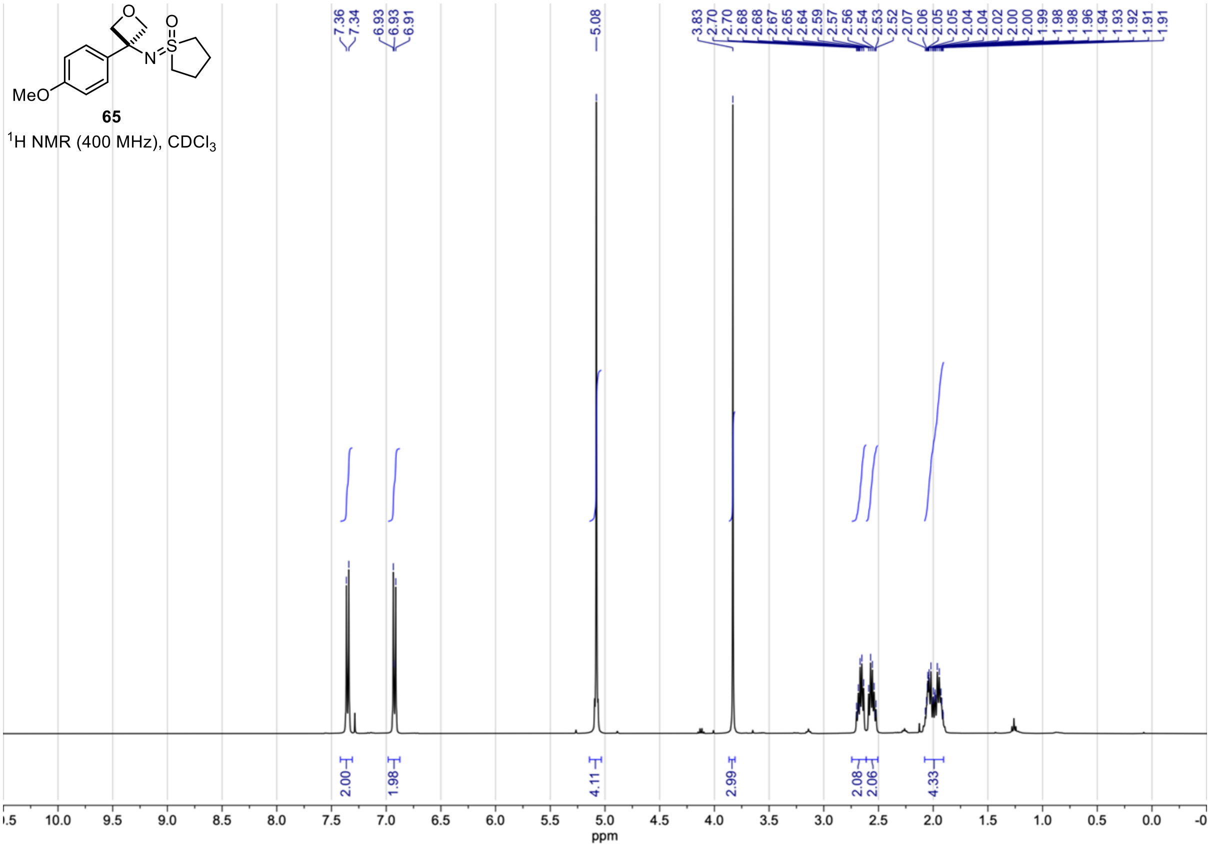
MeO
O
N
S
O
65
1H NMR (400 MHz), CDCl3
7.36
7.34
6.93
6.93
6.91
5.08
3.83
2.70
2.70
2.68
2.68
2.67
2.65
2.64
2.59
2.57
2.56
2.54
2.53
2.52
2.07
2.06
2.05
2.05
2.04
2.04
2.02
2.00
1.99
1.98
1.98
1.96
1.94
1.93
1.92
1.91
1.91
2.00
1.98
4.11
2.99
2.08
2.06
4.33
ppm

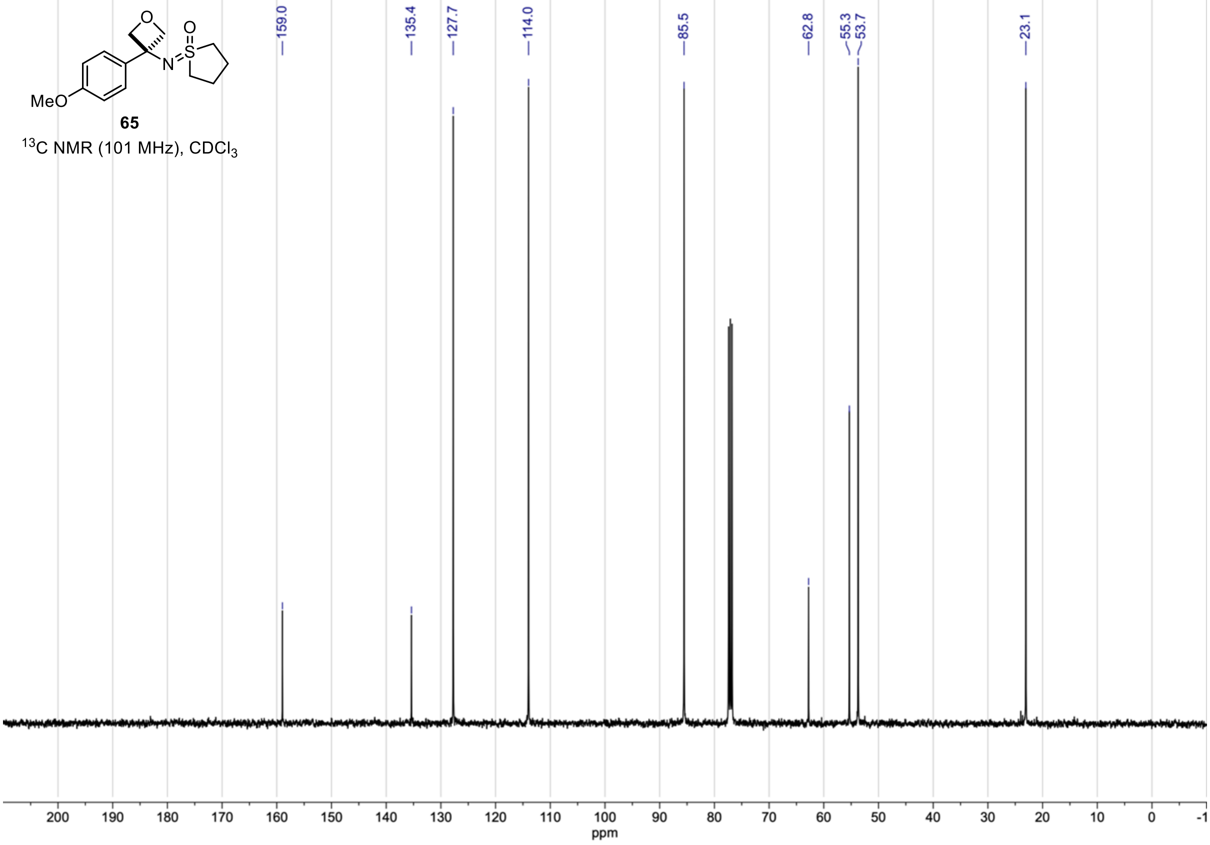
MeO
O
N
S
O
65
13C NMR (101 MHz), CDCl3
159.0
135.4
127.7
114.0
85.5
62.8
55.3
53.7
23.1
ppm

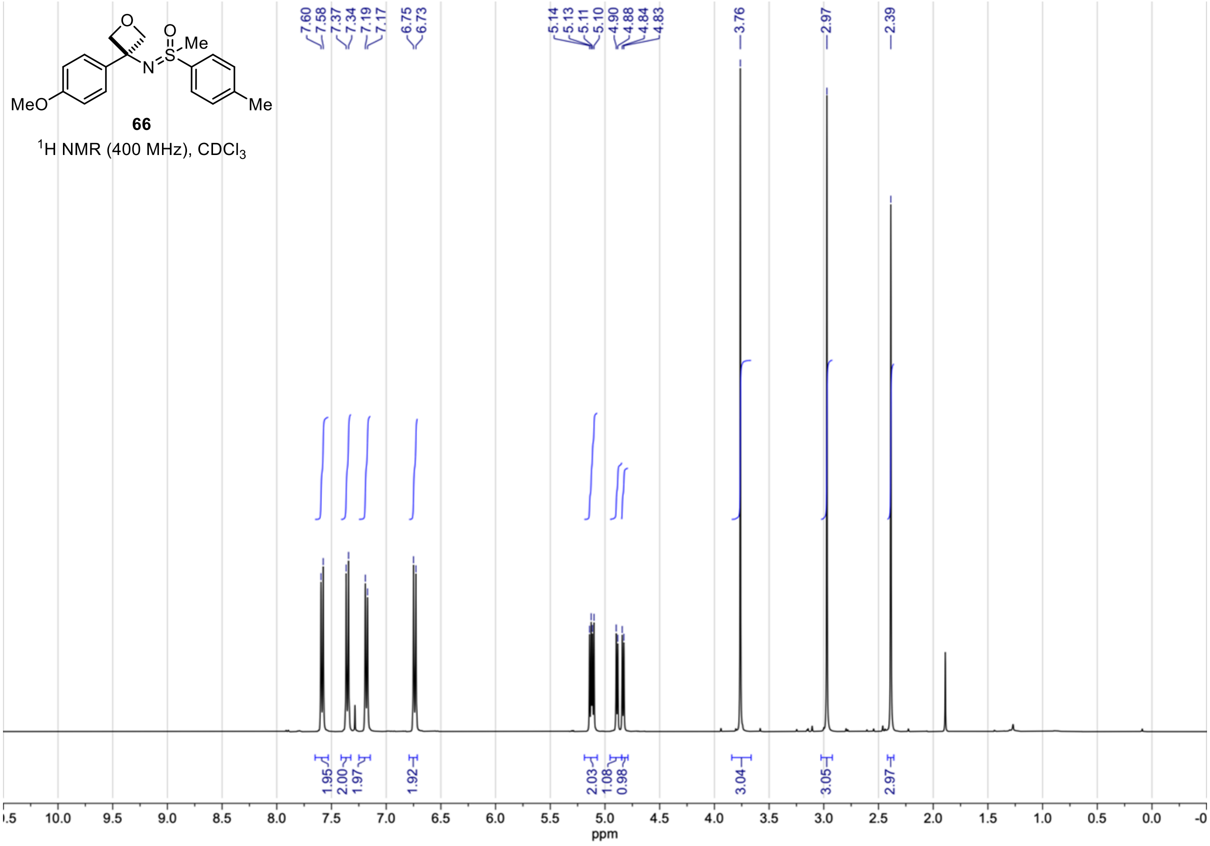
MeO
O
O
Me
N
S
Me
66
^{1}H NMR (400 MHz), CDCl$_3$
7.60
7.58
7.37
7.34
7.19
7.17
6.75
6.73
5.14
5.13
5.11
5.10
4.90
4.88
4.84
4.83
3.76
2.97
2.39
1.95
2.00
1.97
1.92
2.03
1.08
0.98
3.04
3.05
2.97
ppm

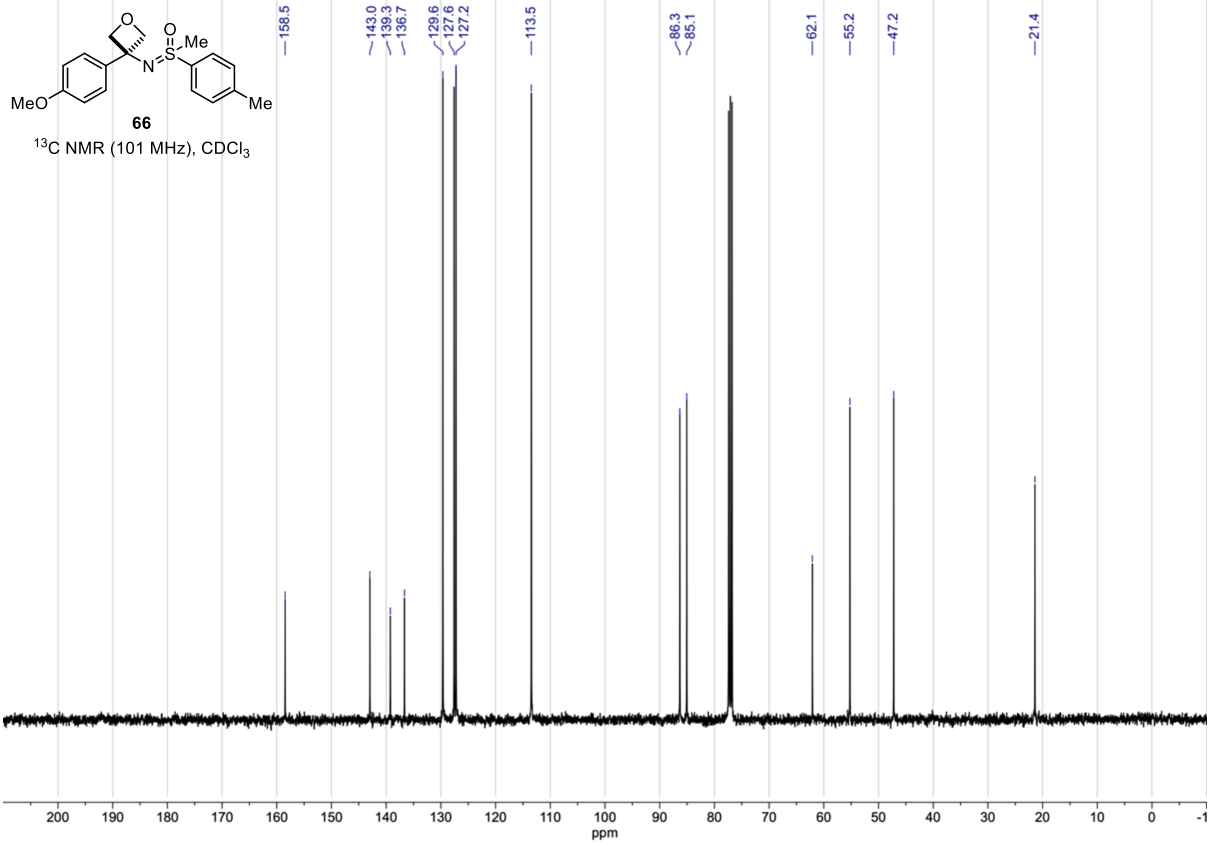
MeO
O
O
Me
N
S
Me
66
^{13}C NMR (101 MHz), CDCl$_3$
158.5
143.0
139.3
136.7
129.6
127.6
127.2
113.5
86.3
85.1
62.1
55.2
47.2
21.4
ppm

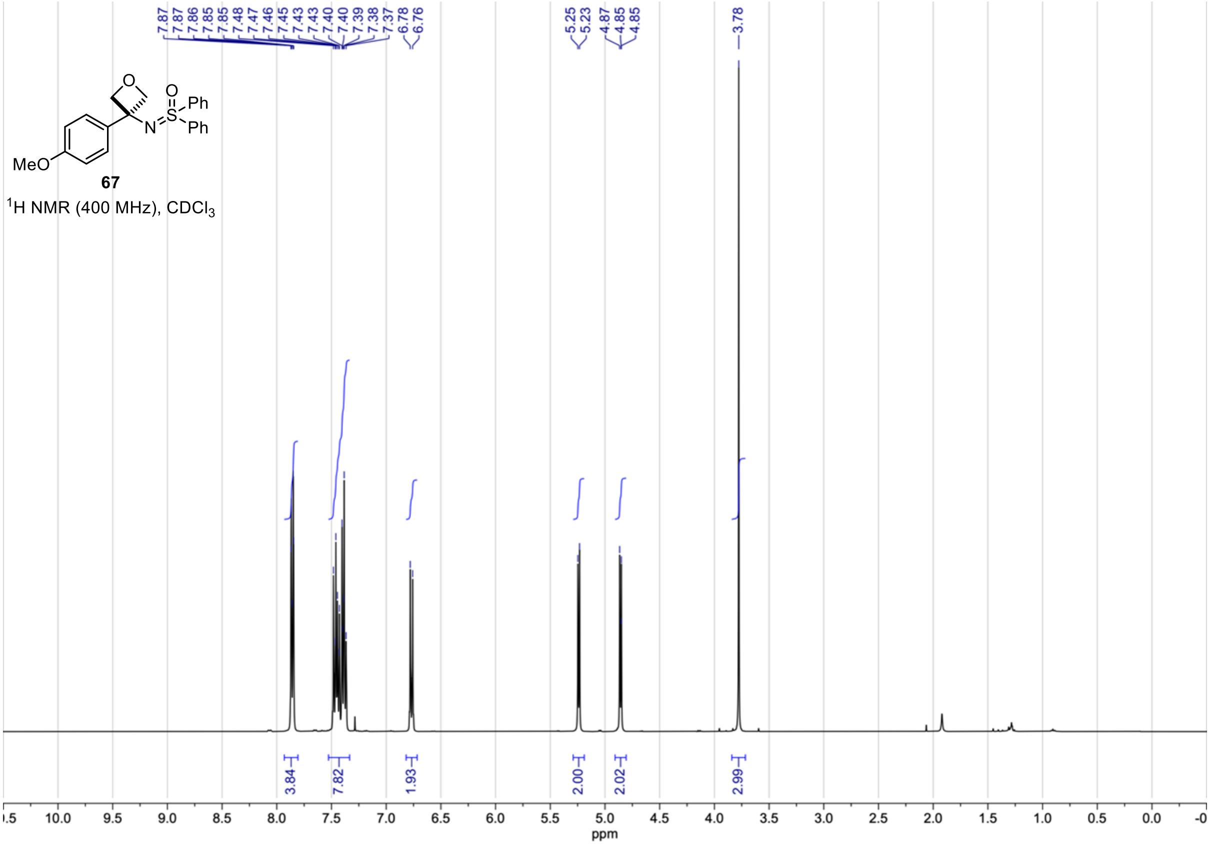
7.87
7.87
7.86
7.85
7.85
7.48
7.47
7.46
7.45
7.43
7.43
7.43
7.40
7.40
7.39
7.38
7.37
6.78
6.76
5.25
5.23
4.87
4.85
4.85
3.78
O
Ph
N
S
Ph
MeO
67
¹H NMR (400 MHz), CDCl₃
3.84
7.82
1.93
2.00
2.02
2.99
ppm

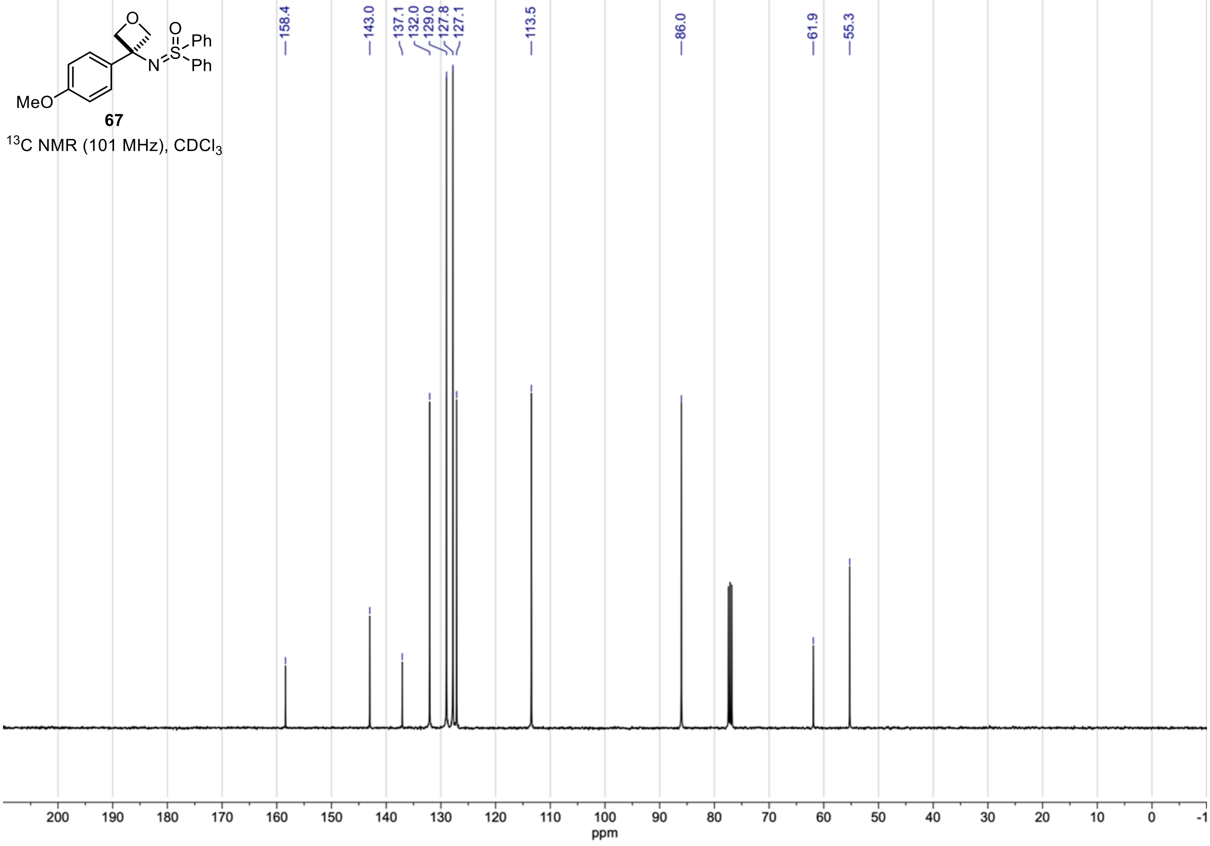
158.4
143.0
137.1
132.0
129.0
127.8
127.1
113.5
86.0
61.9
55.3
O
Ph
N
S
Ph
MeO
67
¹³C NMR (101 MHz), CDCl₃
ppm

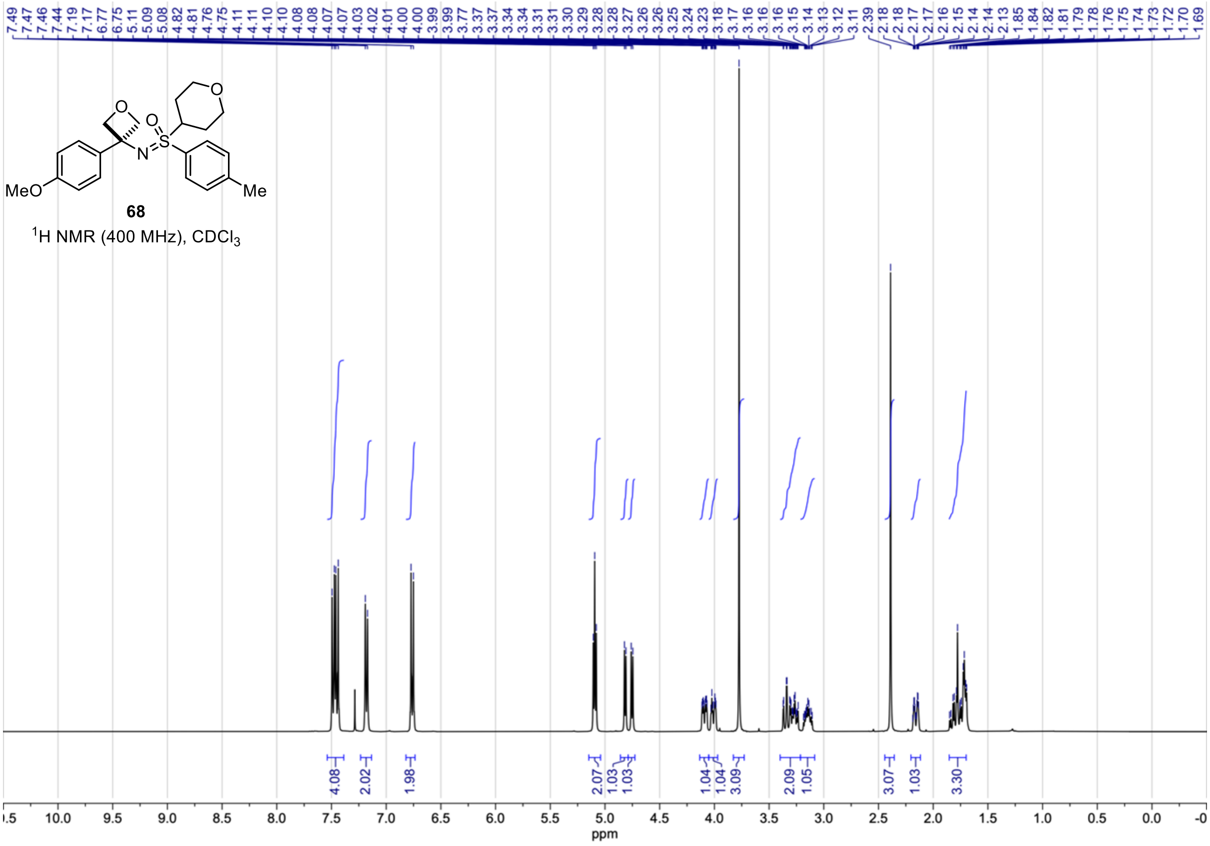
7.49
7.47
7.46
7.44
7.19
7.17
6.77
6.75
5.11
5.09
5.08
4.82
4.81
4.76
4.75
4.11
4.11
4.10
4.08
4.08
4.07
4.03
4.02
4.01
4.00
4.00
3.99
3.99
3.77
3.37
3.37
3.34
3.34
3.31
3.31
3.30
3.29
3.28
3.27
3.26
3.25
3.24
3.23
3.18
3.17
3.16
3.16
3.15
3.14
3.13
3.12
3.11
2.39
2.18
2.18
2.17
2.16
2.15
2.14
2.13
1.85
1.84
1.82
1.81
1.79
1.78
1.76
1.75
1.74
1.73
1.72
1.70
1.69
MeO
Me
68
1H NMR (400 MHz), CDCl3
4.08
2.02
1.98
2.07
1.03
1.03
1.04
1.04
3.09
2.09
1.05
3.07
1.03
3.30
ppm

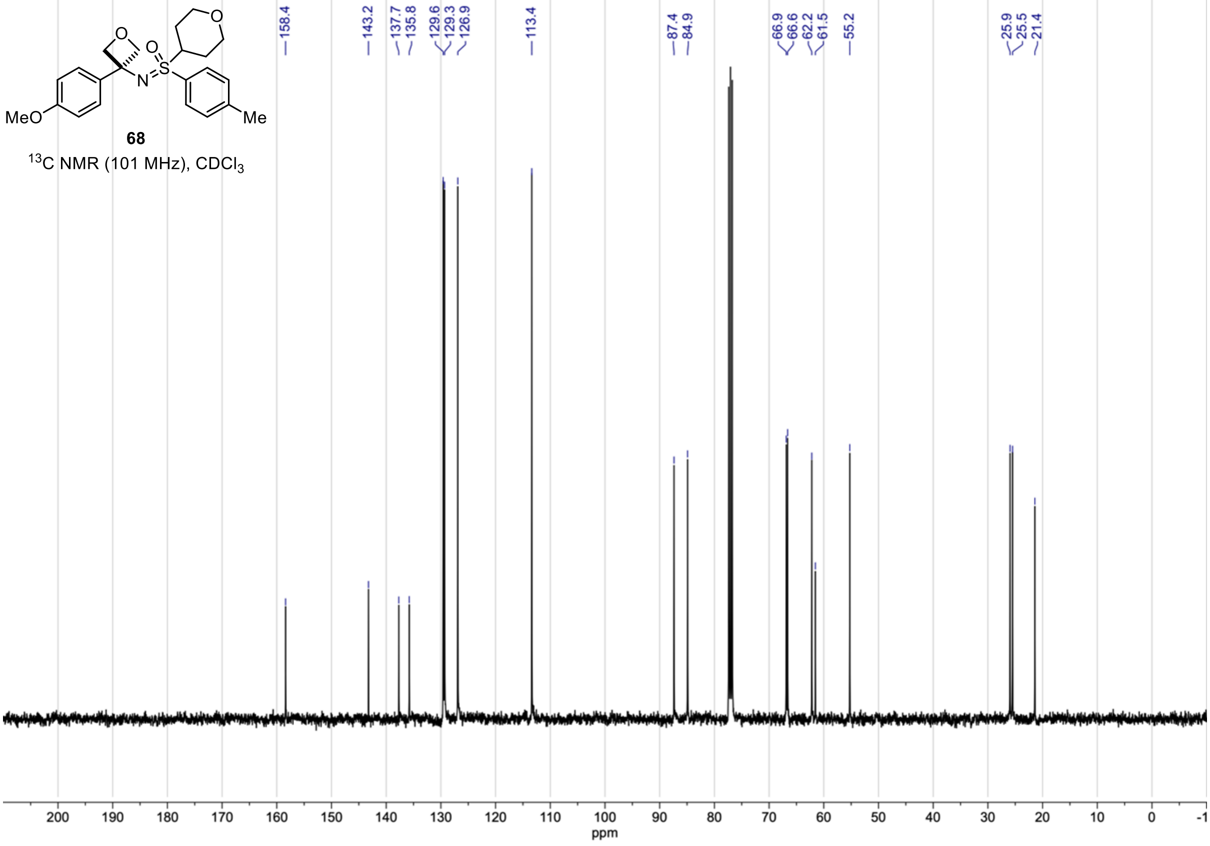
158.4
143.2
137.7
135.8
129.6
129.3
126.9
113.4
87.4
84.9
66.9
66.6
62.2
61.5
55.2
25.9
25.5
21.4
MeO
Me
68
13C NMR (101 MHz), CDCl3
ppm

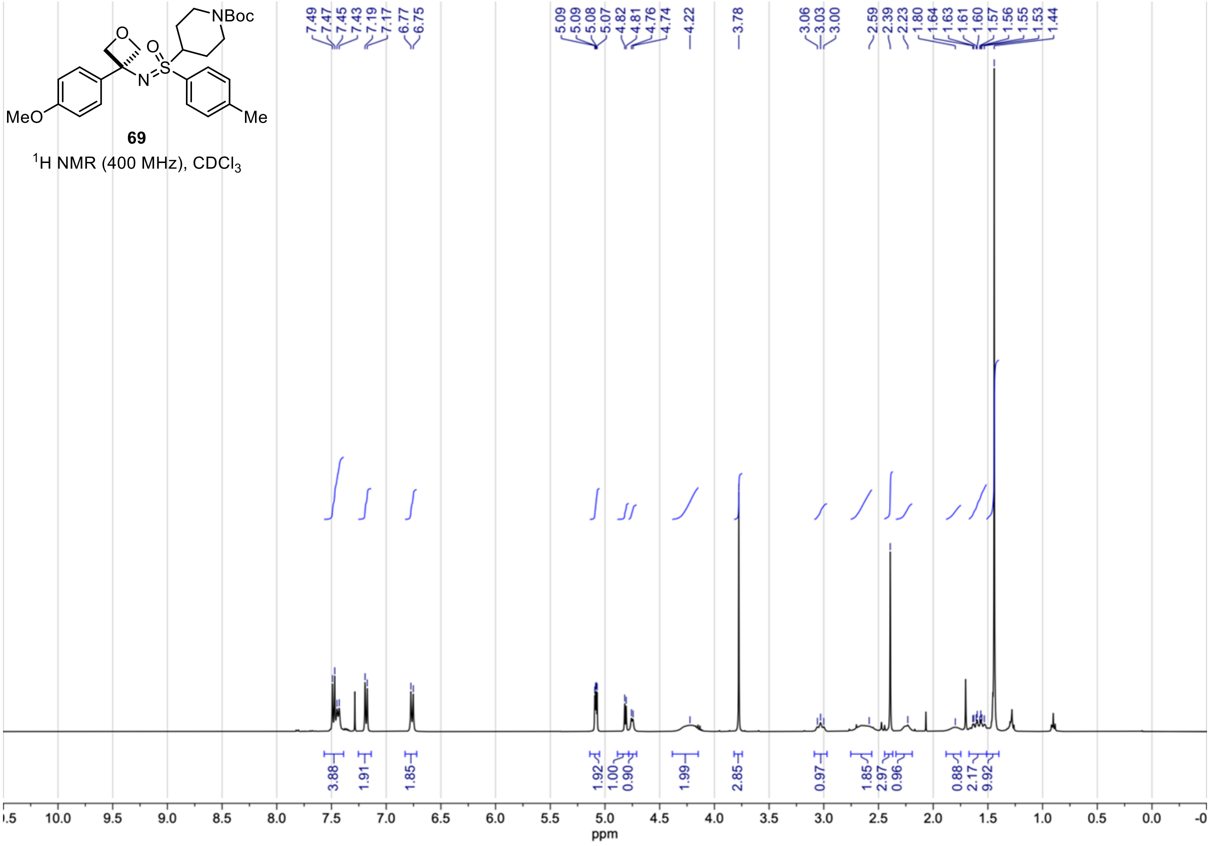

69

^{1}H NMR (400 MHz), $CDCl_3$

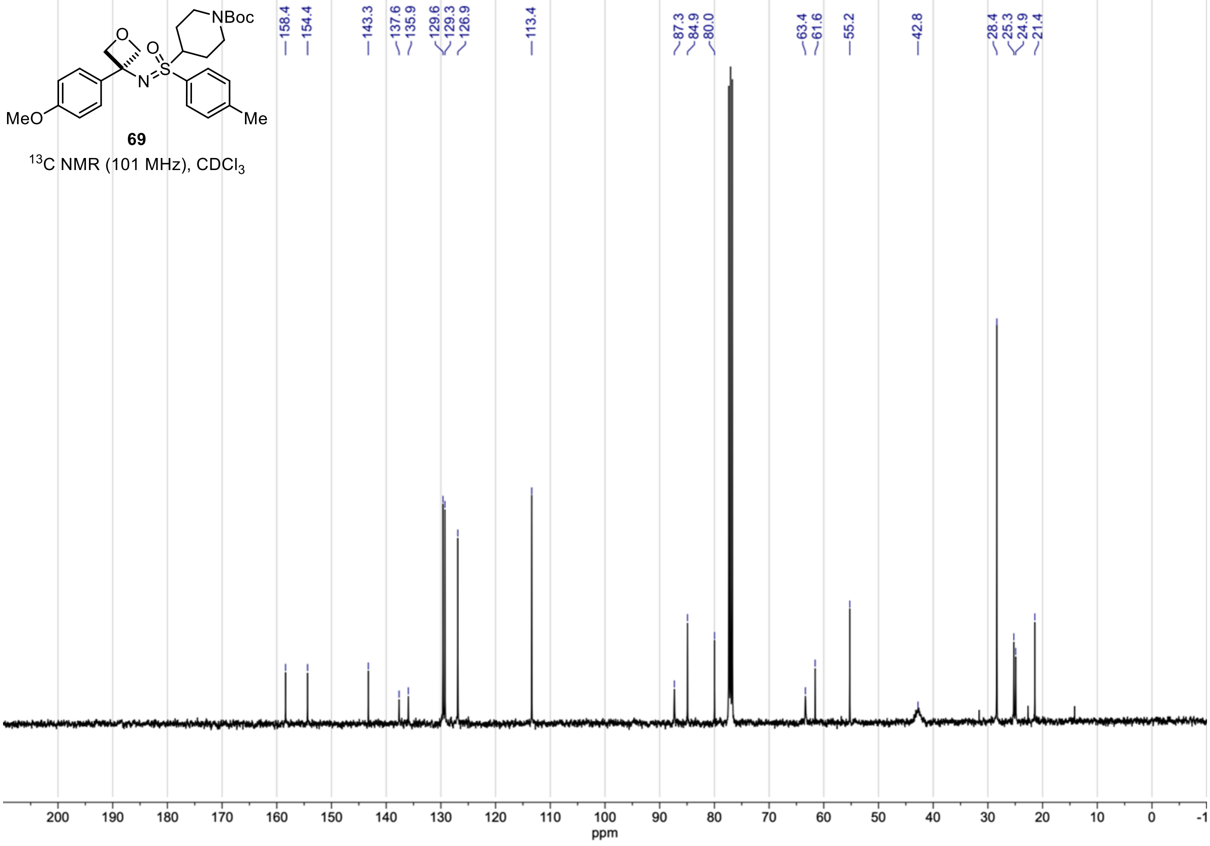

69

^{13}C NMR (101 MHz), $CDCl_3$

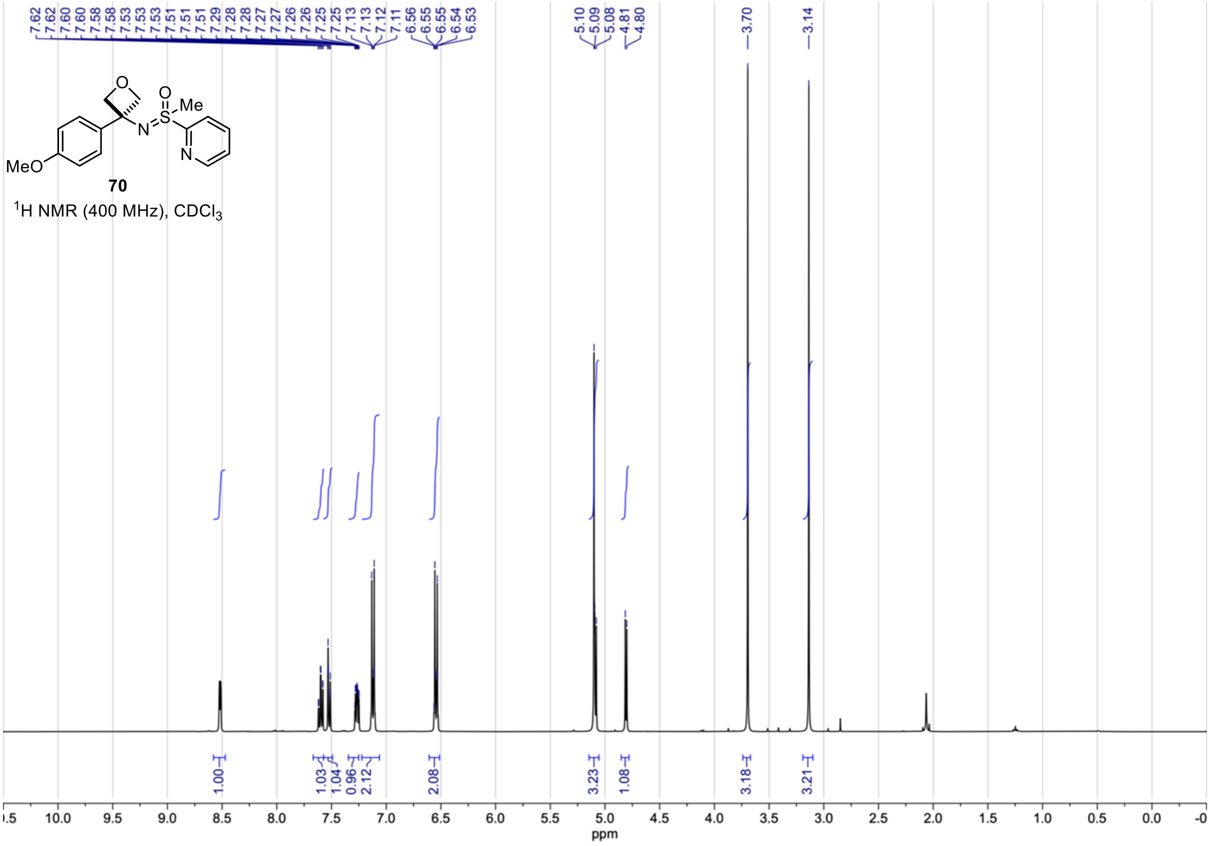

MeO
O
N
S
Me
70
1H NMR (400 MHz), CDCl3
7.62 7.62 7.60 7.58 7.58 7.53 7.53 7.53 7.51 7.51 7.51 7.29 7.28 7.28 7.27 7.27 7.26 7.26 7.25 7.25 7.25 7.13 7.13 7.12 7.11 6.56 6.55 6.55 6.54 6.53
5.10 5.09 5.08 4.81 4.80
3.70
3.14
1.00 1.03 1.04 0.96 2.12 2.08 3.23 1.08 3.18 3.21
ppm

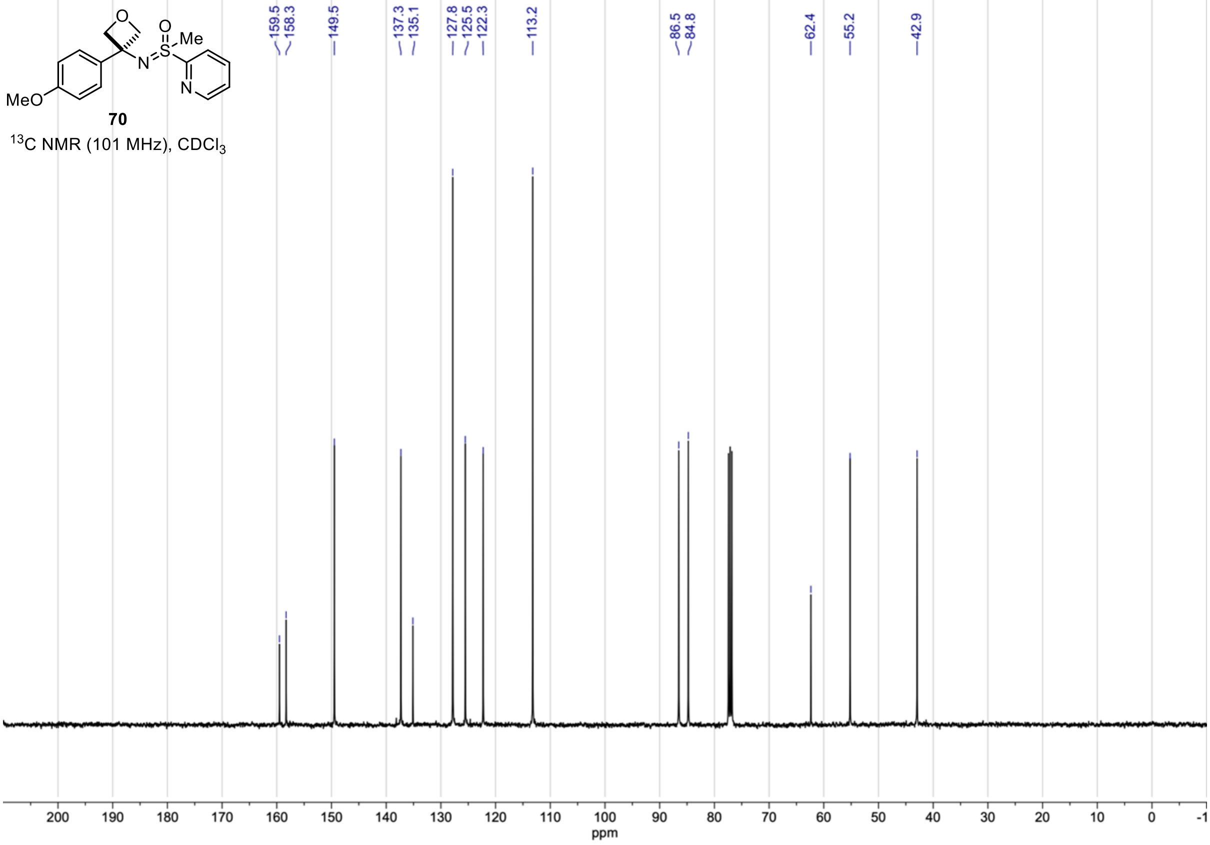

MeO
O
N
S
Me
70
13C NMR (101 MHz), CDCl3
159.5 158.3 149.5 137.3 135.1 127.8 125.5 122.3 113.2 86.5 84.8 62.4 55.2 42.9
ppm

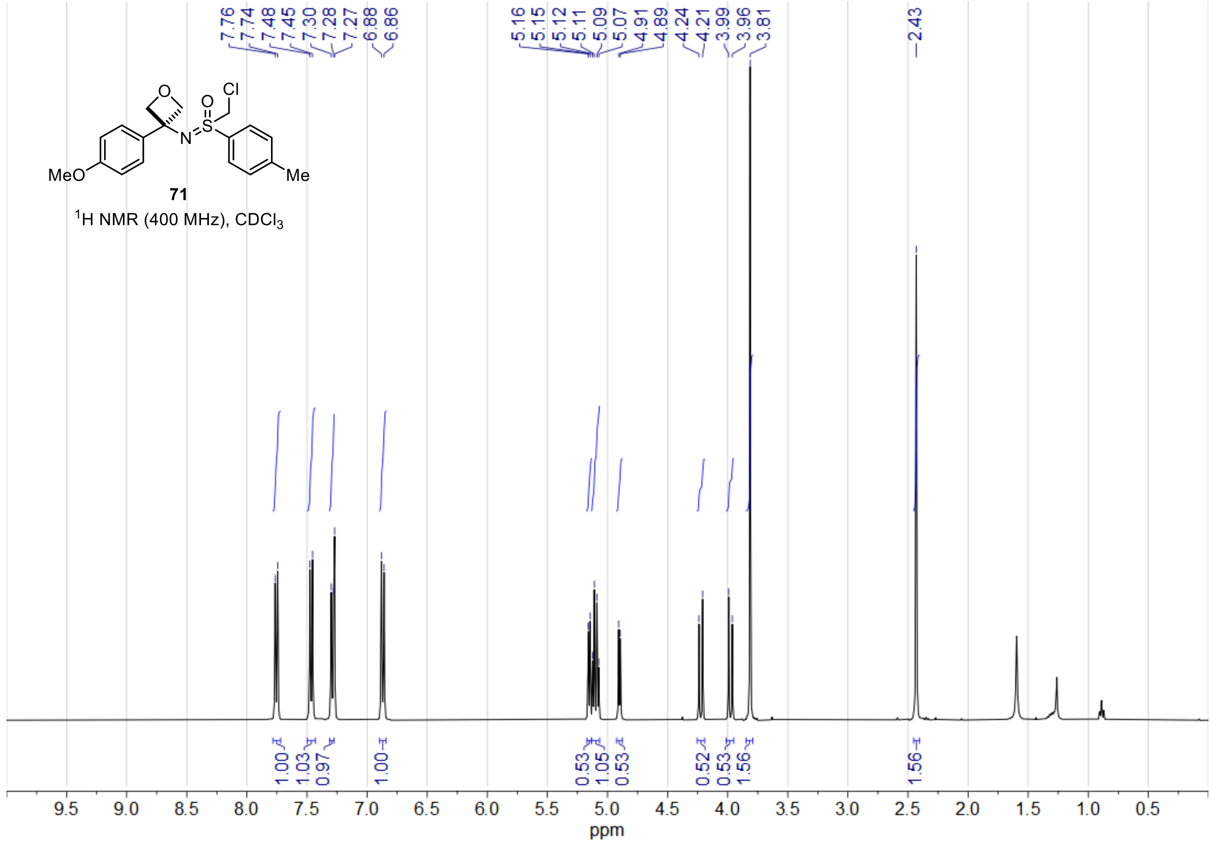
7.76
7.74
7.48
7.45
7.30
7.28
7.27
6.88
6.86
5.16
5.15
5.12
5.11
5.09
5.07
4.91
4.89
4.24
4.21
3.99
3.96
3.81
2.43
O
O
Cl
N
S
MeO
Me
71
^{1}H NMR (400 MHz), CDCl$_3$
1.00
1.03
0.97
1.00
0.53
1.05
0.53
0.52
0.53
1.56
1.56
9.5
9.0
8.5
8.0
7.5
7.0
6.5
6.0
5.5
5.0
4.5
4.0
3.5
3.0
2.5
2.0
1.5
1.0
0.5
ppm

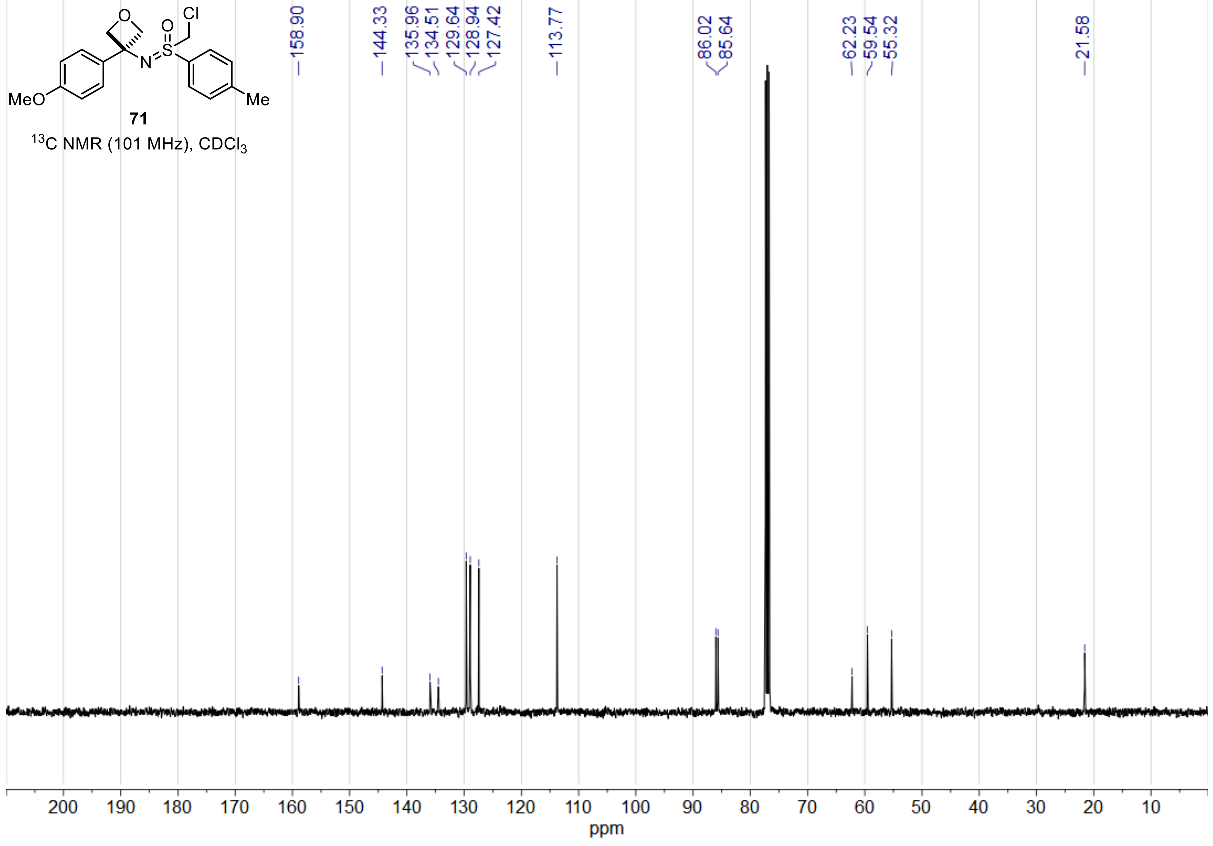
158.90
144.33
135.96
134.51
129.64
128.94
127.42
113.77
86.02
85.64
62.23
59.54
55.32
21.58
O
O
Cl
N
S
MeO
Me
71
^{13}C NMR (101 MHz), CDCl$_3$
200
190
180
170
160
150
140
130
120
110
100
90
80
70
60
50
40
30
20
10
ppm

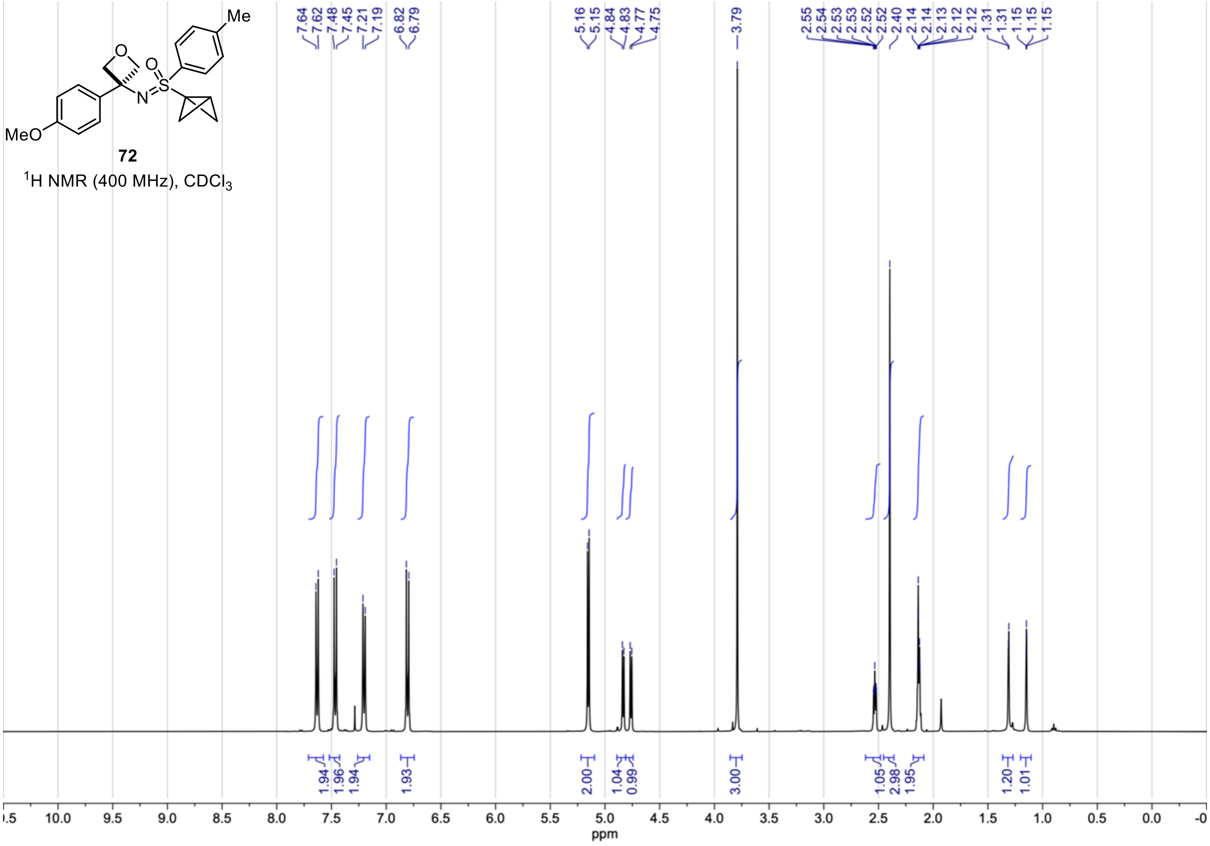
Me
O
O
N=S
MeO
72
^{1}H NMR (400 MHz), CDCl$_3$
7.64
7.62
7.48
7.45
7.21
7.19
6.82
6.79
5.16
5.15
4.84
4.83
4.77
4.75
3.79
2.55
2.54
2.53
2.53
2.52
2.52
2.40
2.14
2.14
2.13
2.12
2.12
1.31
1.31
1.15
1.15
1.94
1.96
1.94
1.93
2.00
1.04
0.99
3.00
1.05
2.98
1.95
1.20
1.01
ppm

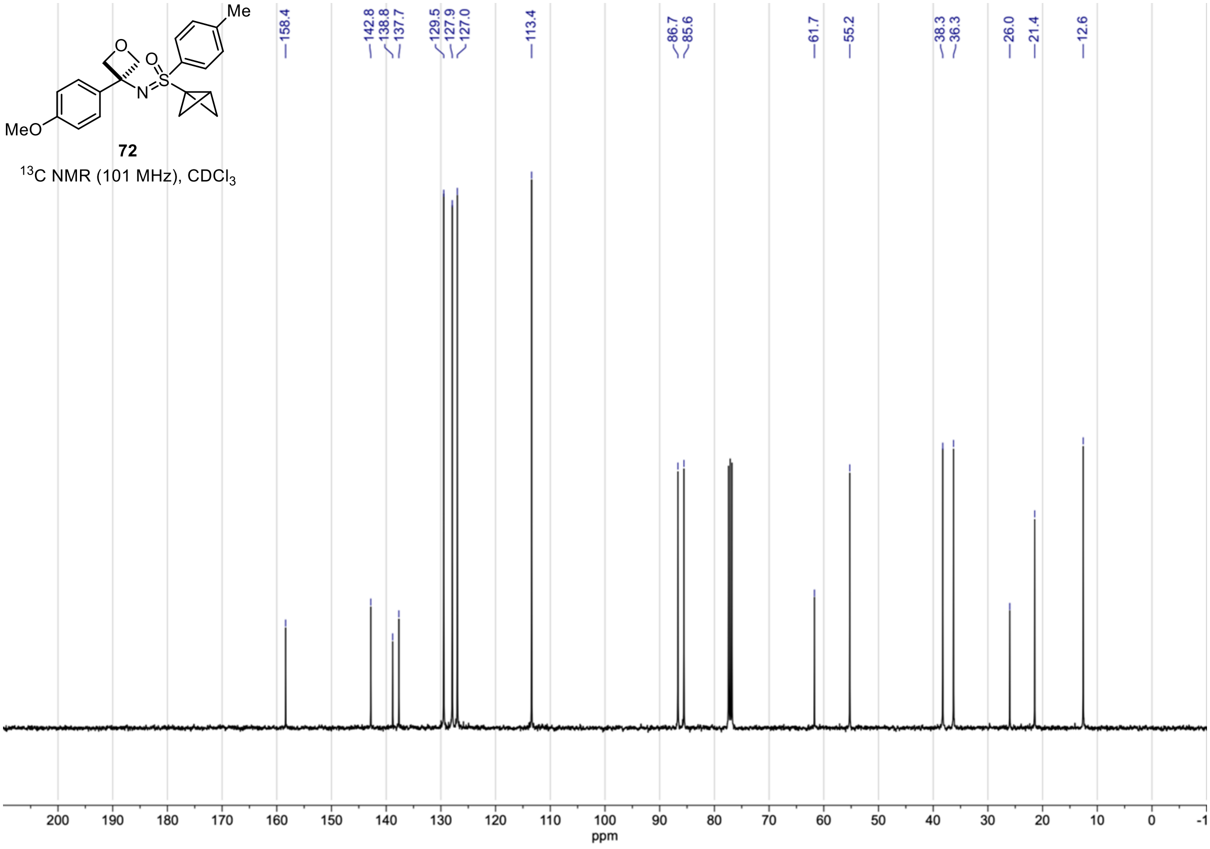
Me
O
O
N=S
MeO
72
^{13}C NMR (101 MHz), CDCl$_3$
158.4
142.8
138.8
137.7
129.5
127.9
127.0
113.4
86.7
85.6
61.7
55.2
38.3
36.3
26.0
21.4
12.6
ppm

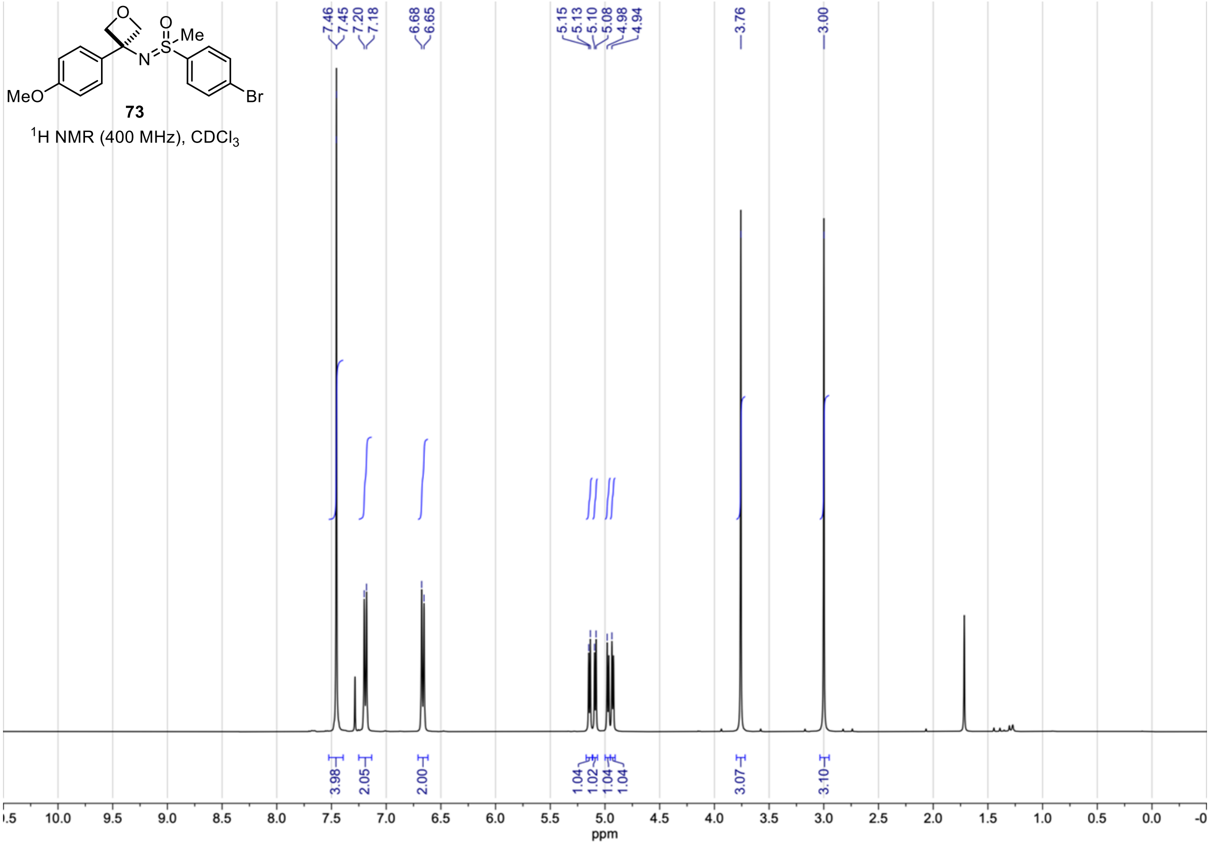
MeO
O
O
S
Me
N
Br
73
¹H NMR (400 MHz), CDCl₃
7.46
7.45
7.20
7.18
6.68
6.65
5.15
5.13
5.10
5.08
4.98
4.94
3.76
3.00
3.98
2.05
2.00
1.04
1.02
1.04
1.04
3.07
3.10
ppm

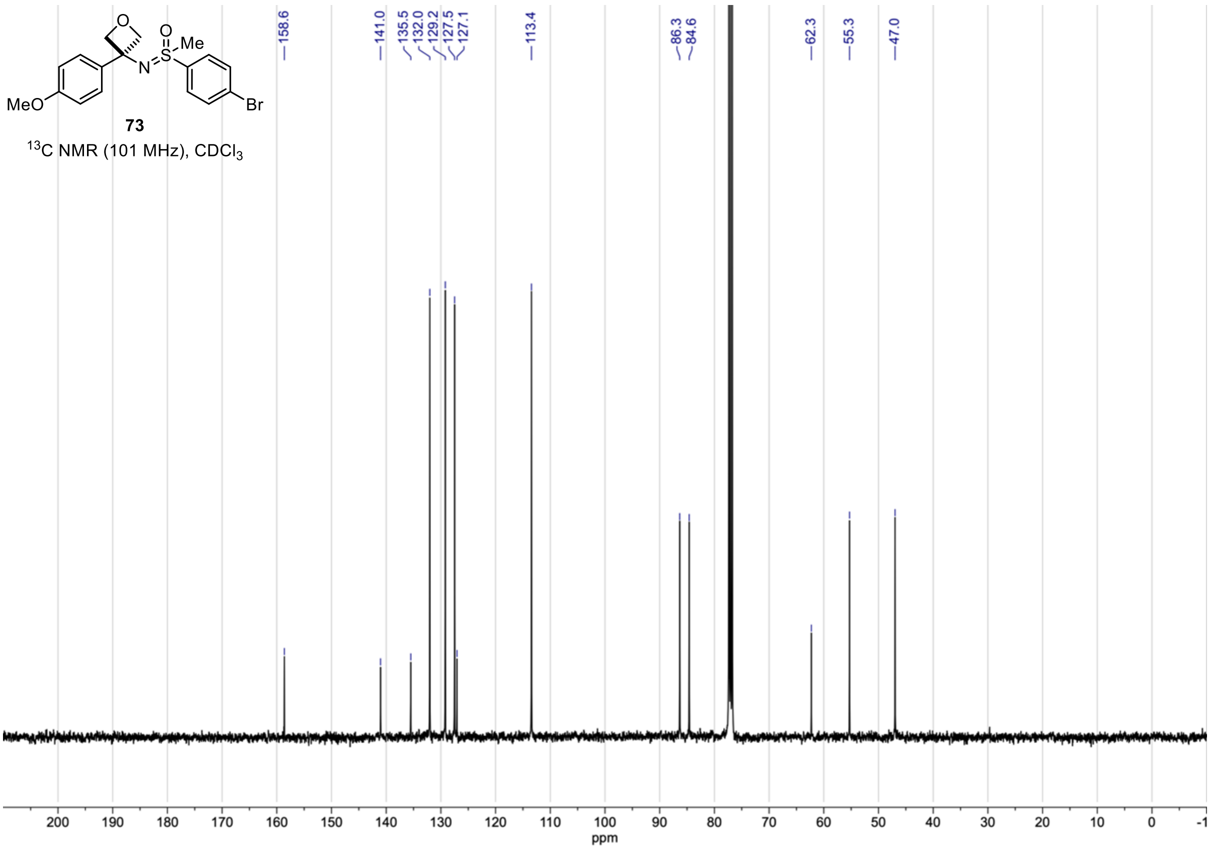
MeO
O
O
S
Me
N
Br
73
¹³C NMR (101 MHz), CDCl₃
158.6
141.0
135.5
132.0
129.2
127.5
127.1
113.4
86.3
84.6
62.3
55.3
47.0
ppm

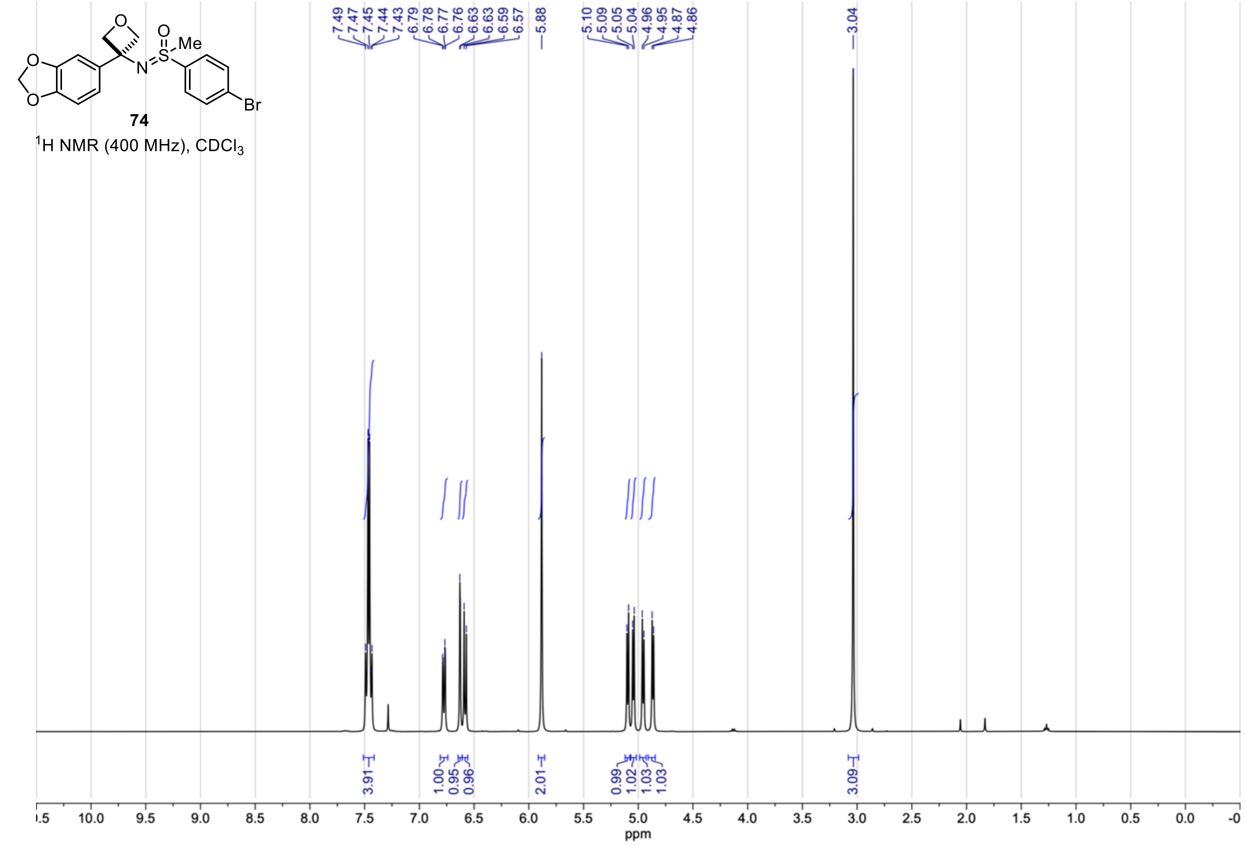
74
^{1}H NMR (400 MHz), CDCl$_3$
7.49 7.47 7.45 7.44 7.43 6.79 6.78 6.77 6.76 6.63 6.63 6.59 6.57 5.88 5.10 5.09 5.05 5.04 4.96 4.95 4.87 4.86 3.04
3.91 1.00 0.95 0.96 2.01 0.99 1.02 1.03 1.03 3.09
ppm

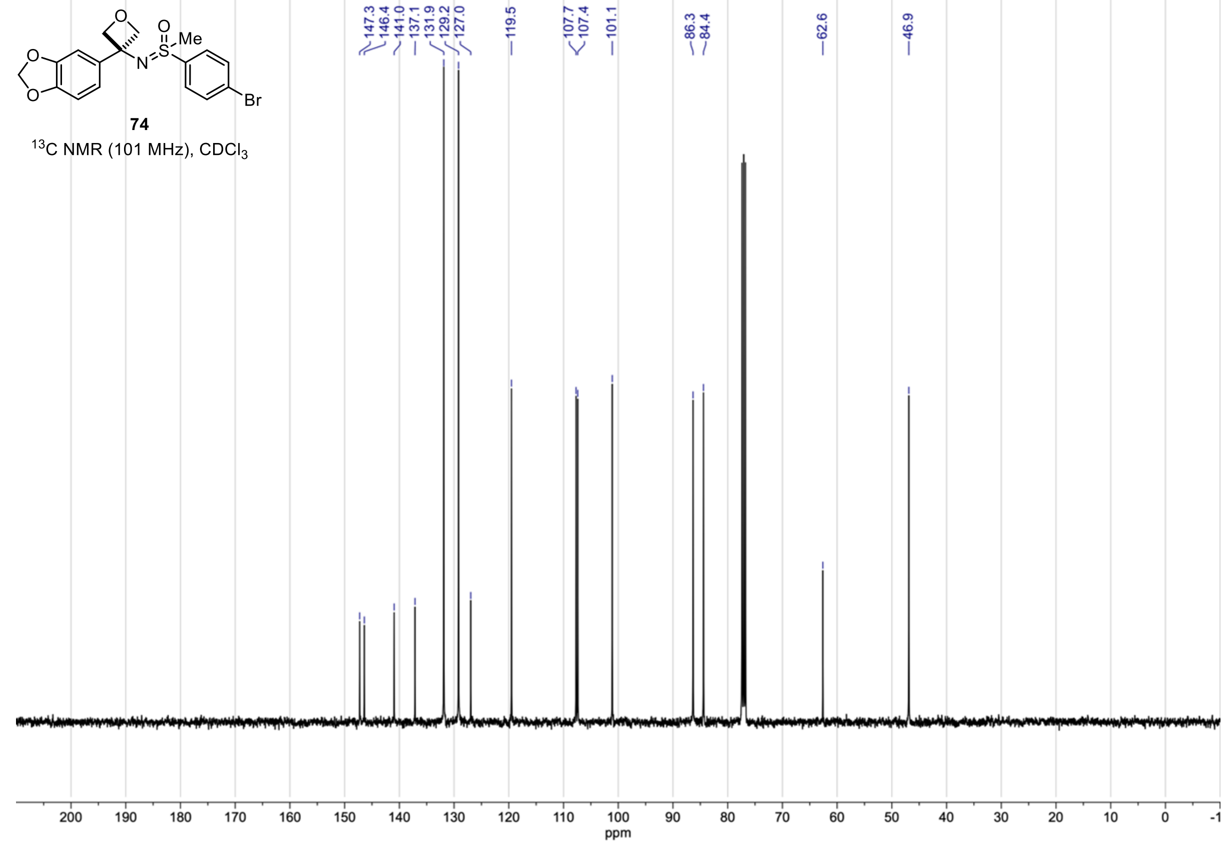
74
^{13}C NMR (101 MHz), CDCl$_3$
147.3 146.4 141.0 137.1 131.9 129.2 127.0 119.5 107.7 107.4 101.1 86.3 84.4 62.6 46.9
ppm

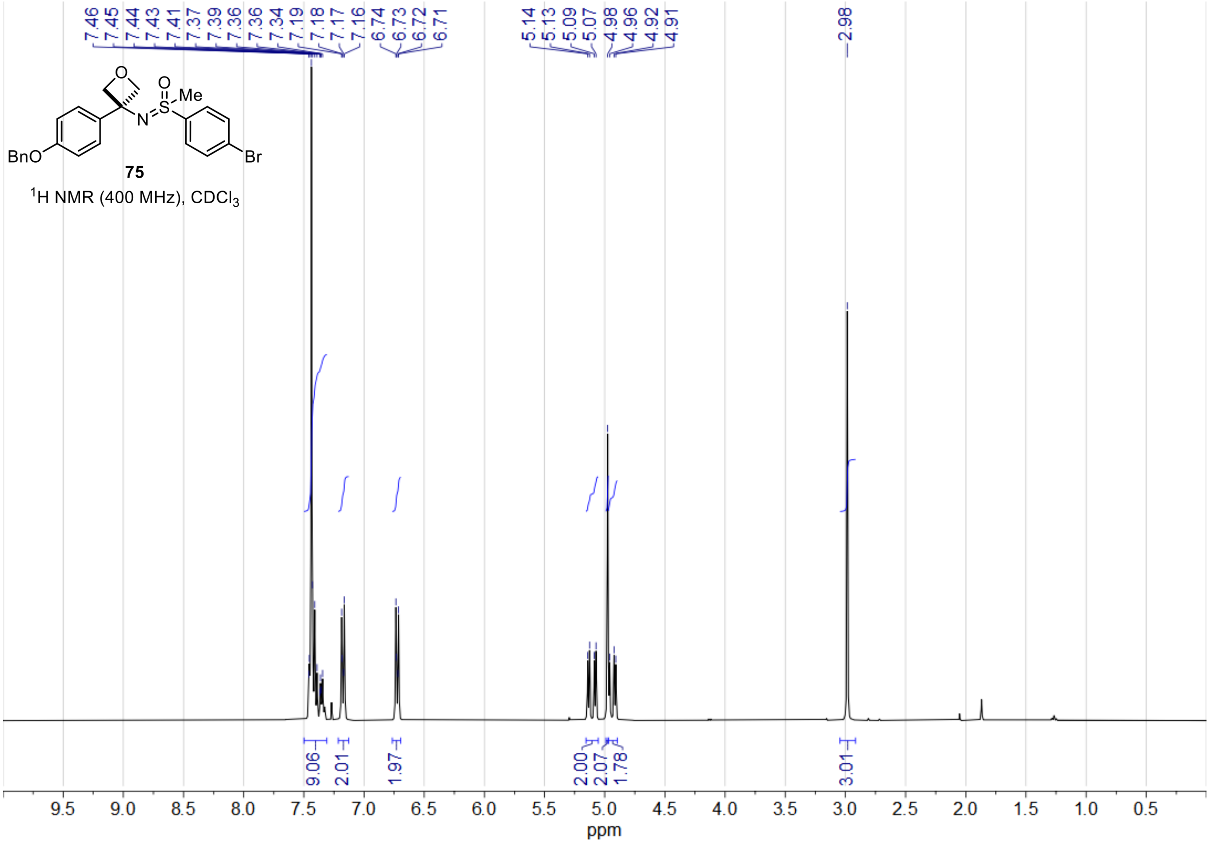
7.46 7.45 7.44 7.43 7.41 7.37 7.39 7.36 7.36 7.34 7.19 7.18 7.17 7.16 6.74 6.73 6.72 6.71
5.14 5.13 5.09 5.07 4.98 4.96 4.92 4.91
2.98
BnO
O
Me
N
S
Br
75
^{1}H NMR (400 MHz), CDCl$_3$
9.06 2.01 1.97 2.00 2.07 1.78 3.01
9.5 9.0 8.5 8.0 7.5 7.0 6.5 6.0 5.5 5.0 4.5 4.0 3.5 3.0 2.5 2.0 1.5 1.0 0.5
ppm

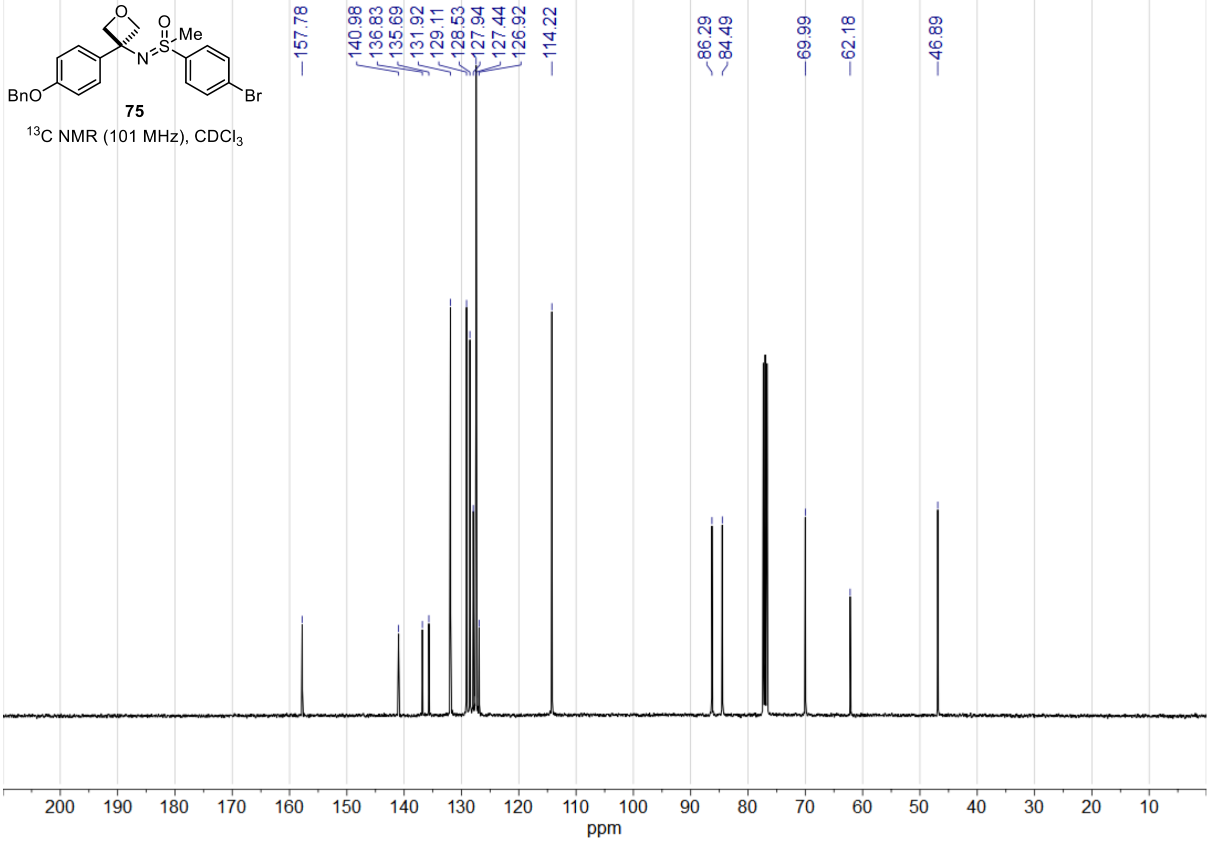
157.78
140.98 136.83 135.69 131.92 129.11 128.53 127.94 127.44 126.92
114.22
86.29 84.49
69.99
62.18
46.89
BnO
O
Me
N
S
Br
75
^{13}C NMR (101 MHz), CDCl$_3$
200 190 180 170 160 150 140 130 120 110 100 90 80 70 60 50 40 30 20 10
ppm

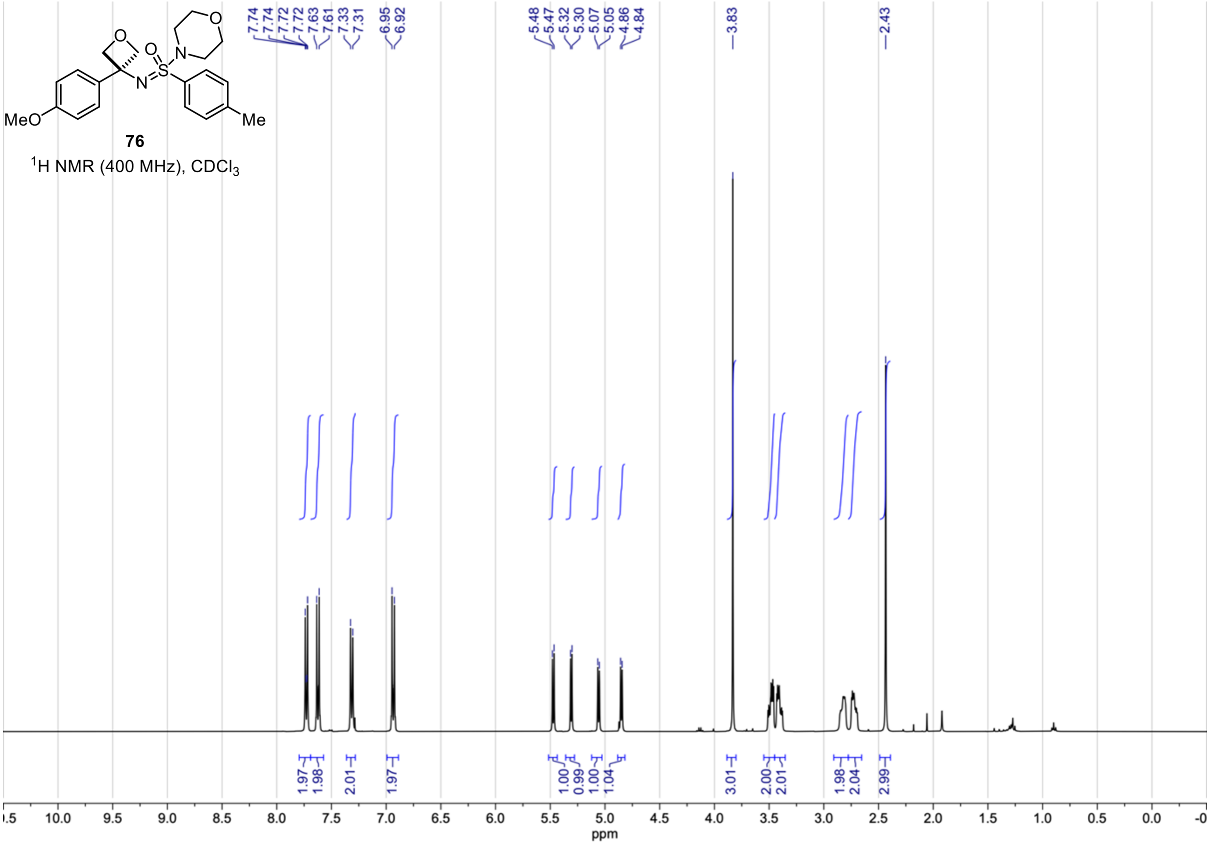
MeO
Me
O
N
S
N
O
76
^{1}H NMR (400 MHz), $CDCl_3$
7.74
7.74
7.72
7.72
7.63
7.61
7.33
7.31
6.95
6.92
5.48
5.47
5.32
5.30
5.07
5.05
4.86
4.84
3.83
2.43
1.97
1.98
2.01
1.97
1.00
0.99
1.00
1.04
3.01
2.00
2.01
1.98
2.04
2.99
ppm

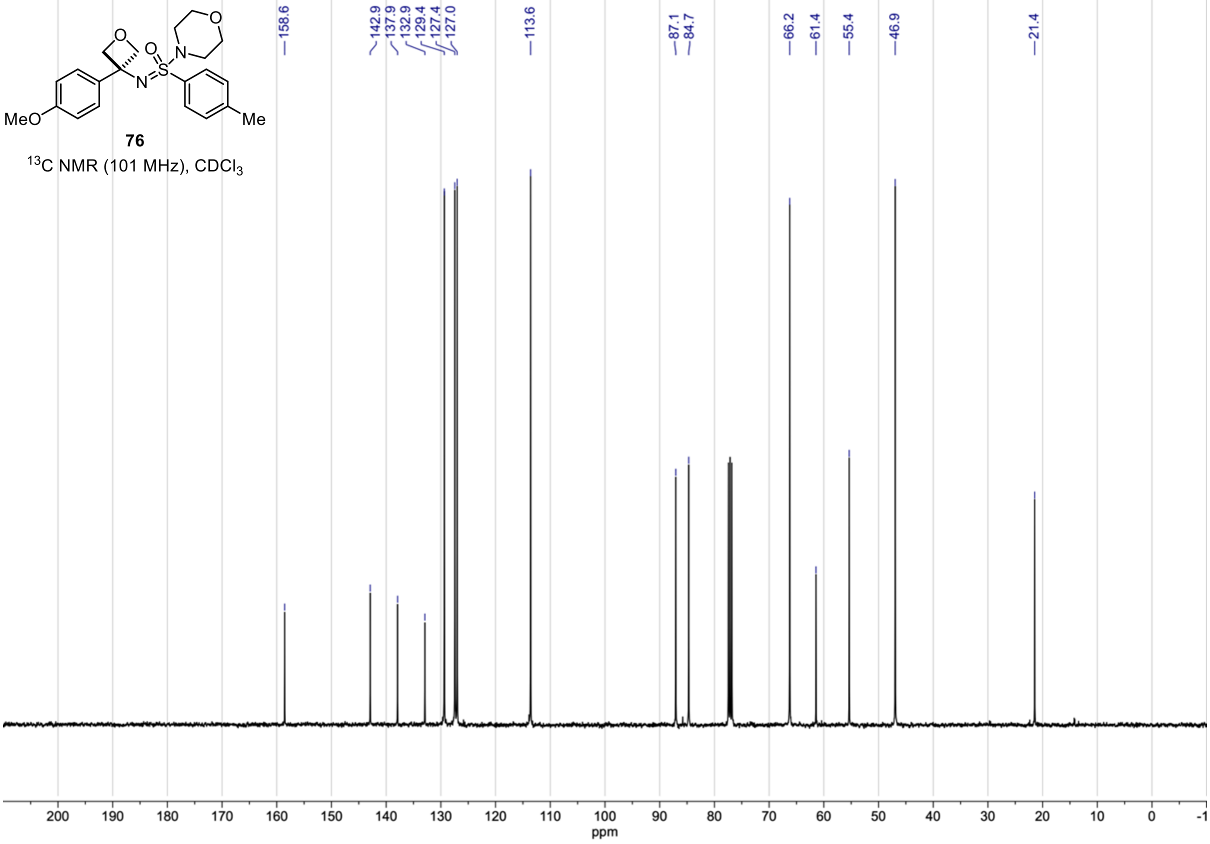
MeO
Me
76
^{13}C NMR (101 MHz), $CDCl_3$
158.6
142.9
137.9
132.9
129.4
127.4
127.0
113.6
87.1
84.7
66.2
61.4
55.4
46.9
21.4
ppm

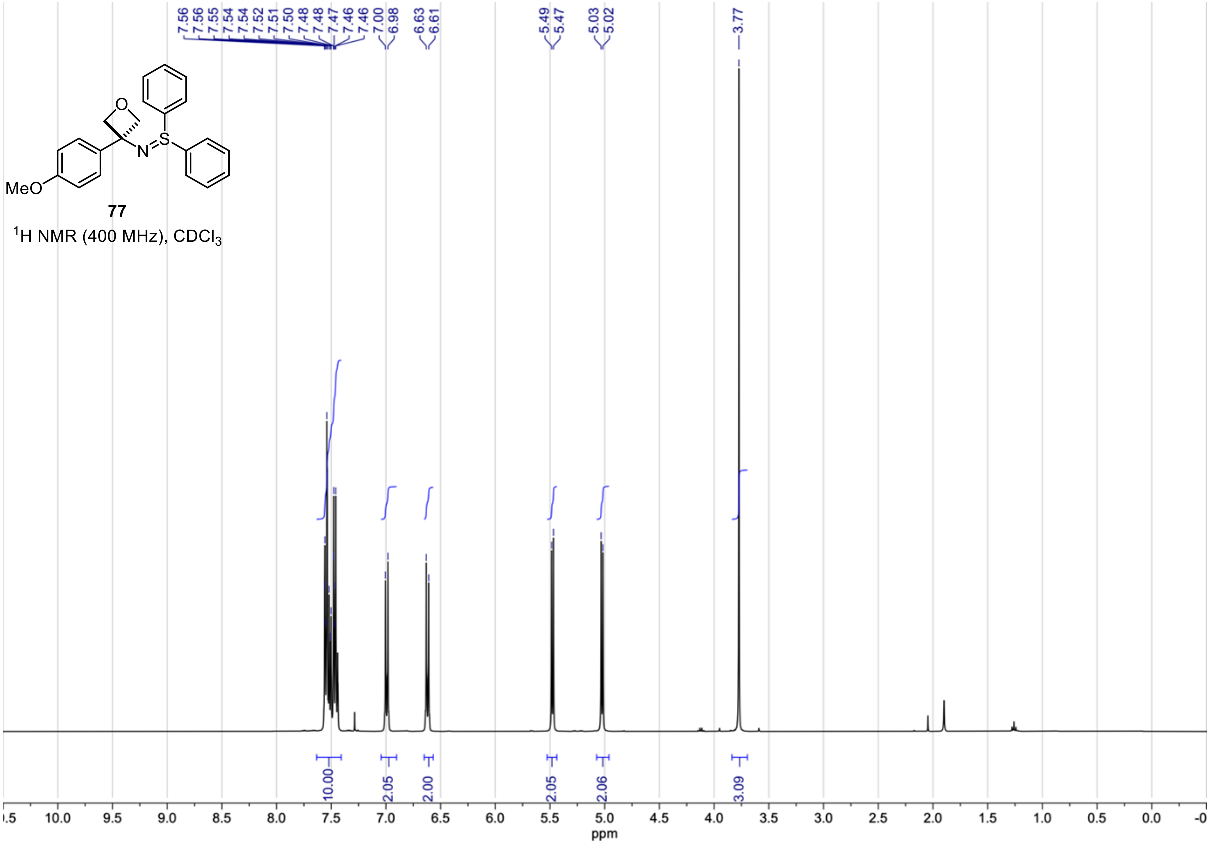
MeO
77
^{1}H NMR (400 MHz), CDCl$_3$
7.56 7.56 7.55 7.54 7.54 7.52 7.51 7.50 7.48 7.48 7.47 7.46 7.46 7.00 6.98 6.63 6.61
5.49 5.47 5.03 5.02 3.77
10.00 2.05 2.00 2.05 2.06 3.09
ppm

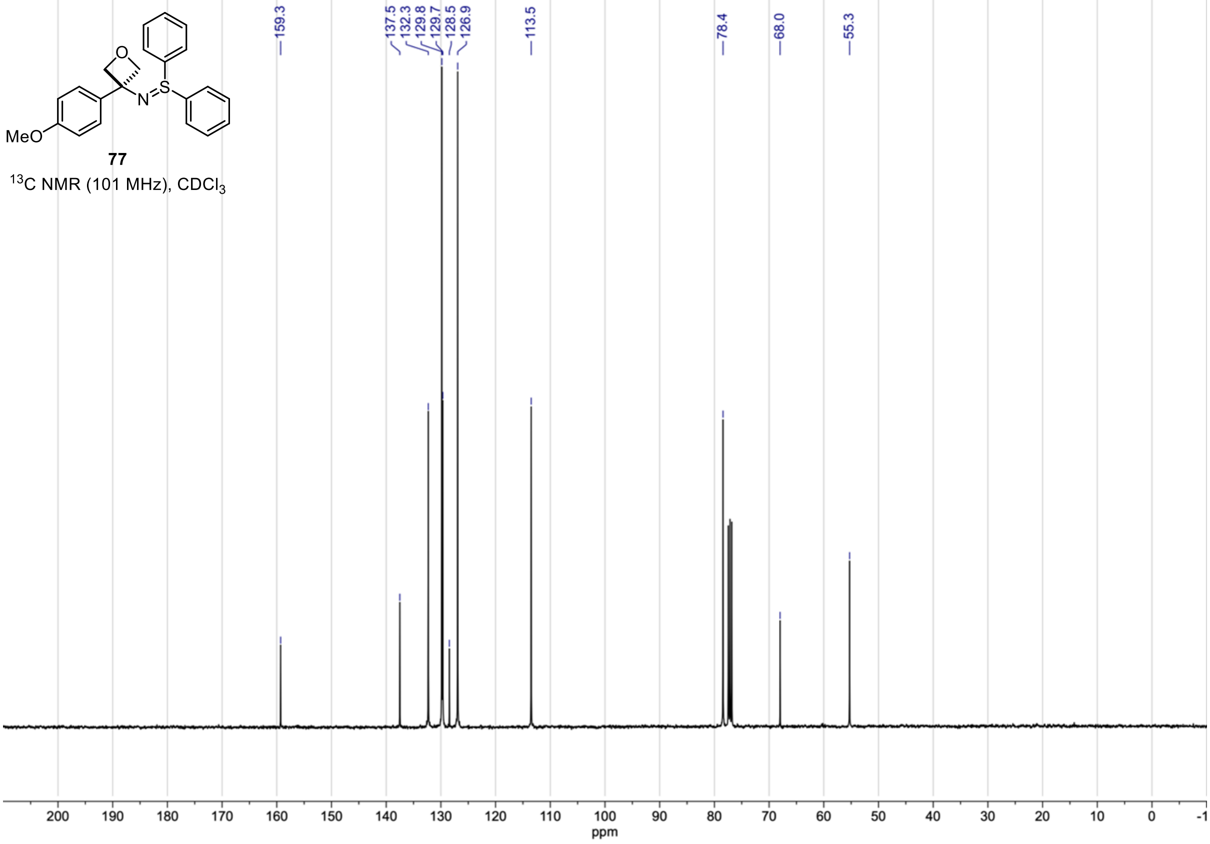
MeO
77
^{13}C NMR (101 MHz), CDCl$_3$
159.3 137.5 132.3 129.8 129.7 128.5 126.9 113.5 78.4 68.0 55.3
ppm

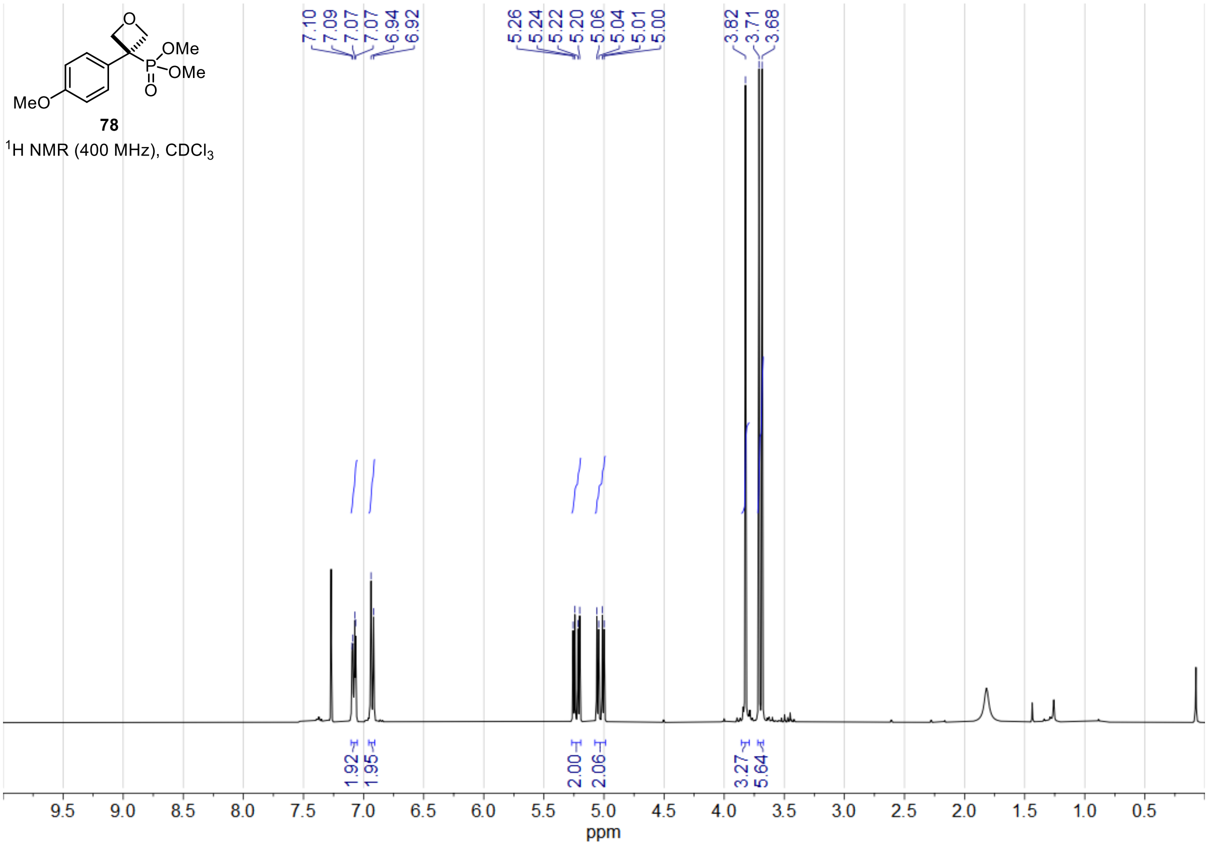

OMe
OMe
P
O
O
MeO
78
1H NMR (400 MHz), CDCl3
7.10
7.09
7.07
7.07
6.94
6.92
5.26
5.24
5.22
5.20
5.06
5.04
5.01
5.00
3.82
3.71
3.68
1.92
1.95
2.00
2.06
3.27
5.64
9.5 9.0 8.5 8.0 7.5 7.0 6.5 6.0 5.5 5.0 4.5 4.0 3.5 3.0 2.5 2.0 1.5 1.0 0.5
ppm

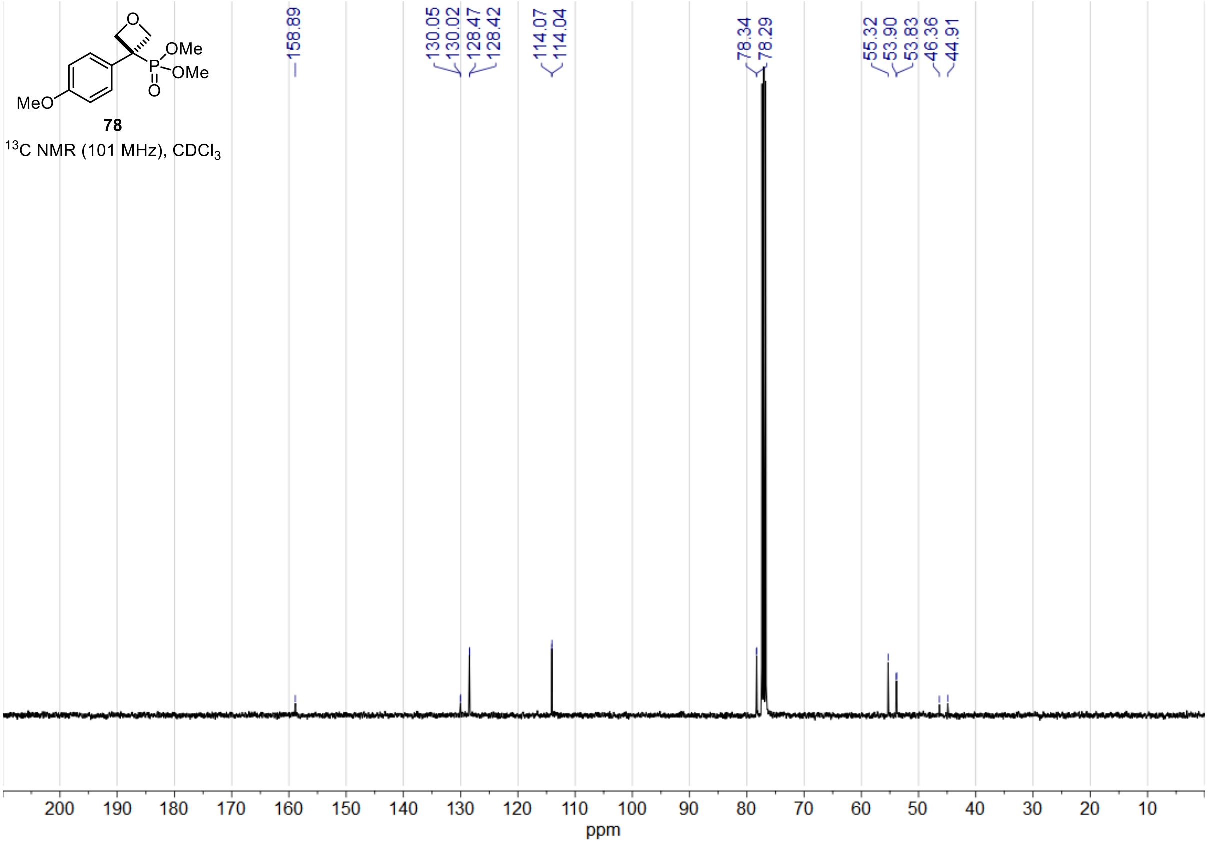

OMe
OMe
P
O
O
MeO
78
13C NMR (101 MHz), CDCl3
158.89
130.05
130.02
128.47
128.42
114.07
114.04
78.34
78.29
55.32
53.90
53.83
46.36
44.91
200 190 180 170 160 150 140 130 120 110 100 90 80 70 60 50 40 30 20 10
ppm

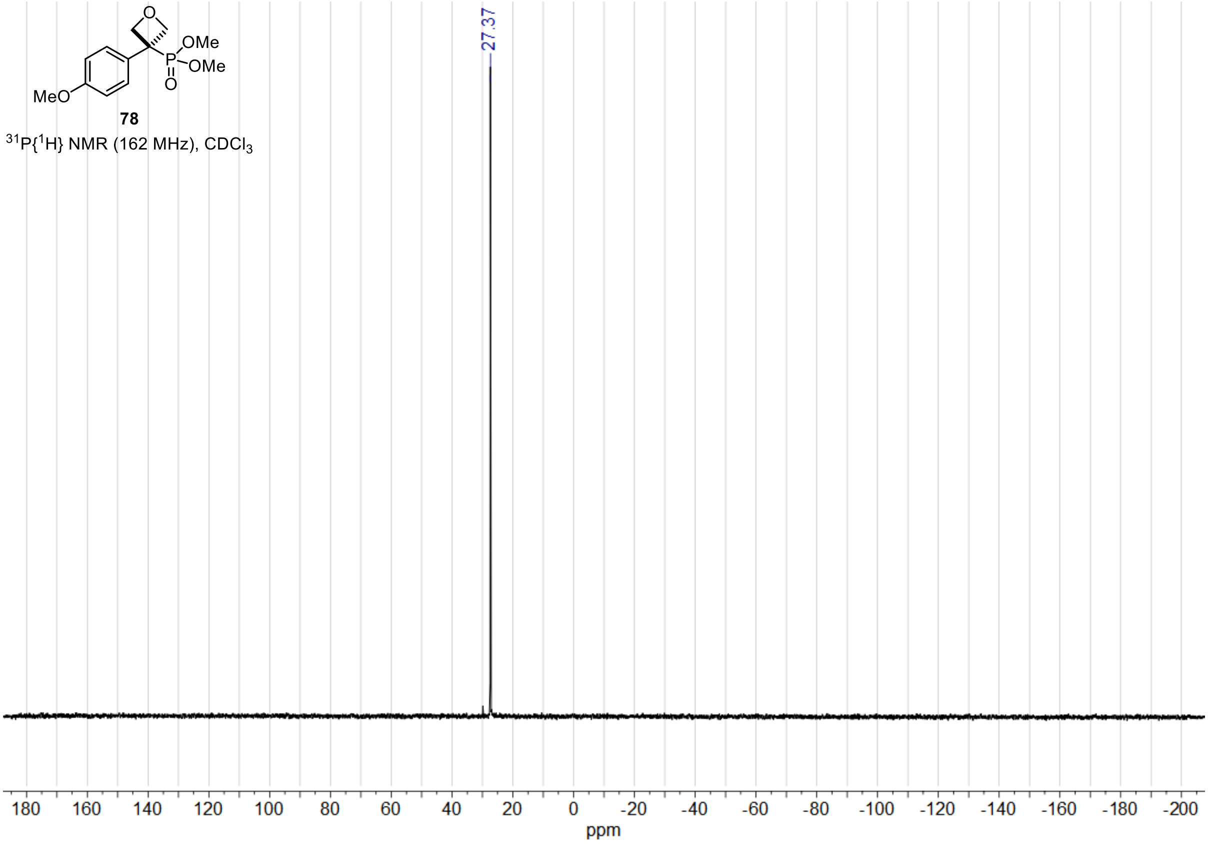

78

$^{31}P\{^{1}H\}$ NMR (162 MHz), $CDCl_3$

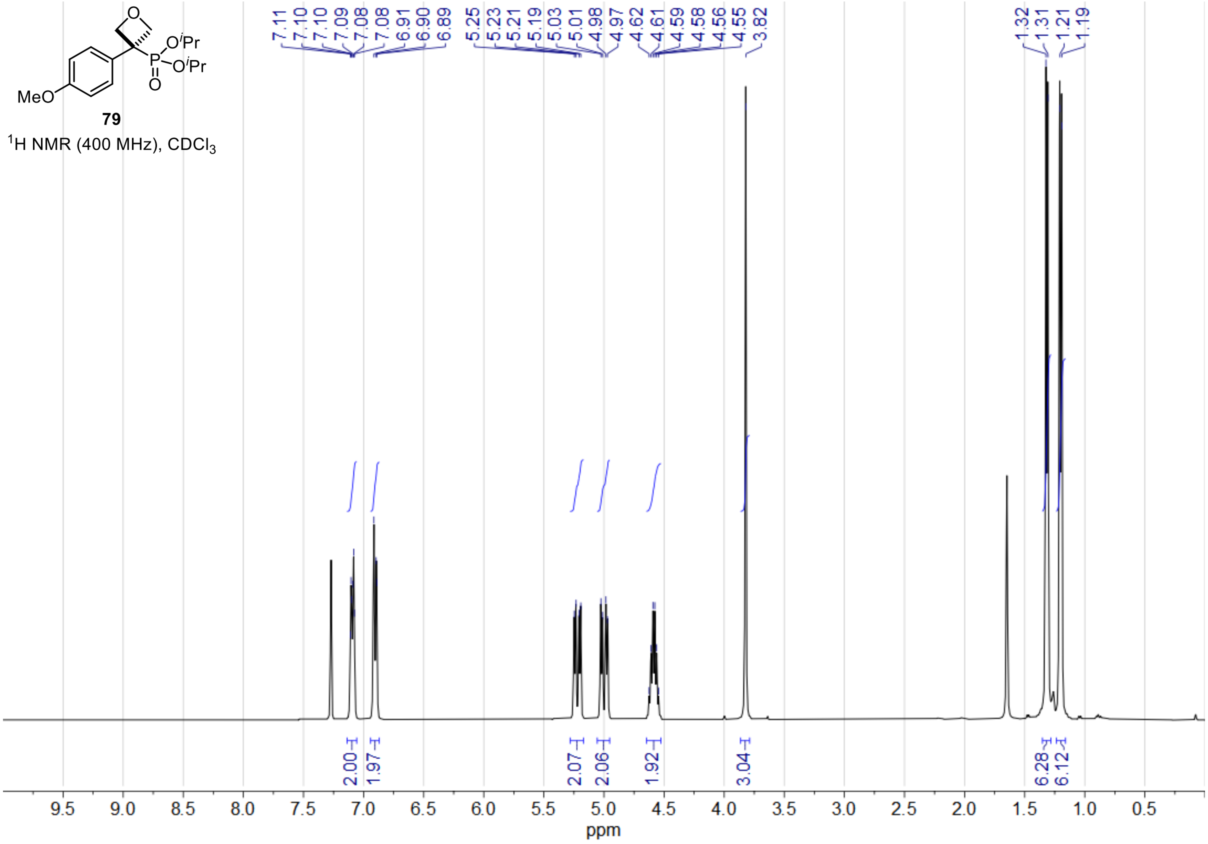
MeO
O
O^{i}Pr
O^{i}Pr
79
^{1}H NMR (400 MHz), CDCl$_3$
7.11 7.10 7.10 7.09 7.08 7.08 6.91 6.90 6.89 5.25 5.23 5.21 5.19 5.03 5.01 4.98 4.97 4.62 4.61 4.59 4.58 4.56 4.55 3.82
1.32 1.31 1.21 1.19
2.00 1.97 2.07 2.06 1.92 3.04 6.28 6.12
9.5 9.0 8.5 8.0 7.5 7.0 6.5 6.0 5.5 5.0 4.5 4.0 3.5 3.0 2.5 2.0 1.5 1.0 0.5
ppm

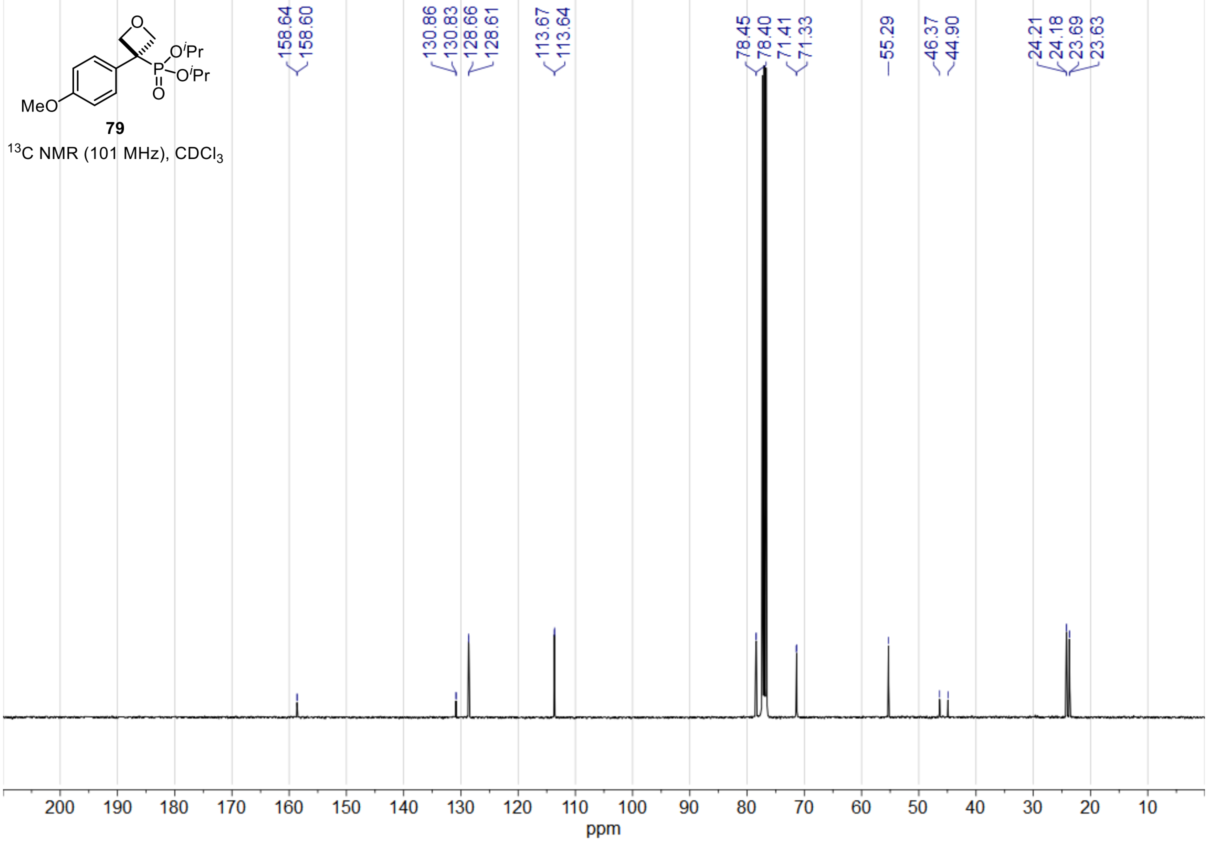
MeO
O
O^{i}Pr
O^{i}Pr
79
^{13}C NMR (101 MHz), CDCl$_3$
158.64 158.60 130.86 130.83 128.66 128.61 113.67 113.64
78.45 78.40 71.41 71.33 55.29 46.37 44.90 24.21 24.18 23.69 23.63
200 190 180 170 160 150 140 130 120 110 100 90 80 70 60 50 40 30 20 10
ppm

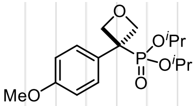

79

$^{31}P\{^1H\}$ NMR (162 MHz), $CDCl_3$

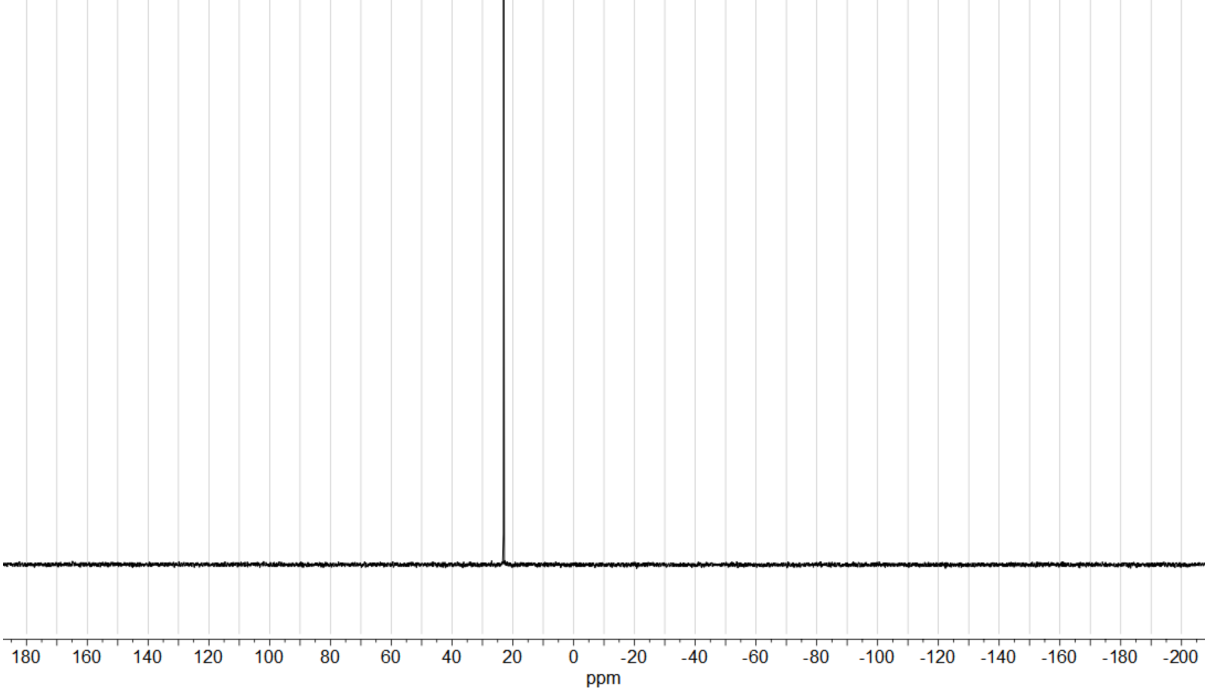

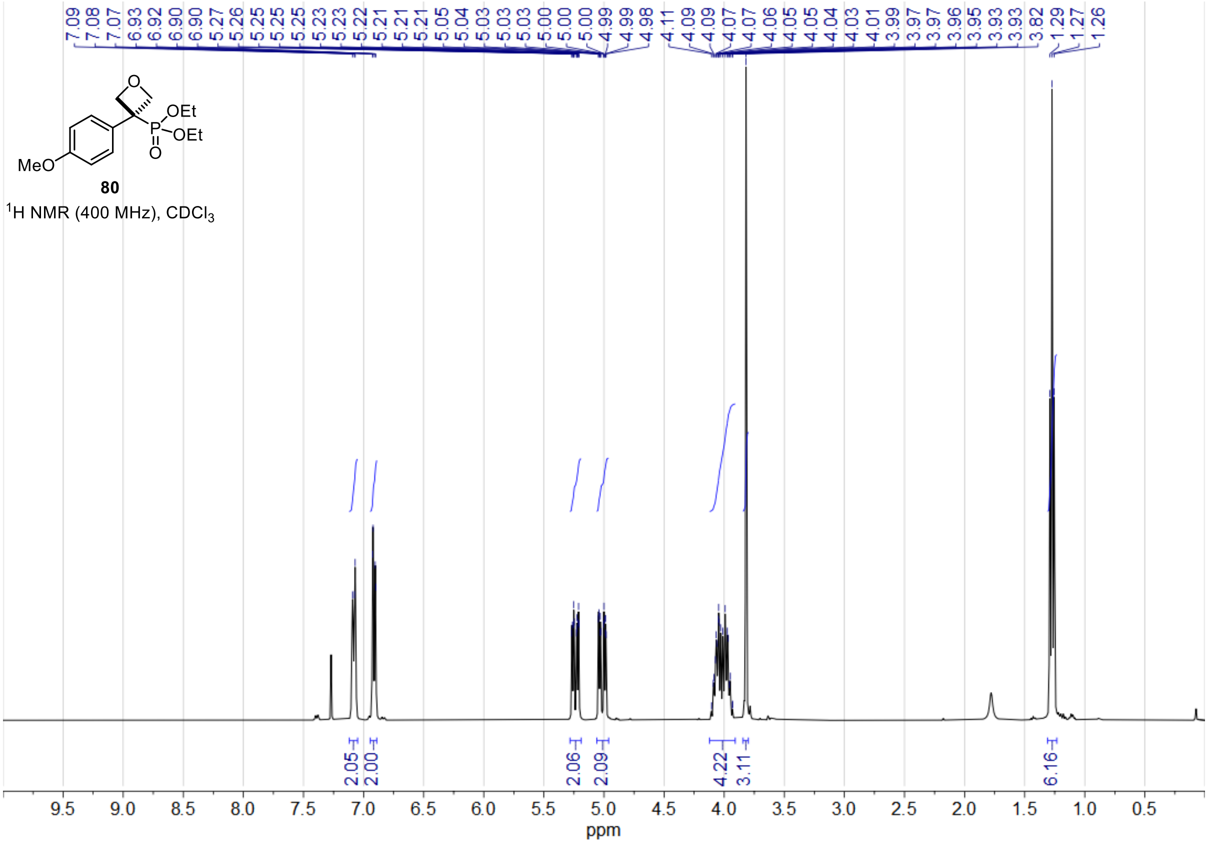

80

^{1}H NMR (400 MHz), $CDCl_3$

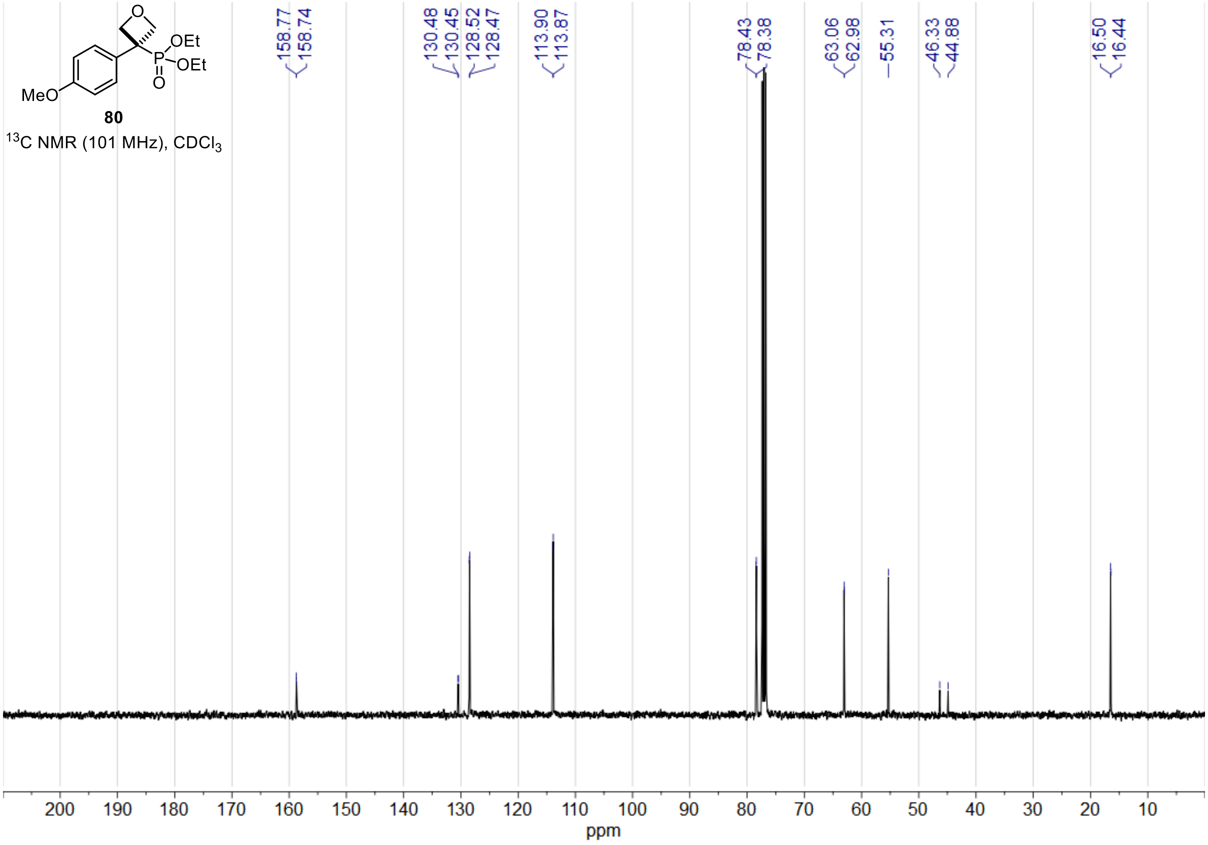

80

^{13}C NMR (101 MHz), $CDCl_3$

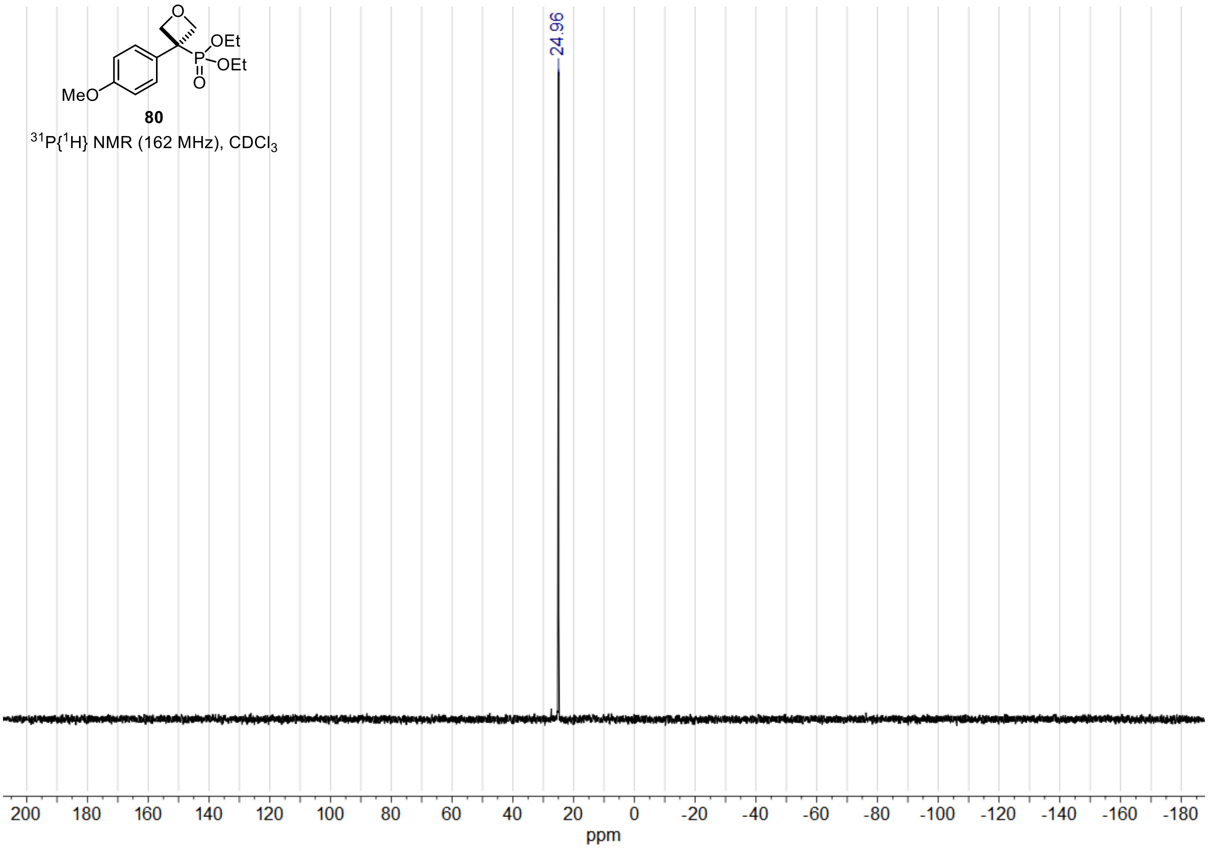
OEt
OEt
MeO
O
P
80
$^{31}P\{^{1}H\}$ NMR (162 MHz), $CDCl_3$
24.96
200 180 160 140 120 100 80 60 40 20 0 -20 -40 -60 -80 -100 -120 -140 -160 -180
ppm

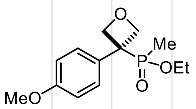

81

^{1}H NMR (400 MHz), $CDCl_3$

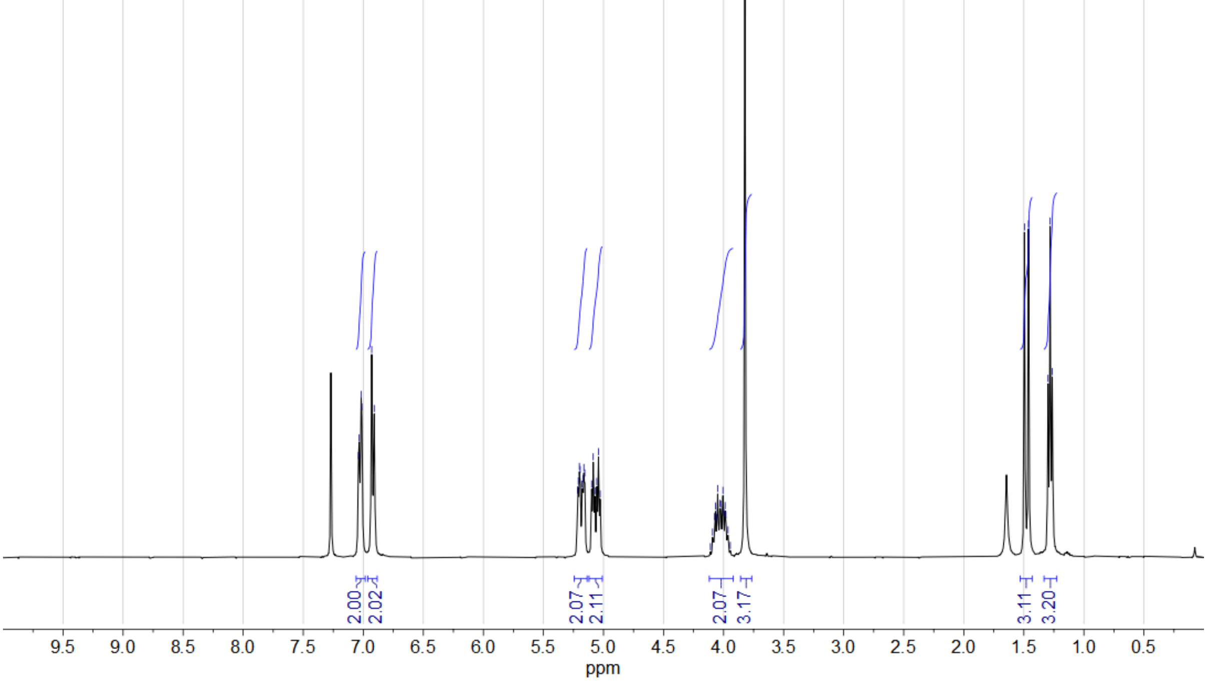

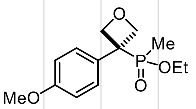

81

^{13}C NMR (126 MHz), $CDCl_3$

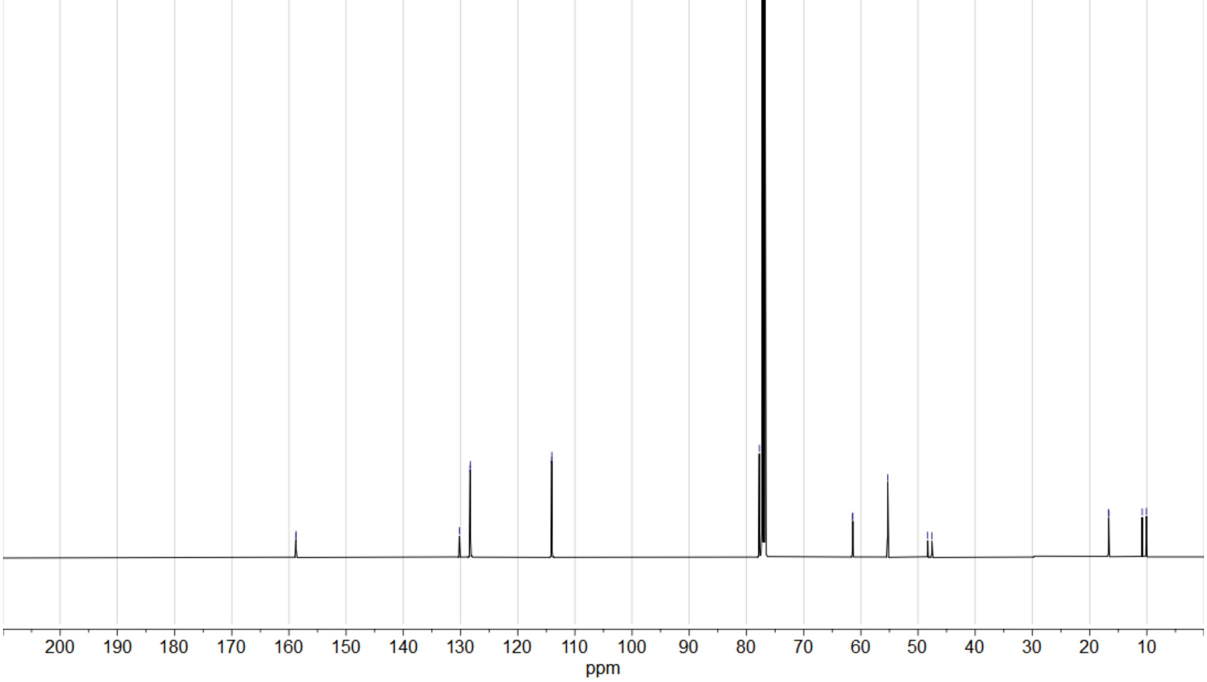

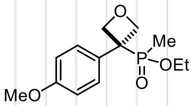

81

$^{31}P\{^{1}H\}$ NMR (162 MHz), $CDCl_3$

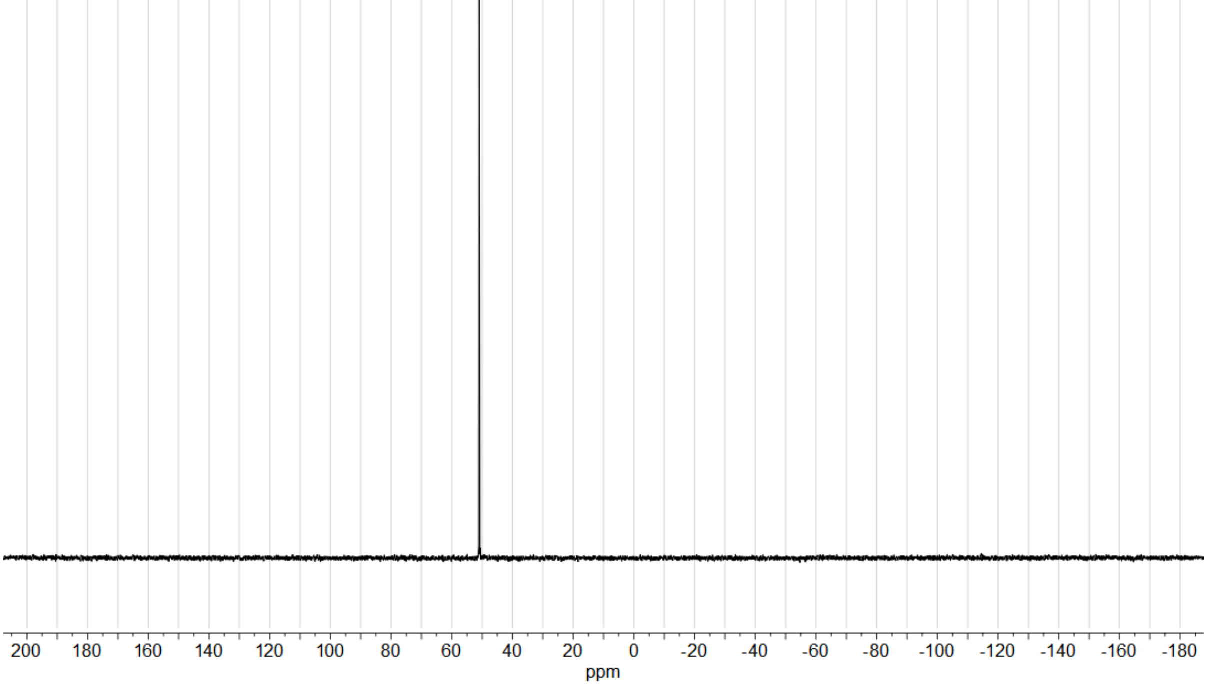

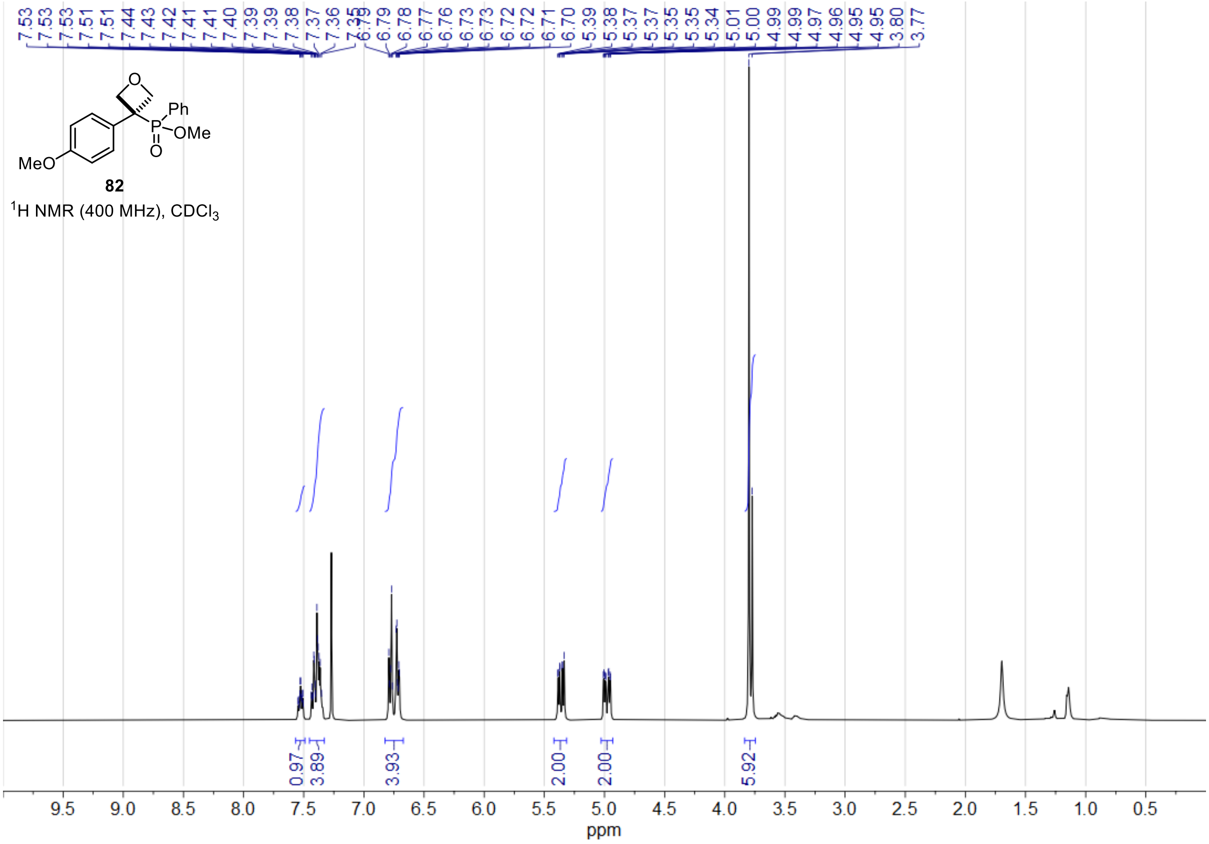
7.53
7.53
7.53
7.51
7.51
7.44
7.43
7.42
7.41
7.41
7.40
7.39
7.39
7.38
7.37
7.36
7.35
6.79
6.78
6.77
6.76
6.73
6.73
6.72
6.72
6.71
6.70
5.39
5.38
5.37
5.37
5.35
5.35
5.34
5.01
5.00
4.99
4.99
4.97
4.96
4.95
4.95
3.80
3.77
Ph
P
OMe
O
MeO
82
^{1}H NMR (400 MHz), CDCl$_3$
0.97
3.89
3.93
2.00
2.00
5.92
9.5
9.0
8.5
8.0
7.5
7.0
6.5
6.0
5.5
5.0
4.5
4.0
3.5
3.0
2.5
2.0
1.5
1.0
0.5
ppm

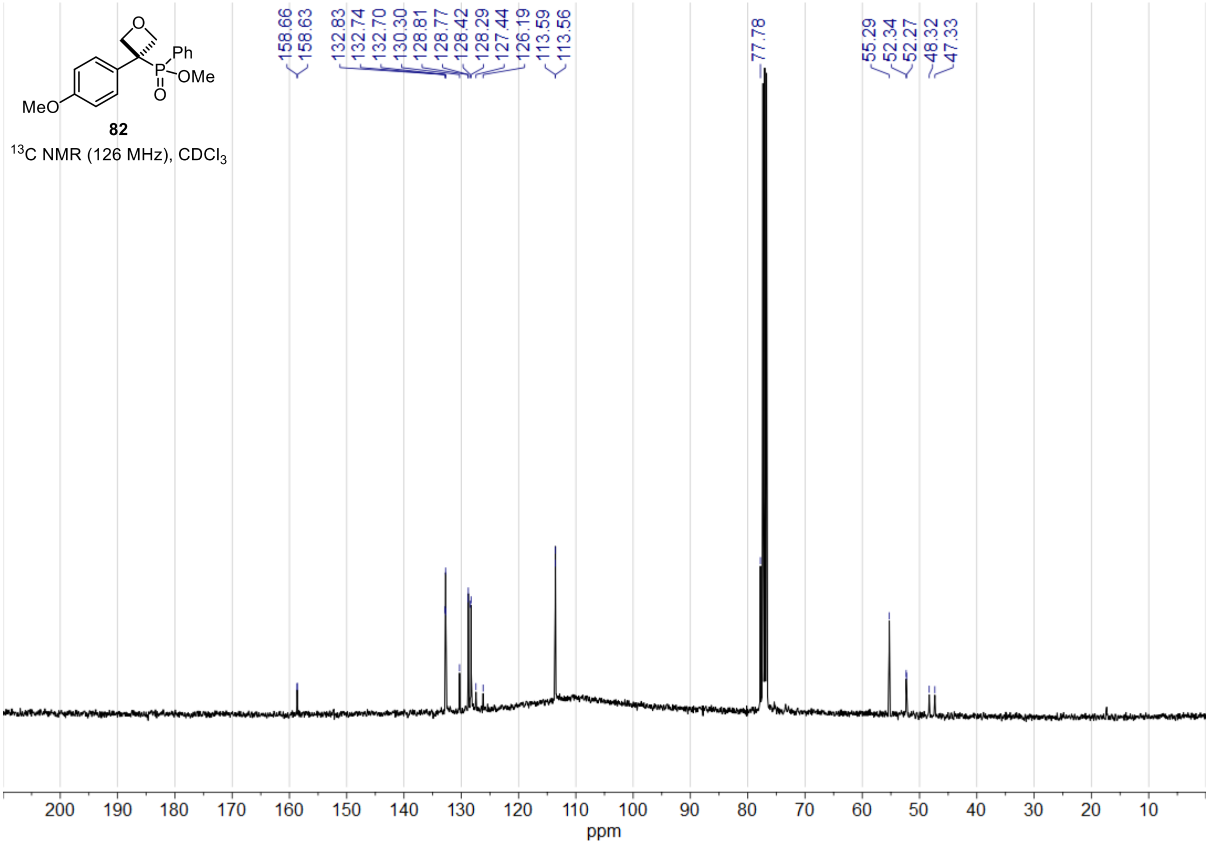
158.66
158.63
132.83
132.74
132.70
130.30
128.81
128.77
128.42
128.29
127.44
126.19
113.59
113.56
77.78
55.29
52.34
52.27
48.32
47.33
Ph
P
OMe
O
MeO
82
^{13}C NMR (126 MHz), CDCl$_3$
200
190
180
170
160
150
140
130
120
110
100
90
80
70
60
50
40
30
20
10
ppm

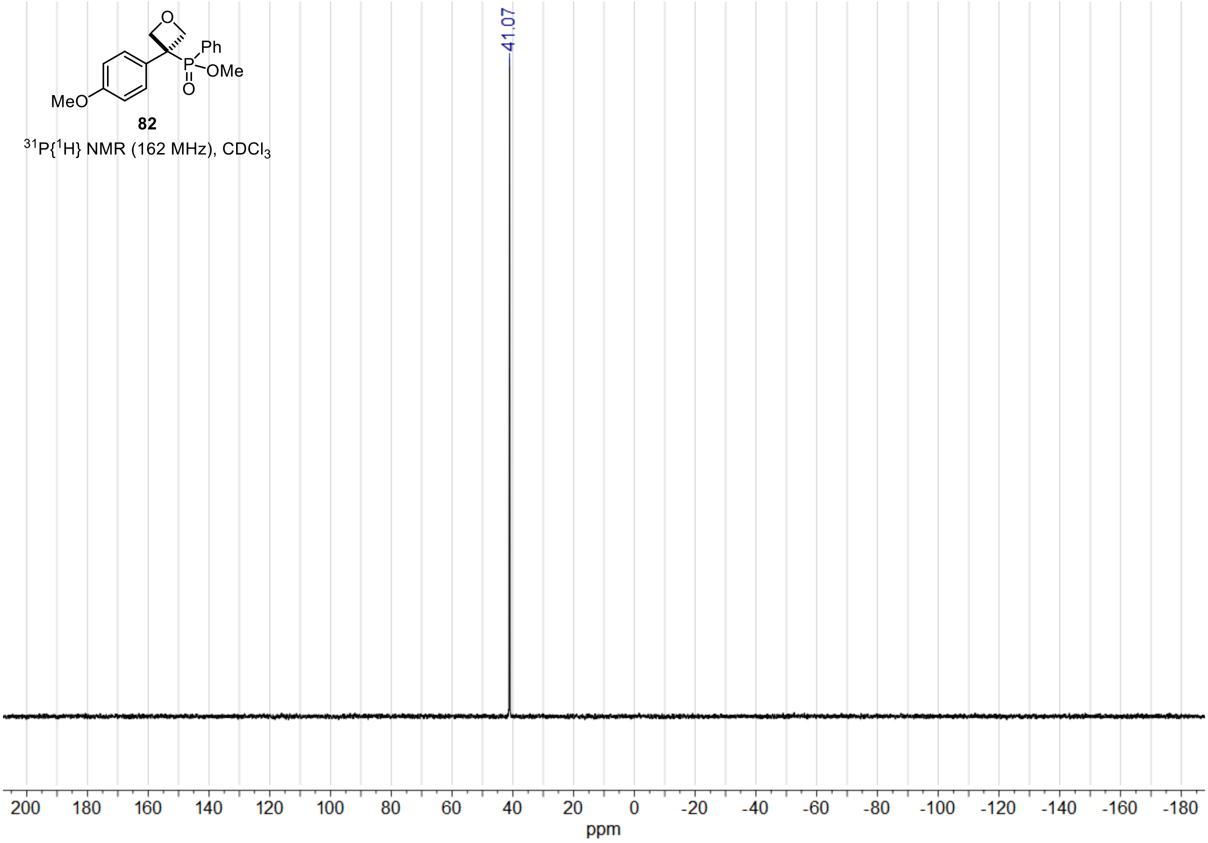

82

$^{31}P\{^1H\}$ NMR (162 MHz), $CDCl_3$

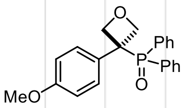

83

^{1}H NMR (400 MHz), $CDCl_3$

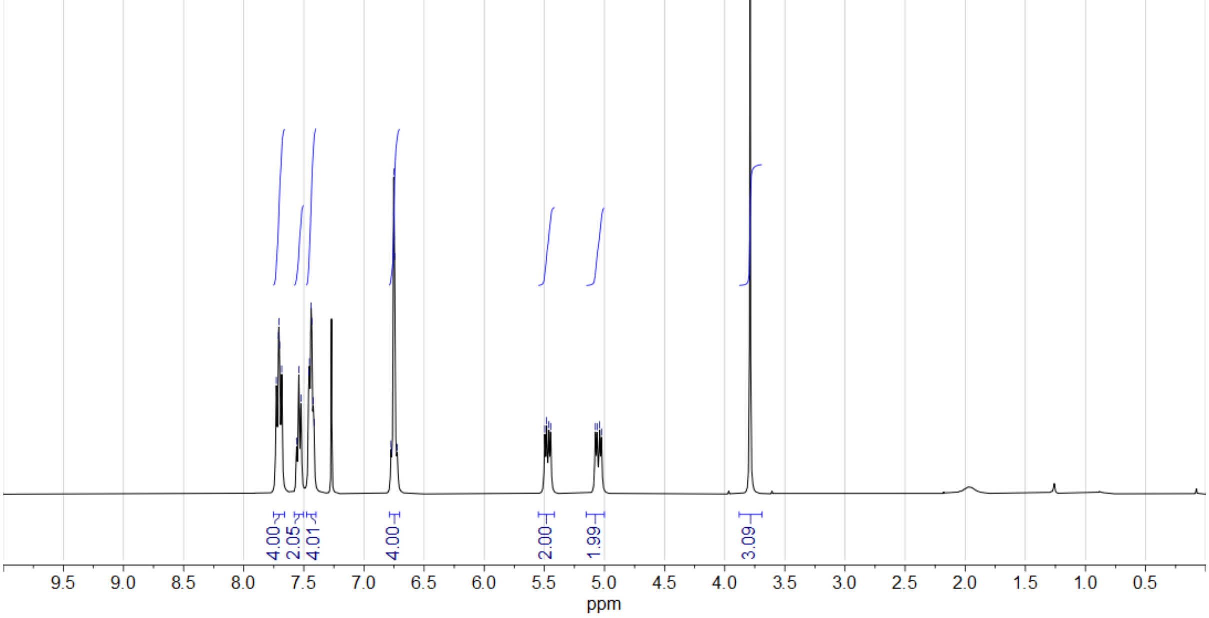

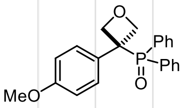

83

^{13}C NMR (126 MHz), $CDCl_3$

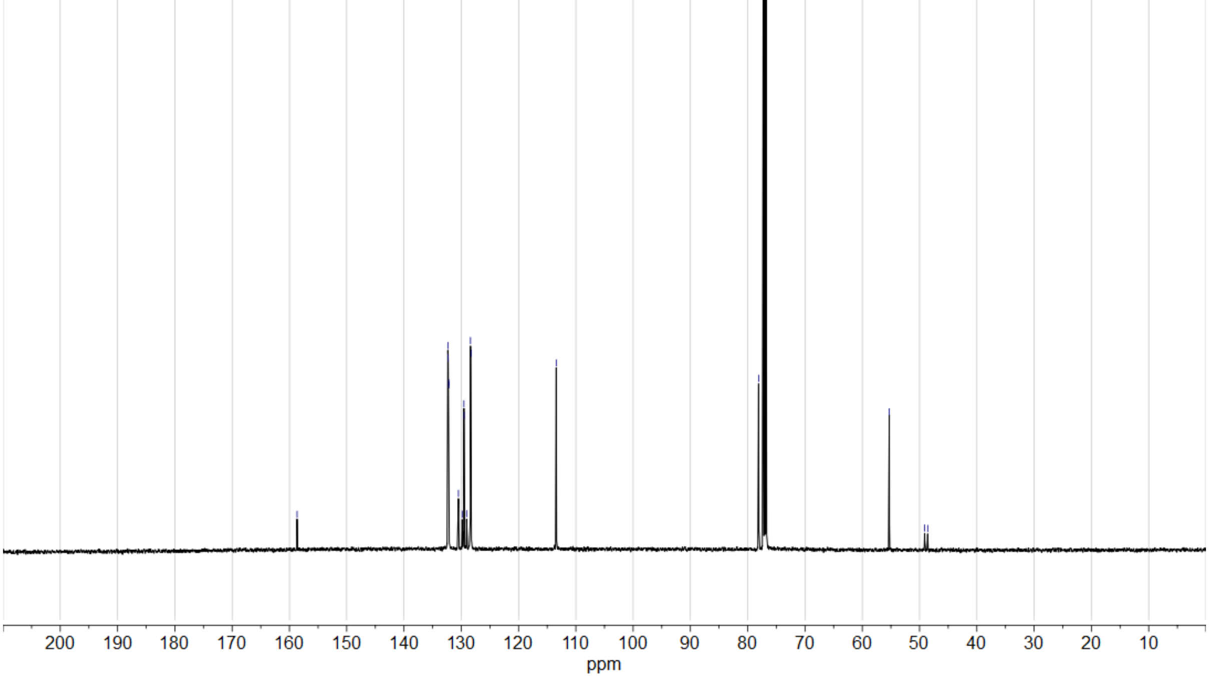

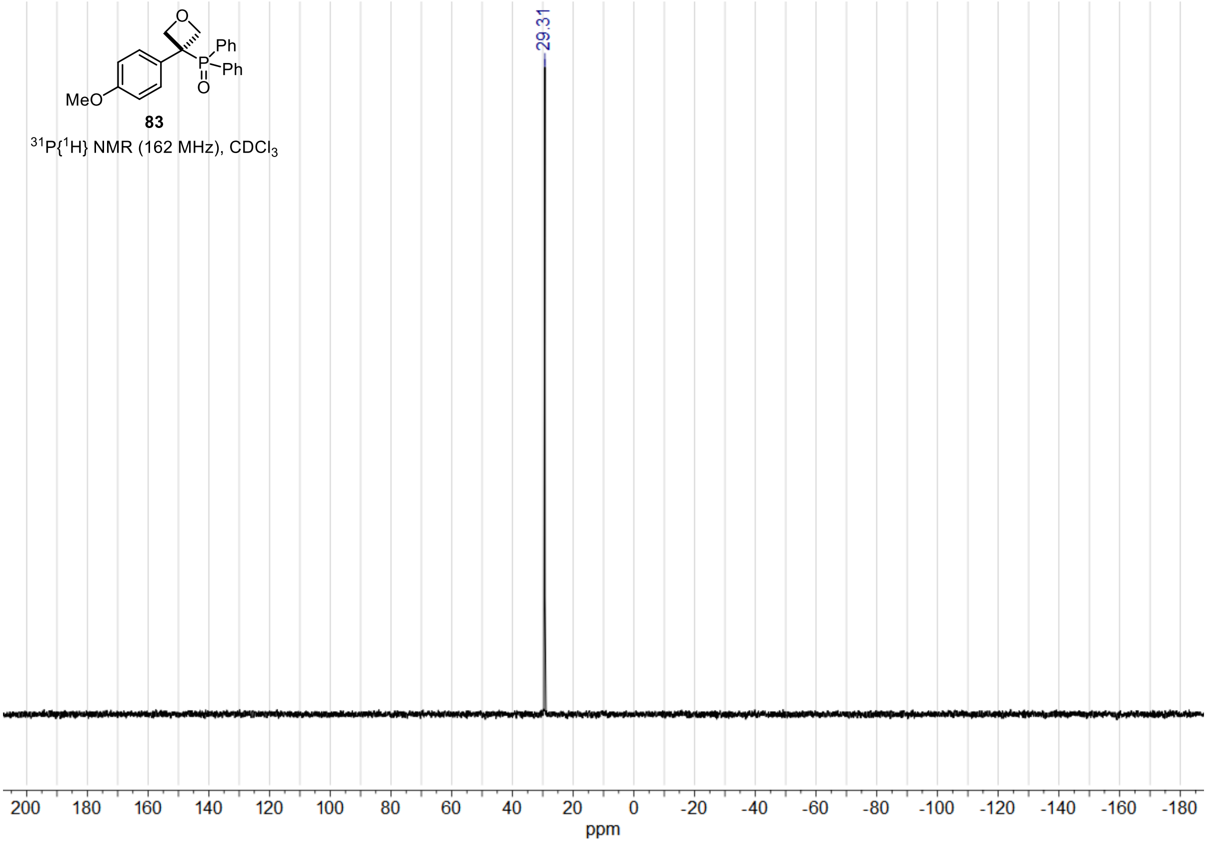
MeO
Ph
P
Ph
O
O
83
^{31}P{^{1}H} NMR (162 MHz), $CDCl_3$
29.31
200 180 160 140 120 100 80 60 40 20 0 -20 -40 -60 -80 -100 -120 -140 -160 -180
ppm

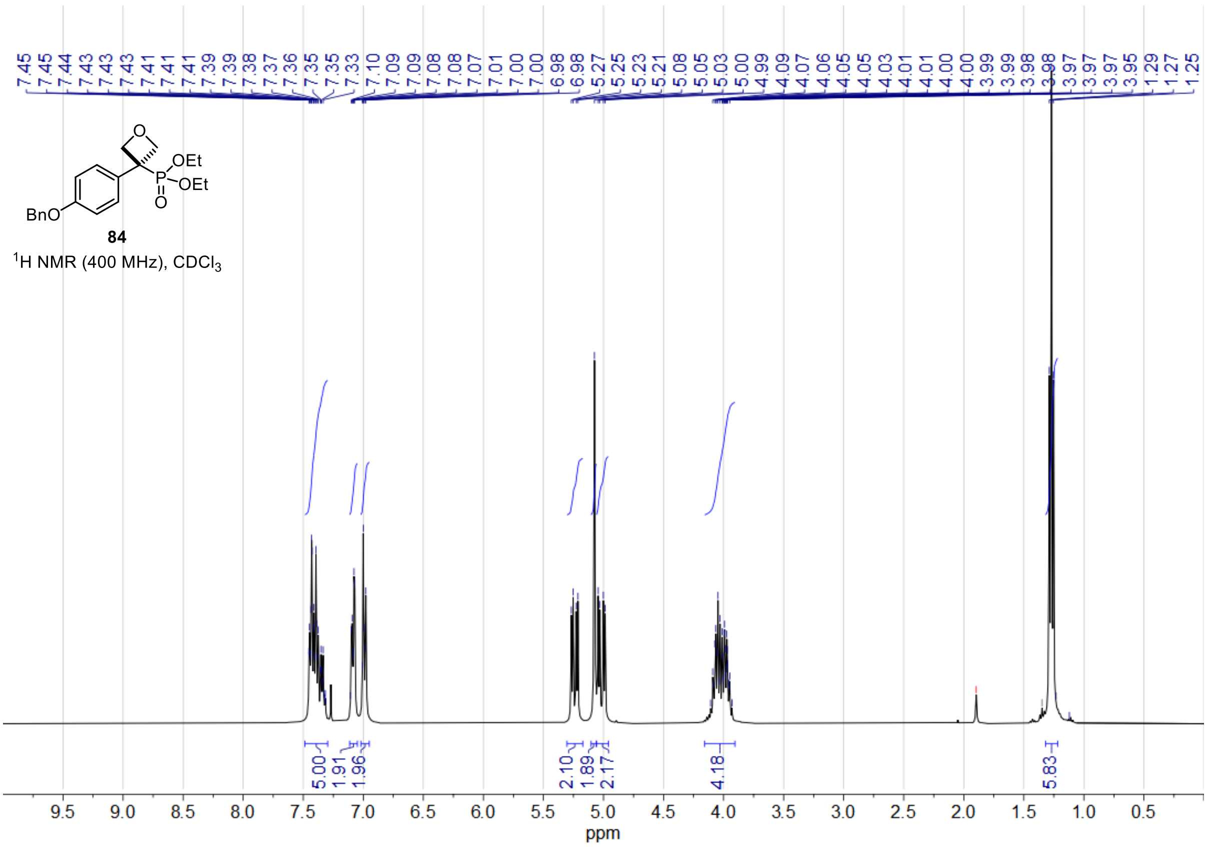
7.45 7.45 7.44 7.43 7.43 7.43 7.41 7.41 7.41 7.39 7.39 7.38 7.37 7.36 7.35 7.35 7.33 7.10 7.09 7.09 7.08 7.08 7.07 7.01 7.00 7.00 6.98 6.98 5.27 5.25 5.23 5.21 5.08 5.05 5.03 5.00 4.99 4.09 4.07 4.06 4.05 4.05 4.03 4.01 4.01 4.00 4.00 3.99 3.99 3.98 3.98 3.97 3.97 3.97 3.95 1.29 1.27 1.25
OEt
OEt
P
O
O
BnO
84
^{1}H NMR (400 MHz), CDCl$_3$
5.00 1.91 1.96 2.10 1.89 2.17 4.18 5.83
9.5 9.0 8.5 8.0 7.5 7.0 6.5 6.0 5.5 5.0 4.5 4.0 3.5 3.0 2.5 2.0 1.5 1.0 0.5
ppm

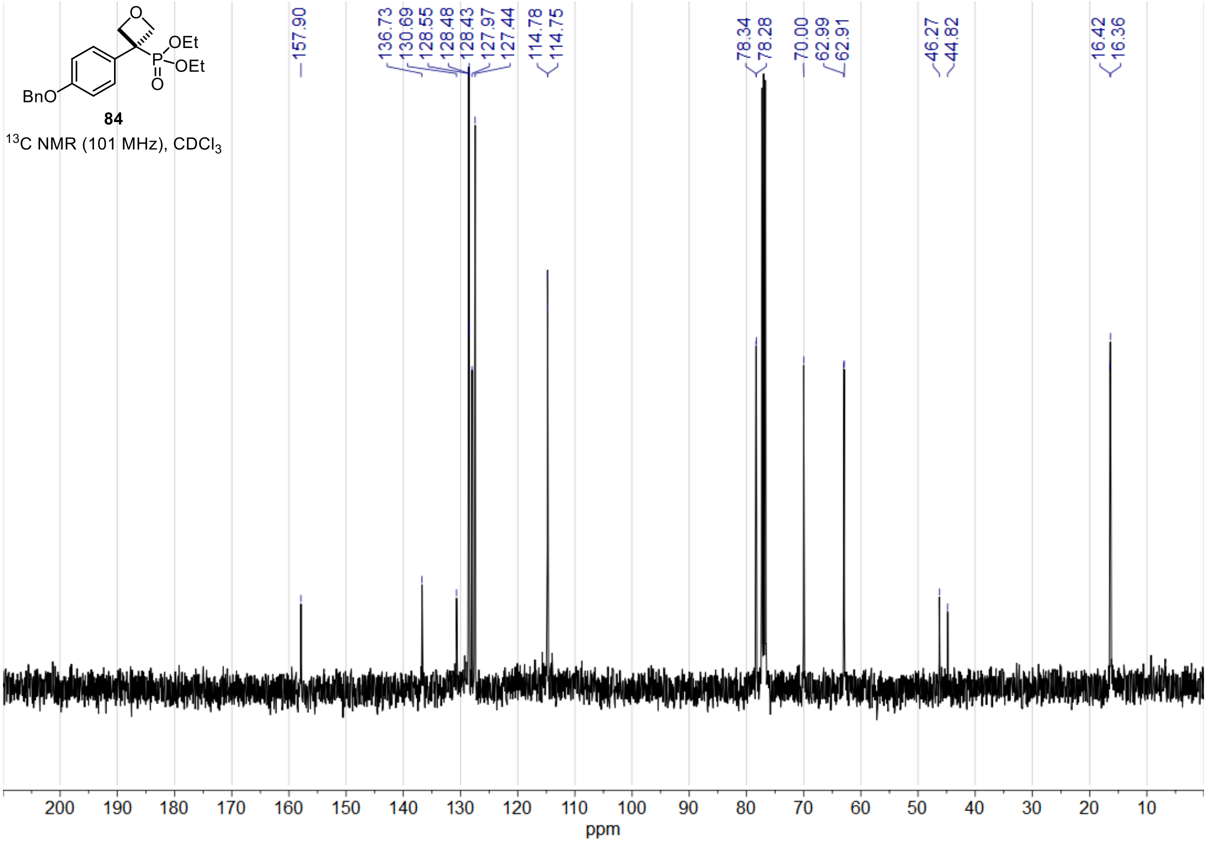
157.90
136.73 130.69 128.55 128.48 128.43 127.97 127.44 114.78 114.75
78.34 78.28 70.00 62.99 62.91 46.27 44.82 16.42 16.36
OEt
OEt
P
O
O
BnO
84
^{13}C NMR (101 MHz), CDCl$_3$
200 190 180 170 160 150 140 130 120 110 100 90 80 70 60 50 40 30 20 10
ppm

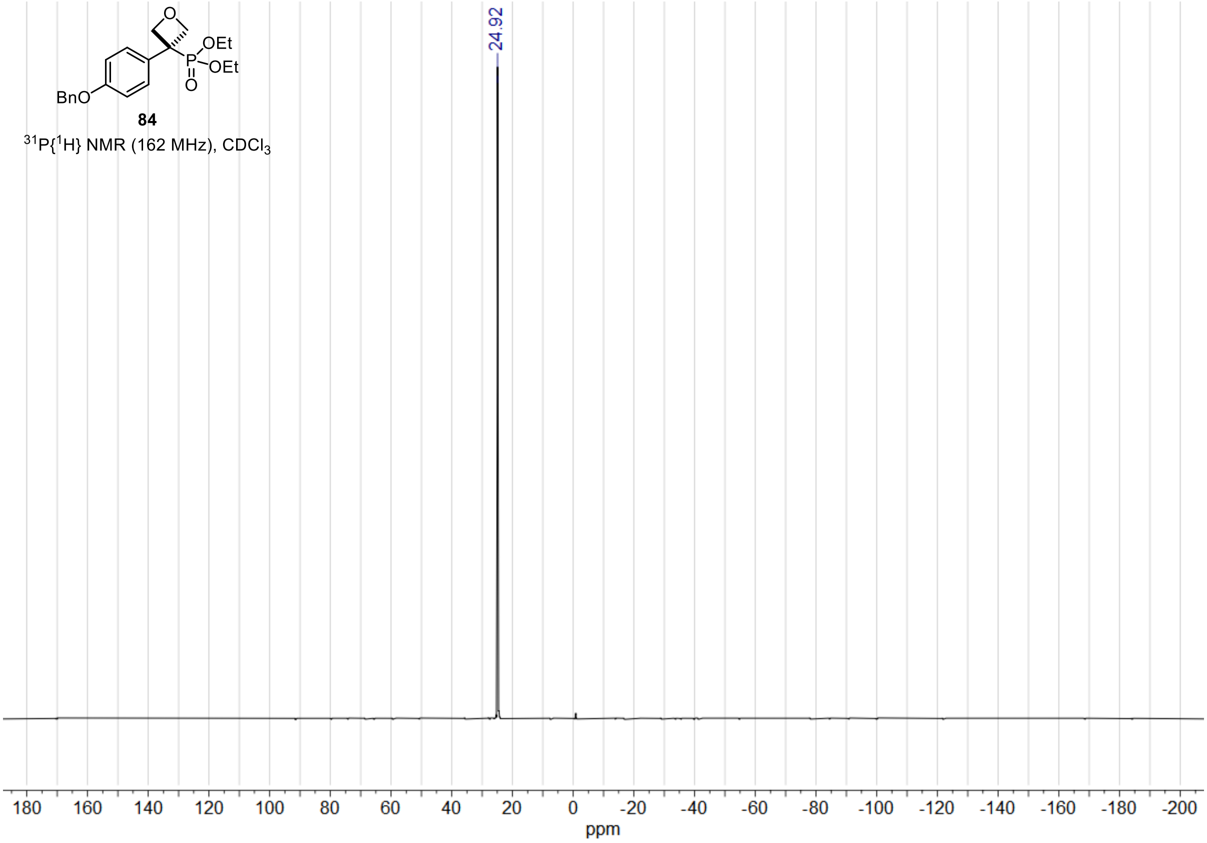

84

$^{31}P\{^{1}H\}$ NMR (162 MHz), $CDCl_3$

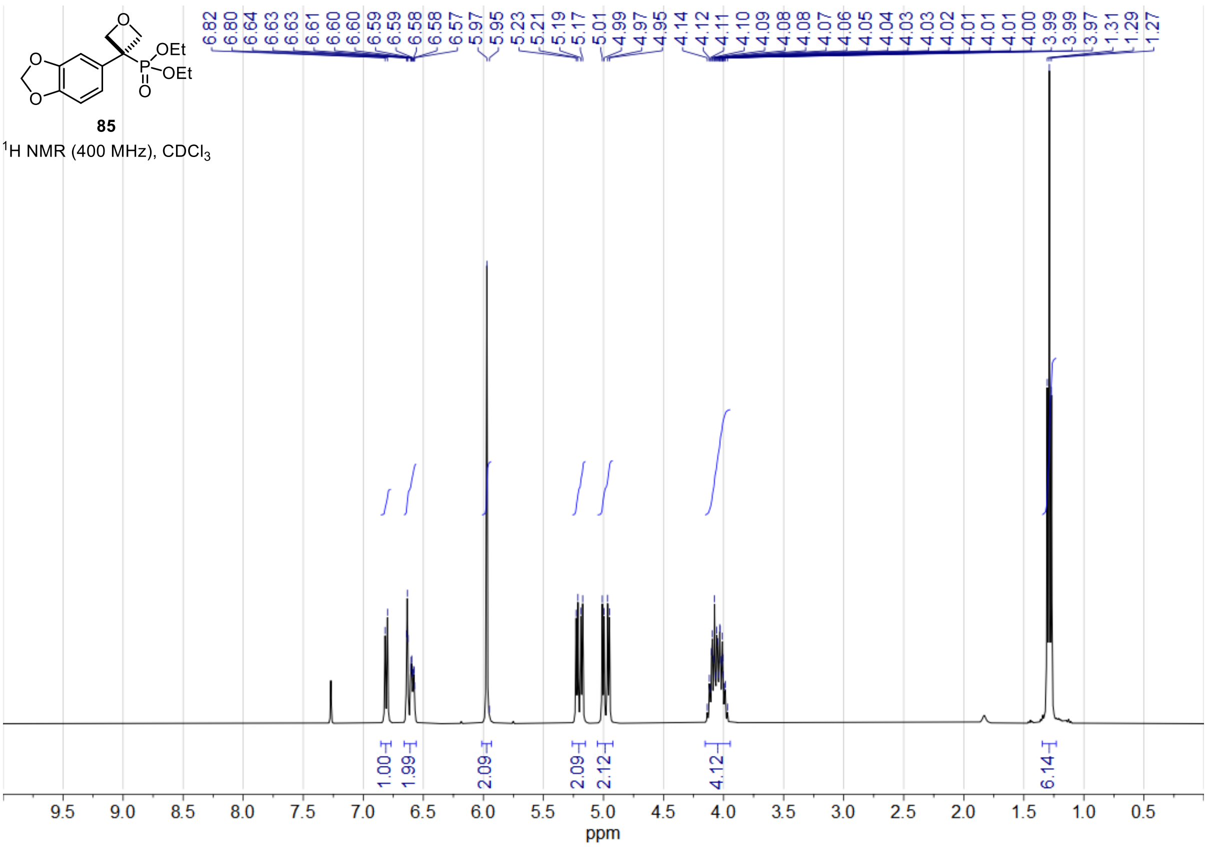
OEt
OEt
O
85
1H NMR (400 MHz), CDCl3
6.82 6.80 6.64 6.63 6.63 6.61 6.60 6.60 6.59 6.59 6.58 6.58 6.57 5.97 5.95 5.23 5.21 5.19 5.17 5.01 4.99 4.97 4.95 4.14 4.12 4.11 4.10 4.09 4.08 4.08 4.07 4.06 4.05 4.04 4.03 4.03 4.02 4.01 4.01 4.01 4.00 3.99 3.99 3.97 1.31 1.29 1.27
1.00 1.99 2.09 2.09 2.12 4.12 6.14
9.5 9.0 8.5 8.0 7.5 7.0 6.5 6.0 5.5 5.0 4.5 4.0 3.5 3.0 2.5 2.0 1.5 1.0 0.5
ppm

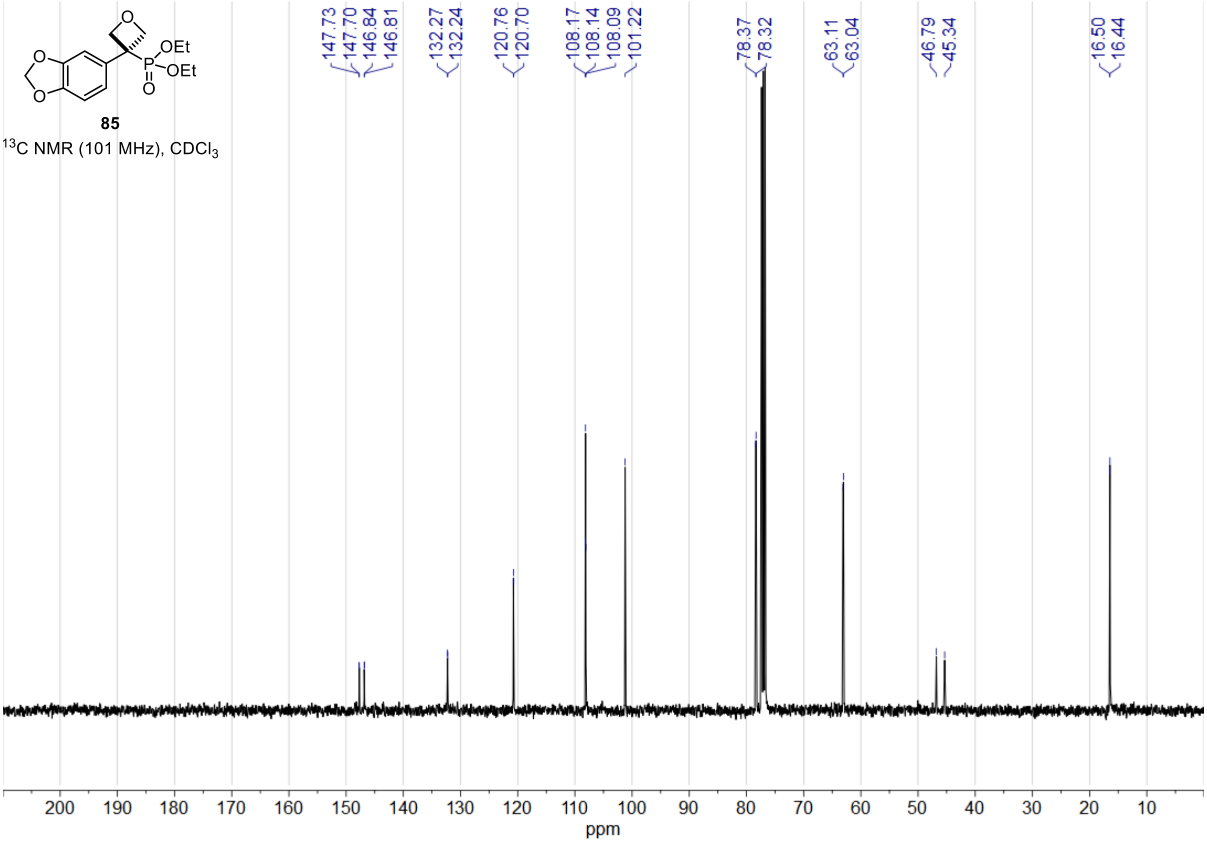
OEt
OEt
O
85
13C NMR (101 MHz), CDCl3
147.73 147.70 146.84 146.81 132.27 132.24 120.76 120.70 108.17 108.14 108.09 101.22 78.37 78.32 63.11 63.04 46.79 45.34 16.50 16.44
200 190 180 170 160 150 140 130 120 110 100 90 80 70 60 50 40 30 20 10
ppm

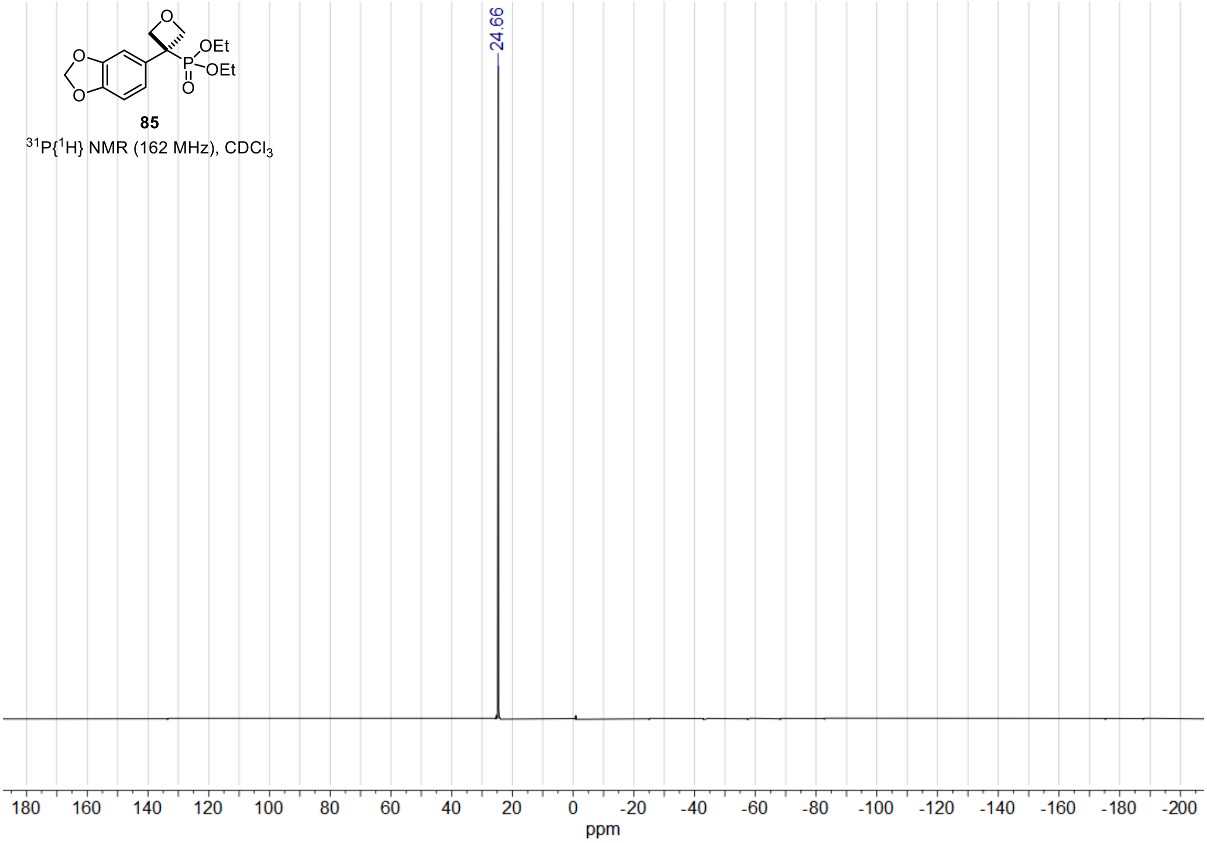
OEt
OEt
85
31P{1H} NMR (162 MHz), CDCl3
24.66
180
160
140
120
100
80
60
40
20
0
-20
-40
-60
-80
-100
-120
-140
-160
-180
-200
ppm

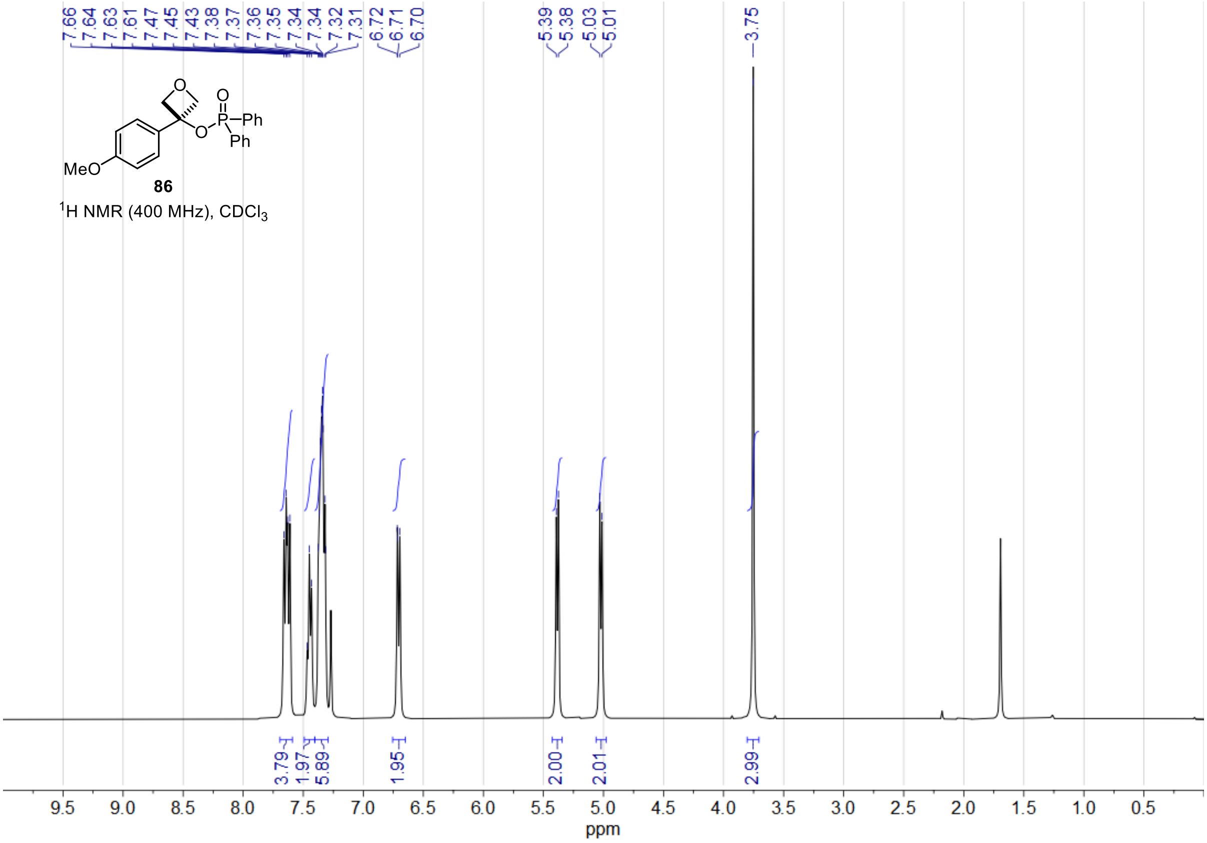
7.66
7.64
7.63
7.61
7.47
7.45
7.43
7.38
7.37
7.36
7.35
7.34
7.34
7.32
7.31
6.72
6.71
6.70
5.39
5.38
5.03
5.01
3.75
O
P
Ph
Ph
MeO
86
^{1}H NMR (400 MHz), CDCl$_3$
3.79
1.97
5.89
1.95
2.00
2.01
2.99
ppm

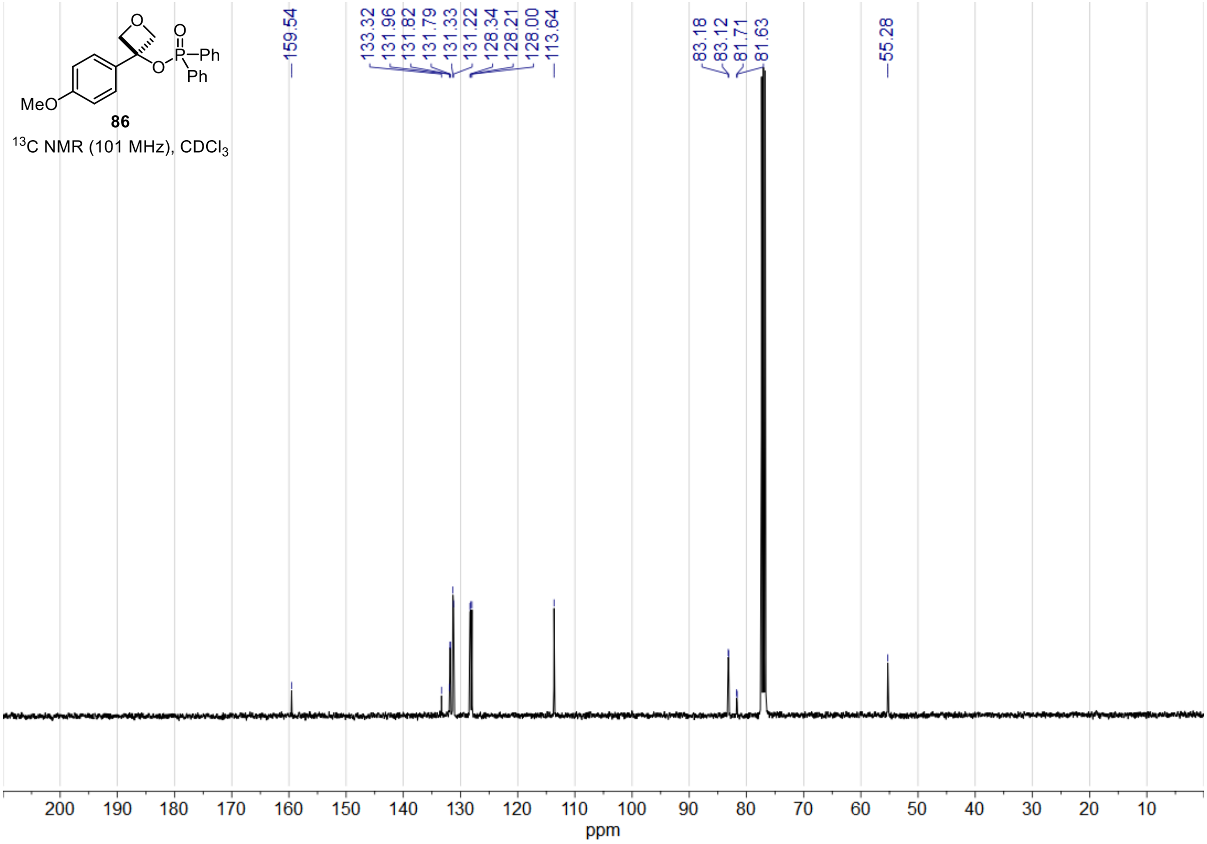
159.54
133.32
131.96
131.82
131.79
131.33
131.22
128.34
128.21
128.00
113.64
83.18
83.12
81.71
81.63
55.28
O
P
Ph
Ph
MeO
86
^{13}C NMR (101 MHz), CDCl$_3$
ppm

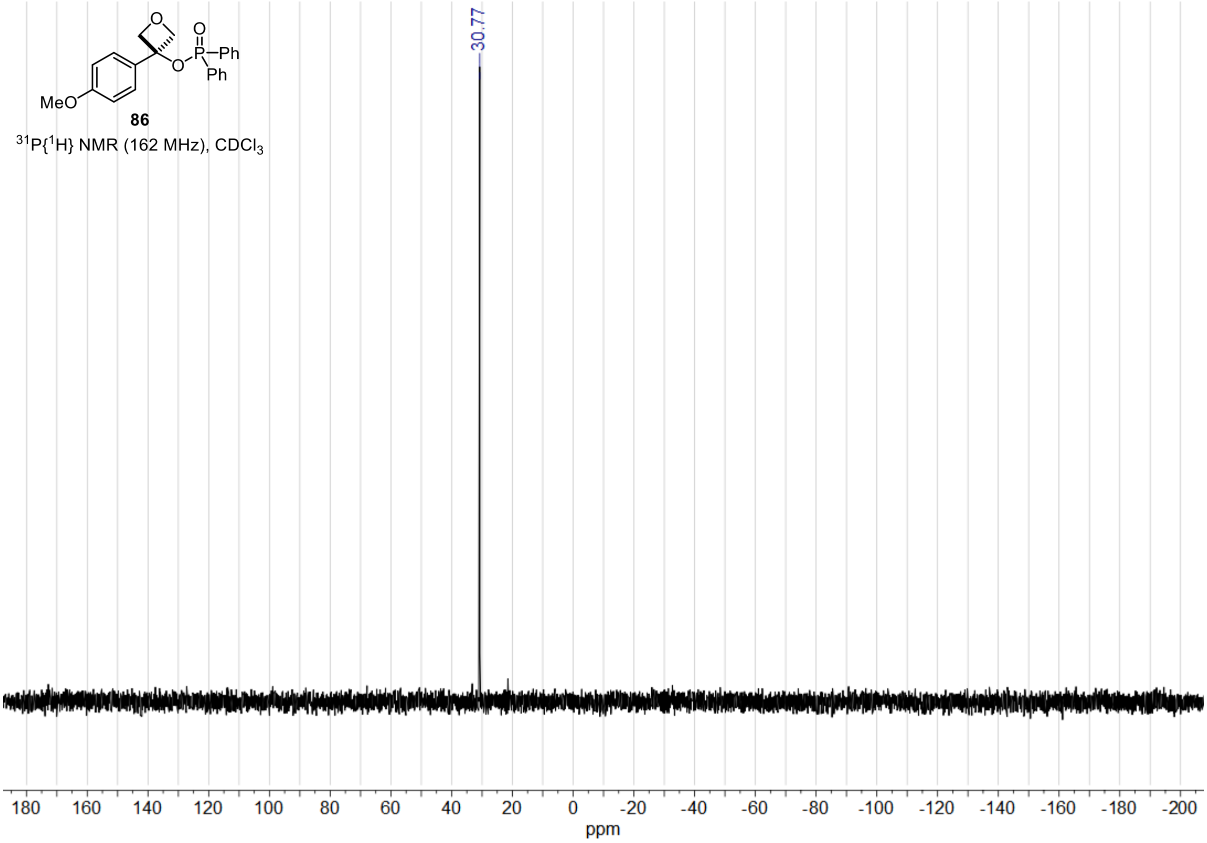

86

$^{31}P\{^{1}H\}$ NMR (162 MHz), $CDCl_3$

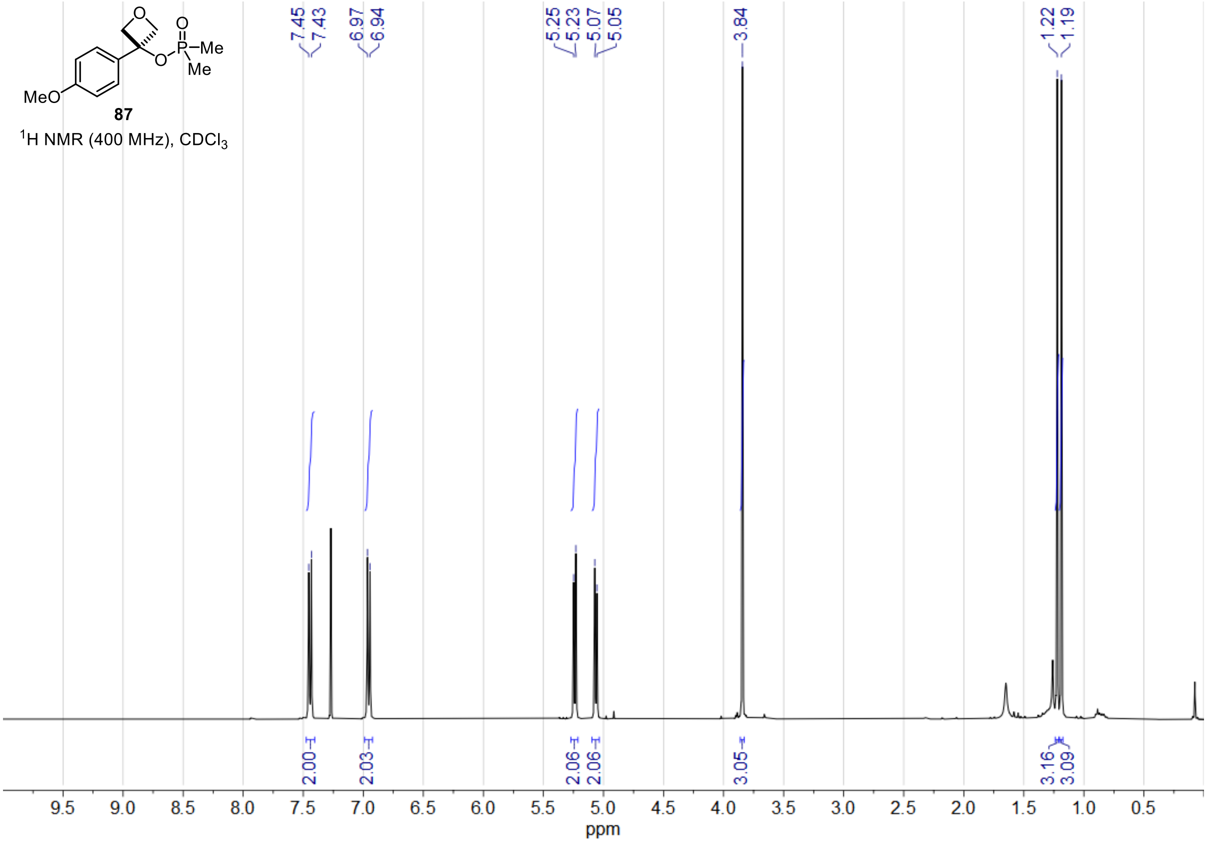
MeO
O
P
Me
87
1H NMR (400 MHz), CDCl3
7.45
7.43
6.97
6.94
5.25
5.23
5.07
5.05
3.84
1.22
1.19
2.00
2.03
2.06
2.06
3.05
3.16
3.09
ppm

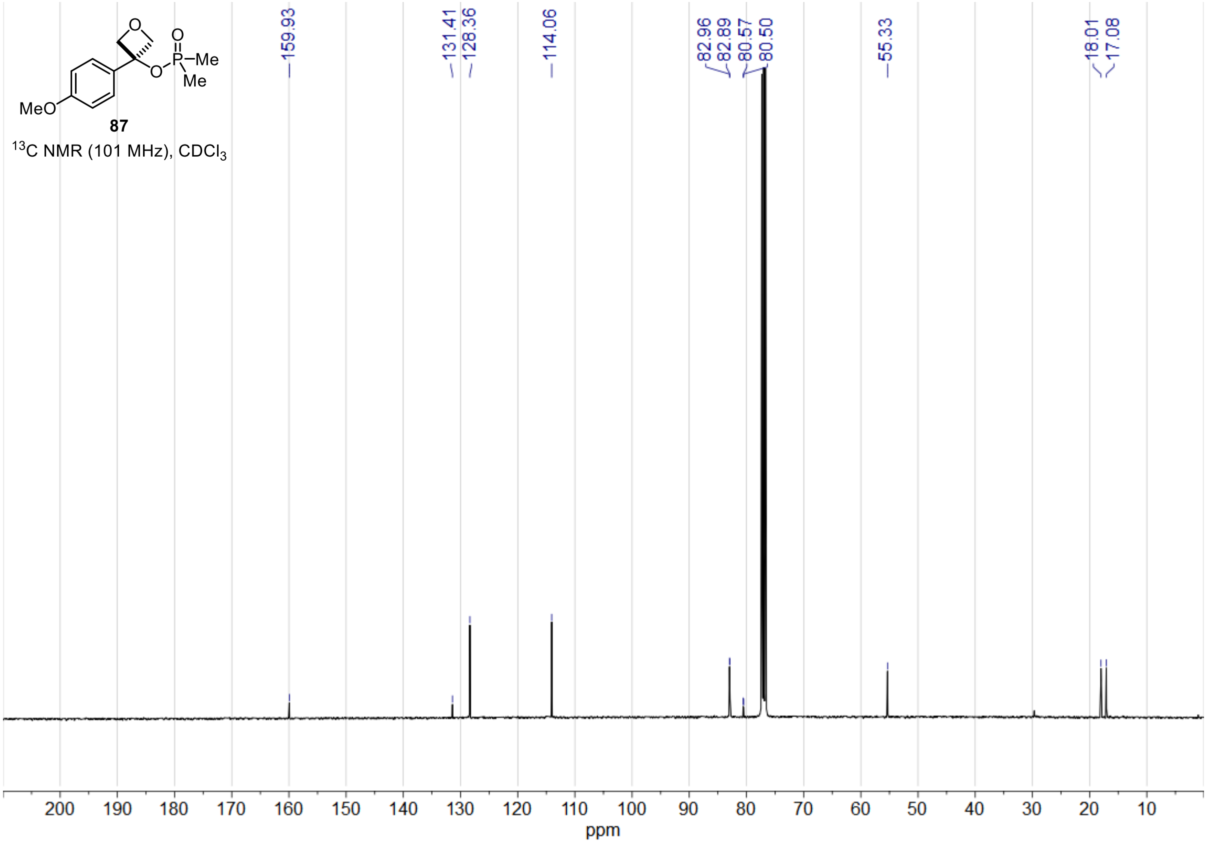
MeO
O
P
Me
87
13C NMR (101 MHz), CDCl3
159.93
131.41
128.36
114.06
82.96
82.89
80.57
80.50
55.33
18.01
17.08
ppm

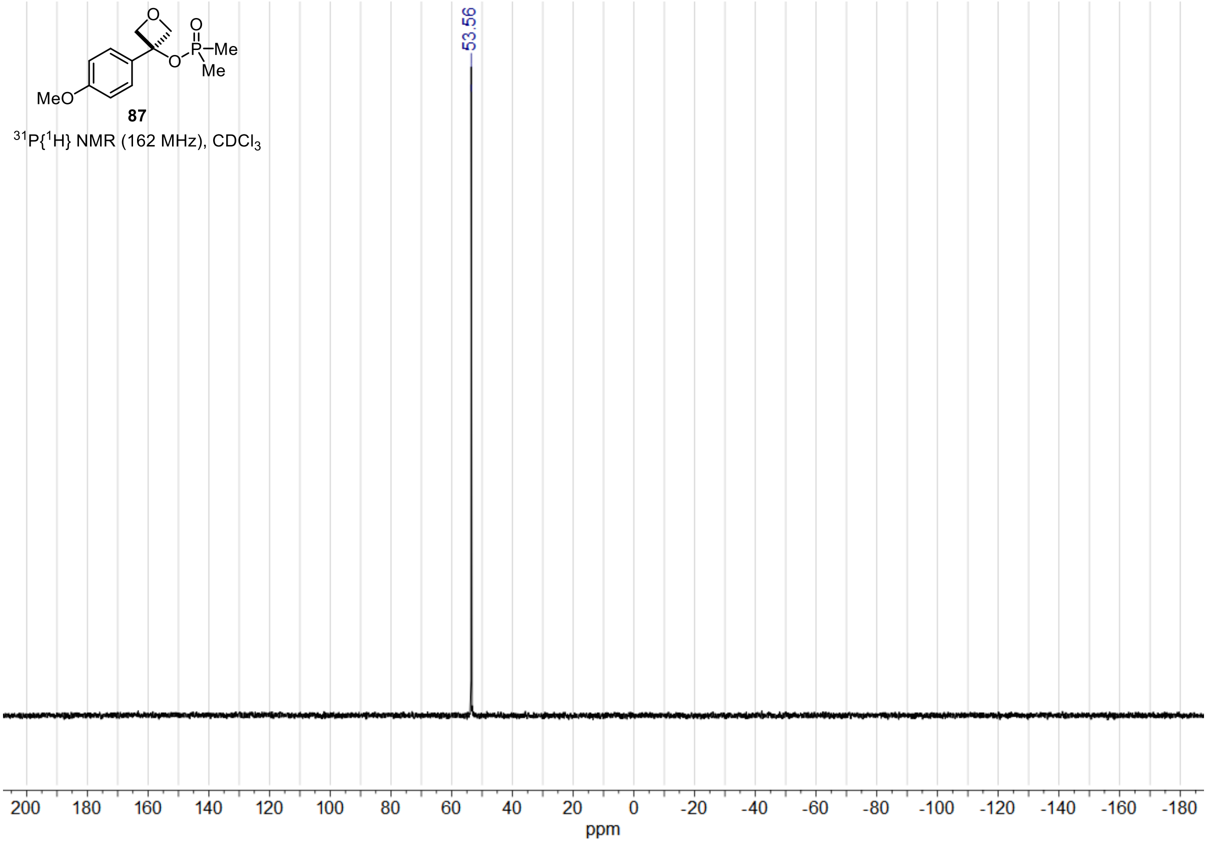

87

$^{31}P\{^{1}H\}$ NMR (162 MHz), $CDCl_3$

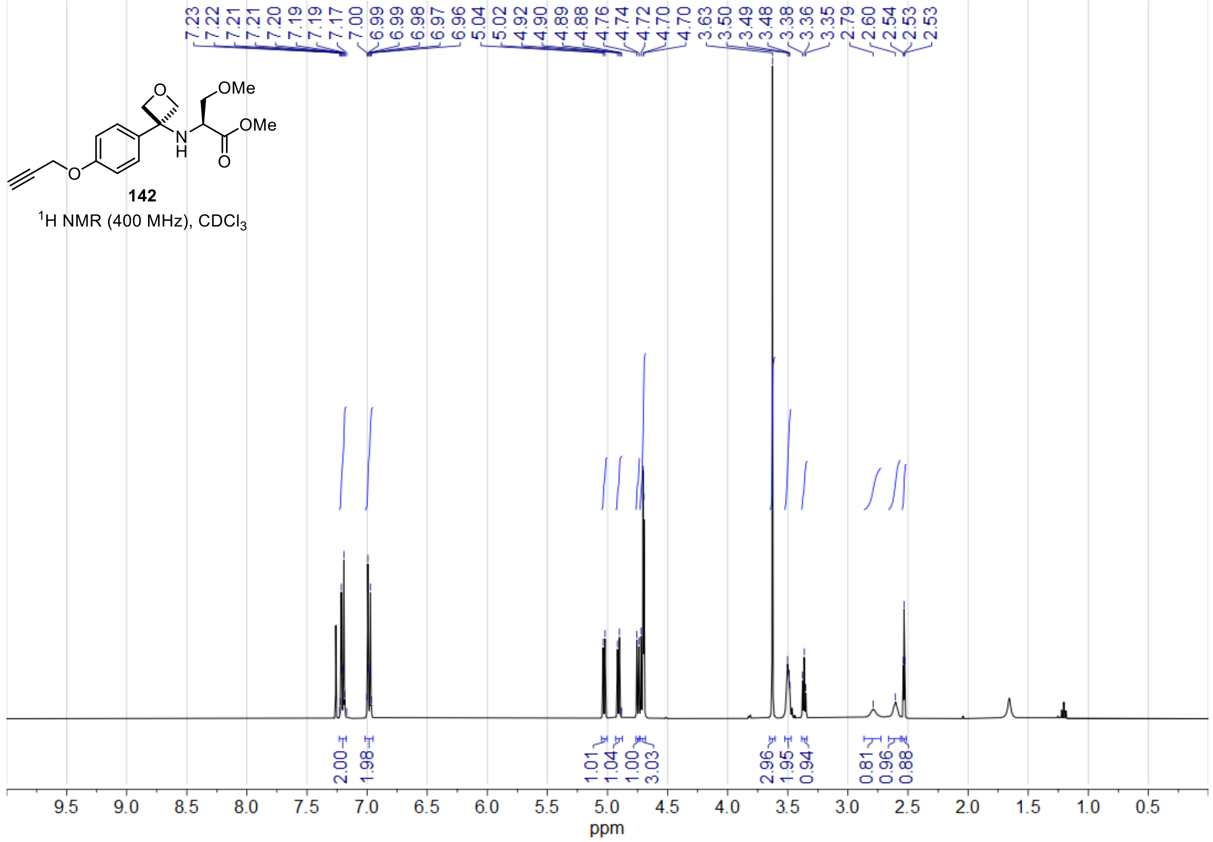
7.23
7.22
7.21
7.21
7.20
7.19
7.19
7.17
7.00
6.99
6.99
6.98
6.97
6.96
5.04
5.02
4.92
4.90
4.89
4.88
4.76
4.74
4.72
4.70
4.70
3.63
3.50
3.49
3.48
3.38
3.36
3.35
2.79
2.60
2.54
2.53
2.53
OMe
OMe
O
N
H
O
142
^{1}H NMR (400 MHz), $CDCl_3$
2.00
1.98
1.01
1.04
1.00
3.03
2.96
1.95
0.94
0.81
0.96
0.88
9.5
9.0
8.5
8.0
7.5
7.0
6.5
6.0
5.5
5.0
4.5
4.0
3.5
3.0
2.5
2.0
1.5
1.0
0.5
ppm

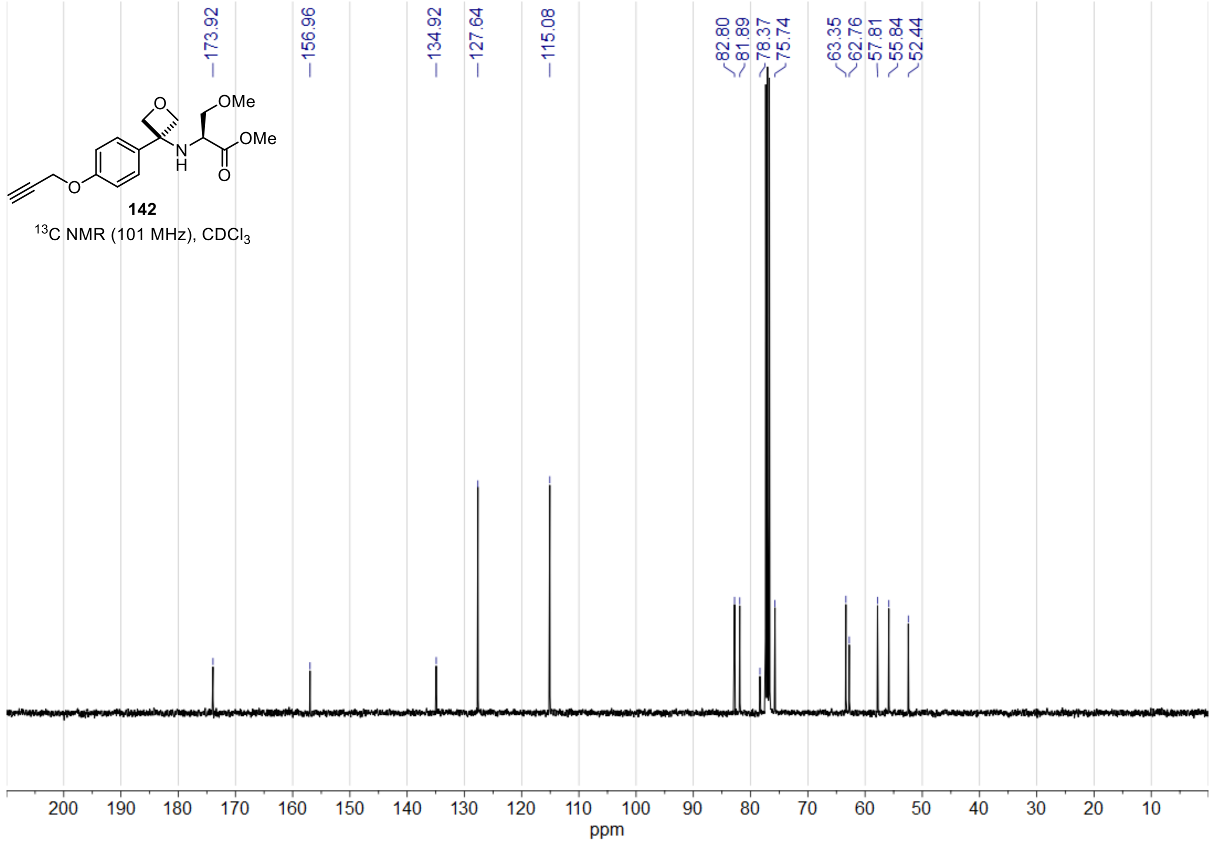
173.92
156.96
134.92
127.64
115.08
82.80
81.89
78.37
75.74
63.35
62.76
57.81
55.84
52.44
OMe
OMe
O
N
H
O
142
^{13}C NMR (101 MHz), $CDCl_3$
200
190
180
170
160
150
140
130
120
110
100
90
80
70
60
50
40
30
20
10
ppm

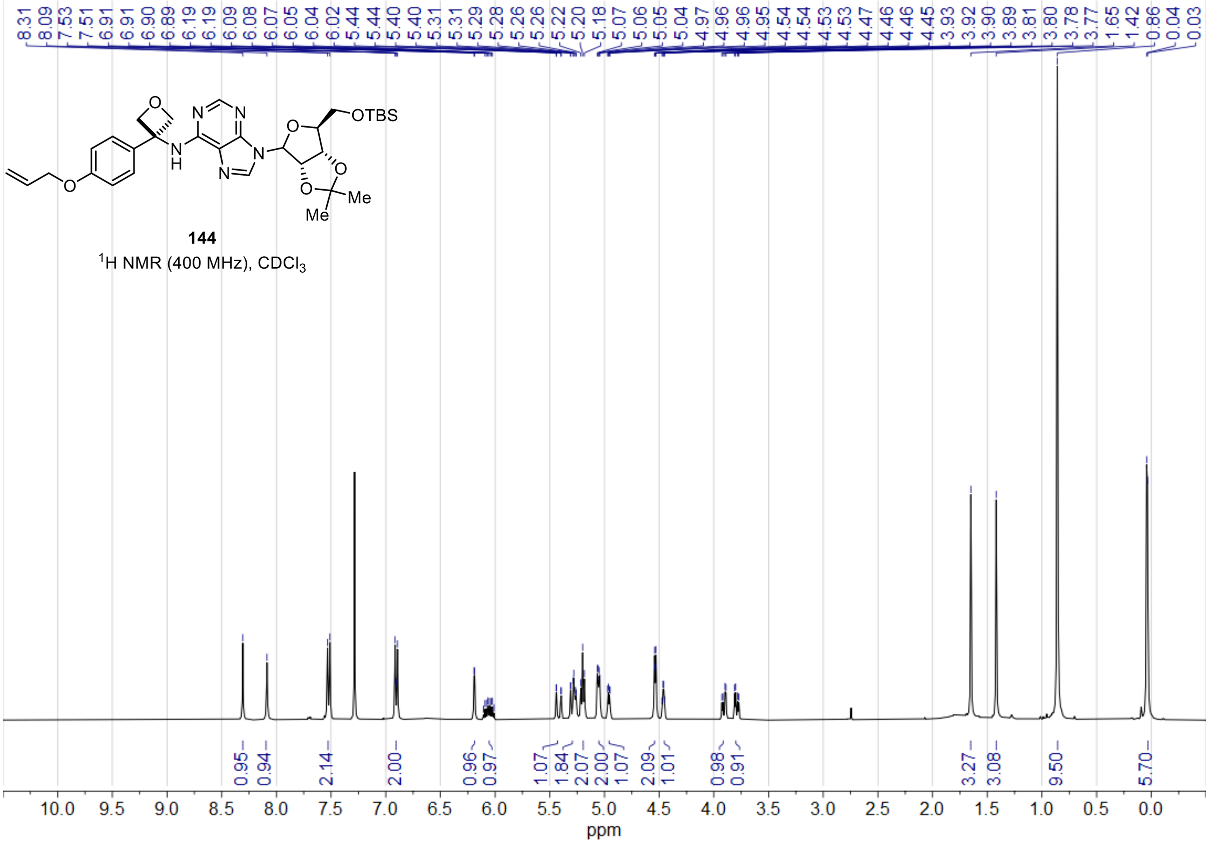
8.31 8.09 7.53 7.51 6.91 6.91 6.90 6.89 6.19 6.19 6.09 6.08 6.07 6.05 6.04 6.02 5.44 5.44 5.40 5.40 5.31 5.31 5.29 5.28 5.26 5.26 5.22 5.20 5.18 5.07 5.06 5.05 5.04 4.97 4.96 4.96 4.95 4.54 4.54 4.53 4.53 4.47 4.46 4.46 4.45 3.93 3.92 3.90 3.89 3.81 3.80 3.78 3.77 1.65 1.42 0.86 0.04 0.03
OTBS
N
N
N
H
O
Me
Me
144
^{1}H NMR (400 MHz), CDCl$_3$
0.95 0.94 2.14 2.00 0.96 0.97 1.07 1.84 2.07 2.00 1.07 2.09 1.01 0.98 0.91 3.27 3.08 9.50 5.70
10.0 9.5 9.0 8.5 8.0 7.5 7.0 6.5 6.0 5.5 5.0 4.5 4.0 3.5 3.0 2.5 2.0 1.5 1.0 0.5 0.0
ppm

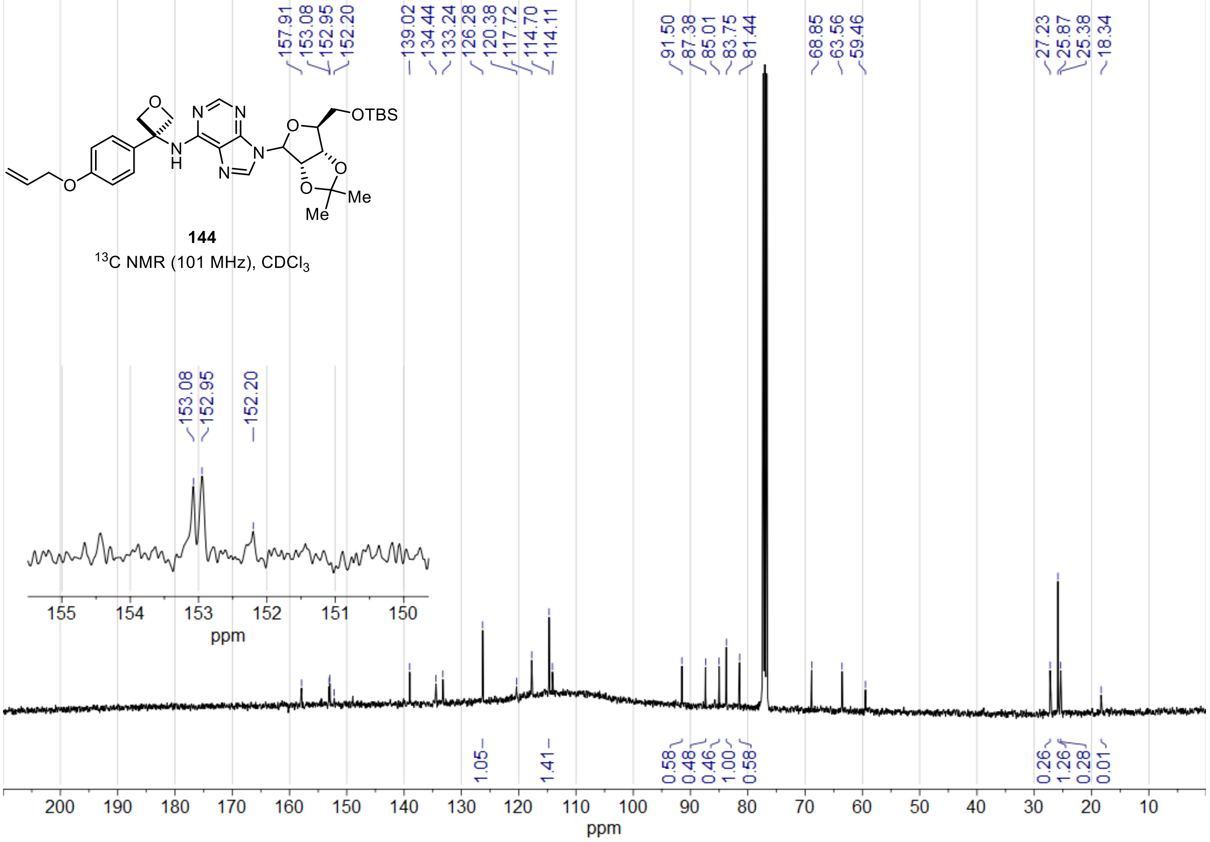
157.91 153.08 152.95 152.20 139.02 134.44 133.24 126.28 120.38 117.72 114.70 114.11 91.50 87.38 85.01 83.75 81.44 68.85 63.56 59.46 27.23 25.87 25.38 18.34
OTBS
Me
Me
144
^{13}C NMR (101 MHz), CDCl$_3$
153.08 152.95 152.20
155 154 153 152 151 150
ppm
1.05 1.41 0.58 0.48 0.46 1.00 0.58 0.26 1.26 0.28 0.01
200 190 180 170 160 150 140 130 120 110 100 90 80 70 60 50 40 30 20 10
ppm

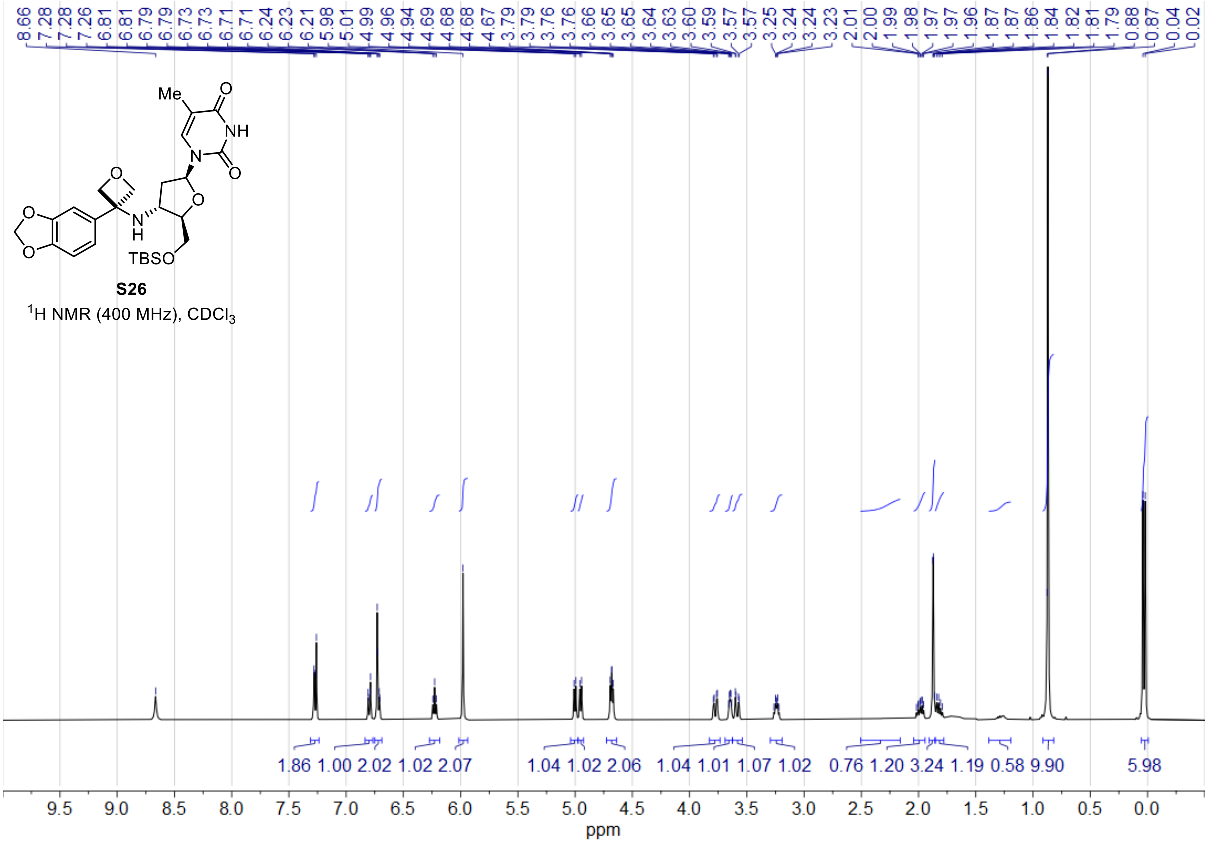

S26

^{1}H NMR (400 MHz), $CDCl_3$

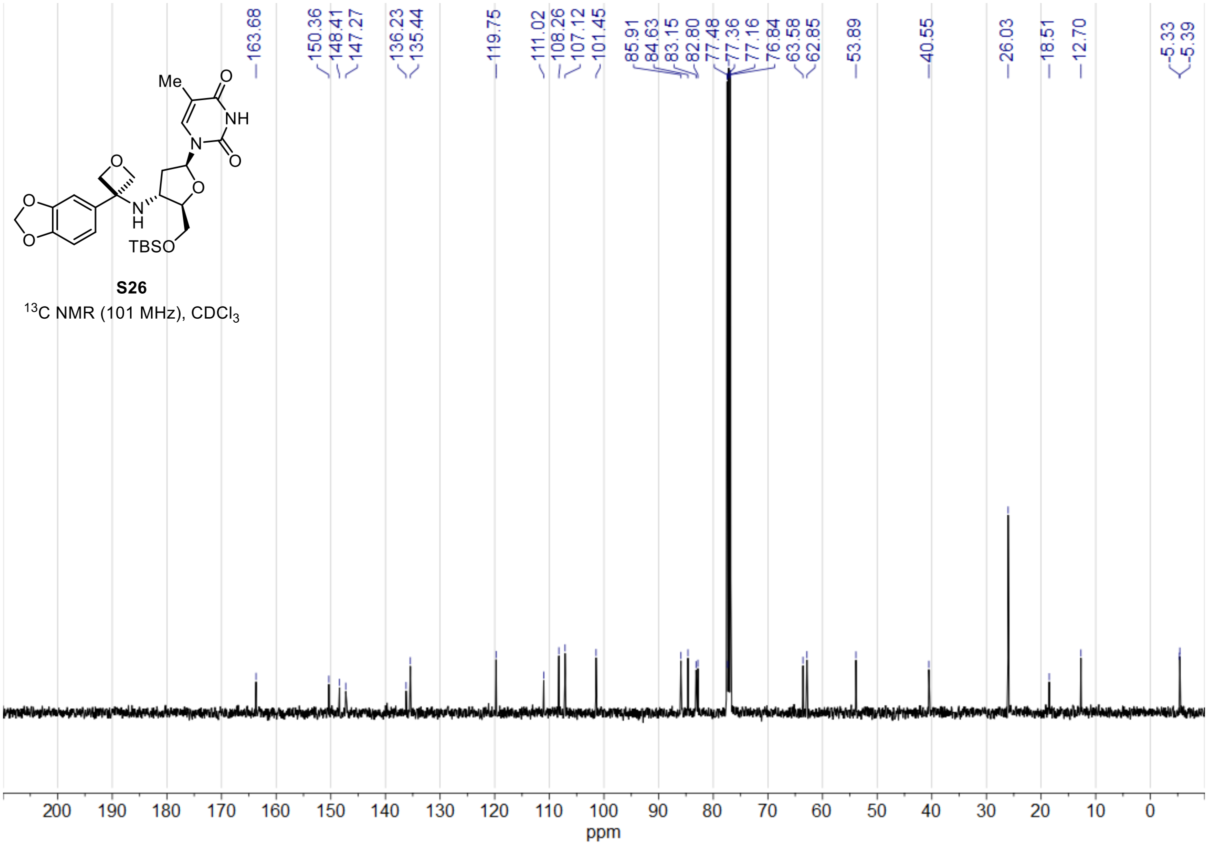

S26

^{13}C NMR (101 MHz), $CDCl_3$

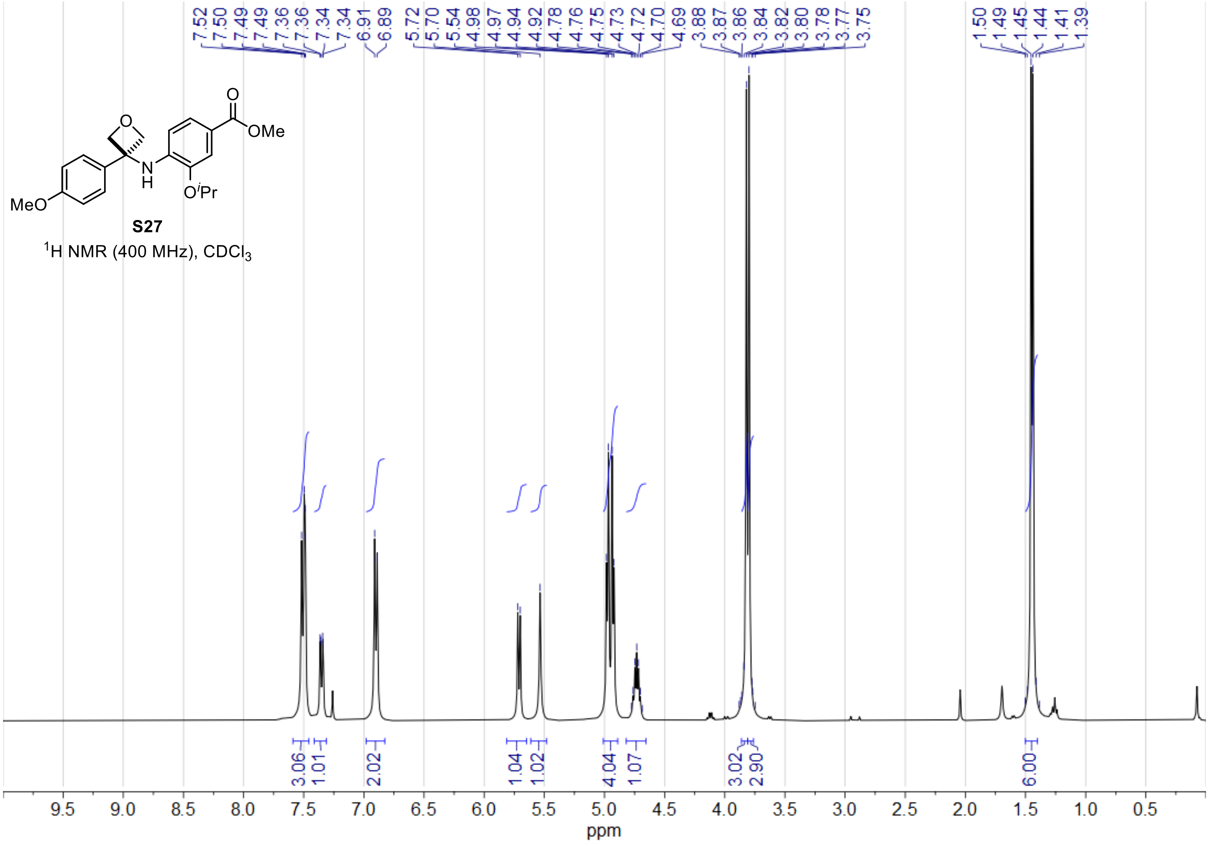
S27
^{1}H NMR (400 MHz), CDCl$_3$
7.52 7.50 7.49 7.49 7.36 7.36 7.34 7.34 6.91 6.89 5.72 5.70 5.54 4.98 4.97 4.94 4.92 4.78 4.76 4.75 4.73 4.72 4.70 4.69 3.88 3.87 3.86 3.84 3.82 3.80 3.78 3.77 3.75 1.50 1.49 1.45 1.44 1.41 1.39
3.06 1.01 2.02 1.04 1.02 4.04 1.07 3.02 2.90 6.00
ppm

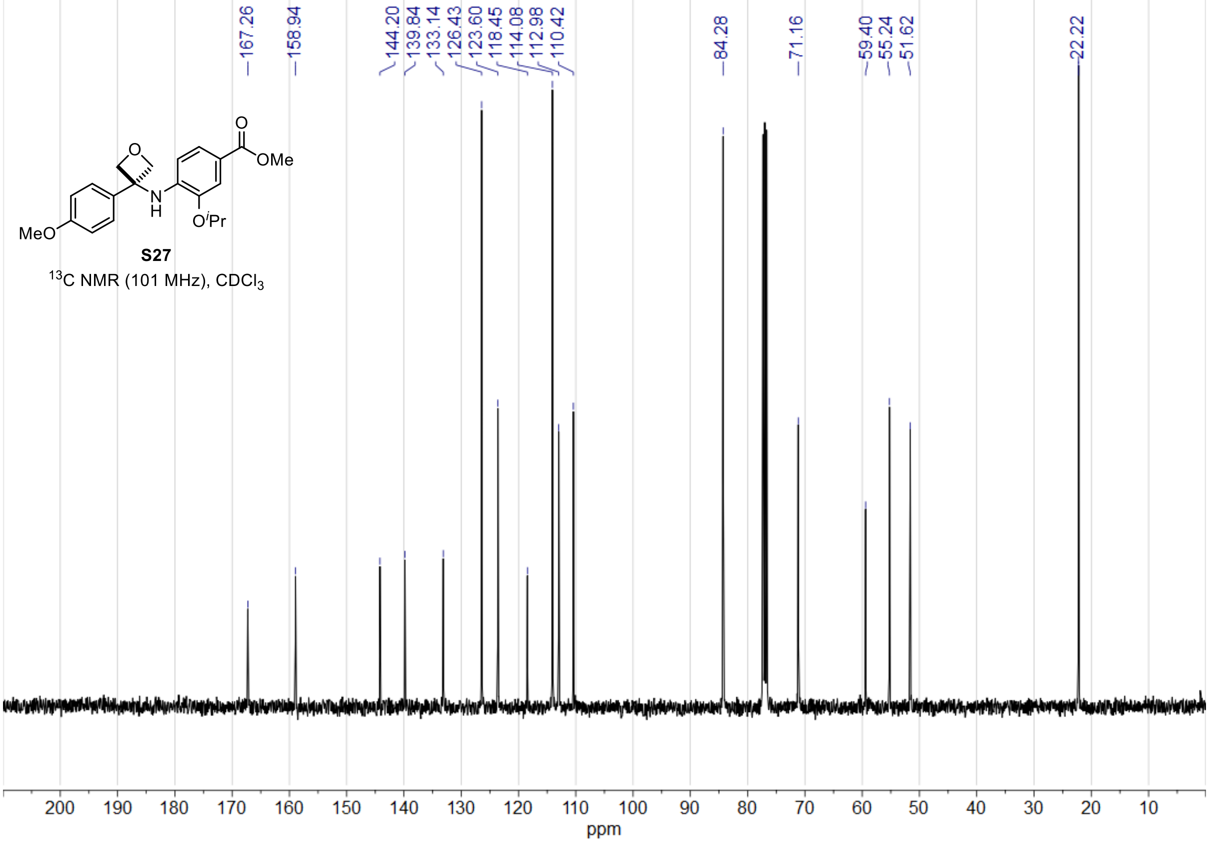
S27
^{13}C NMR (101 MHz), CDCl$_3$
167.26 158.94 144.20 139.84 133.14 126.43 123.60 118.45 114.08 112.98 110.42 84.28 71.16 59.40 55.24 51.62 22.22
ppm

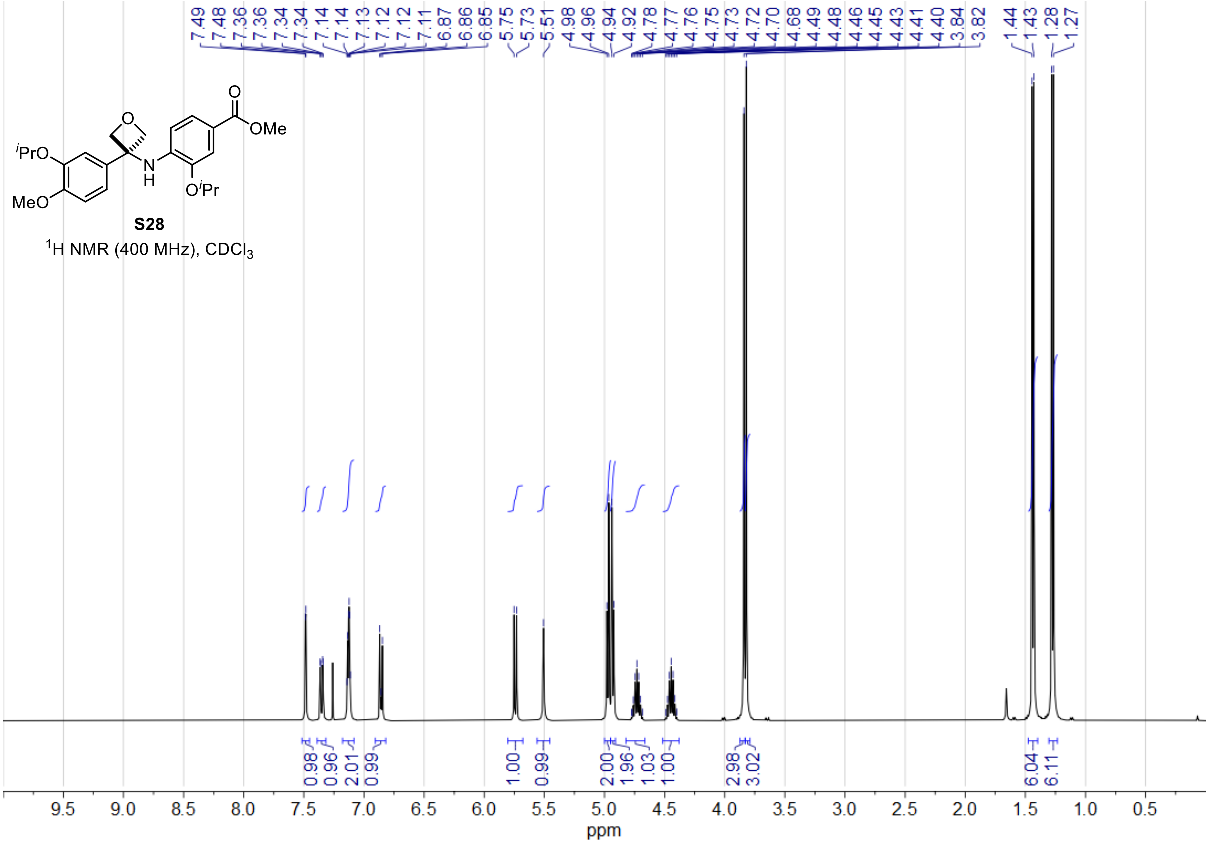

7.49 7.48 7.36 7.36 7.34 7.34 7.14 7.14 7.13 7.12 7.12 7.11 6.87 6.86 6.85 5.75 5.73 5.51 4.98 4.96 4.94 4.92 4.78 4.77 4.76 4.75 4.73 4.72 4.70 4.68 4.49 4.48 4.46 4.45 4.43 4.41 4.40 3.84 3.82 1.44 1.43 1.28 1.27
iPrO
MeO
O
OMe
N
H
OiPr
S28
1H NMR (400 MHz), CDCl3
0.98 0.96 2.01 0.99 1.00 0.99 2.00 1.96 1.03 1.00 2.98 3.02 6.04 6.11
9.5 9.0 8.5 8.0 7.5 7.0 6.5 6.0 5.5 5.0 4.5 4.0 3.5 3.0 2.5 2.0 1.5 1.0 0.5
ppm

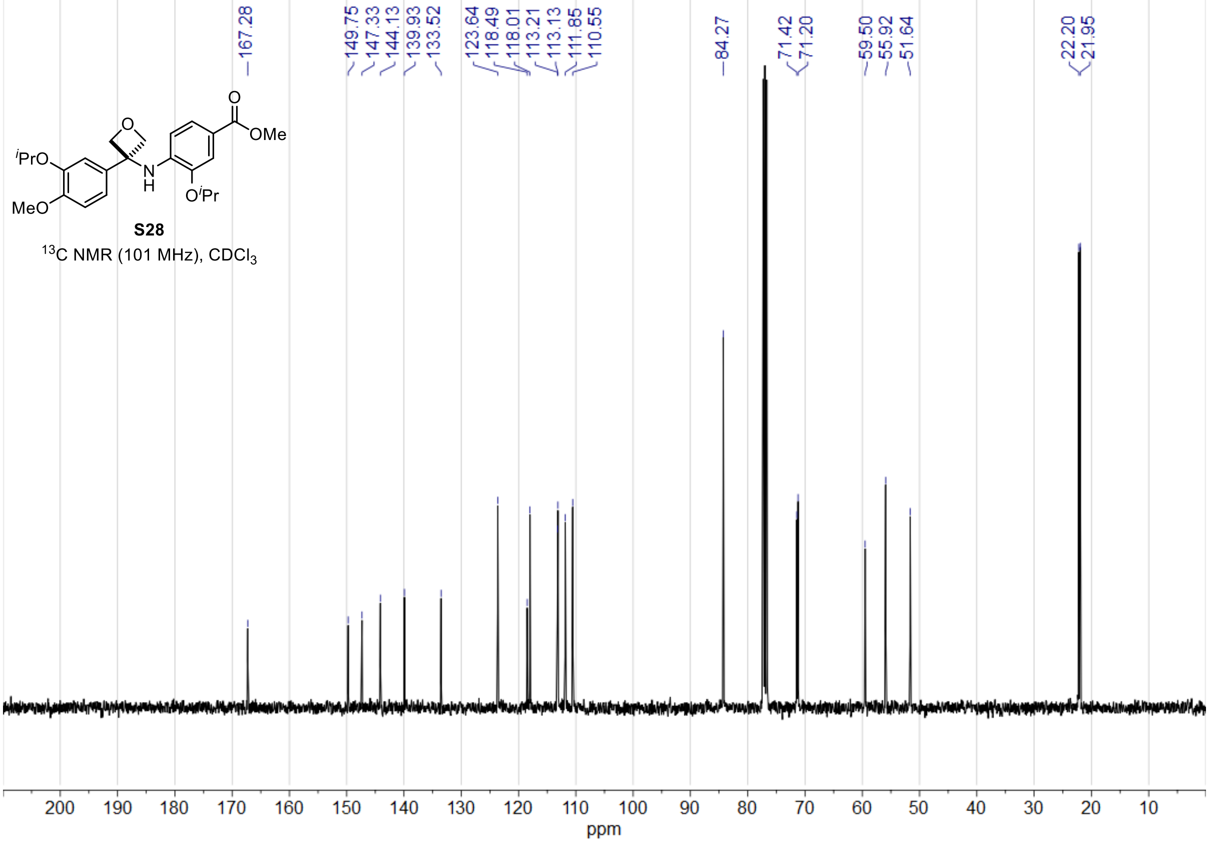

167.28
149.75 147.33 144.13 139.93 133.52
123.64 118.49 118.01 113.21 113.13 111.85 110.55
84.27
71.42 71.20
59.50 55.92 51.64
22.20 21.95
iPrO
MeO
O
OMe
N
H
OiPr
S28
13C NMR (101 MHz), CDCl3
200 190 180 170 160 150 140 130 120 110 100 90 80 70 60 50 40 30 20 10
ppm

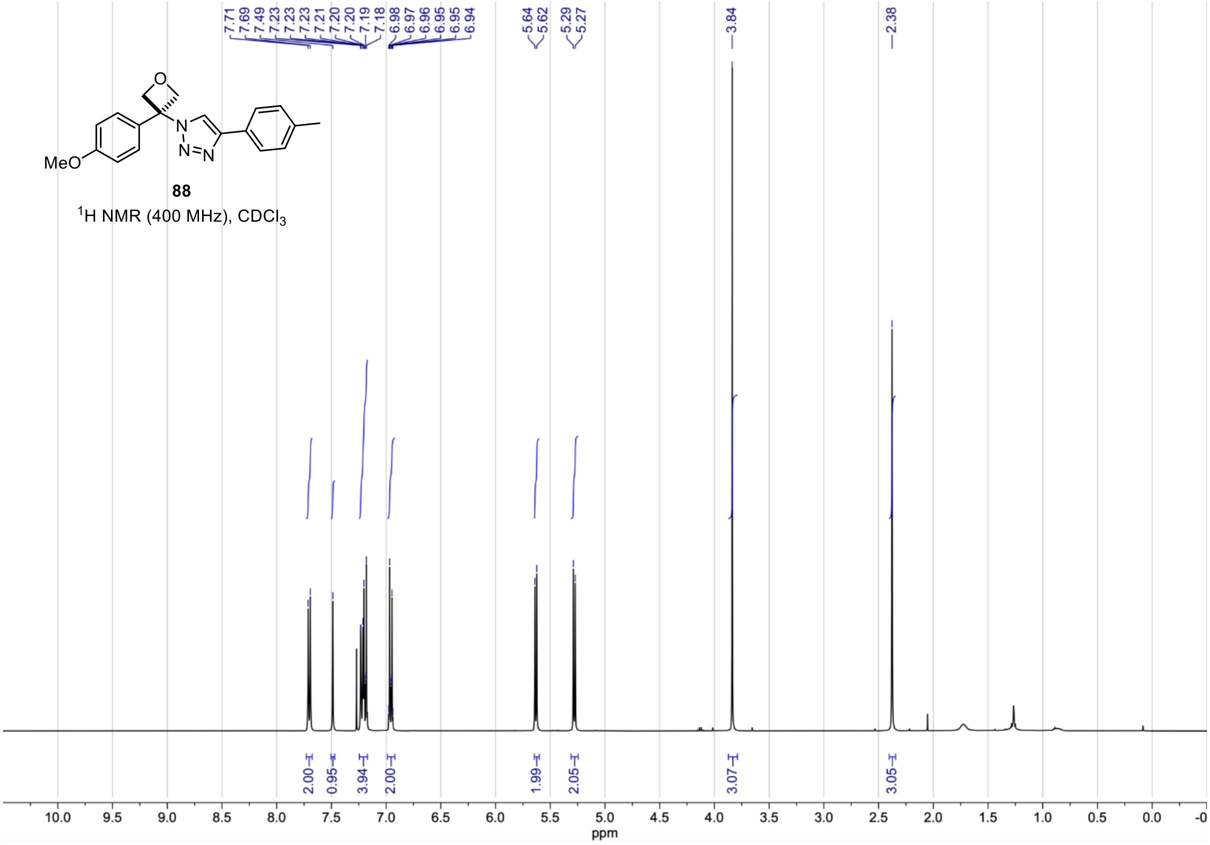

88
^{1}H NMR (400 MHz), CDCl$_3$
7.71
7.69
7.49
7.23
7.23
7.23
7.21
7.20
7.20
7.19
7.18
6.98
6.97
6.96
6.95
6.95
6.94
5.64
5.62
5.29
5.27
3.84
2.38
2.00
0.95
3.94
2.00
1.99
2.05
3.07
3.05
ppm

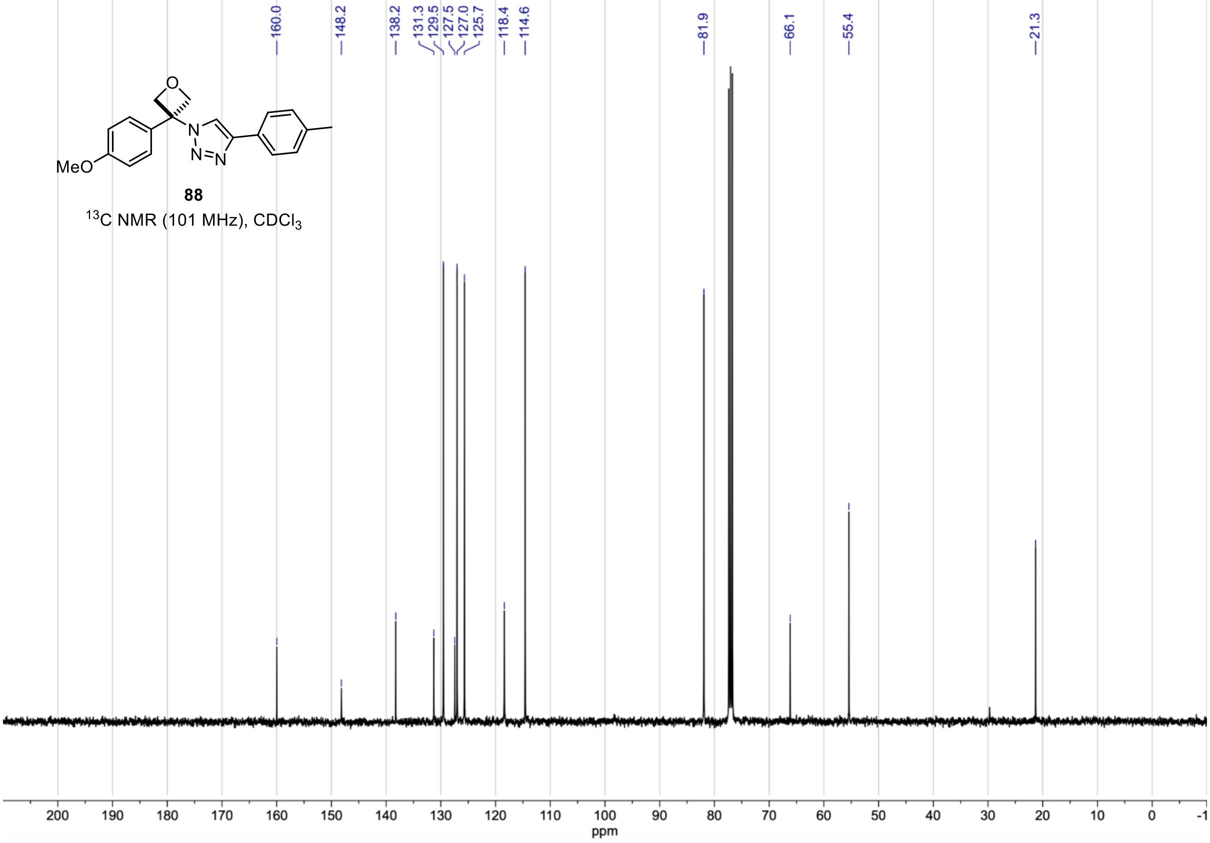

88
^{13}C NMR (101 MHz), CDCl$_3$
160.0
148.2
138.2
131.3
129.5
127.5
127.0
125.7
118.4
114.6
81.9
66.1
55.4
21.3
ppm

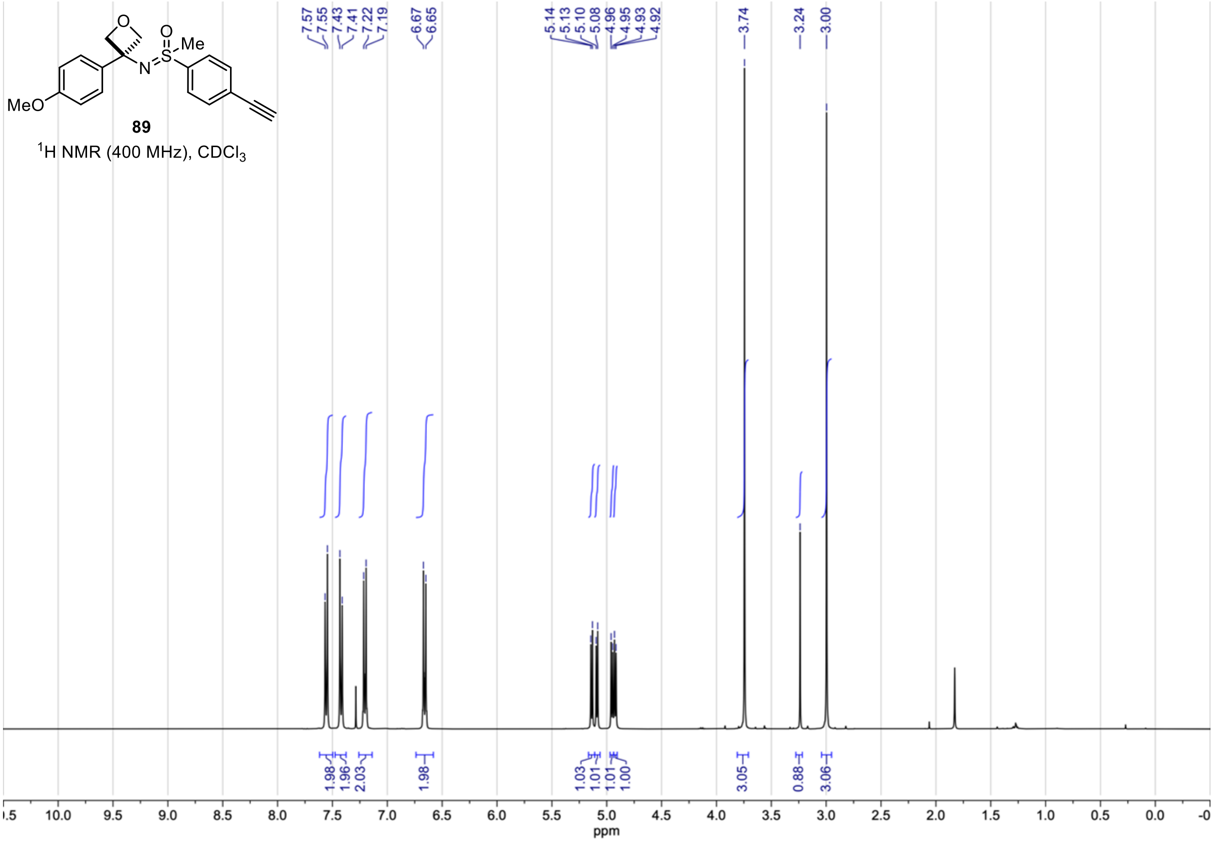

89

^{1}H NMR (400 MHz), $CDCl_3$

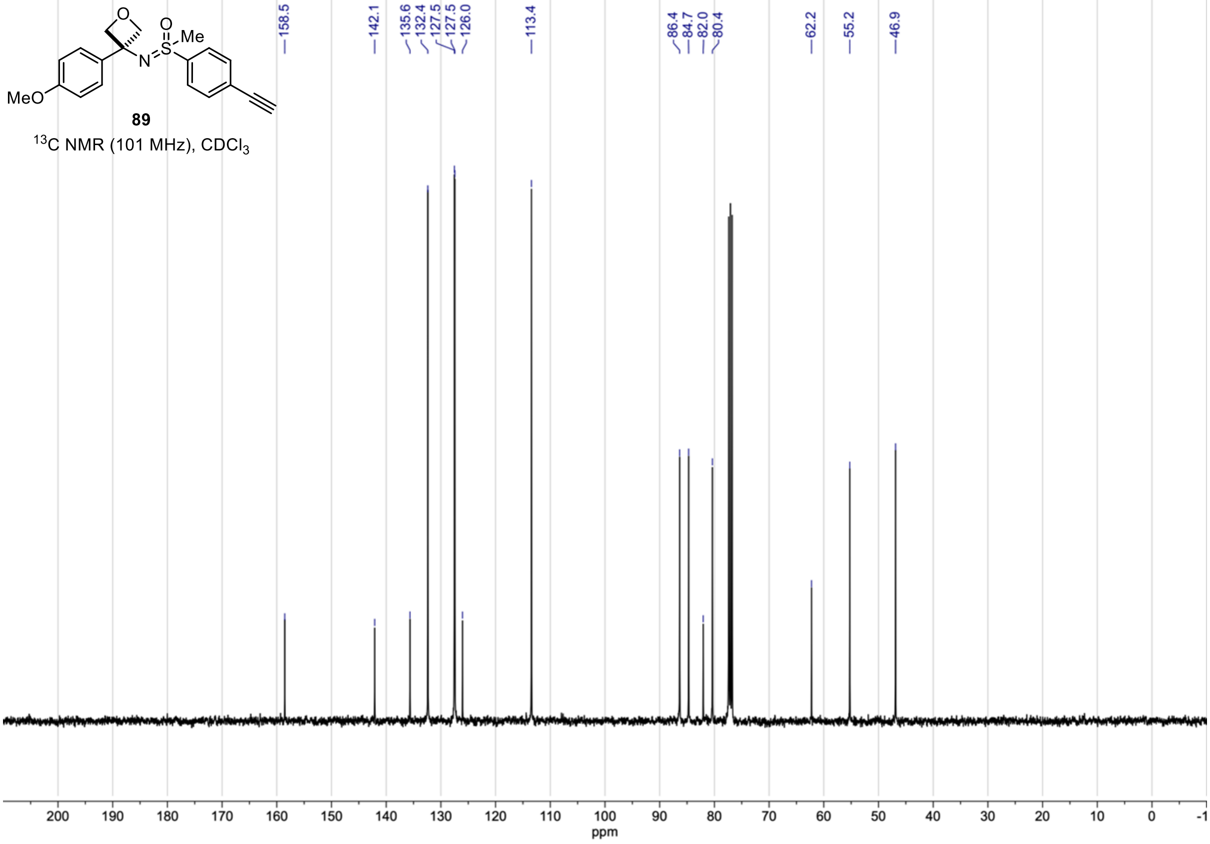

89

^{13}C NMR (101 MHz), $CDCl_3$

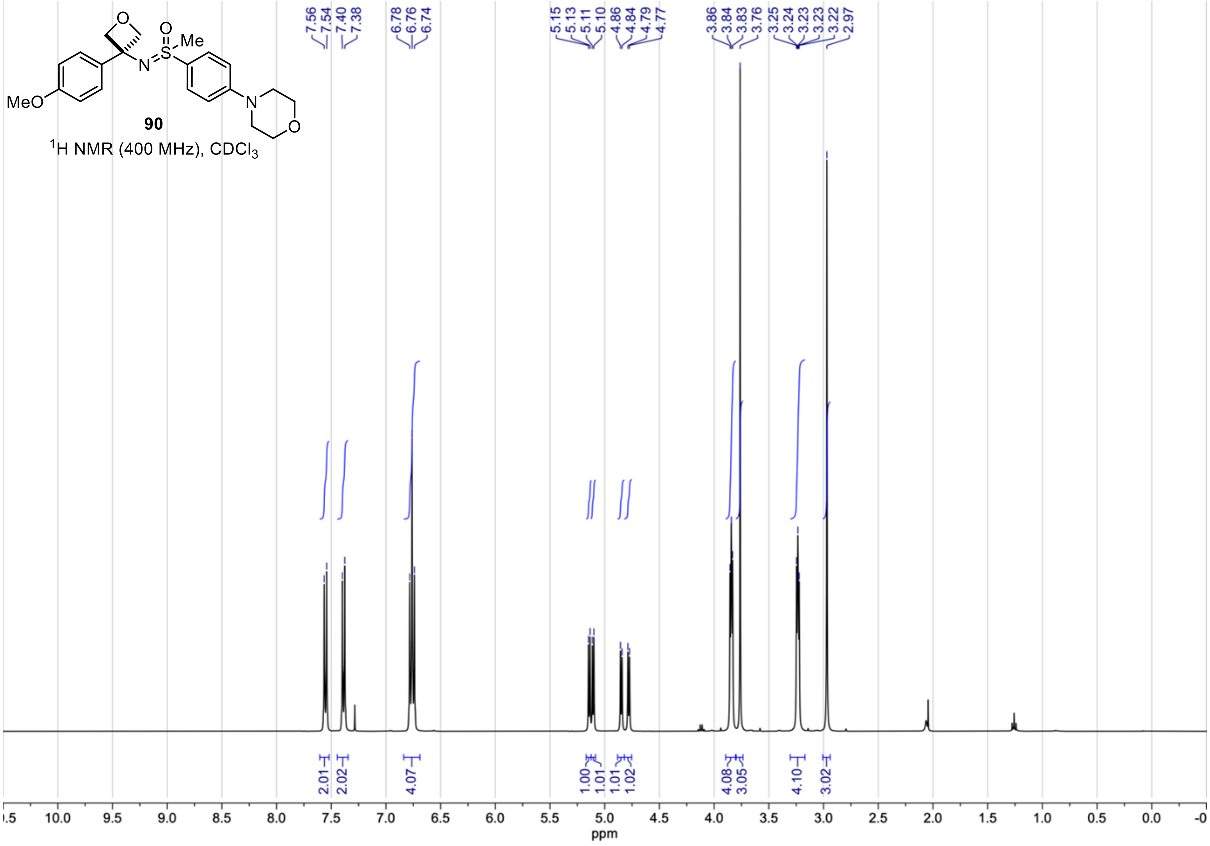
MeO
Me
90
^{1}H NMR (400 MHz), CDCl3
7.56
7.54
7.40
7.38
6.78
6.76
6.74
5.15
5.13
5.11
5.10
4.86
4.84
4.79
4.77
3.86
3.84
3.83
3.76
3.25
3.24
3.23
3.23
3.22
2.97
2.01
2.02
4.07
1.00
1.01
1.01
1.02
4.08
3.05
4.10
3.02
ppm

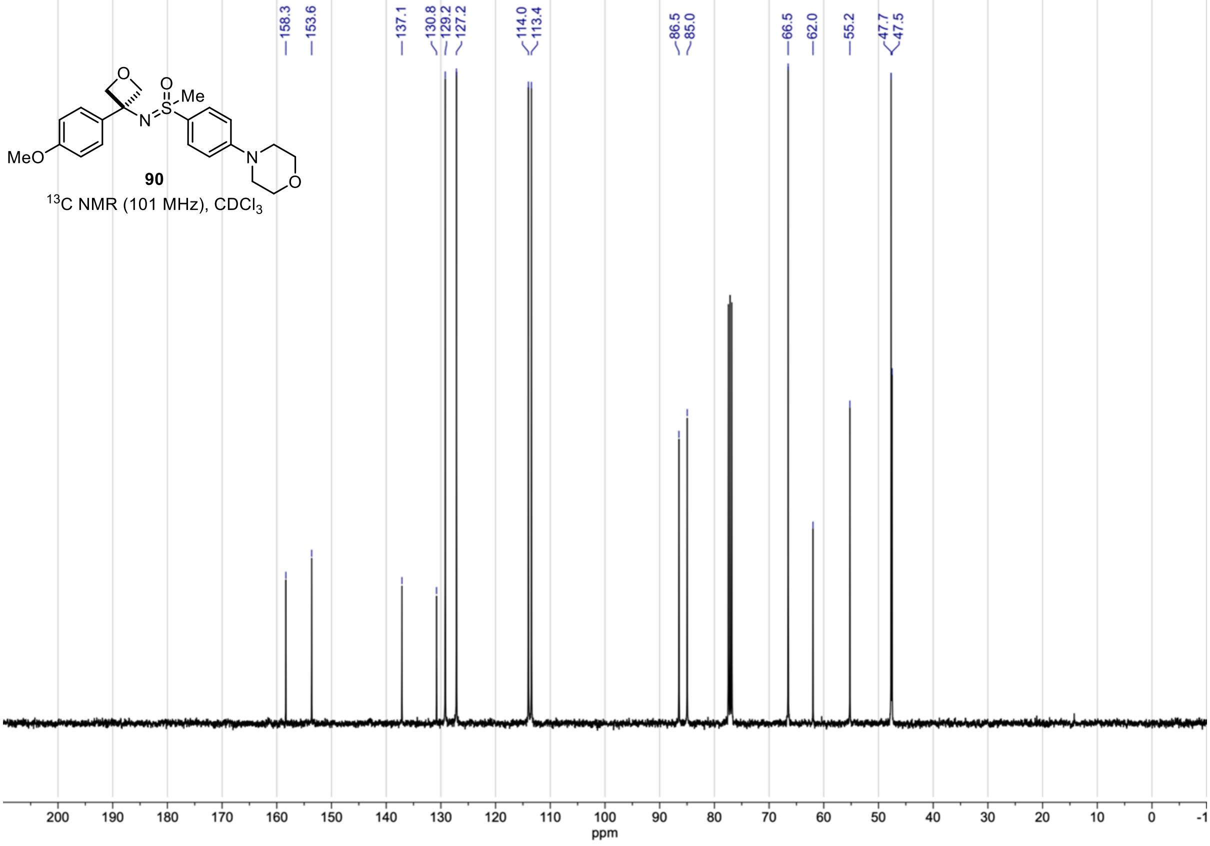
MeO
Me
90
^{13}C NMR (101 MHz), CDCl3
158.3
153.6
137.1
130.8
129.2
127.2
114.0
113.4
86.5
85.0
66.5
62.0
55.2
47.7
47.5
ppm

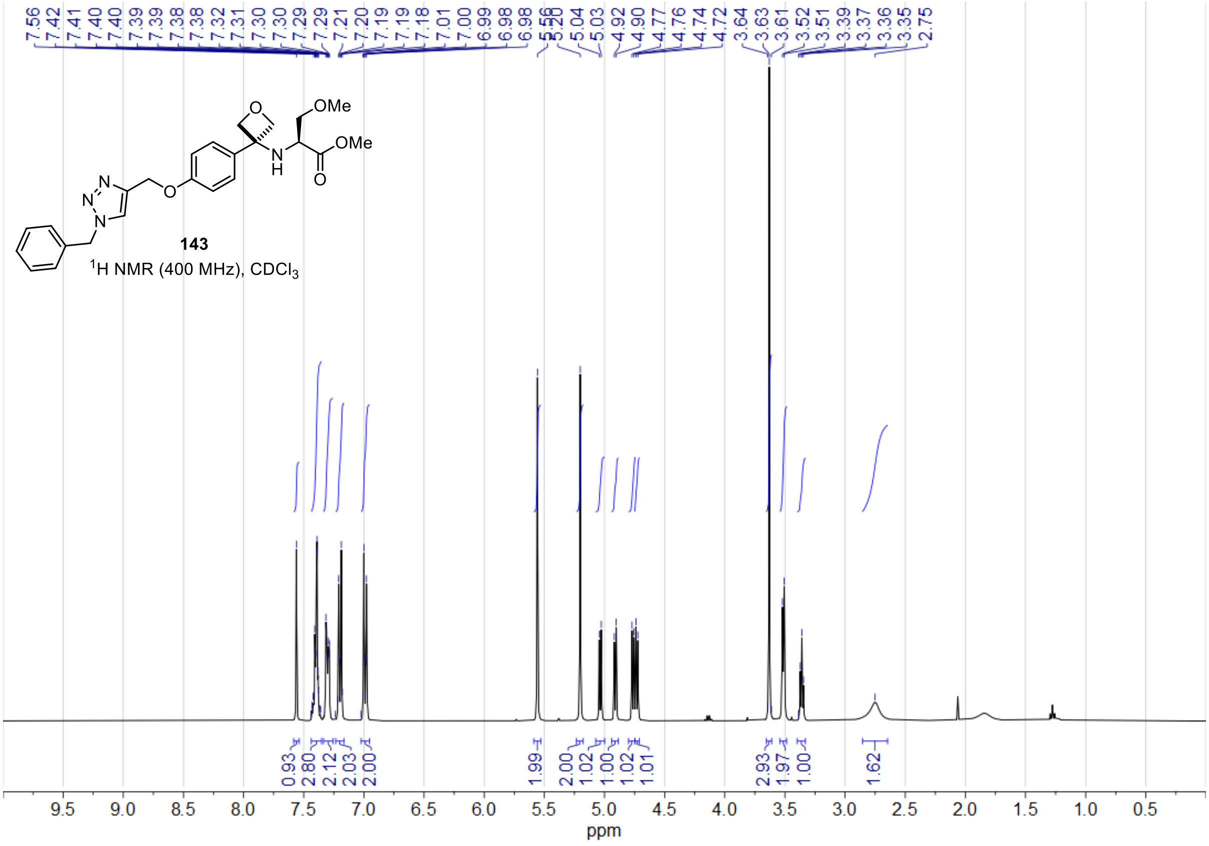
7.56
7.42
7.41
7.40
7.40
7.39
7.39
7.38
7.38
7.32
7.31
7.30
7.30
7.29
7.29
7.21
7.20
7.19
7.19
7.18
7.01
7.00
6.99
6.98
6.98
5.56
5.20
5.04
5.03
4.92
4.90
4.77
4.76
4.74
4.72
3.64
3.63
3.61
3.52
3.51
3.39
3.37
3.36
3.35
2.75
OMe
O
N
H
OMe
O
N
N
N
O
143
^{1}H NMR (400 MHz), CDCl3
0.93
2.80
2.12
2.03
2.00
1.99
2.00
1.02
1.00
1.02
1.01
2.93
1.97
1.00
1.62
9.5
9.0
8.5
8.0
7.5
7.0
6.5
6.0
5.5
5.0
4.5
4.0
3.5
3.0
2.5
2.0
1.5
1.0
0.5
ppm

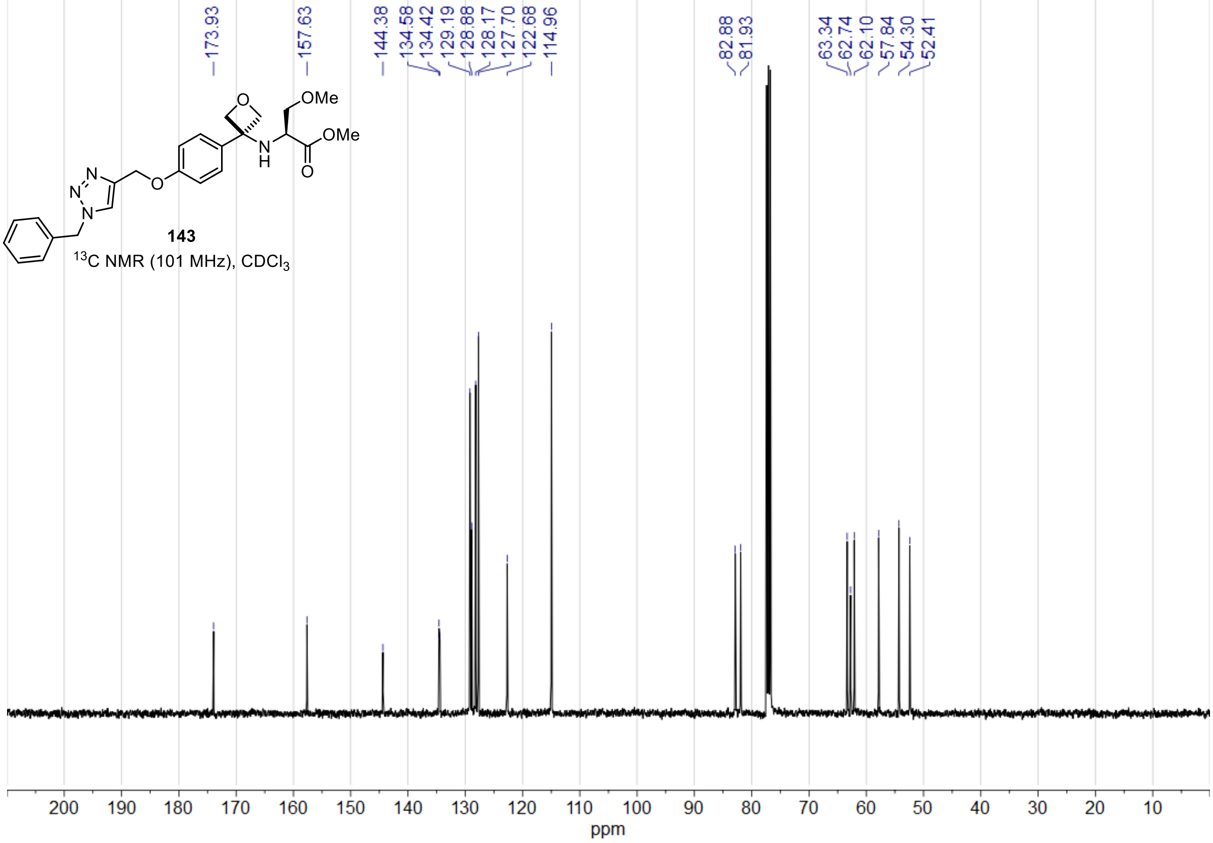
173.93
157.63
144.38
134.58
134.42
129.19
128.88
128.17
127.70
122.68
114.96
82.88
81.93
63.34
62.74
62.10
57.84
54.30
52.41
OMe
O
N
H
OMe
O
N
N
N
O
143
^{13}C NMR (101 MHz), CDCl3
200
190
180
170
160
150
140
130
120
110
100
90
80
70
60
50
40
30
20
10
ppm

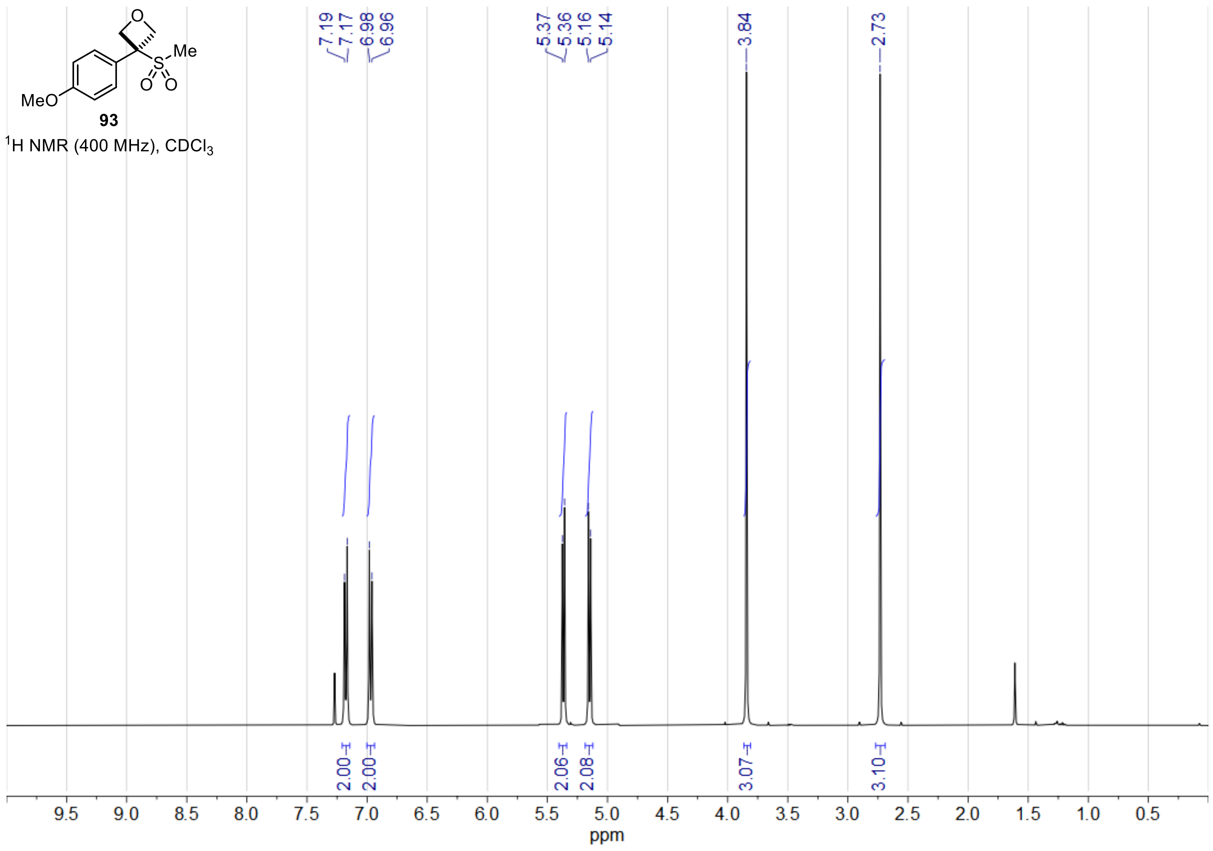

MeO
Me
93
1H NMR (400 MHz), CDCl3
7.19
7.17
6.98
6.96
5.37
5.36
5.16
5.14
3.84
2.73
2.00
2.00
2.06
2.08
3.07
3.10
ppm

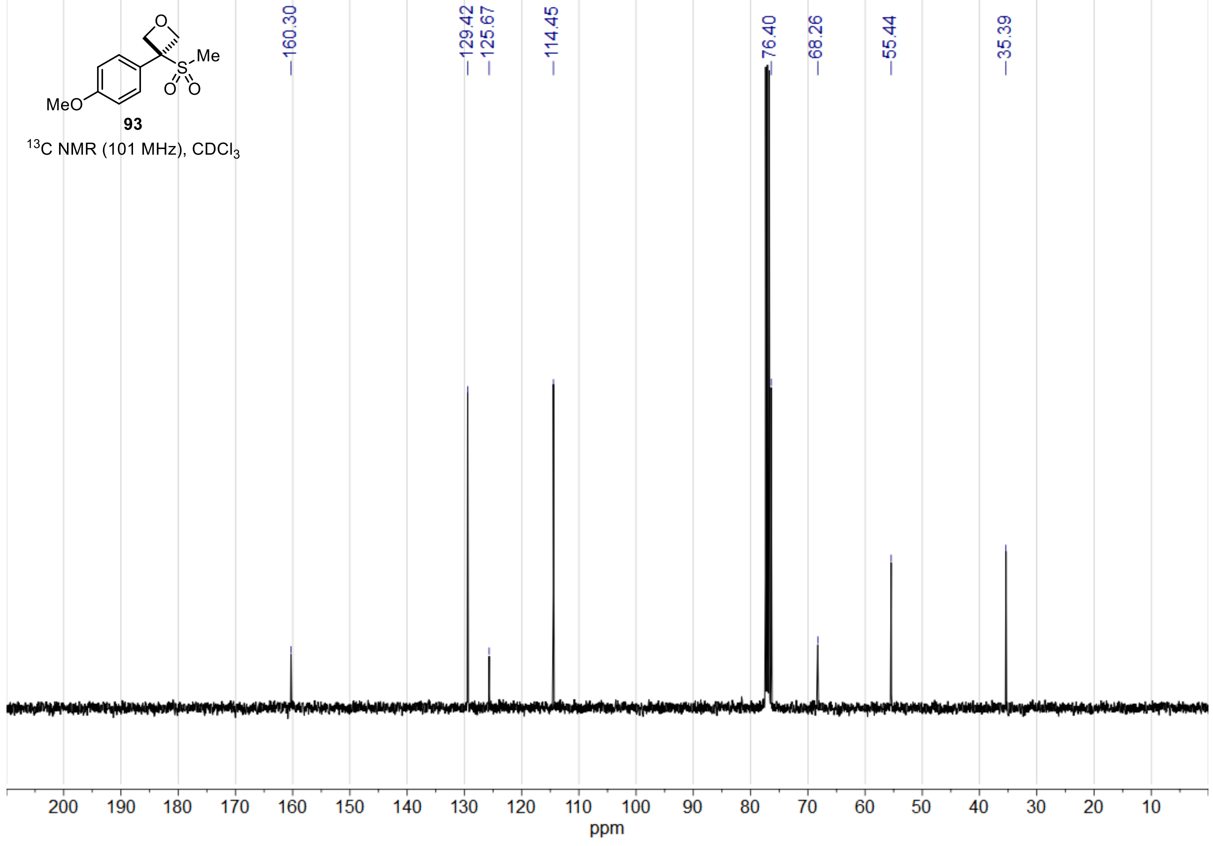

MeO
Me
93
13C NMR (101 MHz), CDCl3
160.30
129.42
125.67
114.45
76.40
68.26
55.44
35.39
ppm

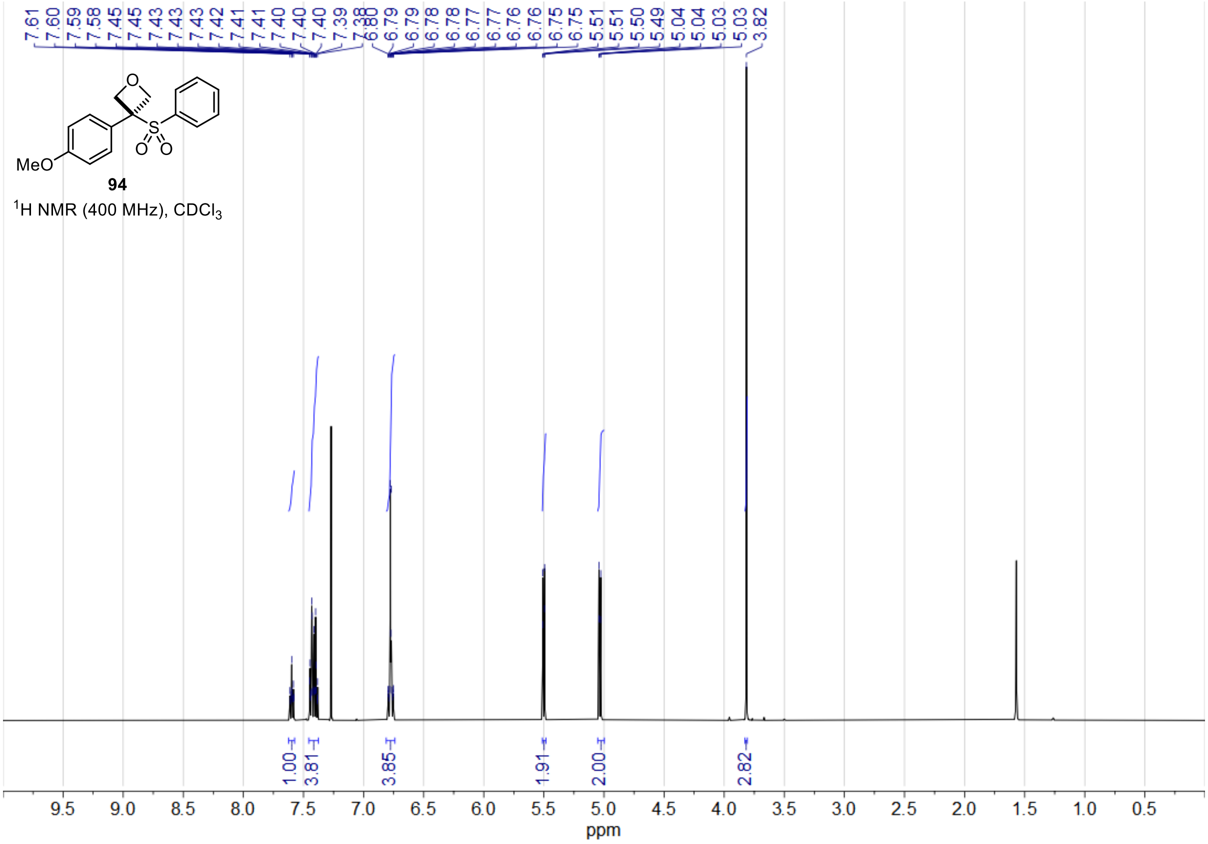
7.61
7.60
7.59
7.58
7.45
7.45
7.43
7.43
7.43
7.42
7.41
7.41
7.40
7.40
7.40
7.39
7.38
6.80
6.79
6.79
6.78
6.78
6.77
6.77
6.76
6.76
6.75
6.75
5.51
5.51
5.50
5.49
5.04
5.04
5.03
5.03
3.82
MeO
O
S
O
O
94
1H NMR (400 MHz), CDCl3
1.00
3.81
3.85
1.91
2.00
2.82
9.5
9.0
8.5
8.0
7.5
7.0
6.5
6.0
5.5
5.0
4.5
4.0
3.5
3.0
2.5
2.0
1.5
1.0
0.5
ppm

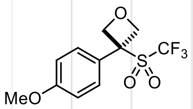

95

^{1}H NMR (400 MHz), CD_3CN

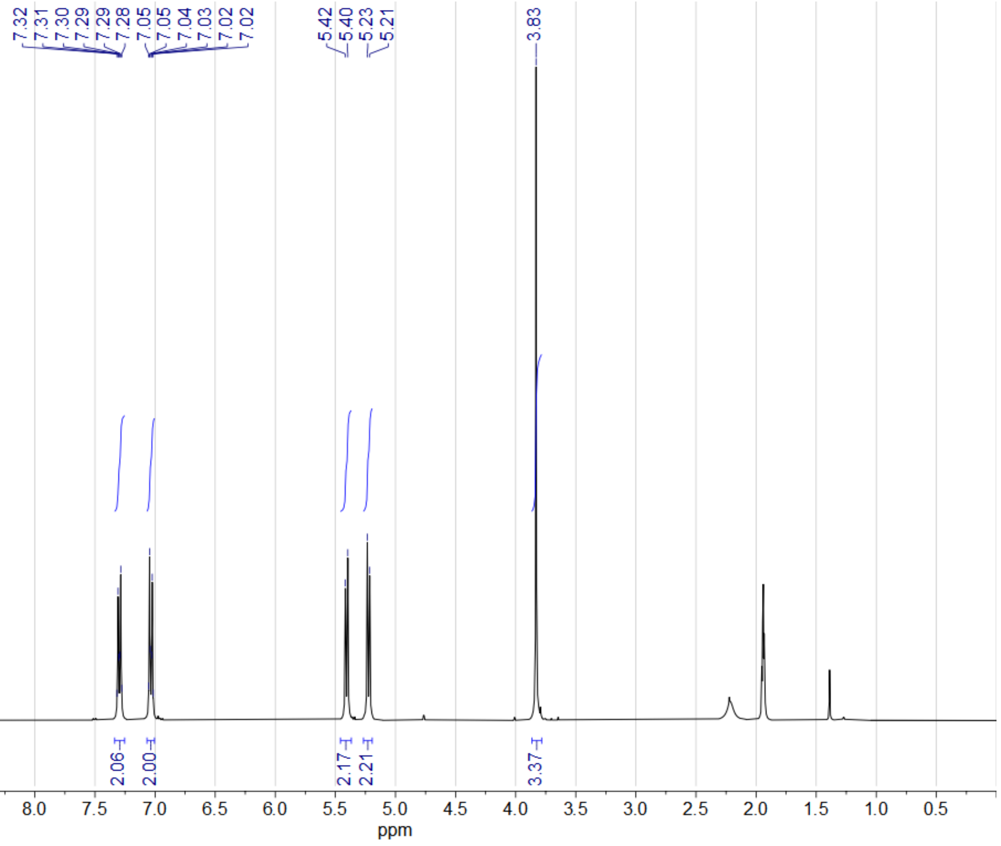

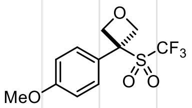

95

^{13}C NMR (101 MHz), CD_3CN

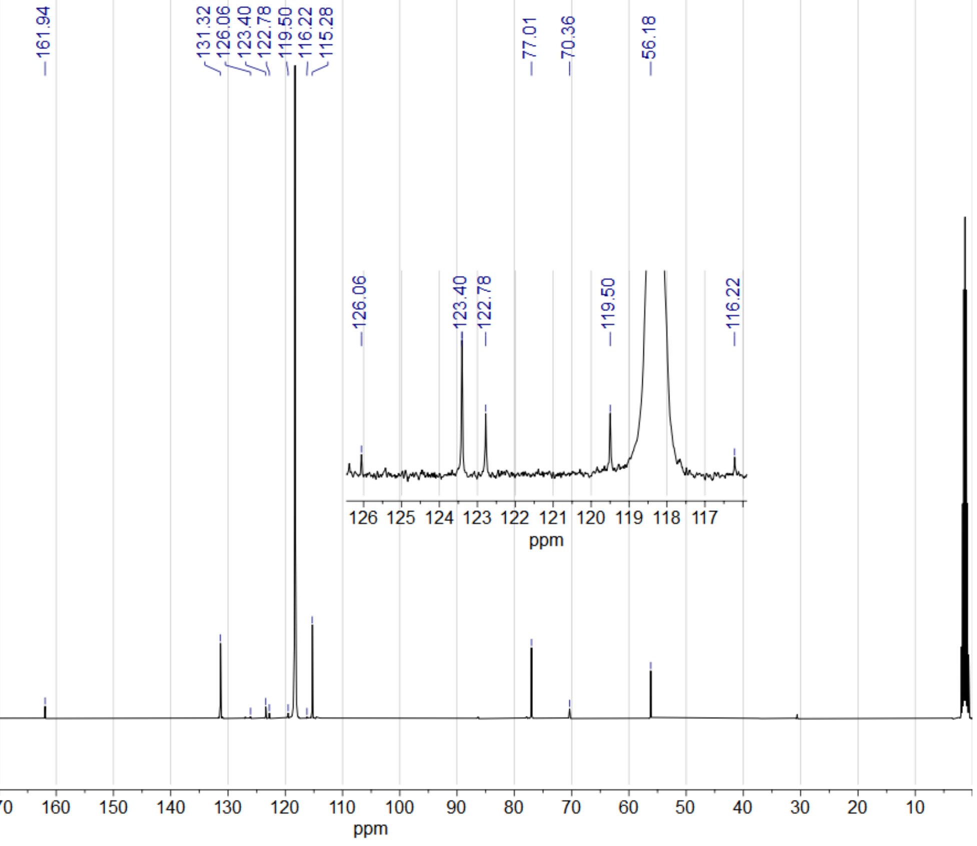

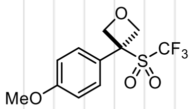

95

$^{19}F\{^{1}H\}$ NMR (377 MHz), CD_3CN

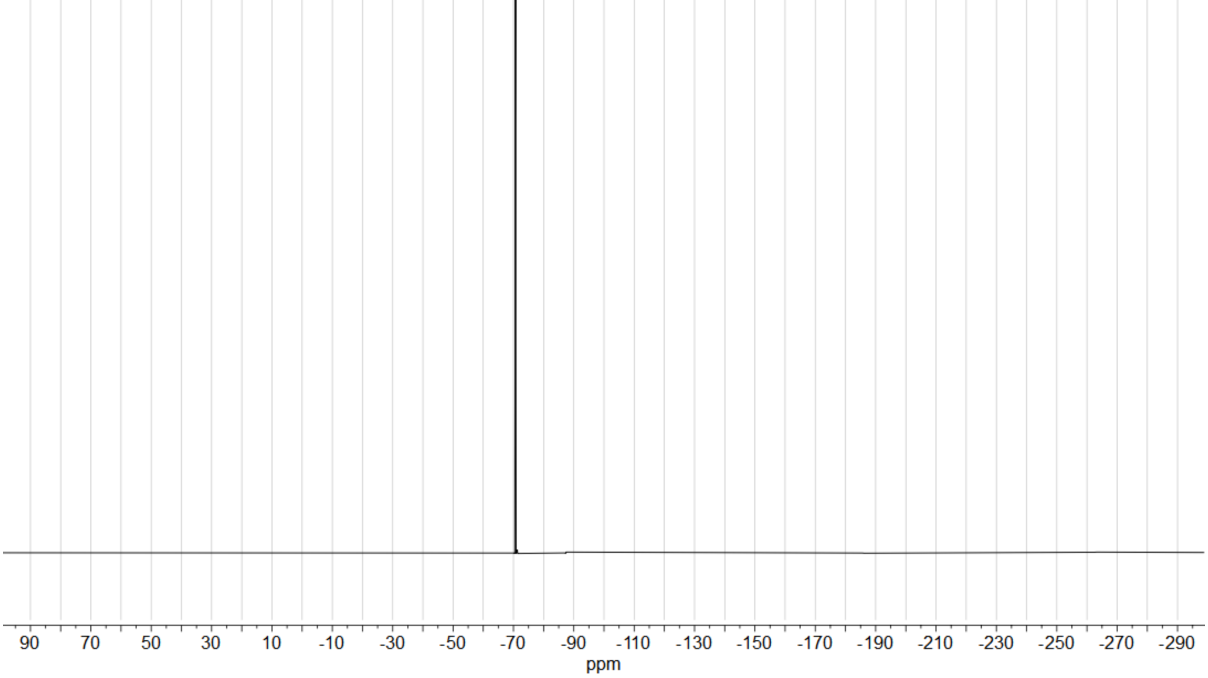

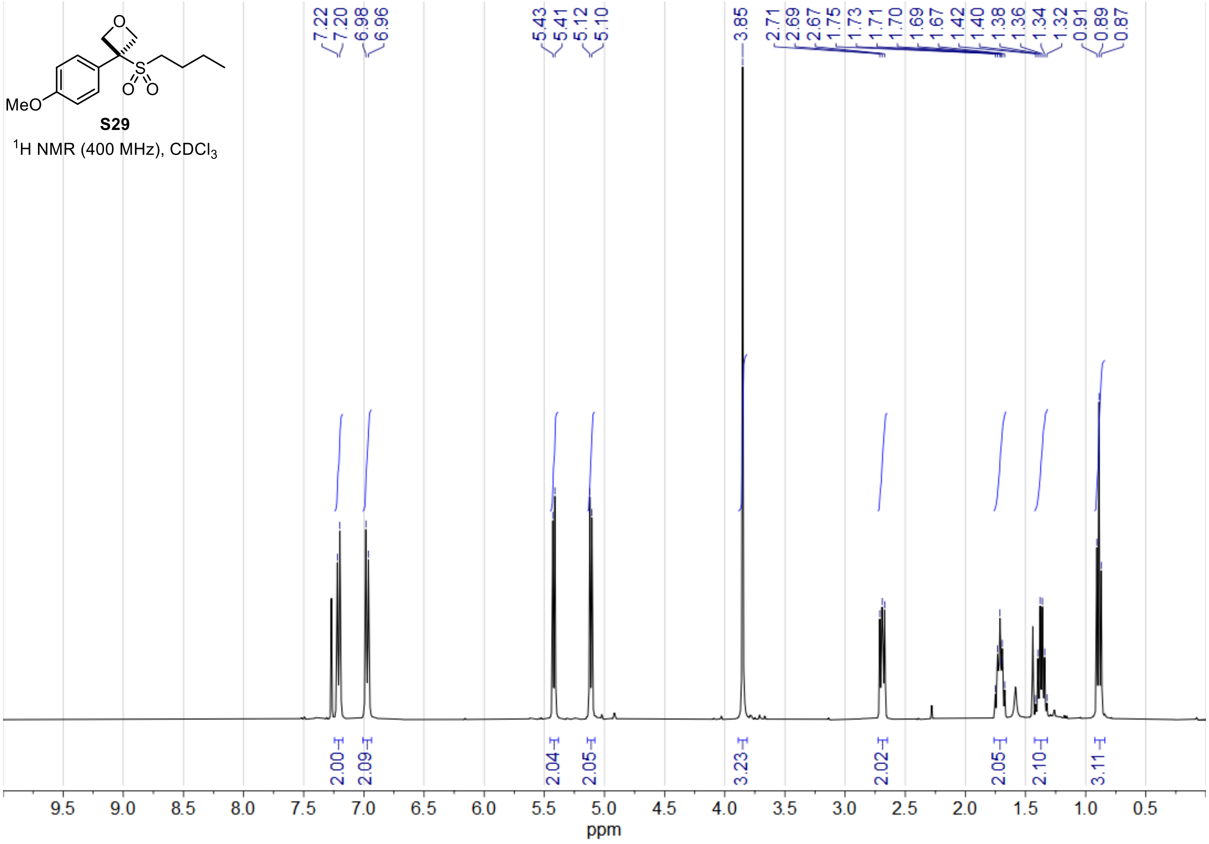

MeO
O
S
O O
S29
^{1}H NMR (400 MHz), CDCl$_3$
7.22
7.20
6.98
6.96
5.43
5.41
5.12
5.10
3.85
2.71
2.69
2.67
1.75
1.73
1.71
1.70
1.69
1.67
1.42
1.40
1.38
1.36
1.34
1.32
0.91
0.89
0.87
2.00
2.09
2.04
2.05
3.23
2.02
2.05
2.10
3.11
9.5 9.0 8.5 8.0 7.5 7.0 6.5 6.0 5.5 5.0 4.5 4.0 3.5 3.0 2.5 2.0 1.5 1.0 0.5
ppm

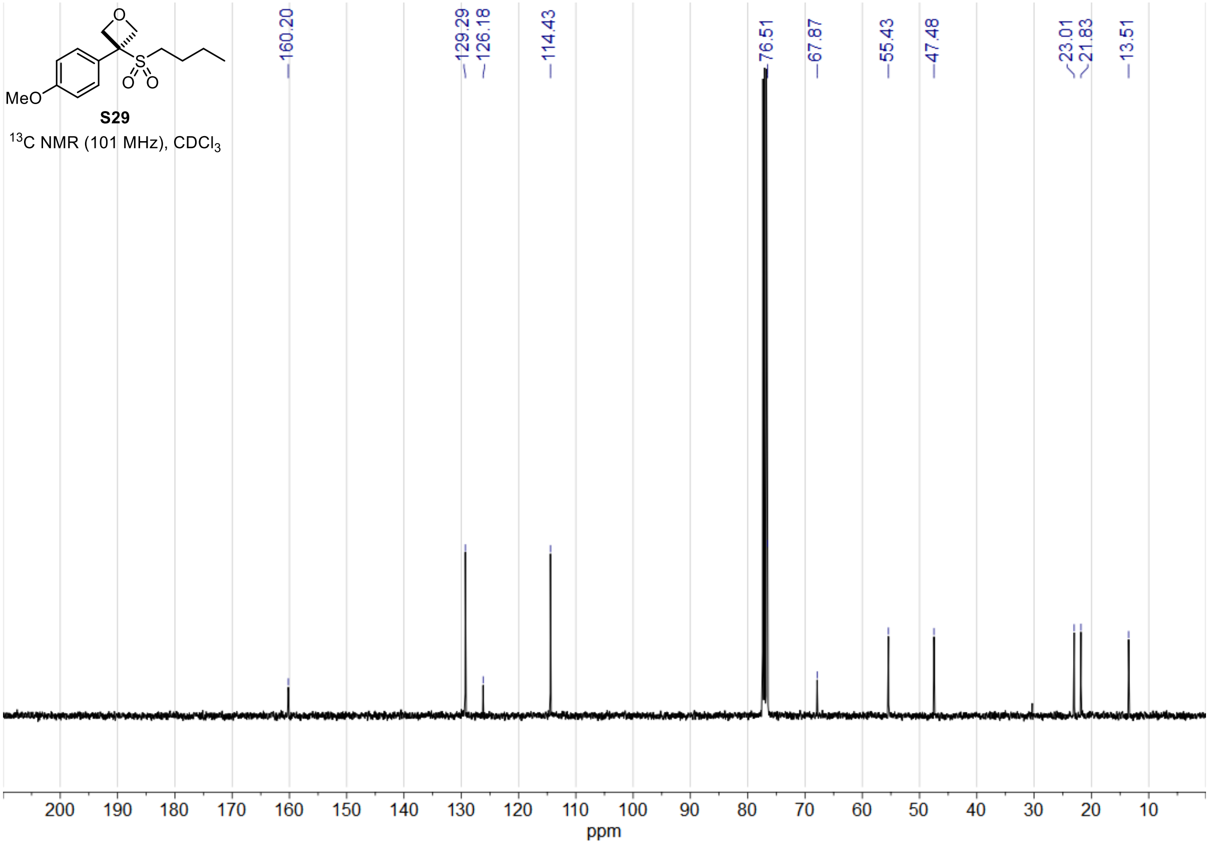

MeO
O
S
O O
S29
^{13}C NMR (101 MHz), CDCl$_3$
160.20
129.29
126.18
114.43
76.51
67.87
55.43
47.48
23.01
21.83
13.51
200 190 180 170 160 150 140 130 120 110 100 90 80 70 60 50 40 30 20 10
ppm

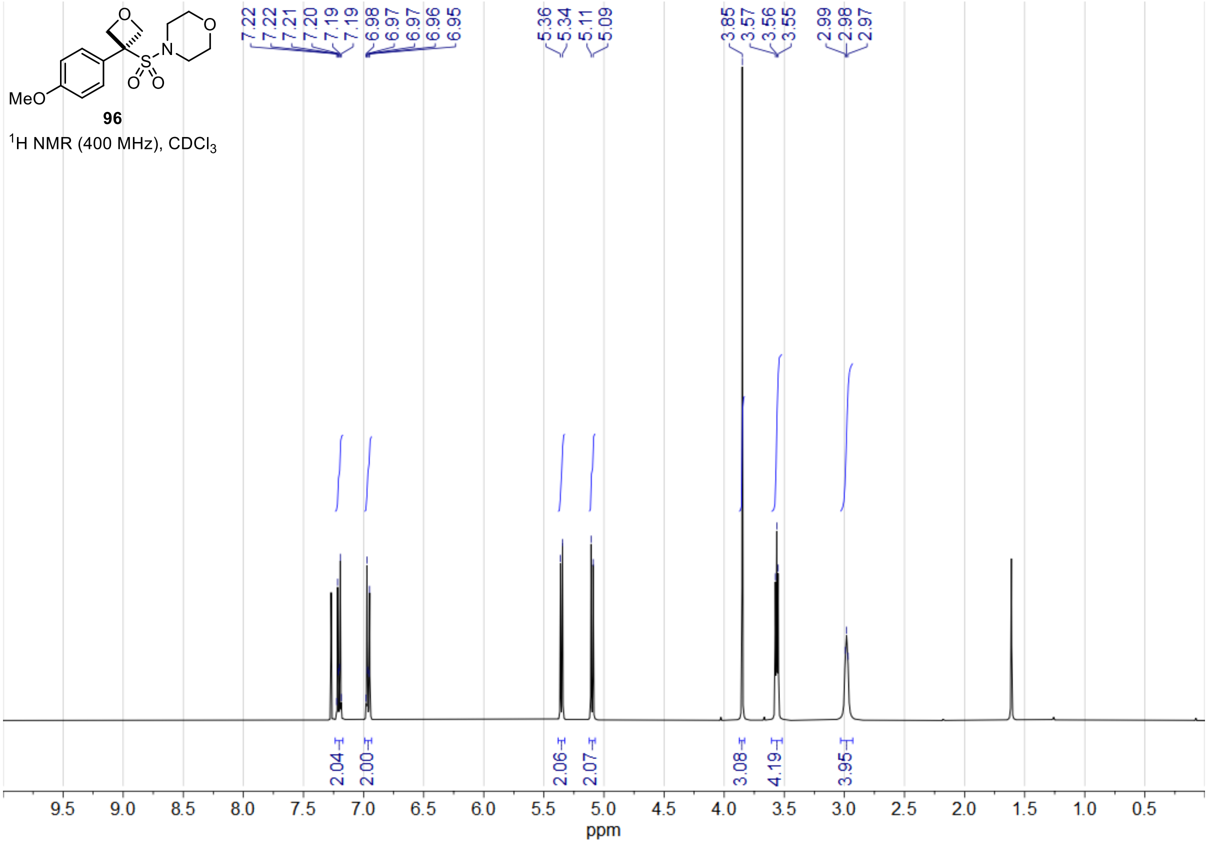
96
1H NMR (400 MHz), CDCl3
7.22
7.22
7.21
7.20
7.19
7.19
6.98
6.97
6.97
6.96
6.95
5.36
5.34
5.11
5.09
3.85
3.57
3.56
3.55
2.99
2.98
2.97
2.04
2.00
2.06
2.07
3.08
4.19
3.95
ppm

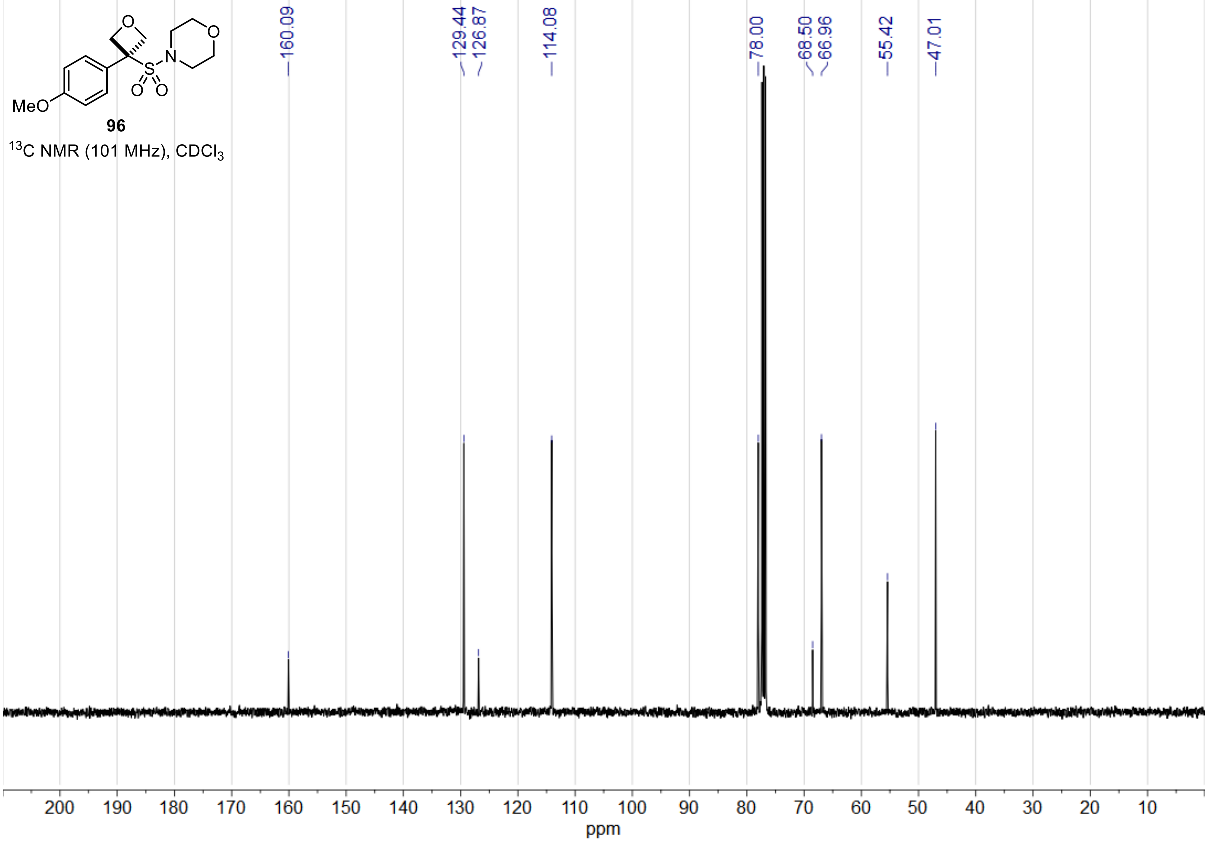
96
13C NMR (101 MHz), CDCl3
160.09
129.44
126.87
114.08
78.00
68.50
66.96
55.42
47.01
ppm

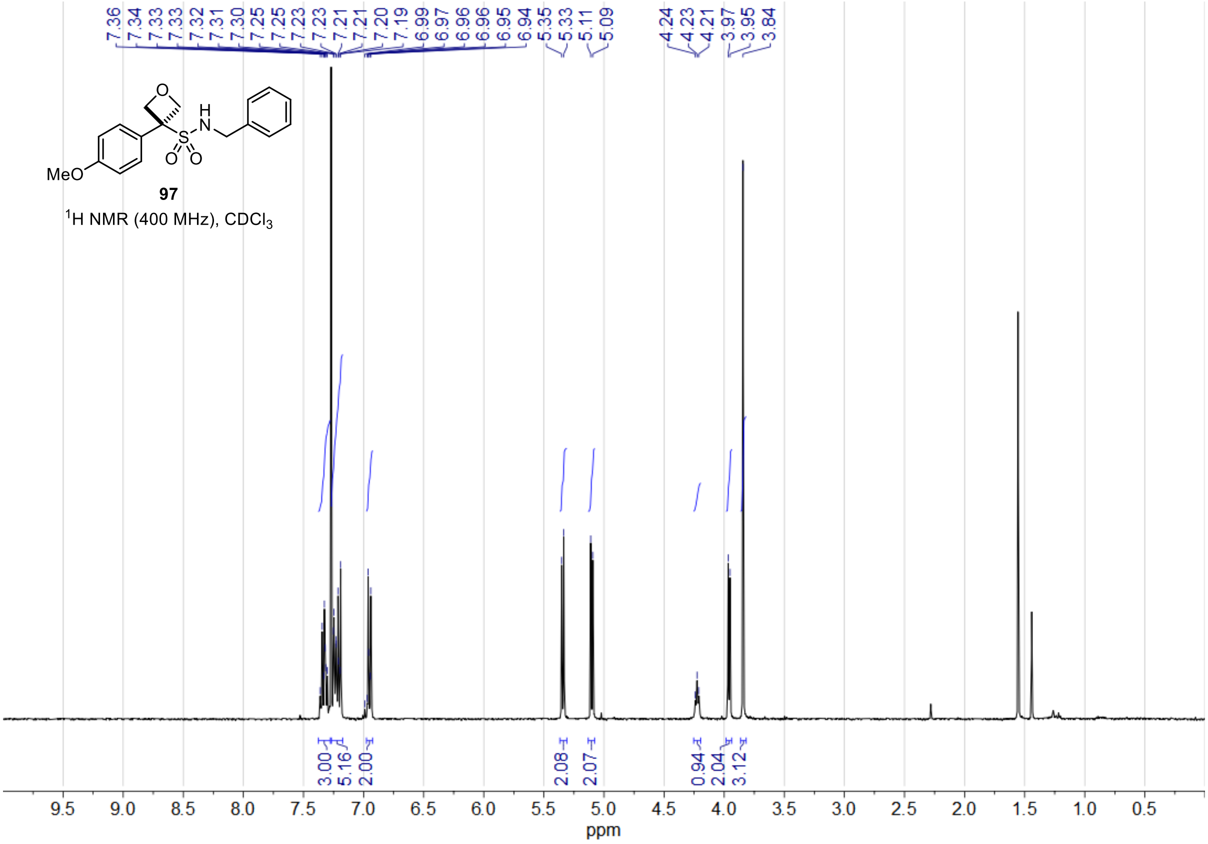
7.36
7.34
7.33
7.33
7.32
7.31
7.30
7.25
7.25
7.23
7.23
7.21
7.21
7.20
7.19
6.99
6.97
6.96
6.96
6.95
6.94
5.35
5.33
5.11
5.09
4.24
4.23
4.21
3.97
3.95
3.84
MeO
H
N
O
S
O
O
97
1H NMR (400 MHz), CDCl3
3.00
5.16
2.00
2.08
2.07
0.94
2.04
3.12
9.5 9.0 8.5 8.0 7.5 7.0 6.5 6.0 5.5 5.0 4.5 4.0 3.5 3.0 2.5 2.0 1.5 1.0 0.5
ppm

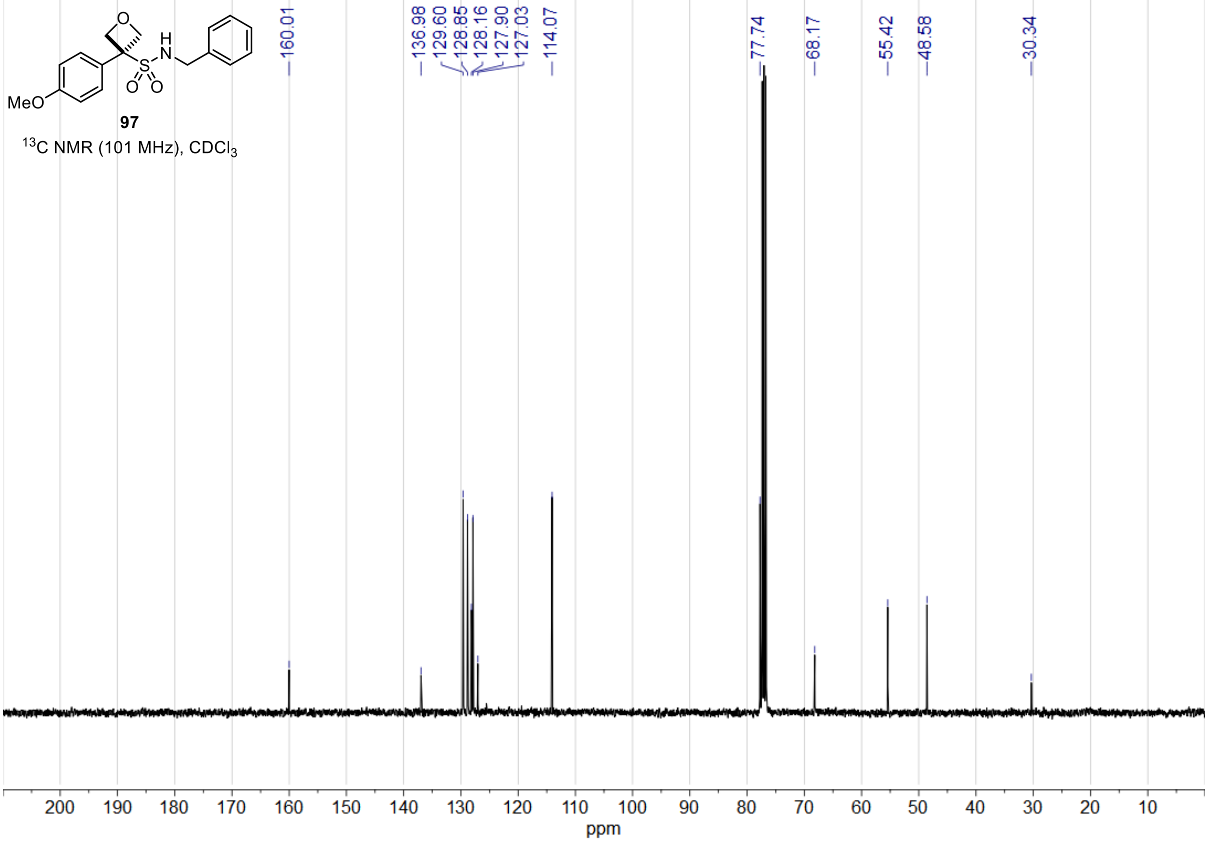
160.01
136.98
129.60
128.85
128.16
127.90
127.03
114.07
77.74
68.17
55.42
48.58
30.34
MeO
H
N
O
S
O
O
97
13C NMR (101 MHz), CDCl3
200 190 180 170 160 150 140 130 120 110 100 90 80 70 60 50 40 30 20 10
ppm

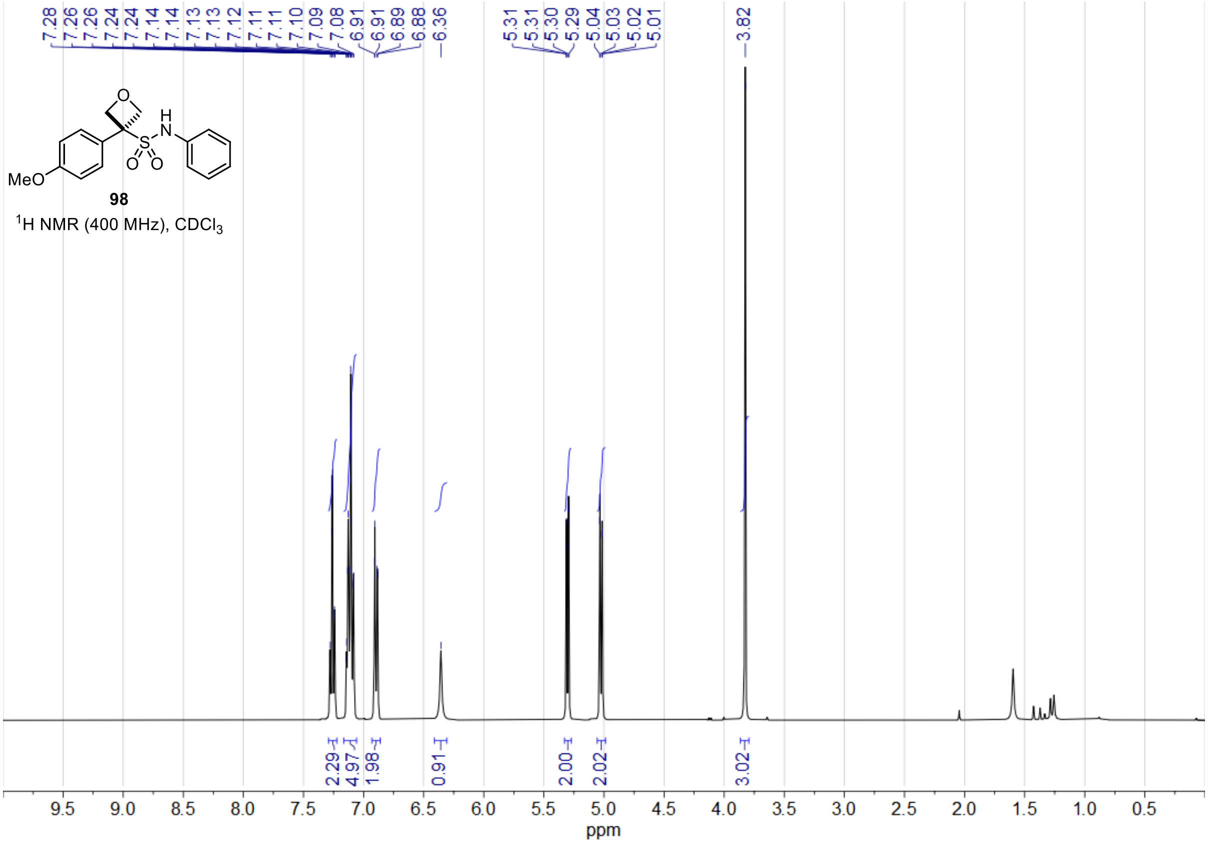

98

^{1}H NMR (400 MHz), $CDCl_3$

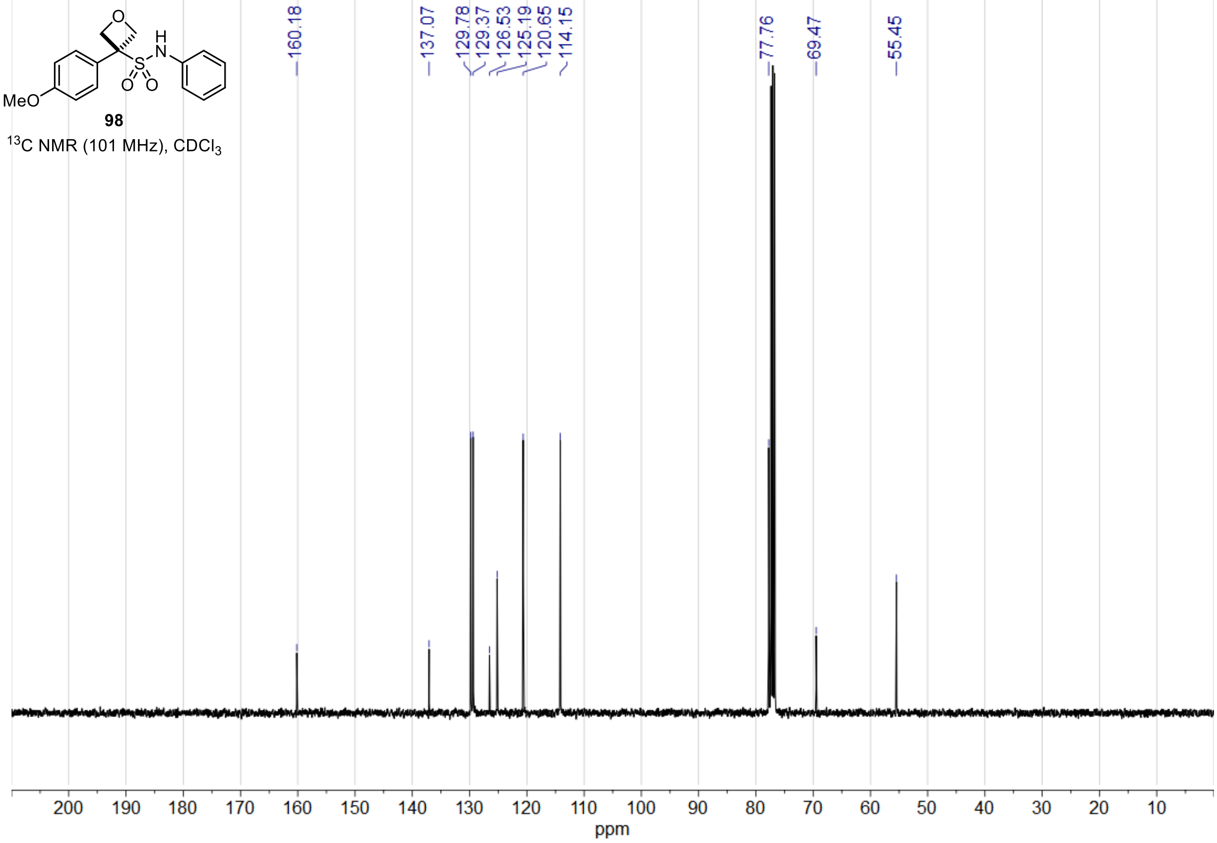

98

^{13}C NMR (101 MHz), $CDCl_3$

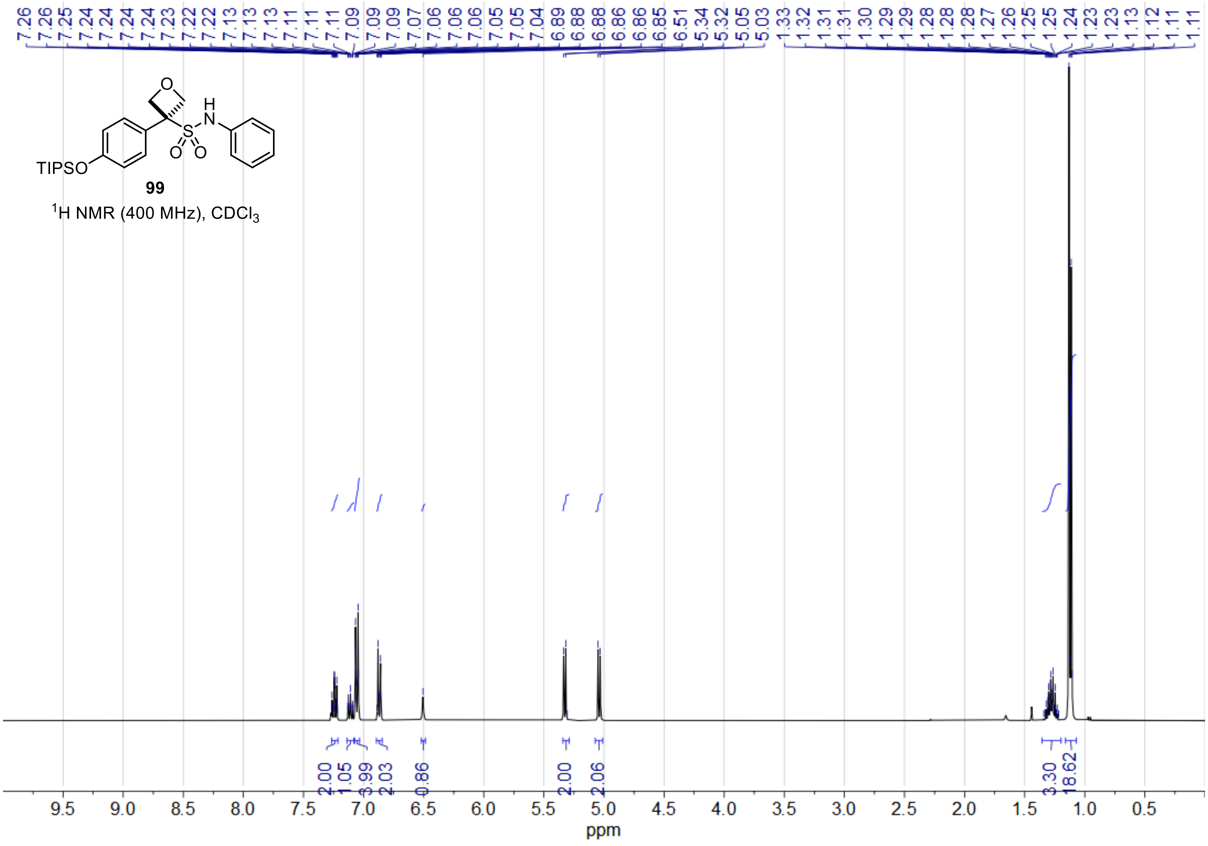

TIPSO
99
^{1}H NMR (400 MHz), CDCl3
7.26 7.26 7.25 7.24 7.24 7.24 7.24 7.23 7.22 7.22 7.13 7.13 7.11 7.11 7.11 7.09 7.09 7.09 7.07 7.06 7.06 7.06 7.05 7.05 7.04 6.89 6.88 6.88 6.86 6.86 6.85 6.51 5.34 5.32 5.05 5.03 1.33 1.32 1.31 1.31 1.30 1.29 1.29 1.28 1.28 1.28 1.27 1.26 1.25 1.25 1.24 1.24 1.23 1.23 1.13 1.12 1.11 1.11
2.00 1.05 3.99 2.03 0.86 2.00 2.06 3.30 18.62
ppm

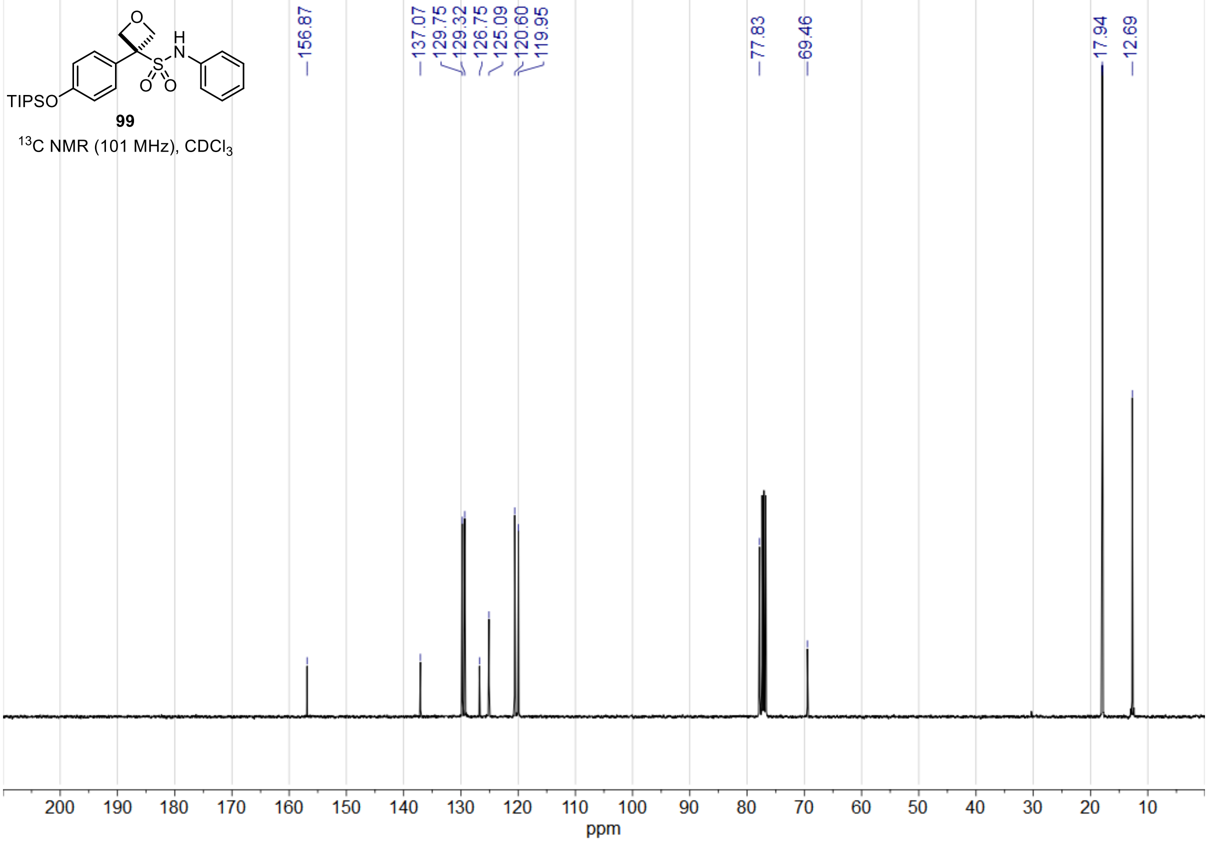

TIPSO
99
^{13}C NMR (101 MHz), CDCl3
156.87 137.07 129.75 129.32 126.75 125.09 120.60 119.95 77.83 69.46 17.94 12.69
ppm

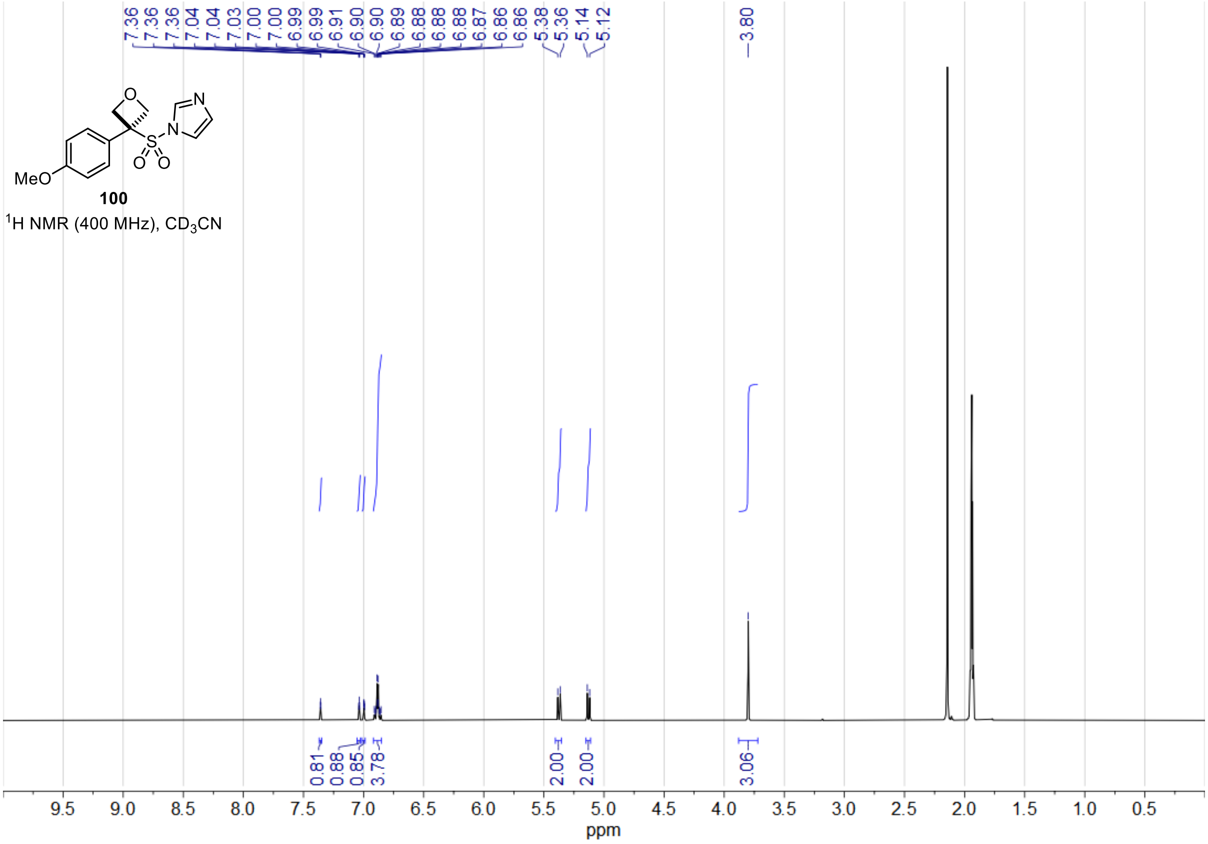

100

^{1}H NMR (400 MHz), CD_3CN

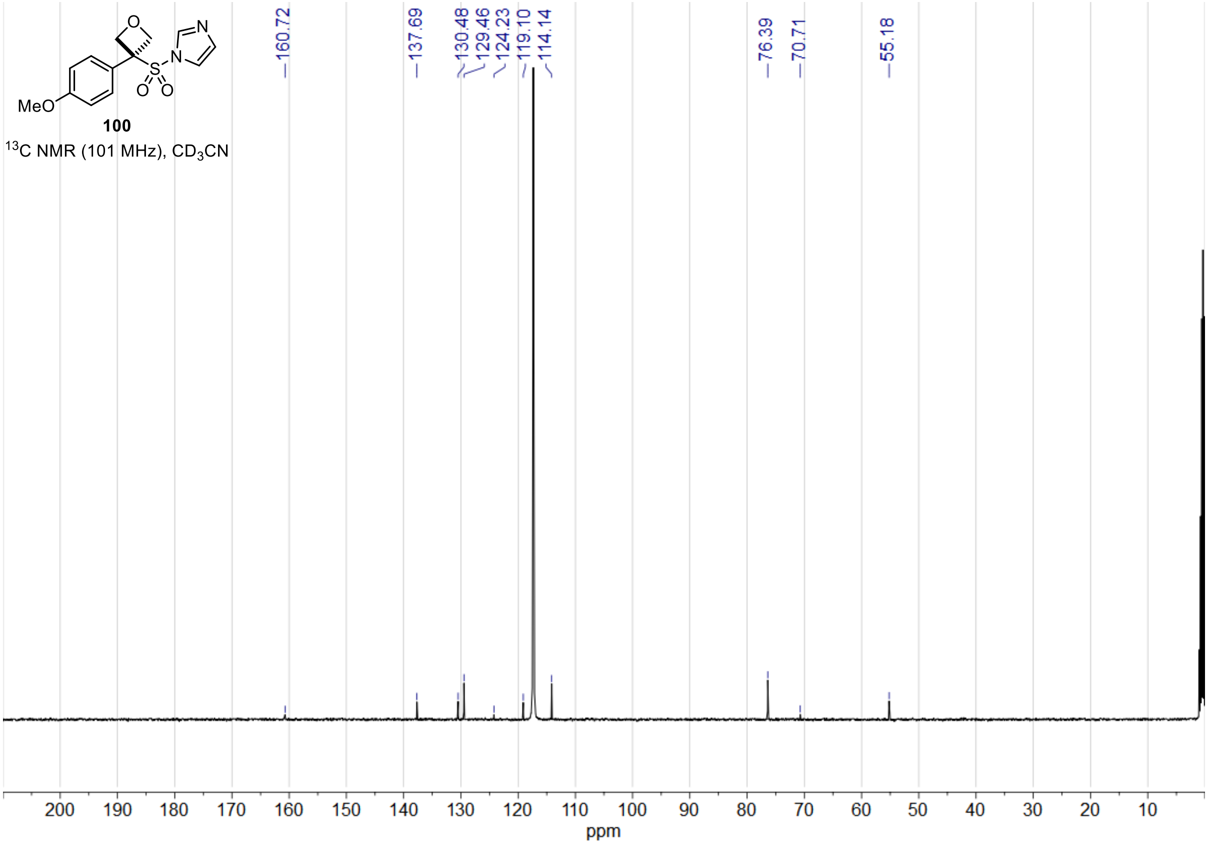

100

^{13}C NMR (101 MHz), CD_3CN

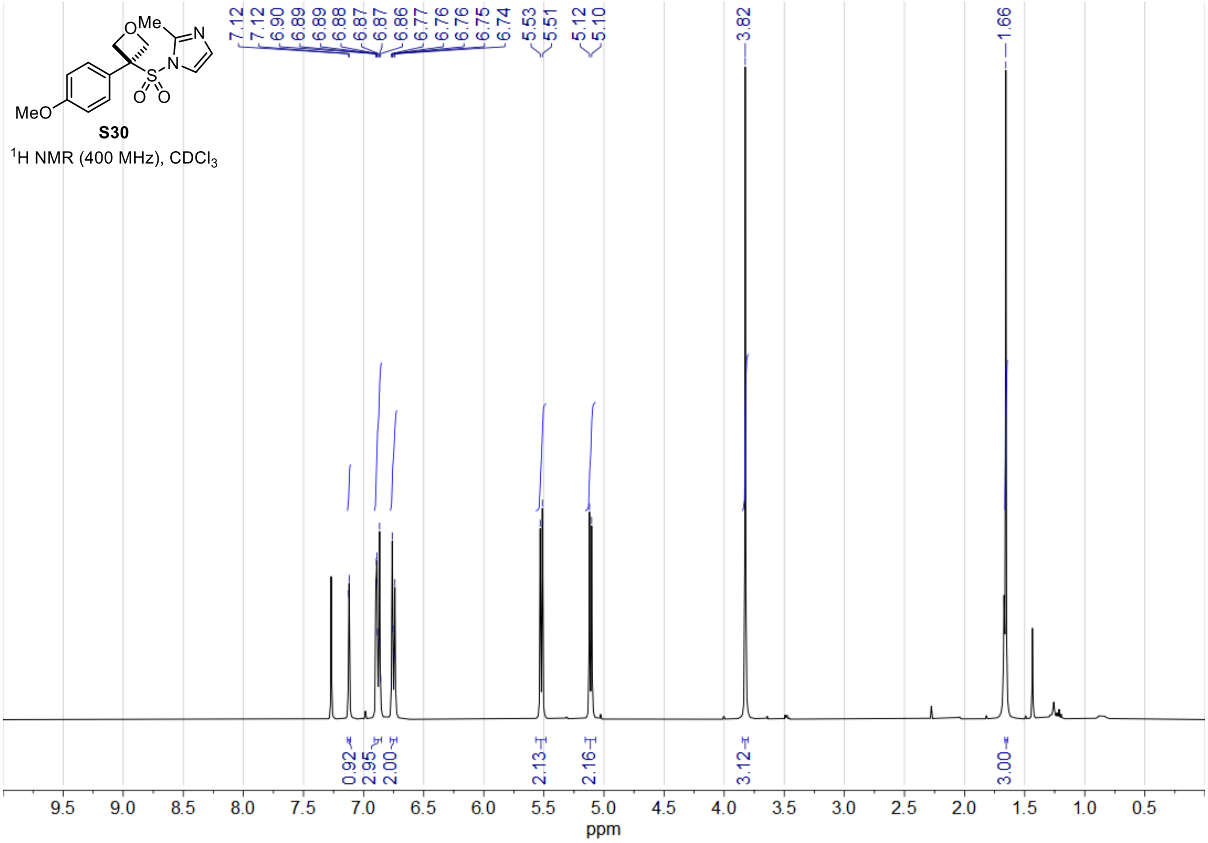
S30
^{1}H NMR (400 MHz), CDCl$_3$
7.12
7.12
6.90
6.89
6.89
6.88
6.87
6.87
6.86
6.77
6.76
6.76
6.75
6.74
5.53
5.51
5.12
5.10
3.82
1.66
0.92
2.95
2.00
2.13
2.16
3.12
3.00
ppm

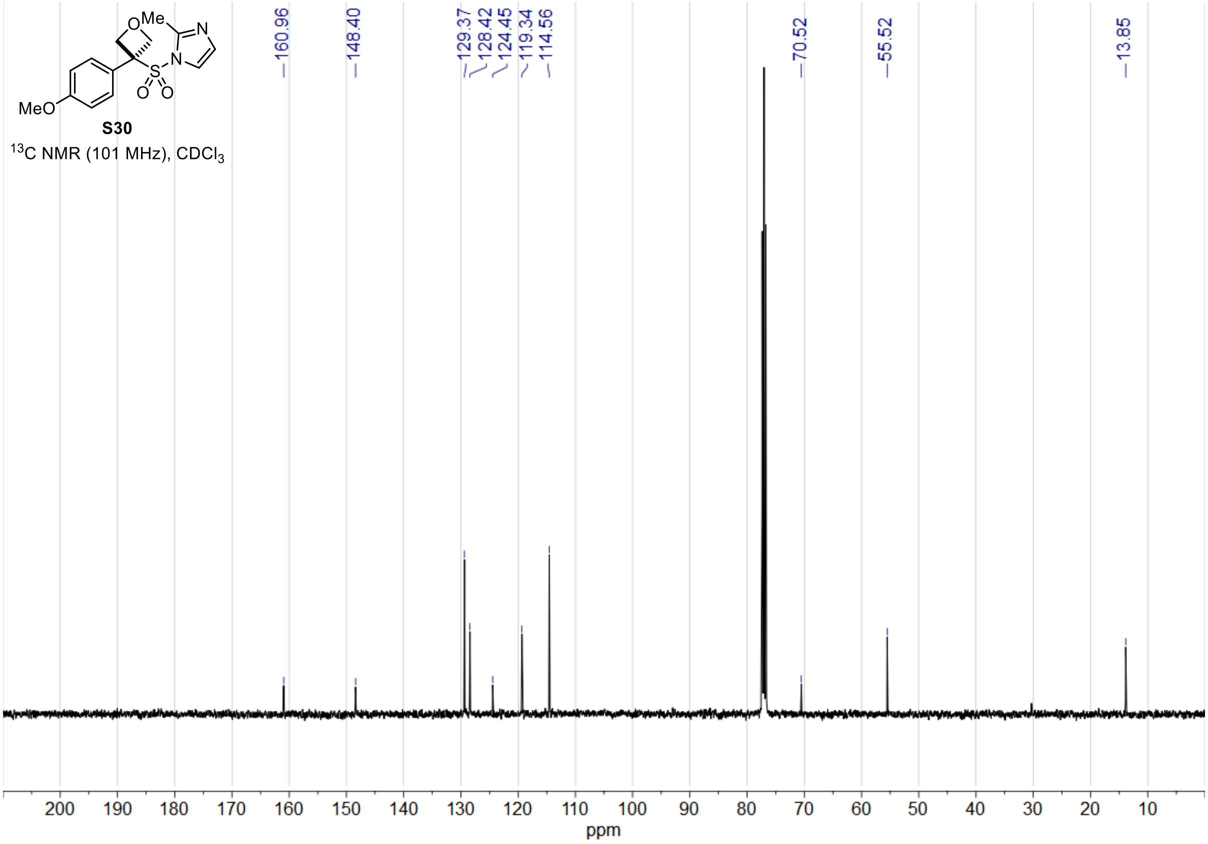
S30
^{13}C NMR (101 MHz), CDCl$_3$
160.96
148.40
129.37
128.42
124.45
119.34
114.56
70.52
55.52
13.85
ppm

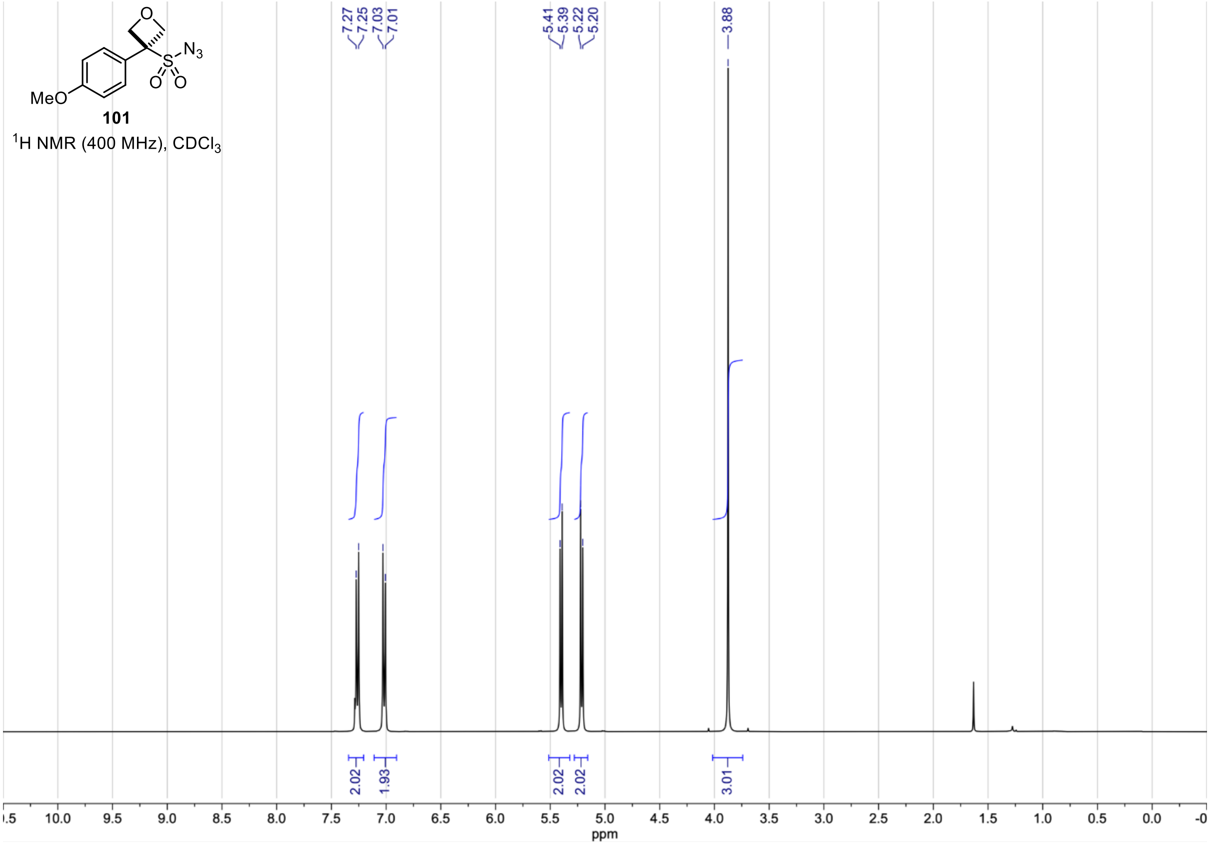
7.27
7.25
7.03
7.01
5.41
5.39
5.22
5.20
3.88
MeO
N3
101
1H NMR (400 MHz), CDCl3
2.02
1.93
2.02
2.02
3.01
ppm

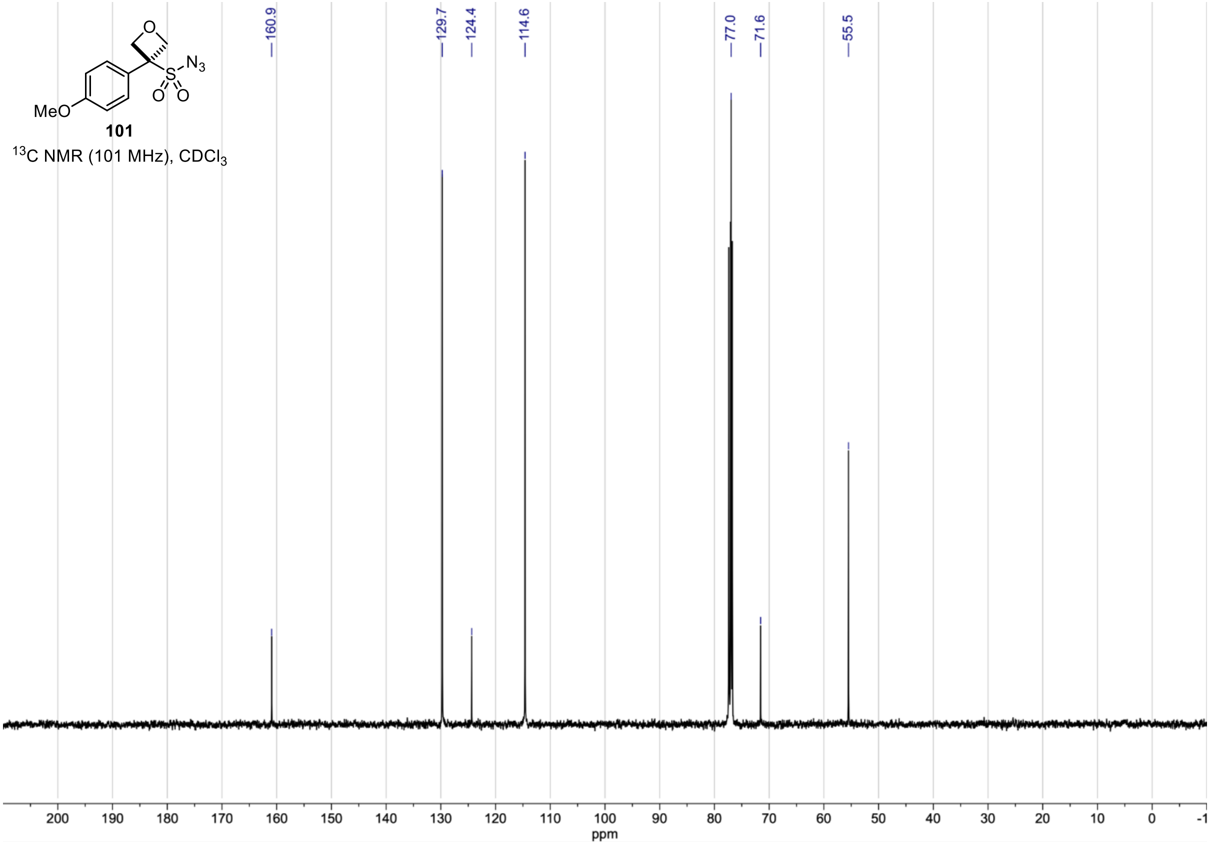
160.9
129.7
124.4
114.6
77.0
71.6
55.5
MeO
N3
101
13C NMR (101 MHz), CDCl3
ppm

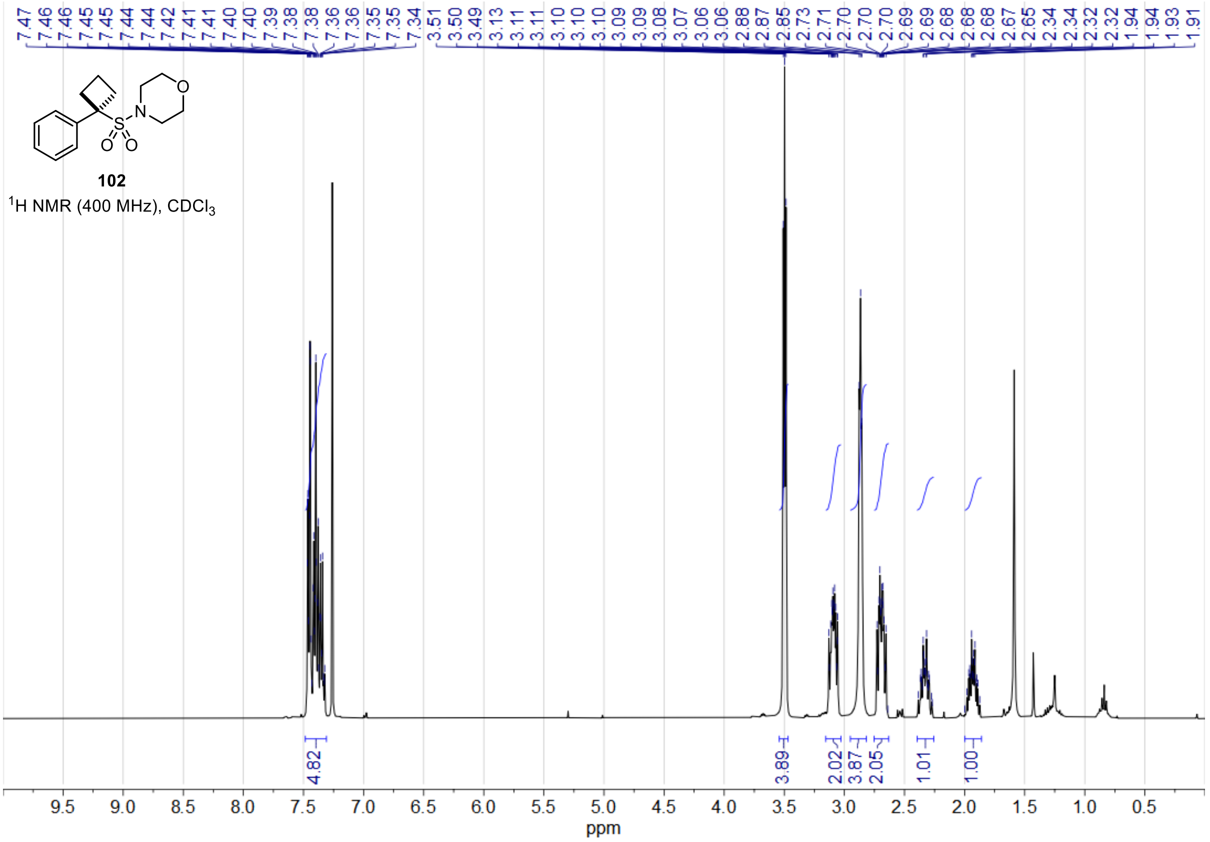
7.47 7.46 7.46 7.45 7.45 7.44 7.44 7.42 7.41 7.41 7.40 7.40 7.39 7.38 7.38 7.36 7.36 7.35 7.35 7.34 3.51 3.50 3.49 3.13 3.11 3.11 3.10 3.10 3.09 3.09 3.08 3.07 3.06 3.06 2.88 2.87 2.85 2.73 2.71 2.70 2.70 2.70 2.69 2.69 2.68 2.68 2.68 2.67 2.65 2.34 2.34 2.32 2.32 1.94 1.94 1.93 1.91
102
^{1}H NMR (400 MHz), CDCl$_3$
4.82
3.89
2.02
3.87
2.05
1.01
1.00
ppm

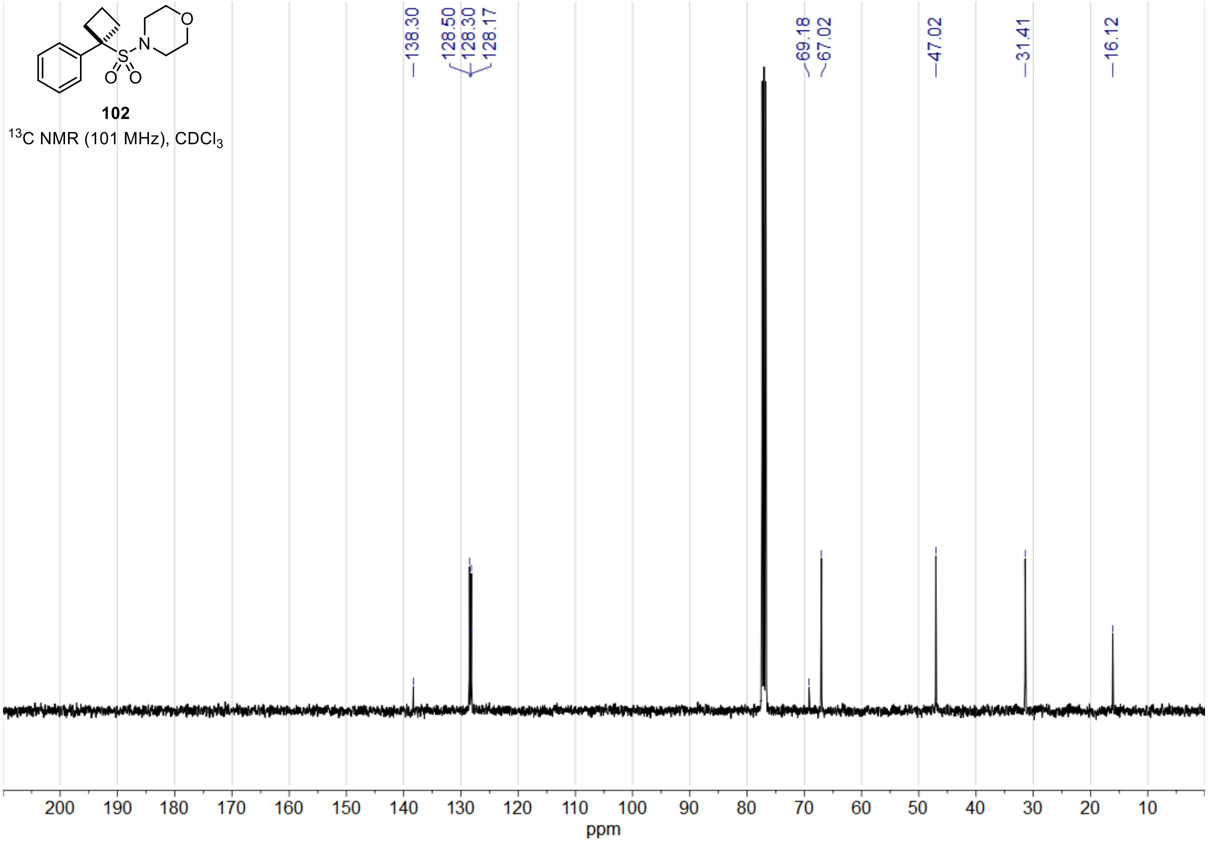
138.30
128.50 128.30 128.17
69.18 67.02
47.02
31.41
16.12
102
^{13}C NMR (101 MHz), CDCl$_3$
ppm

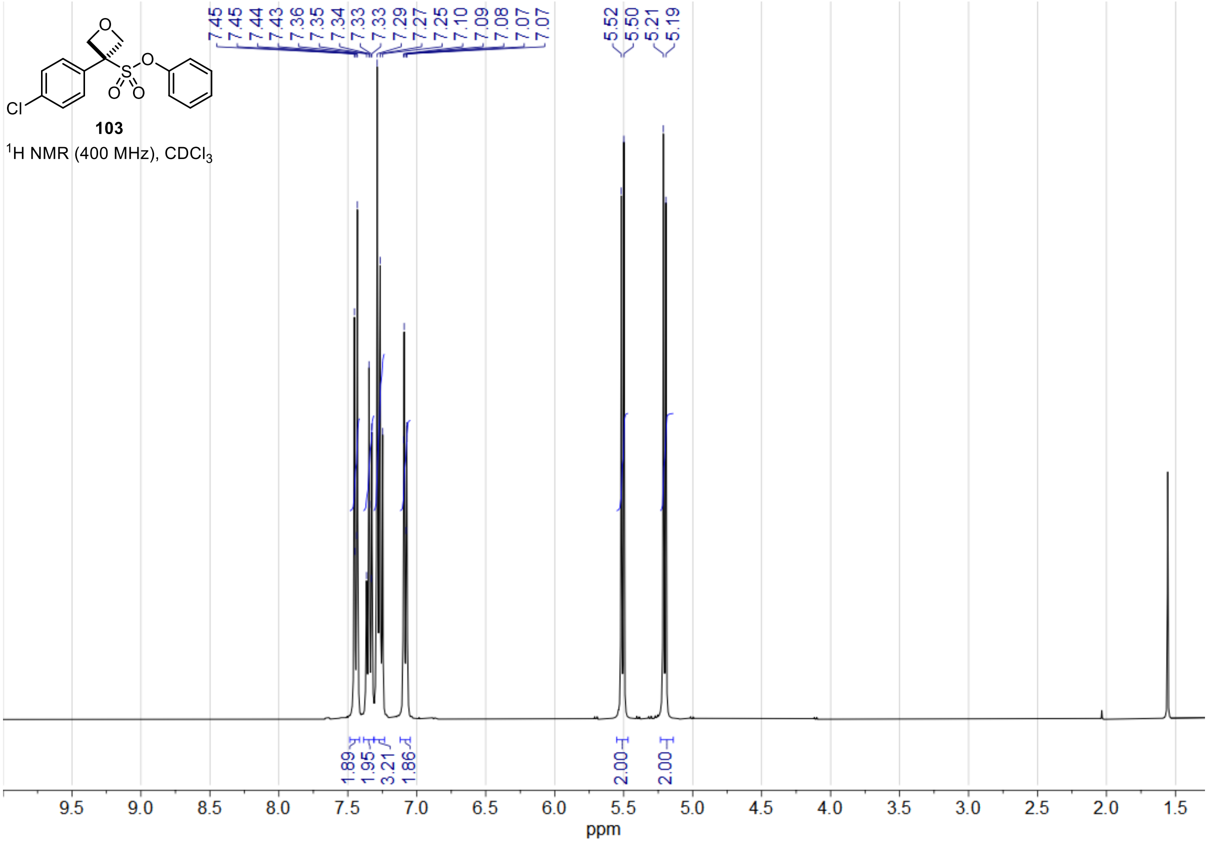
103
¹H NMR (400 MHz), CDCl3
7.45 7.45 7.44 7.43 7.36 7.35 7.34 7.33 7.33 7.29 7.27 7.25 7.10 7.09 7.08 7.07 7.07
5.52 5.50 5.21 5.19
1.89 1.95 3.21 1.86
2.00 2.00
9.5 9.0 8.5 8.0 7.5 7.0 6.5 6.0 5.5 5.0 4.5 4.0 3.5 3.0 2.5 2.0 1.5
ppm

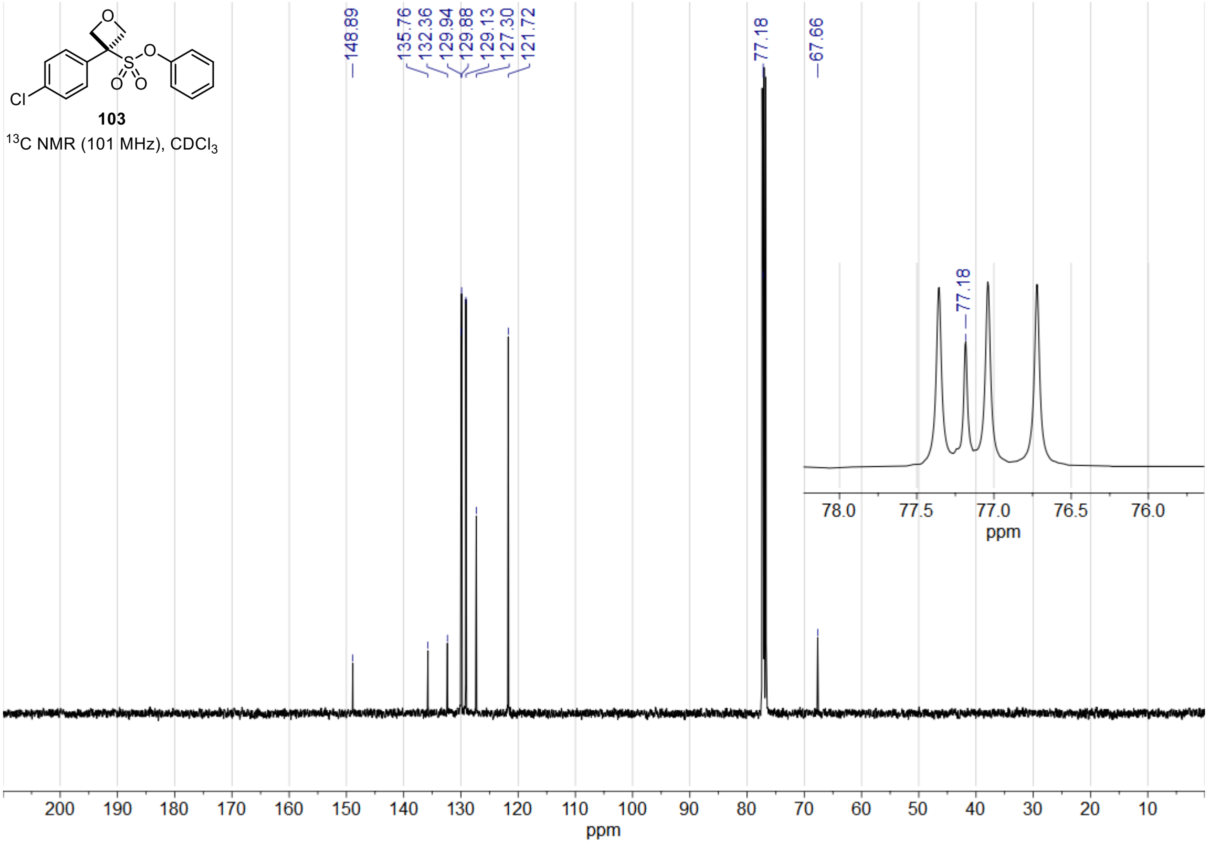
103
¹³C NMR (101 MHz), CDCl3
148.89
135.76 132.36 129.94 129.88 129.13 127.30 121.72
77.18
67.66
77.18
78.0 77.5 77.0 76.5 76.0
ppm
200 190 180 170 160 150 140 130 120 110 100 90 80 70 60 50 40 30 20 10
ppm

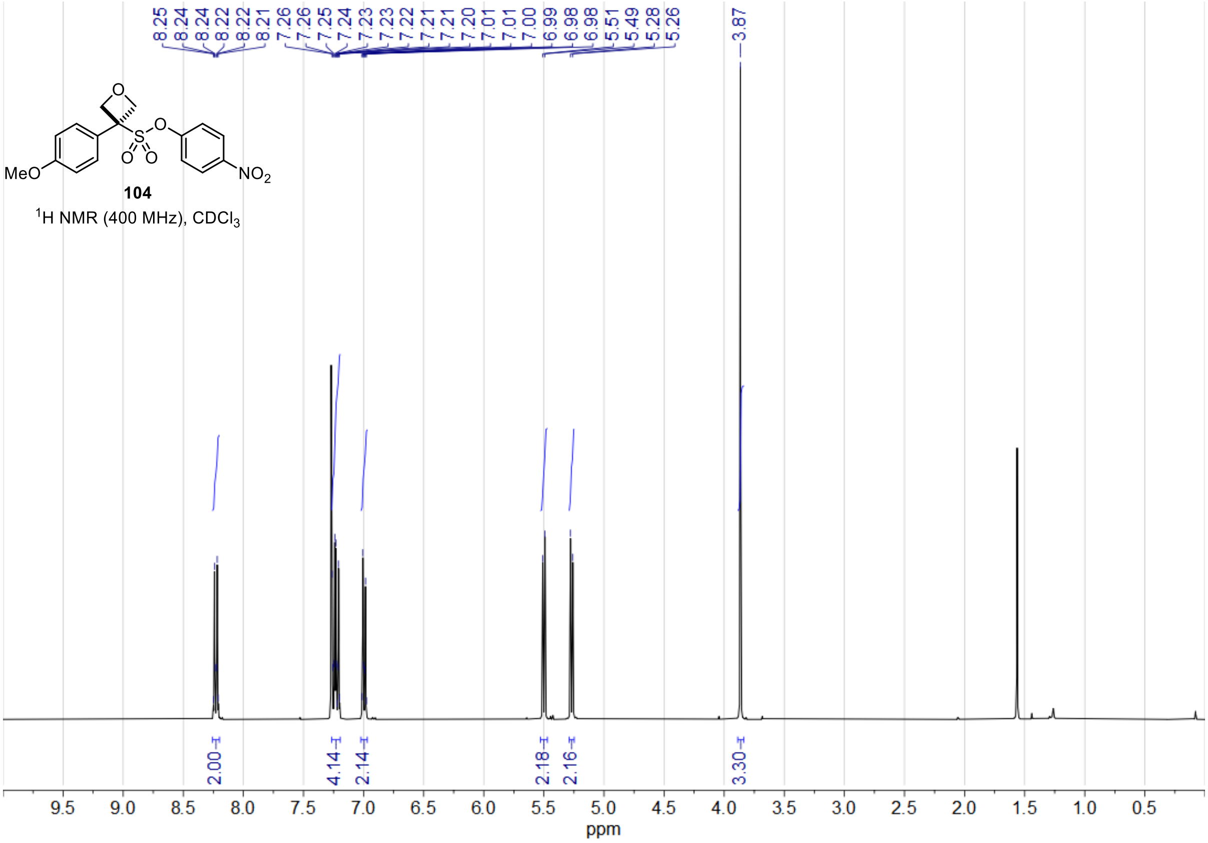
8.25
8.24
8.24
8.22
8.22
8.21
7.26
7.26
7.25
7.24
7.23
7.23
7.22
7.21
7.21
7.20
7.01
7.01
7.00
6.99
6.98
6.98
5.51
5.49
5.28
5.26
3.87
O
S
O
O O
MeO
NO2
104
1H NMR (400 MHz), CDCl3
2.00
4.14
2.14
2.18
2.16
3.30
9.5 9.0 8.5 8.0 7.5 7.0 6.5 6.0 5.5 5.0 4.5 4.0 3.5 3.0 2.5 2.0 1.5 1.0 0.5
ppm

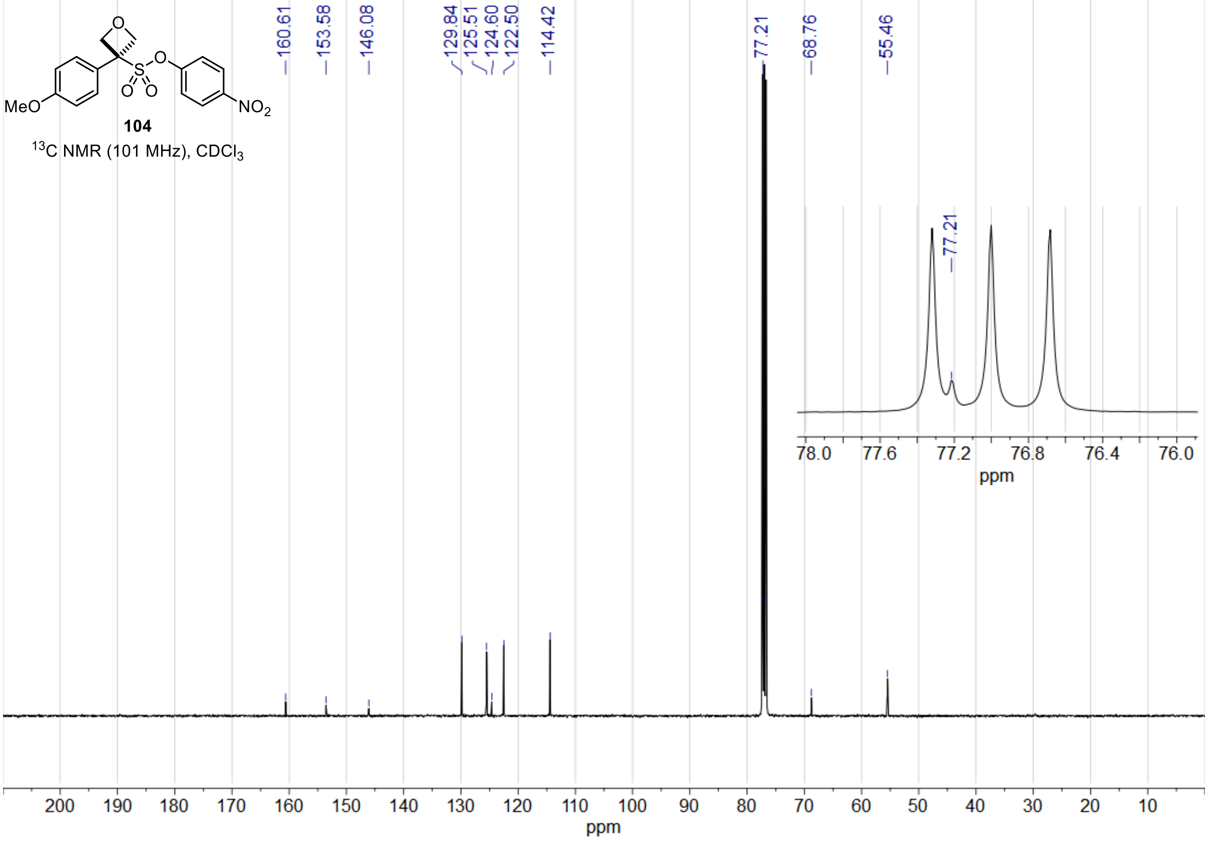
160.61
153.58
146.08
129.84
125.51
124.60
122.50
114.42
77.21
68.76
55.46
O
S
O
O O
MeO
NO2
104
13C NMR (101 MHz), CDCl3
77.21
78.0 77.6 77.2 76.8 76.4 76.0
ppm
200 190 180 170 160 150 140 130 120 110 100 90 80 70 60 50 40 30 20 10
ppm

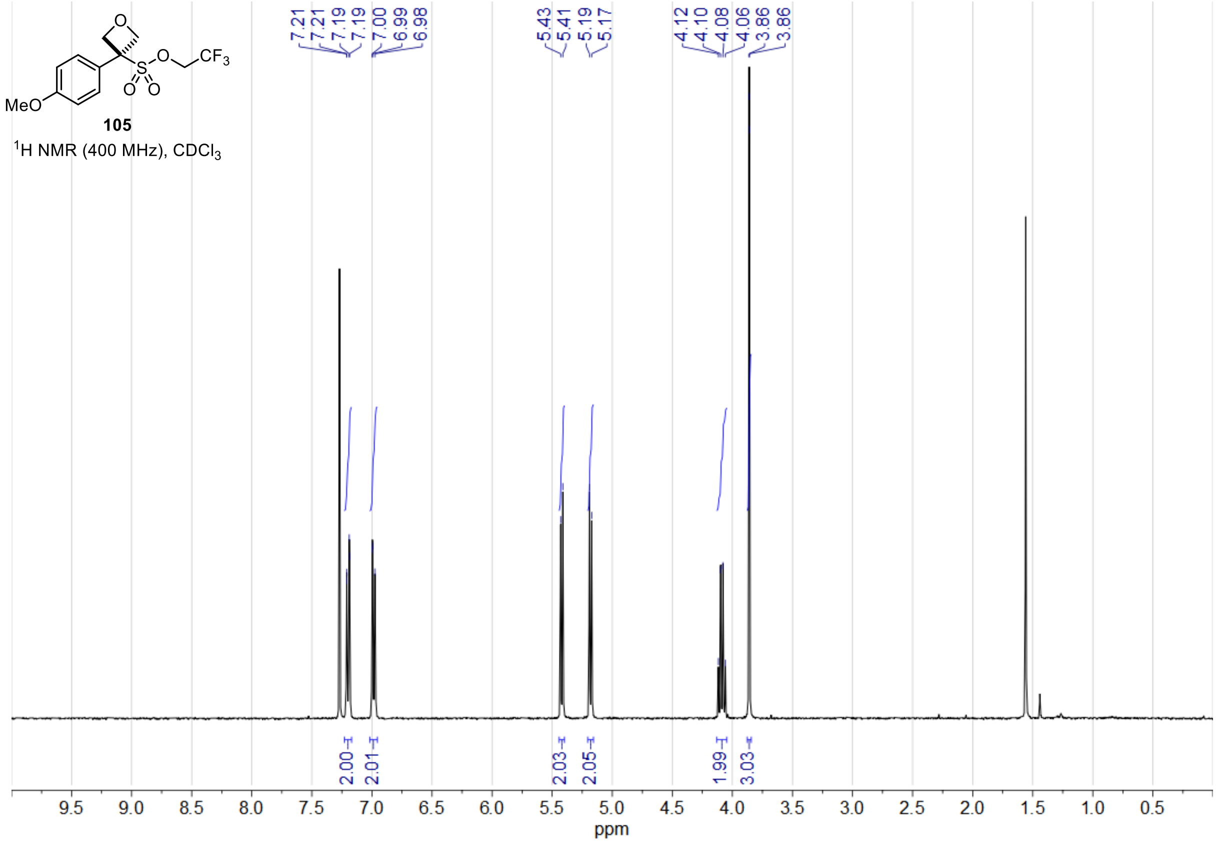

105

^{1}H NMR (400 MHz), $CDCl_3$

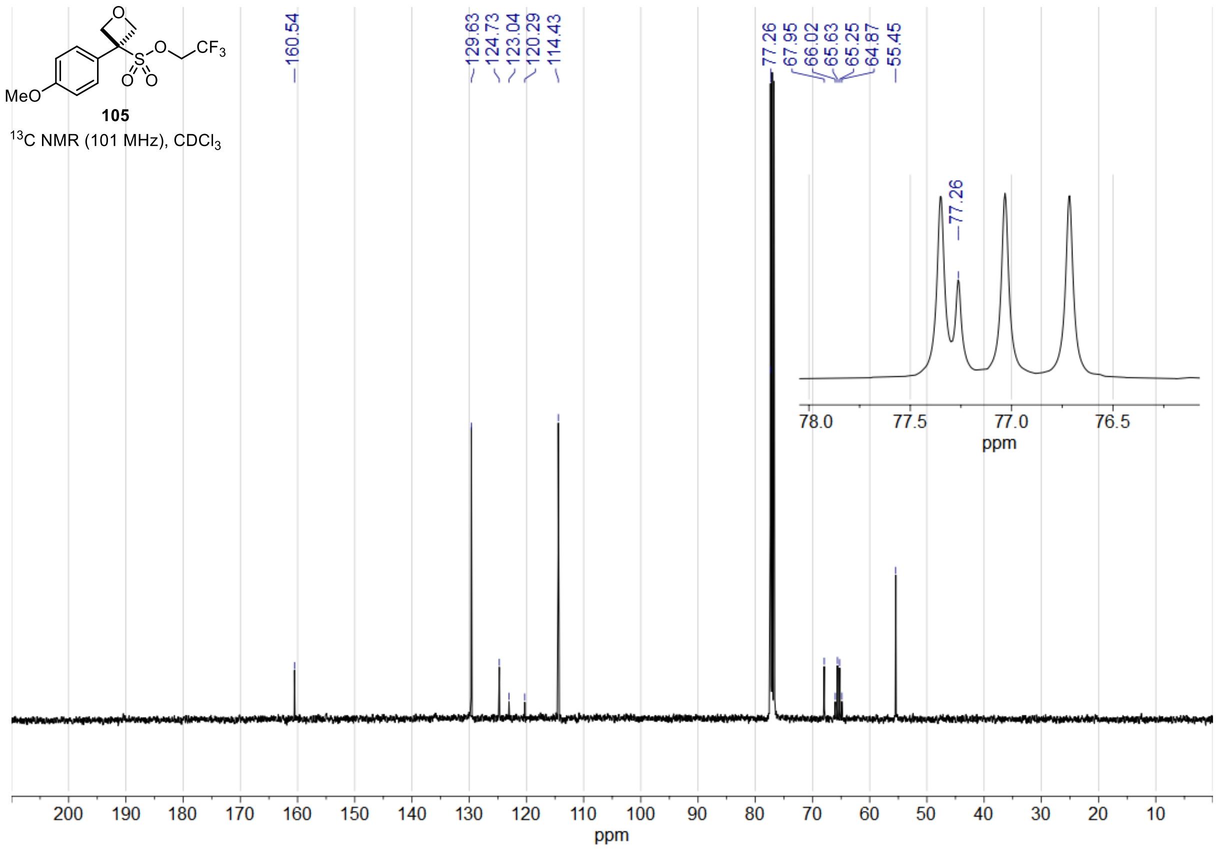

105

^{13}C NMR (101 MHz), $CDCl_3$

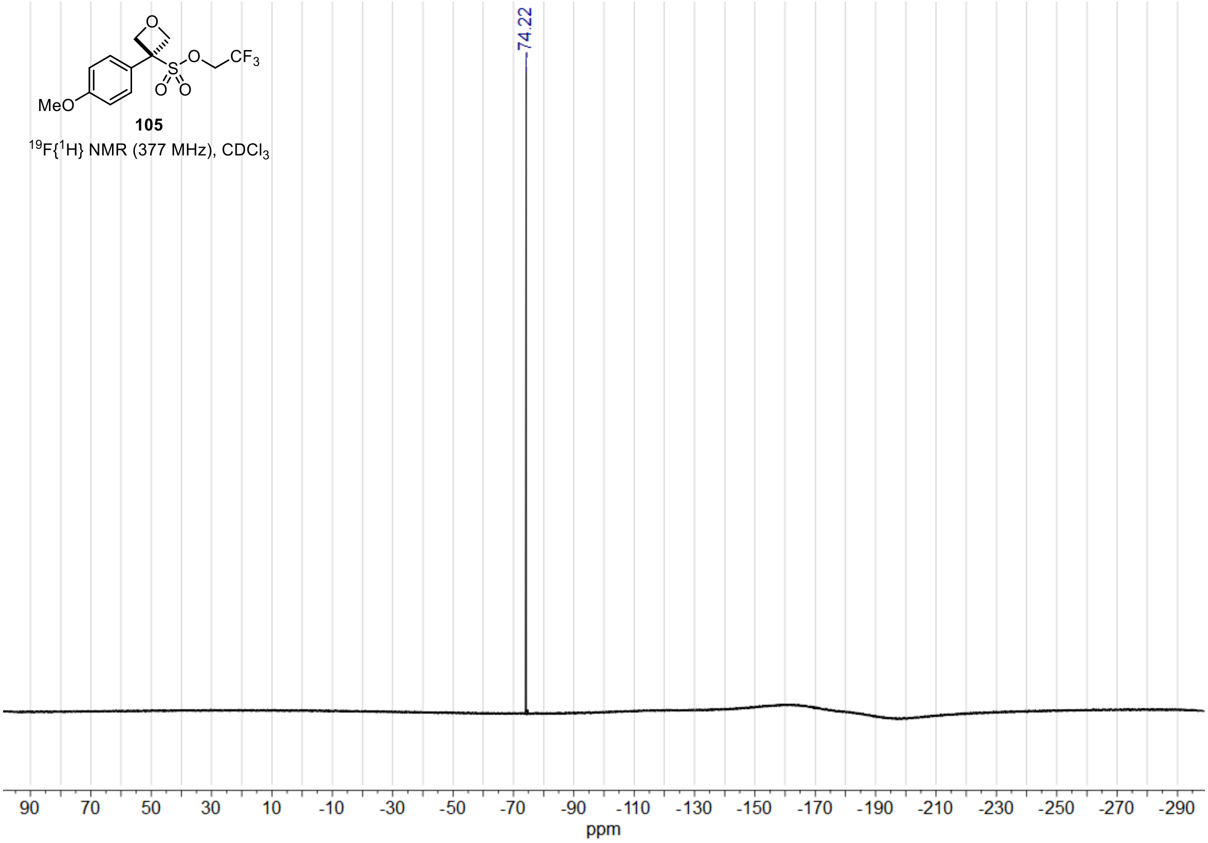
MeO
O
S
O
O
O
CF3
105
19F{1H} NMR (377 MHz), CDCl3
-74.22
90
70
50
30
10
-10
-30
-50
-70
-90
-110
-130
-150
-170
-190
-210
-230
-250
-270
-290
ppm

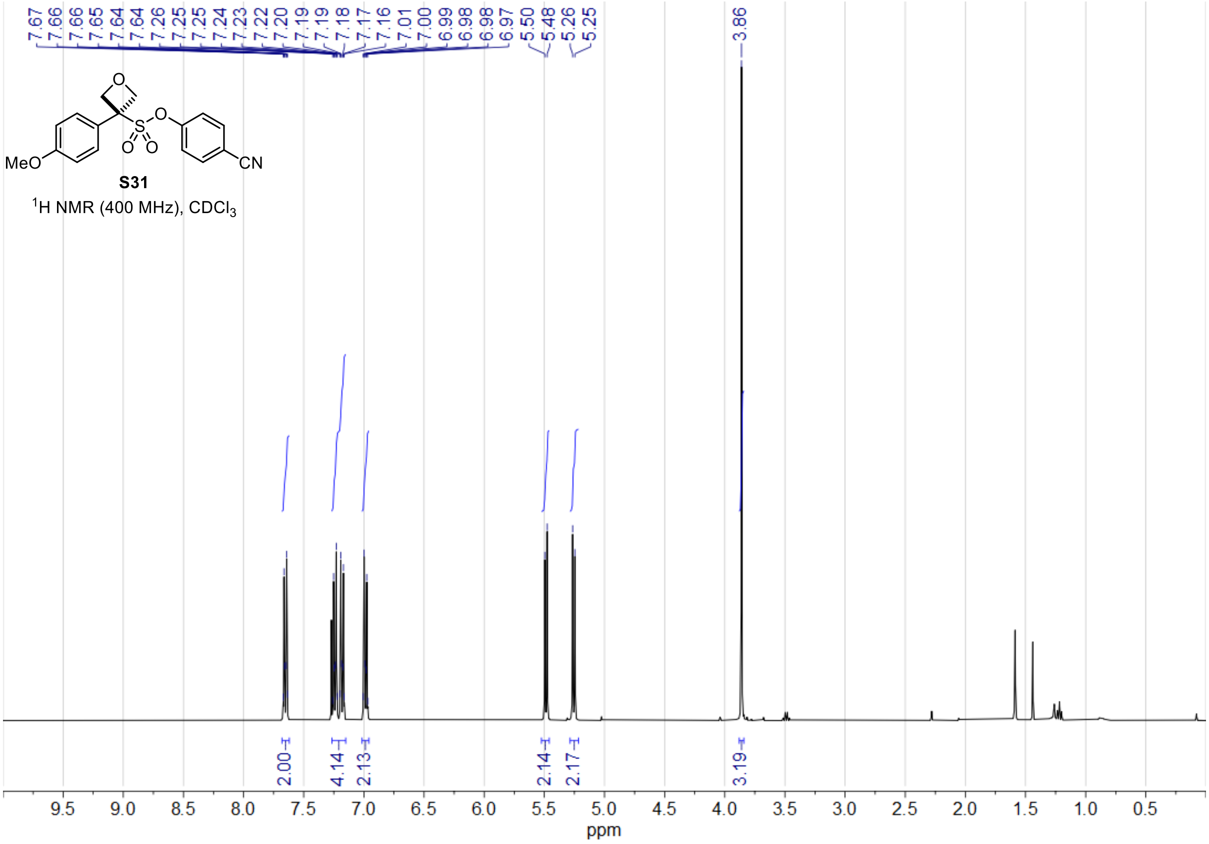
7.67
7.66
7.65
7.64
7.64
7.26
7.25
7.25
7.24
7.23
7.22
7.20
7.19
7.19
7.18
7.17
7.16
7.01
7.00
6.99
6.98
6.98
6.97
5.50
5.48
5.26
5.25
3.86
MeO
O
S
O
O
O
CN
S31
^{1}H NMR (400 MHz), $CDCl_3$
2.00
4.14
2.13
2.14
2.17
3.19
ppm

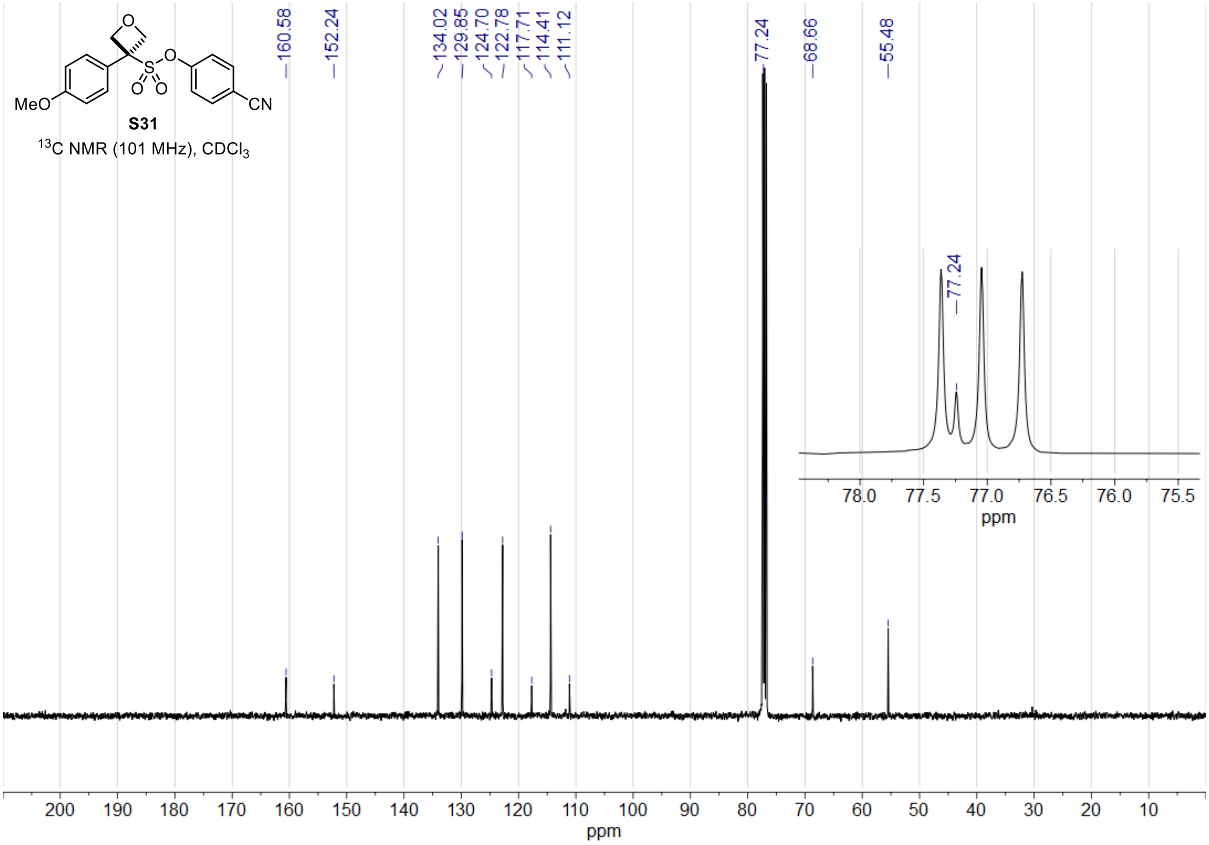
160.58
152.24
134.02
129.85
124.70
122.78
117.71
114.41
111.12
77.24
68.66
55.48
MeO
O
S
O
O
O
CN
S31
^{13}C NMR (101 MHz), $CDCl_3$
77.24
ppm
ppm

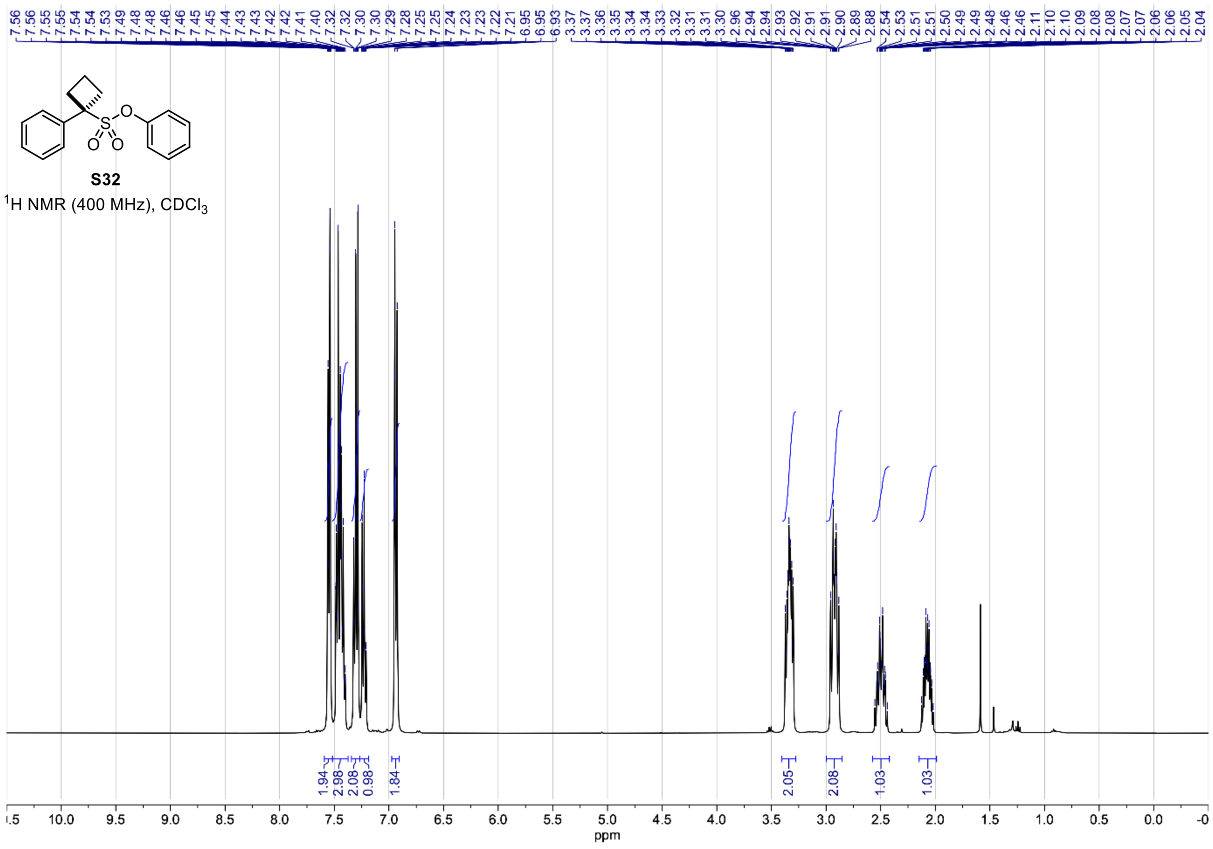
S32
^{1}H NMR (400 MHz), CDCl3
ppm

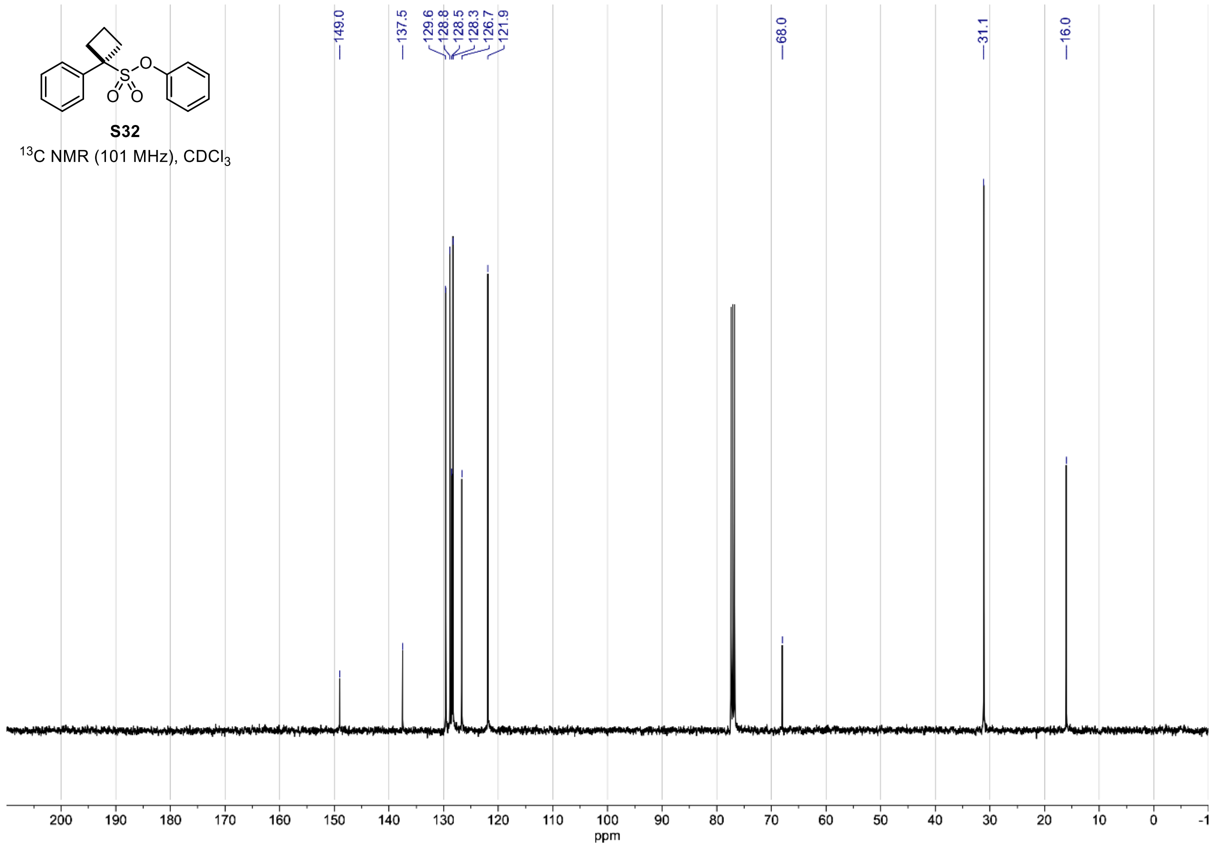
S32
^{13}C NMR (101 MHz), CDCl3
149.0
137.5
129.6
128.8
128.5
128.3
126.7
121.9
68.0
31.1
16.0
ppm

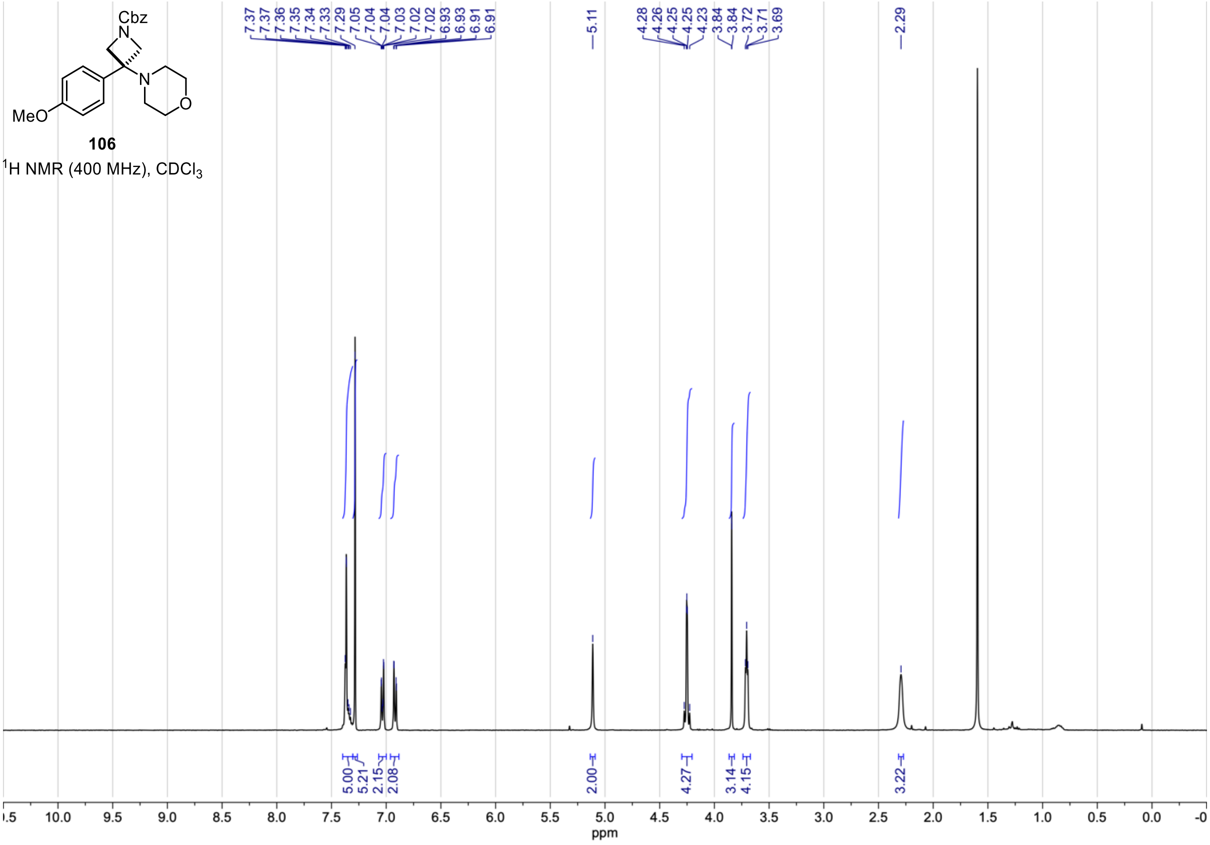
Cbz
N
N
O
MeO
106
¹H NMR (400 MHz), CDCl3
ppm

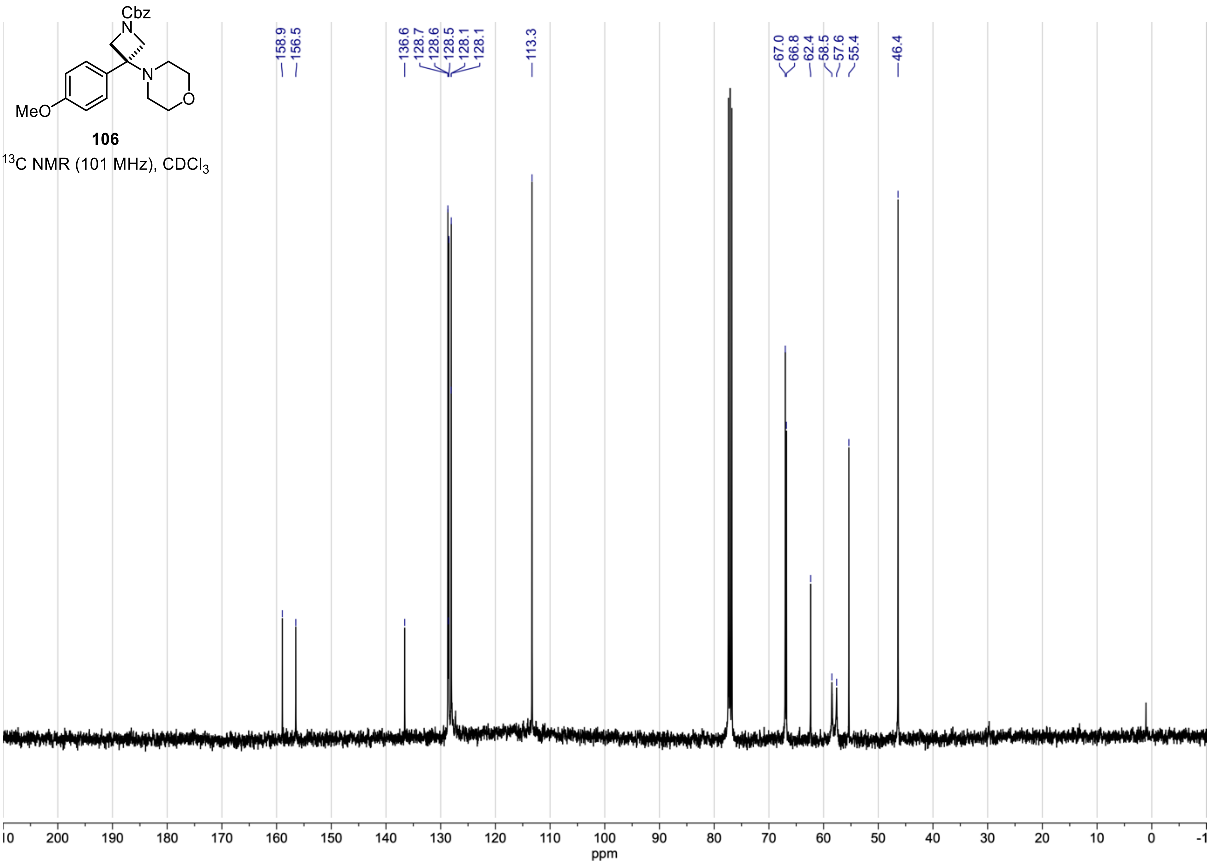
Cbz
N
N
O
MeO
106
¹³C NMR (101 MHz), CDCl3
ppm

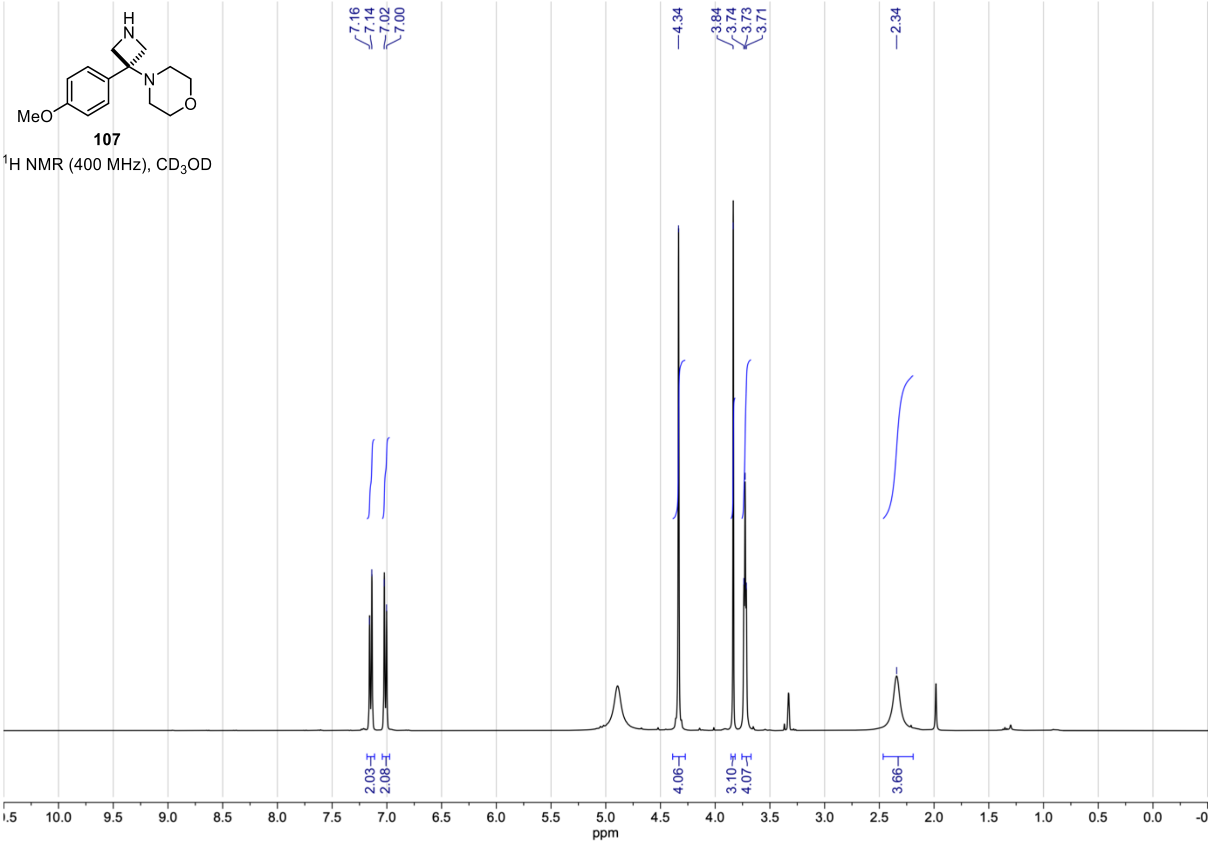
107
1H NMR (400 MHz), CD3OD
7.16
7.14
7.02
7.00
4.34
3.84
3.74
3.73
3.71
2.34
2.03
2.08
4.06
3.10
4.07
3.66
ppm

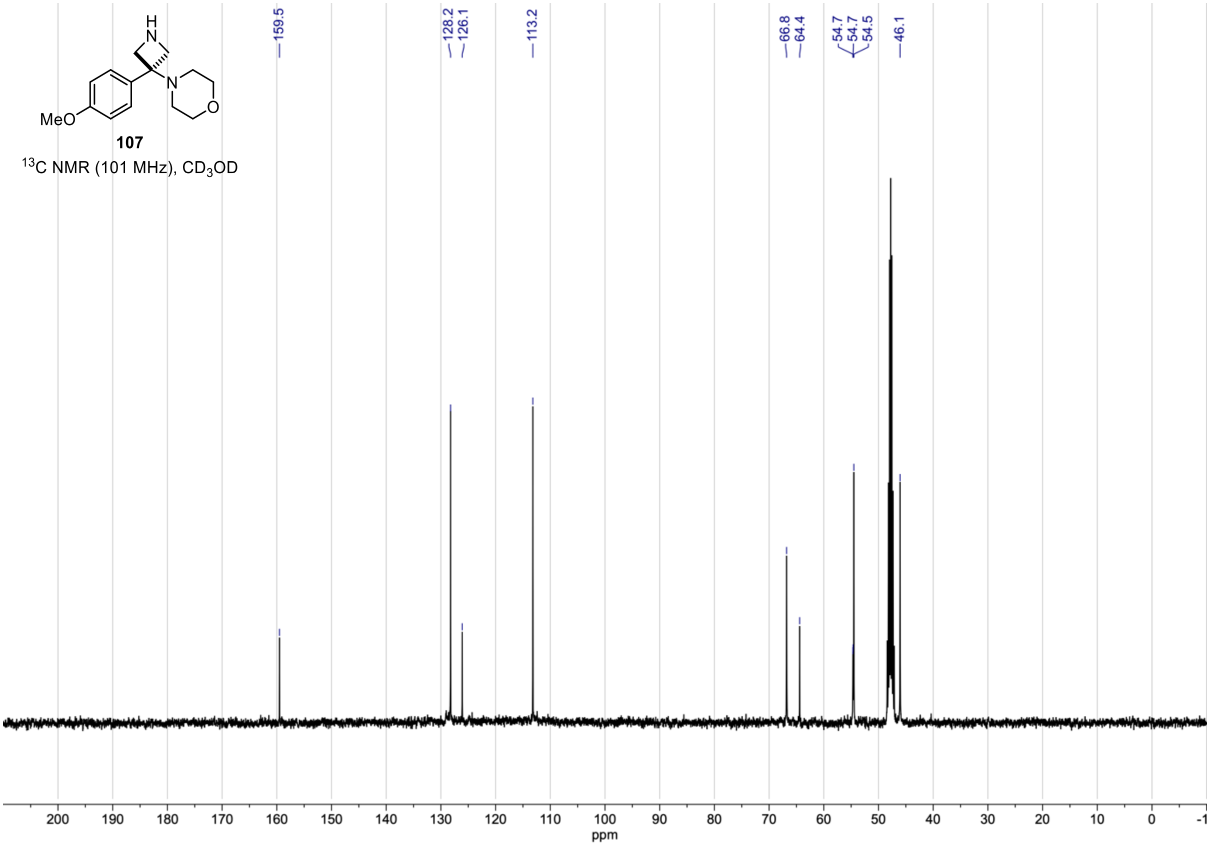
107
13C NMR (101 MHz), CD3OD
159.5
128.2
126.1
113.2
66.8
64.4
54.7
54.7
54.5
46.1
ppm

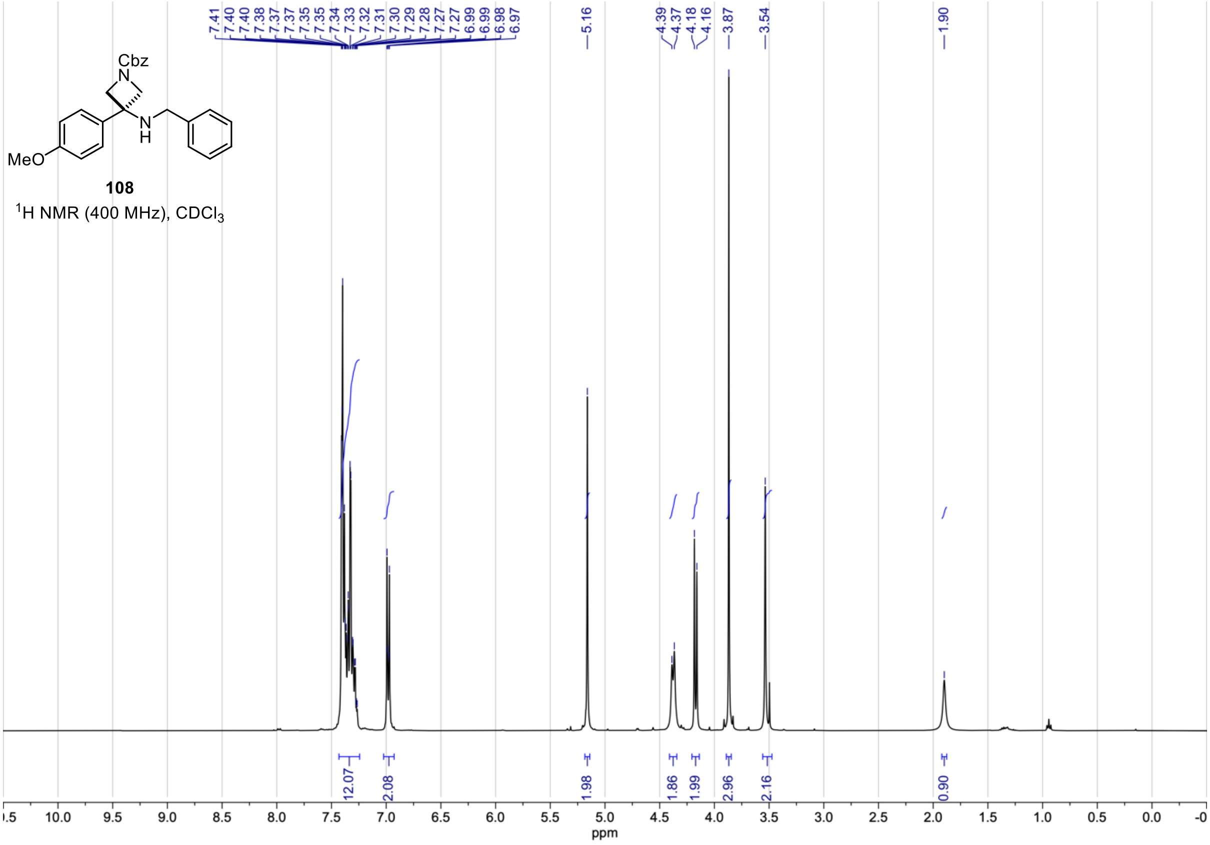

108

^{1}H NMR (400 MHz), $CDCl_3$

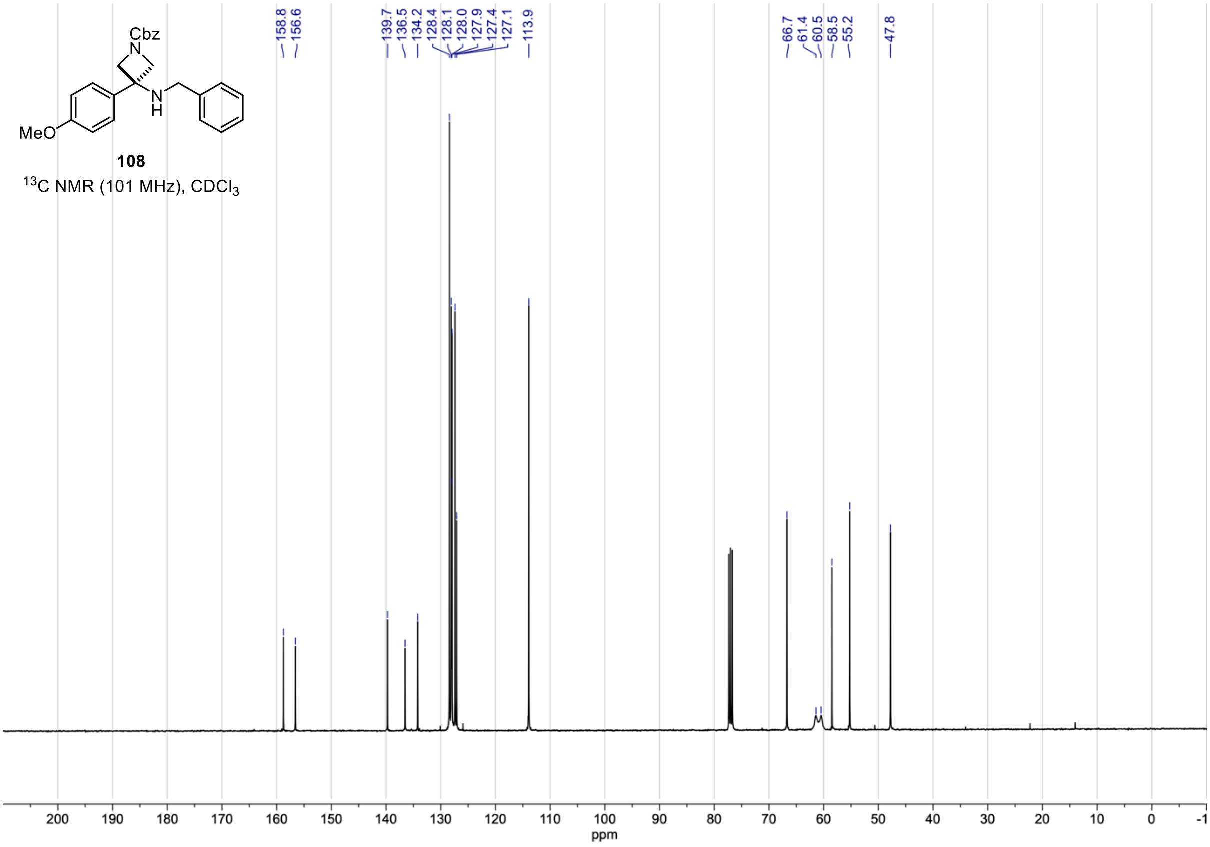

108

^{13}C NMR (101 MHz), $CDCl_3$

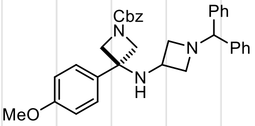

109

^{1}H NMR (400 MHz), $CDCl_3$

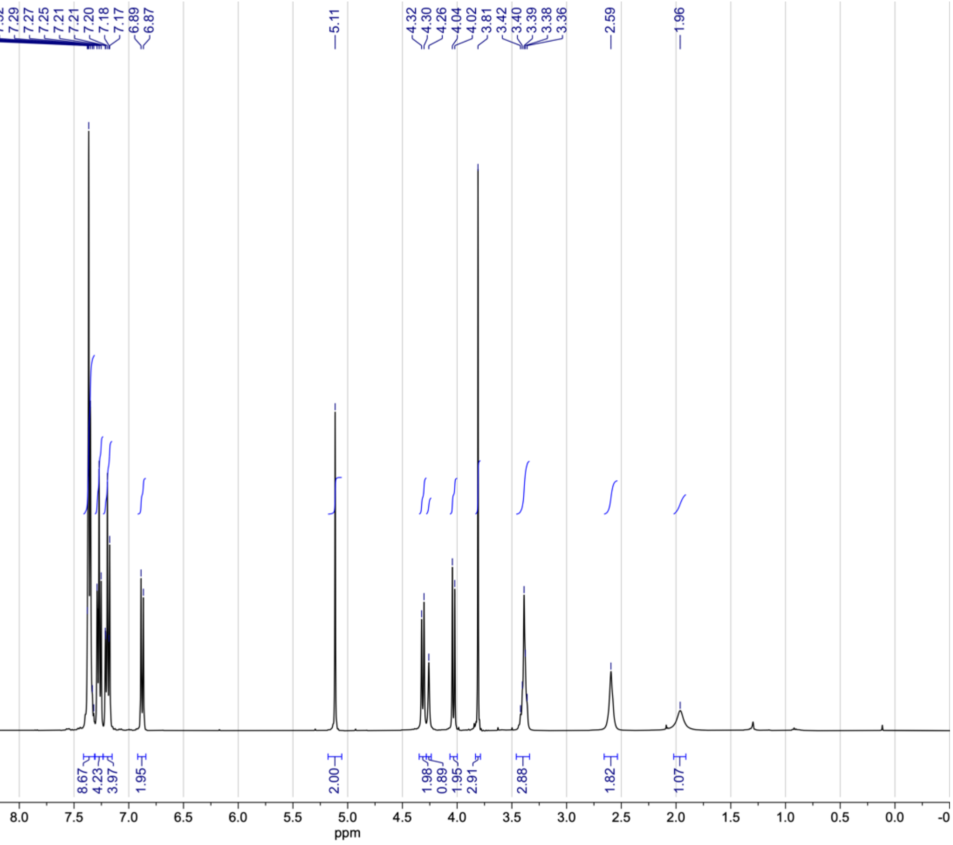

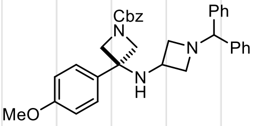

109

^{13}C NMR (101 MHz), $CDCl_3$

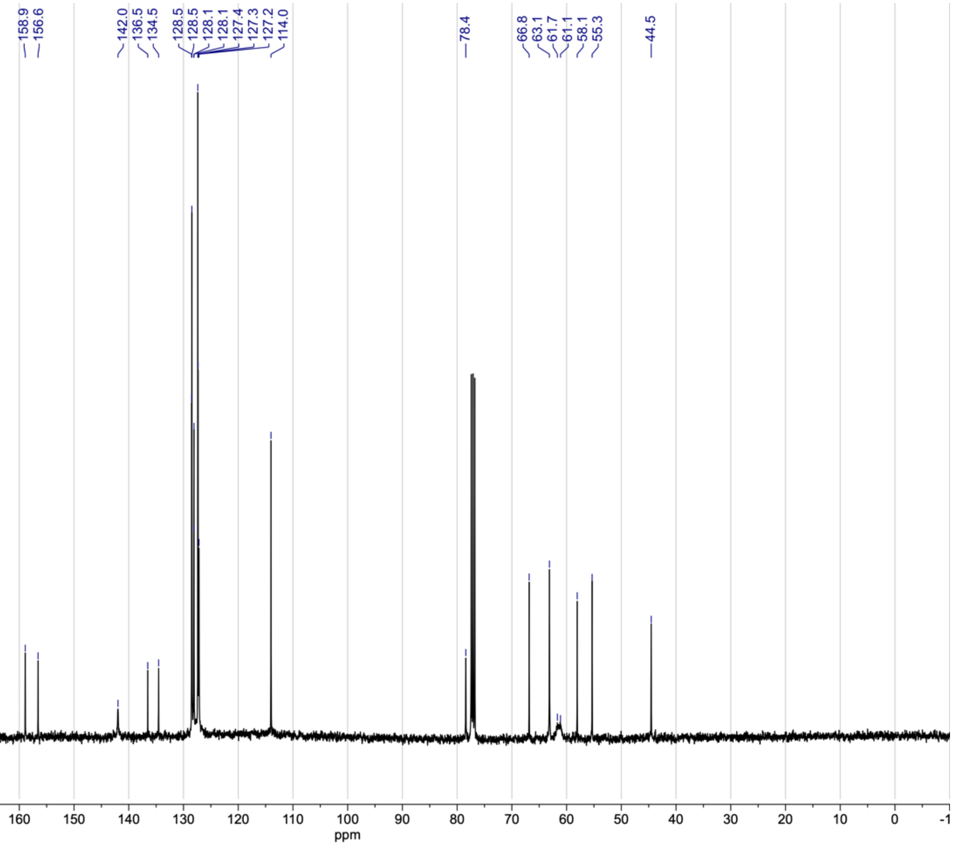

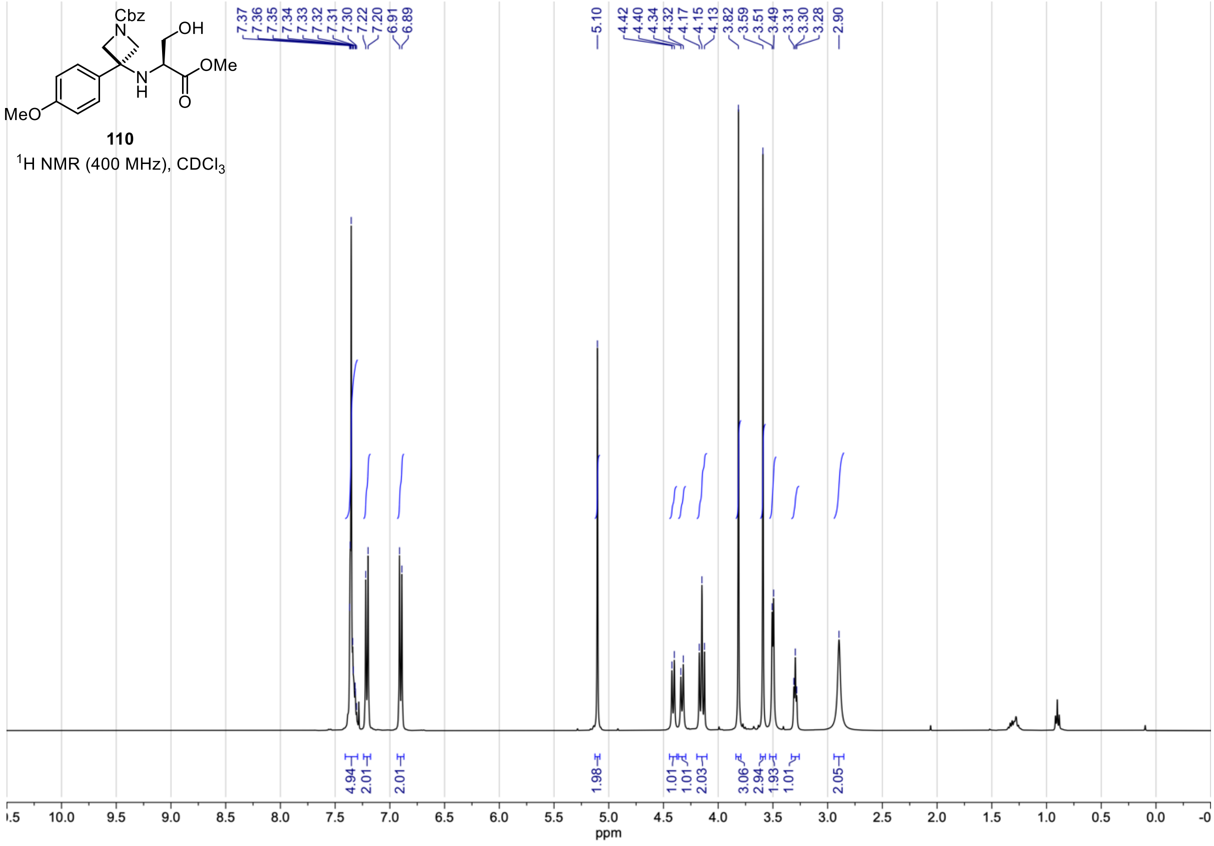

110

^{1}H NMR (400 MHz), $CDCl_3$

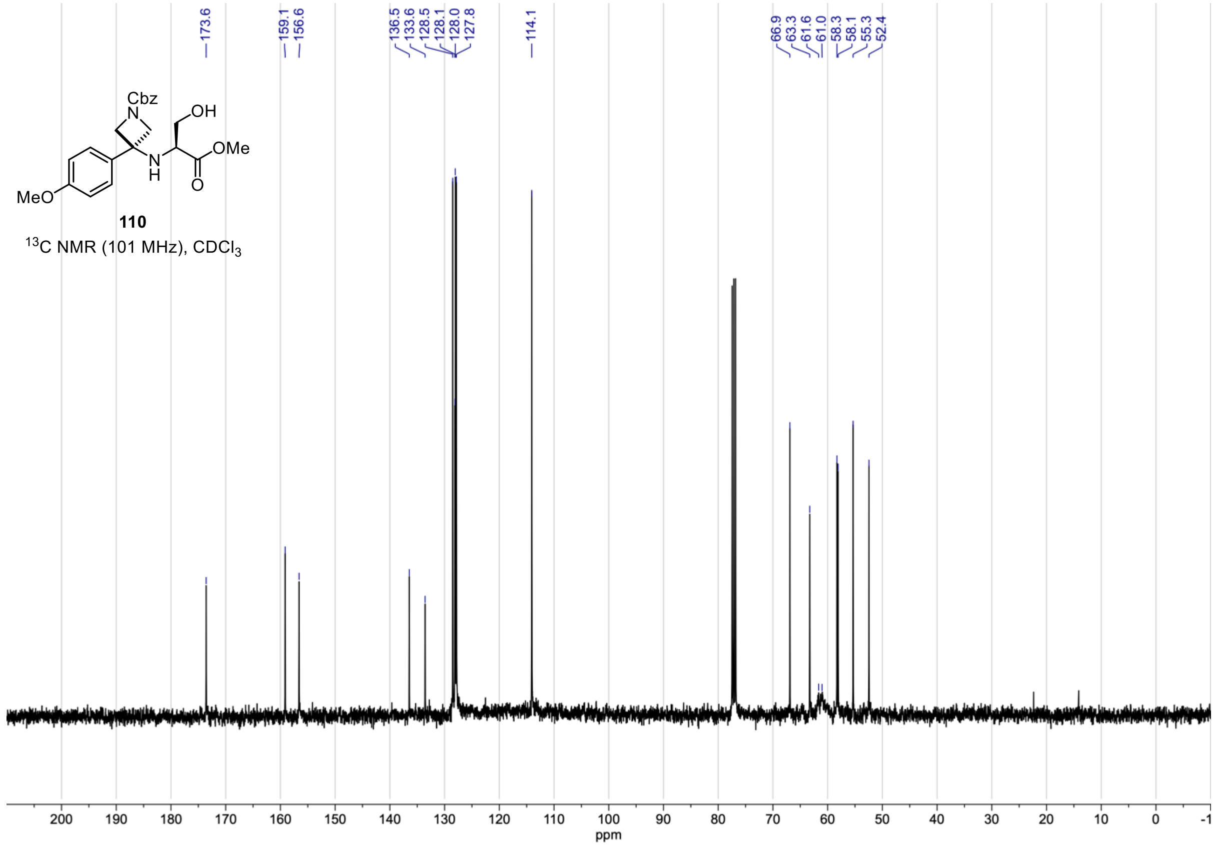

110

^{13}C NMR (101 MHz), $CDCl_3$

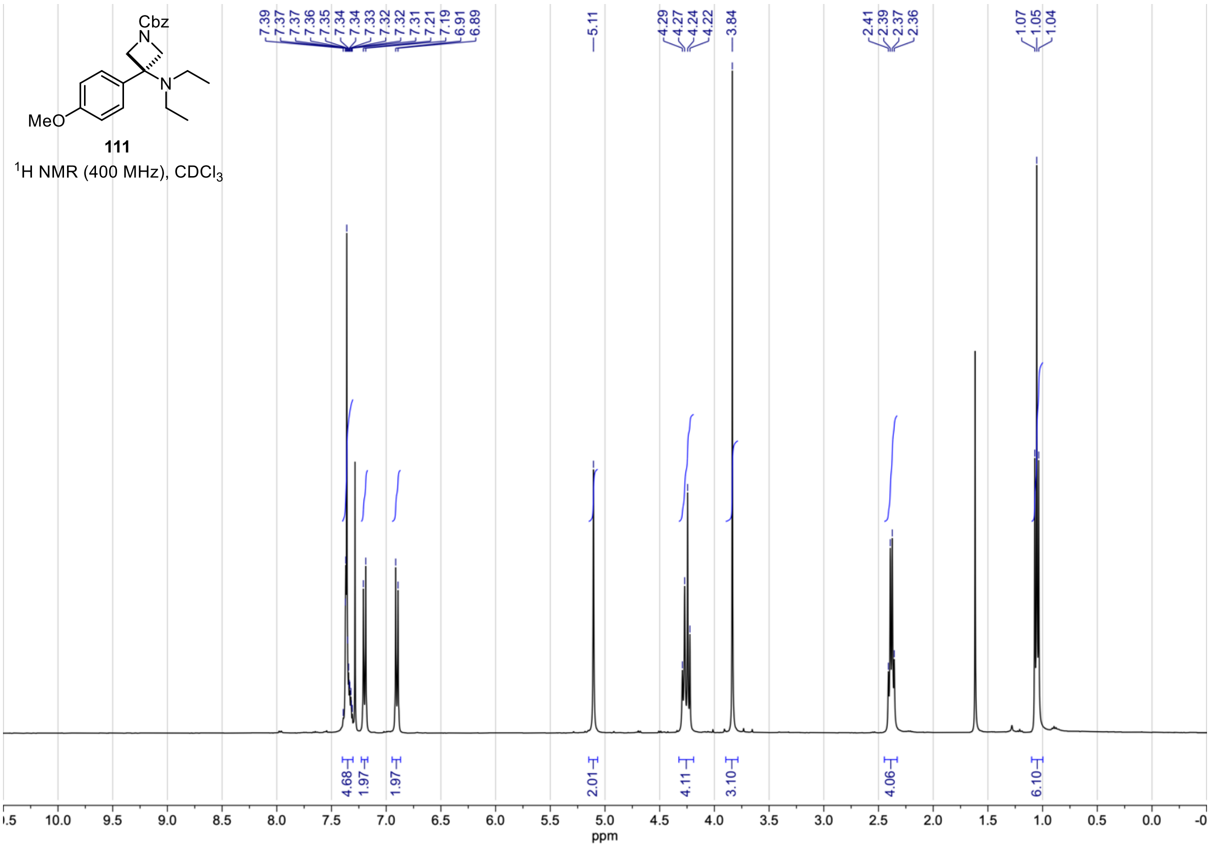
Cbz
N
MeO
111
^{1}H NMR (400 MHz), CDCl$_3$
7.39 7.37 7.37 7.36 7.35 7.34 7.34 7.33 7.32 7.32 7.31 7.21 7.19 6.91 6.89
5.11
4.29 4.27 4.24 4.22
3.84
2.41 2.39 2.37 2.36
1.07 1.05 1.04
4.68 1.97 1.97 2.01 4.11 3.10 4.06 6.10
ppm

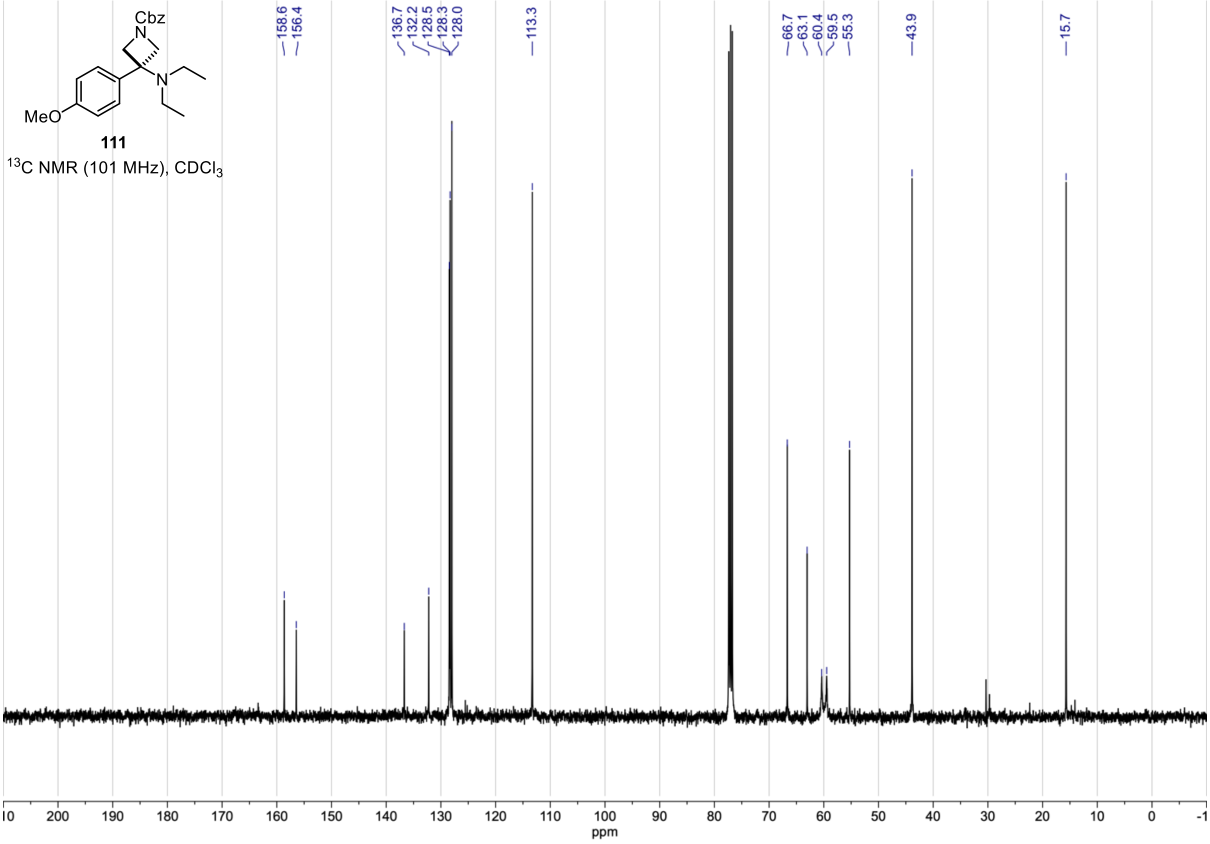
Cbz
N
MeO
111
^{13}C NMR (101 MHz), CDCl$_3$
158.6 156.4
136.7 132.2 128.5 128.3 128.0
113.3
66.7 63.1 60.4 59.5 55.3
43.9
15.7
ppm

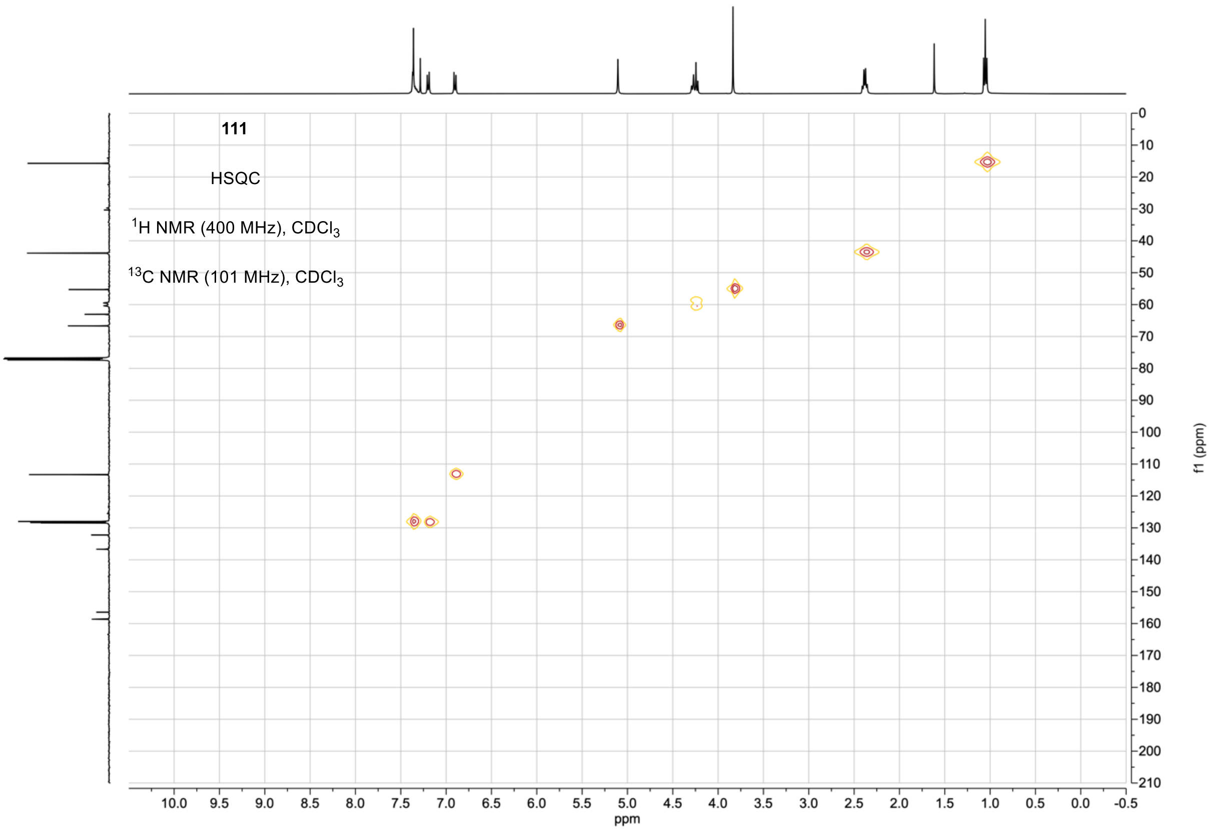
111
HSQC
1H NMR (400 MHz), CDCl3
13C NMR (101 MHz), CDCl3
ppm
f1 (ppm)

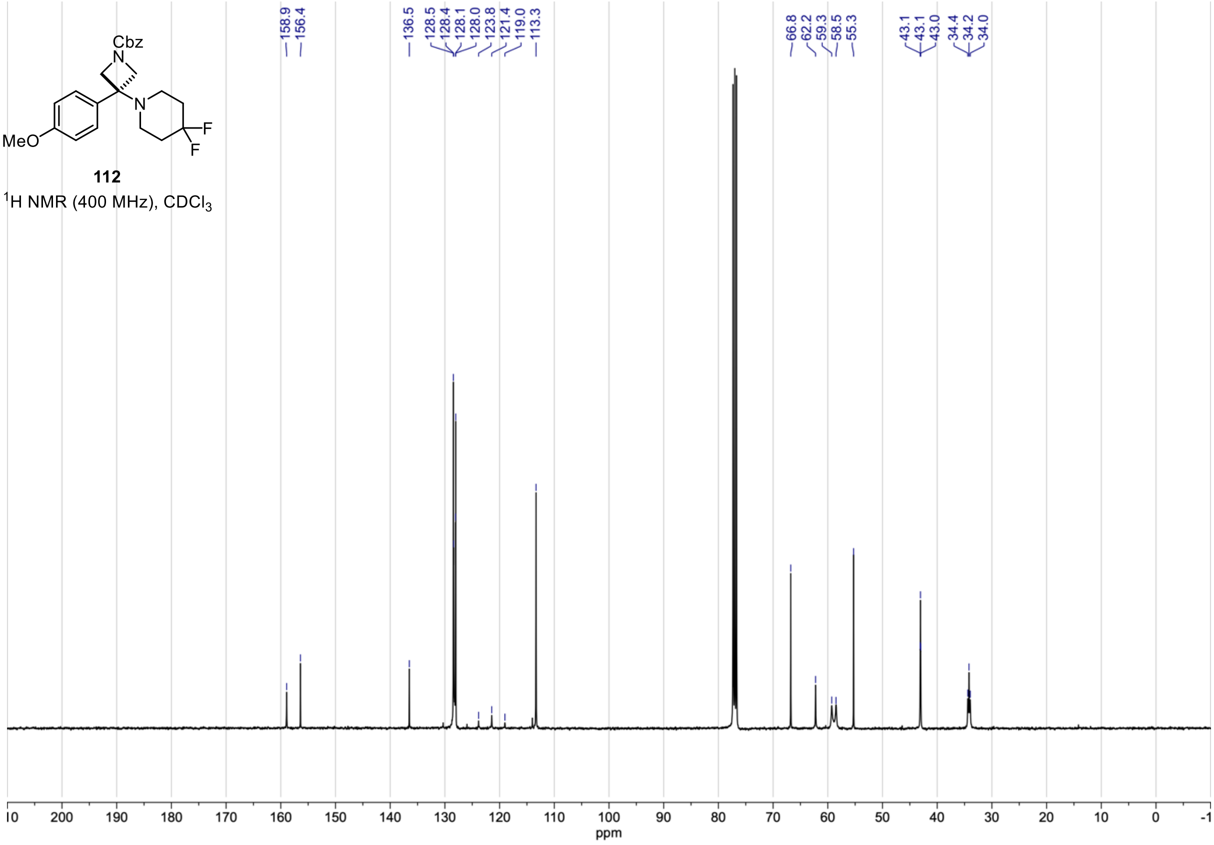

112

^{1}H NMR (400 MHz), $CDCl_3$

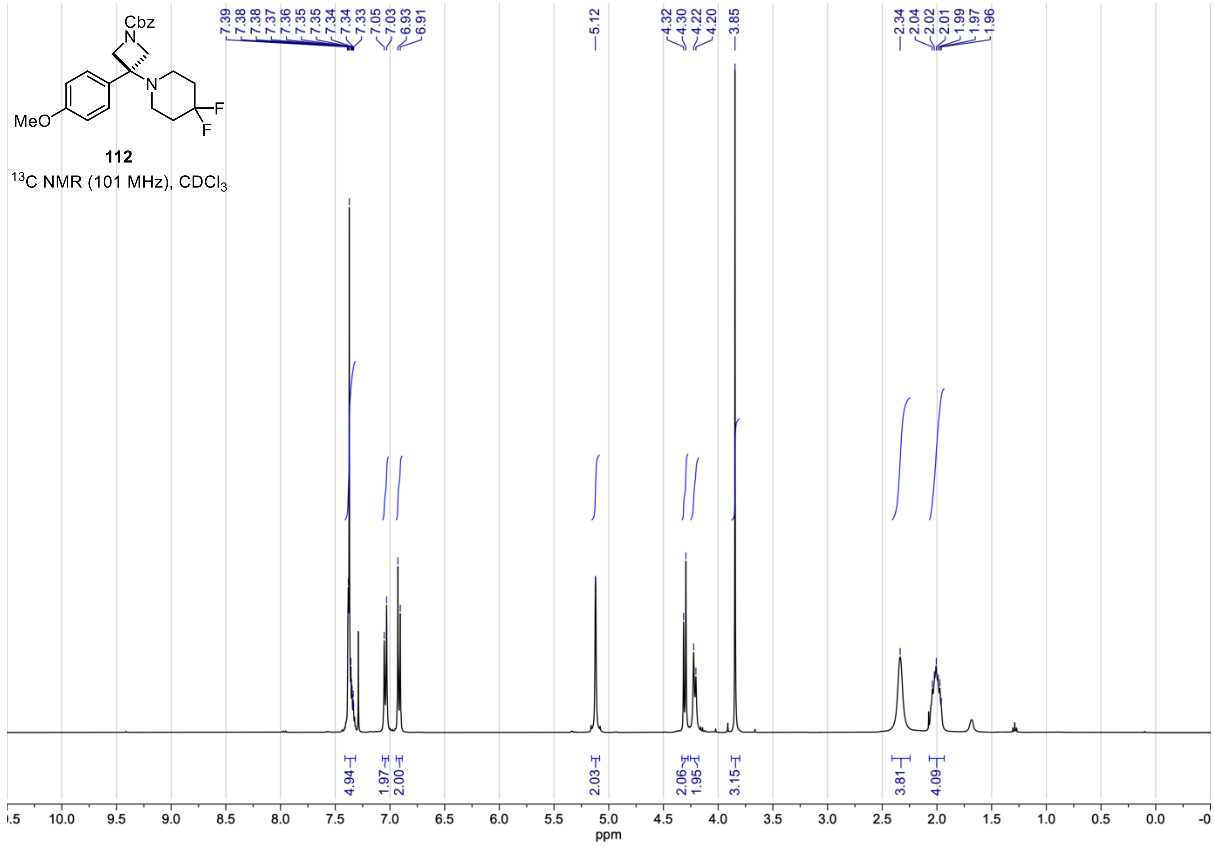

112

^{13}C NMR (101 MHz), $CDCl_3$

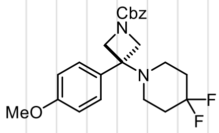

112

$^{19}F\{^{1}H\}$ NMR (377 MHz), $CDCl_3$

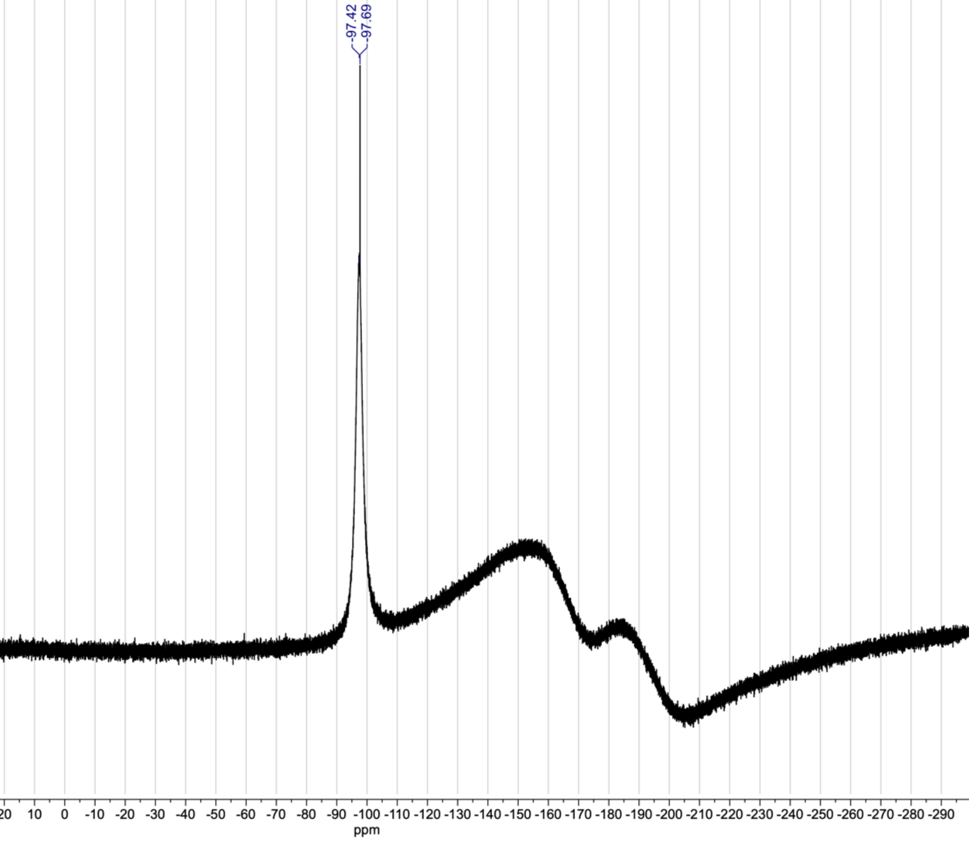

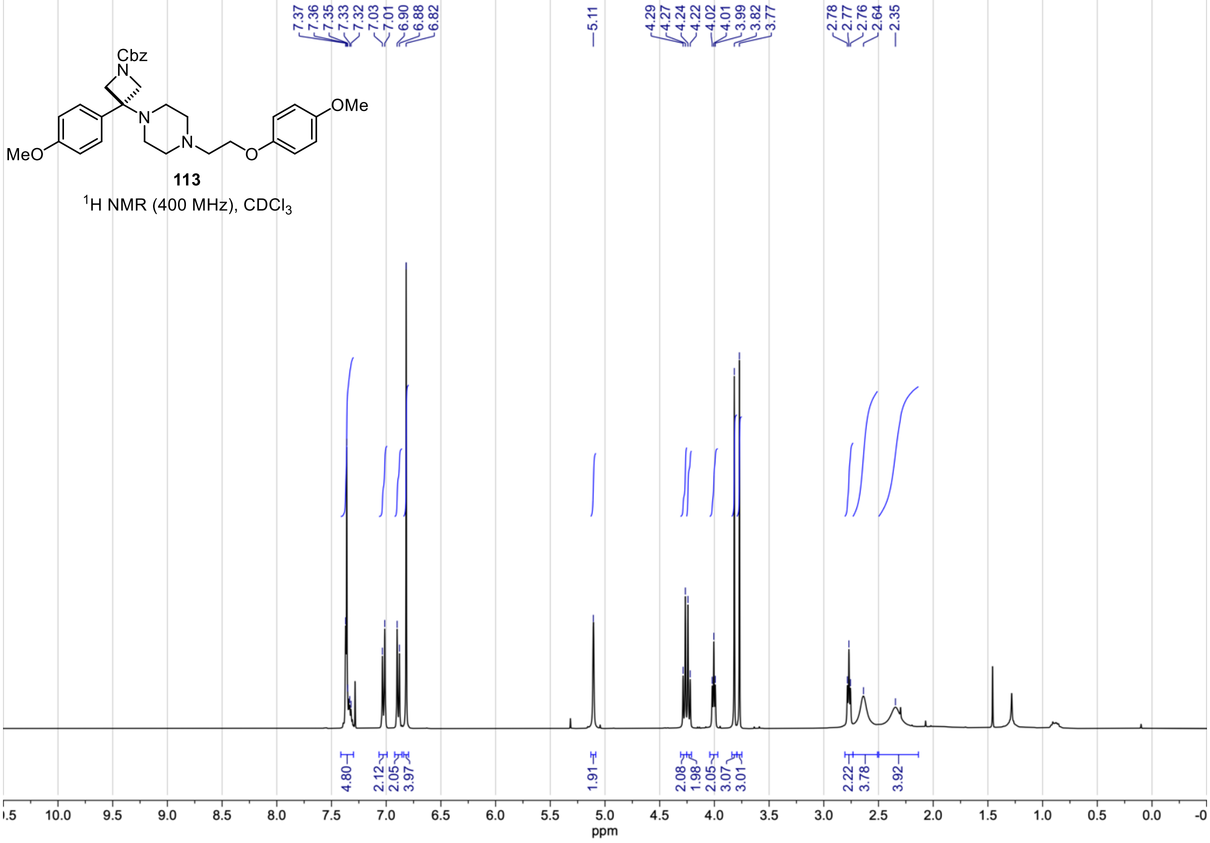

113
^{1}H NMR (400 MHz), CDCl$_3$
7.37 7.36 7.35 7.33 7.32 7.03 7.01 6.90 6.88 6.82
5.11
4.29 4.27 4.24 4.22 4.02 4.01 3.99 3.82 3.77
2.78 2.77 2.76 2.64 2.35
4.80 2.12 2.05 3.97 1.91 2.08 1.98 2.05 3.07 3.01 2.22 3.78 3.92
ppm

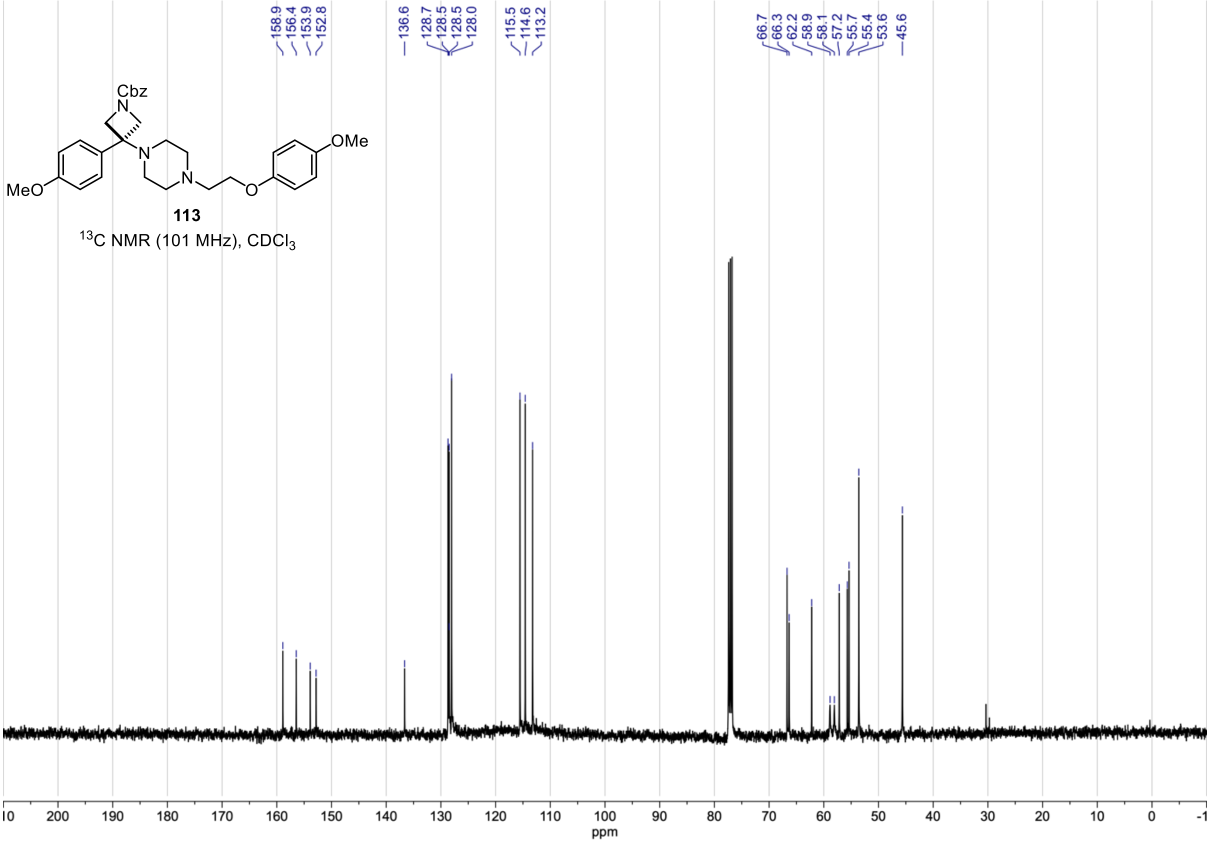

113
^{13}C NMR (101 MHz), CDCl$_3$
158.9 156.4 153.9 152.8
136.6 128.7 128.5 128.5 128.0
115.5 114.6 113.2
66.7 66.3 62.2 58.9 58.1 57.2 55.7 55.4 53.6 45.6
ppm

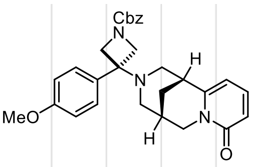

114

^{1}H NMR (400 MHz), $CDCl_3$

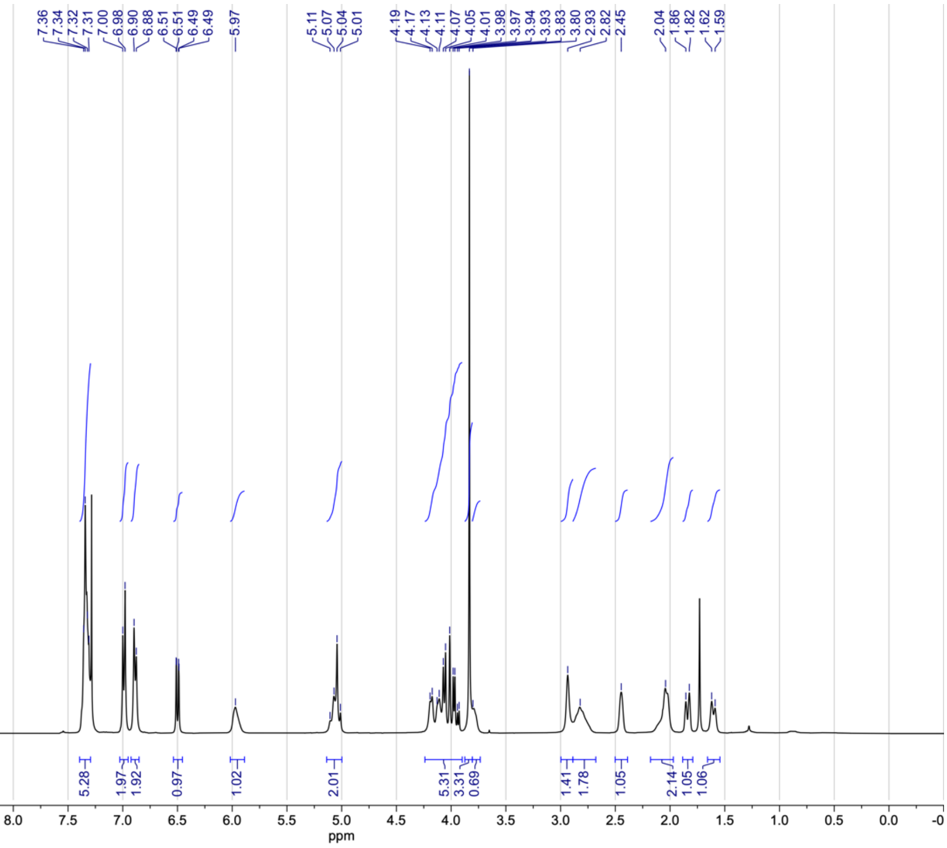

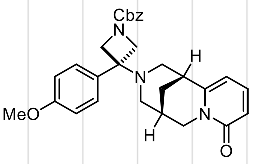

114

^{13}C NMR (101 MHz), $CDCl_3$

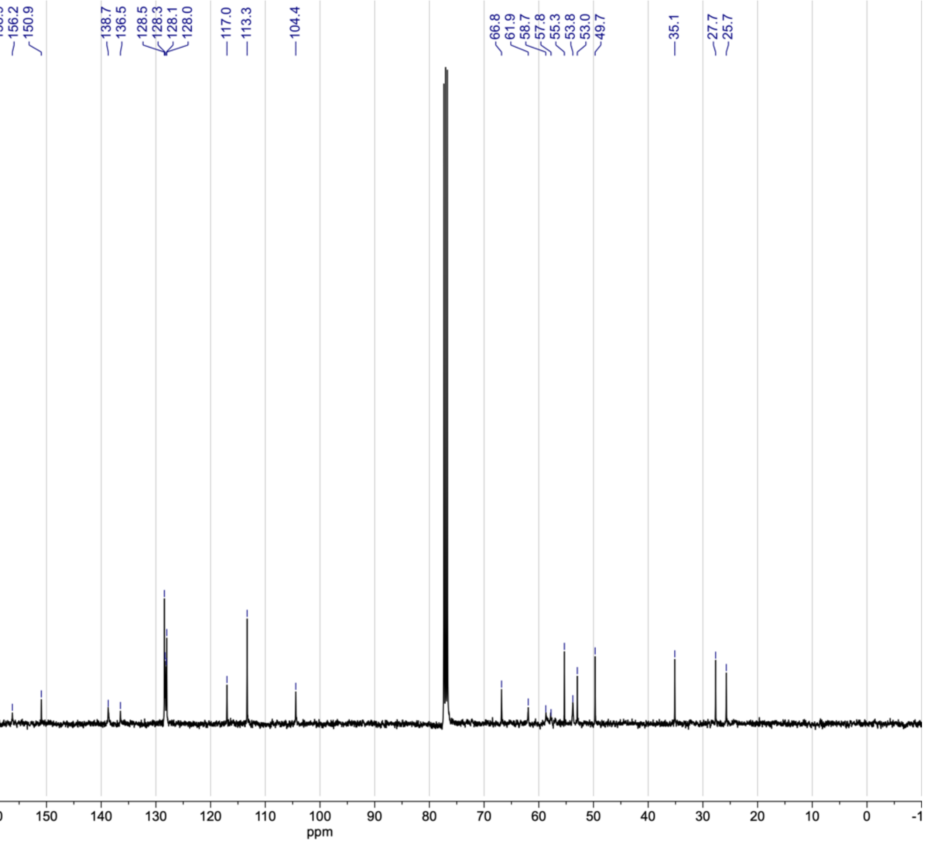

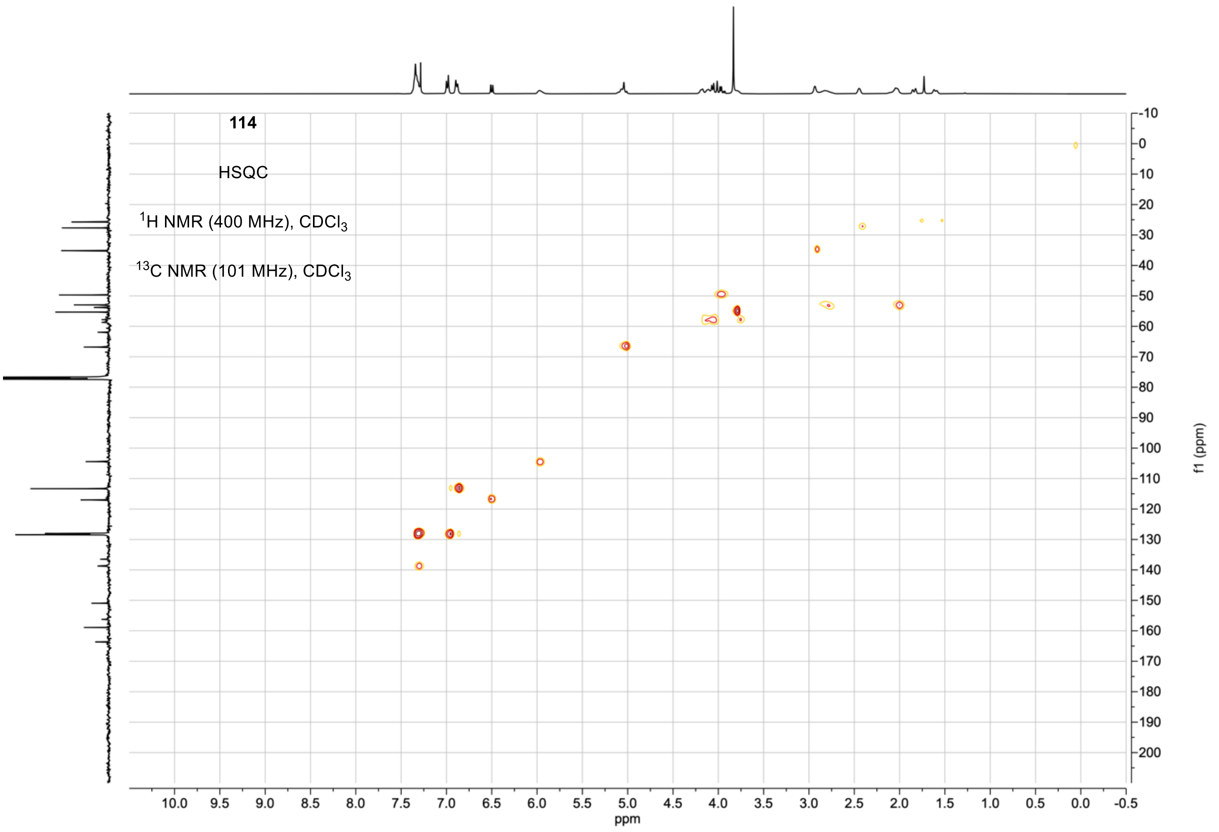
114
HSQC
1H NMR (400 MHz), CDCl3
13C NMR (101 MHz), CDCl3
ppm
f1 (ppm)

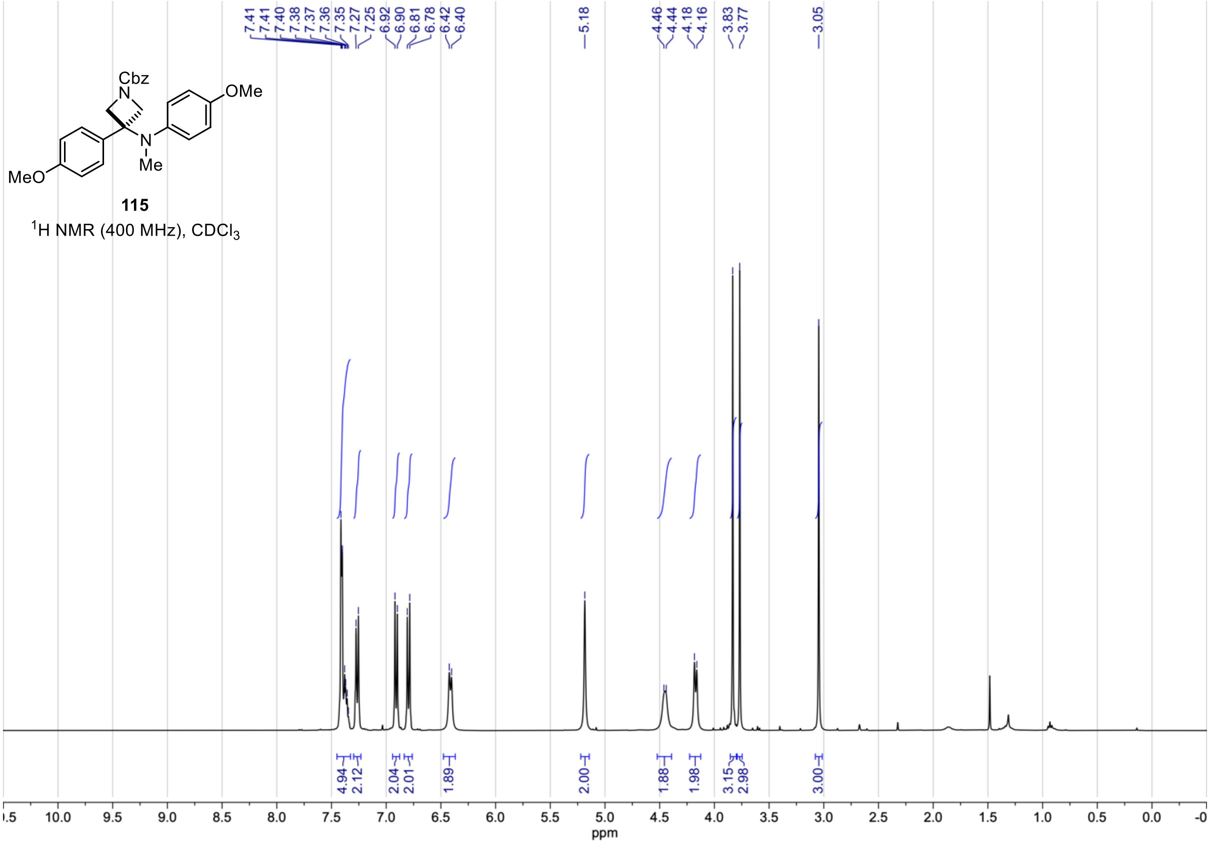
7.41
7.41
7.40
7.38
7.37
7.36
7.35
7.27
7.25
6.92
6.90
6.81
6.78
6.42
6.40
5.18
4.46
4.44
4.18
4.16
3.83
3.77
3.05
Cbz
N
OMe
N
Me
MeO
115
1H NMR (400 MHz), CDCl3
4.94
2.12
2.04
2.01
1.89
2.00
1.88
1.98
3.15
2.98
3.00
ppm

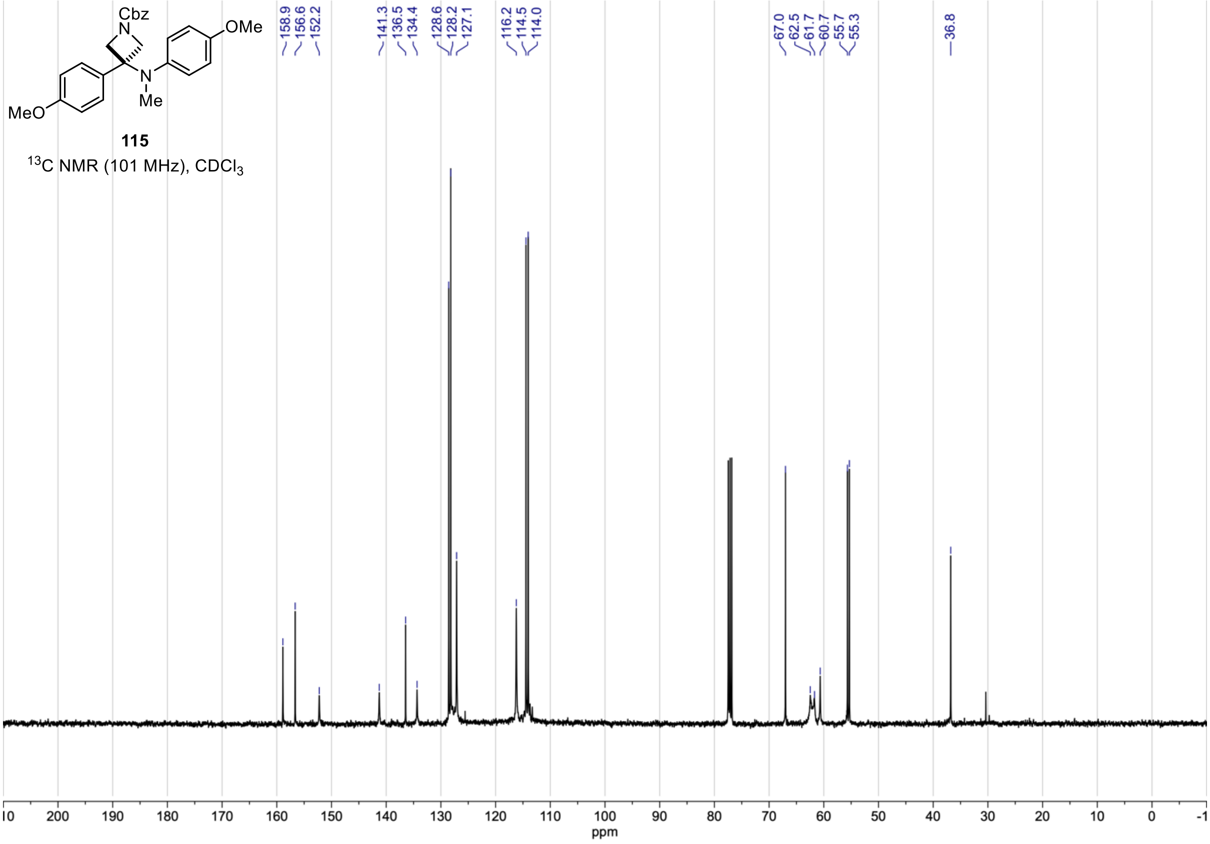
158.9
156.6
152.2
141.3
136.5
134.4
128.6
128.2
127.1
116.2
114.5
114.0
67.0
62.5
61.7
60.7
55.7
55.3
36.8
Cbz
N
OMe
N
Me
MeO
115
13C NMR (101 MHz), CDCl3
ppm

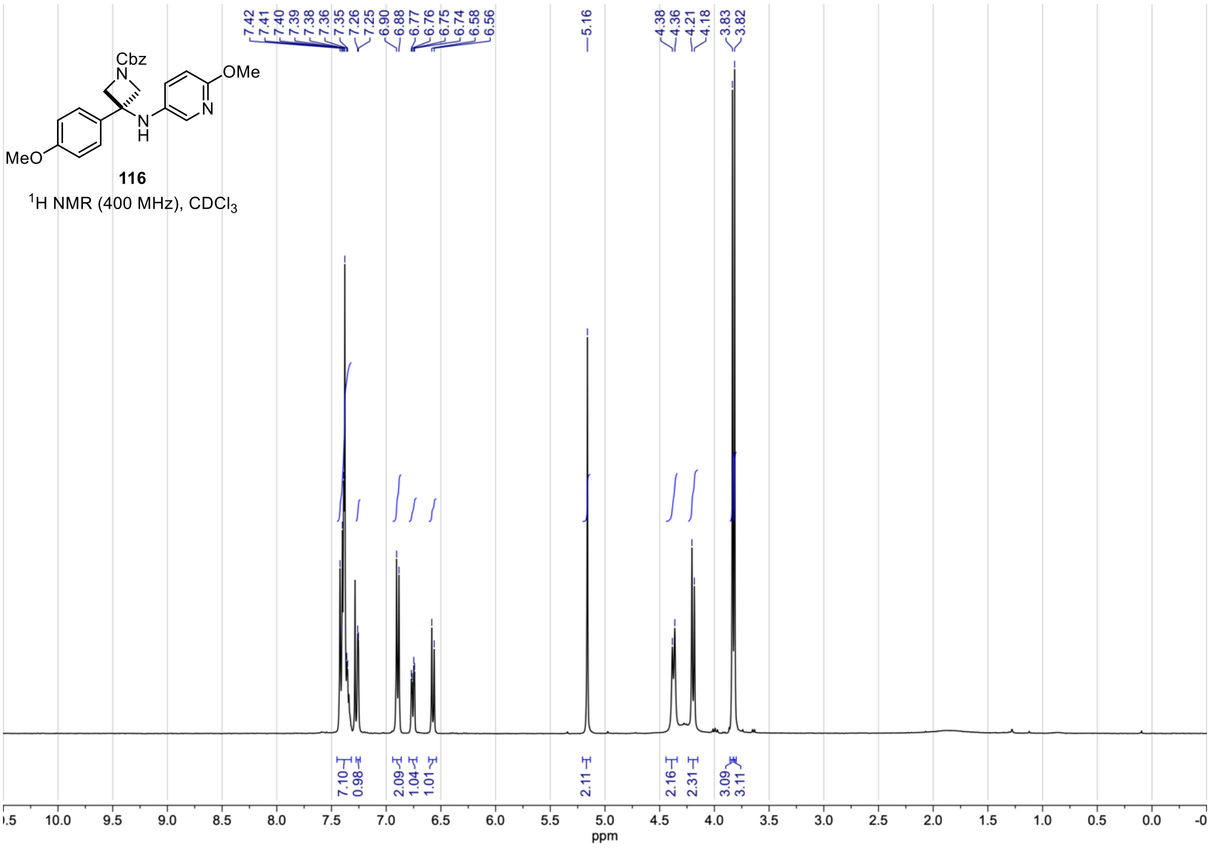

Cbz
N
OMe
N
MeO
N
H
116
^{1}H NMR (400 MHz), CDCl$_3$
7.42 7.41 7.40 7.39 7.38 7.36 7.35 7.26 7.25 6.90 6.88 6.77 6.76 6.75 6.74 6.58 6.56
5.16
4.38 4.36 4.21 4.18 3.83 3.82
7.10 0.98 2.09 1.04 1.01 2.11 2.16 2.31 3.09 3.11
10.0 9.5 9.0 8.5 8.0 7.5 7.0 6.5 6.0 5.5 5.0 4.5 4.0 3.5 3.0 2.5 2.0 1.5 1.0 0.5 0.0
ppm

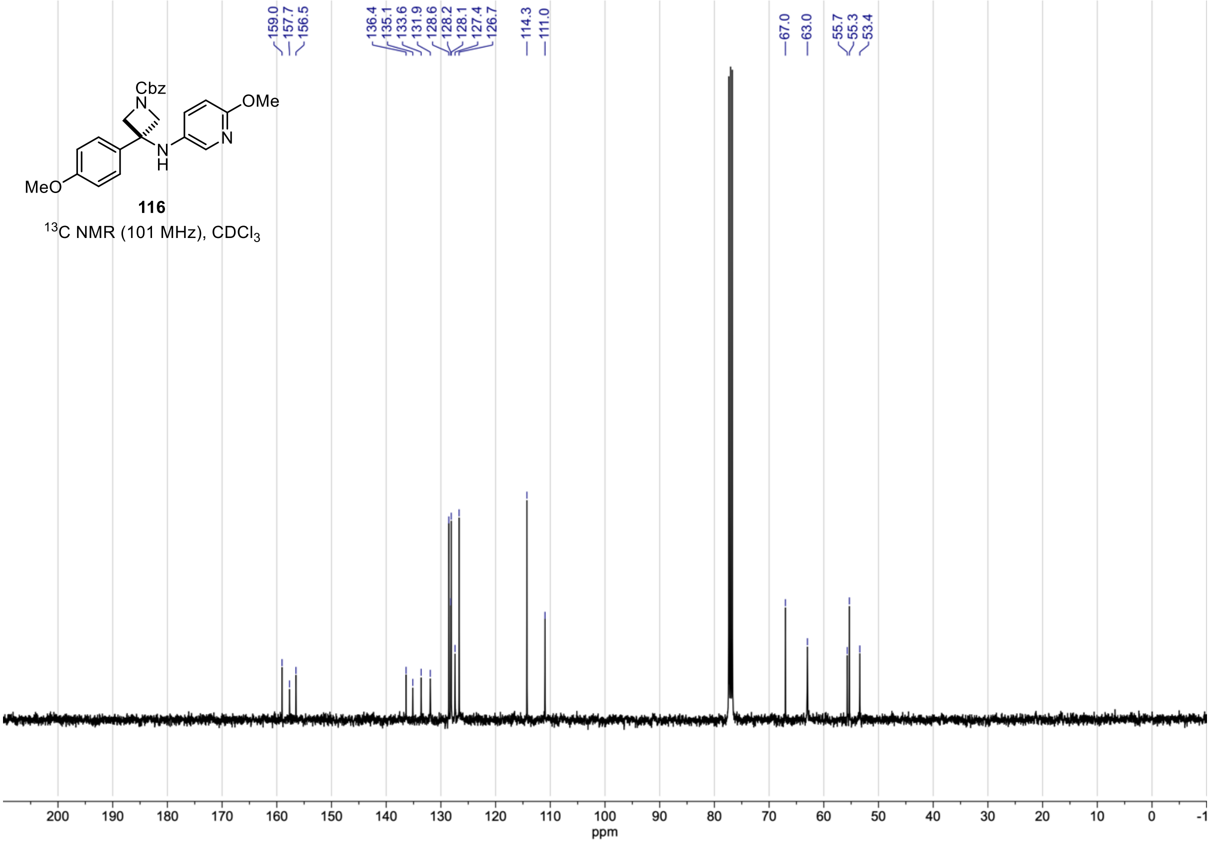

Cbz
N
OMe
N
MeO
N
H
116
^{13}C NMR (101 MHz), CDCl$_3$
159.0 157.7 156.5
136.4 135.1 133.6 131.9 128.6 128.2 128.1 127.4 126.7
114.3 111.0
67.0 63.0 55.7 55.3 53.4
200 190 180 170 160 150 140 130 120 110 100 90 80 70 60 50 40 30 20 10 0
ppm

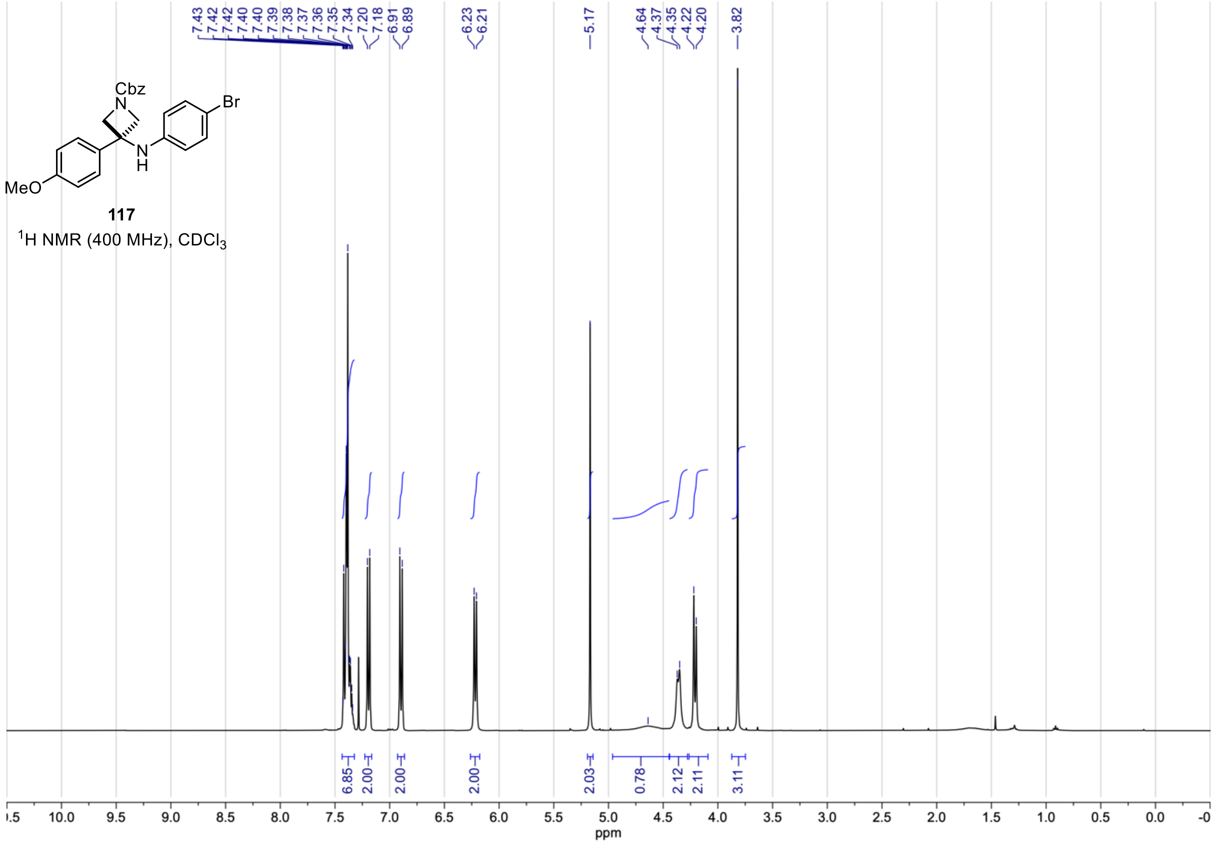
Cbz
N
Br
N
H
MeO
117
1H NMR (400 MHz), CDCl3
ppm

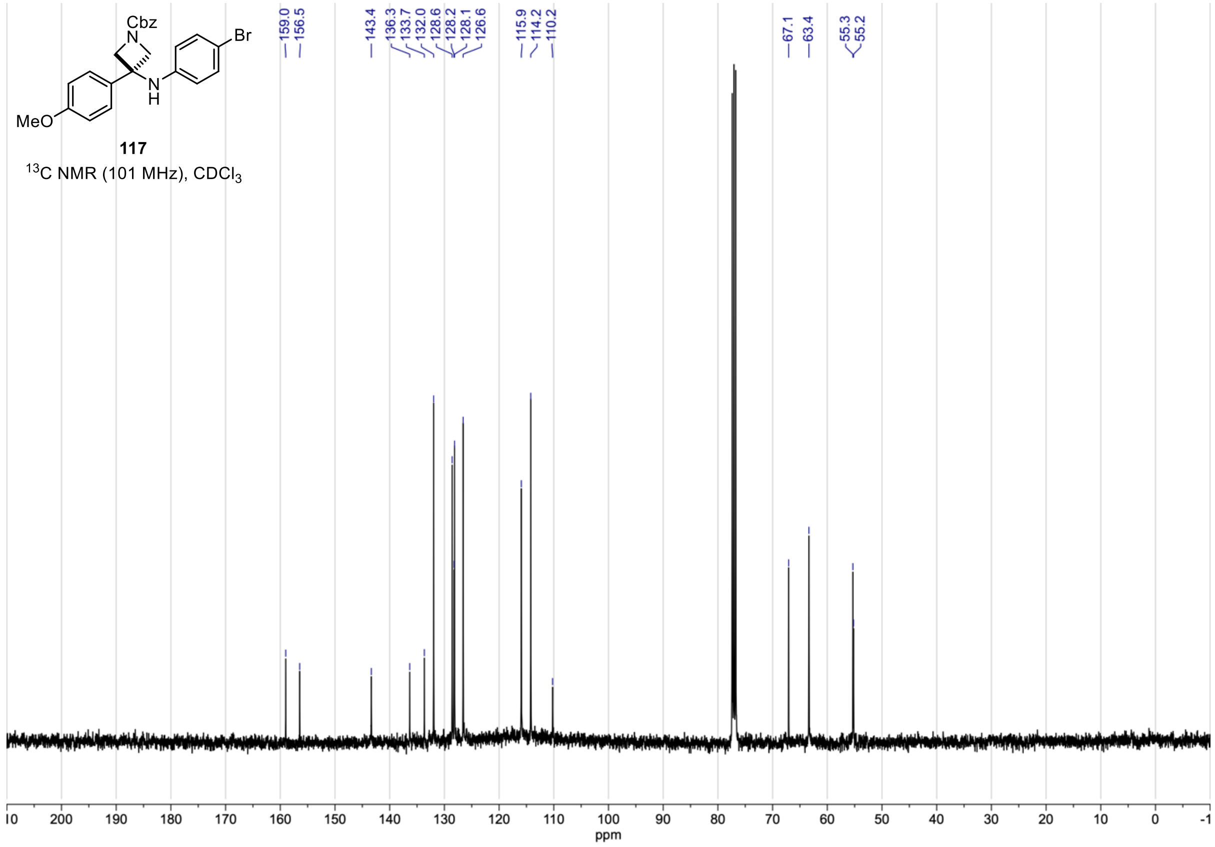
Cbz
N
Br
N
H
MeO
117
13C NMR (101 MHz), CDCl3
ppm

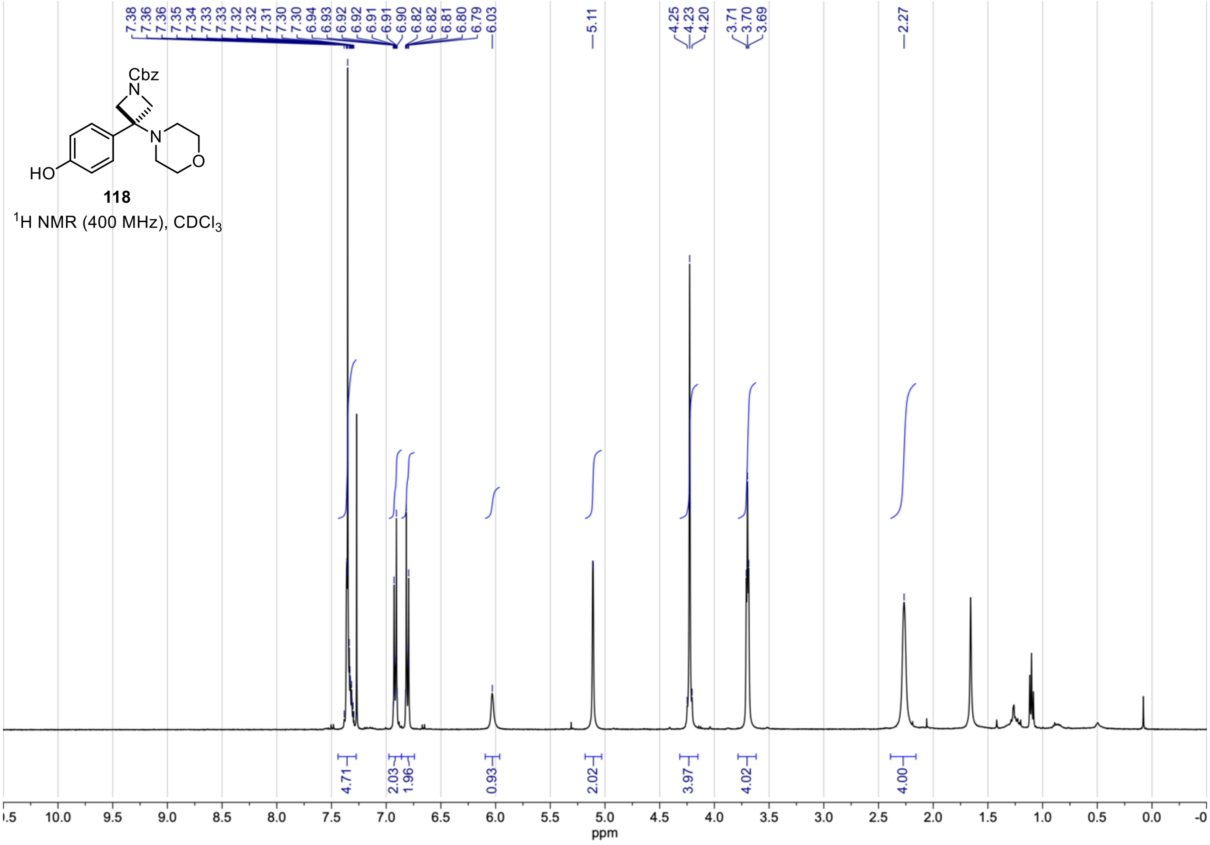

118

^{1}H NMR (400 MHz), $CDCl_3$

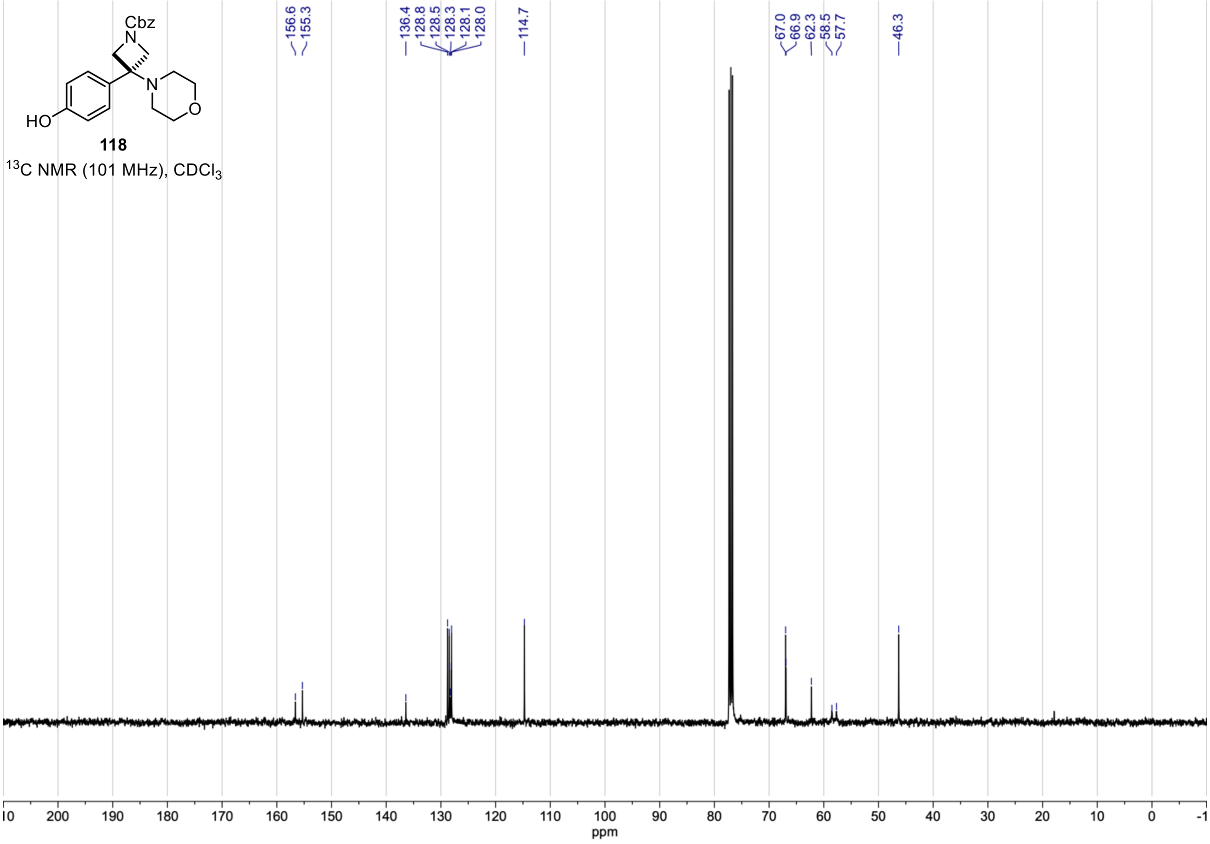

118

^{13}C NMR (101 MHz), $CDCl_3$

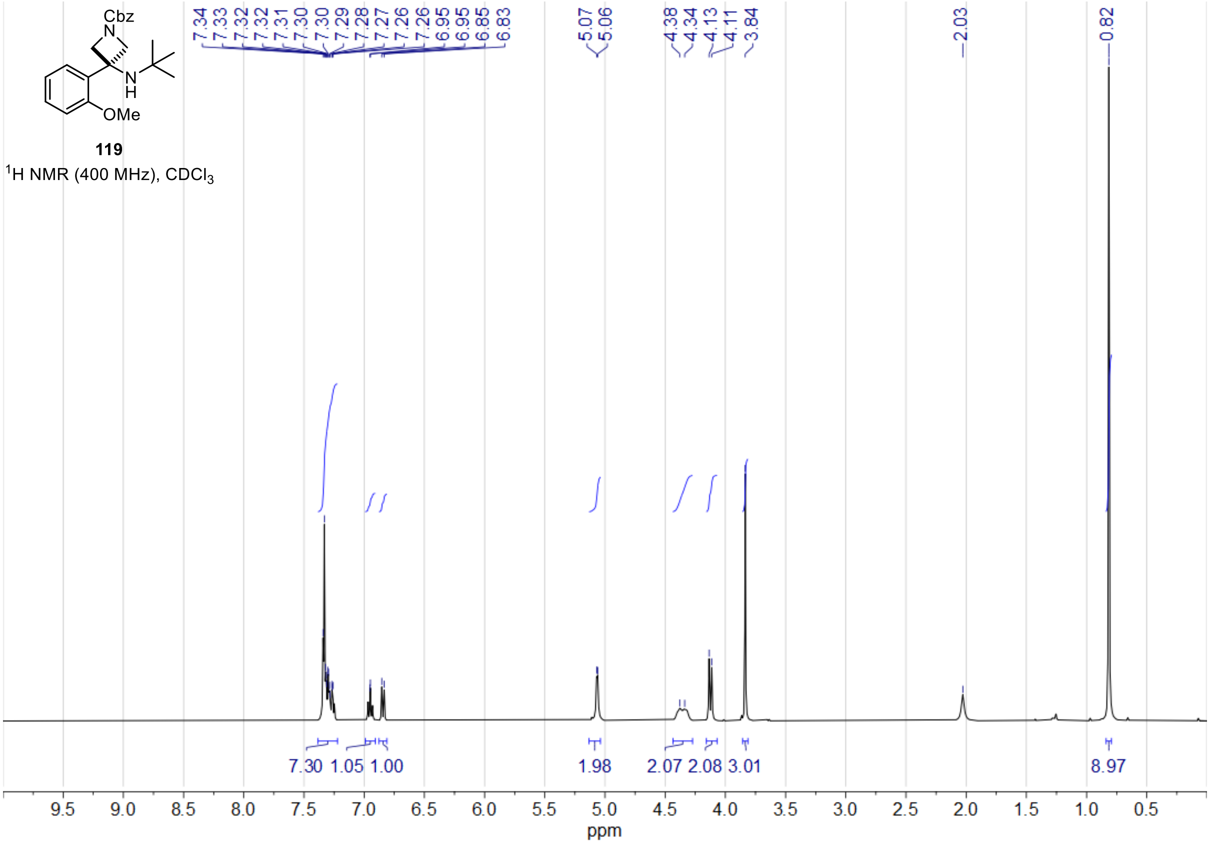

119

^{1}H NMR (400 MHz), $CDCl_3$

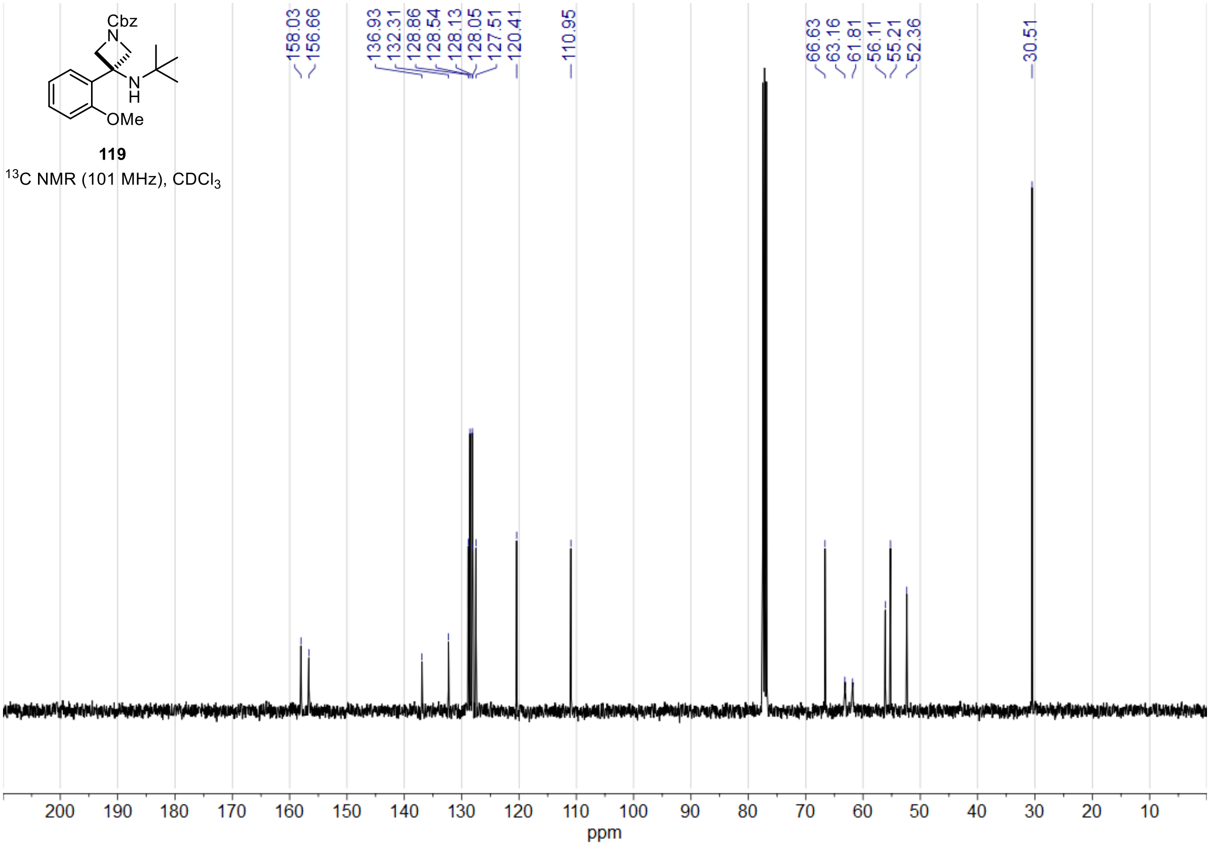

119

^{13}C NMR (101 MHz), $CDCl_3$

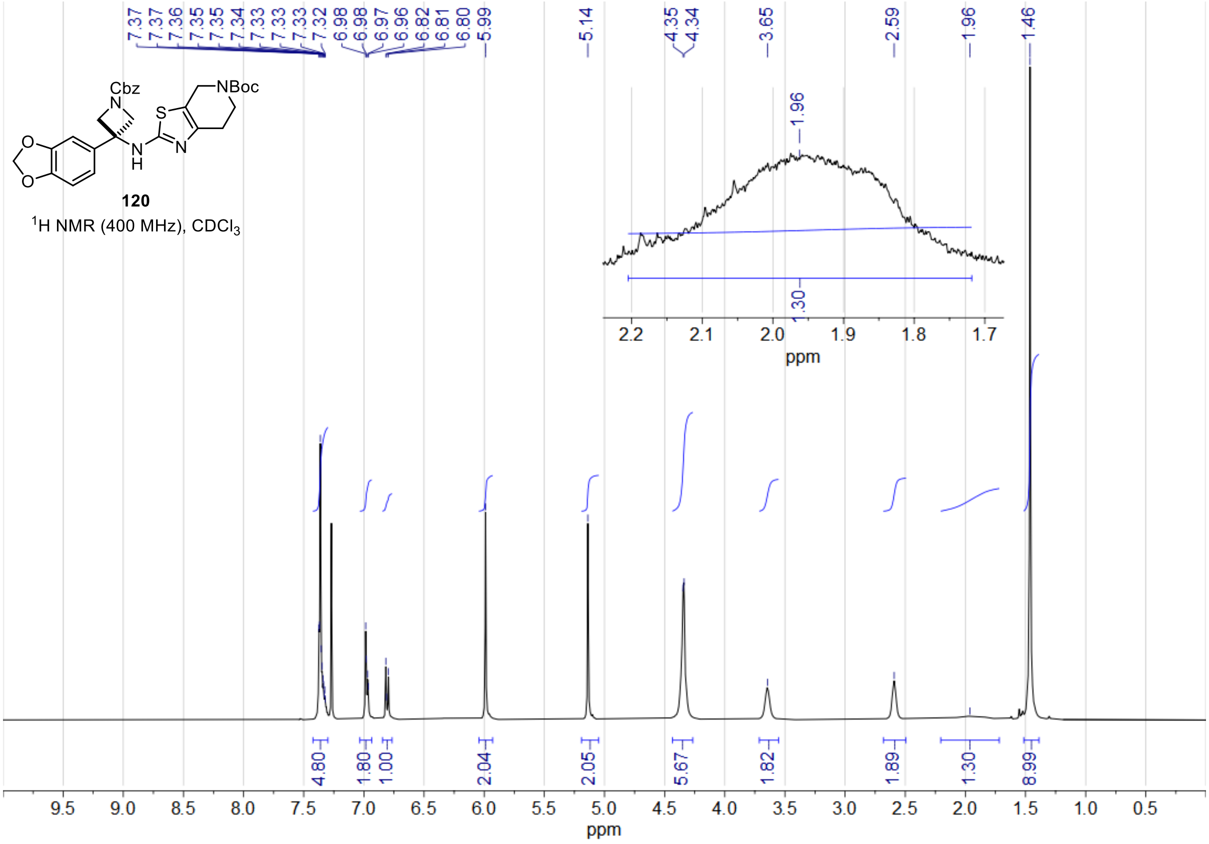
Cbz
NBoc
120
^{1}H NMR (400 MHz), CDCl$_3$
ppm

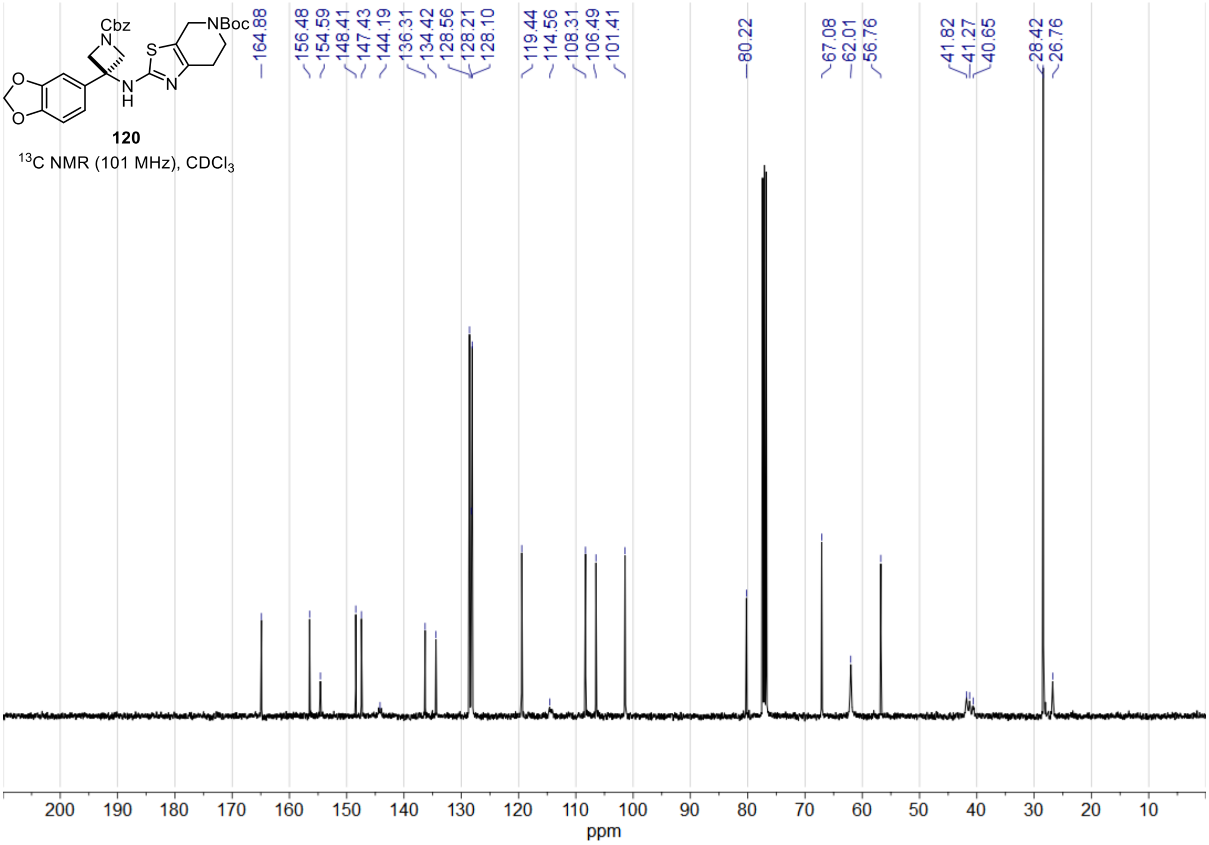
Cbz
NBoc
120
^{13}C NMR (101 MHz), CDCl$_3$
ppm

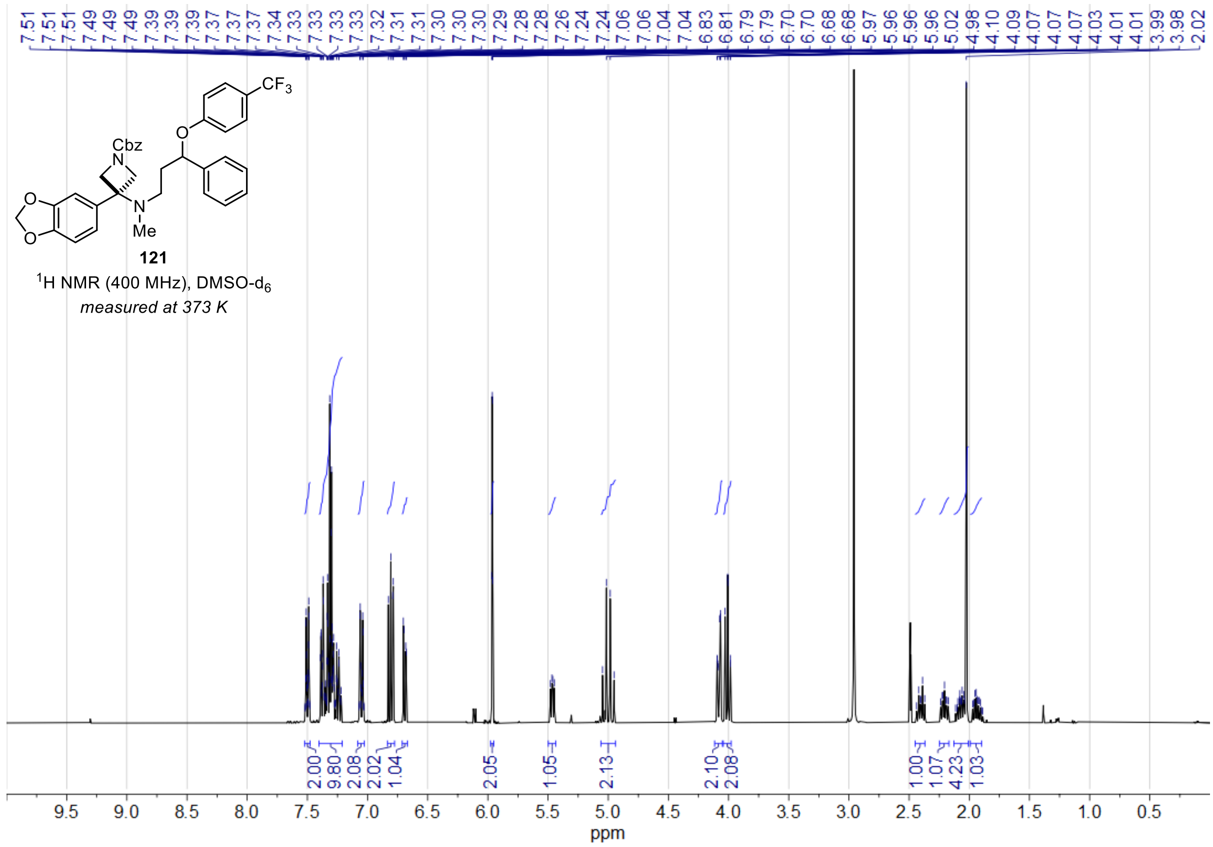
CF3
Cbz
N
O
O
O
N
Me
121
1H NMR (400 MHz), DMSO-d6
measured at 373 K
ppm

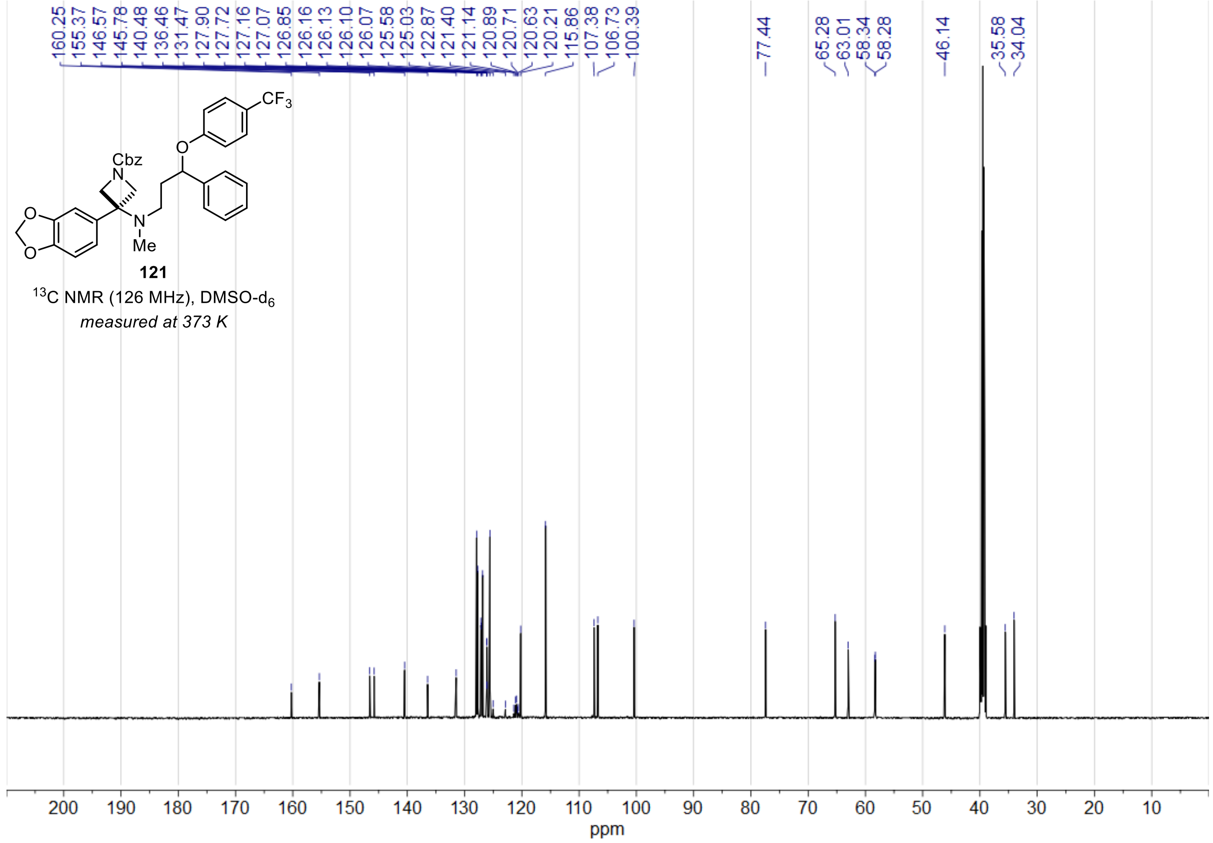
CF3
Cbz
N
O
O
O
N
Me
121
13C NMR (126 MHz), DMSO-d6
measured at 373 K
ppm

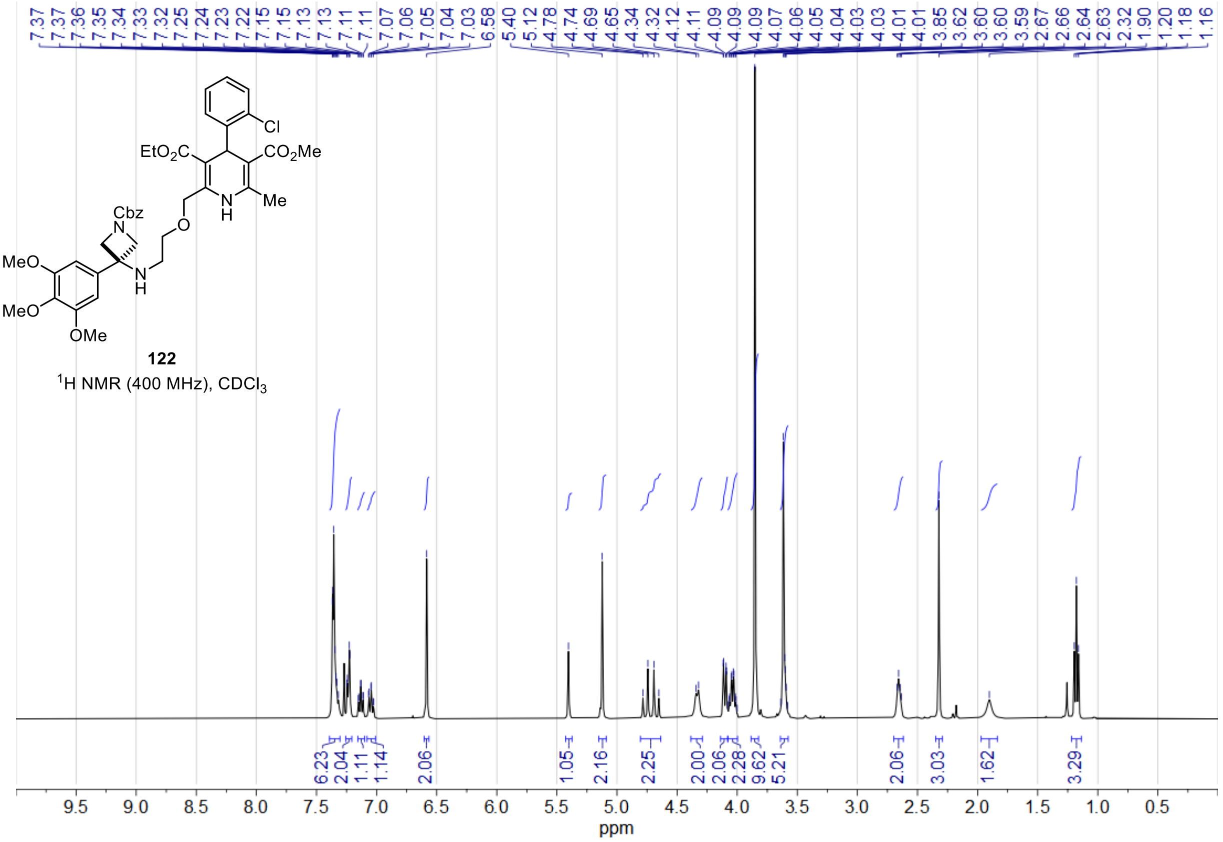

122

^{1}H NMR (400 MHz), $CDCl_3$

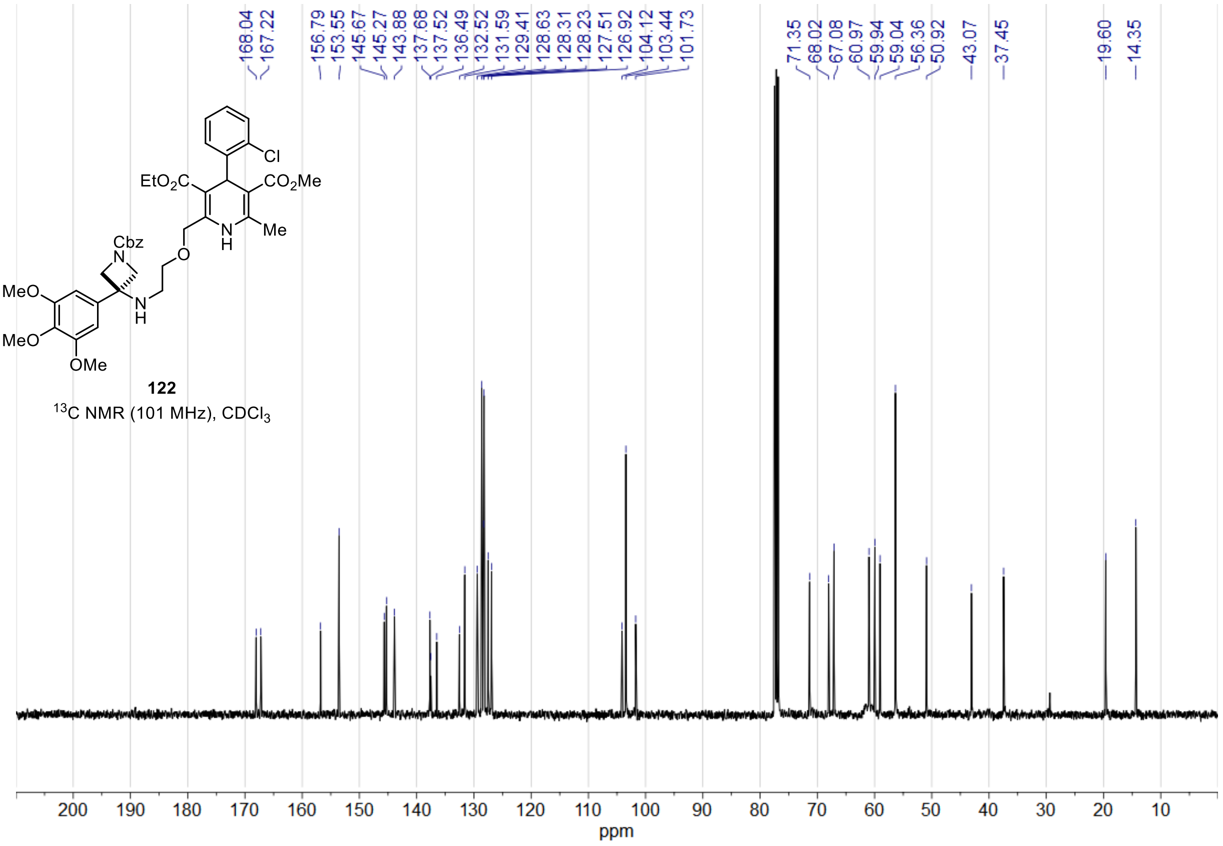

122

^{13}C NMR (101 MHz), $CDCl_3$

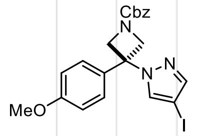

123

^{1}H NMR (400 MHz), $CDCl_3$

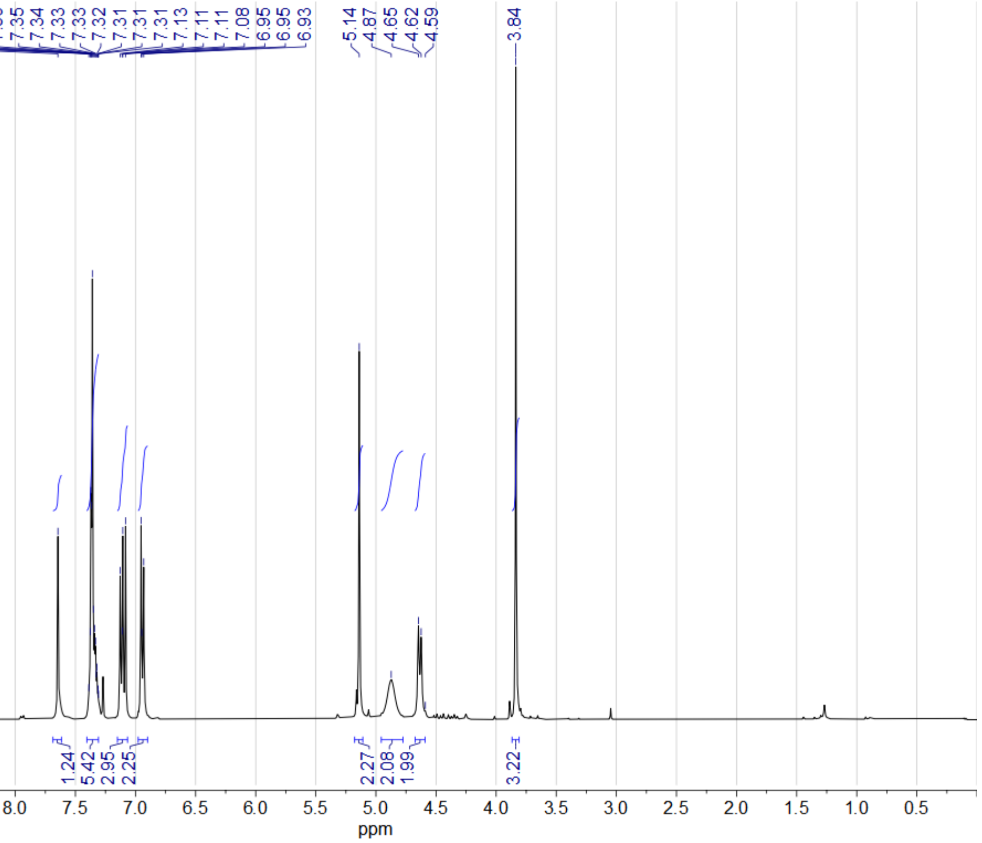

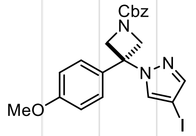

123

^{13}C NMR (101 MHz), $CDCl_3$

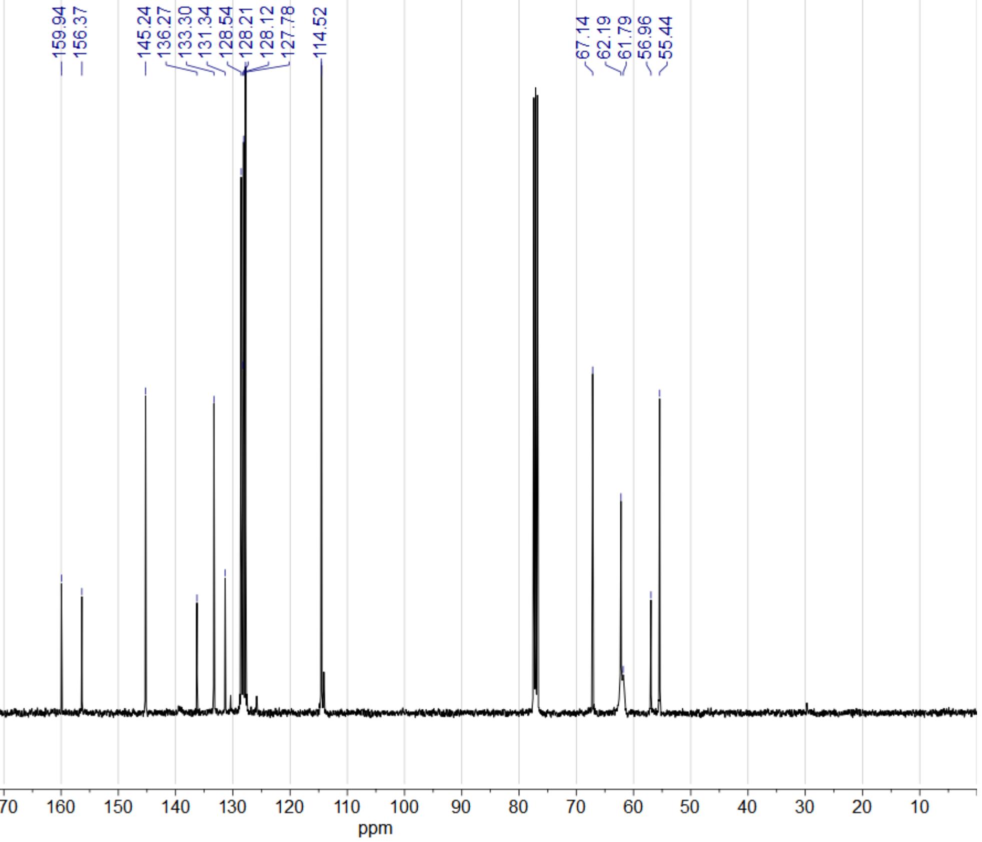

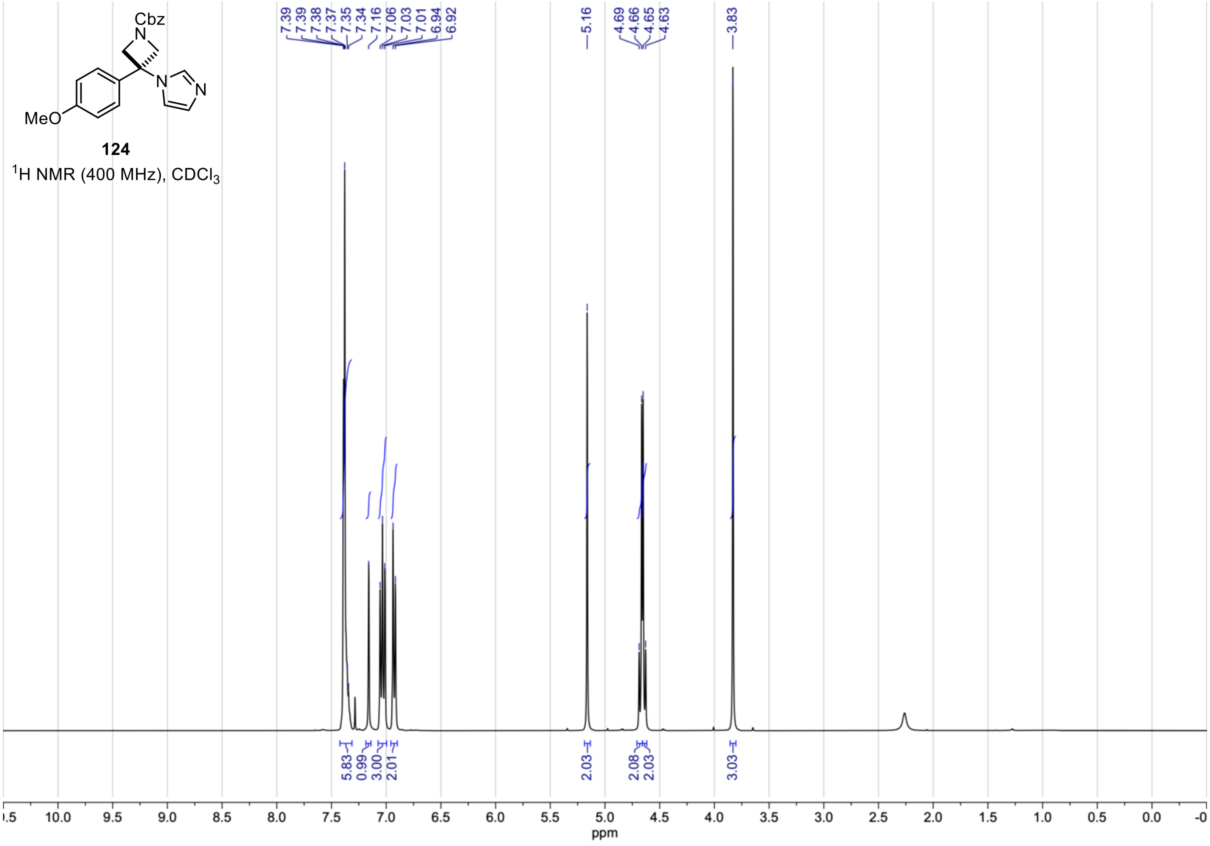
Cbz
N
N
N
MeO
124
^{1}H NMR (400 MHz), CDCl3
7.39
7.39
7.38
7.37
7.35
7.34
7.16
7.06
7.03
7.01
6.94
6.92
5.16
4.69
4.66
4.65
4.63
3.83
5.83
0.99
3.00
2.01
2.03
2.08
2.03
3.03
ppm

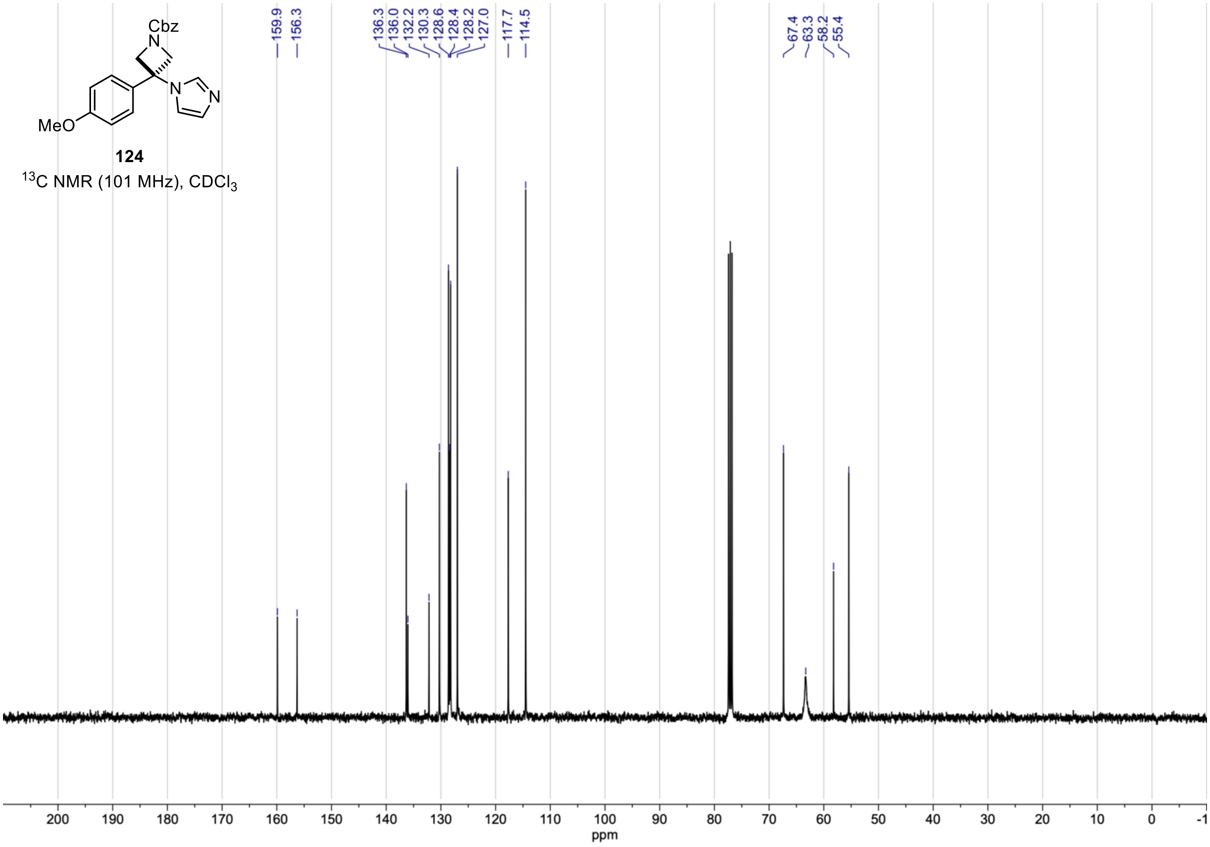
Cbz
N
N
N
MeO
124
^{13}C NMR (101 MHz), CDCl3
159.9
156.3
136.3
136.0
132.2
130.3
128.6
128.4
128.2
127.0
117.7
114.5
67.4
63.3
58.2
55.4
ppm

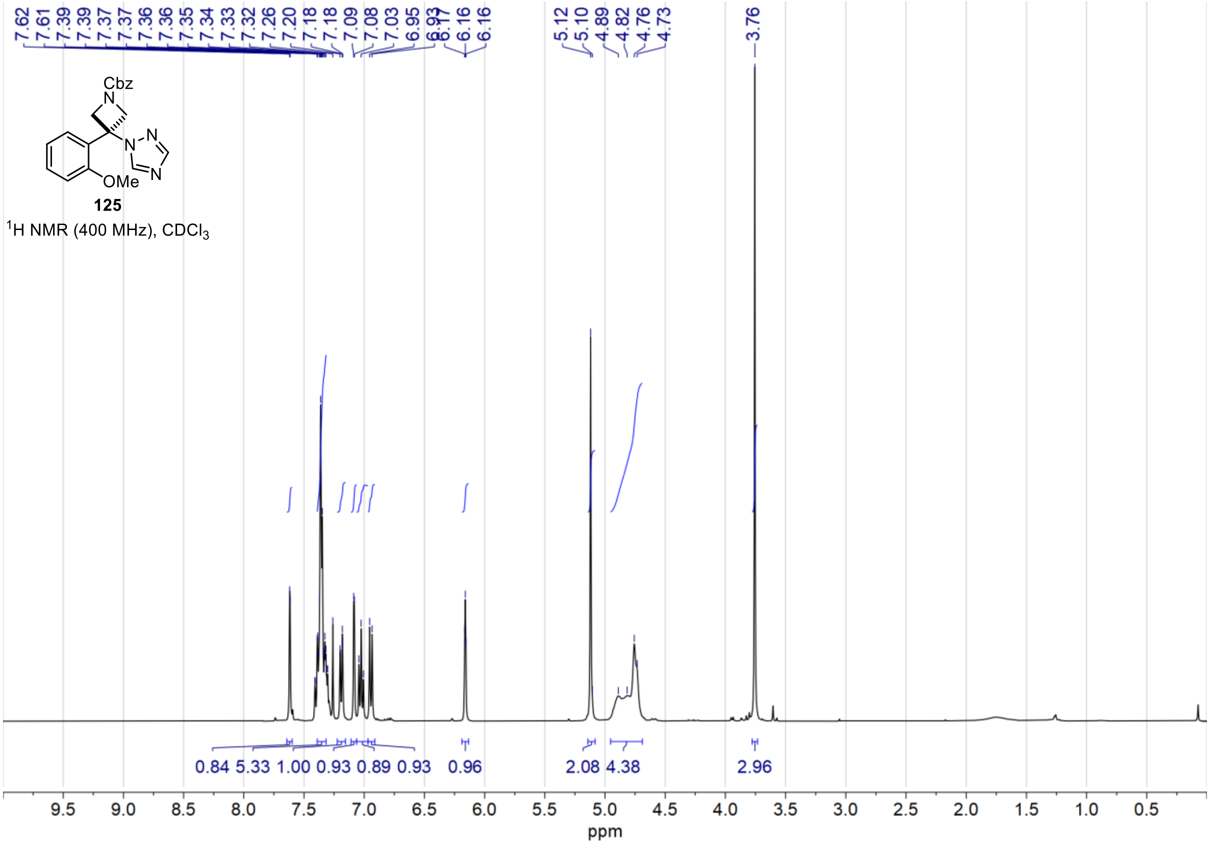
7.62
7.61
7.39
7.39
7.37
7.37
7.36
7.36
7.35
7.34
7.33
7.32
7.26
7.20
7.18
7.18
7.09
7.08
7.03
6.95
6.93
6.17
6.16
6.16
5.12
5.10
4.89
4.82
4.76
4.73
3.76
Cbz
N
N
N
N
OMe
125
^{1}H NMR (400 MHz), CDCl$_3$
0.84
5.33
1.00
0.93
0.89
0.93
0.96
2.08
4.38
2.96
9.5
9.0
8.5
8.0
7.5
7.0
6.5
6.0
5.5
5.0
4.5
4.0
3.5
3.0
2.5
2.0
1.5
1.0
0.5
ppm

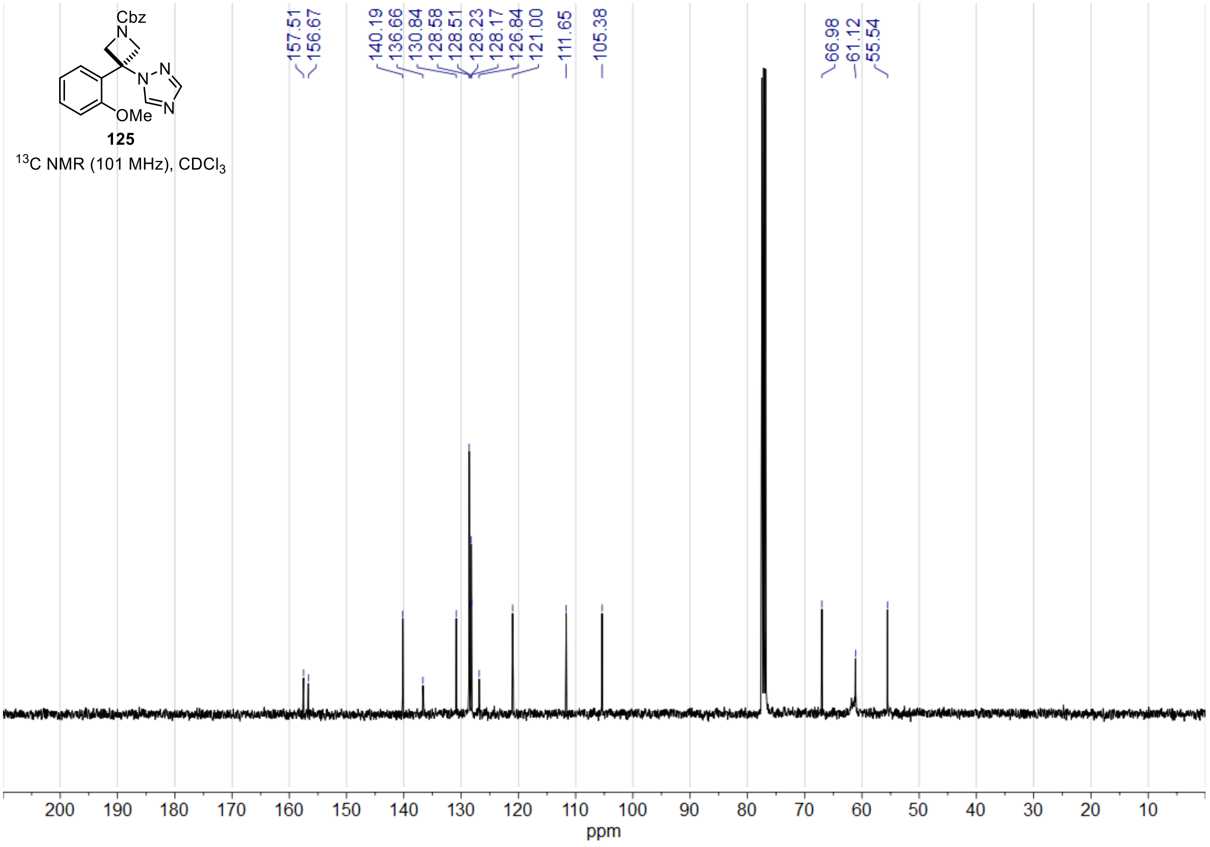
Cbz
N
N
N
N
OMe
125
^{13}C NMR (101 MHz), CDCl$_3$
157.51
156.67
140.19
136.66
130.84
128.58
128.51
128.23
128.17
126.84
121.00
111.65
105.38
66.98
61.12
55.54
200
190
180
170
160
150
140
130
120
110
100
90
80
70
60
50
40
30
20
10
ppm

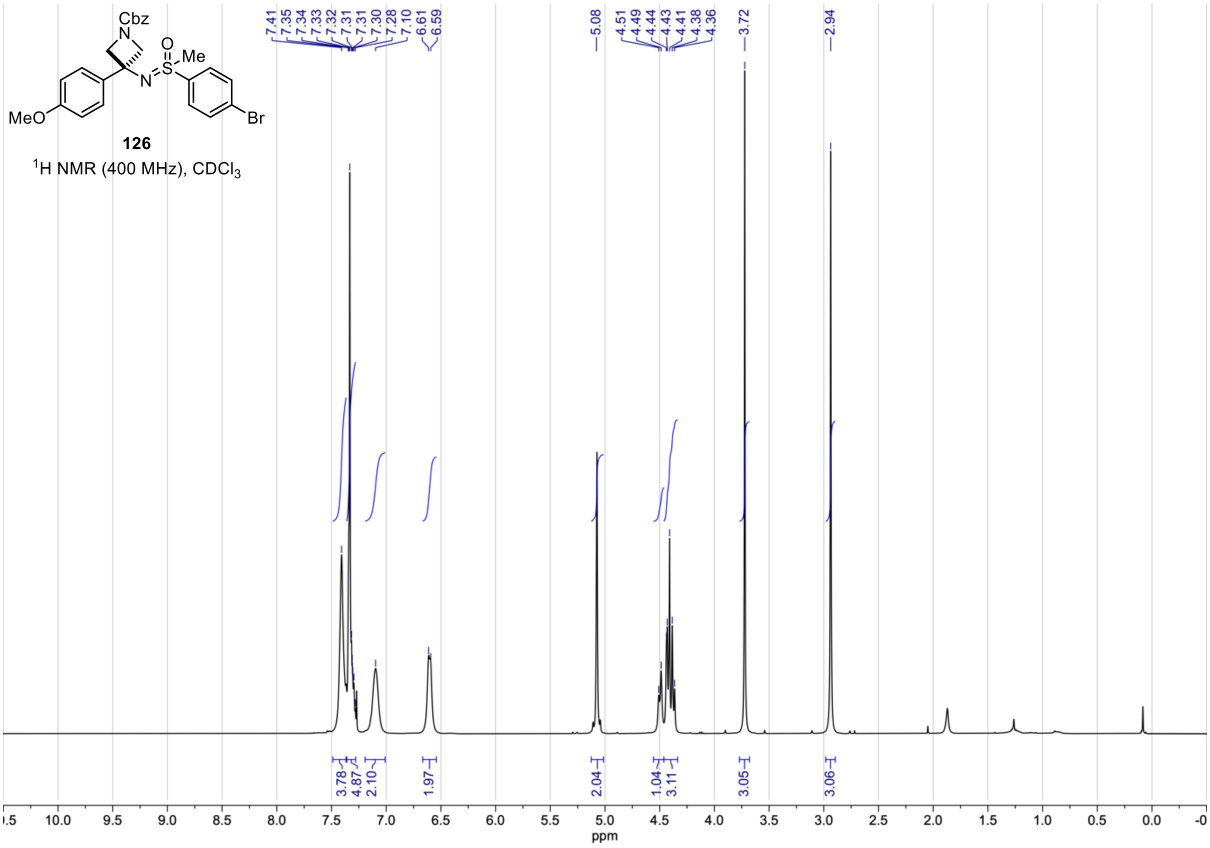

Cbz
N
O
S
Me
N
MeO
Br
126
1H NMR (400 MHz), CDCl3
7.41
7.35
7.34
7.33
7.32
7.31
7.31
7.30
7.28
7.10
6.61
6.59
5.08
4.51
4.49
4.44
4.43
4.41
4.38
4.36
3.72
2.94
3.78
4.87
2.10
1.97
2.04
1.04
3.11
3.05
3.06
10.0
9.5
9.0
8.5
8.0
7.5
7.0
6.5
6.0
5.5
5.0
4.5
4.0
3.5
3.0
2.5
2.0
1.5
1.0
0.5
0.0
ppm

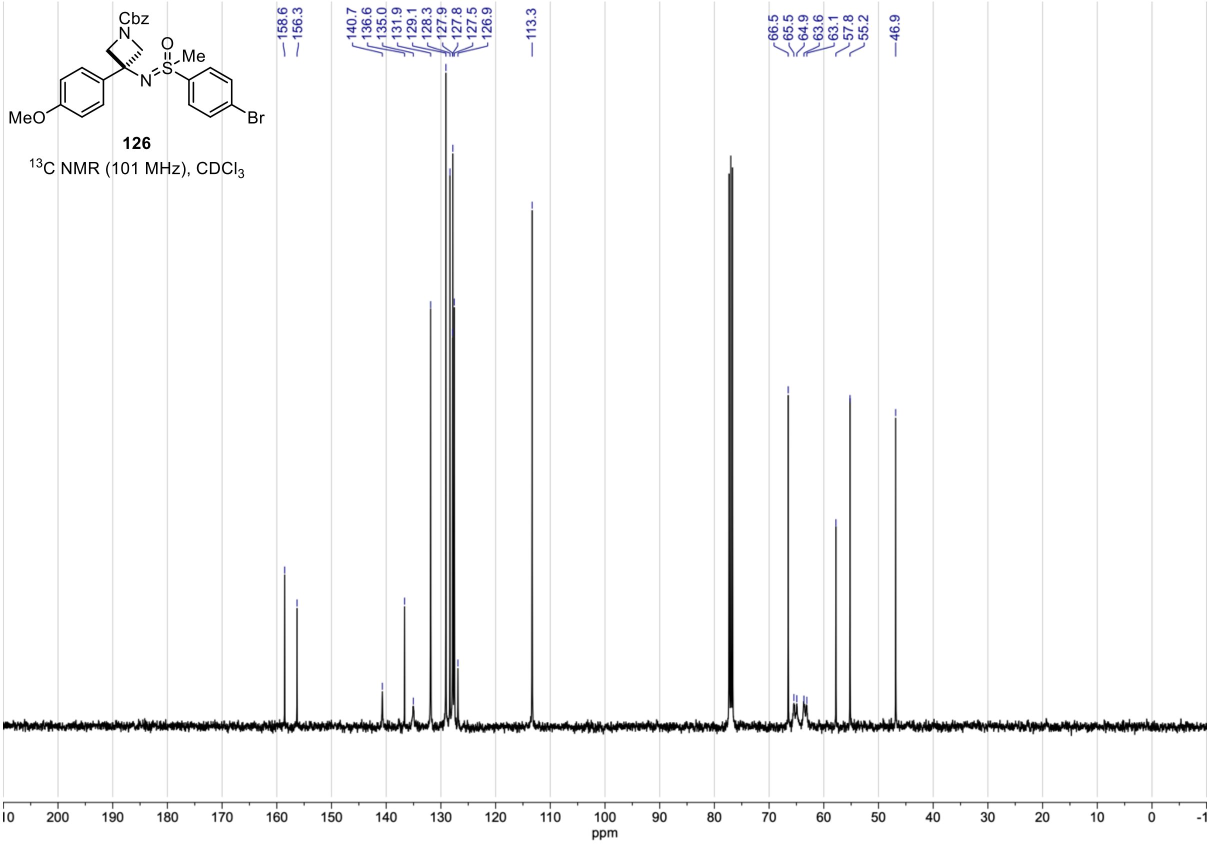

126

^{13}C NMR (101 MHz), $CDCl_3$

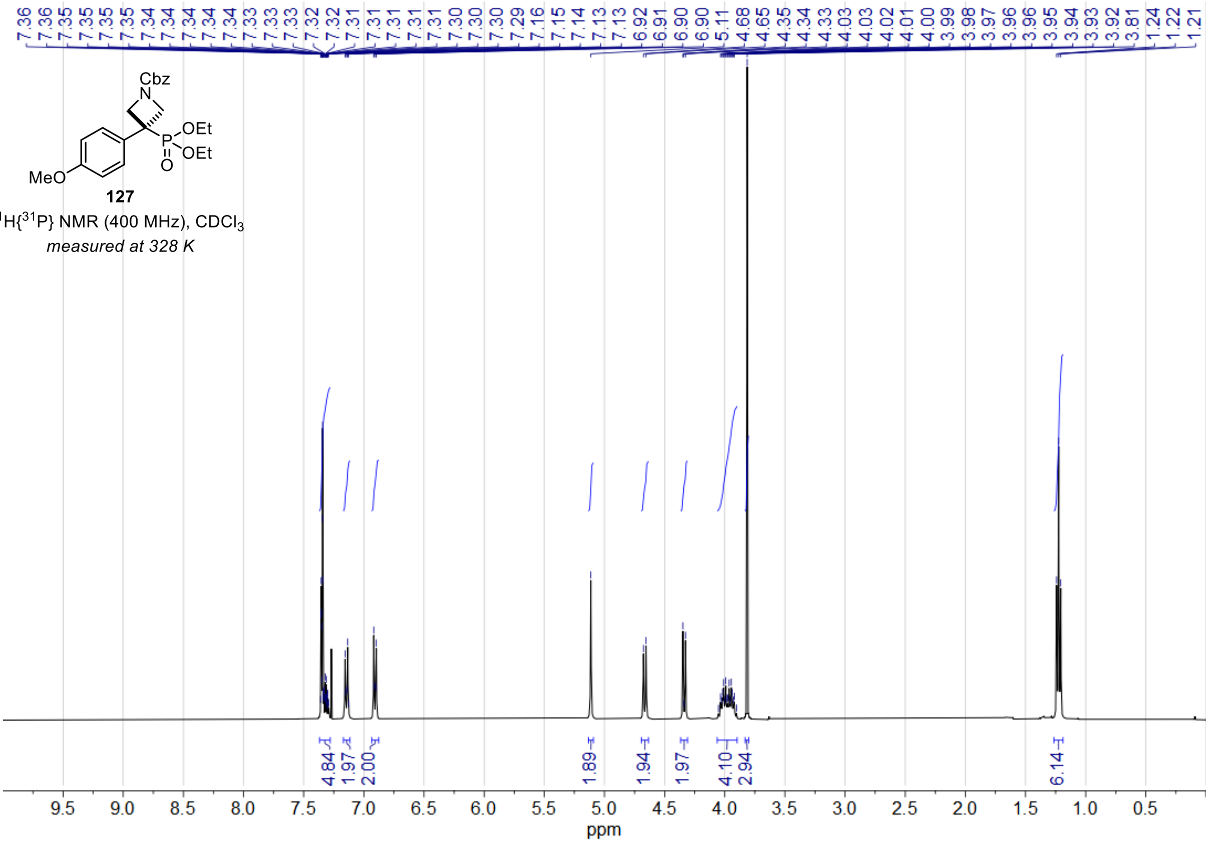

127

$^{1}H\{^{31}P\}$ NMR (400 MHz), $CDCl_3$

measured at 328 K

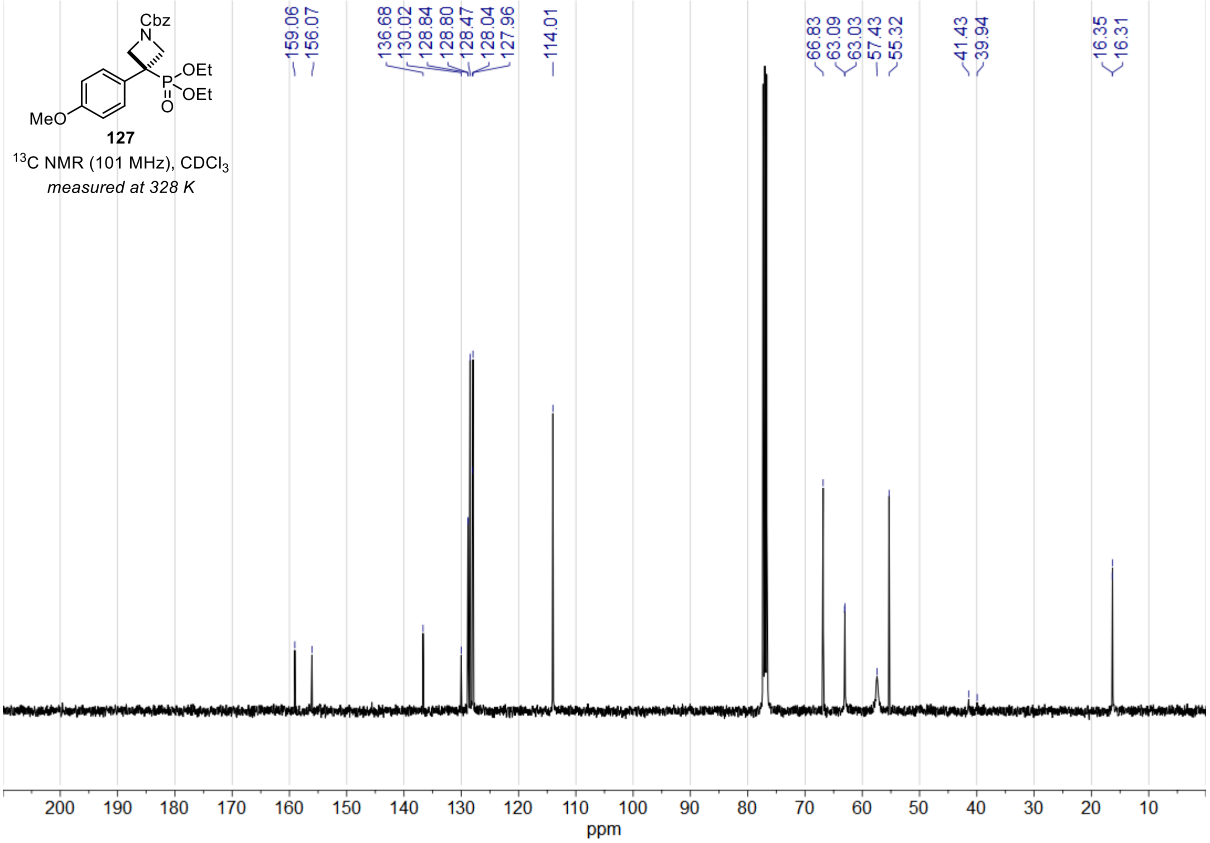

127

^{13}C NMR (101 MHz), $CDCl_3$

measured at 328 K

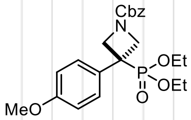

127

$^{31}P\{^1H\}$ NMR (162 MHz), $CDCl_3$

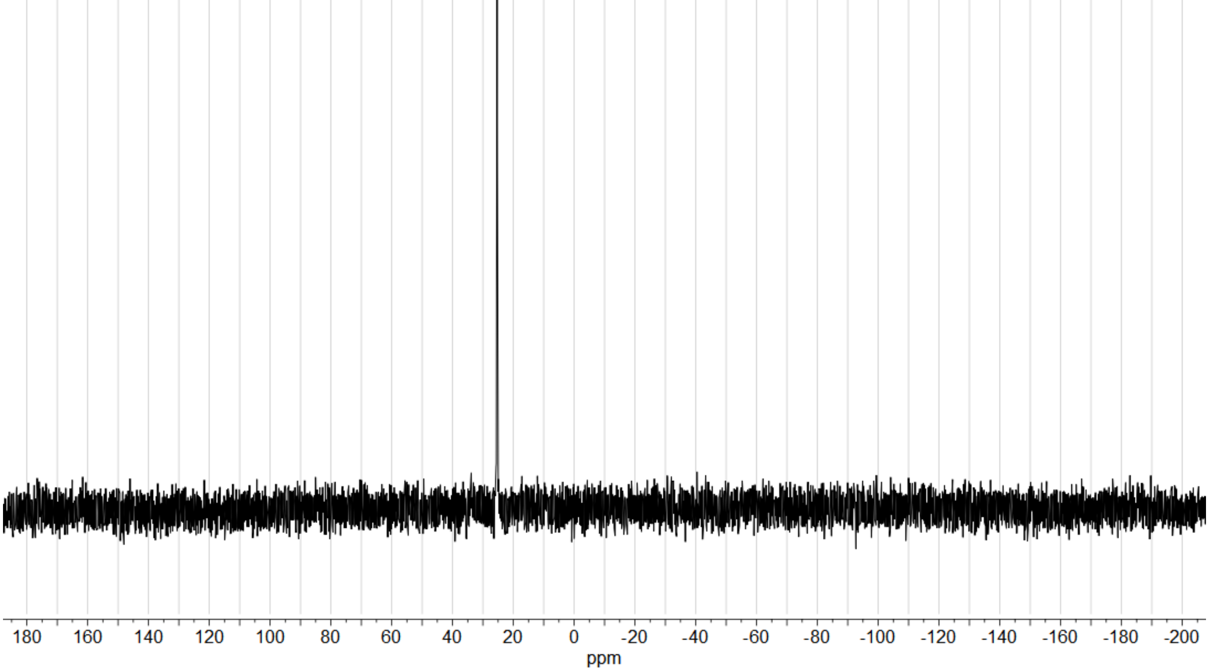

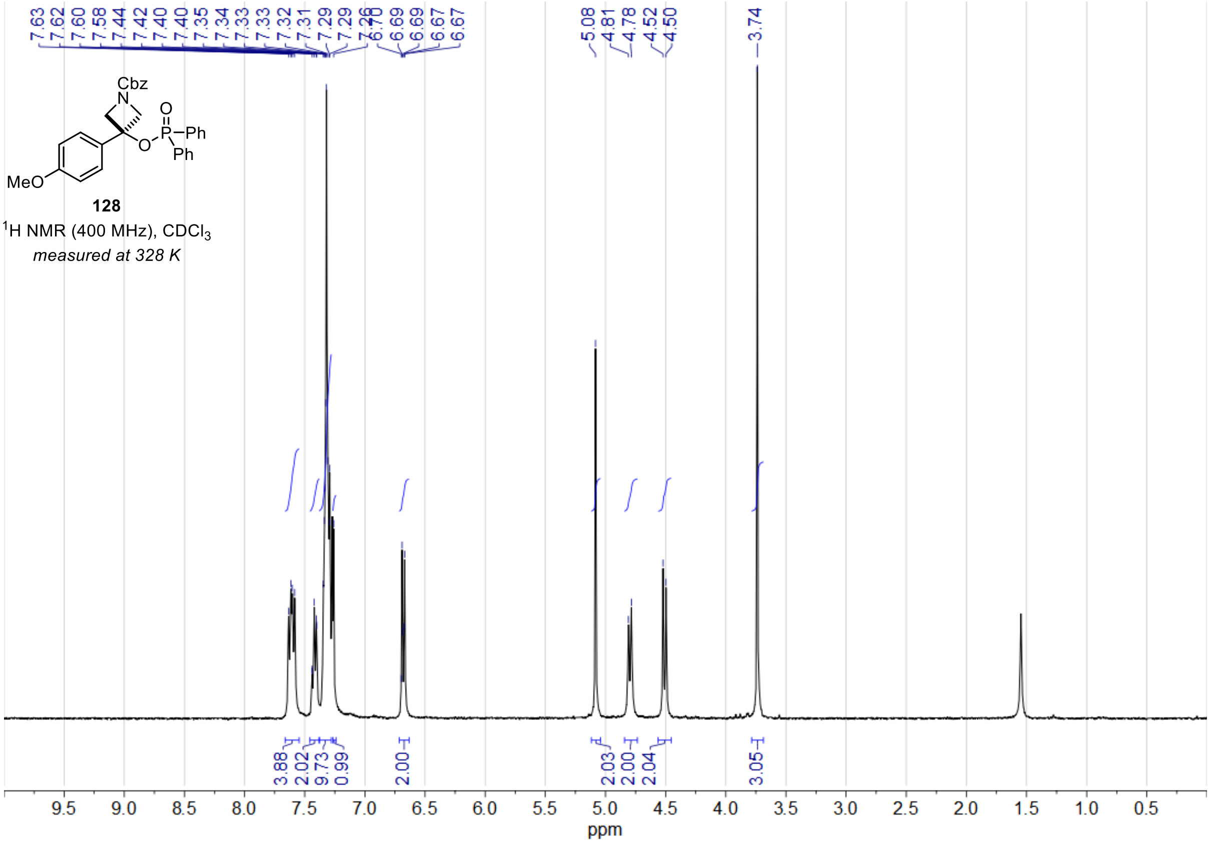

128

^{1}H NMR (400 MHz), $CDCl_3$

measured at 328 K

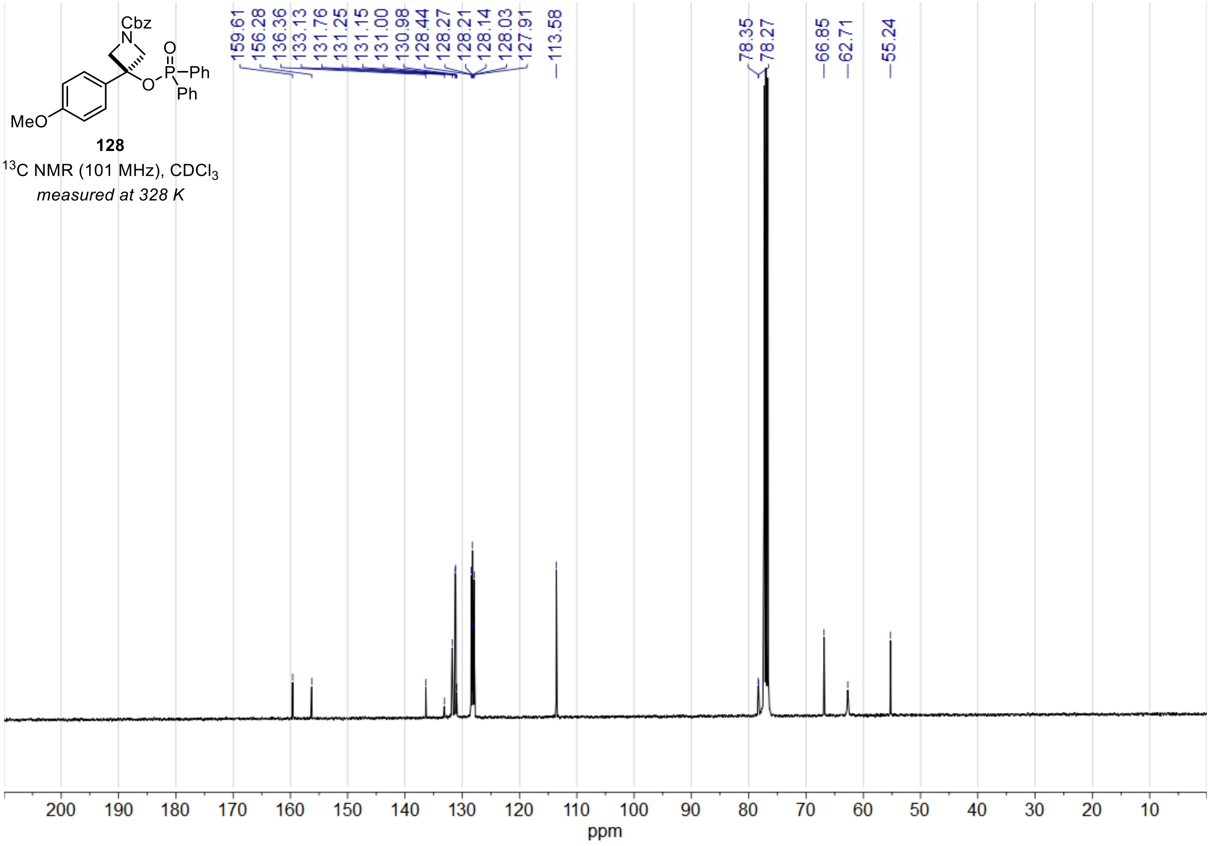

128

^{13}C NMR (101 MHz), $CDCl_3$

measured at 328 K

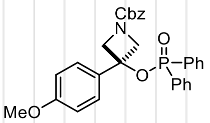

128

$^{31}P\{^{1}H\}$ NMR (162 MHz), $CDCl_3$

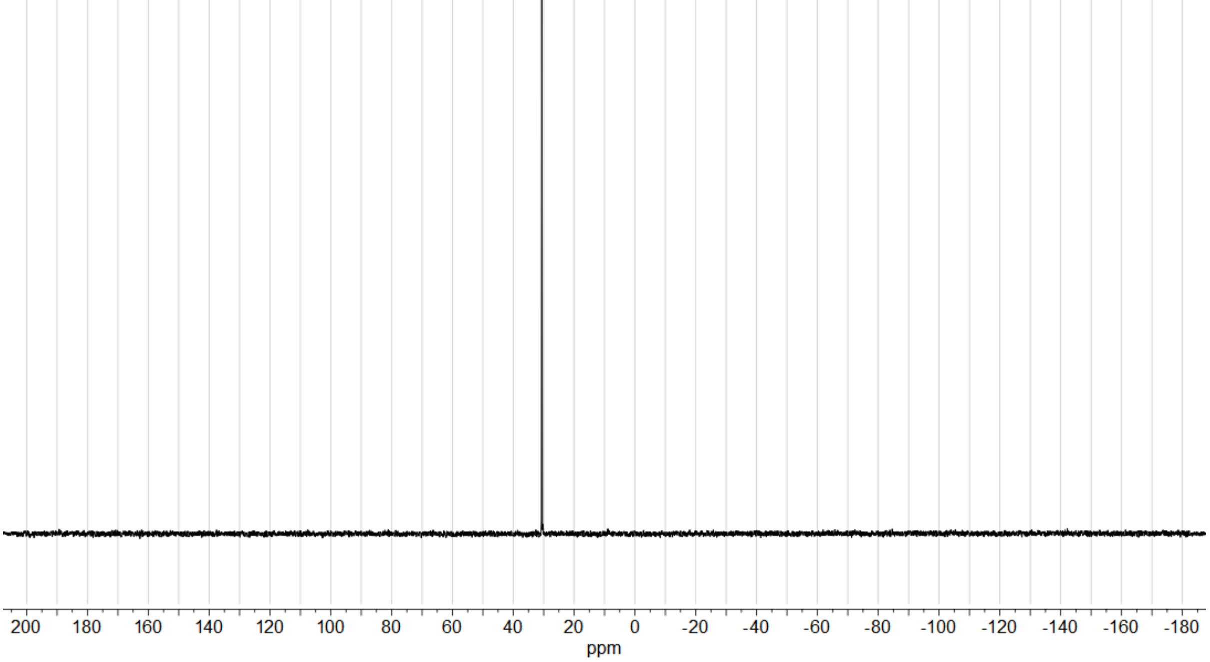

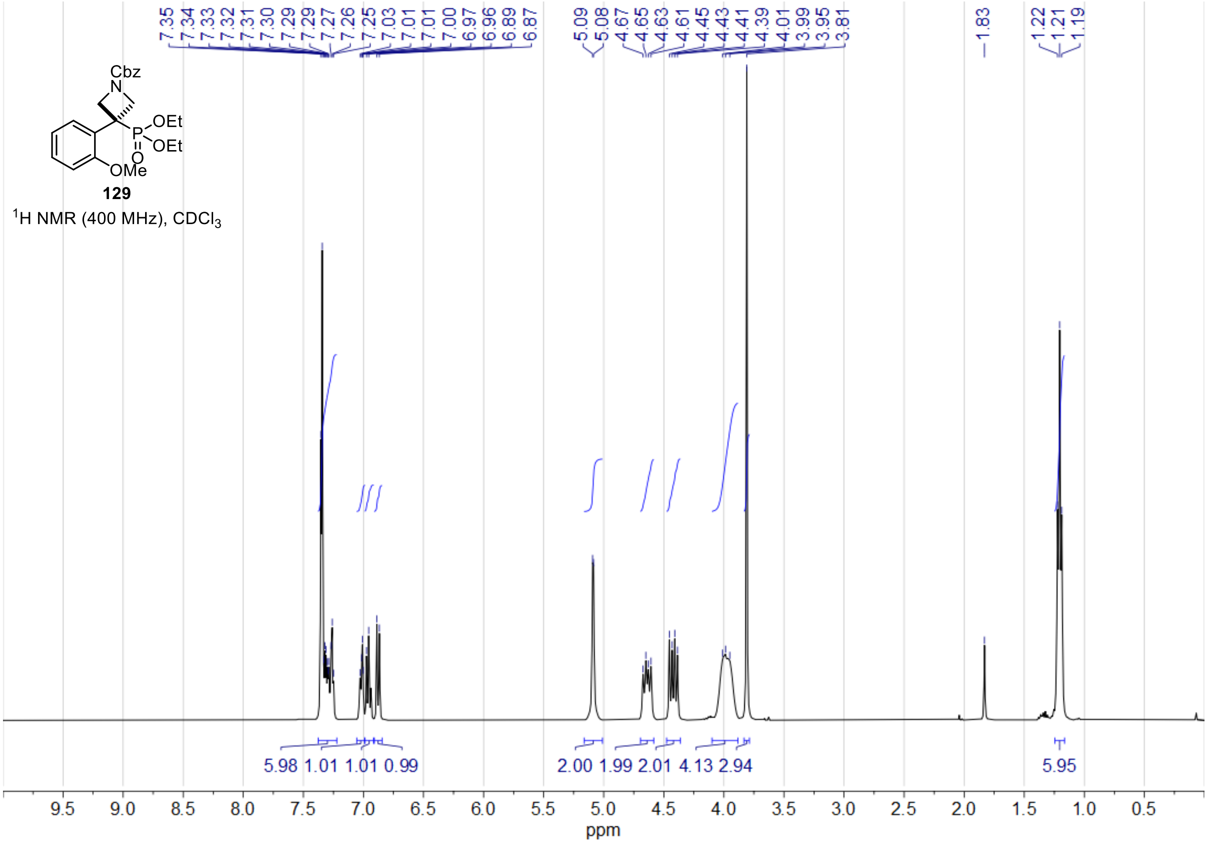
7.35
7.34
7.33
7.32
7.31
7.30
7.29
7.29
7.27
7.26
7.25
7.03
7.01
7.01
7.00
6.97
6.96
6.89
6.87
5.09
5.08
4.67
4.65
4.63
4.61
4.45
4.43
4.41
4.39
4.01
3.99
3.95
3.81
1.83
1.22
1.21
1.19
Cbz
N
P
OEt
OEt
O
OMe
129
^{1}H NMR (400 MHz), $CDCl_3$
5.98 1.01 1.01 0.99
2.00 1.99 2.01 4.13 2.94
5.95
9.5 9.0 8.5 8.0 7.5 7.0 6.5 6.0 5.5 5.0 4.5 4.0 3.5 3.0 2.5 2.0 1.5 1.0 0.5
ppm

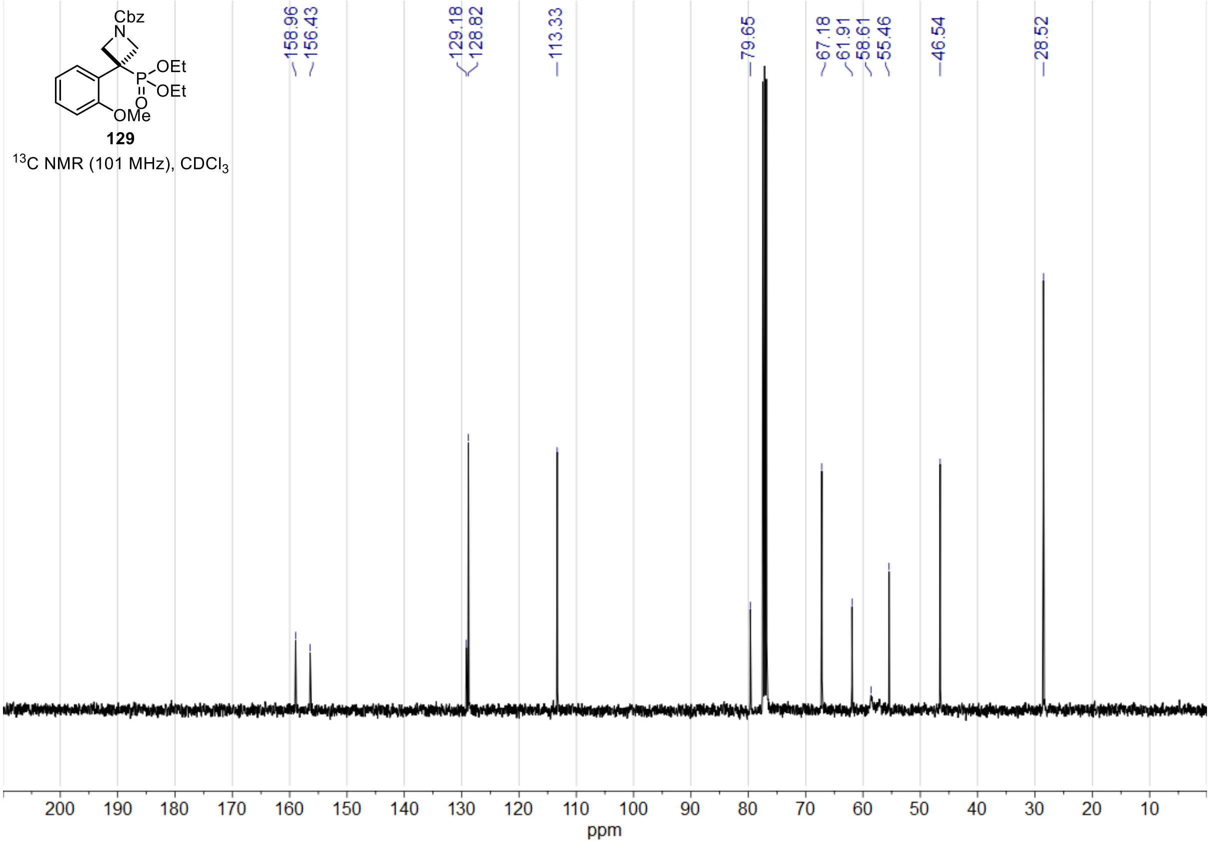
158.96
156.43
129.18
128.82
113.33
79.65
67.18
61.91
58.61
55.46
46.54
28.52
Cbz
N
P
OEt
OEt
O
OMe
129
^{13}C NMR (101 MHz), $CDCl_3$
200 190 180 170 160 150 140 130 120 110 100 90 80 70 60 50 40 30 20 10
ppm

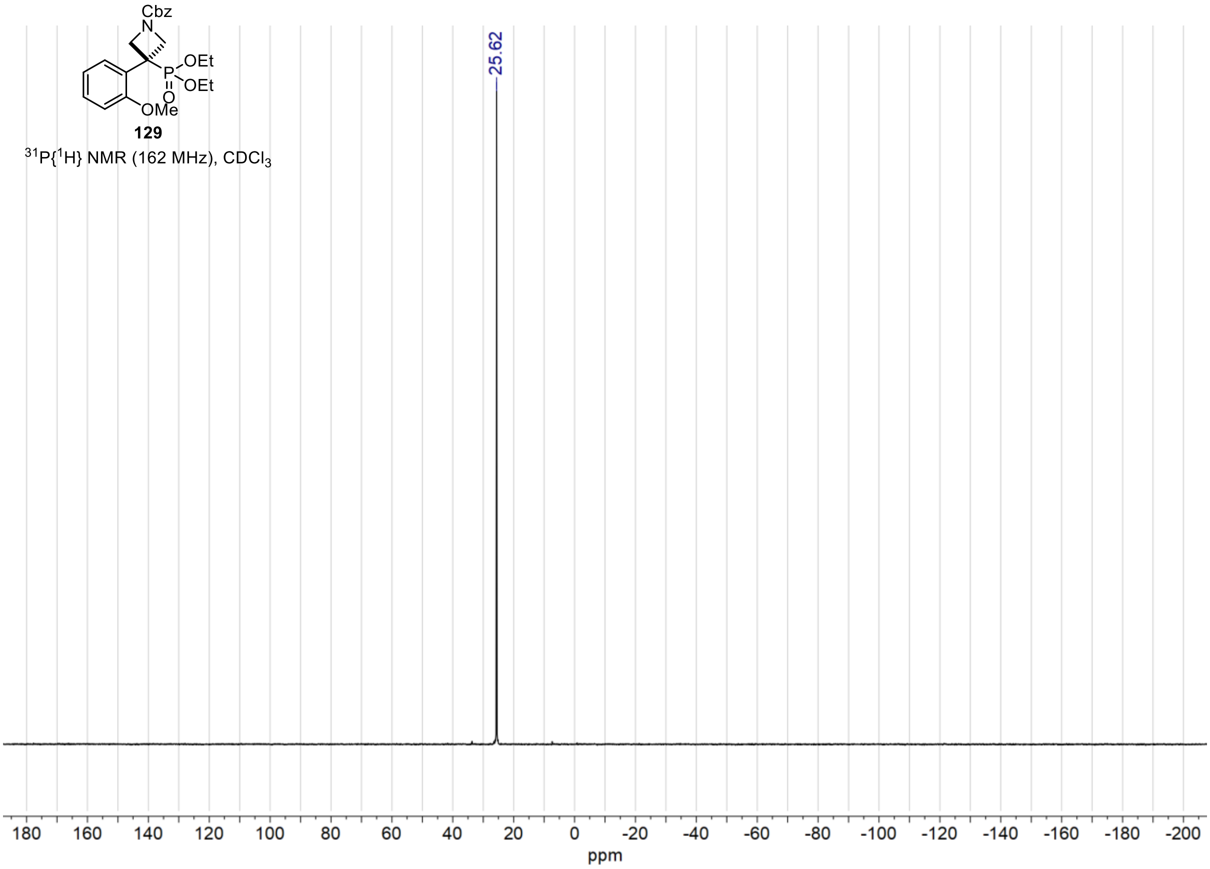

129

$^{31}P\{^{1}H\}$ NMR (162 MHz), $CDCl_3$

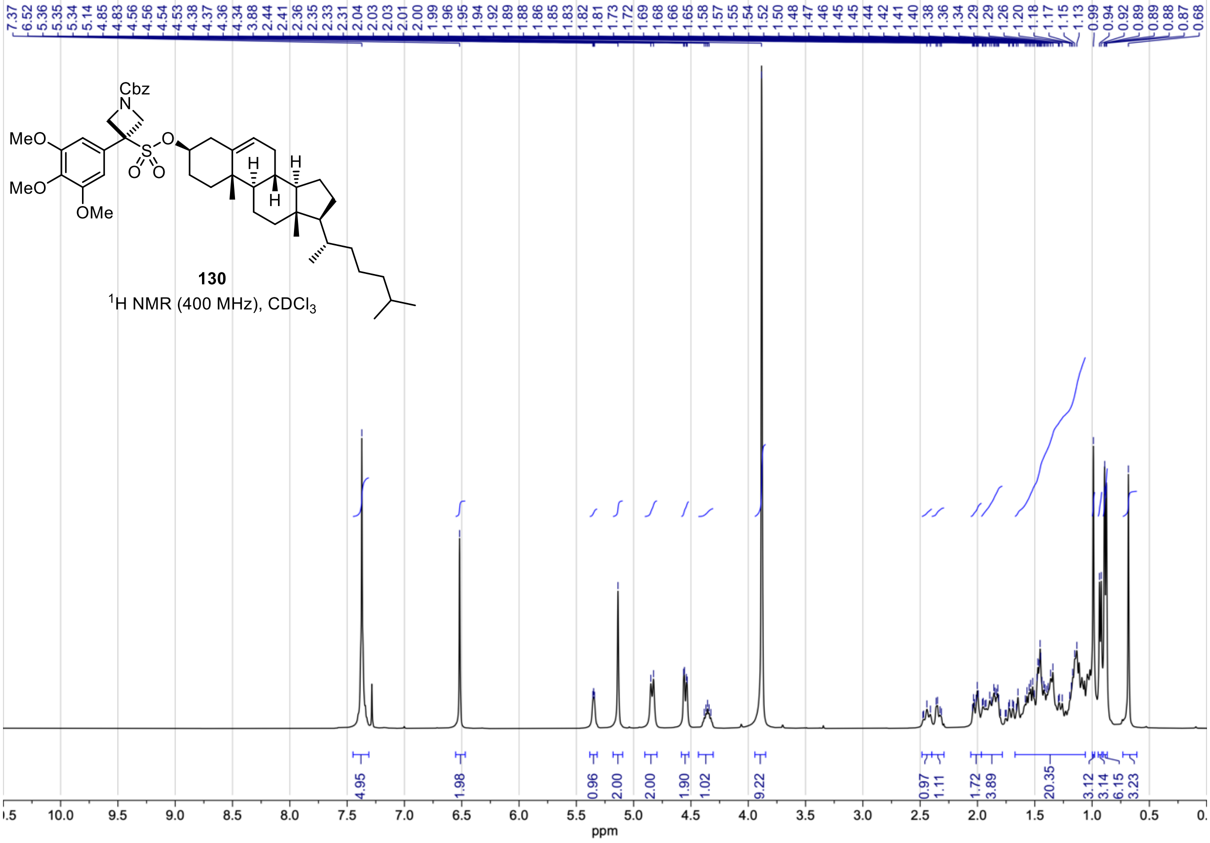
7.37
6.52
5.36
5.35
5.34
5.14
4.85
4.83
4.56
4.56
4.54
4.53
4.38
4.37
4.36
4.34
3.88
2.44
2.41
2.36
2.35
2.33
2.31
2.04
2.03
2.03
2.01
2.00
1.99
1.96
1.95
1.94
1.92
1.89
1.88
1.86
1.85
1.83
1.82
1.81
1.73
1.72
1.69
1.68
1.66
1.65
1.58
1.57
1.55
1.54
1.52
1.50
1.48
1.47
1.46
1.45
1.45
1.44
1.42
1.41
1.40
1.38
1.36
1.34
1.29
1.29
1.26
1.20
1.18
1.17
1.15
1.13
0.99
0.94
0.92
0.89
0.88
0.87
0.68
Cbz
N
MeO
MeO
OMe
S
O
O
O
H
H
H
130
^{1}H NMR (400 MHz), $CDCl_3$
4.95
1.98
0.96
2.00
2.00
1.90
1.02
9.22
0.97
1.11
1.72
3.89
20.35
3.12
3.14
6.15
3.23
ppm

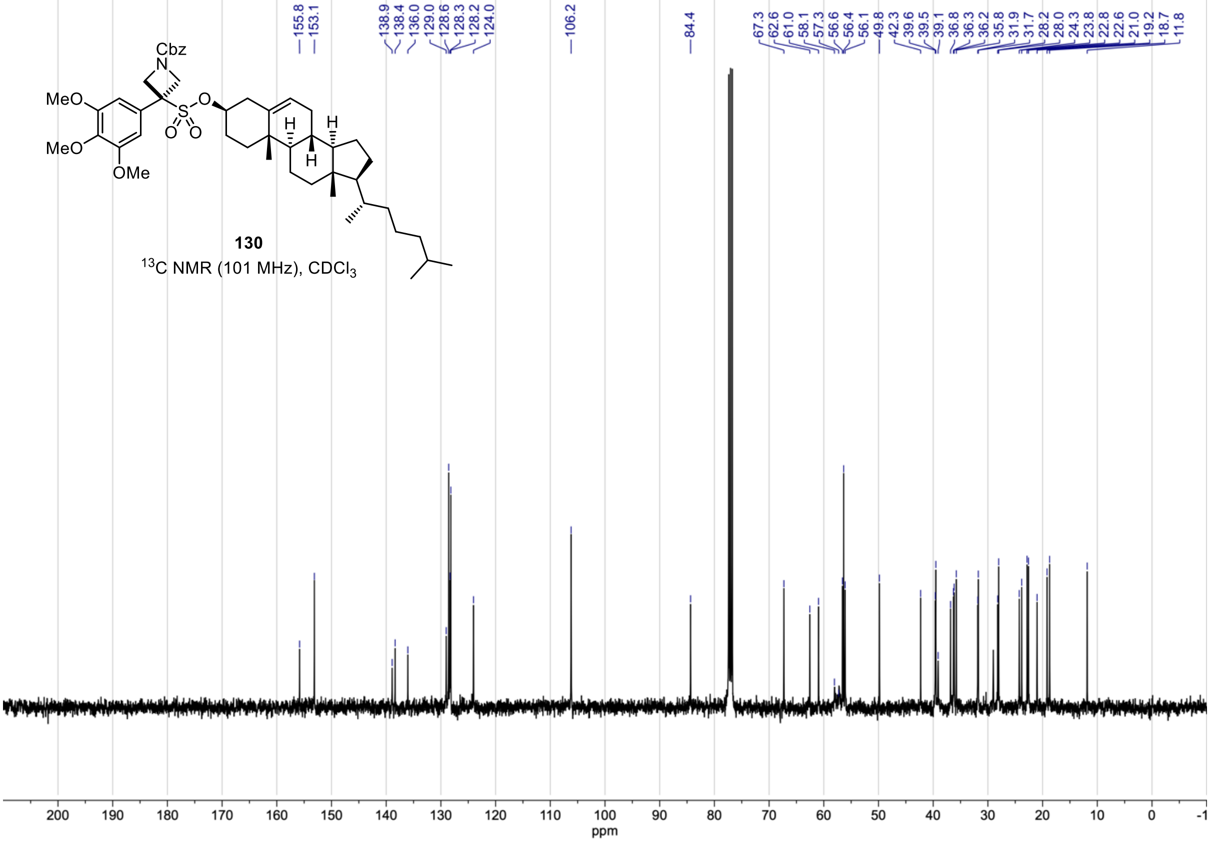
155.8
153.1
138.9
138.4
136.0
129.0
128.6
128.3
128.2
124.0
106.2
84.4
67.3
62.6
61.0
58.1
57.3
56.6
56.4
56.1
49.8
42.3
39.6
39.5
39.1
36.8
36.3
36.2
35.8
31.9
31.7
28.2
28.0
24.3
23.8
22.8
22.6
21.0
19.2
18.7
11.8
Cbz
N
MeO
MeO
OMe
S
O
O
O
H
H
H
130
^{13}C NMR (101 MHz), $CDCl_3$
ppm

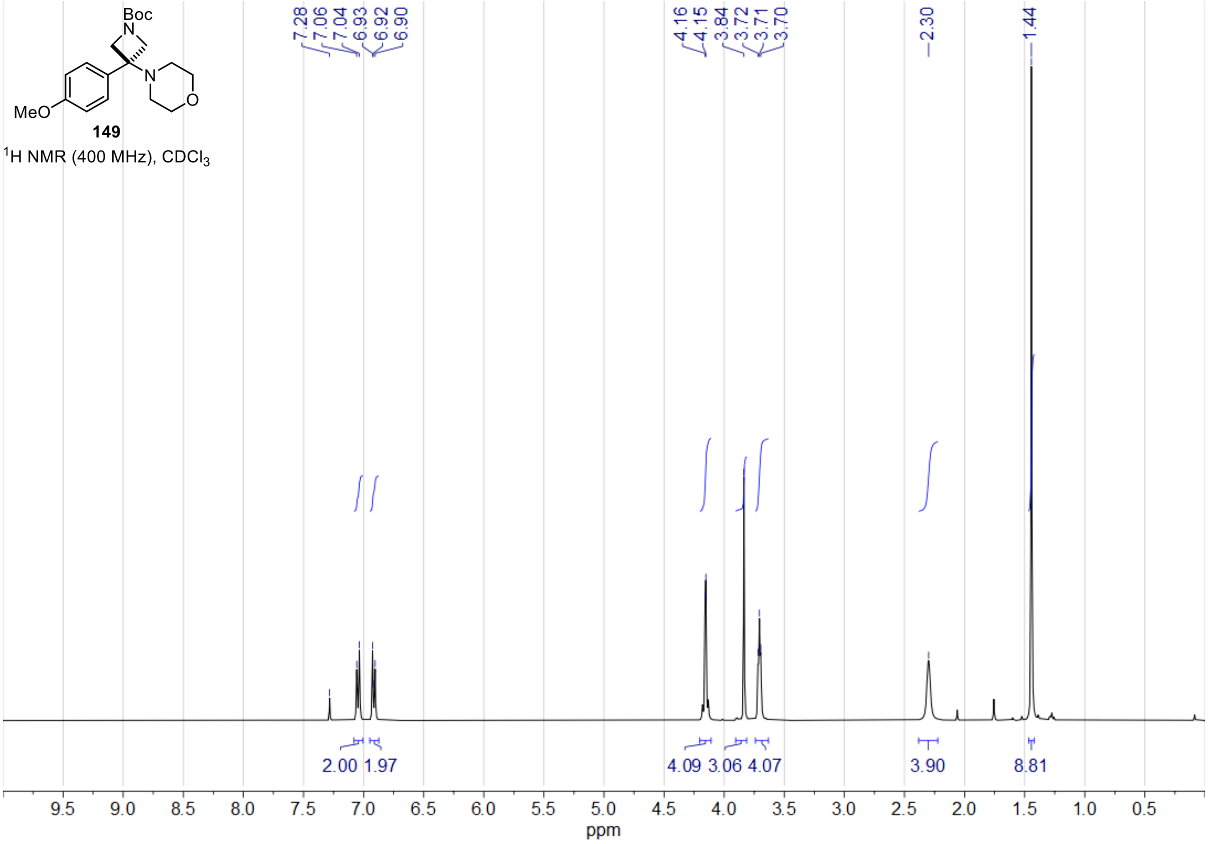

149

^{1}H NMR (400 MHz), $CDCl_3$

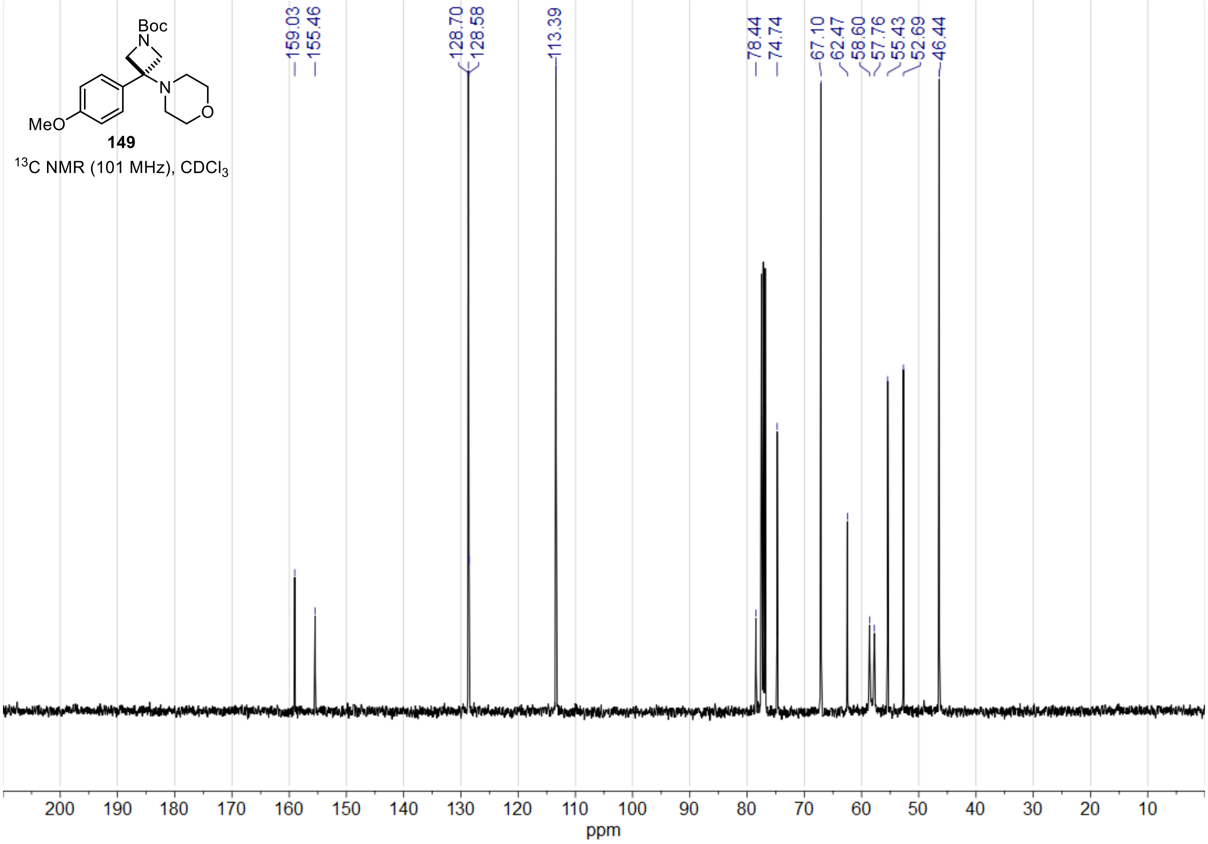

149

^{13}C NMR (101 MHz), $CDCl_3$

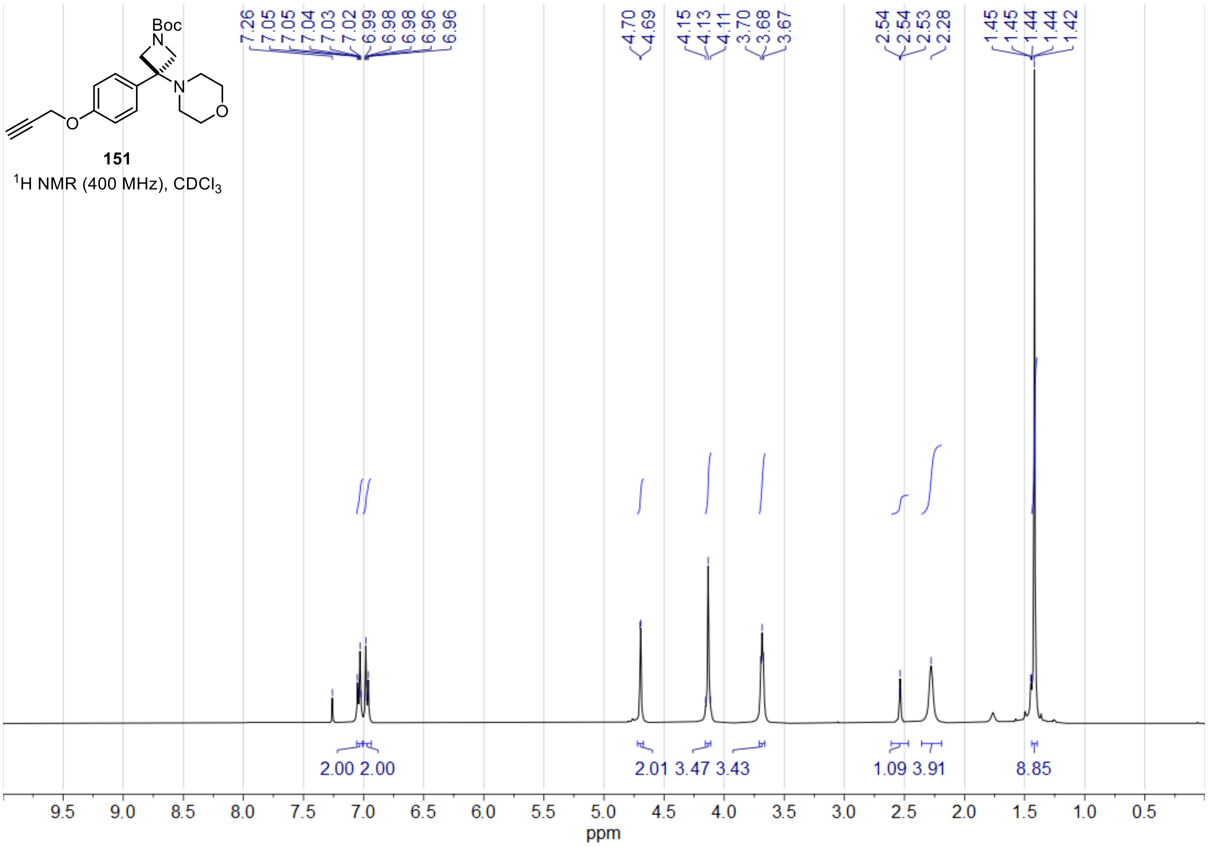

151

^{1}H NMR (400 MHz), $CDCl_3$

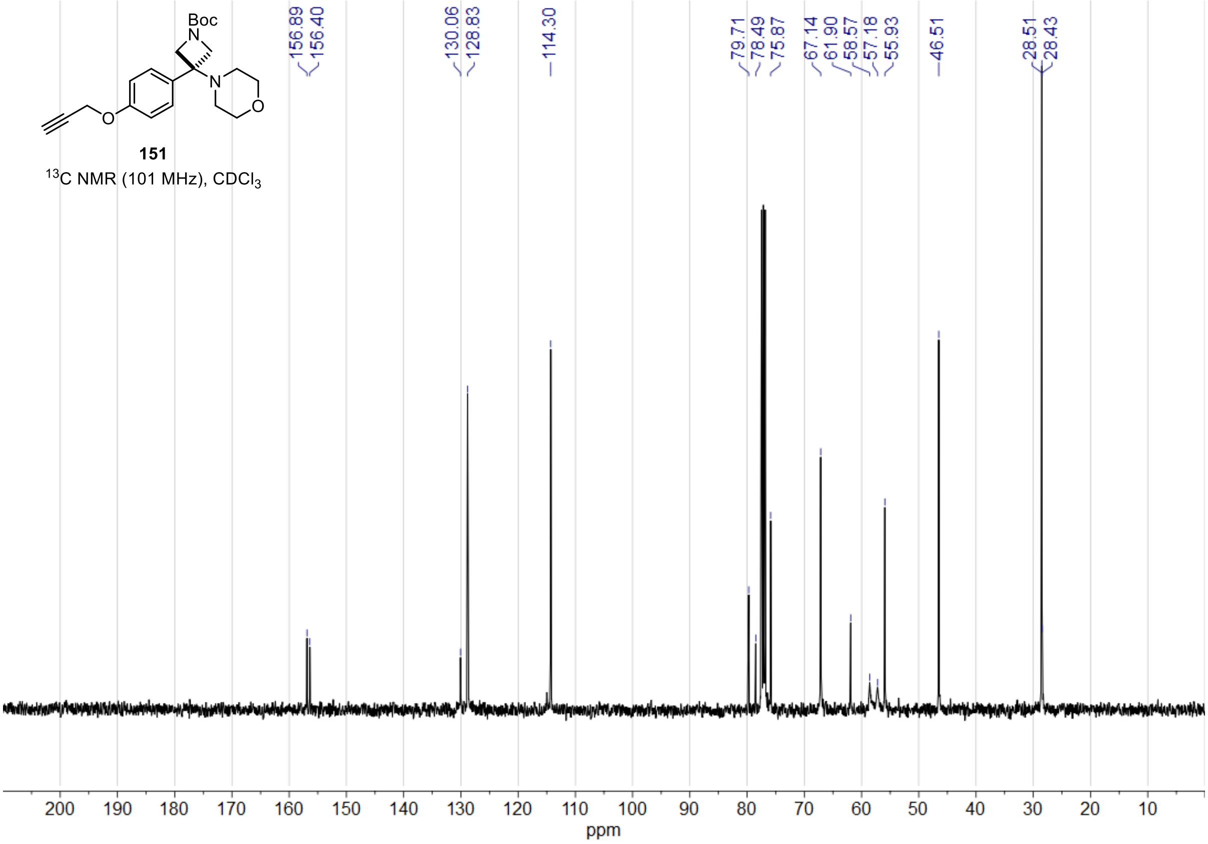

151

^{13}C NMR (101 MHz), $CDCl_3$

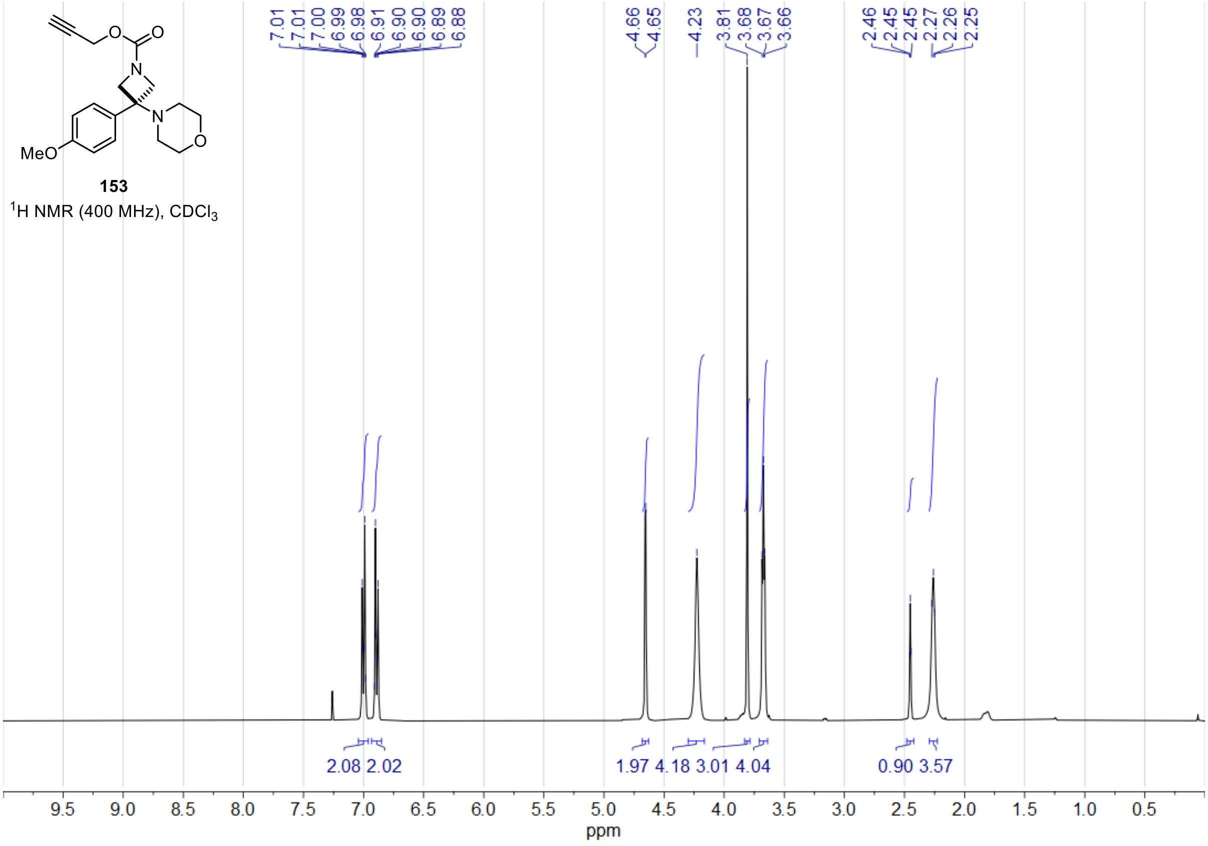

153

^{1}H NMR (400 MHz), $CDCl_3$

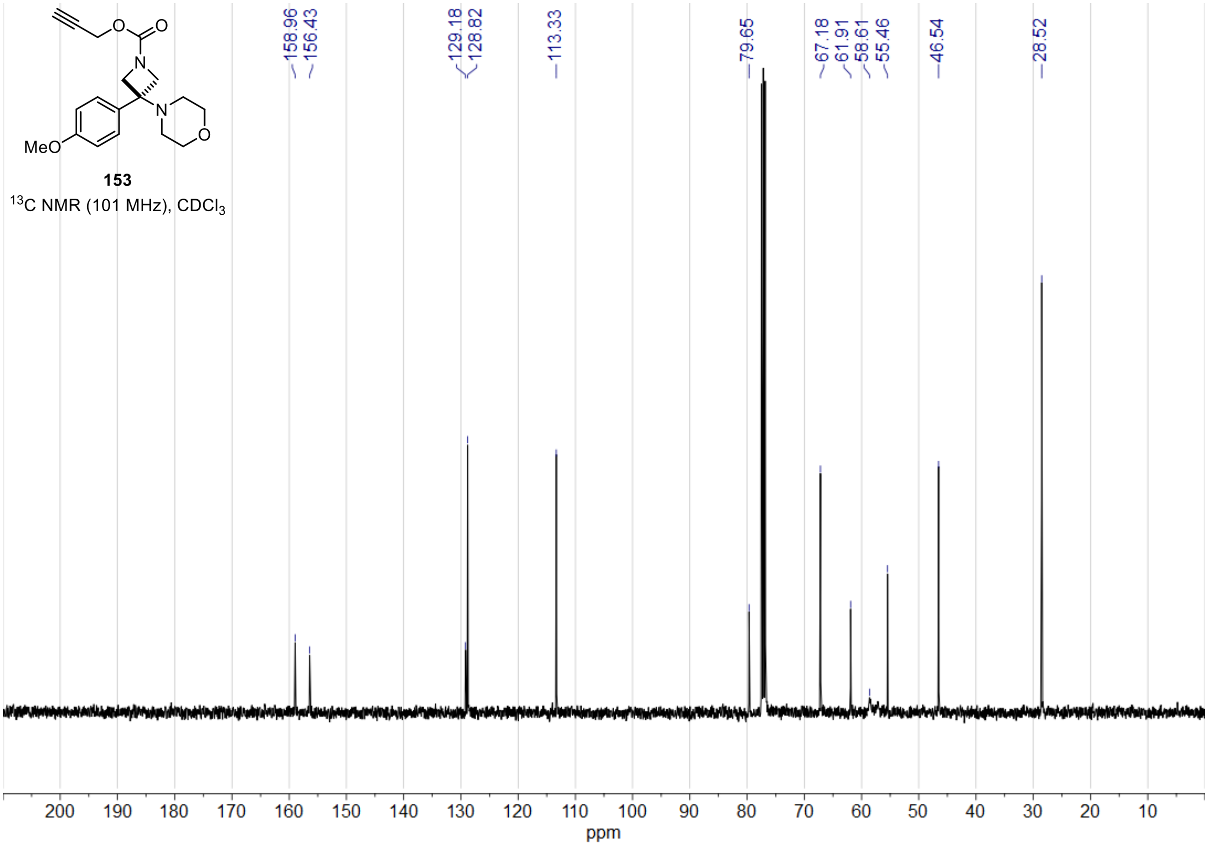

153

^{13}C NMR (101 MHz), $CDCl_3$

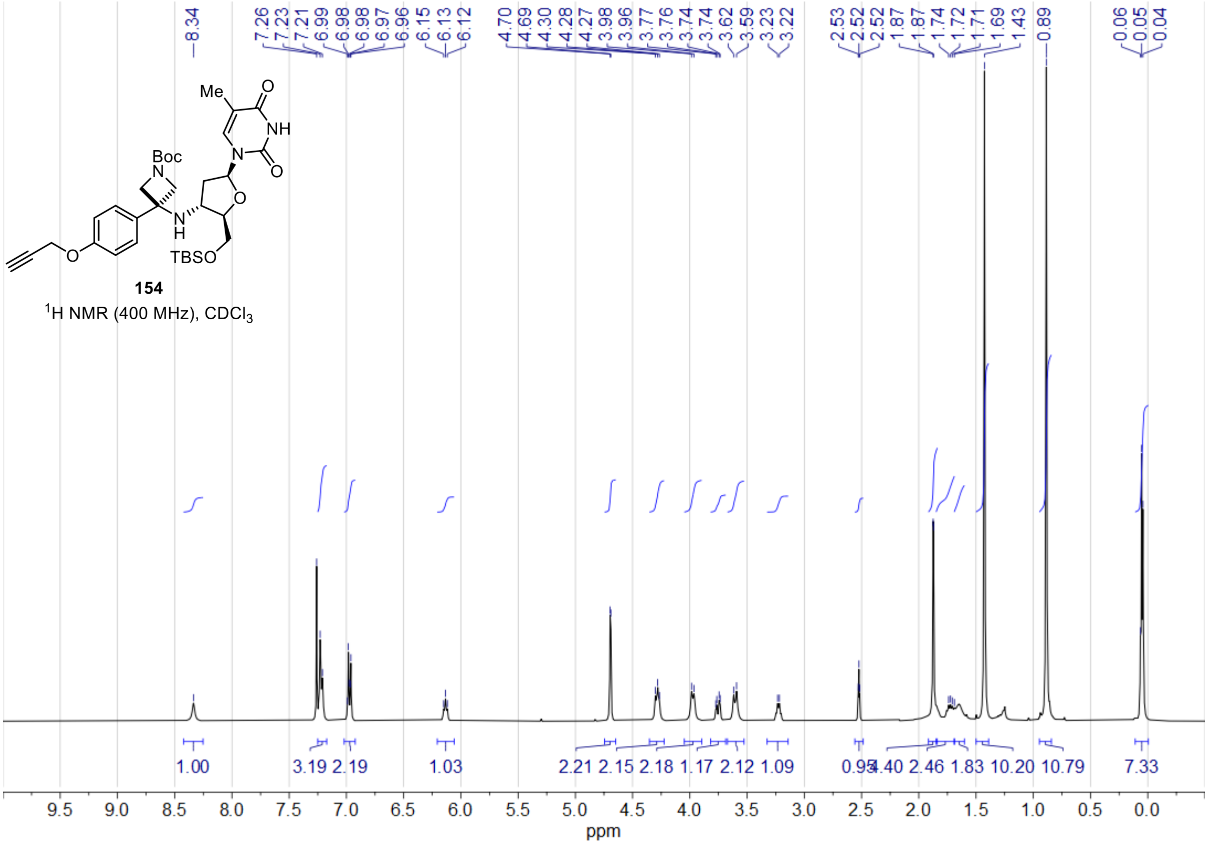

154

^{1}H NMR (400 MHz), $CDCl_3$

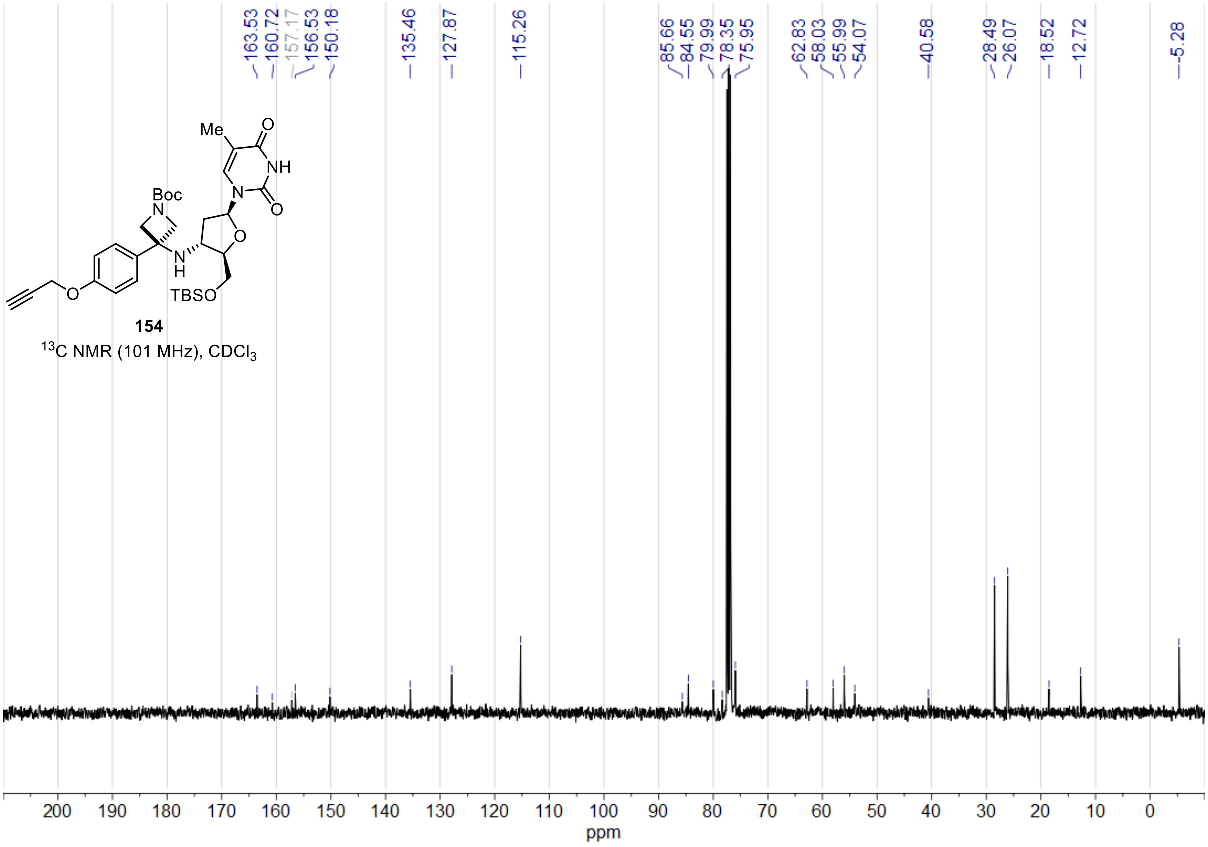

154

^{13}C NMR (101 MHz), $CDCl_3$

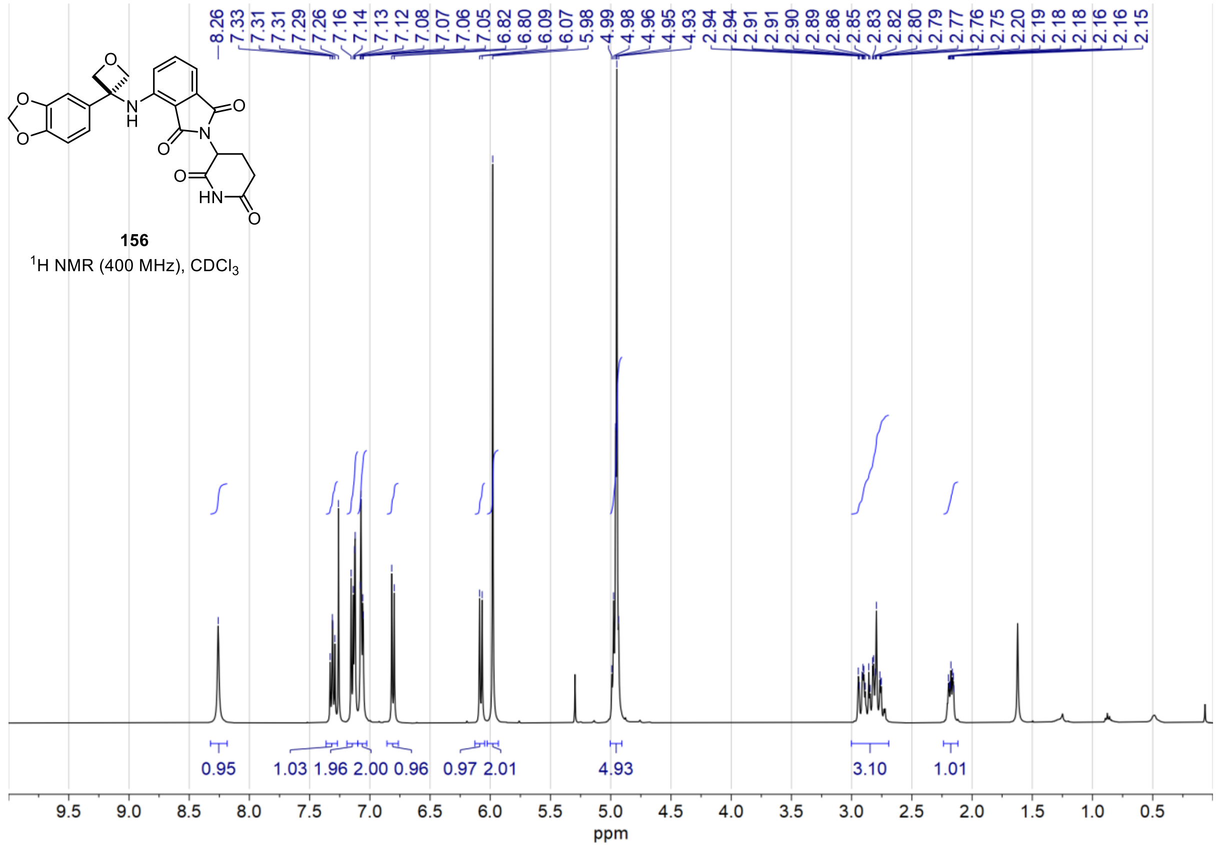
8.26
7.33
7.31
7.31
7.29
7.26
7.16
7.14
7.13
7.12
7.08
7.07
7.06
7.05
6.82
6.80
6.09
6.07
5.98
4.99
4.98
4.96
4.95
4.93
2.94
2.94
2.91
2.91
2.90
2.89
2.86
2.85
2.83
2.82
2.80
2.79
2.77
2.76
2.75
2.20
2.19
2.18
2.18
2.16
2.15
156
^{1}H NMR (400 MHz), $CDCl_3$
0.95
1.03
1.96
2.00
0.96
0.97
2.01
4.93
3.10
1.01
9.5 9.0 8.5 8.0 7.5 7.0 6.5 6.0 5.5 5.0 4.5 4.0 3.5 3.0 2.5 2.0 1.5 1.0 0.5
ppm

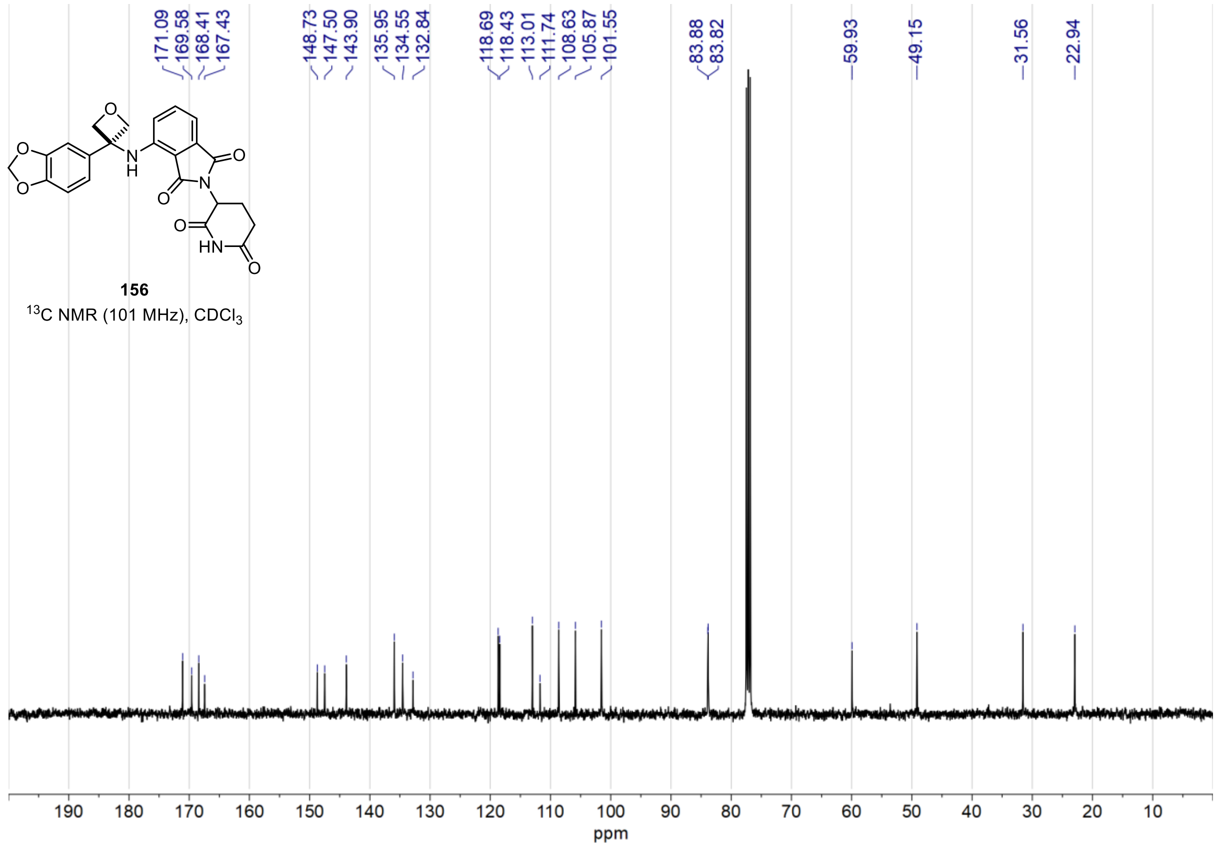
171.09
169.58
168.41
167.43
148.73
147.50
143.90
135.95
134.55
132.84
118.69
118.43
113.01
111.74
108.63
105.87
101.55
83.88
83.82
59.93
49.15
31.56
22.94
156
^{13}C NMR (101 MHz), $CDCl_3$
190 180 170 160 150 140 130 120 110 100 90 80 70 60 50 40 30 20 10
ppm

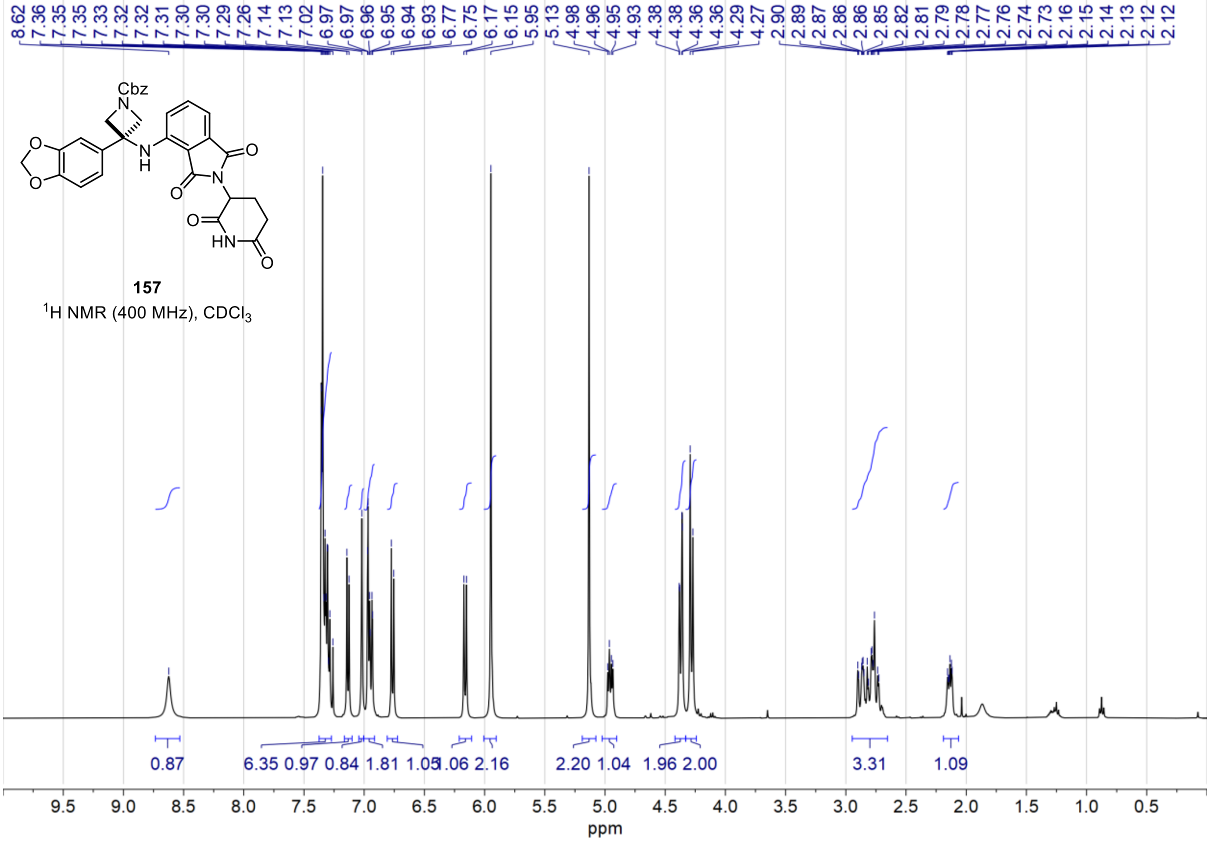

157

^{1}H NMR (400 MHz), $CDCl_3$

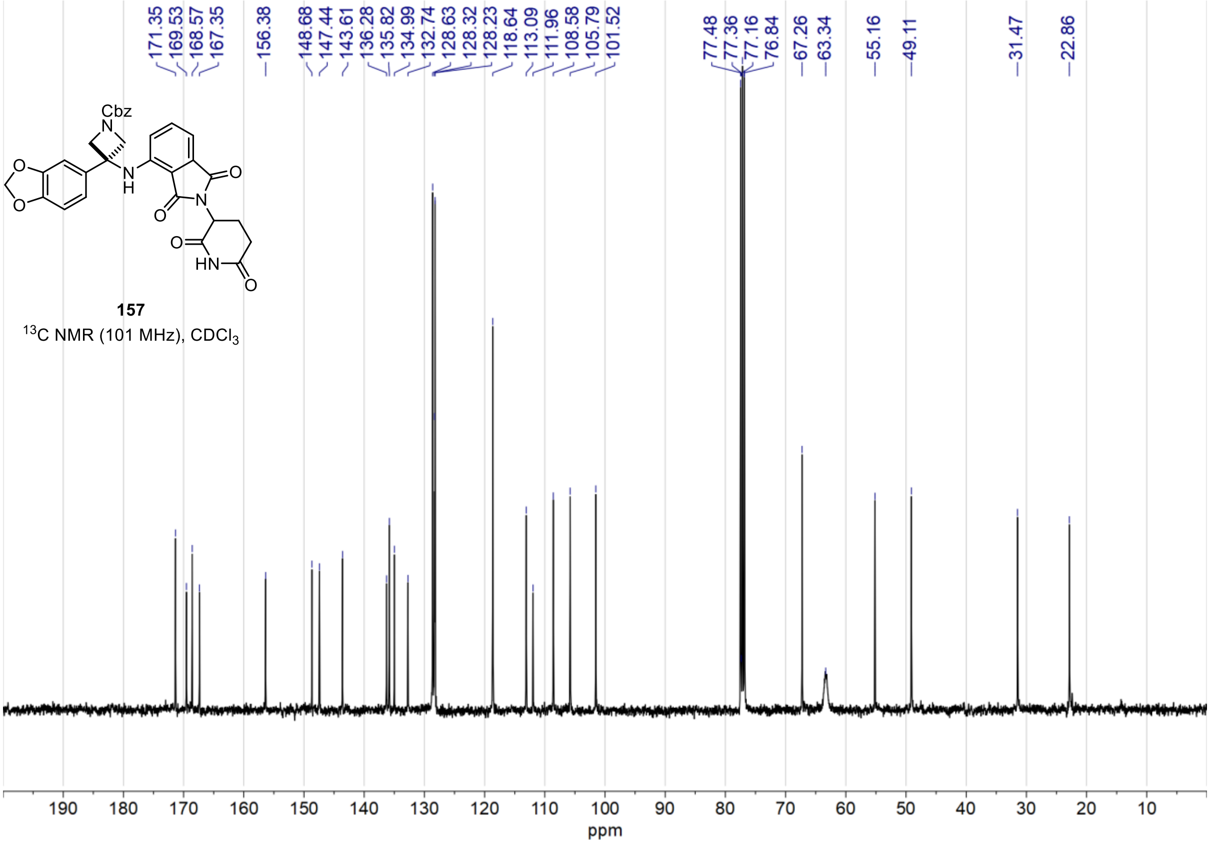

157

^{13}C NMR (101 MHz), $CDCl_3$

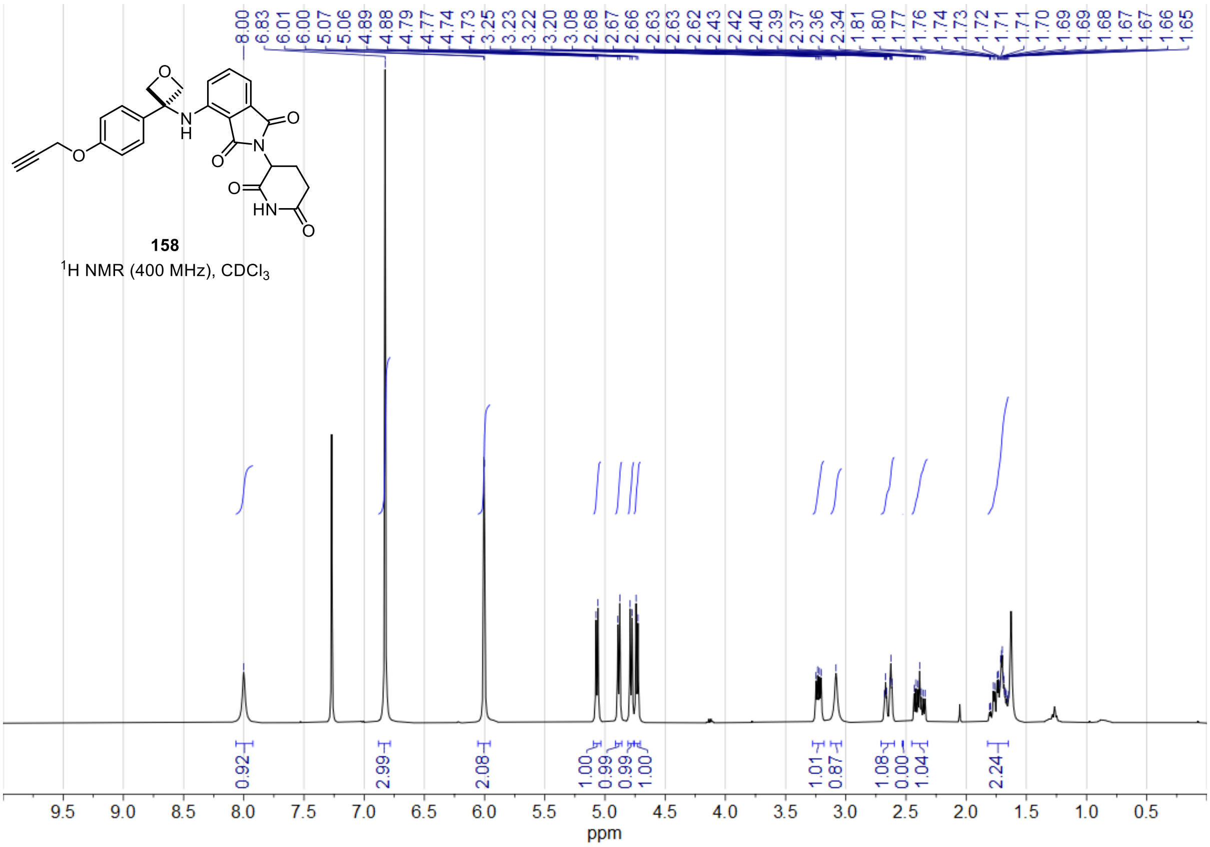
8.00
6.83
6.01
6.00
5.07
5.06
4.89
4.88
4.79
4.77
4.74
4.73
3.25
3.23
3.22
3.20
3.08
2.68
2.67
2.66
2.63
2.63
2.62
2.43
2.42
2.40
2.39
2.37
2.36
2.34
1.81
1.80
1.77
1.76
1.74
1.73
1.72
1.71
1.71
1.70
1.69
1.69
1.68
1.67
1.67
1.66
1.65
158
1H NMR (400 MHz), CDCl3
0.92
2.99
2.08
1.00
0.99
0.99
1.00
1.01
0.87
1.08
0.00
1.04
2.24
9.5
9.0
8.5
8.0
7.5
7.0
6.5
6.0
5.5
5.0
4.5
4.0
3.5
3.0
2.5
2.0
1.5
1.0
0.5
ppm

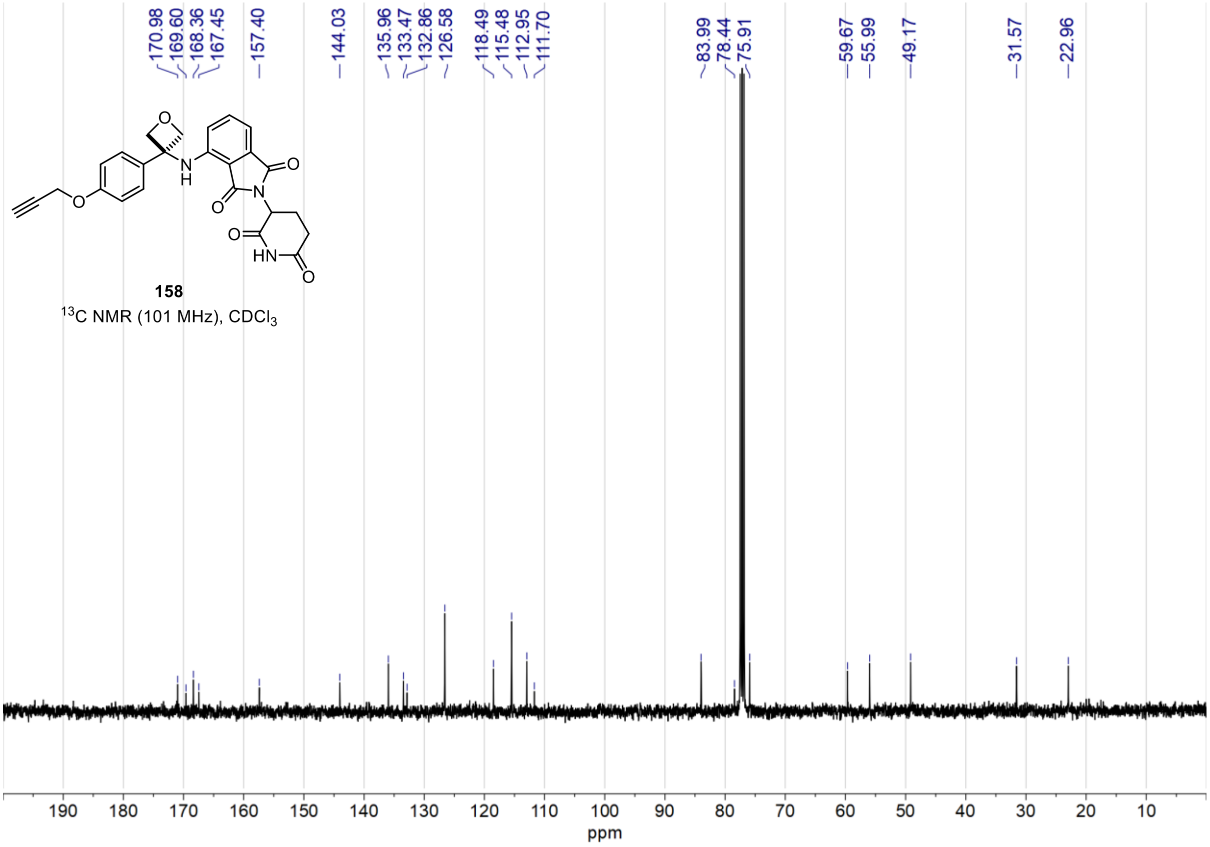
170.98
169.60
168.36
167.45
157.40
144.03
135.96
133.47
132.86
126.58
118.49
115.48
112.95
111.70
83.99
78.44
75.91
59.67
55.99
49.17
31.57
22.96
158
13C NMR (101 MHz), CDCl3
190
180
170
160
150
140
130
120
110
100
90
80
70
60
50
40
30
20
10
ppm

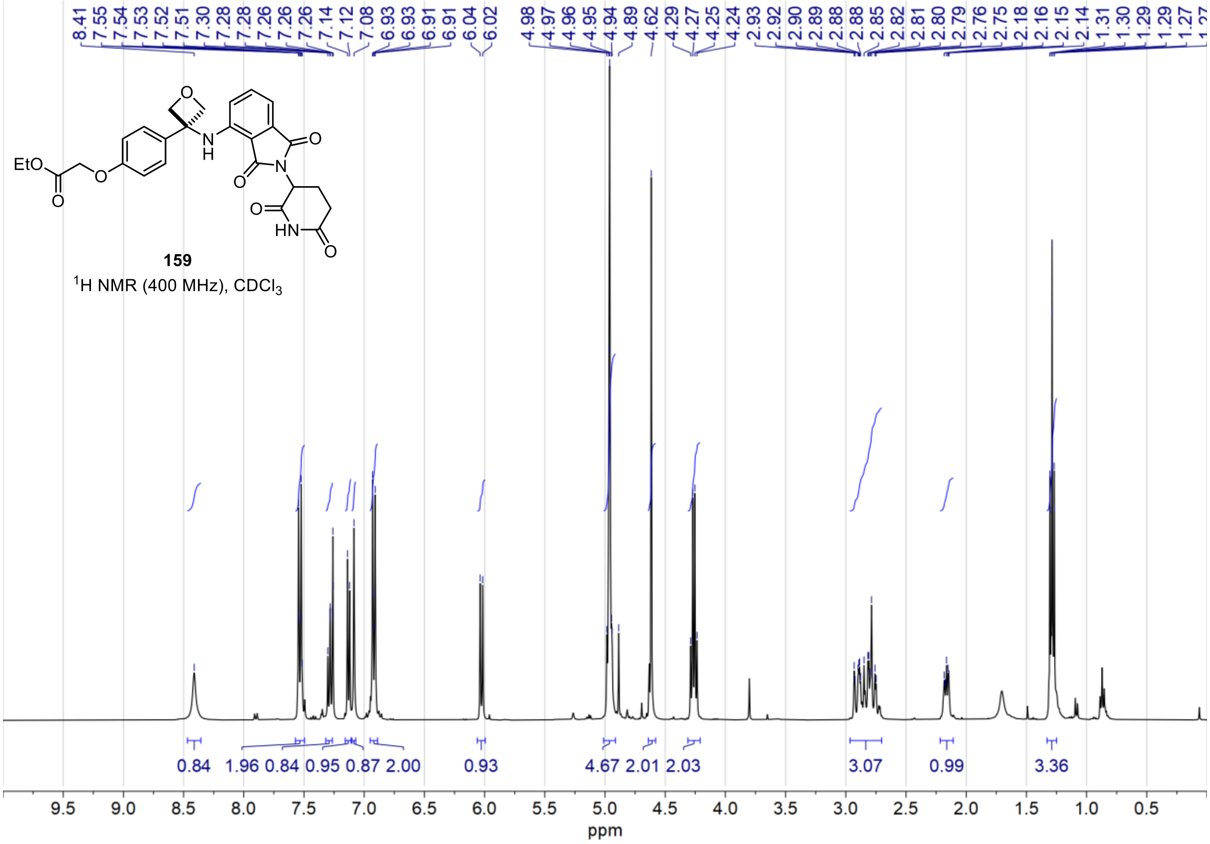
8.41
7.55
7.54
7.53
7.52
7.51
7.30
7.28
7.26
7.26
7.26
7.14
7.12
7.08
6.93
6.93
6.91
6.91
6.04
6.02
4.98
4.97
4.96
4.95
4.94
4.89
4.62
4.29
4.27
4.25
4.24
2.93
2.92
2.90
2.89
2.88
2.88
2.85
2.82
2.81
2.80
2.79
2.76
2.75
2.18
2.16
2.15
2.14
1.31
1.30
1.29
1.27
EtO
O
N
H
O
O
N
O
O
HN
O
159
¹H NMR (400 MHz), CDCl₃
0.84
1.96
0.84
0.95
0.87
2.00
0.93
4.67
2.01
2.03
3.07
0.99
3.36
9.5
9.0
8.5
8.0
7.5
7.0
6.5
6.0
5.5
5.0
4.5
4.0
3.5
3.0
2.5
2.0
1.5
1.0
0.5
ppm

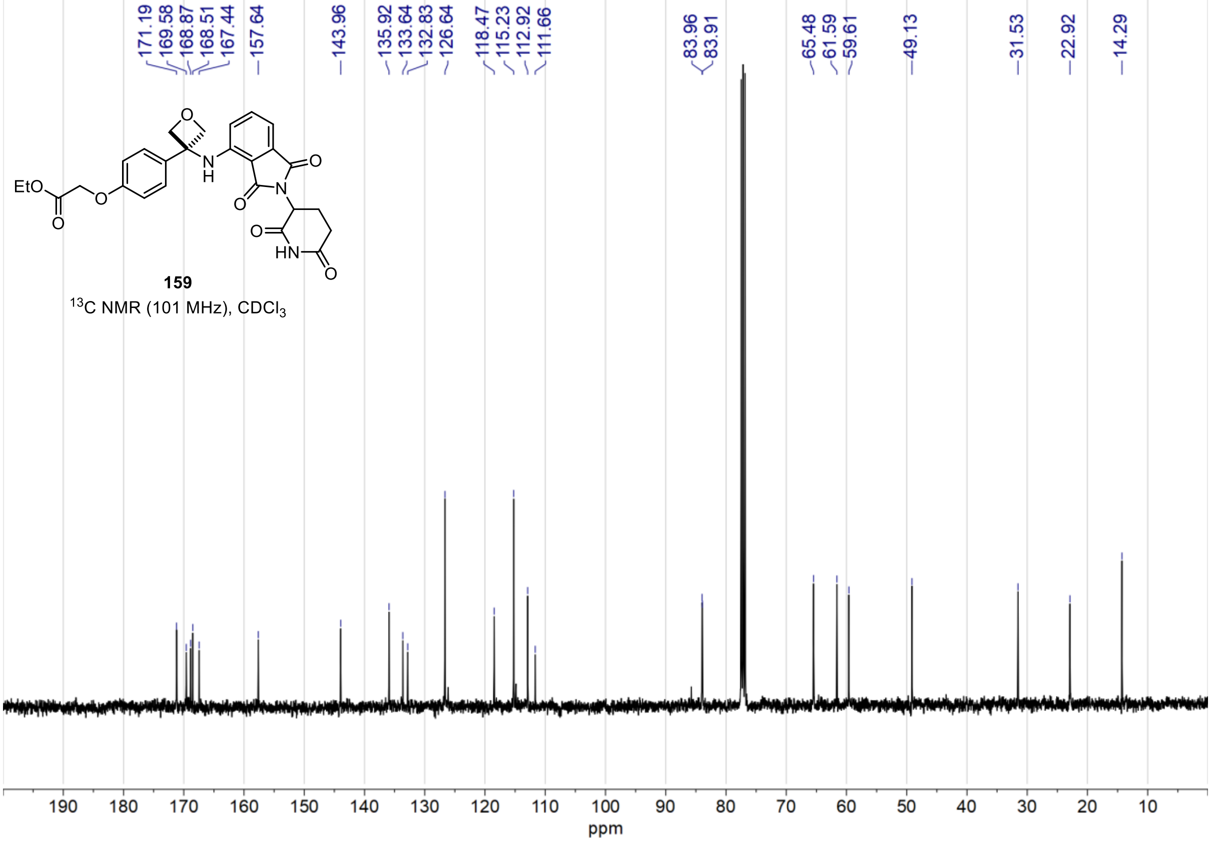
171.19
169.58
168.87
168.51
167.44
157.64
143.96
135.92
133.64
132.83
126.64
118.47
115.23
112.92
111.66
83.96
83.91
65.48
61.59
59.61
49.13
31.53
22.92
14.29
EtO
O
N
H
O
O
N
O
O
HN
O
159
¹³C NMR (101 MHz), CDCl₃
190
180
170
160
150
140
130
120
110
100
90
80
70
60
50
40
30
20
10
ppm

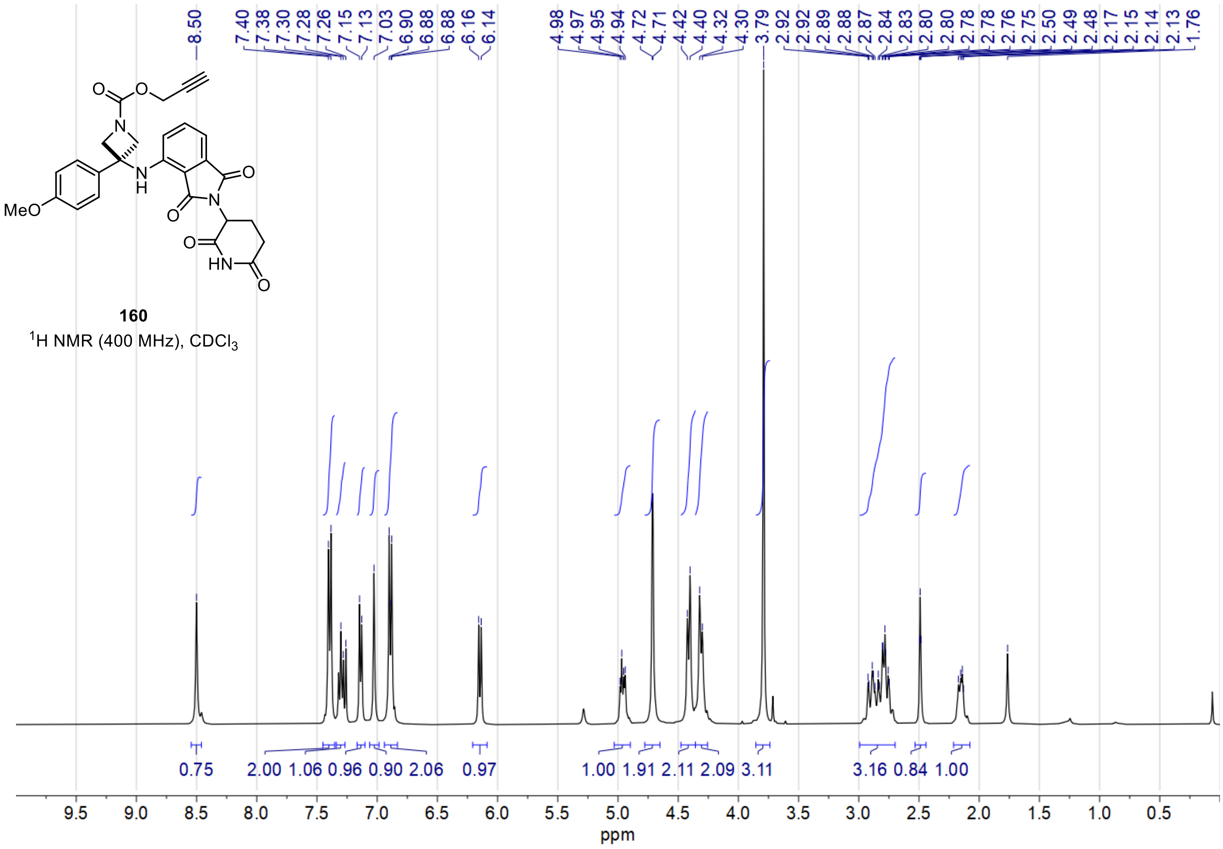

160

^{1}H NMR (400 MHz), $CDCl_3$

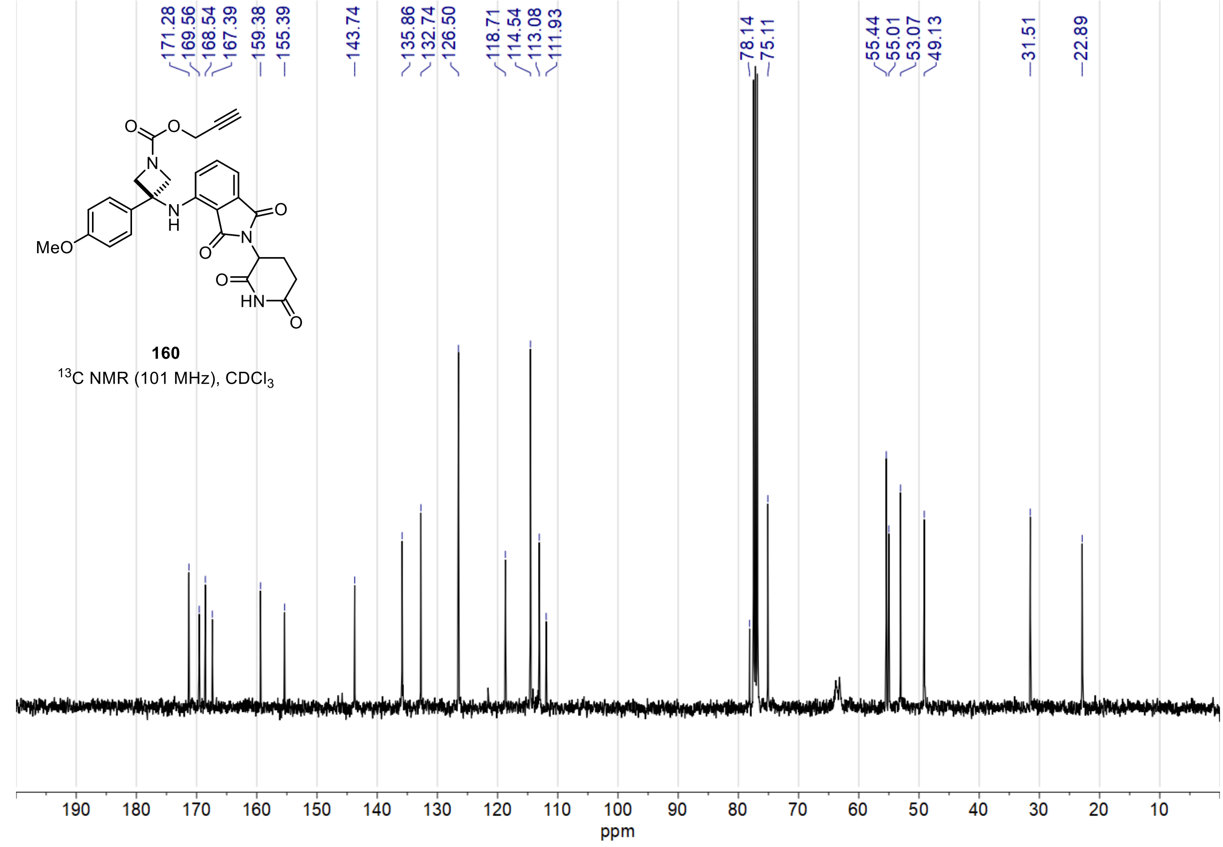

160

^{13}C NMR (101 MHz), $CDCl_3$

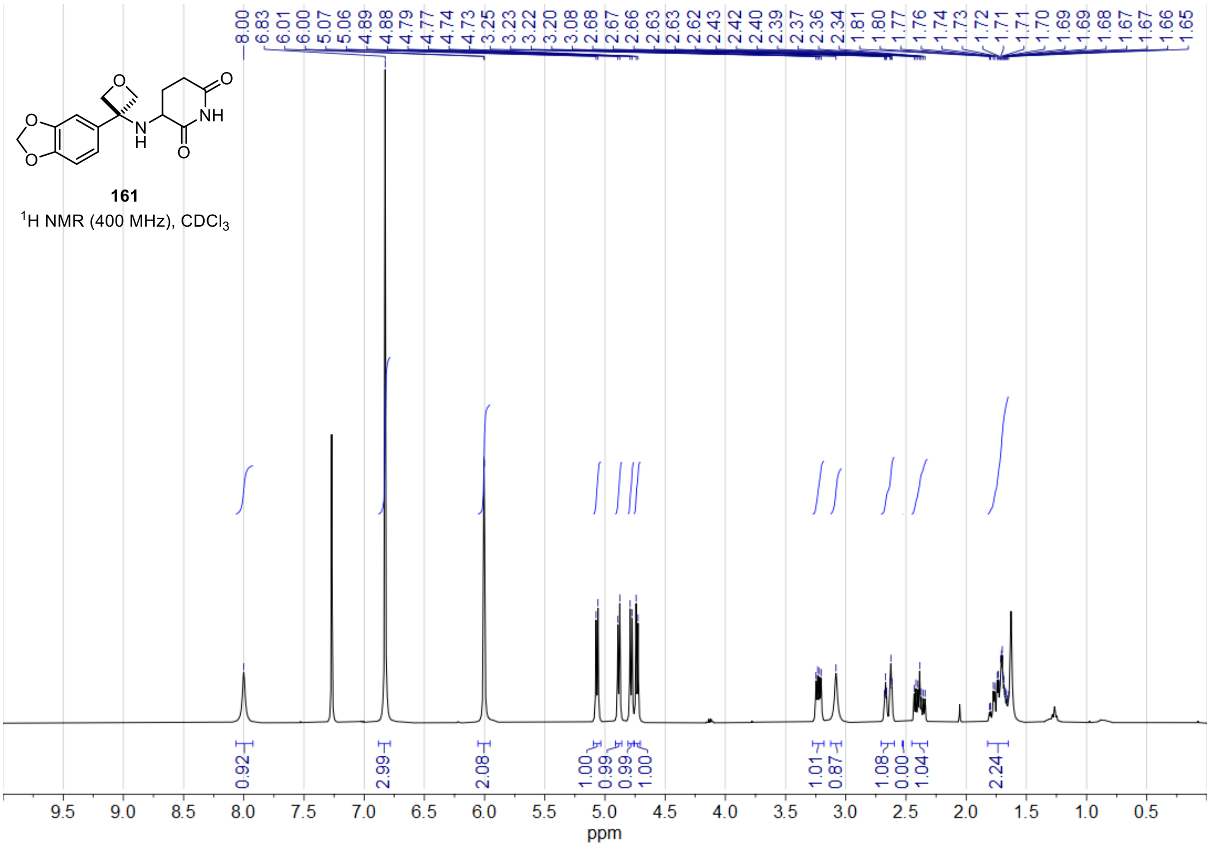

161

^{1}H NMR (400 MHz), $CDCl_3$

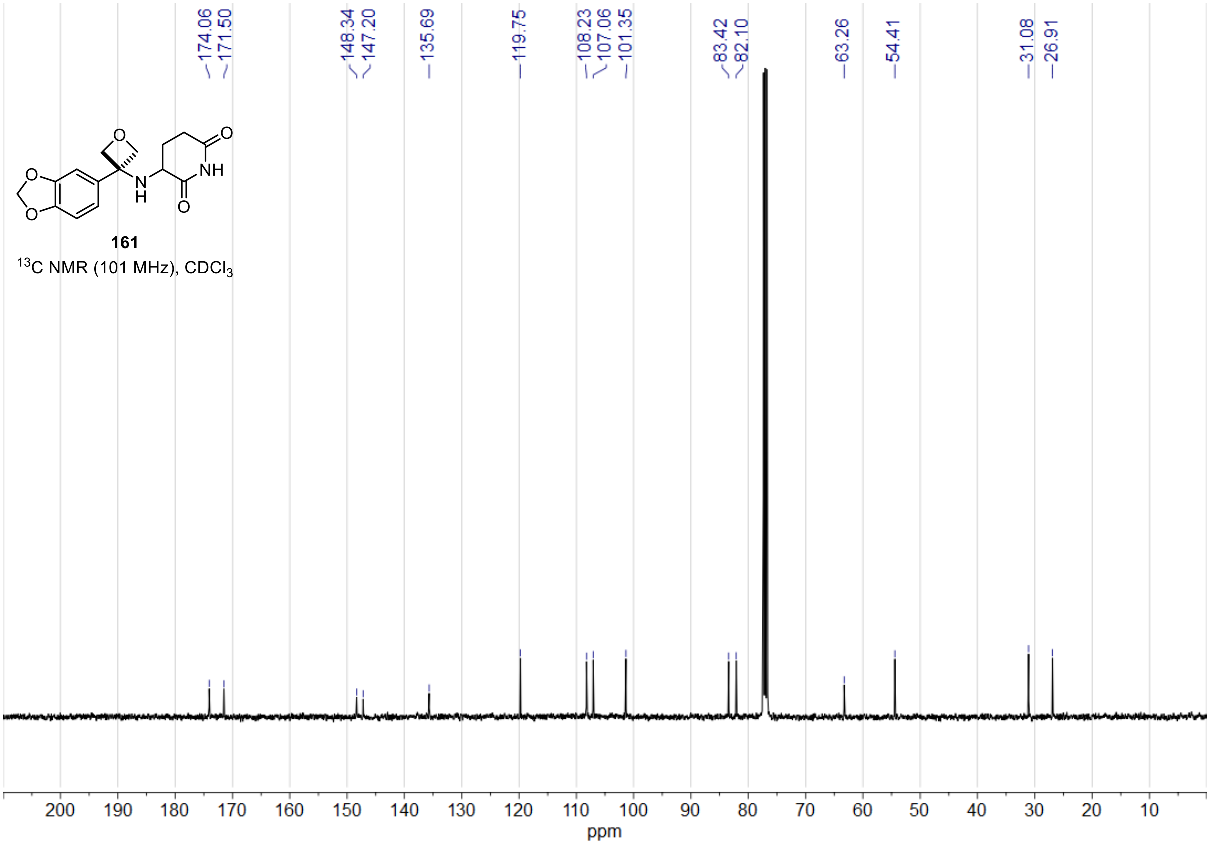

161

^{13}C NMR (101 MHz), $CDCl_3$

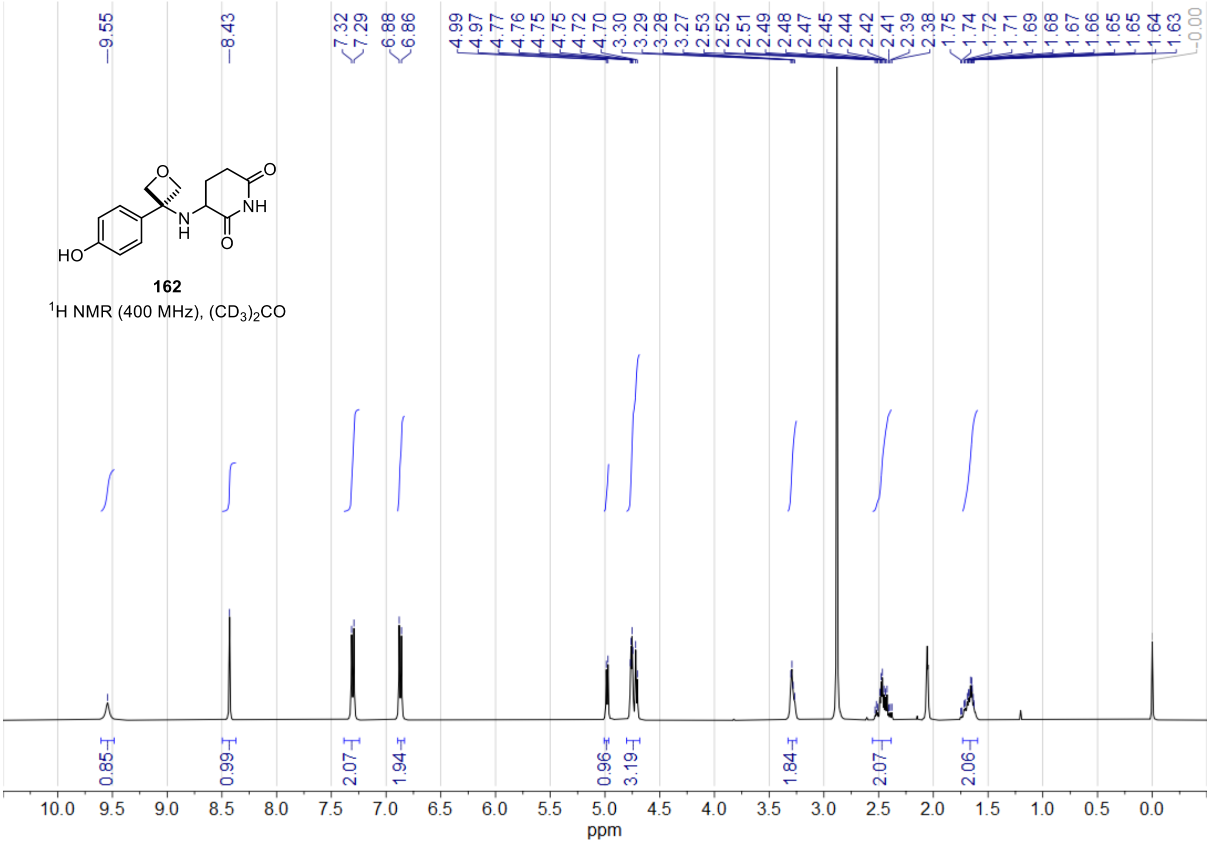
9.55
8.43
7.32
7.29
6.88
6.86
4.99
4.97
4.77
4.76
4.75
4.75
4.72
4.70
3.30
3.29
3.28
3.27
2.53
2.52
2.51
2.49
2.48
2.47
2.45
2.44
2.42
2.41
2.39
2.38
1.75
1.74
1.72
1.71
1.69
1.68
1.67
1.66
1.65
1.65
1.64
1.63
0.00
HO
O
N
H
NH
O
O
162
^{1}H NMR (400 MHz), $(CD_3)_2CO$
0.85
0.99
2.07
1.94
0.96
3.19
1.84
2.07
2.06
ppm

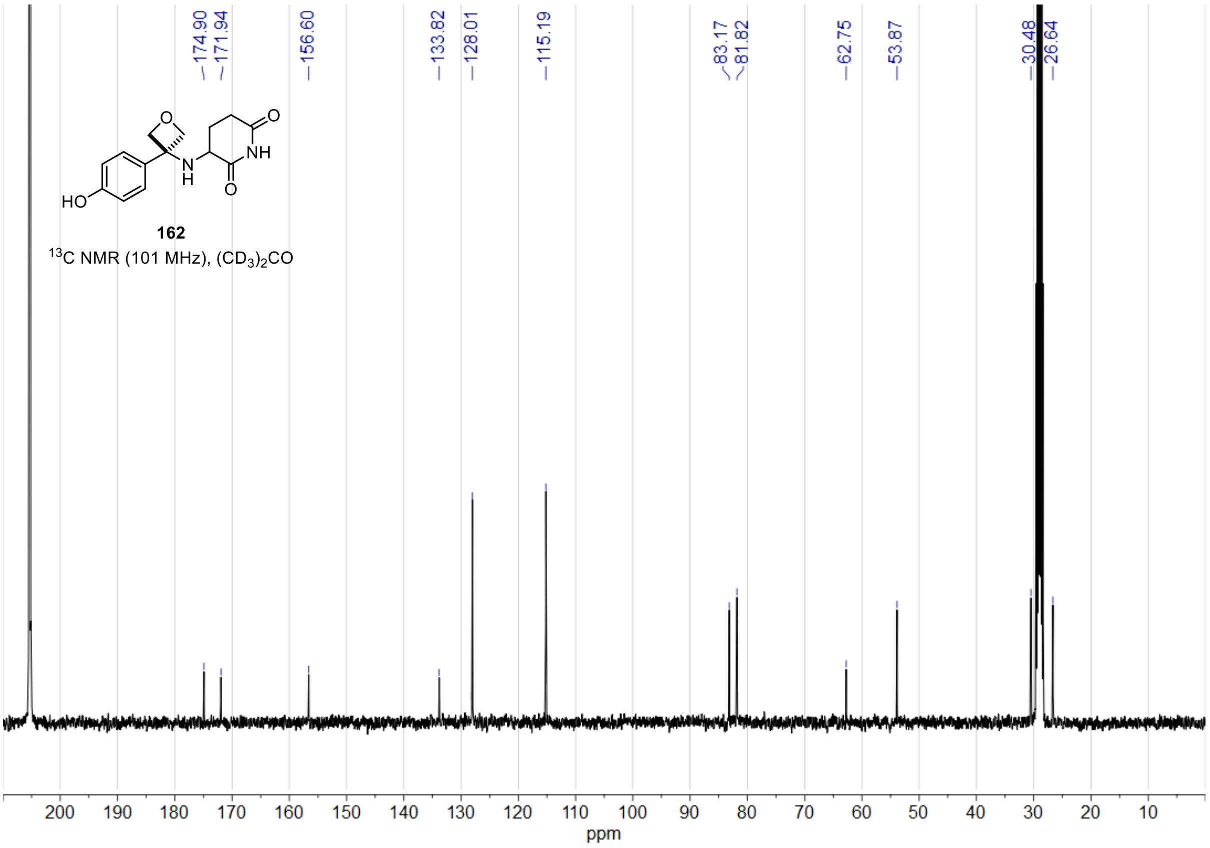
174.90
171.94
156.60
133.82
128.01
115.19
83.17
81.82
62.75
53.87
30.48
26.64
HO
O
N
H
NH
O
O
162
^{13}C NMR (101 MHz), $(CD_3)_2CO$
ppm

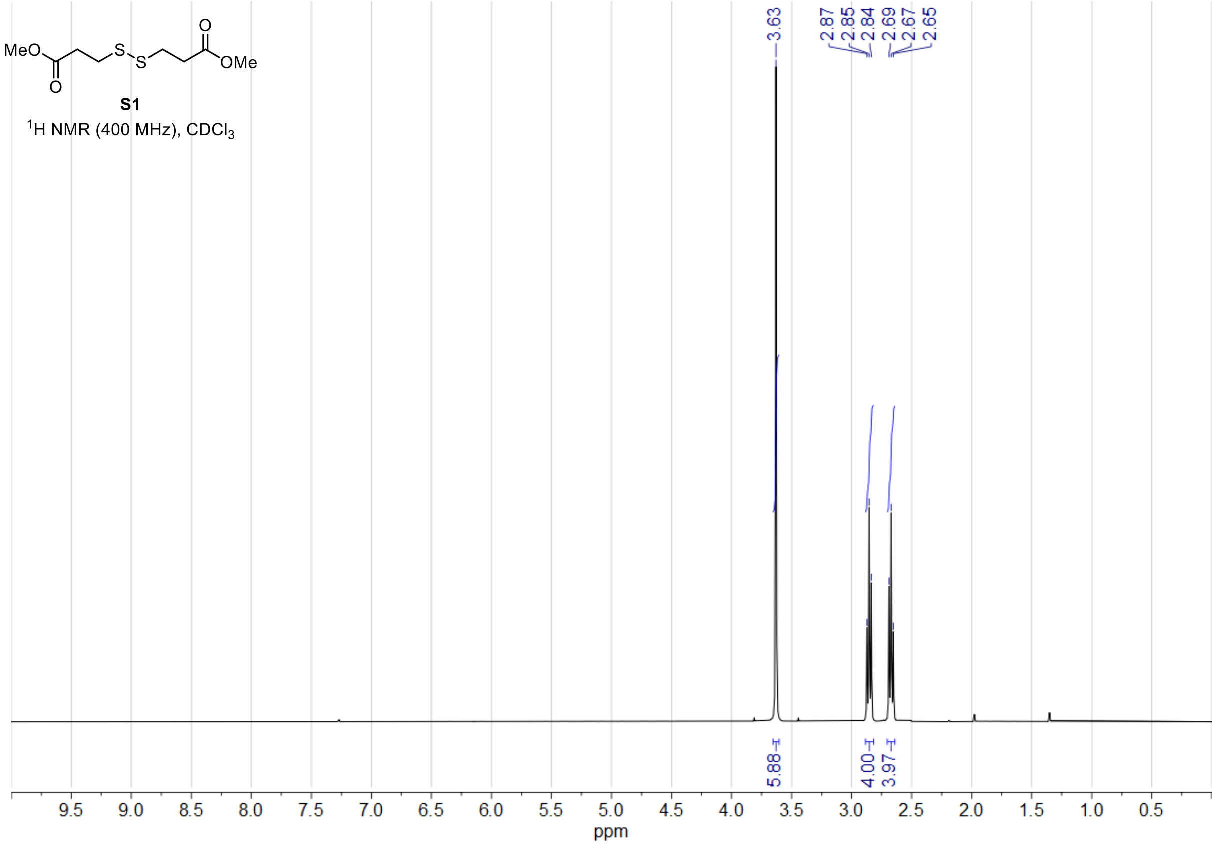
MeO
O
S
S
O
OMe
S1
1H NMR (400 MHz), CDCl3
3.63
2.87
2.85
2.84
2.69
2.67
2.65
5.88
4.00
3.97
9.5 9.0 8.5 8.0 7.5 7.0 6.5 6.0 5.5 5.0 4.5 4.0 3.5 3.0 2.5 2.0 1.5 1.0 0.5
ppm

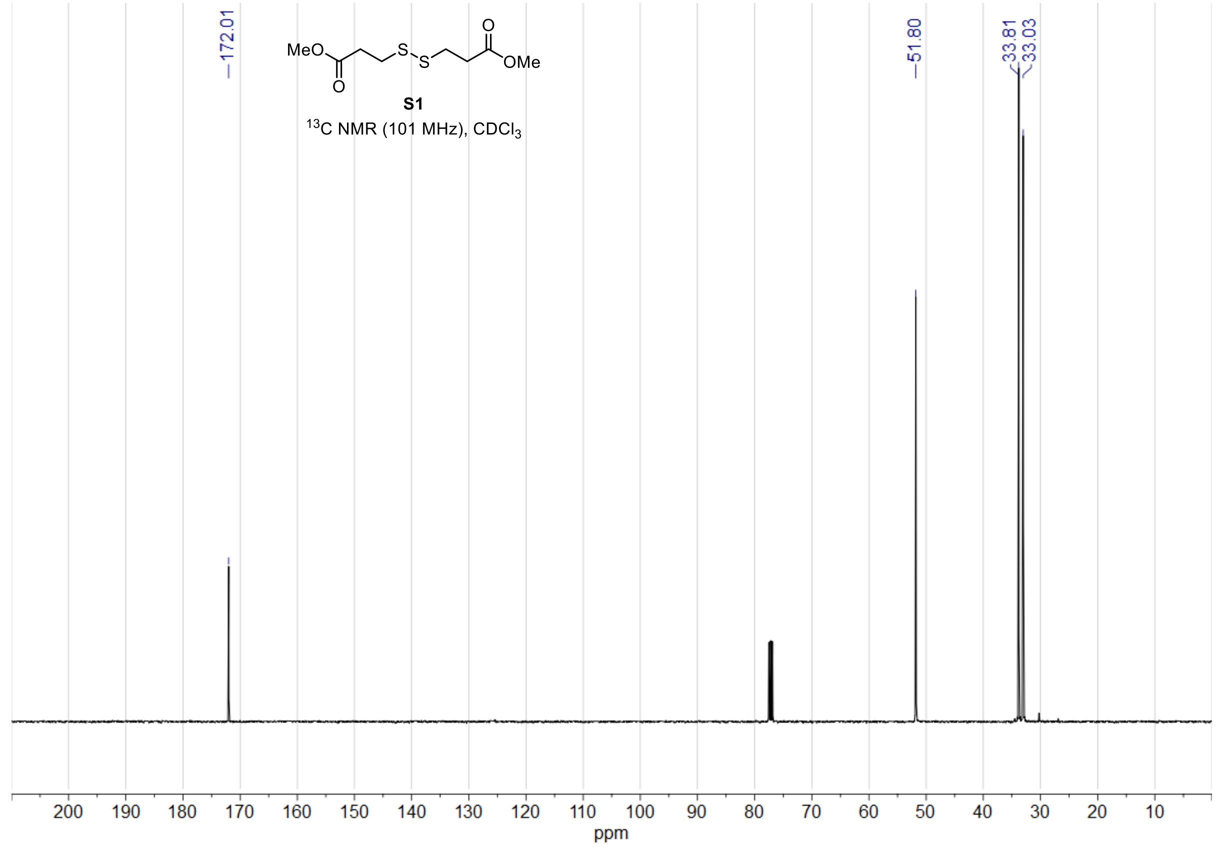
172.01
MeO
O
S
S
O
OMe
S1
13C NMR (101 MHz), CDCl3
51.80
33.81
33.03
200 190 180 170 160 150 140 130 120 110 100 90 80 70 60 50 40 30 20 10
ppm
